# Organoytterbium Ate Complexes Extend the Value of Cyclobutenediones as Isoprene Equivalents^**^

**DOI:** 10.1002/anie.201307193

**Published:** 2013-10-23

**Authors:** Emma Packard, David D Pascoe, Jacques Maddaluno, Théo P Gonçalves, David C Harrowven

**Affiliations:** Chemistry, University of Southampton Highfield, Southampton, SO17 1BJ (United Kingdom); FR CNRS 3038, IRCOF, Université de Rouen 76821 Mont St. Aignan Cedex (France)

**Keywords:** density functional calculations, flow chemistry, natural products, rearrangement, ytterbium

3-Methyl-4-methoxycyclobuten-1,2-dione (**1 a**) has long been recognized as a valuable isoprene equivalent in natural products total synthesis, particularly for the preparation of hydroquinones, quinones, and their benzannulated analogues (e.g. Scheme 1).^1^, 2a It is readily introduced to a substrate as the electrophilic component in organolithium or Grignard addition reactions, for example, **1 a**→**2**. Here, the differential reactivity of the C1 and C2 carbonyl groups in **1 a** adds to its value as a synthon by providing a reliable and predictable means of achieving the ubiquitous head to tail connectivity of isoprene units.^1^, 2a

**Scheme 1:**
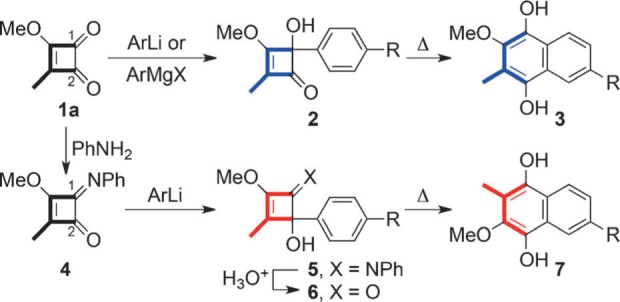
Use of 1 a as an isoprene equivalent in the synthesis of benzohydroquinones.

Though regiocontrol in the addition of Grignard and organolithium reagents to **1 a** is of critical importance, it also imposes a severe limitation. As evidenced by the example in Scheme 1, while the method is convenient for the synthesis of benzohydroquinones such as **3**, it proves cumbersome when targeting the regioisomeric series **7**. In this case addition of a carbon nucleophile to the vinylogous ester carbonyl (C2) of **1 a** is required to achieve the desired outcome. Consequently, a protecting group strategy must be invoked to mask the more reactive C1 carbonyl (e.g. **1 a**→**4**).^2^ Addition of the Grignard or organolithium reagent to C1 is then followed by deprotection (e.g. **5**→**6**), which can be difficult to achieve efficiently because of the presence of the acid sensitive tertiary alcohol and enol ether functions.

Herein we report an expedient solution to that longstanding problem and reveal some hitherto unknown facets of organoytterbium reactivity. In essence, while organolithium and Grignard reagents favor addition to the C1 carbonyl of cyclobutenedione (**1 a**), the corresponding organoytterbium reagents give exclusive addition to the C2 carbonyl of **1 b** (Scheme 2).^3^

**Scheme 2:**
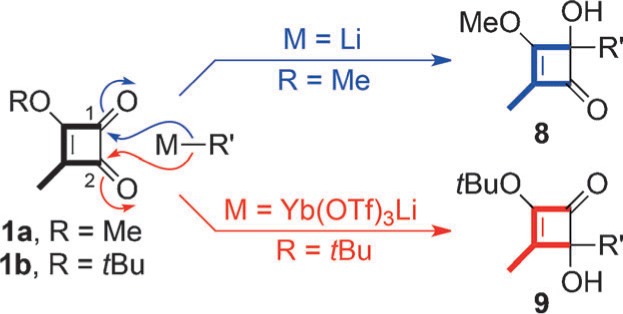
Dichotomous regioselectivity in the addition of organolithium and organoytterbium reagents to cyclobutenediones. Tf=trifluoromethanesulfonyl.

The discovery was made during an optimization study aimed at reducing side reactions resulting from deprotonation of the C3 methyl substituent in **1 a**. In the addition of PhLi, for example (Scheme 3), these appeared to limit the yield of the adduct **2 a** to approximately 70–80 % when employing standard protocols (e.g. THF at −78 °C).^4^ Inspired by reports from the groups of Molander and Procter on the efficient addition of organoytterbium reagents to carbonyl compounds,^5^ we decided to examine the addition of phenylytterbium triflate to **1 a**.^2^ The result we attained was quite unexpected, as the reaction led to the formation of both C1 and C2 adducts, **2 a** and **11 a**, respectively, in the corresponding yields of 50 % and 41 % (Scheme 3). Having noted similar anomalies in the additions of PhLi and PhMgBr to **1 b**, we were delighted to find that the addition of phenylytterbium triflate to **1 b** resulted in a complete reversal of the normal regiochemical course, thus giving the C2 adduct **12 a** in a remarkable 92 % yield upon isolation.^5^, ^6^

**Scheme 3:**
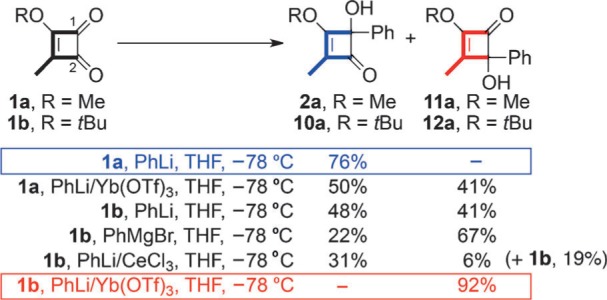
Regioselectivity in the additions of various phenyl organometallics to 1 a and 1 b. THF=tetrahydrofuran.

To demonstrate the value of the method, a series of arylytterbium reagents were prepared and reacted with **1 b**. These included *ortho*-,^7^ *meta*-,^7^ and *para*-substituted aromatics with a variety of substituents (+I, −I, and +M), and a heteroaromatic example^7^ (see Scheme 4 and the Supporting Information). In each case the C2 adducts **12** were formed in excellent yield. This result was in stark contrast to the corresponding organolithium additions to **1 a** where the major product **2** resulted from addition to the C1 carbonyl.^7^ For completeness, all adducts were subjected to thermolysis and aerial oxidation, thus leading to the complementary series of benzoquinones **15** and **16**.

**Scheme 4:**
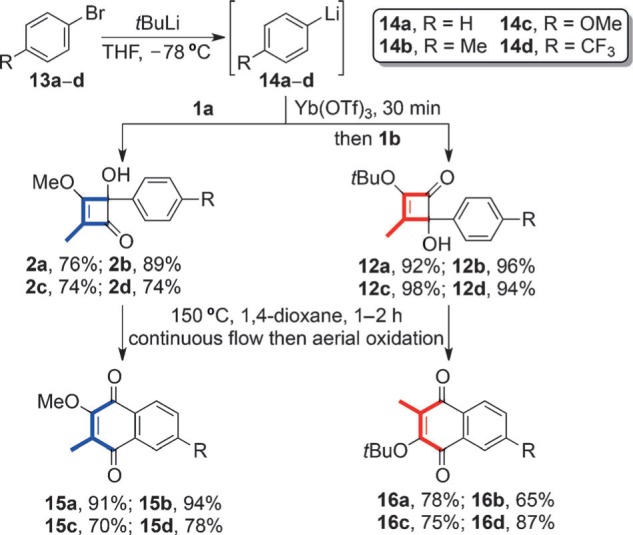
Synthesis of regioisomeric series of benzoquinones using aryllithium and arylytterbium additions to 1 a and 1 b.

The complementary reactivity extended to vinyllithium and vinylytterbium triflate additions to **1 a** and **1 b** as demonstrated by the syntheses of the hydroquinones **23**–**25** and **27** (Scheme 5).^1^ Notably, vinylytterbium triflate derived from vinylmagnesium bromide displayed the same regiochemical preference as those derived from vinyllithium intermediates. Interestingly, the addition of the vinyllithium **18** to **1 a** proved unusual in that it gave **27** directly after a bicarbonate quench at −78 °C and warming to ambient temperature. The minor product of the reaction was the C2 adduct **21 b**,^7^ rather than the corresponding hydroquinone.

**Scheme 5:**
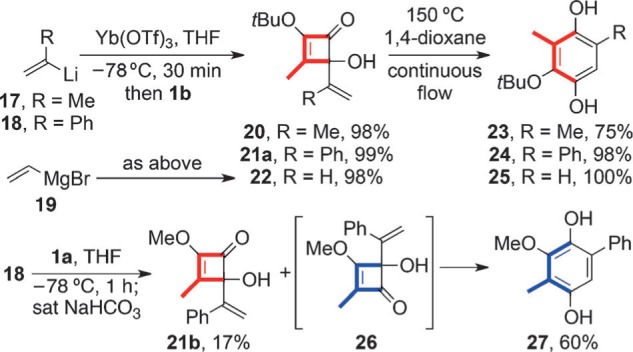
Synthesis of regioisomeric series of hydroquinones by vinyllithium and vinylytterbium additions to 1 a and 1 b.

The addition of methylytterbium triflate to **1 b** also proved facile (Scheme 6). In contrast, the analogous sequence with *n*BuLi required a 2:1 equivalence with ytterbium(III) triflate to achieve a high yield of the C2 adduct **28 b**.^8^ The photochemical rearrangement of the adduct **28 a** into the furanone **29**,^9^ and the facile generation of the diorganylcyclobutendiones **30** by exposure of these adducts to acid, provide further illustrations of the method’s utility.

**Scheme 6:**
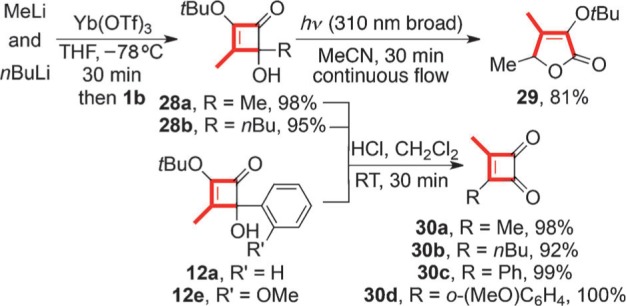
Alkylytterbium additions to 1 b for the synthesis of 5*H*-furanones and diorganylcyclobutenediones.

For completeness, the addition of phenylalkynylytterbium triflate to **1 b** was examined (Scheme 7). The reaction proved sluggish (9 h at −78 °C) in comparison with other additions and regioselectivity was attenuated, with the formation of cyclobutenones **34** and **33** in 69 % and 15 % yield, respectively. In contrast, the addition of the organolithium **31** to **1 a** gave the cyclobutenone **32** as the sole isolated product. Thermolyses of the adduct **32** and **34** exposed a further anomaly, with the former giving a 5:4 mixture of the quinone **35** and cyclopentenedione (*E*)-**36**, while the a latter gave only the cyclopentenedione (*Z*)-**37**.

**Scheme 7:**
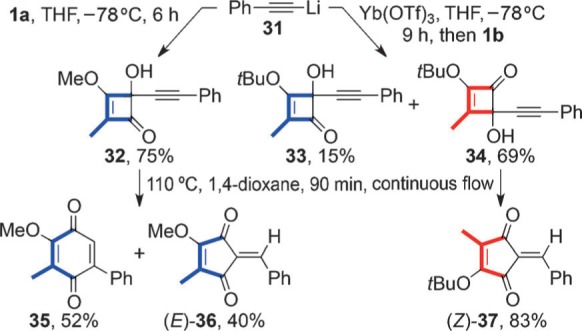
Phenylalkynyllithium and ytterbium additions to 1 a and 1 b, respectively.

To gain insights into the mechanistic course of organoytterbium addition reactions we decided to initiate a computational study of the reaction of PhYb(OTf)_2_ with **1 b**. However, these investigations proved fruitless, with DFT calculations predicting a preference for reaction at the ketonic C1 carbonyl (i.e. Δ*G*^≠^_**A**_<Δ*G*^≠^_**D**_, Figure 1).^10^ The results led us to question the nature of the organometallic reagent. Based on limited literature precedent,^11^ we had assumed that the reaction of PhLi with Yb(OTf)_3_ would first give the ate complex PhYb(OTf)_3_Li, which in turn would collapse to PhYb(OTf)_2_ and LiOTf. When this premise was tested computationally, our calculations indicated that loss of a triflate ligand from the ate complex would cost approximately 25 kcal mol^−1^ at −78 °C! Moreover, when the reactivity of [PhYb(OTf)_3_]^−^ towards **1 b** was examined, the predicted outcome mirrored our experimental findings by revealing a kinetic preference for phenyl addition to the C2 carbonyl (i.e. Δ*G*^≠^_**H**_<Δ*G*^≠^_**E**_, Figure  1).

**Figure 1:**
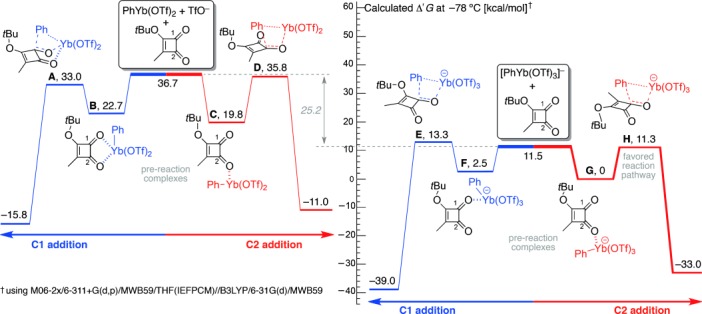
Calculated free energy barriers for C1 and C2 addition in the reactions of 1 b with PhYb(OTf)_2_ and [PhYb(OTf)_3_]^−^ in THF.

The modeling also exposed some seminal facets of organoytterbium reactivity (Figure 1). In particular, the pre-reaction complexes of [PhYb(OTf)_3_]^−^⋅**1 b** (**F** and **G**) each showed single ytterbium–carbonyl interactions leading to trapezoidal transition states. The strength of those interactions was a key factor in determining the regiochemical course of addition, with Δ*G*_**G**_ less than Δ*G*_**F**_ by about 2.5 kcal mol^−1^. Additionally, steric interactions in the addition phase play an important role, as evidenced by 1) the need to rotate the *t*Bu ether during C1 addition (**F**→**E**, Figure 1) at a cost of about 2.6 kcal mol^−1^ 12 and 2) the low selectivity observed for addition of [PhYb(OTf)_3_]^−^ to **1 a** compared to that for **1 b** (Scheme 3).

Finally, to demonstrate the method’s potential in terpenoid synthesis we decided to target mansonone B, a natural product whose identity has yet to be determined with rigor but seemed likely to be as depicted in **43**.^13^ Our synthesis began with (−)-menthone (**39**; Scheme 8), which was readily transformed into the required vinyllithium reagent **41** by means of a Shapiro reaction.^14^ Transmetallation with ytterbium triflate, and subsequent addition of the resulting vinylytterbium intermediate to **1 b**, gave a 7:3 diastereomeric mixture of the C2 adduct **42** in 94 % yield. Thermolysis of that mixture under continuous flow at 110 °C with concomitant aerial oxidation, gave the quinone **44**, from which our target **43** was readily derived. Pleasingly, the physical and spectral characteristics displayed by our synthetic sample (−)-**43** matched those reported for mansonone B in its isolation paper,13a thus confirming the regiochemical and relative stereochemical identity of the natural product. Its absolute configuration remains uncertain because of a lack of available optical rotation data for comparison.

**Scheme 8:**
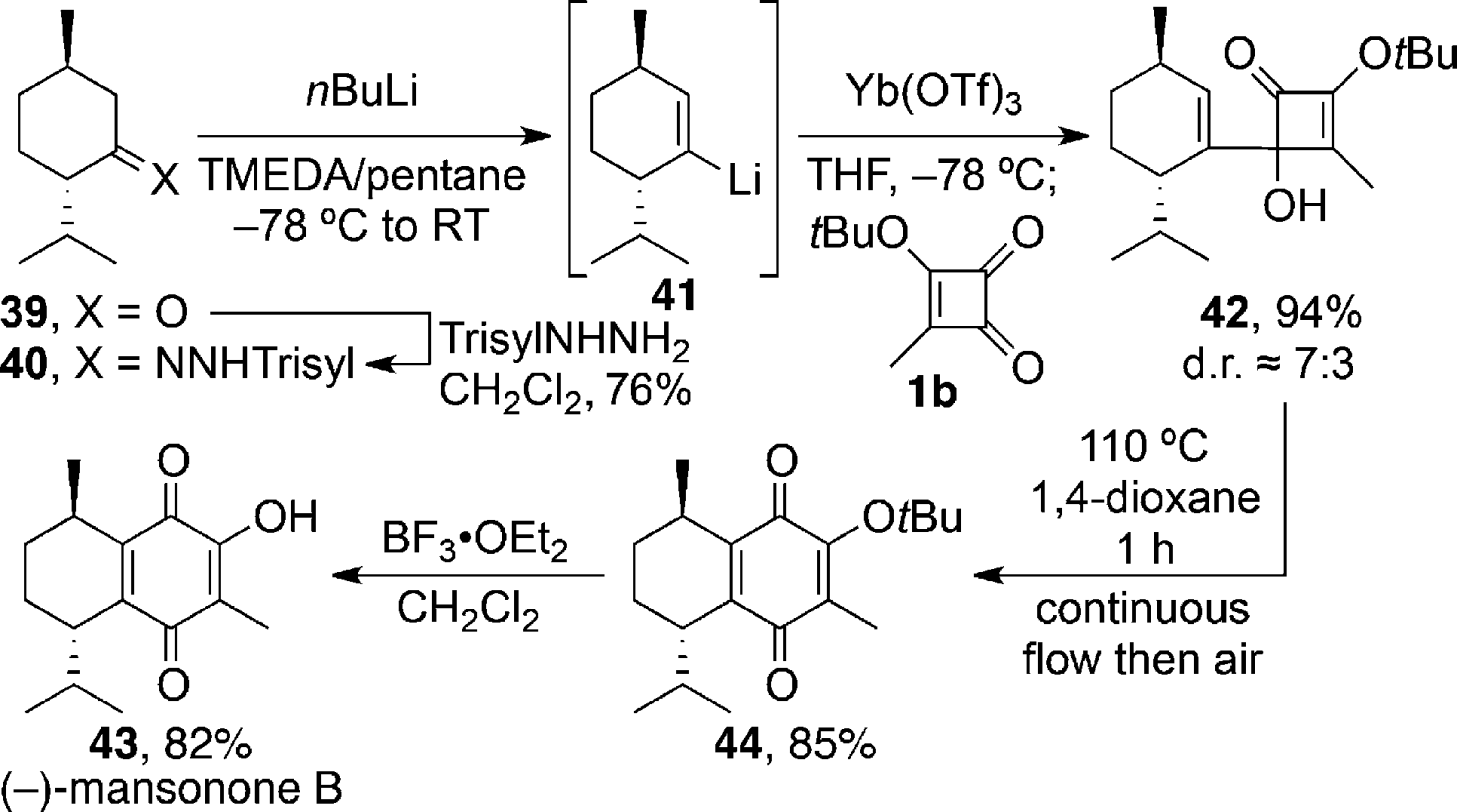
First total synthesis of (−)-mansonone B. TMEDA= *N*,*N*,*N*′,*N*′-tetramethylethylenediamine, Trisyl=2,4,6-triisopropylphenylsulfonyl.

In conclusion, we have shown that organoytterbium additions to **1 b** are easy to effect, proceed in excellent yield, and follow a complementary regiochemical course to related organolithium additions to **1 a**. Alkyl, aryl, hetaryl, vinyl, and alkynyl ytterbium reagents participate in the reaction, greatly extending the utility of cyclobutenone rearrangements by providing a predictable means of introducing this isoprene unit in a regioselective manner. High-level computational studies have provided seminal insights into the nature of organoytterbium intermediates and their reactivity. Of particular note is the realization that reactions proceed via ytterbium ate complexes.

In memory of Margaret Harrowven

## Supporting Information

As a service to our authors and readers, this journal provides supporting information supplied by the authors. Such materials are peer reviewed and may be re-organized for online delivery, but are not copy-edited or typeset. Technical support issues arising from supporting information (other than missing files) should be addressed to the authors.

miscellaneous_information
