# Supplementary material for: Organoytterbium Ate Complexes Extend the Value of Cyclobutenediones as Isoprene Equivalents
Source: Angew Chem Int Ed Engl. 2013 Oct 23;52(49):13076–9. doi: 10.1002/anie.201307193 (PMC4285119; doi:10.1002/anie.201307193)
Supplement: Supplementary file 1 — miscellaneous_information [file anie0052-13076-sd1.pdf]

Angewandte Chemie
Eine Zeitschrift der Gesellschaft Deutscher Chemiker

Supporting Information

© Wiley-VCH 2013

69451 Weinheim, Germany

Organoytterbium Ate Complexes Extend the Value of Cyclobutenediones as Isoprene Equivalents**

*Emma Packard, David D. Pascoe, Jacques Maddaluno, Théo P. Gonçalves, and David C. Harrowven**

anie_201307193_sm_miscellaneous_information.pdf

ANIMATION OF THE LOWEST ENERGY PATHWAY

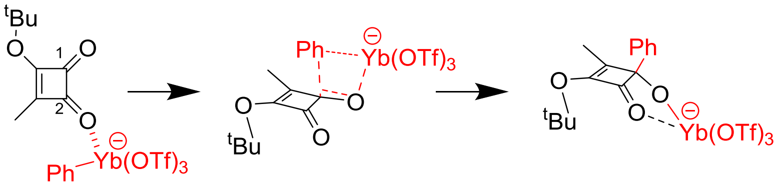

G H C-2 addition product

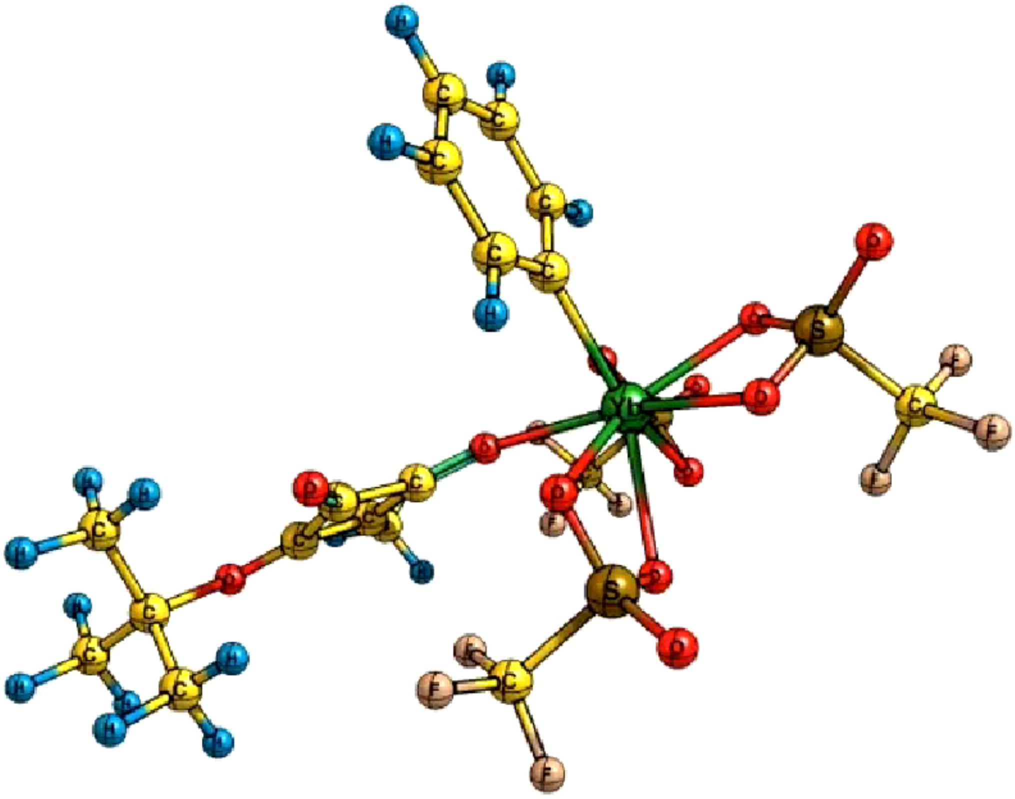

Animation base on IRC and PES at B3LYP/6-31G(d)/WMB59
requires Adobe Reader version 10 or higher

Table of Contents

Further Examples and Observations S7

General Experimental S8

Drying and Storage of Ytterbium(III) Triflate S9

Experimental Procedures S9

Additions of Organoytterbium Reagents to Cyclobutenediones **1a** and **1b** S9

4-Hydroxy-2-methoxy-3-methyl-4-phenylcyclobut-2-enone (**11a**) and 4-Hydroxy-3-methoxy-2-methyl-4-phenylcyclobut-2-enone (**2a**) S9
2-(*tert*-Butoxy)-4-hydroxy-3-methyl-4-phenylcyclobut-2-enone (**12a**) S10
2-(*tert*-Butoxy)-4-hydroxy-3-methyl-4-(p-tolyl)cyclobut-2-enone (**12b**) S10
2-(*tert*-Butoxy)-4-hydroxy-4-(4-methoxyphenyl)-3-methylcyclobut-2-enone (**12c**) S11
2-(*tert*-Butoxy)-4-hydroxy-3-methyl-4-(4-(trifluoromethyl)phenyl)cyclobut-2-enone (**12d**) S11
2-(*tert*-Butoxy)-4-hydroxy-3-methyl-4-(2-methoxyphenyl)cyclobut-2-enone (**12e**) S12
2-(*tert*-Butoxy)-4-hydroxy-3-methyl-4-(pyridin-2-yl)cyclobut-2-enone (**12f**) S12
2-(*tert*-Butoxy)-4-hydroxy-3-methyl-4-(m-tolyl)cyclobut-2-enone (**12g**) S12
2-(*tert*-Butoxy)-4-hydroxy-3-methyl-4-(3-(trifluoromethyl)phenyl)cyclobut-2-enone (**12h**) S13
2-(*tert*-Butoxy)-4-hydroxy-3-methyl-4-(propen-2-yl)cyclobut-2-enone (**20**) S13
2-(*tert*-Butoxy)-4-hydroxy-3-methyl-4-(1-phenylvinyl)cyclobut-2-enone (**21a**) S14
2-(*tert*-Butoxy)-4-hydroxy-3-methyl-4-vinylcyclobut-2-enone (**22**) S14
2-(*tert*-Butoxy)-4-hydroxy-3,4-dimethylcyclobut-2-enone (**28a**) S15
2-(*tert*-Butoxy)-4-butyl-4-hydroxy-3-methylcyclobut-2-enone (**28b**) S15
2-(*tert*-Butoxy)-4-hydroxy-3-methyl-4-(phenylethynyl)cyclobut-2-enone (**34**) and 3-(*tert*-butoxy)-4-hydroxy-2-methyl-4-(phenylethynyl)cyclobut-2-enone (**33**) S15

Comparative Additions of Organolithium Reagents to Cyclobutenediones **1** S16

4-Hydroxy-3-methoxy-2-methyl-4-phenylcyclobut-2-enone (**2a**) S16
4-Hydroxy-3-methoxy-2-methyl-4-(p-tolyl)cyclobut-2-enone (**2b**) S16
4-Hydroxy-3-methoxy-4-(4-methoxyphenyl)-2-methylcyclobut-2-enone (**2c**) S17
4-Hydroxy-3-methoxy-2-methyl-4-(4-(trifluoromethyl)phenyl)cyclobut-2-enone (**2d**) S17
3-Methoxy-4-methyl-[1,1'-biphenyl]-2,5-diol (**27**) and 4-hydroxy-2-methoxy-3-methyl-4-(1-phenylvinyl)cyclobut-2-enone (**21b**) S17
4-Hydroxy-3-methoxy-2-methyl-4-(phenylethynyl)cyclobut-2-enone (**32**) S18

Additional experiments as noted on page S7 and in reference 7 of the article: S18

4-Hydroxy-3-methoxy-2,4-dimethylcyclobut-2-enone (**50**) S18
4-Hydroxy-3-methoxy-4-(2-methoxyphenyl)-2-methylcyclobut-2-enone (**2e**) S19
3-(*tert*-Butoxy)-4-hydroxy-4-(4-methoxyphenyl)-2-methylcyclobut-2-enone (**12c**) and 2-(*tert*-butoxy)-4-hydroxy-4-(4-methoxyphenyl)-3-methylcyclobut-2-enone (**10c**) S19

Comparative Addition of an Organocerium Reagent to Cyclobutenedione **1b** S20

2-(*tert*-Butoxy)-4-hydroxy-3-methyl-4-phenylcyclobut-2-enone (**12a**) and 3-(*tert*-butoxy)-4-hydroxy-2-methyl-4-phenylcyclobut-2-enone (**10a**) S20

Thermochemical Rearrangements S20

2-Methoxy-3-methylnaphthalene-1,4-dione (**15a**) S20
2-Methoxy-3,6-dimethylnaphthalene-1,4-dione (**15b**) S21
2,6-Dimethoxy-3-methylnaphthalene-1,4-dione (**15c**) S21
2-Methoxy-3-methyl-6-(trifluoromethyl)naphthalene-1,4-dione (**15d**) S21
2-(*tert*-Butoxy)-3-methylnaphthalene-1,4-dione (**16a**) S22
3-(*tert*-Butoxy)-2,6-dimethylnaphthalene-1,4-dione (**16b**) S22

3-(*tert*-Butoxy)-6-methoxy-2-methylnaphthalene-1,4-dione (**16c**) S22
2-(*tert*-Butoxy)-3-methyl-6-(trifluoromethyl)naphthalene-1,4-dione (**16d**) S23
2-(*tert*-Butoxy)-5-methoxy-3-methylnaphthalene-1,4-dione (**16e**) S23
3-(*tert*-Butoxy)-1-hydroxy-2-methyl-4*H*-quinolizin-4-one (**16f**) S23
2-(*tert*-Butoxy)-3,6-dimethylnaphthalene-1,4-dione (**16g**) and 2-(*tert*-butoxy)-3,8-dimethylnaphthalene-1,4-dione (**16i**) S24
2-(*tert*-Butoxy)-3-methyl-6-(trifluoromethyl)naphthalene-1,4-dione (**16h**) S24
2-(*tert*-Butoxy)-3,5-dimethylbenzene-1,4-diol (**23**) and 2-(*tert*-butoxy)-3,5-dimethylcyclohexa-2,5-diene-1,4-dione (**58**) S25
4-(*tert*-Butoxy)-3-methyl-[1,1'-biphenyl]-2,5-diol (**24**) S25
2-(*tert*-Butoxy)-3-methylbenzene-1,4-diol (**25**) S25
4-Methoxy-3-methyl-[1,1'-biphenyl]-2,5-dione (**35**) and (*E*)-2-benzylidene-4-methoxy-5-methylcyclopent-4-ene-1,3-dione (**36**) S26
(*Z*)-2-Benzylidene-4-(*tert*-butoxy)-5-methylcyclopent-4-ene-1,3-dione (**37**) S26

Additional experiments as noted on page S7 and in reference 7 of the article: S26

(*E*)-2-Benzylidene-4-(*tert*-butoxy)-5-methylcyclopent-4-ene-1,3-dione (**53**) and 4-(*tert*-butoxy)-3-methyl-[1,1'-biphenyl]-2,5-dione (**54**) S26
3-Methoxy-4-methyl-[1,1'-biphenyl]-2,5-diol (**51**) and 3-methoxy-4-methyl-[1,1'-biphenyl]-2,5-dione (**52**) S27

Photochemical Rearrangement S27

3-(*tert*-Butoxy)-4,5-dimethylfuran-2(5*H*)-one (**29**) S27

Elimination to Cyclobutenediones S28

3,4-Dimethylcyclobut-3-ene-1,2-dione (**30a**) S28
3-Butyl-4-methylcyclobut-3-ene-1,2-dione (**30b**) S28
3-Methyl-4-phenylcyclobut-3-ene-1,2-dione (**30c**) S28
3-Methyl-4-(2-methoxyphenyl)cyclobut-3-ene-1,2-dione (**30d**) S29

Total Synthesis of Mansonone B S29

(*E*)-2,4,6-Triisopropyl-N'-((2*S*,5*R*)-2-isopropyl-5-methylcyclohexylidene)benzenesulfonohydra-zone (**40**) S29
2-(*tert*-Butoxy)-4-hydroxy-4-((3*R*,6*S*)-6-isopropyl-3-methylcyclohex-1-en-1-yl)-3-methylcyclobut-2-enone (**42**) S29
(5*S*,8*R*)-2-(*tert*-Butoxy)-5-isopropyl-3,8-dimethyl-5,6,7,8-tetrahydronaphthal-ene-1,4-dione (**44**) S30
(5*S*,8*R*)-2-Hydroxy-5-isopropyl-3,8-dimethyl-5,6,7,8-tetrahydronaphthalene-1,4-dione, (–)-Mansonone B (**43**) S30

^{1}H AND ^{13}C NMR SPECTRA S32

4-Hydroxy-2-methoxy-3-methyl-4-phenylcyclobut-2-enone (**11a**) S32
4-Hydroxy-3-methoxy-2-methyl-4-phenylcyclobut-2-enone (**2a**) S33
2-(*tert*-Butoxy)-4-hydroxy-3-methyl-4-phenylcyclobut-2-enone (**12a**) S34
2-(*tert*-Butoxy)-4-hydroxy-3-methyl-4-(*p*-tolyl)cyclobut-2-enone (**12b**) S35
2-(*tert*-Butoxy)-4-hydroxy-4-(4-methoxyphenyl)-3-methylcyclobut-2-enone (**12c**) S36
2-(*tert*-Butoxy)-4-hydroxy-3-methyl-4-(4-(trifluoromethyl)phenyl)cyclobut-2-enone (**12d**) S37
2-(*tert*-Butoxy)-4-hydroxy-3-methyl-4-(2-methoxyphenyl)cyclobut-2-enone (**12e**) S38
2-(*tert*-Butoxy)-4-hydroxy-3-methyl-4-(pyridin-2-yl)cyclobut-2-enone (**12f**) S39
2-(*tert*-Butoxy)-4-hydroxy-3-methyl-4-(m-tolyl)cyclobut-2-enone (**12g**) S40
2-(*tert*-Butoxy)-4-hydroxy-3-methyl-4-(3-(trifluoromethyl)phenyl)cyclobut-2-enone (**12h**) S41
2-(*tert*-Butoxy)-4-hydroxy-3-methyl-4-(propen-2-yl)cyclobut-2-enone (**20**) S42
2-(*tert*-Butoxy)-4-hydroxy-3-methyl-4-(1-phenylvinyl)cyclobut-2-enone (**21a**) S43
2-(*tert*-Butoxy)-4-hydroxy-3-methyl-4-vinylcyclobut-2-enone (**22**) S44
2-(*tert*-Butoxy)-4-hydroxy-3,4-dimethylcyclobut-2-enone (**28a**) S45
2-(*tert*-Butoxy)-4-butyl-4-hydroxy-3-methylcyclobut-2-enone (**28b**) S46
2-(*tert*-Butoxy)-4-hydroxy-3-methyl-4-(phenylethynyl)cyclobut-2-enone (**34**) S47
3-(*tert*-butoxy)-4-hydroxy-2-methyl-4-(phenylethynyl)cyclobut-2-enone (**33**) S48
4-Hydroxy-3-methoxy-2-methyl-4-(*p*-tolyl)cyclobut-2-enone (**2b**) S49
4-Hydroxy-3-methoxy-4-(4-methoxyphenyl)-2-methylcyclobut-2-enone (**2c**) S50

4-Hydroxy-3-methoxy-2-methyl-4-(4-(trifluoromethyl)phenyl)cyclobut-2-enone (**2d**) S51
3-Methoxy-4-methyl-[1,1'-biphenyl]-2,5-diol (**27**) S52
4-Hydroxy-2-methoxy-3-methyl-4-(1-phenylvinyl)cyclobut-2-enone (**21b**) S53
4-Hydroxy-3-methoxy-2-methyl-4-(phenylethynyl)cyclobut-2-enone (**32**) S54
4-Hydroxy-3-methoxy-2,4-dimethylcyclobut-2-enone (**50**) S55
4-Hydroxy-3-methoxy-4-(2-methoxyphenyl)-2-methylcyclobut-2-enone (**2e**) S56
2-(*tert*-butoxy)-4-hydroxy-4-(4-methoxyphenyl)-3-methylcyclobut-2-enone (**10c**) S57
3-(*tert*-butoxy)-4-hydroxy-2-methyl-4-phenylcyclobut-2-enone (**10a**) S58
2-Methoxy-3-methylnaphthalene-1,4-dione (**15a**) S59
2-Methoxy-3,6-dimethylnaphthalene-1,4-dione (**15b**) S60
2,6-Dimethoxy-3-methylnaphthalene-1,4-dione (**15c**) S61
2-Methoxy-3-methyl-6-(trifluoromethyl)naphthalene-1,4-dione (**15d**) S62
2-(*tert*-Butoxy)-3-methylnaphthalene-1,4-dione (**16a**) S63
3-(*tert*-Butoxy)-2,6-dimethylnaphthalene-1,4-dione (**16b**) S64
3-(*tert*-Butoxy)-6-methoxy-2-methylnaphthalene-1,4-dione (**16c**) S65
2-(*tert*-Butoxy)-3-methyl-6-(trifluoromethyl)naphthalene-1,4-dione (**16d**) S66
2-(*tert*-Butoxy)-5-methoxy-3-methylnaphthalene-1,4-dione (**16e**) S67
3-(*tert*-Butoxy)-1-hydroxy-2-methyl-4H-quinolizin-4-one (**16f**) S68
2-(*tert*-Butoxy)-3,6-dimethylnaphthalene-1,4-dione (**16g**) and 2-(*tert*-butoxy)-3,8-dimethylnaphthalene-S1,4-dione (**16i**) S69
2-(*tert*-Butoxy)-3-methyl-6-(trifluoromethyl)naphthalene-1,4-dione (**16h**) S70
2-(*tert*-Butoxy)-3,5-dimethylbenzene-1,4-diol (**23**) and 2-(*tert*-butoxy)-3,5-dimethylcyclohexa-2,5-diene-1,4-dione (**55**) S71
4-(*tert*-Butoxy)-3-methyl-[1,1'-biphenyl]-2,5-diol (**24**) S72
2-(*tert*-Butoxy)-3-methylbenzene-1,4-diol (**25**) S73
4-Methoxy-3-methyl-[1,1'-biphenyl]-2,5-dione (**35**) S74
(*E*)-2-benzylidene-4-methoxy-5-methylcyclopent-4-ene-1,3-dione (**36**) S75
(*Z*)-2-Benzylidene-4-(*tert*-butoxy)-5-methylcyclopent-4-ene-1,3-dione (**37**) S76
(*E*)-2-Benzylidene-4-(*tert*-butoxy)-5-methylcyclopent-4-ene-1,3-dione (**53**) and 4-(*tert*-butoxy)-3-methyl-[1,1'-biphenyl]-2,5-dione (**54**) S77
3-methoxy-4-methyl-[1,1'-biphenyl]-2,5-dione (**52**) S78
3-Methoxy-4-methyl-[1,1'-biphenyl]-2,5-diol (**51**) S79
3-(*tert*-Butoxy)-4,5-dimethylfuran-2(5*H*)-one (**29**) S80
3,4-Dimethylcyclobut-3-ene-1,2-dione (**30a**) S81
3-Butyl-4-methylcyclobut-3-ene-1,2-dione (**30b**) S82
3-Methyl-4-phenylcyclobut-3-ene-1,2-dione (**30c**) S83
3-Methyl-4-(2-methoxyphenyl)cyclobut-3-ene-1,2-dione (**30d**) S84
(*E*)-2,4,6-Triisopropyl-*N*'-((2*S*,5*R*)-2-isopropyl-5-methylcyclohexylidene)benzenesulfonohydra-zone (**40**) S85
2-(*tert*-Butoxy)-4-hydroxy-4-((3*R*,6*S*)-6-isopropyl-3-methylcyclohex-1-en-1-yl)-3-methylcyclobut-2-enone (**42**) S86
(5*S*,8*R*)-2-(*tert*-Butoxy)-5-isopropyl-3,8-dimethyl-5,6,7,8-tetrahydronaphthal-ene-1,4-dione (**44**) S87
(5*S*,8*R*)-2-Hydroxy-5-isopropyl-3,8-dimethyl-5,6,7,8-tetrahydronaphthalene-1,4-dione, (–)-Mansonone B (**43**) S88

COMPUTATIONAL ANALYSIS S89

Summary of Calculated Reaction Pathways S90

Energies and Cartesian Coordinates S91

Cyclobutenedione [**1b**] S91
Transition state for interconversion of conformers 1 & 2 ..... S91
$Yb(OTf)_3$ S92
$[PhYb(OTf)_3]^-$ S93
$PhYb[OTf]_2$ S94
Triflate S95
Phenyl anion S95
Pre-reaction complex 1b•$PhYb(OTf)_2$:- double chelation S96
Pre-reaction complex 1b•$PhYb(OTf)_2$:- chelation to C1 carbonyl only S100
Pre-reaction complex 1b•$PhYb(OTf)_2$:- chelation to C2 carbonyl only S100
Trapezoidal Transition State for $PhYb(OTf)_2$ addition to C-1 carbonyl of **1b** S101
Butterfly Transition State for $PhYb(OTf)_2$ addition to C-1 carbonyl of **1b** S104

Trapezoidal Transition State for $PhYb(OTf)_2$ addition to C-2 carbonyl of **1b** S111
Butterfly Transition State for $PhYb(OTf)_2$ addition to C-2 carbonyl of **1b** S114
C-1 addition product from $PhYb(OTf)_2$ + **1b** S119
C-2 addition product from $PhYb(OTf)_2$ + **1b** S121
Pre-reaction complex 1b•$[PhYb(OTf)_3]^-$:- chelation to C1 carbonyl only S124
Pre-reaction complex 1b•$[PhYb(OTf)_3]^-$:- chelation to C2 carbonyl only S132
Trapezoidal Transition State for $[PhYb(OTf)_3]^-$ addition to C-1 carbonyl of **1b** S141
Trapezoidal Transition State for $[PhYb(OTf)_3]^-$ addition to C-2 carbonyl of **1b** S145
C-1 addition product from $[PhYb(OTf)_3]^-$ + **1b** S150
C-2 addition product from $[PhYb(OTf)_3]^-$ + **1b** S154

References and Notes for Supporting Information S159

Continuation of Reference 1 and 5 from Main Article S159

Further Examples and Observations

As noted in the manuscript, various *ortho*-, *meta*- and hetero-arylytterbium reagents were also prepared and reacted with cyclobutenedione **1b** (Schemes A and B). In each case C-2 adducts were formed in excellent yield.

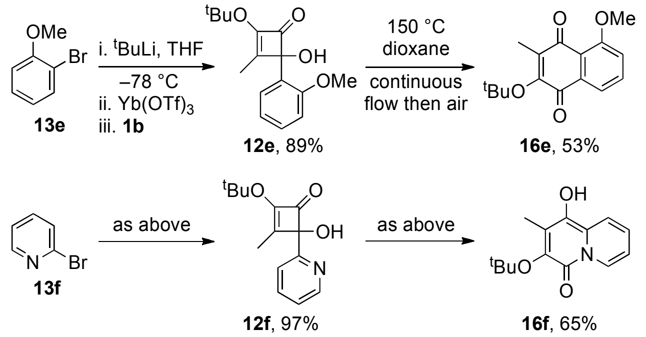

Scheme A. Examples of *ortho*- and hetero-arylytterbium additions to **1b** and thermal rearrangement of the resulting C-2 adducts.

The thermal rearrangements of the *meta*-substituted arylcyclobutenones (Scheme B) deserve further comment to explain why the tolyl derivative **12g** gave a 1 : 1 mixture of benzoquinones **16g** and **16i** while the analogous α,α,α-trifluorotolyl derivative **12h** displayed exquisite regioselectivity and gave only benzoquinone **16h**. The dichotomy is instructive as it provides a further illustration of the importance of inductive effects on the course of arylcyclobutenone rearrangements. In a detailed study of the reaction,[1] we showed that its rate-determining step involves the electrocyclic ring closure of a vinylketene intermediate (*e.g.* **45**, Scheme C) while the absolute rate is largely dictated by the inductive effect of substituents on the arene [substituents exhibiting a –I effect slow the reaction considerably compared to the parent system (X = H)].

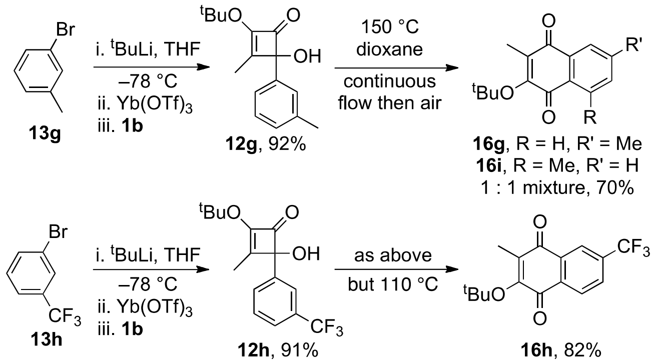

Scheme B. Examples of *meta*-substituted arylytterbium additions to **1b** and thermal rearrangement of the resulting C-2 adducts.

Here we see an important manifestation of that effect in respect of *meta*-substituted arenes. Since the inductive influence drops off rapidly through extended σ-frameworks, the rate of cyclisation via rotamer **45B** will be influenced to a far greater extent than the rate of cyclisation via rotamer **45A**. Thus, substrates bearing an inductive electron-withdrawing group (–I) as a *meta*-substituent will display a strong preference for cyclisation to **46** via rotamer **45A**. By contrast, substrates baring an inductive electron-releasing group (+I) on the *meta*-carbon will be more inclined to undergo cyclisation to **47** via rotamer **45B**.

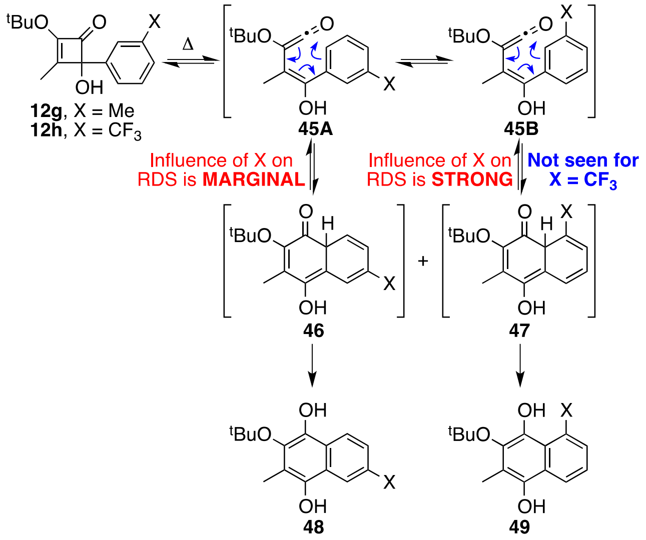

Scheme C. Understanding regioselectivity in the thermal rearrangement of *meta*-substituted arylcyclobutenones.

The additional examples of organolithium additions to **1a** and **1b** noted in the manuscript are illustrated in Scheme D while the additional thermochemical rearrangements are illustrated in Scheme E. Accounts for these additional experiments are included in the relevant sections of the Experimental.

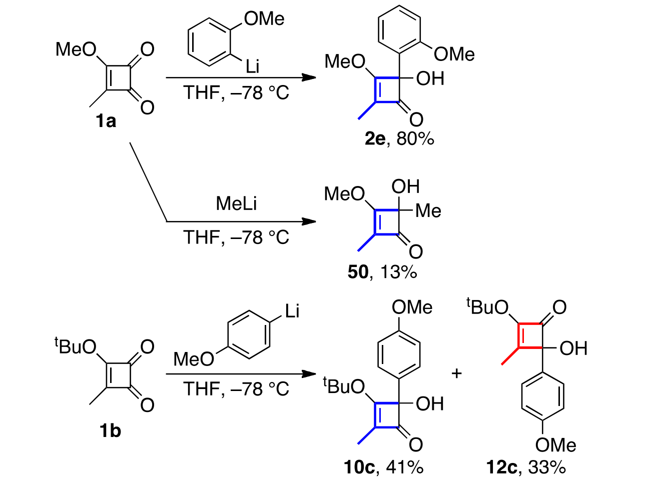

Scheme D. Further examples of RLi additions to **1**.

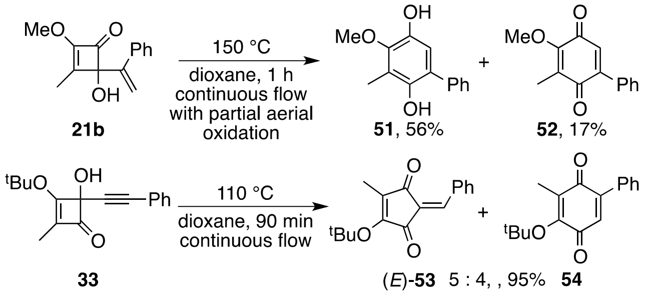

Scheme E. Additional thermochemical rearrangements.

GENERAL EXPERIMENTAL

Melting points were recorded on an electrothermal digital melting point apparatus and are uncorrected.

Ultraviolet-Visible measurements were recorded via a silica cuvette (quartz Suprasill 300 with a path length of 1 mm) on an Ocean Optics DH-2000-BAL at room temperature using Spectral suits.

Infrared Spectra were recorded neat as a thin film or a solid compression using the Golden Gate ATR method on a Nicolet 380 FT-IR. Absorption maxima (υ max) are expressed as s, strong; m, medium; w, weak; br, broad and are quoted in wavenumbers (cm^{-1}).

^{1}H and ^{13}C NMR Spectra were recorded on a Bruker AV300 (300/75 MHz) or Bruker DPX400 (400/100 MHz) spectrometer at 298 K. Experiments were run in deuterated chloroform ($CDCl_3$) unless otherwise stated, with this supplied by Goss Scientific or Sigma Aldrich and stored over dried K_2CO_3 to neutralise trace acidity. Chemical shifts are reported in parts per million (ppm) downfield of tetramethylsilane with residual solvent as the internal standard. Assignments were made on the basis of chemical shifts, coupling constants and DEPT experiments and by comparison with literature values where available. Multiplicities are reported as s (singlet), d (doublet), t (triplet), q (quartet), quin (quintet), m (multiplet) and may also be described as br (broad) or app (apparent). Coupling constants (*J*) are given in Hz and are rounded to the nearest 0.1 Hz.

Mass spectra: Electrospray mass spectra were recorded using a Waters ZMD single quadrupole mass spectrometer with a 2700 autosampler and a 600 pump with acetonitrile as the eluent. Electron Impact mass spectra were measured on a thermoquest trace single quadrupole GC-MS at 70 eV with acetonitrile as the eluent. The EI method used was Fast EI-MS non-polar with the details and specifications as follows: 40 °C for 3 min then ramped at 40 °C per min to a final temperature of 320 °C, held at final temperature for 6 min. The carrier gas was He which was pumped at 1 $mLmin^{-1}$ constant flow. 70 eV EI-MS were run with a trap current of 150 µA. The source temperature was 200 °C and the mass range recorded was $^{m}/_{z}$ 40–500 with 2 scans/second. The column used was a Zebron ZB5-MS 30 mm x 0.25 mm x 0.25 µm film thickness. High resolution mass spectra were recorded on either a Bruker Apex III FT-ICR mass spectrometer equipped with a 4.7 T actively shielded superconducting magnet and Apollo ESI ion source or a Bruker maXis ESI-ToF coupled to a Dionex Ultimate 3000 HPLC and Apollo ESI ion source. High resolution mass spectra were recorded by Dr. G. J. Langley or J. Herniman (Chemistry, University of Southampton) and are reported to four decimal places.

Chromatography: Thin layer chromatography was performed on Merck DC-Alufolien 60 F254 0.2 mm aluminium backed plates. Product spots were visualised by shortwave UV and one of the following stains: *p*-anisaldehyde, CAM (Hannessian's stain) or 10% PMA in EtOH. Flash column chromatography was carried out under slight positive pressure on silica gel (200–400 mesh) using the solvent system stated in the experimental account.

Optical Rotation: Optical rotations were recorded on an Optical Activity Polar 2001 polarimeter at 589 nm.

Flow Chemistry: Thermal rearrangements under continuous flow were conducted in stainless-steel tubing (internal diameter of 1 mm, capacity of 10 mL) using a Vapourtec R4/R2+ instrument.

Solvents and Reagents: Commercial reagents and the solvents used in work-up procedures were used as supplied. THF and pentane were dried over, and distilled from, sodium using sodium benzophenone ketyl as an indicator. Acetonitrile was dried over, and distilled from, sodium. The cyclobutenediones **1a** and **1b** were each prepared in 2 steps from squaric acid using the literature methods indicated below (Scheme F).[2,3,4]

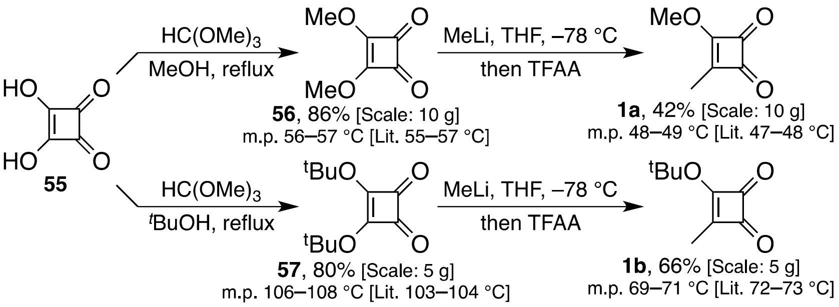

Scheme F. Preparation of cyclobutenediones **1a** and **1b**.

DRYING AND STORAGE OF YTTERBIUM(III) TRIFLATE

$Yb(OTf)_3.xH_2O$ (25 g) [from Sigma-Aldrich: ytterbium(III) trifluoromethanesulfonate hydrate, 25–28% Yb] was placed in a drying tube then heated at 240 °C in a Kugelrohr oven for 4 days under vacuum (0.1–0.5 mmHg). After cooling to RT under vacuum, the oven was filled with Ar and the resulting fine white powder was transferred to an Ar filled glove bag. The solid was weighed into dried sample vials, which were then sealed and stored over P_2O_5 in a desiccator within an Ar filled glove bag until needed.
The drying phase could also be effected by placing the commercial hydrate in a Schlenk flask then heating it under vacuum at 240 °C for 4 days using a DrySyn block with continuous stirring.

EXPERIMENTAL PROCEDURES

ADDITIONS OF ORGANOYTTERBIUM REAGENTS TO CYCLOBUTENEDIONES 1A AND 1B

*4-Hydroxy-2-methoxy-3-methyl-4-phenylcyclobut-2-enone (**11a**) and*
*4-Hydroxy-3-methoxy-2-methyl-4-phenylcyclobut-2-enone (**2a**).*

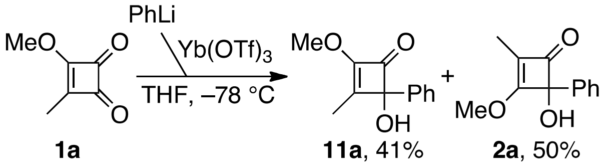

To a solution of $Yb(OTf)_3$ (350 mg, 0.56 mmol) in THF (3 mL) at –78 °C was added PhLi (0.94 M in dibutyl ether, 600 µL, 0.56 mmol) dropwise over 3 min. Over the course of addition the colourless solution turned from pale pink to deep orange to bright yellow. After 30 min a solution of cyclobutenedione **1a** (70 mg, 0.56 mmol) in THF (2.5 mL) was added dropwise over 5 min and the colour remained bright yellow. After 1 h the reaction mixture was quenched with sat. $NaHCO_3$ (10 mL) then warmed to RT and diluted with ether (15 mL). The aqueous phase was separated and extracted with ether (10 mL). The organic phases were combined, washed with brine (20 mL), dried over $MgSO_4$ and concentrated under reduced pressure to give a pale yellow oil (127 mg). Purification by flash column chromatography (2.5–10% ethyl acetate/petroleum ether) afforded firstly cyclobutenone **11a** (45 mg, 0.22 mmol, 41%) as a pale yellow oil; ^{1}H NMR (300 MHz, $CDCl_3$): δ = 7.47–7.30 (5 H, m, Ar**H**), 4.05 (3 H, s, OC**H₃**), 3.02 (1 H, br s, O**H**), 2.06 (3 H, s, C**H₃**) ppm; ^{13}C NMR (100 MHz, $CDCl_3$): δ = 187.8 (**C**=O), 155.3 (**C**), 153.9 (**C**), 137.8 (**C**), 128.6 (2 x **C**H), 128.1 (**C**H), 125.7 (2 x **C**H), 89.9 (**C**), 58.2 (**C**H_3), 9.2 (**C**H_3) ppm; IR: ν 3407 (br), 2946 (w), 2922 (w), 2852 (w), 2362 (w), 2333 (w), 1757 (s), 1631 (s), 1447 (m), 1382 (m), 1328 (s), 1206 (w), 1169 (w), 1051 (s), 846 (w), 699 (m), 552 (w), 483 (w) cm^{-1}; MS (EI) *m/z* (%): 204 ($[M]^{+\bullet}$, 46%), 202 (100%); HRMS (ES+) found 227.0673 $[M+Na]^+$, $C_{12}H_{12}NaO_3$ requires 227.0679; followed by the cyclobutenone **2a** (55 mg, 0.27 mmol, 50%) as a colourless oil.[5] ^{1}H NMR (400 MHz, $CDCl_3$): δ = 7.40–7.36 (2 H, m, Ar**H**), 7.30–7.18 (3 H, m, Ar**H**), 4.58 (1 H, br s, O**H**), 3.89 (3 H, s, OC**H₃**), 1.67 (3 H, s, C**H₃**) ppm; ^{13}C NMR (100 MHz, $CDCl_3$): δ = 191.3 (**C**=O), 182.6 (**C**), 136.9 (**C**), 128.5 (2 x **C**H), 128.1 (**C**H), 125.6 (2 x **C**H), 124.9 (**C**), 92.2 (**C**), 59.6 (**C**H_3), 6.3 (**C**H_3) ppm; IR: ν 3354 (br), 2954 (w), 2860 (w), 2366 (m), 2341 (w), 1753 (m), 1610 (s), 1451 (m), 1386 (m), 1333 (s),1173 (w), 1034 (w), 993 (m), 924 (w), 887 (w), 863 (w), 728 (w), 699 (m), 671 (w), 622 (w) cm^{-1}; MS (EI) *m/z* (%): 204 ($[M]^{+\bullet}$, 41%), 202 (100%); HRMS (ES+) found 227.0680 $[M+Na]^+$ and 431.1465 $[2M+Na]^+$, $C_{12}H_{12}NaO_3$ requires 227.0679 and $C_{24}H_{24}NaO_6$ requires 431.1465.

*2-(tert-Butoxy)-4-hydroxy-3-methyl-4-phenylcyclobut-2-enone (**12a**)*

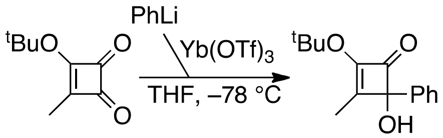

1b **12a**, 92%

To a solution of $Yb(OTf)_3$ (609 mg, 0.98 mmol) in THF (10 mL) at –78 °C was added PhLi (0.91 M in $^nBu_2O$, 1.20 mL, 1.08 mmol) dropwise over 1 min. Over the course of addition the solution turned from colourless to deep pink to deep orange. After 1 h a solution of cyclobutenedione **1b** (156 mg, 0.93 mmol) in THF (10 mL) was added dropwise over 10 min during which time the colour faded to pale yellow. After 1 h sat. $NaHCO_3$ (10 mL) was added then the reaction mixture was allowed to warm to RT and diluted with ether (15 mL). The aqueous phase was separated and extracted with ether (10 mL). The organic phases were combined, washed with brine (20 mL), dried over $MgSO_4$ and concentrated under reduced pressure to give the title compound **12a** (175 mg, 0.95 mmol, 92%) as a pale yellow oil that was used without further purification. ^{1}H NMR (300 MHz, $CDCl_3$): δ = 7.44–7.35 (4H, m, Ar**H**), 7.31 (1H, ddt, *J* = 8.1, 6.5, 1.8 Hz, Ar**H**), 2.70 (1H, br s, O**H**), 2.03 (3H, s, C**H3**), 1.49 (9H, s, C(C**H3**)**3**) ppm; ^{13}C NMR (100 MHz, $CDCl_3$): δ = 186.9 (**C**=O) 157.8 (**C**) 154.5 (**C**) 138.4 (**C**) 128.6 (2 x **C**H), 127.9 (**C**H), 125.7 (2 x **C**H), 88.8 (**C**), 80.4 (**C**), 28.6 (C(**C**H_3)$_3$), 8.8 (**C**H_3) ppm; IR: ν 3418 (br), 2978 (w), 2360 (w), 1755 (s), 1629 (m), 1601 (w), 1449 (w), 1371 (m), 1323 (m), 1260 (w), 1164 (m),1046 (m), 973 (w), 903 (m), 836 (w), 738 (w), 696 (s), 650 (w), 603 (w), 552 (w), 476 (w) cm^{-1}; MS (EI) *m/z* (%): 190 ([M – $Me_2C{=}CH_2$]$^{+\bullet}$, 26%), 161 (100%); HRMS (EI) Found 246.1267 [M]$^{+\bullet}$, $C_{15}H_{18}O_3$ requires 246.1256.

*2-(tert-Butoxy)-4-hydroxy-3-methyl-4-(p-tolyl)cyclobut-2-enone (**12b**)*

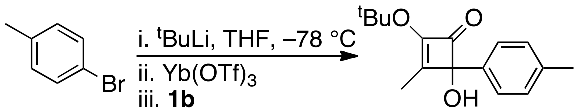

13b **12b**, 96%

To a solution of ^{t}BuLi (1.39 M in pentane, 1.54 mL, 2.14 mmol) in THF (10 mL) at –78 °C was added *p*-bromotoluene (134 µL, 1.07 mmol) dropwise over 1 min. After 40 min the resulting solution was added via cannula to a solution of $Yb(OTf)_3$ (663 mg, 1.07 mmol) in THF (10 mL) at –78 °C over 10 min. Over the course of addition the solution turned from colourless to orange then intensified to become brown/black on complete addition. After 30 min a solution of cyclobutenedione **1b** (157 mg, 0.93 mmol) in THF (5 mL) was added dropwise over 5 min during which time the colour became pale olive green. After a further 30 min sat. $NaHCO_3$ (15 mL) was added, then the reaction mixture was warmed to RT and ether (30 mL) was added. The aqueous phase was separated and extracted with ether (15 mL). The organic phases were combined, washed with brine (30 mL), dried over $MgSO_4$ and concentrated under reduced pressure to give the title compound **12b** (241 mg, 0.93 mmol, 96%) as a white solid that was used without further purification. MP (ether/petroleum ether) 107–108 °C; ^{1}H NMR (400 MHz, $CDCl_3$): δ = 7.31 (2H, d, *J* = 8.2 Hz, 2 x Ar**H**), 7.19 (2H, d, *J* = 8.0 Hz, 2 x Ar**H**), 2.15 (1H, s, O**H**), 2.36 (3H, s, C**H3**), 2.03 (3H, s, C**H3**), 1.49 (9H, s, C(C**H3**)**3**) ppm; ^{13}C NMR (100 MHz, $CDCl_3$): δ = 187.2 (**C**=O), 157.9 (**C**), 154.5 (**C**), 137.8 (**C**), 135.5 (**C**), 129.3 (2 x **C**H), 125.6 (2 x **C**H), 88.8 (**C**), 80.3 (**C**), 28.7 (C(**C**H_3)$_3$), 21.1 (**C**H_3), 8.8 (**C**H_3) ppm; IR: ν 2976 (br), 2360 (m), 2340 (w), 1774 (s), 1757 (s), 1589 (s), 1367 (m), 1337 (m), 1309 (w),1154 (m), 1076 (m), 909 (w), 855 (w), 829 (m), 804 (w), 720 (w), 589 (w), 476 (m), 459 (m) cm^{-1}; MS (EI) *m/z* (%): 186 ([M – ^{t}BuOH]$^{+\bullet}$, 26%), 130 (100%); X-Ray (thermal ellipsoids drawn at 35% probability level, selected hydrogens omitted for clarity).

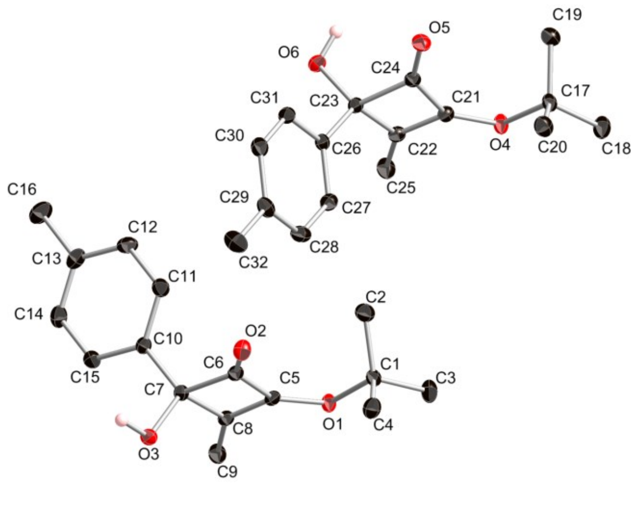

*2-(tert-Butoxy)-4-hydroxy-4-(4-methoxyphenyl)-3-methylcyclobut-2-enone (**12c**)*

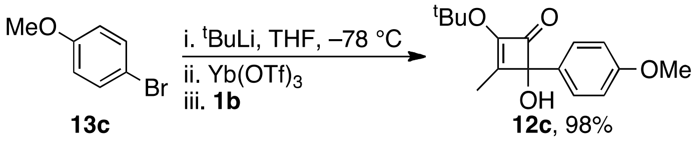

To a solution of ^{t}BuLi (1.39 M in pentane, 1.45 mL, 2.02 mmol) in THF (10 mL) at –78 °C was added *p*-bromoanisole (128 μL, 1.01 mmol) dropwise over 1 min. After 40 min the resulting solution was added via cannula over 10 min to a solution of Yb(OTf)$_3$ (625 mg, 1.01 mmol) in THF (10 mL) at –78 °C. Over the course of addition the solution turned from colourless to orange to dark orange/brown. After 30 min a solution of cyclobutenedione **1b** (151 mg, 0.90 mmol) in THF (5 mL) was added dropwise over 5 min during which time the colour became olive green. After 40 min a 1 : 1 solution of sat. $NaHCO_3$ and sat. Rochelle salt (25 mL) was added then the reaction mixture was allowed to warm to RT and diluted with ether (30 mL). The aqueous phase was separated and extracted with ether (15 mL). The organic phases were combined, washed with brine (30 mL), dried over $MgSO_4$ and concentrated under reduced pressure to give the title compound **12c** (245 mg, 0.89 mmol, 98%) as a pale yellow oil that was used without further purification. ^{1}H NMR (400 MHz, $CDCl_3$): δ = 7.31 (2H, d, *J* = 8.2 Hz, 2 x Ar**H**), 7.19 (2H, d, *J* = 8.0 Hz, 2 x Ar**H**), 3.80 (3H, s, OC**H$_3$**), 2.91 (1H, br s, O**H**), 2.01 (3H, s, C**H$_3$**), 1.47 (9H, s, C(C**H$_3$**)$_3$) ppm; ^{13}C NMR (100 MHz, $CDCl_3$): δ = 187.6 (**C**=O), 159.3 (**C**), 158.4 (**C**), 154.2 (**C**), 130.6 (**C**), 126.9 (2 x **C**H), 113.9 (2 x **C**H), 88.4 (**C**), 80.3 (**C**), 55.2 (**C**H$_3$), 28.6 (C(**C**H$_3$)$_3$), 8.8 (**C**H$_3$) ppm; IR: ν 3097 (w), 3072 (w), 2966 (w), 2938 (w), 2917 (w), 2840 (w), 2361 (m), 2337 (m), 1773 (s), 1757 (s), 1597 (s), 1573 (s), 1507 (s), 1467 (w), 1426 (m), 1348 (s), 1311 (m), 1258 (s), 1181 (s), 1123 (m), 1070 (m) 1009 (m), 841 (s), 722 (w), 600 (m), 498 (m) cm^{-1}; MS (ES$^+$) *m/z* (%): 575 ([2M+Na]$^+$, 100%); HRMS (ES$^+$) Found 299.1256 [M+Na]$^+$ $C_{16}H_{20}NaO_4$ requires 299.1254.

*2-(tert-Butoxy)-4-hydroxy-3-methyl-4-(4-(trifluoromethyl)phenyl)cyclobut-2-enone (**12d**)*

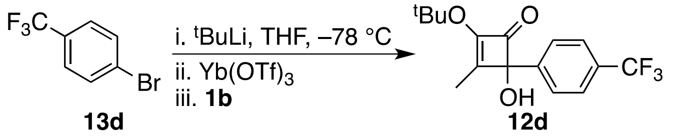

To a solution of ^{t}BuLi (2.06 M in pentane, 941 μL, 1.94 mmol) in THF (10 mL) at –78 °C was added 1-bromo-4-(trifluoromethyl)benzene (136 μL, 0.97 mmol) dropwise over 1 min. After 1 h the resulting solution was added via cannula over 10 min to a solution of Yb(OTf)$_3$ (602 mg, 0.97 mmol) in THF (10 mL) at –78 °C. Over the course of addition the solution turned from colourless to green to dark green/black. After 1 h a solution of cyclobutenedione **1b** (85 mg, 0.51 mmol) in THF (5 mL) was added dropwise over 5 min during which time the colour became pale green. After 1h sat. $NaHCO_3$ was added then the reaction mixture was allowed to warm to RT and diluted with ether (30 mL). The aqueous phase was separated and extracted with ether (15 mL). The organic phases were combined, washed with brine (30 mL), dried over $MgSO_4$ and concentrated under reduced pressure to give the title compound **12d** (150 mg, 0.48 mmol, 94%) as a white crystalline solid that was used without further purification. ^{1}H NMR (300 MHz, $CDCl_3$): δ = 7.61 (2H, d with fine splitting, *J* = 8.2 Hz, 2 x Ar**H**), 7.50 (2H, d with fine splitting, *J* = 8.2 Hz, 2 x Ar**H**), 3.44 (1H, br s, O**H**), 1.99 (3H, s, C**H$_3$**), 1.47 (9H, s, C(C**H$_3$**)$_3$) ppm; ^{13}C NMR (75 MHz, $CDCl_3$): δ = 186.4 (**C**=O), 157.8 (**C**), 154.9 (**C**), 142.5 (**C**, q, *J* = 1.3 Hz), 131.0 (**C**, q, $J_{C\text{-}F}$ = 32.6 Hz), 126.2 (2 x **C**H, s), 125.4 (2 x **C**H, q, $J_{C\text{-}F}$ = 3.9 Hz), 124.0 (**C**F$_3$, q, $J_{C\text{-}F}$ = 272.3 Hz), 88.6 (**C**), 80.8 (**C**), 28.6 (C(**C**H$_3$)$_3$), 8.8 (**C**H$_3$) ppm; ^{19}F NMR (282 MHz, $CDCl_3$) δ = −62.69 (3F, s, C**F$_3$**); IR: ν 3359 (br), 2991 (w), 2966 (w), 2938 (w), 2909 (w), 2362 (w), 2333 (w), 1749 (s), 1623 (s), 1373 (m), 1324 (s), 1263 (w), 1157 (s), 1124 (s), 1055 (m), 1010 (w), 912 (m), 859 (s) cm^{-1}; MS (EI) *m/z* (%): 314 ([M]$^{+\bullet}$, 1%), 57 (100%); HRMS (ES$^+$) Found 337.1027 [M+Na]$^+$ $C_{16}H_{17}F_3NaO_3$ requires 337.0122.

*2-(tert-Butoxy)-4-hydroxy-3-methyl-4-(2-methoxyphenyl)cyclobut-2-enone (**12e**)*

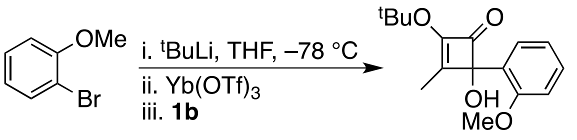

13e **12e**, 89%

To a solution of ^{t}BuLi (1.39 M in pentane, 1.62 mL, 2.25 mmol) in THF (10 mL) at –78 °C was added 2-bromoanisole (143 μL, 1.12 mmol) dropwise over 1 min. After 1 h the resulting solution was added via cannula over 10 min to a solution of $Yb(OTf)_3$ (697 mg, 1.12 mmol) in THF (10 mL) at –78 °C. Over the course of addition the solution turned from colourless to dark brown. After 30 min a solution of cyclobutenedione **1b** (180 mg, 1.07 mmol) in THF (5 mL) was added dropwise over 5 min during which time the colour faded to grey/violet. After a further 40 min a 1 : 1 solution of sat. $NaHCO_3$ and sat. Rochelle salt (25 mL) was added then the reaction mixture was allowed to warm to RT and diluted with ether (30 mL). The aqueous phase was separated and extracted with ether (15 mL). The organic phases were combined, washed with brine (30 mL), dried over $MgSO_4$ and concentrated under reduced pressure to give the title compound **12e** (263 mg, 0.95 mmol, 89%) as a pale yellow solid that was used without further purification. ^{1}H NMR (400 MHz, $CDCl_3$): δ = 7.29 (1H, ddd, *J* = 8.2, 7.6, 1.8 Hz, Ar**H**), 7.21 (1H, dd, *J* = 8.0, 1.8 Hz, Ar**H**), 6.96 (1H, dd, *J* = 8.4, 1.1 Hz, Ar**H**), 6.96 (1H, app td, *J* = 7.6, 1.0 Hz, Ar**H**), 4.60 (1H, br s, O**H**), 3.92 (3H, s, ArOC**H₃**), 2.09 (3H, s, C**H₃**), 1.47 (9H, s, C(C**H₃**)₃) ppm; ^{13}C NMR (100 MHz, $CDCl_3$): δ = 186.8 (**C**=O), 157.3 (**C**), 156.2 (**C**), 154.0 (**C**), 129.4 (**C**H), 127.4 (**C**H), 126.8 (**C**), 121.2 (**C**H), 111.9 (**C**H), 89.0 (**C**), 80.1 (**C**), 56.0 (**C**H_3), 28.7 (C(**C**H_3)$_3$), 9.3 (**C**H_3) ppm; MS (EI) *m/z* (%): 202 ([M – ^{t}BuOH]$^{+•}$, 26%), 146 (100%); HRMS (ES+) Found 299.1252 [M+Na]$^{+}$, $C_{16}H_{20}NaO_4$ requires 299.1254.

*2-(tert-Butoxy)-4-hydroxy-3-methyl-4-(pyridin-2-yl)cyclobut-2-enone (**12f**)*

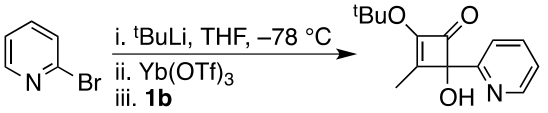

13f **12f**, 97%

To a solution of ^{t}BuLi (1.39 M in pentane, 1.47 mL, 2.05 mmol) in THF (10 mL) at –78 °C was added 2-bromopyridine (98.5 μL, 1.02 mmol) dropwise over 1 min. After 45 min the resulting solution was added via cannula over 10 min to a solution of $Yb(OTf)_3$ (635 mg, 1.02 mmol) in THF (10 mL) at –78 °C. Over the course of addition the solution turned from colourless to a deep orange/brown. After 30 min a solution of cyclobutenedione **1b** (151 mg, 0.90 mmol) in THF (5 mL) was added dropwise over 5 min during which time the colour faded to yellow/brown. After a further 30 min a 1 : 1 solution of sat. $NaHCO_3$ and sat. Rochelle salt (25 mL) was added then the reaction mixture was allowed to warm to RT and diluted with ether (30 mL). The aqueous phase was separated and extracted with ether (15 mL). The organic phases were combined, washed with brine (30 mL), dried over $MgSO_4$ and concentrated under reduced pressure to give the title compound **12f** (220 mg, 0.89 mmol, 97%) as a pale brown solid that was used without further purification. MP 78–80 °C; ^{1}H NMR (400 MHz, $CDCl_3$): δ = 8.62 (1H, ddd, *J* = 4.8, 1.7, 1.0 Hz, Ar**H**), 7.72 (1H, ddd, *J* = 8.4, 8.0, 1.7 Hz, Ar**H**), 7.27 (1H, ddd, *J* = 8.4, 4.8, 1.0 Hz, Ar**H**), 7.23 (1H, dt, *J* = 8.0, 1.0 Hz, Ar**H**), 6.02 (1H, br s, O**H**), 1.95 (3H, s, C**H₃**), 1.52 (9H, s, C(C**H₃**)₃) ppm; ^{13}C NMR (100 MHz, $CDCl_3$): δ = 186.0 (**C**=O), 156.4 (**C**), 156.31 (**C**), 156.29 (**C**), 148.2 (**C**H), 137.2 (**C**H), 122.8 (**C**H), 119.7 (**C**H), 88.8 (**C**), 80.5 (**C**), 28.6 (C(**C**H_3)$_3$), 8.6 (**C**H_3) ppm; IR: ν 3743 (w), 3726 (w), 3150 (br), 2983 (w), 2962 (w), 2938 (w), 1753 (s), 1626 (s), 1589 (w), 1475 (w), 1434 (w), 1385 (m), 1324 (s), 1258 (w), 1217 (w), 1160 (s), 1119 (m), 1054 (s), 980 (m), 915 (s), 845 (m), 792 (w), 772 (m), 707 (m), 653 (m), 625 (m), 559 (w), 539 (w), 465 (w), 445 (w) cm^{-1}; MS (ES^{+}) *m/z* (%): 311 ([M+Na+MeCN]$^{+}$, 100%).

*2-(tert-Butoxy)-4-hydroxy-3-methyl-4-(m-tolyl)cyclobut-2-enone (**12g**)*

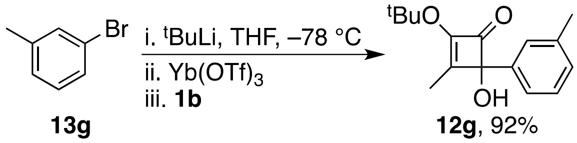

13g **12g**, 92%

To a solution of ^{t}BuLi (1.49 M in pentane, 1.32 mL, 1.97 mmol) in THF (10 mL) at –78 °C was added 2-bromotoluene (120 μL, 0.98 mmol) dropwise over 1 min. After 1 h the resulting solution was added via cannula over 10 min to a solution of $Yb(OTf)_3$ (610 mg, 0.98 mmol) in THF (10 mL) at –78 °C. Over the course of addition the solution turned from colourless to orange then intensified to a dark brown. After 45 min a solution of cyclobutenedione **1b** (154 mg, 0.92 mmol) in THF (5 mL) was added dropwise over 10 min during which time the

colour faded to pale yellow. After a further 30 min sat. $NaHCO_3$ (15 mL) was added then the reaction mixture was allowed to warm to RT and diluted with ether (30 mL). The aqueous phase was separated and extracted with ether (15 mL). The organic phases were combined, washed with water (30 mL) and brine (30 mL), dried over $MgSO_4$ and concentrated under reduced pressure to give the title compound **12f** (220 mg, 0.85 mmol, 92%) as a pale yellow oil that was used without further purification. ^{1}H NMR (300 MHz, $CDCl_3$): δ = 7.26 (1H, br t, J = 7.4 Hz, Ar**H**), 7.25 (1H, s with fine splitting, Ar**H**), 7.19 (1H, d with fine splitting, J = 7.7 Hz, Ar**H**), 7.12 (1H, d with fine splitting, J = 7.4 Hz, Ar**H**), 2.67 (1H, br s, O**H**), 2.37 (3H, d, J = 0.6 Hz, ArC**H₃**), 2.03 (3H, s, C**H₃**), 1.49 (9H, s, C(C**H₃**)₃) ppm; ^{13}C NMR (100 MHz, $CDCl_3$): δ = 187.0 (**C**=O), 157.8 (**C**), 154.4 (**C**), 138.4 (**C**), 138.3 (**C**), 128.7 (**C**H), 128.4 (**C**H), 126.3 (**C**H), 122.7 (**C**H), 88.8 (**C**), 80.3 (**C**), 28.7 (C(**C**H₃)₃), 21.5 (**C**H₃), 8.9 (**C**H₃) ppm; IR: ν 3428 (br), 2978 (w), 2917 (w), 2357 (m), 2341 (w), 1761 (s), 1630 (s), 1454 (w), 1368 (m), 1319 (m), 1254 (w), 1168 (m), 1050 (m), 931 (m), 813 (w), 690 (w) cm^{-1}; MS (EI) *m/z* (%): 260 ([M]$^{+\bullet}$, 2%), 175 (100%); HRMS (ES$^+$) Found 283.1306 [M+Na]$^+$ $C_{16}H_{20}NaO_3$ requires 283.1305.

*2-(tert-Butoxy)-4-hydroxy-3-methyl-4-(3-(trifluoromethyl)phenyl)cyclobut-2-enone (**12h**)*

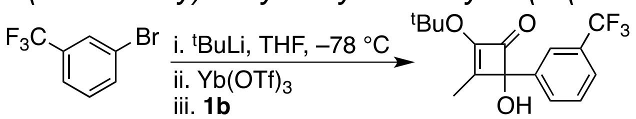

13h **12h**, 91%

To a solution of ^{t}BuLi (1.49 M in pentane, 1.29 mL, 1.92 mmol) in THF (10 mL) at –78 °C was added 1-bromo-3-(trifluoromethyl)benzene (134 μL, 0.96 mmol) dropwise over 1 min. After 40 min the resulting solution was added via cannula over 10 min to a solution of $Yb(OTf)_3$ (594 mg, 0.96 mmol) in THF (10 mL) at –78 °C. Over the course of addition the colourless solution became dark brown. After 30 min a solution of cyclobutenedione **1b** (146 mg, 0.87 mmol) in THF (5 mL) was added dropwise over 5 min during which time the colour became pale blue/green. After a further 30 min sat. $NaHCO_3$ (15 mL) was added then the reaction mixture was warmed to RT and diluted with ether (30 mL). The aqueous phase was separated and extracted with ether (15 mL). The organic phases were combined, washed with brine (30 mL), dried over $MgSO_4$ and concentrated under reduced pressure to give the title compound **12h** (246 mg, 0.78 mmol, 91%) as a pale yellow oil that was used without further purification. ^{1}H NMR (400 MHz, $CDCl_3$): δ = 7.71 (1H, app s with fine splitting, Ar**H**), 7.59 (1H, app br s, Ar**H**), 7.57 (1H, dt, J = 1.7, 0.6 Hz, Ar**H**), 7.53–7.47 (1H, m, Ar**H**), 2.62 (1H, br s, O**H**), 2.02 (3H, s, C**H₃**), 1.50 (9H, s, C(C**H₃**)₃) ppm; ^{13}C NMR (100 MHz, $CDCl_3$): δ = 186.0 (**C**=O), 157.3 (**C**), 155.2 (**C**), 139.5 (**C**), 131.0 (**C**, q, $J_{C\text{-}F}$ = 32.2 Hz), 129.1 (**C**H, q, $J_{C\text{-}F}$ = 1.5 Hz), 129.0 (**C**H), 124.8 (**C**H, q, $J_{C\text{-}F}$ = 3.7 Hz), 122.7 (**C**H, q, $J_{C\text{-}F}$ = 3.7 Hz), 88.8 (**C**), 80.9 (**C**), 28.6 (C(**C**H₃)₃), 8.8 (**C**H₃) ppm [**C**F₃ not observed]; ^{19}F NMR (282 MHz, $CDCl_3$) δ = −62.75 (3F, s, C**F₃**); IR: ν 3408 (br), 2978 (w), 2929 (w), 2357 (w), 2337 (w), 1756 (s), 1630 (m) 1442 (w), 1368 (m), 1327 (s), 1274 (w), 1165 (s), 1126 (s), 1074 (m), 1050 (m), 988 (w), 923 (w), 850 (w), 805 (w), 702 (m) cm^{-1}; HRMS (ES$^+$) Found 337.1023 [M+Na]$^+$ $C_{16}H_{17}F_3NaO_4$ requires 337.1022.

*2-(tert-Butoxy)-4-hydroxy-3-methyl-4-(propen-2-yl)cyclobut-2-enone (**20**)*

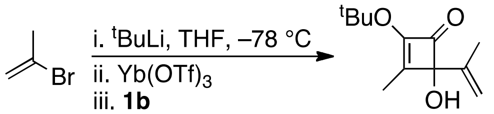

20, 98%

To a solution of ^{t}BuLi (1.49 M in pentane, 1.39 mL, 2.07 mmol) in THF (10 mL) at –78 °C was added 2-bromopropene (92 μL, 1.04 mmol) dropwise over 30 s. After 30 min the solution was warmed to 0 °C for 15 min then added via cannula over 10 min to a solution of $Yb(OTf)_3$ (617 mg, 1.00 mmol) in THF (10 mL) at –78 °C. Over the course of addition the solution turned from colourless to maroon to brown/black. After 45 min a solution of cyclobutenedione **1b** (86 mg, 0.51 mmol) in THF (3 mL) was added dropwise over 5 min during which time the colour became golden yellow. After a further 45 min sat. $NaHCO_3$ (20 mL) was added then the reaction mixture was warmed to RT and diluted with ether (30 mL). The aqueous phase was separated and extracted with ether (15 mL). The organic phases were combined, washed with water (30 mL) and brine (30 mL), dried over $MgSO_4$ and concentrated under reduced pressure to give the title compound **20** (105 mg, 0.50 mmol, 98%) as a colourless oil that was used without further purification. ^{1}H NMR (300 MHz, $CDCl_3$): δ = 5.20 (1H, br s, C**H**H), 5.09 (1H, app quin, J = 1.4 Hz, CH**H**), 2.19 (1H, s, O**H**), 2.00 (3H, s, C**H₃**), 1.74 (3H,

app d, J = 0.6 Hz, C**H$_3$**), 1.46 (9H, s, C(C**H$_3$**)$_3$) ppm; ^{13}C NMR (100 MHz, $CDCl_3$): δ = 187.8 (**C**=O), 157.4 (**C**), 154.1 (**C**), 142.3 (**C**), 113.5 (**C**H$_2$), 80.2 (**C**), 28.6 (C(**C**H$_3$)$_3$), 19.6 (**C**H$_3$), 9.0 (**C**H$_3$) [one **C** not observed] ppm; IR: ν 3401 (m), 2980 (w), 2938 (w), 2360 (w), 1744 (s), 1622 (s), 1462 (w), 1443 (w), 1373 (m), 1320 (m), 1260 (w), 1215 (w), 1170 (m), 1119 (w), 1074 (m),1033 (w), 1002 (w), 940 (m), 903 (s), 846 (m), 788 (w), 549 (m), 458 (m) cm^{-1}; MS (EI) *m/z* (%): 210 ([M]$^{+\bullet}$, 2%), 154 (100%); HRMS (ES$^+$) Found 233.1148 [M+Na]$^+$ $C_{12}H_{18}NaO_3$ requires 233.1148.

*2-(tert-Butoxy)-4-hydroxy-3-methyl-4-(1-phenylvinyl)cyclobut-2-enone (**21a**)*

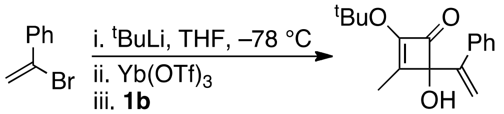

21a, 99%

To a solution of ^{t}BuLi (1.29 M in pentane, 1.54 mL, 1.99 mmol) in THF (10 mL) at –78 °C was added α-bromostyrene (136 μL, 1.00 mmol) dropwise over 1 min. After 1 h the resulting solution was added via cannula over 10 min to a solution of Yb(OTf)$_3$ (617 mg, 1.00 mmol) in THF (10 mL) at –78 °C. Over the course of addition the solution turned from colourless to deep maroon. After 40 min a solution of cyclobutenedione **1b** (162 mg, 0.95 mmol) in THF (5 mL) was added dropwise over 5 min during which time the colour became golden brown. After a further 40 min sat. $NaHCO_3$ (20 mL) was added then the reaction mixture was allowed to warm to RT and diluted with ether (30 mL). The aqueous phase was separated and extracted with ether (15 mL). The organic phases were combined, washed with brine (30 mL), dried over $MgSO_4$ and concentrated under reduced pressure to give the title compound **21a** (255 mg, 0.94 mmol, 99%) as a pale yellow oil that was used without further purification. ^{1}H NMR (300 MHz, $CDCl_3$): δ = 7.51–7.45 (2H, m, Ar**H**), 7.38–7.29 (3H, m, Ar**H**), 5.40 (1H, d, J = 0.8 Hz, C**H**H), 5.35 (1H, d, J = 0.8 Hz, CH**H**), 2.46 (1H, br s, O**H**), 2.02 (3H, s, C**H$_3$**), 1.39 (9H, s, C(C**H$_3$**)$_3$) ppm; ^{13}C NMR (100 MHz, $CDCl_3$): δ = 186.8 (**C**=O), 155.9 (**C**), 154.5 (**C**), 148.2 (**C**), 138.7 (**C**), 128.3 (2 x **C**H), 128.1 (2 x **C**H), 127.9 (**C**H), 116.0 (**C**H$_2$), 90.0 (**C**), 80.3 (**C**), 28.6 (C(**C**H$_3$)$_3$), 9.0 (**C**H$_3$) ppm; IR: ν 3413 (br), 2977 (w), 2361 (w),1753 (s), 1632 (m), 1371 (m), 1323 (m), 1260 (w), 1165 (m), 1051 (m), 1028 (m), 916 (s), 850 (w), 775 (w), 700 (s), 506 (w) cm^{-1}; MS (EI) *m/z* (%): 272 ([M]$^{+\bullet}$, 2%), 216 (100%); HRMS (EI) Found 272.1398 [M]$^{+\bullet}$, $C_{17}H_{20}O_3$ requires 272.1412.

*2-(tert-Butoxy)-4-hydroxy-3-methyl-4-vinylcyclobut-2-enone (**22**)*

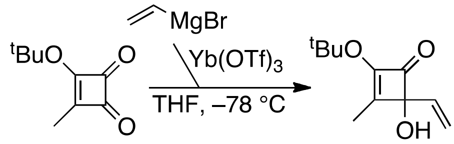

1b **22**, 98%

To a solution of Yb(OTf)$_3$ (587 mg, 0.95 mmol) in THF (10 mL) at –78 °C was added vinylmagnesium bromide (1 M in THF, 947 μL, 0.95 mmol) dropwise over 1 min. Over the course of addition the solution turned from colourless to bright orange. After 45 min a solution of cyclobutenedione **1b** (78 mg, 0.47 mmol) in THF (3 mL) was added dropwise over 5 min, during which time the colour faded to pale yellow. After 45 min sat. $NaHCO_3$ (10 mL) was added then the reaction mixture was allowed to warm to RT and diluted with ether (20 mL). The aqueous phase was separated and extracted with ether (15 mL). The organic phases were combined, washed with water (20 mL) and brine (20 mL), dried over $MgSO_4$ and concentrated under reduced pressure to give the title compound **22** (90 mg, 0.47 mmol, 98%) as a colourless oil that was used without further purification. ^{1}H NMR (300 MHz, $CDCl_3$): δ = 5.92 (1H, dd, J = 17.3, 10.7 Hz, C**H**), 5.43 (1H, dd, J = 17.4, 0.9 Hz, C**H**H), 5.33 (1H, dd, J = 10.7, 1.0 Hz, CH**H**), 2.28 (1H, br s, O**H**), 2.01 (3H, s, C**H$_3$**), 1.45 (9H, s, C(C**H$_3$**)$_3$) ppm; ^{13}C NMR (100 MHz, $CDCl_3$): δ = 187.5 (**C**=O), 157.9 (**C**), 153.6 (**C**), 135.6 (**C**H), 117.4 (**C**H$_2$), 87.9 (**C**), 80.3 (**C**), 28.6 (C(**C**H$_3$)$_3$), 8.8 (**C**H$_3$) ppm; IR: ν 3404 (br), 2975 (m), 2934 (w), 2881 (w), 2362 (w), 2333 (w), 1749 (s), 1627 (m), 1475 (w), 1369 (m), 1324 (m), 1255 (w), 1161 (m), 1059 (w), 998 (w), 920 (s), 846 (w) cm^{-1}; MS (EI) *m/z* (%): 210 ([M − ^{t}Bu]$^{+\bullet}$, 100%); HRMS (ES$^+$) Found 219.0991 [M+Na]$^+$ $C_{11}H_{16}NaO_3$ requires 219.0992.

*2-(tert-Butoxy)-4-hydroxy-3,4-dimethylcyclobut-2-enone (**28a**)*

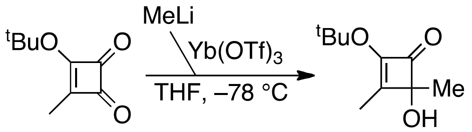

1b **28a**, 98%

To a solution of $Yb(OTf)_3$ (631 mg, 1.02 mmol) in THF (10 mL) at –78 °C was added MeLi (1.02 M in hexanes, 1.10 mL, 1.12 mmol) dropwise over 1 min. Over the course of addition the solution turned from colourless to deep purple to deep red. After 1 h a solution of cyclobutenedione **1b** (162 mg, 0.97 mmol) in THF (10 mL) was added dropwise over 10 min, during which time the colour faded to pale red. After 1 h sat. $NaHCO_3$ (10 mL) was added then the reaction mixture was allowed to warm to RT and diluted with ether (15 mL). The aqueous phase was separated and extracted with ether (10 mL). The organic phases were combined, washed with brine (20 mL), dried over $MgSO_4$ and concentrated under reduced pressure to give the title compound **28a** (175 mg, 0.95 mmol, 98%) as a white solid that was used without further purification. MP 101–103 °C; ^{1}H NMR (400 MHz, $CDCl_3$): δ = 2.11 (1H, br s, O**H**), 2.02 (3H, s, C**H₃**), 1.45 (3H, s, C**H₃**), 1.43 (9H, s, C(C**H₃**)₃) ppm; ^{13}C NMR (100 MHz, $CDCl_3$): δ = 190.4 (**C**=O), 160.2 (**C**), 152.1 (**C**), 84.6 (**C**(CH_3)₃), 79.9 (**C**), 28.6 (C(**C**H_3)₃), 19.4 (**C**H_3), 8.4 (**C**H_3) ppm; UV/Vis: (acetonitrile): λ_{max} (ε) = 247 (1850), 200 (375); IR: ν 3244 (br), 2978 (w), 2934 (w), 1752 (s), 1628 (s), 1442 (w), 1371 (m), 1323 (s), 1173 (m), 1119 (s), 1089 (w), 1052 (m), 930 (m), 884 (m) cm^{-1}; MS (EI) *m/z* (%): 166 ($[M - H_2O]^{+\bullet}$, 7%), 110 ($[M - Me_2C{=}CH_2]^{+\bullet}$, 59%), 54 (100%); HRMS (EI) found 166.0998 $[M - H_2O]^{+\bullet}$, $C_{10}H_{14}O_2$ requires 166.0994.

*2-(tert-Butoxy)-4-butyl-4-hydroxy-3-methylcyclobut-2-enone (**28b**)*

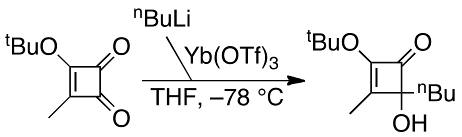

1b **28b**, 95%

To a solution of $Yb(OTf)_3$ (614 mg, 0.99 mmol) in THF (10 mL) at –78 °C was added ^{n}BuLi (2.32 M in hexanes, 427 µL, 0.99 mmol) dropwise over 1 min. Over the course of addition the solution turned from colourless to royal blue to blue/black and 15 min after addition was deep maroon. After 30 min a solution of cyclobutenedione **1b** (74.6 mg, 0.44 mmol) in THF (5 mL) was added dropwise over 5 min during which time the colour faded to pale violet. After a further 1 h a 1 : 1 : 1 solution of sat. $NaHCO_3$, sat. Rochelle salt and water (15 mL) was added then the reaction mixture was allowed to warm to RT and diluted with ether (20 mL). The aqueous phase was separated and extracted with ether (15 mL). The organic phases were combined, washed with brine (20 mL), dried over $MgSO_4$ and concentrated under reduced pressure to give the title compound **28b** (95 mg, 0.42 mmol, 95%) as a colourless oil that was used without further purification. ^{1}H NMR (400 MHz, $CDCl_3$): δ = 2.49 (1H, br s, O**H**), 2.00 (3H, s, C**H₃**), 1.83 (1H, ddd, *J* = 13.9, 9.7, 6.8 Hz, C**H**H), 1.75 (1H, ddd, *J* = 13.8, 9.6, 6.6 Hz, CH**H**), 1.41 (9H, s, C(C**H₃**)₃), 1.35–1.16 (4H, m, 2 x C**H₂**), 0.87 (3H, t, *J* = 7.2 Hz, C**H₃**) ppm; ^{13}C NMR (100 MHz, $CDCl_3$): δ = 190.4 (**C**=O), 159.2 (**C**), 152.7 (**C**), 87.6 (**C**), 79.8 (**C**), 32.8 (**C**H_2), 28.6 (C(**C**H_3)₃), 27.2 (**C**H_2), 22.9 (**C**H_2), 13.8 (**C**H_3), 8.9 (**C**H_3) ppm; IR: ν 3441 (br), 2960 (m), 2933 (m), 2873 (w), 2361 (s), 2340 (s), 2157 (w), 2018 (w), 1761 (s), 1634 (m), 1602 (m), 1459 (w), 1372 (m), 1323 (m),1260 (w), 1168 (m), 1031 (w), 937 (w), 772 (w), 670 (w), 421 (w) cm^{-1}; MS (EI) *m/z* (%): 208 ($[M - Me_2C{=}CH_2]^{+\bullet}$, 2%), 141 (100%).

*2-(tert-Butoxy)-4-hydroxy-3-methyl-4-(phenylethynyl)cyclobut-2-enone (**34**) and 3-(tert-butoxy)-4-hydroxy-2-methyl-4-(phenylethynyl)cyclobut-2-enone (**33**)*

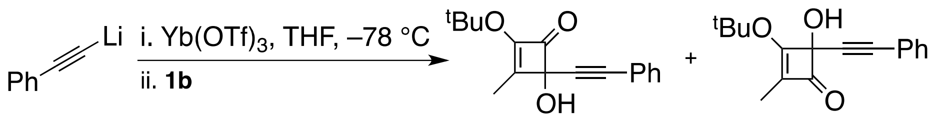

34, 75% **33**, 15%

To a solution of $Yb(OTf)_3$ (904 mg, 1.46 mmol) in THF (10 mL) at –78 °C was added lithium phenyl-acetelide (1.00 M in THF, 1.46 mL, 1.46 mmol) dropwise over 1 min. After 30 min a solution of cyclobutenedione **1b** (196 mg, 1.17 mmol) in THF (10 mL) was added dropwise over 10 min. After a further 9 h $NaHCO_3$ was added then the reaction mixture was allowed to warm to RT and diluted with ether (20 mL). The aqueous phase was separated and extracted with ether (15 mL). The organic phases were combined, washed with brine (20 mL), dried over $MgSO_4$ and concentrated under reduced pressure to give an orange brown oil. Purification by flash column chromatography (0.05–0.3% ethyl acetate/dichloromethane with 1% NH_4OH) afforded firstly cyclobutenone **34** (237 mg, 0.88 mmol, 75%) as a pale yellow solid: MP 100–101 °C (diethyl ether/hexane); ^{1}H NMR (400 MHz, $CDCl_3$): δ = 7.47–7.43 (2 H, m,

ArH), 7.35–7.30 (3 H, m, ArH), 2.72 (1 H, br s, OH), 2.15 (3 H, s, C**H$_3$**), 1.48 (9 H, s, C(C**H$_3$**)$_3$) ppm; ^{13}C NMR (100 MHz, $CDCl_3$): δ = 183.5 (**C**=O), 157.0 (**C**), 154.7 (**C**), 131.8 (2 x **C**H), 128.8 (2 x **C**H), 128.3 (**C**H), 121.9 (**C**), 88.5 (**C**), 84.4 (**C**), 81.0 (**C**), 80.5 (**C**), 28.6 (C(**C**H$_3$)$_3$), 9.0 (**C**H$_3$) ppm; IR: ν 3391 (br), 2979 (w), 2930 (w), 2362 (w), 2337 (w), 1753 (s), 1631 (s), 1590 (m), 1386 (m), 1382 (m), 1324 (s), 1267 (w), 1149 (m), 1079 (w), 1026 (m), 920 (s), 846 (w), 757 (s), 687 (m) cm^{-1}; HRMS (ES$^+$) Found 293.1149 $[M+Na]^+$ $C_{17}H_{18}NaO_3$ requires 293.1148; X-Ray (thermal ellipsoids drawn at 35% probability level, selected hydrogens omitted for clarity). Then cyclobutenone **33** (47 mg, 0.17 mmol, 15%) as a pale yellow oil. ^{1}H NMR (400 MHz, $CDCl_3$): δ = 7.45–7.40 (2 H, m, Ar**H**), 7.33–7.24 (3 H, m, Ar**H**), 4.56 (1 H, br s, O**H**), 1.71 (3 H, s, C**H$_3$**), 1.64 (9 H, s, C(C**H$_3$**)$_3$) ppm; ^{13}C NMR (100 MHz, $CDCl_3$): δ = 188.9 (**C**=O), 180.0 (**C**), 131.7 (2 x **C**H), 128.6 (**C**H), 128.1 (2 x **C**H), 126.6 (**C**), 122.0 (**C**), 89.2 (**C**), 85.7 (**C**), 84.6 (**C**), 84.4 (**C**), 28.9 (C(**C**H$_3$)$_3$), 7.3 (**C**H$_3$) ppm; IR: ν 2922 (s), 2366 (m), 1757 (w), 1602 (m), 1459 (w), 1398 (w),1341 (w), 1145 (w), 757 (w), 687 (w) cm^{-1}; HRMS (ES$^+$) Found 293.1144 $[M+Na]^+$ $C_{17}H_{18}NaO_3$ requires 293.1148.

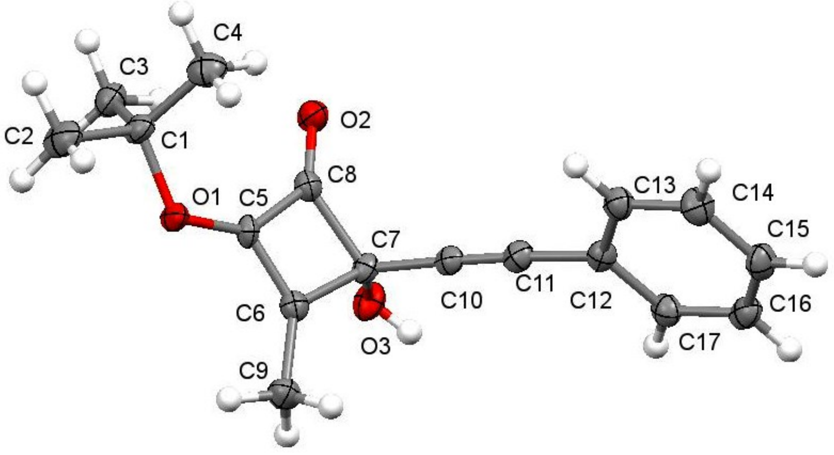

Comparative Additions of Organolithium Reagents to Cyclobutenediones 1

*4-Hydroxy-3-methoxy-2-methyl-4-phenylcyclobut-2-enone (**2a**)*

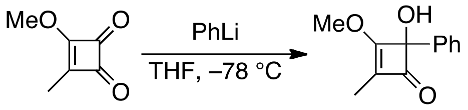

1a **2a**, 76%

To a solution of PhLi (0.94 M in dibutyl ether, 559 µL, 0.53 mmol) in THF (2.5 mL) at –78 °C was added a solution of cyclobutenedione **1a** (63 mg, 0.5 mmol) in THF (2.5 mL) was added dropwise over 5 min and the solution turned from pale pink to deep orange. After 30 min the reaction mixture was quenched with sat. $NaHCO_3$ (10 mL) then warmed to RT and diluted with dichloromethane (15 mL). The aqueous phase was separated and extracted with dichloromethane (10 mL). The organic phases were combined, washed with brine (20 mL), dried over $MgSO_4$ and concentrated under reduced pressure to give a yellow oil (127 mg). Purification by flash column chromatography (0.2–0.4% ethyl acetate/dichloromethane) afforded cyclobutenone **2a** as a colourless oil (77 mg, 0.38 mmol, 76%, data as above).[5]

*4-Hydroxy-3-methoxy-2-methyl-4-(p-tolyl)cyclobut-2-enone (**2b**)*

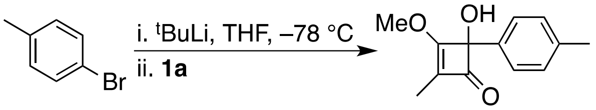

13b **2b**, 89%

To a solution of ^{t}BuLi (2.06 M in pentane, 509 µL, 2.14 mmol) in THF (7 mL) at –78 °C was added *p*-bromotoluene (66 µL, 0.53 mmol) dropwise over 1 min. After 30 min a solution of cyclobutenedione **1a** (65 mg, 0.52 mmol) in THF (5 mL) was added dropwise over 5 min. Over the course of addition the solution turned from colourless to yellow then intensified to become bright orange. After 1 h sat. $NaHCO_3$ (10 mL) was added then the reaction mixture was warmed to RT and dichloromethane (15 mL) was added. The aqueous phase was separated and extracted with dichloromethane (10 mL). The organic phases were combined, dried over $MgSO_4$ and concentrated under reduced pressure to give a pale orange oil (114 mg). Purification by flash column chromatography (4–30% ethyl acetate/ dichloromethane) afforded the title compound **2b** (100 mg, 0.46 mmol, 89%) as a colourless oil. ^{1}H NMR (400 MHz, $CDCl_3$): δ = 7.36 (2H, d with fine splitting, *J* = 8.3 Hz, 2 x Ar**H**), 7.17 (2H, d with fine splitting, *J* = 8.0 Hz, 2 x Ar**H**), 4.52 (1H, s, O**H**), 3.99 (3H, s, OC**H$_3$**), 2.35 (3H, s, C**H$_3$**), 1.76 (3H, s, C**H$_3$**) ppm; ^{13}C NMR (100 MHz, $CDCl_3$): δ = 191.5 (**C**=O), 182.7 (**C**), 137.8 (**C**), 133.9 (**C**), 129.2 (2 x **C**H), 125.5 (2 x **C**H), 124.7 (**C**), 92.1 (**C**), 59.6 (O**C**H$_3$), 21.1 (**C**H$_3$), 6.3 (**C**H$_3$) ppm; MS (EI) *m/z* (%): 218 ([M]$^{+\bullet}$, 79%), 216 (100%); HRMS

(ES+) Found 241.0834 $[M+Na]^+$ and 219.1014 $[M+H]^+$, $C_{13}H_{14}NaO_3$ requires 241.0835 and $C_{13}H_{15}O_3$ requires 219.1016.

*4-Hydroxy-3-methoxy-4-(4-methoxyphenyl)-2-methylcyclobut-2-enone (**2c**)*

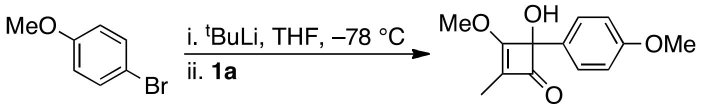

13c **2c**, 74%

To a solution of ^tBuLi (2.06 M in heptane, 509 μL, 1.05 mmol) in THF (7 mL) at –78 °C was added *para*-bromoanisole (66 μL, 0.53 mmol) dropwise over 1 min. After 45 min a solution of cyclobutenedione **1a** (62 mg, 0.49 mmol) in THF (5 mL) was added dropwise over 10 min and the solution became bright orange. After 1 h sat. $NaHCO_3$ (10 mL) was added. The solution was warmed to RT and dichloromethane (10 mL) was added. The aqueous phase was separated and extracted with dichloromethane (10 mL) then the organic phases were combined, dried over $MgSO_4$ and concentrated under reduced pressure to a pale yellow oil (199 mg). Purification by flash column chromatography (5–30% ethyl acetate/chloroform with 1% NH_4OH) afforded cyclobutenone **2c** as a colourless oil (85 mg, 0.36 mmol, 74%). ^1H NMR (400 MHz, $CDCl_3$): δ = 7.42–7.37 (2 H, m, Ar**H**), 6.91–6.86 (2 H, m, Ar**H**), 4.21 (1 H, br s, O**H**), 4.01 (3 H, s, OC**H$_3$**), 3.80 (3 H, s, OC**H$_3$**), 1.77 (3 H, s, C**H$_3$**) ppm; ^{13}C NMR (100 MHz, $CDCl_3$): δ = 191.5 (**C**=O), 182.7 (**C**), 159.5 (**C**), 129.0 (**C**), 126.9 (2 x **C**H), 124.6 (**C**), 114.0 (2 x **C**H), 91.8 (**C**), 59.6 (O**C**H$_3$), 55.2 (O**C**H$_3$), 6.4 (**C**H$_3$) ppm; IR: ν 3285 (br w), 2922 (w), 2852 (w), 2366 (w), 2337 (w), 1757 (m), 1614 (s), 1455 (m), 1377 (m), 1337 (s), 1247 (m), 1153 (w), 1006 (m), 867 (w), 765 (m), 659 (m) cm^{-1}; MS (EI) *m/z* (%): 234 ($[M]^{+\bullet}$, 100%).

*4-Hydroxy-3-methoxy-2-methyl-4-(4-(trifluoromethyl)phenyl)cyclobut-2-enone (**2d**)*

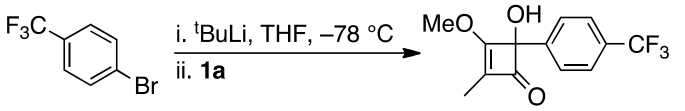

13d **2d**, 74%

To a solution of ^tBuLi (1.49 M in pentane, 1.45 mL, 2.16 mmol) in THF (10 mL) at –78 °C was added 1-bromo-4-(trifluoromethyl)benzene (150 μL, 1.05 mmol) dropwise over 1 min. After 1 h a solution of cyclobutenedione **1a** (127 mg, 1.01 mmol) in THF (10 mL) was added over 15 min during which time the solution became dark brown/black. After 1 h sat. $NaHCO_3$ (15 mL) was added. The solution was warmed to RT and dichloromethane (30 mL) was added. The aqueous phase was separated and extracted with dichloromethane (15 mL) then the organic phases were combined, washed with brine (30 mL), dried over $MgSO_4$ and concentrated under reduced pressure to a pale yellow solid (251 mg). Purification by flash column chromatography (2–10% ethyl acetate/dichloromethane with 0.5% NH_4OH) afforded **2d** as an off-white solid (200 mg, 0.738 mmol, 74%). MP 147–148 °C (ethyl acetate/hexane); ^1H NMR (400 MHz, $CDCl_3$): δ = 7.64 (2 H, d, *J* = 8.7 Hz, Ar**H**), 7.60 (2 H, d, *J* = 8.7 Hz, Ar**H**), 4.18 (1 H, br s, O**H**), 4.02 (3 H, s, OC**H$_3$**), 1.82 (3 H, s, C**H$_3$**) ppm; ^{13}C NMR (100 MHz, $CDCl_3$): δ = 189.7 (**C**=O), 181.7 (**C**), 140.8 (**C**), 130.5 (**C**, q, $J_{C\text{-}F}$ = 32.2 Hz), 126.1 (2 x **C**H), 125.8 (**C**), 125.6 (2 x **C**H, q, $J_{C\text{-}F}$ = 3.9 Hz), 92.1 (**C**), 59.8 (O**C**H$_3$), 6.5 (**C**H$_3$) ppm [**C**F$_3$ not observed]; IR: ν 3310 (br), 2954 (w), 2358 (w),1753 (m), 1602 (s), 1455 (w),1418 (w), 1390 (m), 1328 (s), 1161 (m), 1108 (m), 1071 (m), 998 (m), 908 (w), 834 (m), 736 (w), 622 (w) cm^{-1}; MS (EI) *m/z* (%): 270 ($[M − 2]^{+\bullet}$, 95%), 115 (100%); HRMS (ES+) found 273.0732 $[M+H]^+$, $C_{13}H_{12}F_3O_3$ requires 273.0733.

*3-Methoxy-4-methyl-[1,1'-biphenyl]-2,5-diol (**27**) and*
*4-hydroxy-2-methoxy-3-methyl-4-(1-phenylvinyl)cyclobut-2-enone (**21b**)*

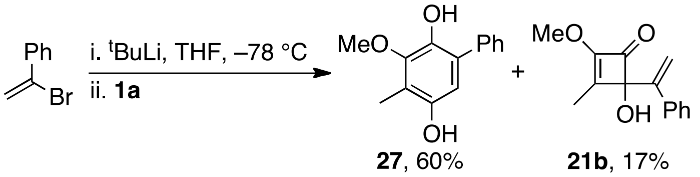

27, 60% **21b**, 17%

To a solution of ^tBuLi (1.49 M in pentane, 705 μL, 1.05 mmol) in THF (7 mL) at –78 °C was added α-bromostyrene (72 μL, 0.55 mmol) dropwise over 1 min. Over the course of addition the solution became deep orange. After 15 min the solution was warmed to 0 °C over 15 min and became bright red. After a further 15 min the solution was cooled to –78 °C and a solution of cyclobutenedione **1a** (63 mg, 0.50 mmol) in THF (5 mL) was added dropwise over 5 min, during which time the colour became dark brown then golden brown. After 30 min sat.

$NaHCO_3$ (10 mL) was added. The solution was warmed to RT and dichloromethane (15 mL) was added. The aqueous phase was separated and extracted with dichloromethane (10 mL) then the organic phases were combined, dried over $MgSO_4$ and concentrated under reduced pressure to a yellow oil (127 mg). Purification by flash column chromatography (0.2–2% ethyl acetate/ dichloromethane with 1% NH_4OH) afforded firstly hydroquinone **27** (70 mg, 0.30 mmol, 60%) as a dark orange oil; ^{1}H NMR (400 MHz, $CDCl_3$): δ = 7.60–7.54 (2 H, m, Ar**H**), 7.47–7.40 (2 H, m, Ar**H**), 7.37–7.30 (1 H, m, Ar**H**), 6.59 (1 H, s, C**H**), 5.50 (1 H, s, O**H**), 4.69 (1 H, s, O**H**), 3.84 (3 H, s, OC**H$_3$**), 2.25 (3 H, s, C**H$_3$**) ppm; ^{13}C NMR (100 MHz, $CDCl_3$): δ = 147.3 (**C**), 146.2 (**C**), 140.1 (**C**), 137.3 (**C**), 129.0 (2 x **C**H), 128.3 (2 x **C**H), 127.1 (**C**H), 125.6 (**C**), 117.0 (**C**), 111.7 (**C**H), 60.9 (O**C**H$_3$), 9.3 (**C**H$_3$) ppm; IR: ν 3404 (br), 2917 (w), 2848 (w), 2362 (w), 2337 (w), 1733 (w), 1606 (w), 1463 (w), 1410 (m), 1263 (w), 1214 (w), 1075 (s), 1018 (w), 769 (w), 695 (m), 659 (w), 507 (w) cm^{-1}; MS (EI) *m/z* (%): 230 ($[M]^{+\bullet}$, 100%); HRMS (ES+) found 231.1010 $[M+H]^+$ and 253.0830 $[M+Na]^+$, $C_{14}H_{15}O_3$ requires 231.1016 and $C_{14}H_{14}NaO_3$ requires 253.0835; then cyclobutenone **21b** (19 mg, 0.08 mmol, 17%) as a yellow oil ^{1}H NMR (400 MHz, $CDCl_3$): δ = 7.54–7.50 (2 H, m, Ar**H**), 7.40–7.30 (3 H, m, Ar**H**), 5.38 (1H, d, *J* = 0.8 Hz, =C**H**H), 5.37 (1H, d, *J* = 0.8 Hz, =CH**H**), 4.00 (3 H, s, OC**H$_3$**), 2.51 (1 H, br s, O**H**), 2.05 (3 H, s, C**H$_3$**) ppm; ^{13}C NMR (100 MHz, $CDCl_3$): δ = 187.3 (**C**=O), 155.2 (**C**), 151.6 (**C**), 147.7 (**C**), 138.4 (**C**), 128.4 (2 x **C**H), 128.0 (**C**H), 127.9 (2 x **C**H), 116.1 (**C**H$_2$), 91.0 (**C**), 58.1 (O**C**H$_3$), 9.3 (**C**H$_3$) ppm; MS (EI) *m/z* (%): 230 ($[M]^{+\bullet}$, 100%); HRMS (EI) found 230.0943 $[M]^{+\bullet}$, $C_{14}H_{14}O_3$ requires 230.0943.

*4-Hydroxy-3-methoxy-2-methyl-4-(phenylethynyl)cyclobut-2-enone (**32**)*

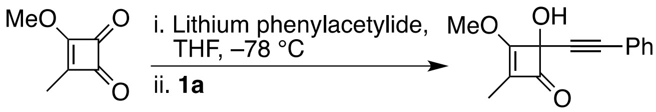

1a **32**, 69%

To a solution of lithium phenylacetylide (1.00 M in THF, 946 µL, 1.05 mmol) in THF (10 mL) at –78 °C was added a solution of cyclobutene-dione **1a** (126 mg, 1.00 mmol) in THF (10 mL) was added dropwise over 10 min. After 6 h $NaHCO_3$ (10 mL) was added then the reaction mixture was allowed to warm to RT and diluted with dichloromethane (20 mL). The aqueous phase was separated and extracted with dichloromethane (15 mL). The organic phases were combined, dried over $MgSO_4$ and concentrated under reduced pressure to give an orange brown oil. Purification by flash column chromatography (1–10% ethyl acetate/dichloromethane with 1% NH_4OH) afforded cyclobutenone **32** (157 mg, 0.69 mmol, 69%) as an off-white solid. MP 98–99 °C (chloroform/hexane); ^{1}H NMR (400 MHz, $CDCl_3$): δ = 7.49–7.44 (2 H, m, Ar**H**), 7.37–7.28 (3 H, m, Ar**H**), 4.32 (3 H, s, OC**H$_3$**), 3.76 (1 H, s, O**H**), 1.74 (3 H, s, C**H$_3$**) ppm; ^{13}C NMR (100 MHz, $CDCl_3$): δ = 187.2 (**C**=O), 180.6 (**C**), 131.8 (2 x **C**H), 129.0 (**C**H), 128.3 (2 x **C**H), 125.4 (**C**), 121.7 (**C**), 89.9 (**C**), 83.1 (**C**), 83.0 (**C**), 59.8 (O**C**H$_3$), 6.4 (**C**H$_3$) ppm; IR: ν 3220 (br), 1749 (m), 1602 (s), 1492 (w), 1463 (w), 1394 (m), 1341 (s), 1267 (w), 1222 (w), 1157 (w), 1083 (m), 993 (m), 920 (m), 761 (m), 695 (m) cm^{-1}; MS (EI) *m/z* (%): 228 ($[M]^{+\bullet}$, 3%), 129 (100%); HRMS (ES+) found 251.0678 $[M+Na]^+$ and 229.0858 $[M+H]^+$, $C_{14}H_{12}NaO_3$ requires 251.0679 and $C_{14}H_{13}O_3$ requires 229.0859.

ADDITIONAL EXPERIMENTS AS NOTED ON PAGE S1 AND IN REFERENCE 7 OF THE ARTICLE:

*4-Hydroxy-3-methoxy-2,4-dimethylcyclobut-2-enone (**50**)*

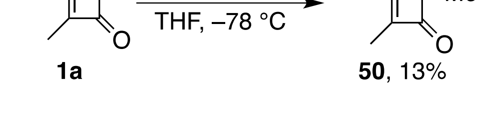

1a **50**, 13%

To a solution of MeLi (2.23 M in Et_2O, 470 µL, 1.05 mmol) in THF (10 mL) at –78 °C was added cyclobutenedione **1a** (126 mg, 1.00 mmol) in THF (5 mL) dropwise over 10 min. After 1 h sat. $NaHCO_3$ (10 mL) was added. The solution was warmed to RT and dichloromethane (15 mL) was added. The aqueous phase was separated and extracted with dichloromethane (10 mL) then the organic phases were combined, dried over $MgSO_4$ and concentrated under reduced pressure to a colourless oil (93 mg). Purification by flash column chromatography (5–30% ethyl acetate/dichloromethane) afforded **50** as a colourless oil (18 mg, 0.127 mmol, 13%). ^{1}H NMR (400 MHz, $CDCl_3$): δ = 4.17 (3 H, s, OC**H$_3$**), 3.89 (1 H, br s, O**H**), 1.67 (3 H, s, C**H$_3$**), 1.54 (3 H, s, C**H$_3$**) ppm; ^{13}C NMR (100 MHz, $CDCl_3$): δ = 194.7 (**C**=O), 184.9 (**C**), 121.3 (**C**), 87.9 (**C**), 59.3 (O**C**H$_3$), 19.3 (**C**H$_3$), 6.3 (**C**H$_3$) ppm; IR: ν 3367 (br), 2962 (w), 2922 (w), 2864 (w), 2358 (w), 2333 (w), 1749 (m), 1618 (s),

1459 (w), 1386 (w), 1337 (s), 1136 (w), 961 (w), 891 (w) cm^{-1}; MS (EI) *m/z* (%): 142 ([M]$^{+•}$, 7%), 43 (100%); HRMS (ES+) found 165.0524 [M+Na]$^+$ and 307.1154 [2M+Na]$^+$, $C_7H_{10}NaO_3$ requires 165.0522 and $C_{14}H_{20}NaO_6$ requires 307.1152.

*4-Hydroxy-3-methoxy-4-(2-methoxyphenyl)-2-methylcyclobut-2-enone (**2e**)*

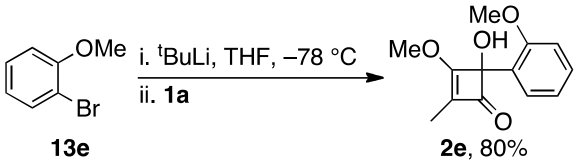

To a solution of ^{t}BuLi (2.06 M in heptane, 509 μL, 1.05 mmol) in THF (7 mL) at –78 °C was added 2-bromoanisole (67 μL, 0.53 mmol) dropwise over 1 min. After 45 min a solution of cyclobutenedione **1a** (60 mg, 0.46 mmol) in THF (5 mL) was added dropwise over 5 min during which time the colour became bright yellow. After 1 h a solution of sat. $NaHCO_3$ (10 mL) was added. The solution was warmed to RT and dichloromethane (15 mL) was added. The aqueous phase was separated and extracted with dichloromethane (10 mL) then the organic phases were combined, washed with brine (20 mL), dried over $MgSO_4$ and concentrated under reduced pressure to a waxy oil (143 mg). Purification by flash column chromatography (2–15% ethyl acetate/chloroform with 1% NH_4OH) afforded **2e** as a white solid (85.8 mg, 0.366 mmol, 80%).[5,6] MP 112–114 °C (ethyl acetate/hexane), Lit. oil;[6] ^{1}H NMR (400 MHz, $CDCl_3$): δ = 7.30 (1 H, ddd, *J* = 8.2, 7.4, 1.7 Hz, Ar**H**), 7.25 (1 H, dd, *J* = 8.0, 1.7 Hz, Ar**H**), 7.00–6.94 (2 H, m Ar**H**), 5.09 (1 H, s, O**H**), 4.11 (3 H, s, ArOC**H₃**), 3.92 (3 H, s, OC**H₃**), 1.77 (3 H, s, C**H₃**) ppm; ^{13}C NMR (100 MHz, $CDCl_3$): δ = 190.0 (**C**=O), 180.6 (**C**), 157.2 (**C**), 129.6 (**C**H), 127.3 (**C**H), 124.9 (**C**), 124.1 (**C**), 121.3 (**C**H), 112.1 (**C**H), 93.1 (**C**), 59.6 (**C**H_3), 56.3 (**C**H_3), 6.5 (**C**H_3) ppm; IR: ν 3285 (br), 2954 (w), 2913 (w), 2844 (w), 2362 (w), 2341 (w), 1749 (m), 1618 (s), 1492 (w), 1459 (m), 1386 (m), 1337 (s), 1255 (s), 1153 (m), 1006 (m), 900 (w), 863 (m), 769 (m), 744 (w), 663 (m), 597 (w), 467 (m) cm^{-1}; MS (EI) *m/z* (%): 234 ([M]$^{+•}$, 100%); HRMS (ES+) found 257.0785 [M+Na]$^+$ and 491.1676 [2M+Na]$^+$, $C_{13}H_{14}NaO_4$ requires 257.0784 and $C_{26}H_{28}NaO_8$ requires 491.1676.

*3-(tert-Butoxy)-4-hydroxy-4-(4-methoxyphenyl)-2-methylcyclobut-2-enone (**12c**) and 2-(tert-butoxy)-4-hydroxy-4-(4-methoxyphenyl)-3-methylcyclobut-2-enone (**10c**)*

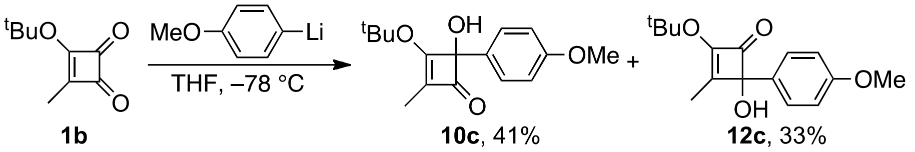

To a solution of ^{t}BuLi (1.39 M in pentane, 1.51 mL, 2.10 mmol) in THF (10 mL) at –78 °C was added *p*-bromoanisole (133 μL, 1.05 mmol) dropwise over 1 min. After 2 h a solution of cyclobutenedione **1b** (162 mg, 0.96 mmol) in THF (10 mL) was added dropwise over 10 min. After 40 min sat. $NaHCO_3$ (15 mL) was added, the reaction mixture was warmed to RT and ether (20 mL) added. The aqueous phase was separated and extracted with ether (15 mL). The organic phases were combined, washed with brine (30 mL), dried over $MgSO_4$ and concentrated under reduced pressure to a pale yellow oil (277 mg). Purification by flash column chromatography (rapidly with gradient elution from 10–70% ethyl acetate/petroleum ether with 1% aq. NH_3) afforded firstly cyclobutenone **12c** (89 mg, 0.32 mmol, 33%, data as above) followed by the cyclobutenone **10c** (120 mg) as a turbid oil. The oil was dissolved in a minimum of diethyl ether and cold petroleum ether was added causing precipitation. The solid was collected by filtration then washed with cold petroleum ether. Residual solvents were removed under high vacuum to give cyclobutenone **10c** as a white solid (110 mg, 0.40 mmol, 41%). MP 98–99 °C (Et_2O/petroleum ether); ^{1}H NMR (300 MHz, $CDCl_3$): δ = 7.42 (2H, d, *J* = 8.8 Hz, 2 x **C**H), 6.88 (2H, d, *J* = 8.8 Hz, 2 x **C**H), 3.80 (3H, s, OC**H₃**), 3.47 (1H, br s, O**H**), 1.83 (3H, s, C**H₃**), 1.47 (9H, s, C(C**H₃**)₃) ppm; ^{13}C NMR

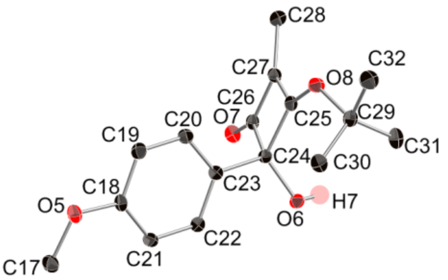

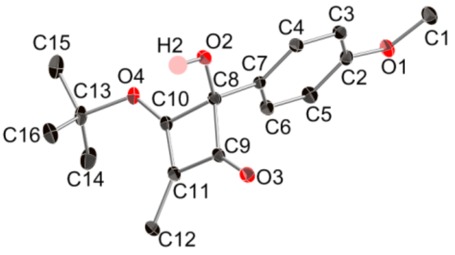

(100 MHz, $CDCl_3$): δ = 192.4 (**C**=O), 179.9 (**C**), 159.5 (**C**), 129.8 (**C**), 127.1 (2 x **C**H), 123.8 (**C**), 113.9 (2 x **C**H), 92.6 (**C**), 84.2 (**C**), 55.3 (**C**H_3), 28.8 (C(**C**H_3)$_3$), 8.5 (**C**H_3) ppm; IR: ν 3350 (b), 2978 (w), 2929 (w), 2831 (w), 2366 (w), 2337 (w), 1744 (m), 1585 (s), 1507 (s), 1462 (w), 1389 (m), 1340 (s), 1299 (w), 1246 (s), 1148 (s), 1021 (m), 997 (m), 907 (w), 870 (m), 837 (m), 727 (m), 608 (w), 527 (w) cm^{-1}; MS (EI) *m*/*z* (%): 218 ([M − $Me_2C=CH_2$]$^{+\bullet}$, 100%); HRMS (ES+) Found 299.1250 [M+Na]$^+$, $C_{16}H_{20}NaO_4$ requires 299.1254; X-Ray (thermal ellipsoids drawn at 35% probability level, selected hydrogens omitted for clarity).

Comparative Addition of an Organocerium Reagent to Cyclobutenedione 1b

*2-(tert-Butoxy)-4-hydroxy-3-methyl-4-phenylcyclobut-2-enone (**12a**) and 3-(tert-butoxy)-4-hydroxy-2-methyl-4-phenylcyclobut-2-enone (**10a**)*

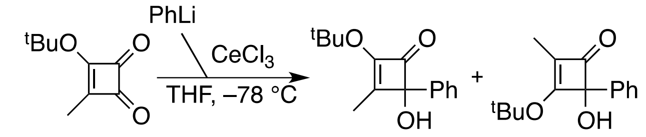

1b **12a**, 6% **10a**, 31%*

To a solution of $CeCl_3$ *(anhydrous beads)* (268 mg, 1.09 mmol) in THF (10 mL) at –78 °C was added PhLi (1.28 M in $^nBu_2O$, 938 μ L, 1.2 mmol) dropwise over 1 min. After 1 h a solution of cyclobutenedione **1b** (170 mg, 1.01 mmol) in THF (10 mL) was added dropwise over 10 min during which time the colour became pale yellow. After 1 h sat. $NaHCO_3$ (10 mL) was added then the reaction mixture was allowed to warm to RT and diluted with ether (15 mL). The aqueous phase was separated and extracted with ether (10 mL). The organic phases were combined, washed with brine (20 mL), dried over $MgSO_4$ and concentrated under reduced pressure to give a pale yellow oil (200 mg). Purification by flash column chromatography (0.5–2% ethyl acetate/chloroform) afforded firstly recovered starting material **1b** (33 mg, 0.20 mmol, 19%), then cyclobutenone **12a** (15 mg, 0.06 mmol, 41%) as a pale yellow oil (data as below), then cyclobutenone **10a** contaminated with *ca.* 10–15% of two unidentified side products (76 mg, 0.31 mmol, 31%*), as a colourless oil. ^{1}H NMR (300 MHz, $CDCl_3$): δ = 7.52–7.47 (2 H, m, Ar**H**), 7.47–7.33 (3 H, m, Ar**H**), 7.33–7.27 (1 H, m, Ar**H**), 1.84 (3 H, s, C**H$_3$**), 1.47 (9 H, s C(C**H$_3$**)$_3$) ppm, with additional signals from two unidentified byproducts (see spectra); ^{13}C NMR (100 MHz, $CDCl_3$): δ = 192.3 (**C**=O), 180.0 (**C**), 137.5 (**C**), 128.3 (2 x **C**H), 127.8 (**C**H), 125.7 (2 x **C**H), 124.2 (**C**), 92.9 (**C**), 84.3 (**C**), 28.7 (C(**C**H_3)$_3$), 8.2 (**C**H_3) ppm, with additional signals from the unidentified byproducts: 194.4 (**C**), 194.1 (**C**), 186.7 (**C**), 185.8 (**C**), 179.7 (**C**), 156.3 (**C**), 155.9 (**C**), 154.4 (**C**), 153.8 (**C**), 138.5 (**C**), 137.7 (**C**), 128.4 (**C**H), 128.3 (**C**H), 127.7 (**C**H), 127.7 (**C**H), 125.9 (**C**H), 125.8 (**C**H), 119.4 (s), 119.2 (s), 88.5 (**C**), 88.2 (**C**), 88.2 (**C**), 87.8 (**C**), 84.2 (**C**), 84.0 (**C**), 81.3 (**C**), 81.2 (**C**), 31.0 (**C**H_3), 29.7 (**C**H_3), 28.6 (**C**H_3), 28.6 (**C**H_3), 28.5 (**C**H_3), 28.5 (**C**H_3), 9.4 (**C**H_3), 9.1 (**C**H_3) ppm; IR: ν 3338 (br), 2983 (w), 2934 (w), 1749 (s), 1600 (s), 1450 (w), 1382 (s), 1345 (s), 1263 (w), 1149 (s), 1010 (w), 895 (w), 867 (w), 830 (w), 757 (w), 703 (m) cm^{-1}.

**The yield calculation is based on the mass obtained and does not take into account the impurities evidenced by NMR. Attempts to purify this material with successive flash column chromatographic separations proved fruitless.*

Thermochemical Rearrangements

*2-Methoxy-3-methylnaphthalene-1,4-dione (**15a**)*

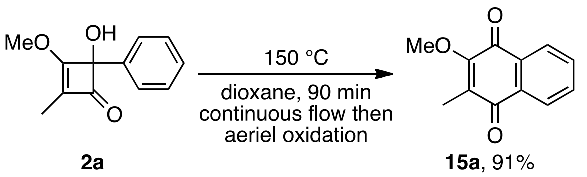

2a **15a**, 91%

Cyclobutenone **2a** (9.0 mg, 0.044 mmol) in dioxane (1 mL) was heated at 150 °C in stainless steel tubing under continuous flow for a residence time of 90 min using a Vapourtec R4/R2+ instrument. The resulting solution was concentrated under reduced pressure to a yellow solid (9 mg), which was then dissolved in $CHCl_3$ (5 mL) and stirred at RT under air for 24 h. Concentration under reduced pressure then gave a yellow solid (9.0 mg) that was purified by flash column chromatography (neat chloroform) to afford benzoquinone **15a** as a yellow crystalline solid (8.1 mg, 0.040 mmol, 91%).[MP 88–90 °C; ^{1}H NMR (400 MHz, $CDCl_3$): δ = 8.11–8.03 (2 H, m, Ar**H**), 7.73–7.67 (2 H, m, Ar**H**), 4.13 (3 H, s, OC**H$_3$**), 2.11 (3 H, s, C**H$_3$**) ppm; ^{13}C NMR (100 MHz, $CDCl_3$): δ = 185.8 (**C**=O), 181.3 (**C**=O), 157.8 (**C**), 133.7 (**C**H), 133.2 (**C**H), 132.0 (**C**), 131.8 (**C**), 131.5 (**C**), 126.2 (**C**H), 126.1 (**C**H), 61.0 (O**C**H_3), 9.3 (**C**H_3) ppm; IR: ν 2950 (w), 2922 (w), 2856 (w), 2362 (w), 2333 (w), 1749 (w), 1672 (s), 1618 (m), 1594 (m), 1459 (w), 1373 (w),

1328 (w), 1272 (s), 1214 (s), 1165 (m), 1083 (m), 1006 (w), 957 (m), 920 (m), 789 (w), 720 (s), 683 (w) cm^{-1}; MS (EI) *m/z* (%): 202 ($[M]^{+\bullet}$, 100%); HRMS (ES+) found 203.0699 $[M+H]^+$ and 427.1147 $[2M+Na]^+$, $C_{12}H_{11}O_3$ requires 203.0703 and $C_{24}H_{20}NaO_6$ requires 427.1152.

*2-Methoxy-3,6-dimethylnaphthalene-1,4-dione (**15b**)*

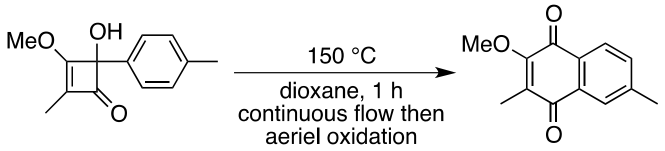

2b **15b**, 94%

Cyclobutenone **2b** (37 mg, 0.17 mmol) in dioxane (3 mL) was heated at 150 °C in stainless steel tubing under continuous flow for a residence time of 1 h using a Vapourtec R4/R2+ instrument. The crude material was stirred at RT under air for 24 h then concentration under reduced pressure to give **15b** as a yellow solid (35 mg, 0.16 mmol, 94%). MP 78–79 °C; ^{1}H NMR (300 MHz, $CDCl_3$): δ = 7.93 (1 H, d, *J* = 7.8 Hz, Ar**H**), 7.86 (1 H, s with fine splitting, Ar**H**), 7.47 (1 H, d with fine splitting, *J* = 7.9 Hz, Ar**H**), 4.12 (3 H, s O**CH₃**), 2.48 (3 H, s, C**H₃**), 2.09 (3 H, s, C**H₃**) ppm; ^{13}C NMR (75 MHz, $CDCl_3$): δ = 186.1 (**C**=O), 181.1 (**C**=O), 157.8 (**C**), 144.8 (**C**), 133.9 (**C**H), 131.9 (**C**), 131.5 (**C**), 129.2 (**C**), 126.6 (**C**H), 126.3 (**C**H), 61.0 (O**C**H₃), 21.8 (**C**H₃), 9.3 (**C**H₃) ppm; IR: ν 2922 (w), 2852 (w), 2362 (m), 2341 (w), 1761 (w), 1655 (s), 1590 (s), 1443 (w), 1369 (w), 1320 (s), 1267 (m), 1214 (m), 1010 (m), 1002 (w), 969 (w), 904 (s), 846 (m), 736 (s), 667 (w), 601 (w) cm^{-1}; MS (EI) *m/z* (%): 216 ($[M]^{+\bullet}$, 100%); HRMS (ES+) found 239.0673 $[M+Na]^+$ and 217.0854 $[M+H]^+$, $C_{13}H_{12}NaO_3$ requires 239.0679 and $C_{13}H_{13}O_3$ requires 217.0859.

*2,6-Dimethoxy-3-methylnaphthalene-1,4-dione (**15c**)*

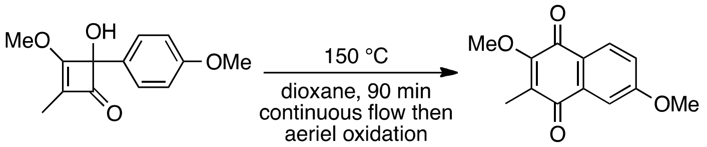

2c **15c**, 70%

Cyclobutenone **2c** (23 mg, 0.10 mmol) in dioxane (2 mL) was heated at 150 °C in stainless steel tubing under continuous flow for a residence time of 90 min using a Vapourtec R4/R2+ instrument. The resulting solution was concentrated under reduced pressure to give a bright yellow solid (19 mg). Purification by flash column chromatography (1% ethyl acetate/dichloromethane) afforded benzoquinone **15c** as a bright yellow solid (16 mg, 0.07 mmol, 70%). MP 111–113 °C; ^{1}H NMR (400 MHz, $CDCl_3$): δ = 7.99 (1 H, d, *J* = 8.6 Hz, Ar**H**), 7.51 (1 H, d, *J* = 2.7 Hz, Ar**H**), 7.14 (1 H, dd, *J* = 8.6, 2.7 Hz, Ar**H**), 4.12 (3 H, s, OC**H₃**), 3.94 (3 H, s, OC**H₃**), 2.08 (3 H, s, C**H₃**) ppm; ^{13}C NMR (100 MHz, $CDCl_3$): δ = 185.8 (**C**=O), 180.3 (**C**=O), 164.1 (**C**), 158.0 (**C**), 134.1 (**C**), 131.1 (**C**), 128.6 (**C**H), 124.9 (**C**), 119.6 (**C**H), 109.7 (**C**H), 61.1 (O**C**H₃), 55.9 (O**C**H₃), 9.2 (**C**H₃) ppm; IR: ν 2954 (w), 2840 (w), 2358 (s), 2346 (m), 2117 (w), 1659 (m), 1643 (w), 1594 (s), 1496 (w), 1439 (w), 1324 (s), 1239 (s), 1083 (m), 973 (m), 928 (w), 740 (m), 671 (w), 581 (w) cm^{-1}; MS (EI) *m/z* (%): 232 ($[M]^{+\bullet}$, 100%); HRMS (ES+) found 233.0805 $[M+H]^+$ and 255.0621 $[M+Na]^+$, $C_{13}H_{13}O_4$ requires 233.0808 and $C_{13}H_{12}NaO_4$ requires 255.0628.

*2-Methoxy-3-methyl-6-(trifluoromethyl)naphthalene-1,4-dione (**15d**)*

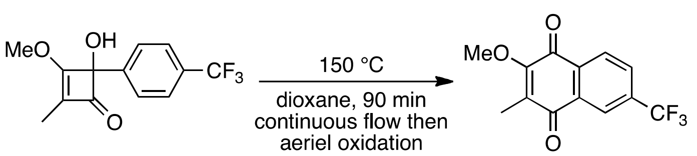

2d **15d**, 78%

Cyclobutenone **2d** (28 mg, 0.10 mmol) in dioxane (2 mL) was heated at 150 °C in stainless steel tubing under continuous flow for a residence time of 90 min using a Vapourtec R4/R2+ instrument. The resulting solution was concentrated under reduced pressure to a brown oil which was then dissolved in $CHCl_3$ (7 mL) and stirred at RT under air for 48 h. Concentration under reduced pressure then gave a yellow solid (27 mg) that was purified by flash column chromatography (1% ethyl acetate/chloroform) afforded benzoquinone **15d** as a bright yellow crystalline solid (22 mg, 0.08 mmol, 78%). MP 68–70 °C; ^{1}H NMR (400 MHz, $CDCl_3$): δ = 8.35 (1 H, s with fine splitting, Ar**H**), 8.18 (1 H, d with fine splitting, *J* = 8.1 Hz, Ar**H**), 7.94 (1 H, d with fine splitting, *J* = 8.1 Hz, Ar**H**), 4.17 (3 H, s, OC**H₃**), 2.14 (3 H, s, C**H₃**) ppm; ^{13}C NMR (100 MHz, $CDCl_3$): δ = 184.2 (**C**=O), 180.2 (**C**=O), 157.9 (**C**), 135.4 (**C**, q, $J_{C\text{-}F}$ = 33.3 Hz), 133.6 (**C**), 132.3 (**C**), 132.2 (**C**), 129.6 (**C**H, q, $J_{C\text{-}F}$ = 3.7 Hz), 126.9 (**C**H), 123.2 (**C**F₃, $J_{C\text{-}F}$ = 273 Hz), 23.4 (**C**H, q, $J_{C\text{-}F}$ = 3.7 Hz), 61.2 (O**C**H₃), 9.4 (**C**H₃) ppm; IR: ν 3730 (w), 3624 (w), 2950 (w), 2860

(w), 2362 (s), 2341 (m), 1672 (m), 1651 (m), 1606 (m), 1488 (m), 1373 (m), 1304 (s), 1214 (s), 1169 (s), 1112 (s), 1001 (m), 924 (s), 859 (s), 699 (m), 642 (m) cm^{-1}; MS (EI) *m/z* (%): 270 ($[M]^{+\bullet}$, 100%); HRMS (ES+) found 293.0392 $[M+Na]^+$, $C_{13}H_9F_3NaO_3$ requires 293.0396.

*2-(tert-Butoxy)-3-methylnaphthalene-1,4-dione (**16a**)*

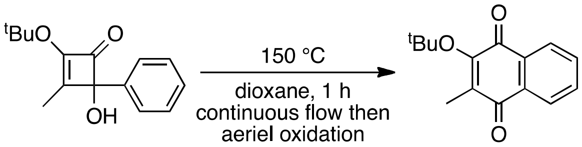

12a **16a**, 78%

Cyclobutenone **12a** (25 mg, 0.10 mmol) in dioxane (2 mL) was heated at 150 °C in stainless steel tubing under continuous flow for a residence time of 1 h using a Vapourtec R4/R2+ instrument. The resulting solution was concentrated under reduced pressure to give a brown oil then dissolved in $CHCl_3$ (5 mL) and stirred under air at RT overnight. The solution was concentrated under reduced pressure to a bright yellow oil (23 mg) then purified by flash column chromatography (0.2% ethyl acetate/dichloromethane) to afford benzoquinone **16a** as a bright yellow oil (17 mg, 0.07 mmol, 78%). ^1H NMR (300 MHz, $CDCl_3$): δ = 8.04–7.96 (2 H, m, Ar**H**), 7.65–7.60 (2 H, m, Ar**H**), 2.07 (3 H, s, C**H₃**), 1.39 (9 H, s, C(C**H₃**)**₃**); ^{13}C NMR (100 MHz, $CDCl_3$): δ = 186.3 (**C**=O), 182.9 (**C**=O), 156.9 (**C**), 137.3 (**C**), 133.5 (**C**H), 133.3 (**C**H), 132.2 (**C**), 131.6 (**C**), 126.4 (**C**H), 126.2 (**C**H), 84.9 (**C**), 29.6 (C(**C**H_3)$_3$), 11.1 (**C**H_3); IR: ν 2975 (w), 2922 (w), 2848 (w), 1786 (m), 1766 (m), 1672 (s), 1655 (s), 1602 (m), 1459 (w),1373 (m), 1333 (m), 1300 (m), 1267 (m), 1198 (w), 1149 (s), 1071 (m), 940 (w), 859 (w), 728 (m), 691 (w) cm^{-1}; MS (EI) *m/z* (%): 244 ($[M]^{+\bullet}$, 2%), 188 ($[M - {}^tBuH]^{+\bullet}$, 100%); HRMS (EI) Found 244.1098 $[M]^{+\bullet}$, $C_{15}H_{16}O_3$ requires 244.1099.

*3-(tert-Butoxy)-2,6-dimethylnaphthalene-1,4-dione (**16b**)*

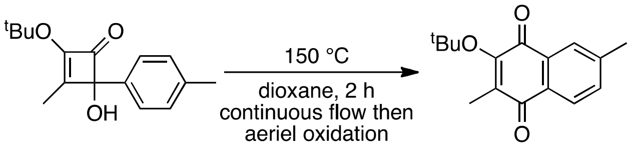

12b **16b**, 65%

Cyclobutenone **12b** (34 mg, 0.13 mmol) in dioxane (3 mL) was heated at 150 °C in stainless steel tubing under continuous flow for a residence time of 2 h using a Vapourtec R4/R2+ instrument. The resulting solution was concentrated under reduced pressure to a bright yellow oil (31 mg). Purification by flash column chromatography (neat chloroform) afforded benzoquinone **16b** directly as a pale yellow solid (25 mg, 0.10 mmol, 65%). MP 71–73 °C; ^1H NMR (400 MHz, $CDCl_3$): δ = 7.98 (1 H, d, *J* = 7.8 Hz, Ar**H**), 7.86 (1 H, s with fine splitting, Ar**H**), 7.50 (1 H, d with fine splitting, *J* = 7.9 Hz, Ar**H**), 2.48 (3 H, s C**H₃**), 2.13 (3 H, s, C**H₃**), 1.46 (9 H, s, C(C**H₃**)**₃**) ppm; ^{13}C NMR (100 MHz, $CDCl_3$): δ = 186.2 (**C**=O), 183.2 (**C**=O), 156.7 (**C**), 144.3 (**C**), 137.3 (**C**), 134.2 (**C**H), 131.5 (**C**), 129.9 (**C**), 126.8 (**C**H), 126.4 (**C**H), 84.7 (**C**), 29.6 (C(**C**H_3)$_3$), 21.7 (**C**H_3), 11.2 (**C**H_3) ppm; IR: ν 2976 (w), 2923 (w), 2359 (w), 1777 (w), 1761 (m), 1658 (s), 1600 (s), 1453 (w), 1368 (m), 1338 (m), 1305 (m), 1263 (m), 1186 (w), 1139 (s), 1075 (s), 984 (w), 952 (s), 887 (m), 855 (m), 811 (w), 761 (w), 732 (m), 687 (m), 659 (w) cm^{-1}; MS (EI) *m/z* (%): 258 ($[M]^{+\bullet}$, 2%), 202 (100%); HRMS (ES+) found 281.1150 $[M+Na]^+$ and 539.2406 $[2M+Na]^+$, $C_{16}H_{18}NaO_3$ requires 281.1148 and $C_{32}H_{36}NaO_6$ requires 539.2404.

*3-(tert-Butoxy)-6-methoxy-2-methylnaphthalene-1,4-dione (**16c**)*

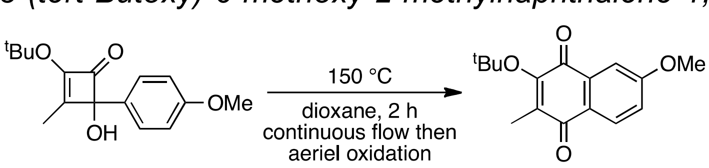

12c **16c**, 75%

Cyclobutenone **12c** (55 mg, 0.20 mmol) in dioxane (4 mL) was heated at 150 °C in stainless steel tubing under continuous flow for a residence time of 2 h using a Vapourtec R4/R2+ instrument. The resulting solution was concentrated under reduced pressure then purified by flash column chromatography (1% ethyl acetate/dichloromethane with 1% aq. NH_3) to afford benzoquinone **16c** (40 mg, 0.15 mmol, 75%) as a bright yellow oil. ^1H NMR (400 MHz, $CDCl_3$): δ = 8.02 (1 H, d, *J* = 8.7 Hz, Ar**H**), 7.50 (1 H, d, *J* = 2.7 Hz, Ar**H**), 7.16 (1 H, dd, *J* = 8.6, 2.7 Hz, Ar**H**), 3.93 (3 H, s, OC**H₃**), 2.12 (3 H, s, C**H₃**), 1.46 (9 H, s C(C**H₃**)**₃**) ppm; ^{13}C NMR (100 MHz, $CDCl_3$): δ = 185.4 (**C**=O), 182.9 (**C**=O), 163.8 (**C**), 156.5 (**C**), 135.2 (**C**), 133.5 (**C**), 128.5 (**C**H), 125.7 (**C**), 120.1 (**C**H), 109.7 (**C**H), 84.6 (**C**), 55.8 (O**C**H_3), 29.5 (C(**C**H_3)$_3$), 11.2 (**C**H_3) ppm; IR: ν 3726 (w), 3698 (w), 3624 (w), 3591 (w), 2970 (w), 2917 (w), 2844 (w), 2366 (m), 2333 (m), 1777 (w), 1675 (m), 1655 (m), 1593 (s), 1491 (w), 1462 (w), 1360 (w), 1303 (s), 1279 (s),

1225 (m), 1144 (s), 1074 (m), 1025 (m), 948 (m), 882 (w), 837 (w), 768 (w), 739 (w), 686 (w), 613 (w) cm^{-1}; MS (EI) *m/z* (%): 270 ($[M - CH_3]^{+\bullet}$, 1%) 218 ($[M - Me_2C{=}CH_2]^{+\bullet}$, 100%).

*2-(tert-Butoxy)-3-methyl-6-(trifluoromethyl)naphthalene-1,4-dione (**16d**)*

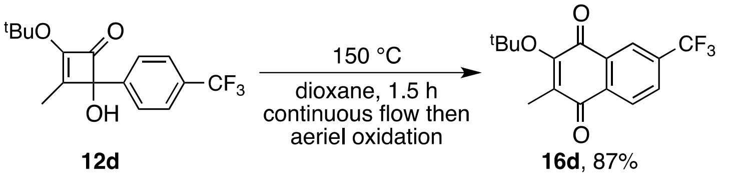

12d **16d**, 87%

Cyclobutenone **12d** (29 mg, 0.09 mmol) in dioxane (2 mL) was heated at 150 °C in stainless steel tubing under continuous flow for a residence time of 90 min using a Vapourtec R4/R2+ instrument. The resulting solution was concentrated under reduced pressure to a brown oil then dissolved in $CHCl_3$ (7 mL), stirred at RT under air for 24 h then concentrated under reduced pressure to give benzoquinone **16d** (25 mg, 0.08 mmol, 87%) as a yellow solid. MP 135–137 °C; ^{1}H NMR (300 MHz, $CDCl_3$): δ = 8.35 (1 H, s with fine splitting, Ar**H**), 8.23 (1 H, d with fine splitting, *J* = 8.0 Hz, Ar**H**), 7.96 (1 H, d with fine splitting, *J* = 8.0 Hz, Ar**H**), 2.18 (3 H, s, C**H₃**), 1.49 (3 H, s, C(C**H₃**)₃) ppm; ^{13}C NMR (75 MHz, $CDCl_3$): δ = 185.1 (**C**=O), 181.6 (**C**=O), 157.3 (**C**), 137.8 (2 x **C**), 135.1 (**C**, q, $J_{C\text{-}F}$ = 33.6 Hz), 131.9 (**C**), 130.0 (**C**H, q, $J_{C\text{-}F}$ = 3.3 Hz), 127.0 (**C**H), 123.6 (**C**H, q, $J_{C\text{-}F}$ = 3.9 Hz), 123.2 (**C**F₃, $J_{C\text{-}F}$ = 274.1 Hz), 85.6 (**C**), 29.6 (C(**C**H₃)₃), 11.2 (**C**H₃) ppm; IR: ν 2909 (w), 2844 (w), 2358 (m), 2337 (w), 1737 (w), 1655 (m), 1614 (w), 1337 (w), 1287 (m), 1247 (m), 1165 (m), 1120 (s), 1067 (m), 953 (m), 871 (m), 740 (w), 699 (m) cm^{-1}; MS (EI) *m/z* (%): 312 ($[M]^{+\bullet}$, 2%), 57 (100%); HRMS (ES+) found 335.0862 $[M+Na]^+$, $C_{16}H_{15}F_3NaO_3$ requires 335.0866.

*2-(tert-Butoxy)-5-methoxy-3-methylnaphthalene-1,4-dione (**16e**)*

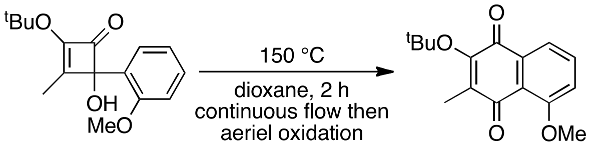

12e **16e**, 53%

Cyclobutenone **12e** (107 mg, 0.20 mmol) in dioxane (7.7 mL) was heated at 150 °C in stainless steel tubing under continuous flow for a residence time of 2 h using a Vapourtec R4/R2+ instrument. The resulting solution was concentrated under reduced pressure to give a bright yellow oil (102 mg). Purification by flash column chromatography (0.2–1% ethyl acetate/dichloromethane with 1% NH_4OH) afforded benzoquinone **16e** directly as a bright yellow crystalline solid (53 mg, 0.19 mmol, 53%). MP 76–78 °C; ^{1}H NMR (400 MHz, $CDCl_3$): δ = 7.72 (1 H, dd, *J* = 7.7, 1.1 Hz, Ar**H**), 7.62 (1 H, dd, *J* = 8.3, 7.6 Hz, Ar**H**), 7.26 (1 H, dd, *J* = 8.5, 1.0 Hz, Ar**H**), 3.99 (3 H, s, OC**H₃**), 2.10 (3 H, s, C**H₃**), 1.43 (9 H, s, C(C**H₃**)₃) ppm; ^{13}C NMR (100 MHz, $CDCl_3$): δ = 185.6 (**C**=O), 183.0 (**C**=O), 159.2 (**C**), 154.9 (**C**), 139.0 (**C**), 134.2 (**C**H), 133.8 (**C**), 120.0 (**C**), 119.1 (**C**H), 117.6 (**C**H), 84.1 (**C**), 56.4 (O**C**H₃), 29.5 (C(**C**H₃)₃), 11.3 (**C**H₃) ppm; IR: ν 3722 (w), 3698 (w), 3628 (w), 3591 (w), 2975 (w), 2938 (w), 2840 (w), 2362 (s), 2340 (m), 1664 (m), 1648 (m), 1620 (m), 1583 (m), 1475 (w), 1270 (m), 1237 (w), 1153 (m), 1105 (m), 1063 (m), 985 (w), 963 (m), 859 (w), 784 (w), 736 (w), 690 (w) cm^{-1}; MS (ES+) *m/z* 297 ($[M+H]^+$, 2%), 338 ($[M+MeCN+Na]^+$, 1%), 417 (100%), 571 ($[2M+Na]^+$, 87%); HRMS (ES^+): found 297.1096 $[M+Na]^+$, $C_{16}H_{18}NaO_4$ requires 297.1097.

*3-(tert-Butoxy)-1-hydroxy-2-methyl-4H-quinolizin-4-one (**16f**)*

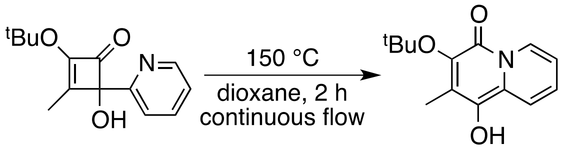

12f **16f**, 65%

Cyclobutenone **12f** (164 mg, 0.66 mmol) in dioxane (13 mL) was heated at 150 °C in stainless steel tubing under continuous flow for a residence time of 2 h using a Vapourtec R4/R2+ instrument. The resulting solution was concentrated under reduced pressure to give a dark green/black solid (170 mg). Purification by flash column chromatography (5–30% ethyl acetate/dichloromethane) afforded quinolizinone **16f** as a black oil (107 mg, 0.43 mmol, 65%). ^{1}H NMR (400 MHz, $CDCl_3$): δ = 8.60 (1 H, ddd, *J* = 4.9, 1.6, 0.9 Hz, Ar**H**), 7.81 (1 H, ddd, *J* = 8.0, 7.7, 1.7 Hz, Ar**H**), 7.41 (1 H, ddd, *J* = 7.5, 4.9, 1.0 Hz, Ar**H**), 7.26 (1 H, dt, *J* = 7.9, 1.0 Hz, Ar**H**), 7.23 (1 H, br s, O**H**), 1.75 (3 H, s, C**H₃**), 1.47 (9 H, s, C(C**H₃**)₃) ppm; ^{13}C NMR (100 MHz, $CDCl_3$): δ = 168.6 (**C**=O), 153.5 (**C**), 147.8 (**C**H), 145.8 (**C**), 141.1 (**C**), 138.3 (**C**H), 124.8 (**C**H), 119.8 (**C**H), 101.4 (**C**), 83.3 (**C**), 29.0 (C(**C**H₃)₃), 9.7 (**C**H₃) ppm; IR: ν 3730 (w), 3698 (w), 3624 (w), 3600 (w), 2975 (m), 2774 (w), 2631 (w), 2354 (s), 2337 (s), 1765 (s), 1684 (m), 1586 (m), 1431 (m), 1394 (m), 1373 (m), 1324 (w), 1259 (m), 1165 (m), 1132 (s), 1047 (m), 949

(s), 891 (m), 797 (m), 667 (m), 618 (m) cm^{-1}; MS (ES+) *m*/*z* 248 ($[M+H]^+$, 1%), 270 ($[M+Na]^+$, 1%), 390 (100%).

*2-(tert-Butoxy)-3,6-dimethylnaphthalene-1,4-dione (**16g**) and 2-(tert-butoxy)-3,8-dimethylnaphthalene-1,4-dione (**16i**)*

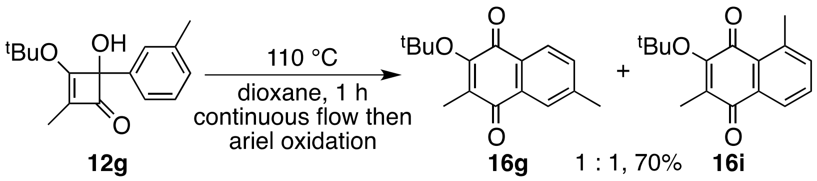

Cyclobutenone **12g** (26 mg, 0.10 mmol) in dioxane (2 mL) was heated at 110 °C in stainless steel tubing under continuous flow for a residence time of 1 h using a Vapourtec R4/R2+ instrument. The resulting solution was concentrated under reduced pressure to give a bright yellow oil (24 mg). Purification by flash column chromatography (5–15% ethyl acetate/petroleum ether) afforded a 1 : 1 mixture of benzoquinones **16g** and **16i** as a bright yellow oil (18 mg, 0.07 mmol, 70%). ^{1}H NMR (400 MHz, $CDCl_3$): δ = 8.02 (1 H, d with fine splitting, *J* = 7.6 Hz, Ar**H**), 7.96 (1 H, d, *J* = 7.8 Hz, Ar**H**), 7.88 (1 H, s with fine splitting, Ar**H**), 7.55 (1 H, app t, *J* = 7.6 Hz, Ar**H**), 7.51–7.47 (2 H, m, 2 x Ar**H**), 2.74 (3 H, s, C**H₃**), 2.49 (3 H, s, C**H₃**), 2.14 (3 H, s, C**H₃**), 2.11 (3 H, s, C**H₃**), 1.47 (9 H, s, C(C**H₃**)₃), 1.46 (9 H, s, C(C**H₃**)₃) ppm; ^{13}C NMR (100 MHz, $CDCl_3$): δ = 186.6 (2 x **C**=O), 184.9 (**C**=O), 182.8 (**C**=O), 157.7 (**C**), 156.9 (**C**), 144.6 (**C**), 140.7 (**C**), 137.1 (**C**H), 137.0 (**C**), 135.2 (**C**), 134.0 (**C**H), 133.7 (**C**), 132.6 (**C**H), 132.1 (**C**), 129.5 (**C**), 129.3 (**C**), 126.6 (**C**H), 126.5 (**C**H), 125.0 (**C**H), 84.8 (**C**), 84.5 (**C**), 29.6 (C(**C**H_3)$_3$), 29.5 (C(**C**H_3)$_3$), 22.5 (**C**H_3), 21.8 (**C**H_3), 11.1 (**C**H_3), 10.8 (**C**H_3) ppm; IR: ν 2979 (w), 2922 (w), 2366 (w), 2337 (w), 1667 (m), 1651 (m), 1598 (w), 1373 (w), 1320 (w), 1267 (w), 1153 (s), 1104 (w), 1079 (w), 957 (m), 728 (w) cm^{-1}; MS (EI) *m*/*z* 258 ($[M]^{+\bullet}$, 1%), 202 (100%).

*2-(tert-Butoxy)-3-methyl-6-(trifluoromethyl)naphthalene-1,4-dione (**16h**)*

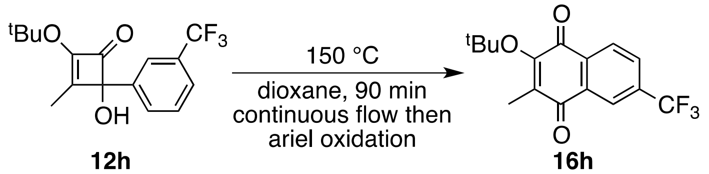

Cyclobutenone **12h** (35 mg, 0.11 mmol) in dioxane (2 mL) was heated at 150 °C in stainless steel tubing under continuous flow for a residence time of 90 min using a Vapourtec R4/R2+ instrument. The resulting solution was concentrated under reduced pressure to give a bright yellow solid (32 mg). Purification by flash column chromatography (neat chloroform) afforded benzoquinone **16h** directly as a bright yellow solid (28 mg, 0.09 mmol, 82%). MP 76–78 °C; ^{1}H NMR (400 MHz, $CDCl_3$): δ = 8.37 (1 H, s with fine splitting, Ar**H**), 8.20 (1 H, d with fine splitting, *J* = 8.1 Hz, Ar**H**), 7.96 (1 H, d with fine splitting, *J* = 8.0 Hz, Ar**H**), 2.18 (3 H, s, C**H₃**), 1.48 (9 H, s, C(C**H₃**)₃) ppm; ^{13}C NMR (100 MHz, $CDCl_3$): δ = 184.9 (**C**=O), 181.8 (**C**=O), 157.2 (**C**), 138.0 (**C**), 135.2 (**C**, q, J_{C-F} = 33.3 Hz), 133.7 (**C**), 132.5 (**C**), 129.8 (**C**H, q, J_{C-F} = 4.0 Hz), 127.1 (**C**H), 123.5 (**C**H, q, J_{C-F} = 3.7 Hz), 85.6 (**C**), 29.6 (C(**C**H_3)$_3$), 11.2 (**C**H_3) ppm [**C**F_3 not observed]; ^{19}F NMR (282 MHz, $CDCl_3$) −63.29 (3F, s, C**F₃**) IR: ν 2979 (w), 2917 (w), 2844 (w), 2366 (w), 2346 (w), 1790 (w), 1676 (m), 1663 (m), 1610 (w), 1316 (w), 1296 (m), 1169 (w), 1145 (s), 1079 (w), 957 (w), 769 (m) cm^{-1}; MS (EI) *m*/*z* 312 ($[M]^{+\bullet}$, 2%), 57 (100%); HRMS (ES^+): found 312.0987 $[M]^{+\bullet}$, $C_{16}H_{15}O_3F_3$ requires 312.0973.

*2-(tert-Butoxy)-3,5-dimethylbenzene-1,4-diol (**23**) and 2-(tert-butoxy)-3,5-dimethylcyclohexa-2,5-diene-1,4-dione (**58**)*

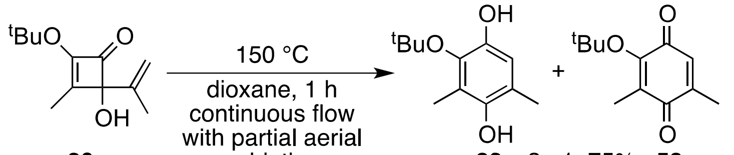

20 **23** 3 : 1, 75% **58**

Cyclobutenone **20** (25 mg, 0.12 mmol) in dioxane (2 mL) was heated at 150 °C in stainless steel tubing under continuous flow for a residence time of 1 h using a Vapourtec R4/R2+ instrument. The resulting solution was concentrated under reduced pressure to a dark yellow oil (29 mg). This was dissolved in chloroform (10 mL) and stirred at RT under air for 48 h. Purification by flash column chromatography (0.2% ethyl acetate in dichloromethane) afforded an inseparable 3 : 1 mixture of hydroquinone **23** and the corresponding quinone **58** as an orange oil (18 mg, 0.09 mmol, 75%). ^{1}H NMR (400 MHz, $CDCl_3$): δ = *signals attributed to hydroquinone **23**:* 6.60 (1 H, s with fine splitting, C**H**), 5.07 (1 H, s, O**H**), 4.24 (1 H, s, O**H**), 2.19 (3 H, d, *J* = 0.6 Hz, C$\mathbf{H_3}$), 2.18 (3 H, d, *J* = 0.4 Hz, C$\mathbf{H_3}$), 1.39 (9 H, s, C(C$\mathbf{H_3}$)$_3$); *signals attributed to quinone **58**:* 6.49 (1 H, q, *J* = 1.6 Hz, C**H**), 2.05 (3 H, d, *J* = 1.6 Hz, C$\mathbf{H_3}$), 2.00 (3 H, s, C$\mathbf{H_3}$), 1.40 (9 H, s, C(C$\mathbf{H_3}$)$_3$) ppm; ^{13}C NMR (100 MHz, $CDCl_3$): δ = *signals attributed to hydroquinone **23**:* 145.5 (**C**), 144.3 (**C**), 139.5 (**C**), 119.4 (**C**), 118.9 (**C**), 113.6 (**C**H), 82.1 (**C**), 29.3 (C(**C**H_3)$_3$), 15.7 (**C**H_3), 11.9 (**C**H_3); *signals attributed to quinone **32**:* 189.1 (**C**=O), 185.1 (**C**=O), 154.5 (**C**), 145.6 (**C**), 134.7 (**C**), 131.8 (**C**H), 84.4 (**C**), 29.5 (C(**C**H_3)$_3$), 15.8 (**C**H_3), 10.7 (**C**H_3) ppm; IR: ν 3448 (br w), 2974 (m), 2922 (w), 2366 (s), 2341 (s), 1651 (s), 1602 (m), 1480 (s), 1365 (m), 1316 (w), 1173 (s), 1149 (s), 1104 (s), 1038 (m), 949 (w), 887 (m), 821 (w), 752 (w), 658 (w) cm^{-1}; MS (EI) *m*/*z* 196 ([M]$^{+\bullet}$, 1%), 140 ([M − Me_2C=CH_2]$^{+\bullet}$, 100%); (ES$^+$): found 231.0988 [M+Na]$^+$, $C_{12}H_{16}NaO_3$ requires 231.0992.

*4-(tert-Butoxy)-3-methyl-[1,1'-biphenyl]-2,5-diol (**24**)*

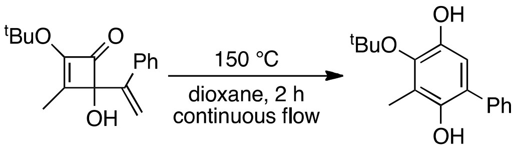

21a **24**, 98%

Cyclobutenone **21a** (98 mg, 0.36 mmol) in dioxane (7 mL) was heated at 150 °C in stainless steel tubing under continuous flow for a residence time of 2 h using a Vapourtec R4/R2+ instrument. The resulting solution was concentrated under reduced pressure to give hydroquinone **24** as a bright yellow oil (96 mg, 0.35 mmol, 98%). ^{1}H NMR (400 MHz, $CDCl_3$): δ = 7.54–7.43 (4 H, m, Ar**H**), 7.43–7.33 (1 H, m, Ar**H**), 6.73 (1 H, s, C**H**), 5.24 (1 H, br s, O**H**), 5.00 (1 H, br s, O**H**), 2.25 (3 H, s, C$\mathbf{H_3}$), 1.46 (9 H, s, C(C$\mathbf{H_3}$)$_3$) ppm; ^{13}C NMR (100 MHz, $CDCl_3$): δ = 144.6 (**C**), 144.3 (**C**), 141.6 (**C**), 137.4 (**C**), 129.3 (2 x **C**H), 129.1 (2 x **C**H), 127.7 (**C**H), 123.4 (**C**), 120.6 (**C**), 112.8 (**C**H), 82.7 (**C**), 29.4 (C(**C**H_3)$_3$), 12.3 (**C**H_3) ppm; IR: ν 3530 (br w), 3452 (br w), 2979 (w), 2926 (w), 2856 (w), 1651 (w), 1598 (w), 1471 (m), 1365 (w), 1234 (w), 1145 (s), 1059 (m), 1014 (w), 944 (w), 851 (m), 814 (w), 757 (m), 695 (m), 667 (w) cm^{-1}; MS (EI) *m*/*z* 272 ([M]$^{+\bullet}$, 2%), 216 ([M − ^{t}BuH]$^{+\bullet}$, 100%); HRMS (ES$^+$): found 295.1298 [M+Na]$^+$, $C_{17}H_{20}NaO_3$ requires 295.1305.

*2-(tert-Butoxy)-3-methylbenzene-1,4-diol (**25**)*

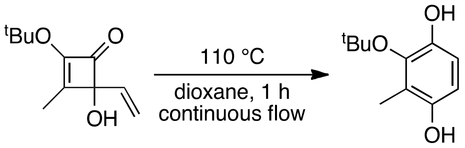

22 **25**, 100%

Cyclobutenone **22** (17 mg, 0.09 mmol) in dioxane (2 mL) was heated at 110 °C in stainless steel tubing under continuous flow for a residence time of 1 h using a Vapourtec R4/R2+ instrument. The resulting solution was concentrated under reduced pressure to give hydroquinone **25** as a pale yellow solid (17 mg, 0.09 mmol, 100%). MP 150–153 °C (diethyl ether/hexane); ^{1}H NMR (400 MHz, $CDCl_3$): δ = 6.68 (1 H, dd, *J* = 8.6, 0.6 Hz, C**H**), 6.49 (1 H, d, *J* = 8.6 Hz, C**H**), 5.12 (1 H, s, O**H**), 4.31 (1 H, s, O**H**), 2.18 (3 H, s, C$\mathbf{H_3}$), 1.41 (9 H, s, C(C$\mathbf{H_3}$)$_3$) ppm; ^{13}C NMR (100 MHz, $CDCl_3$): δ = 147.5 (**C**), 144.9 (**C**), 141.8 (**C**), 120.1 (**C**), 111.8 (**C**H), 110.5 (**C**H), 82.6 (**C**), 29.3 (C(**C**H_3)$_3$), 11.8 (**C**H_3) ppm; IR: ν 3730 (w), 3698 (w), 3624 (w), 3591 (w), 3375 (w), 3232 (w), 2917 (s), 2852 (m), 2358 (s), 2346 (s), 1729 (m), 1655 (w), 1594 (m), 1500 (m), 1463 (m), 1398 (w), 1365 (w), 1320 (s), 1259 (w), 1153 (m), 1067 (s), 1010 (w), 903 (m), 818 (m), 765 (m), 732 (m), 683 (w), 605 (w) cm^{-1}; MS (EI) *m*/*z* 196 ([M]$^{+\bullet}$, 1%), 140 ([M − Me_2C=CH_2]$^{+\bullet}$, 100%); HRMS (ES$^+$): found 415.2083 [2M+Na]$^+$, $C_{22}H_{32}NaO_6$ requires 415.2091.

*4-Methoxy-3-methyl-[1,1'-biphenyl]-2,5-dione (**35**) and*
*(E)-2-benzylidene-4-methoxy-5-methylcyclopent-4-ene-1,3-dione (**36**)*

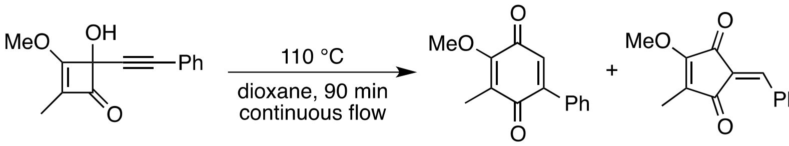

32 **35**, 52% **36**, 40%

Cyclobutenone **32** (20 mg, 0.09 mmol) in dioxane (2 mL) was heated at 110 °C in stainless steel tubing under continuous flow for a residence time of 90 min using a Vapourtec R4/R2+ instrument. The resulting solution was concentrated under reduced pressure to give an orange-yellow oil (18 mg). Purification by flash column chromatography (5% ether/petroleum ether) afforded firstly quinone **35** (10 mg, 0.05 mmol, 52%) as a yellow oil: ^{1}H NMR (400 MHz, $CDCl_3$): δ = 7.49–7.40 (5 H, m, Ar**H**), 6.70 (1 H, s, C**H**), 4.09 (3 H, s, OC**H$_3$**), 2.04 (3 H, s, C**H$_3$**) ppm; ^{13}C NMR (100 MHz, $CDCl_3$): δ = 187.4 (**C**=O), 183.6 (**C**=O), 155.5 (**C**), 145.9 (**C**), 133.1 (**C**), 131.2 (**C**H), 129.9 (**C**H), 129.4 (2 x **C**H), 129.0 (**C**), 128.4 (2 x **C**H), 60.9 (O**C**H$_3$), 9.2 (**C**H$_3$) ppm; IR: ν 2922 (w), 2856 (w), 2362 (s), 2337 (m), 1753 (w), 1647 (s), 1598 (w), 1447 (w), 1386 (w), 1320 (w), 1255 (w), 1206 (w), 1149 (w), 891 (w), 781 (w), 703 (w) cm^{-1}; MS (EI) *m/z* 228 ([M]$^{+\bullet}$, 100%); HRMS (ES$^+$): found 251.0675 [M+Na]$^+$ and 229.0857 [M+H]$^+$, $C_{14}H_{12}NaO_3$ requires 251.0679 and $C_{14}H_{13}O_3$ requires 229.0859; then cyclopentenedione **36** (8 mg, 0.04 mmol, 40%) an off-white solid: MP 114–116 °C; ^{1}H NMR (400 MHz, $CDCl_3$): δ = 8.31–8.25 (2 H, m, Ar**H**), 7.51–7.43 (3 H, m, Ar**H**), 7.41 (1 H, s, C**H**), 4.38 (3 H, s, OC**H$_3$**), 2.07 (3 H, s, C**H$_3$**) ppm; ^{13}C NMR (100 MHz, $CDCl_3$): δ = 189.8 (**C**=O), 188.6 (**C**=O), 164.6 (**C**), 139.2 (**C**H), 135.3 (**C**), 133.3 (2 x **C**H), 132.9 (**C**), 131.8 (**C**H), 128.6 (2 x **C**H), 126.4 (**C**), 59.6 (O**C**H$_3$), 7.4 (**C**H$_3$) ppm; IR: ν 2962 (w), 2917 (w), 2848 (w), 2366 (w), 2337 (w), 1737 (w), 1672 (s), 1627 (s), 1459 (w), 1386 (m), 1328 (m), 1251 (w), 1198 (w), 1146 (m), 1087 (w), 1010 (w), 793 (w), 777 (w), 732 (w), 695 (w), 569 (w) cm^{-1}; MS (EI) *m/z* 228 ([M]$^{+\bullet}$, 100%); HRMS (ES$^+$): found 251.0676 [M+Na]$^+$ and 229.0859 [M+H]$^+$, $C_{14}H_{12}NaO_3$ requires 251.0679 and $C_{14}H_{13}O_3$ requires 229.0859.

*(Z)-2-Benzylidene-4-(tert-butoxy)-5-methylcyclopent-4-ene-1,3-dione (**37**)*

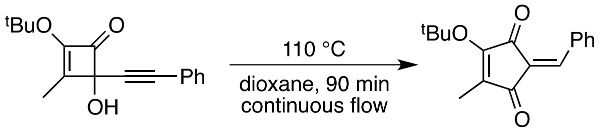

31a **33**, 90%

Cyclobutenone **34** (27 mg, 0.1 mmol) in dioxane (2 mL) was heated at 110 °C in stainless steel tubing under continuous flow for a residence time of 90 min using a Vapourtec R4/R2+ instrument. The resulting solution was concentrated under reduced pressure to give cyclopentenedione **37** as a yellow solid (24 mg, 0.09 mmol, 90%). MP 80–81 °C; ^{1}H NMR (400 MHz, $CDCl_3$): δ = 8.31–8.26 (2 H, m, Ar**H**), 7.50–7.44 (3 H, m, Ar**H**), 7.42 (1 H, s, C**H**), 2.02 (3 H, s, C**H$_3$**), 1.57 (9 H, s, C(C**H$_3$**)$_3$) ppm; ^{13}C NMR (100 MHz, $CDCl_3$): δ = 191.9 (**C**=O), 188.1 (**C**=O), 168.9 (**C**), 140.9 (**C**), 139.3 (**C**H), 133.1 (2 x **C**H), 133.0 (**C**), 131.8 (**C**H), 128.6 (2 x **C**H), 126.5 (**C**), 85.7 (**C**), 29.6 (C(**C**H$_3$)$_3$), 7.9 (**C**H$_3$) ppm; IR: ν 2983 (w), 2913 (w), 1733 (w), 1684 (s), 1623 (s), 1455 (w), 1365 (m), 1328 (s), 1251 (w), 1161 (m), 1112 (s), 1075 (s), 985 (m), 830 (w), 769 (m), 683 (m) cm^{-1}; MS (EI) *m/z* 213 ([M − C_4H_9]$^{+\bullet}$, 100%); HRMS (ES$^+$): found 293.1146 [M+Na]$^+$ and 215.0700 [M+H–C_4H_8]$^+$, $C_{17}H_{18}NaO_3$ requires 293.1148 and $C_{13}H_{11}O_3$ requires 215.0703.

ADDITIONAL EXPERIMENTS AS NOTED ON PAGE S1 AND IN REFERENCE 7 OF THE ARTICLE:

*(E)-2-Benzylidene-4-(tert-butoxy)-5-methylcyclopent-4-ene-1,3-dione (**53**) and*
*4-(tert-butoxy)-3-methyl-[1,1'-biphenyl]-2,5-dione (**54**)*

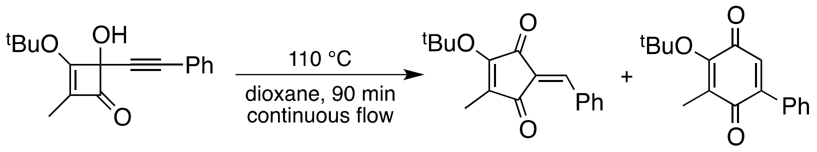

33 **53** 5 : 4, 95% **54**

Cyclobutenone **33** (63 mg, 0.23 mmol) in dioxane (4.7 mL) was heated at 110 °C in stainless steel tubing under continuous flow for a residence time of 90 min using a Vapourtec R4/R2+ instrument. The resulting solution was concentrated under reduced pressure to give an 5 : 4 mixture of cyclopentenedione **53** and quinone **54** (59.6 mg, 0.023 mmol, 95%) as a bright yellow solid. MP 116–118 °C; ^{1}H NMR

(400 MHz, $CDCl_3$): δ = 8.32–8.24 (2 H, m, Ar**H**), 7.50–7.42 (8 H, m, Ar**H**), 7.41 (1 H, s, C**H**, **34a**), 6.74 (1 H, s, C**H**, **34b**), 2.07 (3 H, s, C**H₃**), 2.02 (3 H, s, C**H₃**), 1.56 (9 H, s, C(C**H₃**)₃), 1.45 (8 H, s, C(C**H₃**)₃) ppm; ^{13}C NMR (100 MHz, $CDCl_3$): δ = 190.2 (**C**=O), 189.4 (**C**=O), 187.8 (**C**=O), 185.0 (**C**=O), 165.9 (**C**), 154.5 (**C**), 145.8 (**C**), 144.6 (**C**), 139.0 (**C**H), 134.9 (**C**), 133.1 (2 x **C**H), 133.0 (**C**), 132.9 (**C**), 131.7 (**C**H), 131.5 (**C**H), 129.8 (**C**H), 129.3 (2 x **C**H), 128.5 (2 x **C**H), 128.3 (2 x **C**H), 126.8 (**C**), 85.2 (**C**), 84.6 (**C**), 29.5 (C(**C**H₃)₃), 29.4 (C(**C**H₃)₃), 10.9 (**C**H₃), 7.8 (**C**H₃) ppm; IR: ν 2983 (w), 2922 (w), 2840 (w), 2362 (w), 2341 (w), 1725 (w), 1680 (s), 1647 (s), 1627 (s), 1602 (s), 1447 (w), 1365 (m), 1324 (m), 1251 (m), 1147 (s), 1116 (s), 985 (w), 957 (w), 900 (w), 846 (w), 797 (w), 781 (w), 744 (w), 683 (m), 634 (w) cm^{-1}; MS (EI) *m/z* 270 ([M]$^{+\bullet}$, 1%), 214 ([M − $Me_2C{=}CH_2$]$^{+\bullet}$, 100%); HRMS (ES$^+$): found 293.1149 [M+Na]$^+$ $C_{17}H_{18}NaO_3$ requires 293.1148.

*3-Methoxy-4-methyl-[1,1'-biphenyl]-2,5-diol (**51**) and 3-methoxy-4-methyl-[1,1'-biphenyl]-2,5-dione (**52**)*

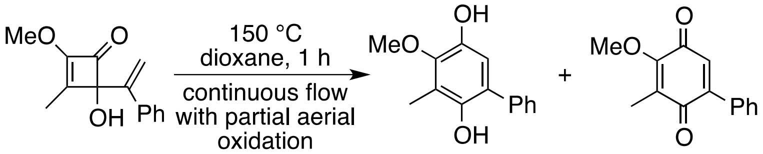

21b **51**, 56% **52**, 17%

Cyclobutenone **21b** (18 mg, 0.08 mmol) in dioxane (2 mL) was heated at 110 °C in stainless steel tubing under continuous flow for a residence time of 1 h using a Vapourtec R4/R2+ instrument. The resulting solution was concentrated under reduced pressure to give a brown oil (18 mg), which was dissolved in $CHCl_3$ (7 mL) and stirred at RT for 72 h. Concentration under reduced pressure then gave an orange oil (15 mg) which was purified by flash column chromatography (1–5% ethyl acetate/chloroform) to afford firstly quinone **52** (3 mg, 0.01 mmol, 17%) as a orange crystalline solid; ^{1}H NMR (400 MHz, $CDCl_3$): δ = 7.49–7.41 (5 H, m, Ar**H**), 6.70 (1 H, s, C**H**), 4.09 (3 H, s, OC**H₃**), 2.04 (3 H, s, C**H₃**) ppm; ^{13}C NMR (100 MHz, $CDCl_3$): δ = 133.0 (**C**), 131.2 (**C**H), 129.9 (**C**H), 129.4 (2 x **C**H), 128.4 (2 x **C**H), 60.9 (O**C**H₃), 9.1 (**C**H₃) ppm [4 **C** not observed]; IR: ν 2922 (w), 2848 (w), 2362 (w), 2337 (w), 1741 (w), 1651 (s), 1598 (m), 1451 (w), 1377 (w), 1328 (w), 1255 (w), 1210 (m), 1157 (m), 1083 (w), 895 (w), 793 (w), 777 (w), 699 (m), 626 (w); MS (EI) *m/z* (%): 228 ([M]$^{+\bullet}$, 6%), 230 (100%); then hydroquinone **51** (10 mg, 0.04 mmol, 56%) as a yellow oil; ^{1}H NMR (400 MHz, $CDCl_3$): δ = 7.53–7.38 (5 H, m, Ar**H**), 6.73 (1 H, d, *J* = 0.5 Hz, C**H**), 5.28 (1 H, s, O**H**), 4.97 (1 H, s, O**H**), 3.84 (3 H, s, OC**H₃**), 2.27 (3 H, s, C**H₃**) ppm; ^{13}C NMR (100 MHz, $CDCl_3$): δ = 145.5 (**C**), 144.4 (**C**), 142.4 (**C**), 137.2 (**C**), 129.3 (2 x **C**H), 129.1 (2 x **C**H), 127.8 (**C**H), 123.7 (**C**), 118.0 (**C**), 113.1 (**C**H), 61.0 (O**C**H₃), 9.7 (**C**H₃) ppm; IR: ν 3415 (br), 2926 (w), 2848 (w), 2362 (w), 2337 (w), 1602 (w), 1480 (s), 1418 (m), 1308 (w), 1239 (w),1194 (w), 1149 (w), 1067 (s), 1022 (w), 989 (w), 908 (w), 867 (w), 818 (s), 769 (s), 699 (s), 638 (w) cm^{-1}; MS (EI) *m/z* (%): 230 ([M]$^{+\bullet}$, 100%); HRMS (ES+) found 229.0854 [M+H]$^+$ and 251.0673 [M+Na]$^+$, $C_{14}H_{13}O_3$ requires 229.0859 and $C_{14}H_{12}NaO_3$ requires 251.0679.

PHOTOCHEMICAL REARRANGEMENT

*3-(tert-Butoxy)-4,5-dimethylfuran-2(5H)-one (**29**)*

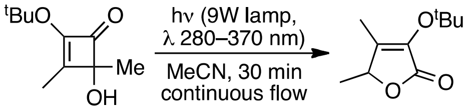

28a **29**, 81%

A solution of cyclobutenone **28a** (17 mg, 0.10 mmol) in degassed acetonitrile (2 mL) was irradiated under continuous flow by a 9 W Philips narrow-spectrum light source (PL-S 9W/01/2P, 310-320 nm) for a residence time of 30 min using the flow-photochemical device detailed in reference 3 and controlled by a Vapourtec R4/R2+ instrument. The resulting solution was concentrated under reduced pressure (under Ar) to give 5*H*-furanone **29** (15 mg, 0.08 mmol, 81%) as a colourless oil.[7] ^{1}H NMR (300 MHz, degassed $CDCl_3$): δ = 4.82 (1H, qq, *J* = 6.7, 1.0 Hz, C**H**), 1.92 (3H, d, *J* = 1.0 Hz, C**H₃**), 1.43 (3H, d, *J* = 6.7 Hz, C**H₃**), 1.38 (9H, s, C(C**H₃**)₃) ppm; ^{13}C NMR (100 MHz, degassed $CDCl_3$): δ = 170.4 (**C**=O), 149.7 (**C**), 138.5 (**C**), 100.0 (**C**H), 82.2 (**C**(CH₃)₃), 28.9 (C(**C**H₃)₃), 18.8 (**C**H₃), 10.8 (**C**H₃) ppm; IR: ν 2978 (w), 2918 (w), 2850 (w), 1763 (s), 1671 (w), 1439 (w), 1391 (w), 1368 (m), 1336 (w), 1217 (w), 1132 (s),1059 (s), 930 (w), 900 (w), 844 (w), 795 (w), 542 (w), 455 (w) cm^{-1}; UV/Vis (acetonitrile): λ_{max} (ε) = 225 (1485); MS (EI) *m/z* (%): 128

([M − $Me_2C{=}CH_2$]$^{+\bullet}$, 26%), 83 (100%); HRMS (EI) found 128.0471 [M – C_4H_8]$^{+\bullet}$, $C_6H_8O_3$ requires 128.0473.

ELIMINATION TO CYCLOBUTENEDIONES

*3,4-Dimethylcyclobut-3-ene-1,2-dione (**30a**)*

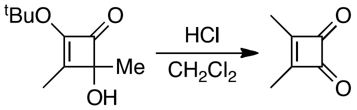

28a **30a**, 98%

To cyclobutenone **28a** (30.5 mg, 0.18 mmol) in dichloromethane (2 mL) was added conc. HCl (10 μL, 0.12 mmol) dropwise over 30 s. After 30 min at RT water (2 mL) was added and the phases separated. The organic phase was dried over $MgSO_4$ and concentrated under reduced pressure to give dione **30a** as a colourless oil (18.0 mg, 0.16 mmol, 98%). ^{1}H NMR (400 MHz, $CDCl_3$): δ = 2.35 (6H, s, 2 x C**H₃**) ppm; ^{13}C NMR (100 MHz, $CDCl_3$): δ = 199.7 (2 x **C**=O), 199.3 (2 x **C**), 10.9 (2 x **C**H_3) ppm; IR: ν 2357 (w), 1765 (s), 1610 (s), 1434 (w), 1381 (w), 1311 (w), 1181 (w), 1013 (w), 682 (w) cm^{-1}; MS (EI) *m/z* (%): 110 ([M]$^{+\bullet}$, 41%), 54 (100%); HRMS (EI$^+$) found 133.0259 [M+Na]$^+$, $C_6H_6NaO_2$ requires 133.0260.

*3-Butyl-4-methylcyclobut-3-ene-1,2-dione (**30b**)*

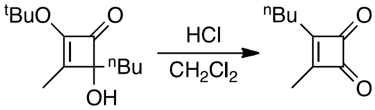

28b **30b**, 92%

Cyclobutenone **28b** (25.7 mg, 0.114 mmol) was dissolved in dichloromethane (2 mL) and conc. HCl (12.2 M in water, 7 μL, 0.09 mmol) was added dropwise. After 30 min water (2 mL) was added and the phases were separated. The organic phase was dried over $MgSO_4$ and concentrated under reduced pressure to a colourless oil (23 mg). Purification by flash column chromatography (5–15% ethyl acetate/petroleum ether) afforded dione **30b** as a colourless oil (16 mg, 0.105 mmol, 92%). ^{1}H NMR (400 MHz, $CDCl_3$): δ = 2.74 (2 H, t, *J* = 7.3 Hz, C**H₂**), 2.35 (3 H, app t, *J* = 0.8 Hz, C**H₃**), 1.72 (1 H, quin, *J* = 7.5 Hz, C**H₂**), 1.41 (2 H, app. sext, *J* = 7.4 Hz, C**H₂**), 0.97 (3 H, t, *J* = 7.4 Hz, C**H₃**) ppm; ^{13}C NMR (100 MHz, $CDCl_3$): δ = 203.5 (**C**=O), 199.6 (**C**=O), 199.3 (**C**), 198.9 (**C**), 27.9 (**C**H_2), 26.1 (**C**H_2), 22.8 (**C**H_2), 13.6 (**C**H_3), 11.2 (**C**H_3) ppm; IR: ν 2958 (w), 2930 (w), 2868 (w), 2362 (w), 2333 (w), 1761 (s), 1602 (s), 1459 (w), 1377 (w), 1333 (w), 1284 (w), 1157 (w), 1034 (w), 969 (w), 744 (w), 687 (w), 605 (w) cm^{-1}; MS (EI) *m/z* (%): 152 ([M]$^{+\bullet}$, 53%), 81 (100%); HRMS (EI) found 152.0835 [M]$^{+\bullet}$, $C_9H_{12}O_2$ requires 152.0837.

*3-Methyl-4-phenylcyclobut-3-ene-1,2-dione (**30c**)*

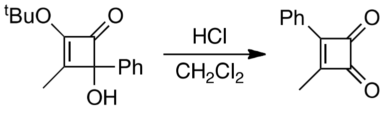

12a **30c**, 99%

Cyclobutenone **12a** (256 mg, 0.106 mmol) was dissolved in 0.08 mmol) was added dropwise. After 30 min, during which time the reaction turned from colourless to yellow, aq. $NaHCO_3$ (2 mL) was added and the phases were separated. The organic phase was dried over $MgSO_4$ and concentrated under reduced pressure to give dione **30c** as a pale yellow crystalline solid (18 mg, 0.105 mmol, 99%). MP 95–97 °C; ^{1}H NMR (400 MHz, $CDCl_3$): δ = 8.07–8.01 (2 H, m, Ar**H**), 7.66–7.54 (3 H, m, Ar**H**), 2.67 (3 H, s, C**H₃**) ppm; ^{13}C NMR (100 MHz, $CDCl_3$): δ = 198.4 (**C**=O), 196.9 (**C**=O), 193.6 (**C**), 191.3 (**C**), 133.5 (**C**H), 129.4 (2 x **C**H), 128.6 (2 x **C**H), 12.5 (**C**H_3) ppm [1 **C** not observed]; IR: ν 2922 (w), 2362 (m), 2333 (w),1761 (s), 1582 (s), 1492 (w), 1447 (m), 1369 (m), 1333 (m), 1177 (w), 1087 (m), 1067 (m), 1010 (w), 773 (s), 728 (w), 687 (s), 605 (w) cm^{-1}; MS (EI) *m/z* (%): 172 ([M]$^{+\bullet}$, 15%), 115 (100%); HRMS (ES+) found 195.0418 [M+Na]$^+$, $C_9H_{12}O_2$ requires 195.0417.

*3-Methyl-4-(2-methoxyphenyl)cyclobut-3-ene-1,2-dione (**30d**)*

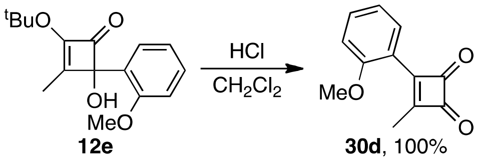

Using the above procedure, cyclobutendione **12e** gave dione **30d** as a pale yellow solid in quantitative yield. MP 105–107 °C; ^{1}H NMR (400 MHz, $CDCl_3$): δ = 8.20 (1 H, dd, J = 7.8, 1.7 Hz, Ar**H**), 7.58 (1 H, ddd, J = 8.6, 7.2, 1.8 Hz, Ar**H**), 7.11 (1 H, td, J = 7.5, 0.9 Hz, Ar**H**), 7.02 (1 H, d, J = 8.5 Hz, Ar**H**), 3.95 (3 H, OC**H**$_3$), 2.58 (3 H, C**H**$_3$) ppm; ^{13}C NMR (100 MHz, $CDCl_3$): δ = 199.4 (**C**=O), 197.3 (**C**=O), 195.5 (**C**), 189.8 (**C**), 157.1 (**C**), 135.2 (**C**H), 130.4 (**C**H), 121.0 (**C**H), 117.9 (**C**), 111.1 (**C**H), 55.3 (O**C**H$_3$), 13.8 (**C**H$_3$)ppm; IR: ν 2926 (br w), 2358 (w), 1770 (s), 1753 (s), 1602 (m), 1574 (m), 1488 (w), 1451 (w), 1365 (m), 1259 (m), 1177 (w), 1132 (m), 1067 (w), 1014 (m), 769 (s), 720 (w), 471 (m) cm^{-1}; MS (EI) m/z (%):202 ([M]$^{+\bullet}$, 32%), 146 (100%); X-Ray (thermal ellipsoids drawn at 35% probability level, selected hydrogens omitted for clarity).

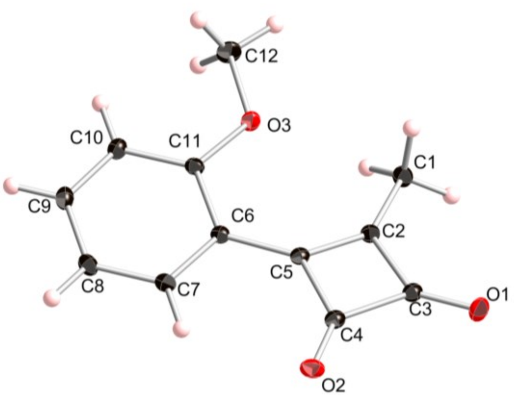

<u>TOTAL SYNTHESIS OF MANSONONE B</u>

*(E)-2,4,6-Triisopropyl-N'-((2S,5R)-2-isopropyl-5-methylcyclohexylidene)benzenesulfonohydra-zone (**40**)*

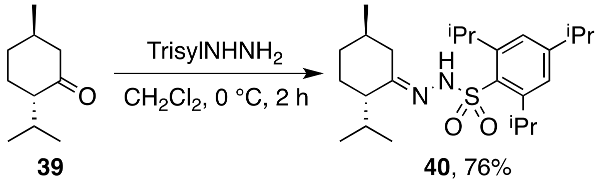

Adapting the method of Garner *et al.*[8] trisylhydrazide (640 mg, 2.1 mmol) was suspended in DCM (3 mL), cooled to 0 °C then (–)-menthone **39** (0.5 mL, 2.9 mmol) was added over 10 sec. After 2 h the reaction mixture was concentrated under reduced pressure at 0 °C to *ca.* half its volume, hexane (2 mL) was added and the mixture concentrated until a white solid formed. The solid was suspended in hexane (5 mL), filtered, washed with further hexane (10 mL) then dried under reduced pressure to give hydrazone **40** (710 mg, 1.6 mmol, 76%) as a white solid that was used without further purification. MP 109–110 °C; ^{1}H NMR (400 MHz, $CDCl_3$): see spectrum ppm; ^{13}C NMR (100 MHz, $CDCl_3$): δ = 153.1 (**C**), 151.04 (2 x **C**), 150.98 (**C**), 123.61 (**C**H), 123.57 (**C**H), 50.2 (**C**H), 50.1 (**C**H), 34.2 (**C**H), 33.2 (**C**H), 32.8 (**C**H), 31.9 (**C**H$_2$), 29.79 (**C**H), 29.76 (**C**H$_3$), 27.5 (**C**H$_2$), 26.9 (**C**H$_2$), 26.8 (**C**H$_3$), 26.1 (**C**H$_3$), 24.8 (**C**H$_3$), 24.75 (**C**H$_3$), 24.71 (**C**H$_3$), 23.6 (**C**H$_3$), 20.3 (**C**H$_3$), 18.5 (**C**H$_3$) ppm; IR: ν 3228 (w), 2946 (m), 2860 (w), 2357 (w), 1630 (w), 1593 (w), 1556 (w), 1454 (w), 1426 (w), 1405 (w), 1381 (w), 1356 (w), 1324 (m), 1254 (w), 1168 (s), 1038 (m), 1017 (m), 931 (m), 911 (m), 886 (w), 845 (w), 743 (w), 662 (s), 559 (s) cm^{-1}; MS (ES+) m/z (%): 435 ([M+H]$^+$, 100%), 891 ([2M+Na]$^+$, 100%); HRMS (ES+) found 457.2859 [M+Na]$^+$ and 891.5806 [2M+Na]$^+$, $C_{25}H_{42}N_2NaO_2S$ requires 457.2859 and $C_{50}H_{84}N_4NaO_4S_2$ requires 891.5826.

*2-(tert-Butoxy)-4-hydroxy-4-((3R,6S)-6-isopropyl-3-methylcyclohex-1-en-1-yl)-3-methylcyclobut-2-enone (**42**)*

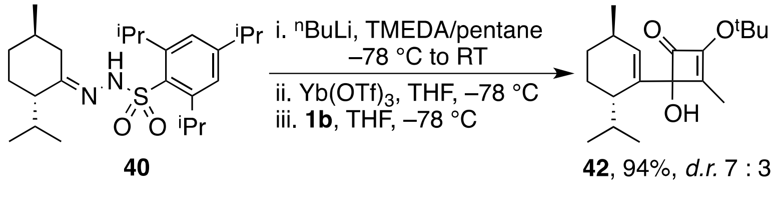

Adapting the method of Garner *et al.*[8] to a suspension of trisyl hydrazone **40** (216 mg, 0.50 mmol) in pentane (5 mL) and TMEDA (300 μL, 2.0 mmol) at –78 °C, was added ^{n}BuLi (2.38 M, 630 μL, 1.50 mmol) dropwise over 30 sec, during which time the reaction mixture turned milky orange. On warming to RT over 1 h, the solution became clear as N_2 evolution was observed. After 1 h at RT the resulting solution was transferred via cannula over 5 min to a solution of Yb(OTf)$_3$ (325 mg, 0.52 mmol) in THF (5 mL) at –78 °C. During the course of addition the colourless solution turned from navy blue to purple to dark

purple/brown. After 30 min a solution of cyclobutenedione **1b** (56 mg, 0.33 mmol) in THF (2 mL) was added dropwise over 2 min and the solution faded to pale violet. After a further 30 min sat. $NaHCO_3$ (5 mL) was added. The resulting biphasic mixture was warmed to RT and ether (10 mL) was added. The aqueous phase was separated and extracted with ether (10 mL) then the organic phases were combined, washed with brine (15 mL), dried over $MgSO_4$ and concentrated under reduced pressure to give adduct **42** (96 mg, 0.31 mmol, 94%) as a yellow oil composed of a 7 : 3 mixture of diastereoisomers. The adduct was used without further purification. ^{1}H NMR (400 MHz, $CDCl_3$): δ = *major diastereoisomer* 6.94 (1 H, br s, O**H**) 5.61 (1 H, d with fine splitting, *J* = 1.5 Hz, C=C**H**) 2.66−2.57 (1 H, m, C**H**) 2.48−2.31 (3 H, m, C**H₃**) 2.20−2.04 (1 H, m, C**H**) 2.03−2.00 (1 H, m, C**H**) 1.95−1.85 (1 H, m, C**H₂**) 1.84−1.66 (2 H, m, C**H₂**) 1.61−1.40 (9 H, m, C(C**H₃**)₃) 1.27−1.11 (1 H, m, C**H₂**) 1.08 (3 H, d, *J* = 7.2 Hz, C**H₃**) 1.00−0.94 (3 H, m, C**H₃**) 0.72 (3 H, d, *J* = 6.8 Hz, C**H₃**) ppm with additional signals attributed to the minor diastereoisomer; ^{13}C NMR (100 MHz, $CDCl_3$): δ = 198.6 (**C**), 197.7 (**C**), 194.5 (**C**), 191.5 (**C**), 186.2 (**C**), 156.8 (**C**), 154.3 (**C**), 148.2 (**C**H), 137.9 (**C**), 134.3 (**C**H), 133.3 (**C**), 90.3 (**C**), 80.1 (**C**), 40.1 (**C**H), 39.8 (**C**H), 31.6 (**C**H), 30.31 (**C**H), 30.25 (**C**H_2), 30.19 (**C**H), 28.9 (**C**H_2), 28.6 (C(**C**H_3)₃), 21.9 (**C**H_3), 21.1 (**C**H_3), 21.0 (**C**H_3), 20.8 (**C**H_2), 20.52 (**C**H_2), 20.45 (**C**H_3), 16.8 (**C**H_3), 16.3 (**C**H_3), 12.3 (**C**H_3), 8.8 (**C**H_3) ppm; IR: ν 3482 (br w), 2957 (m), 2931 (m), 2869 (w), 2361 (w), 1757 (s), 1635 (w), 1605 (m), 1569 (m), 1458 (w), 1370 (m), 1312 (m), 1168 (m), 1049 (m), 927 (m), 876 (w) cm^{-1}; MS (EI) *m/z* (%): 306 ([M]$^{+\bullet}$, 2%), 207 (100%).

*(5S,8R)-2-(tert-Butoxy)-5-isopropyl-3,8-dimethyl-5,6,7,8-tetrahydronaphthal-ene-1,4-dione (**44**)*

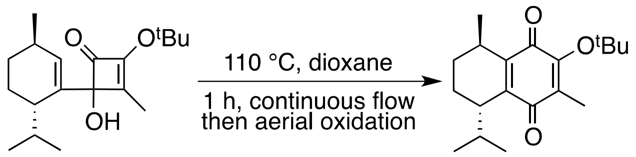

42, *d.r.* 7 : 3 **44**, 84%

A solution of alcohol **42** (28 mg, 0.09 mmol) in dioxane (2 mL) was heated at 110 °C in stainless steel tubing under continuous flow for a residence time of 1 h. The resulting solution was concentrated under reduced pressure to a yellow oil (27 mg) which was dissolved in $CHCl_3$ (10 mL) and stirred at RT under air for 5 h. The solution was concentrated under reduced pressure to a bright yellow oil (25 mg) then purified by flash column chromatography (0.1–1% ethyl acetate/dichloromethane) to afforded quinone **44** (23 mg, 0.08 mmol, 84%) as a bright yellow oil. ^{1}H NMR (300 MHz, $CDCl_3$): δ = 2.96 (1H, app quin with fine splitting, *J* = 7.0 Hz, C**H**), 2.70 (1H, td, *J* = 5.8, 1.6 Hz, C**H** or C**H**H), 1.96 (3H, s, C**H₃**), 1.94−1.73 (3H, m, C**H** + 2 x C**H**H), 1.65−1.40 (2H, m, C**H** or CH**H** + CH**H**), 1.38 (9H, s, C(C**H₃**)₃), 1.06 (3H, d, *J* = 7.0 Hz, C**H₃**), 0.87 (3H, d, *J* = 6.9 Hz, C**H₃**), 0.86 (3H, d, *J* = 7.0 Hz, C**H₃**) ppm; ^{13}C NMR (100 MHz, $CDCl_3$): δ = 189.2 (**C**=O), 185.1 (**C**=O), 154.1 (**C**), 145.9 (**C**), 145.1 (**C**), 134.1 (**C**), 83.8 (**C**(CH_3)₃), 36.6 (**C**H), 31.8 (**C**H), 29.5 (C(**C**H₃)₃), 26.3 (**C**H), 25.6 (**C**H_2), 21.8 (**C**H_3), 20.9 (**C**H_3), 20.5 (**C**H_3), 18.7 (**C**H_2), 10.7 (**C**H_3) ppm; MS (EI) *m/z* (%): 248 ([M − $Me_2C=CH_2$]$^{+\bullet}$, 9%), 41 (100%); HRMS (ES+) found 327.1936 [M+Na]$^+$, $C_{19}H_{28}NaO_3$ requires 327.1931.

*(5S,8R)-2-Hydroxy-5-isopropyl-3,8-dimethyl-5,6,7,8-tetrahydronaphthalene-1,4-dione, (–)-Mansonone B (**43**)*

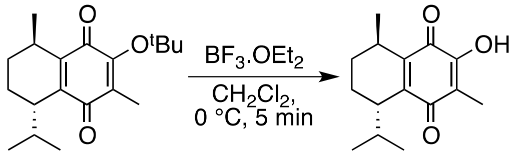

44 **43**, 82%

To a solution of quinone **44** (60 mg, 0.197 mmol) in DCM (20 mL) at 0 °C was added $BF_3.OEt_2$ (50 µL, 0.394 mmol) over 20 s. After 5 min the resulting deep blue solution was concentrated under reduced pressure and the residual dark yellow oil was purified by flash column chromatography (5% diethyl ether/petroleum ether) to give (–)-mansonone B **43** (40 mg, 0.161 mmol, 82%) as a yellow crystalline solid. MP 67–68 °C (hexane), Lit. 68–69 °C;[9] $[\alpha]_D^{24}$ −456.5 (*c* = 0.001 in chloroform) (units for [*α*] = degcm^{3}g^{-1}dm^{-1} and *c* = gcm^{-3}); ^{1}H NMR (400 MHz, $CDCl_3$): δ = 6.98 (1H, br s, O**H**), 2.94 (1H, app quin with fine splitting, *J* = 7.1 Hz, C**H**), 2.77 (1H, td, *J* = 5.7, 1.6 Hz, C**H** or C**H**H), 1.93 (3H, s, C**H₃**), 1.92−1.84 (2H, m, C**H** or C**H**H + C**H**H), 1.79 (1H, dtd, *J* = 14.0, 3.7, 1.8 Hz, CH**H**), 1.65−1.59 (1H, ddd, *J* = 14.1, 5.7, 3.4 Hz, C**H** or CH**H**), 1.47 (1H, dtdd, *J* = 13.5, 3.5, 1.0, 0.6 Hz, CH**H**), 1.10 (3H, d, *J* = 7.1 Hz, C**H₃**), 0.89 (3H, d, *J* = 6.9 Hz, C**H₃**), 0.87 (3H, d, *J* = 6.8 Hz, C**H₃**) ppm; ^{13}C NMR (100 MHz, $CDCl_3$): δ = 188.1 (**C**=O), 183.0 (**C**=O), 150.6 (2 x **C**), 148.6 (**C**), 142.5 (**C**), 36.9 (**C**H), 32.3 (**C**H), 26.2 (**C**H), 25.5 (**C**H_2), 22.0 (**C**H_3), 20.9 (**C**H_3), 20.6 (**C**H_3), 18.7 (**C**H_2), 8.2 (**C**H_3) ppm; UV/Vis (ethanol): λ_{max} (ε) = 405

(629), 332 (406); UV/Vis (ethanol + NaOH): λ_{max} (ε) = 530 (2771), λ_2 (ε) = 327 (930); IR: ν 3389 br, 2959 m, 2872 w, 2360 s, 2340 s, 2020 w, 1988 w, 1641 s, 1463 w, 1396 w, 1336 m, 1305 w, 1235 w, 1155 w, 1139 w, 1042 w, 961 w cm^{-1}; MS (EI) *m/z* (%): 248 ($[M]^{+\bullet}$, 8%), 205 ($[M - C_3H_7]^{+\bullet}$,100%); HRMS (EI) found 248.1405 $[M]^{+\bullet}$, $C_{15}H_{20}O_3$ requires 248.1412; X-Ray displayed four *crystallographically independent molecules* in the asymmetric unit cell, thermal ellipsoids are drawn at the 35% probability level.

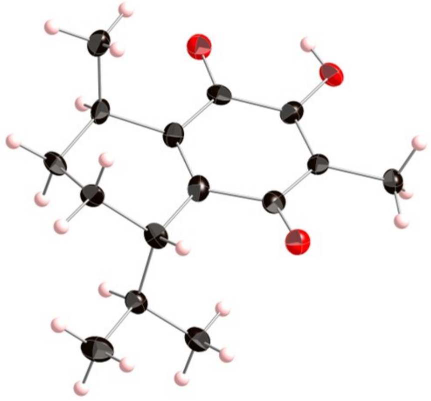

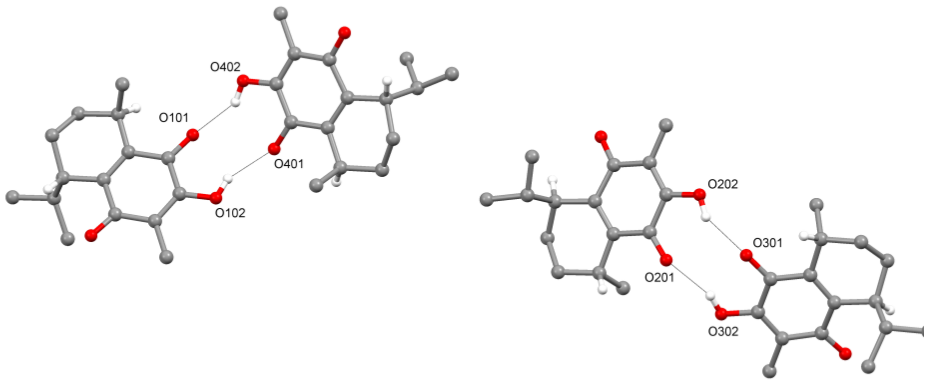

^{1}H AND ^{13}C NMR SPECTRA

*4-Hydroxy-2-methoxy-3-methyl-4-phenylcyclobut-2-enone (**11a**)*

^{1}H NMR

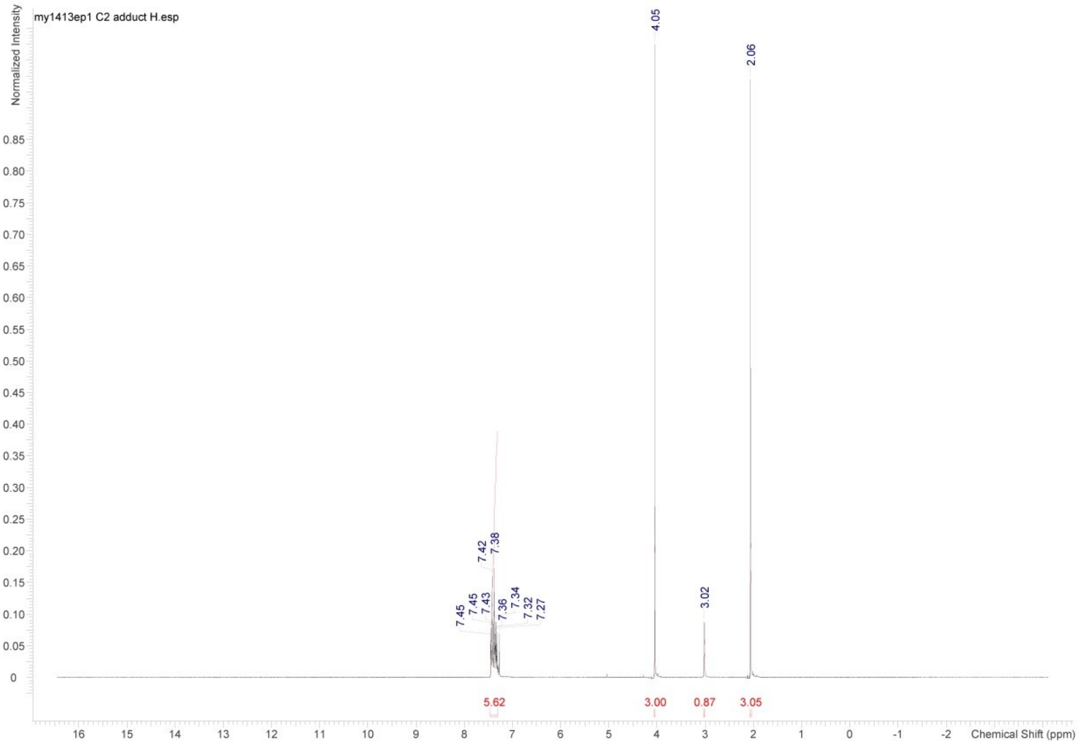

^{13}C NMR

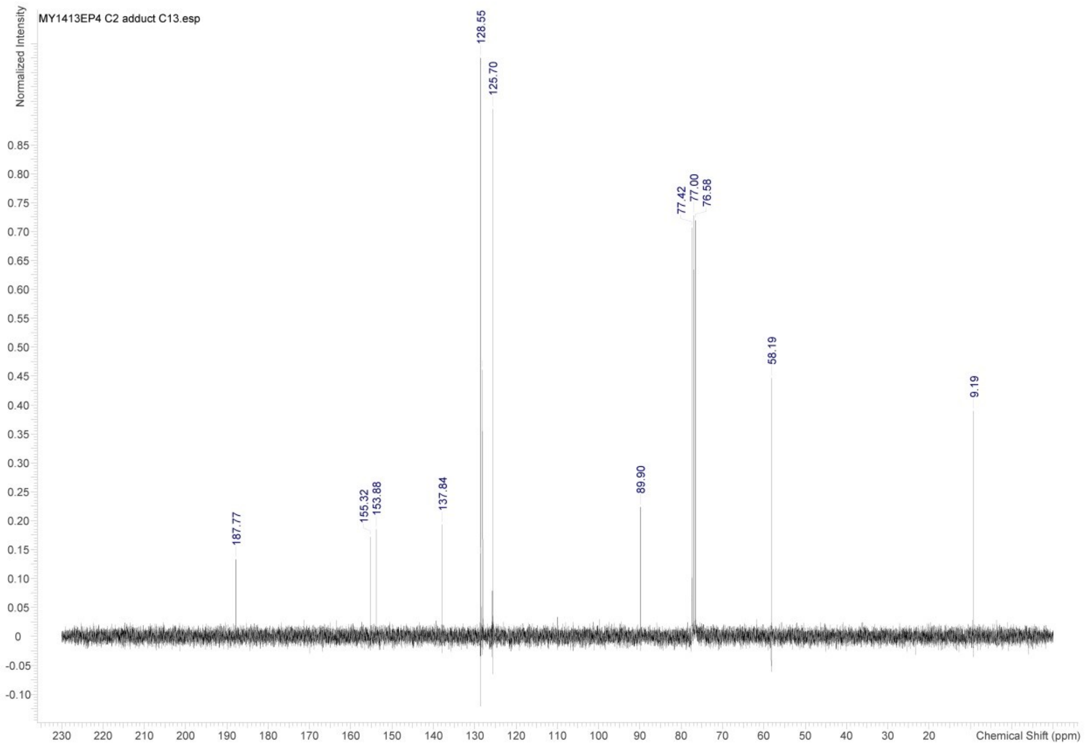

*4-Hydroxy-3-methoxy-2-methyl-4-phenylcyclobut-2-enone (**2a**)*

^{1}H NMR

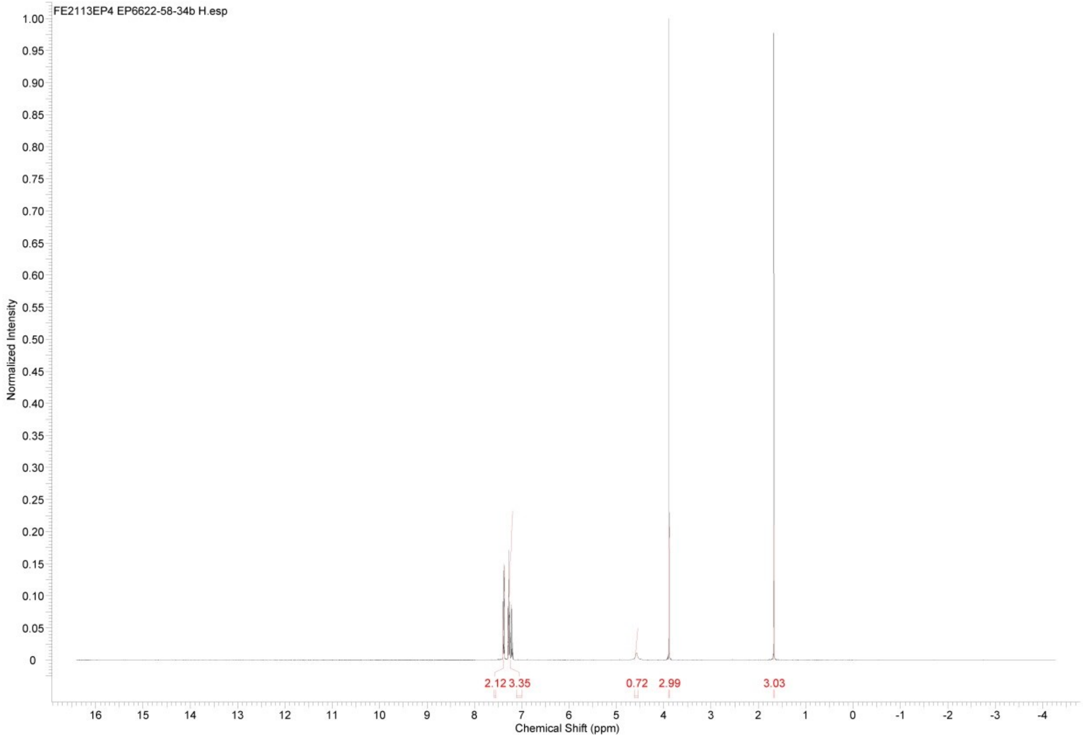

^{13}C NMR

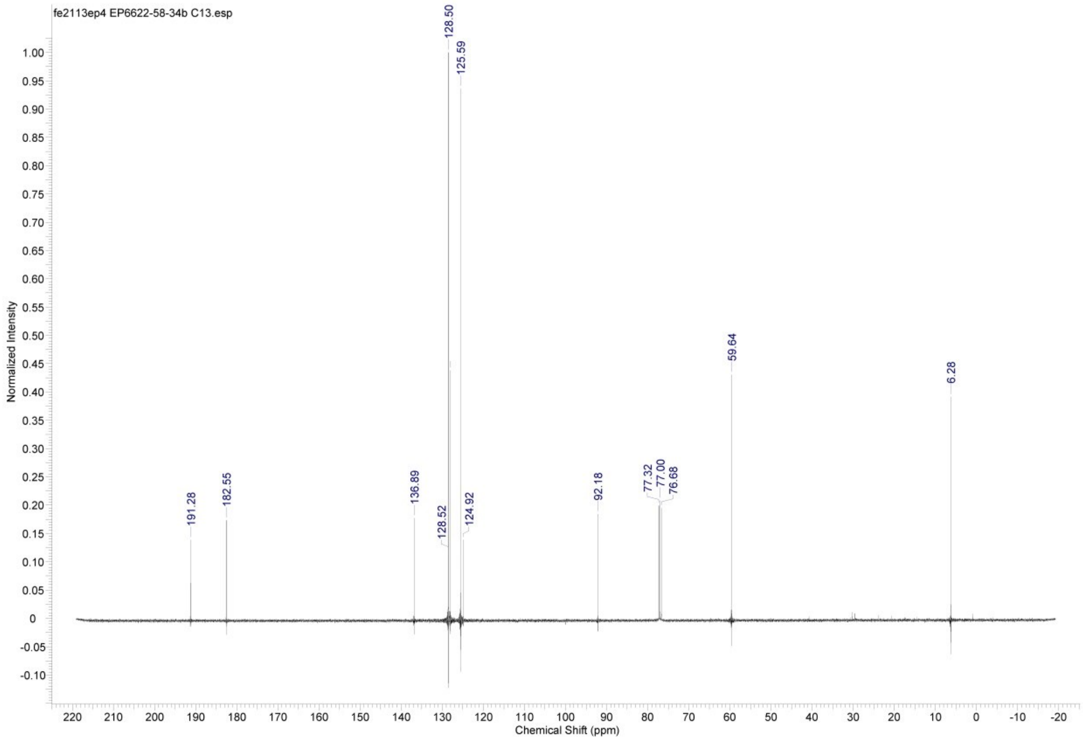

*2-(tert-Butoxy)-4-hydroxy-3-methyl-4-phenylcyclobut-2-enone (**12a**)*

^{1}H NMR

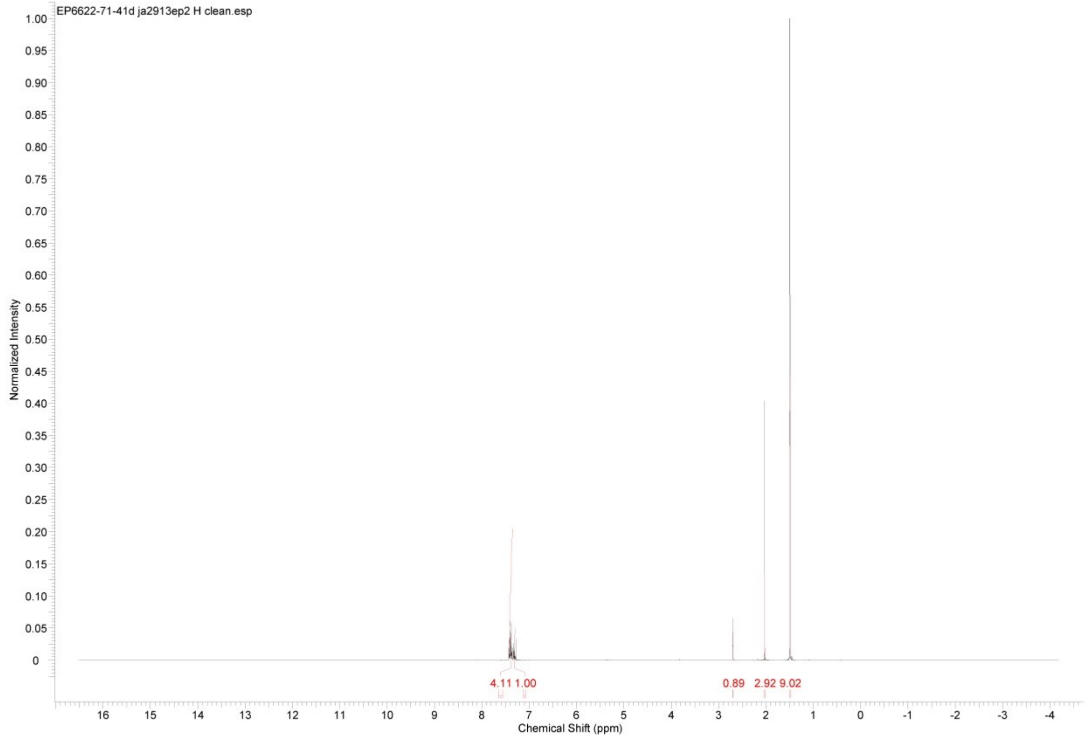

^{13}C NMR

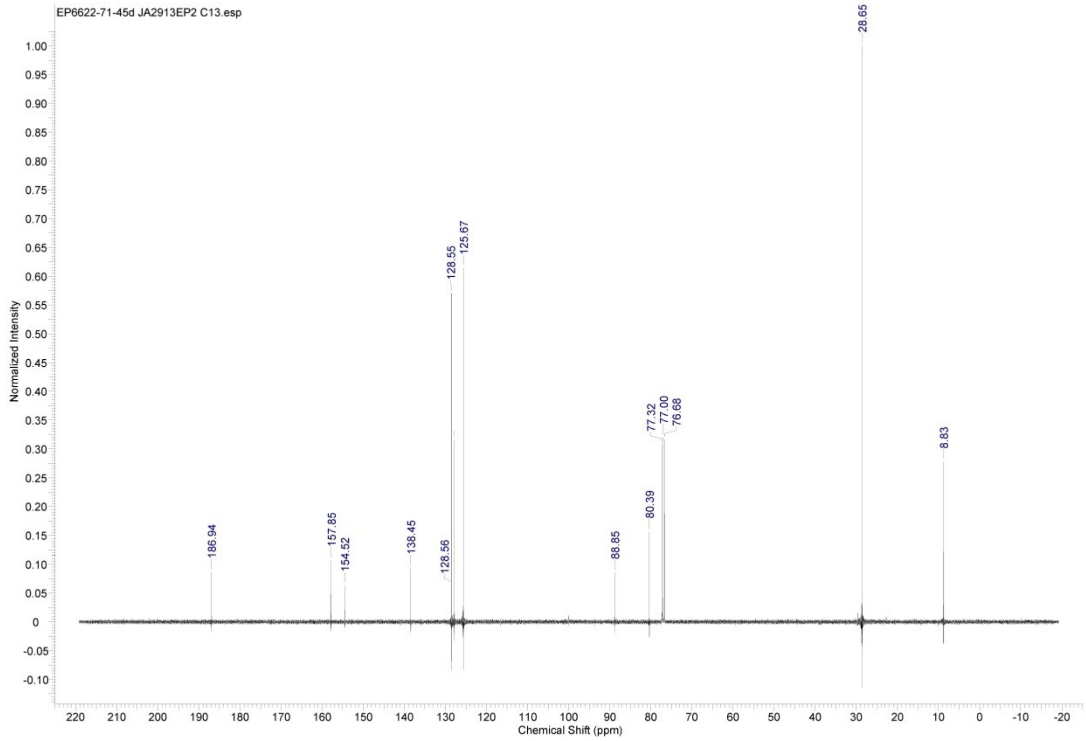

*2-(tert-Butoxy)-4-hydroxy-3-methyl-4-(p-tolyl)cyclobut-2-enone (**12b**)*

^{1}H NMR

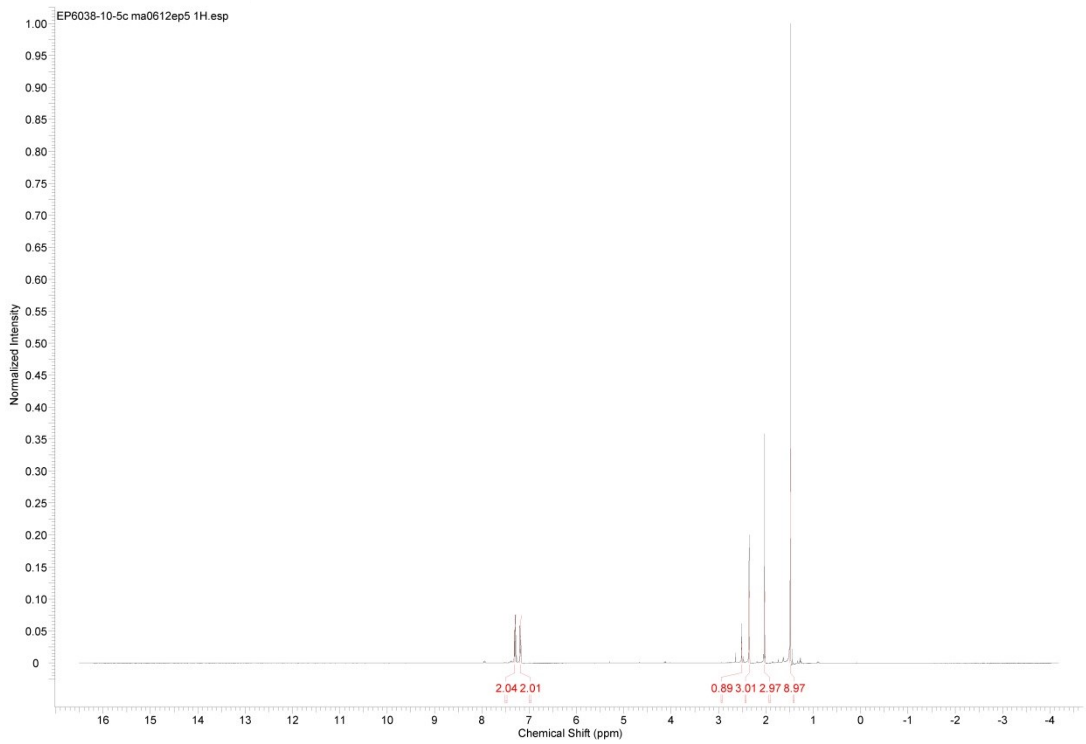

^{13}C NMR

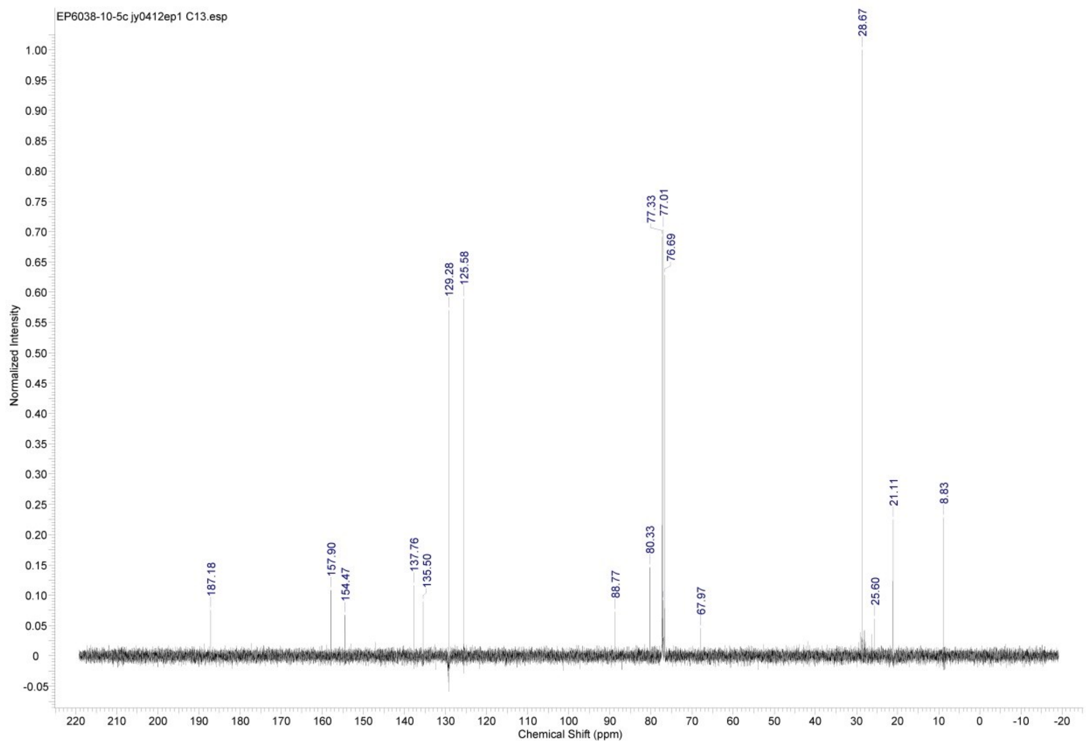

2-(tert-Butoxy)-4-hydroxy-4-(4-methoxyphenyl)-3-methylcyclobut-2-enone (*12c*)

^{1}H NMR

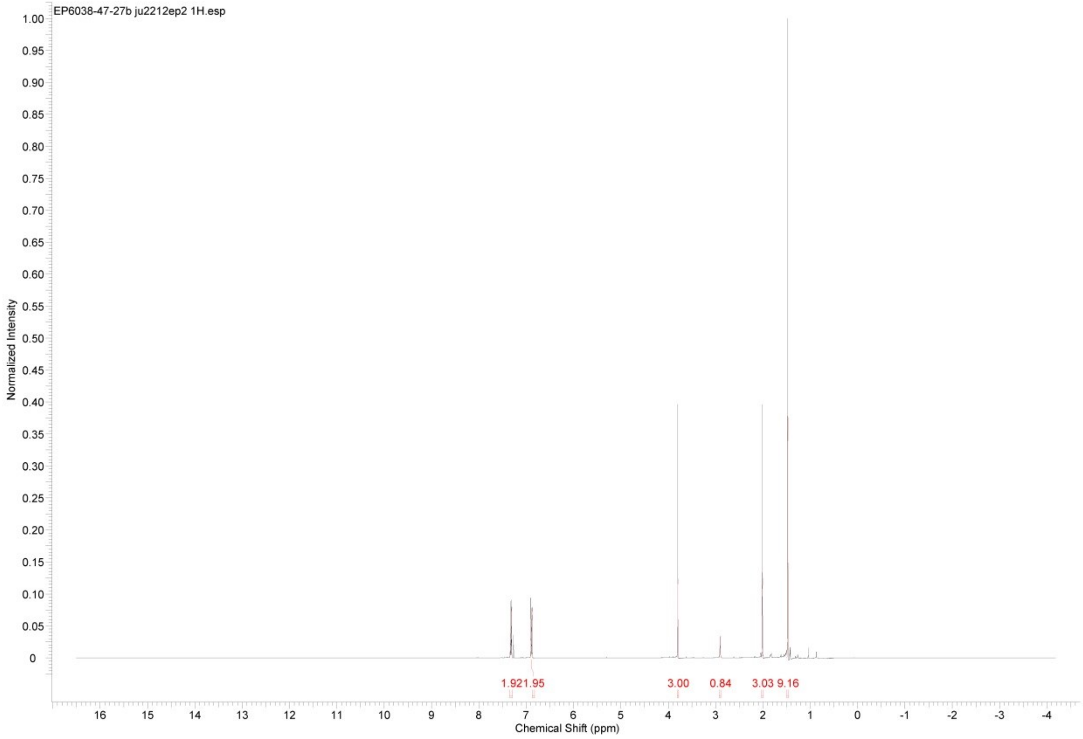

^{13}C NMR

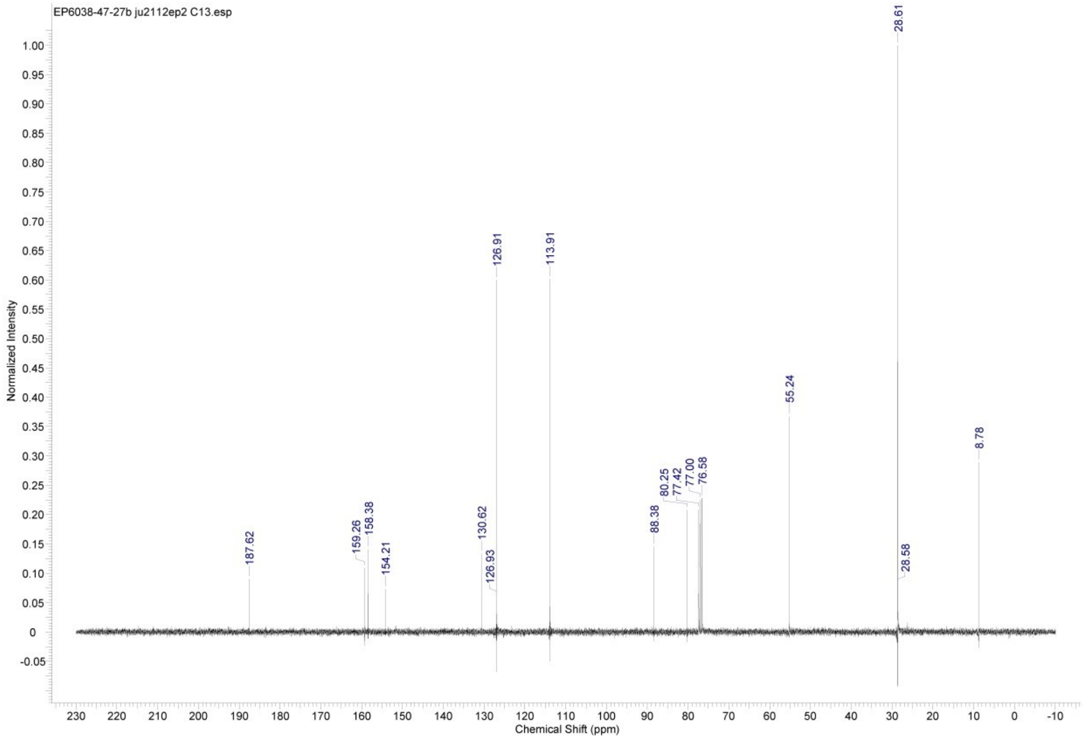

*2-(tert-Butoxy)-4-hydroxy-3-methyl-4-(4-(trifluoromethyl)phenyl)cyclobut-2-enone (**12d**)*

^{1}H NMR

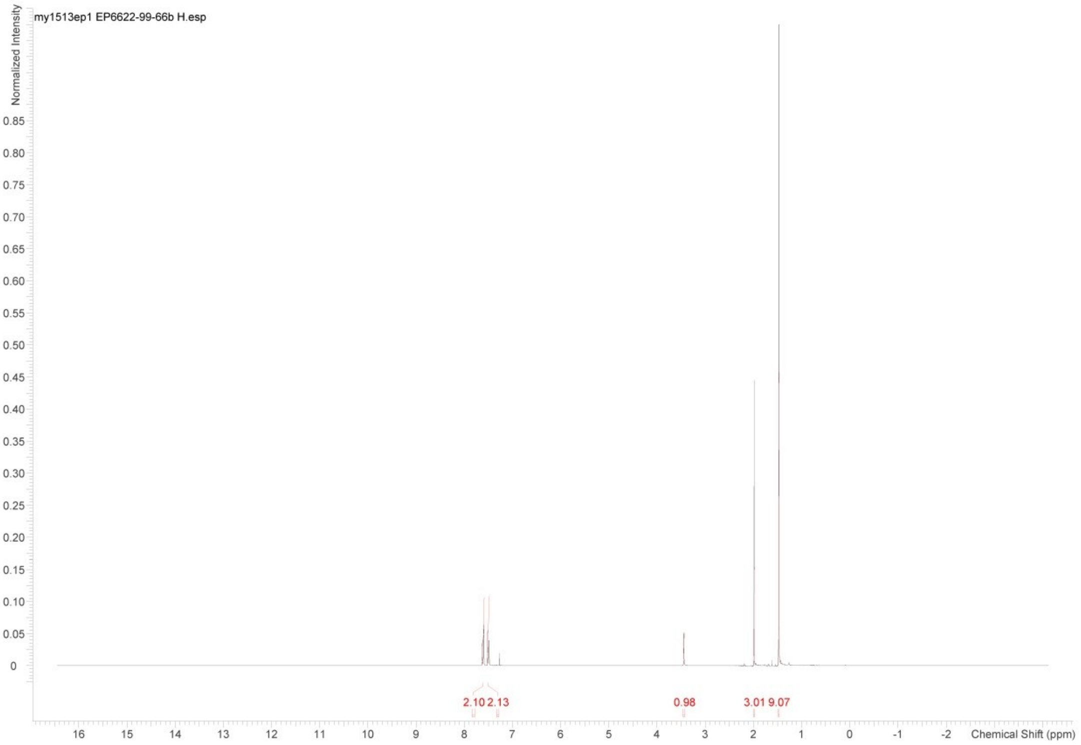

^{13}C NMR

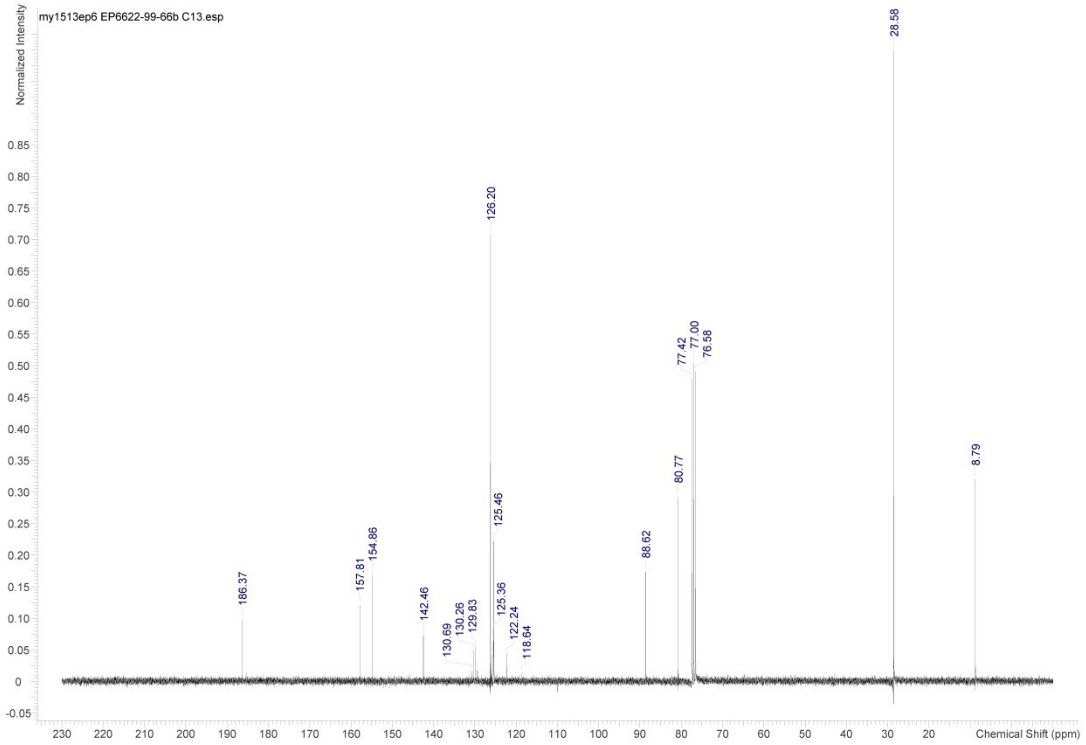

*2-(tert-Butoxy)-4-hydroxy-3-methyl-4-(2-methoxyphenyl)cyclobut-2-enone (**12e**)*

^{1}H NMR

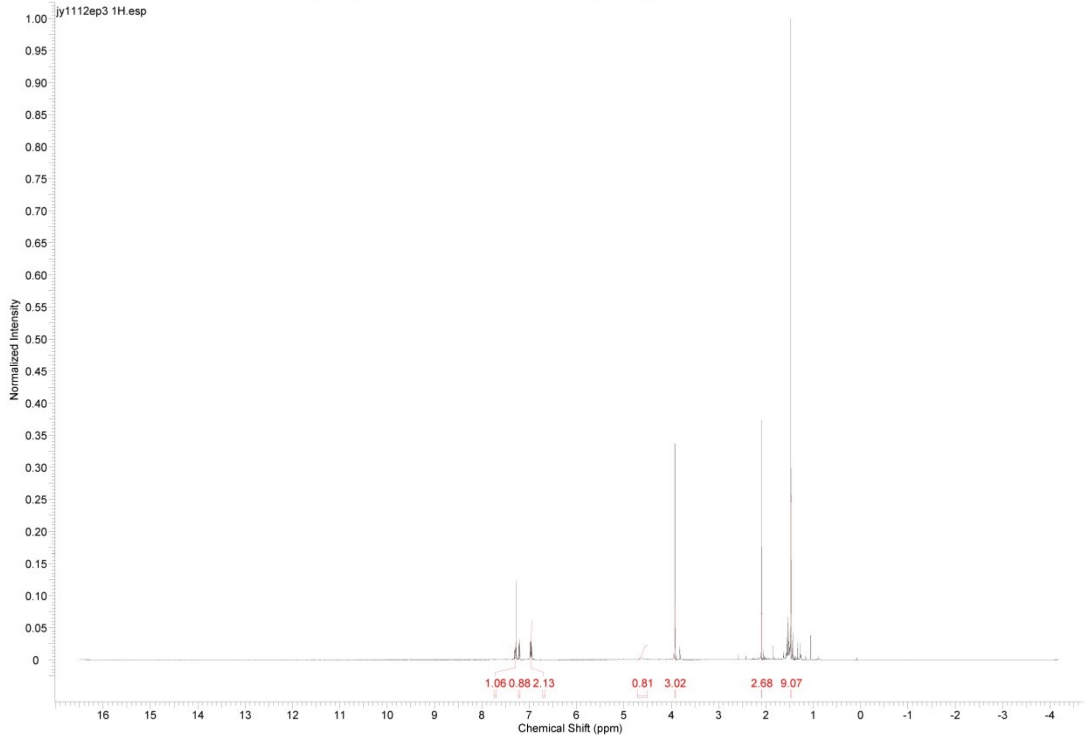

^{13}C NMR

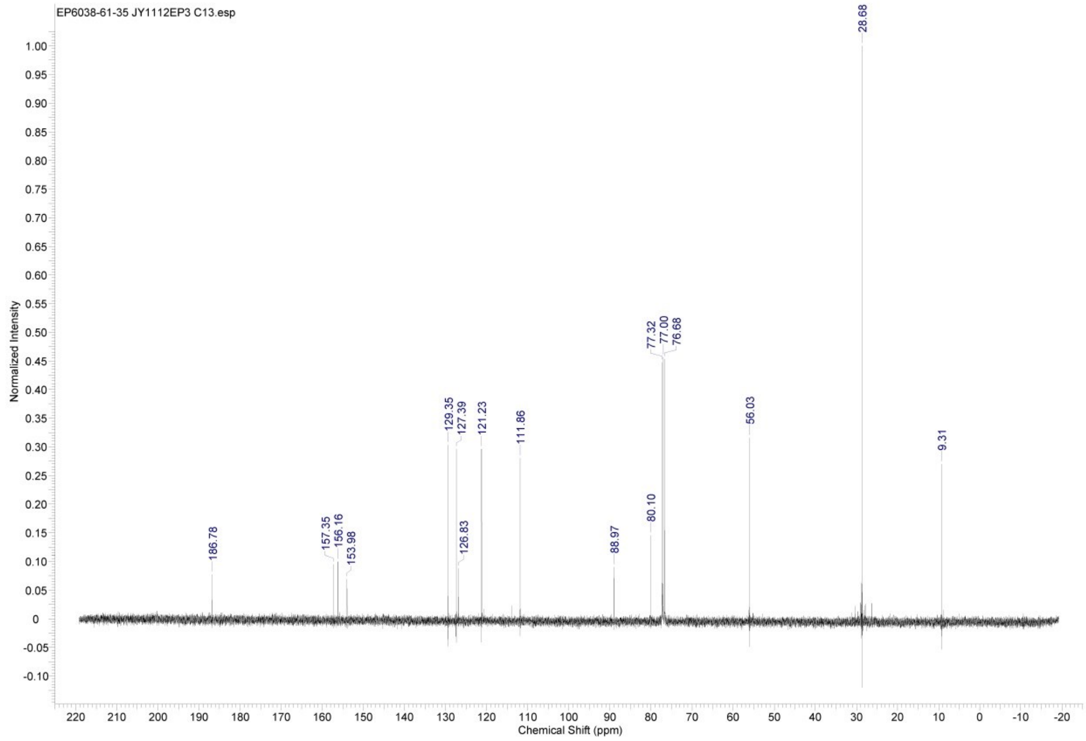

*2-(tert-Butoxy)-4-hydroxy-3-methyl-4-(pyridin-2-yl)cyclobut-2-enone (**12f**)*

^{1}H NMR

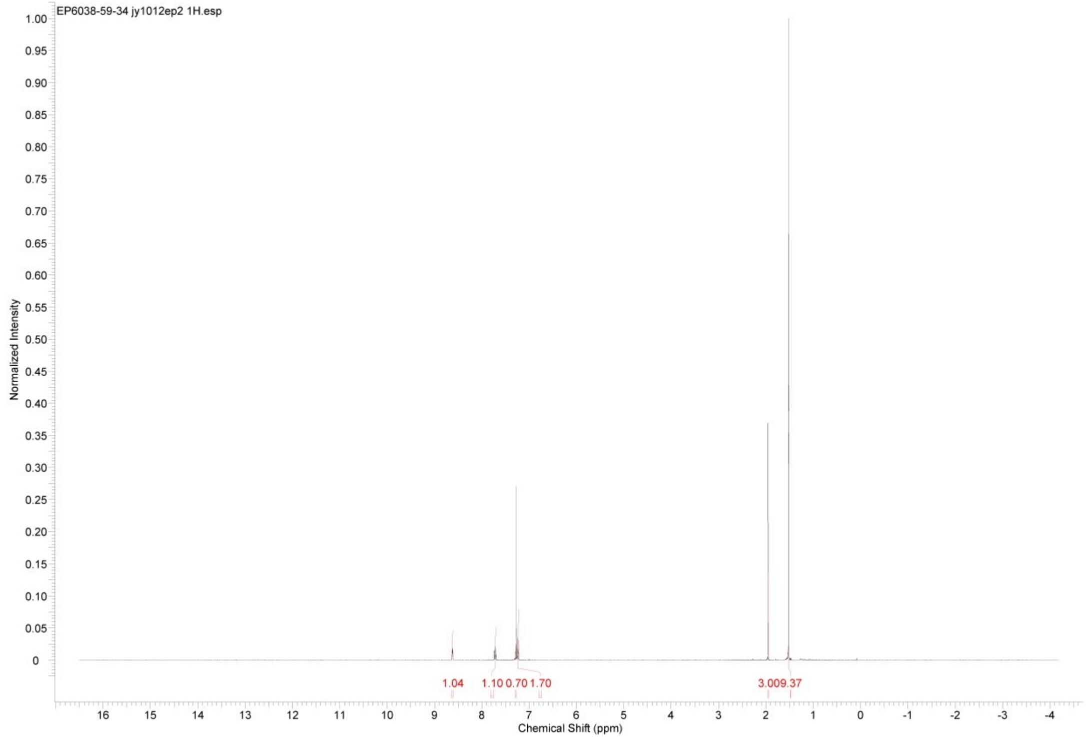

^{13}C NMR

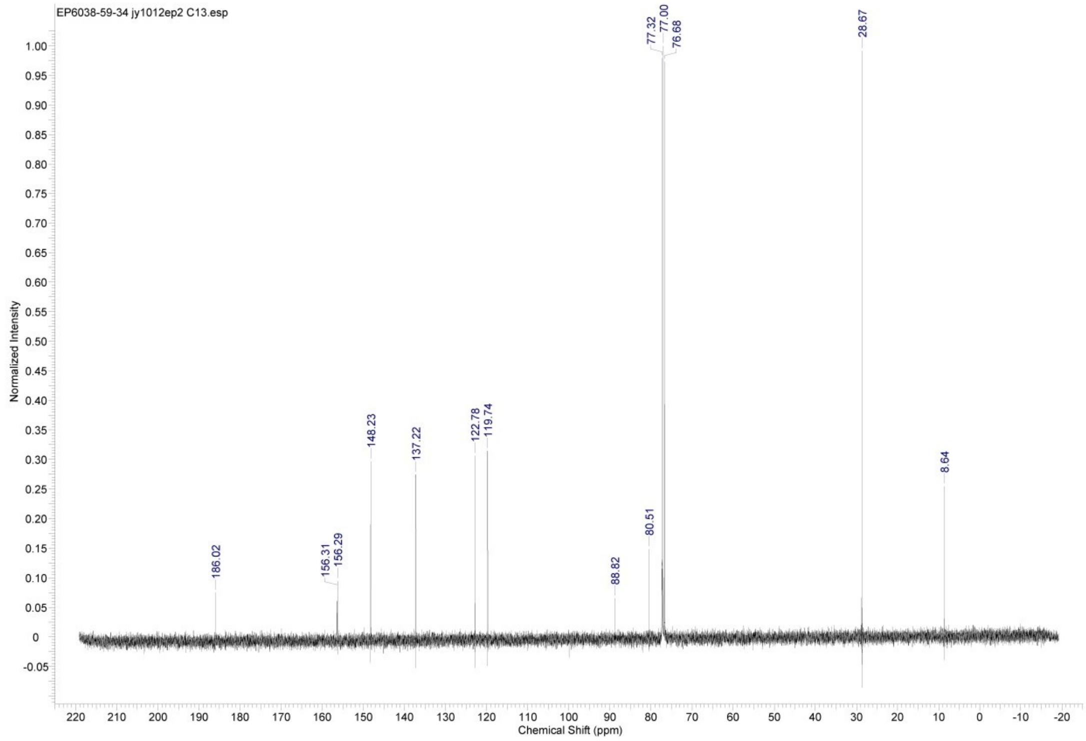

*2-(tert-Butoxy)-4-hydroxy-3-methyl-4-(m-tolyl)cyclobut-2-enone (**12g**)*

^{1}H NMR

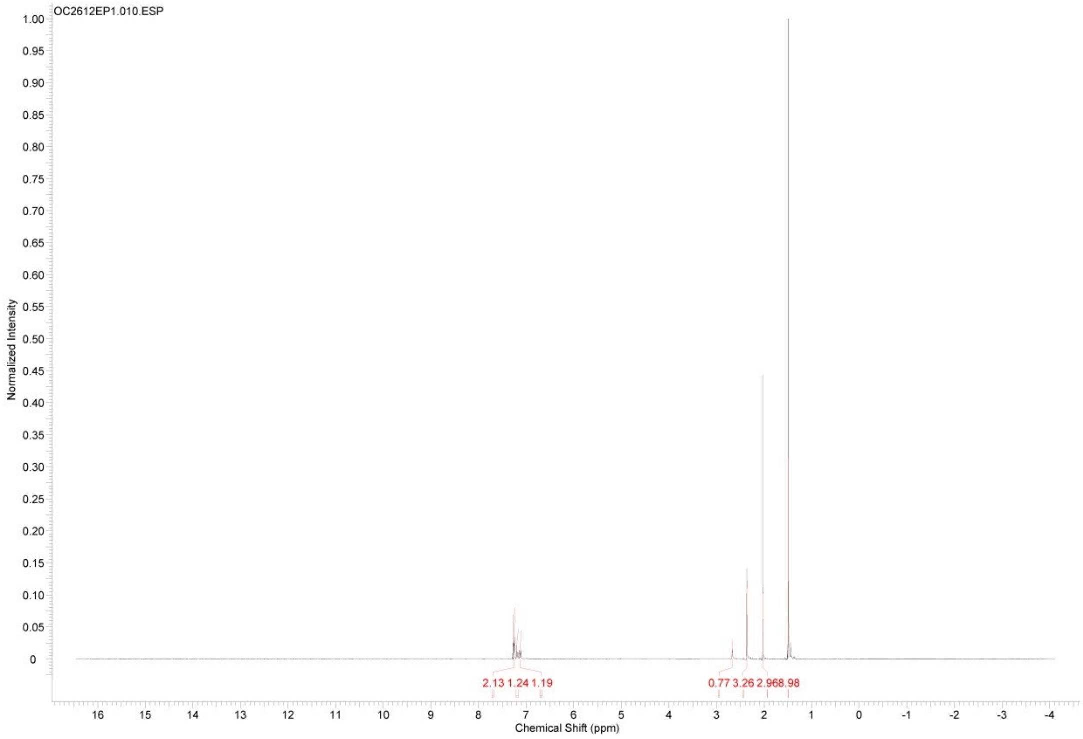

^{13}C NMR

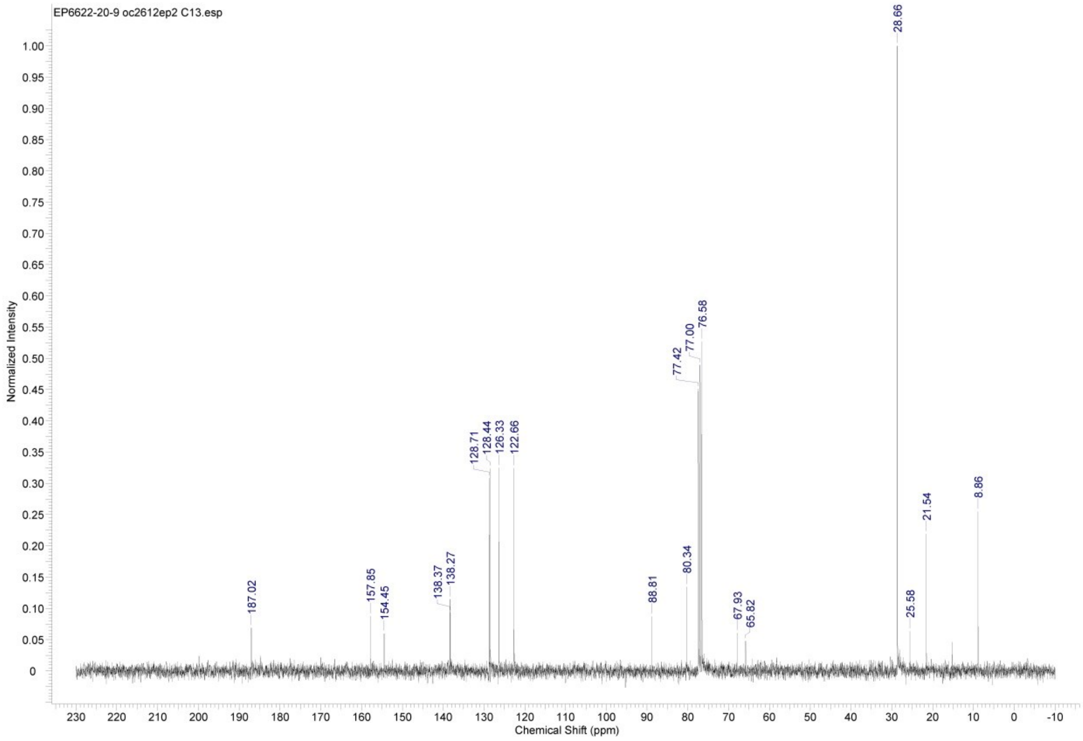

2-(tert-Butoxy)-4-hydroxy-3-methyl-4-(3-(trifluoromethyl)phenyl)cyclobut-2-enone (**12h**)

^{1}H NMR

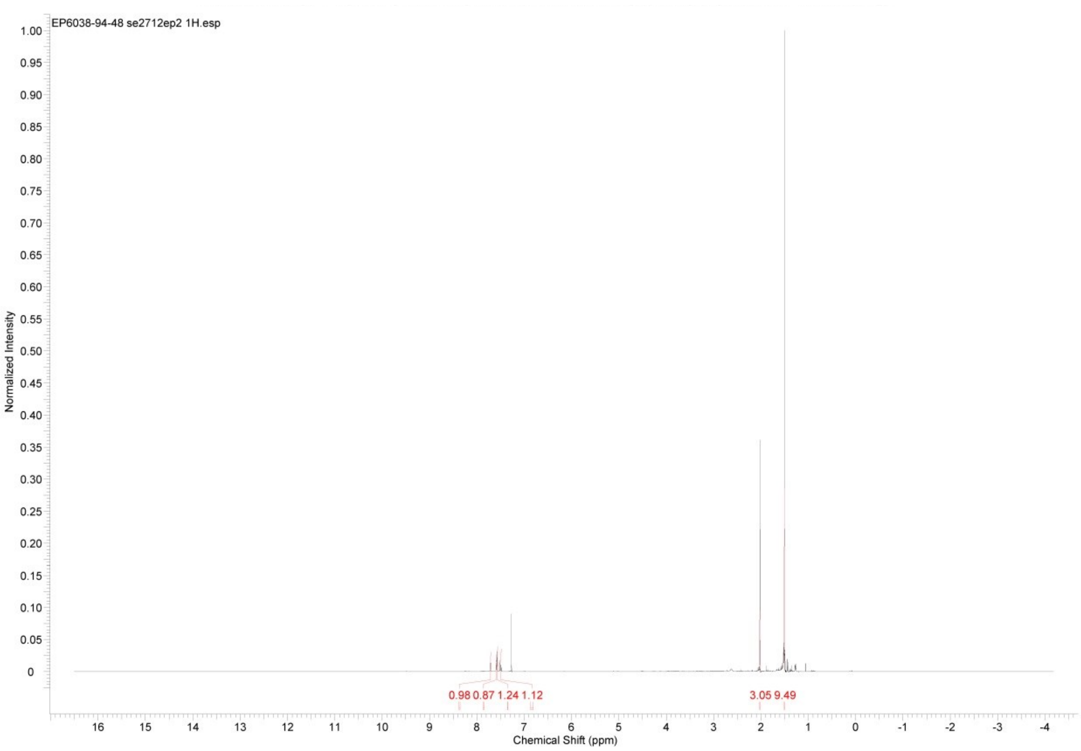

^{13}C NMR

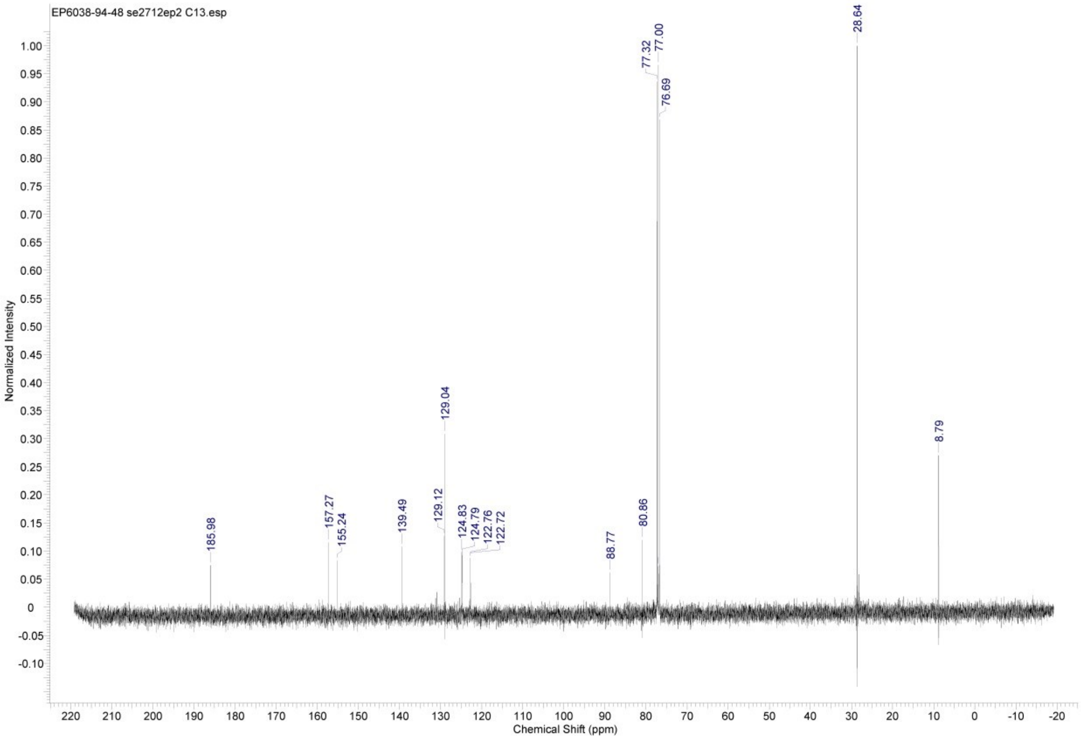

2-(tert-Butoxy)-4-hydroxy-3-methyl-4-(propen-2-yl)cyclobut-2-enone (**20**)

^{1}H NMR

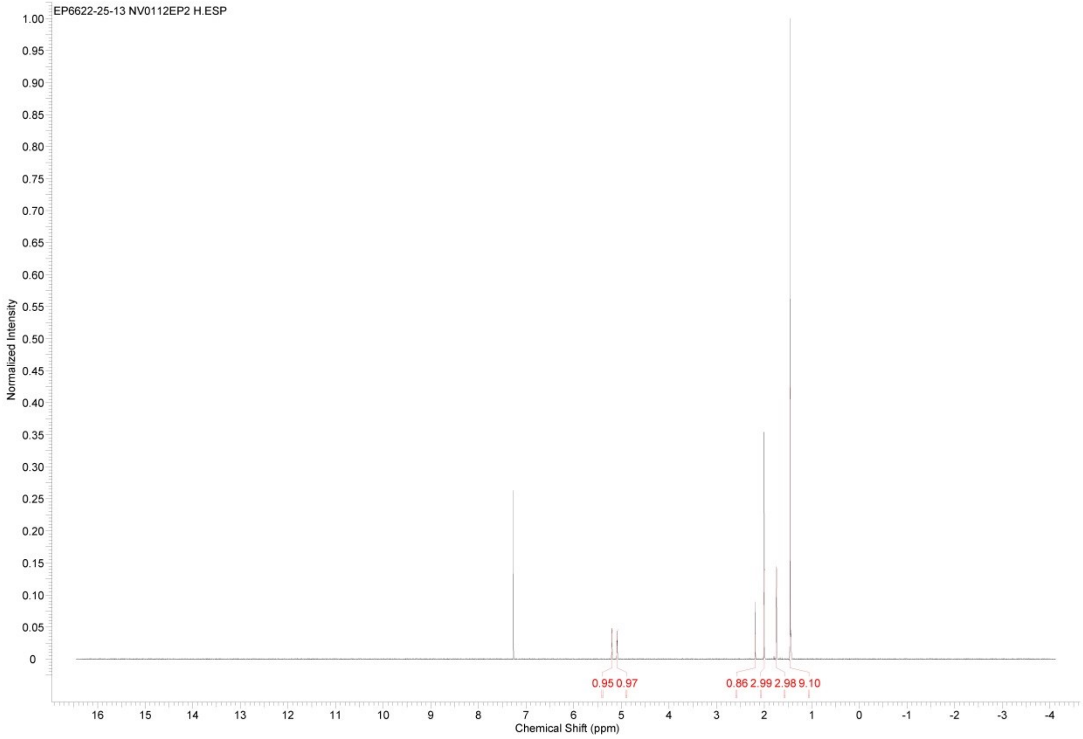

^{13}C NMR

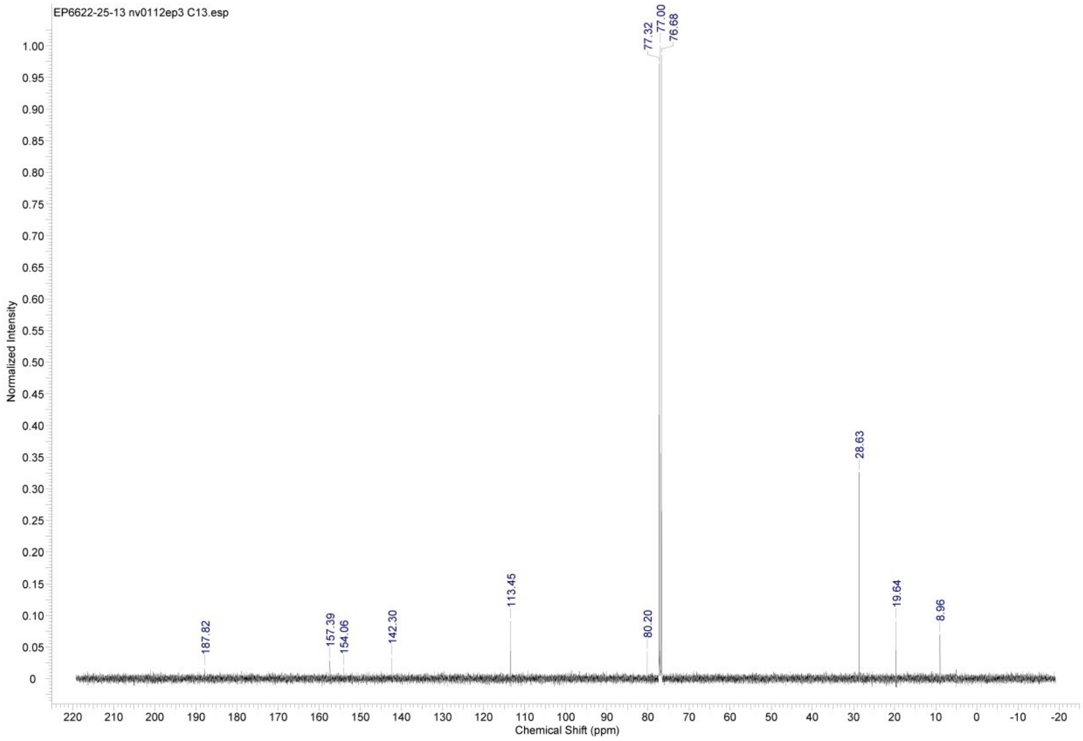

*2-(tert-Butoxy)-4-hydroxy-3-methyl-4-(1-phenylvinyl)cyclobut-2-enone (**21a**)*

^{1}H NMR

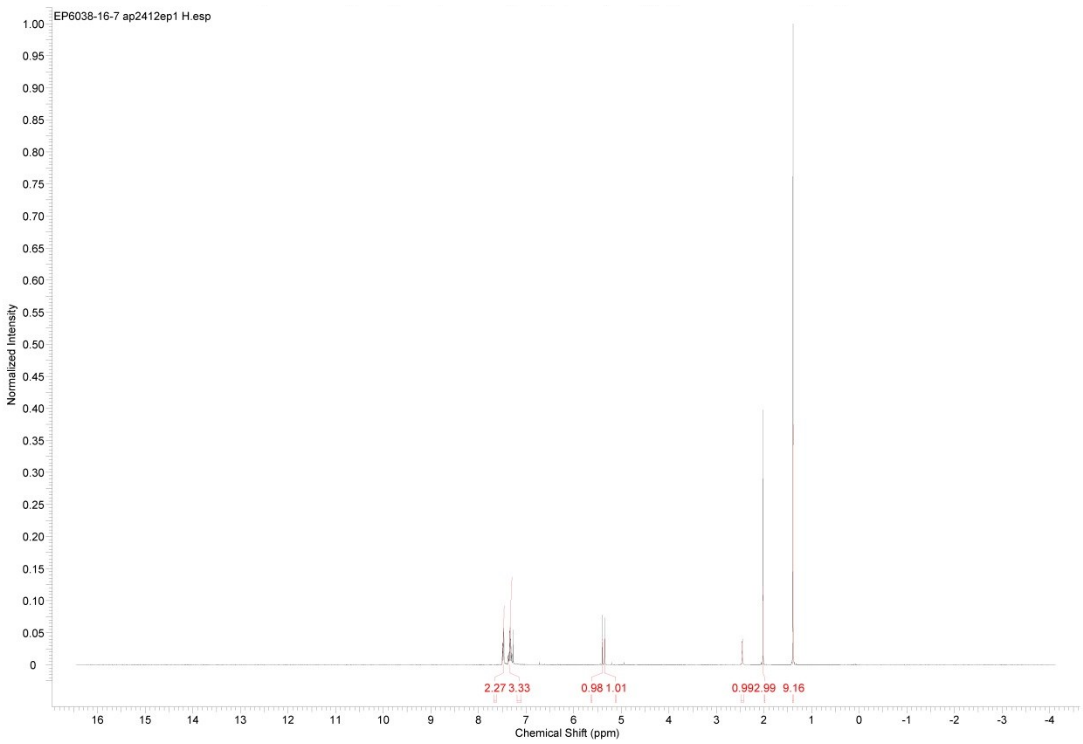

^{13}C NMR

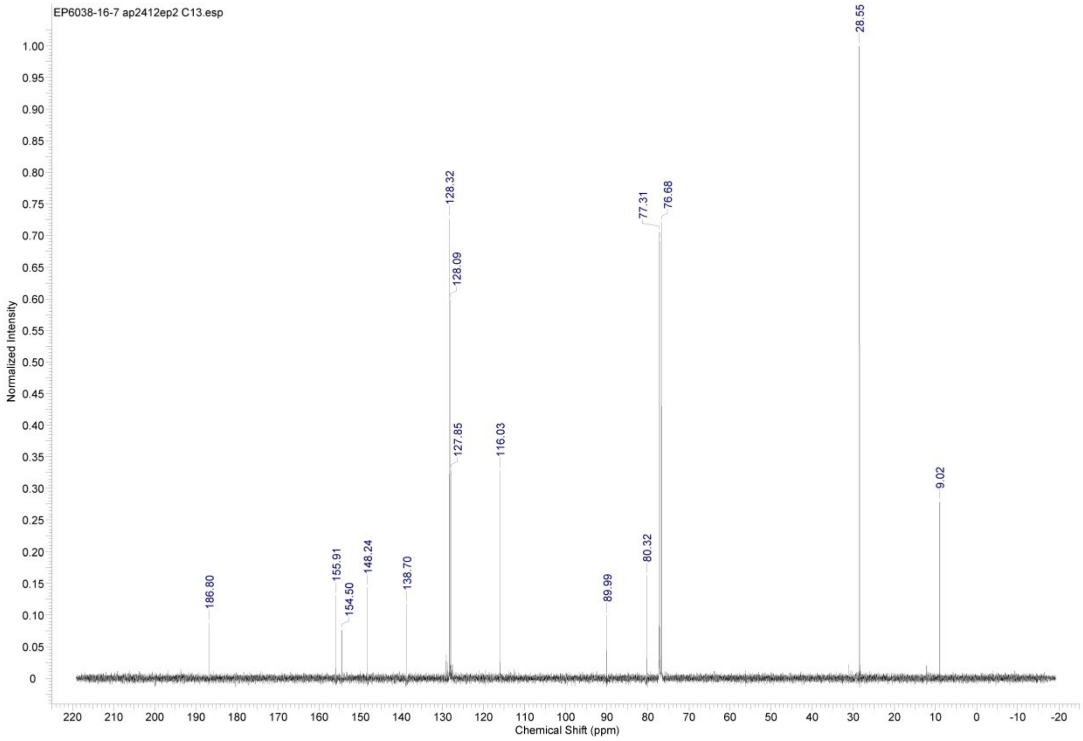

2-(tert-Butoxy)-4-hydroxy-3-methyl-4-vinylcyclobut-2-enone (**22**)

^{1}H NMR

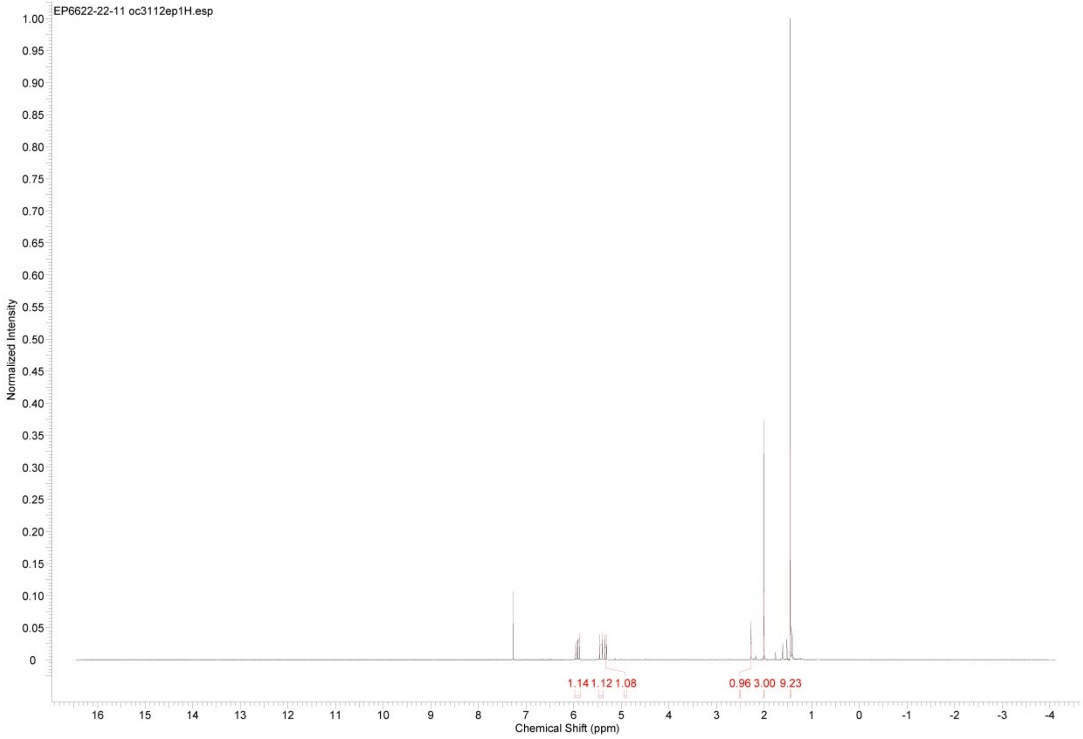

^{13}C NMR

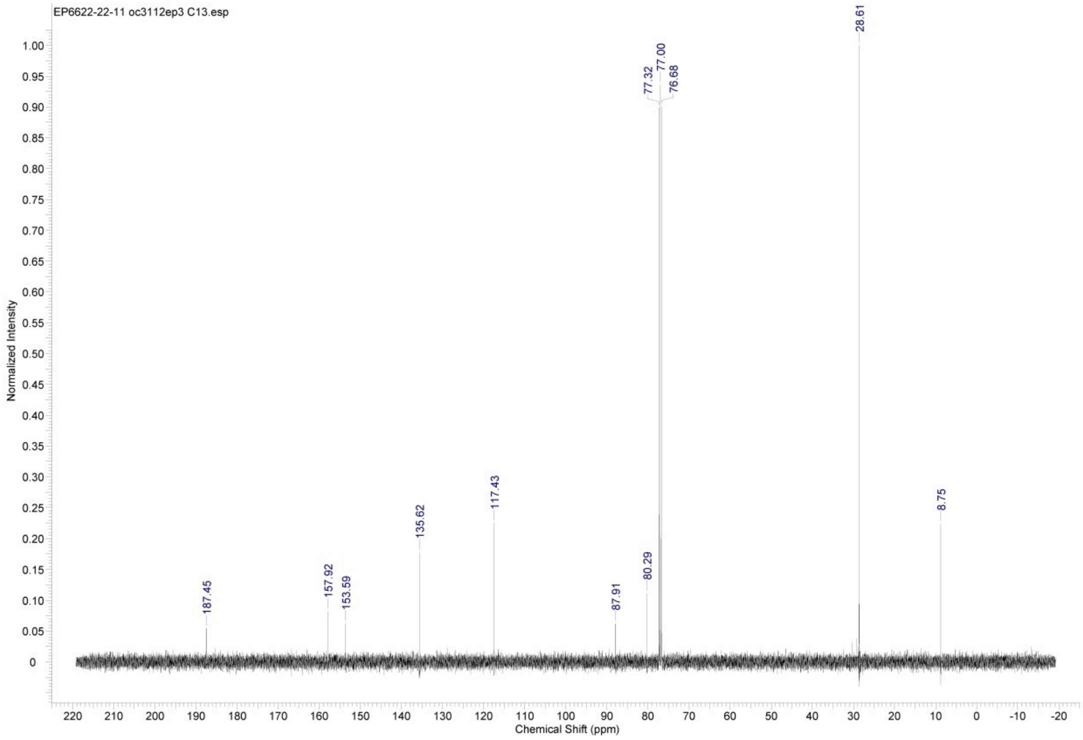

*2-(tert-Butoxy)-4-hydroxy-3,4-dimethylcyclobut-2-enone (**28a**)*

^{1}H NMR

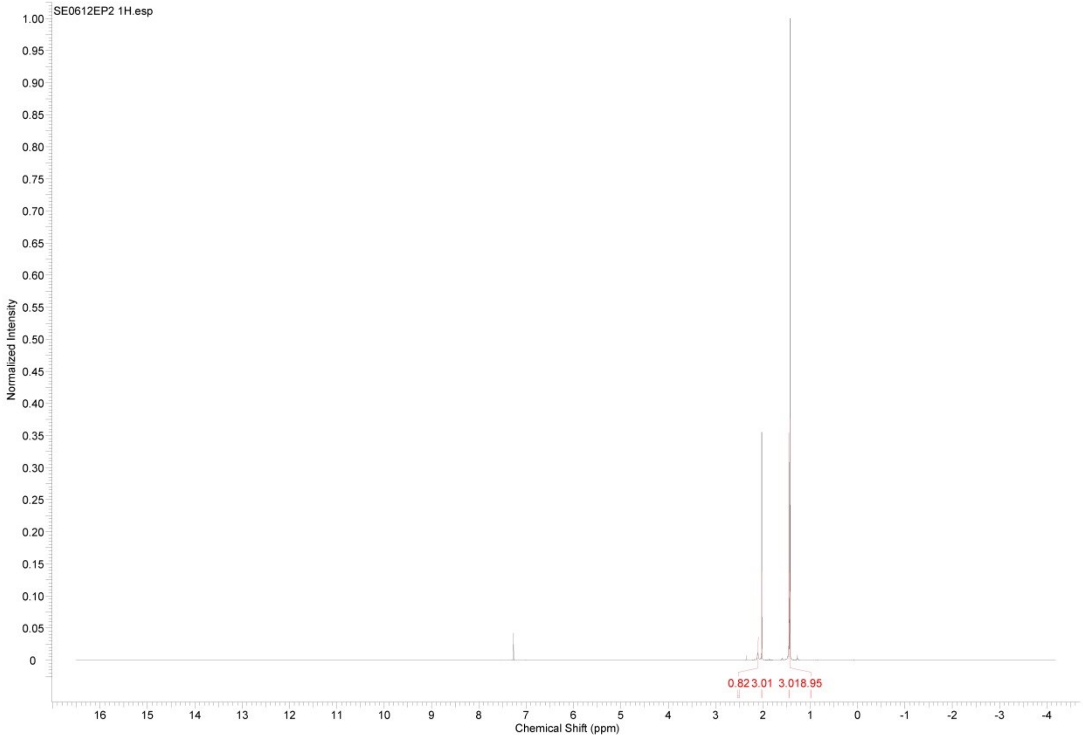

^{13}C NMR

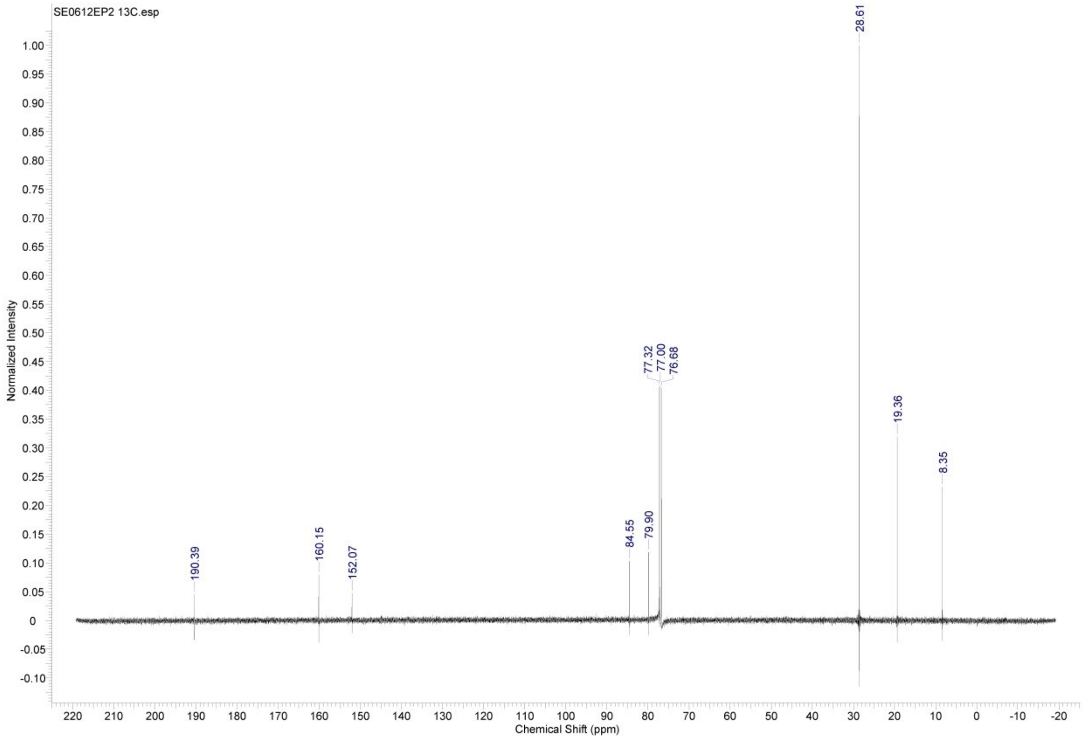

*2-(tert-Butoxy)-4-butyl-4-hydroxy-3-methylcyclobut-2-enone (**28b**)*

^{1}H NMR

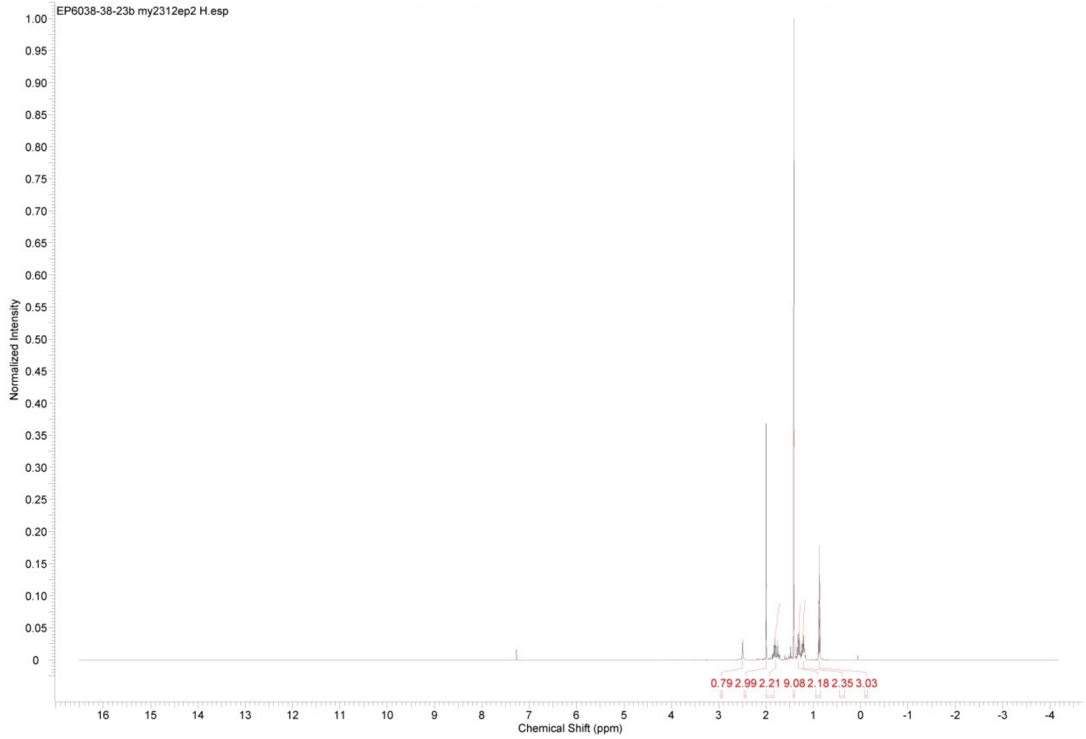

^{13}C NMR

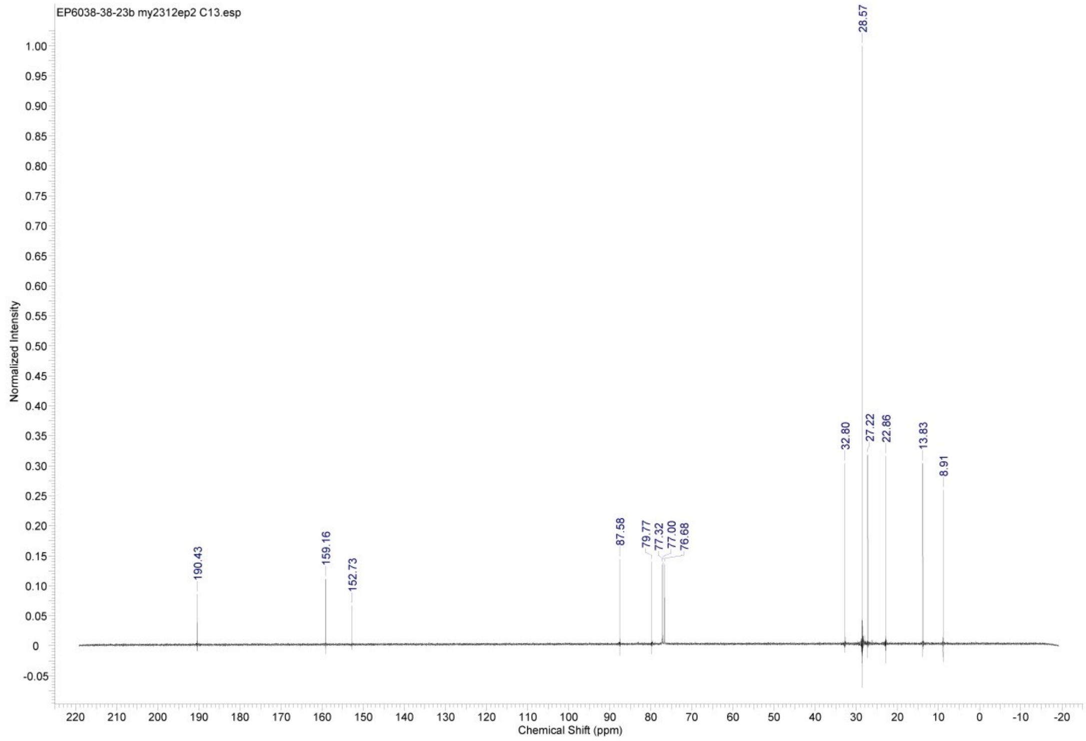

*2-(tert-Butoxy)-4-hydroxy-3-methyl-4-(phenylethynyl)cyclobut-2-enone (**34**)*

^{1}H NMR

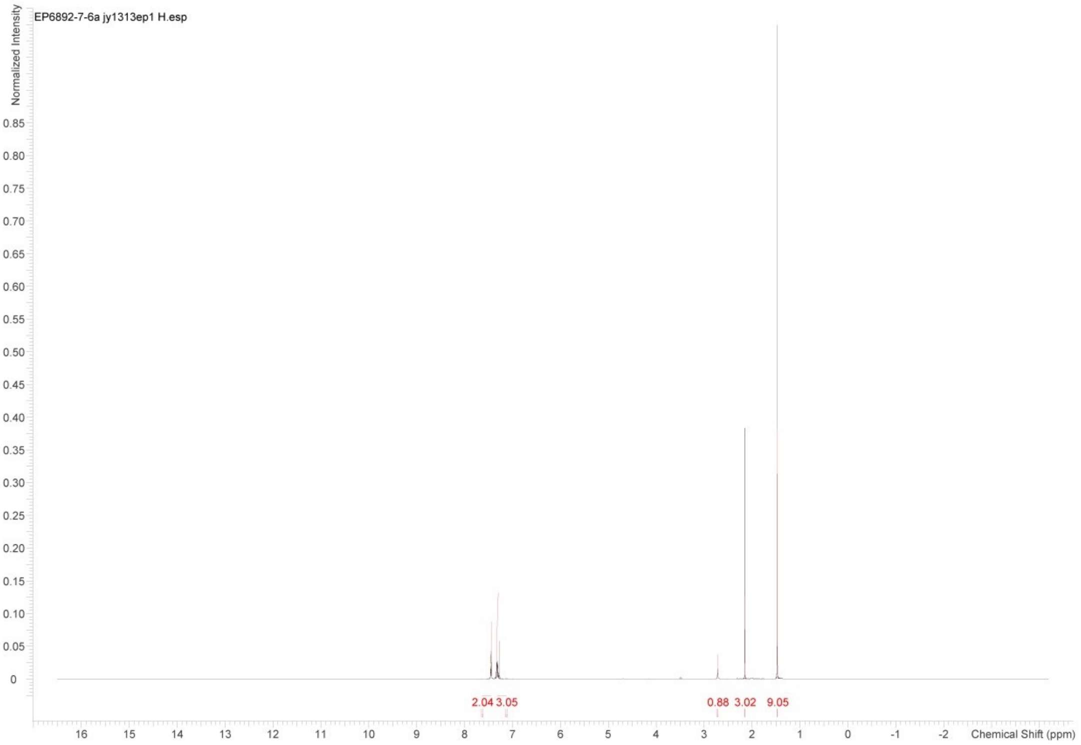

^{13}C NMR

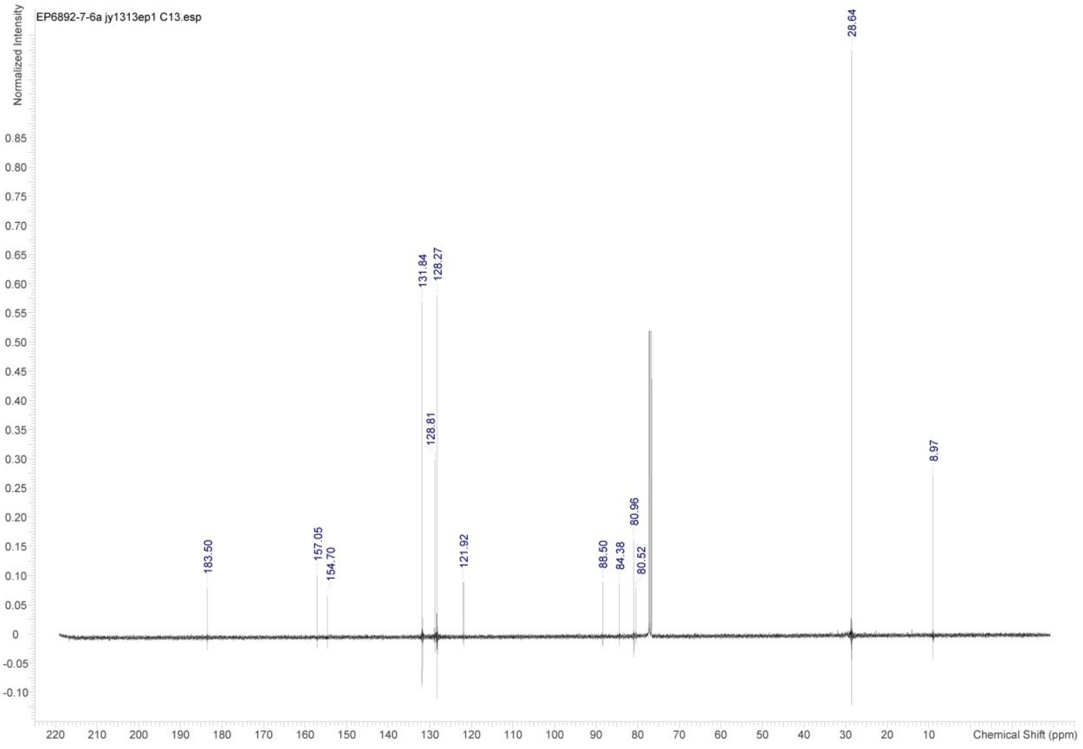

*3-(tert-butoxy)-4-hydroxy-2-methyl-4-(phenylethynyl)cyclobut-2-enone (**33**)*

^{1}H NMR

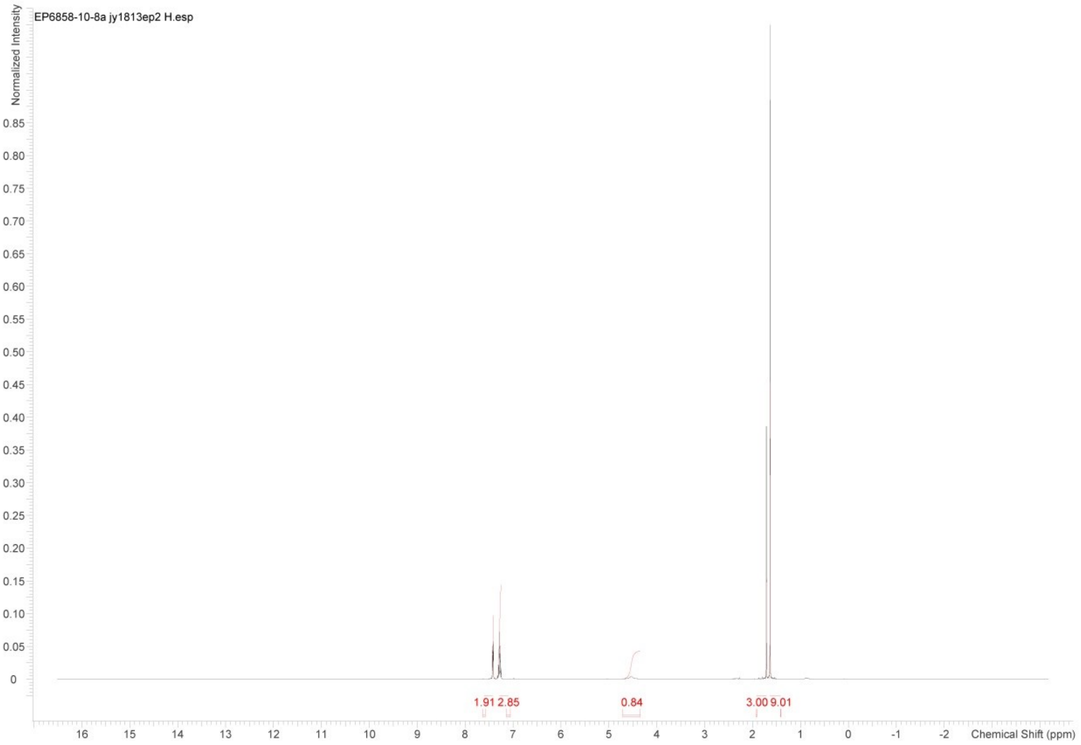

^{13}C NMR

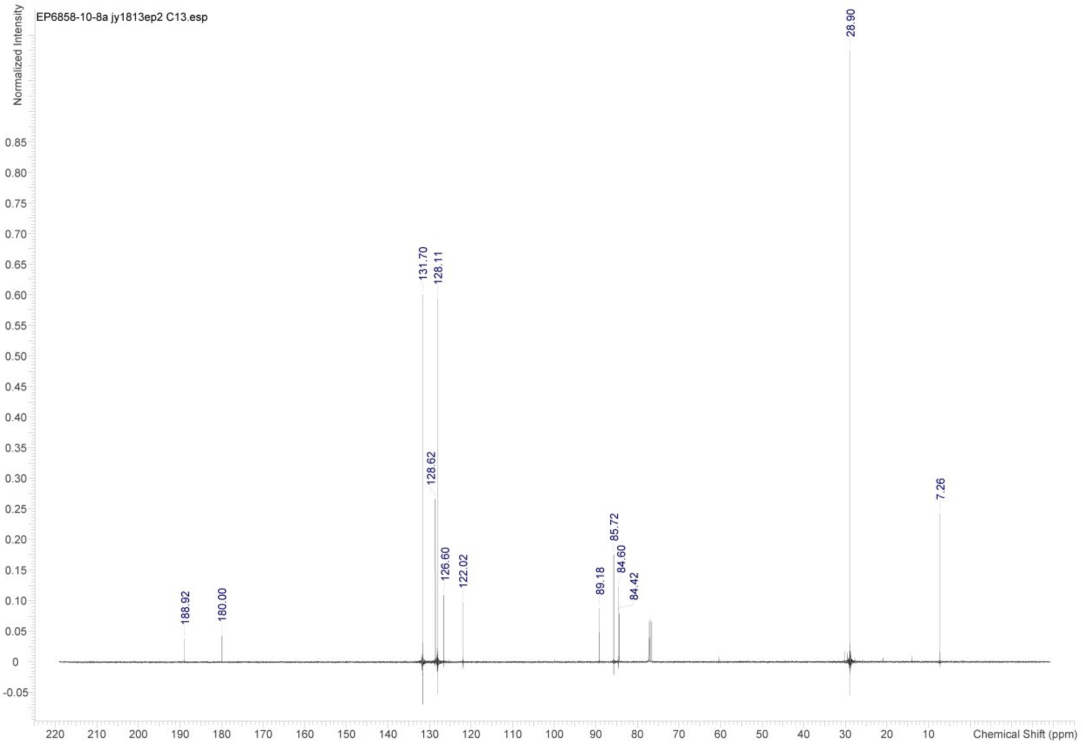

*4-Hydroxy-3-methoxy-2-methyl-4-(p-tolyl)cyclobut-2-enone (**2b**)*

^{1}H NMR

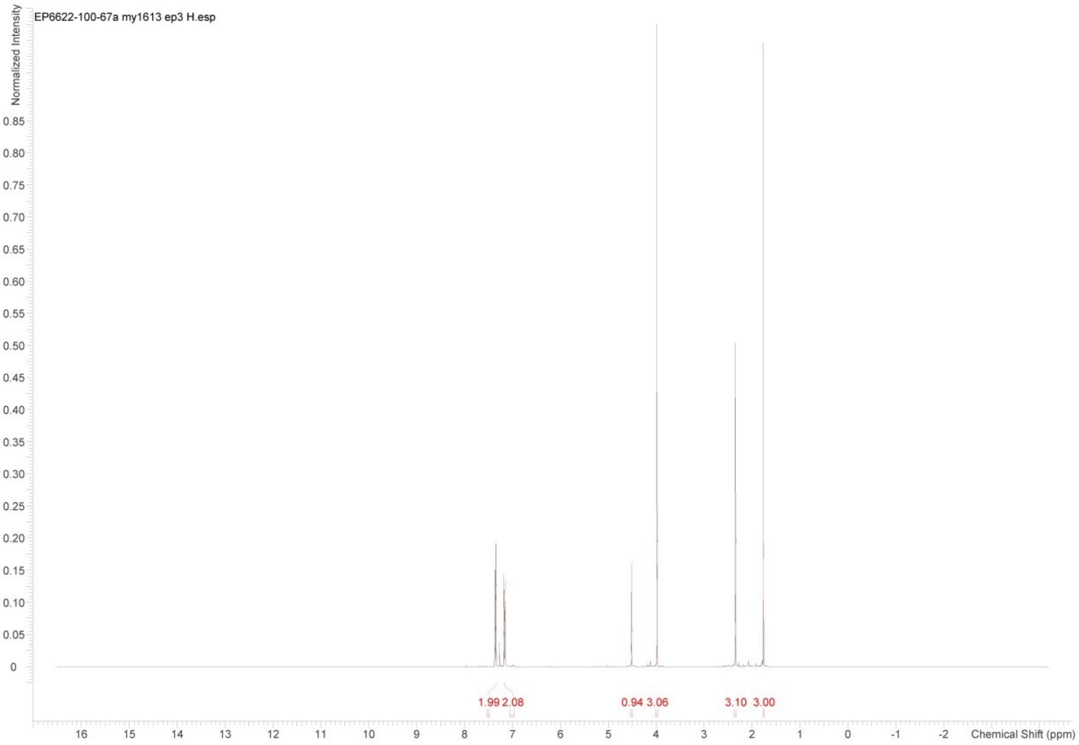

^{13}C NMR

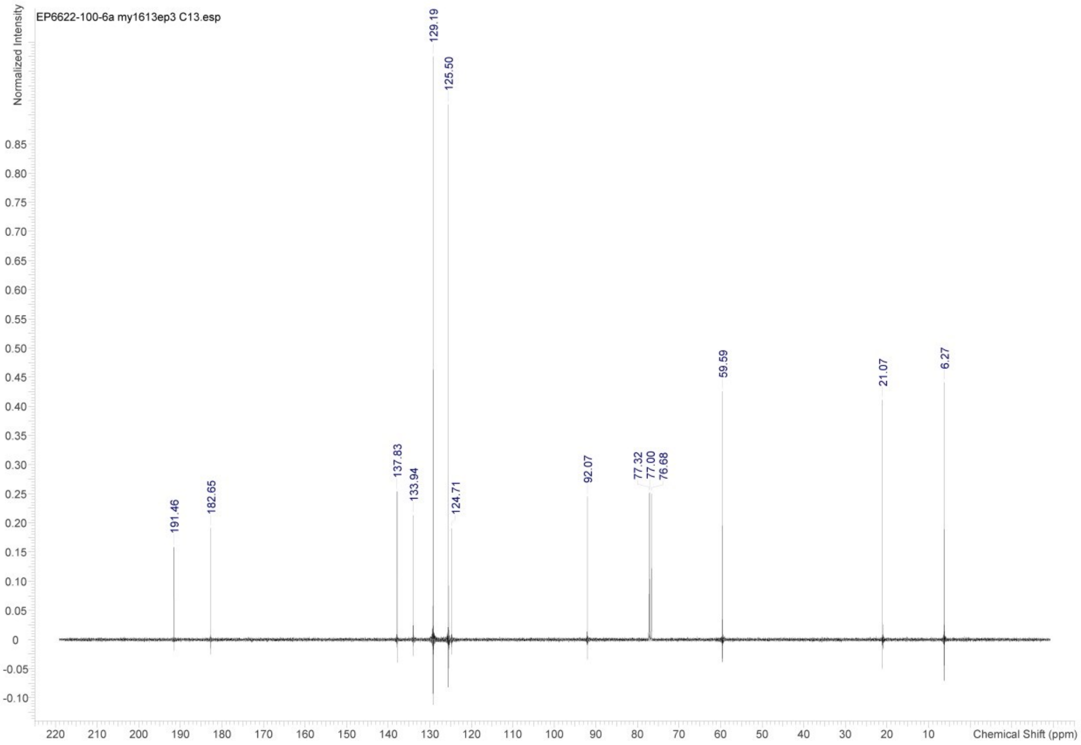

*4-Hydroxy-3-methoxy-4-(4-methoxyphenyl)-2-methylcyclobut-2-enone (**2c**)*

^{1}H NMR

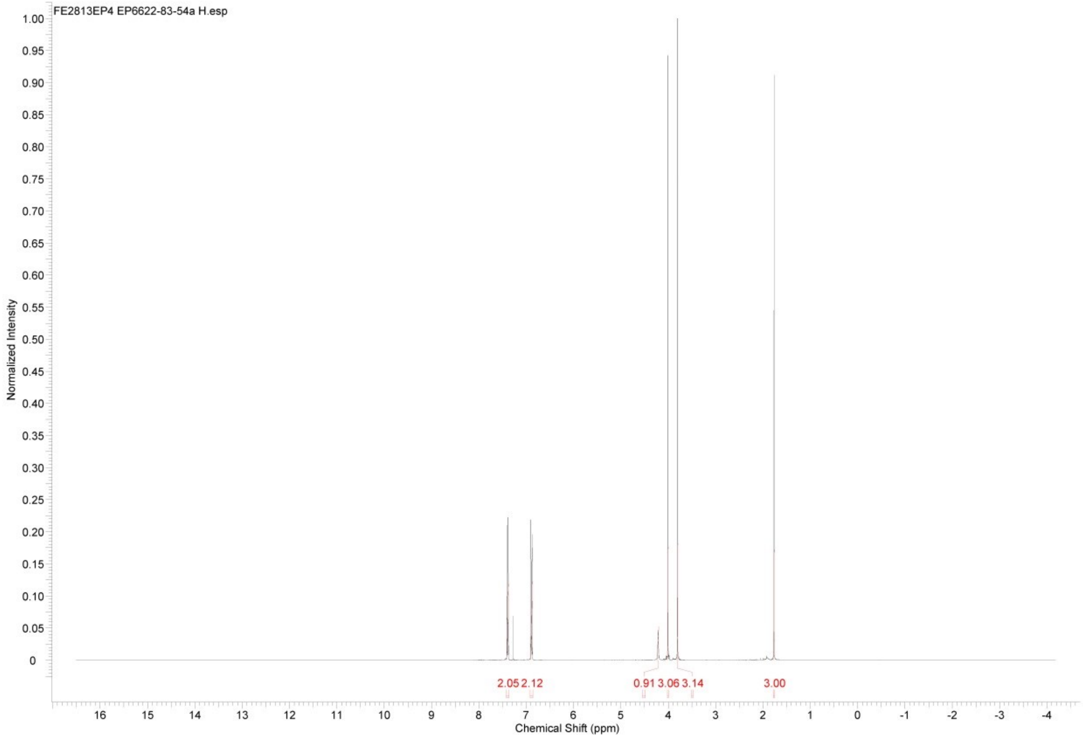

^{13}C NMR

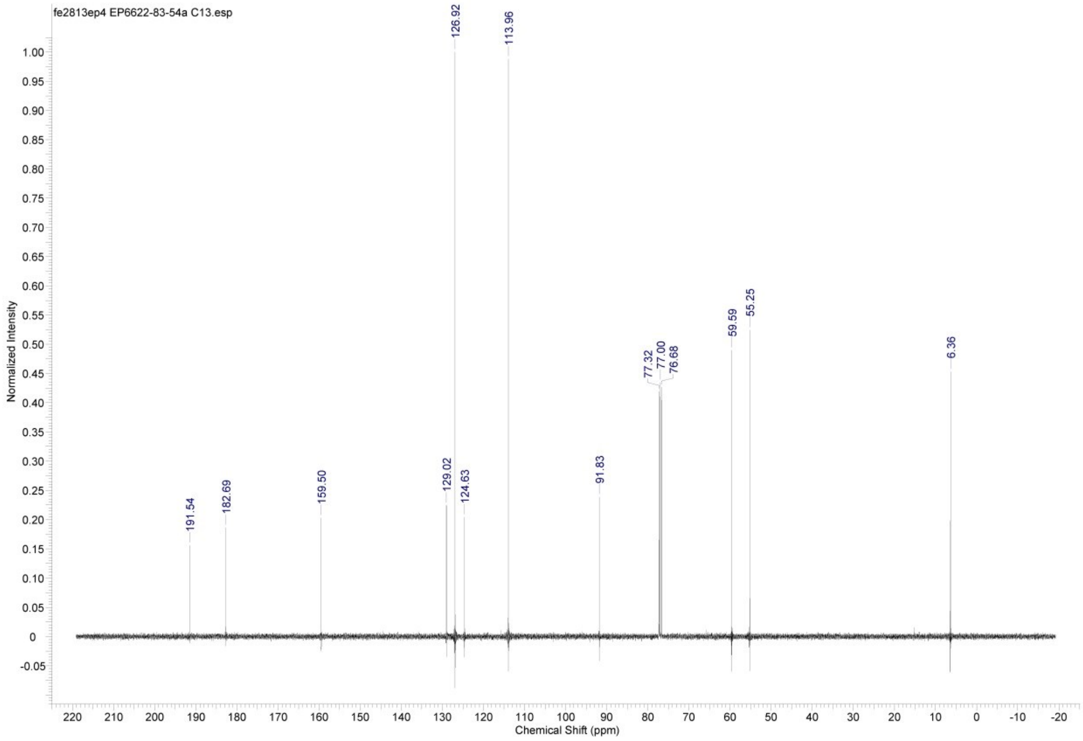

*4-Hydroxy-3-methoxy-2-methyl-4-(4-(trifluoromethyl)phenyl)cyclobut-2-enone (**2d**)*

^{1}H NMR

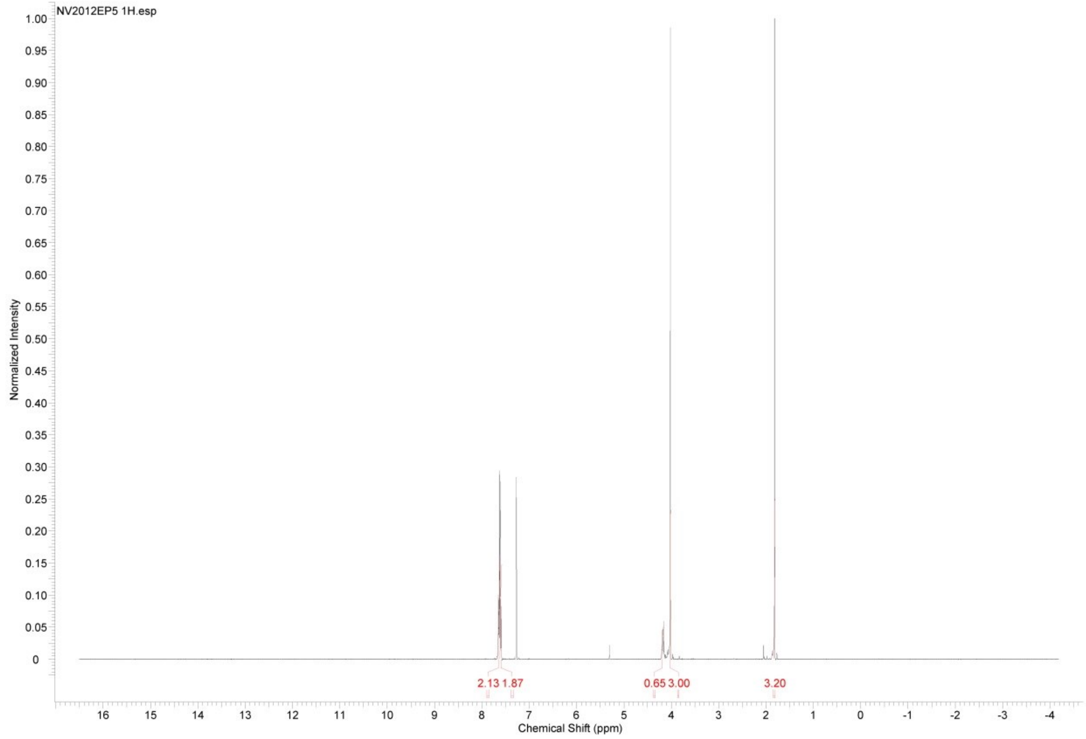

^{13}C NMR

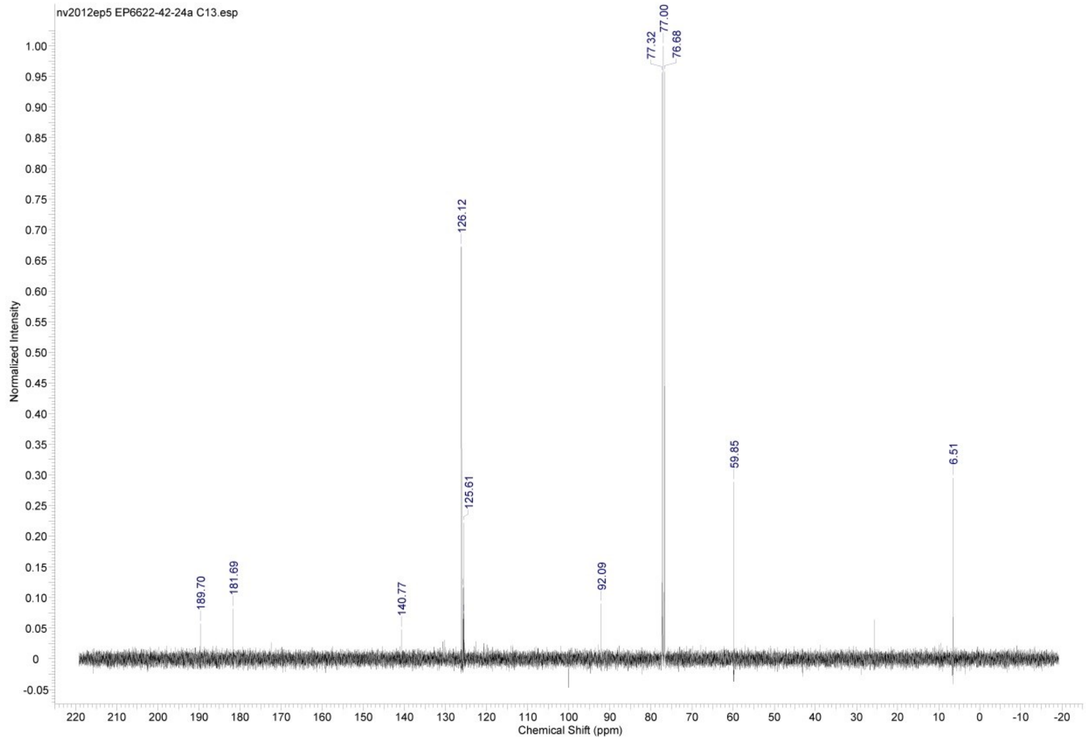

*3-Methoxy-4-methyl-[1,1'-biphenyl]-2,5-diol (**27**)*

^{1}H NMR

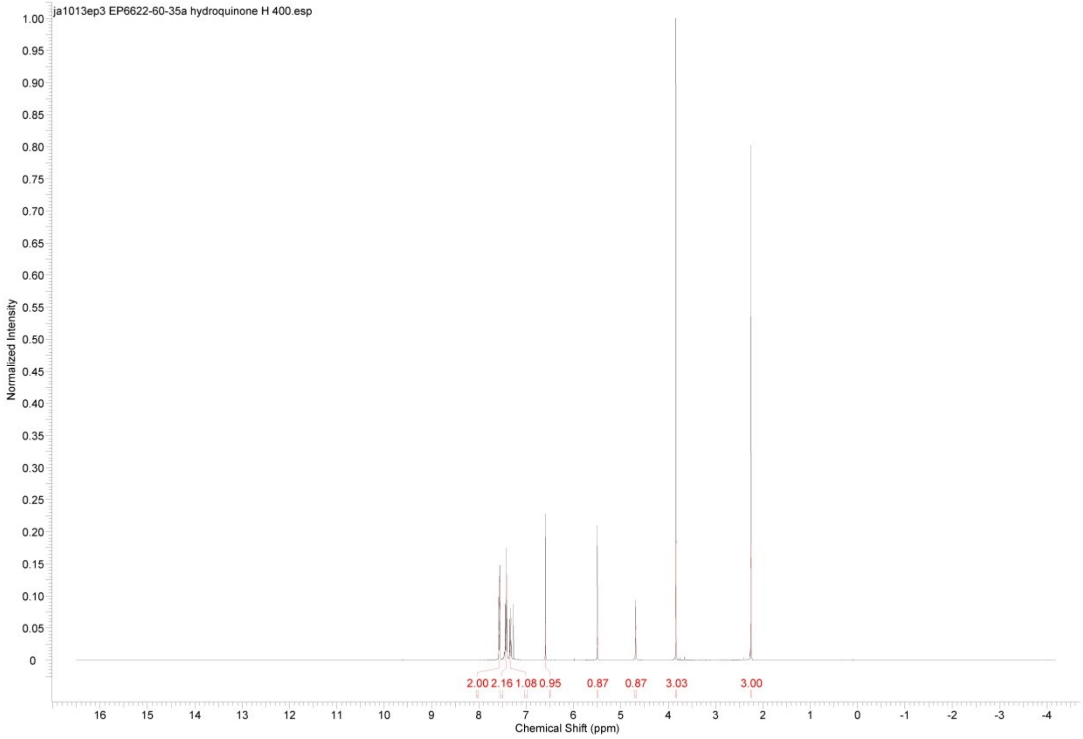

^{13}C NMR

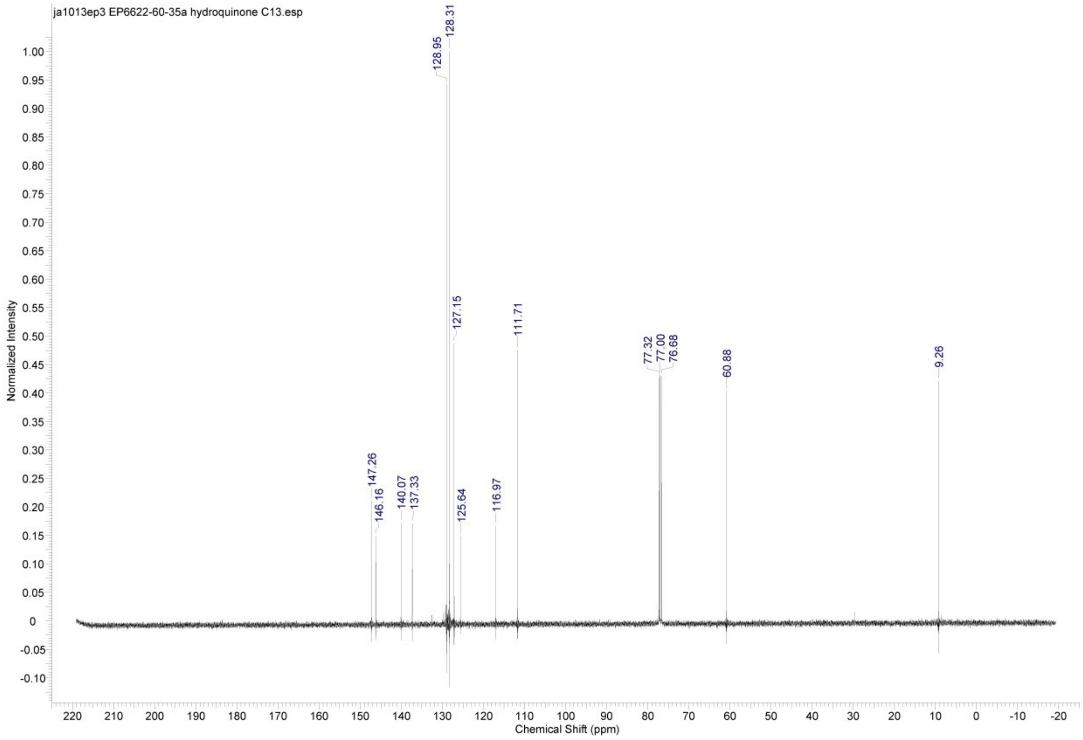

*4-Hydroxy-2-methoxy-3-methyl-4-(1-phenylvinyl)cyclobut-2-enone (**21b**)*

^{1}H NMR

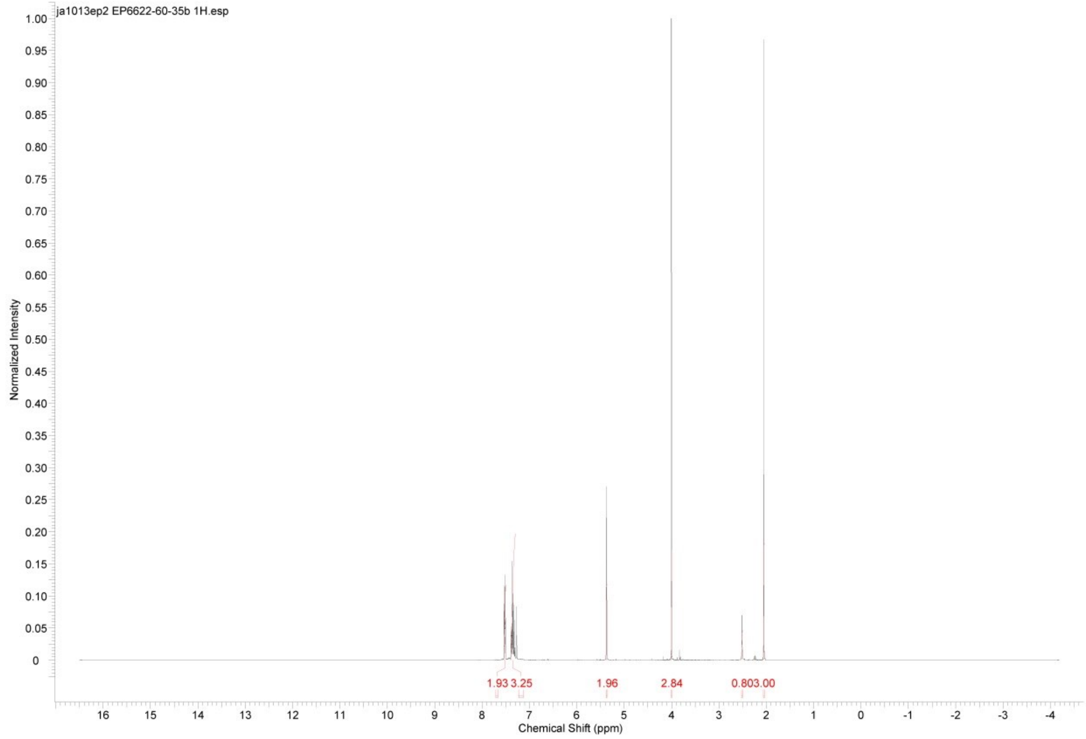

^{13}C NMR

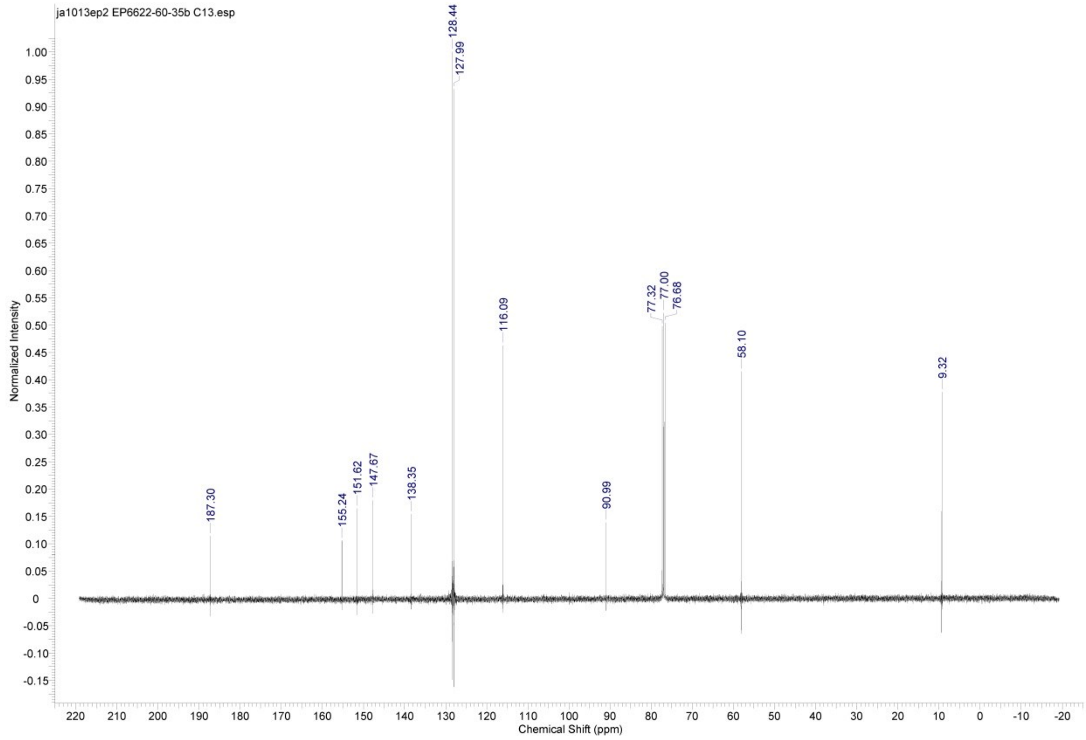

*4-Hydroxy-3-methoxy-2-methyl-4-(phenylethynyl)cyclobut-2-enone (**32**)*

^{1}H NMR

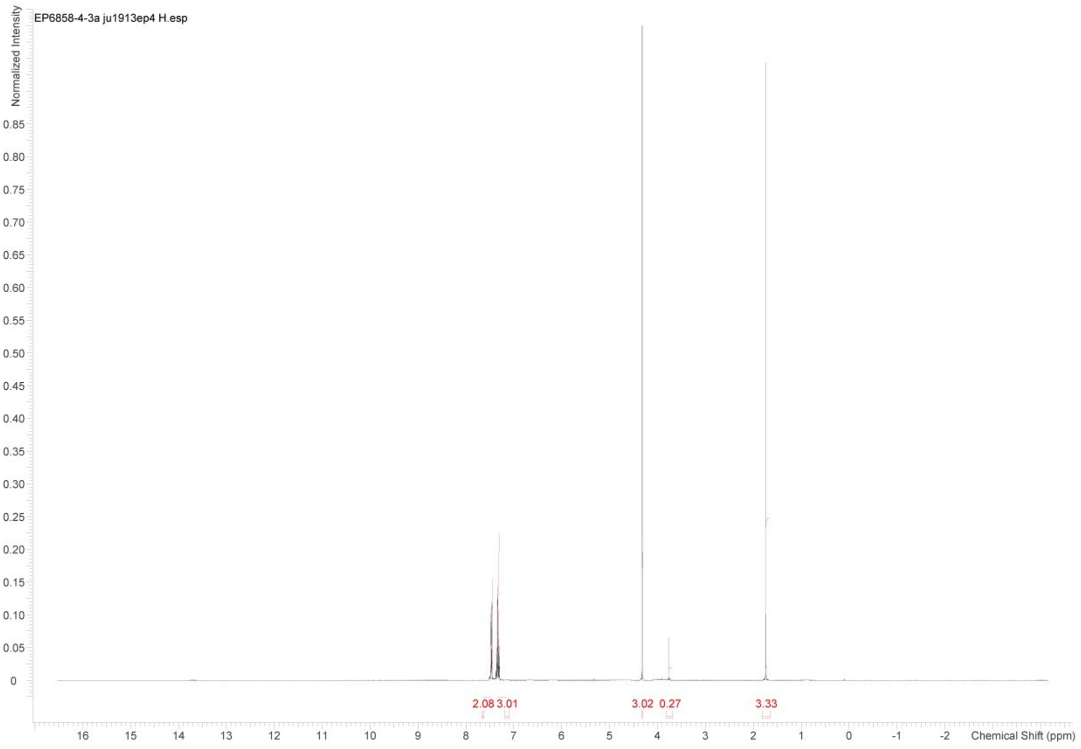

^{13}C NMR

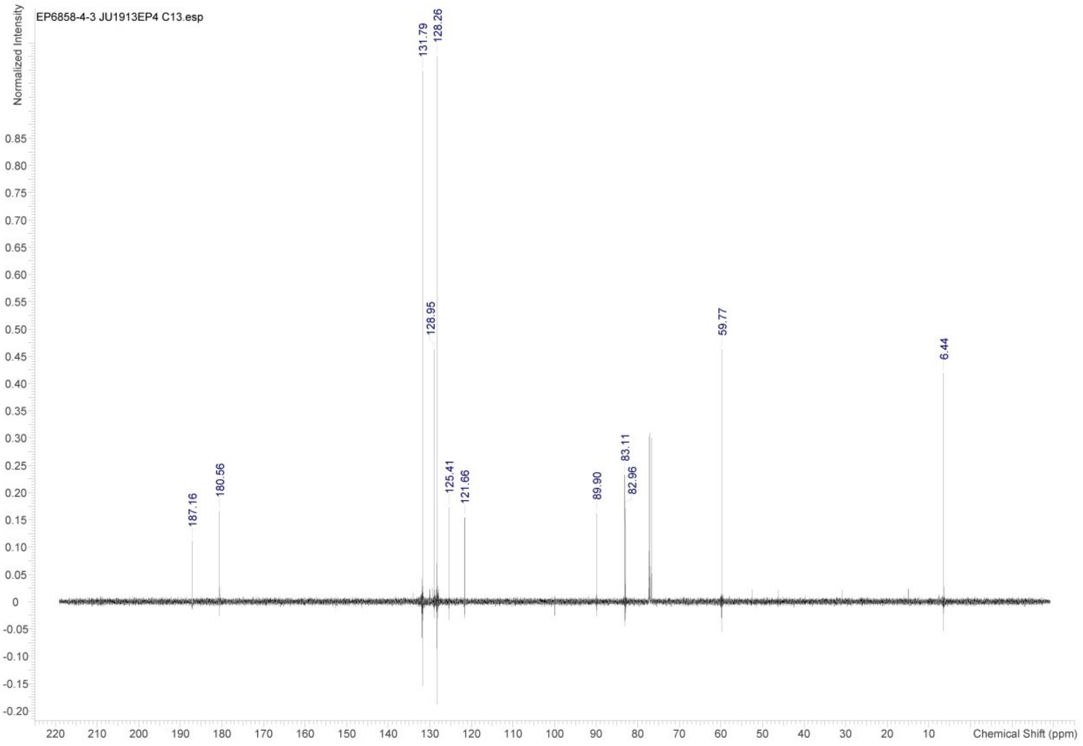

*4-Hydroxy-3-methoxy-2,4-dimethylcyclobut-2-enone (**50**)*

^{1}H NMR

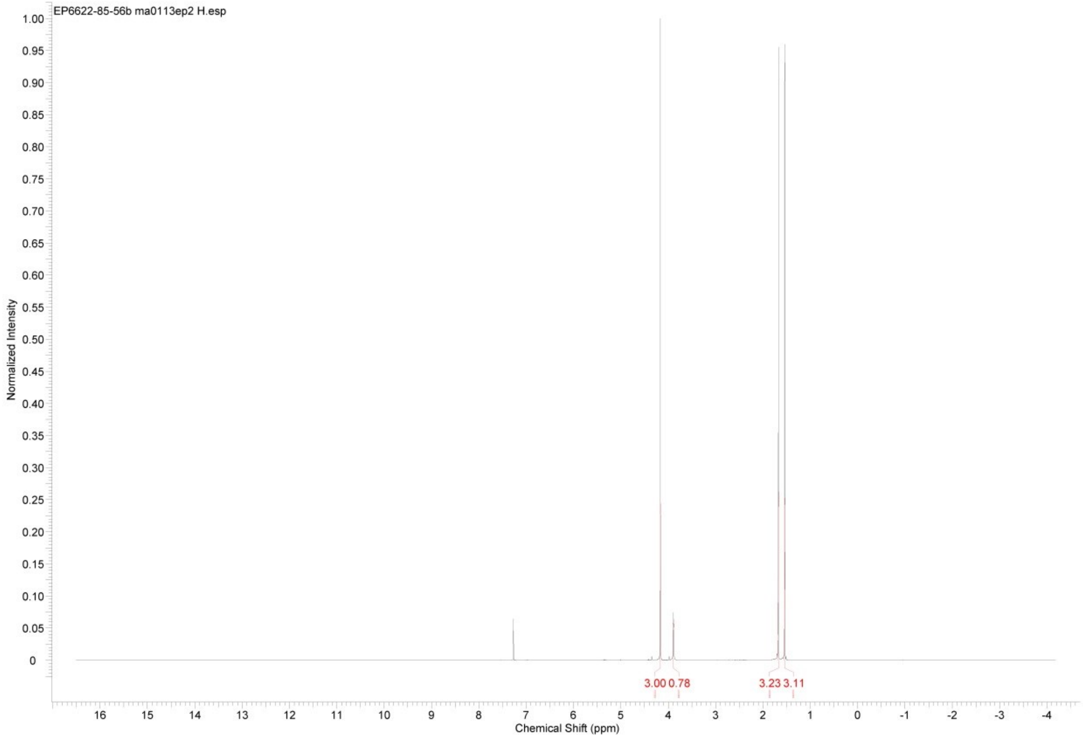

^{13}C NMR

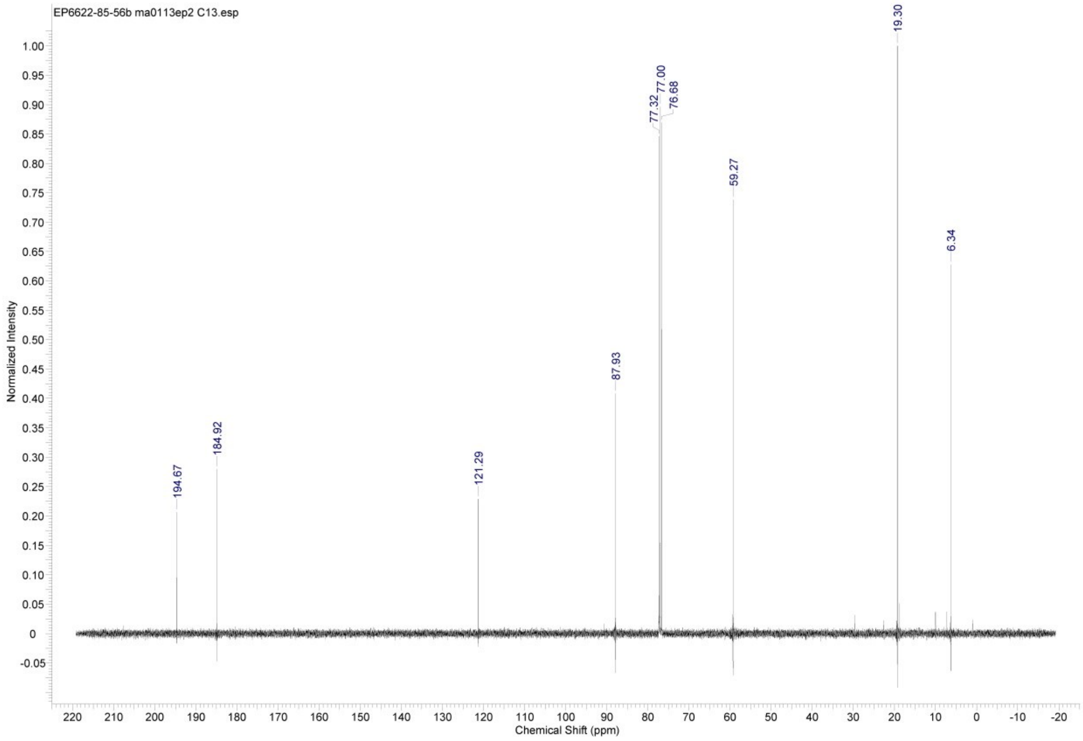

*4-Hydroxy-3-methoxy-4-(2-methoxyphenyl)-2-methylcyclobut-2-enone (**2e**)*

^{1}H NMR

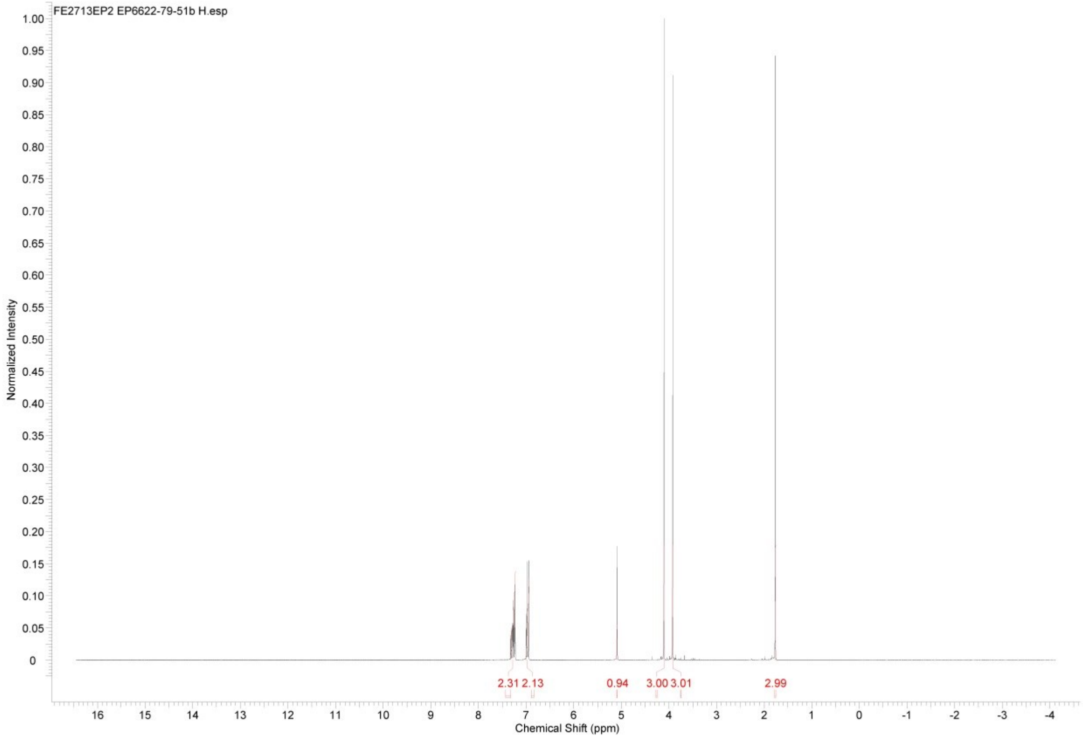

^{13}C NMR

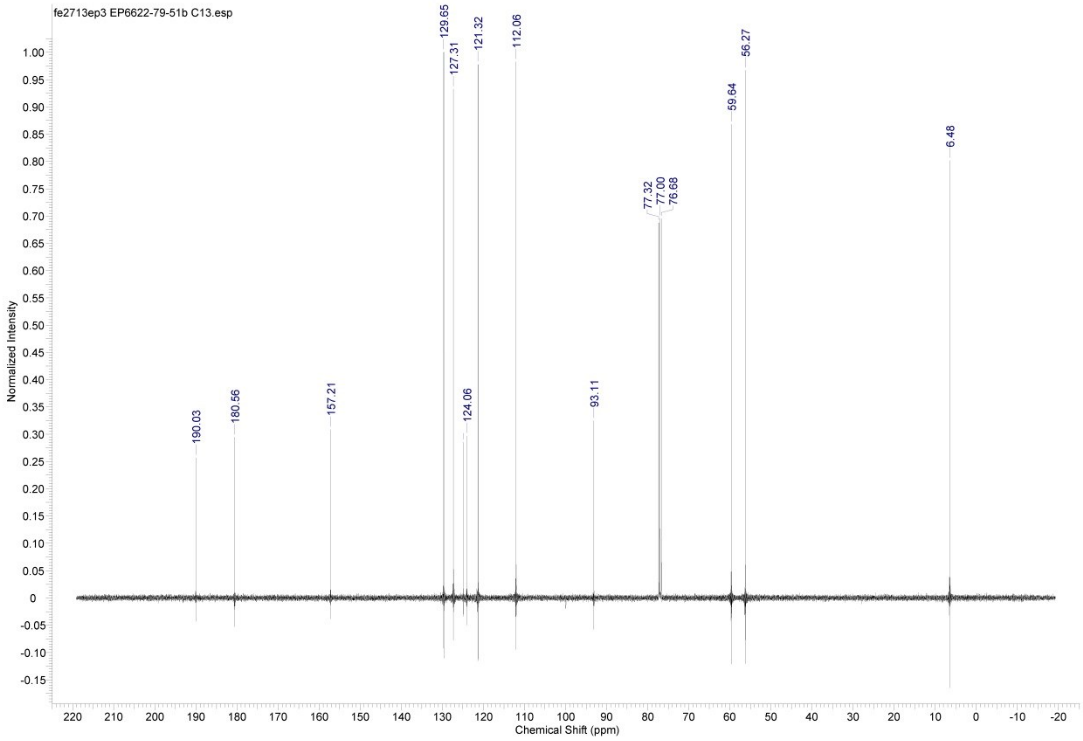

*2-(tert-butoxy)-4-hydroxy-4-(4-methoxyphenyl)-3-methylcyclobut-2-enone (**10c**)*

^{1}H NMR

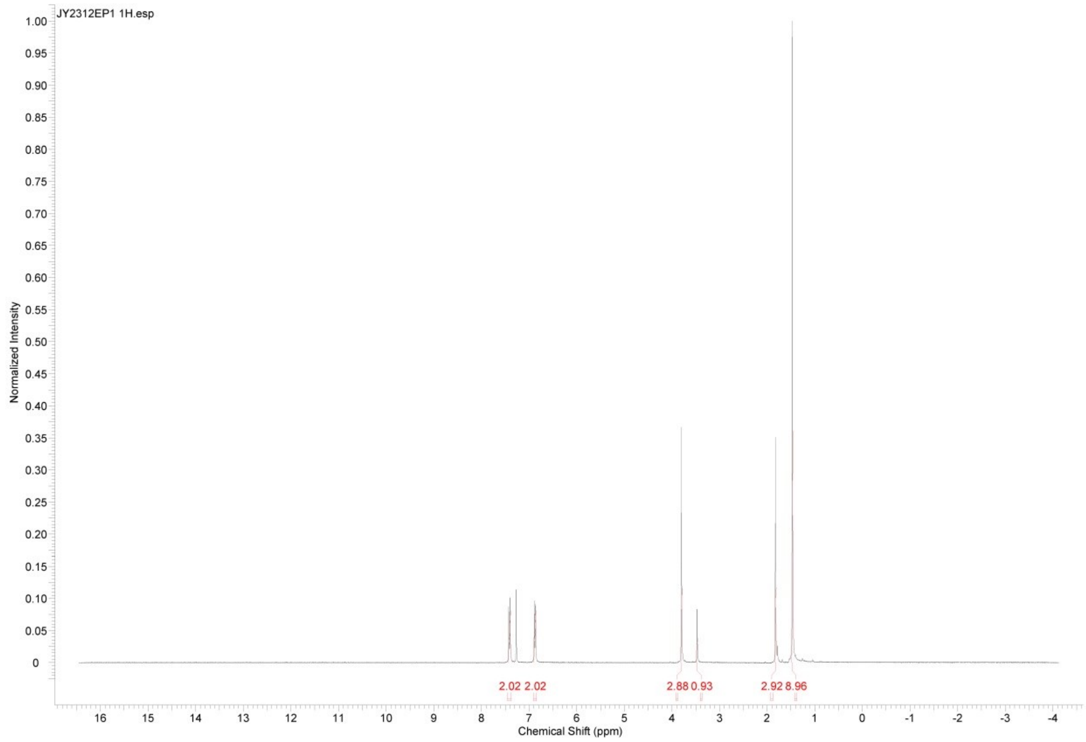

^{13}C NMR

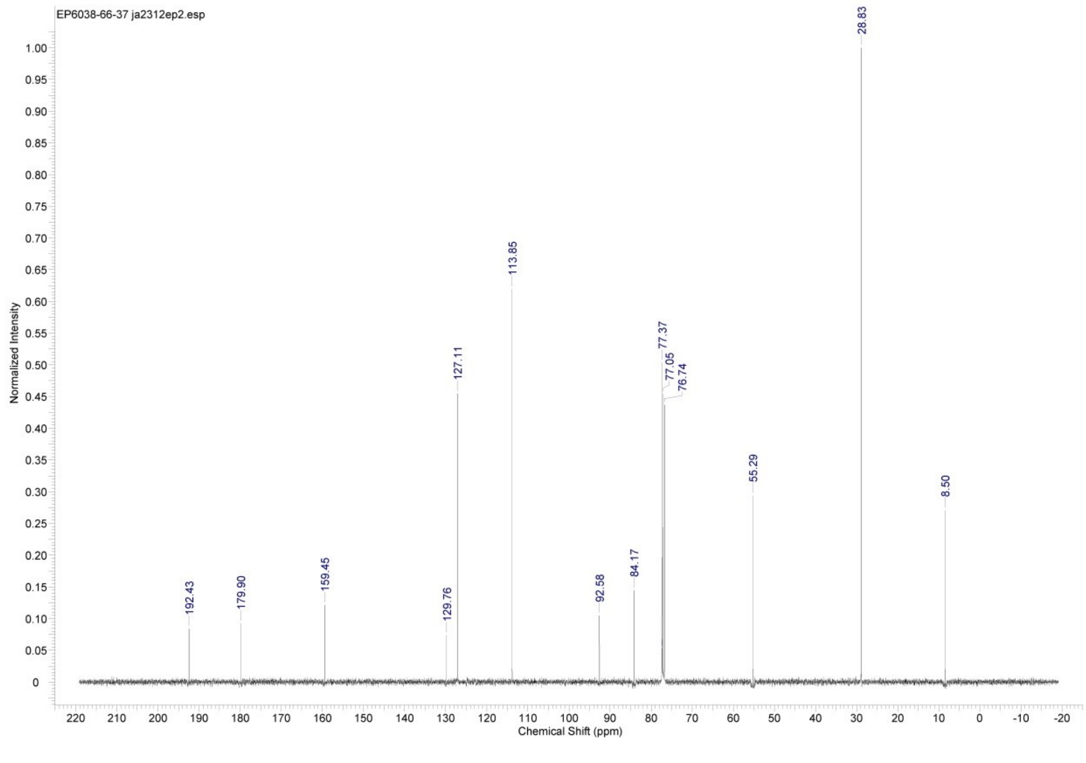

3-(tert-butoxy)-4-hydroxy-2-methyl-4-phenylcyclobut-2-enone (**10a**)

^{1}H NMR

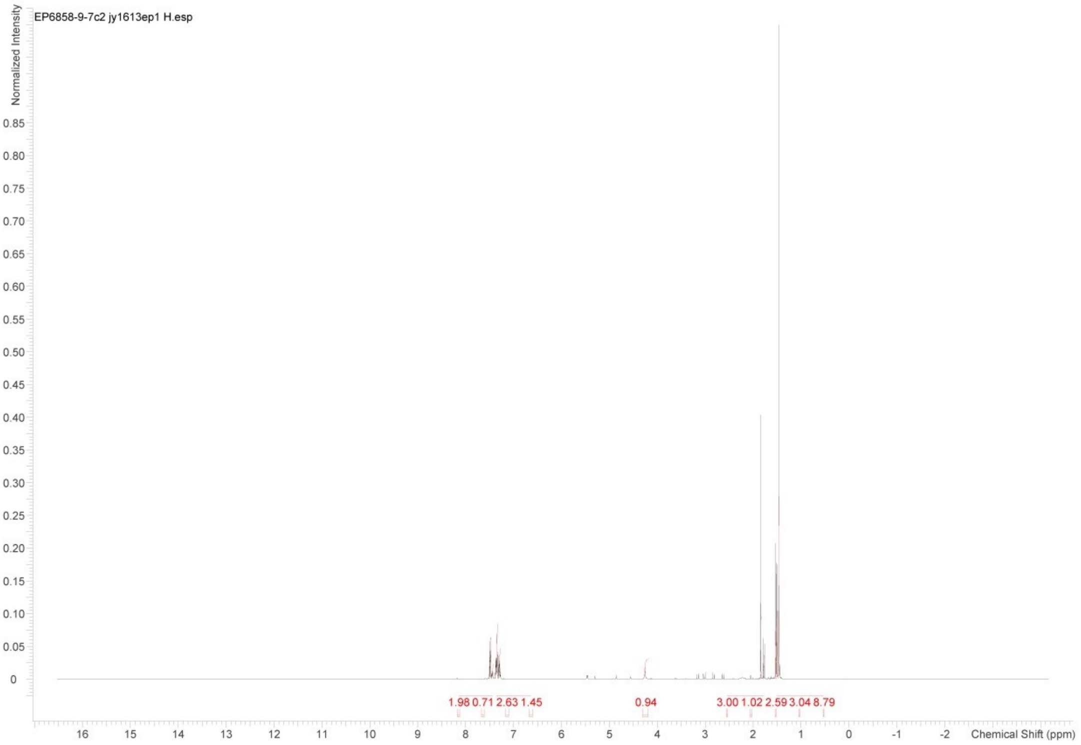

^{13}C NMR

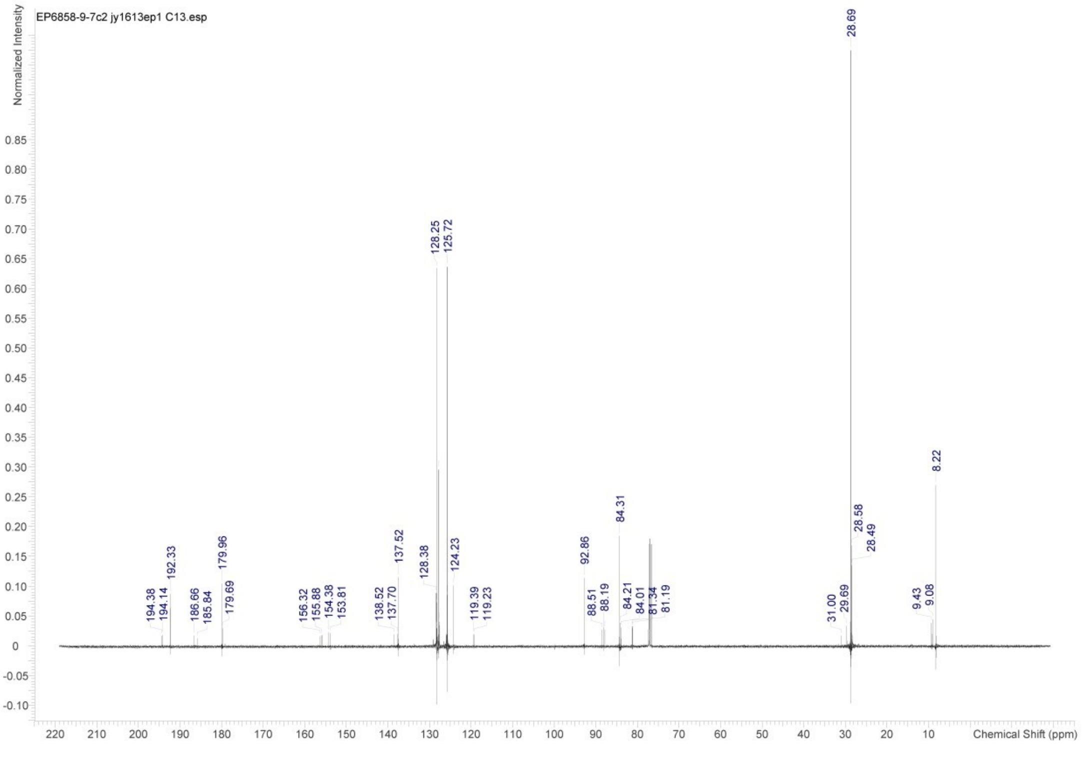

2-Methoxy-3-methylnaphthalene-1,4-dione (**15a**)

^{1}H NMR

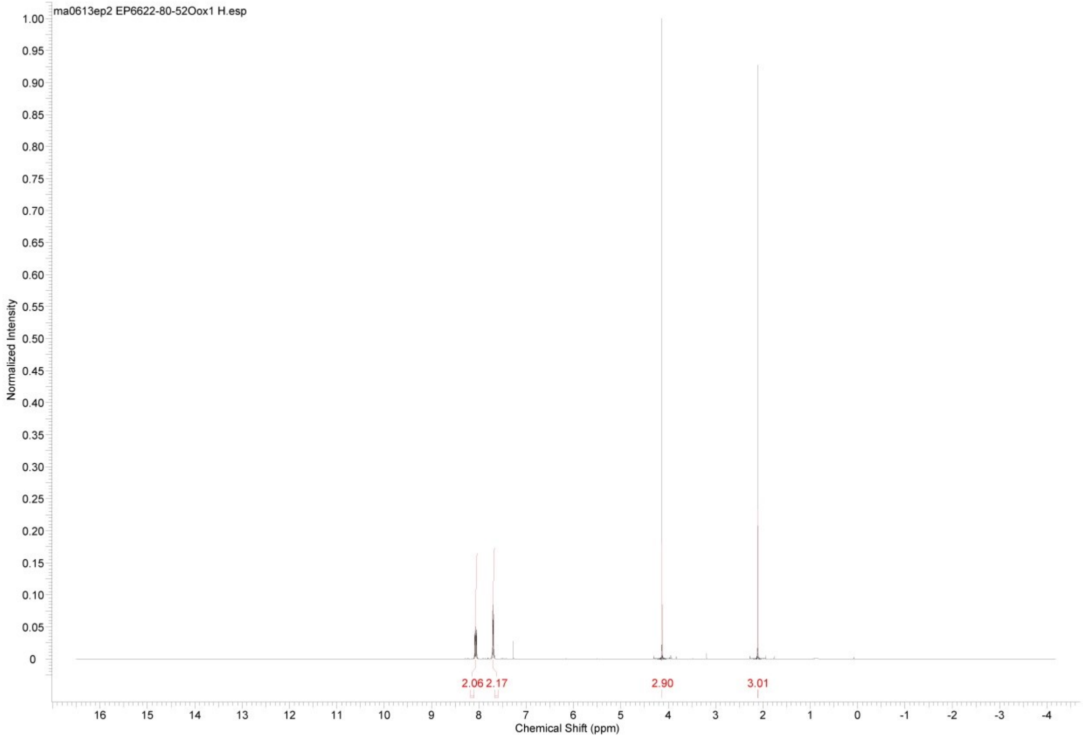

^{13}C NMR

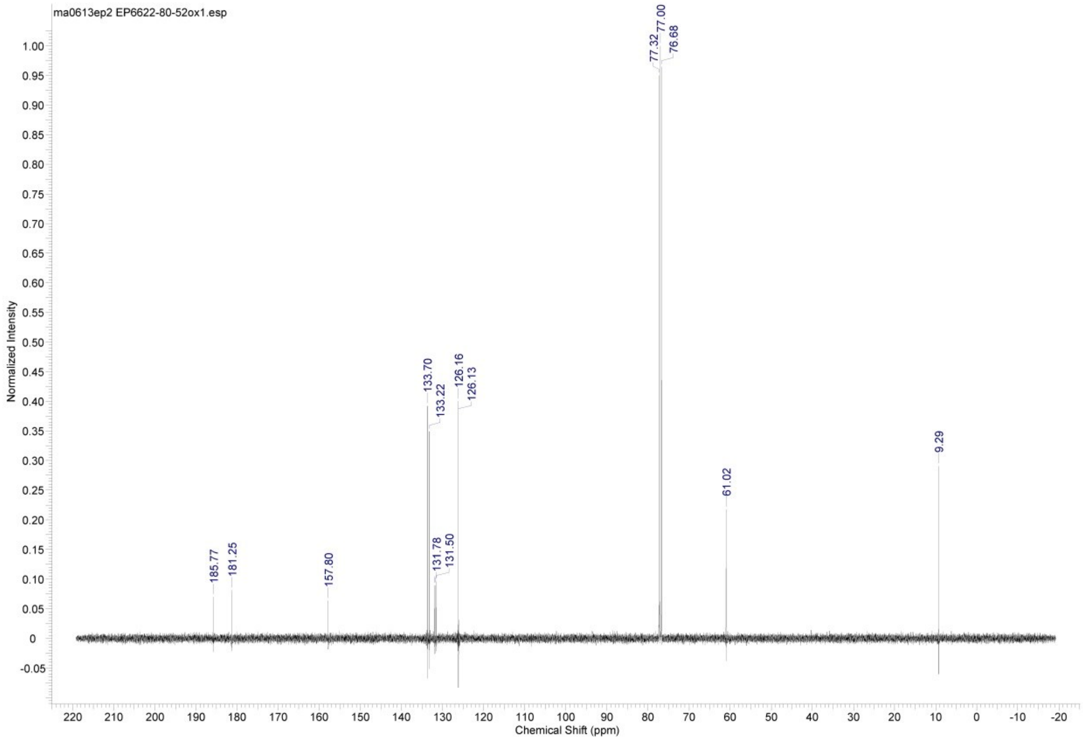

*2-Methoxy-3,6-dimethylnaphthalene-1,4-dione (**15b**)*

^{1}H NMR

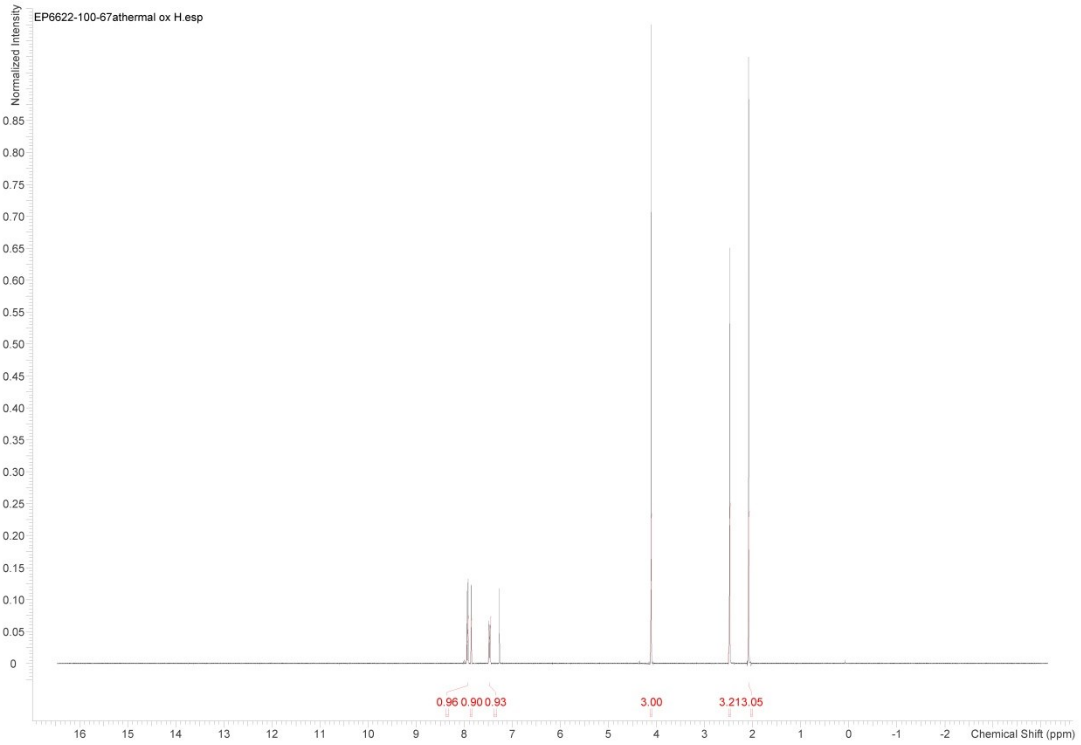

^{13}C NMR

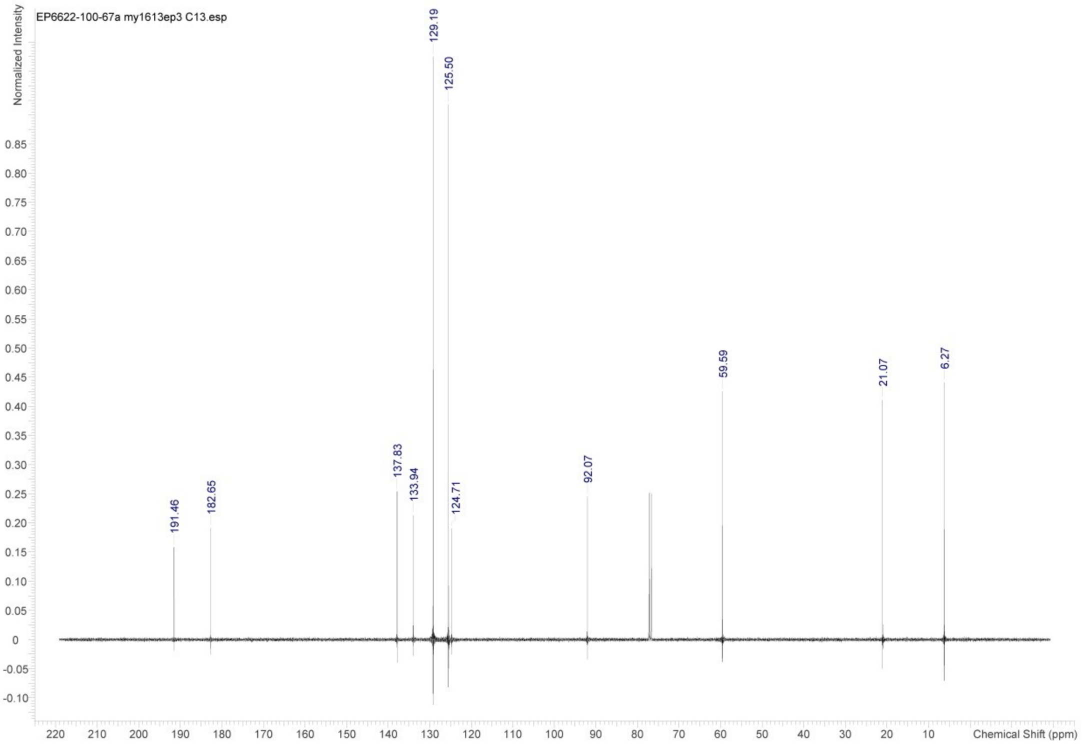

*2,6-Dimethoxy-3-methylnaphthalene-1,4-dione (**15c**)*

^{1}H NMR

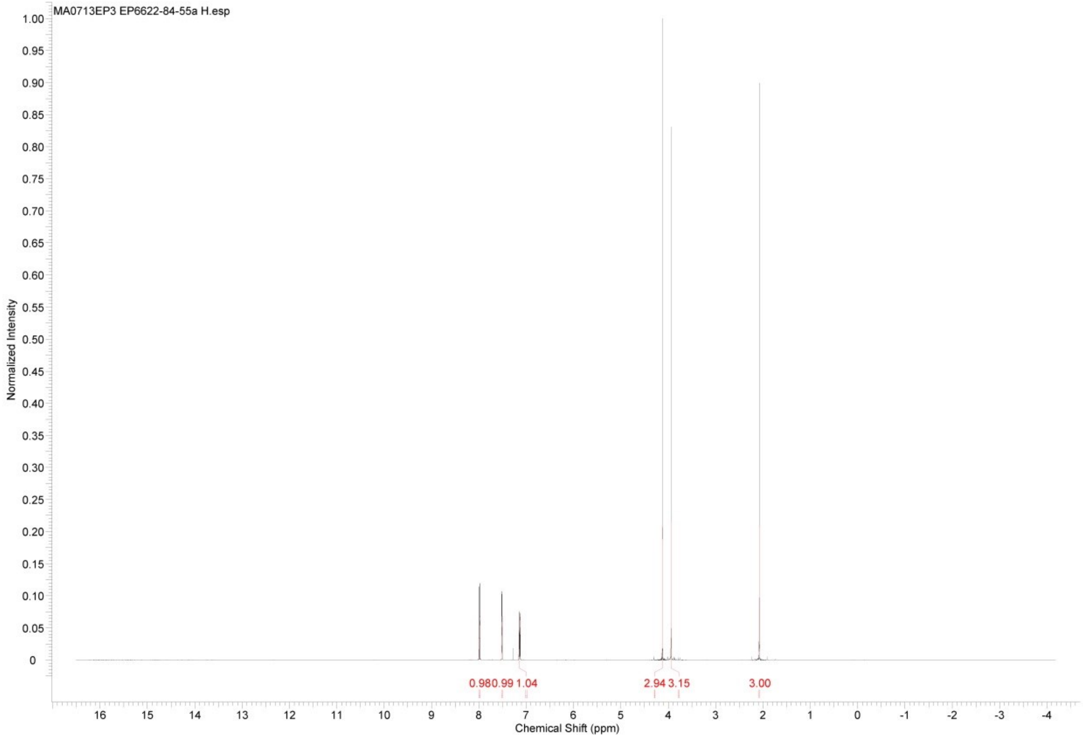

^{13}C NMR

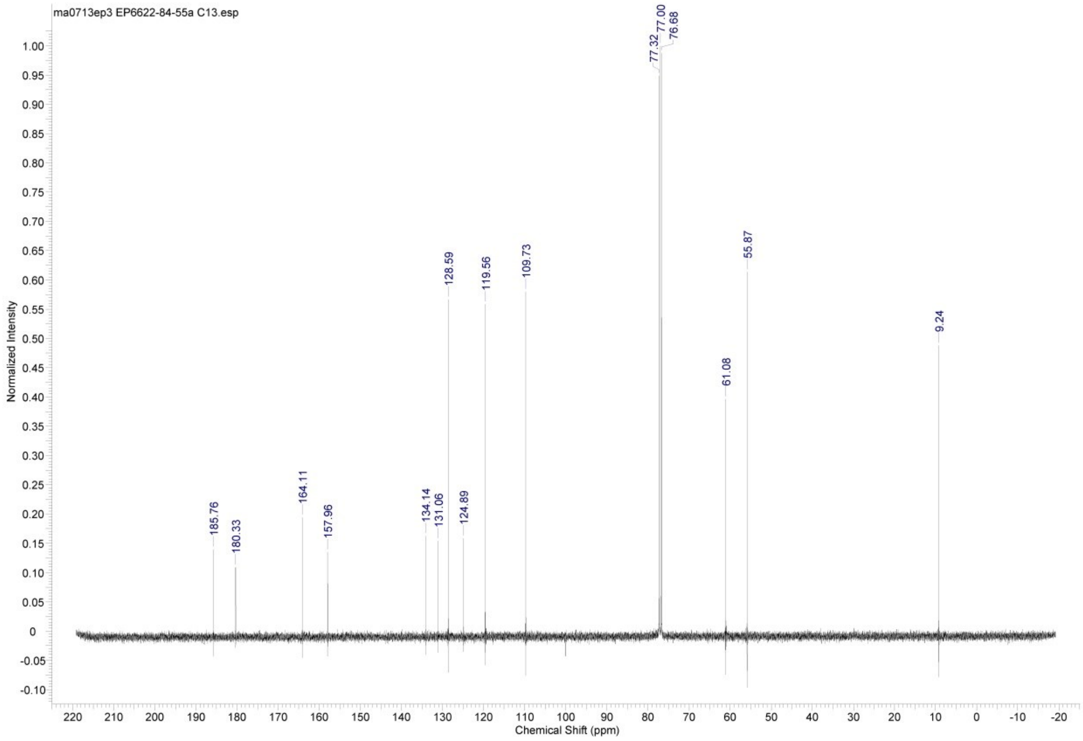

*2-Methoxy-3-methyl-6-(trifluoromethyl)naphthalene-1,4-dione (**15d**)*

^{1}H NMR

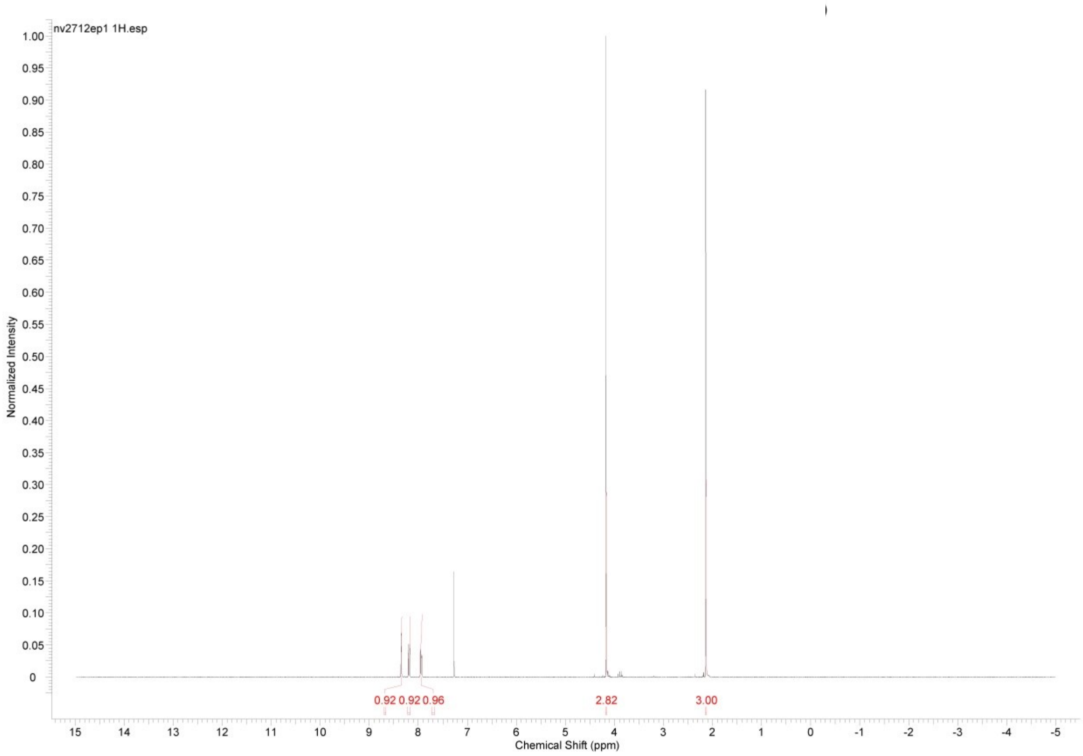

^{13}C NMR

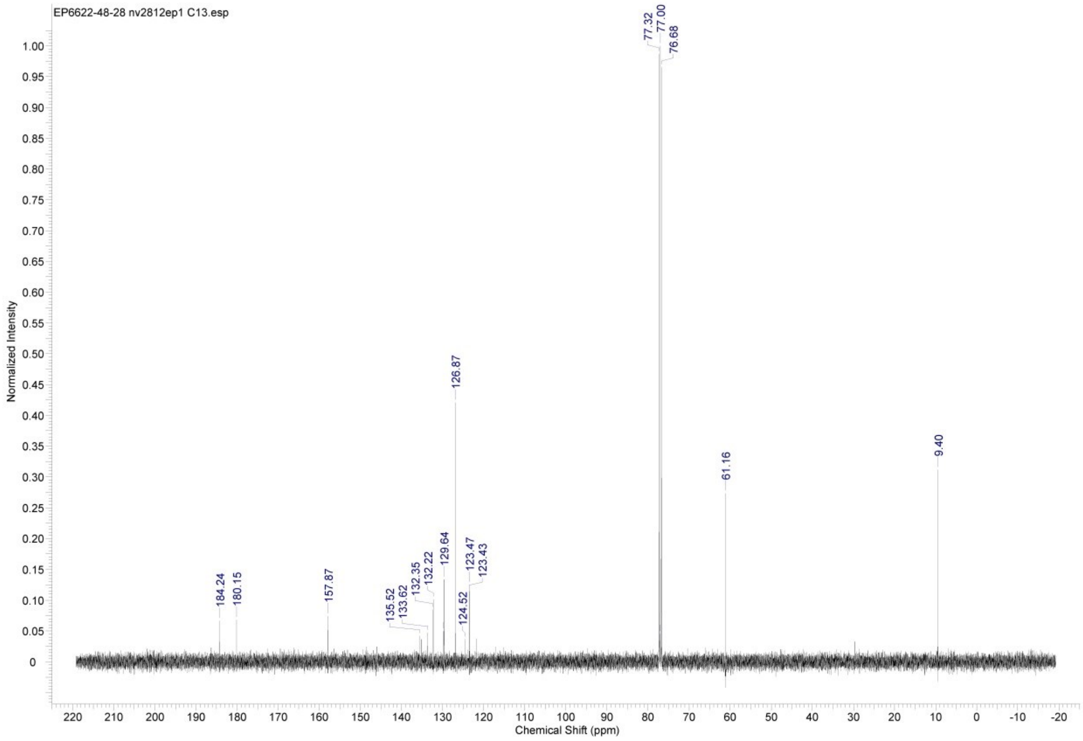

*2-(tert-Butoxy)-3-methylnaphthalene-1,4-dione (**16a**)*

^{1}H NMR

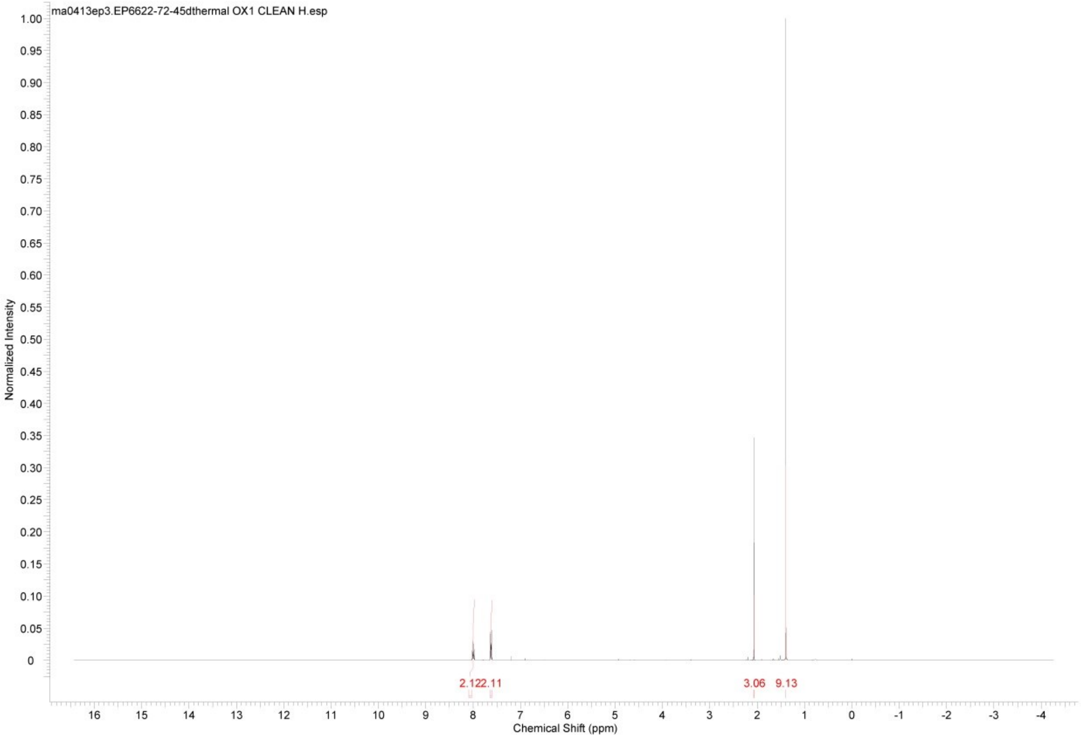

^{13}C NMR

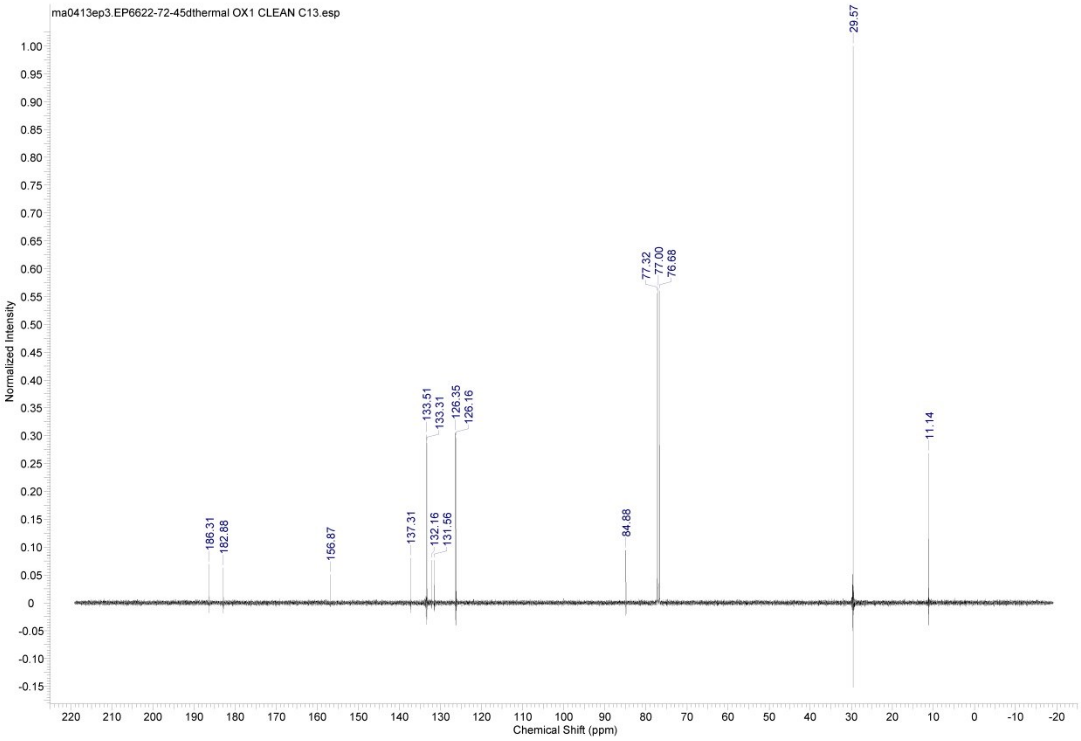

*3-(tert-Butoxy)-2,6-dimethylnaphthalene-1,4-dione (**16b**)*

^{1}H NMR

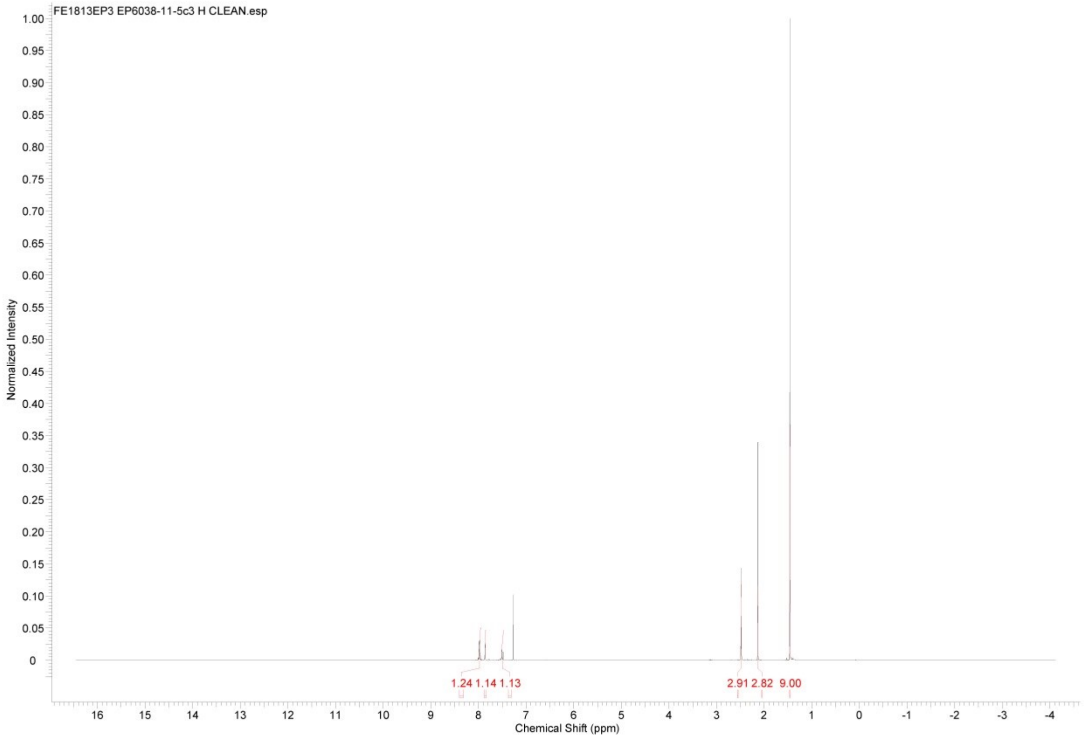

^{13}C NMR

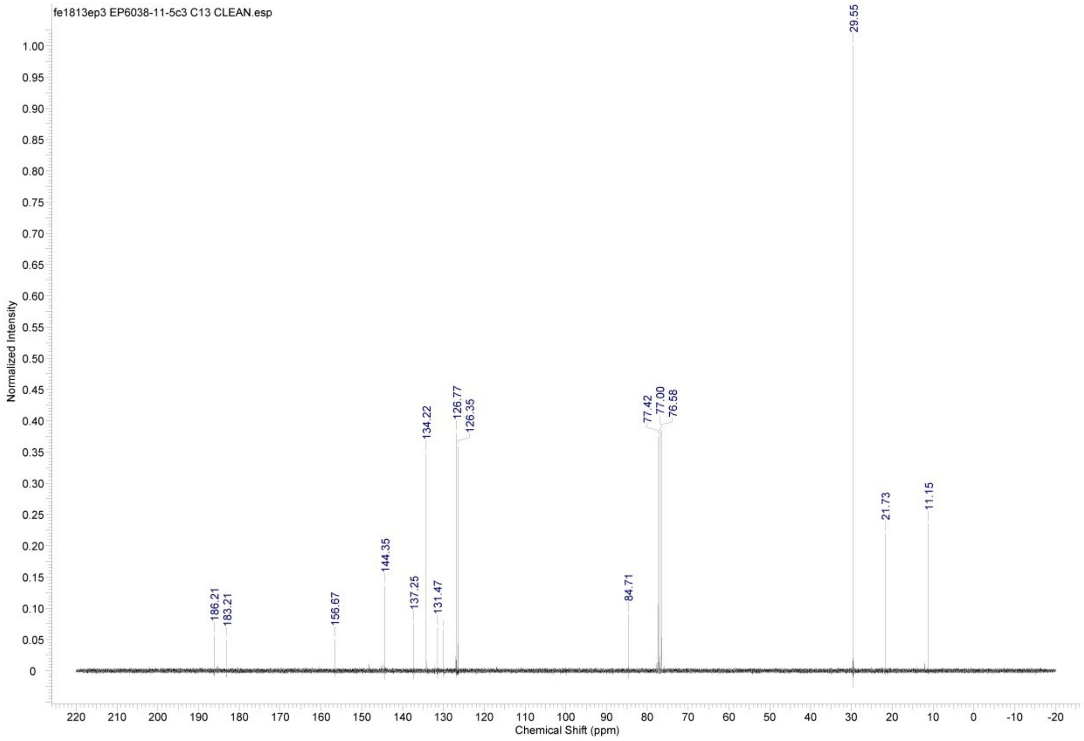

*3-(tert-Butoxy)-6-methoxy-2-methylnaphthalene-1,4-dione (**16c**)*

^{1}H NMR

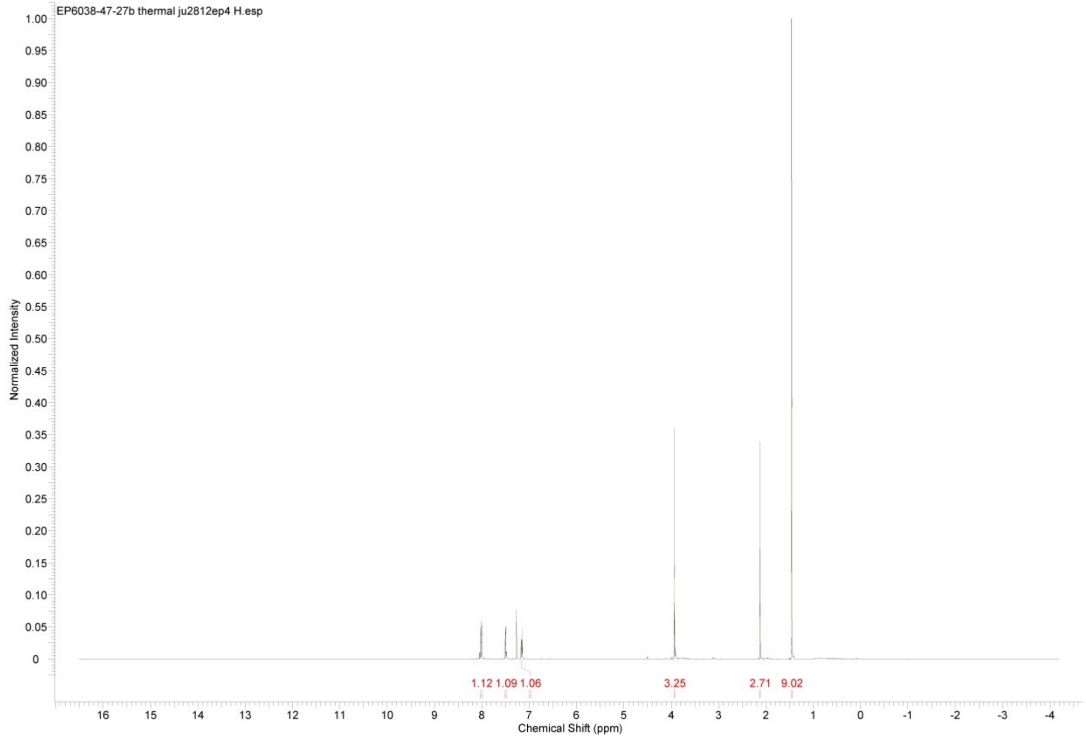

^{13}C NMR

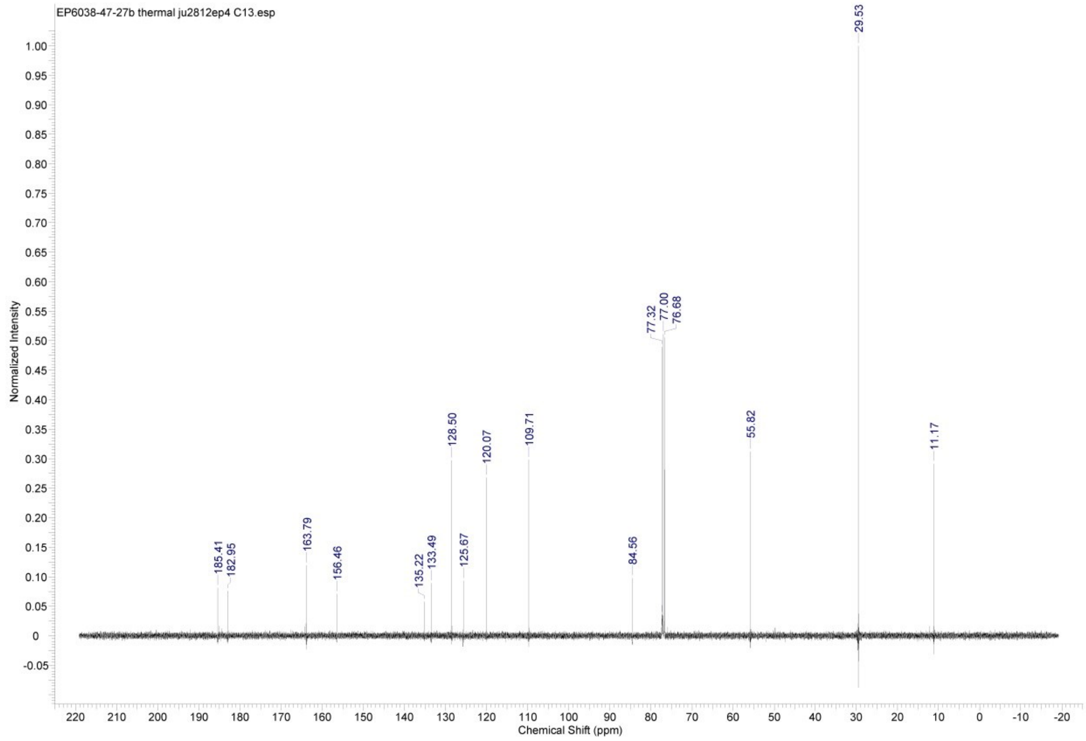

*2-(tert-Butoxy)-3-methyl-6-(trifluoromethyl)naphthalene-1,4-dione (**16d**)*

^{1}H NMR

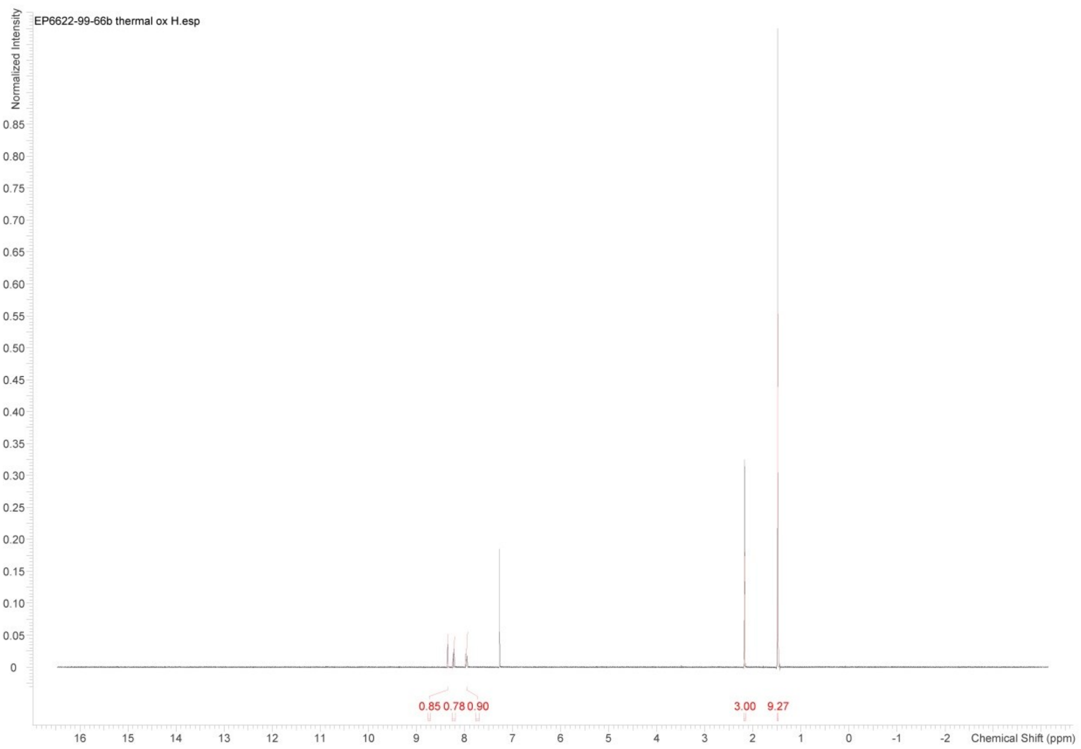

^{13}C NMR

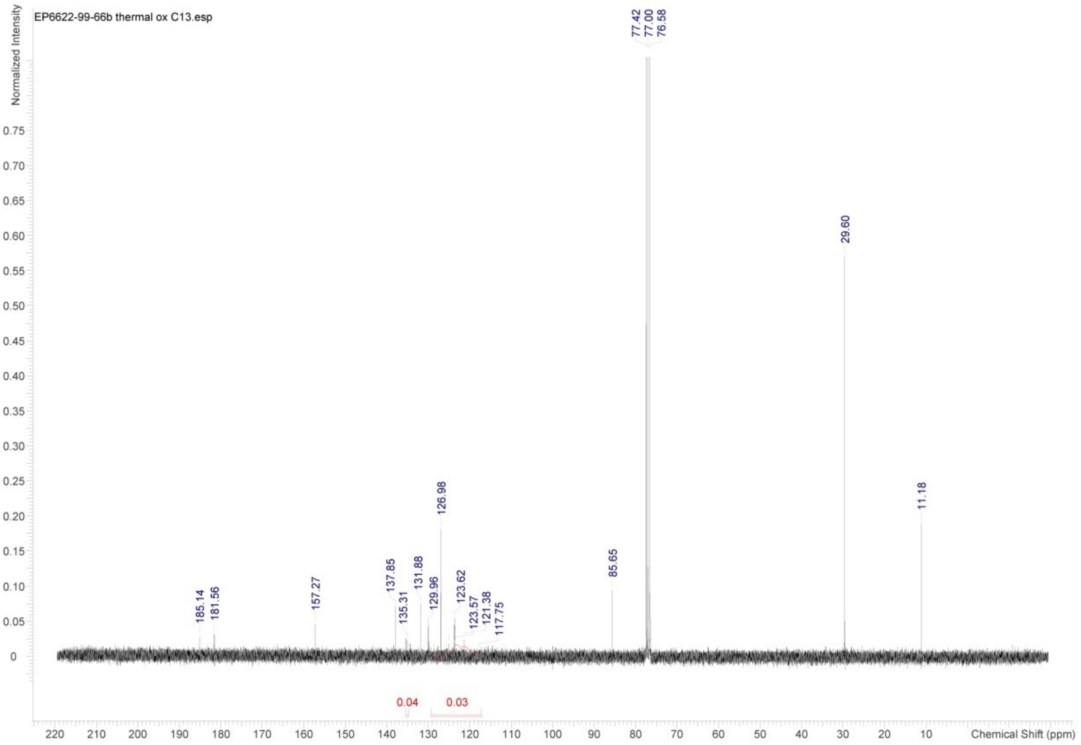

*2-(tert-Butoxy)-5-methoxy-3-methylnaphthalene-1,4-dione (**16e**)*

^{1}H NMR

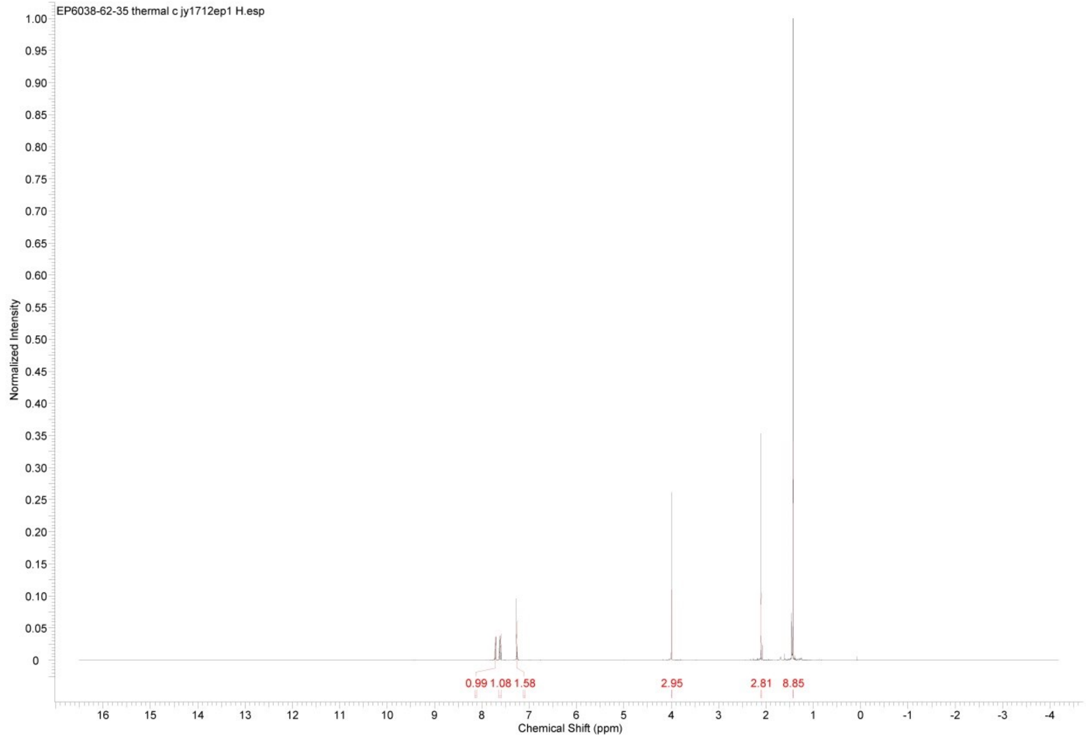

^{13}C NMR

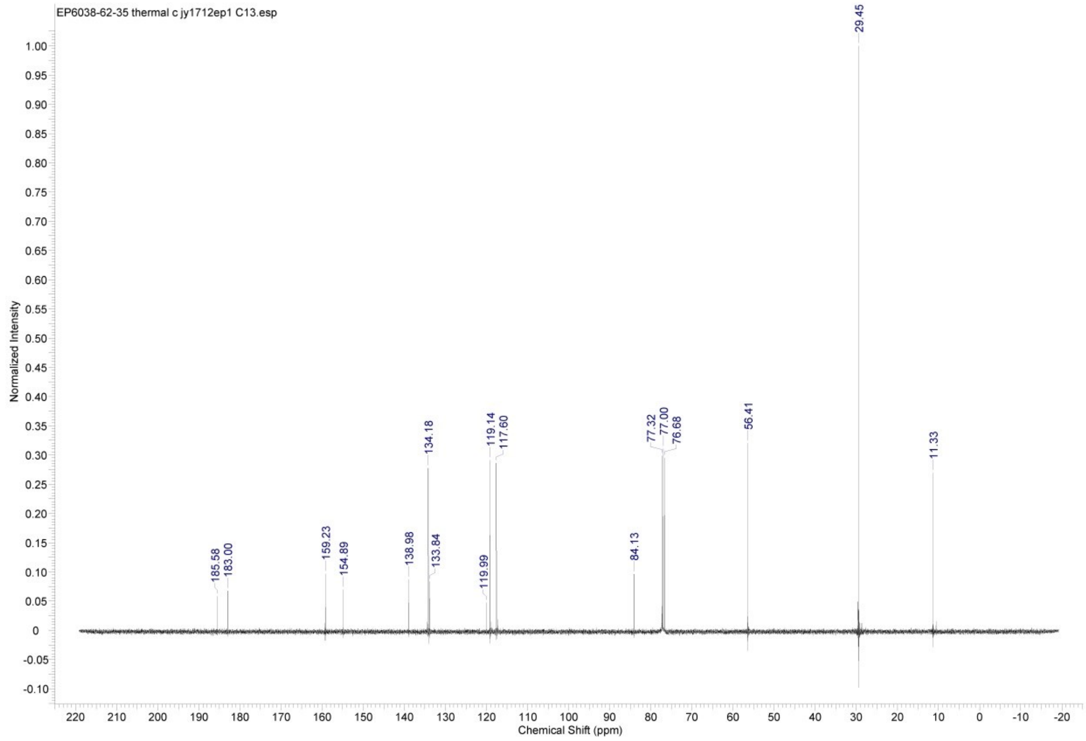

*3-(tert-Butoxy)-1-hydroxy-2-methyl-4H-quinolizin-4-one (**16f**)*

^{1}H NMR

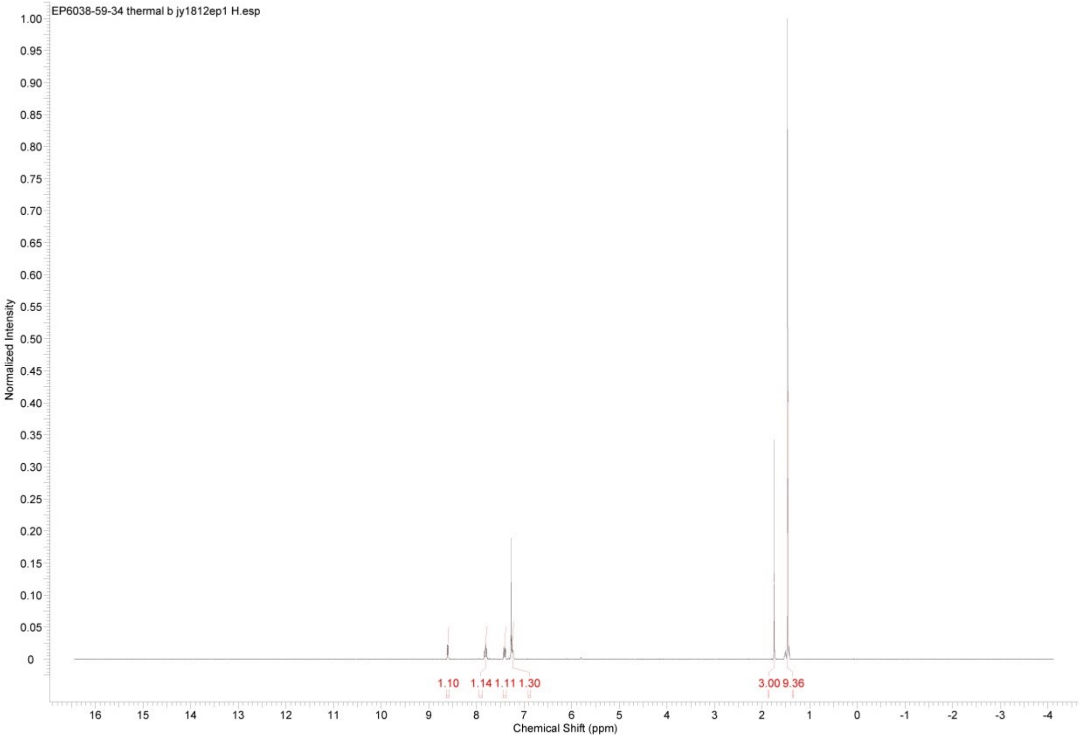

^{13}C NMR

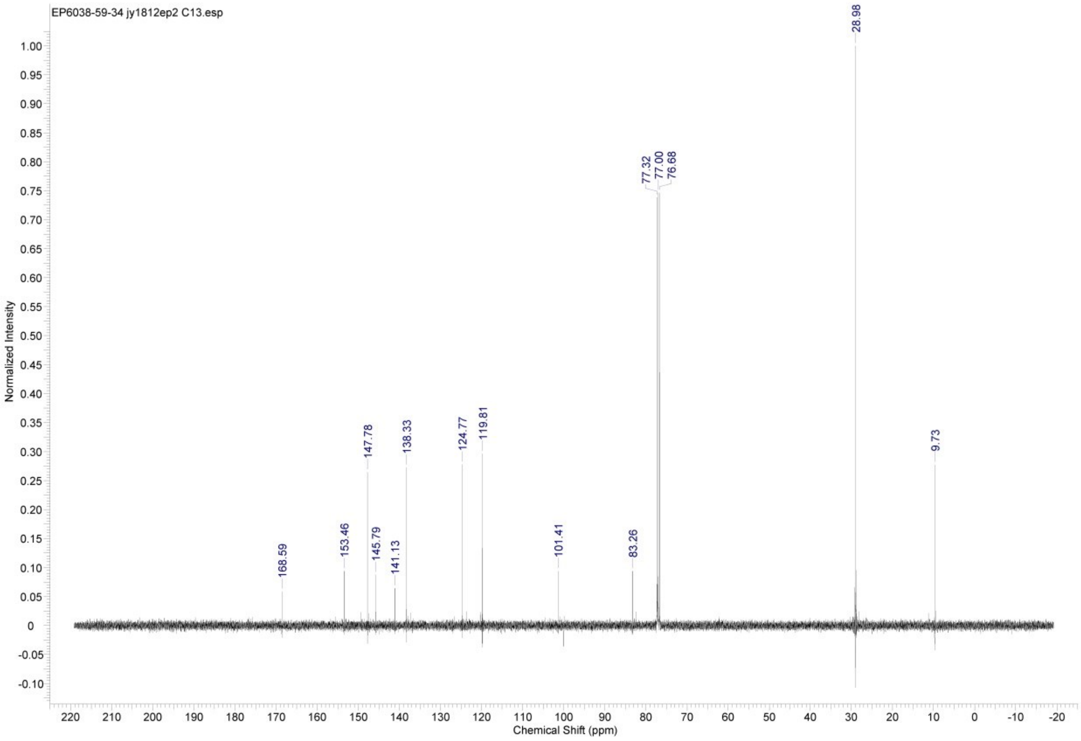

*2-(tert-Butoxy)-3,6-dimethylnaphthalene-1,4-dione (**16g**) and 2-(tert-butoxy)-3,8-dimethylnaphthalene-1,4-dione (**16i**)*

^{1}H NMR

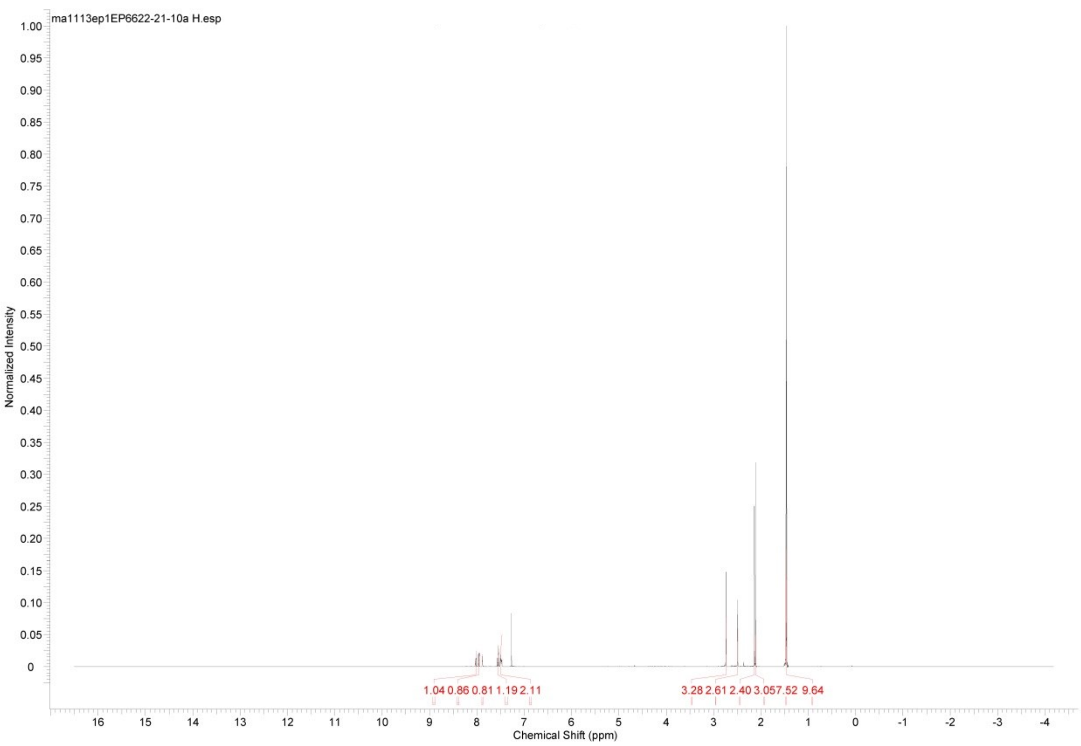

^{13}C NMR

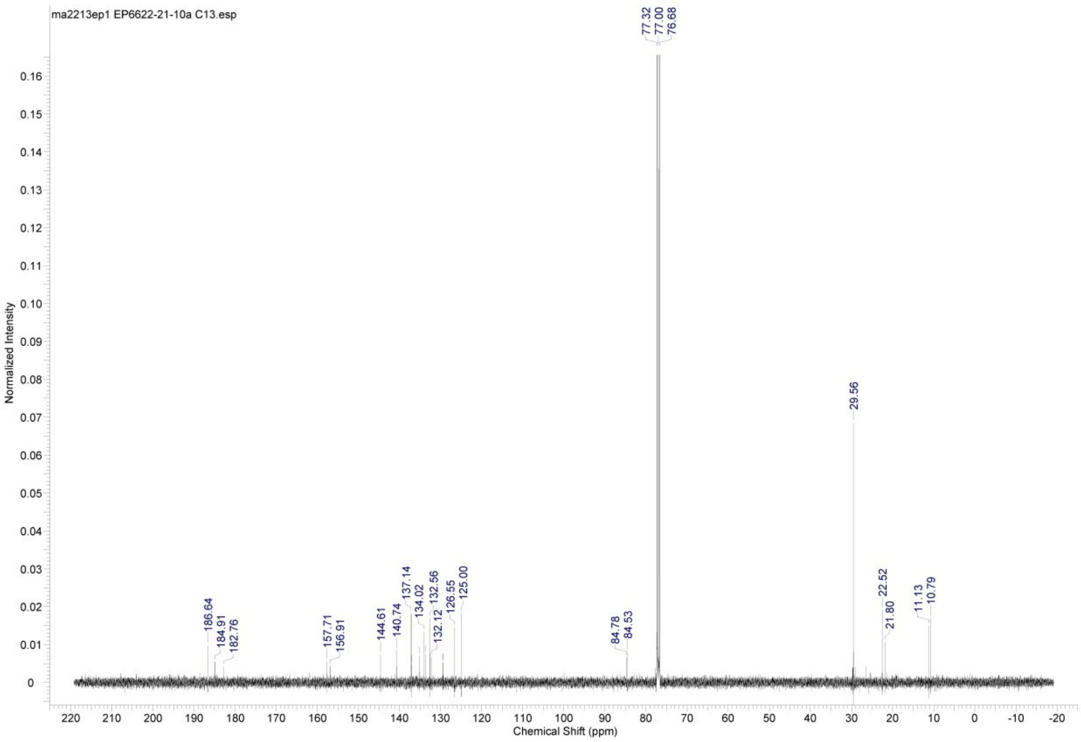

2-(tert-Butoxy)-3-methyl-6-(trifluoromethyl)naphthalene-1,4-dione (**16h**)

^{1}H NMR

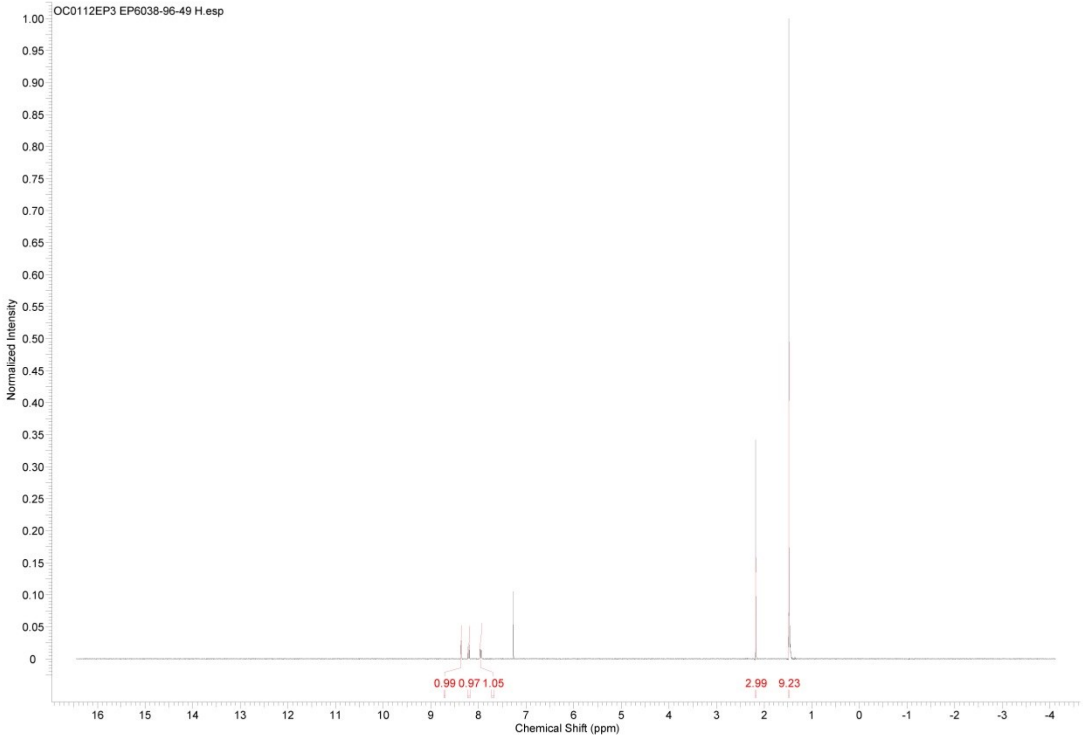

^{13}C NMR

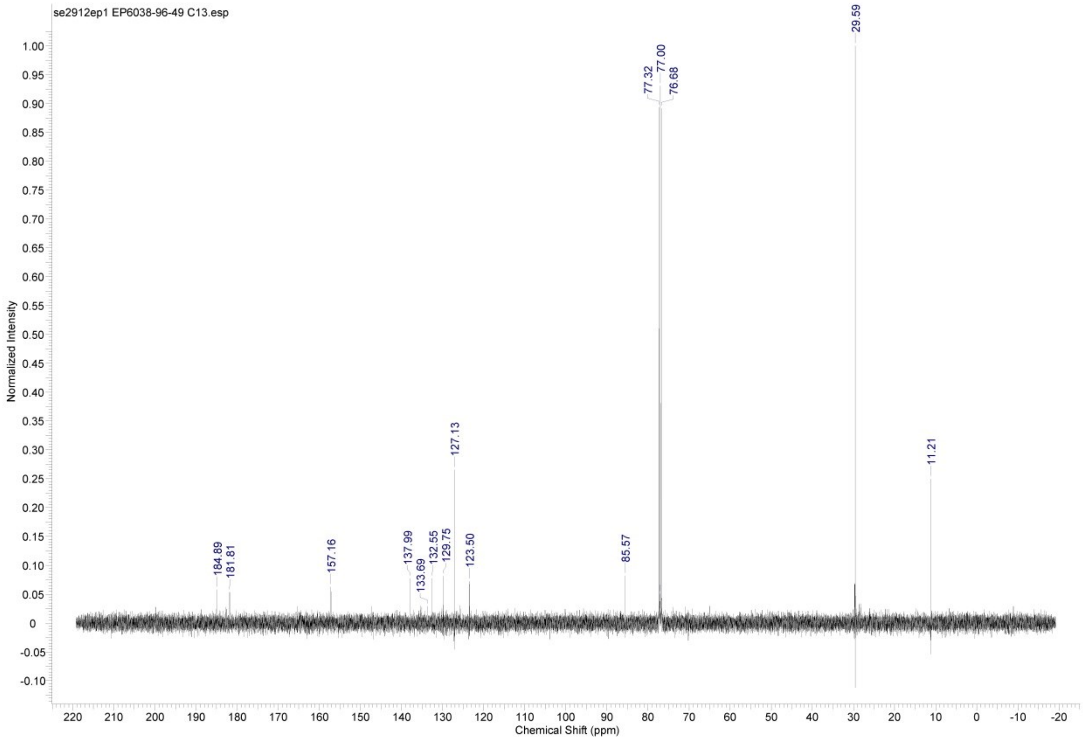

*2-(tert-Butoxy)-3,5-dimethylbenzene-1,4-diol (**23**) and 2-(tert-butoxy)-3,5-dimethylcyclohexa-2,5-diene-1,4-dione (**55**)*

^{1}H NMR

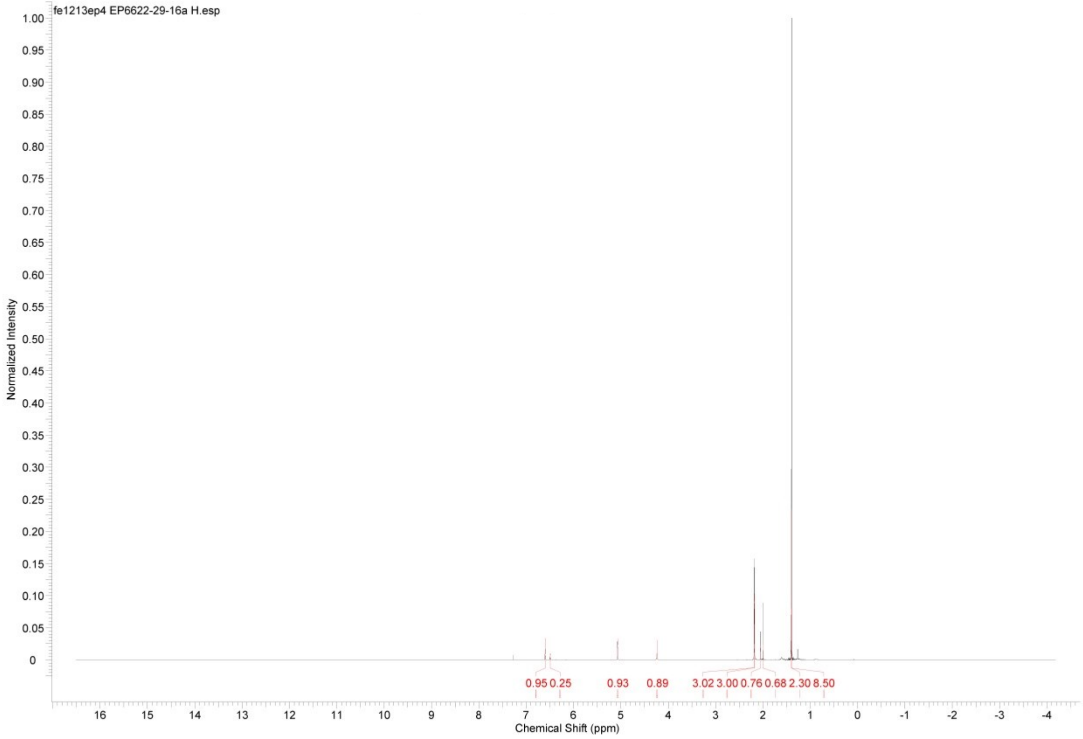

^{13}C NMR

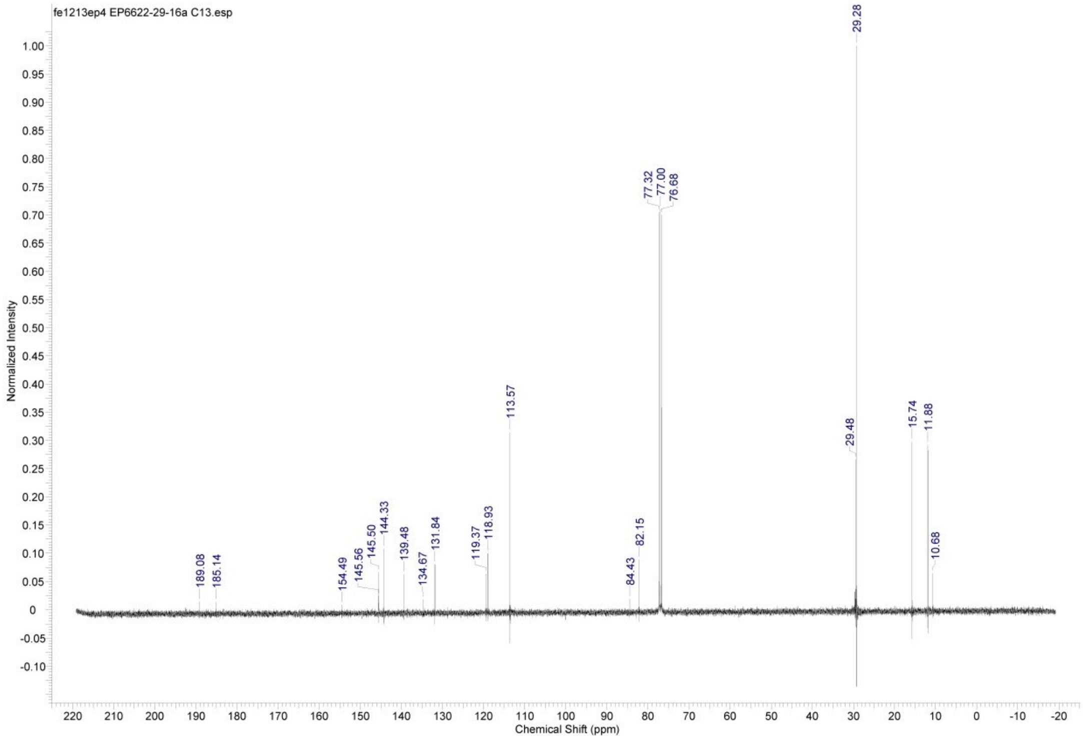

*4-(tert-Butoxy)-3-methyl-[1,1'-biphenyl]-2,5-diol (**24**)*

^{1}H NMR

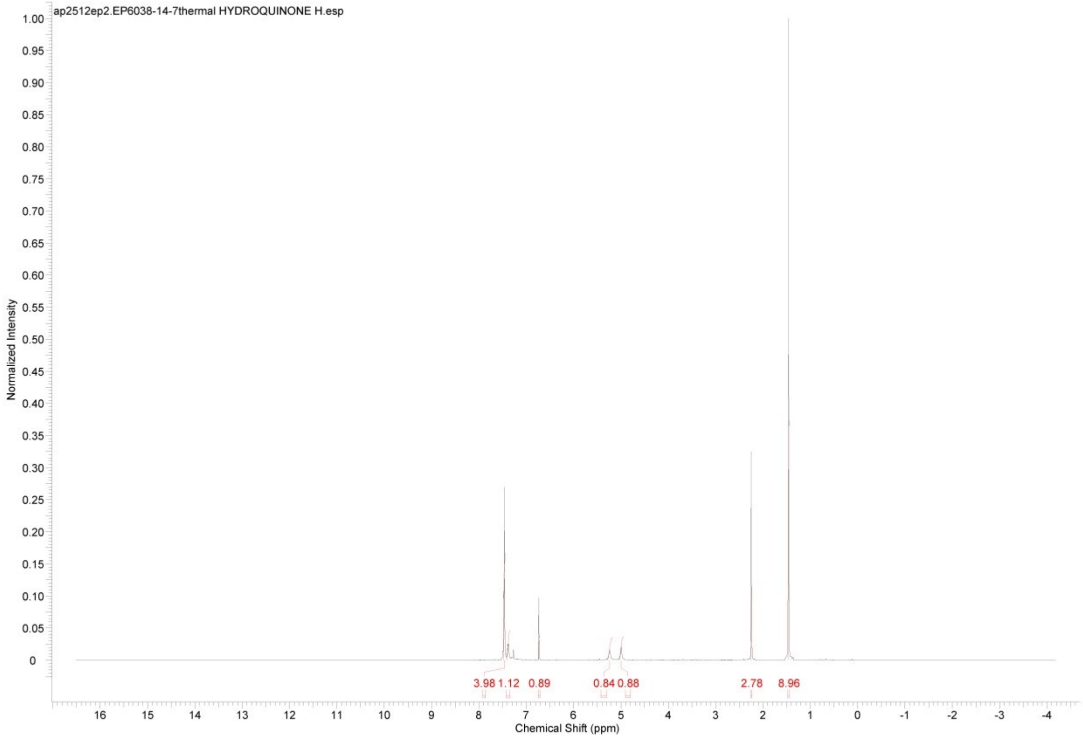

^{13}C NMR

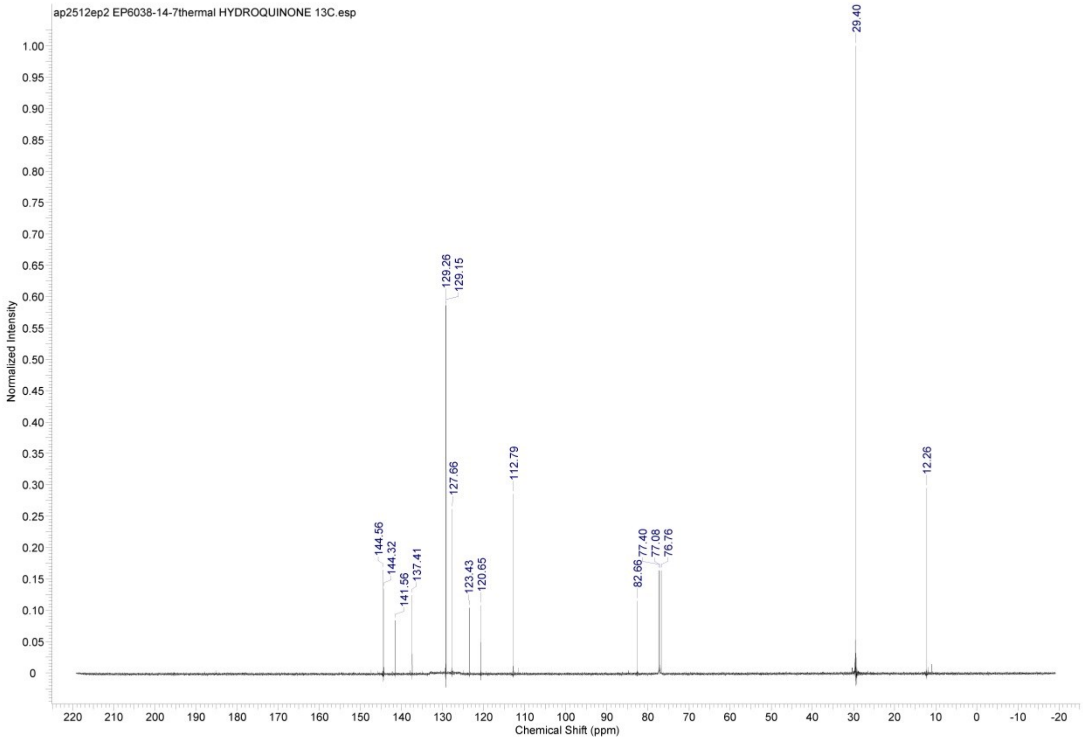

*2-(tert-Butoxy)-3-methylbenzene-1,4-diol (**25**)*

^{1}H NMR

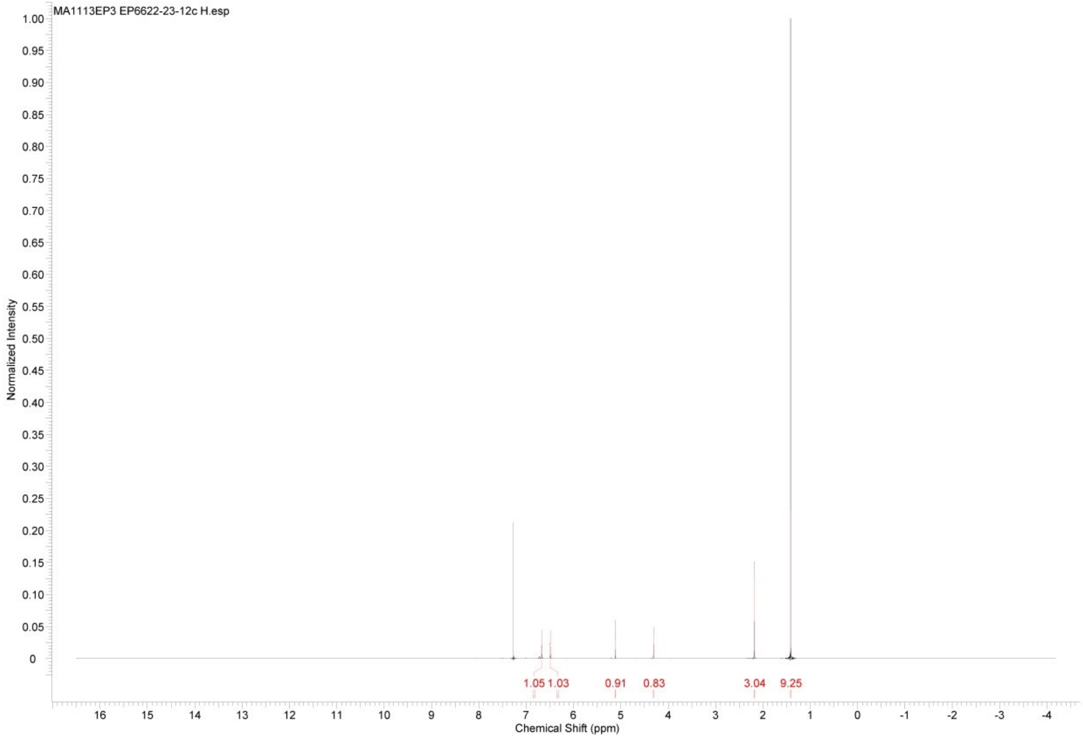

^{13}C NMR

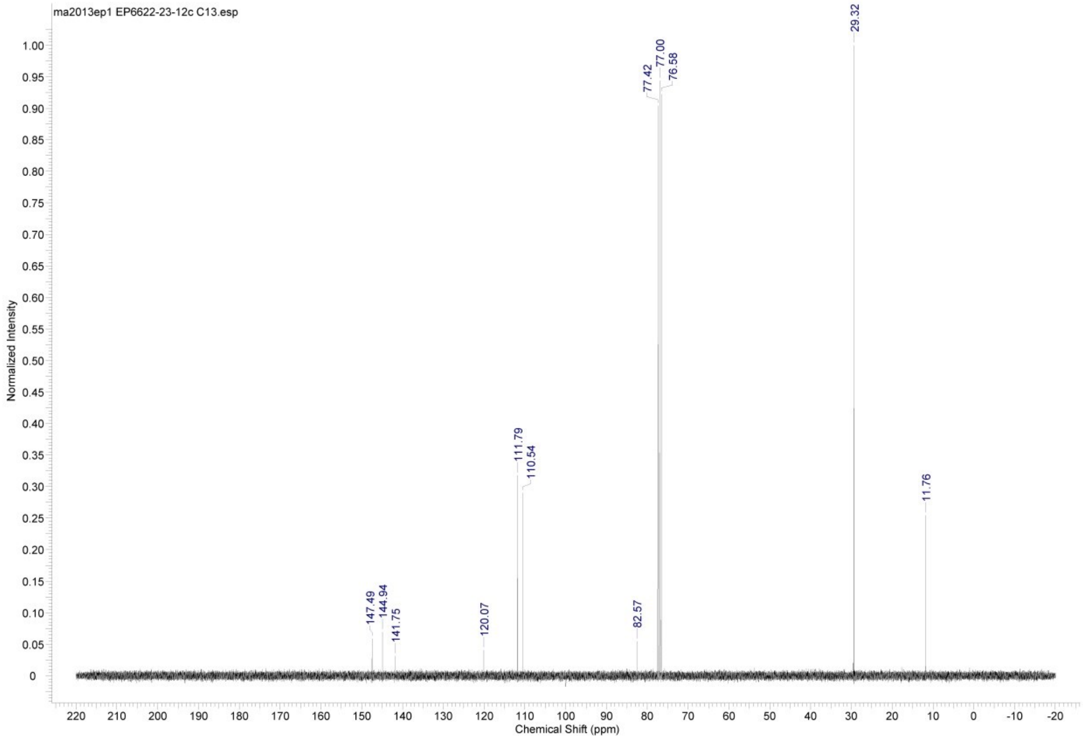

*4-Methoxy-3-methyl-[1,1'-biphenyl]-2,5-dione (**35**)*

^{1}H NMR

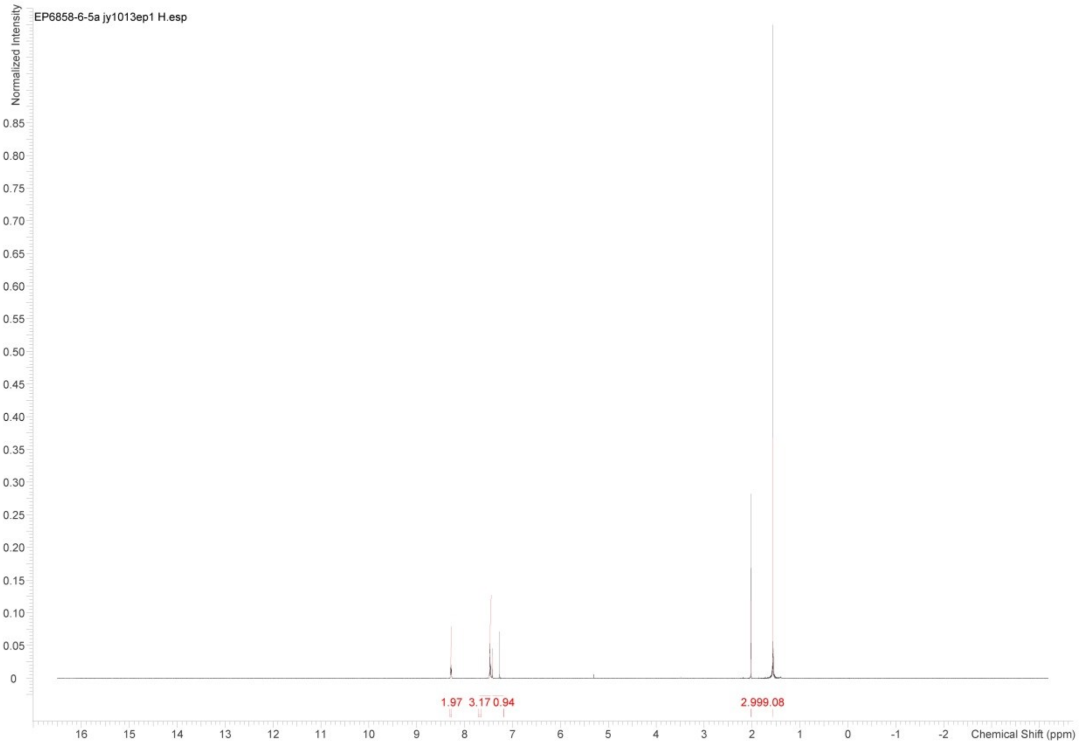

^{13}C NMR

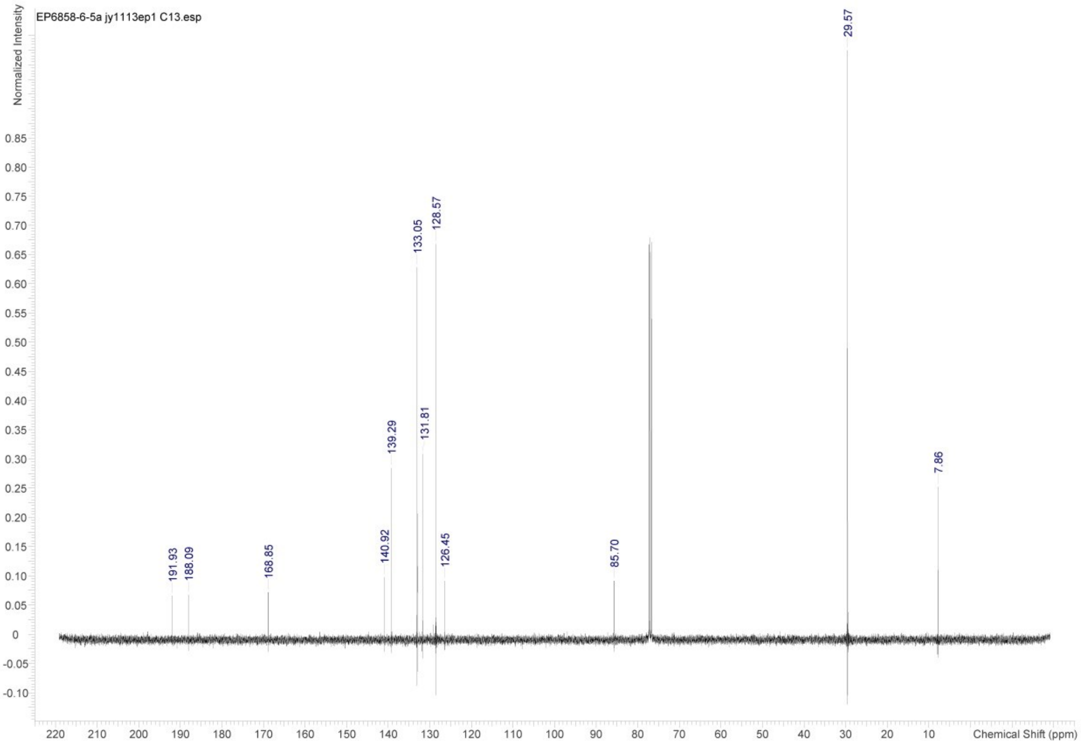

*(E)-2-benzylidene-4-methoxy-5-methylcyclopent-4-ene-1,3-dione (**36**)*

^{1}H NMR

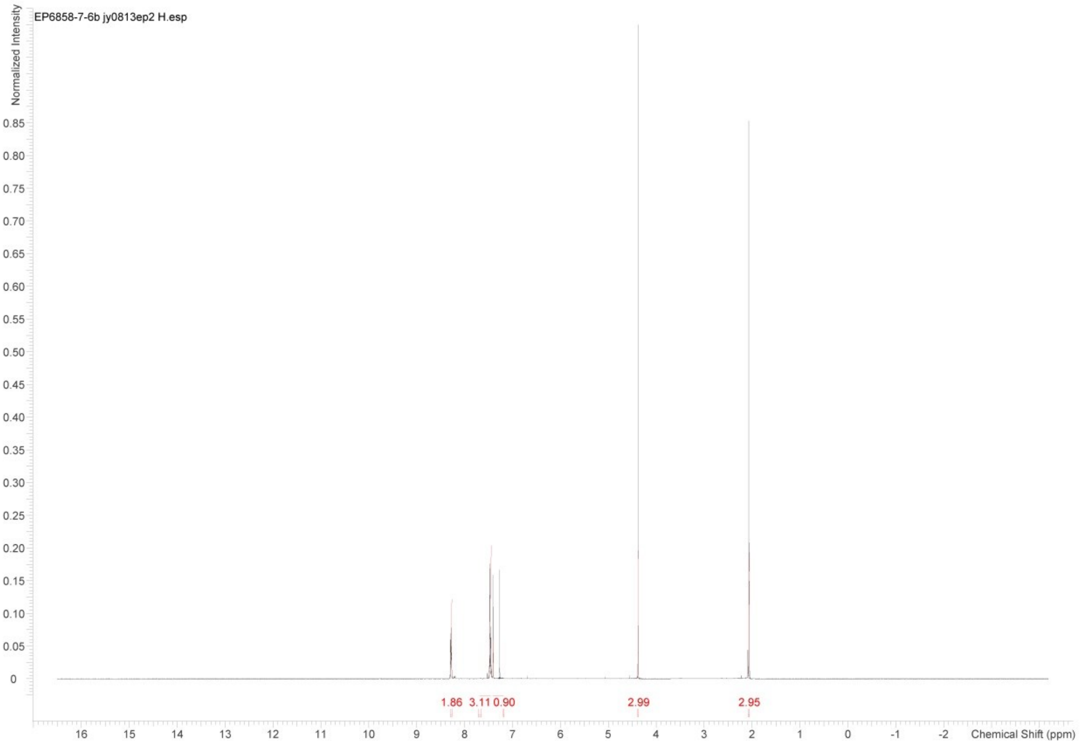

^{13}C NMR

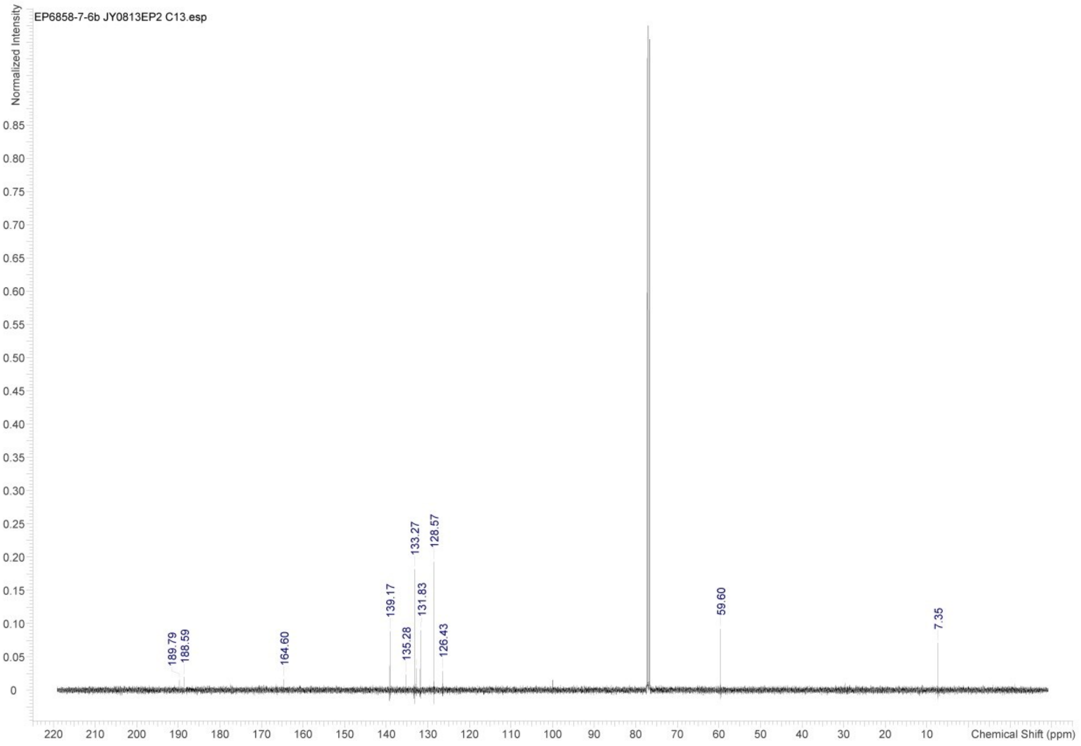

*(Z)-2-Benzylidene-4-(tert-butoxy)-5-methylcyclopent-4-ene-1,3-dione (**37**)*

^{1}H NMR

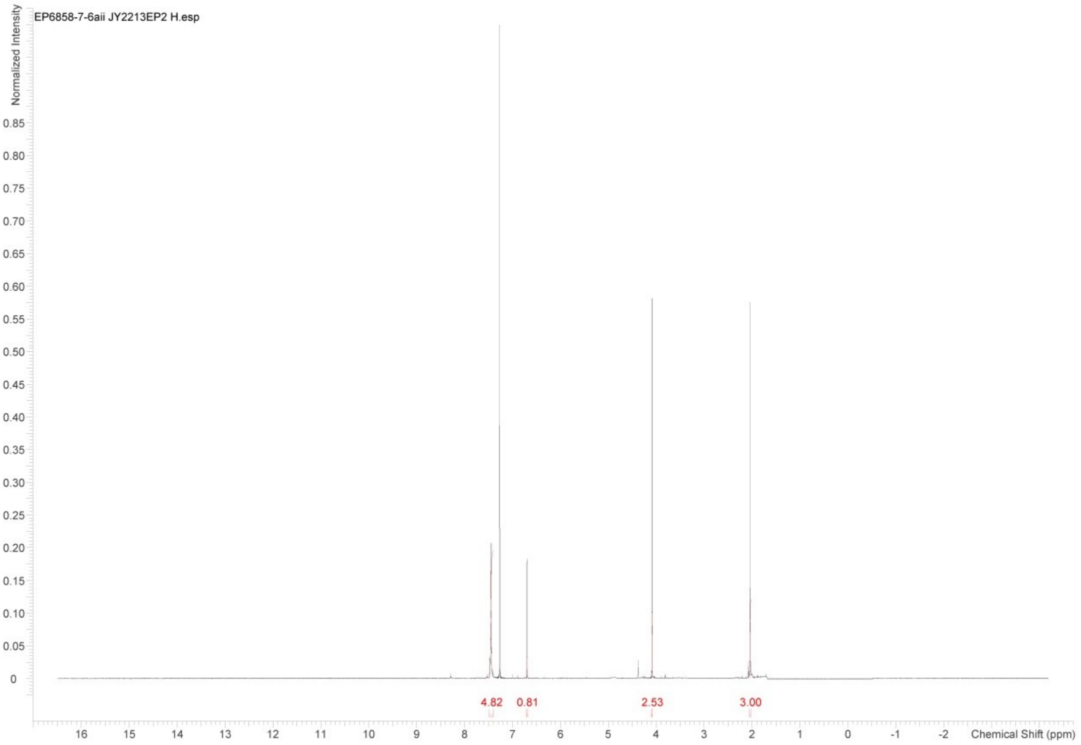

^{13}C NMR

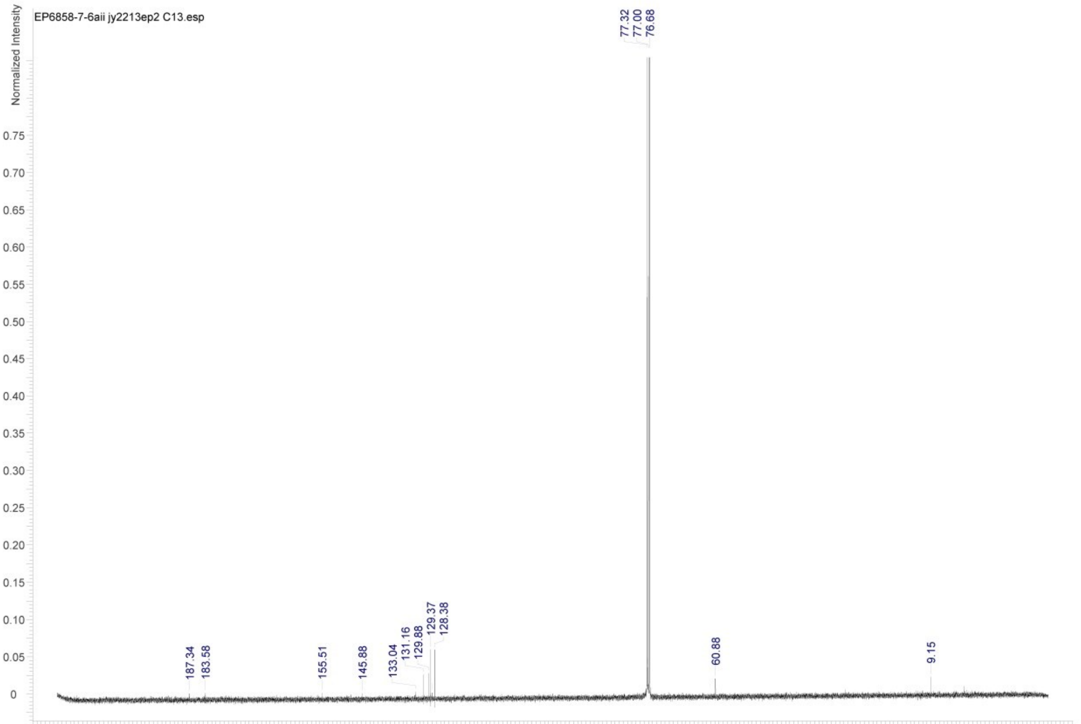

*(E)-2-Benzylidene-4-(tert-butoxy)-5-methylcyclopent-4-ene-1,3-dione (**53**) and 4-(tert-butoxy)-3-methyl-[1,1'-biphenyl]-2,5-dione (**54**)*

^{1}H NMR

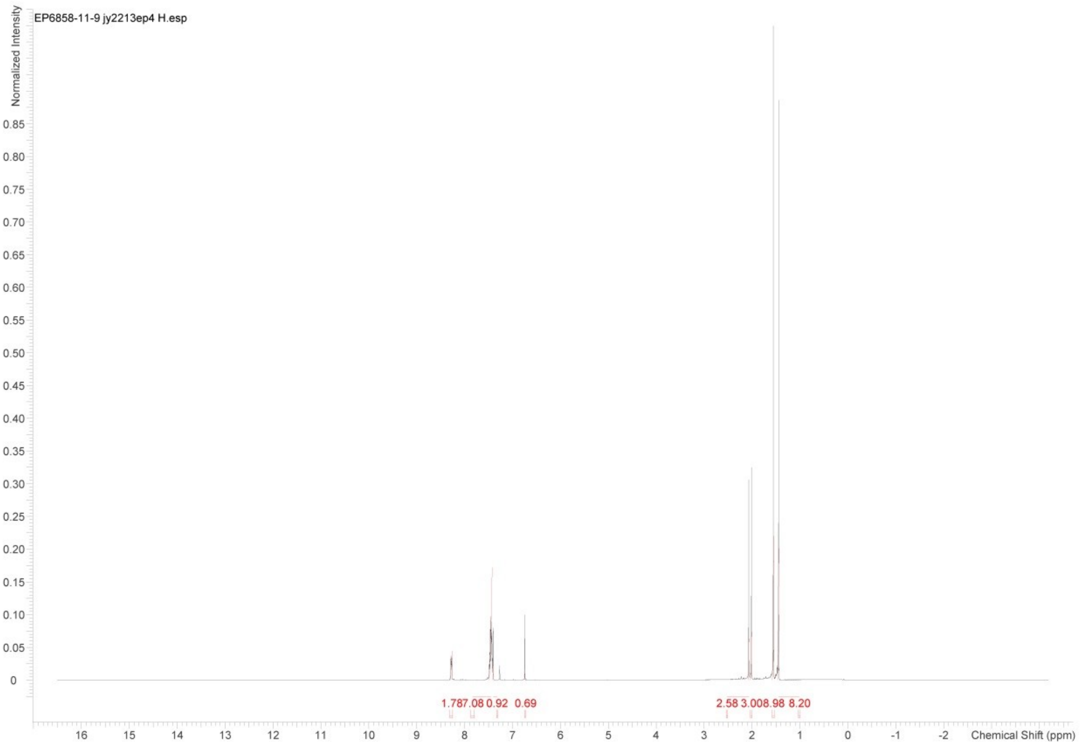

^{13}C NMR

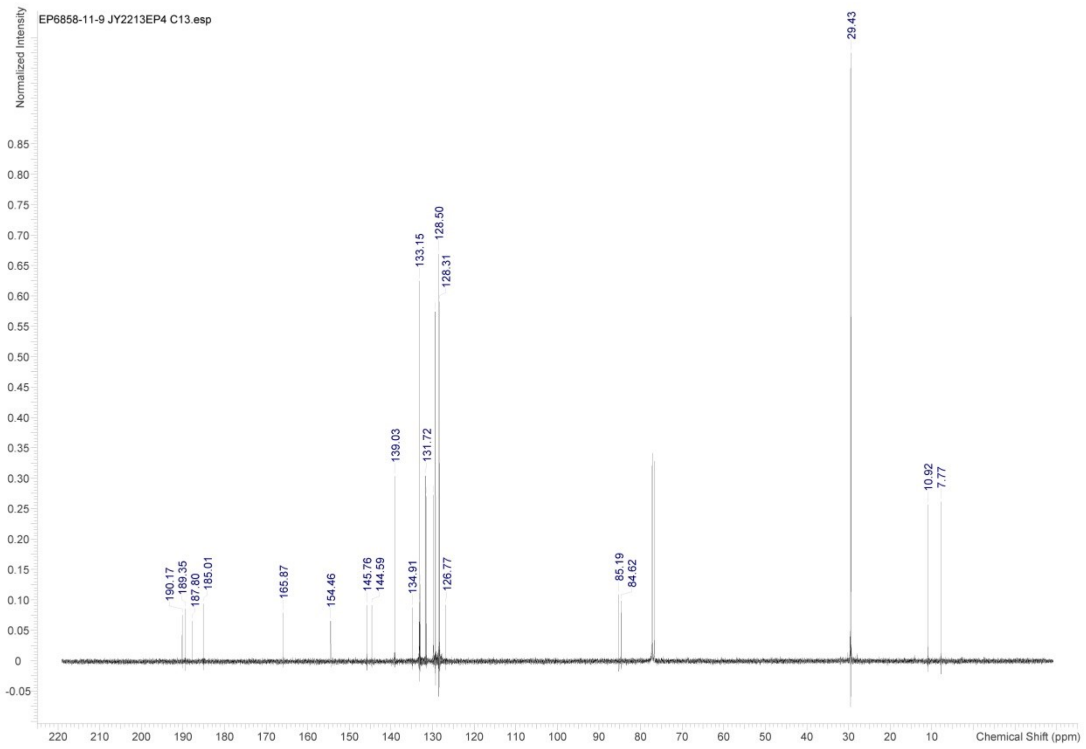

*3-methoxy-4-methyl-[1,1'-biphenyl]-2,5-dione (**52**)*

¹H NMR

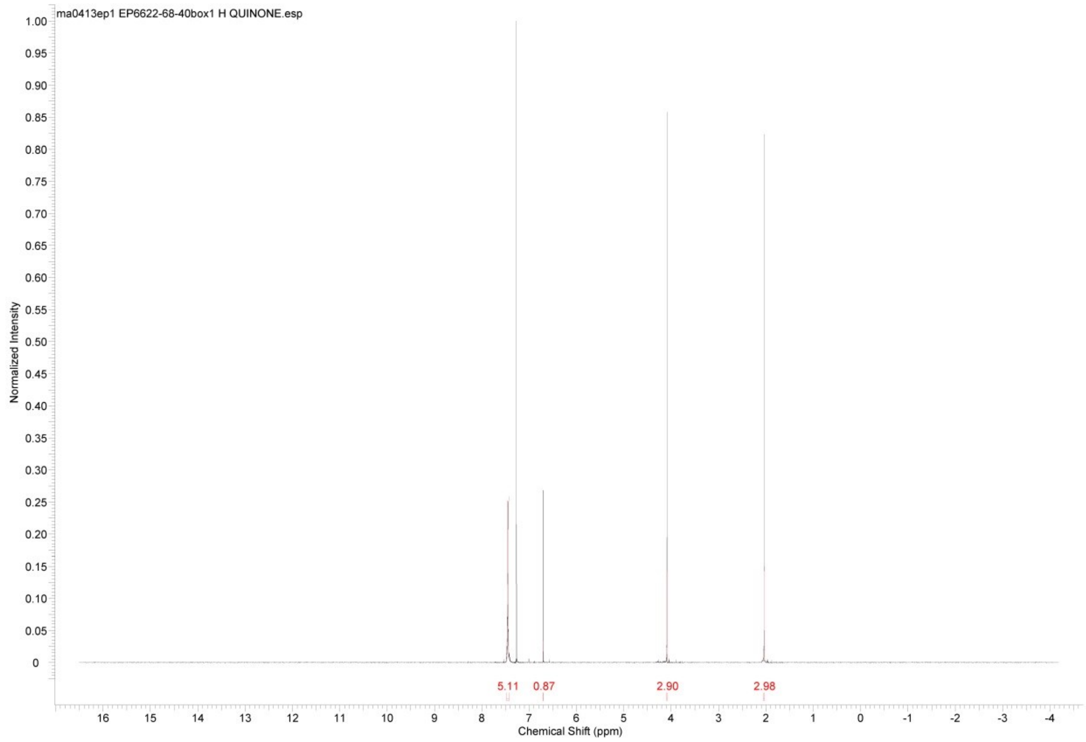

¹³C NMR

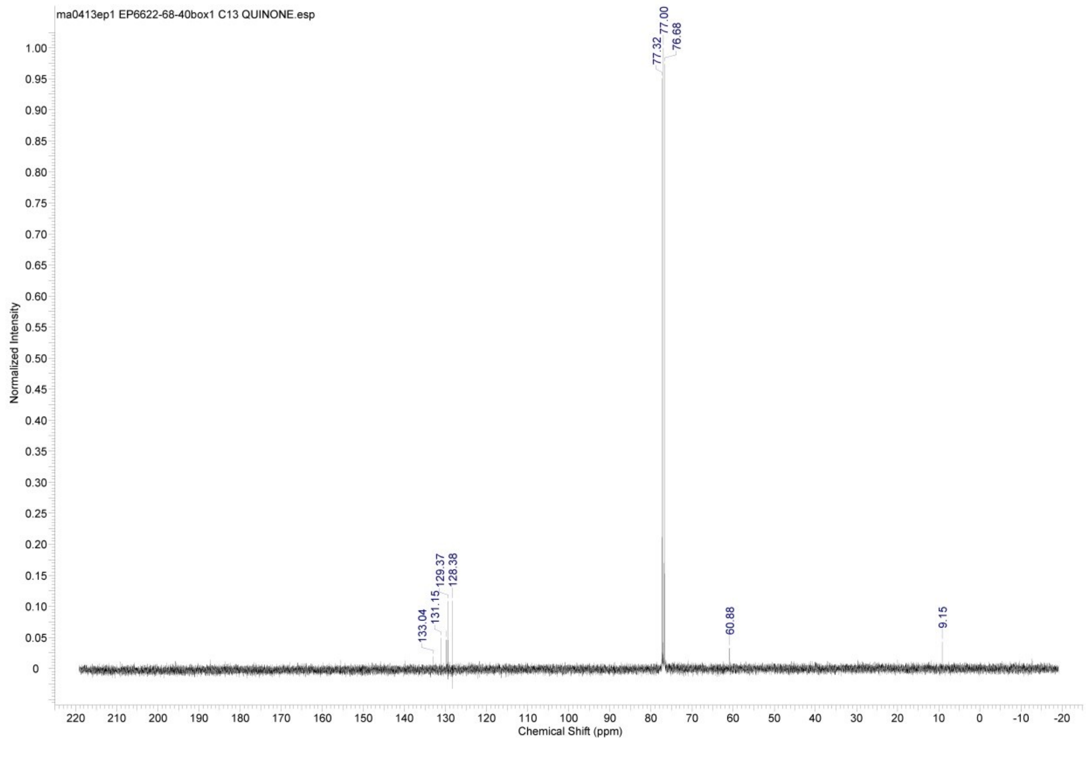

*3-Methoxy-4-methyl-[1,1'-biphenyl]-2,5-diol (**51**)*

^{1}H NMR

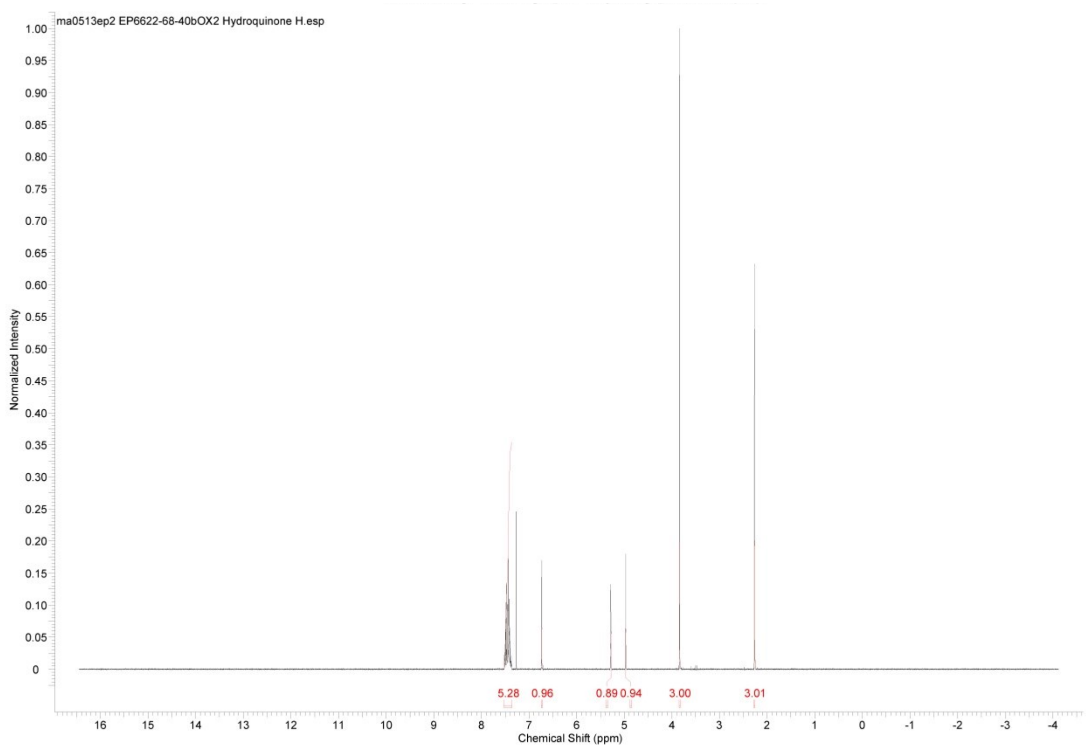

^{13}C NMR

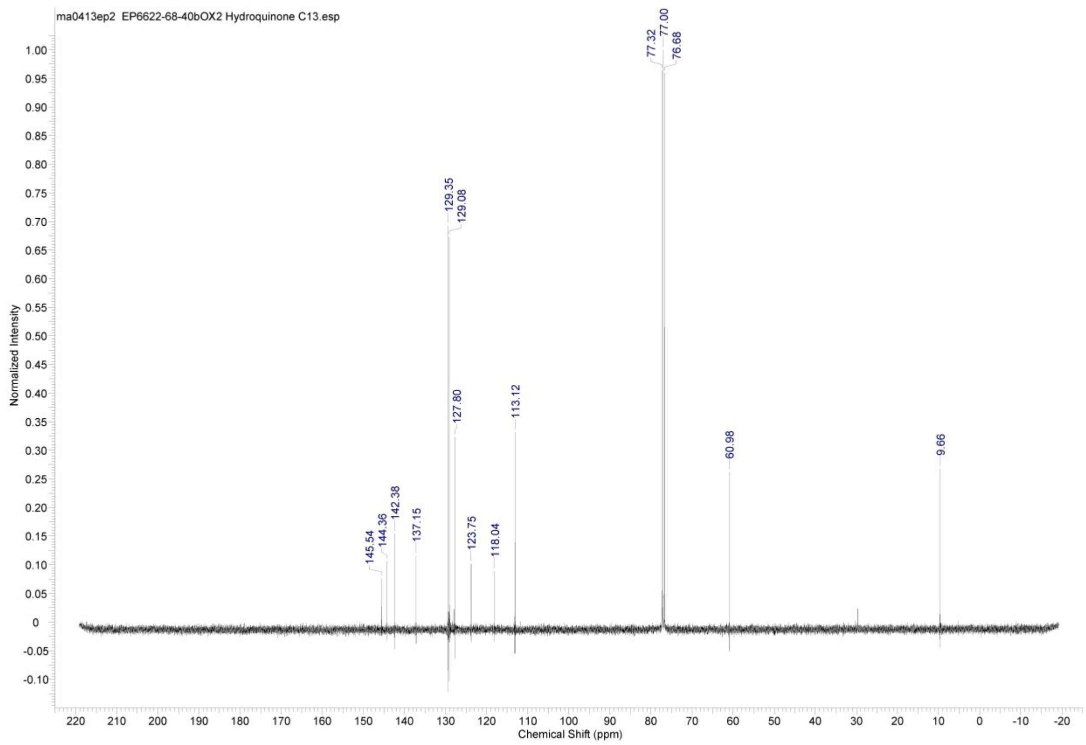

*3-(tert-Butoxy)-4,5-dimethylfuran-2(5H)-one (**29**)*

^{1}H NMR

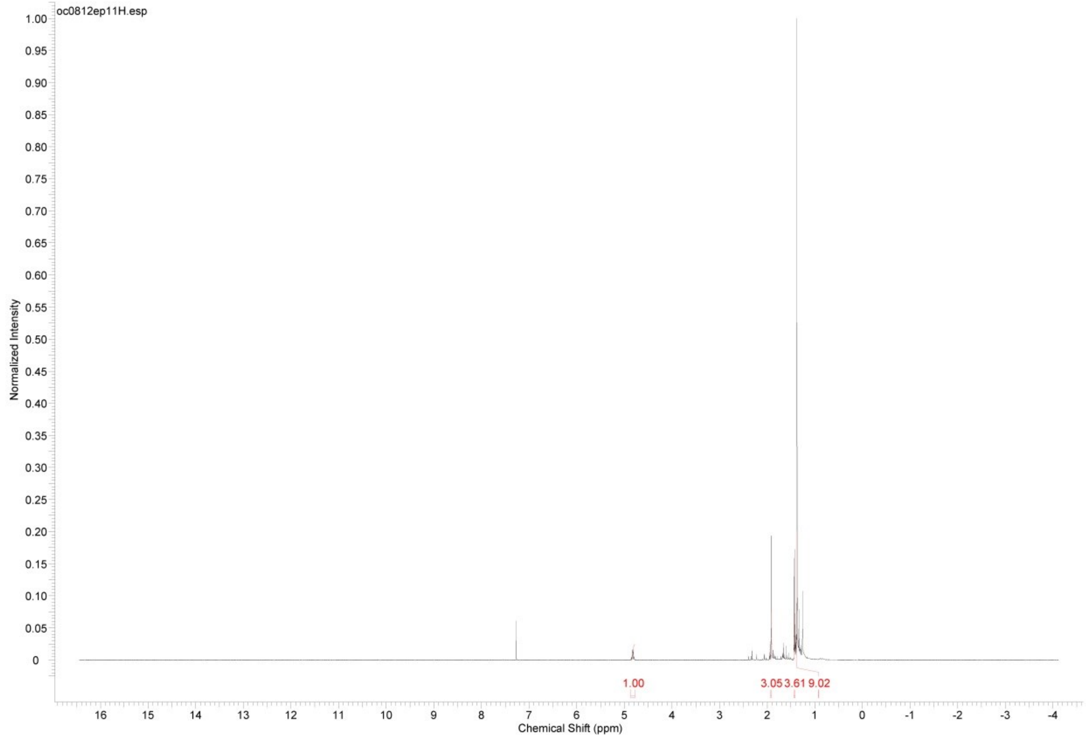

^{13}C NMR

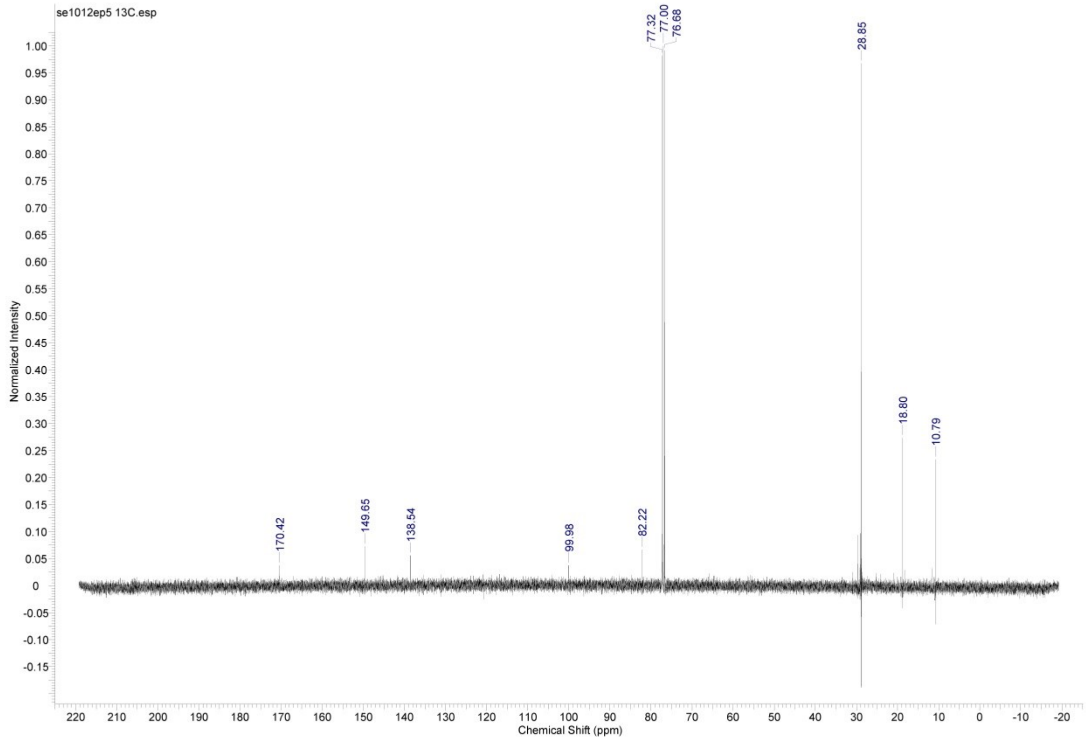

*3,4-Dimethylcyclobut-3-ene-1,2-dione (**30a**)*

^{1}H NMR

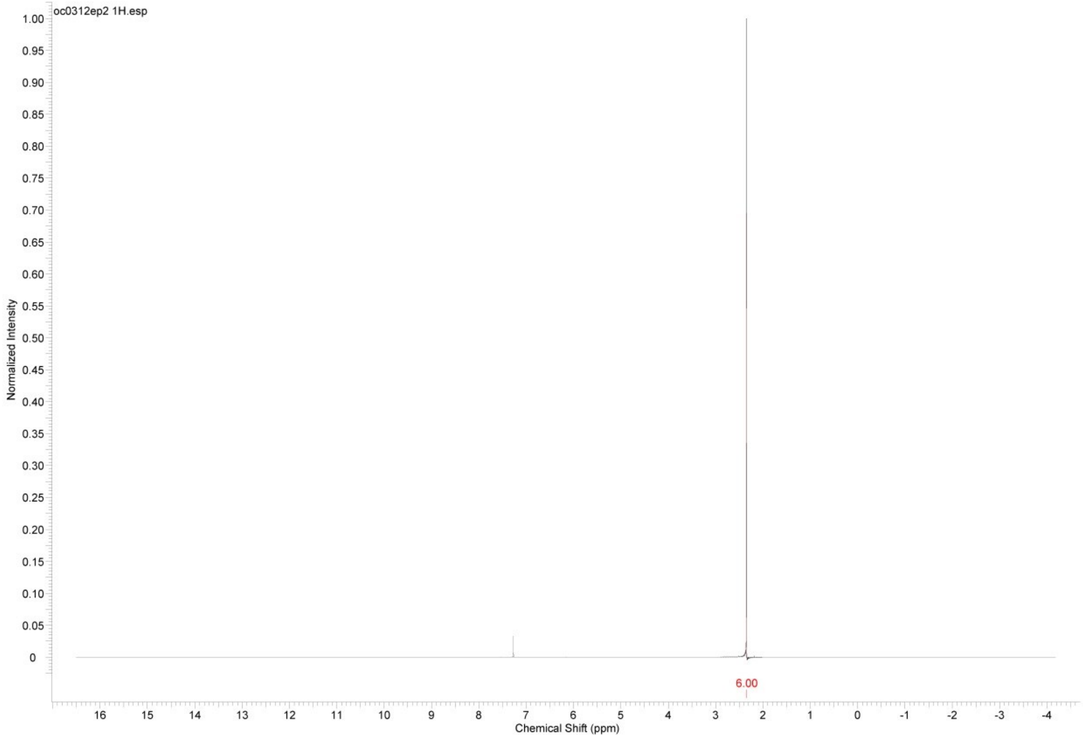

^{13}C NMR

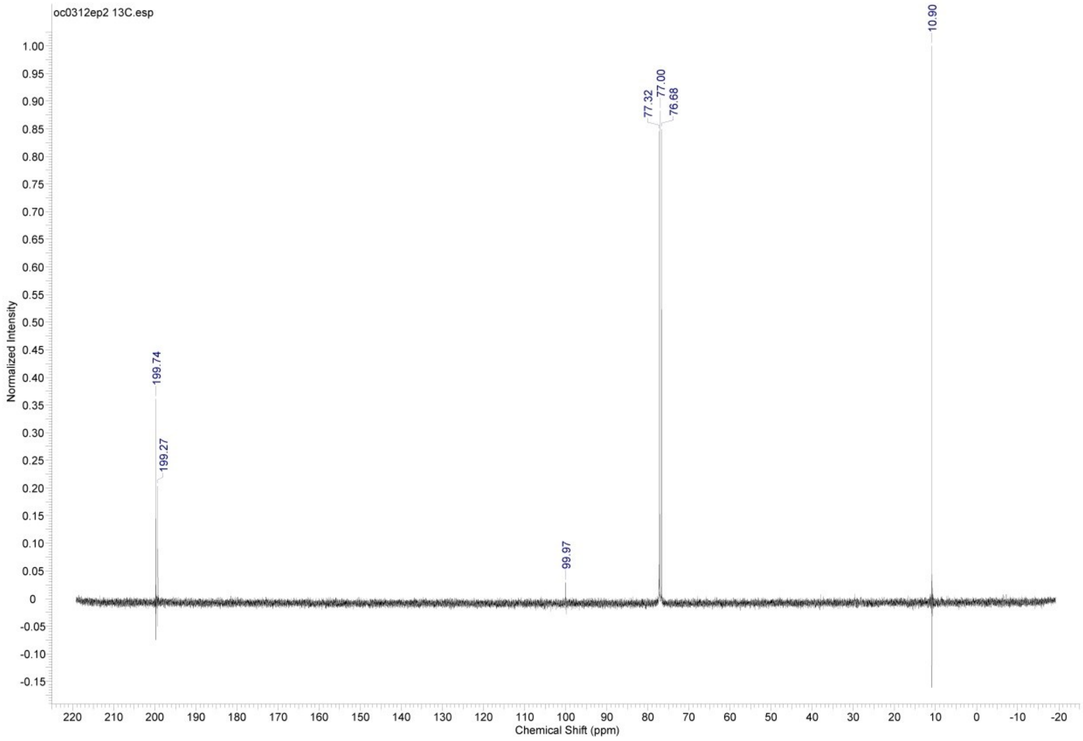

*3-Butyl-4-methylcyclobut-3-ene-1,2-dione (**30b**)*

^{1}H NMR

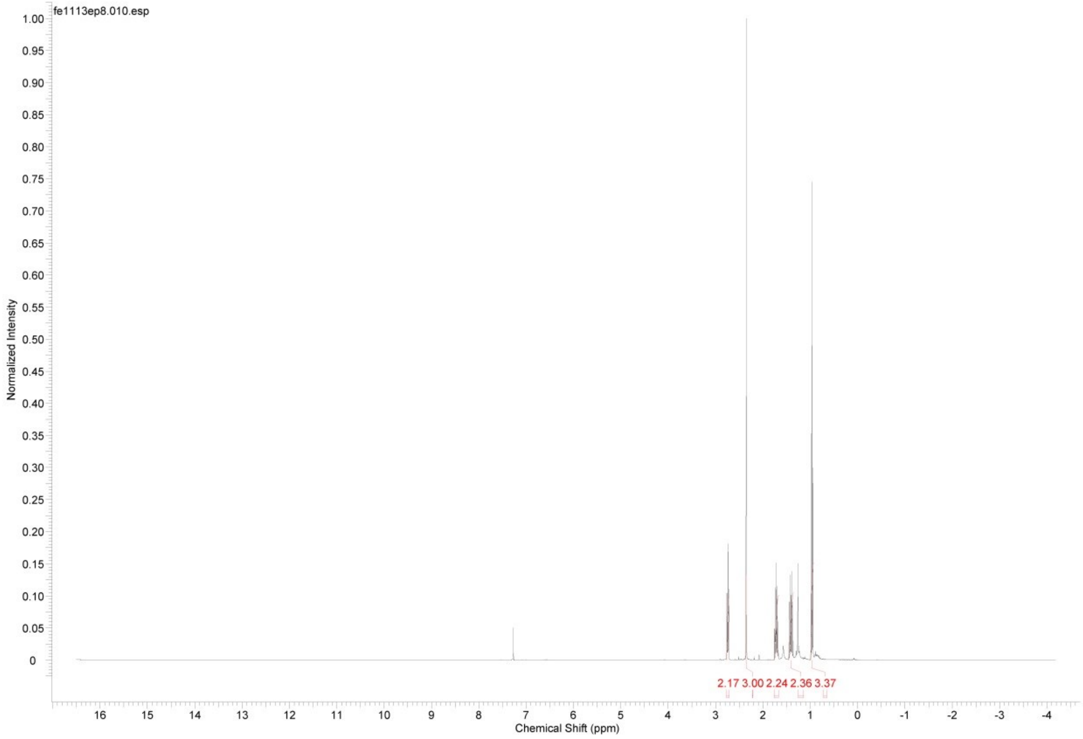

^{13}C NMR

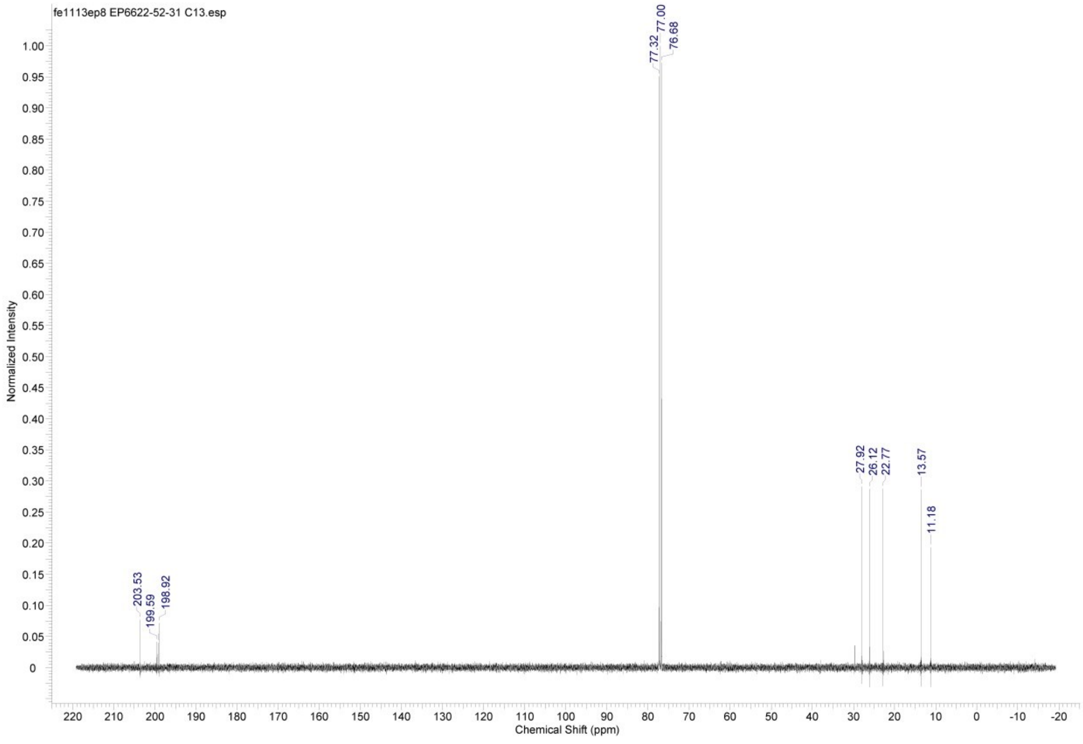

*3-Methyl-4-phenylcyclobut-3-ene-1,2-dione (**30c**)*

^{1}H NMR

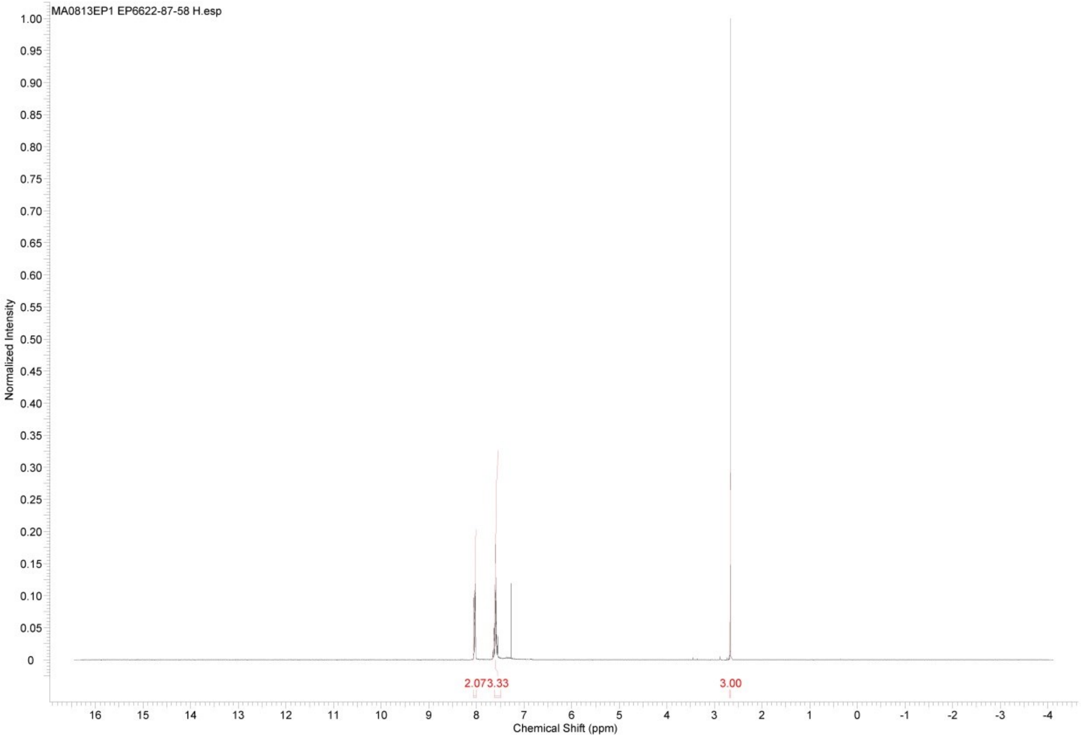

^{13}C NMR

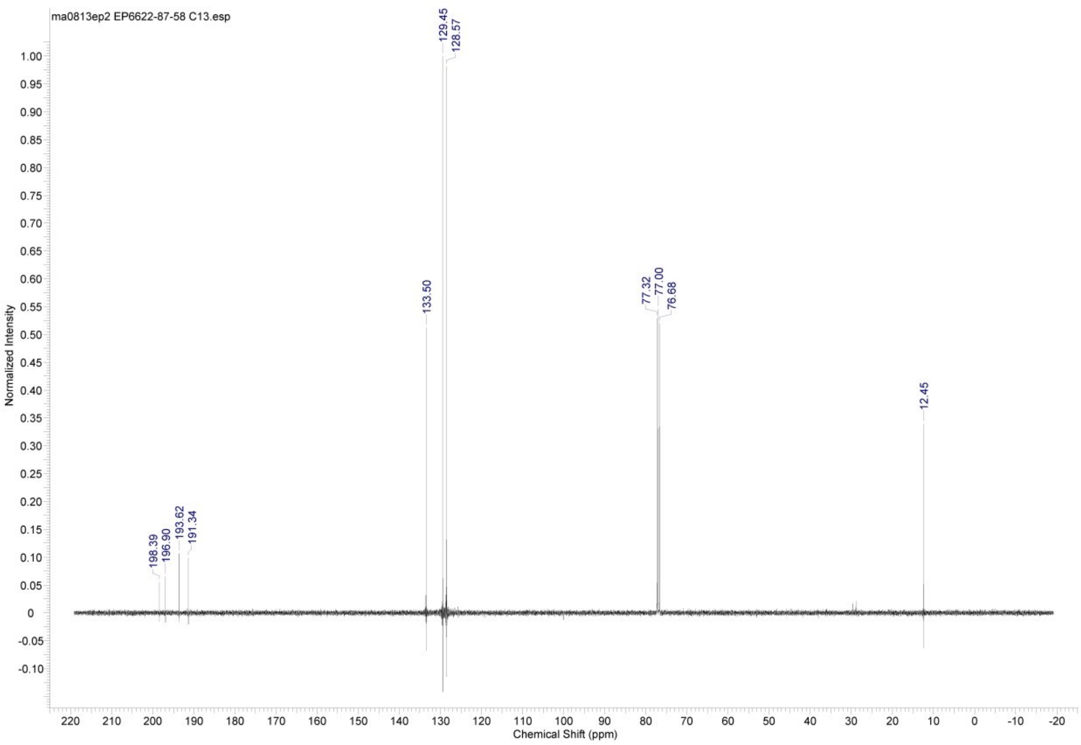

*3-Methyl-4-(2-methoxyphenyl)cyclobut-3-ene-1,2-dione (**30d**)*

^{1}H NMR

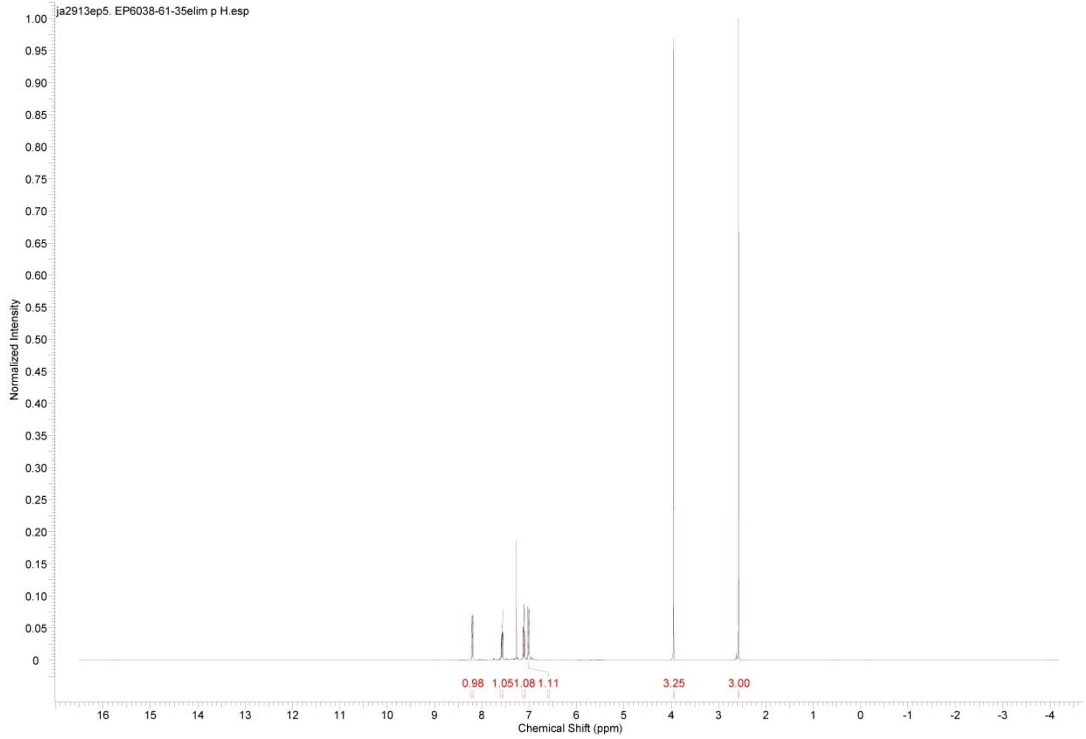

^{13}C NMR

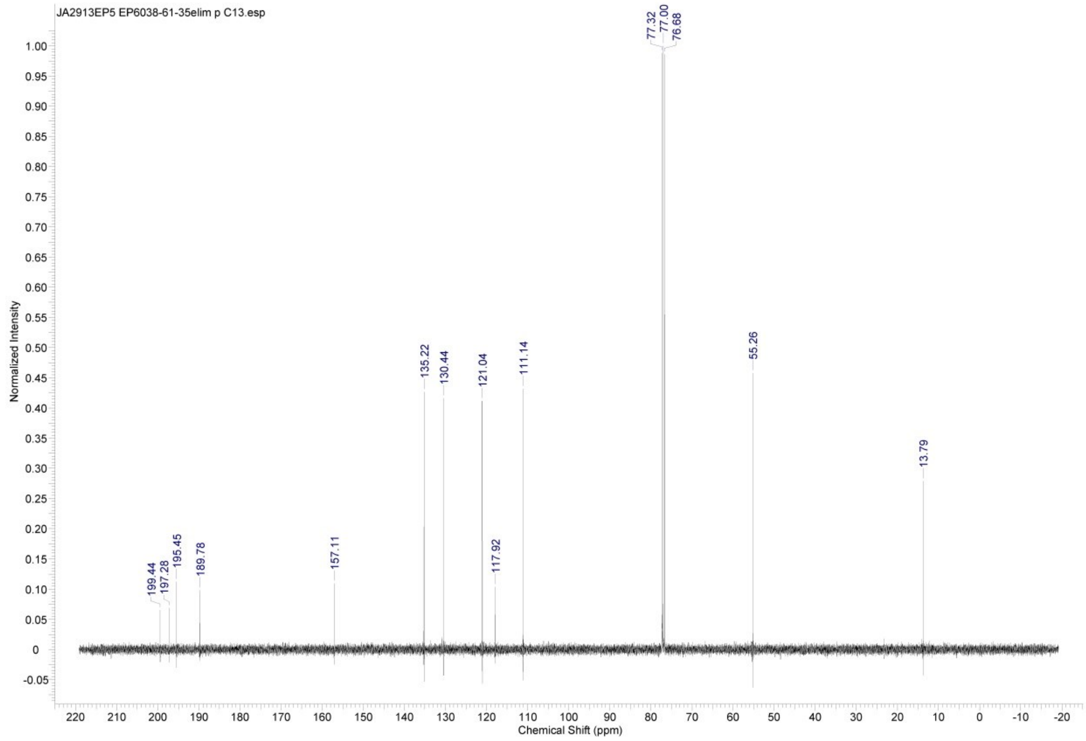

*(E)-2,4,6-Triisopropyl-N'-((2S,5R)-2-isopropyl-5-methylcyclohexylidene)benzenesulfonohydrazone (**40**)*

^{1}H NMR

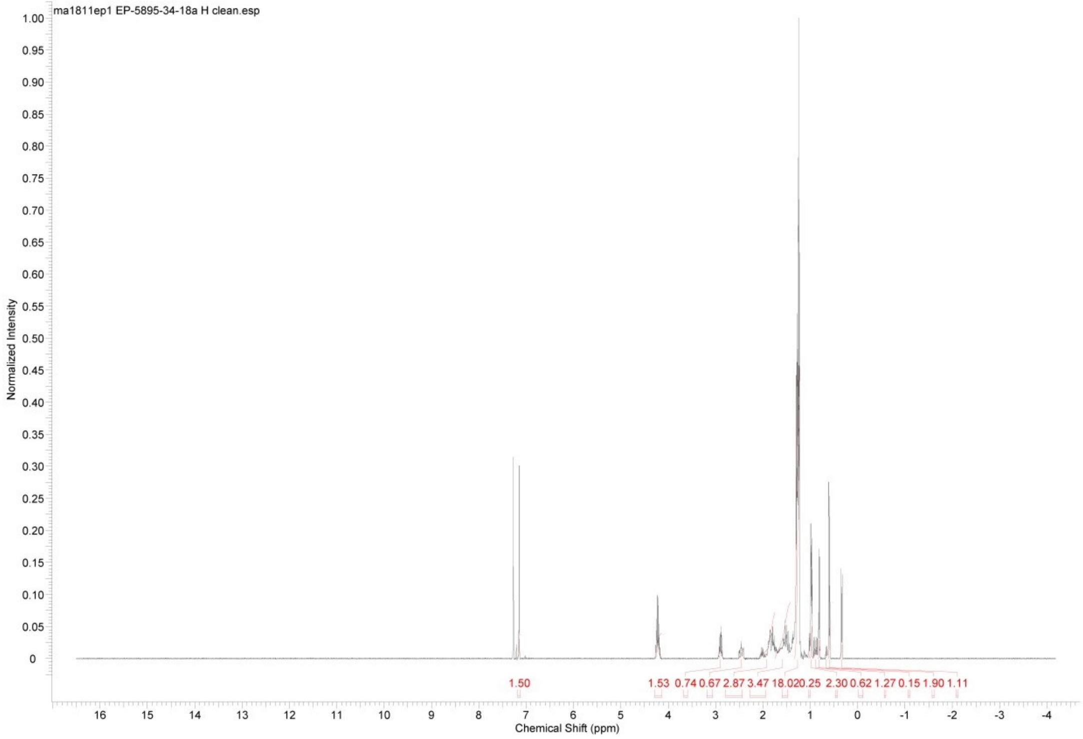

^{13}C NMR

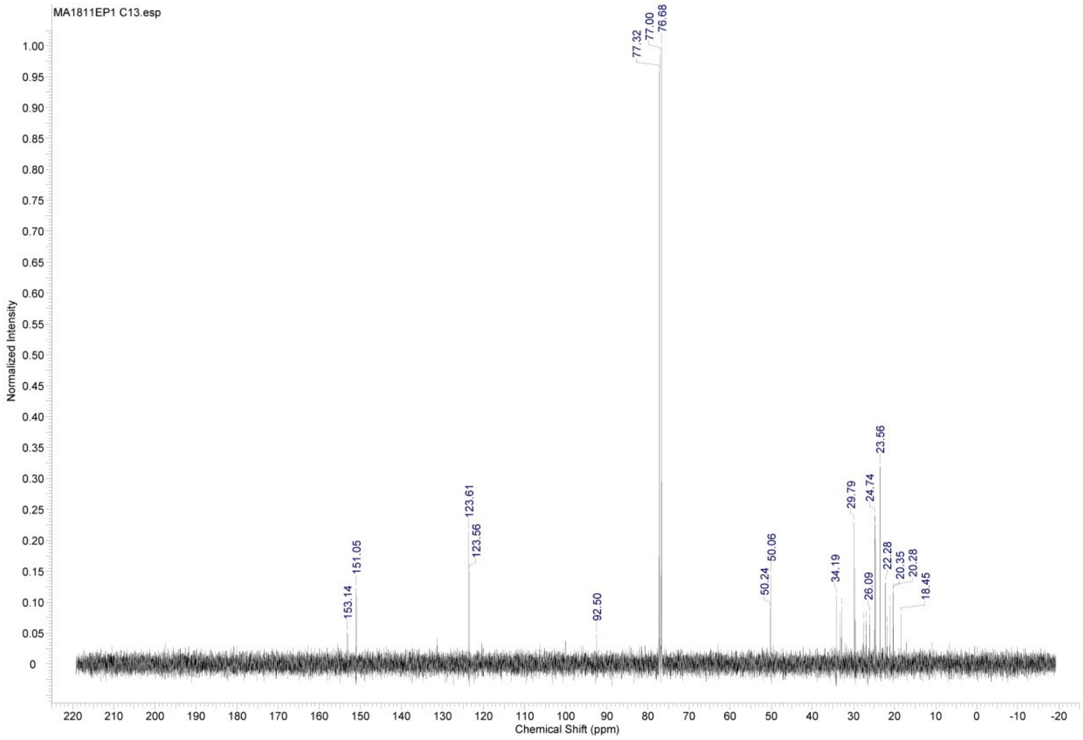

*2-(tert-Butoxy)-4-hydroxy-4-((3R,6S)-6-isopropyl-3-methylcyclohex-1-en-1-yl)-3-methylcyclobut-2-enone (**42**)*

^{1}H NMR

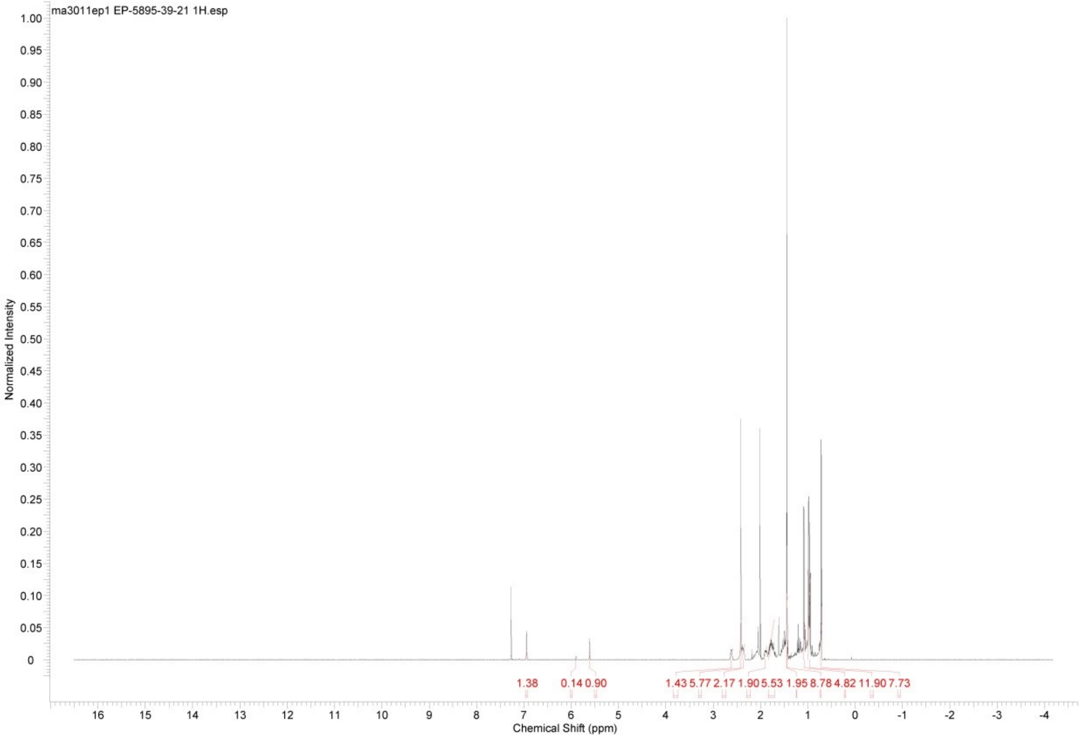

^{13}C NMR

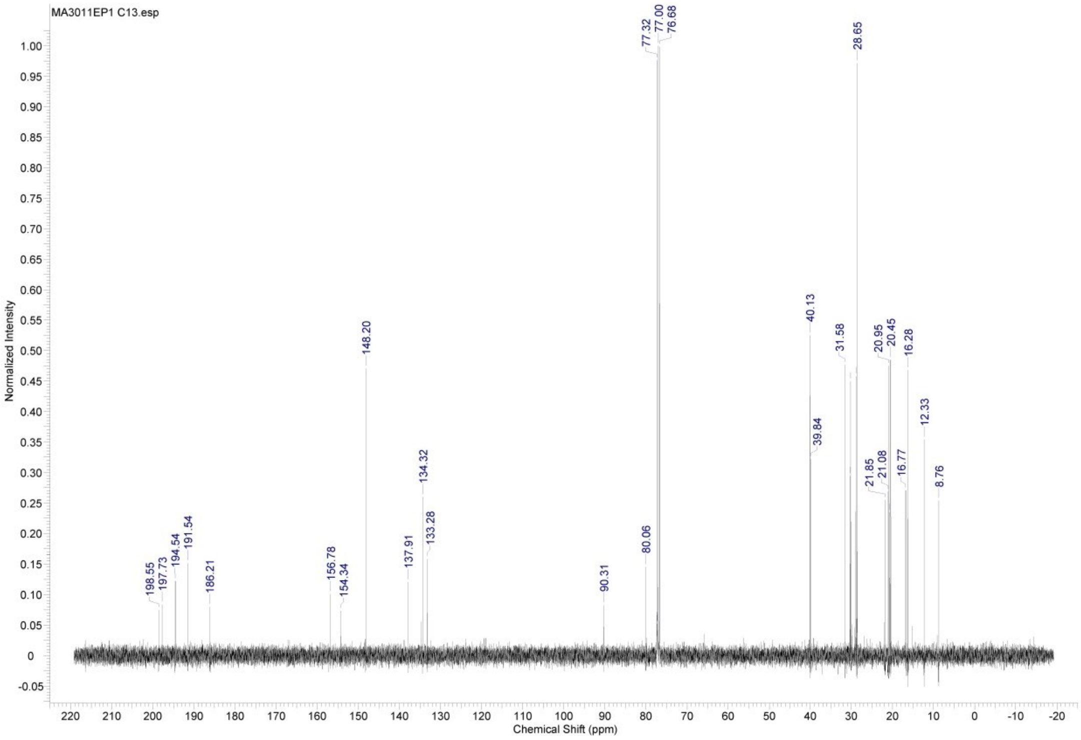

*(5S,8R)-2-(tert-Butoxy)-5-isopropyl-3,8-dimethyl-5,6,7,8-tetrahydronaphthal-ene-1,4-dione (**44**)*

^{1}H NMR

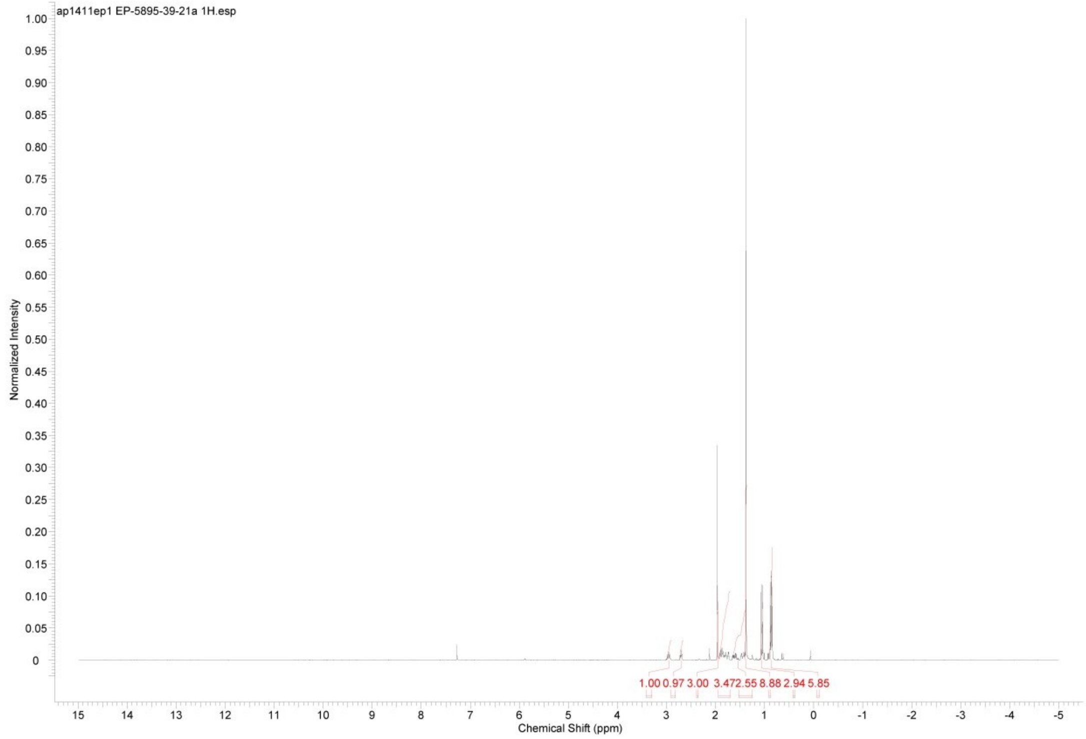

^{13}C NMR

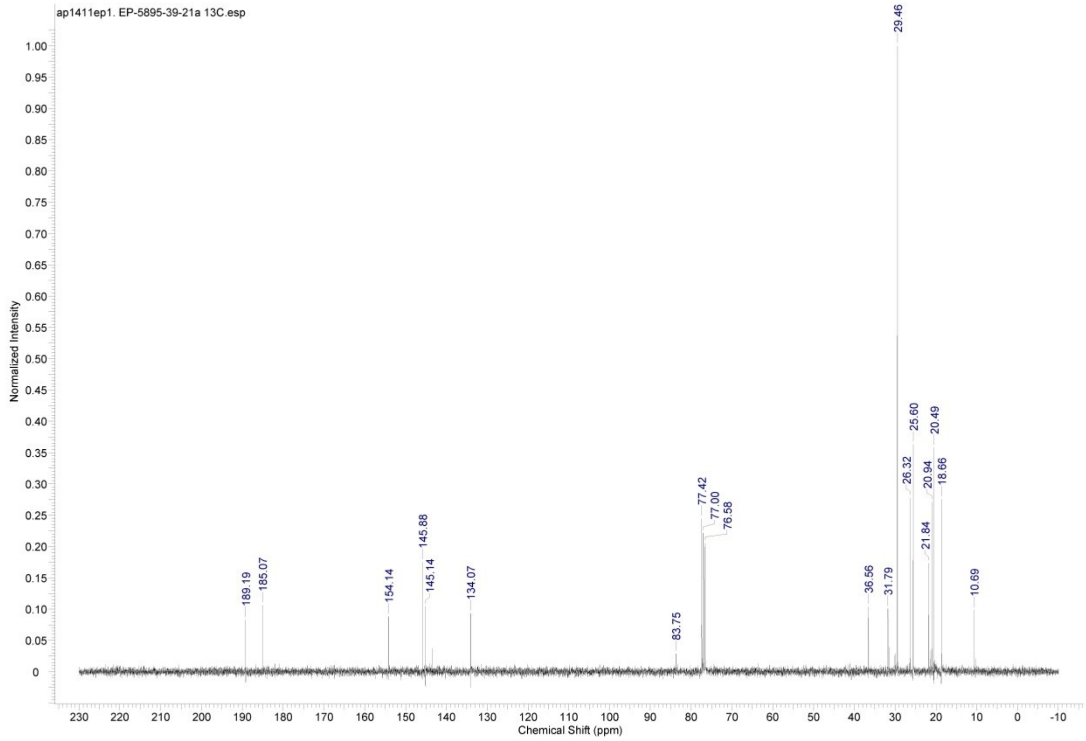

*(5S,8R)-2-Hydroxy-5-isopropyl-3,8-dimethyl-5,6,7,8-tetrahydronaphthalene-1,4-dione, (–)-Mansonone B (**43**)*

^{1}H NMR

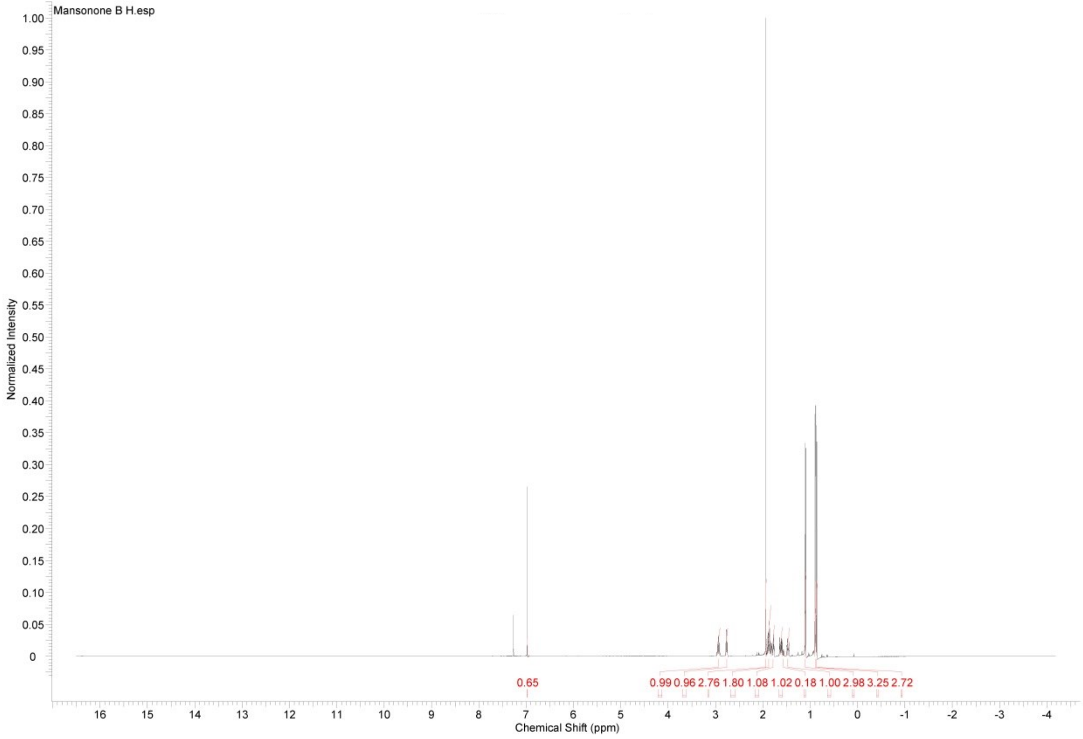

^{13}C NMR

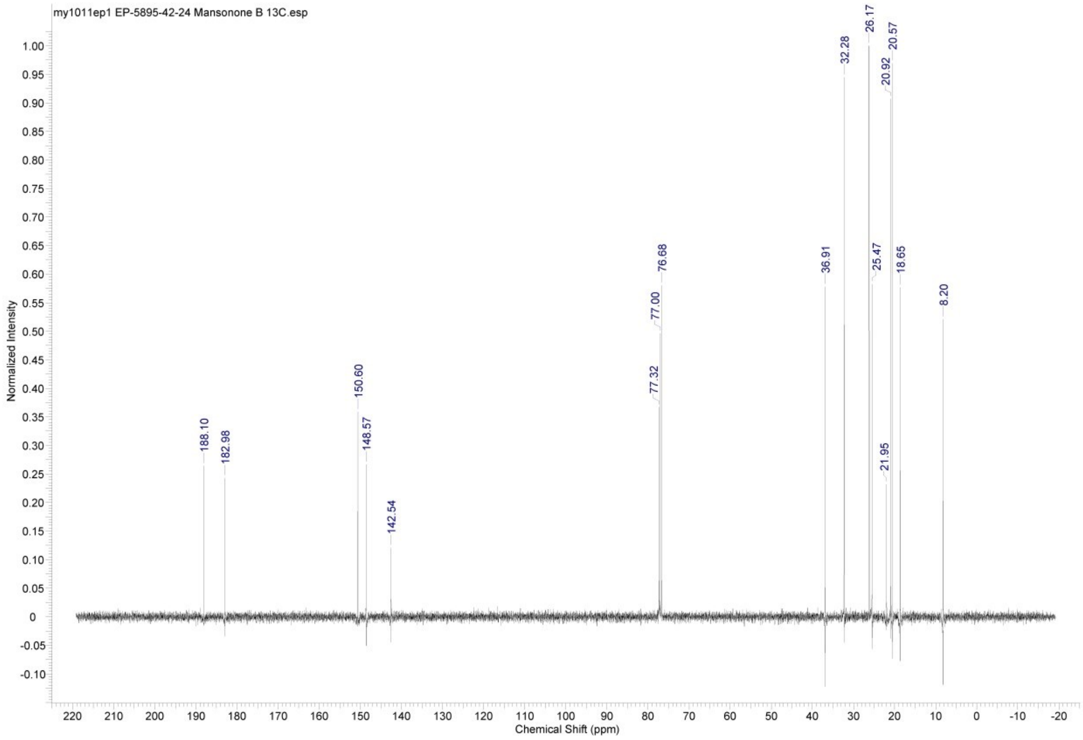

COMPUTATIONAL ANALYSIS

Geometry optimization calculations were performed using B3LYP with the mixed basis set MWB59 for Yb,[10] and 6-31G(d) for other atoms. Single point energies were calculated at the M06-2x/6-311+G(d,p) level (MWB59 for Yb)[10,11] using the IEFPCM solvation model (THF)[12] on B3LYP-optimized geometries (Gaussian 09).[13] Gibbs free energy (ΔG) at 195.150 K (–78 °C, 1 atm) in THF was calculated using DFT at the M06-2x/6-311+G(d,p)/MWB59 level with vibrational correction from B3LYP/6-31G(d)/MWB59. The lowest energy conformers were determined using M06-2x/6-311+G(d,p)/MWB59/THF(IEFPCM)//B3LYP/6-31G(d)/MWB59 using ultra fine grid for numerical integration of the density. Some pre-optimization was carried out with MOPAC PM7/SPARKLE.[14] Energies reported herein are in atomic units and Cartesian coordinates are in angstroms. ChemCraft was used for post-processing visualization.[15]

SUMMARY OF CALCULATED REACTION PATHWAYS

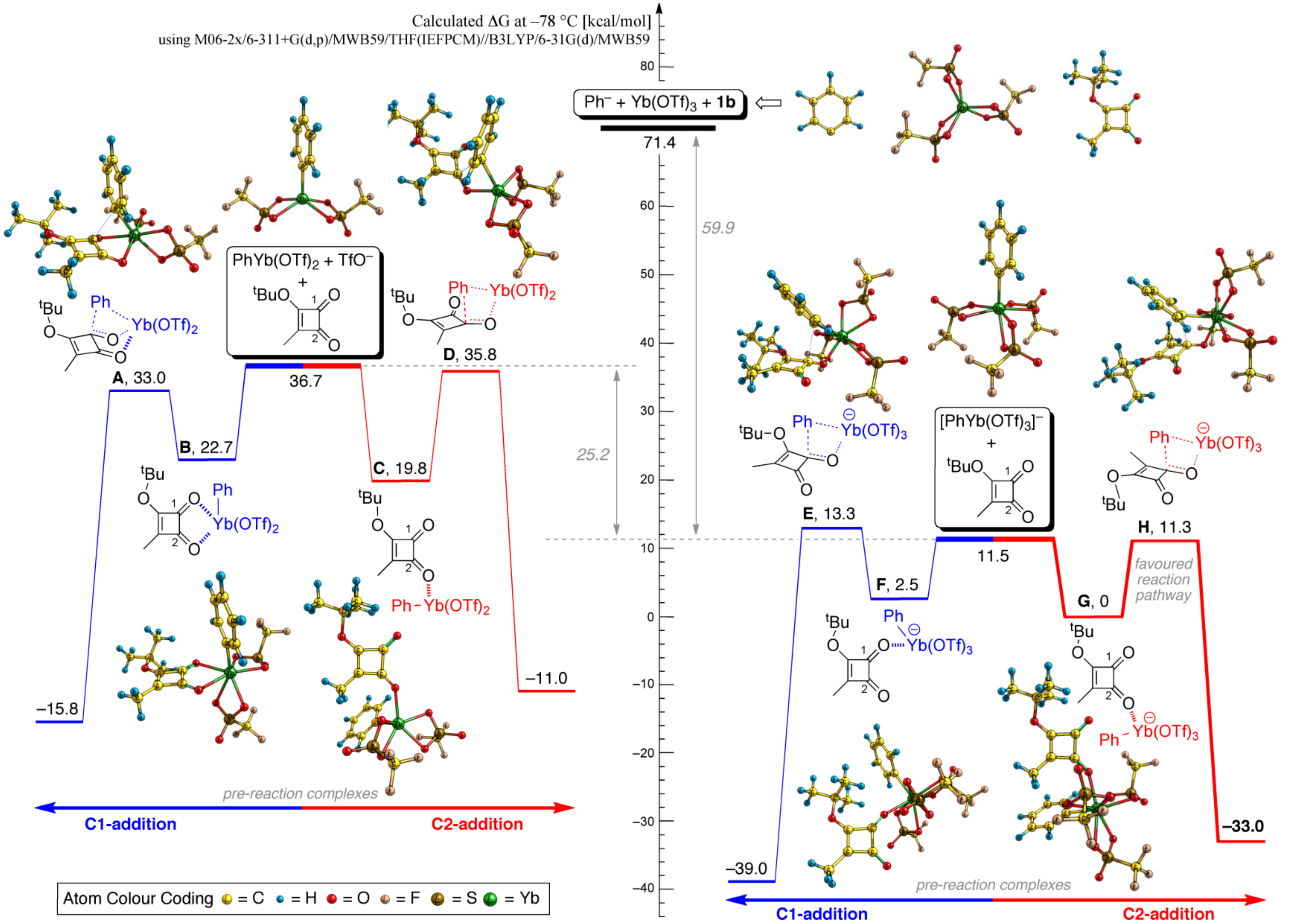

Figure. Calculated free energy barriers for C-1 and C-2 addition in the reactions of **1b** with $PhYb(OTf)_2$ and $[PhYb(OTf)_3]^-$ in THF.

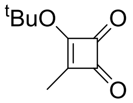	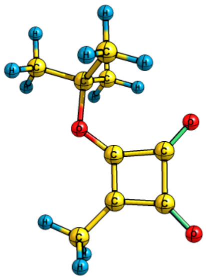	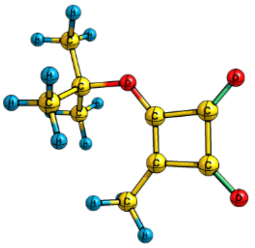	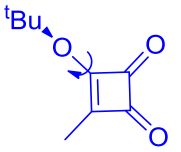	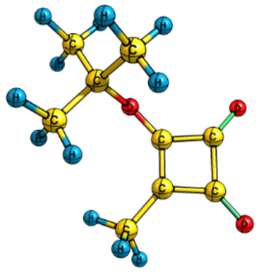
**Cyclobutenedione [1b]**	*Conformer 1*	*Conformer 2*	**Transition state for interconversion of conformers 1 & 2**	*Conformer 1*
Lowest energy conformation is:<br>Conformer 1	E(RB3LYP/6-31G(d)) = -575.817497673<br>Convg = 0.3607D-08 -V/T = 2.0092<br><br>Temperature 195.150 Kelvin. Pressure 1.00000 Atm.<br>elec. and zero-point Energies= -575.622261<br>elec. and thermal Energies= -575.615815<br>elec. and thermal Enthalpies= -575.615197<br>elec. and thermal Free Energies= -575.644893<br><br>RB3LYP/6-31G(d)/MWB59/THF(IEFPCM) = -575.825727348<br><br>RM06-2x/6-311+G(d,p)/MWB59/THF(IEFPCM) = -575.742116830	E(RB3LYP/6-31G(d)) = -575.808196165<br>Convg = 0.3438D-08 -V/T = 2.0092<br><br>Temperature 195.150 Kelvin. Pressure 1.00000 Atm.<br>elec. and zero-point Energies= -575.613041<br>elec. and thermal Energies= -575.606632<br>elec. and thermal Enthalpies= -575.606014<br>elec. and thermal Free Energies= -575.635404<br><br>RB3LYP/6-31G(d)/MWB59/THF(IEFPCM) = -575.818990981<br><br>RM06-2x/6-311+G(d,p)/MWB59/THF(IEFPCM) = -575.738099653		E(RB3LYP/6-31G(d)) = -575.802683590<br>Convg = 0.3279D-08 -V/T = 2.0092<br><br>Temperature 195.150 Kelvin. Pressure 1.00000 Atm.<br>elec. and zero-point Energies= -575.608118<br>elec. and thermal Energies= -575.602146<br>elec. and thermal Enthalpies= -575.601528<br>elec. and thermal Free Energies= -575.630117<br><br>RB3LYP/6-31G(d)/MWB59/THF(IEFPCM) = -575.812645146<br><br>RM06-2x/6-311+G(d,p)/MWB59/THF(IEFPCM) = -575.732196762
	Geometry at RB3LYP/6-31G(d)<br>Charge = 0 Multiplicity = 1<br>C 1.4615112622 -2.5144935325 0.9542462204<br>C 0.160763958 -2.2818839232 0.5523750145<br>C 0.3634140057 -0.8204369816 0.4237659523<br>C 1.8383534665 -1.0426992222 0.8741052686<br>O 2.8249488382 -0.3766673561 1.0712947869<br>O -0.3136067049 0.1296293673 0.1087356401<br>O 2.0237445349 -3.6644499398 1.2620263499<br>C 3.4469248154 -3.774693676 1.6829909606<br>C -1.0173369109 -3.1545270177 0.3267369199<br>H -0.7853854265 -4.2052119041 0.5226200993<br>H -1.373926242 -3.0574789255 -0.7064175356<br>H -1.849651595 -2.8496575563 0.9735566316<br>C 4.3494313977 -3.2922239055 0.5462783273<br>H 5.3951533101 -3.4961972835 0.8011779612<br>H 4.1164895798 -3.8236160936 -0.3825376203<br>H 4.2473490265 -2.2161918426 0.3858644922<br>C 3.5983692512 -5.2773942349 1.9181660051<br>H 4.6202169899 -5.5028482311 2.239054372<br>H 2.9074735969 -5.6195636793 2.695106605<br>H 3.3921099556 -5.8360198383 0.9997679476<br>C 3.6550494498 -2.982114327 2.9744616293<br>H 3.5563149421 -1.907522357 2.8027013726<br>H 2.9375315415 -3.2970741623 3.7395178808<br>H 4.6641449574 -3.1698403772 3.3573467185<br><br>Frequency: 44.5895; 56.7593; 68.9542; 113.7256; 130.4876; 187.8884; 201.5167; 219.7890; 250.1832; 271.2168; 276.3812; 288.5306; 327.8654; 346.5425; 395.9424; 453.0818; 470.8518; 491.0972; 588.5406; 600.0985; 629.8520; 730.1502; 771.7605; 786.4301; 855.2987; 935.6303; 941.1140; 954.5731; 978.9200; 987.9096; 1056.4236; 1060.9778; 1069.8487; 1080.8350; 1207.9472; 1237.7450; 1286.1771; 1303.6509; 1386.3858; 1432.4219; 1433.0150; 1442.4351; 1459.7246; 1502.9588; 1503.8768; 1505.8214; 1517.7392; 1518.3537; 1535.1476; 1537.5830; 1554.7082; 1654.2738; 1852.3751; 1880.0075; 3046.4469; 3060.4836; 3060.6675; 3067.9733; 3097.9977; 3126.3231; 3128.1496; 3138.0008; 3138.8175; 3141.1606; 3156.7739; 3160.2848.<br><br>Cartesian Forces: Max 0.000001068 RMS 0.000000383<br><br>Item Value Threshold Converged?<br>Maximum Force 0.000000 0.000450 YES<br>RMS Force 0.000000 0.000300 YES<br>Maximum Displacement 0.000047 0.001800 YES<br>RMS Displacement 0.000008 0.001200 YES	Geometry at RB3LYP/6-31G(d)<br>Charge = 0 Multiplicity = 1<br>C 1.2314721011 -2.6070179385 0.8726880445<br>C -0.0243018375 -2.1711758064 0.4838611086<br>C 0.4276012896 -0.7582945477 0.3432717326<br>C 1.8466225106 -1.2219616981 0.7845627352<br>O 2.9384558043 -0.7615705672 0.9831775809<br>O -0.1155744939 0.2691321883 0.0133664892<br>O 1.9051687221 -3.6786608698 1.2288552152<br>C 1.374071224 -5.0580295811 1.2683823654<br>C -1.4075899034 -2.6676538024 0.2479283506<br>H -1.4466187944 -3.603281943 -0.3187000679<br>H -1.9615065595 -1.9017850372 -0.3057156502<br>H -1.9400114346 -2.8323086149 1.1940953764<br>C 0.1950725557 -5.1381467673 2.239849044<br>H -0.0995004141 -6.184917705 2.3703769279<br>H 0.4801018678 -4.7398548209 3.2187966713<br>H -0.6785150003 -4.5904878049 1.8804264427<br>C 2.5689014527 -5.852227175 1.8001239782<br>H 2.3165820042 -6.9157920631 1.8601090473<br>H 3.4324594312 -5.73470889 1.1388187758<br>H 2.8494670915 -5.503246202 2.7984830547<br>C 1.0176641945 -5.4971233659 -0.1531748938<br>H 0.2053304653 -4.9006433746 -0.5758965908<br>H 1.8888814928 -5.4075649119 -0.8095470428<br>H 0.6978314402 -6.5445848281 -0.1479275782<br><br>Frequency: 51.4212; 67.0276; 95.9743; 123.6570; 133.3089; 185.5698; 208.1619; 212.2290; 233.9731; 245.7059; 260.7482; 280.6795; 327.7204; 348.9508; 393.4931; 448.1293; 477.0914; 479.7893; 589.8499; 599.5865; 643.1480; 758.9906; 779.6483; 782.5151; 843.2691; 932.7471; 934.1966; 965.3755; 979.4071; 990.4162; 1053.4753; 1058.9561; 1060.2146; 1079.7601; 1188.9452; 1209.6825; 1284.3450; 1294.6922; 1369.2618; 1430.7780; 1432.7224; 1439.7565; 1458.2384; 1500.8417; 1504.2819; 1513.8193; 1515.9270; 1519.2072; 1527.8602; 1530.1106; 1548.3495; 1651.3465; 1873.2711; 1889.8268; 3043.0817; 3064.4480; 3067.2951; 3073.8596; 3102.0451; 3128.8242; 3132.6301; 3134.4165; 3142.8562; 3144.9530; 3154.2783; 3165.9520.<br><br>Cartesian Forces: Max 0.000000861 RMS 0.000000432<br><br>Item Value Threshold Converged?<br>Maximum Force 0.000000 0.000450 YES<br>RMS Force 0.000000 0.000300 YES<br>Maximum Displacement 0.000005 0.001800 YES<br>RMS Displacement 0.000001 0.001200 YES		Geometry at RB3LYP/6-31G(d)<br>Charge = 0 Multiplicity = 1<br>C 1.4401653784 -2.5807075248 1.0116385879<br>C 0.2153732023 -2.2459704035 0.4773244109<br>C 0.4202957521 -0.7838298054 0.7284526931<br>C 1.81157644 -1.1513888911 1.3447556758<br>O 2.754437038 -0.5997971863 1.8467927929<br>O -0.2321682481 0.2099848015 0.5230050524<br>O 2.1081686476 -3.692001274 1.3033709011<br>C 2.6948686295 -4.5774605524 0.2708986115<br>C -0.9676679993 -2.9369768813 -0.0937362202<br>H -1.0485174197 -3.9742728065 0.2435255691<br>H -0.9292962434 -2.938818677 -1.1913483659<br>H -1.8770404001 -2.393547086 0.1863630534<br>C 1.5943423349 -5.2449212 -0.5538599432<br>H 2.038815988 -5.9786734943 -1.2352108281<br>H 0.8858496686 -5.7684931832 0.0960004052<br>H 1.0478500627 -4.5188991624 -1.1625763161<br>C 3.4621405648 -5.6022275221 1.1044210002<br>H 3.9678516741 -6.318441026 0.4487274928<br>H 4.2128382366 -5.1044236386 1.7249894464<br>H 2.7803673588 -6.1522377796 1.7606571668<br>C 3.6365690673 -3.7418569235 -0.5993098184<br>H 3.0842267574 -2.991572119 -1.175972989<br>H 4.3750211342 -3.2264865349 0.0221941186<br>H 4.1656280233 -4.3869657908 -1.308918642<br><br>Frequency: -48.4179; 37.6429; 77.0254; 120.9655; 136.6519; 185.5733; 213.8047; 227.6547; 229.4974; 265.6946; 274.2465; 283.7098; 320.4253; 340.7327; 392.3666; 436.7822; 447.9180; 468.7972; 559.7781; 611.7515; 682.7911; 749.0528; 759.7470; 794.1474; 842.9110; 929.3810; 935.0488; 950.5605; 969.8828; 979.7281; 1053.0834; 1057.1334; 1058.1918; 1068.2188; 1191.4143; 1204.3075; 1278.9746; 1296.0645; 1364.4808; 1428.5101; 1431.6423; 1438.4754; 1456.2041; 1499.4801; 1500.8730; 1506.9694; 1515.3232; 1518.2098; 1526.9716; 1529.5187; 1547.6167; 1652.0735; 1875.9243; 1888.6738; 3044.5500; 3056.8123; 3060.7589; 3067.8278; 3101.7493; 3123.6550; 3128.4853; 3137.7754; 3139.0387; 3140.8435; 3143.8762; 3146.3933.<br><br>Cartesian Forces: Max 0.000001084 RMS 0.000000446<br><br>Item Value Threshold Converged?<br>Maximum Force 0.000000 0.000450 YES<br>RMS Force 0.000000 0.000300 YES<br>Maximum Displacement 0.000005 0.001800 YES<br>RMS Displacement 0.000001 0.001200 YES
	FILE:<br>Yb_Sq2_PhYbOTf3_GS_Anion_B3LYP_M062x_Sq2_Conf1.out	FILE:<br>Yb_Sq2_PhYbOTf3_GS_Anion_B3LYP_M062x_Sq2_Conf2.out		FILE:<br>Yb_Sq2_PhYbOTf3_TS_Anion_B3LYP_M062x_Sq2_Conf1-Conf2.out

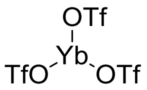	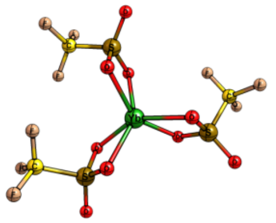	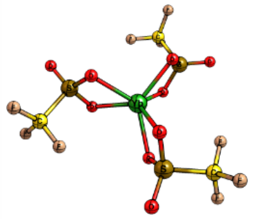
**$Yb(OTf)_3$**	*Conformer 1*	*Conformer 2*
Lowest energy conformation is:<br>Conformer 2	E(RB3LYP/6-31G(d)/MWB59) = -2923.92531547<br>A.U. after 1 cycles Convg = 0.5132D-08 -V/T = 2.0137<br><br>Temperature 195.150 Kelvin. Pressure 1.00000 Atm.<br>elec. and zero-point Energies= -2923.839206<br>elec. and thermal Energies= -2923.825746<br>elec. and thermal Enthalpies= -2923.825128<br>elec. and thermal Free Energies= -2923.874558<br><br>RB3LYP/6-31G(d)/MWB59/THF(IEFPCM) = -2923.94931071<br><br>RM06-2x/6-311+G(d,p)/MWB59/THF(IEFPCM) = -2923.85302428	E(RB3LYP/6-31G(d)/MWB59) = -2923.92604172<br>A.U. after 1 cycles Convg = 0.3144D-08 -V/T = 2.0137<br><br>Temperature 195.150 Kelvin. Pressure 1.00000 Atm.<br>elec. and zero-point Energies= -2923.839958<br>elec. and thermal Energies= -2923.826457<br>elec. and thermal Enthalpies= -2923.825839<br>elec. and thermal Free Energies= -2923.876272<br><br>RB3LYP/6-31G(d)/MWB59/THF(IEFPCM) = -2923.94989808<br><br>RM06-2x/6-311+G(d,p)/MWB59/THF(IEFPCM) = -2923.85316277
	Geometry at RB3LYP/6-31G(d)/MWB59<br>Charge = 0 Multiplicity = 1<br>Yb 0.4105070924 0.4136978192 0.0010221409<br>O 1.3145844892 2.1502133445 1.1925026272<br>S 1.1836574607 1.5205290226 2.5795051296<br>O 2.3410361651 1.5529276333 3.447081258<br>O 0.5509170813 0.1628778656 2.279578435<br>C -0.1918948731 2.4917068652 3.3925806256<br>F 0.2043216137 3.7475312364 3.5554702455<br>F -1.2673662643 2.4534551197 2.6017056607<br>F -0.4740054786 1.9397500775 4.5656948667<br>O -1.5661760893 1.4465188601 -0.574995622<br>O -3.688835356 0.1930129222 0.0653605809<br>O -1.4317915056 -0.9265395108 -0.3119819661<br>S -2.4253735845 0.1862295712 -0.6407217697<br>C -2.7777203853 -0.0349953271 -2.4632383177<br>F -3.5551214029 0.9580689656 -2.8752601512<br>F -1.6215195654 -0.0129775215 -3.1324482174<br>F -3.3818650567 -1.2023368427 -2.6474424808<br>O 2.0766100804 -1.0742085789 -0.5416601203<br>S 2.4817760434 -0.4336173293 -1.8676381659<br>C 1.8773697035 -1.6414247152 -3.1601040887<br>O 3.8890011774 -0.1903790876 -2.1001217815<br>O 1.5234896768 0.7503981416 -1.9838494035<br>F 2.0230546086 -1.0975449999 -4.3620214183<br>F 0.5857869516 -1.9027792839 -2.9354894369<br>F 2.5845614796 -2.760119458 -3.0699195561<br><br>Frequency: 18.8561; 21.5282; 23.6063; 26.1356; 27.9372; 29.1137; 40.9929; 45.3644; 46.1417; 55.4708; 59.0740; 69.4642; 161.9186; 164.5465; 165.6433; 169.7910; 190.0321; 200.9730; 215.4324; 226.9825; 232.0165; 247.9682; 254.1427; 257.9373; 301.0439; 304.1764; 306.3042; 317.6150; 317.8751; 317.9754; 368.5651; 371.7922; 373.4997; 490.5161; 491.1814; 491.5496; 535.5149; 536.0423; 541.2056; 557.6509; 557.9161; 558.0158; 578.4908; 580.8304; 589.1415; 622.3220; 626.8777; 649.7241; 766.6954; 766.9872; 767.5319; 938.1824; 940.3793; 959.3734; 1026.6491; 1029.4515; 1043.6029; 1188.0763; 1189.9124; 1192.0989; 1252.3737; 1260.9697; 1263.5726; 1284.8289; 1285.4972; 1286.4805; 1334.1386; 1335.6026; 1348.1826.<br><br>Cartesian Forces: Max 0.000000720 RMS 0.000000333<br><br>Item Value Threshold Converged?<br>Maximum Force 0.000000 0.000450 YES<br>RMS Force 0.000000 0.000300 YES<br>Maximum Displacement 0.000002 0.001800 YES<br>RMS Displacement 0.000001 0.001200 YES	Geometry at RB3LYP/6-31G(d)/MWB59<br>Charge = 0 Multiplicity = 1<br>Yb 0.3100943014 0.4723770479 0.0321633232<br>O 1.1385415353 2.3007792372 1.1530808395<br>S 1.261543534 1.642534882 2.5249090023<br>O 2.479606406 1.8592341316 3.2752026679<br>O 0.8330084242 0.2025594426 2.2546289002<br>C -0.1731050764 2.3555983881 3.486549696<br>F 0.0142728605 3.6576983967 3.6532253381<br>F -1.2928021435 2.1451967199 2.7819962557<br>F -0.2605195534 1.7457904984 4.6607913413<br>O -1.4845517389 1.574500903 -0.8902591209<br>O -3.8053006467 0.9273215501 -0.0582561791<br>O -1.7429046574 -0.554093853 0.1641376403<br>S -2.5333240604 0.5033464256 -0.6022077816<br>C -2.8245748453 -0.2877072312 -2.2697588552<br>F -3.3764204381 0.5995979705 -3.0861490171<br>F -1.6398268813 -0.6778881883 -2.7582509245<br>F -3.6183395721 -1.3398295145 -2.1237998747<br>O 1.6751369429 0.7085227343 -1.8029281882<br>S 2.2039257633 -0.7231687086 -1.8312914373<br>C 3.9435337786 -0.5642214754 -1.1680752645<br>O 2.2516227113 -1.4042123135 -3.1070250463<br>O 1.4435272157 -1.3929027969 -0.6895286722<br>F 4.4643281267 -1.7732319595 -1.0085536492<br>F 3.8833221974 0.0630752296 0.0139444021<br>F 4.6734445652 0.1466353132 -2.0167726028<br><br>Frequency: 4.9477; 21.1435; 21.5423; 24.1560; 24.2709; 32.7097; 42.5850; 43.2750; 43.4335; 54.3252; 63.3296; 63.4332; 164.3305; 168.2853; 169.8552; 169.9118; 199.0497; 199.1016; 214.3900; 232.5435; 232.6042; 243.0633; 248.4710; 248.6503; 301.1644; 307.0209; 307.1649; 316.8652; 319.0567; 319.1070; 370.6303; 371.0717; 371.0990; 490.9172; 490.9535; 491.2549; 535.8876; 535.8964; 540.5345; 556.8494; 556.8697; 556.9484; 583.3248; 583.3503; 585.7522; 625.4812; 625.5129; 645.0300; 766.3904; 766.3992; 766.7707; 942.3902; 942.4312; 957.2622; 1030.0636; 1030.0885; 1052.8928; 1185.5461; 1188.5865; 1188.6529; 1252.9828; 1253.0600; 1255.0403; 1288.5070; 1288.5540; 1290.1298; 1337.2543; 1337.2897; 1349.1349.<br><br>Cartesian Forces: Max 0.000001112 RMS 0.000000380<br><br>Item Value Threshold Converged?<br>Maximum Force 0.000000 0.000450 YES<br>RMS Force 0.000000 0.000300 YES<br>Maximum Displacement 0.000053 0.001800 YES<br>RMS Displacement 0.000019 0.001200 YES
	FILE: Yb_YbOTf3_GS_Neutral_B3LYP_M062x_Conf1_A.out	FILE: Yb_YbOTf3_GS_Neutral_B3LYP_M062x_Conf2_A.out

[PhYb(OTf)$_3$]$^{\ominus}$	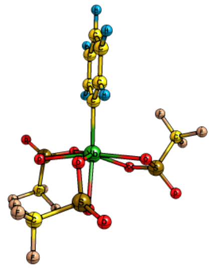	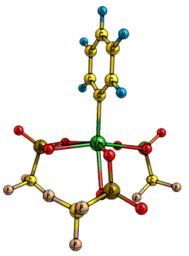	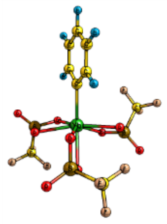
**[PhYb(OTf)$_3$]$^-$**	*Conformer 1*	*Conformer 2*	*Conformer 3*
Lowest energy conformation is:<br>Conformer 2	E(RB3LYP/6-31G(d)/MWB59) = -3155.64832524<br>Convg = 0.4827D-08 -V/T = 2.0132<br><br>Temperature 195.150 Kelvin. Pressure 1.00000 Atm.<br>elec. and zero-point Energies= -3155.473397<br>elec. and thermal Energies= -3155.456744<br>elec. and thermal Enthalpies= -3155.456126<br>elec. and thermal Free Energies= -3155.513147<br><br>RB3LYP/6-31G(d)/MWB59/THF(IEFPCM) = -3155.70619568<br><br>RM06-2x/6-311+G(d,p)/MWB59/THF(IEFPCM) = -3155.59801452	E(RB3LYP/6-31G(d)/MWB59) = -3155.64825488<br>Convg = 0.6353D-08 -V/T = 2.0132<br><br>Temperature 195.150 Kelvin. Pressure 1.00000 Atm.<br>elec. and zero-point Energies= -3155.473338<br>elec. and thermal Energies= -3155.456666<br>elec. and thermal Enthalpies= -3155.456048<br>elec. and thermal Free Energies= -3155.513268<br><br>RB3LYP/6-31G(d)/MWB59/THF(IEFPCM) = -3155.70634055<br><br>RM06-2x/6-311+G(d,p)/MWB59/THF(IEFPCM) = -3155.59831334	E(RB3LYP/6-31G(d)/MWB59) = -3155.64859015<br>Convg = 0.9235D-08 -V/T = 2.0132<br><br>Temperature 195.150 Kelvin. Pressure 1.00000 Atm.<br>elec. and zero-point Energies= -3155.473688<br>elec. and thermal Energies= -3155.457025<br>elec. and thermal Enthalpies= -3155.456407<br>elec. and thermal Free Energies= -3155.513559<br><br>RB3LYP/6-31G(d)/MWB59/THF(IEFPCM) = -3155.70640944<br><br>RM06-2x/6-311+G(d,p)/MWB59/THF(IEFPCM) = -3155.59778115
	Geometry at RB3LYP/6-31G(d)/MWB59<br>Charge = -1 Multiplicity = 1<br>Yb 0.2540115402 2.9054851217 0.7972620338<br>O 1.6041565363 1.9039740055 -0.8932878633<br>S 2.9495544725 1.9569720908 -0.2039676873<br>O 3.7182657538 0.7206561147 -0.1436072935<br>O 2.7318845531 2.7196116963 1.0748352987<br>C 3.9699158145 3.093115954 -1.27342569<br>F 4.1331791198 2.5394560861 -2.481367068<br>F 3.3871145473 4.2831186021 -1.4175441156<br>F 5.1732684408 3.2649659709 -0.7099984681<br>O -0.5878258488 4.9930557771 1.6299662748<br>O -0.7657440015 6.9528389776 0.0214614004<br>O 0.7800471087 4.9693362535 -0.3450833311<br>S 0.0476830627 5.8909632743 0.595641729<br>C 1.4100640679 6.7404230298 1.5391531563<br>F 0.8861238502 7.5424266848 2.4716839823<br>F 2.188659897 5.830348345 2.1350882658<br>F 2.154729594 7.4714618067 0.7037514928<br>C -1.7199598638 2.3789076781 -0.4017365893<br>C -1.7577985523 1.3108489277 -1.3275150219<br>C -2.9322436659 3.089000744 -0.2418490972<br>H -0.8568020307 0.7220973101 -1.5031954219<br>H -2.9776622648 3.928199289 0.4536319963<br>C -2.9123025244 0.9706153435 -2.0400288606<br>C -4.0971355388 2.7621752431 -0.9453479483<br>H -2.8955162699 0.1390624659 -2.7436966233<br>H -5.008902013 3.338015122 -0.7905522478<br>C -4.0897048214 1.6980215802 -1.8493050348<br>H -4.9919346074 1.4386460553 -2.4009180244<br>O 0.9472155038 0.4964288157 4.1893403395<br>S 0.092155415 1.0371560731 3.1421267535<br>C -1.6069088003 0.3552732798 3.4893710973<br>O 0.3774619048 0.6476088068 1.7169449041<br>O -0.117062224 2.5366936306 3.1171025956<br>F -2.4965110017 0.8356718741 2.6197405243<br>F -1.9797787922 0.6978952248 4.7285278549<br>F -1.5794777043 -0.9786992064 3.397292124<br><br>Frequency: 13.3767; 18.6940; 21.6351; 24.0913; 26.7694; 27.9371; 28.5506; 33.6262; 37.0278; 43.3980; 51.3690; 58.8904; 68.2283; 73.7190; 78.6705; 115.7860; 126.1366; 138.5500; 148.1371; 152.5639; 156.4271; 172.8151; 184.7058; 196.8914; 207.5050; 214.7536; 215.5781; 218.5190; 223.8766; 228.8298; 303.3680; 306.0762; 306.7979; 321.2043; 322.3183; 323.7597; 354.4224; 356.6389; 361.2432; 393.4076; 445.1299; 489.5217; 489.6785; 492.0592; 524.7370; 527.1756; 532.0888; 557.3981; 558.1173; 558.4669; 570.8267; 575.6466; 580.2538; 621.4640; 623.6514; 633.5901; 634.8596; 653.1232; 717.7240; 737.4336; 761.6429; 762.0334; 762.6134; 870.2948; 915.2745; 969.1013; 970.9200; 973.0509; 980.9057; 987.4503; 1011.2102; 1037.5612; 1086.0216; 1087.0476; 1096.6768; 1105.6707; 1115.0425; 1185.1371; 1212.3841; 1212.9835; 1214.5073; 1224.5764; 1234.5038; 1239.6689; 1240.1394; 1243.3790; 1249.1768; 1253.7772; 1295.7734; 1307.9356; 1313.5622; 1323.7205; 1369.7590; 1459.0939; 1522.7619; 1615.4620; 1628.7737; 3134.4594; 3139.3157; 3152.2070; 3161.6109; 3178.2985.<br><br>Cartesian Forces: Max 0.000000619 RMS 0.000000305<br><br>Item Value Threshold Converged?<br>Maximum Force 0.000000 0.000450 YES<br>RMS Force 0.000000 0.000300 YES<br>Maximum Displacement 0.000105 0.001800 YES<br>RMS Displacement 0.000021 0.001200 YES	Geometry at RB3LYP/6-31G(d)/MWB59<br>Charge = -1 Multiplicity = 1<br>Yb -0.2927940897 3.0489823391 0.661899952<br>O 1.5747938535 2.9778095798 -0.8315618478<br>S 1.9594411349 1.5318224512 -0.6192954048<br>O 2.2605876959 0.728862843 -1.7956791443<br>O 0.9668610038 0.9917187481 0.3751643588<br>C 3.5538751579 1.6439225141 0.3372926148<br>F 4.5134964421 2.1568272093 -0.4405754254<br>F 3.3969543899 2.4300382418 1.4075144429<br>F 3.9265220185 0.4254540563 0.7421244865<br>O -1.5563585872 4.8539540054 1.7002440464<br>O -1.711953043 7.0069128087 0.3602216448<br>O 0.1628910029 5.3147647821 0.0877900153<br>S -0.8776886831 5.9778334656 0.9642405197<br>C 0.1324389687 6.8549438503 2.2622285677<br>F -0.6758545867 7.3391320083 3.2097326999<br>F 1.0099231892 6.0175855909 2.8196586511<br>F 0.7992065522 7.8695312062 1.6980177886<br>C -1.9881139773 2.4758525722 -0.881124889<br>C -1.8218671325 2.6654817399 -2.2724250568<br>C -3.2280643304 1.9258478919 -0.4835300599<br>H -0.888106203 3.0824407548 -2.6535099792<br>H -3.4214448092 1.7517811289 0.5752511441<br>C -2.8143574687 2.3360167119 -3.2024786034<br>C -4.2306415453 1.5905183142 -1.3990793864<br>H -2.6431390973 2.499025529 -4.2658201228<br>H -5.1724305599 1.169617193 -1.0487202909<br>C -4.0254649092 1.7958701521 -2.7656567832<br>H -4.8022925677 1.5368446232 -3.4832440943<br>O -1.1216747767 2.7056922896 4.7618066487<br>S -0.5046692616 2.1708285523 3.5542723767<br>C 0.1967099609 0.5242991811 4.0758819678<br>O 0.6603764002 2.9225455254 2.9686996852<br>O -1.4162120843 1.8398719119 2.3956235808<br>F 0.788370271 -0.100573275 3.0586087211<br>F -0.7895429231 -0.249047302 4.5469658726<br>F 1.0970452523 0.7179702596 5.0493099036<br><br>Frequency: 13.2551; 14.4257; 21.1370; 23.3046; 26.1377; 27.3209; 31.0719; 34.6520; 35.2064; 45.3162; 48.5106; 60.4957; 66.8708; 74.6937; 81.1490; 112.5131; 125.3739; 137.1153; 146.3811; 148.6487; 154.5477; 179.8395; 188.7123; 192.8780; 208.3240; 213.3410; 216.5146; 217.8980; 223.7145; 227.8269; 303.0040; 305.6559; 306.9327; 320.5940; 322.0840; 324.5999; 353.9722; 355.7721; 361.4729; 392.8222; 444.7610; 489.7689; 489.9019; 492.3151; 524.6476; 527.0749; 531.8882; 557.5496; 558.4528; 558.6191; 570.8091; 575.0381; 580.0518; 621.5114; 624.3509; 633.3943; 635.4993; 653.6114; 717.1231; 737.3799; 761.6344; 762.1983; 762.4007; 869.7876; 915.3954; 969.3262; 971.4418; 971.9296; 982.5148; 987.7931; 1011.2092; 1038.0334; 1085.7957; 1087.7718; 1097.4721; 1107.9179; 1116.0236; 1185.4287; 1212.4130; 1212.6475; 1215.0355; 1224.4566; 1233.7278; 1238.6792; 1241.3105; 1242.6887; 1249.0357; 1254.7955; 1296.5913; 1306.6731; 1313.0989; 1323.4169; 1370.0348; 1459.2862; 1523.0852; 1615.8895; 1629.1114; 3130.0564; 3141.0886; 3153.1240; 3162.6107; 3178.9248.<br><br>Cartesian Forces: Max 0.000001724 RMS 0.000000608<br><br>Item Value Threshold Converged?<br>Maximum Force 0.000000 0.000450 YES<br>RMS Force 0.000000 0.000300 YES<br>Maximum Displacement 0.000225 0.001800 YES<br>RMS Displacement 0.000040 0.001200 YES	Geometry at RB3LYP/6-31G(d)/MWB59<br>Charge = -1 Multiplicity = 1<br>Yb 0.2597177518 2.8825158124 0.8945086634<br>O 2.7233299306 2.7435363582 1.2820448391<br>S 3.0399679777 2.1975962119 -0.0840224415<br>O 4.0627415214 2.8777888903 -0.8688124308<br>O 1.7064751746 1.9453680431 -0.7521497853<br>C 3.7337689262 0.4937216443 0.2197462093<br>F 4.8769283727 0.6003997068 0.9089490571<br>F 2.881844767 -0.2656893543 0.9057349406<br>F 3.9906763436 -0.0938889431 -0.9563700021<br>O -0.6159945182 4.9908446873 1.5994061183<br>O -0.6840429251 6.8987113913 -0.0767658385<br>O 0.8829448966 4.9072528823 -0.2758846926<br>S 0.091430931 5.8583781129 0.5831601888<br>C 1.3826291851 6.7431119282 1.5927617683<br>F 0.7892511532 7.5798601131 2.4512233794<br>F 2.1113795553 5.8586720261 2.2808774187<br>F 2.1872101241 7.4425396998 0.7877897996<br>C -1.6696201779 2.307563138 -0.3574632714<br>C -1.6918939204 1.1638234534 -1.1884303694<br>C -2.8678141611 3.0576412991 -0.3244990812<br>H -0.8009192786 0.539059821 -1.2650870456<br>H -2.9246197599 3.9551743738 0.2926757638<br>C -2.8171670303 0.7901477953 -1.930116887<br>C -4.004135827 2.6986685633 -1.0585403454<br>H -2.7880614897 -0.0997336345 -2.5579642749<br>H -4.9056316706 3.3078118234 -1.0027802887<br>C -3.9813725396 1.559822515 -1.8659543016<br>H -4.8612126192 1.2752416083 -2.4407852992<br>O 0.821477958 0.5512908047 4.3645323529<br>S -0.0041559233 1.1043180025 3.2995864197<br>C -1.7341933277 0.5093327762 3.651922045<br>O 0.2714151362 0.6597763641 1.8880428244<br>O -0.1438741907 2.6086699341 3.2315278695<br>F -2.5958125482 1.0146226545 2.76834805<br>F -2.0958259234 0.8926373595 4.8822271465<br>F -1.7692586209 -0.8260866822 3.5850429684<br><br>Frequency: 11.7888; 17.7126; 22.8493; 24.7530; 26.0649; 28.8247; 31.2200; 33.8484; 35.6998; 40.9773; 45.6417; 56.0496; 68.9072; 73.6172; 80.2486; 116.5415; 125.8010; 140.3320; 148.7463; 155.2242; 156.8328; 173.6958; 183.7627; 195.1783; 209.3353; 213.0513; 215.9533; 219.8734; 223.3348; 226.8759; 303.4350; 305.8135; 307.3406; 321.0940; 322.6305; 324.0772; 354.6170; 356.5770; 360.9712; 392.7604; 445.0319; 489.6496; 489.9950; 492.0797; 525.0982; 527.1643; 531.8679; 557.5080; 558.3916; 558.5292; 571.3500; 574.9579; 579.5293; 623.3661; 624.9934; 633.1358; 633.8673; 653.0138; 716.8865; 736.9684; 762.2196; 762.4166; 762.7497; 869.3091; 913.6316; 969.7770; 970.9038; 971.5654; 980.2481; 986.5479; 1011.3889; 1037.5111; 1086.0707; 1087.2217; 1097.0666; 1105.2906; 1114.8896; 1185.1028; 1211.2525; 1212.9580; 1213.6669; 1224.4464; 1234.8721; 1240.0635; 1240.8482; 1246.6788; 1248.8672; 1255.7345; 1295.8498; 1306.9511; 1312.4913; 1321.8205; 1369.3586; 1459.1077; 1522.6017; 1615.5362; 1628.7578; 3134.0457; 3137.0474; 3151.5220; 3161.1857; 3178.3688.<br><br>Cartesian Forces: Max 0.000002140 RMS 0.000000745<br><br>Item Value Threshold Converged?<br>Maximum Force 0.000000 0.000450 YES<br>RMS Force 0.000000 0.000300 YES<br>Maximum Displacement 0.000067 0.001800 YES<br>RMS Displacement 0.000012 0.001200 YES
	FILE:<br>Yb_Sq2_PhYbOTf3_GS_Anion_B3LYP_M062x_PhYbOTf3_Conf1.out	FILE:<br>Yb_Sq2_PhYbOTf3_GS_Anion_B3LYP_M062x_PhYbOTf3_Conf2.out	FILE:<br>Yb_Sq2_PhYbOTf3_GS_Anion_B3LYP_M062x_PhYbOTf3_Conf3.out

PhYb(OTf)$_2$	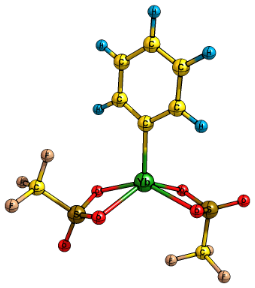	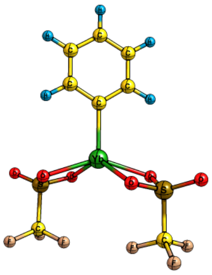	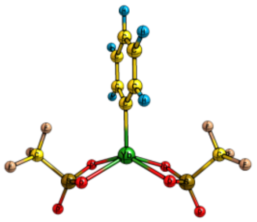
**PhYb[OTf]$_2$**	*Conformer 1*	*Conformer 2*	*Conformer 3*
Lowest energy conformation is: Conformer 3	E(RB3LYP/6-31G(d)/MWB59) = -2194.06304205<br>Convg = 0.8910D-08 -V/T = 2.0171<br><br>Temperature 195.150 Kelvin. Pressure 1.00000 Atm.<br>elec. and zero-point Energies= -2193.916280<br>elec. and thermal Energies= -2193.904200<br>elec. and thermal Enthalpies= -2193.903582<br>elec. and thermal Free Energies= -2193.950216<br><br>RB3LYP/6-31G(d)/MWB59/THF(IEFPCM) = -2194.09289422<br><br>RM06-2x/6-311+G(d,p)/MWB59/THF(IEFPCM) = -2193.95902765	E(RB3LYP/6-31G(d)/MWB59) = -2194.06304365<br>Convg = 0.5119D-08 -V/T = 2.0171<br><br>Temperature 195.150 Kelvin. Pressure 1.00000 Atm.<br>elec. and zero-point Energies= -2193.916257<br>elec. and thermal Energies= -2193.904169<br>elec. and thermal Enthalpies= -2193.903551<br>elec. and thermal Free Energies= -2193.950208<br><br>RB3LYP/6-31G(d)/MWB59/THF(IEFPCM) = -2194.09297527<br><br>RM06-2x/6-311+G(d,p)/MWB59/THF(IEFPCM) = -2193.95944018	E(RB3LYP/6-31G(d)/MWB59) = -2194.06228644<br>Convg = 0.5274D-08 -V/T = 2.0171<br><br>Temperature 195.150 Kelvin. Pressure 1.00000 Atm.<br>elec. and zero-point Energies= -2193.915675<br>elec. and thermal Energies= -2193.903541<br>elec. and thermal Enthalpies= -2193.902923<br>elec. and thermal Free Energies= -2193.950047<br><br>RB3LYP/6-31G(d)/MWB59/THF(IEFPCM) = -2194.09393825<br><br>RM06-2x/6-311+G(d,p)/MWB59/THF(IEFPCM) = -2193.96018966
	Geometry at RB3LYP/6-31G(d)/MWB59<br>Charge = 0 Multiplicity = 1<br>Yb 2.0814765686 0.5000154462 -1.1935081537<br>O 0.2131777204 0.2114093722 0.1271510073<br>S -0.151527229 1.6886936193 0.2489845808<br>O -1.5428253466 2.0559087349 0.0939457176<br>O 0.8770379088 2.3900645627 -0.6304799278<br>C 0.3922298426 2.1233067449 1.9818437896<br>F -0.3408220365 1.4492206345 2.8583353142<br>F 1.6818756638 1.7796410553 2.1138714961<br>F 0.256683408 3.4289491377 2.1738408571<br>O 3.6222565321 -0.8733237845 -0.1428651458<br>O 5.617139588 0.3354506065 0.8736317941<br>O 4.2643279025 1.2402908964 -1.0818772<br>S 4.8242538981 0.0505104122 -0.3048366781<br>C 5.9215458972 -0.826082148 -1.5375777997<br>F 6.3744939321 -1.9475555711 -0.9903708088<br>F 5.2092450604 -1.1113457458 -2.6304208838<br>F 6.9336697803 -0.0275035259 -1.8568778663<br>C 1.5728371873 -0.1885945425 -3.3296386184<br>C 2.457552986 -0.0936454695 -4.4286362872<br>C 0.2877004943 -0.7143480906 -3.6018882855<br>C 2.0883817601 -0.4942243113 -5.7148147395<br>C -0.092094613 -1.1223436957 -4.8836865003<br>C 0.810569789 -1.0108900763 -5.9428945556<br>H 3.4650518159 0.2992097648 -4.2871861196<br>H -0.4466513985 -0.8130162568 -2.8001662238<br>H 2.793702234 -0.4057601467 -6.537949806<br>H -1.0880442938 -1.5232580992 -5.0565761217<br>H 0.5198603089 -1.3245870437 -6.9423938815<br><br>Frequency: 16.1740; 19.7736; 22.4321; 24.9333; 28.1136; 35.0811; 41.6015; 46.2118; 49.4118; 63.8662; 102.3049; 119.0239; 165.0898; 165.7320; 178.9388; 197.3629; 207.9785; 223.3817; 233.0489; 250.1642; 260.9345; 303.4530; 305.6385; 318.5013; 321.5331; 369.1113; 369.7304; 391.1194; 436.9268; 491.0336; 491.7889; 534.4650; 538.4388; 556.7538; 558.3004; 579.4082; 584.8332; 623.8652; 630.7437; 634.3971; 660.2169; 717.1321; 738.7463; 765.3262; 766.7932; 872.0868; 925.1894; 941.3049; 950.7243; 972.8253; 1005.8095; 1010.1039; 1035.0089; 1041.4337; 1045.8947; 1087.4644; 1094.4997; 1189.0388; 1192.5855; 1194.2132; 1226.2603; 1249.8672; 1258.9296; 1281.8211; 1286.9475; 1299.3756; 1331.4387; 1340.8155; 1371.2817; 1460.7255; 1525.7224; 1617.3702; 1632.3510; 3129.0565; 3140.7213; 3178.5711; 3188.3619; 3203.5548.<br><br>Cartesian Forces: Max 0.000000702 RMS 0.000000188<br><br>Item Value Threshold Converged?<br>Maximum Force 0.000001 0.000450 YES<br>RMS Force 0.000000 0.000300 YES<br>Maximum Displacement 0.000164 0.001800 YES<br>RMS Displacement 0.000036 0.001200 YES	Geometry at RB3LYP/6-31G(d)/MWB59<br>Charge = 0 Multiplicity = 1<br>Yb 2.2112804861 0.3476995492 -1.308881179<br>O 0.6086974787 -0.2463364884 0.2523576388<br>S -0.0320987075 1.1303721852 0.3846195948<br>O -1.4759875257 1.2148389733 0.321366621<br>O 0.7729182811 2.0005292271 -0.5767198436<br>C 0.5118680295 1.7167563328 2.071883704<br>F -0.0127276881 0.9276906379 3.001198786<br>F 1.8487792322 1.6581497471 2.1339095351<br>F 0.1118313564 2.9693777613 2.2512222452<br>O 4.2850570184 1.3756461418 -1.3334246826<br>O 6.2876359975 -0.1611863275 -1.6703472517<br>O 4.0458208664 -0.9405237094 -0.7492929226<br>S 5.0955662919 0.1553107053 -0.9122188473<br>C 5.6067096081 0.5413596791 0.841745875<br>F 6.4164678092 1.5929031979 0.8417843386<br>F 4.5077327666 0.8194430621 1.55556793<br>F 6.2210569311 -0.5136400653 1.3621343137<br>C 1.5611385574 -0.2493615172 -3.4273053759<br>C 2.2627809349 -1.223624853 -4.1749780899<br>C 0.4375001744 0.3381979622 -4.0540179069<br>C 1.8706692452 -1.591156523 -5.4646872497<br>C 0.0375573285 -0.0201313398 -5.3439166402<br>C 0.7557135708 -0.9874598074 -6.050439885<br>H 3.1388139112 -1.7155456913 -3.7493107121<br>H -0.1474356109 1.0969410228 -3.5317347684<br>H 2.4323520468 -2.3443173417 -6.0122769094<br>H -0.8313203189 0.4515177463 -5.7968977283<br>H 0.4475698295 -1.2698545645 -7.0540583958<br><br>Frequency: 17.9616; 20.8108; 22.3964; 24.9601; 27.0073; 33.8346; 41.0065; 41.8124; 46.5416; 67.5917; 100.8062; 116.3886; 164.5560; 169.1237; 179.4974; 202.5169; 204.9051; 227.2112; 230.7773; 247.8356; 260.2748; 302.5711; 306.3857; 319.7734; 320.4811; 367.7841; 371.0324; 391.5985; 436.5581; 490.8692; 491.4639; 534.3906; 539.0807; 557.2290; 557.5036; 579.3376; 584.3700; 619.9077; 630.4406; 638.2202; 660.8841; 717.6960; 739.6407; 765.5928; 766.5216; 873.9753; 928.5155; 938.6321; 954.9018; 975.3328; 1006.8713; 1009.7604; 1037.0801; 1041.6382; 1046.9676; 1088.0233; 1094.0751; 1189.3970; 1194.1968; 1195.2737; 1225.9468; 1244.7391; 1258.7778; 1283.4025; 1286.0365; 1299.9880; 1333.0004; 1340.7339; 1370.7691; 1460.7476; 1525.0627; 1617.2645; 1631.9854; 3135.0032; 3135.3013; 3178.8242; 3188.8912; 3203.6450.<br><br>Cartesian Forces: Max 0.000000509 RMS 0.000000124<br><br>Item Value Threshold Converged?<br>Maximum Force 0.000000 0.000450 YES<br>RMS Force 0.000000 0.000300 YES<br>Maximum Displacement 0.000032 0.001800 YES<br>RMS Displacement 0.000009 0.001200 YES	Geometry at RB3LYP/6-31G(d)/MWB59<br>Charge = 0 Multiplicity = 1<br>Yb 1.9100300735 0.5221410241 -1.0645429478<br>O 0.8273553471 2.5108346963 -0.6121315235<br>S -0.3234461266 1.9103720772 0.1903791039<br>O -0.6291945325 2.5057815294 1.4747519557<br>O -0.0415642623 0.4094468707 0.1576936824<br>C -1.8165535417 2.1223442238 -0.914097565<br>F -2.0765093695 3.4185736551 -1.0399658248<br>F -1.554187319 1.5946404579 -2.110575659<br>F -2.8505239984 1.4970575326 -0.3622717298<br>O 3.2927722142 -0.9455734936 0.0603562531<br>O 5.3469564681 0.1033659809 1.1358189766<br>O 4.1308477256 1.1065743825 -0.8615027596<br>S 4.5707580902 -0.1211018686 -0.0661549383<br>C 5.629123845 -1.0905425794 -1.263773298<br>F 5.9712883372 -2.2424738502 -0.6984241776<br>F 4.9311995506 -1.3217125993 -2.3765545135<br>F 6.7133771837 -0.3788867189 -1.5510558341<br>C 1.502792665 -0.1885334959 -3.2178420692<br>C 2.1723557229 0.373963463 -4.3287573993<br>C 0.5689981014 -1.2117443259 -3.5006106271<br>C 1.9280661363 -0.0514629446 -5.6372435136<br>C 0.3181796776 -1.6496872229 -4.8037302106<br>C 0.9990607587 -1.0669056695 -5.8748884933<br>H 2.9094258601 1.1663388243 -4.1819335264<br>H 0.0134791636 -1.6875048059 -2.6895757393<br>H 2.460191865 0.4046892936 -6.4688038101<br>H -0.4059238614 -2.4406264089 -4.9848735237<br>H 0.8064769109 -1.4027239419 -6.890763715<br><br>Frequency: 14.1717; 18.1437; 22.2676; 23.1834; 23.8054; 28.0920; 39.3833; 40.9661; 58.0528; 60.3828; 98.0243; 124.2030; 164.7561; 165.5015; 171.3493; 188.0388; 213.4557; 216.7989; 232.6691; 260.8602; 262.6917; 303.5343; 303.8212; 318.4032; 319.0842; 368.3590; 370.8069; 389.6915; 438.7010; 491.2275; 492.3857; 534.2982; 539.1232; 558.3446; 558.5643; 576.8069; 586.1316; 623.2280; 631.1628; 636.2124; 660.2214; 717.1553; 737.6169; 766.5877; 767.1126; 871.6710; 923.6052; 939.3575; 950.8026; 971.3800; 1004.8832; 1010.1018; 1030.6822; 1041.3425; 1041.5345; 1088.5825; 1093.1700; 1191.0542; 1192.2668; 1193.9996; 1225.0791; 1261.6120; 1263.1047; 1280.1867; 1280.7420; 1298.9626; 1329.4778; 1338.2377; 1369.4902; 1460.4853; 1525.4144; 1617.6310; 1632.1646; 3121.1584; 3121.2779; 3178.3866; 3188.0533; 3203.5838.<br><br>Cartesian Forces: Max 0.000000609 RMS 0.000000189<br><br>Item Value Threshold Converged?<br>Maximum Force 0.000000 0.000450 YES<br>RMS Force 0.000000 0.000300 YES<br>Maximum Displacement 0.000337 0.001800 YES<br>RMS Displacement 0.000088 0.0012030 YES
	FILE: Yb_PhYbOTf2_GS_Neutral_B3LYP_M062x_Conf1.out	FILE: Yb_PhYbOTf2_GS_Neutral_B3LYP_M062x_Conf2.out	FILE: Yb_PhYbOTf2_GS_Neutral_B3LYP_M062x_Conf3.out

TfO$^{\ominus}$	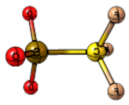	Ph$^{\ominus}$	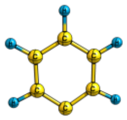
**Triflate**	*Conformer 1*	**Phenyl anion**	*Conformer 1*
	E(RB3LYP/6-31G(d)/MWB59) = -961.487307673<br>Convg = 0.9953D-08 -V/T = 2.0043<br><br>Temperature 195.150 Kelvin. Pressure 1.00000 Atm.<br>elec. and zero-point Energies= -961.460005<br>elec. and thermal Energies= -961.456382<br>elec. and thermal Enthalpies= -961.455764<br>elec. and thermal Free Energies= -961.479272<br><br>RB3LYP/6-31G(d)/MWB59/THF(IEFPCM) = -961.563646560<br><br>RM06-2x/6-311+G(d,p)/MWB59/THF(IEFPCM) = -961.583337085		E(RB3LYP/6-31G(d)/MWB59) = -231.565244846<br>Convg = 0.9069D-08 -V/T = 2.0061<br><br>Temperature 195.150 Kelvin. Pressure 1.00000 Atm.<br>elec. and zero-point Energies= -231.480157<br>elec. and thermal Energies= -231.477941<br>elec. and thermal Enthalpies= -231.477323<br>elec. and thermal Free Energies= -231.496815<br><br>RB3LYP/6-31G(d)/MWB59/THF(IEFPCM) = -231.647053503<br><br>RM06-2x/6-311+G(d,p)/MWB59/THF(IEFPCM) = -231.632849216
	Geometry at RB3LYP/6-31G(d)/MWB59<br>Charge = -1 Multiplicity = 1<br>O 0.5959629431 2.3842317308 -0.6469993396<br>S -0.1641982997 1.7702308575 0.4652908589<br>O -0.0891401629 2.4836551514 1.7604419135<br>O -0.0984153024 0.2932949162 0.5450042261<br>C -1.9311884549 2.0634498396 -0.0496327957<br>F -2.2053599243 3.3806721845 -0.1820820929<br>F -2.2134962795 1.480599924 -1.2363872596<br>F -2.8077175194 1.5669003959 0.8519944893<br><br>Frequency: 55.4118; 193.9591; 194.0530; 292.7772; 332.2917; 332.3049; 499.0742; 499.0925; 556.6455; 556.6764; 622.8454; 741.9516; 999.5414; 1181.6249; 1181.6523; 1229.2517; 1257.6564; 1257.6906.<br><br>Cartesian Forces: Max 0.000001181 RMS 0.000000724<br><br>Item Value Threshold Converged?<br>Maximum Force 0.000001 0.000450 YES<br>RMS Force 0.000000 0.000300 YES<br>Maximum Displacement 0.000006 0.001800 YES<br>RMS Displacement 0.000003 0.001200 YES		Geometry at RB3LYP/6-31G(d)/MWB59<br>Charge = -1 Multiplicity = 1<br>C -0.1922846253 0.0002627601 -4.9763210168<br>C -1.3636302763 -0.0005616157 -4.1662423067<br>C 0.9782573369 -0.000611493 -4.1650814423<br>C -1.3931413173 -0.0020326004 -2.7618998365<br>C 1.0063760393 -0.0020837059 -2.7607103286<br>C -0.1937401844 -0.0028140655 -2.039986674<br>H -2.3512764455 -0.0000252681 -4.6583416416<br>H 1.966390824 -0.0001172169 -4.6562015773<br>H -2.3472854081 -0.0025797446 -2.2206849972<br>H 1.9599831995 -0.0026714564 -2.2185500488<br>H -0.1942814108 -0.0039584215 -0.9480952601<br><br>Frequency: 345.1583; 407.9376; 597.3683; 635.6461; 686.3944; 728.4613; 845.9485; 864.5125; 942.3006; 959.7884; 965.7268; 1008.9743; 1049.1066; 1064.8436; 1164.0649; 1206.4025; 1265.1962; 1355.2382; 1446.0776; 1471.6563; 1589.1840; 1589.9319; 2965.0080; 2967.1588; 3049.5326; 3054.3699; 3123.2527.<br><br>Cartesian Forces: Max 0.000000021 RMS 0.000000008<br><br>Item Value Threshold Converged?<br>Maximum Force 0.000000 0.000450 YES<br>RMS Force 0.000000 0.000300 YES<br>Maximum Displacement 0.000000 0.001800 YES<br>RMS Displacement 0.000000 0.001200 YES
	FILE: Yb_OTf_GS_Anion_B3LYP_M062x_Conf1.out		FILE: Yb_Ph-Anion_GS_Anion_B3LYP_M062x_Conf1.out

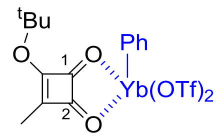

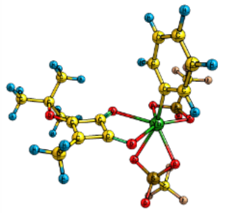

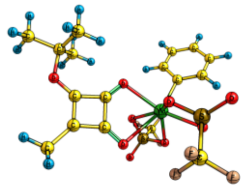

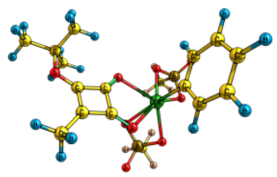

Pre-reaction complex 1b•PhYb(OTf)$_2$:- double chelation

Lowest energy conformation is: Conformer 2

Conformer 1

E(RB3LYP/6-31G(d)/MWB59) = -2769.90750985
Convg = 0.9369D-10 -V/T = 2.0149

Temperature 195.150 Kelvin. Pressure 1.00000 Atm.
elec. and zero-point Energies= -2769.564326
elec. and thermal Energies= -2769.544857
elec. and thermal Enthalpies= -2769.544239
elec. and thermal Free Energies= -2769.607389

RB3LYP/6-31G(d)/MWB59/THF(IEFPCM) = -2769.93373942

RM06-2x/6-311+G(d,p)/MWB59/THF(IEFPCM) = -2769.73876429

Geometry at RB3LYP/6-31G(d)/MWB59
Charge = 0 Multiplicity = 1

```
Yb -1.7261064657 1.5551549038 1.4991233286
O -2.273696621 -0.6393384877 2.4468073424
S -3.7255106257 -0.3011774215 2.7140116329
O -4.7278951222 -1.2699612546 2.3017090783
O -3.8987828069 1.1314195333 2.242553201
C -3.8245588295 -0.1707457651 4.5715946944
F -3.6256916649 -1.3758149424 5.1066279371
F -2.8827712844 0.6692683739 5.0096373541
F -5.0252864931 0.2834184664 4.9224601756
O 0.1670044701 2.0040669376 2.8043805608
O -0.2915232939 3.0126350883 5.0903032241
O -1.8404210621 3.2774224798 3.0922587534
S -0.4395004047 3.1136179017 3.6494788829
C 0.4724944508 4.6575157048 3.1307479788
F 1.7573281062 4.5308910482 3.4644166087
F 0.3702298286 4.8325585909 1.8142329887
F -0.0554031424 5.703548695 3.7631452687
C -0.273472328 -1.8933989895 -1.0939651658
C -1.5943625357 -1.8359589819 -1.5667533782
C -1.9162191672 -0.7106434356 -0.6856873674
C -0.5095670914 -0.7406671803 -0.1676661579
O 0.0574290919 -0.0409223658 0.6607358413
O -2.8389049277 0.0324923114 -0.3828269067
O 0.6824841999 -2.7043384231 -1.4320867207
C 2.0565903934 -2.6507866087 -0.8095251492
C -2.3589433906 -2.6191209732 -2.5623487454
H -1.7496536109 -3.4105452487 -3.0058295707
H -2.727355489 -1.9606326996 -3.3591380936
H -3.2430412592 -3.0660657961 -2.090505505
C -1.4193102548 2.8421392298 -0.4535849132
C -0.1448474456 3.1297663236 -0.9943563496
C -2.5171227682 3.3914972338 -1.1557665269
H 0.7515813978 2.7478969248 -0.5011820699
H -3.5344122765 3.2055900581 -0.8047631534
C 0.0291906738 3.9064319982 -2.1450914676
C -2.3626838968 4.1756221707 -2.303189611
H 1.0296577494 4.1059360863 -2.5250973821
H -3.2360499023 4.5834013644 -2.8084536243
C -1.0840179587 4.4338988216 -2.8025154711
H -0.9565406042 5.0423373993 -3.695051802
C 1.9253941571 -2.9131348657 0.6897805787
H 1.388314603 -3.8487523163 0.8755882566
H 2.9264981356 -3.0054738639 1.1238534868
H 1.4184190107 -2.0951769694 1.2085829258
C 2.6852839919 -1.2958459359 -1.1298524177
H 3.7271859264 -1.2981142677 -0.7927933356
H 2.6784731904 -1.1089108599 -2.2084920208
H 2.1767434938 -0.4757679583 -0.6160527741
C 2.7714502348 -3.7932344851 -1.526507951
H 3.8007640607 -3.8663748157 -1.1623958342
H 2.2692735407 -4.7468616304 -1.3372403186
H 2.7982260164 -3.6195351033 -2.6066033163
```

Frequency: 11.0861; 14.8636; 15.4743; 21.2339; 25.4027; 25.9381; 27.9779; 33.6387; 35.1315; 40.1872; 49.9233; 51.8436; 61.7436; 64.0824; 70.4298; 74.3708; 78.0497; 91.7916; 109.3018; 114.0376; 135.9451; 145.5731; 155.4918; 159.9154; 163.7852; 172.4779; 187.0604; 194.7296; 198.6204; 208.9908; 216.5706; 221.8579; 229.2714; 240.7338; 245.7193; 262.6854; 270.6005; 274.7048; 303.4317; 307.2489; 315.5402; 319.1934; 325.0879; 344.8807; 346.8234; 361.2552; 366.1122; 390.3974; 396.3434; 447.3033; 450.4927; 479.9099; 489.8024; 490.0128; 492.0147; 528.9525; 534.5979; 557.8687; 559.0211; 575.5088; 578.5594; 601.6758; 604.4620; 624.1569; 631.6940; 633.5845; 637.8266; 654.2076; 718.6317; 737.7296; 744.1747; 765.0181; 766.4716; 774.2057; 792.3099; 848.1564; 872.1241; 917.4566; 936.5431; 942.5048; 956.3780; 964.2044; 967.2636; 985.8155; 988.6306; 994.5841; 1011.0508; 1032.0931; 1039.0304; 1050.8329; 1060.7897; 1066.1440; 1072.2119; 1081.2021; 1087.4750; 1088.7046; 1128.3100; 1189.7516; 1193.2631; 1201.4396; 1204.2469; 1224.3533; 1243.9133; 1249.9653; 1258.7465; 1263.8207; 1265.8817; 1289.9830; 1294.1918; 1304.9911; 1317.4219; 1325.6431; 1368.6633; 1411.0807; 1436.5304; 1437.4129; 1449.8385; 1459.4983; 1463.4057; 1495.7685; 1503.8025; 1504.8726; 1514.9483; 1515.1767; 1523.8456; 1533.4757; 1534.9726; 1549.2548; 1616.1048; 1620.7330; 1630.8167; 1775.8306; 1865.1947; 3051.2251; 3066.0170; 3066.8627; 3073.3354; 3104.6666; 3119.6055; 3121.4422; 3134.6639; 3136.2449; 3143.7818; 3147.0013; 3152.7948; 3154.1355; 3157.4100; 3164.8203; 3174.6869; 3193.4354.

Cartesian Forces: Max 0.000000501 RMS 0.000000214

Item	Value	Threshold	Converged?
Maximum Force	0.000000	0.000450	YES
RMS Force	0.000000	0.000300	YES
Maximum Displacement	0.000013	0.001800	YES
RMS Displacement	0.000002	0.001200	YES

FILE: Yb_Sq2_PhYbOTf2_GS_Neutral_B3LYP_M062x_Conf1.out

Conformer 2

E(RB3LYP/6-31G(d)/MWB59) = -2769.91264662
Convg = 0.4303D-09 -V/T = 2.0149

Temperature 195.150 Kelvin. Pressure 1.00000 Atm.
elec. and zero-point Energies= -2769.569289
elec. and thermal Energies= -2769.549831
elec. and thermal Enthalpies= -2769.549213
elec. and thermal Free Energies= -2769.612002

RB3LYP/6-31G(d)/MWB59/THF(IEFPCM) = -2769.93670162

RM06-2x/6-311+G(d,p)/MWB59/THF(IEFPCM) = -2769.74036259

Geometry at RB3LYP/6-31G(d)/MWB59
Charge = 0 Multiplicity = 1

```
Yb -2.4872930771 1.0344836102 1.7692647243
O -1.143269173 2.408542755 0.3666136877
S -1.839871779 3.7179114262 0.6916988267
O -1.0068241142 4.8522618656 1.0466180683
O -2.9560716996 3.3300164596 1.6418606475
C -2.7243077344 4.1769643848 -0.8856285693
F -1.8267991612 4.3194758095 -1.8634088816
F -3.5893803419 3.215802287 -1.2186103379
F -3.3790316968 5.3214098339 -0.7074324498
O -3.3795835284 -1.1758826264 1.8824786663
O -5.821151939 -1.3787526913 1.2265082561
O -4.7843103124 0.7550840702 2.1422755087
S -4.8258944619 -0.759788025 2.085749048
C -5.2266370771 -1.2845488333 3.8312336042
F -5.1243214817 -2.6119931703 3.915801328
F -4.3784006364 -0.7169266525 4.6862505966
F -6.4716887212 -0.9112531807 4.1214778404
C -0.5351655141 -1.6578355304 -1.3751493542
C -1.6539456024 -1.3032461685 -2.1444682339
C -2.1852145839 -0.4464002444 -1.0788768455
C -1.000444735 -0.8151528052 -0.2288013086
O -0.6831899433 -0.4882969727 0.9064883045
O -3.1286984801 0.2901218368 -0.8348797903
O 0.4539487788 -2.440846105 -1.6822637376
C 1.5974649473 -2.7246550814 -0.7375217595
C -2.1139639147 -1.6642026966 -3.5039316048
H -1.4165624793 -2.3461284698 -3.9968080036
H -2.2280826948 -0.7618125948 -4.1176522297
H -3.1032069334 -2.1365798732 -3.4539889352
C -1.4596229489 1.091769454 3.8612606066
C -0.8631758958 -0.0591659371 4.4264409036
C -1.39535092 2.2666926184 4.6457551795
H -0.8811062943 -1.0030260608 3.8783149846
H -1.8360672441 3.1916452022 4.273379658
C -0.245007953 -0.0466587734 5.680024217
C -0.7811811754 2.2955583629 5.9018896935
H 0.2004347753 -0.9555148193 6.0806396689
H -0.7528523401 3.2207799257 6.4739112774
C -0.2031085654 1.1360742501 6.4220516944
H 0.2756804848 1.1531383742 7.3985802037
C 1.0415555843 -3.40921308 0.5097907906
H 0.4574867136 -4.2949225444 0.2399671264
H 1.8763768974 -3.732925238 1.140205557
H 0.4226535307 -2.7347611515 1.107352767
C 2.3183566518 -1.4125897153 -0.4335424378
H 3.2224747506 -1.6289819395 0.145123728
H 2.6210122055 -0.9115654794 -1.3586614163
H 1.7040510995 -0.7329909596 0.1630032018
C 2.4627041766 -3.6699494694 -1.5669984097
H 3.3447869707 -3.9605287258 -0.9883044009
H 1.9083077807 -4.5758402163 -1.8305627785
H 2.7984608054 -3.183906695 -2.4881288802
```

Frequency: 11.9768; 16.5629; 20.9323; 23.1875; 25.3580; 27.2268; 29.4898; 32.2287; 37.2163; 41.6883; 49.5950; 53.0113; 57.1362; 65.0009; 65.9478; 75.8550; 81.6745; 89.7596; 107.4373; 118.9731; 126.5580; 145.7433; 148.4102; 158.8551; 164.9586; 170.4976; 191.3621; 194.3506; 203.8684; 209.6391; 214.7095; 221.7334; 229.3823; 240.1653; 243.6926; 262.8915; 271.5948; 274.2188; 305.0829; 305.8928; 312.3252; 321.8405; 323.0721; 345.9947; 347.5739; 362.3506; 365.6145; 392.9493; 395.8898; 442.1603; 450.6798; 480.9783; 490.0227; 491.1680; 491.8621; 529.9031; 534.2084; 558.6560; 559.2113; 575.7401; 579.7071; 601.0403; 606.0421; 625.2160; 632.5391; 633.3796; 637.7260; 656.8255; 718.4309; 739.0671; 742.4778; 765.1387; 766.3282; 774.9634; 792.0826; 846.8081; 873.3257; 923.0188; 936.9942; 942.2173; 958.0262; 964.7054; 973.8643; 983.7968; 988.9184; 998.3480; 1011.2860; 1029.9033; 1040.2136; 1050.5822; 1065.8079; 1066.8145; 1071.8268; 1077.1532; 1088.4568; 1091.2092; 1121.8614; 1190.2802; 1192.6880; 1202.3781; 1204.6140; 1225.6138; 1243.4937; 1249.3186; 1258.7836; 1263.3028; 1264.6417; 1290.3589; 1296.7734; 1304.8831; 1318.6229; 1327.4051; 1369.9548; 1410.8768; 1436.5070; 1437.8429; 1449.5283; 1460.1886; 1463.5273; 1496.1875; 1503.9964; 1504.8105; 1515.0589; 1515.3520; 1524.2106; 1533.6754; 1534.9862; 1549.3423; 1616.3992; 1622.2777; 1631.7122; 1779.4986; 1865.8366; 3051.1448; 3066.1273; 3067.1379; 3073.4938; 3104.2497; 3125.2582; 3134.8400; 3136.3350; 3143.7653; 3147.1396; 3147.9813; 3152.4064; 3154.6461; 3157.9932; 3166.1473; 3176.9626; 3194.0484.

Cartesian Forces: Max 0.000000911 RMS 0.000000329

Item	Value	Threshold	Converged?
Maximum Force	0.000000	0.000450	YES
RMS Force	0.000000	0.000300	YES
Maximum Displacement	0.000035	0.001800	YES
RMS Displacement	0.000005	0.001200	YES

FILE: Yb_Sq2_PhYbOTf2_GS_Neutral_B3LYP_M062x_Conf2.out

Conformer 3

E(RB3LYP/6-31G(d)/MWB59) = -2769.90770609
Convg = 0.8045D-08 -V/T = 2.0149

Temperature 195.150 Kelvin. Pressure 1.00000 Atm.
elec. and zero-point Energies= -2769.564288
elec. and thermal Energies= -2769.544888
elec. and thermal Enthalpies= -2769.544270
elec. and thermal Free Energies= -2769.606706

RB3LYP/6-31G(d)/MWB59/THF(IEFPCM) = -2769.93448845

RM06-2x/6-311+G(d,p)/MWB59/THF(IEFPCM) = -2769.73955260

Geometry at RB3LYP/6-31G(d)/MWB59
Charge = 0 Multiplicity = 1

```
Yb -1.7580759315 1.5933987362 1.4604679829
O -2.4037452157 -0.5619975131 2.4653522631
S -3.8732054198 -0.2114863266 2.5748069043
O -4.8288297787 -1.1712002945 2.0444779505
O -3.9812412901 1.2241048961 2.0978505486
C -4.2008898854 -0.1063587867 4.407680338
F -3.9986360867 -1.3059305149 4.9557228342
F -3.3768464477 0.7803681505 4.9706073403
F -5.461827822 0.2707249937 4.6080134139
O -1.8228497138 3.15989342 3.2066775002
O 0.2947283739 4.5239183008 3.5746646964
O 0.2616697391 2.2172544625 2.5011715541
S -0.3318286619 3.218882142 3.4760947347
C -0.126658136 2.3946297644 5.1371419818
F -0.8026505849 3.0750596096 6.0593204862
F -0.5927795515 1.1415952892 5.077176679
F 1.1668407581 2.3659577251 5.4555513976
C -0.2423239649 -1.9400225838 -1.0116412344
C -1.5464766119 -1.8831991828 -1.5313201433
C -1.8866897105 -0.7346108727 -0.6877676629
C -0.503529322 -0.7676328149 -0.117419477
O 0.0319381032 -0.0518165196 0.7183017345
O -2.8057638684 0.0309931257 -0.4334917566
O 0.719476475 -2.7623102716 -1.3004263607
C 2.07570236 -2.6951696876 -0.6393900771
C -2.2821330358 -2.6826545604 -2.5356134098
H -1.6629602288 -3.4858508463 -2.9427315722
H -2.6208465578 -2.0387832318 -3.3571221325
H -3.1836556391 -3.1156308145 -2.0840794538
C -1.5924754491 2.9767002549 -0.431993169
C -0.4219998395 3.7359843395 -0.6721058546
C -2.6396657167 3.154196445 -1.366865137
H 0.4250863594 3.6584833725 0.0109573383
H -3.5707862008 2.5992824676 -1.2461311289
C -0.2995121648 4.604898016 -1.7619138108
C -2.5362891459 4.0248005873 -2.4555321705
H 0.6183798286 5.1710387377 -1.908293746
H -3.3696013178 4.1372859036 -3.1466437713
C -1.3605764567 4.7522005704 -2.65689124
H -1.2740583117 5.4307194126 -3.5026941066
C 1.8998309846 -2.9261954428 0.8604360179
H 1.3572300773 -3.8578921236 1.0498926663
H 2.8876004047 -3.0087528295 1.3258888064
H 1.3778511557 -2.0973387663 1.3461133055
C 2.7136912372 -1.3468621797 -0.9690865573
H 3.7460829692 -1.3429052023 -0.6040100923
H 2.7368274118 -1.1812813566 -2.0509668114
H 2.1931562014 -0.5159334318 -0.4854372822
C 2.8104572468 -3.8522143339 -1.3112289954
H 3.8284412088 -3.9179901885 -0.915273394
H 2.3021435247 -4.801715631 -1.1176033442
H 2.869608648 -3.7005454147 -2.3933255829
```

Frequency: 10.0421; 15.0996; 21.7355; 24.6406; 26.5613; 28.7233; 30.9601; 35.4166; 40.8486; 49.0889; 51.6817; 54.0663; 60.4486; 67.6291; 71.0207; 76.5125; 81.3041; 91.7922; 108.4993; 117.6918; 131.8396; 143.9920; 148.3919; 153.6822; 160.5509; 173.6732; 193.7783; 195.7603; 199.8736; 209.7180; 214.9992; 220.4519; 228.8316; 237.3232; 243.6592; 263.4552; 271.1979; 274.3997; 304.4325; 306.4980; 315.6877; 320.9788; 324.4234; 345.2132; 347.1236; 359.5302; 367.3252; 393.1629; 396.6898; 442.4841; 450.4201; 480.4257; 489.9061; 490.0243; 490.9686; 528.3111; 534.1788; 558.2032; 558.4434; 573.3357; 579.9860; 602.1768; 604.3486; 621.1778; 632.8217; 634.8930; 638.1141; 654.9129; 719.6446; 739.1376; 744.5185; 764.7419; 765.6996; 773.8085; 792.3052; 847.6370; 875.0661; 925.1270; 936.5662; 942.5996; 955.3750; 968.5493; 974.8563; 986.0654; 988.8216; 997.7112; 1010.7799; 1033.2361; 1038.7156; 1050.5120; 1066.1313; 1067.1624; 1072.3498; 1081.9324; 1086.5686; 1091.1795; 1131.8213; 1190.2361; 1192.8801; 1202.7712; 1206.6447; 1226.3908; 1241.1518; 1242.9601; 1253.6933; 1264.2102; 1268.0722; 1290.0793; 1294.7605; 1305.2208; 1314.0639; 1329.8758; 1370.8922; 1411.1414; 1436.6512; 1437.5646; 1449.6265; 1459.5591; 1463.5170; 1495.5105; 1503.9025; 1504.9105; 1514.9305; 1515.1873; 1524.0237; 1533.5129; 1535.1444; 1549.2666; 1615.5740; 1620.3513; 1630.9122; 1775.7449; 1866.5016; 3051.2455; 3066.1511; 3066.8403; 3073.3637; 3104.6896; 3134.8331; 3136.3915; 3138.1401; 3139.5613; 3143.9734; 3147.1561; 3152.9157; 3153.5769; 3156.8705; 3166.5732; 3175.9534; 3193.3221.

Cartesian Forces: Max 0.000000664 RMS 0.000000226

Item	Value	Threshold	Converged?
Maximum Force	0.000000	0.000450	YES
RMS Force	0.000000	0.000300	YES
Maximum Displacement	0.000021	0.001800	YES
RMS Displacement	0.000004	0.001200	YES

FILE: Yb_Sq2_PhYbOTf2_GS_Neutral_B3LYP_M062x_Conf3.out

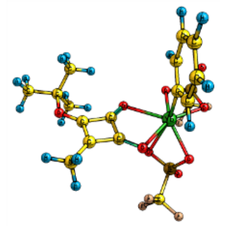

Conformer 4

E(RB3LYP/6-31G(d)/MWB59) = -2769.90417050
Convg = 0.9994D-08 -V/T = 2.0149

Temperature 195.150 Kelvin. Pressure 1.00000 Atm.
elec. and zero-point Energies= -2769.560875
elec. and thermal Energies= -2769.541412
elec. and thermal Enthalpies= -2769.540794
elec. and thermal Free Energies= -2769.604006

RB3LYP/6-31G(d)/MWB59/THF(IEFPCM) = -2769.93164791

RM06-2x/6-311+G(d,p)/MWB59/THF(IEFPCM) = -2769.73674881

Geometry at RB3LYP/6-31G(d)/MWB59
Charge = 0 Multiplicity = 1

```
Yb -1.8629928723  1.48673  1.4479554548
O  -4.0590884324  1.180984418  2.2238042281
S  -3.8849136672 -0.1475507465  2.9372239867
O  -4.2083726176 -0.1861066512  4.3541759482
O  -2.5190988081 -0.6469847508  2.5198178094
C  -5.1118011728 -1.3006936716  2.1302793274
F  -6.3457006601 -0.8727681923  2.3892044801
F  -4.9333730742 -1.3540047157  0.8078044743
F  -4.9443976226 -2.5241161221  2.6394126927
O  -1.938997137  3.2514270052  2.9919620786
O   0.2868152556  4.3464722509  3.5718123971
O   0.0618275246  1.9727119914  2.6890056631
S  -0.5103358799  3.1371000319  3.4835251678
C  -0.677437419  2.4580480623  5.2128773091
F  -1.300230086  3.3486710815  5.9797048165
F  -1.3847392884  1.325164272  5.1759689557
F   0.5351425809  2.2073762265  5.7059578688
C  -0.2557893905 -1.8845285349 -1.1673943263
C  -1.5499230409 -1.7865260053 -1.7067824707
C  -1.9192321722 -0.732352552 -0.7615706228
C  -0.5476175754 -0.8063996335 -0.1670626374
O  -0.0372072469 -0.1682701824  0.7413280488
O  -2.8529730988 -0.0019155523 -0.4549548678
O   0.7201716684 -2.6637605466 -1.5160878937
C   2.0682161296 -2.6413220039 -0.8306874097
C  -2.2500965108 -2.4810265454 -2.810534953
H  -1.6154052683 -3.2410138004 -3.2730041745
H  -2.5576500813 -1.7578383578 -3.5765043459
H  -3.1670058954 -2.9554749972 -2.4393032844
C  -1.4690566171  2.7926345175 -0.4768808682
C  -0.2390572741  3.4774140285 -0.6261623105
C  -2.4168496716  3.0123941904 -1.5037823366
H   0.5390454443  3.3675478249  0.1310557024
H  -3.3874997872  2.5163300133 -1.4574924922
C   0.0310229842  4.3138778306 -1.7148412111
C  -2.1674325483  3.8523341883 -2.5932332234
H   0.9906096422  4.8221175529 -1.7879684241
H  -2.9298760582  3.999933411 -3.3559887827
C  -0.9369163357  4.5046532455 -2.7024516363
H  -0.7363474851  5.1590498069 -3.5478691059
C   1.8744734841 -3.0142141609  0.6378236083
H   1.3432871293 -3.966869991  0.7313306264
H   2.8568657333 -3.1272061911  1.1081728306
H   1.334826002 -2.2411249338  1.1914202821
C   2.6894105597 -1.2593613443 -1.0234941941
H   3.7168576523 -1.2757218549 -0.6450544972
H   2.7238546277 -0.9920807551 -2.0844788115
H   2.1503687706 -0.4849891296 -0.4712263932
C   2.8276658921 -3.7203347247 -1.5977295254
H   3.8411645564 -3.8095864524 -1.1949699125
H   2.3308281401 -4.6905508853 -1.5013361365
H   2.8990320177 -3.4666829646 -2.6597849093
```

Frequency: 8.5569; 12.9442; 15.7597; 23.8396; 24.6976; 26.0406; 30.9338; 32.4701; 37.7336; 39.9393; 49.3518; 51.3755; 59.8533; 65.9730; 67.1425; 77.8345; 88.1082; 96.1537; 115.0900; 117.3045; 132.5929; 141.3375; 145.4158; 157.0928; 159.9984; 166.1477; 191.2846; 198.7654; 202.4137; 208.6320; 212.0996; 220.2631; 235.2575; 238.5342; 243.0068; 263.4150; 271.6682; 274.5606; 305.2611; 307.2893; 316.3355; 319.8506; 323.2110; 343.8091; 345.6199; 358.3369; 366.8491; 392.7923; 396.9745; 444.6269; 450.0318; 478.1889; 489.3240; 489.5075; 491.1451; 527.4215; 534.3074; 558.1104; 558.4721; 569.7759; 580.4111; 601.2954; 604.4895; 627.1819; 632.3173; 633.4373; 636.6277; 653.9752; 719.8827; 738.6960; 744.3339; 764.8367; 765.5421; 772.0718; 792.3852; 845.4126; 874.7846; 923.1287; 936.5728; 942.5940; 957.2933; 966.1468; 973.1850; 985.7510; 989.1232; 996.6753; 1010.8576; 1033.1816; 1038.3561; 1050.9907; 1063.2005; 1066.4392; 1072.3551; 1083.5481; 1086.1331; 1090.6662; 1130.8681; 1189.9143; 1191.5388; 1202.1278; 1206.5716; 1225.8805; 1243.8242; 1247.2124; 1250.7515; 1260.8201; 1268.1997; 1290.4941; 1294.0451; 1305.4779; 1315.3446; 1328.7954; 1370.0969; 1412.3179; 1436.8106; 1437.8554; 1451.1901; 1459.5297; 1463.7608; 1496.4216; 1503.7491; 1505.2847; 1514.9972; 1515.1211; 1523.9599; 1533.6131; 1535.1001; 1549.2707; 1615.5359; 1620.2835; 1630.9175; 1779.0017; 1866.1338; 3051.6344; 3066.2417; 3067.1226; 3073.5000; 3105.1064; 3133.2804; 3133.5055; 3135.0624; 3136.5922; 3144.0382; 3147.4180; 3152.8131; 3153.9486; 3157.3036; 3165.7519; 3175.1550; 3193.1125.

Cartesian Forces: Max 0.000000996 RMS 0.000000456

Item	Value	Threshold	Converged?
Maximum Force	0.000001	0.000450	YES
RMS Force	0.000000	0.000300	YES
Maximum Displacement	0.000052	0.001800	YES
RMS Displacement	0.000013	0.001200	YES

FILE: Yb_Sq2_PhYbOTf2_GS_Neutral_B3LYP_M062x_Conf4.out

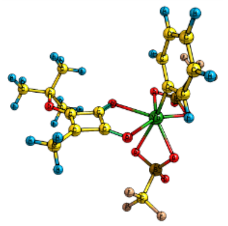

Conformer 5

E(RB3LYP/6-31G(d)/MWB59) = -2769.90241877
Convg = 0.5975D-08 -V/T = 2.0149

Temperature 195.150 Kelvin. Pressure 1.00000 Atm.
elec. and zero-point Energies= -2769.559269
elec. and thermal Energies= -2769.539748
elec. and thermal Enthalpies= -2769.539130
elec. and thermal Free Energies= -2769.602844

RB3LYP/6-31G(d)/MWB59/THF(IEFPCM) = -2769.93046499

RM06-2x/6-311+G(d,p)/MWB59/THF(IEFPCM) = -2769.73474630

Geometry at RB3LYP/6-31G(d)/MWB59
Charge = 0 Multiplicity = 1

```
Yb -1.6951924952  1.4729241344  1.372328637
O  -3.9091210479  1.1428961735  2.0759104732
S  -3.7367672881 -0.174331029  2.8100495161
O  -4.092914588 -0.2055134613  4.2183832081
O  -2.3557639243 -0.6628745134  2.4258991403
C  -4.9295023279 -1.3516944057  1.9862450197
F  -6.1718179425 -0.9050078584  2.1590801942
F  -4.6811870568 -1.4633186968  0.6776335183
F  -4.8012804308 -2.5539988255  2.5531069513
O   0.3203730699  2.0383126376  2.4418552659
O   0.1292675706  2.6296825784  4.903280433
O  -1.7626669785  2.9121322629  3.2263300782
S  -0.278298481  2.9057602157  3.5376202756
C   0.3021164726  4.6380398501  3.1535887087
F   1.634828294  4.6660440416  3.1998491079
F  -0.1086716024  5.0042610802  1.9403828176
F  -0.1966238789  5.4719578513  4.0638038502
C  -0.1338566947 -1.9638604555 -1.21433004
C  -1.4235177958 -1.8559837772 -1.7636330082
C  -1.7705432453 -0.7566827536 -0.8600616044
C  -0.4017514112 -0.8359916198 -0.2650503444
O   0.1193469457 -0.1639551963  0.6136915765
O  -2.6839581759  0.0062495715 -0.5758281033
O   0.8227554401 -2.7835590388 -1.5225388203
C   2.1590348792 -2.7810974831 -0.8142568808
C  -2.1384119019 -2.5804935328 -2.8379574509
H  -1.5252762299 -3.3807466047 -3.2597944831
H  -2.420288007 -1.8857859283 -3.6394525065
H  -3.0714495786 -3.0075724948 -2.4497654952
C  -1.5255057488  2.9068916561 -0.4893675498
C  -0.2814138913  3.4154234067 -0.9308612
C  -2.651359439  3.3044315034 -1.2478437092
H   0.6308108897  3.1624342816 -0.3873864809
H  -3.6443197659  2.9449131232 -0.971667588
C  -0.1623983442  4.2548125419 -2.0440438002
C  -2.5526278769  4.1518390404 -2.3552887999
H   0.8150892626  4.6242938651 -2.3489422751
H  -3.4466607434  4.4401540601 -2.904911829
C  -1.3025135799  4.627840679 -2.7583472581
H  -1.2185051424  5.2858181792 -3.620421074
C   1.9282229826 -3.0898214147  0.6637712544
H   1.3617787661 -4.0188031193  0.784143566
H   2.8980552612 -3.219062684  1.1554607801
H   1.4078587582 -2.2778487958  1.17888596
C   2.8315208359 -1.4298349577 -1.0484169096
H   3.851108308 -1.4666245642 -0.6507055394
H   2.8933962593 -1.2056215664 -2.1181604915
H   2.3104585072 -0.6169739137 -0.5355124502
C   2.8930320905 -3.9143879574 -1.5257662925
H   3.8959895513 -4.0225638822 -1.1018291356
H   2.3611450373 -4.8625001733 -1.4009913002
H   2.9906244325 -3.7050030303 -2.5953829122
```

Frequency: 7.0026; 11.6557; 14.9758; 19.1374; 22.4613; 28.2502; 29.5890; 33.2849; 36.3334; 41.2257; 47.5677; 50.8588; 60.0436; 66.4621; 69.2632; 75.2218; 88.6774; 93.8390; 110.8034; 118.4380; 131.3440; 139.4670; 145.8443; 159.1108; 159.7145; 164.0628; 178.4907; 197.0688; 201.8205; 209.9814; 212.6392; 219.7592; 233.9217; 235.9108; 244.2664; 264.0157; 272.0841; 274.2812; 304.5402; 307.1189; 315.2005; 319.6733; 323.4096; 344.6175; 346.0697; 358.8751; 365.6992; 391.9628; 396.9634; 445.2727; 450.0893; 479.7070; 488.7812; 489.5056; 491.8818; 526.8963; 534.8548; 557.9079; 558.9179; 568.9653; 580.7314; 601.4419; 604.9114; 625.0207; 633.2781; 635.8722; 637.4764; 654.6957; 719.3825; 738.4639; 743.8262; 764.3423; 766.5997; 773.7812; 792.2767; 845.6119; 873.5435; 920.3831; 936.4699; 942.4963; 957.3254; 966.1800; 969.9662; 985.8362; 989.0959; 995.4789; 1011.0943; 1033.7986; 1038.8626; 1050.4737; 1062.8849; 1065.9780; 1072.4615; 1079.0442; 1087.0124; 1089.3399; 1133.6549; 1189.8756; 1191.5247; 1201.7577; 1206.7023; 1224.7925; 1241.2847; 1244.7545; 1257.8482; 1260.7168; 1263.4895; 1290.4218; 1294.4966; 1305.4466; 1316.0127; 1327.5086; 1369.2281; 1411.5747; 1436.8525; 1437.7553; 1450.2542; 1459.4616; 1463.7056; 1495.8528; 1503.8295; 1505.1642; 1514.7951; 1515.1479; 1523.5656; 1533.5670; 1535.1458; 1549.1047; 1615.7113; 1620.1511; 1630.6943; 1777.8039; 1866.5426; 3051.3313; 3066.2642; 3067.0860; 3073.5056; 3104.7373; 3127.2194; 3128.2251; 3135.1907; 3136.7852; 3144.0601; 3147.5227; 3152.8540; 3153.4508; 3156.7877; 3165.3230; 3174.6392; 3193.1575.

Cartesian Forces: Max 0.000001021 RMS 0.000000319

Item	Value	Threshold	Converged?
Maximum Force	0.000000	0.000450	YES
RMS Force	0.000000	0.000300	YES
Maximum Displacement	0.000029	0.001800	YES
RMS Displacement	0.000005	0.001200	YES

FILE: Yb_Sq2_PhYbOTf2_GS_Neutral_B3LYP_M062x_Conf5.out

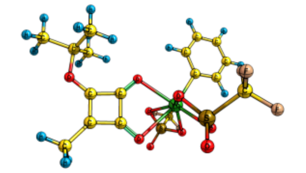

Conformer 6

E(RB3LYP/6-31G(d)/MWB59) = -2769.91347306
Convg = 0.9344D-09 -V/T = 2.0149

Temperature 195.150 Kelvin. Pressure 1.00000 Atm.
elec. and zero-point Energies= -2769.570197
elec. and thermal Energies= -2769.550734
elec. and thermal Enthalpies= -2769.550116
elec. and thermal Free Energies= -2769.613111

RB3LYP/6-31G(d)/MWB59/THF(IEFPCM) = -2769.93629748

RM06-2x/6-311+G(d,p)/MWB59/THF(IEFPCM) = -2769.73957634

Geometry at RB3LYP/6-31G(d)/MWB59
Charge = 0 Multiplicity = 1

```
Yb -2.4241348646  0.9905643498  1.8644617232
O  -2.9315147964  3.232780323  1.4106993361
S  -1.7311868766  3.5501360134  0.5394425055
O  -1.9759879612  4.1875261287 -0.743042183
O  -0.9128627423  2.2710180836  0.5352575342
C  -0.718096495  4.7511371533  1.5465258835
F  -1.4138965482  5.8747470025  1.7116036832
F  -0.4296142382  4.2244115708  2.735199243
F   0.4128986341  5.0152723836  0.890339726
O  -3.3922901744 -1.1894805815  1.8682237629
O  -5.8760308624 -1.2549064714  1.3542325035
O  -4.7017579467  0.7615805117  2.3634089358
S  -4.806493333 -0.742025713  2.1932496714
C  -5.1200973497 -1.3930966516  3.9138306519
F  -5.1278049527 -2.726456979  3.8742527348
F  -4.163648901 -0.9785083188  4.7426277218
F  -6.302557957 -0.9499960014  4.3373111407
C  -0.542839871 -1.5919218125 -1.3794826323
C  -1.6190647542 -1.1166164881 -2.1437654013
C  -2.1361311148 -0.3225064757 -1.025099474
C  -0.9915097296 -0.8165871602 -0.1806578743
O  -0.6819789387 -0.594551737  0.9825542961
O  -3.0523418175  0.4364158674 -0.747119204
O   0.4108348946 -2.4024635678 -1.7267868413
C   1.5096638962 -2.8259417861 -0.7837301577
C  -2.0577798825 -1.335276738 -3.5397170449
H  -1.3869580672 -2.0149030565 -4.0710629849
H  -2.0997947849 -0.3787842812 -4.0759120449
H  -3.0744397683 -1.7479665916 -3.5551760651
C  -1.4549802371  1.1150379987  3.9860110862
C  -0.2835051235  0.4145761964  4.3540209702
C  -2.0312657373  1.9209904126  4.9953257968
H   0.2154047704 -0.2269341713  3.6257970266
H  -2.9378346923  2.4916190845  4.783990679
C   0.2765732671  0.5068272812  5.631777081
C  -1.4845032113  2.0246342533  6.277916189
H   1.1802019684 -0.0491770165  5.8751304995
H  -1.959697944  2.6564467372  7.0256741601
C  -0.3254027001  1.3148449131  6.5986862191
H   0.105361467  1.3908738458  7.5945165881
C   0.8833614276 -3.5727733583  0.392702239
H   0.258901863 -4.399916237  0.0401902636
H   1.6814253513 -3.9921915022  1.0142953183
H   0.2855976447 -2.9132925813  1.0274163107
C   2.2959596177 -1.5863423336 -0.3604761436
H   3.1696874351 -1.8997984326  0.2205089727
H   2.652130222 -1.034018091 -1.235951288
H   1.7038545415 -0.91939699  0.2717084821
C   2.3422529074 -3.7525307088 -1.6662373607
H   3.1907200774 -4.1387021355 -1.0931741462
H   1.7446568462 -4.6009500085 -2.0133585367
H   2.7292555416 -3.2163841326 -2.5381355524
```

Frequency: 10.9662; 15.1825; 21.8569; 22.0910; 26.1439; 26.8843; 28.6830; 29.7962; 31.0589; 41.5495; 50.9054; 51.8700; 61.3934; 65.3757; 67.6752; 72.4933; 82.8330; 90.6264; 106.3476; 119.5936; 128.6643; 146.0785; 156.1964; 156.7747; 165.2718; 170.9396; 186.4138; 195.6045; 200.5689; 209.1291; 215.1868; 222.2676; 229.3973; 243.4132; 243.8464; 263.0089; 271.1785; 275.1755; 304.5892; 305.8067; 313.8488; 322.5047; 323.4235; 345.9751; 346.6788; 362.8764; 365.8360; 391.1463; 395.8520; 443.1443; 450.8711; 481.3880; 489.8731; 491.5028; 492.0074; 529.4241; 534.7760; 559.0681; 559.2717; 574.5090; 581.3585; 601.2645; 605.6748; 624.3742; 633.0779; 633.3763; 637.9568; 656.5669; 718.2756; 738.4093; 742.9629; 766.1270; 766.3550; 773.9315; 792.0146; 848.2971; 871.7730; 920.1903; 936.3194; 942.1972; 957.1779; 965.3053; 968.9308; 984.8097; 988.5776; 996.5369; 1011.1378; 1028.9566; 1040.3860; 1050.4100; 1064.9377; 1066.6780; 1071.6494; 1076.7669; 1088.2738; 1090.6348; 1120.6532; 1190.4054; 1193.5673; 1202.1746; 1203.0502; 1225.0498; 1244.9312; 1257.5612; 1259.0876; 1262.5030; 1263.7672; 1290.0128; 1295.8594; 1304.6644; 1317.2470; 1324.4541; 1369.3753; 1410.8717; 1436.4875; 1437.3493; 1449.2963; 1460.0497; 1463.2739; 1495.6100; 1503.9509; 1504.5573; 1515.1594; 1515.5408; 1524.4224; 1533.7492; 1534.9396; 1549.4463; 1616.6457; 1621.5006; 1631.9617; 1776.8217; 1864.3323; 3051.2257; 3066.1439; 3067.0393; 3073.4883; 3104.4494; 3124.9938; 3130.9927; 3134.6442; 3136.1890; 3143.9528; 3146.9913; 3152.9827; 3154.5700; 3157.7811; 3166.1577; 3175.9574; 3194.1820.

Cartesian Forces: Max 0.000000817 RMS 0.000000314

Item	Value	Threshold	Converged?
Maximum Force	0.000000	0.000450	YES
RMS Force	0.000000	0.000300	YES
Maximum Displacement	0.000049	0.001800	YES
RMS Displacement	0.000008	0.001200	YES

FILE: Yb_Sq2_PhYbOTf2_GS_Neutral_B3LYP_M062x_Conf6.out

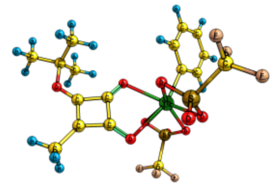

Conformer 7

E(RB3LYP/6-31G(d)/MWB59) = -2769.91263941
Convg = 0.2109D-09 -V/T = 2.0149

Temperature 195.150 Kelvin. Pressure 1.00000 Atm.
elec. and zero-point Energies= -2769.569289
elec. and thermal Energies= -2769.549829
elec. and thermal Enthalpies= -2769.549211
elec. and thermal Free Energies= -2769.611999

RB3LYP/6-31G(d)/MWB59/THF(IEFPCM) = -2769.93648003

RM06-2x/6-311+G(d,p)/MWB59/THF(IEFPCM) = -2769.74008729

Geometry at RB3LYP/6-31G(d)/MWB59
Charge = 0 Multiplicity = 1

```
Yb -2.4818811032  0.9936859918  1.7681451786
O  -2.9944128165  3.2021685942  1.1730358124
S  -1.7208433342  3.5181402975  0.4128553707
O  -1.8495114165  4.1002857547 -0.912479291
O  -0.8771656947  2.2616858391  0.5388630441
C  -0.8443190514  4.7829730877  1.4688070817
F  -1.5446928639  5.9157415627  1.4597606971
F  -0.730333436  4.337253368  2.7182255145
F   0.3695480693  5.0012083361  0.9602013125
O  -4.6858698412  0.8706586856  2.5627996429
O  -5.4484968006 -1.3925524689  3.4388888004
O  -3.6130615005 -1.0988706541  1.7075219957
S  -4.9043318223 -0.6134952034  2.3417904163
C  -6.1566490435 -0.6876880681  0.9607809924
F  -7.3375212706 -0.2743213272  1.4134050714
F  -5.7676435841  0.0948199676 -0.0488767823
F  -6.2559261094 -1.9468342333  0.5288621196
C  -0.436092131 -1.6027583482 -1.3970326436
C  -1.4254612744 -1.0754142349 -2.2410299549
C  -2.0210060818 -0.297134821 -1.1495405603
C  -0.9682038538 -0.8411675251 -0.2232960523
O  -0.7570538441 -0.6503041497  0.9663818948
O  -2.9400949091  0.476110728 -0.9268257976
O   0.521103713 -2.4336946091 -1.6782024149
C   1.519142764 -2.911219886 -0.6502721208
C  -1.7360349584 -1.2418941524 -3.6783502215
H  -1.0392180542 -1.9299689909 -4.1634438565
H  -1.6948307941 -0.2707562318 -4.1876855092
H  -2.7588595382 -1.6187244469 -3.8041258202
C  -1.450614399  1.0791724881  3.8572955845
C  -0.0429453995  1.0579640993  3.9908017774
C  -2.1823394463  1.1602148415  5.0647815803
H   0.5847229786  1.002204423  3.0994156807
H  -3.2719510274  1.1823028477  5.0377312614
C   0.5950668756  1.111628226  5.2333025739
C  -1.5591465297  1.215304354  6.3155892053
H   1.6818743213  1.0953841228  5.2926257851
H  -2.1589073967  1.2767898888  7.2214324298
C  -0.1659251473  1.1904759524  6.4020157771
H   0.3236831874  1.2331501631  7.372377078
C   0.768899952 -3.6604756148  0.4494310173
H   0.1532552542 -4.4601349543  0.0251793812
H   1.4962888871 -4.117736772  1.1283431552
H   0.139076042 -2.995221968  1.0459739792
C   2.30041248 -1.7052323072 -0.1318936493
H   3.1098726407 -2.0571809102  0.5162068059
H   2.7481270737 -1.1463936126 -0.9599496085
H   1.6741698657 -1.0341953086  0.4618618217
C   2.3985640175 -3.8456955898 -1.4770570588
H   3.1812060832 -4.2694771745 -0.8404425141
H   1.8093006712 -4.6681030599 -1.8940369991
H   2.8768855972 -3.3049519969 -2.2994099844
```

Frequency: 12.0612; 16.6162; 21.4244; 23.3689; 25.0123; 26.5550; 29.4819; 32.0704; 37.2082; 41.3674; 49.7498; 53.3210; 57.4145; 65.4057; 65.9233; 75.7905; 81.5252; 89.9167; 107.5870; 119.1859; 126.4950; 145.5617; 148.4099; 158.8970; 164.9106; 170.3713; 191.3635; 194.4152; 203.8405; 209.0953; 214.7148; 221.6604; 229.3259; 240.0010; 243.6237; 262.4569; 271.0144; 274.0219; 304.9974; 305.8114; 312.3701; 321.6659; 323.0516; 345.6874; 347.6627; 362.3174; 365.5986; 393.0417; 396.2026; 442.2995; 450.5688; 480.9806; 489.8944; 491.1695; 491.8703; 529.8388; 534.1481; 558.6356; 559.1535; 575.7421; 579.7042; 601.1036; 605.8765; 625.1834; 632.5459; 633.4038; 637.6710; 656.8618; 718.4993; 739.0576; 742.5094; 765.1308; 766.2826; 774.8921; 792.0798; 846.8469; 873.3194; 923.0228; 936.6672; 942.6214; 958.0144; 964.7006; 973.8808; 983.8297; 988.8651; 998.2768; 1011.3060; 1029.8762; 1040.2450; 1050.5703; 1065.7839; 1066.7511; 1071.7502; 1077.1485; 1088.5344; 1091.2558; 1121.8243; 1190.2830; 1192.6409; 1202.2853; 1204.6656; 1225.6192; 1243.5564; 1249.2963; 1258.7463; 1263.2638; 1264.5968; 1290.3283; 1296.7378; 1304.9616; 1318.6153; 1327.4494; 1369.9403; 1410.9381; 1436.6545; 1437.6039; 1449.5797; 1460.1978; 1463.4316; 1496.2133; 1503.8729; 1504.8360; 1515.0770; 1515.2924; 1524.2386; 1533.5082; 1534.9758; 1549.3535; 1616.4186; 1622.2556; 1631.7394; 1779.4726; 1865.7892; 3051.1519; 3066.0688; 3067.1426; 3073.4822; 3104.2563; 3125.3853; 3134.7257; 3136.2253; 3143.7366; 3147.0602; 3147.6547; 3152.4121; 3154.9415; 3158.0925; 3166.1165; 3176.9109; 3194.0383.

Cartesian Forces: Max 0.000000430 RMS 0.000000163

Item	Value	Threshold	Converged?
Maximum Force	0.000000	0.000450	YES
RMS Force	0.000000	0.000300	YES
Maximum Displacement	0.000031	0.001800	YES
RMS Displacement	0.000004	0.001200	YES

FILE: Yb_Sq2_PhYbOTf2_GS_Neutral_B3LYP_M062x_Conf7.out

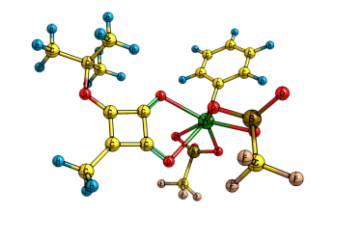

Conformer 8

E(RB3LYP/6-31G(d)/MWB59) = -2769.91042796
Convg = 0.9540D-08 -V/T = 2.0149

Temperature 195.150 Kelvin. Pressure 1.00000 Atm.
elec. and zero-point Energies= -2769.567147
elec. and thermal Energies= -2769.547628
elec. and thermal Enthalpies= -2769.547010
elec. and thermal Free Energies= -2769.610320

RB3LYP/6-31G(d)/MWB59/THF(IEFPCM) = -2769.93531180

RM06-2x/6-311+G(d,p)/MWB59/THF(IEFPCM) = -2769.73862727

Geometry at RB3LYP/6-31G(d)/MWB59
Charge = 0 Multiplicity = 1
Yb -2.5244722717 1.0086465833 1.6571958361
O -1.0871168853 2.3034868517 0.2526046914
S -1.7447134197 3.6420865262 0.5310077378
O -0.8824867657 4.7538528611 0.8879884734
O -2.9017162105 3.3145570545 1.454742131
C -2.5674990608 4.10326317 -1.0790197349
F -1.6435209091 4.1617666176 -2.040921613
F -3.4825442896 3.1857385026 -1.4037347461
F -3.1563828088 5.2893191447 -0.9503055568
O -4.7761718067 0.8871819642 2.3070519154
O -5.4656877602 -1.2888830984 3.4340800561
O -3.5865502897 -1.1170219826 1.7341834102
S -4.9205721874 -0.621020047 2.2666186877
C -6.1255404536 -0.9326439931 0.8758113674
F -7.3435313594 -0.5555524907 1.2570805188
F -5.7670117923 -0.2421113839 -0.2095463809
F -6.1285552876 -2.2358846719 0.5858101178
C -0.3842581595 -1.649485995 -1.4223688147
C -1.4191156183 -1.2453892136 -2.2794504442
C -2.0541853346 -0.4429021738 -1.2269262728
C -0.959755538 -0.8639742716 -0.2853041951
O -0.7533780752 -0.5964696498 0.8897569854
O -3.0161865377 0.284935438 -1.0384952967
O 0.6292215024 -2.4218653104 -1.6702539659
C 1.6767471935 -2.7567631503 -0.633925803
C -1.7336870924 -1.5244002621 -3.6987561401
H -0.9921287905 -2.1871753322 -4.1517830341
H -1.7728625275 -0.5878829015 -4.2692725827
H -2.7257124572 -1.985576622 -3.784011239
C -1.4617127329 1.0417473842 3.7249181442
C -0.9971122439 -0.1419803975 4.3425752256
C -1.2582855744 2.2383365062 4.450287313
H -1.128800324 -1.1026663559 3.8417474515
H -1.598475825 3.1882677949 4.037453575
C -0.3715548159 -0.1395959803 5.5926713121
C -0.6317175435 2.2571537223 5.7002213567
H -0.0331004208 -1.0735990116 6.0373814691
H -0.4928170324 3.1994810551 6.2262258874
C -0.1859831761 1.065155357 6.2745036085
H 0.2997519284 1.0742083706 7.2476830752
C 1.0029411256 -3.4986085491 0.5188600203
H 0.4482032186 -4.3678665419 0.1511978276
H 1.7730383036 -3.856253758 1.2103251733
H 0.32952936 -2.8512872356 1.0867294421
C 2.3645351344 -1.4642927963 -0.1982561472
H 3.2083891904 -1.7129272307 0.4537969009
H 2.7552117273 -0.92032331 -1.0642284618
H 1.6957662331 -0.8114706291 0.3688604929
C 2.6178109747 -3.664538003 -1.4215999585
H 3.4398328918 -3.9871459884 -0.7754072564
H 2.0915433332 -4.5539460966 -1.7814740223
H 3.0410682611 -3.1356804707 -2.2810165378

Frequency: 12.2148; 13.0960; 16.0580; 22.6852; 23.9808; 24.5661; 30.2254; 33.2750; 36.7711; 39.4681; 48.7244; 52.5503; 59.1665; 59.9762; 64.5999; 77.2681; 79.5412; 85.5351; 110.0845; 116.2358; 126.8067; 142.6068; 149.0287; 153.9393; 162.8135; 174.1815; 193.1951; 198.8111; 202.9578; 208.2188; 216.9829; 218.2797; 230.8341; 238.9303; 242.3172; 262.3981; 271.0662; 272.9894; 305.0209; 306.5057; 310.1809; 321.4989; 322.2210; 345.5305; 348.2365; 361.2089; 365.8398; 392.4582; 396.0928; 442.7547; 450.3232; 480.7076; 489.7564; 490.8026; 491.5799; 529.6897; 533.9022; 558.5740; 558.8223; 574.7204; 579.2580; 600.7429; 606.1439; 623.2105; 632.7489; 635.8128; 637.9240; 657.3007; 717.3305; 739.4340; 741.8726; 764.7771; 765.0069; 775.6871; 791.9039; 845.5122; 872.2377; 922.9038; 936.2422; 942.0815; 956.7920; 966.5249; 973.5641; 982.5771; 988.6237; 998.1810; 1011.3006; 1029.9490; 1040.4544; 1050.7886; 1065.7208; 1066.6209; 1071.6153; 1078.7987; 1088.5859; 1091.9753; 1121.3089; 1190.4764; 1192.0263; 1204.4636; 1205.6840; 1225.9373; 1242.4571; 1245.5147; 1250.6518; 1262.5036; 1264.6270; 1290.3519; 1297.7103; 1304.7193; 1322.9643; 1329.5175; 1370.4802; 1410.8671; 1436.3420; 1437.4350; 1449.7791; 1460.4104; 1463.3584; 1496.7915; 1503.7275; 1504.9468; 1514.8657; 1515.2057; 1524.7411; 1533.4201; 1534.7686; 1549.2662; 1616.8529; 1623.4820; 1631.8123; 1782.4347; 1867.3705; 3051.0094; 3066.1157; 3067.2565; 3073.5570; 3103.8938; 3128.9265; 3134.8094; 3136.3878; 3143.6311; 3147.1872; 3147.2469; 3151.9095; 3155.1555; 3158.3797; 3167.1704; 3177.7172; 3194.4849.

Cartesian Forces: Max 0.000001221 RMS 0.000000410

Item	Value	Threshold	Converged?
Maximum Force	0.000000	0.000450	YES
RMS Force	0.000000	0.000300	YES
Maximum Displacement	0.000048	0.001800	YES
RMS Displacement	0.000007	0.001200	YES

FILE: Yb_Sq2_PhYbOTf2_GS_Neutral_B3LYP_M062x_Conf8.out

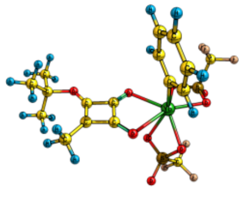

Conformer 9

E(RB3LYP/6-31G(d)/MWB59) = -2769.89802257
Convg = 0.7516D-08 -V/T = 2.0149

Temperature 195.150 Kelvin. Pressure 1.00000 Atm.
elec. and zero-point Energies= -2769.554819
elec. and thermal Energies= -2769.535370
elec. and thermal Enthalpies= -2769.534752
elec. and thermal Free Energies= -2769.597862

RB3LYP/6-31G(d)/MWB59/THF(IEFPCM) = -2769.92669278

RM06-2x/6-311+G(d,p)/MWB59/THF(IEFPCM) = -2769.73200164

Geometry at RB3LYP/6-31G(d)/MWB59
Charge = 0 Multiplicity = 1
Yb -1.7197823603 1.5041669969 1.5401238656
O -2.4731742319 -0.6790610972 2.333354803
S -3.8684577578 -0.213039334 2.6934947613
O -4.9859910603 -1.0390568509 2.2675546841
O -3.9040474713 1.260587614 2.3307885352
C -3.8731146958 -0.2069477695 4.5581481281
F -3.7777936675 -1.4603497743 5.0022256517
F -2.8299731187 0.5008367588 5.0020071752
F -5.0029556834 0.3408115623 4.9993133093
O 0.2378418874 1.7155912258 2.8031153395
O -0.0451688032 2.7158085515 5.1221590085
O -1.6199805554 3.1775230087 3.1810086462
S -0.225827937 2.8611215088 3.6890418238
C 0.81991114 4.3157297708 3.1649939268
F 2.0933992888 4.062503372 3.4665066848
F 0.7041489261 4.5144383129 1.8526391968
F 0.4098212875 5.4007163202 3.8191567002
C -0.6653261909 -1.8020425904 -1.3843187937
C -1.99984995 -1.6418522746 -1.8068163936
C -2.2290082054 -0.5767917154 -0.8163771713
C -0.8107838634 -0.7314474116 -0.3530239952
O -0.1557445982 -0.1616956958 0.5052261982
O -3.1168526869 0.1707740579 -0.4424030056
O 0.4195581106 -2.482824094 -1.6072390636
C 0.5964049175 -3.577317668 -2.6105169496
C -2.9550379087 -2.1801700425 -2.8090615624
H -2.8010346851 -3.2375187545 -3.0356008013
H -2.8744380966 -1.6177283231 -3.749545673
H -3.9762937427 -2.0416463419 -2.4396616343
C -1.4128083483 2.838376861 -0.3790545901
C -0.1695490964 2.9833695103 -1.0367658335
C -2.4851359294 3.5763720982 -0.9316602397
H 0.7071123228 2.4511754964 -0.6620032438
H -3.4788667334 3.5096635559 -0.4835293215
C -0.0011360661 3.8021439701 -2.1589415707
C -2.33465057 4.403282889 -2.0492033763
H 0.9756382914 3.8882870542 -2.6318834803
H -3.1867620464 4.9574664025 -2.4380171892
C -1.0874231103 4.5168026302 -2.667795592
H -0.9630918241 5.1585398857 -3.537214682
C 0.2897622141 -3.0416406044 -4.0085757495
H 0.8876449968 -2.1492106167 -4.2180807148
H 0.5487623586 -3.8049520915 -4.7496060286
H -0.7660946375 -2.7970748818 -4.1444068061
C -0.2764082545 -4.7612446983 -2.1951129557
H -0.0950619794 -5.6022061921 -2.8724350871
H -0.0300691035 -5.0817671873 -1.1783476971
H -1.3433952837 -4.5267168914 -2.2340558493
C 2.0815462964 -3.9071740091 -2.4614532056
H 2.3475858877 -4.7255825824 -3.1376802857
H 2.6983685472 -3.037816432 -2.7075058902
H 2.3078357804 -4.2147604893 -1.4364460062

Frequency: 10.1481; 12.9363; 16.1139; 21.8430; 25.8856; 28.2898; 29.8028; 32.6113; 34.7410; 39.9112; 46.1799; 49.7541; 60.1839; 62.3434; 76.4803; 83.5926; 85.9366; 106.6006; 111.5947; 118.7618; 134.4770; 142.3973; 156.7051; 159.2025; 164.6749; 172.9263; 185.4416; 188.2516; 197.4238; 216.7769; 222.2000; 228.2031; 232.0061; 240.2627; 241.3065; 246.1756; 260.0007; 264.9389; 295.7201; 304.6545; 308.8550; 319.1471; 324.7651; 341.9611; 349.8979; 361.6512; 366.2503; 390.5300; 397.5214; 447.8923; 450.4948; 480.0448; 482.4074; 489.9992; 492.0455; 529.3282; 534.9413; 557.7371; 558.9553; 576.1184; 579.2459; 601.4131; 603.4197; 624.0741; 631.6083; 633.4088; 643.7792; 654.3722; 719.3612; 738.0290; 764.8460; 766.4408; 767.6879; 783.1152; 795.6895; 835.6529; 872.9576; 918.6604; 935.2480; 936.5610; 956.4737; 964.1981; 968.6486; 982.2651; 993.1784; 994.7132; 1010.8566; 1024.1953; 1039.0822; 1049.0783; 1060.5783; 1060.6211; 1068.3488; 1080.9231; 1087.5883; 1088.9469; 1149.0220; 1189.6810; 1196.9724; 1200.2938; 1201.4316; 1204.2398; 1224.7418; 1248.9348; 1258.9060; 1263.6442; 1265.9929; 1288.7001; 1294.1698; 1296.2764; 1318.4672; 1326.5398; 1369.2216; 1401.8876; 1434.5355; 1436.9300; 1443.3711; 1459.5400; 1461.5683; 1498.1326; 1503.7700; 1506.7465; 1514.6734; 1517.0627; 1524.1538; 1527.8681; 1530.5067; 1548.1076; 1616.1588; 1616.9874; 1630.7625; 1798.9401; 1872.1043; 3047.9806; 3068.7751; 3071.5219; 3077.6994; 3114.3600; 3120.8895; 3123.2037; 3138.2959; 3140.1669; 3148.0232; 3149.1862; 3150.9672; 3157.3180; 3164.4451; 3164.5558; 3174.4548; 3192.9427.

Cartesian Forces: Max 0.000000847 RMS 0.000000237

Item	Value	Threshold	Converged?
Maximum Force	0.000000	0.000450	YES
RMS Force	0.000000	0.000300	YES
Maximum Displacement	0.000049	0.001800	YES
RMS Displacement	0.000012	0.001200	YES

FILE: Yb_Sq2_PhYbOTf2_GS_Neutral_B3LYP_M062x_Conf9.out

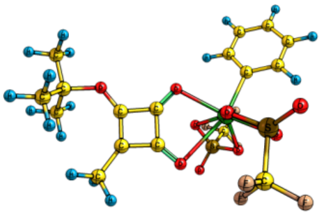

Conformer 10

E(RB3LYP/6-31G(d)/MWB59) = -2769.90313115
Convg = 0.2083D-09 -V/T = 2.0149

Temperature 195.150 Kelvin. Pressure 1.00000 Atm.
elec. and zero-point Energies= -2769.559747
elec. and thermal Energies= -2769.540311
elec. and thermal Enthalpies= -2769.539693
elec. and thermal Free Energies= -2769.602522

RB3LYP/6-31G(d)/MWB59/THF(IEFPCM) = -2769.92992056

RM06-2x/6-311+G(d,p)/MWB59/THF(IEFPCM) = -2769.73365818

Geometry at RB3LYP/6-31G(d)/MWB59
Charge = 0 Multiplicity = 1
Yb -2.4760374556 1.041217011 1.7381340274
O -1.2213274896 2.4048125138 0.2521297593
S -1.8336208452 3.7297864752 0.6715708059
O -0.9279067069 4.8233688914 0.9721267946
O -2.8712309369 3.3568186164 1.7116200728
C -2.8419335052 4.2630935595 -0.8048149174
F -2.0363585844 4.3887323596 -1.8620546946
F -3.7786555979 3.3497629798 -1.0711329122
F -3.4229816035 5.4314075604 -0.5432361416
O -3.4937465364 -1.1151351729 1.781592135
O -5.9853453225 -1.1460156872 1.3074235295
O -4.7590280658 0.869270314 2.2578495357
S -4.8941408596 -0.6342547126 2.1195761566
C -5.1924409526 -1.242342363 3.8583021626
F -5.1779591221 -2.57630798 3.8568533908
F -4.2415347901 -0.7873353323 4.6717191337
F -6.3814614659 -0.807487207 4.2722428308
C -0.9193909286 -1.6512645433 -1.6010882703
C -2.0596158608 -1.238291919 -2.3161160816
C -2.4907791947 -0.3625053796 -1.210903705
C -1.2786486203 -0.7964181763 -0.4314258876
O -0.8563697144 -0.5187105569 0.6794071184
O -3.394931679 0.4091552437 -0.9448754829
O 0.1307435906 -2.4145179614 -1.6704285348
C 0.5408162203 -3.2663868834 -2.828767298
C -2.7458785973 -1.4792284316 -3.6114909506
H -2.0589394173 -1.646668527 -4.4445313504
H -3.3832707649 -0.6202284195 -3.8441023201
H -3.4038733726 -2.3558170658 -3.5358382799
C -1.3294588333 1.0049896519 3.760046986
C -0.6428653139 -0.1494324847 4.2020730496
C -1.2843738365 2.1215474573 4.6265370797
H -0.636990061 -1.0480577343 3.5828011513
H -1.7908531912 3.0454686681 4.3466321078
C 0.041212762 -0.193811445 5.4202200306
C -0.6049571361 2.0919317034 5.8483761695
H 0.5575090633 -1.1020596985 5.725415162
H -0.5928899382 2.972642517 6.4873222722
C 0.0606020409 0.9312894733 6.2476991337
H 0.5913185254 0.9034937489 7.196762326
C 0.90532655 -2.3516669205 -3.9977115254
H 1.6846108746 -1.6426523955 -3.7022594093
H 1.2886985414 -2.9556564928 -4.826600729
H 0.0457582766 -1.7865294995 -4.3678424381
C -0.5719304227 -4.2641331104 -3.1476332924
H -0.2123297354 -4.9733243166 -3.9003453093
H -0.8485254591 -4.830749245 -2.2530980877
H -1.466388344 -3.7836803831 -3.5504223204
C 1.7764255088 -3.9776485182 -2.277435359
H 2.2002672434 -4.6308124074 -3.0465705124
H 2.538277747 -3.2521638069 -1.9779381524
H 1.5164853173 -4.5876089677 -1.4073009597

Frequency: 10.5608; 14.9871; 20.1198; 23.6464; 24.9157; 27.2483; 30.2313; 31.3621; 37.3740; 40.2782; 48.5233; 50.0895; 55.9747; 63.8826; 74.3310; 90.7904; 91.4682; 101.4405; 113.4609; 120.3848; 126.1717; 135.7623; 150.3459; 159.1730; 164.7164; 170.1431; 187.3353; 191.7538; 204.6064; 215.0497; 221.5907; 230.0784; 231.8235; 239.3010; 241.4383; 244.8369; 260.2267; 265.2554; 297.0061; 305.3684; 306.6460; 321.4575; 322.9191; 342.6770; 349.7761; 362.4344; 365.1495; 392.7223; 397.4107; 442.0288; 450.4139; 481.1089; 483.4553; 491.1284; 491.9690; 529.9196; 534.1007; 558.6182; 559.2010; 575.9283; 579.7944; 602.8542; 604.5742; 625.3117; 632.3767; 633.0702; 643.0997; 657.5815; 717.8573; 739.2500; 765.0384; 766.3273; 767.0356; 783.8080; 795.0606; 835.2225; 872.8331; 923.5073; 935.3406; 936.5484; 958.4473; 965.0708; 973.7768; 982.7769; 991.8645; 998.2341; 1011.1331; 1022.2428; 1040.5270; 1048.3859; 1061.0100; 1066.4825; 1067.9206; 1078.0032; 1088.7055; 1091.8147; 1143.8505; 1190.5350; 1196.4927; 1200.0839; 1202.8527; 1204.9759; 1225.9346; 1249.2093; 1258.6704; 1262.7209; 1263.5322; 1288.4740; 1296.5838; 1297.4800; 1318.6942; 1327.7768; 1370.5841; 1401.3641; 1434.3263; 1436.8593; 1442.9688; 1460.3117; 1461.4215; 1498.6268; 1504.1921; 1506.6763; 1514.6978; 1516.7862; 1524.5895; 1527.8993; 1530.8012; 1547.9165; 1616.7541; 1619.2092; 1631.8189; 1803.0566; 1873.0374; 3048.6586; 3068.8438; 3071.3969; 3077.6938; 3114.4092; 3129.7519; 3138.5218; 3140.1319; 3147.3983; 3148.2019; 3148.3315; 3150.1963; 3155.2734; 3163.9994; 3167.1643; 3177.4621; 3194.2246.

Cartesian Forces: Max 0.000000866 RMS 0.000000318

Item	Value	Threshold	Converged?
Maximum Force	0.000000	0.000450	YES
RMS Force	0.000000	0.000300	YES
Maximum Displacement	0.000028	0.001800	YES
RMS Displacement	0.000005	0.001200	YES

FILE: Yb_Sq2_PhYbOTf2_GS_Neutral_B3LYP_M062x_Conf10.out

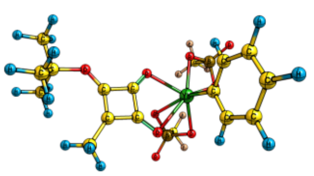

Conformer 11

E(RB3LYP/6-31G(d)/MWB59) = -2769.89806121
Convg = 0.8924D-08 -V/T = 2.0149

Temperature 195.150 Kelvin. Pressure 1.00000 Atm.
elec. and zero-point Energies= -2769.554770
elec. and thermal Energies= -2769.535299
elec. and thermal Enthalpies= -2769.534681
elec. and thermal Free Energies= -2769.597936

RB3LYP/6-31G(d)/MWB59/THF(IEFPCM) = -2769.92652778

RM06-2x/6-311+G(d,p)/MWB59/THF(IEFPCM) = -2769.73180463

Geometry at RB3LYP/6-31G(d)/MWB59
Charge = 0 Multiplicity = 1
Yb -1.76468205 1.5601362639 1.51706387
O -2.5997751062 -0.5875956324 2.3424909082
S -4.0206209393 -0.1060537576 2.5565035466
O -5.0930581563 -0.9204748929 2.0080638061
O -3.9996624533 1.3672838308 2.1992965589
C -4.2468141579 -0.1200173126 4.4074079064
F -4.1587072745 -1.3758324814 4.848818853
F -3.2987156758 0.619350207 4.988628204
F -5.4442439302 0.3776828831 4.7088440098
O -1.632271134 3.0293953791 3.3364171765
O 0.5942822434 4.2155603767 3.677455092
O 0.3416772158 1.9878078993 2.4724012712
S -0.1292354456 2.9663425118 3.5333504907
C 0.0863493836 2.0258416955 5.1298530365
F -0.4872790601 2.6985436498 6.12449726
F -0.4864975534 0.8210514831 5.0189981223
F 1.3856380707 1.8728543344 5.3788528683
C -0.6376319359 -1.8364753883 -1.3118120201
C -1.955734925 -1.6937808603 -1.789311244
C -2.2251168043 -0.600378079 -0.8385829594
C -0.8266896256 -0.7379237173 -0.3184194574
O -0.2078308958 -0.1375406735 0.5465326989
O -3.1245421721 0.1570066624 -0.5194162605
O 0.4573256335 -2.5185506411 -1.4737628276
C 0.679579488 -3.6331669837 -2.4453943806
C -2.8704504973 -2.2648832924 -2.8107594962
H -2.6984858403 -3.325271957 -3.0079730593
H -2.7632994326 -1.7227192076 -3.7605090586
H -3.9051184783 -2.128520935 -2.4797000088
C -1.6254594979 3.0023278586 -0.3286112261
C -0.3975767285 3.6221920066 -0.6622604972
C -2.7316885659 3.357128886 -1.1357424411
H 0.4975352247 3.4039966022 -0.0778124487
H -3.7086276523 2.9152468193 -0.936765067
C -0.2763262802 4.5262896527 -1.7234035309
C -2.6286104881 4.2649102166 -2.1935571958
H 0.686900215 4.9816442066 -1.9454293477
H -3.5067389039 4.5165428195 -2.7854115192
C -1.3956911974 4.8509595242 -2.4915888389
H -1.3096808444 5.5579990482 -3.3138030612
C 0.4242909509 -3.1298588051 -3.8656276262
H 1.0242304065 -2.237869586 -4.0710764531
H 0.7170265708 -3.9069896239 -4.5792121111
H -0.6270253071 -2.8960863379 -4.0479201743
C -0.2015071658 -4.8138970001 -2.0383945507
H 0.0110803703 -5.6679745125 -2.6897189834
H 0.007213271 -5.1108260222 -1.0061544212
H -1.2676204663 -4.587732577 -2.1235938581
C 2.1598141318 -3.9494075545 -2.2319202432
H 2.4568767723 -4.7801858391 -2.8795373428
H 2.7803629702 -3.0812163194 -2.4725713939
H 2.3480527231 -4.2335488279 -1.1925425751

Frequency: 7.4322; 9.2442; 18.9774; 23.8233; 27.8450; 28.8393; 31.5528; 33.2273; 35.1931; 43.1405; 46.6068; 55.1964; 57.4632; 63.3879; 77.4839; 80.9640; 87.7932; 104.9169; 110.0911; 119.2603; 131.2634; 136.6199; 148.6497; 154.1733; 160.1537; 173.5316; 184.9207; 194.7870; 200.6106; 215.9502; 220.5572; 226.5435; 232.0240; 236.9276; 239.6093; 243.8979; 260.0277; 264.3798; 295.7937; 304.6961; 308.6591; 320.6710; 323.4676; 342.4228; 349.8531; 359.9657; 367.4844; 392.9278; 397.5267; 442.9380; 450.5260; 480.3334; 482.8953; 489.8317; 490.9746; 528.8785; 534.5974; 558.0424; 558.2447; 574.1560; 580.7551; 601.3414; 603.8114; 620.9282; 632.6393; 634.9745; 643.8874; 655.3154; 719.3480; 739.1516; 764.7690; 765.5761; 767.8118; 783.4276; 795.3070; 835.7328; 874.7873; 924.9657; 935.0784; 936.4522; 955.2927; 968.3103; 974.7383; 982.3189; 992.4472; 997.2694; 1010.7677; 1024.5390; 1038.9421; 1048.4987; 1060.5606; 1066.6287; 1068.3853; 1080.9445; 1086.5518; 1091.2615; 1150.6811; 1190.0583; 1196.7556; 1199.1029; 1202.4050; 1206.7611; 1226.0853; 1241.1195; 1253.3977; 1264.0943; 1268.5859; 1288.6734; 1295.2908; 1296.1808; 1315.9978; 1330.9443; 1370.8545; 1401.1250; 1434.4847; 1436.8880; 1442.9367; 1459.6094; 1461.5644; 1497.7558; 1503.3411; 1506.5986; 1514.6906; 1516.9201; 1524.1322; 1527.8809; 1530.4522; 1548.0704; 1615.6040; 1617.6145; 1630.8291; 1799.2001; 1874.3476; 3047.6258; 3068.8403; 3071.5103; 3077.7150; 3113.8858; 3136.7421; 3138.4612; 3140.1081; 3141.1601; 3148.2126; 3149.1403; 3150.9321; 3157.3491; 3164.1584; 3166.3172; 3175.9275; 3192.9648.

Cartesian Forces: Max 0.000001383 RMS 0.000000258

Item	Value	Threshold	Converged?
Maximum Force	0.000001	0.000450	YES
RMS Force	0.000000	0.000300	YES
Maximum Displacement	0.000076	0.001800	YES
RMS Displacement	0.000018	0.001200	YES

FILE: Yb_Sq2_PhYbOTf2_GS_Neutral_B3LYP_M062x_Conf11.out

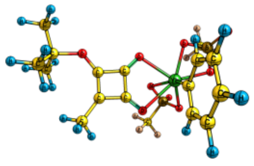

Conformer 12

E(RB3LYP/6-31G(d)/MWB59) = -2769.89409091
Convg = 0.6955D-08 -V/T = 2.0149

Temperature 195.150 Kelvin. Pressure 1.00000 Atm.
elec. and zero-point Energies= -2769.550822
elec. and thermal Energies= -2769.531368
elec. and thermal Enthalpies= -2769.530750
elec. and thermal Free Energies= -2769.594037

RB3LYP/6-31G(d)/MWB59/THF(IEFPCM) = -2769.92431061

RM06-2x/6-311+G(d,p)/MWB59/THF(IEFPCM) = -2769.72996197

Geometry at RB3LYP/6-31G(d)/MWB59
Charge = 0 Multiplicity = 1
Yb -1.8568572247 1.4298989643 1.4886982265
O -4.0426252074 1.2806709805 2.3472407456
S -3.9643870577 -0.1040115173 2.9633425096
O -4.2216331404 -0.2106725059 4.3903946362
O -2.6742317588 -0.697518128 2.4411317133
C -5.3350680992 -1.0802237193 2.1542280876
F -6.507946024 -0.5679501224 2.5220629026
F -5.2394202863 -1.0462200748 0.8227509135
F -5.2519103082 -2.3495152124 2.5621039136
O -1.7300645002 3.1295256327 3.0967267215
O 0.5961516789 4.017364661 3.6351521946
O 0.1475369318 1.7125218902 2.6568066255
S -0.2975588552 2.8796933524 3.5245005914
C -0.4584514175 2.132877884 5.2261675283
F -0.9808469728 3.0313356129 6.0565720699
F -1.257329002 1.063215985 5.1630564044
F 0.7449927717 1.7622724228 5.6612089237
C -0.6461935656 -1.8564209683 -1.4006225447
C -1.954158809 -1.6422715385 -1.8851445217
C -2.2124284556 -0.6297136545 -0.8525170823
C -0.8297308467 -0.8364578544 -0.3212679491
O -0.2250779448 -0.3116493417 0.5974656967
O -3.0968494746 0.1273556853 -0.4805960235
O 0.4342771882 -2.546263728 -1.6042647701
C 0.6570855963 -3.5747545388 -2.6694232784
C -2.8568707311 -2.1001648257 -2.9730012837
H -2.712703051 -3.1475840137 -3.2473389687
H -2.7041009928 -1.4907154349 -3.874283216
H -3.8964157477 -1.9557186568 -2.6617028738
C -1.4647735503 2.7794259387 -0.4049598698
C -0.1721096435 3.3028353353 -0.6462025926
C -2.4661263619 3.1814870926 -1.3193841675
H 0.6510651744 3.0465308413 0.0228284004
H -3.4868065395 2.8151929246 -1.1980007289
C 0.1063126228 4.1584467161 -1.7179997924
C -2.2076834889 4.0431966391 -2.3894918397
H 1.115391247 4.5387419039 -1.8649692792
H -3.0111540429 4.3345012374 -3.0637070775
C -0.914996476 4.5326101785 -2.5929652773
H -0.7071300115 5.2032719075 -3.4238118153
C 0.4389958702 -2.9402486478 -4.0421986943
H 1.0566385259 -2.0438228517 -4.1544449283
H 0.7345724845 -3.6547471844 -4.8173960909
H -0.6047072973 -2.6733236098 -4.2227604083
C -0.2535077108 -4.7688188532 -2.3859263532
H -0.0413088343 -5.5662950441 -3.1055151952
H -0.0731158498 -5.159528649 -1.3799213133
H -1.3132675697 -4.5151157321 -2.4737809916
C 2.1267098079 -3.936666353 -2.4566084261
H 2.4226003437 -4.7114190405 -3.1707736227
H 2.7669113862 -3.0623773227 -2.6062852062
H 2.2885422205 -4.3165896623 -1.4437426223

Frequency: 7.9182; 11.5291; 14.4192; 23.8618; 24.2982; 24.9701; 32.6705; 33.4435; 37.3167; 38.9179; 44.3449; 51.0419; 60.0111; 69.4213; 83.2199; 84.3722; 92.2776; 109.5315; 114.8077; 118.1375; 131.9994; 139.6670; 145.3325; 156.0053; 160.3869; 166.1369; 186.7440; 191.7200; 202.3445; 211.0642; 220.6388; 230.5914; 235.3247; 238.6723; 241.0449; 244.8106; 259.4459; 263.7326; 296.4908; 305.3614; 307.4503; 319.7073; 323.0530; 340.9178; 349.7949; 358.1989; 367.0619; 392.4143; 396.9970; 445.4950; 449.9326; 478.9108; 482.0963; 489.2607; 491.0361; 527.4814; 534.5546; 558.0303; 558.4673; 569.6616; 580.9538; 602.4555; 602.6794; 627.2806; 632.5608; 633.5422; 642.8789; 654.1427; 719.7027; 738.7349; 764.8800; 765.4855; 768.2209; 782.0864; 796.5899; 833.7576; 874.4688; 922.5377; 935.2295; 936.7446; 957.4070; 966.5845; 972.8575; 982.6434; 993.7112; 996.1304; 1010.8195; 1025.9000; 1038.4515; 1048.9840; 1060.8544; 1063.0252; 1068.6083; 1083.7146; 1086.2398; 1090.6390; 1152.0579; 1189.7322; 1195.7296; 1201.3303; 1201.8663; 1206.7914; 1225.7719; 1247.1442; 1250.3400; 1259.8830; 1268.3021; 1288.7728; 1294.3675; 1296.4954; 1315.2967; 1329.6835; 1370.1815; 1404.2312; 1434.6064; 1437.1843; 1444.2437; 1459.5631; 1461.7076; 1498.3928; 1503.8489; 1506.8223; 1514.5851; 1516.7693; 1524.0773; 1527.8726; 1530.4992; 1547.8740; 1614.6609; 1616.3727; 1630.8062; 1800.5736; 1872.9832; 3049.1468; 3069.1312; 3071.7055; 3077.9819; 3114.8383; 3131.4598; 3133.8042; 3139.0100; 3140.4384; 3148.6998; 3149.0406; 3150.8899; 3156.8896; 3164.2719; 3165.1796; 3174.9164; 3192.6143.

Cartesian Forces: Max 0.000001107 RMS 0.000000373

Item	Value	Threshold	Converged?
Maximum Force	0.000000	0.000450	YES
RMS Force	0.000000	0.000300	YES
Maximum Displacement	0.000025	0.001800	YES
RMS Displacement	0.000003	0.001200	YES

FILE: Yb_Sq2_PhYbOTf2_GS_Neutral_B3LYP_M062x_Conf12.out

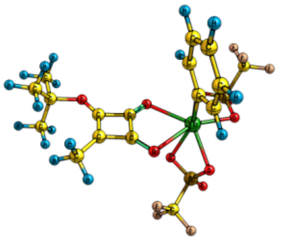

Conformer 13

E(RB3LYP/6-31G(d)/MWB59) = -2769.89240374
Convg = 0.7500D-08 -V/T = 2.0149

Temperature 195.150 Kelvin. Pressure 1.00000 Atm.
elec. and zero-point Energies= -2769.549325
elec. and thermal Energies= -2769.529796
elec. and thermal Enthalpies= -2769.529178
elec. and thermal Free Energies= -2769.593167

RB3LYP/6-31G(d)/MWB59/THF(IEFPCM) = -2769.92302052

RM06-2x/6-311+G(d,p)/MWB59/THF(IEFPCM) = -2769.72806845

Geometry at RB3LYP/6-31G(d)/MWB59
Charge = 0 Multiplicity = 1
Yb -1.7609883858 1.3676292887 1.4998378866
O -3.961497562 1.1962068812 2.3085794169
S -3.8667222047 -0.1765329225 2.9492482284
O -4.1327249206 -0.2658287217 4.3751588687
O -2.5613870215 -0.7559084276 2.4448113867
C -5.2139096302 -1.1904276863 2.1462764519
F -6.3965662984 -0.6646930071 2.460375827
F -5.0838313089 -1.2155796079 0.8173939824
F -5.1401017484 -2.4399874637 2.6116575235
O 0.2792010909 1.6467117596 2.6158024842
O 0.1176344823 2.4346913135 5.0260824663
O -1.6453095614 2.9126401055 3.2563749979
S -0.1931755139 2.6768438056 3.6293105334
C 0.6888321288 4.2529618102 3.1571837054
F 2.0021892103 4.070609044 3.2971589787
F 0.4164790135 4.5734164181 1.8929937385
F 0.2826008169 5.2324250114 3.9628488443
C -0.5665974646 -1.8639450569 -1.4729386926
C -1.8858116692 -1.6723803215 -1.9350413154
C -2.1455062584 -0.6660509085 -0.8942556633
C -0.7500302849 -0.8494725405 -0.3897962802
O -0.1383964986 -0.3152383113 0.5201929314
O -3.0346402856 0.0736086717 -0.5034341462
O 0.5229644298 -2.5345627676 -1.6955835085
C 0.7408026415 -3.566496076 -2.757783153
C -2.7989278947 -2.144623059 -3.0077412767
H -2.64108452 -3.1888106471 -3.2868454825
H -2.6727334737 -1.5308759032 -3.9102842709
H -3.835198088 -2.0187898522 -2.6779824178
C -1.4814730352 2.7634461999 -0.381699375
C -0.2163613035 3.0056254711 -0.9656152251
C -2.5733313653 3.4170749797 -0.9986152671
H 0.67572587 2.5452009163 -0.5362970681
H -3.5828411359 3.2724071074 -0.6083760724
C -0.0464295161 3.835113325 -2.0798719745
C -2.4215511564 4.2563252168 -2.1065642009
H 0.9467122781 3.9966576959 -2.4954526259
H -3.2891660023 4.7460951007 -2.5446395495
C -1.1527859726 4.4655937197 -2.6525457044
H -1.0276193762 5.1169211316 -3.5147115585
C 0.4892039789 -2.9444178015 -4.1305773217
H 1.0931395966 -2.0410125851 -4.2602959105
H 0.7794199249 -3.6603738075 -4.9064585344
H -0.5609604163 -2.691842181 -4.2934088265
C -0.1475714963 -4.7714750427 -2.4505710386
H 0.0627335068 -5.5704379954 -3.1690635519
H 0.0565675191 -5.1532315038 -1.4456739154
H -1.2122578855 -4.5332775767 -2.5205042697
C 2.218939953 -3.9068686973 -2.5695544411
H 2.5125759521 -4.6822291465 -3.2839877219
H 2.8442340059 -3.0249228943 -2.7365099882
H 2.4043498552 -4.2777134607 -1.5573799037

Frequency: 6.8811; 10.8056; 11.3981; 17.6636; 22.5874; 28.8868; 29.3511; 33.2200; 33.9390; 39.6135; 44.1836; 46.1063; 61.5995; 66.0521; 82.0472; 82.8786; 90.5785; 109.5680; 115.0174; 115.9080; 131.5458; 139.9128; 144.4974; 157.3166; 162.1613; 163.9137; 178.7973; 186.8501; 202.3060; 211.4716; 219.9589; 231.3804; 233.4025; 236.4537; 242.6793; 246.7401; 259.6335; 263.5971; 295.8090; 304.5299; 307.0759; 318.8851; 323.0321; 341.3313; 349.7477; 358.9012; 365.9025; 390.8003; 397.1827; 447.7030; 450.1328; 479.3933; 482.1813; 488.9216; 491.9776; 527.2040; 535.4571; 558.0626; 558.9687; 568.7465; 581.2319; 601.7651; 603.0085; 625.1702; 633.3315; 636.9958; 643.1524; 654.4615; 719.4704; 738.1586; 764.8045; 766.5589; 768.0809; 782.9721; 796.3442; 834.2219; 873.1500; 918.7116; 935.2659; 936.6239; 956.9124; 966.3035; 968.6258; 982.4060; 993.3926; 994.5167; 1010.9539; 1025.8278; 1038.8803; 1048.6143; 1060.2247; 1060.6020; 1068.6385; 1079.4871; 1087.2139; 1088.8070; 1152.9984; 1189.5674; 1195.9180; 1199.7477; 1201.7421; 1206.4107; 1224.5262; 1247.2923; 1258.4632; 1259.7467; 1263.0980; 1288.6545; 1293.9662; 1296.4682; 1315.1097; 1328.7328; 1369.0219; 1403.1611; 1434.5646; 1437.1182; 1443.7012; 1459.4163; 1461.6725; 1498.2386; 1503.7159; 1506.7362; 1514.5557; 1516.8357; 1523.7135; 1527.9321; 1530.4599; 1548.0360; 1615.5171; 1616.0525; 1630.5229; 1800.3327; 1873.3155; 3048.7589; 3069.1041; 3071.6750; 3077.9193; 3114.6452; 3123.1666; 3124.0977; 3138.8018; 3140.4848; 3148.5437; 3149.0601; 3150.9099; 3156.7891; 3164.2648; 3164.4153; 3173.9587; 3192.5942.

Cartesian Forces: Max 0.000001081 RMS 0.000000407

Item	Value	Threshold	Converged?
Maximum Force	0.000001	0.000450	YES
RMS Force	0.000000	0.000300	YES
Maximum Displacement	0.000033	0.001800	YES
RMS Displacement	0.000008	0.001200	YES

FILE: Yb_Sq2_PhYbOTf2_GS_Neutral_B3LYP_M062x_Conf13.out

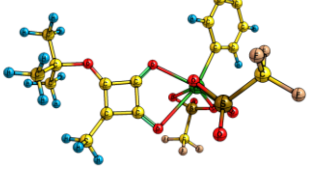

Conformer 14

E(RB3LYP/6-31G(d)/MWB59) = -2769.90312128
Convg = 0.7532D-08 -V/T = 2.0149

Temperature 195.150 Kelvin. Pressure 1.00000 Atm.
elec. and zero-point Energies= -2769.559749
elec. and thermal Energies= -2769.540305
elec. and thermal Enthalpies= -2769.539686
elec. and thermal Free Energies= -2769.602569

RB3LYP/6-31G(d)/MWB59/THF(IEFPCM) = -2769.92996195

RM06-2x/6-311+G(d,p)/MWB59/THF(IEFPCM) = -2769.73371863

Geometry at RB3LYP/6-31G(d)/MWB59
Charge = 0 Multiplicity = 1
Yb -2.5622412392 0.8937026057 1.788518813
O -2.992670146 3.1445662193 1.2780691693
S -1.7790756739 3.3990214499 0.4060960736
O -1.9923760716 4.0240961842 -0.8885269631
O -1.0121725024 2.0882713993 0.4250349214
C -0.7236482626 4.5748223302 1.398914391
F -1.353334718 5.7443118708 1.4989579106
F -0.5053543017 4.0785604069 2.6150380959
F 0.4407803718 4.7443067096 0.7703425645
O -4.7183043199 0.9097174702 2.7209649401
O -5.6079096913 -1.3279405718 3.5428399864
O -3.8626525036 -1.0922901168 1.7118091152
S -5.0695284607 -0.5396012813 2.4495765379
C -6.4051586397 -0.4474273109 1.1502441332
F -7.5212694677 0.0273701075 1.6978442558
F -6.0197139817 0.3568454239 0.1566462033
F -6.628107129 -1.6710126002 0.6649311593
C -0.8907312333 -1.6826769251 -1.5862829927
C -1.8906595605 -1.0936602583 -2.3832496971
C -2.40520917 -0.3152574334 -1.2413997848
C -1.3395798367 -0.9326910082 -0.3764017914
O -1.0416634369 -0.806069388 0.8002931554
O -3.287533425 0.4877162994 -0.9954437718
O 0.1044721562 -2.5186267551 -1.6168513945
C 0.5774019221 -3.3016624517 -2.7995607449
C -2.3740031575 -1.0826214449 -3.787944765
H -2.2824556807 -2.0492696711 -4.2888301579
H -1.8133767801 -0.3433813129 -4.3764862985
H -3.4238231951 -0.7727836513 -3.802957077
C -1.4138207393 0.8809957094 3.809585183
C -0.018857708 0.6565873588 3.8664541303
C -2.0547371947 1.110129511 5.0490315801
H 0.5395241589 0.4735782776 2.9467874502
H -3.1298474958 1.288411496 5.0784130835
C 0.6920603681 0.6597171566 5.069954852
C -1.3568832885 1.1171107434 6.2607785635
H 1.7660706616 0.4833080512 5.0718648524
H -1.8864594218 1.2967521398 7.1942282654
C 0.0209186362 0.8911396858 6.2728389613
H 0.5681317108 0.8947361196 7.2128908521
C 0.9584127153 -2.3423053917 -3.9264344672
H 1.6774031621 -1.5977057132 -3.5709661951
H 1.4296885803 -2.909247053 -4.7359491014
H 0.0946691358 -1.8236648992 -4.3484998867
C -0.509218202 -4.3059254357 -3.1825198357
H -0.1434850634 -4.9466582847 -3.9915521833
H -0.761409887 -4.9429894777 -2.3293918853
H -1.4229590438 -3.8180759942 -3.5321514036
C 1.8106965499 -4.009379366 -2.2385520585
H 2.2532039625 -4.6471458175 -3.0100920859
H 2.5612798274 -3.2821030711 -1.9153925093
H 1.5413637097 -4.6343580411 -1.3822041493

Frequency: 10.6044; 14.7428; 19.2375; 23.9199; 25.3012; 27.0583; 30.0399; 31.0684; 37.2449; 40.4555; 48.8643; 49.8828; 55.4752; 63.8167; 74.1455; 90.4806; 91.2092; 101.2151; 113.0189; 119.9945; 125.7640; 135.7048; 150.2533; 159.0627; 164.8053; 170.1262; 186.8689; 191.7379; 204.5269; 215.1515; 221.5164; 229.9387; 231.5355; 239.2697; 241.0697; 244.5776; 260.3000; 265.0573; 296.8734; 305.3699; 306.5616; 321.5839; 322.8465; 342.5308; 349.7450; 362.4101; 365.0941; 392.6334; 397.5095; 441.8958; 450.3659; 481.2423; 483.5132; 491.0832; 491.9440; 529.9405; 534.1236; 558.6337; 559.1560; 575.9524; 579.8167; 603.0329; 604.1721; 625.2734; 632.3538; 633.0533; 643.0770; 657.5838; 717.7979; 739.3113; 765.0976; 766.2651; 767.0271; 783.6917; 794.9540; 835.3079; 872.8786; 923.4974; 935.1752; 936.7163; 958.4094; 965.0318; 973.7778; 982.8230; 991.8734; 998.3418; 1011.1085; 1022.1987; 1040.5180; 1048.2548; 1061.2250; 1066.4993; 1067.8751; 1078.0052; 1088.6151; 1091.6954; 1143.9594; 1190.5378; 1196.6046; 1200.0338; 1202.8060; 1205.0775; 1225.6999; 1249.2572; 1258.6111; 1262.7487; 1263.5823; 1288.6165; 1296.5795; 1297.3903; 1318.6201; 1327.8362; 1370.2876; 1401.3525; 1434.6621; 1436.8988; 1442.9763; 1460.3339; 1461.5650; 1498.5595; 1503.9880; 1506.7615; 1514.7514; 1516.7785; 1524.4872; 1527.9681; 1530.6100; 1547.8318; 1616.7364; 1619.2426; 1631.7988; 1803.0222; 1873.0243; 3048.6406; 3068.8359; 3071.4600; 3077.7194; 3114.3605; 3129.5569; 3138.5628; 3140.1670; 3147.4995; 3148.2369; 3148.5905; 3150.3272; 3155.9201; 3163.9298; 3167.2176; 3177.5630; 3194.2942.

Cartesian Forces: Max 0.000001314 RMS 0.000000498

Item	Value	Threshold	Converged?
Maximum Force	0.000000	0.000450	YES
RMS Force	0.000000	0.000300	YES
Maximum Displacement	0.000025	0.001800	YES
RMS Displacement	0.000003	0.001200	YES

FILE: Yb_Sq2_PhYbOTf2_GS_Neutral_B3LYP_M062x_Conf14.out

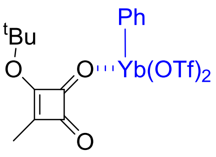	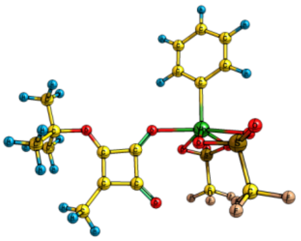	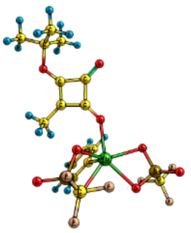	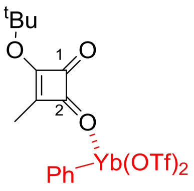
**Pre-reaction complex 1b•PhYb(OTf)$_2$:- chelation to C1 carbonyl only**	*Conformer 1*	*Conformer 2*	**Pre-reaction complex 1b•PhYb(OTf)$_2$:- chelation to C2 carbonyl only**
Lowest energy conformation is:<br>Conformer 2	E(RB3LYP/6-31G(d)/MWB59) = -2769.90190722<br>Convg = 0.6212D-08 -V/T = 2.0149<br><br>Temperature 195.150 Kelvin. Pressure 1.00000 Atm.<br>elec. and zero-point Energies= -2769.558834<br>elec. and thermal Energies= -2769.539188<br>elec. and thermal Enthalpies= -2769.538570<br>elec. and thermal Free Energies= -2769.603356<br><br>RB3LYP/6-31G(d)/MWB59/THF(IEFPCM) = -2769.93295062<br><br>RM06-2x/6-311+G(d,p)/MWB59/THF(IEFPCM) = -2769.73229651	E(RB3LYP/6-31G(d)/MWB59) = -2769.91620261<br>Convg = 0.2918D-09 -V/T = 2.0149<br><br>Temperature 195.150 Kelvin. Pressure 1.00000 Atm.<br>elec. and zero-point Energies= -2769.572643<br>elec. and thermal Energies= -2769.553207<br>elec. and thermal Enthalpies= -2769.552589<br>elec. and thermal Free Energies= -2769.616517<br><br>RB3LYP/6-31G(d)/MWB59/THF(IEFPCM) = -2769.94300220<br><br>RM06-2x/6-311+G(d,p)/MWB59/THF(IEFPCM) = -2769.74558905	Lowest energy conformation is:<br>Conformer 1
	Geometry at RB3LYP/6-31G(d)/MWB59<br>Charge = 0 Multiplicity = 1<br>Yb -2.6443594386 1.0536719144 1.7381753546<br>O -1.6737746359 2.3565309176 0.0122049932<br>S -2.0846534603 3.7147128986 0.555173852<br>O -1.0868146267 4.7680520319 0.5619494014<br>O -2.8041853676 3.3997056765 1.8505232289<br>C -3.4545681737 4.2534855455 -0.5885215249<br>F -2.9738012618 4.3961360346 -1.8227694727<br>F -4.4147074323 3.3205411831 -0.5913549052<br>F -3.9612625064 5.408677227 -0.1662518203<br>O -4.7576944461 1.1654703368 2.7637561486<br>O -5.8723435914 -1.0391705573 3.382430204<br>O -4.0925108023 -0.8100789119 1.5806039108<br>S -5.2480131983 -0.2124004225 2.3669042784<br>C -6.5371177425 0.1318358538 1.0643722569<br>F -7.5770914962 0.7502595189 1.61769538<br>F -6.0064326066 0.9131082695 0.1163742579<br>F -6.9341262648 -1.0169098198 0.5181525626<br>C -0.9781010474 -1.6679728261 -1.7918156813<br>C -1.8723939438 -1.3622048084 -2.8224725519<br>C -2.6686057534 -0.4591010292 -1.9484521731<br>C -1.6844797891 -0.7873107374 -0.8246286051<br>O -1.5098241774 -0.4864970746 0.3468188043<br>O -3.6593159974 0.219934115 -2.0700682431<br>O 0.086459139 -2.3629664184 -1.4994859014<br>C 0.8207504573 -3.2798031151 -2.4165828286<br>C -2.1170285722 -1.6579764697 -4.2567279094<br>H -1.9088849388 -2.6959923135 -4.5294572149<br>H -1.4943873193 -1.0142655907 -4.8931695215<br>H -3.1615785406 -1.430046546 -4.4925347187<br>C -1.169801033 0.7139612144 3.4913093668<br>C -0.4987361984 -0.5116214479 3.7026564392<br>C -0.918132235 1.7348716406 4.4362276749<br>H -0.6479553672 -1.3396733827 3.0082440462<br>H -1.4037631784 2.7054549216 4.3302014214<br>C 0.3636978605 -0.7120708324 4.7842206835<br>C -0.0560208216 1.5485750737 5.5210937598<br>H 0.8608188627 -1.6710067788 4.9171598631<br>H 0.1140193113 2.3577892338 6.2281383863<br>C 0.5867369208 0.3215270447 5.696753869<br>H 1.2571563765 0.1712175422 6.539865481<br>C 1.2975186395 -2.5040892495 -3.6445167186<br>H 1.8701166232 -1.6215033595 -3.342799954<br>H 1.9526744032 -3.145210291 -4.2433792495<br>H 0.4729044053 -2.1861015879 -4.2866366605<br>C -0.0858065052 -4.4642700193 -2.7523088687<br>H 0.4694687862 -5.1901236033 -3.3554007799<br>H -0.4193643561 -4.9635698076 -1.8375057451<br>H -0.968151613 -4.1630042571 -3.3234296753<br>C 2.0028031632 -3.7181309951 -1.551505346<br>H 2.6265732005 -4.4253496566 -2.1070886286<br>H 2.6169086995 -2.8574400954 -1.2706798982<br>H 1.6518595901 -4.2061381894 -0.6375880283<br><br>Frequency: 8.3827; 11.8194; 12.8582; 15.1701; 20.6152; 21.8757; 23.1480; 30.3512; 33.5585; 40.3291; 40.5817; 44.9937; 48.9180; 57.4570; 62.6703; 70.7592; 88.7546; 99.8615; 102.6979; 116.7358; 124.8348; 141.6234; 157.8768; 159.2558; 161.7573; 177.8957; 187.8925; 200.2235; 203.4311; 218.8670; 221.9224; 226.5254; 231.5713; 233.6162; 235.7710; 241.7750; 261.2309; 276.3872; 294.7320; 303.8823; 306.9563; 320.9375; 321.8211; 339.2081; 348.8571; 361.9619; 365.6396; 392.5250; 398.1483; 440.5949; 450.5359; 481.9543; 485.7038; 490.3210; 491.2944; 530.5381; 534.3581; 557.4708; 557.8684; 577.9046; 582.7719; 599.7336; 614.9964; 620.5890; 631.9359; 634.3682; 644.0105; 658.1751; 716.8274; 738.4651; 764.0818; 764.7398; 764.9872; 783.7244; 786.3338; 837.2481; 871.8096; 923.5429; 935.1707; 935.9340; 955.0884; 966.8813; 972.5415; 978.0122; 981.6363; 999.0487; 1008.2730; 1010.9999; 1040.5548; 1047.3521; 1060.1170; 1065.1503; 1065.8245; 1078.9749; 1087.9351; 1092.5338; 1130.9952; 1191.1339; 1193.7230; 1200.7985; 1201.8442; 1204.9830; 1225.0198; 1239.4305; 1252.1965; 1266.1990; 1269.1709; 1287.5390; 1296.3702; 1299.5793; 1326.1122; 1333.4590; 1369.8367; 1394.2089; 1433.5216; 1436.3410; 1440.3123; 1460.8765; 1461.1637; 1498.9263; 1503.2148; 1507.4198; 1514.7193; 1516.7215; 1524.4663; 1527.9720; 1530.7162; 1548.2108; 1617.3099; 1631.4350; 1632.3577; 1813.3524; 1890.5992; 3046.6678; 3068.2129; 3070.5287; 3076.9495; 3111.0889; 3135.1344; 3137.7210; 3139.1139; 3144.8343; 3144.8604; 3147.6848; 3148.9488; 3152.7348; 3162.2866; 3169.2263; 3179.6593; 3196.1008.<br><br>Cartesian Forces: Max 0.000000817 RMS 0.000000288<br><br>Item Value Threshold Converged?<br>Maximum Force 0.000000 0.000450 YES<br>RMS Force 0.000000 0.000300 YES<br>Maximum Displacement 0.000062 0.001800 YES<br>RMS Displacement 0.000013 0.001200 YES	Geometry at RB3LYP/6-31G(d)/MWB59<br>Charge = 0 Multiplicity = 1<br>Yb -5.2023121397 -1.058013565 -1.699569553<br>O -5.5479847549 -0.3317582414 -3.9384033966<br>S -4.3652896078 0.603623424 -4.0292417023<br>O -3.5116101794 0.5211314282 -5.2026623948<br>O -3.6817475876 0.4882878531 -2.6759954375<br>C -5.1206925468 2.3080706825 -3.9999499584<br>F -5.8865143855 2.4753303743 -5.07305066<br>F -5.8628924038 2.4359985019 -2.8937166791<br>F -4.1502753731 3.2202638952 -3.9884899544<br>O -7.5014227784 -0.5493116523 -1.56984954<br>O -8.3451494647 0.2964254304 0.6737468707<br>O -6.2639001997 -1.1333980242 0.3950894436<br>S -7.6554844798 -0.7404603838 -0.0723639777<br>C -8.6615418635 -2.3022308725 0.0976300417<br>F -9.8860909916 -2.0873422045 -0.3773244163<br>F -8.0803171388 -3.285480153 -0.5936604175<br>F -8.7284067337 -2.6413370055 1.3835184668<br>C -0.0843674772 -1.3508589656 -0.5202125755<br>C -1.1539524985 -1.5403419519 -1.4020246866<br>C -2.1275703222 -1.1674545268 -0.396061543<br>C -1.0052776062 -0.945306568 0.6205271958<br>O -0.9031537188 -0.6101623629 1.7720568653<br>O -3.3582761263 -1.0649175932 -0.3260523649<br>O 1.1832618963 -1.5085620596 -0.7488373822<br>C 2.2438991662 -1.2748831053 0.2950375513<br>C -1.2356782689 -1.9604665929 -2.8207612636<br>H -0.2688959314 -2.326460101 -3.1768538572<br>H -1.5447129423 -1.1147379906 -3.4479411092<br>H -1.9922127213 -2.7438139216 -2.9471701556<br>C -4.8570415562 -3.2784534487 -2.3252745811<br>C -4.9606769723 -3.7434259178 -3.65672097<br>C -4.5219131547 -4.2500140683 -1.3529437748<br>H -5.2240443828 -3.0478500921 -4.4531813142<br>H -4.4347289783 -3.9616333009 -0.3035231611<br>C -4.7411345151 -5.0821211082 -3.9979827625<br>C -4.3025210882 -5.5920073187 -1.6794300672<br>H -4.8310341005 -5.4022474183 -5.0339836357<br>H -4.0525975968 -6.310760976 -0.9015007884<br>C -4.4107383432 -6.0099967541 -3.0078197802<br>H -4.2421416444 -7.0522125319 -3.2687701893<br>C 2.0165502728 -2.2477935543 1.4508627521<br>H 1.9689930112 -3.2792540964 1.08665657<br>H 2.8562694305 -2.1743771178 2.1501415193<br>H 1.1028313217 -2.0115839279 2.0020715602<br>C 2.191001979 0.1894002577 0.7278118332<br>H 3.0406596897 0.3979994605 1.386668184<br>H 2.2644253703 0.8535417459 -0.1395144085<br>H 1.2765463951 0.4124876456 1.2832167095<br>C 3.5220786534 -1.5949503032 -0.4763793683<br>H 4.3885398912 -1.4621878331 0.1787914178<br>H 3.5133153789 -2.6294522808 -0.8331593243<br>H 3.634266118 -0.9293278097 -1.3376858303<br><br>Frequency: 7.7300; 11.0702; 13.4610; 19.2728; 22.4555; 23.9437; 26.6665; 30.2027; 33.6434; 39.3134; 43.1299; 48.4816; 56.6880; 63.0663; 67.1284; 71.6101; 100.1693; 105.9299; 118.7204; 128.6884; 134.7934; 143.8671; 156.2884; 161.2279; 163.5413; 171.3170; 188.7989; 205.1986; 211.0160; 214.2918; 217.4445; 220.3263; 227.1876; 232.0540; 237.5247; 254.6830; 274.0137; 274.8066; 303.9926; 305.9819; 318.0937; 319.9396; 322.9764; 346.2190; 353.7981; 362.0440; 364.9902; 393.8970; 405.7365; 441.5906; 452.7038; 481.0195; 490.8916; 491.3724; 492.2271; 530.2666; 534.7403; 557.7369; 558.3432; 578.2307; 582.3035; 600.1404; 618.5189; 625.3722; 629.6584; 632.0243; 633.5545; 656.1857; 720.7414; 739.2925; 750.7252; 765.9885; 766.5722; 774.3185; 789.0579; 844.3579; 877.1786; 927.7725; 937.7980; 943.6906; 959.0751; 965.2826; 967.0064; 977.8263; 990.7523; 1002.0936; 1010.5829; 1011.3765; 1039.0371; 1055.2116; 1065.2458; 1068.6868; 1069.7507; 1081.8542; 1086.6251; 1091.6988; 1094.5184; 1191.1501; 1195.5598; 1201.3137; 1202.4397; 1224.2286; 1247.6547; 1253.9835; 1264.5445; 1267.8651; 1272.5331; 1290.3529; 1296.4882; 1307.7755; 1318.8769; 1327.2613; 1368.5699; 1416.3792; 1435.7345; 1435.8476; 1452.2370; 1460.3570; 1462.4520; 1499.1957; 1503.8202; 1508.1194; 1516.6201; 1517.8883; 1522.9318; 1535.4133; 1535.6263; 1552.6734; 1606.0564; 1615.9605; 1630.0834; 1789.7381; 1884.3662; 3053.6890; 3064.8131; 3064.9471; 3071.8992; 3112.8027; 3125.7521; 3132.0664; 3133.6605; 3143.2531; 3145.5521; 3146.8196; 3154.4801; 3156.7532; 3160.2150; 3170.7781; 3180.4114; 3197.0062.<br><br>Cartesian Forces: Max 0.000001019 RMS 0.000000343<br><br>Item Value Threshold Converged?<br>Maximum Force 0.000000 0.000450 YES<br>RMS Force 0.000000 0.000300 YES<br>Maximum Displacement 0.000029 0.001800 YES<br>RMS Displacement 0.000005 0.001200 YES	
	FILE: Yb_Sq2_PhYbOTf2_GS_Neutral_B3LYP_M062x_Conf15.out	FILE: Yb_Sq2_PhYbOTf2_GS_Neutral_B3LYP_M062x_Conf16.out	

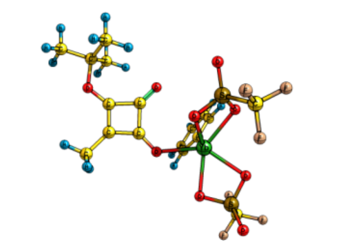

Conformer 1

E(RB3LYP/6-31G(d)/MWB59) = -2769.91493000
Convg = 0.2914D-09 -V/T = 2.0149

Temperature 195.150 Kelvin. Pressure 1.00000 Atm.
elec. and zero-point Energies= -2769.571746
elec. and thermal Energies= -2769.552082
elec. and thermal Enthalpies= -2769.551464
elec. and thermal Free Energies= -2769.616369

RB3LYP/6-31G(d)/MWB59/THF(IEFPCM) = -2769.94363087

RM06-2x/6-311+G(d,p)/MWB59/THF(IEFPCM) = -2769.74289588

Geometry at RB3LYP/6-31G(d)/MWB59
Charge = 0 Multiplicity = 1

```
Yb 1.6303787273 0.4151968983 3.1562646975
O 2.5204397004 1.2765608751 1.1036309074
S 3.9822479353 1.020738869 1.4263583343
O 4.8102187447 0.4378722407 0.3858450474
O 3.9588985764 0.3475045598 2.7825839655
C 4.6603430298 2.7220173907 1.7770662982
F 4.6626691293 3.4355979568 0.651041568
F 3.8859240667 3.3285793728 2.6832126991
F 5.9002330557 2.6205160592 2.2477072028
O -0.0732899341 1.5567662669 4.3122599045
O 0.6386421662 3.6131378559 5.6212462906
O 2.2008832905 1.6903746719 5.053408925
S 0.8061038769 2.1915626602 5.3767361522
C 0.3432072143 1.3084477218 6.9534459144
F -0.9316430519 1.5691826151 7.2399927827
F 0.5053941958 -0.0062590707 6.7970348177
F 1.1246186693 1.7402983824 7.9407325034
C -0.2190539601 -1.20717988 -1.3311516774
C -1.0633870972 -0.3823632083 -0.5799873046
C -0.1305206519 -0.3166619162 0.5218937408
C 0.8369499775 -1.2298781013 -0.2330861053
O 1.9114715202 -1.7314878123 -0.033104782
O -0.1147167999 0.2407948414 1.625623002
O -0.405048074 -1.6947725722 -2.5172931157
C 0.6131524848 -2.5655061327 -3.2172905211
C -2.4008574438 0.2129926063 -0.8160263359
H -2.7838611065 -0.0476849115 -1.805939934
H -2.3530883868 1.3051974192 -0.7282607483
H -3.1133369942 -0.1340771022 -0.0575503259
C 1.4373655874 -1.8241430551 3.7771854875
C 0.1789776739 -2.3778017485 4.1092355155
C 2.542504401 -2.7011993941 3.8644745958
H -0.7173813799 -1.7551307671 4.0684104292
H 3.5405618959 -2.3382716938 3.6190749167
C 0.0267155955 -3.7112308663 4.502641594
C 2.4061318842 -4.0361303159 4.2571814932
H -0.9585781038 -4.0991526131 4.7544685847
H 3.2814122484 -4.6801729435 4.3140983968
C 1.1451294764 -4.5443006464 4.5773815271
H 1.0349163047 -5.5817535213 4.8850927684
C 0.8090143912 -3.8400061584 -2.3985763774
H -0.1497491844 -4.3358968544 -2.2150368838
H 1.4444373433 -4.5308756994 -2.962835502
H 1.30458487 -3.6372896692 -1.4458840419
C 1.9009402423 -1.7640708121 -3.3972324267
H 2.5941871669 -2.3407704115 -4.0187361654
H 1.701035144 -0.8153465117 -3.9058450963
H 2.3968123923 -1.5693522106 -2.443025322
C -0.0769957267 -2.8437968454 -4.5501783033
H 0.5648199496 -3.4793625773 -5.1679449196
H -1.0295422225 -3.3602183839 -4.3964169283
H -0.2667988101 -1.9129018566 -5.0933202461
```

Frequency: 6.8787; 11.6153; 12.6273; 18.1577; 23.1194; 24.2849; 26.9221; 27.5870; 32.2121; 38.2641; 39.9990; 48.1854; 49.6365; 50.7919; 60.0071; 63.8145; 66.5021; 106.3546; 114.8333; 119.1236; 129.8322; 134.5548; 149.1071; 159.5068; 161.5401; 168.2761; 190.7396; 203.3268; 205.8126; 212.5192; 217.7576; 220.1259; 224.8351; 229.0123; 236.5962; 253.7928; 274.0541; 274.8013; 303.9314; 305.7357; 319.9268; 321.8700; 329.2007; 346.4316; 352.1143; 361.9589; 365.8540; 392.8888; 397.1092; 441.8499; 451.3712; 476.1203; 490.2637; 491.5095; 492.2499; 530.3271; 534.5544; 557.4236; 558.5231; 578.0248; 581.6317; 597.6048; 616.8367; 624.1731; 628.0574; 631.3363; 632.5425; 656.7385; 718.9515; 738.7283; 754.2256; 765.0842; 766.6177; 779.9500; 789.5614; 838.4971; 874.1338; 923.8527; 937.4790; 942.9822; 958.9694; 962.0098; 966.5811; 973.3043; 989.6634; 999.1055; 1010.7668; 1014.2127; 1039.7295; 1056.8407; 1065.9667; 1068.0814; 1068.3005; 1077.8776; 1087.4245; 1090.2686; 1091.9378; 1190.3713; 1192.8540; 1202.0685; 1204.0930; 1224.6758; 1247.6685; 1256.0596; 1265.8154; 1267.3066; 1267.9234; 1290.8260; 1297.4807; 1307.1607; 1321.7950; 1328.4514; 1369.4962; 1418.0740; 1436.0196; 1436.7103; 1459.0460; 1460.2441; 1463.7108; 1501.0183; 1503.4133; 1505.1044; 1516.0056; 1517.2656; 1523.7159; 1535.0702; 1535.2981; 1552.3057; 1610.3041; 1616.5183; 1630.8783; 1794.2318; 1885.3690; 3053.8755; 3064.9753; 3065.7095; 3072.2762; 3107.8544; 3119.8818; 3132.4240; 3134.0888; 3142.5079; 3145.8258; 3150.6327; 3150.6477; 3158.3072; 3161.7712; 3167.0873; 3177.4290; 3194.1655.

Cartesian Forces: Max 0.000001421 RMS 0.000000571

Item	Value	Threshold	Converged?
Maximum Force	0.000000	0.000450	YES
RMS Force	0.000000	0.000300	YES
Maximum Displacement	0.000013	0.001800	YES
RMS Displacement	0.000003	0.001200	YES

FILE: Yb_Sq2_PhYbOTf2_GS_Neutral_B3LYP_M062x_Conf17.out

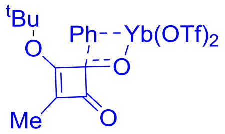

Trapezoidal Transition State for $PhYb(OTf)_2$ addition to C-1 carbonyl of 1b

Lowest energy conformation is:
Conformer 10

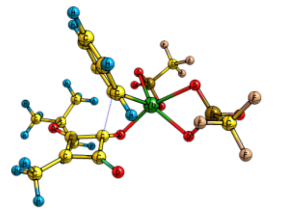

Conformer 1

E(RB3LYP/6-31G(d)/MWB59) = -2769.89154003
Convg = 0.7004D-09 -V/T = 2.0149

Temperature 195.150 Kelvin. Pressure 1.00000 Atm.
elec. and zero-point Energies= -2769.548541
elec. and thermal Energies= -2769.529759
elec. and thermal Enthalpies= -2769.529141
elec. and thermal Free Energies= -2769.589710

RB3LYP/6-31G(d)/MWB59/THF(IEFPCM) = -2769.91384919

RM06-2x/6-311+G(d,p)/MWB59/THF(IEFPCM) = -2769.71639792

Geometry at RB3LYP/6-31G(d)/MWB59
Charge = 0 Multiplicity = 1

```
Yb 1.0581537111 2.1671776752 0.5514450042
O 3.1616257435 2.0048264472 -0.4370657472
S 4.0066395828 2.0731086631 0.831092353
O 5.0317007082 1.0564829144 0.9904207237
O 2.9918295707 2.2434396481 1.9404964487
C 4.8698487247 3.7238121304 0.713342781
F 5.7060289682 3.7041983161 -0.3222079643
F 3.9619940348 4.6852526136 0.5383538293
F 5.5450754384 3.9421093821 1.8378673413
O 0.6529073155 4.4618397024 0.2667581068
O 0.3001377393 5.7340777102 2.440267713
O -0.2443457393 3.2666550323 2.1396200568
S -0.1281769627 4.6664792494 1.556887025
C -1.8594919812 5.0828499154 0.9969201918
F -1.840062072 6.2509872994 0.3600148816
F -2.3043934332 4.1324819539 0.1693409801
F -2.6544634114 5.1570500662 2.0591809203
C -0.2273511449 -1.7300348777 -0.257409238
C -1.6099180924 -1.7165968934 -0.2596209278
C -1.6714147351 -0.3971305721 0.3949469046
C -0.1348264013 -0.375473906 0.4044151333
O 0.6988409762 0.1128206058 1.2354734016
O -2.5221087282 0.3534240952 0.8172924154
O 0.5727913858 -2.6711817223 -0.7035730941
C 1.9560680892 -2.9130187143 -0.1914235567
C -2.6452841543 -2.6826500778 -0.700814521
H -2.211542525 -3.6652154545 -0.908213801
H -3.1354207308 -2.3225152556 -1.6155797092
H -3.4275521776 -2.7856585075 0.0596942123
C 2.8866782393 -1.8005039166 -0.6640141316
H 3.9150416374 -2.0314511987 -0.3671023546
H 2.8568702451 -1.7080210472 -1.7539298239
H 2.6286680982 -0.8406246565 -0.2143226099
C 2.3087749037 -4.2474566135 -0.8482377891
H 3.3211774813 -4.5462153094 -0.5588397262
H 1.6120549334 -5.0310012308 -0.5345992502
H 2.2705754475 -4.1637630154 -1.9388597631
C 1.9082352888 -3.0329190306 1.3323794817
H 1.6475753317 -2.0814438187 1.8040520647
H 1.1866030214 -3.7979393076 1.6380566018
H 2.8952187204 -3.3290480779 1.7030189219
C -0.0262558685 1.1881547301 -1.4100051664
C 0.909215763 0.8599923641 -2.4179351784
C -1.2770514627 1.689394672 -1.8407476974
H 1.8899239664 0.4753851876 -2.1494135906
H -2.0470080783 1.9283026938 -1.1123227283
C 0.6248235545 1.0395142508 -3.7755415768
C -1.5651846947 1.8767663411 -3.1942986192
H 1.3693281539 0.7820139805 -4.5251864632
H -2.5316629905 2.2763535628 -3.4922935828
C -0.6150088029 1.5477900001 -4.1652820414
H -0.8409855874 1.6878060012 -5.2193258415
```

Frequency: -190.4119; 13.1355; 20.0148; 22.5931; 23.2100; 24.5537; 31.8938; 36.2390; 40.5482; 43.5774; 50.7107; 53.8856; 57.2469; 64.7173; 68.4561; 77.1282; 79.1832; 87.2047; 99.3771; 110.3965; 134.3253; 142.4297; 154.7529; 159.4234; 171.0023; 175.2076; 188.5247; 191.5019; 208.3807; 214.9906; 219.1959; 222.1489; 234.2451; 245.9184; 260.6824; 274.0645; 276.9071; 293.7465; 304.0199; 306.2842; 317.3715; 319.0463; 320.4175; 341.5411; 347.7745; 364.5553; 367.2970; 393.7786; 400.4730; 443.4930; 450.7635; 484.0526; 489.5651; 490.4678; 510.9607; 532.0946; 537.0942; 557.4737; 558.3323; 576.8494; 579.7991; 591.4336; 620.0817; 626.3349; 627.2129; 633.9789; 637.9034; 645.7614; 719.8791; 728.0218; 734.5269; 763.5688; 766.6372; 767.1056; 789.0043; 852.8690; 873.7619; 935.8653; 937.5855; 943.8984; 952.7412; 959.2793; 961.8076; 985.5285; 988.4545; 991.8254; 1008.2408; 1009.5331; 1015.1851; 1053.2885; 1054.7572; 1063.0753; 1064.5308; 1067.8320; 1072.2508; 1075.8128; 1100.4099; 1194.3433; 1197.3845; 1199.2893; 1200.0128; 1227.0693; 1240.3122; 1251.9699; 1258.6117; 1271.3932; 1273.5160; 1289.3877; 1300.4609; 1303.3767; 1320.8732; 1329.5012; 1372.6431; 1394.1836; 1430.6882; 1433.7544; 1442.4930; 1458.8430; 1463.3209; 1501.9301; 1502.6135; 1504.6557; 1513.2980; 1517.3014; 1519.7372; 1527.9044; 1531.3885; 1543.3540; 1549.6178; 1614.8047; 1622.6625; 1665.6958; 1867.7611; 3044.5410; 3062.3600; 3065.9808; 3075.8064; 3102.2170; 3131.2619; 3135.8341; 3140.3932; 3140.8519; 3144.0618; 3151.3242; 3174.5194; 3176.9004; 3184.4435; 3196.0009; 3202.8831; 3208.6884.

Cartesian Forces: Max 0.000000526 RMS 0.000000193

Item	Value	Threshold	Converged?
Maximum Force	0.000001	0.000450	YES
RMS Force	0.000000	0.000300	YES
Maximum Displacement	0.000024	0.001800	YES
RMS Displacement	0.000005	0.001200	YES

FILE:
Yb_Sq2_PhYbOTf2_TS_Neutral_Add12_B3LYP_M062x_Conf1.out

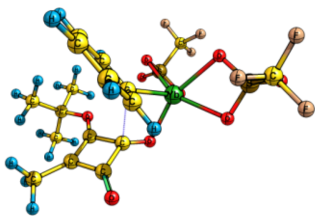

Conformer 2

E(RB3LYP/6-31G(d)/MWB59) = -2769.89155939
Convg = 0.9677D-08 -V/T = 2.0149

Temperature 195.150 Kelvin. Pressure 1.00000 Atm.
elec. and zero-point Energies= -2769.548645
elec. and thermal Energies= -2769.529842
elec. and thermal Enthalpies= -2769.529224
elec. and thermal Free Energies= -2769.589888

RB3LYP/6-31G(d)/MWB59/THF(IEFPCM) = -2769.91504826

RM06-2x/6-311+G(d,p)/MWB59/THF(IEFPCM) = -2769.71802783

Geometry at RB3LYP/6-31G(d)/MWB59
Charge = 0 Multiplicity = 1

```
Yb 0.7391735917 1.8794226214 0.5049300117
O 2.6048803238 1.0769530755 -0.6757962798
S 3.5058428901 0.8087905037 0.5209740451
O 4.1428354799 -0.4961074944 0.5891487257
O 2.6910889449 1.2639137274 1.7135525143
C 4.8694229133 2.0737257896 0.3644768134
F 5.5739117276 1.8173566589 -0.736709229
F 4.338692343 3.2936980218 0.282250825
F 5.6578180259 1.9975802164 1.4334091467
O 1.2306622658 4.166085885 0.1947663547
O 1.0642679142 5.5593197001 2.3156574516
O -0.2784684168 3.4382886648 1.9087585834
S 0.4092834979 4.6827549835 1.3633794057
C -0.9674911 5.6655204794 0.5749901169
F -0.4492785355 6.732848683 -0.0265428213
F -1.6004006822 4.9088210041 -0.3262575222
F -1.8254997068 6.0463816011 1.517152331
C -0.6422719669 -1.6194216007 -0.3610659869
C -1.9796575303 -1.9629289712 -0.5061263995
C -2.4348046769 -0.7390688809 0.2024058405
C -0.9514493268 -0.3932319481 0.43052625
O -0.3072902922 0.1592598526 1.3679467293
O -3.5018205957 -0.2703443196 0.5151757219
O 0.5816951286 -1.9944863123 -0.6262401885
C 0.9884046168 -3.2322552864 -1.34289284
C -2.8363128507 -3.040389155 -1.0707448595
H -2.4442627488 -4.0460330871 -0.8919886109
H -2.955672593 -2.9149803072 -2.1555661502
H -3.8336713491 -2.9688985439 -0.6248236948
C 0.6640994811 -4.4311261414 -0.4507347713
H 1.0157375902 -5.3515218376 -0.9287932447
H 1.1648428979 -4.3341144883 0.5173889339
H -0.4110345994 -4.5336369402 -0.2767326272
C 2.4983505806 -3.0419333992 -1.4903671841
H 2.9399060549 -3.9321203943 -1.9500669434
H 2.719609431 -2.1763718816 -2.1218160059
H 2.9670365353 -2.876009161 -0.516661531
C 0.3047452325 -3.290142749 -2.7095679479
H -0.7673812965 -3.4849627549 -2.6346866933
H 0.4536722303 -2.3516314315 -3.2524705006
H 0.7478131925 -4.0999575247 -3.2987731829
C -0.6925226794 1.2850567719 -1.3470138645
C -0.035013545 0.9226685208 -2.547058513
C -1.9539724372 1.9110768617 -1.4748621544
H 0.9389052062 0.4384524571 -2.5042908526
H -2.520508777 2.182469624 -0.5858038829
C -0.5925931582 1.1903562428 -3.799581948
C -2.5194680058 2.1804263462 -2.7246839433
H -0.0569903714 0.9153435808 -4.7056464153
H -3.4910949156 2.6641327417 -2.7895977418
C -1.8386965045 1.8182491913 -3.8888364126
H -2.2762314344 2.0244708045 -4.8625158578
```

Frequency: -165.0129; 13.8153; 19.5675; 20.6646; 22.6891; 28.3751; 30.9119; 35.6218; 38.2060; 42.2959; 43.2788; 52.3435; 55.6672; 61.9762; 66.8065; 71.5845; 84.1203; 99.3794; 118.9143; 126.6833; 138.6685; 139.1802; 155.6799; 157.0875; 165.8193; 172.3732; 188.0806; 193.1125; 209.1231; 215.4569; 220.1714; 225.8624; 231.4553; 235.5283; 244.6821; 248.1785; 259.2204; 296.1127; 304.1708; 306.2038; 319.5073; 320.3994; 329.5068; 341.9921; 349.9110; 363.3195; 366.9427; 394.2207; 397.7367; 436.9771; 454.7000; 482.3039; 489.8357; 490.3493; 509.0472; 531.2537; 536.3312; 558.0390; 558.6009; 576.2036; 579.8884; 596.6844; 620.9941; 626.1056; 627.0427; 633.9783; 640.7430; 646.2166; 717.1651; 732.1547; 763.1025; 763.8369; 766.4623; 766.7773; 782.1447; 838.6170; 871.8665; 935.3353; 936.3665; 936.9143; 953.7655; 961.9688; 968.6499; 981.6371; 982.4165; 994.8020; 1006.0334; 1010.9060; 1018.1801; 1050.0641; 1058.3354; 1060.1384; 1067.1020; 1069.1540; 1072.7171; 1093.9013; 1115.1820; 1193.2756; 1193.7030; 1199.8806; 1200.6947; 1203.9788; 1224.1079; 1253.0259; 1259.3149; 1268.3822; 1272.2786; 1284.7216; 1296.3897; 1302.1740; 1316.3031; 1328.6565; 1370.1558; 1388.8303; 1432.5787; 1437.0075; 1439.7508; 1461.4733; 1462.3920; 1500.4024; 1505.5965; 1510.0462; 1514.1901; 1516.3427; 1517.8002; 1527.8224; 1531.0665; 1545.4353; 1574.5620; 1613.6240; 1622.5637; 1680.0177; 1881.9921; 3043.5058; 3066.6446; 3068.9336; 3075.9298; 3107.0330; 3135.7935; 3136.5364; 3138.3572; 3144.6903; 3146.5824; 3155.9107; 3163.2811; 3167.4589; 3169.9639; 3182.3466; 3190.6548; 3204.2711.

Cartesian Forces: Max 0.000001271 RMS 0.000000405

Item	Value	Threshold	Converged?
Maximum Force	0.000001	0.000450	YES
RMS Force	0.000000	0.000300	YES
Maximum Displacement	0.000063	0.001800	YES
RMS Displacement	0.000012	0.001200	YES

FILE: Yb_Sq2_PhYbOTf2_TS_Neutral_Add12_B3LYP_M062x_Conf1-
.out

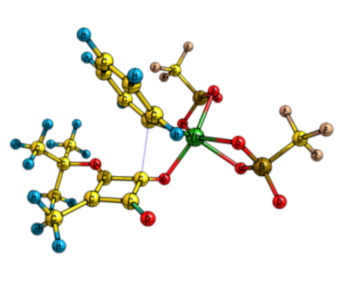

Conformer 3

E(RB3LYP/6-31G(d)/MWB59) = -2769.88921452
Convg = 0.3072D-08 -V/T = 2.0149

Temperature 195.150 Kelvin. Pressure 1.00000 Atm.
elec. and zero-point Energies= -2769.546388
elec. and thermal Energies= -2769.527532
elec. and thermal Enthalpies= -2769.526914
elec. and thermal Free Energies= -2769.588307

RB3LYP/6-31G(d)/MWB59/THF(IEFPCM) = -2769.91492204

RM06-2x/6-311+G(d,p)/MWB59/THF(IEFPCM) = -2769.71649579

Geometry at RB3LYP/6-31G(d)/MWB59
Charge = 0 Multiplicity = 1
Yb 1.5461249159 1.7447125274 -0.2273188612
O 1.6303435764 3.3348203618 1.5168514169
S 0.8629428547 4.4305294295 0.7989207573
O -0.227197261 5.0735648459 1.5068271912
O 0.5586239334 3.8294000357 -0.5676514255
C 2.1403667338 5.7451645365 0.4451482924
F 2.570900492 6.2533381388 1.5971692386
F 3.1647654613 5.2069994161 -0.2185932051
F 1.5845867698 6.7037967824 -0.2929147878
O 3.4156601034 0.401297288 0.2295394399
O 5.6300536404 1.6486200355 0.1515459016
O 3.6056917283 2.2721135559 -1.2556109004
S 4.4369453943 1.2153890542 -0.5511844932
C 5.0073577123 0.0721545864 -1.9109277867
F 5.6288269528 -0.9728459571 -1.3682619167
F 3.9560516487 -0.3566628993 -2.6177056107
F 5.8403829231 0.7293499672 -2.7123527377
H -2.1950789394 -3.5345193435 -1.8352989994
C -1.0250246517 -1.4098190536 -0.1674411184
C -2.3649487981 -1.0887167099 -0.3381602978
C -0.6454381795 -0.010533336 0.1935622088
C -2.117212879 0.3499810853 -0.0625444987
O 0.24989042 0.4314980844 0.9664339866
O -2.792641639 1.3482045032 -0.0071414368
O -0.1704406787 -2.4022882988 -0.1870424741
C -0.4948953155 -3.8209970935 -0.4791709173
C -3.6743069099 -1.7194313462 -0.6566880049
H -3.8204094227 -2.6923515164 -0.1788264153
H -3.7841504692 -1.8580812228 -1.7409874985
H -4.4764854619 -1.0473100039 -0.3348851763
C -1.3381258416 -4.3750876031 0.6703718744
H -2.3052773195 -3.8714986881 0.7496217812
H -1.5277995449 -5.4413843772 0.507819661
H -0.8103637582 -4.2611540522 1.6222802343
C 0.8891595193 -4.4716585042 -0.5063350742
H 0.7910942091 -5.5493302653 -0.6710829079
H 1.4991647933 -4.0513201135 -1.311785561
H 1.4091955087 -4.3092990995 0.4423300344
C -1.1777498052 -3.9311926416 -1.8435017761
H -0.6014952084 -3.3964160078 -2.6048353207
H -1.2363584002 -4.9855842142 -2.1335706011
C 0.2025103715 0.7653304486 -1.9869757568
C 0.7517268508 -0.3274675391 -2.697894183
C -0.6803842888 1.6101819222 -2.6986528349
H 1.4257384091 -1.020136074 -2.1954664511
H -1.1414654112 2.4571990867 -2.1952789235
C 0.4592504155 -0.5535380285 -4.0459082703
C -0.9818332776 1.3887066164 -4.0452744411
H 0.9111392253 -1.3922752951 -4.5714450364
H -1.6591866606 2.0598298872 -4.5680250019
C -0.4132598812 0.3053515324 -4.7200904408
H -0.6464525624 0.1323625554 -5.7678448753

Frequency: -156.5661; 9.3327; 13.1033; 17.7819; 20.4677; 27.2515; 30.5212; 36.2147; 37.9289; 40.0515; 44.9644; 52.2854; 56.0848; 61.8620; 66.3805; 76.1941; 88.9477; 98.8770; 112.3820; 121.2023; 130.3016; 141.6794; 151.8630; 159.4083; 166.2284; 170.3138; 188.5114; 192.8772; 206.9203; 217.0736; 221.7150; 228.4909; 232.4935; 236.0266; 242.8554; 250.2395; 263.5312; 291.0580; 303.6804; 306.1704; 318.9802; 320.0802; 330.8742; 337.8255; 350.6667; 364.4312; 366.2669; 395.2179; 397.6757; 435.7389; 454.9798; 482.9564; 489.4959; 490.3169; 508.0232; 531.8265; 536.4210; 557.8732; 558.1780; 575.8215; 580.4341; 599.7473; 616.7905; 626.2650; 626.9996; 634.6669; 641.2831; 646.9774; 716.6572; 730.7781; 763.2800; 763.8981; 766.1281; 766.2616; 783.2935; 842.4383; 871.1638; 933.3894; 935.1420; 936.9517; 953.1540; 959.9729; 970.7892; 980.2987; 981.6317; 995.8789; 1005.9160; 1010.4790; 1018.0212; 1049.5511; 1056.6323; 1060.4186; 1064.2133; 1065.5363; 1072.2320; 1093.1559; 1109.7661; 1192.6177; 1193.3160; 1200.1052; 1202.3605; 1205.3411; 1223.5194; 1248.9555; 1257.9815; 1268.5668; 1270.5913; 1285.8925; 1296.1368; 1302.6272; 1324.8345; 1331.6729; 1369.9992; 1386.7829; 1432.8190; 1435.0689; 1439.7488; 1459.9015; 1462.1402; 1500.4075; 1505.0292; 1510.9741; 1514.9940; 1518.0488; 1518.1394; 1527.7789; 1530.8699; 1547.7672; 1581.3353; 1613.4467; 1621.9459; 1679.4760; 1882.7658; 3043.4946; 3065.8424; 3069.1005; 3075.8159; 3107.6992; 3136.4989; 3136.5789; 3139.2765; 3143.7426; 3146.9260; 3151.9610; 3156.2700; 3166.1236; 3175.4725; 3181.6704; 3192.1991; 3203.9131.

Cartesian Forces: Max 0.000004786 RMS 0.000000884

Item	Value	Threshold	Converged?
Maximum Force	0.000005	0.000450	YES
RMS Force	0.000001	0.000300	YES
Maximum Displacement	0.000559	0.001800	YES
RMS Displacement	0.000097	0.001200	YES

FILE: Yb_Sq2_PhYbOTf2_TS_Neutral_Add12_B3LYP_M062x_Conf2-.out

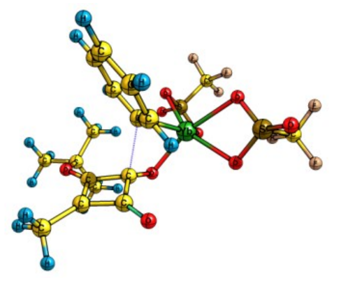

Conformer 4

E(RB3LYP/6-31G(d)/MWB59) = -2769.89228146
Convg = 0.4534D-08 -V/T = 2.0149

Temperature 195.150 Kelvin. Pressure 1.00000 Atm.
elec. and zero-point Energies= -2769.549497
elec. and thermal Energies= -2769.530657
elec. and thermal Enthalpies= -2769.530039
elec. and thermal Free Energies= -2769.590812

RB3LYP/6-31G(d)/MWB59/THF(IEFPCM) = -2769.91249495

RM06-2x/6-311+G(d,p)/MWB59/THF(IEFPCM) = -2769.71596594

Geometry at RB3LYP/6-31G(d)/MWB59
Charge = 0 Multiplicity = 1
Yb 1.0825891044 2.1078077824 0.6308653747
O 3.1983383301 1.875185283 -0.3465968368
S 4.0252514984 1.8986732285 0.9316930786
O 5.0083733741 0.8423819676 1.1001191926
O 3.0055084986 2.0997785019 2.0336205184
C 4.9584266638 3.5126265135 0.8438829819
F 5.8218379026 3.4616361614 -0.1674320715
F 4.0980454762 4.51359095 0.647949793
F 5.6106977103 3.7004624962 1.9882623816
O -0.4916551461 3.2650459544 1.8814100017
O -1.3697351555 5.4435309057 0.9048970773
O 0.8900768569 4.4379825313 0.3122995275
S -0.2250515262 4.6558768097 1.3198470927
C 0.5965425096 5.559485374 2.7312919587
F -0.279555536 5.7219266109 3.7180281705
F 1.6350815242 4.8402753452 3.1684889972
F 1.0239253694 6.7441166531 2.3027977457
C -0.1905546387 -1.7526882756 -0.2626674229
C -1.5715663655 -1.7730025069 -0.3071033502
C -1.6900674882 -0.4793315619 0.3902530418
C -0.1514395706 -0.4176692193 0.44452394
O 0.6481969266 0.0669215935 1.3110626443
O -2.5767534785 0.2218505009 0.8190104516
O 0.6411938385 -2.6643909274 -0.7149573041
C 2.0183957388 -2.8915044805 -0.1833766323
C -2.5680002027 -2.7512393777 -0.8066200763
H -2.1042850139 -3.717727694 -1.0245787193
H -3.0370793392 -2.3787054832 -1.7274358032
H -3.3717762895 -2.8913747473 -0.0750413533
C 2.9373495617 -1.7647131819 -0.6430496248
H 3.9619471299 -1.9633333531 -0.3122500622
H 2.9378505217 -1.6861725642 -1.7346691623
H 2.6430443306 -0.8050266004 -0.2170125798
C 2.3981893538 -4.2191160981 -0.8392455502
H 3.4135589757 -4.5009573746 -0.5431851324
H 1.7127408871 -5.0149163666 -0.5315870804
H 2.3662733074 -4.1345226635 -1.9299898078
C 1.9549832358 -3.0181650177 1.3393105164
H 1.6797557064 -2.0720284959 1.8130076013
H 1.2376030477 -3.7917780038 1.6333904737
H 2.9407369418 -3.3069494215 1.7188739176
C -0.025345864 1.2093126907 -1.3223836806
C 0.8065136903 0.855060595 -2.4080659649
C -1.2521483977 1.8433999821 -1.6324395182
H 1.7648462651 0.3745349134 -2.2263454429
H -1.9445453431 2.1125637642 -0.8372878129
C 0.443698049 1.1275498829 -3.7299324373
C -1.6161086514 2.1259993574 -2.9516990835
H 1.1067788693 0.8469468895 -4.5449288422
H -2.5594116727 2.6254254309 -3.158378029
C -0.7701055588 1.762611442 -4.0025349104
H -1.0546070705 1.9762985258 -5.0296954707

Frequency: -186.4788; 15.8084; 17.7836; 21.3249; 25.3197; 28.2697; 28.6590; 34.0862; 37.5319; 41.7459; 48.3563; 51.2751; 58.8459; 61.9740; 68.2002; 69.8550; 76.5829; 85.7269; 96.3148; 111.7552; 133.0319; 139.9883; 158.4634; 164.7654; 167.0635; 174.9597; 189.7570; 197.5969; 206.9554; 215.5408; 217.0266; 222.7740; 233.1369; 240.7863; 258.6907; 272.3522; 275.2124; 293.7253; 303.1796; 306.4907; 317.1485; 319.5245; 321.0572; 339.7729; 347.8766; 362.5067; 367.0830; 391.6550; 395.7348; 444.6044; 451.3526; 484.1560; 490.3171; 491.1844; 511.3737; 531.5938; 536.5678; 557.9751; 558.3578; 576.4635; 582.0762; 589.6006; 619.4715; 622.4755; 626.3145; 634.5212; 639.0893; 643.2388; 717.8599; 727.9131; 734.3076; 762.4890; 765.9245; 766.7580; 788.8459; 853.5123; 871.3876; 935.6599; 936.3796; 940.5489; 951.4396; 959.3615; 962.6903; 983.9734; 985.6110; 987.9058; 1008.5849; 1009.3611; 1015.6057; 1053.0610; 1057.1556; 1062.1795; 1062.7949; 1068.0238; 1070.5515; 1074.4355; 1099.5182; 1194.4462; 1197.4323; 1199.1366; 1200.7647; 1224.4892; 1241.7896; 1251.5858; 1256.7324; 1270.8210; 1274.0767; 1289.9068; 1299.4976; 1303.1319; 1318.6457; 1331.2957; 1369.6119; 1392.3729; 1430.4886; 1433.8286; 1441.9039; 1458.8007; 1463.3100; 1501.6861; 1502.4418; 1504.2636; 1511.9913; 1517.3252; 1518.2115; 1526.9407; 1530.3255; 1540.4188; 1549.4002; 1614.3552; 1621.8180; 1666.8402; 1872.6648; 3044.6969; 3062.2587; 3065.9976; 3076.4179; 3102.3395; 3130.8096; 3135.8907; 3140.2127; 3140.7413; 3143.8189; 3152.9580; 3175.1725; 3178.8202; 3179.7401; 3190.1467; 3197.0164; 3207.4188.

Cartesian Forces: Max 0.000001612 RMS 0.000000531

Item	Value	Threshold	Converged?
Maximum Force	0.000001	0.000450	YES
RMS Force	0.000000	0.000300	YES
Maximum Displacement	0.000059	0.001800	YES
RMS Displacement	0.000010	0.001200	YES

FILE: Yb_Sq2_PhYbOTf2_TS_Neutral_Add12_B3LYP_M062x_Conf7.out

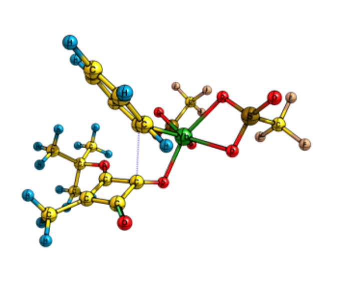

Conformer 5

E(RB3LYP/6-31G(d)/MWB59) = -2769.89287712
Convg = 0.7874D-08 -V/T = 2.0149

Temperature 195.150 Kelvin. Pressure 1.00000 Atm.
elec. and zero-point Energies= -2769.550005
elec. and thermal Energies= -2769.531180
elec. and thermal Enthalpies= -2769.530562
elec. and thermal Free Energies= -2769.591262

RB3LYP/6-31G(d)/MWB59/THF(IEFPCM) = -2769.91594725

RM06-2x/6-311+G(d,p)/MWB59/THF(IEFPCM) = -2769.71899373

Geometry at RB3LYP/6-31G(d)/MWB59
Charge = 0 Multiplicity = 1
Yb 0.7963766362 1.8513960936 0.5458007781
O 2.653336148 1.052518919 -0.6671255216
S 3.566378944 0.780782593 0.5170025875
O 4.2141601031 -0.5193529867 0.5715620166
O 2.7647276405 1.2239295966 1.7236248984
C 4.9175537249 2.0575428405 0.3568548189
F 5.620385558 1.8136434663 -0.7474768723
F 4.3732944074 3.2738961852 0.2802729751
F 5.7119329307 1.9898990031 1.4225015774
O -0.4098175591 3.4031291769 1.7981723982
O -0.6053007118 5.7606119806 0.862244112
O 1.2564560811 4.1418975468 0.2400512665
S 0.2543647136 4.6611332203 1.2556024903
C 1.3117254143 5.2496200383 2.6756106706
F 0.5297010834 5.6390158874 3.6778687768
F 2.0928622152 4.2422771039 3.0835239886
F 2.0703898624 6.2639806327 2.2702535826
C -0.6161566635 -1.6325504996 -0.337637421
C -1.9591859483 -1.9498987096 -0.4903928351
C -2.3940673277 -0.7286227912 0.2370909261
C -0.9049318066 -0.4140674199 0.4711469903
O -0.2462869888 0.1247091723 1.4053800497
O -3.4529199558 -0.2468734712 0.5557261265
O 0.601999773 -2.0246626461 -0.6066730574
C 0.9887623123 -3.2589067366 -1.339549793
C -2.8336674224 -3.0014554949 -1.0754461474
H -2.4597071554 -4.0170262535 -0.9148361059
H -2.9486341012 -2.8536673071 -2.1579576997
H -3.8305040309 -2.9202785536 -0.630023971
C 0.6412076335 -4.4645196868 -0.4653205641
H 0.9785221939 -5.3840553431 -0.9552033625
H 1.1403529414 -4.3897564624 0.5056204482
H -0.43610338 -4.550960222 -0.2960011818
C 2.5022381449 -3.0925142311 -1.4804783653
H 2.9298231635 -3.9826165592 -1.9533348015
H 2.7401641798 -2.2206638724 -2.0969846534
H 2.971067485 -2.9507726984 -0.5029397862
C 0.308170568 -3.2865120624 -2.7086872729
H -0.7676710335 -3.4617422986 -2.6389942272
H 0.4762638345 -2.3443532879 -3.2393997279
H 0.7373875015 -4.0971701253 -3.3069209038
C -0.6258248823 1.2934923929 -1.3052152661
C -0.0220070772 0.9015126138 -2.5229137821
C -1.8521887656 1.9926280682 -1.3937536572
H 0.9259558444 0.3667250987 -2.506906832
H -2.3750553331 2.2961726919 -0.4878089738
C -0.5983822824 1.2056859512 -3.757944536
C -2.4347517961 2.3014762143 -2.6270224094
H -0.1046432171 0.9061419461 -4.6799223248
H -3.3762761233 2.8438022296 -2.6631384839
C -1.8085561874 1.9049322192 -3.810115461
H -2.2593390789 2.1417313413 -4.7706904422

Frequency: -157.1578; 16.2678; 18.2759; 22.5199; 23.7598; 26.0581; 29.3795; 34.5428; 40.4777; 43.5207; 44.8377; 50.0984; 51.1282; 62.1331; 66.0803; 67.6555; 77.1194; 98.2160; 119.1171; 125.3961; 137.7091; 140.0660; 156.5026; 163.9315; 164.6521; 173.2919; 192.0621; 196.8011; 207.8896; 217.2315; 218.4106; 226.6840; 229.8195; 235.1785; 238.9906; 247.8776; 258.7671; 295.5677; 303.4386; 306.2849; 319.9244; 320.9540; 329.5479; 341.9600; 349.4776; 362.1307; 366.5457; 393.6271; 396.4813; 437.4904; 454.5710; 482.3566; 489.9434; 491.1877; 508.3955; 531.0040; 535.9438; 557.7296; 558.4443; 576.7412; 582.8328; 595.5901; 619.8279; 622.1199; 626.7723; 634.1882; 641.0153; 646.0884; 716.8143; 732.4582; 762.6025; 763.7268; 765.5195; 766.3152; 781.6935; 838.8934; 871.5838; 935.3347; 936.3819; 936.8229; 953.2142; 963.5570; 967.7119; 981.3543; 982.9665; 994.9755; 1006.6510; 1010.8070; 1019.1440; 1049.7844; 1059.7961; 1060.7806; 1067.0780; 1070.6788; 1072.8886; 1094.3823; 1116.1866; 1192.8320; 1193.6370; 1198.9300; 1201.8166; 1204.1568; 1223.0913; 1248.7344; 1254.7495; 1268.5885; 1274.4513; 1284.7593; 1296.4030; 1302.7485; 1316.5351; 1331.9929; 1369.1484; 1388.5757; 1432.5400; 1436.8581; 1439.5552; 1461.3221; 1462.6157; 1500.2887; 1505.4167; 1509.7560; 1513.9988; 1516.0999; 1517.1692; 1527.7996; 1530.8998; 1545.3073; 1577.0656; 1613.2928; 1622.5312; 1681.6954; 1883.9919; 3043.3366; 3066.5253; 3069.0702; 3076.1339; 3106.9585; 3135.8935; 3136.8919; 3138.3322; 3145.0416; 3146.3311; 3154.7913; 3163.5576; 3164.8120; 3167.7246; 3181.8525; 3191.2180; 3205.0117.

Cartesian Forces: Max 0.000000981 RMS 0.000000335

Item	Value	Threshold	Converged?
Maximum Force	0.000001	0.000450	YES
RMS Force	0.000000	0.000300	YES
Maximum Displacement	0.000018	0.001800	YES
RMS Displacement	0.000004	0.001200	YES

FILE: Yb_Sq2_PhYbOTf2_TS_Neutral_Add12_B3LYP_M062x_Conf7-.out

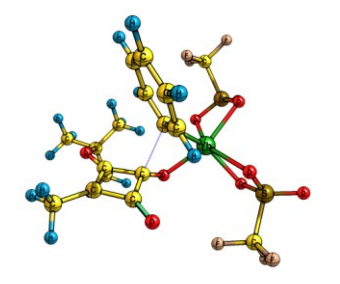

Conformer 6

E(RB3LYP/6-31G(d)/MWB59) = -2769.89038639
Convg = 0.1066D-08 -V/T = 2.0149

Temperature 195.150 Kelvin. Pressure 1.00000 Atm.
elec. and zero-point Energies= -2769.547630
elec. and thermal Energies= -2769.528829
elec. and thermal Enthalpies= -2769.528211
elec. and thermal Free Energies= -2769.588742

RB3LYP/6-31G(d)/MWB59/THF(IEFPCM) = -2769.91237690

RM06-2x/6-311+G(d,p)/MWB59/THF(IEFPCM) = -2769.71505097

Geometry at RB3LYP/6-31G(d)/MWB59
Charge = 0 Multiplicity = 1
Yb 1.4641695905 1.967929401 -0.310752099
O 0.3527139485 3.9638883168 -0.7172555736
S 0.6868395205 4.6634562693 0.5956775012
O 1.2129062089 6.0125625972 0.5185475973
O 1.4861467088 3.6274425481 1.3668924942
C -0.9460882555 4.7563338094 1.4972599896
F -1.805212717 5.4650749796 0.7700376154
F -1.422800688 3.525970324 1.6870777763
F -0.7540355709 5.3470979602 2.675113439
O 3.4411129503 0.8241222596 0.1939147441
O 5.5349728462 2.264509971 0.122315062
O 3.478047233 2.6810336644 -1.3189122033
S 4.3956790704 1.718609773 -0.5895221047
C 5.0813044842 0.6078998065 -1.9232994675
F 5.7681326564 -0.3808667822 -1.3536743834
F 4.0801273634 0.092333468 -2.6445001745
F 5.8784785685 1.3174257463 -2.7152024721
H 0.3481785934 -1.4416829389 2.3876881041
C -0.9993827698 -1.3896255268 -0.0669414239
C -2.288572338 -1.0126760625 -0.3893258333
C -0.544212674 0.0342326486 0.1404485178
C -1.9942057046 0.4223579804 -0.2101018643
O 0.3198440535 0.5475228041 0.9221080959
O -2.6048918366 1.4651112765 -0.2455449399
O -0.5012132464 -2.6055456771 -0.0074575193
C 0.6192583372 -3.0103166748 0.8928196392
C -3.5217882027 -1.7588468465 -0.7371162399
H -3.4152184077 -2.8270697846 -0.5271159539
H -3.7490141711 -1.6383226716 -1.805079285
H -4.3813278693 -1.3643511899 -0.1834652987
C 1.9403420076 -2.4620923551 0.3599891322
H 1.9815118048 -1.3728983169 0.4170779619
H 2.7657805823 -2.8510184694 0.9660715045
H 2.1013588934 -2.7807926432 -0.6747403799
C 0.5716570522 -4.5351242909 0.793010225
H 1.3636218018 -4.9723819227 1.4091619771
H -0.3921867104 -4.9171766032 1.1432621237
H 0.7187786582 -4.8595179586 -0.2419363057
C 0.317834533 -2.5323100514 2.3140349288
H -0.6634551987 -2.8902279827 2.6431950656
H 1.0723548907 -2.9340186995 2.9986186233
C 0.2588222936 0.7259176321 -2.0169861141
C 0.9254213262 -0.3361711889 -2.6679477597
C -0.5932839324 1.5298894777 -2.8108087788
H 1.5847345029 -0.9933017642 -2.1048358481
H -1.1490759662 2.3460922474 -2.3552495911
C 0.7719531511 -0.5746153273 -4.0362514853
C -0.7467674173 1.3011086979 -4.1807559511
H 1.3018270773 -1.3979495054 -4.5096463935
H -1.3986095311 1.9433684196 -4.7679569303
C -0.067027861 0.2455906605 -4.7941687745
H -0.1907878219 0.0630234714 -5.858611855

Frequency: -185.2129; 14.4872; 16.4027; 22.2829; 25.0451; 30.0421; 34.1142; 38.3583; 40.2269; 45.0559; 48.9641; 52.0220; 57.6452; 61.8893; 68.9219; 75.4720; 77.7992; 85.7488; 91.8267; 112.7149; 129.6993; 139.3078; 150.7473; 161.1997; 174.2000; 176.0590; 184.7457; 191.2575; 212.5167; 214.9028; 220.2462; 223.3969; 238.1509; 244.4192; 256.4271; 275.5122; 281.3129; 292.6982; 305.1233; 305.6912; 316.9847; 318.9017; 320.0533; 340.9017; 348.4524; 365.9760; 366.1807; 392.9771; 395.3523; 442.1863; 449.9477; 484.0811; 489.1424; 490.1690; 509.8561; 531.3268; 537.0014; 557.6687; 558.0282; 574.0668; 581.8493; 587.7271; 618.1168; 624.5460; 626.6811; 635.0498; 640.2703; 645.9349; 716.0420; 726.9900; 733.3459; 761.3903; 765.9800; 766.4620; 787.8237; 853.3662; 868.6526; 934.2954; 935.8941; 938.8959; 950.0846; 957.6613; 960.5506; 980.9540; 985.9396; 986.8800; 1007.1465; 1009.0756; 1016.2533; 1052.5409; 1053.4722; 1058.8517; 1063.7242; 1064.6441; 1071.2901; 1075.8640; 1098.3888; 1194.0611; 1197.9597; 1199.1137; 1201.1276; 1224.2767; 1239.1577; 1248.7472; 1258.4719; 1269.0753; 1271.6975; 1288.3131; 1300.6360; 1303.5306; 1325.5914; 1332.9061; 1369.5727; 1391.9215; 1431.3576; 1433.5331; 1441.6693; 1458.8301; 1463.1177; 1502.0769; 1503.0560; 1504.0387; 1514.9402; 1517.1128; 1518.3815; 1530.1679; 1532.6760; 1546.3302; 1551.0365; 1614.2512; 1622.0466; 1669.0036; 1873.2556; 3044.8921; 3062.2493; 3064.7661; 3072.0147; 3102.3883; 3129.4670; 3131.5467; 3140.1993; 3141.0111; 3142.8914; 3150.8250; 3171.8583; 3172.8878; 3181.3141; 3189.0179; 3196.6621; 3206.9978.

Cartesian Forces: Max 0.000000970 RMS 0.000000352

Item	Value	Threshold	Converged?
Maximum Force	0.000000	0.000450	YES
RMS Force	0.000000	0.000300	YES
Maximum Displacement	0.000059	0.001800	YES
RMS Displacement	0.000010	0.001200	YES

FILE: Yb_Sq2_PhYbOTf2_TS_Neutral_Add12_B3LYP_M062x_Conf8.out

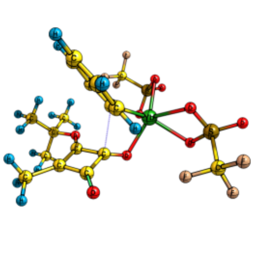

Conformer 7

E(RB3LYP/6-31G(d)/MWB59) = -2769.88981338
Convg = 0.8516D-09 -V/T = 2.0149

Temperature 195.150 Kelvin. Pressure 1.00000 Atm.
elec. and zero-point Energies= -2769.546930
elec. and thermal Energies= -2769.528095
elec. and thermal Enthalpies= -2769.527477
elec. and thermal Free Energies= -2769.588525

RB3LYP/6-31G(d)/MWB59/THF(IEFPCM) = -2769.91700312

RM06-2x/6-311+G(d,p)/MWB59/THF(IEFPCM) = -2769.71754494

Geometry at RB3LYP/6-31G(d)/MWB59
Charge = 0 Multiplicity = 1
Yb 1.3812746134 1.7459248239 -0.3513450891
O 1.1133636432 3.9581652937 -1.061912736
S 1.1866961909 4.6179962419 0.3059967086
O 2.0057442689 5.8057596736 0.4466515363
O 1.4359069051 3.4609057323 1.257346405
C -0.5825135983 5.1045691594 0.6437151879
F -0.9452243472 6.0682508122 -0.1984643783
F -1.3686282291 4.0346149528 0.4594814696
F -0.6983536511 5.5294630666 1.8976776305
O 2.9558416049 0.2623654546 0.5922962179
O 5.3800418652 0.9792651485 0.2993500895
O 3.5176148349 1.5795860865 -1.3255058725
S 4.1184063979 0.6160585754 -0.3167567489
C 4.4390316736 -0.9477760309 -1.2833179803
F 4.8748550661 -1.8935764416 -0.454941704
F 3.3029263953 -1.3690499931 -1.8647758335
F 5.3477575435 -0.7117734405 -2.2232141631
H -1.6663146805 -3.6720593053 -1.9275096125
C -1.0356952695 -1.3365700096 -0.2748286618
C -2.3988957634 -1.2619111606 -0.5241168764
C -0.9238857999 0.1167218032 0.0520698538
C -2.4244787622 0.2041170307 -0.2823558029
O -0.1589419734 0.7285937754 0.8468859524
O -3.2731594351 1.0616026307 -0.2815147379
O -0.0159588385 -2.1569472498 -0.216747649
C -0.063528663 -3.6255713362 -0.433099061
C -3.5547396799 -2.1230146436 -0.8930495709
H -3.5739771376 -3.0806867401 -0.3646399038
H -3.5523649179 -2.3356266932 -1.9708622719
H -4.4809443818 -1.5829754035 -0.6713827334
C -0.8873225591 -4.2604179317 0.6882864612
H -1.9355017568 -3.9519299228 0.6555869382
H -0.8564475286 -5.3510584777 0.5940914274
H -0.4762792115 -3.9889225936 1.6654744083
C 1.4100462322 -4.0194276784 -0.3183202286
H 1.5188684071 -5.0990173386 -0.4626599541
H 2.0108573774 -3.5024843653 -1.0715826324
H 1.8012844326 -3.7583824419 0.6695245553
C -0.6080168277 -3.9246092795 -1.8304700875
H -0.0466541282 -3.3699186483 -2.5885261871
H -0.5017659184 -4.9943124889 -2.0394300773
C -0.0783266296 0.9447035188 -2.1104600474
C 0.3882579007 -0.0385032925 -3.014000933
C -1.0104344855 1.8794737188 -2.6181841527
H 1.1003909438 -0.7905727661 -2.680684749
H -1.4205398939 2.6479206544 -1.9661527159
C -0.0269185255 -0.0736880708 -4.3471222381
C -1.4354836024 1.8499046568 -3.9492470768
H 0.3645564461 -0.8320964146 -5.021700339
H -2.1507860235 2.5859647475 -4.3077685421
C -0.9440347132 0.8715945527 -4.8157173632
H -1.2720323214 0.8457888691 -5.8518840795

Frequency: -159.6288; 6.0401; 17.4788; 20.5641; 23.6967; 25.0764; 34.6596; 43.5672; 44.7344; 46.5681; 47.8781; 53.1348; 57.1434; 63.3913; 64.1033; 73.0719; 77.5477; 89.9977; 112.1529; 124.1235; 133.6652; 141.5473; 147.7644; 159.1210; 165.5724; 176.2913; 186.7147; 189.3594; 209.5527; 213.5579; 221.3809; 228.0145; 230.1174; 234.7544; 237.2484; 249.6335; 265.7952; 295.8267; 304.1018; 305.4465; 318.7159; 319.5710; 331.6325; 339.7661; 350.0208; 364.0556; 365.3643; 392.1582; 396.5482; 435.5346; 454.7439; 482.8995; 489.2931; 490.0382; 505.9554; 531.5130; 536.5876; 556.5675; 557.1274; 575.0593; 584.2481; 596.2845; 618.8131; 622.2802; 627.9460; 635.1261; 640.9676; 646.8485; 714.4201; 731.5949; 762.7070; 763.5300; 764.2520; 764.6123; 782.4763; 841.1106; 867.9751; 932.1080; 935.0084; 935.9634; 953.6109; 962.0749; 969.4244; 979.8675; 981.9056; 994.7877; 1006.2810; 1011.3490; 1018.5523; 1049.3647; 1060.1560; 1060.9745; 1066.5959; 1071.9591; 1072.9520; 1094.2785; 1110.8188; 1192.3320; 1193.5735; 1195.9344; 1197.4932; 1205.0274; 1224.5120; 1234.7021; 1244.6717; 1272.8769; 1274.4534; 1285.9361; 1296.5555; 1302.7190; 1327.1244; 1336.0855; 1370.8169; 1385.3650; 1432.8913; 1436.3342; 1439.4142; 1461.0614; 1462.6729; 1500.7153; 1504.4595; 1511.9038; 1514.0113; 1517.8945; 1519.9018; 1528.4656; 1530.7957; 1547.3616; 1583.5734; 1614.5499; 1623.7907; 1680.7574; 1882.8749; 3043.5980; 3068.5950; 3070.1081; 3076.9909; 3107.5202; 3136.6753; 3137.8852; 3138.4094; 3145.9917; 3152.9839; 3155.2017; 3162.8731; 3170.1040; 3176.6531; 3185.8611; 3192.9519; 3205.5385.

Cartesian Forces: Max 0.000001195 RMS 0.000000425

Item	Value	Threshold	Converged?
Maximum Force	0.000000	0.000450	YES
RMS Force	0.000000	0.000300	YES
Maximum Displacement	0.000415	0.001800	YES
RMS Displacement	0.000097	0.001200	YES

FILE: Yb_Sq2_PhYbOTf2_TS_Neutral_Add12_B3LYP_M062x_Conf8-.out

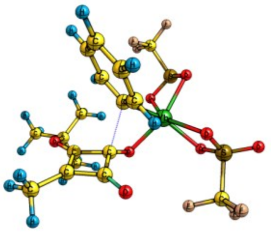

Conformer 8

E(RB3LYP/6-31G(d)/MWB59) = -2769.89038790
Convg = 0.5020D-08 -V/T = 2.0149

Temperature 195.150 Kelvin. Pressure 1.00000 Atm.
elec. and zero-point Energies= -2769.547608
elec. and thermal Energies= -2769.528823
elec. and thermal Enthalpies= -2769.528205
elec. and thermal Free Energies= -2769.588645

RB3LYP/6-31G(d)/MWB59/THF(IEFPCM) = -2769.91274396

RM06-2x/6-311+G(d,p)/MWB59/THF(IEFPCM) = -2769.71541785

Geometry at RB3LYP/6-31G(d)/MWB59
Charge = 0 Multiplicity = 1
Yb 1.2058122479 2.205791181 0.3058687126
O 3.4081298415 1.4783061324 0.6107966109
S 4.1056528574 2.7350852822 0.1020767283
O 5.0901293214 3.3507182989 0.9706791452
O 2.9710016385 3.5965432981 -0.4187417342
C 5.0188494838 2.1723745596 -1.4251960734
F 5.9337985426 1.2699866355 -1.0753437561
F 4.1615659126 1.620056253 -2.2902613928
F 5.607634082 3.2200102161 -1.9924985249
O -0.362103601 3.9148350674 0.3561215278
O 0.0021185784 5.7404060872 2.0876976397
O 0.8640554217 3.357609629 2.3378604743
S -0.1791643995 4.3282807873 1.8123586657
C -1.7660056518 3.8008075135 2.6440972345
F -2.7871879187 4.4369641259 2.0769671769
F -1.9243876347 2.4838340744 2.5109682243
F -1.6986677581 4.1136034505 3.9367157899
C -0.3830522789 -1.5894638206 -0.3848097512
C -1.7301111088 -1.4568443116 -0.6603013231
C -1.7816743583 -0.083182853 -0.1230323906
C -0.2752615946 -0.1957855936 0.1837361196
O 0.4557285801 0.2953151333 1.103140222
O -2.6228587912 0.7629460526 0.0717920854
O 0.3904759118 -2.6307990544 -0.6028101966
C 1.5889525242 -2.9726365972 0.219656819
C -2.7568260478 -2.3606741806 -1.2325385266
H -2.3965043357 -3.3921344373 -1.2855895958
H -3.0239786083 -2.0360597665 -2.2474088332
H -3.6760079931 -2.3277402669 -0.6367364969
C 2.733229243 -2.015106415 -0.1023006899
H 3.6369601941 -2.3390130487 0.4252490657
H 2.9489200057 -2.0181181208 -1.1754397525
H 2.5151345273 -0.9964618012 0.2235294468
C 1.9043604563 -4.3917464346 -0.2535525083
H 2.7868355483 -4.7706385225 0.2715356397
H 1.0646035297 -5.0638639806 -0.0518086063
H 2.1089011068 -4.4041574217 -1.3287238929
C 1.204261757 -2.9481528116 1.6997323653
H 0.9756012506 -1.9344650674 2.0402943206
H 0.3412694687 -3.596047843 1.8863997936
H 2.0429298763 -3.3197947432 2.2979343293
C 0.3041910297 1.1769169015 -1.7030295424
C 1.1914959877 0.4935640679 -2.5642990634
C -0.7269947391 1.930516014 -2.3124731153
H 1.9969630235 -0.1096371883 -2.1507487057
H -1.4517922146 2.4569418277 -1.6958570665
C 1.0752394759 0.5705637352 -3.9546849026
C -0.8455299416 2.0171268496 -3.7021253175
H 1.7762971092 0.0360391921 -4.5915139697
H -1.6404691709 2.61426842 -4.1422404402
C 0.0534468171 1.3334666436 -4.5248197892
H -0.0419831095 1.3950592414 -5.6059237368

Frequency: -185.3472; 13.8014; 17.1584; 22.3320; 25.0482; 30.4634; 35.1888; 38.7581; 40.5189; 45.2411; 49.6529; 52.9335; 58.2643; 62.6202; 68.8882; 76.3648; 77.9892; 85.8912; 92.7527; 112.3713; 130.1694; 138.9949; 150.6413; 161.5588; 174.2143; 176.1635; 184.8628; 191.2811; 212.6708; 214.9287; 220.1082; 223.3728; 238.1985; 244.1891; 256.7097; 275.2536; 281.7800; 292.7145; 305.0794; 305.7051; 317.0399; 319.0356; 320.0125; 340.9258; 348.4696; 365.9213; 366.2471; 393.3202; 396.2841; 442.1122; 450.0793; 484.2175; 489.1671; 490.2307; 509.6408; 531.3814; 537.0636; 557.6776; 558.0248; 574.0760; 581.9189; 587.7138; 618.1370; 624.4831; 626.7146; 634.9628; 640.1156; 645.9630; 717.0063; 726.9924; 733.3016; 761.3822; 765.9299; 766.4820; 787.8145; 853.4196; 869.6043; 935.0271; 935.9381; 938.8861; 950.0288; 957.6383; 960.4875; 981.7927; 986.1787; 986.8149; 1007.5606; 1009.0050; 1016.1702; 1052.5035; 1053.5197; 1058.7356; 1063.8579; 1064.5788; 1070.8551; 1075.9404; 1098.2966; 1194.0312; 1197.9777; 1199.1582; 1201.0404; 1223.7705; 1239.0576; 1248.7918; 1258.3255; 1269.1506; 1271.7106; 1288.3575; 1300.6263; 1303.3399; 1325.6098; 1332.9364; 1368.9368; 1391.8826; 1431.3944; 1433.5399; 1441.6284; 1458.8585; 1463.1654; 1502.0048; 1503.0811; 1504.0466; 1514.9205; 1517.0048; 1518.4533; 1530.2118; 1532.6593; 1546.6203; 1551.0302; 1614.2546; 1622.0667; 1669.0177; 1873.2917; 3044.8951; 3062.2246; 3064.7534; 3071.9932; 3102.4120; 3129.4782; 3131.5587; 3140.2218; 3141.0298; 3142.9499; 3150.5723; 3171.4615; 3172.5565; 3181.1531; 3188.8160; 3196.5364; 3206.9732.

Cartesian Forces: Max 0.000012607 RMS 0.000002086

Item	Value	Threshold	Converged?
Maximum Force	0.000012	0.000450	YES
RMS Force	0.000001	0.000300	YES
Maximum Displacement	0.001703	0.001800	YES
RMS Displacement	0.000232	0.001200	YES

FILE: Yb_Sq2_PhYbOTf2_TS_Neutral_Add12_B3LYP_M062x_Confx7.out

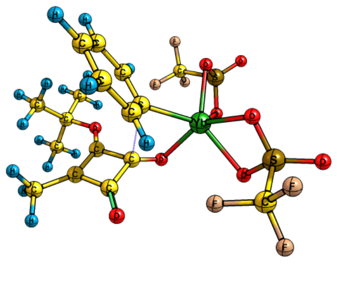

Conformer 9

E(RB3LYP/6-31G(d)/MWB59) = -2769.89014529
Convg = 0.8908D-08 -V/T = 2.0149

Temperature 195.150 Kelvin. Pressure 1.00000 Atm.
elec. and zero-point Energies= -2769.547140
elec. and thermal Energies= -2769.528357
elec. and thermal Enthalpies= -2769.527739
elec. and thermal Free Energies= -2769.588140

RB3LYP/6-31G(d)/MWB59/THF(IEFPCM) = -2769.91804626

RM06-2x/6-311+G(d,p)/MWB59/THF(IEFPCM) = -2769.71900359

Geometry at RB3LYP/6-31G(d)/MWB59
Charge = 0 Multiplicity = 1
Yb 1.0600468294 2.1806750232 0.4046232315
O 3.1504664263 1.685886108 1.4094754402
S 4.0068779419 1.9434566541 0.1871723267
O 5.2190065809 2.7222883881 0.3476105099
O 3.0136019915 2.3811659268 -0.8766117683
C 4.5191987394 0.2397583182 -0.3744465595
F 5.281672256 -0.3306180741 0.5526836655
F 3.4201490165 -0.5127761062 -0.5655489373
F 5.1882959412 0.3280582702 -1.5211046269
O 0.4154918849 4.4103688815 0.1711593498
O 0.4252473366 5.8216317047 2.2871587727
O 0.1591688714 3.2893753735 2.2693386014
S -0.0530136948 4.636441588 1.6022212713
C -1.9079541716 4.786884593 1.4583065534
F -2.2065722478 5.8243392921 0.6807700019
F -2.4012313263 3.6664631117 0.9175800225
F -2.4285378182 4.9608054327 2.6685400992
C -0.502579229 -1.5052883512 -0.3603538666
C -1.8893269223 -1.5764372382 -0.3788972079
C -2.0247614154 -0.2626084488 0.3004601439
C -0.4911831704 -0.2100725409 0.3830267777
O 0.3377095358 0.2604370397 1.2091125666
O -2.9415902696 0.429361183 0.6728977703
O 0.5895688122 -2.1408340727 -0.7010347135
C 0.6584171812 -3.4363774548 -1.4220436061
C -2.9929733811 -2.4683021183 -0.8258198779
H -2.7899652332 -3.5308666114 -0.6646471639
H -3.1979199456 -2.324031854 -1.8955086091
H -3.905818843 -2.2000464801 -0.2840156901
C 0.1593391418 -4.5408451298 -0.4892362851
H 0.2729020857 -5.5147762757 -0.9770579053
H 0.7402156757 -4.5512599244 0.4381984665
H -0.8964629792 -4.4156818254 -0.2341174986
C 2.1584467276 -3.5778165565 -1.6873170335
H 2.3602754079 -4.5292218246 -2.1893585817
H 2.5176079482 -2.7637439256 -2.3238812003
H 2.7184276333 -3.5520636945 -0.7478821721
C -0.124968221 -3.346624751 -2.7325435604
H -1.2046950603 -3.3119063497 -2.5715450705
H 0.1720129074 -2.4571961383 -3.296752334
H 0.0899786787 -4.2301794114 -3.3428092073
C -0.1758683661 1.3348614903 -1.5095180689
C 0.6230045737 0.737737865 -2.5131381066
C -1.3756910257 1.9479824643 -1.937173695
H 1.5580875314 0.2492565865 -2.2448555304
H -2.0393371824 2.4105476324 -1.2107241043
C 0.2607055942 0.7673098688 -3.8628401444
C -1.7475528974 1.9805857689 -3.2836667461
H 0.9078645067 0.3165959222 -4.6123158521
H -2.672773286 2.4691036694 -3.5801631827
C -0.930372967 1.3872494189 -4.2491664897
H -1.2172669698 1.4127393733 -5.2975173331

Frequency: -154.4253; 11.8069; 16.3991; 20.3638; 24.2133; 31.5614; 36.2422; 38.2912; 44.3766; 47.7178; 49.1573; 53.8536; 58.8982; 64.2015; 67.2708; 74.9457; 86.8458; 96.6079; 109.8806; 121.1509; 129.1753; 142.1383; 151.8466; 160.8018; 166.2582; 179.9064; 186.3010; 187.9915; 208.3420; 214.1202; 222.8747; 225.0563; 230.3476; 236.7608; 238.3833; 248.6842; 256.0855; 291.7605; 304.5854; 305.2188; 318.2832; 319.3362; 330.9709; 336.2357; 349.9043; 363.3492; 366.8033; 394.8737; 397.5630; 436.0331; 454.5638; 483.2876; 490.1245; 490.3268; 506.8507; 531.7238; 536.7969; 556.6128; 557.6034; 576.4479; 585.0325; 600.1040; 615.6342; 621.5393; 628.3736; 634.2076; 640.6864; 646.0661; 717.5001; 731.4520; 762.5780; 763.7710; 764.2951; 765.6698; 783.4583; 841.9962; 873.5155; 935.0642; 937.0669; 938.6600; 953.6560; 962.6151; 971.2143; 981.8148; 985.9278; 996.6503; 1005.9395; 1011.6386; 1017.4874; 1049.3893; 1057.9161; 1060.5316; 1066.2068; 1074.0729; 1075.9826; 1095.5449; 1112.0754; 1192.8365; 1193.2713; 1193.7929; 1197.8301; 1205.3308; 1226.7781; 1235.8258; 1247.7034; 1273.0803; 1274.1640; 1286.2230; 1295.9571; 1302.9482; 1327.9713; 1337.0121; 1374.8000; 1388.3856; 1433.0377; 1435.8135; 1439.9136; 1460.4489; 1462.5743; 1500.3994; 1504.6102; 1510.1643; 1514.9006; 1516.2456; 1521.7807; 1527.9125; 1530.8365; 1546.6534; 1585.7544; 1614.6587; 1623.1277; 1680.9916; 1879.2590; 3043.5367; 3066.6842; 3069.0777; 3075.8530; 3107.6039; 3136.5167; 3137.7217; 3139.2869; 3146.0629; 3146.9330; 3151.2967; 3163.8076; 3166.3576; 3178.2199; 3184.9471; 3196.4370; 3205.0497.

Cartesian Forces: Max 0.000002504 RMS 0.000000651

Item	Value	Threshold	Converged?
Maximum Force	0.000003	0.000450	YES
RMS Force	0.000000	0.000300	YES
Maximum Displacement	0.000280	0.001800	YES
RMS Displacement	0.000052	0.001200	YES

FILE: Yb_Sq2_PhYbOTf2_TS_Neutral_Add12_B3LYP_M062x_Confx7-.out

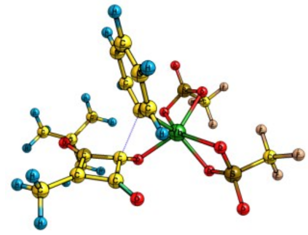

Conformer 10

E(RB3LYP/6-31G(d)/MWB59) = -2769.89232796
Convg = 0.7075D-08 -V/T = 2.0149

Temperature 195.150 Kelvin. Pressure 1.00000 Atm.
elec. and zero-point Energies= -2769.549501
elec. and thermal Energies= -2769.530653
elec. and thermal Enthalpies= -2769.530035
elec. and thermal Free Energies= -2769.591223

RB3LYP/6-31G(d)/MWB59/THF(IEFPCM) = -2769.91499027

RM06-2x/6-311+G(d,p)/MWB59/THF(IEFPCM) = -2769.71847877

Geometry at RB3LYP/6-31G(d)/MWB59
Charge = 0 Multiplicity = 1
Yb 1.5202234938 2.0475073194 -0.2618963176
O 1.3652150947 3.6895687828 1.4018788558
S 0.5931278489 4.7179267362 0.5869777507
O -0.5698396954 5.3297351926 1.1985055244
O 0.422813966 4.0546546763 -0.7699163329
C 1.829888572 6.0819522824 0.2789843089
F 2.1559888047 6.6459969847 1.4396417382
F 2.925265947 5.5726333126 -0.2923176285
F 1.288012653 6.988935794 -0.5293275286
O 3.4511056543 2.6786530964 -1.4547874402
O 5.2033627144 0.8685291397 -1.8128381039
O 3.5019627432 0.8359470841 0.0738809579
S 4.381501205 1.6242115392 -0.8861239242
C 5.5287854263 2.5763633903 0.2358656071
F 6.2409358317 3.4346664322 -0.4876717992
F 4.8033452262 3.2445946904 1.1389880722
F 6.3390039222 1.7249848263 0.8592745569
H 0.6110945091 -1.5741090808 2.3884619424
C -0.9256129253 -1.3356696535 0.0624838496
C -2.2164199109 -0.875367349 -0.1177411456
C -0.3714328961 0.0530332284 0.2723392588
C -1.8180843256 0.5313436343 0.0813667374
O 0.580732355 0.4962038979 0.9929771287
O -2.3518336988 1.6158764971 0.1397936562
O -0.4943270509 -2.5771427895 0.0323992718
C 0.6868893565 -3.0709995321 0.8013668091
C -3.5187266369 -1.5388036589 -0.3693513325
H -3.4527969006 -2.6195937808 -0.2138725174
H -3.842847432 -1.3584157265 -1.4033382464
H -4.2958722958 -1.1257923795 0.2836881752
C 1.9771360028 -2.5441845142 0.1789433757
H 2.080246188 -1.4648037994 0.3063331069
H 2.8345377134 -3.0155315791 0.6717610774
H 2.0204354723 -2.7928746715 -0.8858959475
C 0.5657311807 -4.5852433812 0.6295977724
H 1.3874838718 -5.0849496636 1.1521474977
H -0.3806578162 -4.9484006255 1.0421489272
H 0.6112247779 -4.8587737017 -0.4292109585
C 0.5330433036 -2.6581450663 2.2660740885
H -0.4275276471 -2.9991918448 2.6665320619
H 1.3307243127 -3.119100986 2.8582712516
C 0.2683927138 0.7876520262 -1.9064613837
C 1.0871112013 -0.1521611358 -2.572209294
C -0.6992745921 1.4642749463 -2.6835435371
H 1.847181651 -0.7047408088 -2.0222078179
H -1.3595589262 2.1892706524 -2.2139064088
C 0.9633023684 -0.39461507 -3.9443594409
C -0.8272294656 1.2271029163 -4.0536499801
H 1.6127057337 -1.1185059201 -4.4306524027
H -1.5712008622 1.7710231575 -4.6307795425
C 0.0014299746 0.2941143724 -4.6848824338
H -0.1011168602 0.1080982372 -5.7509427192

Frequency: -192.2591; 11.7135; 16.4782; 19.7567; 21.2978; 24.9654; 30.7989; 33.4597; 36.4636; 41.1799; 46.1959; 54.1603; 57.3542; 62.1822; 65.2019; 73.0232; 79.9921; 88.4957; 93.4808; 107.5523; 131.7402; 141.5986; 161.0878; 162.0592; 168.8189; 180.1192; 189.3712; 197.5327; 209.6438; 218.2001; 219.0400; 223.7698; 230.3866; 237.5804; 260.5990; 275.1150; 282.4019; 291.4586; 302.7404; 306.3481; 316.8122; 318.9885; 320.6608; 341.5868; 348.1707; 363.1846; 366.4931; 394.7382; 397.1210; 445.4559; 450.3800; 484.2617; 489.9867; 490.8989; 508.2151; 532.1788; 536.3968; 557.6184; 557.7964; 577.9465; 582.5713; 589.8573; 618.5305; 621.7620; 626.3070; 634.6525; 639.1267; 647.6856; 719.4864; 727.5132; 734.0500; 763.4211; 765.4564; 766.2198; 788.5229; 854.2226; 873.7869; 936.1720; 938.0852; 942.5479; 951.2985; 960.3613; 962.3791; 986.6449; 988.8955; 992.9304; 1007.3167; 1010.9425; 1015.9564; 1053.4024; 1055.1016; 1062.9857; 1065.1996; 1066.5598; 1070.5413; 1076.6197; 1099.1026; 1194.2229; 1198.4892; 1199.8431; 1200.7523; 1223.2732; 1237.8097; 1248.2473; 1254.2999; 1270.6184; 1273.9246; 1288.4482; 1301.0008; 1305.4200; 1325.3803; 1334.6516; 1368.9220; 1393.0330; 1432.0563; 1433.2387; 1442.2016; 1459.0339; 1463.8688; 1502.3306; 1502.8934; 1504.5791; 1515.6558; 1516.8518; 1518.3885; 1530.6296; 1533.3573; 1546.9970; 1552.0354; 1614.2751; 1621.7747; 1666.6279; 1869.3990; 3044.5192; 3061.6074; 3064.4039; 3071.6955; 3102.0874; 3129.6938; 3131.4576; 3140.0474; 3140.5269; 3142.6796; 3148.8116; 3167.9448; 3169.6720; 3180.2995; 3189.5403; 3198.2201; 3207.4938.

Cartesian Forces: Max 0.000000837 RMS 0.000000274

Item	Value	Threshold	Converged?
Maximum Force	0.000000	0.000450	YES
RMS Force	0.000000	0.000300	YES
Maximum Displacement	0.000031	0.001800	YES
RMS Displacement	0.000009	0.001200	YES

FILE: Yb_Sq2_PhYbOTf2_TS_Neutral_Add12_B3LYP_M062x_Confx8.out

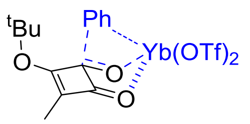

Butterfly Transition State for PhYb(OTf)$_2$ addition to C-1 carbonyl of 1b

Lowest energy conformation is:
Conformer 1

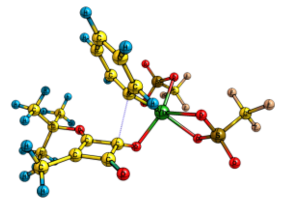

Conformer 11

E(RB3LYP/6-31G(d)/MWB59) = -2769.89182861
Convg = 0.5381D-08 -V/T = 2.0149

Temperature 195.150 Kelvin. Pressure 1.00000 Atm.
elec. and zero-point Energies= -2769.548980
elec. and thermal Energies= -2769.530128
elec. and thermal Enthalpies= -2769.529510
elec. and thermal Free Energies= -2769.590677

RB3LYP/6-31G(d)/MWB59/THF(IEFPCM) = -2769.91592788

RM06-2x/6-311+G(d,p)/MWB59/THF(IEFPCM) = -2769.71783007

Geometry at RB3LYP/6-31G(d)/MWB59
Charge = 0 Multiplicity = 1
Yb 1.4438124845 1.8720921637 -0.2766782948
O 1.6260100509 3.5170404396 1.4148557722
S 0.8504283426 4.6041857523 0.6923385885
O -0.1863308192 5.3022488367 1.4278448654
O 0.4688146153 3.9671670763 -0.6357618237
C 2.1453452358 5.8664460031 0.2318338674
F 2.6575553621 6.3930436506 1.3418558059
F 3.1157725906 5.2730208497 -0.4694400445
F 1.584861299 6.8217927639 -0.505501899
O 3.4724668625 2.2692578475 -1.4091369452
O 4.9284517215 0.2348001226 -1.8702464064
O 3.1995304898 0.3396208251 -0.0115517498
S 4.2110862357 1.0446279408 -0.9028978878
C 5.4648124328 1.730221857 0.2964156894
F 6.3238229648 2.5054437184 -0.3589877017
F 4.8311039453 2.4506982773 1.2276630775
F 6.1133410461 0.7243605614 0.8778748547
H -2.0000691343 -3.5059376366 -1.8349782039
C -1.0054360782 -1.317561658 -0.1411641605
C -2.3631928041 -1.089526888 -0.3271647057
C -0.7273512868 0.1064443456 0.2099641535
C -2.2165139439 0.3650185154 -0.0628530197
O 0.135450522 0.6252662277 0.971231594
O -2.9582722754 1.3160648533 -0.0245382185
O -0.0848204385 -2.2475561499 -0.1459627564
C -0.2984501963 -3.682770522 -0.4619098055
C -3.6217973703 -1.8115837921 -0.6545595595
H -3.700493893 -2.793440386 -0.1792405018
H -3.71409071 -1.9555691888 -1.7398591544
H -4.4723192395 -1.1991901217 -0.3376403999
C -1.1121975076 -4.3137597801 0.6690218324
H -2.1154700357 -3.8851514307 0.7413989107
H -1.2192724528 -5.3889626373 0.4904553388
H -0.6064078094 -4.1737984956 1.6293654438
C 1.1307797732 -4.2271403659 -0.4797456563
H 1.1156407194 -5.3064400704 -0.6617903011
H 1.7181268437 -3.7489319484 -1.2693344938
H 1.6251098778 -4.0412651028 0.4782622338
C -0.9550506887 -3.8230510118 -1.8362072947
H -0.4126424488 -3.2340019899 -2.5821258042
H -0.9291195875 -4.8742284134 -2.1422223486
C 0.0976482993 0.8848228497 -2.008583992
C 0.7456947517 -0.1481668268 -2.7272756052
C -0.8614283422 1.6485700792 -2.7111142902
H 1.4898760275 -0.7735001263 -2.2336004884
H -1.3941541933 2.448395498 -2.2013117117
C 0.4687844247 -0.3933736604 -4.0757068652
C -1.1490492576 1.404871033 -4.0567075646
H 0.9982461924 -1.1803242157 -4.6084391715
H -1.8880208909 2.0127985224 -4.5733586865
C -0.4852673982 0.3822473702 -4.7398823289
H -0.7061690639 0.1940705641 -5.7876777764

Frequency: -147.6904; 10.5291; 18.1046; 19.3380; 22.1679; 23.9671; 30.3607; 33.2316; 40.7566; 43.1974; 45.3223; 51.5268; 53.6230; 60.8852; 63.4046; 69.6606; 82.7356; 98.4058; 111.6015; 122.4348; 129.7914; 141.4306; 158.6682; 162.6510; 166.4298; 171.4432; 193.3088; 197.6053; 206.5859; 217.9105; 221.1118; 228.2872; 230.3670; 235.7395; 238.7648; 250.5603; 263.8969; 292.1618; 303.1112; 306.0988; 319.2593; 320.9213; 332.5954; 336.9409; 350.8557; 362.9394; 366.2409; 393.7348; 396.9596; 436.8108; 455.1134; 483.2766; 489.9216; 491.3508; 508.4033; 531.6591; 536.0456; 557.7187; 558.1104; 577.8244; 582.8076; 599.1956; 615.6181; 621.8760; 626.9055; 633.9904; 641.2995; 646.2881; 716.6105; 731.5319; 763.0036; 764.1257; 765.6521; 765.9977; 782.9632; 841.6465; 872.0967; 934.5137; 935.1741; 936.9291; 952.9167; 961.7738; 970.6810; 981.4671; 983.1013; 995.8975; 1006.0029; 1010.1031; 1018.6873; 1048.9019; 1060.4855; 1060.5526; 1065.6313; 1066.2241; 1072.1049; 1093.5780; 1111.8284; 1192.8249; 1193.6291; 1200.0344; 1201.4555; 1205.0230; 1223.2441; 1249.8497; 1252.8645; 1269.8504; 1272.7358; 1285.8095; 1296.1026; 1302.4283; 1324.0441; 1332.7418; 1369.5854; 1388.3165; 1432.7810; 1435.6085; 1439.6077; 1460.4420; 1462.1030; 1500.3536; 1504.6109; 1510.6630; 1515.2241; 1517.5686; 1517.6087; 1527.7863; 1531.3590; 1547.7333; 1583.2465; 1613.1145; 1621.6342; 1680.6234; 1882.6214; 3043.3768; 3066.3212; 3069.0557; 3076.0127; 3107.4924; 3136.3294; 3137.3409; 3139.4213; 3145.2036; 3147.1931; 3149.0424; 3151.0155; 3165.3554; 3175.2573; 3181.9141; 3191.9354; 3203.8338.

Cartesian Forces: Max 0.000009926 RMS 0.000001924

Item	Value	Threshold	Converged?
Maximum Force	0.000010	0.000450	YES
RMS Force	0.000002	0.000300	YES
Maximum Displacement	0.001167	0.001800	YES
RMS Displacement	0.000302	0.001200	YES

FILE: Yb_Sq2_PhYbOTf2_TS_Neutral_Add12_B3LYP_M062x_Confx8-.out

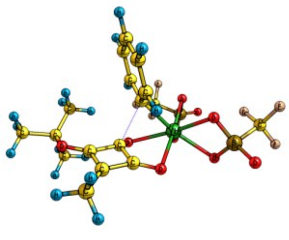

Conformer 1

E(RB3LYP/6-31G(d)/MWB59) = -2769.89505739
Convg = 0.4592D-08 -V/T = 2.0149

Temperature 195.150 Kelvin. Pressure 1.00000 Atm.
elec. and zero-point Energies= -2769.552056
elec. and thermal Energies= -2769.533341
elec. and thermal Enthalpies= -2769.532723
elec. and thermal Free Energies= -2769.592762

RB3LYP/6-31G(d)/MWB59/THF(IEFPCM) = -2769.91852722

RM06-2x/6-311+G(d,p)/MWB59/THF(IEFPCM) = -2769.72553630

Geometry at RB3LYP/6-31G(d)/MWB59
Charge = 0 Multiplicity = 1
Yb 0.4541011592 2.3105553366 -0.5470791733
O -0.4947677222 4.4378897508 -0.2678798676
S -0.7780229915 4.7204343425 -1.7332827243
O -2.1062515887 5.1840617141 -2.0920343439
O -0.2348200904 3.5011576319 -2.4564209885
C 0.4128425436 6.0900101444 -2.168553733
F 0.0851922501 7.1865461857 -1.4880832626
F 1.6551969608 5.7210651139 -1.8505992989
F 0.3411586343 6.3327384285 -3.4763134464
O 1.8949856832 2.9998874353 1.2340477782
O 3.9037478456 4.3841616768 0.5139187436
O 2.6958198167 2.7455622952 -1.0104246241
S 3.1354273014 3.1599202881 0.3865607038
C 4.2609355002 1.7726236013 0.9245965303
F 4.6052367021 1.9495383121 2.1980101079
F 3.6274553568 0.5964172905 0.8003917312
F 5.3503524658 1.7629888325 0.1594624426
H 0.3980209335 -1.6323103837 2.5556647797
C -1.2216266475 -1.4701531102 0.2343402834
C -2.3995553576 -0.8176437544 -0.1478624272
C -0.4411922495 -0.1676407654 0.2667348789
C -1.7276154795 0.4660579149 -0.1571560991
O 0.5047436828 0.3445564574 0.9044774523
O -1.929584075 1.6723468422 -0.3517328183
O -1.0369957846 -2.7352923103 0.4638716815
C 0.1843879185 -3.2856701782 1.1497613963
C -3.7797847191 -1.2818010782 -0.4365162641
H -3.9276539969 -2.3106083962 -0.0965207185
H -3.9759170586 -1.2452075382 -1.5163695969
H -4.5194040026 -0.6346102999 0.0472265391
C 1.4309972236 -2.9408526644 0.3411021246
H 1.6654686298 -1.8759435774 0.3940323834
H 2.2829162511 -3.491251874 0.755108673
H 1.312805589 -3.2294121951 -0.7074831097
C -0.1001020922 -4.7857072012 1.145622082
H 0.7139807678 -5.3148398067 1.6505162082
H -1.0346395118 -5.0066249191 1.6704137204
H -0.1760703205 -5.1640295004 0.1215381734
C 0.2241350683 -2.712561163 2.5661842503
H -0.7080471051 -2.92527349 3.0995839439
H 1.046922599 -3.1765025162 3.1203848012
C 0.3939218898 0.2841418607 -1.9970635158
C 1.6479505216 -0.3467059203 -2.1162642222
C -0.473870763 0.1929974423 -3.1057107693
H 2.3523207352 -0.3181366241 -1.2848259195
H -1.4555437692 0.6653716491 -3.067060662
C 2.0411521778 -0.9973917441 -3.2919565146
C -0.0994299819 -0.4606281599 -4.2807533501
H 3.0243838632 -1.4576124731 -3.3606434079
H -0.7821358958 -0.4988738095 -5.1266835812
C 1.1623360696 -1.0584432538 -4.3742590415
H 1.4566462877 -1.5688564581 -5.2879872935

Frequency: -183.3324; 12.9218; 18.3602; 25.5170; 27.7423; 31.2446; 34.1396; 36.1296; 43.8814; 45.7353; 48.9808; 53.1939; 61.9093; 66.4726; 66.9666; 75.3042; 80.1579; 97.2729; 102.6802; 118.0992; 126.8477; 148.3111; 157.5934; 161.7617; 169.1126; 179.3855; 190.4914; 200.1102; 214.9920; 216.0161; 216.8892; 220.5183; 233.4947; 237.8055; 265.8994; 269.8700; 275.9618; 279.9244; 303.2892; 307.0020; 319.3133; 320.5425; 332.0674; 341.2398; 350.2868; 362.3003; 365.8888; 397.4653; 401.3437; 411.9799; 452.2692; 465.2170; 488.3620; 489.6068; 490.6650; 531.1868; 534.7729; 557.3290; 558.0377; 576.7302; 578.6518; 594.8893; 613.1276; 625.6390; 626.8847; 631.4614; 635.3233; 646.7590; 712.7580; 717.2832; 742.0823; 750.0399; 763.3477; 765.7767; 795.0286; 853.3954; 873.9165; 937.3115; 939.9744; 943.9455; 956.5863; 963.3818; 978.8383; 984.8629; 990.8336; 1001.9663; 1009.0295; 1016.8758; 1026.1811; 1055.5612; 1060.5748; 1068.5603; 1069.2936; 1072.4879; 1081.4905; 1097.0578; 1119.4352; 1191.5469; 1194.8495; 1201.4156; 1202.6608; 1219.1073; 1237.6206; 1241.6577; 1255.3870; 1265.6234; 1267.7035; 1289.5945; 1306.5088; 1311.4998; 1323.5975; 1330.8108; 1365.2658; 1414.4918; 1435.2664; 1437.3416; 1453.3193; 1462.7905; 1464.6400; 1503.1001; 1503.5385; 1506.5272; 1512.6023; 1516.2883; 1517.6676; 1532.4963; 1534.7756; 1548.1505; 1608.3878; 1615.4175; 1619.7224; 1650.5195; 1800.0073; 3049.0593; 3062.6474; 3066.4219; 3073.3844; 3108.6343; 3133.4221; 3136.4942; 3142.4567; 3145.4989; 3145.7049; 3146.2771; 3150.0677; 3153.4515; 3170.2384; 3177.3166; 3185.9093; 3202.1667.

Cartesian Forces: Max 0.000003920 RMS 0.000000748

Item	Value	Threshold	Converged?
Maximum Force	0.000002	0.000450	YES
RMS Force	0.000000	0.000300	YES
Maximum Displacement	0.000478	0.001800	YES
RMS Displacement	0.000081	0.001200	YES

FILE:
Yb_Sq2_PhYbOTf2_TS_Neutral_Add12_B3LYP_M062x_Conf2_A.out

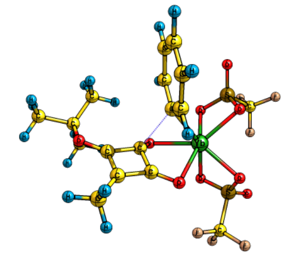

Conformer 2

E(RB3LYP/6-31G(d)/MWB59) = -2769.89438813
Convg = 0.2258D-09 -V/T = 2.0149

Temperature 195.150 Kelvin. Pressure 1.00000 Atm.
elec. and zero-point Energies= -2769.551201
elec. and thermal Energies= -2769.532605
elec. and thermal Enthalpies= -2769.531987
elec. and thermal Free Energies= -2769.591831

RB3LYP/6-31G(d)/MWB59/THF(IEFPCM) = -2769.91669942

RM06-2x/6-311+G(d,p)/MWB59/THF(IEFPCM) = -2769.72488378

Geometry at RB3LYP/6-31G(d)/MWB59
Charge = 0 Multiplicity = 1
Yb 1.2454715554 2.0332023653 -0.5459691327
O 0.4136961564 4.2275421646 -0.3821588873
S 0.6692755311 4.4322072592 1.1004082666
O 1.4089423608 5.6191313567 1.4921070776
O 1.1813857408 3.0888666414 1.5856388824
C -1.0221161266 4.6101791779 1.8731952359
F -1.6495463145 5.6413806416 1.3098109258
F -1.7425698889 3.5014601524 1.6955212905
F -0.8743834501 4.8323751643 3.1792341362
O 2.6001158929 3.0010027378 -2.1907536233
O 4.8564844591 1.8912263469 -2.5890508298
O 3.5641048891 1.7275987133 -0.4055514636
S 3.933428763 2.5105173676 -1.6555438894
C 4.7606973798 4.0516548633 -1.0036282262
F 4.9977443487 4.882053246 -2.015999834
F 3.9596709964 4.6432322363 -0.1153919025
F 5.9075061512 3.7156266456 -0.4164043373
C -0.7360673044 -1.5896405244 0.1888130048
C -1.874221715 -0.8269842773 -0.0872644032
C -1.0892126872 0.3888403359 -0.1555495545
C 0.1653764714 -0.3650451081 0.1739053099
O 1.2013712462 0.0173640332 0.7661915559
O -1.200828276 1.6014986307 -0.3806780705
O -0.6471465114 -2.8723172783 0.3770670987
C 0.5720118491 -3.5462388688 0.9470791175
C -3.3093647987 -1.15943776 -0.2767577042
H -3.5210363773 -2.1821168901 0.0476293887
H -3.5860610748 -1.0708688625 -1.3357480014
H -3.9492309627 -0.468537389 0.2829719359
C 1.7756443614 -3.287417886 0.0461047542
H 2.1086182329 -2.2493778898 0.1057680196
H 2.6054100857 -3.9227015689 0.3745242082
H 1.5473017305 -3.5333441056 -0.9953022925
C 0.1559146471 -5.0152317576 0.9326436638
H 0.9574052089 -5.6275952182 1.3571988896
H -0.7497259515 -5.1695068343 1.5273305895
H -0.0348270551 -5.3554474492 -0.0899211045
C 0.7764477117 -3.0211561204 2.3678740833
H -0.12453795 -3.1699348662 2.9719472836
H 1.597919662 -3.5698631019 2.8406461058
H 1.0423326476 -1.9598840348 2.3670279796
C 0.8109904971 0.1170799009 -2.1024705477
C 1.9658656521 -0.6299230573 -2.4072436145
C -0.1406088482 0.2729004597 -3.1326062848
H 2.7270887472 -0.7865458891 -1.6432917566
H -1.0603225879 0.8291020551 -2.9461264922
C 2.1874248716 -1.1593801563 -3.6834422873
C 0.0680415523 -0.2503822335 -4.4110266571
H 3.0984089627 -1.7146042644 -3.8940624461
H -0.6725464373 -0.0963927633 -5.1927889509
C 1.2347912969 -0.9705387611 -4.6863223049
H 1.3990296581 -1.3820645795 -5.6790012045

Frequency: -189.4713; 12.0704; 16.3797; 25.0046; 29.7535; 32.0399; 32.4222; 37.0170; 39.1400; 45.0101; 49.0422; 53.2516; 63.6526; 65.2519; 75.7013; 82.8591; 86.1525; 97.4799; 106.9010; 119.0939; 131.4056; 157.4880; 161.0523; 162.7913; 168.4214; 183.7396; 191.9400; 203.0392; 212.5253; 217.9942; 220.5716; 224.4801; 239.2369; 244.2779; 271.5393; 273.0302; 277.6084; 283.4994; 304.2369; 307.3996; 318.0379; 321.2292; 331.9203; 342.5885; 352.8969; 359.9666; 366.5601; 399.1185; 403.4870; 419.3967; 452.6237; 463.9416; 488.8945; 489.8644; 491.2662; 531.1620; 535.1512; 557.8928; 558.3821; 574.4600; 578.8960; 593.0361; 615.0854; 626.3193; 626.9096; 629.8748; 636.9484; 650.7213; 712.9554; 721.1446; 741.4969; 750.2179; 765.5201; 766.0206; 795.2490; 853.0030; 876.6840; 937.7318; 940.5261; 944.4306; 955.0617; 964.1552; 977.6531; 985.4054; 991.6061; 1003.4524; 1009.3105; 1016.0525; 1024.2909; 1055.0289; 1057.0127; 1068.7359; 1069.3910; 1072.5520; 1077.4052; 1097.2120; 1110.8776; 1192.2592; 1195.1463; 1199.5930; 1203.2287; 1218.4711; 1242.0302; 1253.7152; 1255.9883; 1262.2386; 1268.7006; 1289.5844; 1306.5404; 1310.0254; 1320.8232; 1326.9696; 1363.5256; 1414.5672; 1435.3181; 1437.4494; 1453.8568; 1462.8271; 1465.0225; 1503.2382; 1504.4367; 1507.3983; 1513.7082; 1515.8040; 1516.5497; 1532.8117; 1535.2416; 1549.1077; 1609.3176; 1615.2595; 1618.8849; 1640.7849; 1797.0948; 3048.4563; 3062.6361; 3066.0649; 3073.0039; 3107.5709; 3132.9787; 3135.6374; 3140.7284; 3141.8594; 3144.8676; 3145.2873; 3147.5666; 3155.0545; 3169.7850; 3178.6727; 3188.2959; 3204.2253.

Cartesian Forces: Max 0.000001174 RMS 0.000000395

Item	Value	Threshold	Converged?
Maximum Force	0.000000	0.000450	YES
RMS Force	0.000000	0.000300	YES
Maximum Displacement	0.000025	0.001800	YES
RMS Displacement	0.000003	0.001200	YES

FILE:
Yb_Sq2_PhYbOTf2_TS_Neutral_Add12_B3LYP_M062x_Conf3.out

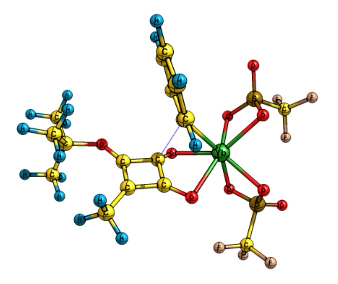

Conformer 3

E(RB3LYP/6-31G(d)/MWB59) = -2769.88703078
Convg = 0.7195D-08 -V/T = 2.0149

Temperature 195.150 Kelvin. Pressure 1.00000 Atm.
elec. and zero-point Energies= -2769.543950
elec. and thermal Energies= -2769.525335
elec. and thermal Enthalpies= -2769.524717
elec. and thermal Free Energies= -2769.584800

RB3LYP/6-31G(d)/MWB59/THF(IEFPCM) = -2769.91266082

RM06-2x/6-311+G(d,p)/MWB59/THF(IEFPCM) = -2769.72022949

Geometry at RB3LYP/6-31G(d)/MWB59
Charge = 0 Multiplicity = 1
Yb 1.1795601324 2.0558221591 -0.4606376966
O 0.5174178459 4.3134970533 -0.4228725895
S 0.7246744718 4.5638474844 1.0604183142
O 1.5454781351 5.7023099235 1.4347326417
O 1.0971678549 3.2074214933 1.627177308
C -0.9768397291 4.9215569975 1.7434148006
F -1.4916018532 5.9679027562 1.098738131
F -1.7807172317 3.8678025066 1.5908377367
F -0.8661914145 5.2036229309 3.0415808064
O 2.6728810546 2.8473969486 -2.0791963366
O 4.8296500887 1.5224353506 -2.3518550566
O 3.4453518211 1.5534904327 -0.2180752298
S 3.9337101537 2.2547361393 -1.476019497
C 4.8748648044 3.7347816634 -0.8373391083
F 5.2303685799 4.5032305803 -1.8641876108
F 4.0969297984 4.4298089442 -0.0049776178
F 5.9599569595 3.3160677653 -0.1893610756
C -1.097365529 -1.3591499842 0.176529854
C -2.1886719628 -0.5288658009 -0.1288725004
C -1.3223907705 0.6445278191 -0.1332505975
C -0.1501544766 -0.1903616268 0.2770145914
O 0.8879594429 0.0995388887 0.902708326
O -1.3595910689 1.8532212651 -0.3854294441
O -0.755853422 -2.5999901779 0.3508692764
C -1.6297912927 -3.7982207586 0.1851863998
C -3.6375255558 -0.6192174963 -0.4639458794
H -4.1631660309 -1.3985935643 0.0932881788
H -3.7751936774 -0.8187152388 -1.5356139842
H -4.1194620433 0.3402333478 -0.2510055605
C -2.6672704249 -3.802650902 1.3076533451
H -3.3514180156 -2.9526728875 1.2418724834
H -3.2649703296 -4.7183337276 1.2490381543
H -2.1756108741 -3.7765794473 2.2849411647
C -0.6319828559 -4.9447666144 0.351159398
H -1.154406486 -5.9038574018 0.278637136
H 0.1347524154 -4.9050478521 -0.428104637
H -0.1390874565 -4.8890911924 1.3261790078
C -2.2362786812 -3.7966733167 -1.2178415628
H -1.4536930409 -3.6749254378 -1.9732135049
H -2.7358747347 -4.7551304231 -1.3936015289
H -2.9795460683 -3.0079586124 -1.352724367
C 0.635540104 0.1562700555 -1.9803561774
C 1.6082332751 -0.8558420029 -2.1068238932
C -0.1965355252 0.3890498736 -3.0969605387
H 2.2542752225 -1.0904039156 -1.2604784354
H -0.9830795061 1.1438736448 -3.0428419618
C 1.779985031 -1.5683600282 -3.2992519189
C -0.0320295249 -0.3113260759 -4.2940895153
H 2.5493826718 -2.3335525381 -3.3730521201
H -0.6687041078 -0.0929299303 -5.1488765543
C 0.958659416 -1.2945603682 -4.3944725249
H 1.0869287776 -1.8445069289 -5.3235939939

Frequency: -158.2379; 12.8120; 16.4324; 16.6660; 24.3081; 29.5705; 31.5353; 35.2007; 42.6456; 44.7699; 51.3563; 53.8664; 60.8371; 71.2651; 79.3121; 87.6932; 101.2843; 106.8988; 111.9645; 118.0096; 139.4156; 147.9749; 159.5941; 160.4433; 167.5383; 187.2910; 191.4832; 192.7052; 210.6044; 219.3125; 221.2571; 232.3767; 239.5088; 243.4414; 251.3807; 261.8028; 267.0681; 276.5954; 304.3091; 307.3780; 312.4165; 318.0447; 321.2473; 346.3840; 356.5662; 359.9804; 367.8451; 394.6571; 399.4704; 422.0986; 450.5181; 477.4285; 482.6718; 489.5270; 491.2386; 531.0965; 535.2729; 557.8927; 558.3750; 574.0425; 579.2545; 592.2228; 615.5369; 626.8775; 627.1399; 633.4405; 639.1105; 652.8078; 715.3331; 720.0985; 749.0550; 765.4758; 765.8923; 768.0780; 799.8028; 837.9180; 875.4409; 935.4944; 935.5911; 937.8553; 955.0666; 964.4093; 981.4611; 982.8398; 992.5574; 1003.6701; 1007.4250; 1014.0639; 1017.6286; 1052.2645; 1055.2427; 1061.7164; 1068.5180; 1069.4220; 1077.7529; 1096.2215; 1135.4549; 1191.4021; 1195.6211; 1199.7661; 1200.9385; 1203.6739; 1218.3024; 1253.4392; 1255.5642; 1261.2312; 1268.3179; 1288.5648; 1296.3686; 1307.8695; 1320.4207; 1327.9646; 1364.4308; 1407.7139; 1434.0078; 1436.4955; 1446.6048; 1461.0502; 1464.2356; 1500.5873; 1506.2924; 1511.1040; 1515.0829; 1515.5462; 1517.9501; 1528.0221; 1530.8278; 1548.0546; 1608.9764; 1614.8852; 1619.5857; 1680.6487; 1802.7926; 3044.4169; 3067.7055; 3070.3394; 3076.6961; 3112.2079; 3133.0547; 3137.7875; 3138.2425; 3146.1329; 3147.0868; 3148.7443; 3152.2035; 3155.2639; 3165.3604; 3175.6687; 3185.5132; 3201.5636.

Cartesian Forces: Max 0.000006937 RMS 0.000001039

Item	Value	Threshold	Converged?
Maximum Force	0.000004	0.000450	YES
RMS Force	0.000000	0.000300	YES
Maximum Displacement	0.000482	0.001800	YES
RMS Displacement	0.000087	0.001200	YES

FILE: Yb_Sq2_PhYbOTf2_TS_Neutral_Add12_B3LYP_M062x_Conf3-.out

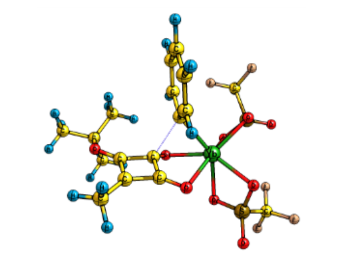

Conformer 4

E(RB3LYP/6-31G(d)/MWB59) = -2769.89343655
Convg = 0.9077D-08 -V/T = 2.0149

Temperature 195.150 Kelvin. Pressure 1.00000 Atm.
elec. and zero-point Energies= -2769.550134
elec. and thermal Energies= -2769.531616
elec. and thermal Enthalpies= -2769.530998
elec. and thermal Free Energies= -2769.590552

RB3LYP/6-31G(d)/MWB59/THF(IEFPCM) = -2769.91645724

RM06-2x/6-311+G(d,p)/MWB59/THF(IEFPCM) = -2769.72370373

Geometry at RB3LYP/6-31G(d)/MWB59
Charge = 0 Multiplicity = 1
Yb 1.452223764 1.9583416566 -0.6077256564
O 1.0366375323 2.9566256267 1.5149146335
S 0.560910264 4.2878655788 0.96914523
O -0.6964971798 4.8185658856 1.4646660073
O 0.7265019589 4.1597688562 -0.5397243419
C 1.8900739109 5.5027492626 1.4604157638
F 1.9170323952 5.6030905911 2.7890257238
F 3.0749278871 5.077100076 1.0210552321
F 1.6154530978 6.6904861278 0.9249174919
O 3.7545675944 2.109548157 -0.177006997
O 4.9014035716 4.0039110901 -1.4300812793
O 2.8789914944 2.7414176728 -2.3134285284
S 4.1531234487 2.7619037461 -1.491050729
C 5.2847357832 1.5220450488 -2.3083109616
F 6.4407635072 1.4919093271 -1.6484679169
F 4.7342605727 0.3033623388 -2.2847354417
F 5.4938539406 1.8874284962 -3.5707417135
C -0.6459428522 -1.5620176324 0.2419913477
C -1.7664512627 -0.7470593311 0.0819172146
C -0.9364787872 0.4328337049 -0.0727677709
C 0.311503801 -0.3857717687 0.1262140387
O 1.4226668138 -0.0730058934 0.6274845659
O -1.0226643855 1.6434764168 -0.3012319384
O -0.6033745869 -2.8506874322 0.4238535815
C 0.6054615874 -3.5801685628 0.9359731369
C -3.228514351 -1.0048002112 0.0490719153
H -3.4546957013 -2.0243918215 0.3732577544
H -3.6180568003 -0.8718854323 -0.9689033541
H -3.7639173821 -0.29854385 0.6932005447
C 1.7744958402 -3.3931370099 -0.0265967566
H 2.1651918471 -2.3742568216 0.0111026035
H 2.5837272534 -4.07239353 0.2628042704
H 1.4786692945 -3.630132336 -1.0531134688
C 0.1159780744 -5.0265584523 0.9591322882
H 0.9057282066 -5.6757548406 1.3496816481
H -0.7650418141 -5.1287871172 1.6001187184
H -0.1440228013 -5.365191822 -0.0486422058
C 0.911209148 -3.0556692074 2.3389431971
H 0.0371491189 -3.1544538993 2.9907877151
H 1.7287539921 -3.6402937152 2.77404426
H 1.2271568105 -2.0084413895 2.3130473167
C 0.7790311258 0.0926258959 -2.1549400833
C 1.8184112375 -0.7720106056 -2.5448281413
C -0.1990770189 0.39844947 -3.1259296761
H 2.5833120509 -1.0556911346 -1.8232317848
H -1.0393016422 1.044157464 -2.8686174139
C 1.9145381371 -1.2729122245 -3.847845477
C -0.11319119 -0.0909222988 -4.4315137139
H 2.7362604977 -1.9301374503 -4.1235123631
H -0.866617238 0.1803255397 -5.1675945659
C 0.9454345193 -0.9308242844 -4.7926316266
H 1.0105119142 -1.3192029546 -5.8058652933

Frequency: -204.0942; 8.6037; 22.6017; 26.4969; 30.2761; 31.6917; 32.7399; 36.1079; 41.4745; 48.4688; 50.9321; 55.4756; 64.1523; 69.7846; 73.7078; 84.1319; 90.8095; 96.7347; 109.8745; 117.4177; 136.5282; 152.3721; 158.1099; 166.5270; 175.7015; 184.2475; 187.5344; 202.0479; 217.4700; 218.9398; 219.4122; 222.0482; 244.9271; 247.2592; 273.3318; 274.2597; 280.2062; 284.8663; 305.4508; 308.4035; 318.7009; 322.1208; 330.6587; 344.2935; 351.8327; 363.0701; 367.8997; 399.3261; 407.3680; 424.6661; 452.3364; 465.0047; 488.4971; 489.7860; 490.3647; 531.2772; 535.1567; 557.5986; 558.4226; 574.1201; 576.9512; 593.7227; 613.5061; 626.5360; 627.5051; 633.5750; 636.8931; 652.2313; 712.9160; 722.7721; 741.4717; 751.4400; 765.6013; 765.7371; 795.3024; 855.9154; 878.5754; 938.1930; 942.8850; 944.6268; 953.1988; 962.7957; 976.8462; 986.6480; 992.5480; 1004.5998; 1008.3200; 1016.2929; 1021.0221; 1055.4279; 1057.4901; 1068.1303; 1069.9450; 1071.9923; 1075.1413; 1096.8192; 1104.5579; 1191.9629; 1196.8186; 1201.4967; 1203.3146; 1217.4188; 1243.3533; 1247.2962; 1257.0031; 1264.5899; 1267.5809; 1289.2302; 1306.6986; 1311.9454; 1322.2385; 1327.8617; 1362.6277; 1411.9530; 1434.8946; 1436.9716; 1451.8716; 1462.4268; 1465.2896; 1502.9697; 1503.6306; 1507.2233; 1514.6664; 1516.0945; 1516.5475; 1532.5670; 1534.9535; 1550.2507; 1601.2215; 1615.8346; 1619.1564; 1631.8768; 1802.2275; 3048.8657; 3062.5134; 3065.4203; 3071.9839; 3107.4591; 3132.8719; 3134.5776; 3141.7254; 3144.9330; 3145.1161; 3146.7452; 3147.6130; 3158.5558; 3167.6543; 3179.3801; 3187.1730; 3204.0892.

Cartesian Forces: Max 0.000000696 RMS 0.000000221

Item	Value	Threshold	Converged?
Maximum Force	0.000000	0.000450	YES
RMS Force	0.000000	0.000300	YES
Maximum Displacement	0.000033	0.001800	YES
RMS Displacement	0.000005	0.001200	YES

FILE: Yb_Sq2_PhYbOTf2_TS_Neutral_Add12_B3LYP_M062x_Conf4.out

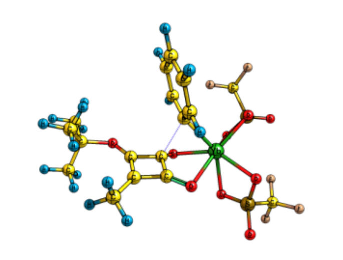

Conformer 5

E(RB3LYP/6-31G(d)/MWB59) = -2769.88742184
Convg = 0.2470D-09 -V/T = 2.0149

Temperature 195.150 Kelvin. Pressure 1.00000 Atm.
elec. and zero-point Energies= -2769.544331
elec. and thermal Energies= -2769.525715
elec. and thermal Enthalpies= -2769.525097
elec. and thermal Free Energies= -2769.585368

RB3LYP/6-31G(d)/MWB59/THF(IEFPCM) = -2769.91293862

RM06-2x/6-311+G(d,p)/MWB59/THF(IEFPCM) = -2769.71982119

Geometry at RB3LYP/6-31G(d)/MWB59
Charge = 0 Multiplicity = 1
Yb 1.4054547616 2.0218294334 -0.5157532768
O 0.962168606 3.2013543099 1.5100909631
S 0.609636596 4.5166710275 0.8468585784
O -0.6190852834 5.1780193184 1.2482625207
O 0.8201605379 4.2623802602 -0.6403542015
C 2.0128300789 5.6610448867 1.2987062567
F 2.0090668092 5.8570801784 2.6166314349
F 3.1735705501 5.1145423102 0.9323026796
F 1.8495663782 6.8238035566 0.6704999162
O 3.6774992997 1.9658980208 0.0366573778
O 5.1219092414 3.6170242703 -1.252515843
O 2.9860110431 2.5852046821 -2.1692742639
S 4.2157604013 2.4840772946 -1.2873600756
C 5.1938948173 1.0548644763 -1.982831876
F 6.2946184734 0.8861525598 -1.2530553496
F 4.4634129274 -0.0639868659 -1.944003551
F 5.5246567286 1.323731155 -3.2438606315
C -0.9739367107 -1.2878745134 0.2393314757
C -2.0574120156 -0.4125099256 0.0787031262
C -1.1511866069 0.729747226 -0.0168675807
C 0.0280204307 -0.162406408 0.2433786241
O 1.1371043269 0.0572236763 0.7809204905
O -1.176625447 1.9358407446 -0.2626103343
O -0.6635334067 -2.54562408 0.3583479638
C -1.6052810448 -3.7000184552 0.3093793812
C -3.5394374892 -0.4350403623 -0.0725530939
H -4.026075849 -1.1991956825 0.5386044471
H -3.8195339128 -0.611446877 -1.1202646142
H -3.9461130315 0.5414517687 0.2088826014
C -2.4866945846 -3.6639142602 1.5578710539
H -3.131956386 -2.7820543163 1.5831025921
H -3.1299759747 -4.549920099 1.577296874
H -1.8708366306 -3.6676093987 2.4624129249
C -0.6495847674 -4.8932219118 0.344297925
H -1.2213287562 -5.8265697778 0.3429213441
H 0.0088879184 -4.8862897048 -0.5293646616
H -0.0305481696 -4.8650014525 1.2458260775
C -2.3901258568 -3.6644915936 -1.0019750479
H -1.708074636 -3.5769007735 -1.8535066489
H -2.953470107 -4.597391499 -1.1090855341
H -3.1060939561 -2.8407517827 -1.0401686922
C 0.6186285498 0.197411232 -2.031502148
C 1.4542025095 -0.9126832072 -2.2647492529
C -0.2381782804 0.5877913922 -3.0845031923
H 2.1016267239 -1.2748357458 -1.4660829823
H -0.9317447189 1.4182459255 -2.9436443238
C 1.4789474891 -1.5715309924 -3.4991675437
C -0.2149571627 -0.0540141995 -4.3247894931
H 2.1401171723 -2.4214946974 -3.6536672269
H -0.8632475589 0.2860879926 -5.1293944741
C 0.6452289194 -1.1381763282 -4.5318212207
H 0.6592643217 -1.6451692756 -5.493528348

Frequency: -177.0140; 7.2175; 17.1877; 19.8410; 27.6926; 29.5574; 31.5938; 32.1624; 41.7937; 44.4160; 50.7643; 59.5928; 64.1168; 71.0211; 75.2406; 91.5608; 95.7066; 103.1069; 106.5688; 116.3333; 141.7255; 148.2480; 156.6331; 165.4573; 170.5303; 186.1108; 187.9598; 190.5853; 210.7145; 218.0759; 220.9140; 232.7724; 242.3788; 244.6448; 250.4305; 262.0852; 271.2579; 275.8309; 305.2545; 308.2984; 313.7715; 318.0293; 322.0978; 346.5785; 354.5638; 363.3778; 368.4708; 395.9718; 402.2924; 426.8021; 450.3388; 477.7557; 482.8830; 488.9324; 490.4466; 531.2543; 535.2042; 557.6756; 558.2699; 575.3841; 577.1481; 593.7636; 618.2036; 626.7272; 627.3932; 634.0008; 639.3064; 653.7551; 715.6430; 722.2281; 750.3452; 765.3571; 765.7104; 768.0463; 799.5785; 839.8388; 877.5773; 935.5244; 937.4773; 938.2509; 953.2894; 962.7880; 982.1757; 983.3074; 992.4770; 1004.8066; 1006.9108; 1011.6187; 1017.3176; 1052.4831; 1056.1256; 1061.0326; 1068.1428; 1069.1903; 1075.6219; 1096.2230; 1131.2429; 1191.5158; 1196.2475; 1201.1535; 1202.1993; 1203.5492; 1218.0612; 1248.9526; 1255.1112; 1264.6265; 1267.0755; 1288.0456; 1296.0585; 1308.3776; 1322.6055; 1328.7779; 1364.0777; 1404.9529; 1433.8452; 1436.0021; 1445.8169; 1460.8027; 1464.3984; 1500.4114; 1505.6275; 1510.8154; 1515.1003; 1515.3158; 1518.0074; 1527.9362; 1530.6265; 1548.2683; 1610.0459; 1614.9494; 1619.6938; 1661.8354; 1810.5279; 3044.0764; 3067.2968; 3070.2482; 3076.4873; 3112.2023; 3137.1726; 3138.0380; 3139.7358; 3146.0553; 3146.5342; 3147.8757; 3151.8736; 3155.6306; 3165.6945; 3176.4686; 3184.9975; 3201.7146.

Cartesian Forces: Max 0.000000737 RMS 0.000000305

Item	Value	Threshold	Converged?
Maximum Force	0.000000	0.000450	YES
RMS Force	0.000000	0.000300	YES
Maximum Displacement	0.000010	0.001800	YES
RMS Displacement	0.000002	0.001200	YES

FILE: Yb_Sq2_PhYbOTf2_TS_Neutral_Add12_B3LYP_M062x_Conf4-.out

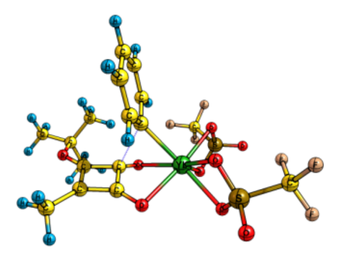

Conformer 6

E(RB3LYP/6-31G(d)/MWB59) = -2769.89506824
Convg = 0.3834D-08 -V/T = 2.0149

Temperature 195.150 Kelvin. Pressure 1.00000 Atm.
elec. and zero-point Energies= -2769.552030
elec. and thermal Energies= -2769.533334
elec. and thermal Enthalpies= -2769.532716
elec. and thermal Free Energies= -2769.592619

RB3LYP/6-31G(d)/MWB59/THF(IEFPCM) = -2769.91796389

RM06-2x/6-311+G(d,p)/MWB59/THF(IEFPCM) = -2769.72491033

Geometry at RB3LYP/6-31G(d)/MWB59
Charge = 0 Multiplicity = 1
Yb 2.5251618876 0.6663418804 -1.2122736242
O 3.1934964989 2.8666164048 -0.7478471912
S 2.63241314 3.51164412 -2.003469908
O 1.8176518566 4.7034394371 -1.8498281312
O 2.046545454 2.3498989122 -2.7855541079
C 4.1481281434 4.0088525865 -2.9722783937
F 4.8005850367 4.9590644446 -2.3058210138
F 4.9433942175 2.9495042696 -3.1336423829
F 3.7735526677 4.4704304209 -4.1642946382
O 4.6105048397 0.0864990975 -0.1933876269
O 6.6261251444 0.1802329139 -1.7421511031
O 4.2399352699 -0.1755003205 -2.5466419696
S 5.3003622107 -0.3415016112 -1.4675365967
C 5.4838284866 -2.1915743202 -1.3101853503
F 6.2912965012 -2.4718544579 -0.2899602825
F 4.2851744665 -2.752202021 -1.0882719909
F 5.9926538629 -2.6845065124 -2.4373381145
C -0.638038104 -1.5599183818 0.4463436516
C -1.2418823906 -0.2986840276 0.5100094792
C -0.0016759681 0.3531084564 0.140717925
C 0.7067669305 -0.9620141981 0.0711276189
O 1.9218227518 -1.2007878271 0.2457465788
O 0.494357121 1.4719510129 -0.0491537261
O -1.1687589678 -2.7223814432 0.6794061056
C -0.3641453547 -3.980256877 0.868095753
C -2.6175677253 0.1632612571 0.822917956
H -3.2092672625 -0.6396415715 1.2714069218
H -3.1256482428 0.4942661445 -0.0926117523
H -2.5970015239 1.018517764 1.5070200261
C 0.448496499 -4.2674968223 -0.3904986036
H 1.2664825713 -3.5552729555 -0.5147629602
H 0.8867668417 -5.2679577524 -0.3063195512
H -0.1846633529 -4.24406895 -1.282302623
C -1.4565131939 -5.0233175421 1.0912381303
H -0.9988428301 -6.0005475943 1.2734076998
H -2.072980421 -4.7628725023 1.9570918492
H -2.103566912 -5.1026419994 0.2122159998
C 0.5133213066 -3.7937374379 2.1056505478
H -0.0933232577 -3.5306714322 2.9782972383
H 1.0342158919 -4.7321061366 2.3236164707
H 1.2721562252 -3.0219983474 1.9448942219
C 0.7657721363 -0.6715111844 -2.3648330548
C 1.2586478454 -1.8688290563 -2.920034147
C -0.3144155472 -0.0563612882 -3.0320639006
H 2.0838787542 -2.3927184013 -2.437608089
H -0.7325847587 0.8733052778 -2.6458587438
C 0.7371572152 -2.406773975 -4.1027906431
C -0.8510575646 -0.5861387764 -4.2065834848
H 1.1527812393 -3.3222786428 -4.5179645407
H -1.6717636998 -0.0799169158 -4.7098977908
C -0.3238596396 -1.7658440108 -4.743770421
H -0.7401572963 -2.1813861075 -5.6581337163

Frequency: -183.5307; 13.9505; 17.7227; 26.3790; 29.2055; 31.3439; 35.5867; 35.8960; 43.9676; 46.1673; 49.5054; 53.1549; 61.4853; 66.9655; 67.3592; 75.0290; 80.9164; 97.6214; 102.8999; 118.2611; 127.2493; 148.3711; 157.5690; 161.6244; 169.5755; 179.6164; 190.5492; 200.2408; 215.5958; 216.0252; 216.9929; 220.6097; 233.5187; 237.7184; 265.8069; 270.0977; 276.9943; 280.1576; 303.4852; 307.1621; 319.2830; 320.4900; 332.1941; 341.0264; 350.5283; 362.3377; 365.9066; 397.8080; 401.7951; 412.0156; 452.2853; 465.2147; 488.5515; 489.6809; 490.6906; 531.1972; 534.8049; 557.2960; 558.0606; 576.7227; 578.6774; 594.8809; 613.2678; 625.6113; 626.8510; 631.4477; 635.2888; 646.8660; 712.8427; 717.3000; 742.0925; 750.0763; 763.2755; 765.7914; 795.0979; 853.3223; 873.6854; 937.4198; 939.8695; 944.4997; 956.5791; 963.3204; 978.7752; 984.7682; 991.3552; 1002.0198; 1008.9370; 1016.8513; 1026.2000; 1055.5890; 1060.6127; 1068.8833; 1069.5222; 1072.6760; 1081.4542; 1097.2412; 1119.3867; 1191.5025; 1194.8554; 1201.3851; 1202.5825; 1219.2858; 1237.5055; 1241.6848; 1255.3702; 1265.6456; 1267.6860; 1289.5628; 1306.8994; 1311.6241; 1323.6357; 1330.8598; 1365.4174; 1414.4343; 1435.2495; 1437.7149; 1453.3487; 1462.8715; 1464.6050; 1503.3337; 1503.5842; 1506.5027; 1512.8080; 1516.5203; 1517.7904; 1532.4774; 1535.0458; 1548.1922; 1608.3457; 1615.4716; 1619.6626; 1650.4169; 1799.8877; 3049.0277; 3062.6449; 3066.4532; 3073.4884; 3108.6312; 3133.3487; 3136.5668; 3142.4745; 3145.4941; 3145.7082; 3146.4801; 3150.0623; 3153.5434; 3170.5407; 3177.3830; 3185.9486; 3202.2576.

Cartesian Forces: Max 0.000006091 RMS 0.000001136

Item	Value	Threshold	Converged?
Maximum Force	0.000002	0.000450	YES
RMS Force	0.000001	0.000300	YES
Maximum Displacement	0.000626	0.001800	YES
RMS Displacement	0.000120	0.001200	YES

FILE: Yb_Sq2_PhYbOTf2_TS_Neutral_Add12_B3LYP_M062x_Conf5.out

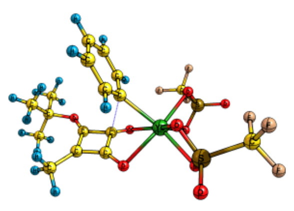

Conformer 7

E(RB3LYP/6-31G(d)/MWB59) = -2769.88854164
Convg = 0.8251D-09 -V/T = 2.0149

Temperature 195.150 Kelvin. Pressure 1.00000 Atm.
elec. and zero-point Energies= -2769.545511
elec. and thermal Energies= -2769.526841
elec. and thermal Enthalpies= -2769.526223
elec. and thermal Free Energies= -2769.586126

RB3LYP/6-31G(d)/MWB59/THF(IEFPCM) = -2769.91414331

RM06-2x/6-311+G(d,p)/MWB59/THF(IEFPCM) = -2769.72157330

Geometry at RB3LYP/6-31G(d)/MWB59
Charge = 0 Multiplicity = 1

```
Yb 2.5248211839 0.717058308 -1.1277921958
O 3.3303834554 2.9045111238 -0.8327072843
S 2.7468816201 3.5033871964 -2.1002043406
O 2.0054502474 4.7468218239 -1.9868325217
O 2.0638428842 2.3330521393 -2.7839148449
C 4.2397313085 3.8565817322 -3.1627410422
F 4.9734973074 4.8053281385 -2.5842425406
F 4.9683847695 2.7468251388 -3.2957126211
F 3.8346121842 4.2697529701 -4.3629090225
O 4.5974711132 0.0728923136 -0.131245387
O 6.5654676396 -0.0486100348 -1.7393639719
O 4.1372527988 -0.2935158331 -2.453789273
S 5.2199238665 -0.4700977489 -1.3968439396
C 5.2884626802 -2.3164123861 -1.1366960921
F 6.1405249364 -2.5910103306 -0.1515932437
F 4.073299161 -2.7740432579 -0.809655716
F 5.6922534484 -2.9066353255 -2.2600056393
C -0.7724636306 -1.1613070182 0.5367820986
C -1.3191459754 0.1355205653 0.5752970633
C -0.0226329296 0.7134863783 0.2433855556
C 0.6127062286 -0.6352660998 0.2705740868
O 1.8008585222 -0.9448608135 0.4813441184
O 0.516231117 1.7968994622 -0.0044671384
O -1.0825250824 -2.4170548441 0.6390910619
C -2.4575606911 -2.9965051392 0.7190448631
C -2.631068541 0.8170487957 0.7531973995
H -3.2962876932 0.3015920517 1.4499502536
H -3.1513968023 0.9061178593 -0.2104226069
H -2.464841915 1.834269659 1.1219111933
C -3.0312953807 -2.6944580993 2.1027886613
H -3.1755887759 -1.6230309155 2.2649184264
H -4.0057832491 -3.1826823374 2.2087184792
H -2.3694833296 -3.0774445559 2.8856311971
C -2.179920663 -4.4901622792 0.5474814518
H -3.1173323108 -5.0511908658 0.6146814003
H -1.7235699675 -4.6865423607 -0.4271008436
H -1.5023216236 -4.8492166989 1.3277503417
C -3.3051341122 -2.4596815996 -0.434150766
H -2.7817372162 -2.5850077915 -1.3872703658
H -4.2426096389 -3.0237752432 -0.4800682397
H -3.5619554563 -1.4053692634 -0.3107910141
C 0.6867545777 -0.5962439651 -2.1692234828
C 0.9029933046 -1.9707687879 -2.3976967843
C -0.281901753 0.0392151564 -2.9779919661
H 1.6188151802 -2.5145535983 -1.7811056262
H -0.4869223091 1.1009471497 -2.8442795897
C 0.2276463337 -2.6710402823 -3.4049676177
C -0.9673051278 -0.6483962225 -3.9809698877
H 0.4300104709 -3.7275669162 -3.5675491529
H -1.6905363965 -0.1274141443 -4.6048430235
C -0.7134135361 -2.00894373 -4.1950897576
H -1.2456067729 -2.5459835531 -4.9767161224
```

Frequency: -156.3857; 13.8209; 18.0249; 23.3699; 26.8411; 29.0562; 32.4325; 36.8408; 41.6817; 45.5760; 50.7856; 58.9557; 60.8008; 65.3943; 69.3164; 79.4187; 99.0031; 105.6426; 112.1013; 124.8820; 131.4739; 146.6322; 157.4054; 160.2658; 168.1845; 183.8881; 187.3950; 195.7161; 204.1823; 217.3008; 220.3146; 233.7059; 234.6483; 237.4291; 251.9195; 260.9548; 267.7876; 275.2314; 303.4827; 307.0695; 312.3779; 319.0298; 320.6754; 342.4875; 354.8677; 362.9746; 366.7002; 392.9512; 398.4244; 417.5233; 450.6164; 475.9684; 482.2593; 489.4664; 490.6846; 531.2707; 534.9296; 557.5003; 558.1293; 577.1058; 578.7652; 593.3638; 614.8573; 626.0864; 627.4235; 632.7459; 638.2765; 647.8323; 715.8023; 717.4502; 749.8176; 764.3138; 765.7551; 768.2433; 798.6288; 836.1462; 872.5061; 933.4062; 935.5824; 938.6363; 956.5155; 963.1423; 979.7817; 982.9573; 993.1924; 999.8225; 1007.9793; 1014.7571; 1017.5228; 1050.9944; 1061.2427; 1061.5321; 1068.9078; 1069.8233; 1079.5795; 1096.2429; 1145.7484; 1190.5662; 1195.4739; 1199.3514; 1202.0955; 1203.4824; 1220.5513; 1243.1450; 1255.4603; 1264.6695; 1266.8731; 1288.8049; 1296.3019; 1308.7037; 1322.8957; 1330.4536; 1367.1649; 1408.9008; 1434.5324; 1436.4150; 1446.9154; 1461.4189; 1463.4723; 1500.2070; 1505.1416; 1510.1289; 1515.2076; 1516.9502; 1517.8049; 1527.9580; 1531.4243; 1548.2080; 1606.8163; 1613.5613; 1619.8111; 1684.3397; 1807.2184; 3045.2875; 3067.4150; 3069.9723; 3076.2677; 3112.5996; 3137.8190; 3138.2034; 3146.7050; 3147.2370; 3148.8278; 3154.5682; 3155.2621; 3157.4376; 3164.9839; 3175.0060; 3183.7223; 3199.2118.

Cartesian Forces: Max 0.000008439 RMS 0.000001660

Item	Value	Threshold	Converged?
Maximum Force	0.000006	0.000450	YES
RMS Force	0.000001	0.000300	YES
Maximum Displacement	0.000667	0.001800	YES
RMS Displacement	0.000153	0.001200	YES

FILE: Yb_Sq2_PhYbOTf2_TS_Neutral_Add12_B3LYP_M062x_Conf5-.out

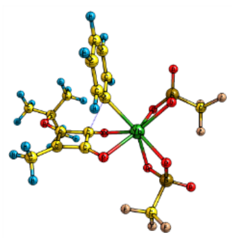

Conformer 8

EE(RB3LYP/6-31G(d)/MWB59) = -2769.89438813
Convg = 0.8494D-08 -V/T = 2.0149

Temperature 195.150 Kelvin. Pressure 1.00000 Atm.
elec. and zero-point Energies= -2769.551201
elec. and thermal Energies= -2769.532605
elec. and thermal Enthalpies= -2769.531987
elec. and thermal Free Energies= -2769.591831

RB3LYP/6-31G(d)/MWB59/THF(IEFPCM) = -2769.91669945

RM06-2x/6-311+G(d,p)/MWB59/THF(IEFPCM) = -2769.72488380

Geometry at RB3LYP/6-31G(d)/MWB59
Charge = 0 Multiplicity = 1

```
Yb 1.2454735197 2.0332036006 -0.5459717025
O 0.4137021131 4.2275443823 -0.3821438559
S 0.6692825366 4.4321970692 1.1004248069
O 1.4089509703 5.6191170977 1.4921330565
O 1.181391103 3.0888520394 1.5856444655
C -1.0221083215 4.6101648738 1.873214178
F -1.6495344986 5.6413770366 1.30984501
F -1.7425666974 3.5014512692 1.6955239739
F -0.8743748552 4.8323410478 3.1792563716
O 2.6001037434 3.0010106064 -2.1907635715
O 4.8564753175 1.8912451816 -2.5890736552
O 3.5641084895 1.7276100665 -0.4055673189
S 3.9334217503 2.5105311766 -1.6555613773
C 4.7606864598 4.0516722871 -1.0036493174
F 4.9977166207 4.8820758348 -2.0160206686
F 3.9596654171 4.6432409729 -0.1154023651
F 5.9075039409 3.7156503036 -0.4164389028
C -0.7360687896 -1.5896380273 0.188811296
C -1.874221422 -0.8269809228 -0.0872710598
C -1.0892108856 0.3888428106 -0.1555565025
C 0.1653761965 -0.365043917 0.1739039773
O 1.2013716676 0.0173641166 0.7661895111
O -1.2008253705 1.601500826 -0.3806874885
O -0.6471502202 -2.872314361 0.3770679492
C 0.5720057604 -3.5462371768 0.9470837572
C -3.3093642931 -1.1594327505 -0.2767686517
H -3.5210365312 -2.1821141701 0.0476106457
H -3.5860591466 -1.07085552 -1.3357585949
H -3.9492310619 -0.4685363762 0.2829653729
C 1.7756408236 -3.2874194565 0.0461118827
H 2.1086161672 -2.2493798878 0.1057742756
H 2.6054047506 -3.9227038923 0.3745344254
H 1.5473003922 -3.5333469599 -0.9952952979
C 0.1559061642 -5.0152293902 0.9326493859
H 0.9573948544 -5.6275935796 1.357207086
H -0.7497359047 -5.1695022143 1.5273346441
H -0.0348339451 -5.3554461106 -0.0899153103
C 0.7764390067 -3.0211527511 2.3678784936
H -0.1245483841 -3.1699291322 2.9719497017
H 1.5979089121 -3.5698603685 2.8406533191
H 1.042325684 -1.9598810568 2.3670315907
C 0.8109971651 0.1170806788 -2.1024735658
C 1.9658706913 -0.6299271596 -2.4072393658
C -0.1405985743 0.272899013 -3.1326125482
H 2.7270911377 -0.7865486765 -1.6432845957
H -1.0603112811 0.829104236 -2.9461383476
C 2.1874321932 -1.1593913015 -3.6834346621
C 0.0680537625 -0.2503906065 -4.4110297066
H 3.0984151728 -1.7146192798 -3.8940495089
H -0.6725314525 -0.0964028154 -5.192794985
C 1.2348021093 -0.9705521357 -4.686318448
H 1.3990420413 -1.3820835308 -5.6789948021
```

Frequency: -189.4681; 12.0705; 16.3794; 25.0049; 29.7534; 32.0400; 32.4224; 37.0172; 39.1396; 45.0100; 49.0421; 53.2515; 63.6543; 65.2520; 75.7015; 82.8590; 86.1527; 97.4800; 106.9008; 119.0939; 131.4053; 157.4879; 161.0522; 162.7911; 168.4211; 183.7396; 191.9401; 203.0390; 212.5254; 217.9938; 220.5717; 224.4803; 239.2369; 244.2779; 271.5387; 273.0298; 277.6081; 283.4993; 304.2369; 307.3996; 318.0379; 321.2292; 331.9203; 342.5885; 352.8971; 359.9665; 366.5601; 399.1185; 403.4870; 419.3964; 452.6237; 463.9415; 488.8944; 489.8643; 491.2662; 531.1619; 535.1512; 557.8927; 558.3821; 574.4600; 578.8960; 593.0359; 615.0854; 626.3192; 626.9095; 629.8746; 636.9481; 650.7210; 712.9563; 721.1450; 741.4969; 750.2181; 765.5200; 766.0206; 795.2490; 853.0029; 876.6838; 937.7318; 940.5259; 944.4307; 955.0618; 964.1553; 977.6530; 985.4048; 991.6062; 1003.4526; 1009.3107; 1016.0530; 1024.2908; 1055.0291; 1057.0124; 1068.7362; 1069.3913; 1072.5522; 1077.4055; 1097.2120; 1110.8776; 1192.2595; 1195.1466; 1199.5930; 1203.2288; 1218.4707; 1242.0303; 1253.7149; 1255.9882; 1262.2386; 1268.7006; 1289.5846; 1306.5406; 1310.0268; 1320.8232; 1326.9697; 1363.5248; 1414.5672; 1435.3184; 1437.4499; 1453.8569; 1462.8275; 1465.0231; 1503.2387; 1504.4359; 1507.3978; 1513.7086; 1515.8038; 1516.5502; 1532.8124; 1535.2421; 1549.1083; 1609.3176; 1615.2597; 1618.8852; 1640.7841; 1797.0950; 3048.4565; 3062.6361; 3066.0650; 3073.0040; 3107.5707; 3132.9787; 3135.6376; 3140.7280; 3141.8595; 3144.8678; 3145.2874; 3147.5664; 3155.0543; 3169.7852; 3178.6725; 3188.2957; 3204.2252.

Cartesian Forces: Max 0.000001193 RMS 0.000000436

Item	Value	Threshold	Converged?
Maximum Force	0.000001	0.000450	YES
RMS Force	0.000000	0.000300	YES
Maximum Displacement	0.000091	0.001800	YES
RMS Displacement	0.000016	0.001200	YES

FILE: Yb_Sq2_PhYbOTf2_TS_Neutral_Add12_B3LYP_M062x_Conf6.out

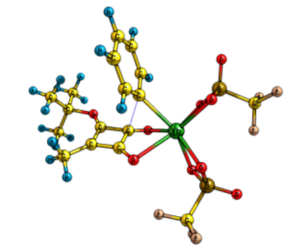

Conformer 9

E(RB3LYP/6-31G(d)/MWB59) = -2769.88703084
Convg = 0.8465D-08 -V/T = 2.0149

Temperature 195.150 Kelvin. Pressure 1.00000 Atm.
elec. and zero-point Energies= -2769.543950
elec. and thermal Energies= -2769.525335
elec. and thermal Enthalpies= -2769.524717
elec. and thermal Free Energies= -2769.584799

RB3LYP/6-31G(d)/MWB59/THF(IEFPCM) = -2769.91266768

RM06-2x/6-311+G(d,p)/MWB59/THF(IEFPCM) = -2769.72023743

Geometry at RB3LYP/6-31G(d)/MWB59
Charge = 0 Multiplicity = 1

```
Yb 1.179750941 2.0556314138 -0.4611363862
O 0.5174183029 4.3132414076 -0.422638419
S 0.7239941712 4.5629341563 1.0608597781
O 1.5445405771 5.7012926379 1.4360540154
O 1.0963443563 3.2062840622 1.6271706275
C -0.9778514901 4.9202011329 1.7432608498
F -1.492417275 5.9667883543 1.0988193761
F -1.7815738639 3.8664464214 1.5898626089
F -0.8678024463 5.201702053 3.0416004268
O 2.6737678608 2.8480575149 -2.078633767
O 4.8307651396 1.5233849101 -2.3508926621
O 3.4454627526 1.5533474324 -0.2177494555
S 3.9343582424 2.2552120479 -1.4751391414
C 4.8750997837 3.7350347928 -0.8353290913
F 5.2309905039 4.5040080221 -1.8616506733
F 4.0967339624 4.4295929494 -0.0029788941
F 5.9599415522 3.3161059776 -0.187071146
C -1.0973303727 -1.3597620755 0.1735477337
C -2.1885296137 -0.5293943051 -0.1320157941
C -1.3223012941 0.6440432077 -0.1354791149
C -0.1502246571 -0.1909753479 0.2749755902
O 0.8876167381 0.0986989943 0.901215239
O -1.3594461754 1.8528405599 -0.3871386961
O -0.7558444482 -2.6006611946 0.3475155694
C -1.6296446262 -3.7988626766 0.1808841597
C -3.6372230196 -0.6196678982 -0.4677970314
H -4.1630772232 -1.3993342977 0.088828689
H -3.7743828189 -0.8186705548 -1.5396224333
H -4.1193095177 0.339657859 -0.2546320423
C -2.6676474209 -3.8038477826 1.3028639845
H -3.3518012834 -2.9538696076 1.2371480443
H -3.2652794628 -4.7195300875 1.2435535682
H -2.1764466032 -3.7781976228 2.2803935332
C -0.6318547785 -4.9454317468 0.3468076067
H -1.1541970557 -5.9045163529 0.273621053
H 0.1352362934 -4.9053302502 -0.4320864498
H -0.1394101118 -4.8901637579 1.3220781334
C -2.2354772269 -3.7967151206 -1.2224258851
H -1.4525483076 -3.6745710658 -1.9773781382
H -2.7349274744 -4.7551252531 -1.3988551998
H -2.9787353979 -3.007989464 -1.3572953462
C 0.6365170107 0.1567602956 -1.9819564913
C 1.6092696644 -0.855299454 -2.1084008702
C -0.1950958383 0.3899446156 -3.0988221619
H 2.254967083 -1.0901709285 -1.261877915
H -0.9816663943 1.1447445456 -3.0447532299
C 1.7815147325 -1.5673818757 -3.3010181774
C -0.0300971699 -0.3099933952 -4.2961396439
H 2.5509483246 -2.3325421241 -3.3747822842
H -0.6664232793 -0.0912880554 -5.1511072746
C 0.9606364557 -1.2931876072 -4.3964763314
H 1.0892896687 -1.8427943217 -5.3257460182
```

Frequency: -158.1746; 12.8129; 16.4381; 16.6718; 24.3100; 29.5679; 31.5305; 35.2033; 42.6481; 44.7750; 51.3597; 53.8686; 60.8435; 71.2733; 79.3127; 87.6916; 101.2789; 106.8987; 111.9567; 118.0130; 139.4172; 147.9702; 159.5970; 160.4435; 167.5378; 187.2906; 191.4897; 192.7075; 210.6063; 219.3114; 221.2551; 232.3726; 239.5103; 243.4399; 251.3741; 261.7988; 267.0639; 276.5917; 304.3057; 307.3759; 312.4140; 318.0455; 321.2490; 346.3839; 356.5658; 359.9800; 367.8447; 394.6621; 399.4752; 422.0999; 450.5192; 477.4317; 482.6699; 489.5271; 491.2389; 531.0955; 535.2722; 557.8927; 558.3751; 574.0426; 579.2547; 592.2175; 615.5309; 626.8782; 627.1406; 633.4395; 639.1107; 652.8033; 715.3327; 720.1050; 749.0539; 765.4752; 765.8921; 768.0782; 799.8010; 837.9160; 875.4451; 935.4977; 935.5913; 937.8557; 955.0674; 964.4101; 981.4644; 982.8391; 992.5575; 1003.6720; 1007.4264; 1014.0663; 1017.6279; 1052.2631; 1055.2430; 1061.7167; 1068.5192; 1069.4234; 1077.7535; 1096.2203; 1135.4619; 1191.4020; 1195.6236; 1199.7654; 1200.9384; 1203.6744; 1218.3043; 1253.4369; 1255.5642; 1261.2311; 1268.3191; 1288.5644; 1296.3700; 1307.8673; 1320.4202; 1327.9650; 1364.4326; 1407.7145; 1434.0085; 1436.4957; 1446.6037; 1461.0503; 1464.2347; 1500.5870; 1506.2914; 1511.1030; 1515.0839; 1515.5476; 1517.9509; 1528.0195; 1530.8293; 1548.0561; 1608.9810; 1614.8841; 1619.5861; 1680.6734; 1802.8142; 3044.4176; 3067.7051; 3070.3393; 3076.6966; 3112.2076; 3133.0509; 3137.7881; 3138.2425; 3146.1329; 3147.0869; 3148.7445; 3152.1989; 3155.2631; 3165.3613; 3175.6665; 3185.5102; 3201.5616.

Cartesian Forces: Max 0.000001026 RMS 0.000000433

Item	Value	Threshold	Converged?
Maximum Force	0.000000	0.000450	YES
RMS Force	0.000000	0.000300	YES
Maximum Displacement	0.000044	0.001800	YES
RMS Displacement	0.000008	0.001200	YES

FILE: Yb_Sq2_PhYbOTf2_TS_Neutral_Add12_B3LYP_M062x_Conf6-.out

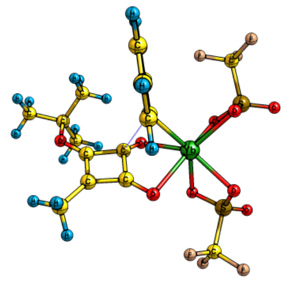

Conformer 10

E(RB3LYP/6-31G(d)/MWB59) = -2769.89015086
Convg = 0.4277D-08 -V/T = 2.0149

Temperature 195.150 Kelvin. Pressure 1.00000 Atm.
elec. and zero-point Energies= -2769.546948
elec. and thermal Energies= -2769.528405
elec. and thermal Enthalpies= -2769.527787
elec. and thermal Free Energies= -2769.587644

RB3LYP/6-31G(d)/MWB59/THF(IEFPCM) = -2769.91415141

RM06-2x/6-311+G(d,p)/MWB59/THF(IEFPCM) = -2769.72158430

Geometry at RB3LYP/6-31G(d)/MWB59
Charge = 0 Multiplicity = 1

```
Yb 1.2983336066 1.9161207894 -0.6238292668
O 0.7278760861 4.1668689879 -0.4179966617
S 0.7575721319 4.257832627 1.102331073
O 1.6047281837 5.2791723104 1.6909486528
O 0.9522013981 2.8264258499 1.5582263956
C -0.9870053787 4.7098579315 1.5942349884
F -1.2463554756 5.9452071698 1.1679310393
F -1.8692748218 3.8646380859 1.06207855
F -1.0796096771 4.6644603305 2.9242466767
O 3.5914919436 2.1902239046 -0.2590291671
O 4.6140079093 4.0996268517 -1.5972914255
O 2.6373189068 2.708875343 -2.3923882379
S 3.9271217968 2.8222338883 -1.602100778
C 5.0995762432 1.6115864561 -2.4061980194
F 6.2754351741 1.6703146015 -1.7846415374
F 4.6177541255 0.3669015735 -2.3158246909
F 5.2494153599 1.9344189988 -3.6887164564
C -0.7323468501 -1.6494886165 0.2121045962
C -1.8605973448 -0.854303481 0.0065242602
C -1.0456744296 0.3368172811 -0.1355488093
C 0.2079074262 -0.459116239 0.11085526
O 1.3085838106 -0.126914039 0.6149628706
O -1.1506587873 1.545848897 -0.3742547017
O -0.6739301104 -2.9336876893 0.4110610613
C 0.5402186945 -3.6411566391 0.9460973188
C -3.3157118102 -1.1382048232 -0.0764614217
H -3.536992202 -2.1544317028 0.2613173278
H -3.6669861882 -1.0351461966 -1.1117614627
H -3.8884132159 -0.4282395245 0.5303528654
C 1.7163221534 -3.4477850381 -0.0063504643
H 2.0918896189 -2.4229962588 0.0228548813
H 2.5322329678 -4.1114638467 0.2998510091
H 1.4353007567 -3.7012750742 -1.0330648012
C 0.0698784676 -5.0935333362 0.9797396965
H 0.8641826617 -5.7273353551 1.3859600598
H -0.8165267385 -5.2010953065 1.6123772677
H -0.1741558898 -5.4467489243 -0.0269928943
C 0.8218911028 -3.0965598309 2.3462659257
H -0.0583718812 -3.1999264418 2.9890067048
H 1.6417052086 -3.6656016344 2.7973793974
H 1.125042062 -2.045814068 2.3127665452
C 0.7029712764 0.0271960801 -2.1870716953
C 1.7584697012 -0.8323575057 -2.5433047619
C -0.2657443656 0.2947831995 -3.1782422367
H 2.5180354021 -1.0885775476 -1.8055923303
H -1.1189926955 0.9342087846 -2.948088565
C 1.8798565996 -1.3618432481 -3.8328565904
C -0.1557319435 -0.2230997839 -4.4710162361
H 2.7146677492 -2.0131996937 -4.0820637184
H -0.9027918944 0.0202738471 -5.2232665867
C 0.9193495546 -1.0557874671 -4.7985532391
H 1.0037581554 -1.4665894942 -5.8014863585
```

Frequency: -191.7911; 7.5832; 18.9147; 25.3800; 28.2877; 29.2189; 34.8580; 37.2821; 41.7545; 43.6308; 51.2238; 52.2346; 66.0024; 68.6116; 81.1297; 85.0434; 91.1646; 97.9930; 112.5144; 118.1232; 134.8925; 152.2371; 159.1378; 161.2914; 168.7199; 180.8417; 189.6468; 204.5845; 213.1967; 218.7648; 219.9422; 226.5909; 242.7879; 251.3912; 273.4052; 273.9225; 278.7575; 286.1976; 304.6833; 307.4450; 318.6820; 321.2708; 331.3214; 344.0097; 353.7752; 361.0769; 367.8757; 399.3272; 406.7922; 423.1934; 452.3431; 464.3490; 488.4280; 489.5475; 490.4112; 529.7070; 535.9136; 557.5533; 558.7427; 570.1604; 579.4039; 591.3263; 612.0609; 624.9159; 626.9897; 633.0981; 640.6931; 651.0729; 710.1795; 721.9294; 740.9866; 749.0976; 765.1639; 765.8654; 795.2672; 853.9907; 877.7093; 937.8620; 941.0185; 944.4551; 952.7644; 963.2816; 977.0197; 985.1534; 992.3895; 1003.8703; 1007.5005; 1016.2872; 1021.2327; 1054.2949; 1056.8993; 1068.1243; 1069.8387; 1071.6296; 1073.3754; 1096.4407; 1104.8226; 1191.6987; 1195.8704; 1202.4091; 1203.7040; 1217.1694; 1243.3126; 1246.7098; 1257.0072; 1260.8770; 1267.0648; 1289.5577; 1306.7319; 1310.9432; 1319.6568; 1332.1148; 1362.2737; 1413.2462; 1435.0784; 1437.2782; 1452.7852; 1462.6223; 1464.9908; 1503.1277; 1503.8139; 1507.1308; 1514.5739; 1515.8800; 1516.4223; 1532.6412; 1535.1880; 1549.9828; 1607.2398; 1615.6029; 1618.8632; 1636.4448; 1798.4119; 3048.4947; 3062.8393; 3065.7933; 3072.4025; 3107.2721; 3133.2289; 3135.0457; 3141.9978; 3142.2993; 3145.2555; 3145.4084; 3147.1880; 3155.3329; 3168.1178; 3178.2256; 3185.9941; 3203.4936.

Cartesian Forces: Max 0.000000665 RMS 0.000000237

Item	Value	Threshold	Converged?
Maximum Force	0.000000	0.000450	YES
RMS Force	0.000000	0.000300	YES
Maximum Displacement	0.000064	0.001800	YES
RMS Displacement	0.000013	0.001200	YES

FILE: Yb_Sq2_PhYbOTf2_TS_Neutral_Add12_B3LYP_M062x_Conf9.out

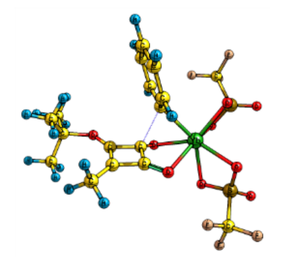

Conformer 11

E(RB3LYP/6-31G(d)/MWB59) = -2769.88376966
Convg = 0.2862D-08 -V/T = 2.0149

Temperature 195.150 Kelvin. Pressure 1.00000 Atm.
elec. and zero-point Energies= -2769.540679
elec. and thermal Energies= -2769.522104
elec. and thermal Enthalpies= -2769.521486
elec. and thermal Free Energies= -2769.581681

RB3LYP/6-31G(d)/MWB59/THF(IEFPCM) = -2769.91065690

RM06-2x/6-311+G(d,p)/MWB59/THF(IEFPCM) = -2769.71788373

Geometry at RB3LYP/6-31G(d)/MWB59
Charge = 0 Multiplicity = 1
Yb 1.2342105814 1.9271390675 -0.5287850602
O 0.8262719711 4.221068421 -0.4619292371
S 0.7791596565 4.3884630716 1.0517758666
O 1.6767401463 5.3669215577 1.6390444384
O 0.8257769761 2.9730642973 1.5871590354
C -0.9437258228 5.0119477908 1.4165508809
F -1.0634644164 6.2475980908 0.9320309311
F -1.8670713424 4.2288630254 0.8598486886
F -1.1191076417 5.0290841606 2.7389278916
O 3.510011805 2.0003095798 -0.0383989066
O 4.8357321318 3.6918694791 -1.4033614003
O 2.7367340065 2.5229986599 -2.2425790934
S 3.9898543465 2.5138689087 -1.388562044
C 5.02574311 1.1180789195 -2.0691468927
F 6.158959189 1.0388759387 -1.3748736293
F 4.3639710762 -0.0391164677 -1.965243698
F 5.2968042582 1.3587690429 -3.350169639
C -1.0739473826 -1.4498726922 0.1573419806
C -2.1626097809 -0.5903919602 -0.054157099
C -1.2703105257 0.5634569166 -0.1167496287
C -0.0902366594 -0.3095724989 0.196051095
O 1.0056882595 -0.0745748847 0.7460626821
O -1.3085163107 1.77125293 -0.3643616888
O -0.750730389 -2.7012686341 0.2935300939
C -1.6690328013 -3.8731304508 0.1974842049
C -3.6348967323 -0.6374802258 -0.2758675451
H -4.1366084109 -1.4123000245 0.3089526036
H -3.860670033 -0.8149174995 -1.3364627924
H -4.0722191423 0.3305953535 -0.0118118706
C -2.6104972715 -3.8541188994 1.401541125
H -3.272342834 -2.9842206935 1.3954924799
H -3.2377654092 -4.7516310532 1.3891605872
H -2.0396288578 -3.8480315713 2.335102259
C -0.6946271462 -5.0486685818 0.2778463152
H -1.2488234089 -5.99203286 0.2473140379
H 0.0055299793 -5.0281165089 -0.5625022435
H -0.1211381039 -5.0113239205 1.2086674926
C -2.3886696053 -3.8477821924 -1.1508608265
H -1.6674568595 -3.7440164883 -1.9675672328
H -2.9277825208 -4.7910147916 -1.2878152969
H -3.1179811237 -3.0380047491 -1.2221629784
C 0.5447529098 0.0857684556 -2.0873272625
C 1.4011495025 -1.013865208 -2.2925526059
C -0.2993309262 0.4467989418 -3.1605504061
H 2.0416932081 -1.3552736654 -1.4790975466
H -1.0085410378 1.2682582269 -3.0440720813
C 1.458305204 -1.688409601 -3.5175692815
C -0.2448945117 -0.2100689822 -4.3919736019
H 2.1364987334 -2.5288075443 -3.6494086014
H -0.8843151944 0.1094106995 -5.2120996518
C 0.6357994897 -1.2828175728 -4.5702497102
H 0.6746538517 -1.8019989323 -5.5247670898

Frequency: -161.4521; 6.6318; 16.0958; 19.9518; 26.2362; 28.7123; 32.4914; 36.4865; 42.2245; 44.2180; 46.9637; 57.2452; 70.3231; 73.3647; 83.7364; 92.9064; 100.0445; 108.1439; 109.5108; 118.9706; 140.6310; 149.4505; 155.6845; 160.0491; 167.6382; 178.9715; 190.3195; 194.9610; 212.0779; 219.0906; 220.3838; 232.0380; 241.1187; 248.3611; 252.7518; 262.2028; 269.6122; 278.6853; 304.9112; 307.3666; 312.9070; 318.3219; 321.5273; 347.3386; 356.9512; 361.2733; 369.0503; 395.5874; 401.6633; 425.7889; 450.1303; 478.0402; 483.0403; 489.0590; 490.5747; 529.8293; 536.0393; 557.7012; 558.8179; 570.5318; 579.9504; 590.3704; 616.0590; 624.9981; 627.7849; 636.5883; 640.9856; 652.6859; 713.0229; 721.6061; 747.6425; 765.2312; 765.8367; 767.8667; 799.4713; 838.1993; 876.7990; 935.5926; 935.9796; 937.7384; 952.9098; 963.4047; 981.6174; 982.5851; 992.6087; 1003.6792; 1006.7350; 1012.2355; 1017.1471; 1052.2473; 1055.0308; 1061.2649; 1068.2682; 1069.3441; 1073.7106; 1095.8923; 1131.5662; 1191.2128; 1196.3255; 1201.3482; 1202.6108; 1203.9029; 1218.2194; 1248.2602; 1257.0693; 1259.9505; 1266.5374; 1288.3267; 1296.2527; 1307.5484; 1319.3953; 1332.4980; 1364.0947; 1406.6915; 1434.0669; 1436.3975; 1446.3677; 1461.0711; 1464.0786; 1500.4148; 1506.0228; 1510.6734; 1515.0077; 1515.3391; 1517.7524; 1528.0364; 1530.6860; 1548.0201; 1610.2092; 1614.9548; 1619.3371; 1673.0146; 1804.9595; 3044.3295; 3067.6478; 3070.4087; 3076.6570; 3112.3573; 3133.7559; 3137.6679; 3138.3887; 3146.6230; 3146.9775; 3148.4076; 3149.2949; 3155.4714; 3165.2906; 3175.3677; 3183.7951; 3201.0309.

Cartesian Forces: Max 0.000002879 RMS 0.000000637

Item	Value	Threshold	Converged?
Maximum Force	0.000002	0.000450	YES
RMS Force	0.000000	0.000300	YES
Maximum Displacement	0.000878	0.001800	YES
RMS Displacement	0.000145	0.001200	YES

FILE: Yb_Sq2_PhYbOTf2_TS_Neutral_Add12_B3LYP_M062x_Conf9-.out

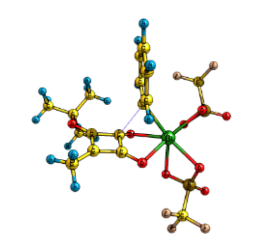

Conformer 12

E(RB3LYP/6-31G(d)/MWB59) = -2769.89014897
Convg = 0.4902D-08 -V/T = 2.0149

Temperature 195.150 Kelvin. Pressure 1.00000 Atm.
elec. and zero-point Energies= -2769.546943
elec. and thermal Energies= -2769.528402
elec. and thermal Enthalpies= -2769.527784
elec. and thermal Free Energies= -2769.587626

RB3LYP/6-31G(d)/MWB59/THF(IEFPCM) = -2769.91399469

RM06-2x/6-311+G(d,p)/MWB59/THF(IEFPCM) = -2769.72141402

Geometry at RB3LYP/6-31G(d)/MWB59
Charge = 0 Multiplicity = 1
Yb 1.3222643301 1.9412223627 -0.6140331285
O 0.7262649497 4.1862266575 -0.4172957487
S 0.7343160284 4.2781403465 1.1032260529
O 1.5628415358 5.3083823804 1.7027507811
O 0.9374478431 2.8489897299 1.5625227066
C -1.0213445994 4.7124223133 1.5711451175
F -1.2878317973 5.9445032822 1.1398139047
F -1.8875524679 3.857418392 1.0283286927
F -1.1312914764 4.6675857944 2.8998539232
O 3.6076485431 2.2374986776 -0.2192985112
O 4.62862422 4.1574755523 -1.5435291331
O 2.6764447872 2.7475097414 -2.3648791953
S 3.9545811121 2.8733272966 -1.557708341
C 5.1494631278 1.6746823927 -2.3466106213
F 6.316467789 1.7449877823 -1.7097493554
F 4.6789371634 0.4252528074 -2.2626947535
F 5.3127946744 1.9993571357 -3.6270139883
C -0.6845136153 -1.6436104536 0.197086529
C -1.8175662414 -0.8597648012 -0.0249836696
C -1.0125345331 0.3392083837 -0.1568433708
C 0.2453013292 -0.4440925166 0.1078394868
O 1.3354874933 -0.1006268179 0.626944945
O -1.1261074316 1.5469229102 -0.3982980991
O -0.6162820655 -2.9270128798 0.3980350497
C 0.5971206048 -3.6221723135 0.9505764613
C -3.2685083261 -1.1580904668 -0.1280350221
H -3.4844728066 -2.1761383075 0.2076948272
H -3.6062400538 -1.0594680871 -1.1682622956
H -3.8566278356 -0.4532517704 0.4699428946
C 1.7844544966 -3.4181386543 0.0144212125
H 2.1496119905 -2.3897210716 0.0478523058
H 2.6024271169 -4.0735855889 0.3326191967
H 1.5202600547 -3.6752081517 -1.0158631064
C 0.1405146572 -5.0790348968 0.9790531977
H 0.9352562976 -5.7047521696 1.396786642
H -0.7534601629 -5.1947257655 1.5994878509
H -0.0861396247 -5.4353976256 -0.0306295405
C 0.8539543329 -3.0737023767 2.3540099016
H -0.0341402243 -3.1850922654 2.9845231216
H 1.6728875756 -3.6343643077 2.8170581013
H 1.1473150945 -2.0200840813 2.32370975
C 0.7677658485 0.0447918753 -2.1832828177
C 1.8367881291 -0.8045080086 -2.5233811104
C -0.1895225431 0.3013121786 -3.1883785107
H 2.588382702 -1.0521050566 -1.7746409164
H -1.0522975655 0.9324243921 -2.9711596642
C 1.9816929165 -1.3344000256 -3.810339282
C -0.0561000727 -0.2171261081 -4.4787164618
H 2.8264683835 -1.9775976297 -4.0468340009
H -0.7949017834 0.017706572 -5.241759778
C 1.031839898 -1.0393379055 -4.7898896558
H 1.1344994611 -1.4505743743 -5.7909408081

Frequency: -191.8806; 7.6286; 18.9422; 25.5752; 28.3542; 29.1816; 35.0675; 37.2256; 41.7313; 43.7742; 51.1507; 52.3770; 65.9743; 68.4034; 81.1204; 85.0532; 91.1866; 97.9530; 112.5471; 118.0877; 134.8390; 152.2424; 159.1777; 161.2846; 168.6947; 180.8212; 189.6060; 204.5781; 213.1576; 219.0448; 219.9644; 226.6050; 242.7681; 251.3924; 273.8805; 274.1233; 278.7999; 286.2068; 304.6553; 307.4252; 318.6703; 321.2750; 331.3798; 344.0221; 353.7956; 361.0685; 367.8944; 399.4042; 406.7764; 423.1321; 452.3721; 464.3440; 488.4308; 489.5303; 490.4019; 529.6925; 535.8956; 557.5578; 558.7372; 570.1654; 579.4072; 591.3302; 612.0696; 624.9226; 627.0038; 633.0973; 640.6978; 651.0639; 710.1568; 721.8528; 740.9858; 749.0849; 765.1623; 765.8568; 795.2494; 853.9605; 877.6415; 937.8542; 940.9326; 944.5327; 952.7642; 963.2807; 977.0151; 985.0795; 992.5287; 1003.8615; 1007.4603; 1016.2759; 1021.2285; 1054.2874; 1056.9012; 1068.0483; 1069.8608; 1071.7045; 1073.4105; 1096.4513; 1104.7920; 1191.6887; 1195.8698; 1202.4087; 1203.7020; 1217.0836; 1243.3295; 1246.7424; 1256.9954; 1260.8743; 1267.0662; 1289.5605; 1306.6944; 1310.9397; 1319.6513; 1332.1091; 1362.1929; 1413.2532; 1435.0979; 1437.3568; 1452.8021; 1462.6312; 1465.0122; 1503.2567; 1503.8151; 1507.1265; 1514.5973; 1515.8922; 1516.4170; 1532.6650; 1535.3219; 1549.9632; 1607.2333; 1615.6347; 1618.8607; 1636.4429; 1798.3958; 3048.4788; 3062.8492; 3065.8021; 3072.4188; 3107.2641; 3133.2345; 3135.0465; 3141.9949; 3142.3179; 3145.2580; 3145.4111; 3147.2056; 3155.2911; 3168.2050; 3178.2237; 3185.9928; 3203.4923.

Cartesian Forces: Max 0.000000621 RMS 0.000000217

Item	Value	Threshold	Converged?
Maximum Force	0.000000	0.000450	YES
RMS Force	0.000000	0.000300	YES
Maximum Displacement	0.000050	0.001800	YES
RMS Displacement	0.000009	0.001200	YES

FILE: Yb_Sq2_PhYbOTf2_TS_Neutral_Add12_B3LYP_M062x_Conf10.out

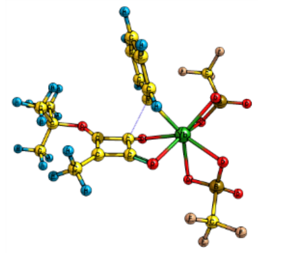

Conformer 13

E(RB3LYP/6-31G(d)/MWB59) = -2769.88377121
Convg = 0.9197D-08 -V/T = 2.0149

Temperature 195.150 Kelvin. Pressure 1.00000 Atm.
elec. and zero-point Energies= -2769.540671
elec. and thermal Energies= -2769.522101
elec. and thermal Enthalpies= -2769.521483
elec. and thermal Free Energies= -2769.581645

RB3LYP/6-31G(d)/MWB59/THF(IEFPCM) = -2769.91055662

RM06-2x/6-311+G(d,p)/MWB59/THF(IEFPCM) = -2769.71773970

Geometry at RB3LYP/6-31G(d)/MWB59
Charge = 0 Multiplicity = 1
Yb 1.2759125884 2.0562088743 -0.6095420553
O 0.863947316 4.3495476929 -0.6642420508
S 0.7830912915 4.5908094004 0.8380819698
O 1.6665296551 5.5987375105 1.3962447733
O 0.8191833188 3.2034748886 1.4431240982
C -0.9480002131 5.2281255078 1.1337207623
F -1.0579566614 6.4383289762 0.5866661044
F -1.8582511817 4.4170249684 0.5959472311
F -1.1525067618 5.30973961 2.4494956537
O 3.5392182065 2.157268384 -0.0681109892
O 4.8984355472 3.779243466 -1.4837449806
O 2.8193614194 2.5688171226 -2.313566786
S 4.0516245682 2.6030434639 -1.4302481719
C 5.1023143608 1.1752248408 -2.014789544
F 6.2180351104 1.1308012709 -1.2897882367
F 4.4366490464 0.0246966715 -1.8693687213
F 5.4050253578 1.3514213043 -3.2991996094
C -1.0441541301 -1.288488094 0.1873229041
C -2.1289187921 -0.4416769607 -0.0862230973
C -1.2362854029 0.70939789 -0.1852640255
C -0.0622724803 -0.1459482679 0.1922797047
O 1.021875831 0.1166448819 0.7531019343
O -1.2702380529 1.9040685936 -0.4902516834
O -0.7229170357 -2.5315839377 0.3887082425
C -1.6383982939 -3.7080845569 0.3296113446
C -3.5965148342 -0.5014380874 -0.3344419092
H -4.1094138117 -1.2474572107 0.2775221191
H -3.8013563323 -0.7306573226 -1.3893373506
H -4.0392462145 0.4777981972 -0.12653371
C -2.6036585506 -3.6339898385 1.5125078871
H -3.2657023814 -2.7663045502 1.4523988067
H -3.2300524959 -4.532025704 1.5297929608
H -2.0515496781 -3.5832167196 2.4559412669
C -0.6650771061 -4.8770590963 0.4843713468
H -1.2179302422 -5.8217007798 0.4867164636
H 0.0519034999 -4.8946019509 -0.3417336366
H -0.110539453 -4.7954393053 1.4238053088
C -2.3311705481 -3.7471817961 -1.0324035822
H -1.5939944388 -3.6807401165 -1.8386746679
H -2.8669011822 -4.69662815 -1.1355396188
H -3.0594356364 -2.9427864413 -1.1560334337
C 0.6195222117 0.1431201916 -2.0939684441
C 1.4803862977 -0.9637656992 -2.2300591712
C -0.2019046142 0.453021517 -3.2002523531
H 2.1037317198 -1.2664243072 -1.3884140346
H -0.9137763742 1.2779897264 -3.1368615279
C 1.5634918177 -1.6941840753 -3.4210643771
C -0.1213463557 -0.2600435635 -4.3985494923
H 2.2445205391 -2.5389519829 -3.4995907962
H -0.743465634 0.0203389306 -5.2458296848
C 0.7632432837 -1.3387308248 -4.5084599832
H 0.8223775341 -1.9014381131 -5.4369129519

Frequency: -161.4101; 6.8066; 15.8653; 19.8260; 26.7536; 28.8106; 32.5408; 36.5377; 42.0897; 44.4513; 47.1096; 57.2099; 70.4737; 73.5136; 83.7527; 93.0628; 100.3506; 108.4267; 109.6617; 118.9603; 140.7814; 149.5486; 155.9441; 160.1144; 167.6643; 179.0148; 190.9133; 194.9658; 212.0859; 219.1528; 220.4749; 231.9620; 241.1120; 248.3760; 252.7381; 261.7950; 269.5622; 278.6593; 304.9638; 307.4693; 312.8795; 318.4879; 321.5045; 347.5092; 357.0746; 361.2781; 369.0591; 395.5863; 401.6193; 425.8227; 450.1634; 478.0773; 482.9103; 489.0721; 490.5522; 529.8447; 536.0762; 557.7121; 558.8021; 570.5263; 579.9878; 590.3890; 615.9776; 624.9791; 627.7667; 636.5540; 640.9688; 652.7141; 713.1102; 721.6321; 747.6614; 765.2360; 765.8304; 767.8604; 799.5045; 838.2040; 876.8138; 935.6541; 935.9773; 937.8431; 952.8740; 963.3713; 981.5918; 982.7303; 992.5912; 1003.7244; 1006.6992; 1012.2421; 1017.1534; 1052.2577; 1055.0172; 1061.6019; 1068.2840; 1069.3976; 1073.6969; 1095.8017; 1131.6106; 1191.1725; 1196.3051; 1201.3181; 1202.6096; 1203.8981; 1218.2338; 1248.1544; 1257.0808; 1259.9350; 1266.6042; 1288.4683; 1296.3572; 1307.5210; 1319.3652; 1332.5114; 1364.0539; 1406.6395; 1434.1044; 1436.3643; 1446.3803; 1460.9978; 1464.0532; 1500.4958; 1506.0683; 1510.7539; 1515.0198; 1515.3256; 1517.7973; 1528.0531; 1530.6329; 1548.0570; 1610.2096; 1614.8919; 1619.3622; 1672.9462; 1805.0861; 3044.3546; 3067.6553; 3070.4161; 3076.6617; 3112.3308; 3133.5358; 3137.6425; 3138.3981; 3146.5788; 3147.0003; 3148.4019; 3149.5522; 3155.4860; 3165.3270; 3175.3725; 3183.8306; 3201.0014.

Cartesian Forces: Max 0.000000334 RMS 0.000000109

Item	Value	Threshold	Converged?
Maximum Force	0.000000	0.000450	YES
RMS Force	0.000000	0.000300	YES
Maximum Displacement	0.000126	0.001800	YES
RMS Displacement	0.000025	0.001200	YES

FILE: Yb_Sq2_PhYbOTf2_TS_Neutral_Add12_B3LYP_M062x_Conf10-.out

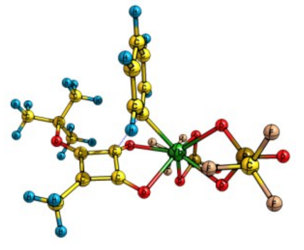

Conformer 14

E(RB3LYP/6-31G(d)/MWB59) = -2769.89365193
Convg = 0.7127D-09 -V/T = 2.0149

Temperature 195.150 Kelvin. Pressure 1.00000 Atm.
elec. and zero-point Energies= -2769.550858
elec. and thermal Energies= -2769.532104
elec. and thermal Enthalpies= -2769.531486
elec. and thermal Free Energies= -2769.592176

RB3LYP/6-31G(d)/MWB59/THF(IEFPCM) = -2769.91486316

RM06-2x/6-311+G(d,p)/MWB59/THF(IEFPCM) = -2769.72068850

Geometry at RB3LYP/6-31G(d)/MWB59
Charge = 0 Multiplicity = 1
Yb 2.438033847 0.6503141485 -1.3253939014
O 2.471157553 1.7413596779 -3.4038545682
S 2.7589213302 3.1363604775 -2.8818467058
O 3.7853210144 3.9180739275 -3.5432245316
O 2.8554568454 2.9524112258 -1.377915399
C 1.1465111508 4.0444386107 -3.1144353804
F 0.8546486877 4.1127328873 -4.4110762161
F 0.1687850807 3.3845316683 -2.4757362328
F 1.2487701398 5.271040833 -2.6091529561
O 4.1388172368 0.5631180892 0.373434083
O 6.4752804695 0.7823098007 -0.6005253382
O 4.5155148838 -0.2273832881 -1.8615878047
S 5.2264774099 0.0448394077 -0.5389412992
C 5.6730212664 -1.6495523958 0.1104331063
F 6.0854191624 -1.5299234906 1.37262405
F 4.6235974825 -2.471502749 0.064539325
F 6.6553547238 -2.1506261574 -0.637688974
C -0.4964456523 -1.7005770117 0.6195811231
C -0.9195726754 -0.4241141796 1.0067967966
C 0.2690990125 0.1823427793 0.45486515
C 0.804433041 -1.1598734582 0.0361364108
O 1.9721318388 -1.548412745 -0.1299856299
O 0.7985752195 1.2966160506 0.3061103262
O -1.1018613334 -2.8351453103 0.795018122
C -0.4634149541 -4.1722959975 0.5241844213
C -2.1299820669 0.0884033123 1.698496644
H -2.7342529211 -0.7337453626 2.0915737261
H -2.7500581954 0.6725924721 1.0062238489
H -1.8554056529 0.7543594522 2.5241516022
C -0.1367004764 -4.2792026059 -0.9617908968
H 0.660413643 -3.5924030328 -1.2519899826
H 0.2023806387 -5.2980554453 -1.1785342829
H -1.0205499507 -4.0748481828 -1.5737252991
C -1.5713239359 -5.1394104393 0.9348160263
H -1.2290167195 -6.169312931 0.7941427743
H -1.8369667726 -5.0040374675 1.987799133
H -2.4672495856 -4.98651024 0.3252429697
C 0.7707001646 -4.305040217 1.4157355328
H 0.5088684248 -4.1510814379 2.4677594634
H 1.1788965284 -5.3161706535 1.3130958461
H 1.5539814873 -3.5979007917 1.1285677339
C 0.4567173604 -0.5057251541 -2.2894313976
C 0.9918904763 -1.5443558722 -3.0748738167
C -0.7333628237 0.0924942598 -2.7485119757
H 1.8863800947 -2.0760098088 -2.7367960188
H -1.1963944502 0.8956850113 -2.174040326
C 0.4153127024 -1.9304484679 -4.2914712587
C -1.3361385055 -0.2980496358 -3.9460121722
H 0.8671005505 -2.719256146 -4.8886114523
H -2.2512612147 0.1850556516 -4.2816741088
C -0.7563070645 -1.3075493569 -4.7231197097
H -1.2215939593 -1.6091561053 -5.6581512437

Frequency: -179.6290; 9.3282; 19.6897; 22.1296; 24.7561; 28.1046; 29.1011; 34.5152; 39.9151; 43.0112; 44.6746; 52.3803; 53.3433; 61.1118; 71.9048; 81.4153; 83.6276; 97.2444; 104.0243; 124.1601; 127.6833; 145.6632; 159.2838; 161.8960; 171.9540; 175.1595; 188.1390; 206.5603; 212.3648; 214.3047; 217.0108; 225.2766; 233.8983; 246.5114; 261.0682; 270.3989; 276.8442; 282.7225; 304.0186; 306.1961; 318.1818; 320.7306; 334.9034; 340.6486; 355.8992; 361.8839; 366.7079; 396.9830; 397.9156; 405.5237; 452.8202; 465.4563; 488.7310; 490.1978; 491.0149; 530.2486; 535.7919; 556.8545; 558.7603; 571.7960; 583.0428; 592.4397; 617.0290; 622.5482; 626.4713; 631.2753; 637.9504; 646.5775; 712.6680; 719.4707; 741.8714; 748.1831; 764.0013; 765.3060; 795.2213; 851.4925; 871.4796; 930.1434; 937.6842; 943.2360; 953.1432; 964.0431; 975.1775; 978.3305; 988.9572; 1002.7152; 1006.3609; 1017.2319; 1024.4644; 1058.1757; 1059.1240; 1067.2282; 1069.2764; 1071.7979; 1077.4451; 1096.5267; 1105.7227; 1191.5583; 1194.4769; 1200.7316; 1203.6022; 1216.0525; 1240.7348; 1246.9065; 1254.5683; 1260.4698; 1269.2320; 1290.5327; 1305.6571; 1310.8450; 1321.5704; 1335.5868; 1361.9157; 1416.8223; 1435.6090; 1437.1424; 1456.1587; 1463.1159; 1464.4501; 1503.2997; 1503.9766; 1507.0332; 1511.4691; 1516.6545; 1517.3508; 1533.0669; 1534.7058; 1549.0403; 1604.0974; 1615.5448; 1619.7221; 1678.4378; 1791.3109; 3050.8844; 3062.9765; 3066.3577; 3073.4257; 3104.7639; 3108.2231; 3131.8908; 3135.4566; 3141.4051; 3142.2416; 3145.2577; 3146.0161; 3151.6521; 3172.6811; 3177.1923; 3185.6291; 3203.2917.

Cartesian Forces: Max 0.000000601 RMS 0.000000223

Item	Value	Threshold	Converged?
Maximum Force	0.000000	0.000450	YES
RMS Force	0.000000	0.000300	YES
Maximum Displacement	0.000073	0.001800	YES
RMS Displacement	0.000014	0.001200	YES

FILE: Yb_Sq2_PhYbOTf2_TS_Neutral_Add12_B3LYP_M062x_Conf11.out

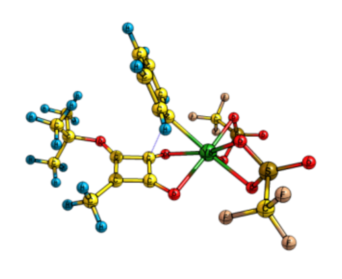

Conformer 15

E(RB3LYP/6-31G(d)/MWB59) = -2769.88612354
Convg = 0.1260D-08 -V/T = 2.0149

Temperature 195.150 Kelvin. Pressure 1.00000 Atm.
elec. and zero-point Energies= -2769.543161
elec. and thermal Energies= -2769.524469
elec. and thermal Enthalpies= -2769.523851
elec. and thermal Free Energies= -2769.584063

RB3LYP/6-31G(d)/MWB59/THF(IEFPCM) = -2769.91199321

RM06-2x/6-311+G(d,p)/MWB59/THF(IEFPCM) = -2769.71909074

Geometry at RB3LYP/6-31G(d)/MWB59
Charge = 0 Multiplicity = 1

```
Yb 2.4631628406 0.7497278952 -1.0844997591
O 2.061448627 2.2622959816 -2.8518114118
S 2.8560548867 3.427914345 -2.293136376
O 3.8502128584 4.0473214198 -3.149909096
O 3.3051900909 2.944240472 -0.9258772067
C 1.5804794575 4.7456889485 -1.9468191618
F 1.0868180213 5.1856171203 -3.1045137662
F 0.5867091267 4.2490608354 -1.2056256001
F 2.1581104647 5.7523951988 -1.2932221637
O 4.4897862973 0.156063233 0.0357241959
O 6.5469180993 0.1006240917 -1.4607058054
O 4.1702798719 -0.2193144662 -2.3097477118
S 5.1988804591 -0.3636353801 -1.1936802409
C 5.3130970959 -2.208809043 -0.9378512123
F 6.0719774074 -2.4589559259 0.127325763
F 4.0919836356 -2.7194438334 -0.7401092852
F 5.8536236613 -2.7702347917 -2.0178998802
C -0.7717076921 -1.2631847102 0.5714007537
C -1.2876028161 0.0380085653 0.7325568087
C 0.0057043172 0.6134125472 0.3979612037
C 0.6172918575 -0.7436096171 0.2975188363
O 1.8013366874 -1.0899427539 0.433882131
O 0.5551633018 1.7079594114 0.2130641412
O -1.1070463823 -2.5149632035 0.5832032097
C -2.4912027742 -3.0748780581 0.667198148
C -2.5779855706 0.7269007627 1.0131462996
H -3.2214787721 0.1747475665 1.7022846342
H -3.1389505211 0.8903421033 0.0827611408
H -2.3772605322 1.7129518548 1.4440436756
C -3.0076372001 -2.8769168365 2.0915597369
H -3.1247646064 -1.8199532274 2.3451272347
H -3.9871463759 -3.3556324381 2.1944085795
H -2.3256521063 -3.3337230676 2.8152252337
C -2.2503607963 -4.5537484682 0.363677614
H -3.1963016324 -5.1016304385 0.4187267521
H -1.8323157821 -4.6767849896 -0.6396650162
H -1.5536270956 -4.9880813557 1.0866905126
C -3.3677924974 -2.429402387 -0.4056413888
H -2.8814222539 -2.4836578194 -1.384695507
H -4.3172788837 -2.9718939106 -0.4632224505
H -3.5992545392 -1.3849605797 -0.1858248272
C 0.6121512245 -0.5374518328 -2.1342812011
C 0.8830770827 -1.8824794868 -2.4587713543
C -0.413031258 0.0991985266 -2.868389807
H 1.6401238616 -2.4303750118 -1.8969689427
H -0.6645755113 1.1389232918 -2.6590146398
C 0.2090417214 -2.5482765362 -3.4901611278
C -1.1022557829 -0.555426506 -3.8902375977
H 0.4560590479 -3.5804531955 -3.7289299033
H -1.8725147091 -0.0327343636 -4.4534985865
C -0.7898830146 -1.884514978 -4.203549329
H -1.3230316746 -2.3955005523 -5.0017699168
```

Frequency: -152.4160; 11.2079; 17.4343; 21.0967; 23.2166; 29.2739; 31.5505; 37.0543; 40.2763; 45.5219; 48.0919; 57.4864; 63.9977; 69.3841; 74.5963; 79.0692; 98.1583; 107.9991; 115.4102; 124.0506; 132.1718; 144.1490; 156.5777; 159.9269; 172.0265; 177.9843; 189.5973; 195.1886; 204.3002; 214.0508; 220.9253; 230.9815; 233.7280; 238.0540; 249.6429; 259.6648; 266.3508; 274.9191; 303.5703; 305.7367; 312.3801; 318.7896; 319.9950; 341.8948; 355.6396; 362.0100; 366.9750; 392.7628; 398.7255; 413.9975; 450.4624; 475.2469; 481.9065; 489.7064; 490.6930; 530.4713; 535.6736; 557.6431; 558.1955; 573.2489; 582.3572; 593.7095; 615.6053; 622.8342; 627.5460; 635.5627; 639.6677; 647.2469; 715.6054; 717.8994; 749.7573; 764.2042; 765.3561; 768.2364; 798.7172; 834.5304; 871.6550; 933.0240; 935.7270; 938.8130; 954.5259; 965.0474; 979.1559; 983.3041; 992.6490; 999.9912; 1007.9670; 1015.4819; 1018.1925; 1052.0454; 1060.9361; 1061.6170; 1068.6921; 1069.8439; 1078.1182; 1096.3941; 1142.0536; 1190.5662; 1195.5520; 1199.6687; 1203.3299; 1205.1326; 1219.6067; 1244.2544; 1250.4394; 1262.0054; 1265.3431; 1288.9594; 1296.3354; 1308.8499; 1322.6956; 1333.5994; 1365.9185; 1411.0495; 1434.6604; 1436.6601; 1448.3059; 1461.5506; 1463.4995; 1500.1943; 1505.8139; 1510.5643; 1514.7587; 1516.6904; 1517.6158; 1527.7187; 1531.2587; 1547.7734; 1603.6932; 1613.5905; 1620.4004; 1698.5187; 1800.8028; 3045.7772; 3067.4866; 3070.0584; 3076.3766; 3112.7257; 3138.0047; 3138.3000; 3146.5331; 3147.2533; 3149.0482; 3150.0682; 3151.4204; 3154.8788; 3164.9734; 3174.6446; 3183.5555; 3199.5511.

Cartesian Forces: Max 0.000000359 RMS 0.000000151

Item	Value	Threshold	Converged?
Maximum Force	0.000000	0.000450	YES
RMS Force	0.000000	0.000300	YES
Maximum Displacement	0.000104	0.001800	YES
RMS Displacement	0.000020	0.001200	YES

FILE: Yb_Sq2_PhYbOTf2_TS_Neutral_Add12_B3LYP_M062x_Conf11-.out

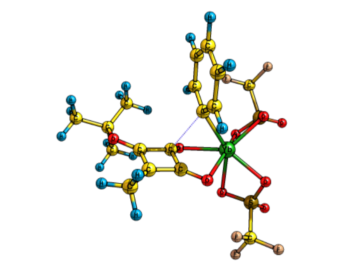

Conformer 16

E(RB3LYP/6-31G(d)/MWB59) = -2769.89014679
Convg = 0.9497D-08 -V/T = 2.0149

Temperature 195.150 Kelvin. Pressure 1.00000 Atm.
elec. and zero-point Energies= -2769.546938
elec. and thermal Energies= -2769.528399
elec. and thermal Enthalpies= -2769.527781
elec. and thermal Free Energies= -2769.587610

RB3LYP/6-31G(d)/MWB59/THF(IEFPCM) = -2769.91388641

RM06-2x/6-311+G(d,p)/MWB59/THF(IEFPCM) = -2769.72129459

Geometry at RB3LYP/6-31G(d)/MWB59
Charge = 0 Multiplicity = 1

```
Yb 1.3268255401 2.0194397419 -0.5044822691
O 0.7034733614 4.2543661406 -0.279117908
S 0.6752654503 4.317049488 1.2426311536
O 1.4788449407 5.3437921918 1.8808666246
O 0.8826057104 2.8814090622 1.6792855888
C -1.0952123066 4.7246372363 1.6776593427
F -1.3652238087 5.9612055918 1.2616307652
F -1.9395930331 3.8702101191 1.1005954272
F -1.2347512167 4.6554525868 3.0025401352
O 3.5993950734 2.3303710988 -0.05144104
O 4.6313385622 4.2847586098 -1.3155093244
O 2.7123740694 2.8723669214 -2.207896598
S 3.970468216 2.9948306328 -1.36931413
C 5.1946160312 1.8225580802 -2.1529119434
F 6.3465305183 1.8925893933 -1.4891237714
F 4.7349384632 0.567337879 -2.1018860188
F 5.3828449205 2.172033763 -3.4233260379
C -0.6640426454 -1.5982314578 0.192340689
C -1.798922028 -0.8208173509 -0.0426117908
C -1.0025162634 0.3876964345 -0.1331555878
C 0.2561361774 -0.3888239396 0.1472840356
O 1.330362142 -0.0451888644 0.6985433728
O -1.1219423729 1.5985773663 -0.3551981937
O -0.5883882584 -2.8844381707 0.3715502499
C 0.6180438896 -3.5786670028 0.9402722605
C -3.2440682966 -1.1303927328 -0.1859994401
H -3.4583425475 -2.1562635953 0.1261717074
H -3.5576523315 -1.0160348353 -1.2321609521
H -3.8530364006 -0.4418461239 0.4100859689
C 1.825049329 -3.3475801549 0.0360957592
H 2.1798744759 -2.3167636183 0.0962278418
H 2.6413408757 -4.0013872662 0.3618932163
H 1.5874266609 -3.5885641377 -1.0044916487
C 0.1742726173 -5.0397422831 0.9323190802
H 0.9647744603 -5.6658462307 1.357451889
H -0.7327857261 -5.1742726021 1.5295318348
H -0.0255839271 -5.3800061625 -0.0885154204
C 0.8369782497 -3.0531171938 2.3588216906
H -0.0645284268 -3.1835316032 2.9662352508
H 1.6499760756 -3.6147930719 2.8310125421
H 1.1213164411 -1.9966068665 2.3540457933
C 0.8282810743 0.1472308443 -2.1211224822
C 1.9127472605 -0.6863891798 -2.4509710516
C -0.1068683892 0.4143447439 -3.1441528287
H 2.6483335058 -0.9415794781 -1.6889973297
H -0.9802321836 1.033902229 -2.9361836105
C 2.0932214543 -1.1908704 -3.7436344573
C 0.0621127271 -0.0787028459 -4.4402599415
H 2.9491512075 -1.8223274043 -3.9715107801
H -0.6602433454 0.1640720685 -5.216450254
C 1.1645353421 -0.8856533696 -4.7402189775
H 1.2948634106 -1.277210173 -5.7459304479
```

Frequency: -192.0560; 7.5766; 19.1020; 25.8930; 28.4211; 29.0902; 35.3142; 37.2340; 41.7888; 43.8556; 51.0595; 52.5733; 65.8039; 68.0567; 81.1203; 85.1436; 91.0815; 97.9083; 112.4759; 118.0343; 134.7699; 152.2561; 159.2839; 161.3545; 168.6916; 180.8561; 189.5878; 204.5350; 213.1176; 219.1742; 220.0308; 226.6409; 242.8095; 251.4464; 273.9778; 274.2390; 278.8623; 286.2144; 304.6341; 307.4035; 318.6698; 321.3314; 331.3848; 344.0776; 353.9584; 361.0734; 367.9466; 399.7278; 406.7583; 423.1191; 452.4731; 464.3416; 488.4466; 489.6123; 490.4181; 529.6840; 535.8791; 557.5583; 558.7396; 570.1829; 579.4227; 591.3038; 612.0503; 624.9315; 627.0295; 633.1090; 640.7024; 651.0736; 710.1265; 721.7382; 740.9722; 748.9938; 765.1638; 765.8548; 795.2584; 853.9724; 877.4803; 937.8617; 940.7501; 944.6358; 952.7614; 963.2770; 976.9819; 984.8824; 992.6192; 1003.8401; 1007.3669; 1016.2645; 1021.2286; 1054.2942; 1056.9372; 1067.9954; 1069.9555; 1071.7682; 1073.4431; 1096.4188; 1104.7627; 1191.6584; 1195.8457; 1202.4035; 1203.6940; 1216.9811; 1243.3417; 1246.7507; 1256.9645; 1260.8726; 1267.0888; 1289.5131; 1306.6682; 1310.9361; 1319.6627; 1332.1264; 1362.0702; 1413.2575; 1435.1880; 1437.4285; 1452.8414; 1462.6798; 1465.0283; 1503.3519; 1503.8029; 1507.1327; 1514.5499; 1515.8545; 1516.4252; 1532.7380; 1535.3808; 1549.9339; 1607.2303; 1615.6255; 1618.8572; 1636.4231; 1798.3535; 3048.4681; 3062.8749; 3065.8202; 3072.4528; 3107.2580; 3133.2311; 3135.0361; 3141.9946; 3142.3701; 3145.2408; 3145.4073; 3147.2900; 3155.1438; 3168.4704; 3178.1856; 3185.9723; 3203.4447.

Cartesian Forces: Max 0.000000694 RMS 0.000000228

Item	Value	Threshold	Converged?
Maximum Force	0.000001	0.000450	YES
RMS Force	0.000000	0.000300	YES
Maximum Displacement	0.000054	0.001800	YES
RMS Displacement	0.000010	0.001200	YES

FILE: Yb_Sq2_PhYbOTf2_TS_Neutral_Add12_B3LYP_M062x_Conf12.out

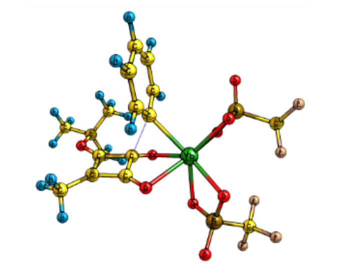

Conformer 17

E(RB3LYP/6-31G(d)/MWB59) = -2769.89480153
Convg = 0.7888D-08 -V/T = 2.0149

Temperature 195.150 Kelvin. Pressure 1.00000 Atm.
elec. and zero-point Energies= -2769.551598
elec. and thermal Energies= -2769.533072
elec. and thermal Enthalpies= -2769.532454
elec. and thermal Free Energies= -2769.591903

RB3LYP/6-31G(d)/MWB59/THF(IEFPCM) = -2769.91683855

RM06-2x/6-311+G(d,p)/MWB59/THF(IEFPCM) = -2769.72493124

Geometry at RB3LYP/6-31G(d)/MWB59
Charge = 0 Multiplicity = 1

```
Yb 1.2382606515 1.9776579745 -0.6268833284
O 0.7140784155 2.8074629891 1.5480362607
S 0.2455309462 4.174286914 1.0923295306
O -1.0595860523 4.6322248914 1.5350397249
O 0.5269706266 4.193732954 -0.4025272477
C 1.4927185454 5.3568711857 1.8215697864
F 1.2845665826 5.4487169904 3.1341830018
F 2.7312724505 4.9067629971 1.6025770842
F 1.3489212992 6.5546270708 1.257835551
O 2.5271872934 2.835475258 -2.4146511059
O 4.8335798997 1.7758301736 -2.6051216227
O 3.5611472568 1.9986714508 -0.4205049604
S 3.8827529853 2.5335898099 -1.8109944852
C 4.6601721789 4.2060587136 -1.5171464244
F 4.9936216694 4.7347751592 -2.6924851721
F 3.7974428683 5.0108357869 -0.8964237381
F 5.7487197133 4.0532659664 -0.7650938414
C -0.777149412 -1.5946453636 0.1910830932
C -1.9052107573 -0.8181862996 -0.0796370919
C -1.1015669053 0.3804401277 -0.2100628664
C 0.1509313989 -0.3932604508 0.1190894354
O 1.2192106082 -0.0383966214 0.6709992198
O -1.2129795015 1.579519628 -0.4923407197
O -0.7119571346 -2.8745652622 0.4118991966
C 0.5013234874 -3.5638709984 0.9719515324
C -3.3521174287 -1.1217726576 -0.2188527418
H -3.5729665469 -2.1403917734 0.1117912897
H -3.6648530388 -1.0232949486 -1.2667278685
H -3.9563538885 -0.4184655728 0.3648165055
C 1.6862170328 -3.3705654795 0.0304672418
H 2.0467312324 -2.3403296045 0.0435193283
H 2.5080069872 -4.0162100155 0.3588069551
H 1.4225245965 -3.6464359901 -0.9950381319
C 0.0438994437 -5.0201040616 1.0174815789
H 0.8395383928 -5.6416386702 1.4397626852
H -0.8484676966 -5.1286898549 1.6415834825
H -0.1855166216 -5.387292213 0.0123198966
C 0.7602825652 -3.0005261563 2.3690770657
H -0.1275062393 -3.1040396406 3.0014357365
H 1.5786409262 -3.5579919087 2.8370735354
H 1.0550336163 -1.9478402515 2.3272884417
C 0.7947823442 0.0484547199 -2.1570473524
C 1.961980844 -0.696466482 -2.4123578541
C -0.1374968884 0.1603577652 -3.209402261
H 2.7048589553 -0.8287829898 -1.6243092207
H -1.0656585288 0.7129090175 -3.0579741756
C 2.2191307885 -1.261404552 -3.6665553016
C 0.1029876601 -0.4030787163 -4.4645481712
H 3.1401724417 -1.8126883753 -3.8404210286
H -0.6236088112 -0.2855810628 -5.2655602551
C 1.2847293116 -1.1159739269 -4.6933520466
H 1.4736362941 -1.5573133101 -5.6687030462
```

Frequency: -191.2357; 12.8312; 18.6165; 26.7946; 27.8739; 29.1730; 34.5822; 35.6724; 38.6236; 48.9678; 54.6494; 61.6082; 63.2590; 70.2100; 75.8483; 83.8478; 91.2321; 96.1020; 105.0965; 120.7746; 135.6460; 153.0528; 158.6440; 162.8300; 172.4154; 183.0488; 191.8678; 203.2071; 215.5772; 216.9192; 221.0019; 223.4471; 243.9035; 246.6685; 269.3000; 274.2019; 280.2290; 286.3103; 304.2591; 308.6314; 319.5150; 322.2859; 332.1653; 343.1741; 352.8836; 361.1988; 368.1291; 397.7003; 403.0507; 418.5774; 452.5064; 465.0060; 489.0432; 490.0334; 490.5082; 530.6551; 535.6191; 558.3155; 558.7576; 573.8769; 578.2425; 591.6780; 615.6459; 622.4408; 626.2682; 633.5302; 639.3537; 650.0468; 711.8753; 720.7147; 741.4563; 749.9175; 765.1372; 766.8115; 795.2484; 854.0403; 876.2762; 936.9720; 939.6943; 944.0089; 951.2616; 965.1190; 977.5849; 984.2807; 991.1861; 1003.6211; 1008.3804; 1016.4460; 1020.4743; 1057.2989; 1058.2132; 1067.6349; 1069.5933; 1071.4630; 1074.7050; 1096.9329; 1102.0098; 1191.9491; 1195.9357; 1200.4754; 1203.8281; 1216.8079; 1244.9267; 1245.5501; 1260.0736; 1267.4955; 1267.7213; 1289.5854; 1306.2649; 1311.1804; 1318.1746; 1326.9875; 1362.1431; 1413.9888; 1435.0713; 1437.0302; 1453.4955; 1462.6060; 1465.2805; 1503.2826; 1503.5744; 1507.2205; 1513.7079; 1515.4581; 1516.6707; 1532.8766; 1535.0472; 1549.1233; 1607.2878; 1615.3252; 1619.1375; 1639.9003; 1797.7734; 3049.3360; 3062.7203; 3065.9774; 3072.9757; 3107.6819; 3132.6976; 3135.1626; 3139.2196; 3141.8291; 3142.6501; 3145.0658; 3145.6640; 3148.4188; 3170.9901; 3178.3333; 3187.9661; 3204.0062.

Cartesian Forces: Max 0.000000731 RMS 0.000000176

Item	Value	Threshold	Converged?
Maximum Force	0.000001	0.000450	YES
RMS Force	0.000000	0.000300	YES
Maximum Displacement	0.000015	0.001800	YES
RMS Displacement	0.000003	0.001200	YES

FILE: Yb_Sq2_PhYbOTf2_TS_Neutral_Add12_B3LYP_M062x_Confx9.out

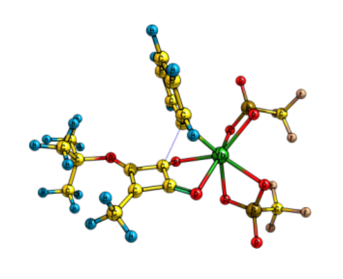

Conformer 18

E(RB3LYP/6-31G(d)/MWB59) = -2769.88786230
Convg = 0.9018D-08 -V/T = 2.0149

Temperature 195.150 Kelvin. Pressure 1.00000 Atm.
elec. and zero-point Energies= -2769.544697
elec. and thermal Energies= -2769.526170
elec. and thermal Enthalpies= -2769.525552
elec. and thermal Free Energies= -2769.585168

RB3LYP/6-31G(d)/MWB59/THF(IEFPCM) = -2769.91281741

RM06-2x/6-311+G(d,p)/MWB59/THF(IEFPCM) = -2769.72005449

Geometry at RB3LYP/6-31G(d)/MWB59
Charge = 0 Multiplicity = 1

```
Yb 1.2665814692 1.9354392986 -0.4702196477
O 0.6572101296 2.8604361149 1.6478859697
S 0.3587808529 4.2537457995 1.1349136413
O -0.9244623587 4.8462624196 1.4692088467
O 0.751255239 4.2121969609 -0.3344334108
C 1.6577815552 5.3289342179 1.9358879071
F 1.3660717921 5.4698561176 3.2281488789
F 2.8600393471 4.7591633539 1.8153292806
F 1.6706125812 6.5223050013 1.3447403738
O 2.7513199219 2.6301800724 -2.1775433598
O 4.9361015777 1.3250205849 -2.2056913073
O 3.5527863924 1.7158388846 -0.1125848105
S 4.0229255301 2.1916541745 -1.4824785219
C 4.956429862 3.7734819361 -1.1460528807
F 5.4121315458 4.2552523297 -2.3006017395
F 4.1499167634 4.6705336982 -0.5778381999
F 5.9770448609 3.512040819 -0.3314004692
C -1.1145192165 -1.4038895715 0.133788479
C -2.1769589669 -0.534803353 -0.155868476
C -1.2683838754 0.606850618 -0.1885299881
C -0.1180600062 -0.2767617147 0.2105574952
O 0.9470868568 -0.0514353457 0.8188938245
O -1.2822288646 1.8086638245 -0.4650836827
O -0.8181934847 -2.6567537096 0.3127952694
C -1.7518624908 -3.8149602967 0.2083589194
C -3.6360811881 -0.5647275271 -0.4547208275
H -4.1823348199 -1.3096559746 0.1292840506
H -3.8096791486 -0.7760626603 -1.5188470929
H -4.0673722604 0.4196678502 -0.2473027106
C -2.7468846264 -3.7434408152 1.3666616827
H -3.3919948079 -2.8632582616 1.3038053874
H -3.3888142347 -4.6305024867 1.3525046404
H -2.2190554741 -3.7176800783 2.324895804
C -0.803783537 -5.0039170989 0.3679221883
H -1.3728183941 -5.9384444772 0.3376031671
H -0.0645186808 -5.0191498192 -0.4383493997
H -0.2740336892 -4.9490811269 1.3234647679
C -2.4100127675 -3.8194301268 -1.1712635231
H -1.6513428689 -3.7599140412 -1.9578237
H -2.9645917037 -4.7546928772 -1.3011680353
H -3.1161501237 -2.9967217265 -1.3020250026
C 0.6931936656 0.086524115 -2.0314295219
C 1.6597805646 -0.9324571911 -2.1442597719
C -0.1059721071 0.341765067 -3.166525908
H 2.2730668128 -1.1927353322 -1.2801579656
H -0.8874584727 1.1020533505 -3.1221358329
C 1.8645170543 -1.6259292031 -3.3427895886
C 0.0875813577 -0.3429184722 -4.3679494008
H 2.6296636938 -2.3962803679 -3.4057472079
H -0.5247363623 -0.1096045821 -5.2364883746
C 1.0766877678 -1.3293238686 -4.4561290872
H 1.2276137664 -1.8659751153 -5.3896089999
```

Frequency: -158.1767; 12.0721; 17.3195; 17.9164; 26.1300; 29.4238; 31.9713; 37.0681; 42.7695; 50.0385; 58.5267; 61.1252; 63.3176; 71.7683; 84.0336; 89.2824; 100.4331; 106.7429; 111.5518; 115.7238; 144.6878; 149.7000; 156.1258; 161.8379; 169.2267; 185.2513; 190.2786; 192.7360; 214.1985; 216.6414; 223.0227; 231.5121; 243.0447; 245.5308; 250.3592; 261.4517; 272.3391; 279.8107; 304.2678; 308.6313; 312.9734; 319.2129; 322.6309; 347.3063; 356.0291; 361.3314; 369.1683; 394.7412; 399.8879; 422.2564; 450.2437; 477.9378; 482.7458; 489.8182; 490.4973; 530.6670; 535.7810; 558.2986; 558.6601; 574.0094; 578.3431; 591.4573; 616.5797; 622.8070; 626.8936; 637.5484; 640.2109; 652.9366; 714.2921; 720.2522; 748.6178; 765.0857; 766.7295; 767.5381; 799.6459; 838.6569; 875.6849; 935.4884; 935.7739; 937.4868; 951.2868; 965.0810; 981.9398; 982.6164; 992.8353; 1004.3130; 1007.0298; 1012.1126; 1017.6671; 1052.6768; 1058.6741; 1061.6822; 1067.9261; 1068.8154; 1075.0713; 1096.6647; 1127.2874; 1191.4043; 1197.0019; 1200.8708; 1202.1001; 1204.3665; 1217.4834; 1245.1664; 1259.8529; 1266.8944; 1267.2007; 1288.3418; 1296.4327; 1308.6643; 1318.6741; 1327.4355; 1363.6102; 1406.6949; 1434.0164; 1436.2609; 1446.3980; 1460.9621; 1464.5506; 1500.5391; 1506.1338; 1510.9053; 1515.0000; 1515.3444; 1517.8712; 1528.0682; 1530.7023; 1547.9266; 1609.5688; 1614.9095; 1619.9000; 1677.1270; 1804.0699; 3044.5824; 3067.5575; 3070.4326; 3076.8091; 3112.6208; 3136.7311; 3137.6410; 3138.2417; 3141.7009; 3146.1167; 3146.8738; 3148.3736; 3155.2235; 3166.0778; 3175.6472; 3185.4889; 3201.6310.

Cartesian Forces: Max 0.000002580 RMS 0.000000820

Item	Value	Threshold	Converged?
Maximum Force	0.000001	0.000450	YES
RMS Force	0.000000	0.000300	YES
Maximum Displacement	0.000386	0.001800	YES
RMS Displacement	0.000061	0.001200	YES

FILE: Yb_Sq2_PhYbOTf2_TS_Neutral_Add12_B3LYP_M062x_Confx9-.out

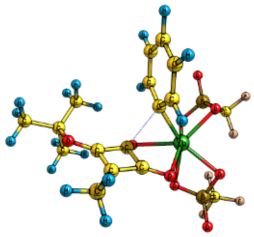

Conformer 19

E(RB3LYP/6-31G(d)/MWB59) = -2769.89480645
Convg = 0.8166D-08 -V/T = 2.0149

Temperature 195.150 Kelvin. Pressure 1.00000 Atm.
elec. and zero-point Energies= -2769.551578
elec. and thermal Energies= -2769.533062
elec. and thermal Enthalpies= -2769.532444
elec. and thermal Free Energies= -2769.591854

RB3LYP/6-31G(d)/MWB59/THF(IEFPCM) = -2769.91684951

RM06-2x/6-311+G(d,p)/MWB59/THF(IEFPCM) = -2769.72492630

Geometry at RB3LYP/6-31G(d)/MWB59
Charge = 0 Multiplicity = 1
Yb 1.1322229206 1.9101145191 -0.6401082488
O 0.6543050643 2.6977764134 1.5608166218
S 0.1481621827 4.0622079291 1.1393244039
O -1.1522742159 4.488856947 1.6249883547
O 0.3873102591 4.1091699572 -0.3622520673
C 1.3932419813 5.2570686211 1.8520063799
F 1.2200454968 5.3254506883 3.1711112535
F 2.633311507 4.8336965398 1.5920838289
F 1.2117706323 6.4601501041 1.310929152
O 2.3571557444 2.8189238607 -2.447519648
O 4.6735004852 1.7994614026 -2.7195953655
O 3.4593023759 1.966219537 -0.4971243629
S 3.7336060669 2.5288396415 -1.8868160168
C 4.4930180531 4.2083930688 -1.5865481615
F 4.784782969 4.7618239199 -2.7615919212
F 3.6358786553 4.9888606771 -0.928178464
F 5.6046840229 4.0605217695 -0.8680745867
C -0.7975299602 -1.7102279419 0.1742971165
C -1.9458803642 -0.9502611345 -0.0546547606
C -1.1672518965 0.2646039516 -0.1867533764
C 0.106975984 -0.4913100895 0.0968439401
O 1.1831228938 -0.1256135263 0.625941726
O -1.307184018 1.4658207505 -0.4463803148
O -0.7038426905 -2.991973295 0.373291129
C 0.5351222589 -3.6671174067 0.8928767783
C -3.3903426565 -1.2778077604 -0.1610506305
H -3.5845767586 -2.3048014098 0.1602735703
H -3.7318258305 -1.1702591944 -1.1990206365
H -3.9915259917 -0.5937566831 0.4480885241
C 1.6929805864 -3.4376966787 -0.0738111859
H 2.0354273408 -2.4014354456 -0.0527642297
H 2.5338493962 -4.0734813903 0.2240543423
H 1.4091102063 -3.702210791 -1.0969170754
C 0.1054016267 -5.1320736069 0.9268285103
H 0.9221886957 -5.7450885115 1.3201932636
H -0.7694180666 -5.2664968141 1.5704390124
H -0.1417004742 -5.4882724262 -0.0780940988
C 0.817975234 -3.1201754994 2.2918890552
H -0.0521048827 -3.249345184 2.9438393598
H 1.6573928934 -3.6694959521 2.7313448864
H 1.0927086398 -2.0617621034 2.2591687449
C 0.6814214062 -0.0030757113 -2.1880755741
C 1.8538569348 -0.7235627597 -2.4858860688
C -0.2800463494 0.1094421 -3.2137835353
H 2.6193557943 -0.8555180564 -1.7197497187
H -1.2130386439 0.643475897 -3.029249271
C 2.0872830999 -1.2640762478 -3.7553978717
C -0.0632592823 -0.4297443779 -4.483807699
H 3.0125519508 -1.7965973816 -3.9621751799
H -0.812565101 -0.3120339808 -5.2635845747
C 1.1238472792 -1.1184746788 -4.7549659832
H 1.2943681905 -1.5409513331 -5.7419869964

Frequency: -190.8194; 12.9360; 18.4926; 26.7329; 27.9118; 29.4196; 34.8369; 35.7199; 38.6426; 49.0219; 54.7464; 61.5855; 64.2853; 70.2602; 76.4238; 84.1238; 91.2139; 96.1936; 105.5567; 120.7989; 136.1432; 153.0698; 158.5550; 162.7830; 172.4774; 183.2120; 191.8617; 203.3413; 215.6052; 217.2070; 221.1185; 223.4516; 243.9847; 246.7255; 269.9846; 274.3206; 280.2459; 286.3546; 304.3122; 308.6119; 319.4377; 322.3482; 332.2983; 343.1191; 353.0030; 361.2135; 368.1652; 397.8344; 403.3668; 418.8954; 452.3457; 465.0909; 489.1343; 490.0490; 490.5194; 530.6494; 535.6549; 558.3087; 558.7590; 573.8667; 578.2313; 591.7435; 615.5361; 622.4609; 626.3034; 633.6575; 639.4050; 650.1700; 711.8767; 720.9537; 741.4527; 749.9773; 765.1489; 766.8242; 795.2826; 854.0682; 876.4220; 936.9746; 939.7957; 944.2264; 951.2511; 965.1312; 977.5839; 984.3841; 991.4593; 1003.6550; 1008.5687; 1016.5406; 1020.5034; 1057.2944; 1058.1496; 1067.9871; 1069.7788; 1071.4081; 1074.7022; 1097.1118; 1102.0884; 1191.9774; 1195.9777; 1200.4907; 1203.8242; 1217.1792; 1244.8993; 1245.5281; 1260.1196; 1267.5056; 1267.7139; 1289.4888; 1306.4001; 1311.4474; 1318.1524; 1326.9664; 1362.5596; 1413.9824; 1435.0701; 1437.1091; 1453.4477; 1462.6952; 1465.3198; 1503.3270; 1503.6085; 1507.2526; 1513.6743; 1515.6434; 1516.6898; 1532.8857; 1535.3324; 1549.1123; 1607.3340; 1615.3855; 1619.1609; 1639.9216; 1797.7649; 3049.3083; 3062.7243; 3065.9392; 3072.9114; 3107.6862; 3132.6834; 3135.1177; 3139.5489; 3141.8336; 3142.6426; 3145.0184; 3145.6490; 3148.3839; 3170.8647; 3178.3023; 3187.9611; 3203.9964.

Cartesian Forces: Max 0.000000749 RMS 0.000000346

Item	Value	Threshold	Converged?
Maximum Force	0.000000	0.000450	YES
RMS Force	0.000000	0.000300	YES
Maximum Displacement	0.000053	0.001800	YES
RMS Displacement	0.000006	0.001200	YES

FILE: Yb_Sq2_PhYbOTf2_TS_Neutral_Add12_B3LYP_M062x_Confx10.out

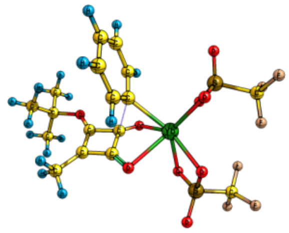

Conformer 20

E(RB3LYP/6-31G(d)/MWB59) = -2769.88784604
Convg = 0.6806D-08 -V/T = 2.0149

Temperature 195.150 Kelvin. Pressure 1.00000 Atm.
elec. and zero-point Energies= -2769.544700
elec. and thermal Energies= -2769.526162
elec. and thermal Enthalpies= -2769.525544
elec. and thermal Free Energies= -2769.585229

RB3LYP/6-31G(d)/MWB59/THF(IEFPCM) = -2769.91305190

RM06-2x/6-311+G(d,p)/MWB59/THF(IEFPCM) = -2769.72032542

Geometry at RB3LYP/6-31G(d)/MWB59
Charge = 0 Multiplicity = 1
Yb 1.2596744856 2.033532322 -0.574894332
O 0.7741223601 2.9864059713 1.5627714297
S 0.4411064518 4.3717350618 1.0493904099
O -0.8229237492 4.9646710018 1.4497023522
O 0.7477714664 4.3113155729 -0.4395575958
C 1.7796966636 5.4621984463 1.7594051163
F 1.5639070932 5.6173951542 3.0648851732
F 2.9758195853 4.8967132913 1.5748859958
F 1.751229804 6.6483796062 1.1544787907
O 2.638248908 2.7080802977 -2.3766620034
O 4.8205677511 1.4061524807 -2.5182643332
O 3.5634986879 1.8217824086 -0.3515833166
S 3.9500264572 2.2805723092 -1.7530824987
C 4.8979201883 3.8681784035 -1.493047629
F 5.2819783289 4.3365181675 -2.678758071
F 4.1245698367 4.770545965 -0.8883135457
F 5.9664370032 3.6189244761 -0.7381929154
C -1.0734804053 -1.3009330607 0.2145382862
C -2.1530705144 -0.437655431 -0.0247211459
C -1.2506204821 0.7047701376 -0.126719811
C -0.0768764136 -0.1713260244 0.216855071
O 1.0216453804 0.0641850483 0.7582306433
O -1.2835337794 1.9025575849 -0.4188203712
O -0.7642372712 -2.5506527913 0.3936951945
C -1.6996664744 -3.7116946189 0.3607724633
C -3.6270674485 -0.4740589559 -0.2373787255
H -4.1364896041 -1.2116448321 0.3877591245
H -3.861941595 -0.7005128033 -1.2865311051
H -4.047890008 0.5124445932 -0.0187820621
C -2.6249221965 -3.6253458589 1.5745684433
H -3.2744163278 -2.7469097375 1.5377998545
H -3.2647901917 -4.513290062 1.6103286443
H -2.0416767065 -3.5857668146 2.4996267816
C -0.741022362 -4.8968001937 0.4809338115
H -1.3087081174 -5.832478205 0.4981200327
H -0.0509386245 -4.9228313528 -0.3675519148
H -0.1555403152 -4.8271977719 1.4023829963
C -2.4379269785 -3.7366857465 -0.9774652107
H -1.7270959945 -3.6860780976 -1.8081123723
H -2.9961291941 -4.6750085281 -1.061796265
H -3.1532348357 -2.9175931042 -1.0774405727
C 0.6006174242 0.1621143554 -2.074144371
C 1.5608451605 -0.8572844723 -2.2295957089
C -0.2635170838 0.4015762793 -3.1642005039
H 2.2238544266 -1.1054997195 -1.399332833
H -1.0424394607 1.1616191074 -3.0844856645
C 1.6969050386 -1.5662090077 -3.4288490567
C -0.1387361715 -0.2985462538 -4.3658371337
H 2.4585072769 -2.336510366 -3.5260890336
H -0.8007961296 -0.0772108624 -5.2003525322
C 0.8453612624 -1.2849911641 -4.4984105678
H 0.9428992776 -1.8336258301 -5.4320290494

Frequency: -158.1743; 11.9868; 17.0228; 17.7394; 25.7862; 29.0816; 31.8196; 36.7953; 42.8127; 49.8501; 58.2231; 61.1796; 62.9087; 71.6999; 83.6999; 89.1430; 100.2055; 105.5193; 111.1233; 115.5532; 144.2972; 149.5128; 156.1532; 161.6427; 168.9542; 185.2105; 191.3445; 192.6371; 214.1160; 216.6492; 222.8041; 232.3195; 242.8240; 245.3087; 250.4369; 261.7916; 272.2555; 279.6578; 304.1492; 308.3597; 313.0199; 319.2406; 322.3389; 347.3619; 356.0618; 361.3133; 369.1479; 394.8656; 399.8741; 422.1569; 450.2656; 477.9580; 482.6921; 489.7399; 490.4374; 530.5775; 535.7401; 558.2682; 558.6339; 573.9654; 578.3015; 591.3533; 616.5090; 622.8172; 626.9016; 637.5542; 640.2150; 652.8930; 714.3241; 720.0069; 748.4510; 765.0617; 766.7491; 767.5503; 799.6404; 838.5548; 875.5001; 935.3933; 935.7091; 937.6545; 951.2829; 965.0556; 981.6290; 982.5919; 992.8533; 1004.1728; 1007.0303; 1012.1296; 1017.6969; 1052.6088; 1058.6783; 1061.6592; 1067.9228; 1068.8278; 1075.0449; 1096.5223; 1127.2858; 1191.3368; 1196.9571; 1200.9143; 1202.0673; 1204.3725; 1217.3495; 1245.2059; 1259.8825; 1266.8615; 1267.1336; 1288.2656; 1296.4270; 1308.6342; 1318.6542; 1327.4308; 1363.4528; 1406.6829; 1434.0394; 1436.3548; 1446.4409; 1460.9660; 1464.5188; 1500.5949; 1506.0164; 1510.8811; 1515.1190; 1515.2504; 1517.9328; 1528.0706; 1530.6922; 1548.2476; 1609.5164; 1614.8395; 1619.8931; 1677.1809; 1803.9403; 3044.5764; 3067.5509; 3070.4390; 3076.7930; 3112.6132; 3136.5567; 3137.6295; 3138.2372; 3141.7135; 3146.1473; 3146.8484; 3148.3605; 3155.1093; 3166.0967; 3175.5532; 3185.4013; 3201.5266.

Cartesian Forces: Max 0.000007919 RMS 0.000001280

Item	Value	Threshold	Converged?
Maximum Force	0.000006	0.000450	YES
RMS Force	0.000001	0.000300	YES
Maximum Displacement	0.000545	0.001800	YES
RMS Displacement	0.000116	0.001200	YES

FILE: Yb_Sq2_PhYbOTf2_TS_Neutral_Add12_B3LYP_M062x_Confx10-.out

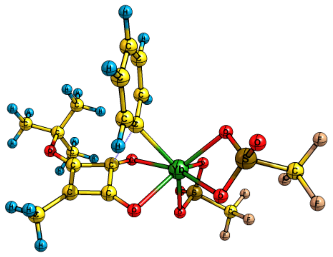

Conformer 21

E(RB3LYP/6-31G(d)/MWB59) = -2769.89582051
Convg = 0.6499D-08 -V/T = 2.0149

Temperature 195.150 Kelvin. Pressure 1.00000 Atm.
elec. and zero-point Energies= -2769.552769
elec. and thermal Energies= -2769.534142
elec. and thermal Enthalpies= -2769.533524
elec. and thermal Free Energies= -2769.593829

RB3LYP/6-31G(d)/MWB59/THF(IEFPCM) = -2769.91747600

RM06-2x/6-311+G(d,p)/MWB59/THF(IEFPCM) = -2769.72374559

Geometry at RB3LYP/6-31G(d)/MWB59
Charge = 0 Multiplicity = 1
Yb 2.2895406359 0.6144307589 -1.4260408665
O 2.2033694772 2.8799032044 -1.9846283688
S 2.3952514031 2.7245077656 -3.4868799925
O 1.4128680478 3.3542226138 -4.3508507893
O 2.6607847734 1.2439291861 -3.6716394871
C 4.0270268116 3.5528219887 -3.8544234765
F 3.9530167367 4.8358681023 -3.506400413
F 5.0050539769 2.9596323407 -3.1680033818
F 4.2760829281 3.451694647 -5.1583106101
O 4.4654133527 -0.2294305432 -1.5063954208
O 5.3349211856 -1.1206374772 0.7098262431
O 3.6729106147 0.7979417815 0.5141816539
S 4.8384065016 0.0131886358 -0.0504522763
C 6.2381627863 1.245743239 -0.1244672898
F 7.229483301 0.7439173121 -0.8587720592
F 5.8128526596 2.3876772537 -0.6705293298
F 6.6698007358 1.4836723758 1.1130509957
C -0.5101501667 -1.7764190145 0.6059806308
C -0.9514973007 -0.5174632597 1.0206453041
C 0.1797262515 0.1309090009 0.3983804773
C 0.7353809325 -1.1965067361 -0.0569260569
O 1.9089095116 -1.5655555376 -0.2638152806
O 0.6446097015 1.2655536963 0.2060145371
O -1.0688958919 -2.931710668 0.8096910638
C -0.3779663815 -4.2465342582 0.5675275068
C -2.137648398 -0.0441521905 1.779461108
H -2.6846988662 -0.8849000462 2.2149218201
H -2.8207062997 0.5078467698 1.1207343193
H -1.8405534031 0.6404806558 2.5816079247
C -0.0234994804 -4.3722605887 -0.9106750273
H 0.7713500108 -3.6799282257 -1.1931839388
H 0.333763349 -5.3896084133 -1.1040717013
H -0.8985784924 -4.1902697233 -1.5419783238
C -1.4530583097 -5.2485751037 0.9819359278
H -1.06680632 -6.2666815071 0.8725671417
H -1.7431870198 -5.0989698865 2.0264779976
H -2.3432996945 -5.1467650858 0.3534785816
C 0.8484334654 -4.3122468483 1.4771992173
H 0.5655542545 -4.157516484 2.5235757545
H 1.3069080647 -5.3032562808 1.3923744584
H 1.6007857998 -3.5715489836 1.1911231629
C 0.2618048008 -0.5312944239 -2.3013009591
C 0.6696518058 -1.5948833087 -3.1273495685
C -0.8925058043 0.1788415951 -2.6887887839
H 1.5379995275 -2.1959967284 -2.8462212195
H -1.2546306631 1.0063929045 -2.0772409647
C -0.0014106948 -1.909032544 -4.3145818253
C -1.5815262702 -0.1320404459 -3.863254051
H 0.3474282755 -2.7240207794 -4.94469638
H -2.4605673718 0.440556429 -4.1500797031
C -1.1326796028 -1.1766815987 -4.6788869901
H -1.6671762612 -1.4193734059 -5.5938183934

Frequency: -199.2585; 9.0911; 13.9099; 23.1363; 26.4054; 28.1238; 31.6457; 35.7367; 37.4068; 45.3557; 54.7478; 57.8104; 59.2499; 67.7602; 74.2993; 83.3201; 86.6500; 92.3678; 112.4194; 124.9348; 129.8016; 148.6050; 153.2172; 161.9465; 169.8824; 177.8141; 194.6525; 203.6399; 215.2811; 215.6484; 222.8730; 224.1368; 242.9480; 247.4927; 264.6517; 274.5170; 277.4739; 289.5594; 304.8342; 309.4599; 318.8442; 322.3025; 334.9152; 341.1940; 355.9492; 361.8951; 368.9582; 397.3733; 401.9712; 408.9951; 452.6618; 464.6898; 489.1771; 489.2987; 490.4729; 530.3262; 536.3001; 558.2459; 558.6030; 573.1222; 578.1461; 592.5224; 615.3985; 623.5503; 625.9381; 634.4409; 640.0748; 649.9639; 713.0698; 720.9798; 741.5125; 750.5580; 765.4869; 767.4550; 795.6127; 852.9214; 875.7586; 936.1179; 939.1426; 943.9943; 951.5368; 966.0574; 975.9522; 982.8864; 990.5491; 1004.3440; 1008.0164; 1017.2264; 1021.6841; 1057.7390; 1058.6852; 1066.2805; 1069.8667; 1071.2640; 1074.3844; 1096.1930; 1100.8457; 1191.8716; 1195.2152; 1201.2668; 1204.5690; 1214.4867; 1245.3731; 1247.4625; 1261.8518; 1266.4616; 1267.8017; 1290.0753; 1306.3812; 1312.4438; 1318.0691; 1326.8614; 1360.2008; 1415.5803; 1435.2447; 1436.7824; 1455.2713; 1462.8002; 1465.4925; 1503.1493; 1504.4712; 1507.2793; 1512.6772; 1514.8474; 1517.0383; 1533.0786; 1534.9288; 1549.1338; 1606.7508; 1616.2433; 1618.7893; 1654.4793; 1790.5598; 3050.2275; 3062.8595; 3065.9583; 3073.1101; 3108.0323; 3117.6098; 3132.3896; 3134.9098; 3141.3944; 3141.6332; 3144.8589; 3145.2205; 3150.1065; 3174.1279; 3178.7281; 3187.6107; 3204.2639.

Cartesian Forces: Max 0.000000775 RMS 0.000000301

Item	Value	Threshold	Converged?
Maximum Force	0.000000	0.000450	YES
RMS Force	0.000000	0.000300	YES
Maximum Displacement	0.000040	0.001800	YES
RMS Displacement	0.000007	0.001200	YES

FILE: Yb_Sq2_PhYbOTf2_TS_Neutral_Add12_B3LYP_M062x_Confx11.out

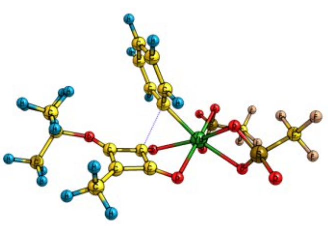

Conformer 22

E(RB3LYP/6-31G(d)/MWB59) = -2769.88776842
Convg = 0.6621D-08 -V/T = 2.0149

Temperature 195.150 Kelvin. Pressure 1.00000 Atm.
elec. and zero-point Energies= -2769.544691
elec. and thermal Energies= -2769.526063
elec. and thermal Enthalpies= -2769.525445
elec. and thermal Free Energies= -2769.585422

RB3LYP/6-31G(d)/MWB59/THF(IEFPCM) = -2769.91315221

RM06-2x/6-311+G(d,p)/MWB59/THF(IEFPCM) = -2769.72134114

Geometry at RB3LYP/6-31G(d)/MWB59
Charge = 0 Multiplicity = 1
Yb 2.438264015 0.7233578866 -1.0277171773
O 3.2684345651 2.9106808285 -0.7899962209
S 2.5461985818 3.5319859884 -1.9732032352
O 1.7933240256 4.7519206472 -1.7401723503
O 1.8278820574 2.3669744983 -2.6243498684
C 3.9041058776 3.9727930915 -3.1764445785
F 4.7415902387 4.8321260907 -2.5977762562
F 4.5715364706 2.8745237247 -3.5371395702
F 3.3602361296 4.5328947078 -4.2561983978
O 4.1030964569 -0.1676194703 -2.3735046981
O 5.6160233851 -1.8948127512 -1.293805091
O 4.4672291333 -0.0258746543 -0.0096548617
S 5.1024060006 -0.5362929446 -1.2829389448
C 6.5662665552 0.5838598087 -1.5812626279
F 7.0520336598 0.3519520817 -2.7995345876
F 6.1959976698 1.8610194204 -1.4836049137
F 7.4987306752 0.3218705268 -0.6668056859
C -0.8573863856 -1.1947470466 0.5854760328
C -1.3914977909 0.1061350484 0.666560709
C -0.0884276754 0.6816026041 0.361669101
C 0.5343566864 -0.6732219992 0.3425093955
O 1.7193408118 -0.9972569358 0.5408976617
O 0.4647345187 1.7683487279 0.158996824
O -1.1801984344 -2.4493397323 0.6444914544
C -2.5605408824 -3.0197274372 0.6988764889
C -2.6976469425 0.7931832693 0.8654372502
H -3.3663071205 0.2623710045 1.5473328969
H -3.218601678 0.9155590645 -0.0941168498
H -2.5225900066 1.7973441505 1.2645551912
C -3.1363227619 -2.7623275079 2.0907115711
H -3.2726609361 -1.6961254096 2.2902000177
H -4.1149896431 -3.2461680504 2.1763231408
H -2.4801038895 -3.1781581581 2.861395206
C -2.2941598864 -4.5083699917 0.4743356075
H -3.2364055959 -5.0637063803 0.5169487192
H -1.83460982 -4.672654402 -0.504653516
H -1.6230191811 -4.9010737145 1.2438719414
C -3.3994624487 -2.4345112971 -0.4369180995
H -2.8740537808 -2.5302500133 -1.3923426839
H -4.3414634909 -2.9886385023 -0.5058186841
H -3.6479270364 -1.3831585917 -0.2764817963
C 0.6308766334 -0.5998996349 -2.0954343737
C 0.8876606828 -1.9698934158 -2.3086255418
C -0.3369352115 0.004503659 -2.9286372056
H 1.595445715 -2.4936587541 -1.6645090704
H -0.5698407972 1.0617435346 -2.8084069083
C 0.256568733 -2.6943616085 -3.3274766209
C -0.9829798276 -0.7089940789 -3.9394993155
H 0.4920934271 -3.7455578604 -3.4785519016
H -1.7083570264 -0.2127487026 -4.5808959095
C -0.6853715132 -2.0629465771 -4.1409356727
H -1.1856140668 -2.6188057405 -4.9304983584

Frequency: -154.7379; 8.2552; 19.0198; 21.1119; 24.5522; 29.9535; 33.2113; 36.1913; 39.2127; 52.1940; 57.7118; 61.9073; 65.1181; 67.1089; 75.0582; 82.1477; 100.9333; 105.8559; 112.4241; 125.7228; 132.9375; 148.5407; 155.2671; 161.6916; 167.0252; 179.3193; 189.8930; 195.0672; 205.3024; 215.3770; 221.3753; 231.3579; 235.1641; 238.2156; 251.5653; 261.1891; 267.2975; 276.0401; 303.1831; 307.4154; 312.5645; 319.4609; 321.7691; 342.1110; 354.9671; 360.8251; 367.8570; 393.7275; 398.7361; 416.8963; 450.7106; 475.7621; 481.8449; 489.9437; 491.1564; 530.7197; 535.1288; 558.3971; 558.8165; 574.9590; 579.3795; 594.0026; 615.0397; 622.6865; 627.1833; 636.5345; 639.4648; 647.4675; 715.7936; 718.2573; 749.9417; 765.2715; 766.4172; 768.3614; 798.7713; 835.7164; 872.8001; 934.4455; 935.7288; 938.7067; 954.3762; 966.7342; 979.7955; 983.1704; 993.3096; 1000.2323; 1007.7294; 1014.9559; 1017.4767; 1051.4192; 1061.5201; 1064.1582; 1068.3452; 1069.7695; 1076.4340; 1096.6559; 1145.4763; 1190.5827; 1195.5584; 1199.4249; 1201.4537; 1203.9487; 1220.0787; 1247.0864; 1257.1671; 1266.3611; 1266.7296; 1288.8417; 1296.3329; 1308.8093; 1318.4640; 1327.5932; 1366.0283; 1409.8377; 1434.5292; 1436.5460; 1447.4642; 1461.4673; 1463.3150; 1500.1062; 1505.3470; 1510.2434; 1515.0218; 1516.1113; 1517.6981; 1527.7376; 1531.1803; 1548.0326; 1605.6624; 1613.1596; 1619.7806; 1687.9106; 1805.4345; 3045.5894; 3067.4301; 3069.8977; 3076.2540; 3112.6129; 3137.8156; 3138.1951; 3142.1312; 3146.4904; 3147.1489; 3149.0682; 3154.7686; 3160.6435; 3164.7596; 3175.3488; 3184.3138; 3199.3608.

Cartesian Forces: Max 0.000000838 RMS 0.000000316

Item	Value	Threshold	Converged?
Maximum Force	0.000001	0.000450	YES
RMS Force	0.000000	0.000300	YES
Maximum Displacement	0.000121	0.001800	YES
RMS Displacement	0.000014	0.001200	YES

FILE: Yb_Sq2_PhYbOTf2_TS_Neutral_Add12_B3LYP_M062x_Confx11-.out

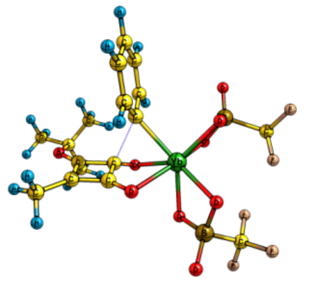

Conformer 23

E(RB3LYP/6-31G(d)/MWB59) = -2769.89480289
Convg = 0.5640D-08 -V/T = 2.0149

Temperature 195.150 Kelvin. Pressure 1.00000 Atm.
elec. and zero-point Energies= -2769.551585
elec. and thermal Energies= -2769.533065
elec. and thermal Enthalpies= -2769.532447
elec. and thermal Free Energies= -2769.591858

RB3LYP/6-31G(d)/MWB59/THF(IEFPCM) = -2769.91663687

RM06-2x/6-311+G(d,p)/MWB59/THF(IEFPCM) = -2769.72469403

Geometry at RB3LYP/6-31G(d)/MWB59
Charge = 0 Multiplicity = 1
Yb 1.1933768947 2.0183730067 -0.440253312
O 0.6142590333 2.7575189606 1.7532798159
S 0.1336520643 4.1336342261 1.3400231474
O -1.1869464306 4.5550168519 1.7730306313
O 0.4443279778 4.2140656699 -0.1469532167
C 1.3475773601 5.3059017633 2.1386673085
F 1.1127097206 5.3438664647 3.4494365406
F 2.5971117634 4.8833614205 1.9272710212
F 1.1956795282 6.5222096232 1.6184554928
O 2.5030417211 2.9627438516 -2.1682248304
O 4.8274487557 1.9423829694 -2.3548375978
O 3.5113750875 2.0636680934 -0.1881406581
S 3.851260197 2.6560667335 -1.5507128814
C 4.5991431077 4.3263106225 -1.1782490826
F 4.9459954607 4.9047779202 -2.3259543175
F 3.7138673969 5.0944539689 -0.5430441507
F 5.6760958418 4.1594084396 -0.4126198536
C -0.7836301463 -1.6112877126 0.2007497353
C -1.9175870325 -0.8414422242 -0.0641803932
C -1.129747613 0.3727206921 -0.1324182471
C 0.1274152168 -0.3947969299 0.1933093569
O 1.1788155451 -0.045730635 0.7802063737
O -1.2535915452 1.5799807337 -0.3714002696
O -0.7035960984 -2.8975784173 0.3747067125
C 0.5082549068 -3.5900756262 0.9338872432
C -3.3565568959 -1.160305473 -0.2451835169
H -3.5687821181 -2.1936275174 0.0427028791
H -3.649006583 -1.0272209853 -1.2951005304
H -3.983274484 -0.4879944552 0.3511631009
C 1.7087364266 -3.3452944528 0.0246791418
H 2.0531030183 -2.3110784242 0.0824855713
H 2.5335507464 -3.9904595371 0.3463080345
H 1.4699543273 -3.5873651285 -1.015352179
C 0.0715978136 -5.0532655792 0.9164077269
H 0.8678559733 -5.678844334 1.3314884441
H -0.8311679437 -5.1974396594 1.517942529
H -0.1325725615 -5.3860436601 -0.1060300013
C 0.7309290681 -3.0754131284 2.3558362078
H -0.1677659956 -3.2145058144 2.9655675395
H 1.5477098543 -3.6385864906 2.8197375586
H 1.0112040585 -2.0179622124 2.3589076248
C 0.8100066289 0.1424217609 -2.0507506736
C 1.9933350695 -0.5752447068 -2.3099222509
C -0.1025024947 0.2809171477 -3.1171827509
H 2.7221617944 -0.7268843219 -1.5122742489
H -1.0417856924 0.8138915574 -2.9641883523
C 2.2842177324 -1.0881370378 -3.5790262103
C 0.171726184 -0.2306809319 -4.3875167633
H 3.2168535841 -1.6190235807 -3.7545502717
H -0.5403103424 -0.0931971939 -5.1983620623
C 1.3685397652 -0.9171882936 -4.6186644651
H 1.583724096 -1.3182124709 -5.6059086349

Frequency: -191.1612; 12.8673; 18.6912; 26.9109; 27.9775; 29.4737; 34.8239; 36.2834; 38.8128; 49.0539; 54.7103; 61.8042; 62.9627; 70.3060; 75.8877; 84.0347; 91.3543; 96.0733; 104.9353; 120.7436; 135.5735; 153.0575; 158.6757; 162.7716; 172.3852; 183.0608; 191.8052; 203.2162; 215.7832; 217.0814; 220.9990; 223.3907; 243.9449; 246.6078; 269.7382; 274.2546; 280.2475; 286.2734; 304.5396; 308.6242; 319.5192; 322.2562; 332.2641; 343.2066; 353.1075; 361.2321; 368.1566; 398.0674; 403.0897; 418.5301; 452.5931; 464.9794; 489.1935; 490.0747; 490.5210; 530.6224; 535.7434; 558.3219; 558.7650; 573.8595; 578.2470; 591.6717; 615.5963; 622.4442; 626.2762; 633.5140; 639.3564; 650.1015; 711.9253; 720.6086; 741.4485; 749.9211; 765.1593; 766.8661; 795.2972; 854.1071; 876.2307; 936.9469; 939.6794; 944.2066; 951.3121; 965.1708; 977.5580; 984.2080; 991.4606; 1003.6059; 1008.4197; 1016.4263; 1020.4841; 1057.3267; 1058.2185; 1067.6932; 1069.8352; 1071.5468; 1074.7528; 1096.9295; 1102.0252; 1191.9526; 1195.9463; 1200.5276; 1203.8759; 1216.8169; 1244.9344; 1245.5582; 1260.1303; 1267.4779; 1267.7337; 1289.5490; 1306.2951; 1311.1472; 1318.1689; 1326.9779; 1362.1832; 1413.9644; 1435.1970; 1437.1762; 1453.5125; 1462.7198; 1465.2782; 1503.3657; 1503.5443; 1507.2307; 1513.6816; 1515.4858; 1516.6768; 1532.9728; 1535.2046; 1549.0992; 1607.2946; 1615.3371; 1619.1526; 1639.8934; 1797.7318; 3049.3101; 3062.7599; 3065.9825; 3073.0044; 3107.6501; 3132.6910; 3135.1433; 3139.1562; 3141.8327; 3142.6570; 3145.0238; 3145.6980; 3148.5280; 3171.2858; 3178.3566; 3187.9916; 3204.0155.

Cartesian Forces: Max 0.000005444 RMS 0.000001067

Item	Value	Threshold	Converged?
Maximum Force	0.000003	0.000450	YES
RMS Force	0.000000	0.000300	YES
Maximum Displacement	0.000260	0.001800	YES
RMS Displacement	0.000050	0.001200	YES

FILE:
Yb_Sq2_PhYbOTf2_TS_Neutral_Add12_B3LYP_M062x_Confx12.out

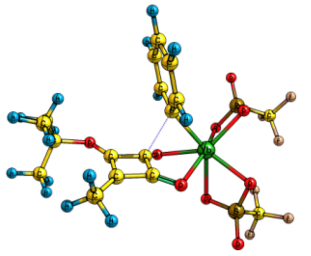

Conformer 24

E(RB3LYP/6-31G(d)/MWB59) = -2769.88786708
A.U. after 1 cycles Convg = 0.3638D-08 -V/T = 2.0149

Temperature 195.150 Kelvin. Pressure 1.00000 Atm.
elec. and zero-point Energies= -2769.544694
elec. and thermal Energies= -2769.526170
elec. and thermal Enthalpies= -2769.525552
elec. and thermal Free Energies= -2769.585153

RB3LYP/6-31G(d)/MWB59/THF(IEFPCM) = -2769.91294624

RM06-2x/6-311+G(d,p)/MWB59/THF(IEFPCM) = -2769.72022191

Geometry at RB3LYP/6-31G(d)/MWB59
Charge = 0 Multiplicity = 1
Yb 1.1309930725 2.0463553135 -0.3906429231
O 0.4894629345 2.9035022005 1.7466403386
S 0.1284133511 4.2918357103 1.2612364712
O -1.1777716376 4.8216082687 1.6116068721
O 0.5155186942 4.2950084637 -0.2100761508
C 1.3835080479 5.4069147876 2.0774306412
F 1.0925427763 5.5102989661 3.3734021401
F 2.6086364337 4.8920670518 1.9407626305
F 1.3418143511 6.6108332215 1.5094731612
O 2.572447747 2.8392006642 -2.0922839497
O 4.816675037 1.6403086092 -2.1534930631
O 3.4269229215 1.9267089408 -0.0478328667
S 3.8669313707 2.449170357 -1.4107425963
C 4.725632706 4.0675247485 -1.0493680529
F 5.1552794354 4.5893989566 -2.1964524967
F 3.8790250905 4.915928298 -0.4649880291
F 5.759442229 3.841131975 -0.2408737315
C -1.0944477409 -1.406873933 0.1644230021
C -2.1957018783 -0.5815916621 -0.1078751964
C -1.3393381703 0.6000377431 -0.1238376661
C -0.149083421 -0.2376709853 0.2569480412
O 0.9075963645 0.0248076521 0.8650360297
O -1.4084102875 1.8045126771 -0.3799317654
O -0.7417720764 -2.6479523159 0.3220341501
C -1.6232515494 -3.8449020292 0.2018135103
C -3.6529847213 -0.6717005548 -0.4035472215
H -4.1639690877 -1.448424085 0.1708584219
H -3.8204434075 -0.8750346013 -1.4702069941
H -4.1266599226 0.2895431671 -0.180612826
C -2.6167910431 -3.8360392141 1.3635740758
H -3.3013158179 -2.9851632938 1.3160066393
H -3.2180114598 -4.750922117 1.3373670344
H -2.0875642947 -3.8011249653 2.3207487926
C -0.6227066299 -4.9927602893 0.3404215648
H -1.1497198996 -5.9510935243 0.2967157747
H 0.1140958217 -4.9625000644 -0.4676819126
H -0.0930523969 -4.9293316308 1.2954875199
C -2.2848844198 -3.8570951646 -1.1760878526
H -1.5320535956 -3.7520681896 -1.9634934462
H -2.7982165257 -4.8137353865 -1.3191978096
H -3.026893466 -3.0643092661 -1.2922804977
C 0.6359705412 0.2001892345 -1.9817200482
C 1.6480674842 -0.7709879874 -2.1151613043
C -0.1785644878 0.43763768 -3.1097112173
H 2.2760967477 -1.0173958108 -1.2576000986
H -0.9939549679 1.1603228138 -3.0497699695
C 1.8798197109 -1.433707234 -3.3261075868
C 0.0414592724 -0.2169152088 -4.3233172435
H 2.6794728379 -2.1666595191 -3.4047955819
H -0.5844666003 0.002597037 -5.1857336417
C 1.0746829586 -1.1549012441 -4.4316388072
H 1.2464648687 -1.6680317493 -5.3746904466

Frequency: -157.5464; 12.2413; 17.3465; 18.0162; 26.0497; 29.0989; 31.8896; 36.9658; 42.5669; 50.1430; 58.5854; 61.4906; 63.3545; 72.0235; 84.2286; 89.4137; 100.5081; 107.1179; 112.1646; 115.6461; 144.6623; 149.7025; 156.3160; 161.9284; 169.2240; 185.2188; 189.8463; 192.8132; 214.2281; 216.6987; 223.0495; 231.7486; 242.9119; 245.3933; 250.4512; 261.6424; 272.4575; 279.8208; 304.3148; 308.6086; 313.0026; 319.2543; 322.5724; 347.4445; 356.0526; 361.3492; 369.1775; 394.8354; 399.8721; 422.2894; 450.2949; 478.0085; 482.7484; 489.8094; 490.4786; 530.6539; 535.7749; 558.2884; 558.6562; 574.0556; 578.3774; 591.4524; 616.6772; 622.7329; 626.9189; 637.5196; 640.1807; 653.0147; 714.3336; 720.2397; 748.5699; 765.0617; 766.7101; 767.5803; 799.7429; 838.6465; 875.6198; 935.5683; 935.7531; 937.4944; 951.3095; 965.0917; 981.8723; 982.6764; 992.9415; 1004.2504; 1007.0559; 1012.1263; 1017.6627; 1052.6043; 1058.7530; 1061.6660; 1067.9457; 1068.8399; 1075.1285; 1096.6937; 1127.2352; 1191.4075; 1196.9540; 1200.8613; 1202.1602; 1204.3846; 1217.4926; 1245.1461; 1259.7780; 1266.8807; 1267.2007; 1288.2747; 1296.2597; 1308.6922; 1318.7119; 1327.4768; 1363.6656; 1406.7225; 1433.9918; 1436.4315; 1446.3458; 1461.0091; 1464.5761; 1500.5621; 1506.2522; 1510.8938; 1514.9714; 1515.3864; 1517.9887; 1528.0848; 1530.6706; 1548.0150; 1609.5471; 1614.9162; 1619.9099; 1677.2922; 1803.9666; 3044.6626; 3067.5580; 3070.4304; 3076.8057; 3112.7634; 3136.8299; 3137.6711; 3138.2097; 3141.5881; 3146.2948; 3146.8742; 3148.3804; 3155.2403; 3166.0353; 3175.6388; 3185.4756; 3201.6650.

Cartesian Forces: Max 0.000001482 RMS 0.000000533

Item	Value	Threshold	Converged?
Maximum Force	0.000000	0.000450	YES
RMS Force	0.000000	0.000300	YES
Maximum Displacement	0.000011	0.001800	YES
RMS Displacement	0.000002	0.001200	YES

FILE: Yb_Sq2_PhYbOTf2_TS_Neutral_Add12_B3LYP_M062x_Confx12-
.out

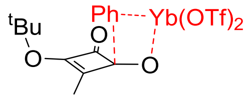

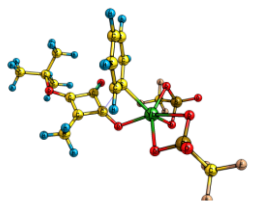

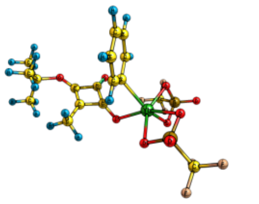

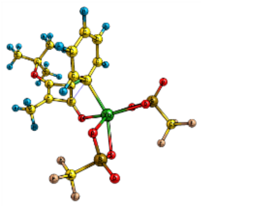

Trapezoidal Transition State for $PhYb(OTf)_2$ addition to C-2 carbonyl of 1b

Lowest energy conformation is: Conformer 7

Conformer 1

E(RB3LYP/6-31G(d)/MWB59) = -2769.89905539
Convg = 0.3482D-08 -V/T = 2.0149

Temperature 195.150 Kelvin. Pressure 1.00000 Atm.
elec. and zero-point Energies= -2769.556054
elec. and thermal Energies= -2769.537302
elec. and thermal Enthalpies= -2769.536684
elec. and thermal Free Energies= -2769.597727

RB3LYP/6-31G(d)/MWB59/THF(IEFPCM) = -2769.91996356

RM06-2x/6-311+G(d,p)/MWB59/THF(IEFPCM) = -2769.72010262

Geometry at RB3LYP/6-31G(d)/MWB59
Charge = 0 Multiplicity = 1
Yb -1.6455416068 -1.4381997365 0.053795304
O -3.6286949941 -2.1770403675 1.1156408694
S -4.4546614796 -0.9376421509 0.8337234065
O -5.1969484498 -0.3481968485 1.932910677
O -3.5071176657 -0.024757677 0.0647816377
C -5.6921980575 -1.4857591652 -0.4509015301
F -6.4932617377 -2.4063329131 0.0767175702
F -5.0401056524 -2.0022019301 -1.496035628
F -6.4065774018 -0.4347523419 -0.8463845995
O -0.5885083072 -3.5292852151 0.0607695754
O -1.5514353632 -5.2847128529 -1.5077329929
O -1.7924378103 -2.7792680625 -1.8726326282
S -0.9911909281 -3.9570690465 -1.3418438282
C 0.6029015541 -3.9021450136 -2.3110599439
F 1.4468002683 -4.8033012633 -1.8141206123
F 1.1403179697 -2.6843441137 -2.2158014392
F 0.3466143857 -4.1779643175 -3.5879337944
C 0.7264884406 1.8149334701 0.5766333257
C 2.0668049273 1.5249196457 0.7467514913
C 1.8994959934 0.1137981987 0.2319875653
C 0.4159380573 0.469575472 0.0651937068
O -0.4247768459 0.1070176089 -0.8373067172
O 2.5946050372 -0.8427620983 -0.0091274068
O 3.0281138117 2.3141067694 1.1709076239
C -0.0985840091 3.0281590977 0.783376573
H -0.8433578656 3.130691238 -0.0134817234
H 0.5225606984 3.9280293908 0.8174964367
H -0.6449797876 2.9581000611 1.7336276633
C 4.447834755 1.8725160728 1.2738815156
C 4.9681140661 1.5239993839 -0.121704315
H 6.0439281732 1.324745599 -0.0682211559
H 4.8116442634 2.3599496183 -0.8115701713
H 4.4821602449 0.6292514836 -0.5188373639
C 4.5411724793 0.6973575144 2.248648851
H 4.0646753282 -0.1989899775 1.8440717984
H 4.0773866201 0.9532582437 3.2069532811
H 5.5953189055 0.462543161 2.432071152
C 5.1332078229 3.1194004677 1.832272772
H 4.7129973502 3.389553076 2.8061324363
H 5.0083170558 3.9678858005 1.1522076444
H 6.2039204801 2.9299249756 1.9570594632
C -0.3317193613 -0.6709614931 1.9460094692
C 0.5728396696 -1.5692853937 2.5536124495
C -1.1158756549 0.1333220182 2.8058564137
H 1.197487786 -2.2089519777 1.9342956046
H -1.8351917662 0.8399883124 2.3909941631
C 0.6841712901 -1.6644719162 3.9433399297
C -1.0122961592 0.0406236263 4.1969619084
H 1.3822862407 -2.3716387747 4.3850215771
H -1.6371402213 0.6627489034 4.8330928531
C -0.1057254268 -0.8567608826 4.7660817452
H -0.0203641227 -0.9317976802 5.8470983964

Frequency: -233.6436; 9.8367; 16.8063; 21.5084; 23.7206; 25.7718; 27.6156; 32.8437; 37.3982; 41.2550; 45.2224; 51.8161; 54.5140; 60.0049; 63.6292; 75.3097; 83.3911; 102.4391; 110.3646; 125.6729; 139.4481; 142.4602; 154.9241; 161.3984; 164.3859; 179.3421; 193.0990; 196.6635; 208.3977; 214.4772; 217.3138; 222.9984; 233.5354; 238.5153; 256.3882; 269.6957; 272.3169; 292.0184; 303.8705; 306.2423; 319.4850; 320.7832; 338.9258; 350.2556; 364.2413; 365.1548; 373.7147; 397.0866; 399.0374; 449.2255; 456.5513; 490.1056; 491.2612; 495.4392; 505.4876; 531.7115; 535.5254; 557.7736; 558.0769; 575.3189; 577.4963; 583.0428; 596.3054; 624.5441; 626.1818; 633.4470; 638.1046; 641.5859; 721.4417; 722.6134; 743.3683; 766.0982; 766.2954; 769.6823; 787.5806; 851.4388; 875.1707; 936.2194; 940.3406; 942.3638; 953.0357; 959.2655; 963.1521; 987.6654; 988.3880; 1005.3921; 1007.1257; 1012.5392; 1017.0160; 1054.1885; 1058.8447; 1064.6198; 1067.0422; 1069.4693; 1070.7760; 1086.4246; 1098.6496; 1194.2427; 1199.3567; 1200.1813; 1204.1572; 1221.8490; 1232.5315; 1254.3871; 1255.4886; 1268.3913; 1272.4396; 1286.8411; 1303.3638; 1305.2132; 1323.7983; 1330.4548; 1367.8198; 1394.6565; 1433.6102; 1434.4086; 1440.4490; 1460.9400; 1464.1991; 1492.4280; 1504.1983; 1504.3325; 1516.4272; 1516.7046; 1517.8808; 1531.7569; 1535.6296; 1536.3696; 1552.9703; 1614.4544; 1622.3498; 1684.6472; 1860.7022; 3046.2090; 3062.2582; 3062.7489; 3069.7590; 3105.8247; 3128.7549; 3130.5992; 3139.5303; 3141.5531; 3142.6731; 3148.3948; 3156.0877; 3159.7191; 3177.9241; 3187.4125; 3195.2649; 3207.1942.

Cartesian Forces: Max 0.000001245 RMS 0.000000494

Item	Value	Threshold	Converged?
Maximum Force	0.000001	0.000450	YES
RMS Force	0.000000	0.000300	YES
Maximum Displacement	0.000090	0.001800	YES
RMS Displacement	0.000015	0.001200	YES

FILE: Yb_Sq2_PhYbOTf2_TS_Neutral_Add56_B3LYP_M062x_Conf1.out

Conformer 2

E(RB3LYP/6-31G(d)/MWB59) = -2769.88992160
Convg = 0.4982D-08 -V/T = 2.0149

Temperature 195.150 Kelvin. Pressure 1.00000 Atm.
elec. and zero-point Energies= -2769.546985
elec. and thermal Energies= -2769.528247
elec. and thermal Enthalpies= -2769.527629
elec. and thermal Free Energies= -2769.588625

RB3LYP/6-31G(d)/MWB59/THF(IEFPCM) = -2769.91303541

RM06-2x/6-311+G(d,p)/MWB59/THF(IEFPCM) = -2769.71583294

Geometry at RB3LYP/6-31G(d)/MWB59
Charge = 0 Multiplicity = 1
Yb 1.6690095596 1.4556695747 -0.0359443038
O 3.616438248 2.3316571734 -1.0577582328
S 4.5133770092 1.1394911824 -0.7899995347
O 5.3029098916 0.6190080807 -1.8912734465
O 3.61528584 0.154863416 -0.0514356218
C 5.7004439606 1.7360351891 0.5203127455
F 6.4494693812 2.7144025791 0.0211109267
F 5.0062578535 2.1899935339 1.5673092189
F 6.473627708 0.7227008019 0.9038711473
O 0.4941281082 3.4807797172 -0.0249848705
O 1.3124575116 5.254456106 1.6043560885
O 1.6994169316 2.7604716479 1.918639276
S 0.8383492723 3.8995395717 1.3959266937
C -0.7704516854 3.7218204142 2.325519189
F -1.6557675595 4.5848627887 1.8340100272
F -1.2296177571 2.4771521259 2.1834173385
F -0.5586808479 3.9753037949 3.6152083999
C -0.4552682253 -1.9523909444 -0.6199149515
C -1.8140675818 -1.7455871422 -0.8053291745
C -1.7573517865 -0.3384170375 -0.2674908349
C -0.2552314499 -0.5865706793 -0.083746279
O 0.5638715773 -0.1931689905 0.8242592743
O -2.5423485329 0.5407871658 -0.0307474085
O -2.9187382506 -2.3298332351 -1.2083059489
C 0.5819111077 -3.0044574332 -0.7913188117
H 1.4086323225 -2.8131983937 -0.0985885094
H 0.2087482385 -4.0157231096 -0.6111171463
H 0.9953852022 -2.9760084642 -1.8085502551
C -3.0408715866 -3.7242738012 -1.6914079318
C -2.1154769128 -3.9458851049 -2.8895500019
H -2.3163738892 -4.9301976931 -3.325685619
H -2.2985984052 -3.1884171011 -3.6581328744
H -1.0588730366 -3.9150977936 -2.6156781956
C -2.7705859197 -4.6781972 -0.5259629234
H -1.7455243091 -4.597614733 -0.1552786344
H -3.4531690107 -4.4695927534 0.3037151298
H -2.9299327902 -5.7121354616 -0.8499615409
C -4.5081069539 -3.7917941747 -2.120534008
H -5.1658086684 -3.5582867556 -1.2780099689
H -4.7081990246 -3.0761700257 -2.9236951578
H -4.7465420307 -4.7973325673 -2.4816168713
C 0.4177257645 0.6348532486 -1.9464233782
C -0.5545204398 1.4653983006 -2.5454130864
C 1.270067776 -0.0878862238 -2.8142259052
H -1.2370682951 2.0364635157 -1.9205555526
H 2.0424812024 -0.7408768206 -2.4066549678
C -0.664902791 1.5753860014 -3.9341006679
C 1.1683463119 0.0211302268 -4.2043106967
H -1.4185092363 2.2279426072 -4.3683165718
H 1.8471732219 -0.5350799075 -4.8463459839
C 0.1944041189 0.8511098593 -4.7646882858
H 0.1098898268 0.9384904899 -5.8448895839

Frequency: -228.9428; 8.7438; 18.4998; 20.4161; 22.0853; 25.0951; 27.2332; 33.2655; 37.6777; 41.7993; 46.1485; 50.0821; 59.7810; 61.5997; 66.5543; 75.9390; 92.5477; 103.4584; 116.6293; 128.7888; 139.5235; 154.9144; 158.8895; 161.6061; 164.8500; 179.2638; 185.5265; 193.5607; 209.6679; 213.6926; 218.3234; 223.2859; 233.1468; 236.5258; 238.2718; 256.9856; 262.6923; 284.2770; 303.8296; 305.9147; 319.4224; 320.9258; 338.6727; 345.0726; 364.3727; 365.2687; 379.6485; 397.9726; 400.7718; 450.5028; 453.3416; 482.3372; 490.1246; 491.2521; 516.1301; 531.6713; 535.4927; 557.7718; 558.0423; 576.5549; 577.6875; 583.0228; 596.7587; 624.5297; 626.2554; 633.2461; 639.9027; 649.1738; 721.9797; 732.0689; 761.3087; 766.1942; 766.3333; 772.3914; 781.3951; 838.6458; 874.9720; 933.4096; 935.2369; 940.7533; 953.0203; 959.8133; 974.5911; 980.5332; 987.8749; 1007.0832; 1008.1456; 1012.3462; 1016.3843; 1051.5604; 1058.0165; 1060.4439; 1064.2146; 1065.2919; 1069.6949; 1088.6210; 1100.0748; 1191.3354; 1194.1796; 1199.0990; 1199.9603; 1205.5782; 1220.9958; 1254.3222; 1256.3151; 1269.0962; 1272.2750; 1285.3115; 1294.5950; 1303.3886; 1323.5316; 1330.3615; 1366.5715; 1381.9186; 1432.2172; 1434.5136; 1438.0524; 1459.7590; 1464.0758; 1498.6883; 1502.2531; 1506.2156; 1515.2272; 1516.3372; 1516.7540; 1523.9391; 1529.7818; 1531.1610; 1548.9838; 1614.5500; 1622.3525; 1684.3334; 1889.4743; 3045.5569; 3065.6274; 3068.8731; 3075.3414; 3108.1693; 3134.6964; 3135.8016; 3141.8937; 3144.2571; 3145.7371; 3147.0436; 3154.4182; 3166.4065; 3179.3846; 3187.6816; 3196.6686; 3207.1695.

Cartesian Forces: Max 0.000019189 RMS 0.000003336

Item	Value	Threshold	Converged?
Maximum Force	0.000016	0.000450	YES
RMS Force	0.000002	0.000300	YES
Maximum Displacement	0.000743	0.001800	YES
RMS Displacement	0.000125	0.001200	YES

FILE: Yb_Sq2_PhYbOTf2_TS_Neutral_Add56_B3LYP_M062x_Conf1-.out

Conformer 3

E(RB3LYP/6-31G(d)/MWB59) = -2769.89835750
Convg = 0.4345D-08 -V/T = 2.0149

Temperature 195.150 Kelvin. Pressure 1.00000 Atm.
elec. and zero-point Energies= -2769.555209
elec. and thermal Energies= -2769.536541
elec. and thermal Enthalpies= -2769.535923
elec. and thermal Free Energies= -2769.596390

RB3LYP/6-31G(d)/MWB59/THF(IEFPCM) = -2769.91911639

RM06-2x/6-311+G(d,p)/MWB59/THF(IEFPCM) = -2769.71872286

Geometry at RB3LYP/6-31G(d)/MWB59
Charge = 0 Multiplicity = 1
Yb 1.7121706708 1.4196726397 -0.5813560267
O 0.9293110563 3.5050821597 0.0680742481
S 1.735238509 4.3508754002 -0.9120605134
O 1.0191334824 5.3683367181 -1.6582221635
O 2.5500818524 3.337329078 -1.6927207073
C 2.9839970594 5.221127168 0.1680499928
F 2.3500520052 6.070152594 0.9726423443
F 3.6399996344 4.3162315465 0.8990917947
F 3.8430601901 5.8817135581 -0.6028544442
O 3.5965519045 1.0300540574 0.8064500456
O 5.7064014739 0.2584004766 -0.3848954999
O 3.3604884514 -0.0970242148 -1.2981632689
S 4.2982386524 0.0469153381 -0.1091146053
C 4.1615806543 -1.5934921751 0.7705516798
F 4.8873040723 -1.560678241 1.8836100087
F 2.8806235443 -1.8334874529 1.0837061275
F 4.6042924418 -2.559676382 -0.032001481
C -0.9245661256 -1.6129586091 -0.4447592494
C -2.2618551834 -1.2846102734 -0.5588455187
C -2.0023989654 0.1750405504 -0.2634001487
C -0.5235605938 -0.2259632766 -0.15587046
O 0.3920767185 0.2194934555 0.6302597342
O -2.6419583297 1.1850452138 -0.1044306516
O -3.2824888753 -2.0781067036 -0.7936222195
C -0.1672542451 -2.8835372163 -0.5426677096
H 0.6848436861 -2.8854048834 0.1441990948
H -0.8092352094 -3.7433970368 -0.328336643
H 0.2305006211 -3.0088174952 -1.5592062924
C -4.6879449002 -1.5852996658 -0.8617498694
C -5.4615587801 -2.862991507 -1.1861260598
H -5.3194277792 -3.6122494879 -0.4009845489
H -6.5303443727 -2.6401048651 -1.2630064868
H -5.1267003269 -3.2882315896 -2.1374585287
C -4.8075789544 -0.5563720303 -1.987244707
H -4.4302019123 -0.9686832263 -2.9288132249
H -5.8622111474 -0.2957172643 -2.1279510943
H -4.2651824199 0.3625392892 -1.7514819761
C -5.0878014598 -1.0180603179 0.5013303215
H -4.5384700246 -0.1016207739 0.7309509091
H -6.1558908675 -0.775025248 0.4934892567
H -4.9132966959 -1.7552251559 1.2921335071
C 0.1294506849 0.6011341925 -2.2298882354
C -0.7432228652 1.5788142369 -2.7607216339
C 0.6685741895 -0.3394757814 -3.1373245696
H -1.1982941046 2.3181853729 -2.1040618127
H 1.3599421577 -1.1026927825 -2.7839735082
C -1.0524575259 1.6216650638 -4.1235806909
C 0.3615136957 -0.3035713552 -4.4998203816
H -1.7190110714 2.3913464777 -4.5048401566
H 0.7991439575 -1.0339498212 -5.1762856835
C -0.5035805301 0.6767135708 -4.9934008593
H -0.743285099 0.7076776741 -6.053182436

Frequency: -232.5028; 11.7114; 19.8494; 22.3549; 23.6568; 25.1820; 29.7442; 37.3739; 38.5532; 42.0976; 47.7532; 51.5458; 58.0362; 63.0935; 65.8698; 73.7380; 90.0486; 105.4221; 116.0318; 122.3636; 143.1273; 145.6596; 154.5330; 161.4523; 164.8481; 179.1962; 193.1501; 201.9127; 208.0384; 214.3911; 216.2246; 222.5340; 230.8027; 239.9060; 256.5422; 269.3165; 272.8883; 293.8096; 303.4680; 305.4870; 319.8612; 320.8879; 339.0225; 350.6595; 362.4493; 365.5168; 374.4188; 397.0586; 399.3509; 449.9736; 457.5917; 490.3277; 491.4067; 494.9312; 506.2176; 531.3320; 535.5388; 557.5429; 558.2160; 575.9260; 576.7124; 583.0784; 596.0433; 624.5561; 626.2220; 633.2193; 638.4137; 641.7504; 721.1446; 722.0090; 743.7881; 764.1678; 766.2716; 770.2337; 787.9489; 850.7584; 874.2208; 936.2883; 940.3186; 942.5186; 952.2715; 959.9024; 964.1451; 986.7685; 988.2424; 1004.9829; 1007.6940; 1012.1206; 1016.7606; 1054.3342; 1059.3293; 1066.9709; 1067.8608; 1069.4588; 1071.0168; 1087.4058; 1099.1235; 1194.4065; 1199.0816; 1200.2746; 1203.8100; 1222.8756; 1232.3447; 1241.0533; 1255.3840; 1271.0724; 1272.3787; 1287.0137; 1302.3385; 1304.7553; 1325.2635; 1331.4862; 1368.5037; 1395.5580; 1433.6463; 1434.4743; 1440.6065; 1460.9697; 1463.9087; 1494.3292; 1504.0769; 1507.4866; 1516.5440; 1517.0550; 1517.9728; 1529.4699; 1535.4314; 1536.3734; 1553.0055; 1614.5123; 1622.6604; 1684.4720; 1863.1454; 3045.9462; 3062.2025; 3062.7166; 3069.7049; 3110.0689; 3128.6583; 3130.5128; 3139.3088; 3142.6545; 3146.5500; 3156.3901; 3159.8939; 3166.4913; 3172.3859; 3184.6303; 3193.8055; 3207.1005.

Cartesian Forces: Max 0.000006323 RMS 0.000001526

Item	Value	Threshold	Converged?
Maximum Force	0.000007	0.000450	YES
RMS Force	0.000001	0.000300	YES
Maximum Displacement	0.001026	0.001800	YES
RMS Displacement	0.000182	0.001200	YES

FILE: Yb_Sq2_PhYbOTf2_TS_Neutral_Add56_B3LYP_M062x_Conf2.out

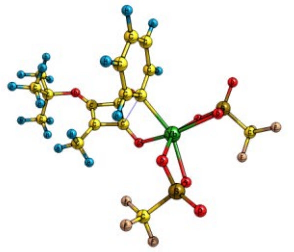

Conformer 4

E(RB3LYP/6-31G(d)/MWB59) = -2769.88883681
Convg = 0.4732D-09 -V/T = 2.0149

Temperature 195.150 Kelvin. Pressure 1.00000 Atm.
elec. and zero-point Energies= -2769.545733
elec. and thermal Energies= -2769.527079
elec. and thermal Enthalpies= -2769.526461
elec. and thermal Free Energies= -2769.586889

RB3LYP/6-31G(d)/MWB59/THF(IEFPCM) = -2769.91210198

RM06-2x/6-311+G(d,p)/MWB59/THF(IEFPCM) = -2769.71443673

Geometry at RB3LYP/6-31G(d)/MWB59
Charge = 0 Multiplicity = 1
Yb 1.5381962166 1.4030297534 -0.4742203655
O 0.4958526416 3.1107832297 0.6963145365
S 0.9641846553 4.2785543818 -0.1656101218
O -0.038123544 5.2381839414 -0.588562039
O 1.8219666978 3.628086635 -1.2345613889
C 2.1804845687 5.182131382 0.9243814569
F 1.5325787478 5.7049716676 1.9620987831
F 3.1051041766 4.3229683077 1.3601352238
F 2.7635090841 6.1478816108 0.2198023754
O 3.6484501373 1.1957262388 0.6022569372
O 5.6650127174 1.1563072732 -0.9464868723
O 3.3304437676 0.4405686522 -1.6524834811
S 4.3882433453 0.5812308627 -0.5681134826
C 4.7280049495 -1.1764218467 -0.040484788
F 5.6073677846 -1.1767322833 0.9561552329
F 3.5914532671 -1.7550942317 0.3725671399
F 5.2146883824 -1.8589789959 -1.0756428116
C -0.4967058272 -2.0767360552 -0.6978967368
C -1.8750184541 -1.9686949952 -0.5890097124
C -1.8048460464 -0.5731009971 -0.0234506634
C -0.2829316802 -0.7108999651 -0.1660709033
O 0.6734418022 -0.2751247968 0.5740463552
O -2.5819804404 0.234629067 0.4090808051
O -2.99685208 -2.6256978055 -0.7726813372
C 0.5457439285 -3.0468942506 -1.1281754523
H 1.488717334 -2.821507167 -0.6202414104
H 0.2831393468 -4.08673076 -0.9181846385
H 0.7278551162 -2.9598610974 -2.2080972121
C -3.1241320834 -4.017995656 -1.2599215708
C -4.6411437796 -4.185868494 -1.3699493887
H -4.8822831881 -5.2005801869 -1.7024746787
H -5.1178767718 -4.0134873428 -0.400459509
H -5.0564126746 -3.4746928506 -2.0903558285
C -2.547082864 -4.9668012671 -0.2072698587
H -3.0497728106 -4.8209410657 0.7538784288
H -2.700940861 -6.0046855813 -0.5211446905
H -1.4745374395 -4.8170319776 -0.0593227262
C -2.4636461027 -4.1539799886 -2.6333985903
H -1.377577577 -4.0496573369 -2.5894969451
H -2.6852831826 -5.1439302588 -3.0461288274
H -2.858644112 -3.4009065122 -3.3224830817
C -0.089773793 0.5998237225 -2.0862010521
C -1.1983344561 1.4454191305 -2.3198496241
C 0.4920050534 -0.0228562988 -3.2148158206
H -1.6958882562 1.9354969484 -1.485182373
H 1.3597372691 -0.6696516184 -3.0944349668
C -1.6918289134 1.6664840462 -3.6090168624
C 0.0022361887 0.1924390381 -4.5056549956
H -2.5411054736 2.3285413034 -3.7582000271
H 0.4773985165 -0.2906146136 -5.3563688998
C -1.0942362691 1.0361080144 -4.702716667
H -1.4769980177 1.206342091 -5.7057808727

Frequency: -226.7657; 11.7289; 17.5011; 20.9354; 23.6767; 26.4851; 29.1480; 33.7870; 39.3120; 44.6049; 50.5966; 50.7218; 60.1119; 60.6891; 68.7594; 76.7843; 95.2834; 107.9808; 118.9953; 139.3209; 143.6160; 156.0558; 159.8893; 163.9574; 164.9741; 178.6760; 184.7823; 192.9589; 209.3430; 214.1083; 215.9874; 221.9673; 230.7143; 237.0518; 240.2684; 258.4954; 265.5383; 285.9605; 302.9768; 305.2342; 319.5460; 320.8745; 338.9922; 346.0637; 362.3229; 365.7937; 379.8280; 398.1817; 400.4677; 450.7470; 454.5821; 482.8847; 490.3609; 491.3694; 515.5711; 531.3832; 535.6292; 557.6642; 558.1986; 576.3422; 576.6697; 582.4965; 596.4809; 624.6957; 626.3560; 633.3434; 640.3593; 648.5008; 721.7417; 732.6849; 761.1688; 764.0770; 766.2605; 772.8940; 781.0237; 838.5721; 873.9566; 933.2674; 935.0426; 940.6936; 952.0560; 960.7886; 974.9349; 980.5395; 986.6235; 1007.6807; 1008.0683; 1012.0079; 1016.5870; 1052.6195; 1058.3761; 1060.2759; 1064.2837; 1068.9738; 1071.3380; 1088.6339; 1100.5278; 1191.5208; 1194.3668; 1198.9983; 1200.6734; 1205.5717; 1223.2913; 1239.6412; 1255.8719; 1270.6603; 1272.0765; 1285.3656; 1294.6442; 1302.6340; 1325.3197; 1331.3391; 1368.8441; 1382.3902; 1432.2040; 1434.4285; 1439.5569; 1459.6711; 1463.7837; 1498.8128; 1502.0764; 1508.6801; 1515.0608; 1516.7287; 1517.2305; 1524.5767; 1530.0443; 1531.8312; 1549.1449; 1614.5627; 1622.6702; 1684.0568; 1891.9707; 3046.3872; 3065.6573; 3068.8486; 3075.3471; 3115.4769; 3134.6809; 3135.9020; 3144.2480; 3145.7908; 3146.8968; 3154.7507; 3163.6435; 3166.5444; 3175.4570; 3183.9099; 3194.0239; 3207.0469.

Cartesian Forces: Max 0.000000563 RMS 0.000000226

Item	Value	Threshold	Converged?
Maximum Force	0.000000	0.000450	YES
RMS Force	0.000000	0.000300	YES
Maximum Displacement	0.000012	0.001800	YES
RMS Displacement	0.000002	0.001200	YES

FILE: Yb_Sq2_PhYbOTf2_TS_Neutral_Add56_B3LYP_M062x_Conf2-.out

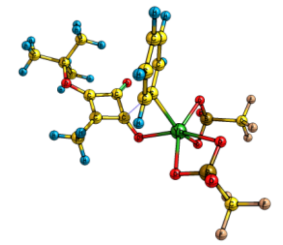

Conformer 5

E(RB3LYP/6-31G(d)/MWB59) = -2769.89864188
Convg = 0.4336D-08 -V/T = 2.0149

Temperature 195.150 Kelvin. Pressure 1.00000 Atm.
elec. and zero-point Energies= -2769.555628
elec. and thermal Energies= -2769.536878
elec. and thermal Enthalpies= -2769.536260
elec. and thermal Free Energies= -2769.597287

RB3LYP/6-31G(d)/MWB59/THF(IEFPCM) = -2769.91925661

RM06-2x/6-311+G(d,p)/MWB59/THF(IEFPCM) = -2769.71979893

Geometry at RB3LYP/6-31G(d)/MWB59
Charge = 0 Multiplicity = 1
Yb -1.667821264 -1.2369612014 0.0289633771
O -3.6370661619 -2.0127139536 1.0948752563
S -4.4514139772 -0.7471389234 0.9119946172
O -5.1667224081 -0.2248708224 2.0617401965
O -3.5098441078 0.2023952259 0.1841713194
C -5.7184115878 -1.1983956768 -0.3809219293
F -6.5147656863 -2.150644442 0.0963557248
F -5.0893486385 -1.6461245313 -1.4727937771
F -6.434321184 -0.1208756998 -0.6910198962
O -1.8450680515 -2.4246195166 -1.9934675673
O 0.0451106988 -4.0467331649 -2.5092551398
O -0.5821487658 -3.3081564204 -0.1569471956
S -0.9894646872 -3.6143403458 -1.5895735179
C -2.2053067058 -5.0208307284 -1.4264618366
F -2.7380044484 -5.279052436 -2.6184186179
F -3.1715033371 -4.6774306622 -0.5701708249
F -1.569976854 -6.0979044736 -0.9713436171
C 0.7717018318 1.9329217777 0.7361135137
C 2.1106383817 1.6068019549 0.8365823978
C 1.8964923207 0.2405681875 0.2263765495
C 0.4160545434 0.6350549148 0.13780462
O -0.4558134335 0.359221328 -0.7673535237
O 2.5614246368 -0.7086848132 -0.1081845319
O 3.1026141502 2.3449981608 1.2818619622
C -0.0196547158 3.1433896339 1.0588849682
H -0.783240075 3.3223023071 0.2938768413
H 0.6226757038 4.0249149533 1.1430494094
H -0.5414596782 3.0122125337 2.01644925
C 4.516364299 1.8731654633 1.2925418348
C 4.9735951698 1.6226960226 -0.1454840039
H 6.0466388039 1.402523502 -0.1514985431
H 4.8058420428 2.5107820131 -0.7638411892
H 4.4553554403 0.7684197341 -0.5882724758
C 4.625557169 0.6259611892 2.1714641771
H 4.116447482 -0.2291729428 1.7200871554
H 4.2053204938 0.8159219522 3.1646097744
H 5.6814408664 0.3607954171 2.2932161846
C 5.2478703998 3.0625370684 1.9143169004
H 4.8724377446 3.2648565726 2.9223592049
H 5.1124569148 3.9621789193 1.3057611055
H 6.3188147664 2.8464863968 1.9799514481
C -0.3042564447 -0.6213639193 1.94127735
C 0.5978127506 -1.5746103603 2.4630991485
C -1.0587420474 0.1298040627 2.8726712816
H 1.1996022114 -2.176302577 1.7857836916
H -1.773698252 0.8756662345 2.5242764245
C 0.7345573911 -1.7714680081 3.8398799021
C -0.929503116 -0.0649198696 4.2509989691
H 1.4305237745 -2.5181062939 4.2150250481
H -1.5313628873 0.5188385337 4.9431997946
C -0.0262439473 -1.0142061213 4.7349205925
H 0.0794613659 -1.168180995 5.8057470352

Frequency: -238.5416; 9.3098; 16.6346; 21.6260; 22.5279; 26.0738; 28.8548; 33.7071; 37.8026; 43.7250; 44.8575; 52.6305; 55.1548; 61.9496; 62.7047; 74.3993; 81.8283; 101.6355; 108.9241; 126.8534; 140.2911; 143.0826; 156.1816; 159.9132; 163.6573; 175.3952; 193.7264; 199.7815; 207.9973; 215.2319; 218.8211; 220.7671; 230.6150; 238.6821; 256.1368; 270.0884; 272.8374; 291.9648; 303.0545; 305.8598; 319.4119; 321.0282; 338.8561; 350.5809; 362.5326; 365.8607; 373.8280; 397.4680; 399.4277; 449.5138; 457.2088; 490.0961; 491.2373; 495.8283; 506.0194; 531.6041; 535.2269; 557.7671; 558.1350; 574.6230; 578.2602; 585.8657; 596.4063; 621.8705; 626.2197; 634.2976; 637.9049; 641.7105; 721.3061; 722.7390; 743.9653; 765.5992; 765.9985; 770.6102; 787.5701; 851.4098; 875.8865; 936.1471; 941.0107; 942.6265; 952.5585; 959.8766; 964.0005; 988.2013; 988.8654; 1005.6535; 1007.3898; 1012.6209; 1016.7790; 1054.6396; 1059.3606; 1066.2530; 1067.1518; 1069.6123; 1070.5468; 1086.4082; 1098.9808; 1194.2423; 1199.9261; 1201.0987; 1204.2099; 1221.8555; 1231.8398; 1249.9048; 1253.5255; 1270.0209; 1273.0408; 1286.9168; 1303.4499; 1305.3308; 1323.7895; 1332.2595; 1368.0986; 1394.3092; 1433.5368; 1434.6378; 1440.3791; 1461.0249; 1464.4693; 1491.1570; 1504.0790; 1504.2407; 1516.5761; 1516.7882; 1517.8540; 1529.8314; 1535.5665; 1536.5906; 1553.0743; 1614.6159; 1622.3953; 1684.3290; 1862.5738; 3045.8636; 3062.1547; 3062.5125; 3069.5712; 3105.3289; 3128.5838; 3130.2973; 3139.1606; 3141.2639; 3142.5160; 3148.6430; 3156.2056; 3159.7405; 3178.2570; 3187.7257; 3195.6167; 3207.0873.

Cartesian Forces: Max 0.000019232 RMS 0.000002362

Item	Value	Threshold	Converged?
Maximum Force	0.000013	0.000450	YES
RMS Force	0.000001	0.000300	YES
Maximum Displacement	0.000872	0.001800	YES
RMS Displacement	0.000230	0.001200	YES

FILE: Yb_Sq2_PhYbOTf2_TS_Neutral_Add56_B3LYP_M062x_Confx5.out

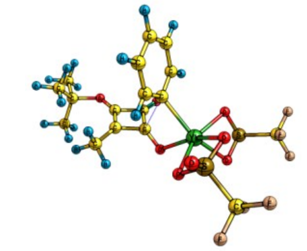

Conformer 6

E(RB3LYP/6-31G(d)/MWB59) = -2769.88925585
Convg = 0.9251D-08 -V/T = 2.0149

Temperature 195.150 Kelvin. Pressure 1.00000 Atm.
elec. and zero-point Energies= -2769.546302
elec. and thermal Energies= -2769.527563
elec. and thermal Enthalpies= -2769.526945
elec. and thermal Free Energies= -2769.587959

RB3LYP/6-31G(d)/MWB59/THF(IEFPCM) = -2769.91305074

RM06-2x/6-311+G(d,p)/MWB59/THF(IEFPCM) = -2769.71642204

Geometry at RB3LYP/6-31G(d)/MWB59
Charge = 0 Multiplicity = 1
Yb 1.6850362696 1.2822332259 0.2201779484
O 3.662122457 2.1609146215 -0.7464929236
S 4.5270660576 0.9333529989 -0.5395214968
O 5.3165508193 0.4589151345 -1.6613406911
O 3.6012255072 -0.0687088387 0.1358442312
C 5.7130779224 1.4289142584 0.8125812297
F 6.4843824567 2.4220537254 0.379583271
F 5.0164642207 1.8347929073 1.8791949745
F 6.4643786977 0.3821577989 1.1444027387
O 1.7260987138 2.4416224899 2.2678545399
O -0.2646278652 3.9539036793 2.7378967421
O 0.4832141457 3.2854584003 0.3994352912
S 0.82279926 3.590540147 1.8501927785
C 1.9674738294 5.0610294512 1.7484976478
F 2.4452999204 5.328500996 2.9615101532
F 2.9792158946 4.7810870676 0.9217631354
F 1.2927878126 6.1104179512 1.2858527
C -0.4766345557 -2.0388728416 -0.623749183
C -1.8312895043 -1.8078029768 -0.8094263102
C -1.7684567031 -0.4381258403 -0.1817872721
C -0.2707194509 -0.7126702983 0.0024239284
O 0.5362619304 -0.3914385367 0.9502767631
O -2.5475141291 0.4313367281 0.1027074369
O -2.9368164865 -2.3554306149 -1.2592818558
C 0.5524778738 -3.0886776047 -0.8502820789
H 1.3753880188 -2.9473203562 -0.1412316665
H 0.168549658 -4.1052392942 -0.7331982808
H 0.9741624566 -3.0044295148 -1.8610316882
C -3.0683334125 -3.719646805 -1.8190130974
C -2.134112131 -3.8852611678 -3.0194203257
H -2.3399138099 -4.8426422717 -3.5098737636
H -2.3034504557 -3.0856949395 -3.747457417
H -1.0798487982 -3.8792961444 -2.7350580916
C -2.8185757538 -4.7388829381 -0.7053805561
H -1.7965763556 -4.6888753507 -0.321087359
H -3.5073081215 -4.5693228948 0.1280922788
H -2.9844011209 -5.7518304557 -1.0872884789
C -4.5320918465 -3.7483201887 -2.2641671063
H -5.1952126894 -3.55422421 -1.4159289199
H -4.7175148531 -2.9877339656 -3.0286096508
H -4.7771628389 -4.7300732919 -2.6818674343
C 0.4439657122 0.6202764356 -1.7570246704
C -0.5047140799 1.5173376857 -2.2953393519
C 1.286757061 -0.0526098808 -2.6729106497
H -1.1787930773 2.0537700978 -1.6317312102
H 2.0407431675 -0.7528334545 -2.31219475
C -0.6016965627 1.7364543798 -3.6722733784
C 1.1979176925 0.1653726932 -4.0507876915
H -1.3377137276 2.4370257899 -4.0593057474
H 1.8685504591 -0.3555635462 -4.7299756378
C 0.2471593522 1.0584790617 -4.551124471
H 0.1725197296 1.2301543814 -5.6219292801

Frequency: -231.4278; 8.3569; 17.4962; 20.4332; 22.6015; 23.6960; 30.1462; 32.3604; 37.6385; 43.7355; 45.8351; 50.2782; 59.3426; 61.4583; 66.0956; 73.8196; 92.4454; 104.6795; 117.7287; 130.0201; 141.2401; 157.0349; 159.5256; 160.2753; 163.9613; 175.7313; 184.2277; 196.4814; 209.4547; 214.3357; 219.1967; 220.8163; 230.3845; 236.1684; 238.5231; 256.4491; 262.6993; 284.2514; 303.1181; 305.8951; 319.4073; 321.0934; 338.8390; 345.4955; 362.5241; 365.8847; 380.0224; 397.9349; 400.5858; 451.0266; 454.3314; 482.6380; 490.0475; 491.2375; 516.5001; 531.5788; 535.2836; 557.7402; 558.1110; 575.7768; 578.2739; 585.7889; 596.8172; 621.8112; 626.2421; 634.1737; 640.0283; 649.4013; 722.1603; 732.4312; 761.5439; 765.5736; 766.0567; 772.9368; 781.5528; 839.0251; 875.5289; 933.3166; 934.9160; 941.5532; 952.4967; 961.4947; 974.4819; 980.9010; 988.6358; 1007.1968; 1008.2202; 1012.3708; 1016.3748; 1052.0670; 1058.9492; 1060.6512; 1064.1716; 1066.8243; 1070.1426; 1088.1700; 1100.1194; 1191.1788; 1194.1448; 1200.0768; 1200.9998; 1205.9627; 1221.8225; 1250.0818; 1253.2082; 1270.3033; 1272.7077; 1285.3625; 1294.5785; 1303.8648; 1323.5724; 1332.5867; 1367.8719; 1381.4838; 1432.2449; 1434.4017; 1438.1166; 1459.6891; 1464.1127; 1497.9065; 1501.6443; 1506.9077; 1514.8684; 1516.5572; 1516.8864; 1523.1981; 1529.3308; 1531.1535; 1548.8176; 1614.5437; 1622.3715; 1684.0512; 1891.7262; 3045.5947; 3065.5590; 3068.7800; 3075.2661; 3108.1449; 3134.5023; 3135.8054; 3141.6928; 3144.0658; 3145.8171; 3147.8176; 3154.3050; 3166.5084; 3179.3750; 3187.6580; 3196.7021; 3206.9668.

Cartesian Forces: Max 0.000001284 RMS 0.000000458

Item	Value	Threshold	Converged?
Maximum Force	0.000001	0.000450	YES
RMS Force	0.000000	0.000300	YES
Maximum Displacement	0.000506	0.001800	YES
RMS Displacement	0.000110	0.001200	YES

FILE: Yb_Sq2_PhYbOTf2_TS_Neutral_Add56_B3LYP_M062x_Confx5-.out

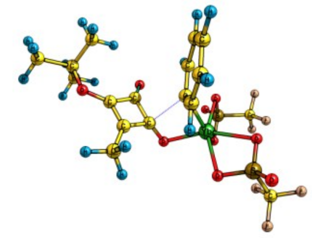

Conformer 7

E(RB3LYP/6-31G(d)/MWB59) = -2769.89864538
Convg = 0.7432D-08 -V/T = 2.0149

Temperature 195.150 Kelvin. Pressure 1.00000 Atm.
elec. and zero-point Energies= -2769.555613
elec. and thermal Energies= -2769.536878
elec. and thermal Enthalpies= -2769.536260
elec. and thermal Free Energies= -2769.597203

RB3LYP/6-31G(d)/MWB59/THF(IEFPCM) = -2769.91975488

RM06-2x/6-311+G(d,p)/MWB59/THF(IEFPCM) = -2769.72034448

Geometry at RB3LYP/6-31G(d)/MWB59
Charge = 0 Multiplicity = 1
Yb 1.6132184708 1.3292611809 -0.2938820611
O 1.873040557 2.9960202636 1.345932574
S 1.1746770318 4.1146550241 0.5899961071
O 0.1735206098 4.8880403211 1.2989835865
O 0.7733430964 3.475311931 -0.7299963225
C 2.5517341218 5.2863560761 0.1275718526
F 3.0784752266 5.801721336 1.2357643536
F 3.4936992338 4.621875977 -0.547710484
F 2.0606125308 6.2596076943 -0.6355758772
O 3.6890053718 1.5687182613 -1.4117812562
O 5.0254371216 -0.5676533179 -1.7590918902
O 3.279793609 -0.2775345589 0.0633506439
S 4.3451055577 0.3269926672 -0.8408498303
C 5.6210165239 0.9862960697 0.349913022
F 6.5358538097 1.677825135 -0.3236598386
F 5.0200083979 1.7849594402 1.2381597553
F 6.1972731174 -0.0306247979 0.9851790865
C -1.1604952524 -1.6387767311 -0.2822245484
C -2.4473397822 -1.2129852305 -0.5506032219
C -2.0928755538 0.2370875688 -0.3126690083
C -0.6732454685 -0.2723055208 -0.0294381573
O 0.1965167543 0.145586481 0.8215094205
O -2.6512955017 1.305971418 -0.2847028379
O -3.5046425361 -1.9354068484 -0.8459689427
C -0.5078156324 -2.9676708601 -0.2211851007
H 0.2066664284 -3.0105501267 0.6082389298
H -1.2465594355 -3.7661219287 -0.1049596668
H 0.0535574946 -3.1581617755 -1.1458585348
C -4.8505951297 -1.3374198611 -1.0749834497
C -5.6966891845 -2.5666051325 -1.4059630075
H -5.6914842797 -3.2765982551 -0.5729455407
H -6.7310973932 -2.2645109979 -1.5974006375
H -5.3127257995 -3.0731760889 -2.2970169803
C -4.7787249447 -0.3710628904 -2.258597114
H -4.3496226089 -0.8666309744 -3.1357034431
H -5.7903418538 -0.0386686219 -2.5157988764
H -4.1870436849 0.5155744143 -2.0178497967
C -5.3248850204 -0.6595209313 0.2115671768
H -4.7238741447 0.2228954433 0.4451603017
H -6.3633430997 -0.3335622336 0.087925154
H -5.2866806355 -1.359280621 1.0530391282
C 0.2476576013 0.3641831873 -2.0547499454
C -0.5182601287 1.2293150763 -2.8669559552
C 0.936745894 -0.6871399441 -2.703579516
H -1.0656947135 2.0524866093 -2.4134227676
H 1.5469079044 -1.381797445 -2.1255624259
C -0.5874271786 1.0577393046 -4.2521770328
C 0.8749006826 -0.8619564065 -4.0892228048
H -1.177981983 1.7433694949 -4.8552852483
H 1.424874177 -1.6720364199 -4.5619340212
C 0.1061230501 0.0098249732 -4.8639115093
H 0.0531236943 -0.1228226352 -5.9414783584

Frequency: -238.0516; 9.6565; 16.4766; 21.5723; 22.9180; 26.4693; 29.0823; 33.6849; 38.0079; 43.6067; 44.8361; 52.9625; 55.1713; 62.0435; 62.9103; 73.8771; 83.9139; 103.2256; 110.7944; 128.1255; 140.2761; 142.9674; 156.0310; 159.8345; 163.3543; 175.2734; 193.9080; 199.9461; 208.0940; 215.4494; 218.9493; 220.8821; 230.5433; 238.5422; 256.2462; 270.4967; 273.1307; 292.0930; 303.2680; 306.0665; 319.3637; 320.9199; 338.8697; 350.5404; 362.5706; 365.9240; 373.8170; 397.4543; 399.3947; 449.3662; 457.0680; 490.0847; 491.3657; 495.7488; 506.0253; 531.6600; 535.2909; 557.7878; 558.1440; 574.5635; 578.2377; 585.8453; 596.3922; 621.8408; 626.1892; 634.3127; 637.8753; 641.6691; 721.3386; 722.9266; 743.9014; 765.6110; 766.0071; 770.4817; 787.5601; 851.2826; 876.0940; 936.0536; 940.9856; 942.5003; 952.4720; 959.9161; 964.0429; 988.1845; 988.9932; 1005.6739; 1007.4156; 1012.6491; 1016.7442; 1054.7385; 1059.3268; 1066.2786; 1067.1716; 1069.3068; 1070.2722; 1086.4364; 1098.7353; 1194.1899; 1199.8775; 1201.0334; 1204.1606; 1221.4284; 1231.9926; 1250.0180; 1253.5628; 1270.1442; 1272.9458; 1286.9331; 1303.3139; 1305.1857; 1323.7462; 1332.2827; 1367.5719; 1394.4922; 1433.4854; 1434.5497; 1440.4260; 1460.9616; 1464.3494; 1491.5793; 1504.1732; 1504.5147; 1516.3713; 1516.7515; 1517.9180; 1530.1810; 1535.5877; 1536.6474; 1552.9947; 1614.5266; 1622.3460; 1684.4704; 1862.6245; 3046.1579; 3062.1866; 3062.5412; 3069.6141; 3105.5828; 3128.6222; 3130.3394; 3139.2149; 3141.2260; 3142.5413; 3148.1145; 3156.2781; 3159.8692; 3178.0818; 3187.5844; 3195.4300; 3207.0389.

Cartesian Forces: Max 0.000001188 RMS 0.000000454

Item	Value	Threshold	Converged?
Maximum Force	0.000001	0.000450	YES
RMS Force	0.000000	0.000300	YES
Maximum Displacement	0.000028	0.001800	YES
RMS Displacement	0.000008	0.001200	YES

FILE: Yb_Sq2_PhYbOTf2_TS_Neutral_Add56_B3LYP_M062x_Confx6.out

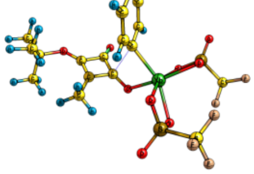

Conformer 8

E(RB3LYP/6-31G(d)/MWB59) = -2769.88946005
Convg = 0.8467D-08 -V/T = 2.0149

Temperature 195.150 Kelvin. Pressure 1.00000 Atm.
elec. and zero-point Energies= -2769.546525
elec. and thermal Energies= -2769.527759
elec. and thermal Enthalpies= -2769.527141
elec. and thermal Free Energies= -2769.588376

RB3LYP/6-31G(d)/MWB59/THF(IEFPCM) = -2769.91227052

RM06-2x/6-311+G(d,p)/MWB59/THF(IEFPCM) = -2769.71630611

Geometry at RB3LYP/6-31G(d)/MWB59
Charge = 0 Multiplicity = 1
Yb 1.4415165572 1.3493031951 -0.4022079801
O 0.5844070727 3.2444542946 0.6369317088
S 1.2779581637 4.2923745019 -0.2251274482
O 0.4681329031 5.3837892484 -0.731958538
O 2.0846475904 3.4818991484 -1.2217775729
C 2.5601702006 5.0253725261 0.9151085424
F 1.9522894284 5.6816571666 1.8989955131
F 3.3049841358 4.0400361068 1.4278918842
F 3.3365544113 5.8572611343 0.2259298127
O 3.0379517468 -0.0049636692 -1.4815712012
O 4.5465698514 -1.3610363725 0.0399124526
O 3.4579625629 0.8313573693 0.7288709484
S 4.0527833763 -0.0515792996 -0.3493950049
C 5.5047611415 0.9227897142 -0.9999463662
F 6.0391140639 0.2746036165 -2.0326929283
F 5.0864937072 2.1272517596 -1.3962693522
F 6.4089515965 1.0590592937 -0.0336545919
C -0.5884020483 -2.0318349134 -0.6208632859
C -1.9741982499 -2.0007002263 -0.6557842156
C -2.0410419311 -0.5893408466 -0.1303976547
C -0.5058030399 -0.6406339195 -0.1193278808
O 0.3484845216 -0.1398878637 0.7022292413
O -2.9036086871 0.1760474026 0.2061316488
O -3.0331139148 -2.7284535808 -0.9278652633
C 0.5491200172 -2.9516961687 -0.8924852391
H 1.412694862 -2.6583967904 -0.2861958117
H 0.3205095543 -3.9985166585 -0.6780481578
H 0.8579110208 -2.8857894116 -1.9445044466
C -3.028807447 -4.1393458494 -1.3758013637
C -4.5134027493 -4.4010549999 -1.638756588
H -4.659982627 -5.437979862 -1.9576186332
H -5.1001562512 -4.228361274 -0.7316573385
H -4.8885822728 -3.7380632716 -2.4242232863
C -2.5150479881 -5.020617729 -0.2352599959
H -3.1255692443 -4.8774649802 0.6617170986
H -2.5747887803 -6.0746187309 -0.5266996503
H -1.4749335952 -4.8020434796 0.0195914615
C -2.2183001655 -4.2764029857 -2.6664232117
H -1.1506289658 -4.1074498837 -2.5118626736
H -2.3393001364 -5.2892989163 -3.065003486
H -2.5783173904 -3.5682148539 -3.4193935846
C -0.211614739 0.6582050974 -2.0260283827
C -1.2483752698 1.6037187253 -2.2006075041
C 0.2957489395 0.036746096 -3.190020516
H -1.6928871588 2.0928660026 -1.3356338431
H 1.1045065722 -0.6872781013 -3.1108120288
C -1.7429313106 1.9224851727 -3.4694001173
C -0.1960780527 0.3490021257 -4.4593501462
H -2.5377297737 2.6567802571 -3.5729589265
H 0.2199531335 -0.1364584673 -5.3392261992
C -1.2189520604 1.2918655379 -4.5992823666
H -1.6025530345 1.5363081395 -5.5865490276

Frequency: -227.6709; 7.0032; 16.4620; 20.3693; 21.9957; 25.3962; 29.8051; 31.2781; 37.6529; 43.4646; 48.3924; 50.5951; 57.3008; 60.7110; 67.6745; 72.7987; 87.4359; 98.2239; 123.0591; 124.9360; 143.6631; 148.2777; 156.0342; 158.9660; 164.5850; 172.7396; 184.5721; 196.6923; 211.9601; 214.2123; 217.6850; 221.6691; 233.3866; 235.7122; 241.0473; 257.7039; 262.6669; 285.4904; 303.4026; 306.3322; 320.1164; 321.3166; 339.6730; 349.2134; 362.7446; 366.1359; 380.7365; 397.1061; 401.5379; 451.5089; 457.1364; 483.9726; 490.3696; 491.4743; 515.8716; 530.9196; 535.1462; 557.9193; 558.5577; 574.8930; 578.6541; 586.7096; 596.1409; 622.3682; 626.0502; 634.0494; 639.9836; 649.0314; 720.5229; 733.6120; 761.3366; 765.6461; 766.3879; 771.5901; 781.0726; 838.6392; 872.9025; 933.2115; 935.1103; 940.8154; 952.3145; 962.6655; 973.2564; 980.5371; 985.8521; 1006.6812; 1008.4756; 1011.3086; 1016.5346; 1049.1366; 1060.2289; 1061.1461; 1064.0350; 1068.4684; 1070.7811; 1088.3510; 1100.3795; 1190.4468; 1194.3421; 1199.4925; 1201.5124; 1205.9354; 1222.5518; 1250.4310; 1254.6036; 1270.3320; 1273.3317; 1285.3189; 1294.5544; 1303.4900; 1319.7161; 1330.5865; 1368.1434; 1381.2429; 1432.2677; 1434.3601; 1438.5301; 1459.6830; 1464.0758; 1492.4481; 1501.2654; 1506.5999; 1514.5541; 1516.3604; 1516.9304; 1522.1655; 1528.9801; 1530.9875; 1549.1505; 1614.5050; 1622.8091; 1683.1830; 1892.5721; 3047.6059; 3065.4848; 3068.7246; 3075.1066; 3110.3809; 3134.2697; 3135.7071; 3143.5443; 3143.9722; 3145.7370; 3154.9966; 3166.1610; 3169.9205; 3173.2412; 3184.9620; 3194.4271; 3207.5358.

Cartesian Forces: Max 0.000001844 RMS 0.000000570

Item	Value	Threshold	Converged?
Maximum Force	0.000001	0.000450	YES
RMS Force	0.000000	0.000300	YES
Maximum Displacement	0.000373	0.001800	YES
RMS Displacement	0.000091	0.001200	YES

FILE: Yb_Sq2_PhYbOTf2_TS_Neutral_Add56_B3LYP_M062x_Confx6-.out

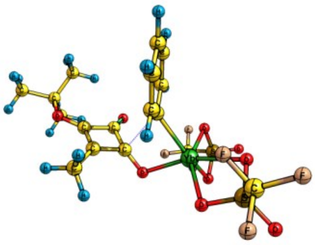

Conformer 9

E(RB3LYP/6-31G(d)/MWB59) = -2769.89612365
Convg = 0.9191D-08 -V/T = 2.0149

Temperature 195.150 Kelvin. Pressure 1.00000 Atm.
elec. and zero-point Energies= -2769.553155
elec. and thermal Energies= -2769.534409
elec. and thermal Enthalpies= -2769.533791
elec. and thermal Free Energies= -2769.595006

RB3LYP/6-31G(d)/MWB59/THF(IEFPCM) = -2769.91853764

RM06-2x/6-311+G(d,p)/MWB59/THF(IEFPCM) = -2769.71897684

Geometry at RB3LYP/6-31G(d)/MWB59
Charge = 0 Multiplicity = 1
Yb -1.6594797048 -1.5222686245 0.0064884544
O -3.6480893282 -0.3019926509 -0.1708084687
S -4.5869615805 -1.3543700503 0.4069043585
O -5.7291601142 -1.7442893544 -0.3978661679
O -3.6567976076 -2.4447865271 0.9019007728
C -5.2762474213 -0.5681137071 1.9527677996
F -5.9931875598 0.5011319531 1.6133916604
F -4.2785744338 -0.1925950115 2.760966148
F -6.046477393 -1.4499189533 2.5827582559
O -0.4701154386 -3.5329768879 -0.0161740944
O -1.3085002373 -5.3193391376 -1.620419887
O -1.6987111564 -2.8251713285 -1.9481617877
S -0.8336936129 -3.9623174973 -1.4308778374
C 0.7626941016 -3.7978792975 -2.3844362365
F 1.6644381078 -4.6317511775 -1.8714249166
F 1.2097228018 -2.5439633573 -2.2957365922
F 0.5395582533 -4.1023863355 -3.6609332711
C 0.6613384533 1.7831008674 0.6093757038
C 2.0024353002 1.4950078216 0.7722351803
C 1.837413174 0.0885215033 0.2447621782
C 0.3507609137 0.4414967465 0.0854179714
O -0.4901545613 0.0954369205 -0.823919891
O 2.5356152397 -0.8627232394 -0.006994875
O 2.9633802782 2.2832189666 1.1994145262
C -0.1624834735 2.9966278398 0.8221134602
H -0.9261570471 3.0875565665 0.0419398474
H 0.4573324475 3.8982127846 0.8282840529
H -0.6832952586 2.9406256427 1.7875040527
C 4.3862086985 1.8475685698 1.2856963959
C 4.8965003465 1.516611502 -0.1177969313
H 5.9737188375 1.3224078601 -0.0753752332
H 4.730019154 2.3589921022 -0.7974060448
H 4.4119545058 0.6236109503 -0.5204927896
C 4.4935296698 0.6626527247 2.2470825037
H 4.0170657013 -0.2314437464 1.83750967
H 4.0378508935 0.906638391 3.2123863412
H 5.5503091891 0.4304226901 2.4182023795
C 5.0700885523 3.0918934915 1.8515798804
H 4.6566474532 3.349762679 2.8316635
H 4.9355829838 3.9468414435 1.1815222339
H 6.1426842383 2.9063865988 1.96562229
C -0.3863833772 -0.7291603703 1.9330142032
C 0.4991918802 -1.6542344336 2.5303178242
C -1.1392844036 0.0918520765 2.8030523
H 1.1034991634 -2.3059507347 1.9035389652
H -1.8374366082 0.8228466623 2.396051723
C 0.6173666277 -1.760289733 3.9188752577
C -1.0286169499 -0.0097220896 4.1925899533
H 1.3000208095 -2.487636651 4.3517342977
H -1.6283454932 0.6286743011 4.8370630989
C -0.1439133252 -0.9354110118 4.7512323384
H -0.0518817843 -1.0170714028 5.8312372357

Frequency: -240.1575; 7.9416; 11.7570; 20.1498; 22.7779; 26.6958; 30.3914; 36.4830; 39.4941; 41.9253; 47.0966; 52.5716; 53.4262; 62.8981; 64.9739; 78.6784; 85.0656; 102.2225; 111.9055; 125.9319; 139.8867; 143.6854; 148.0117; 155.9116; 162.9445; 180.0262; 187.6653; 196.4581; 209.2806; 214.2600; 215.6731; 223.6944; 236.3023; 243.7519; 256.5171; 269.3457; 272.1147; 290.8051; 305.1303; 305.7362; 319.0284; 320.0495; 338.8446; 350.1882; 364.3101; 365.6869; 373.8231; 397.5757; 400.3442; 448.0696; 456.2840; 488.8933; 490.3789; 495.6426; 506.3726; 530.9742; 536.0241; 557.5597; 557.9194; 572.4378; 576.1619; 583.4533; 596.0618; 624.9073; 626.5225; 635.7402; 638.4014; 642.2013; 720.9192; 722.1053; 743.2038; 765.8639; 766.1499; 769.7315; 787.7216; 851.0558; 874.0143; 936.1769; 939.0921; 942.1071; 951.6076; 959.0682; 963.4143; 984.9224; 988.2702; 1004.1023; 1006.8759; 1011.8600; 1017.1511; 1054.9024; 1056.7660; 1062.6660; 1066.7560; 1069.4186; 1070.8113; 1086.0071; 1098.2110; 1193.9062; 1199.7827; 1202.0878; 1204.0087; 1221.4088; 1232.1267; 1248.3841; 1256.0514; 1267.8676; 1270.9458; 1286.8999; 1303.7417; 1305.6035; 1324.6672; 1332.1341; 1367.3539; 1394.1202; 1433.5758; 1434.4272; 1440.0785; 1460.9034; 1464.2310; 1493.1519; 1504.1792; 1505.3915; 1516.5687; 1516.8146; 1517.8187; 1530.0023; 1535.3812; 1536.3294; 1552.9618; 1614.9320; 1622.5372; 1684.8611; 1861.2046; 3045.6669; 3062.3279; 3062.7038; 3069.7571; 3105.4783; 3128.7846; 3130.5518; 3139.4294; 3140.3248; 3142.7195; 3153.2258; 3156.2041; 3159.7923; 3178.7636; 3186.6315; 3195.5776; 3206.9139.

Cartesian Forces: Max 0.000001354 RMS 0.000000479

Item	Value	Threshold	Converged?
Maximum Force	0.000001	0.000450	YES
RMS Force	0.000000	0.000300	YES
Maximum Displacement	0.000114	0.001800	YES
RMS Displacement	0.000017	0.001200	YES

FILE: Yb_Sq2_PhYbOTf2_TS_Neutral_Add56_B3LYP_M062x_Conf5.out

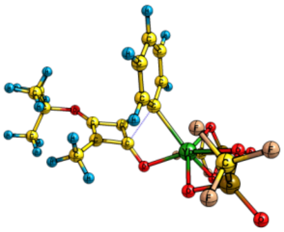

Conformer 10

E(RB3LYP/6-31G(d)/MWB59) = -2769.88688897
Convg = 0.9618D-09 -V/T = 2.0149

Temperature 195.150 Kelvin. Pressure 1.00000 Atm.
elec. and zero-point Energies= -2769.543954
elec. and thermal Energies= -2769.525240
elec. and thermal Enthalpies= -2769.524622
elec. and thermal Free Energies= -2769.585641

RB3LYP/6-31G(d)/MWB59/THF(IEFPCM) = -2769.91170102

RM06-2x/6-311+G(d,p)/MWB59/THF(IEFPCM) = -2769.71474014

Geometry at RB3LYP/6-31G(d)/MWB59
Charge = 0 Multiplicity = 1
Yb 1.752387485 1.374262603 -0.0705674265
O 3.7658134654 0.1834756187 0.0495414401
S 4.6831175988 1.2765681153 -0.4858644438
O 5.8212649039 1.6514663302 0.3318392674
O 3.7319210905 2.3696249528 -0.9309750524
C 5.3811443196 0.5704372773 -2.0660157637
F 6.1027511761 -0.5108996518 -1.776956074
F 4.3882641769 0.2297599383 -2.8950639116
F 6.1490265564 1.485586732 -2.6494416767
O 0.5237064943 3.3593647997 -0.0474645599
O 1.3036315682 5.1466975843 1.5854449609
O 1.7409343977 2.6580140892 1.8942346015
S 0.8588702958 3.7823867394 1.3762391933
C -0.7464883953 3.5736720996 2.3060861249
F -1.6570742641 4.3971040839 1.7928336564
F -1.1661920681 2.3126891502 2.1936182328
F -0.5452612004 3.8645113806 3.589564876
C -0.4604741138 -2.0070233948 -0.6529198796
C -1.8124210648 -1.7502262068 -0.8200002865
C -1.694595074 -0.3415206312 -0.2969266568
C -0.1987063674 -0.6445149278 -0.1315751677
O 0.6464142904 -0.2860762514 0.7677101864
O -2.4437073643 0.5677928105 -0.0588587017
O -2.9453786158 -2.2953134284 -1.1993429519
C 0.5300361924 -3.1040449645 -0.8207211946
H 1.3797185056 -2.9279133736 -0.1520291357
H 0.1211851702 -4.0936481872 -0.6011142582
H 0.9181280452 -3.1236920274 -1.8479954183
C -3.1316090286 -3.6913301452 -1.6560089218
C -2.232993995 -3.9722444225 -2.8620811626
H -2.4815178838 -4.9541475101 -3.2786727955
H -2.394164605 -3.2204976042 -3.6411461411
H -1.1724795062 -3.9823504962 -2.602083938
C -2.8845769174 -4.635579583 -0.477590634
H -1.8514139613 -4.5927191397 -0.1233739386
H -3.5447999019 -4.3833772552 0.3580222249
H -3.0929161189 -5.6672123128 -0.7804522472
C -4.6062228981 -3.7053456207 -2.0644876006
H -5.2418573773 -3.4301329883 -1.2176539834
H -4.7878253049 -2.9960128656 -2.8775750604
H -4.8910099482 -4.7062179047 -2.4043217865
C 0.4905481133 0.5460446838 -1.9871047578
C -0.4366001338 1.429934882 -2.5832117037
C 1.2758102955 -0.2436245151 -2.8579008029
H -1.0703135404 2.0522004914 -1.9559053463
H 2.0065145259 -0.9427324756 -2.4517211796
C -0.5631483677 1.5281171547 -3.9715325277
C 1.1575517676 -0.148923603 -4.2473329385
H -1.2802352151 2.2221269576 -4.4031726437
H 1.7839672798 -0.7604674852 -4.8926575468
C 0.2316498217 0.736518661 -4.8047529584
H 0.1332640802 0.8126283665 -5.88465051

Frequency: -235.4615; 8.6906; 11.6563; 20.4646; 22.9406; 25.0772; 29.9422; 35.2156; 38.9435; 44.7804; 47.7564; 50.7209; 60.1172; 61.7647; 68.5129; 81.3460; 94.4793; 102.2291; 116.3555; 131.3000; 141.6977; 148.2890; 157.7991; 160.7238; 163.5251; 179.9114; 185.8840; 189.2835; 209.9295; 213.9178; 217.6549; 223.9183; 236.5119; 236.9579; 243.5972; 257.4432; 263.7728; 283.3161; 305.1704; 305.4326; 319.0247; 320.1329; 338.1438; 344.6191; 364.4678; 365.8768; 379.9319; 399.0810; 400.9675; 449.7818; 453.1090; 482.3491; 488.9435; 490.3764; 517.0778; 530.9583; 536.0591; 557.5870; 557.9417; 572.9170; 577.2444; 583.7550; 596.5510; 624.8911; 626.5310; 635.5484; 640.9464; 649.2205; 720.8315; 731.2849; 761.1776; 765.8482; 766.2588; 772.5496; 781.9607; 838.7089; 873.2262; 933.4161; 935.2933; 938.9138; 951.4319; 959.9689; 974.5793; 980.6759; 984.2764; 1006.7189; 1007.1425; 1011.7045; 1016.8433; 1052.6137; 1055.7327; 1060.5420; 1063.4239; 1064.0823; 1070.2631; 1088.0556; 1099.4297; 1190.8489; 1193.8407; 1199.1245; 1202.3526; 1205.7680; 1221.0956; 1248.2183; 1257.2528; 1268.4759; 1270.4697; 1285.3158; 1294.6062; 1304.7978; 1324.5233; 1331.9694; 1366.7677; 1380.9557; 1432.1927; 1434.4604; 1437.9499; 1459.7602; 1464.0602; 1499.0458; 1502.1723; 1507.2019; 1515.0520; 1516.4645; 1516.7796; 1523.4005; 1529.5927; 1531.2549; 1548.9887; 1614.8651; 1622.5368; 1684.6288; 1889.9633; 3045.2382; 3065.6823; 3068.8582; 3075.3144; 3107.4667; 3134.5971; 3135.9402; 3140.6346; 3144.2187; 3145.9417; 3152.0600; 3154.0789; 3166.1870; 3179.3982; 3186.7150; 3196.7940; 3207.0333.

Cartesian Forces: Max 0.000000649 RMS 0.000000252

Item	Value	Threshold	Converged?
Maximum Force	0.000000	0.000450	YES
RMS Force	0.000000	0.000300	YES
Maximum Displacement	0.000195	0.001800	YES
RMS Displacement	0.000029	0.001200	YES

FILE: Yb_Sq2_PhYbOTf2_TS_Neutral_Add56_B3LYP_M062x_Conf5-.out

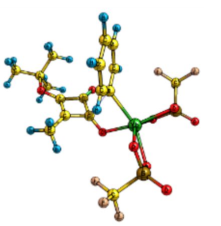

Conformer 11

E(RB3LYP/6-31G(d)/MWB59) = -2769.89652878
Convg = 0.8286D-08 -V/T = 2.0149

Temperature 195.150 Kelvin. Pressure 1.00000 Atm.
elec. and zero-point Energies= -2769.553307
elec. and thermal Energies= -2769.534656
elec. and thermal Enthalpies= -2769.534038
elec. and thermal Free Energies= -2769.594685

RB3LYP/6-31G(d)/MWB59/THF(IEFPCM) = -2769.91854918

RM06-2x/6-311+G(d,p)/MWB59/THF(IEFPCM) = -2769.71791763

Geometry at RB3LYP/6-31G(d)/MWB59
Charge = 0 Multiplicity = 1
Yb 1.698500287 1.4362734828 -0.7268988302
O 2.408548437 3.4057450037 -1.819432911
S 1.8593070879 4.3807876186 -0.7914689272
O 2.7652612057 5.3734091967 -0.2465498228
O 1.1054579422 3.4948312462 0.1904104593
C 0.5177577069 5.3143921338 -1.6922903607
F 1.0645837395 6.0203108175 -2.6782539366
F -0.369062823 4.4523181418 -2.2024932357
F -0.0967780432 6.127022109 -0.8378997594
O 3.5854632017 1.1363902901 0.6838520264
O 5.7186098605 0.3573017931 -0.4653831144
O 3.384698224 -0.0468143777 -1.389774236
S 4.3109841074 0.139178234 -0.1945724561
C 4.1813045037 -1.4818345673 0.7218540422
F 4.8790725318 -1.409942838 1.8504596659
F 2.8970141441 -1.736291861 1.0092615116
F 4.6593066417 -2.4591323721 -0.0464832343
C -0.9558816003 -1.6094231909 -0.4927358547
C -2.2885379847 -1.2619283537 -0.6050158093
C -1.9986474699 0.2039742001 -0.3796551651
C -0.5279691232 -0.2189103529 -0.2638974007
O 0.3963342142 0.2394317077 0.5039191306
O -2.6144184081 1.2360491014 -0.2701487378
O -3.3261882136 -2.0461350552 -0.7933650664
C -0.2179079295 -2.893352219 -0.5478409399
H 0.6316616816 -2.8850045397 0.1426897234
H -0.8724321009 -3.7372171782 -0.3098010243
H 0.1827384427 -3.0535362131 -1.5583932066
C -4.723994663 -1.5328117767 -0.8574077125
C -5.5244150842 -2.8095858987 -1.1133438684
H -5.3801663921 -3.5272693856 -0.2996405067
H -6.5906477944 -2.5719223189 -1.1808421311
H -5.2139887268 -3.2804157816 -2.0513997298
C -4.8477944883 -0.5504674704 -2.0234059983
H -4.4926215575 -1.0079037024 -2.9526864491
H -5.900678091 -0.279499631 -2.157625558
H -4.2875939422 0.3687416776 -1.8351838413
C -5.0894928754 -0.9015297185 0.4870441628
H -4.521381439 0.0144312743 0.6679742273
H -6.1534418437 -0.6409122276 0.4878533637
H -4.9123365871 -1.6072390844 1.3054177133
C 0.1357063804 0.4937616734 -2.365406298
C -0.7972494161 1.3374374685 -3.010152799
C 0.7687745942 -0.4860164449 -3.1663773092
H -1.3311076144 2.0929799784 -2.4393748492
H 1.5036894911 -1.1584907196 -2.7274531531
C -1.0704291736 1.2227514062 -4.3757646798
C 0.4998211743 -0.6071632366 -4.5327958524
H -1.786831475 1.8934149293 -4.8443544144
H 1.0111609492 -1.3639548067 -5.1229571359
C -0.4238226222 0.2472007129 -5.1388771333
H -0.6361449054 0.155819819 -6.2010082757

Frequency: -238.3097; 8.1330; 13.0785; 22.2672; 25.6637; 27.6343; 30.4157; 36.3730; 41.7802; 43.4539; 49.9938; 53.3965; 59.5827; 62.5053; 69.7750; 79.3598; 95.3151; 104.9805; 116.9377; 121.6223; 144.1127; 145.6414; 150.9999; 156.0428; 161.5013; 182.4950; 186.2247; 200.3104; 207.7000; 213.4905; 215.9356; 223.6456; 230.1968; 245.1267; 256.6506; 270.6277; 272.8449; 293.3402; 304.2465; 305.2939; 319.0744; 320.0714; 339.1015; 350.9898; 362.9780; 365.8678; 374.6611; 398.6152; 402.4859; 448.2871; 456.9319; 489.2087; 490.6465; 495.8873; 507.0832; 531.1256; 536.1987; 557.2817; 557.5353; 573.5933; 576.3836; 584.2613; 596.1681; 623.9478; 626.8674; 635.0977; 638.6543; 641.7843; 721.4012; 723.6239; 744.2729; 764.1323; 766.0459; 770.9015; 787.9221; 851.6450; 875.9636; 936.0373; 941.6993; 943.0912; 951.9996; 960.0958; 964.9428; 987.8695; 988.5532; 1006.0077; 1007.6828; 1012.5307; 1015.7821; 1054.4723; 1057.6777; 1066.9030; 1067.6148; 1069.5993; 1072.0725; 1087.4624; 1099.7084; 1194.3541; 1199.5173; 1200.8327; 1204.0395; 1224.7683; 1232.1418; 1241.1440; 1248.8248; 1271.6089; 1272.5177; 1286.8681; 1302.1040; 1304.7266; 1326.2210; 1333.8501; 1370.7471; 1394.9776; 1433.5061; 1434.7069; 1440.3282; 1461.0768; 1463.8363; 1494.1936; 1504.2555; 1506.2600; 1516.6376; 1517.8069; 1518.6823; 1530.2698; 1535.6277; 1536.4936; 1552.8965; 1614.9916; 1623.3562; 1683.8318; 1860.2725; 3044.5558; 3062.3639; 3062.7302; 3069.7882; 3108.6103; 3128.9056; 3130.6065; 3139.4404; 3142.7195; 3144.7264; 3156.1631; 3159.7136; 3168.8098; 3179.1541; 3188.7140; 3197.3858; 3207.0591.

Cartesian Forces: Max 0.000001787 RMS 0.000000627

Item	Value	Threshold	Converged?
Maximum Force	0.000001	0.000450	YES
RMS Force	0.000000	0.000300	YES
Maximum Displacement	0.000497	0.001800	YES
RMS Displacement	0.000114	0.001200	YES

FILE: Yb_Sq2_PhYbOTf2_TS_Neutral_Add56_B3LYP_M062x_Conf6.out

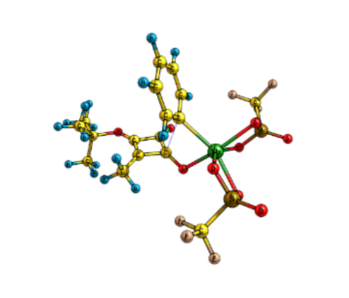

Conformer 12

E(RB3LYP/6-31G(d)/MWB59) = -2769.88731905
Convg = 0.8073D-08 -V/T = 2.0149

Temperature 195.150 Kelvin. Pressure 1.00000 Atm.
elec. and zero-point Energies= -2769.544149
elec. and thermal Energies= -2769.525512
elec. and thermal Enthalpies= -2769.524894
elec. and thermal Free Energies= -2769.585422

RB3LYP/6-31G(d)/MWB59/THF(IEFPCM) = -2769.91185091

RM06-2x/6-311+G(d,p)/MWB59/THF(IEFPCM) = -2769.71389412

Geometry at RB3LYP/6-31G(d)/MWB59
Charge = 0 Multiplicity = 1
Yb 1.5351446319 1.4913949021 -0.5199499848
O 1.6163335117 3.7655546891 -1.1319076634
S 1.0910800351 4.3189280912 0.1832648899
O 1.8663901782 5.3536816461 0.8398424059
O 0.7386118197 3.0771671197 0.9892816172
C -0.5609313304 5.0732004179 -0.2471215801
F -0.3703202694 6.0927200269 -1.0803886398
F -1.3358401941 4.1565018678 -0.8369566147
F -1.1482954179 5.4998985175 0.8664596576
O 3.6494707682 1.3312925528 0.5613548498
O 5.6806598509 1.374262349 -0.9720710775
O 3.3608759925 0.6471812019 -1.7162891928
S 4.4111717158 0.7689506036 -0.6192179926
C 4.7695161489 -1.0024472221 -0.1528975989
F 5.6200006019 -1.0290952597 0.8677498101
F 3.6312819257 -1.6156210272 0.2023262501
F 5.2970964838 -1.6339935521 -1.2003775066
C -0.5251815832 -2.0142195776 -0.7463265146
C -1.8975266802 -1.8675013205 -0.6095788608
C -1.7769491751 -0.4646593791 -0.0718695951
C -0.2641739236 -0.6479322203 -0.236831086
O 0.7148467104 -0.233996771 0.4852187034
O -2.5211082749 0.3806510932 0.3492044031
O -3.0401294994 -2.4970867698 -0.7593647478
C 0.4852263675 -3.0125204224 -1.1873022579
H 1.4400690168 -2.8090334498 -0.6914995457
H 0.1993585156 -4.0451068032 -0.9726503836
H 0.6556144414 -2.9291233305 -2.2695208138
C -3.2118032853 -3.894930258 -1.2169341024
C -4.7344751327 -4.0289353998 -1.2871353466
H -5.007032157 -5.04336073 -1.595324661
H -5.1831417983 -3.8285851732 -0.3096538869
H -5.1508902732 -3.3210279734 -2.0101167941
C -2.6311289217 -4.837890607 -0.1610464872
H -3.1066806064 -4.6631467802 0.8090495424
H -2.8165383878 -5.8773023556 -0.4519234092
H -1.5521268007 -4.7108935614 -0.0415739516
C -2.5883869748 -4.0707875556 -2.6031548132
H -1.4996603172 -3.9880603998 -2.5877080836
H -2.840186315 -5.0635463574 -2.9910580071
H -2.9847513885 -3.322812023 -3.297000571
C -0.0584449647 0.61706891 -2.1710292725
C -1.2170106163 1.3470054513 -2.5206068464
C 0.6344628872 -0.0215895687 -3.2269261593
H -1.804778604 1.8351917797 -1.7478789269
H 1.5388656296 -0.5919268272 -3.02149319
C -1.648107757 1.450661071 -3.8455597345
C 0.2091514846 0.0776407078 -4.5550983719
H -2.5394663431 2.0267575072 -4.0822494323
H 0.7716149301 -0.4146868196 -5.3450233768
C -0.9363751677 0.8136111464 -4.8653262545
H -1.2712580768 0.8931239857 -5.8964380801

Frequency: -234.0086; 8.4561; 13.0036; 20.9276; 25.6226; 27.6293; 29.3141; 37.2362; 43.4299; 44.2410; 51.5596; 55.6086; 59.0970; 60.1452; 73.9264; 83.1105; 97.4388; 106.9344; 117.3055; 137.2843; 143.2582; 151.7164; 159.0316; 161.2586; 163.1499; 183.0519; 184.7204; 186.4808; 208.1162; 213.4147; 215.4382; 223.3425; 229.7535; 237.6152; 245.1910; 258.4949; 264.1996; 285.5959; 303.7193; 305.1058; 318.8894; 320.0752; 338.6960; 345.3682; 362.8045; 366.4269; 379.8809; 400.6398; 402.6662; 448.9862; 453.5340; 482.5000; 489.3069; 490.6240; 517.0403; 531.3080; 536.4373; 557.2438; 557.5205; 573.9440; 576.8597; 584.0840; 596.5916; 623.8843; 627.0281; 635.0440; 640.0880; 649.3045; 723.2149; 732.2151; 761.8318; 764.0424; 766.2202; 773.4616; 781.4973; 838.9799; 875.9681; 933.3179; 935.0224; 942.5298; 952.0351; 960.9359; 975.4306; 980.6160; 988.2054; 1008.1539; 1008.4540; 1012.6026; 1015.2063; 1052.6922; 1056.6247; 1060.4191; 1064.3936; 1068.7612; 1071.5960; 1089.3088; 1101.4311; 1191.4970; 1194.2704; 1199.6722; 1200.6287; 1205.4803; 1224.6771; 1239.7406; 1249.6537; 1271.3375; 1272.6847; 1285.4172; 1294.5770; 1301.9862; 1326.2181; 1333.9103; 1370.4635; 1382.0207; 1432.3464; 1434.4187; 1439.0306; 1459.7288; 1463.6966; 1499.0284; 1502.3161; 1507.4072; 1515.2120; 1516.6754; 1519.0259; 1524.7201; 1529.9641; 1531.8014; 1549.1763; 1615.0617; 1623.4501; 1683.5955; 1888.6011; 3045.2310; 3065.6808; 3068.9324; 3075.3796; 3112.7077; 3134.7690; 3136.0165; 3144.2557; 3145.3442; 3146.1066; 3154.6976; 3166.0356; 3166.1625; 3180.0358; 3188.5472; 3200.3147; 3207.7072.

Cartesian Forces: Max 0.000000945 RMS 0.000000274

Item	Value	Threshold	Converged?
Maximum Force	0.000001	0.000450	YES
RMS Force	0.000000	0.000300	YES
Maximum Displacement	0.000069	0.001800	YES
RMS Displacement	0.000015	0.001200	YES

FILE: Yb_Sq2_PhYbOTf2_TS_Neutral_Add56_B3LYP_M062x_Conf6-.out

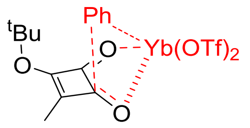

Butterfly Transition State for $PhYb(OTf)_2$ addition to C-2 carbonyl of 1b

Lowest energy conformation is: Conformer 7

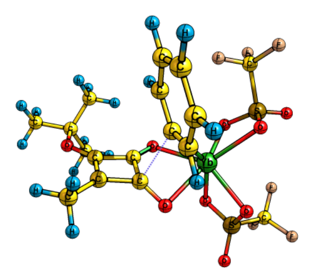

Conformer 1

E(RB3LYP/6-31G(d)/MWB59) = -2769.89113189
Convg = 0.8807D-08 -V/T = 2.0149

Temperature 195.150 Kelvin. Pressure 1.00000 Atm.
elec. and zero-point Energies= -2769.548301
elec. and thermal Energies= -2769.529713
elec. and thermal Enthalpies= -2769.529095
elec. and thermal Free Energies= -2769.589182

RB3LYP/6-31G(d)/MWB59/THF(IEFPCM) = -2769.91363467

RM06-2x/6-311+G(d,p)/MWB59/THF(IEFPCM) = -2769.72035151

Geometry at RB3LYP/6-31G(d)/MWB59
Charge = 0 Multiplicity = 1
Yb 1.2197758278 2.228481288 -0.5092597534
O 1.2805142557 2.8779576555 1.7728698391
S 0.7124415326 4.2663197361 1.5526297149
O -0.3916091148 4.6940562449 2.3920426419
O 0.5347530888 4.3708608789 0.0438254436
C 2.1436136859 5.4109002702 1.9044718703
F 2.4779679006 5.3115012901 3.1899893908
F 3.1863185806 5.0674273155 1.1444639995
F 1.7837107296 6.6623154417 1.6277811828
O 3.5655055307 2.0317520569 -0.4753540837
O 4.8075190634 4.012391466 -1.4797514634
O 2.4688762521 3.305638287 -2.1819813424
S 3.8464554321 2.940150039 -1.6599569029
C 4.5655181899 1.8049598072 -2.9550890116
F 5.7870546925 1.4333633746 -2.5763476124
F 3.7974791209 0.7188557594 -3.09058096
F 4.6294944352 2.4496552561 -4.1174538469
C -0.6731867358 -1.5281874243 0.1829523989
C -1.691700337 -0.7160981898 -0.3016296468
C 0.2716753502 -0.380007631 0.2477147919
C -0.8470752169 0.5039977456 -0.2254496387
O 1.442684659 -0.0937941132 0.4952086824
O -1.0393514119 1.7234585765 0.0333652007
O -0.6798039897 -2.8212709947 0.4143260669
C 0.4951491947 -3.5369000401 0.9937714066
C -3.0777851558 -0.9563561497 -0.7560057164
H -3.3882660716 -1.9896032409 -0.5806028747
H -3.1553560271 -0.7377855981 -1.8298900906
H -3.7697465503 -0.2763538998 -0.2443207382
C 1.6661467168 -3.4516310293 0.0135029036
H 2.0515070358 -2.4319890655 -0.0724213052
H 2.4851106907 -4.0843632644 0.3719878031
H 1.3668854338 -3.8112738158 -0.9764473154
C -0.0283546864 -4.9670516426 1.1181903444
H 0.7515017709 -5.6123530099 1.5342192011
H -0.8992999961 -5.0048895532 1.7797932874
H -0.3177645591 -5.3601932161 0.1386595297
C 0.8253892409 -2.939671594 2.3626268127
H -0.0588931624 -2.9390996102 3.008181312
H 1.5975920603 -3.5481994564 2.8450321026
H 1.2141828901 -1.9207616124 2.2830641468
C 0.1573439048 0.8011373199 -2.2802497996
C 0.8139279977 -0.1968532623 -3.0287894343
C -0.733859388 1.6422726088 -2.9743966536
H 1.4996977609 -0.8866282088 -2.5356928305
H -1.314731696 2.3841809783 -2.4214709075
C 0.6295361091 -0.3165182686 -4.4071535441
C -0.9149217497 1.5453629537 -4.3599512581
H 1.1580581494 -1.086103944 -4.9655633445
H -1.596687377 2.2197531347 -4.8727849144
C -0.2322011171 0.5615217884 -5.0755426819
H -0.3756859403 0.4695385625 -6.1491464028

Frequency: -246.3555; 7.9215; 16.4963; 25.5771; 28.4421; 31.2213; 32.7763; 34.1726; 37.3961; 44.7405; 51.4254; 56.8275; 61.8930; 67.9194; 72.8518; 81.6299; 84.0476; 107.0269; 112.7126; 128.9346; 134.4259; 141.6392; 159.3689; 166.2841; 174.8111; 179.8151; 192.4661; 199.7516; 209.8058; 211.9962; 219.7131; 222.4470; 242.5644; 250.2544; 263.9926; 270.1753; 276.0695; 295.4670; 304.2527; 308.1475; 318.0742; 321.5701; 326.7612; 344.9518; 362.4223; 365.0823; 372.3976; 397.9202; 400.6878; 419.1625; 451.2414; 489.0567; 490.8457; 492.0089; 504.5484; 531.3427; 533.5794; 538.1062; 557.6982; 558.2613; 575.8940; 577.7024; 600.3227; 626.0463; 626.6923; 633.5001; 638.0037; 644.1432; 714.2135; 721.0084; 732.6392; 753.2058; 765.2615; 765.4578; 791.7755; 862.0281; 875.2138; 935.0889; 936.1307; 942.2254; 952.4461; 962.2431; 979.2822; 980.4634; 987.6890; 1005.7269; 1006.9127; 1016.9634; 1030.7540; 1050.8491; 1053.5369; 1066.2159; 1066.6107; 1072.5488; 1075.1138; 1097.6401; 1116.6350; 1192.1240; 1200.8849; 1202.2579; 1203.2798; 1215.3699; 1220.4163; 1247.7248; 1253.2855; 1266.2095; 1267.6239; 1286.5617; 1303.3152; 1311.5045; 1323.8207; 1329.4448; 1361.2270; 1394.5167; 1434.7965; 1435.6879; 1442.1104; 1461.8456; 1465.8238; 1495.2572; 1500.5054; 1504.5297; 1514.9145; 1515.8247; 1516.6080; 1534.3251; 1535.2421; 1550.2851; 1597.4827; 1616.2305; 1621.9955; 1664.4125; 1789.9486; 3044.3280; 3063.3753; 3064.4200; 3071.0640; 3099.3034; 3127.6277; 3131.2544; 3133.6112; 3141.1808; 3142.2602; 3144.6604; 3149.2228; 3150.4838; 3153.6722; 3178.3734; 3187.3194; 3204.3045.

Cartesian Forces: Max 0.000000684 RMS 0.000000295

Item	Value	Threshold	Converged?
Maximum Force	0.000000	0.000450	YES
RMS Force	0.000000	0.000300	YES
Maximum Displacement	0.000049	0.001800	YES
RMS Displacement	0.000010	0.001200	YES

FILE: Yb_Sq2_PhYbOTf2_TS_Neutral_Add56_B3LYP_M062x_Conf3.out

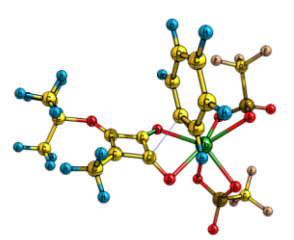

Conformer 2

E(RB3LYP/6-31G(d)/MWB59) = -2769.88316007
Convg = 0.6070D-08 -V/T = 2.0149

Temperature 195.150 Kelvin. Pressure 1.00000 Atm.
elec. and zero-point Energies= -2769.540315
elec. and thermal Energies= -2769.521733
elec. and thermal Enthalpies= -2769.521115
elec. and thermal Free Energies= -2769.581261

RB3LYP/6-31G(d)/MWB59/THF(IEFPCM) = -2769.90717774

RM06-2x/6-311+G(d,p)/MWB59/THF(IEFPCM) = -2769.71458188

Geometry at RB3LYP/6-31G(d)/MWB59
Charge = 0 Multiplicity = 1
Yb 1.1956425496 2.1973497608 -0.5438264151
O 1.1718355091 2.7755513911 1.7604838792
S 0.7429406593 4.2137175561 1.5511067623
O -0.3747617958 4.715092078 2.329819566
O 0.676740747 4.3753122082 0.0374627226
C 2.2383871109 5.2207932312 2.033071459
F 2.4795757197 5.0506161484 3.3320257096
F 3.2963009916 4.8182081298 1.3260470506
F 1.9992573971 6.5067764594 1.783991773
O 3.5267211208 1.9270362951 -0.3916998574
O 4.8938886201 3.8153146824 -1.4125943781
O 2.5543425801 3.1849123604 -2.1828313336
S 3.8933490703 2.7800701671 -1.5933016943
C 4.6045762875 1.5662941189 -2.8193774714
F 5.7913824874 1.1503184728 -2.382441084
F 3.7878849604 0.5144513331 -2.9453903427
F 4.7420597495 2.1633134235 -4.0009665639
C -0.9817864759 -1.4025092238 -0.0709405582
C -1.9460044248 -0.5008459295 -0.5209294417
C 0.0338758525 -0.3255792216 0.0675725147
C -1.0082448953 0.6523105844 -0.383482512
O 1.215378773 -0.1564482579 0.3559521818
O -1.1359928655 1.8732133958 -0.1015193281
O -0.7638187779 -2.6730935851 0.1826176454
C -1.7575147594 -3.7660250343 0.0504243027
C -3.3385265504 -0.455808111 -1.0297023767
H -3.9299565154 -1.3437105628 -0.801972427
H -3.3189345972 -0.3210401223 -2.1208017097
H -3.8439798966 0.4239146998 -0.6143926731
C -2.8992182979 -3.5289052593 1.0408998263
H -3.463774756 -2.6198556723 0.8188371919
H -3.5967297837 -4.3723094909 1.0053291075
H -2.5092946237 -3.4504147906 2.0604498944
C -0.9403388749 -4.9966082714 0.4480795595
H -1.5685001772 -5.8918723879 0.4026492379
H -0.0917490439 -5.1316844323 -0.2292171466
H -0.5565727905 -4.8912393872 1.4671390362
C -2.2154755501 -3.8664225066 -1.406017427
H -1.3533453282 -3.9912503595 -2.0685397466
H -2.8658477135 -4.7395358038 -1.5239334787
H -2.7771450554 -2.9882498621 -1.7326338637
C 0.0771203472 0.9154994137 -2.3983538164
C 0.7025596635 -0.1056206806 -3.1426133613
C -0.734003457 1.8236669327 -3.1049086399
H 1.3254796071 -0.845279989 -2.6386501192
H -1.2867239181 2.5898971226 -2.5566505267
C 0.5658836416 -0.1846796059 -4.5297497647
C -0.8659861418 1.7659082597 -4.4980805257
H 1.070552441 -0.9720495622 -5.0855453115
H -1.4841668929 2.4916802222 -5.0214248644
C -0.2152367605 0.7574804401 -5.2097324514
H -0.3207628969 0.6974816335 -6.2900326226

Frequency: -240.8343; 7.4785; 17.8898; 21.7039; 27.9399; 30.4802; 32.0091; 33.1380; 38.3462; 46.1990; 49.2899; 57.0479; 61.3127; 71.6899; 75.1491; 80.1402; 90.2357; 106.3735; 114.1003; 125.4582; 153.7421; 160.3052; 161.4713; 166.3240; 177.0117; 179.1550; 183.9276; 191.3588; 201.9864; 218.5250; 222.1213; 232.7383; 240.8431; 247.5024; 249.7373; 259.9512; 267.7239; 291.5538; 302.8197; 307.1119; 310.9843; 318.1482; 322.2973; 345.6600; 362.7566; 364.7345; 373.0734; 397.7887; 404.8350; 418.8703; 451.2237; 480.5359; 489.1524; 490.8128; 511.1430; 531.4307; 534.6785; 542.3074; 557.6302; 558.3681; 576.2596; 577.7608; 593.0574; 626.0473; 626.7647; 633.4073; 637.9715; 651.9616; 716.2249; 723.2265; 750.7607; 763.4561; 765.3216; 765.3703; 792.8309; 841.8133; 876.8468; 933.8419; 934.7817; 937.5646; 952.4255; 962.5157; 980.9887; 981.5994; 988.9635; 1005.4109; 1006.9552; 1016.9836; 1024.3070; 1048.1223; 1054.4985; 1060.8272; 1066.3995; 1068.5309; 1075.1469; 1097.4028; 1140.7013; 1181.0869; 1191.8861; 1201.2868; 1203.1499; 1204.8764; 1215.0640; 1247.0164; 1254.6266; 1265.5655; 1267.6078; 1285.8195; 1294.7911; 1311.5016; 1323.6673; 1329.8312; 1361.0016; 1383.7014; 1433.9735; 1435.1281; 1438.9933; 1460.5485; 1465.8999; 1495.3550; 1502.7566; 1505.4036; 1514.7705; 1516.2379; 1517.6345; 1528.2795; 1529.7005; 1549.2042; 1596.3423; 1616.2186; 1621.3576; 1672.0065; 1802.9030; 3041.1013; 3066.6679; 3069.8926; 3076.1052; 3100.7062; 3129.3503; 3136.1583; 3137.2489; 3144.5507; 3145.5794; 3146.6986; 3152.9443; 3158.3882; 3167.7004; 3177.6805; 3186.5066; 3203.5576.

Cartesian Forces: Max 0.000001004 RMS 0.000000351

Item	Value	Threshold	Converged?
Maximum Force	0.000000	0.000450	YES
RMS Force	0.000000	0.000300	YES
Maximum Displacement	0.000034	0.001800	YES
RMS Displacement	0.000006	0.001200	YES

FILE: Yb_Sq2_PhYbOTf2_TS_Neutral_Add56_B3LYP_M062x_Conf3-.out

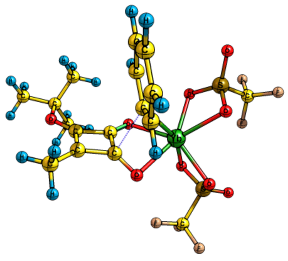

Conformer 3

E(RB3LYP/6-31G(d)/MWB59) = -2769.89203285
Convg = 0.5760D-08 -V/T = 2.0149

Temperature 195.150 Kelvin. Pressure 1.00000 Atm.
elec. and zero-point Energies= -2769.549200
elec. and thermal Energies= -2769.530571
elec. and thermal Enthalpies= -2769.529953
elec. and thermal Free Energies= -2769.590059

RB3LYP/6-31G(d)/MWB59/THF(IEFPCM) = -2769.91345432

RM06-2x/6-311+G(d,p)/MWB59/THF(IEFPCM) = -2769.72048692

Geometry at RB3LYP/6-31G(d)/MWB59
Charge = 0 Multiplicity = 1
Yb 1.0100292532 2.3321025888 -0.441786188
O 0.2219873681 4.4472420767 0.2009521457
S 0.7378069354 4.4256548925 1.6284430268
O 1.5103246899 5.5698254669 2.078132686
O 1.3603973028 3.0493050555 1.7883940577
C -0.7925789275 4.3796314863 2.6977612614
F -1.5160978132 5.4737600829 2.4661806029
F -1.5222529663 3.2975865009 2.424176407
F -0.4207274947 4.3485587802 3.9776014752
O 2.2062964244 3.5749785692 -2.0077678249
O 4.2408862557 2.451578447 -3.0464913655
O 3.2812828847 1.8212432624 -0.7769458999
S 3.5423436461 2.8693449809 -1.8450565101
C 4.6163811821 4.1351109338 -0.9919946919
F 4.8046253187 5.1708616666 -1.8055322381
F 4.0121777539 4.546348585 0.1254726278
F 5.78805857 3.5807307917 -0.6871420226
C -0.7429265301 -1.5221676567 0.1419807945
C -1.7593294316 -0.7530531116 -0.4142535744
C 0.1334862025 -0.3247882296 0.3021037484
C -0.9808297232 0.4993633359 -0.2648916389
O 1.2506700085 0.0258529543 0.6735518327
O -1.2344944979 1.7168725154 -0.0424232755
O -0.7023109073 -2.8153024529 0.3584412443
C 0.4730507609 -3.4849770025 0.993837547
C -3.0891552804 -1.0556577087 -0.9859768719
H -3.3601166562 -2.1052619135 -0.845743567
H -3.0842556104 -0.8292063218 -2.0610352093
H -3.8572384877 -0.4184552933 -0.5311152902
C 1.6965446481 -3.3084602709 0.093537118
H 2.0338190363 -2.2689150652 0.0622016665
H 2.5230063928 -3.9109013577 0.4853725385
H 1.4794772757 -3.6496073891 -0.9238679894
C 0.0210793427 -4.9434740147 1.0476167314
H 0.8064752219 -5.5579576568 1.4983810718
H -0.8876491365 -5.0462972044 1.6488115644
H -0.181796913 -5.3235101749 0.0415722421
C 0.68169596 -2.9093515212 2.395297166
H -0.2399640475 -2.9763304933 2.982591838
H 1.4556874191 -3.4875202182 2.9110119307
H 1.0163403591 -1.8688035736 2.3647692842
C 0.196044689 0.8224655782 -2.2568792151
C 1.0653106787 -0.0726979672 -2.9153115215
C -0.765391884 1.4830867976 -3.0467638635
H 1.8263939005 -0.6102890324 -2.3493991368
H -1.4872500704 2.1494451378 -2.5697064704
C 1.005130897 -0.2648818399 -4.296762235
C -0.829878411 1.3059512389 -4.4347003214
H 1.7016705201 -0.9426160511 -4.785065794
H -1.5729965235 1.8401955186 -5.0223655532
C 0.056628119 0.4277752188 -5.0584853837
H 0.0103362957 0.2788810584 -6.134291957

Frequency: -233.2996; 11.7723; 18.5452; 24.5593; 25.3912; 27.9011; 31.8499; 33.7005; 38.3191; 42.5135; 48.0734; 52.5249; 61.7378; 64.9335; 76.3625; 80.2964; 82.0162; 107.0306; 116.3807; 129.6011; 137.0116; 140.5669; 163.2958; 165.3840; 169.9800; 179.5583; 190.4361; 203.0720; 207.9781; 211.7287; 218.0099; 222.1029; 242.1655; 247.0091; 262.9492; 269.5318; 273.8227; 292.6035; 302.9285; 306.7264; 318.5430; 321.2631; 327.4287; 344.2506; 360.4845; 363.7450; 369.6878; 397.8583; 399.9012; 425.1242; 451.2001; 489.7990; 490.9281; 491.6920; 499.3694; 531.4493; 535.1186; 537.8387; 557.9390; 558.6423; 575.4869; 579.5067; 599.7894; 626.1481; 626.6395; 632.2114; 638.0335; 643.2420; 714.4366; 721.7009; 731.5515; 750.3665; 765.4762; 765.6596; 791.8402; 859.9520; 875.7684; 935.4979; 936.8201; 942.3146; 953.8246; 962.6519; 978.9330; 981.9722; 987.9857; 1005.2045; 1008.2474; 1016.7025; 1032.0497; 1051.6363; 1053.8305; 1066.3323; 1067.3416; 1072.7838; 1074.8289; 1096.7528; 1121.7075; 1192.3119; 1199.7110; 1200.8665; 1202.5037; 1215.9011; 1222.1872; 1253.7650; 1255.7936; 1262.6879; 1269.0824; 1287.1220; 1303.4872; 1309.7004; 1321.9396; 1328.5198; 1361.8672; 1396.5502; 1434.8189; 1436.1593; 1443.3732; 1461.9993; 1465.3478; 1496.1607; 1501.2465; 1504.3650; 1514.2531; 1515.8810; 1516.1341; 1534.2885; 1534.9643; 1550.0733; 1594.0632; 1615.6416; 1620.9701; 1669.2106; 1796.9425; 3044.3529; 3063.9625; 3064.3438; 3071.2100; 3099.5054; 3129.9520; 3132.0184; 3133.6483; 3141.2397; 3144.8809; 3148.5418; 3149.1602; 3150.7284; 3154.3474; 3178.8727; 3187.5253; 3204.2513.

Cartesian Forces: Max 0.000006635 RMS 0.000000947

Item	Value	Threshold	Converged?
Maximum Force	0.000004	0.000450	YES
RMS Force	0.000000	0.000300	YES
Maximum Displacement	0.000515	0.001800	YES
RMS Displacement	0.000089	0.001200	YES

FILE: Yb_Sq2_PhYbOTf2_TS_Neutral_Add56_B3LYP_M062x_Conf4.out

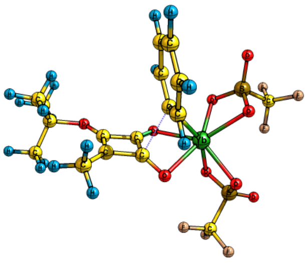

Conformer 4

E(RB3LYP/6-31G(d)/MWB59) = -2769.88367756
Convg = 0.8054D-08 -V/T = 2.0149

Temperature 195.150 Kelvin. Pressure 1.00000 Atm.
elec. and zero-point Energies= -2769.540865
elec. and thermal Energies= -2769.522233
elec. and thermal Enthalpies= -2769.521615
elec. and thermal Free Energies= -2769.581795

RB3LYP/6-31G(d)/MWB59/THF(IEFPCM) = -2769.90757311

RM06-2x/6-311+G(d,p)/MWB59/THF(IEFPCM) = -2769.71526349

Geometry at RB3LYP/6-31G(d)/MWB59
Charge = 0 Multiplicity = 1
Yb 0.9771263927 2.2970535675 -0.4186189582
O 0.3389545501 4.4605666574 0.2402373485
S 0.7806021591 4.3631519132 1.6889215349
O 1.6226739826 5.4275413636 2.2049103391
O 1.2768566323 2.9357348749 1.8409136808
C -0.7980761555 4.4227012087 2.6848222525
F -1.4212656824 5.5747251506 2.4410544868
F -1.5977131511 3.4069603968 2.3566681747
F -0.4924038065 4.3420347561 3.9797683332
O 2.3001970979 3.4788341245 -1.9257920436
O 4.291450802 2.2416293117 -2.9175506647
O 3.2173691384 1.6413012391 -0.6906906366
S 3.5820007751 2.6850114944 -1.731866308
C 4.7057561344 3.8659657107 -0.8231138416
F 4.9835791857 4.9015473349 -1.611125978
F 4.093135035 4.2958349059 0.2828515673
F 5.8303662311 3.2344655785 -0.4931070786
C -1.110253173 -1.3791385511 -0.009562284
C -2.0561014271 -0.5198259126 -0.5715467132
C -0.1536082737 -0.2582443979 0.2158010895
C -1.1748902596 0.6622683716 -0.367880702
O 0.9718085242 -0.0274991182 0.6422415703
O -1.3499854592 1.8931178697 -0.1534489733
O -0.8750051834 -2.6349201873 0.287997246
C -1.7941029059 -3.7745178526 0.0439628427
C -3.3885689932 -0.5382974503 -1.2240613773
H -3.9689060399 -1.4423521045 -1.0344232925
H -3.2576953125 -0.429400172 -2.3103449512
H -3.9670143625 0.3304995806 -0.8889238949
C -3.049611182 -3.5957921004 0.8996307967
H -3.6264853572 -2.7119656545 0.6156656088
H -3.6991535454 -4.4698497777 0.784699775
H -2.7811478509 -3.5059907949 1.956798574
C -0.9682391521 -4.9660491644 0.5319205298
H -1.5441921933 -5.8899123908 0.4183091093
H -0.0442440729 -5.0580050265 -0.0466290553
H -0.7055233468 -4.8464872458 1.5871823865
C -2.0794865595 -3.8886084358 -1.4546733515
H -1.1431243817 -3.9615777902 -2.016395606
H -2.6636712006 -4.795049393 -1.6451853126
H -2.6486106369 -3.0402439731 -1.8414925812
C 0.1243926551 0.8786312424 -2.2963347852
C 0.9509463264 -0.0931951461 -2.8986429208
C -0.7343656784 1.6084161338 -3.1409188461
H 1.6311362858 -0.6861025287 -2.2868340306
H -1.4206567503 2.33938521 -2.708017836
C 0.9505400147 -0.2938260702 -4.2805481946
C -0.7374238642 1.4222918556 -4.5290253003
H 1.6146744156 -1.0312992162 -4.7256277751
H -1.3988770927 2.0110875604 -5.1606616724
C 0.1059477225 0.466899808 -5.0974821006
H 0.1068156696 0.3118073456 -6.1734691297

Frequency: -226.5847; 11.7347; 18.7033; 19.5689; 25.0496; 28.4698; 31.5902; 34.2694; 38.9276; 43.3732; 46.2403; 52.3762; 61.8408; 66.6595; 77.5469; 82.0631; 90.1359; 106.3099; 114.1534; 127.5926; 152.7907; 157.9128; 163.8593; 167.0213; 170.4311; 178.3790; 182.2759; 192.3066; 204.4790; 216.7654; 221.2163; 230.8514; 241.1178; 243.3560; 249.0694; 258.9438; 263.9423; 288.5323; 302.6235; 306.8580; 309.4845; 318.4914; 321.3554; 345.0239; 360.5598; 364.5432; 371.3911; 396.7362; 403.3756; 424.0260; 450.9727; 480.6071; 489.8668; 491.7243; 508.3188; 531.5166; 535.3227; 542.3731; 557.8827; 558.5939; 575.5328; 579.5982; 592.1944; 626.2230; 626.6417; 632.4457; 638.0900; 651.8203; 716.5858; 723.0954; 749.1297; 762.7628; 765.4633; 765.6734; 792.5750; 840.3349; 876.7019; 934.0779; 935.0502; 937.2640; 954.0733; 963.1097; 981.1491; 982.3905; 988.8279; 1004.4415; 1008.4418; 1016.6677; 1024.6165; 1048.4978; 1054.2134; 1060.6226; 1066.9691; 1068.7567; 1075.3593; 1096.3209; 1144.5792; 1183.6185; 1192.0065; 1199.8097; 1202.9724; 1203.9760; 1215.5573; 1253.5348; 1255.1568; 1262.1270; 1269.0141; 1286.0044; 1294.4662; 1309.7327; 1322.0531; 1328.8177; 1361.5640; 1386.0749; 1434.0486; 1435.4721; 1439.7449; 1460.7717; 1465.2716; 1495.9996; 1502.6339; 1505.6341; 1514.1221; 1516.0635; 1517.1745; 1528.3071; 1529.6138; 1548.9524; 1592.6200; 1615.5581; 1620.2857; 1675.7117; 1811.7868; 3041.1697; 3066.8463; 3069.9610; 3076.2946; 3100.8886; 3129.5075; 3136.7085; 3137.3123; 3146.0111; 3146.8331; 3150.5559; 3152.0994; 3159.0330; 3167.7155; 3178.0125; 3186.8736; 3203.4511.

Cartesian Forces: Max 0.000004567 RMS 0.000000740

Item	Value	Threshold	Converged?
Maximum Force	0.000002	0.000450	YES
RMS Force	0.000000	0.000300	YES
Maximum Displacement	0.000168	0.001800	YES
RMS Displacement	0.000032	0.001200	YES

FILE: Yb_Sq2_PhYbOTf2_TS_Neutral_Add56_B3LYP_M062x_Conf4-.out

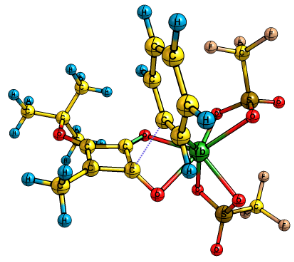

Conformer 5

E(RB3LYP/6-31G(d)/MWB59) = -2769.89113189
Convg = 0.8807D-08 -V/T = 2.0149

Temperature 195.150 Kelvin. Pressure 1.00000 Atm.
elec. and zero-point Energies= -2769.548301
elec. and thermal Energies= -2769.529713
elec. and thermal Enthalpies= -2769.529095
elec. and thermal Free Energies= -2769.589182

RB3LYP/6-31G(d)/MWB59/THF(IEFPCM) = -2769.91363467

RM06-2x/6-311+G(d,p)/MWB59/THF(IEFPCM) = -2769.72035151

Geometry at RB3LYP/6-31G(d)/MWB59
Charge = 0 Multiplicity = 1
Yb 1.2197758278 2.228481288 -0.5092597534
O 1.2805142557 2.8779576555 1.7728698391
S 0.7124415326 4.2663197361 1.5526297149
O -0.3916091148 4.6940562449 2.3920426419
O 0.5347530888 4.3708608789 0.0438254436
C 2.1436136859 5.4109002702 1.9044718703
F 2.4779679006 5.3115012901 3.1899893908
F 3.1863185806 5.0674273155 1.1444639995
F 1.7837107296 6.6623154417 1.6277811828
O 3.5655055307 2.0317520569 -0.4753540837
O 4.8075190634 4.012391466 -1.4797514634
O 2.4688762521 3.305638287 -2.1819813424
S 3.8464554321 2.940150039 -1.6599569029
C 4.5655181899 1.8049598072 -2.9550890116
F 5.7870546925 1.4333633746 -2.5763476124
F 3.7974791209 0.7188557594 -3.09058096
F 4.6294944352 2.4496552561 -4.1174538469
C -0.6731867358 -1.5281874243 0.1829523989
C -1.691700337 -0.7160981898 -0.3016296468
C 0.2716753502 -0.380007631 0.2477147919
C -0.8470752169 0.5039977456 -0.2254496387
O 1.442684659 -0.0937941132 0.4952086824
O -1.0393514119 1.7234585765 0.0333652007
O -0.6798039897 -2.8212709947 0.4143260669
C 0.4951491947 -3.5369000401 0.9937714066
C -3.0777851558 -0.9563561497 -0.7560057164
H -3.3882660716 -1.9896032409 -0.5806028747
H -3.1553560271 -0.7377855981 -1.8298900906
H -3.7697465503 -0.2763538998 -0.2443207382
C 1.6661467168 -3.4516310293 0.0135029036
H 2.0515070358 -2.4319890655 -0.0724213052
H 2.4851106907 -4.0843632644 0.3719878031
H 1.3668854338 -3.8112738158 -0.9764473154
C -0.0283546864 -4.9670516426 1.1181903444
H 0.7515017709 -5.6123530099 1.5342192011
H -0.8992999961 -5.0048895532 1.7797932874
H -0.3177645591 -5.3601932161 0.1386595297
C 0.8253892409 -2.939671594 2.3626268127
H -0.0588931624 -2.9390996102 3.008181312
H 1.5975920603 -3.5481994564 2.8450321026
H 1.2141828901 -1.9207616124 2.2830641468
C 0.1573439048 0.8011373199 -2.2802497996
C 0.8139279977 -0.1968532623 -3.0287894343
C -0.733859388 1.6422726088 -2.9743966536
H 1.4996977609 -0.8866282088 -2.5356928305
H -1.314731696 2.3841809783 -2.4214709075
C 0.6295361091 -0.3165182686 -4.4071535441
C -0.9149217497 1.5453629537 -4.3599512581
H 1.1580581494 -1.086103944 -4.9655633445
H -1.596687377 2.2197531347 -4.8727849144
C -0.2322011171 0.5615217884 -5.0755426819
H -0.3756859403 0.4695385625 -6.1491464028

Frequency: -246.3555; 7.9215; 16.4963; 25.5771; 28.4421; 31.2213; 32.7763; 34.1726; 37.3961; 44.7405; 51.4254; 56.8275; 61.8930; 67.9194; 72.8518; 81.6299; 84.0476; 107.0269; 112.7126; 128.9346; 134.4259; 141.6392; 159.3689; 166.2841; 174.8111; 179.8151; 192.4661; 199.7516; 209.8058; 211.9962; 219.7131; 222.4470; 242.5644; 250.2544; 263.9926; 270.1753; 276.0695; 295.4670; 304.2527; 308.1475; 318.0742; 321.5701; 326.7612; 344.9518; 362.4223; 365.0823; 372.3976; 397.9202; 400.6878; 419.1625; 451.2414; 489.0567; 490.8457; 492.0089; 504.5484; 531.3427; 533.5794; 538.1062; 557.6982; 558.2613; 575.8940; 577.7024; 600.3227; 626.0463; 626.6923; 633.5001; 638.0037; 644.1432; 714.2135; 721.0084; 732.6392; 753.2058; 765.2615; 765.4578; 791.7755; 862.0281; 875.2138; 935.0889; 936.1307; 942.2254; 952.4461; 962.2431; 979.2822; 980.4634; 987.6890; 1005.7269; 1006.9127; 1016.9634; 1030.7540; 1050.8491; 1053.5369; 1066.2159; 1066.6107; 1072.5488; 1075.1138; 1097.6401; 1116.6350; 1192.1240; 1200.8849; 1202.2579; 1203.2798; 1215.3699; 1220.4163; 1247.7248; 1253.2855; 1266.2095; 1267.6239; 1286.5617; 1303.3152; 1311.5045; 1323.8207; 1329.4448; 1361.2270; 1394.5167; 1434.7965; 1435.6879; 1442.1104; 1461.8456; 1465.8238; 1495.2572; 1500.5054; 1504.5297; 1514.9145; 1515.8247; 1516.6080; 1534.3251; 1535.2421; 1550.2851; 1597.4827; 1616.2305; 1621.9955; 1664.4125; 1789.9486; 3044.3280; 3063.3753; 3064.4200; 3071.0640; 3099.3034; 3127.6277; 3131.2544; 3133.6112; 3141.1808; 3142.2602; 3144.6604; 3149.2228; 3150.4838; 3153.6722; 3178.3734; 3187.3194; 3204.3045.

Cartesian Forces: Max 0.000000684 RMS 0.000000295

Item	Value	Threshold	Converged?
Maximum Force	0.000000	0.000450	YES
RMS Force	0.000000	0.000300	YES
Maximum Displacement	0.000049	0.001800	YES
RMS Displacement	0.000010	0.001200	YES

FILE: Yb_Sq2_PhYbOTf2_TS_Neutral_Add56_B3LYP_M062x_Conf3.out

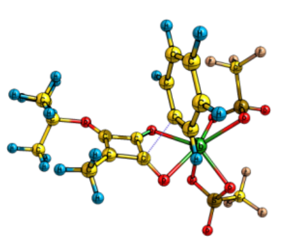

Conformer 6

E(RB3LYP/6-31G(d)/MWB59) = -2769.88316007
Convg = 0.6070D-08 -V/T = 2.0149

Temperature 195.150 Kelvin. Pressure 1.00000 Atm.
elec. and zero-point Energies= -2769.540315
elec. and thermal Energies= -2769.521733
elec. and thermal Enthalpies= -2769.521115
elec. and thermal Free Energies= -2769.581261

RB3LYP/6-31G(d)/MWB59/THF(IEFPCM) = -2769.90717774

RM06-2x/6-311+G(d,p)/MWB59/THF(IEFPCM) = -2769.71458188

Geometry at RB3LYP/6-31G(d)/MWB59
Charge = 0 Multiplicity = 1
Yb 1.1956425496 2.1973497608 -0.5438264151
O 1.1718355091 2.7755513911 1.7604838792
S 0.7429406593 4.2137175561 1.5511067623
O -0.3747617958 4.715092078 2.329819566
O 0.676740747 4.3753122082 0.0374627226
C 2.2383871109 5.2207932312 2.033071459
F 2.4795757197 5.0506161484 3.3320257096
F 3.2963009916 4.8182081298 1.3260470506
F 1.9992573971 6.5067764594 1.783991773
O 3.5267211208 1.9270362951 -0.3916998574
O 4.8938886201 3.8153146824 -1.4125943781
O 2.5543425801 3.1849123604 -2.1828313336
S 3.8933490703 2.7800701671 -1.5933016943
C 4.6045762875 1.5662941189 -2.8193774714
F 5.7913824874 1.1503184728 -2.382441084
F 3.7878849604 0.5144513331 -2.9453903427
F 4.7420597495 2.1633134235 -4.0009665639
C -0.9817864759 -1.4025092238 -0.0709405582
C -1.9460044248 -0.5008459295 -0.5209294417
C 0.0338758525 -0.3255792216 0.0675725147
C -1.0082448953 0.6523105844 -0.383482512
O 1.215378773 -0.1564482579 0.3559521818
O -1.1359928655 1.8732133958 -0.1015193281
O -0.7638187779 -2.6730935851 0.1826176454
C -1.7575147594 -3.7660250343 0.0504243027
C -3.3385265504 -0.455808111 -1.0297023767
H -3.9299565154 -1.3437105628 -0.801972427
H -3.3189345972 -0.3210401223 -2.1208017097
H -3.8439798966 0.4239146998 -0.6143926731
C -2.8992182979 -3.5289052593 1.0408998263
H -3.463774756 -2.6198556723 0.8188371919
H -3.5967297837 -4.3723094909 1.0053291075
H -2.5092946237 -3.4504147906 2.0604498944
C -0.9403388749 -4.9966082714 0.4480795595
H -1.5685001772 -5.8918723879 0.4026492379
H -0.0917490439 -5.1316844323 -0.2292171466
H -0.5565727905 -4.8912393872 1.4671390362
C -2.2154755501 -3.8664225066 -1.406017427
H -1.3533453282 -3.9912503595 -2.0685397466
H -2.8658477135 -4.7395358038 -1.5239334787
H -2.7771450554 -2.9882498621 -1.7326338637
C 0.0771203472 0.9154994137 -2.3983538164
C 0.7025596635 -0.1056206806 -3.1426133613
C -0.734003457 1.8236669327 -3.1049086399
H 1.3254796071 -0.845279989 -2.6386501192
H -1.2867239181 2.5898971226 -2.5566505267
C 0.5658836416 -0.1846796059 -4.5297497647
C -0.8659861418 1.7659082597 -4.4980805257
H 1.070552441 -0.9720495622 -5.0855453115
H -1.4841668929 2.4916802222 -5.0214248644
C -0.2152367605 0.7574804401 -5.2097324514
H -0.3207628969 0.6974816335 -6.2900326226

Frequency: -240.8343; 7.4785; 17.8898; 21.7039; 27.9399; 30.4802; 32.0091; 33.1380; 38.3462; 46.1990; 49.2899; 57.0479; 61.3127; 71.6899; 75.1491; 80.1402; 90.2357; 106.3735; 114.1003; 125.4582; 153.7421; 160.3052; 161.4713; 166.3240; 177.0117; 179.1550; 183.9276; 191.3588; 201.9864; 218.5250; 222.1213; 232.7383; 240.8431; 247.5024; 249.7373; 259.9512; 267.7239; 291.5538; 302.8197; 307.1119; 310.9843; 318.1482; 322.2973; 345.6600; 362.7566; 364.7345; 373.0734; 397.7887; 404.8350; 418.8703; 451.2237; 480.5359; 489.1524; 490.8128; 511.1430; 531.4307; 534.6785; 542.3074; 557.6302; 558.3681; 576.2596; 577.7608; 593.0574; 626.0473; 626.7647; 633.4073; 637.9715; 651.9616; 716.2249; 723.2265; 750.7607; 763.4561; 765.3216; 765.3703; 792.8309; 841.8133; 876.8468; 933.8419; 934.7817; 937.5646; 952.4255; 962.5157; 980.9887; 981.5994; 988.9635; 1005.4109; 1006.9552; 1016.9836; 1024.3070; 1048.1223; 1054.4985; 1060.8272; 1066.3995; 1068.5309; 1075.1469; 1097.4028; 1140.7013; 1181.0869; 1191.8861; 1201.2868; 1203.1499; 1204.8764; 1215.0640; 1247.0164; 1254.6266; 1265.5655; 1267.6078; 1285.8195; 1294.7911; 1311.5016; 1323.6673; 1329.8312; 1361.0016; 1383.7014; 1433.9735; 1435.1281; 1438.9933; 1460.5485; 1465.8999; 1495.3550; 1502.7566; 1505.4036; 1514.7705; 1516.2379; 1517.6345; 1528.2795; 1529.7005; 1549.2042; 1596.3423; 1616.2186; 1621.3576; 1672.0065; 1802.9030; 3041.1013; 3066.6679; 3069.8926; 3076.1052; 3100.7062; 3129.3503; 3136.1583; 3137.2489; 3144.5507; 3145.5794; 3146.6986; 3152.9443; 3158.3882; 3167.7004; 3177.6805; 3186.5066; 3203.5576.

Cartesian Forces: Max 0.000001004 RMS 0.000000351

Item	Value	Threshold	Converged?
Maximum Force	0.000000	0.000450	YES
RMS Force	0.000000	0.000300	YES
Maximum Displacement	0.000034	0.001800	YES
RMS Displacement	0.000006	0.001200	YES

FILE: Yb_Sq2_PhYbOTf2_TS_Neutral_Add56_B3LYP_M062x_Conf3-.out

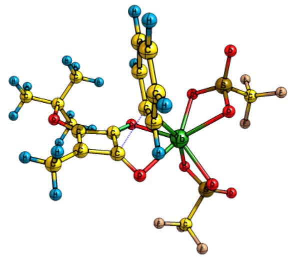

Conformer 7

E(RB3LYP/6-31G(d)/MWB59) = -2769.89203285
Convg = 0.5760D-08 -V/T = 2.0149

Temperature 195.150 Kelvin. Pressure 1.00000 Atm.
elec. and zero-point Energies= -2769.549200
elec. and thermal Energies= -2769.530571
elec. and thermal Enthalpies= -2769.529953
elec. and thermal Free Energies= -2769.590059

RB3LYP/6-31G(d)/MWB59/THF(IEFPCM) = -2769.91345432

RM06-2x/6-311+G(d,p)/MWB59/THF(IEFPCM) = -2769.72048692

Geometry at RB3LYP/6-31G(d)/MWB59
Charge = 0 Multiplicity = 1
Yb 1.0100292532 2.3321025888 -0.441786188
O 0.2219873681 4.4472420767 0.2009521457
S 0.7378069354 4.4256548925 1.6284430268
O 1.5103246899 5.5698254669 2.078132686
O 1.3603973028 3.0493050555 1.7883940577
C -0.7925789275 4.3796314863 2.6977612614
F -1.5160978132 5.4737600829 2.4661806029
F -1.5222529663 3.2975865009 2.424176407
F -0.4207274947 4.3485587802 3.9776014752
O 2.2062964244 3.5749785692 -2.0077678249
O 4.2408862557 2.451578447 -3.0464913655
O 3.2812828847 1.8212432624 -0.7769458999
S 3.5423436461 2.8693449809 -1.8450565101
C 4.6163811821 4.1351109338 -0.9919946919
F 4.8046253187 5.1708616666 -1.8055322381
F 4.0121777539 4.546348585 0.1254726278
F 5.78805857 3.5807307917 -0.6871420226
C -0.7429265301 -1.5221676567 0.1419807945
C -1.7593294316 -0.7530531116 -0.4142535744
C 0.1334862025 -0.3247882296 0.3021037484
C -0.9808297232 0.4993633359 -0.2648916389
O 1.2506700085 0.0258529543 0.6735518327
O -1.2344944979 1.7168725154 -0.0424232755
O -0.7023109073 -2.8153024529 0.3584412443
C 0.4730507609 -3.4849770025 0.993837547
C -3.0891552804 -1.0556577087 -0.9859768719
H -3.3601166562 -2.1052619135 -0.845743567
H -3.0842556104 -0.8292063218 -2.0610352093
H -3.8572384877 -0.4184552933 -0.5311152902
C 1.6965446481 -3.3084602709 0.093537118
H 2.0338190363 -2.2689150652 0.0622016665
H 2.5230063928 -3.9109013577 0.4853725385
H 1.4794772757 -3.6496073891 -0.9238679894
C 0.0210793427 -4.9434740147 1.0476167314
H 0.8064752219 -5.5579576568 1.4983810718
H -0.8876491365 -5.0462972044 1.6488115644
H -0.181796913 -5.3235101749 0.0415722421
C 0.68169596 -2.9093515212 2.395297166
H -0.2399640475 -2.9763304933 2.982591838
H 1.4556874191 -3.4875202182 2.9110119307
H 1.0163403591 -1.8688035736 2.3647692842
C 0.196044689 0.8224655782 -2.2568792151
C 1.0653106787 -0.0726979672 -2.9153115215
C -0.765391884 1.4830867976 -3.0467638635
H 1.8263939005 -0.6102890324 -2.3493991368
H -1.4872500704 2.1494451378 -2.5697064704
C 1.005130897 -0.2648818399 -4.296762235
C -0.829878411 1.3059512389 -4.4347003214
H 1.7016705201 -0.9426160511 -4.785065794
H -1.5729965235 1.8401955186 -5.0223655532
C 0.056628119 0.4277752188 -5.0584853837
H 0.0103362957 0.2788810584 -6.134291957

Frequency: -233.2996; 11.7723; 18.5452; 24.5593; 25.3912; 27.9011; 31.8499; 33.7005; 38.3191; 42.5135; 48.0734; 52.5249; 61.7378; 64.9335; 76.3625; 80.2964; 82.0162; 107.0306; 116.3807; 129.6011; 137.0116; 140.5669; 163.2958; 165.3840; 169.9800; 179.5583; 190.4361; 203.0720; 207.9781; 211.7287; 218.0099; 222.1029; 242.1655; 247.0091; 262.9492; 269.5318; 273.8227; 292.6035; 302.9285; 306.7264; 318.5430; 321.2631; 327.4287; 344.2506; 360.4845; 363.7450; 369.6878; 397.8583; 399.9012; 425.1242; 451.2001; 489.7990; 490.9281; 491.6920; 499.3694; 531.4493; 535.1186; 537.8387; 557.9390; 558.6423; 575.4869; 579.5067; 599.7894; 626.1481; 626.6395; 632.2114; 638.0335; 643.2420; 714.4366; 721.7009; 731.5515; 750.3665; 765.4762; 765.6596; 791.8402; 859.9520; 875.7684; 935.4979; 936.8201; 942.3146; 953.8246; 962.6519; 978.9330; 981.9722; 987.9857; 1005.2045; 1008.2474; 1016.7025; 1032.0497; 1051.6363; 1053.8305; 1066.3323; 1067.3416; 1072.7838; 1074.8289; 1096.7528; 1121.7075; 1192.3119; 1199.7110; 1200.8665; 1202.5037; 1215.9011; 1222.1872; 1253.7650; 1255.7936; 1262.6879; 1269.0824; 1287.1220; 1303.4872; 1309.7004; 1321.9396; 1328.5198; 1361.8672; 1396.5502; 1434.8189; 1436.1593; 1443.3732; 1461.9993; 1465.3478; 1496.1607; 1501.2465; 1504.3650; 1514.2531; 1515.8810; 1516.1341; 1534.2885; 1534.9643; 1550.0733; 1594.0632; 1615.6416; 1620.9701; 1669.2106; 1796.9425; 3044.3529; 3063.9625; 3064.3438; 3071.2100; 3099.5054; 3129.9520; 3132.0184; 3133.6483; 3141.2397; 3144.8809; 3148.5418; 3149.1602; 3150.7284; 3154.3474; 3178.8727; 3187.5253; 3204.2513.

Cartesian Forces: Max 0.000006635 RMS 0.000000947

Item	Value	Threshold	Converged?
Maximum Force	0.000004	0.000450	YES
RMS Force	0.000000	0.000300	YES
Maximum Displacement	0.000515	0.001800	YES
RMS Displacement	0.000089	0.001200	YES

FILE: Yb_Sq2_PhYbOTf2_TS_Neutral_Add56_B3LYP_M062x_Conf4.out

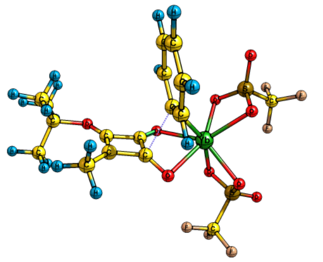

Conformer 8

E(RB3LYP/6-31G(d)/MWB59) = -2769.88367756
Convg = 0.8054D-08 -V/T = 2.0149

Temperature 195.150 Kelvin. Pressure 1.00000 Atm.
elec. and zero-point Energies= -2769.540865
elec. and thermal Energies= -2769.522233
elec. and thermal Enthalpies= -2769.521615
elec. and thermal Free Energies= -2769.581795

RB3LYP/6-31G(d)/MWB59/THF(IEFPCM) = -2769.90757311

RM06-2x/6-311+G(d,p)/MWB59/THF(IEFPCM) = -2769.71526349

Geometry at RB3LYP/6-31G(d)/MWB59
Charge = 0 Multiplicity = 1
Yb 0.9771263927 2.2970535675 -0.4186189582
O 0.3389545501 4.4605666574 0.2402373485
S 0.7806021591 4.3631519132 1.6889215349
O 1.6226739826 5.4275413636 2.2049103391
O 1.2768566323 2.9357348749 1.8409136808
C -0.7980761555 4.4227012087 2.6848222525
F -1.4212656824 5.5747251506 2.4410544868
F -1.5977131511 3.4069603968 2.3566681747
F -0.4924038065 4.3420347561 3.9797683332
O 2.3001970979 3.4788341245 -1.9257920436
O 4.291450802 2.2416293117 -2.9175506647
O 3.2173691384 1.6413012391 -0.6906906366
S 3.5820007751 2.6850114944 -1.731866308
C 4.7057561344 3.8659657107 -0.8231138416
F 4.9835791857 4.9015473349 -1.611125978
F 4.093135035 4.2958349059 0.2828515673
F 5.8303662311 3.2344655785 -0.4931070786
C -1.110253173 -1.3791385511 -0.009562284
C -2.0561014271 -0.5198259126 -0.5715467132
C -0.1536082737 -0.2582443979 0.2158010895
C -1.1748902596 0.6622683716 -0.367880702
O 0.9718085242 -0.0274991182 0.6422415703
O -1.3499854592 1.8931178697 -0.1534489733
O -0.8750051834 -2.6349201873 0.287997246
C -1.7941029059 -3.7745178526 0.0439628427
C -3.3885689932 -0.5382974503 -1.2240613773
H -3.9689060399 -1.4423521045 -1.0344232925
H -3.2576953125 -0.429400172 -2.3103449512
H -3.9670143625 0.3304995806 -0.8889238949
C -3.049611182 -3.5957921004 0.8996307967
H -3.6264853572 -2.7119656545 0.6156656088
H -3.6991535454 -4.4698497777 0.784699775
H -2.7811478509 -3.5059907949 1.956798574
C -0.9682391521 -4.9660491644 0.5319205298
H -1.5441921933 -5.8899123908 0.4183091093
H -0.0442440729 -5.0580050265 -0.0466290553
H -0.7055233468 -4.8464872458 1.5871823865
C -2.0794865595 -3.8886084358 -1.4546733515
H -1.1431243817 -3.9615777902 -2.016395606
H -2.6636712006 -4.795049393 -1.6451853126
H -2.6486106369 -3.0402439731 -1.8414925812
C 0.1243926551 0.8786312424 -2.2963347852
C 0.9509463264 -0.0931951461 -2.8986429208
C -0.7343656784 1.6084161338 -3.1409188461
H 1.6311362858 -0.6861025287 -2.2868340306
H -1.4206567503 2.33938521 -2.708017836
C 0.9505400147 -0.2938260702 -4.2805481946
C -0.7374238642 1.4222918556 -4.5290253003
H 1.6146744156 -1.0312992162 -4.7256277751
H -1.3988770927 2.0110875604 -5.1606616724
C 0.1059477225 0.466899808 -5.0974821006
H 0.1068156696 0.3118073456 -6.1734691297

Frequency: -226.5847; 11.7347; 18.7033; 19.5689; 25.0496; 28.4698; 31.5902; 34.2694; 38.9276; 43.3732; 46.2403; 52.3762; 61.8408; 66.6595; 77.5469; 82.0631; 90.1359; 106.3099; 114.1534; 127.5926; 152.7907; 157.9128; 163.8593; 167.0213; 170.4311; 178.3790; 182.2759; 192.3066; 204.4790; 216.7654; 221.2163; 230.8514; 241.1178; 243.3560; 249.0694; 258.9438; 263.9423; 288.5323; 302.6235; 306.8580; 309.4845; 318.4914; 321.3554; 345.0239; 360.5598; 364.5432; 371.3911; 396.7362; 403.3756; 424.0260; 450.9727; 480.6071; 489.8668; 491.7243; 508.3188; 531.5166; 535.3227; 542.3731; 557.8827; 558.5939; 575.5328; 579.5982; 592.1944; 626.2230; 626.6417; 632.4457; 638.0900; 651.8203; 716.5858; 723.0954; 749.1297; 762.7628; 765.4633; 765.6734; 792.5750; 840.3349; 876.7019; 934.0779; 935.0502; 937.2640; 954.0733; 963.1097; 981.1491; 982.3905; 988.8279; 1004.4415; 1008.4418; 1016.6677; 1024.6165; 1048.4978; 1054.2134; 1060.6226; 1066.9691; 1068.7567; 1075.3593; 1096.3209; 1144.5792; 1183.6185; 1192.0065; 1199.8097; 1202.9724; 1203.9760; 1215.5573; 1253.5348; 1255.1568; 1262.1270; 1269.0141; 1286.0044; 1294.4662; 1309.7327; 1322.0531; 1328.8177; 1361.5640; 1386.0749; 1434.0486; 1435.4721; 1439.7449; 1460.7717; 1465.2716; 1495.9996; 1502.6339; 1505.6341; 1514.1221; 1516.0635; 1517.1745; 1528.3071; 1529.6138; 1548.9524; 1592.6200; 1615.5581; 1620.2857; 1675.7117; 1811.7868; 3041.1697; 3066.8463; 3069.9610; 3076.2946; 3100.8886; 3129.5075; 3136.7085; 3137.3123; 3146.0111; 3146.8331; 3150.5559; 3152.0994; 3159.0330; 3167.7155; 3178.0125; 3186.8736; 3203.4511.

Cartesian Forces: Max 0.000004567 RMS 0.000000740

Item	Value	Threshold	Converged?
Maximum Force	0.000002	0.000450	YES
RMS Force	0.000000	0.000300	YES
Maximum Displacement	0.000168	0.001800	YES
RMS Displacement	0.000032	0.001200	YES

FILE: Yb_Sq2_PhYbOTf2_TS_Neutral_Add56_B3LYP_M062x_Conf4-.out

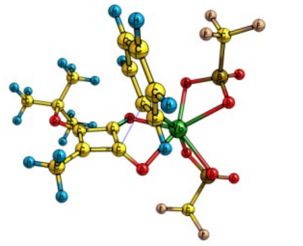

Conformer 9

E(RB3LYP/6-31G(d)/MWB59) = -2769.88835428
Convg = 0.9351D-08 -V/T = 2.0149

Temperature 195.150 Kelvin. Pressure 1.00000 Atm.
elec. and zero-point Energies= -2769.545564
elec. and thermal Energies= -2769.526945
elec. and thermal Enthalpies= -2769.526327
elec. and thermal Free Energies= -2769.586474

RB3LYP/6-31G(d)/MWB59/THF(IEFPCM) = -2769.91041375

RM06-2x/6-311+G(d,p)/MWB59/THF(IEFPCM) = -2769.71776321

Geometry at RB3LYP/6-31G(d)/MWB59
Charge = 0 Multiplicity = 1
Yb 1.3575853794 2.2734975353 -0.5017048958
O 0.6209657408 4.4015404056 0.1790824426
S 1.3042552961 4.4097789184 1.5322855869
O 2.1126008859 5.5650637593 1.8748281988
O 1.9543815197 3.0377920335 1.6390579512
C -0.0863508563 4.3581885987 2.7782645762
F -0.8747109713 5.4159309848 2.5941719075
F -0.8017074198 3.2405692813 2.6343383486
F 0.4326869935 4.3920403362 4.005467658
O 3.5574678711 1.5508501703 -0.975478172
O 5.1809900324 3.4718749808 -1.3389834958
O 2.6990268036 3.5943171989 -1.8792304188
S 3.97831829 2.7790528534 -1.7619767259
C 4.3176754674 2.168501709 -3.4937128669
F 5.2743294689 1.2413526476 -3.4439381891
F 3.2155044875 1.6407618407 -4.0270423606
F 4.7226316019 3.1942600321 -4.2394132014
C -0.6452691162 -1.4321986399 0.2332331552
C -1.6297185209 -0.5979967629 -0.2864088554
C 0.3235801861 -0.3028845632 0.3241935939
C -0.7543901342 0.5963462462 -0.199666882
O 1.4818067324 -0.0288772396 0.6290696121
O -0.9038957047 1.828708656 0.0322575457
O -0.6900590392 -2.7209183996 0.4759473266
C 0.4588770866 -3.4621806456 1.0793777143
C -3.0086280915 -0.8083899206 -0.7771118443
H -3.3469874715 -1.8335047344 -0.6056329184
H -3.0507821941 -0.5939905592 -1.8537491294
H -3.6999200084 -0.111179171 -0.2882052371
C 1.6524060923 -3.398172466 0.1253504807
H 2.0634638344 -2.3875655709 0.0527494152
H 2.4486761378 -4.0507797926 0.4988960485
H 1.3674489633 -3.746831456 -0.8726692399
C -0.0966238342 -4.8812192158 1.1892081763
H 0.6601226069 -5.5424689093 1.6224484397
H -0.9834124872 -4.9022166927 1.8302312307
H -0.3706598951 -5.2675498586 0.2026001549
C 0.7699621589 -2.8725961788 2.455850237
H -0.1281218799 -2.8559189292 3.0819292138
H 1.5196548481 -3.4962179367 2.954166822
H 1.1804556694 -1.8615391926 2.3853101097
C 0.2984725827 0.816741085 -2.2555475839
C 0.9352420339 -0.2322612115 -2.9506307413
C -0.5949124192 1.621886715 -2.9895610926
H 1.6302604113 -0.8889378988 -2.4263683903
H -1.1607761915 2.4040616147 -2.4775600148
C 0.7208374888 -0.4427073578 -4.3132134526
C -0.8012410155 1.4376198687 -4.3632438416
H 1.2324045903 -1.2521475506 -4.8295286764
H -1.4833334606 2.0862717013 -4.9080068518
C -0.1450736589 0.3984809876 -5.0230032602
H -0.3106842492 0.2363502066 -6.0851028675

Frequency: -244.8895; 9.7141; 18.2437; 24.3796; 25.6527; 27.9607; 29.4071; 36.6476; 39.2451; 40.3992; 47.6062; 54.4993; 65.2902; 70.2126; 79.5178; 81.1979; 85.1701; 108.7327; 113.7441; 127.2703; 134.9622; 140.3359; 158.3152; 166.5779; 172.1084; 173.8774; 186.8743; 200.3813; 210.5954; 212.9128; 216.7552; 221.2676; 240.5106; 253.5061; 263.4957; 270.3462; 274.2256; 293.2953; 303.8394; 307.0373; 318.5676; 321.4622; 326.1008; 344.4992; 361.0964; 363.2727; 371.4743; 396.2360; 400.5532; 419.6647; 451.2260; 489.8761; 491.4000; 492.0828; 502.2294; 530.5525; 535.0340; 538.2132; 558.1552; 558.8004; 572.0720; 581.2642; 600.1506; 624.1193; 626.3120; 636.0805; 639.6950; 643.9662; 714.5847; 720.0807; 732.6728; 753.2986; 764.9496; 766.4359; 791.7936; 860.4545; 875.6996; 935.2543; 935.7742; 942.3779; 952.6364; 963.0958; 978.9788; 980.1744; 987.9705; 1005.1949; 1007.5400; 1016.3858; 1032.2089; 1051.3572; 1052.7259; 1066.1405; 1066.4604; 1071.9185; 1073.1920; 1096.8867; 1121.6686; 1191.9770; 1200.8008; 1201.8712; 1203.2801; 1215.2965; 1221.1302; 1252.4326; 1256.2770; 1262.7915; 1263.7943; 1286.9254; 1303.5713; 1310.7638; 1320.8137; 1331.6489; 1361.0627; 1396.0113; 1435.0046; 1435.9454; 1443.0497; 1462.0346; 1465.2353; 1495.7966; 1500.7903; 1504.4720; 1514.4550; 1515.8457; 1516.3039; 1534.2612; 1535.2817; 1550.2070; 1594.1805; 1615.1204; 1621.8892; 1667.4659; 1796.9954; 3044.7045; 3063.7974; 3064.3510; 3071.1397; 3099.4397; 3123.2464; 3131.7860; 3133.7105; 3141.2747; 3144.3003; 3144.9578; 3149.2379; 3150.4770; 3153.8204; 3177.9248; 3186.6671; 3203.6743.

Cartesian Forces: Max 0.000003942 RMS 0.000000685

Item	Value	Threshold	Converged?
Maximum Force	0.000003	0.000450	YES
RMS Force	0.000000	0.000300	YES
Maximum Displacement	0.000461	0.001800	YES
RMS Displacement	0.000107	0.001200	YES

FILE: Yb_Sq2_PhYbOTf2_TS_Neutral_Add56_B3LYP_M062x_Conf7.out

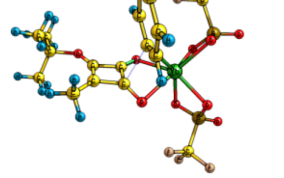

Conformer 10

E(RB3LYP/6-31G(d)/MWB59) = -2769.88034128
Convg = 0.7457D-09 -V/T = 2.0149

Temperature 195.150 Kelvin. Pressure 1.00000 Atm.
elec. and zero-point Energies= -2769.537586
elec. and thermal Energies= -2769.518971
elec. and thermal Enthalpies= -2769.518353
elec. and thermal Free Energies= -2769.578883

RB3LYP/6-31G(d)/MWB59/THF(IEFPCM) = -2769.90489641

RM06-2x/6-311+G(d,p)/MWB59/THF(IEFPCM) = -2769.71206740

Geometry at RB3LYP/6-31G(d)/MWB59
Charge = 0 Multiplicity = 1
Yb 1.0845533011 2.2057920611 -0.4975576175
O 0.7116333703 4.4265593249 0.0201533486
S 0.8544752356 4.3315077931 1.5368693046
O 1.7881941672 5.2451187927 2.1694549261
O 1.0147579076 2.8516927042 1.8069713524
C -0.831628365 4.7881455267 2.1997316513
F -1.0459713138 6.0830960581 1.9684804371
F -1.7861523189 4.0702193433 1.610830933
F -0.849536948 4.557426399 3.5131799924
O 3.4062587692 2.0818552233 -0.2026851947
O 4.7753906443 3.8255835738 -1.455565289
O 2.4794108975 3.0199644544 -2.200586504
S 3.8023444184 2.7516693791 -1.5071996413
C 4.5768389309 1.3757371982 -2.5013296137
F 5.7514195915 1.0526475264 -1.9654258806
F 3.7760160914 0.3017074386 -2.486186215
F 4.7490653906 1.7782727868 -3.7582172988
C -1.0139978047 -1.4454300032 -0.0763290451
C -2.0092628916 -0.5609748753 -0.4941509015
C -0.0302009754 -0.3409599868 0.0851655556
C -1.1037333818 0.6128140335 -0.3368931257
O 1.1448832418 -0.143941794 0.383549153
O -1.2540730536 1.8290335778 -0.0568771666
O -0.7555570452 -2.7138023541 0.1405069976
C -1.7128052587 -3.8360116157 -0.0242661182
C -3.4051656871 -0.5471322159 -0.9957778036
H -3.9722991704 -1.4516567547 -0.7714284368
H -3.3943229858 -0.4047828281 -2.0860671892
H -3.9314023386 0.3160967556 -0.5721382472
C -2.855067961 -3.6705522573 0.9798701316
H -3.4504677264 -2.7734879882 0.7919041095
H -3.5248599067 -4.5346923343 0.918892891
H -2.4615132122 -3.6143653411 1.9995052203
C -0.8513936859 -5.0501873086 0.3271732074
H -1.4493922713 -5.9642081526 0.2560406188
H -0.0039039942 -5.1340383734 -0.3596568653
H -0.4638386566 -4.9655646234 1.3467180343
C -2.1762769663 -3.9018985443 -1.4807486618
H -1.314701061 -3.9702710641 -2.1521012847
H -2.7936446674 -4.7946524594 -1.6257262089
H -2.7735478092 -3.0351523846 -1.7728930246
C -0.0073628556 0.9296189962 -2.3727640548
C 0.6330808059 -0.0635965833 -3.1418060285
C -0.8538779245 1.8236881559 -3.0549989717
H 1.2846411358 -0.7926833232 -2.6595874207
H -1.419519211 2.5676078056 -2.4889356754
C 0.4765926241 -0.1274822536 -4.5275599847
C -1.0055813057 1.7827925926 -4.4470277502
H 0.9934047161 -0.8934083723 -5.1019719952
H -1.6503403917 2.4996258453 -4.9503958214
C -0.3393655292 0.8026694031 -5.1829027554
H -0.4592494657 0.7551798494 -6.2623293438

Frequency: -224.6417; 4.5036; 19.1592; 22.3935; 25.1353; 28.5180; 32.1898; 36.1509; 37.8903; 43.0424; 47.5579; 54.1090; 64.1917; 72.6684; 79.1590; 82.9938; 91.6591; 105.8721; 116.8015; 125.0643; 152.3845; 158.4697; 160.8069; 162.4124; 172.0951; 178.1966; 183.1965; 189.6781; 205.3578; 214.2319; 221.4264; 231.7700; 238.8462; 245.6332; 252.7696; 259.8656; 266.6445; 290.4548; 302.8715; 306.6167; 308.3806; 318.1168; 321.3601; 345.3205; 361.9056; 363.4802; 372.1594; 397.1535; 404.2744; 418.9426; 450.8192; 480.3705; 489.5140; 491.4719; 508.5775; 530.6606; 535.9860; 541.2388; 557.2562; 559.1357; 572.0177; 582.1121; 592.4922; 623.3435; 626.5807; 635.5818; 639.1168; 651.6231; 715.0446; 721.6011; 747.9692; 763.0828; 764.5243; 765.9346; 792.8228; 840.6375; 874.7148; 933.6047; 934.4435; 934.8763; 951.8164; 963.2028; 978.9023; 980.8303; 989.1077; 1004.2594; 1006.2249; 1016.9034; 1023.9010; 1047.6550; 1055.0956; 1060.3196; 1066.7378; 1068.5105; 1074.6954; 1097.1790; 1142.5714; 1182.7981; 1191.7203; 1201.6252; 1202.7839; 1204.0470; 1215.3604; 1242.9794; 1259.1401; 1260.9107; 1268.1834; 1286.0377; 1294.9125; 1310.4573; 1321.2669; 1334.2140; 1361.1750; 1385.4626; 1433.4759; 1435.4622; 1439.2399; 1460.5516; 1465.4965; 1495.5675; 1502.6605; 1505.5556; 1514.8869; 1516.1781; 1517.2882; 1528.0294; 1529.8011; 1548.8953; 1601.6337; 1615.8232; 1621.4446; 1678.3408; 1802.0156; 3041.1460; 3066.9962; 3070.0155; 3076.2952; 3101.5850; 3124.7200; 3136.6597; 3137.4971; 3146.0534; 3146.4533; 3147.0253; 3152.1416; 3158.5457; 3167.1923; 3176.9797; 3185.4393; 3203.0813.

Cartesian Forces: Max 0.000000506 RMS 0.000000204

Item	Value	Threshold	Converged?
Maximum Force	0.000000	0.000450	YES
RMS Force	0.000000	0.000300	YES
Maximum Displacement	0.000095	0.001800	YES
RMS Displacement	0.000018	0.001200	YES

FILE: Yb_Sq2_PhYbOTf2_TS_Neutral_Add56_B3LYP_M062x_Conf7-.out

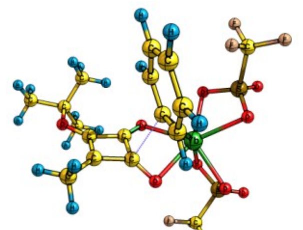

Conformer 11

E(RB3LYP/6-31G(d)/MWB59) = -2769.88834600
Convg = 0.8304D-08 -V/T = 2.0149

Temperature 195.150 Kelvin. Pressure 1.00000 Atm.
elec. and zero-point Energies= -2769.545558
elec. and thermal Energies= -2769.526937
elec. and thermal Enthalpies= -2769.526319
elec. and thermal Free Energies= -2769.586509

RB3LYP/6-31G(d)/MWB59/THF(IEFPCM) = -2769.91023982

RM06-2x/6-311+G(d,p)/MWB59/THF(IEFPCM) = -2769.71759047

Geometry at RB3LYP/6-31G(d)/MWB59
Charge = 0 Multiplicity = 1
Yb 0.8965848857 2.3137073785 -0.3507607951
O -0.1422202576 4.3268658676 0.2848034617
S 0.3705123482 4.3575372361 1.7110361246
O 1.0008953332 5.5785356077 2.1771089072
O 1.1497948987 3.0591597425 1.8636937295
C -1.1439959089 4.1126688657 2.7765373371
F -2.0161614013 5.08706679 2.5234030298
F -1.7104071939 2.9316926074 2.5194545615
F -0.7809980497 4.1555349573 4.0583291002
O 3.2020592409 1.8439257465 -0.575121689
O 4.6395126414 3.9355896007 -0.7041948194
O 2.2369726238 3.8157705569 -1.5290932155
S 3.5749534747 3.1354388557 -1.2802102955
C 4.1737107866 2.6232393445 -2.9732593698
F 5.2119932939 1.7990609198 -2.8320089761
F 3.2026459195 2.0029035642 -3.6440708876
F 4.5493711809 3.7105820441 -3.6431958763
C -0.7649652768 -1.6048468954 0.0600324665
C -1.765764144 -0.8621569247 -0.5579334709
C 0.058310778 -0.3835451007 0.2899517178
C -1.041446524 0.4141459031 -0.3420543401
O 1.1359879261 0.0004079766 0.7379200901
O -1.350331994 1.6154938137 -0.1049473257
O -0.6983099328 -2.8983922174 0.269834921
C 0.4440984289 -3.5339494146 0.9942207733
C -3.04634506 -1.2002942548 -1.2154024143
H -3.2907500745 -2.2598341639 -1.1040091789
H -2.9835838589 -0.9592348018 -2.2853845831
H -3.861320147 -0.5940342855 -0.8015775531
C 1.7286861205 -3.3140702039 0.194074957
H 2.0334032289 -2.2639736472 0.1913383362
H 2.5408664102 -3.8907475686 0.6493587267
H 1.6041011984 -3.6587346462 -0.837666822
C 0.0362800232 -5.0062899723 1.008065668
H 0.8030780211 -5.5980373755 1.5174443511
H -0.913042251 -5.1409878991 1.5356898928
H -0.0748570765 -5.3872099789 -0.011881408
C 0.5234699542 -2.9596182753 2.4094536968
H -0.4392912685 -3.0574686385 2.9216301533
H 1.2714771457 -3.5168004941 2.9832928851
H 0.8277305892 -1.9093184547 2.4088971815
C 0.2167250336 0.8129217448 -2.2499468349
C 1.0402300922 -0.1403035862 -2.8841294499
C -0.6666552613 1.5427718377 -3.0697891984
H 1.7362912726 -0.7365629638 -2.2932800005
H -1.3701047289 2.2438660114 -2.6139837101
C 1.0106643174 -0.328130837 -4.266417542
C -0.6896724172 1.382340861 -4.4616287575
H 1.6636749508 -1.0622236413 -4.7334935837
H -1.3695935215 1.9728534336 -5.0713616443
C 0.1477664452 0.4397847203 -5.0582261498
H 0.1259983059 0.2954173542 -6.1354723842

Frequency: -245.1011; 9.4173; 17.2755; 24.3459; 25.4783; 27.9179; 29.1678; 37.3543; 39.7962; 40.2953; 47.4750; 54.9334; 65.6136; 70.1260; 79.7393; 81.2378; 85.2811; 108.7078; 112.1556; 127.6744; 135.0557; 140.5325; 158.0429; 166.5880; 172.0147; 173.9502; 187.0352; 200.3134; 210.8934; 213.2268; 216.5612; 221.0819; 240.0824; 253.1902; 264.1591; 270.4718; 274.6386; 293.4097; 303.6346; 306.6566; 318.3196; 321.3381; 326.0160; 344.4726; 361.0680; 363.2952; 371.5810; 396.3711; 400.8062; 419.5881; 451.2018; 489.8121; 491.3333; 492.0985; 502.3469; 530.4408; 534.9148; 538.1944; 558.1171; 558.7565; 572.0951; 581.2457; 600.1351; 624.0580; 626.3018; 636.0335; 639.6716; 643.9936; 714.5271; 720.2454; 732.6190; 753.3300; 764.9159; 766.3788; 791.7820; 860.3100; 875.9538; 935.2792; 935.9666; 942.5187; 952.5966; 963.0370; 979.0728; 980.1912; 988.0253; 1005.2203; 1007.5679; 1016.4175; 1032.2553; 1051.3330; 1052.7534; 1065.9936; 1066.5395; 1071.9490; 1073.2530; 1096.9028; 1121.6046; 1191.9503; 1200.7373; 1201.7665; 1203.1911; 1215.4192; 1221.1555; 1252.3070; 1256.2297; 1262.7777; 1263.7397; 1286.9477; 1303.5717; 1310.7805; 1320.8397; 1331.6449; 1361.2132; 1396.0565; 1434.9982; 1435.8674; 1443.0504; 1461.9853; 1465.1808; 1495.8318; 1500.6936; 1504.4769; 1514.5178; 1515.9002; 1516.3652; 1534.2527; 1535.2529; 1550.1418; 1594.1247; 1615.0930; 1621.8911; 1667.4438; 1797.0581; 3044.7249; 3063.8117; 3064.3320; 3071.1346; 3099.5336; 3123.2871; 3131.8046; 3133.6869; 3141.2851; 3144.2803; 3144.9467; 3149.1543; 3150.5382; 3153.8757; 3177.9066; 3186.6208; 3203.6609.

Cartesian Forces: Max 0.000008718 RMS 0.000001982

Item	Value	Threshold	Converged?
Maximum Force	0.000007	0.000450	YES
RMS Force	0.000001	0.000300	YES
Maximum Displacement	0.001624	0.001800	YES
RMS Displacement	0.000210	0.001200	YES

FILE: Yb_Sq2_PhYbOTf2_TS_Neutral_Add56_B3LYP_M062x_Conf8.out

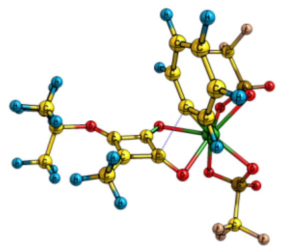

Conformer 12

E(RB3LYP/6-31G(d)/MWB59) = -2769.88034042
Convg = 0.9239D-08 -V/T = 2.0149

Temperature 195.150 Kelvin. Pressure 1.00000 Atm.
elec. and zero-point Energies= -2769.537606
elec. and thermal Energies= -2769.518979
elec. and thermal Enthalpies= -2769.518361
elec. and thermal Free Energies= -2769.579000

RB3LYP/6-31G(d)/MWB59/THF(IEFPCM) = -2769.90529441

RM06-2x/6-311+G(d,p)/MWB59/THF(IEFPCM) = -2769.71251535

Geometry at RB3LYP/6-31G(d)/MWB59
Charge = 0 Multiplicity = 1
Yb 1.0665923447 2.257934354 -0.4053256347
O 0.6336002147 4.4717793686 0.0949948151
S 0.7285396984 4.3819168178 1.6157843904
O 1.6232833759 5.3144057426 2.2765309981
O 0.9083214409 2.9059174311 1.8942650923
C -0.9868602042 4.8067860479 2.2220826453
F -1.2181178961 6.0972676767 1.9823350889
F -1.9074710536 4.070400882 1.6027575512
F -1.0437965993 4.5764963066 3.5344893568
O 3.379066323 2.1805212997 -0.0331868654
O 4.755471754 3.9478360723 -1.2443955395
O 2.5011345013 3.0971820825 -2.0629153556
S 3.8052337383 2.855487108 -1.3255003882
C 4.6379783683 1.4925933299 -2.2899996861
F 5.8003415403 1.1930803504 -1.7151982838
F 3.8575527596 0.4035299714 -2.2978903906
F 4.843476215 1.8955105923 -3.541751397
C -0.9705416895 -1.4344381706 -0.0465409224
C -1.9693729557 -0.5706915963 -0.4978020193
C -0.0150417996 -0.3101345073 0.1450953034
C -1.0932311463 0.6213224775 -0.3133128799
O 1.145393241 -0.0890069257 0.4815180372
O -1.277109909 1.8345262366 -0.040225285
O -0.6936789676 -2.6971171018 0.1805732329
C -1.6221088824 -3.8386693119 -0.0134444676
C -3.348384543 -0.5855381869 -1.0441076024
H -3.9038538748 -1.5013615965 -0.8371371726
H -3.3053496894 -0.4434015549 -2.1336280046
H -3.9054907677 0.2669255959 -0.6385106416
C -2.7994379509 -3.6956777818 0.9529476037
H -3.406522126 -2.8110436523 0.7445505479
H -3.4492049505 -4.5733041674 0.8714776238
H -2.4403464782 -3.6307662903 1.984709608
C -0.7480766438 -5.0348073828 0.367506186
H -1.3248440894 -5.960836182 0.2785698672
H 0.1226598452 -5.102085658 -0.2915047814
H -0.3954185614 -4.9414096032 1.3988846415
C -2.0367056835 -3.915291695 -1.4840763053
H -1.1526197683 -3.9672960001 -2.1270280353
H -2.6312445242 -4.8202875084 -1.647419925
H -2.6412162143 -3.0609081853 -1.7970067912
C 0.061748588 0.9582634511 -2.3130010456
C 0.7473915091 -0.0219323401 -3.0594797298
C -0.7807308018 1.8339008339 -3.0235231364
H 1.3981870646 -0.7367624291 -2.5554265432
H -1.3796704604 2.5662947117 -2.4770412874
C 0.6371163755 -0.0901915901 -4.4494632334
C -0.8865262437 1.788679944 -4.4196624096
H 1.1881473342 -0.8455558472 -5.0057675412
H -1.5295370414 2.4914374202 -4.9446516034
C -0.1765607736 0.8221040777 -5.1321745027
H -0.26052357 0.7712176867 -6.2148336367

Frequency: -224.9519; 4.1201; 19.1349; 22.1935; 25.1446; 28.3131; 32.0225; 35.8341; 37.7055; 42.8155; 47.1046; 54.0250; 63.5737; 72.2949; 78.8933; 83.0049; 91.6065; 105.7648; 116.7273; 124.8847; 152.4615; 158.3579; 160.9431; 162.3280; 171.9930; 177.5542; 182.3160; 189.6860; 205.3046; 214.2403; 221.3607; 231.0113; 238.8114; 245.5695; 252.7260; 259.3805; 266.5255; 290.4480; 302.8628; 306.5803; 308.3507; 318.1702; 321.2975; 345.2483; 361.9225; 363.4665; 372.1546; 397.0449; 404.1243; 418.9886; 450.7340; 480.3048; 489.5050; 491.4874; 508.4959; 530.6365; 535.9576; 541.2532; 557.2503; 559.1221; 572.0221; 582.1497; 592.4674; 623.3190; 626.5590; 635.6130; 639.1247; 651.6303; 715.0714; 721.5584; 747.9373; 763.0426; 764.5197; 765.9009; 792.7524; 840.6781; 874.5494; 933.5816; 934.3925; 934.7203; 951.8233; 963.2047; 978.8321; 980.8958; 989.0408; 1004.2608; 1006.1949; 1016.9447; 1023.8899; 1047.5149; 1055.1240; 1060.3043; 1066.8200; 1068.4639; 1074.7373; 1097.0628; 1142.5569; 1182.7752; 1191.7106; 1201.6327; 1202.7909; 1204.0823; 1215.3515; 1242.9177; 1259.1439; 1260.8973; 1268.2090; 1285.9908; 1294.8591; 1310.5721; 1321.2623; 1334.2415; 1361.2027; 1385.3489; 1433.3841; 1435.4038; 1439.1966; 1460.4700; 1465.4746; 1495.5279; 1502.5690; 1505.5090; 1514.8472; 1516.0621; 1517.2283; 1528.0073; 1529.6880; 1548.7923; 1601.6119; 1615.7793; 1621.4116; 1678.2686; 1802.0015; 3041.1438; 3066.9641; 3070.0312; 3076.3037; 3101.5412; 3124.9304; 3136.6761; 3137.5483; 3146.0294; 3146.7467; 3147.0011; 3152.1062; 3158.5778; 3167.2237; 3176.9745; 3185.4390; 3203.0766.

Cartesian Forces: Max 0.000000721 RMS 0.000000255

Item	Value	Threshold	Converged?
Maximum Force	0.000000	0.000450	YES
RMS Force	0.000000	0.000300	YES
Maximum Displacement	0.000061	0.001800	YES
RMS Displacement	0.000013	0.001200	YES

FILE: Yb_Sq2_PhYbOTf2_TS_Neutral_Add56_B3LYP_M062x_Conf8-.out

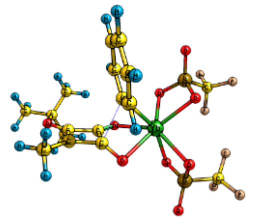

Conformer 13

E(RB3LYP/6-31G(d)/MWB59) = -2769.89177183
Convg = 0.8956D-08 -V/T = 2.0149

Temperature 195.150 Kelvin. Pressure 1.00000 Atm.
elec. and zero-point Energies= -2769.548784
elec. and thermal Energies= -2769.530234
elec. and thermal Enthalpies= -2769.529616
elec. and thermal Free Energies= -2769.589261

RB3LYP/6-31G(d)/MWB59/THF(IEFPCM) = -2769.91333575

RM06-2x/6-311+G(d,p)/MWB59/THF(IEFPCM) = -2769.72016456

Geometry at RB3LYP/6-31G(d)/MWB59
Charge = 0 Multiplicity = 1
Yb 1.154887112 2.2785542498 -0.4466775622
O 1.4438394382 3.1337149546 1.7360080634
S 0.6192539705 4.3962006687 1.5326385959
O -0.4529732959 4.6544396874 2.4768856348
O 0.2807472191 4.3972317262 0.0540251135
C 1.8388950051 5.7949441889 1.7283438771
F 2.347856791 5.7619698772 2.9591590471
F 2.8247401142 5.671711004 0.8367859351
F 1.2072686853 6.9505365499 1.5325344313
O 2.4870206818 3.4980631224 -1.9330504822
O 4.1981006422 2.1356287271 -3.2318051654
O 3.3332800384 1.4958135407 -0.9263029655
S 3.6872992537 2.5657753047 -1.942861706
C 5.0535818832 3.5419393976 -1.1268055937
F 5.2610293125 4.6653433961 -1.8093654772
F 4.7098084512 3.8382509891 0.1292634396
F 6.1637770217 2.8064560554 -1.1184075688
C -0.6560801333 -1.5317919973 0.1921529392
C -1.680206131 -0.7307414873 -0.3007717427
C 0.2521491771 -0.358404137 0.3320229495
C -0.8650572665 0.5030222811 -0.1711757862
O 1.3892605267 -0.0322179738 0.6646801707
O -1.0749644026 1.7169176638 0.1099127128
O -0.6358857476 -2.8300601653 0.3873985696
C 0.5519456632 -3.533500413 0.9565080152
C -3.0478935157 -0.9910900134 -0.7985696128
H -3.3322584261 -2.0388431349 -0.6716441265
H -3.1044753884 -0.7316773715 -1.8645731067
H -3.7725292949 -0.3526876147 -0.2784408109
C 1.737109598 -3.3672035246 0.0041094055
H 2.0945970759 -2.3343800544 -0.0272805734
H 2.5678357225 -3.9924207742 0.3484360067
H 1.4670100816 -3.6877566392 -1.007338072
C 0.0729826576 -4.9835781039 1.0078398134
H 0.8650534185 -5.6212974315 1.4123349085
H -0.8094636904 -5.0781210492 1.6482574181
H -0.1834622121 -5.3424477363 0.0062361259
C 0.8371098418 -2.9868691859 2.356279186
H -0.0581196188 -3.0443592578 2.9840031631
H 1.6210942504 -3.5899172982 2.8264724789
H 1.192012033 -1.9530083506 2.3274504319
C 0.1451537596 0.8593397495 -2.2250979952
C 0.9184211283 -0.0403764833 -2.9891951625
C -0.8344291398 1.6083234119 -2.9071742163
H 1.6888214548 -0.6425525066 -2.5079326725
H -1.4874450618 2.2776686893 -2.3435859359
C 0.7488955386 -0.1555375962 -4.3697872759
C -1.0049461151 1.5100964508 -4.2942725246
H 1.3713156571 -0.8402570739 -4.9412138378
H -1.7591283189 2.1103331987 -4.7980139217
C -0.2127776739 0.6238340491 -5.0241179962
H -0.3430344996 0.5350795508 -6.0996749552

Frequency: -244.5593; 11.5020; 20.4868; 23.7577; 27.4261; 29.0935; 29.9892; 36.3430; 40.4007; 50.3615; 52.4416; 59.1294; 62.4396; 69.0964; 76.4124; 80.7931; 86.2782; 105.7575; 116.8137; 129.2226; 138.0931; 139.3611; 156.1584; 163.9102; 171.7433; 177.1339; 194.7102; 202.3695; 208.4483; 211.8480; 217.5146; 223.7805; 245.6817; 250.5367; 263.1731; 270.0180; 274.0589; 294.8892; 303.9951; 308.4769; 320.5298; 321.7944; 325.8025; 344.3305; 361.5925; 364.6861; 370.7516; 397.9478; 399.8322; 424.5007; 451.2861; 489.4208; 490.7515; 492.1716; 502.2918; 530.4274; 534.7913; 538.3194; 558.2868; 558.7224; 573.8649; 579.0852; 600.3412; 622.8592; 626.0801; 636.8823; 639.3743; 644.2841; 715.8119; 722.0683; 733.0723; 753.1538; 765.4001; 766.5958; 791.7323; 861.9489; 875.9756; 935.1638; 938.8091; 942.1967; 950.5256; 965.0539; 980.1199; 983.4414; 987.4612; 1005.8663; 1008.7861; 1016.4501; 1032.5572; 1051.7134; 1055.8869; 1066.2112; 1067.0119; 1071.9213; 1073.1918; 1097.2843; 1123.0925; 1192.5985; 1200.8675; 1201.7063; 1204.4016; 1215.9511; 1220.4042; 1244.8323; 1259.9240; 1266.2603; 1268.6935; 1286.7667; 1303.2468; 1310.3844; 1319.7985; 1328.5200; 1362.1489; 1395.0744; 1434.7064; 1435.8974; 1442.7354; 1461.9585; 1465.4984; 1495.7351; 1500.7682; 1504.4976; 1514.1263; 1515.9470; 1516.3225; 1534.3915; 1535.2895; 1550.2470; 1592.8725; 1615.4672; 1621.3650; 1665.7675; 1796.5485; 3044.5243; 3063.7507; 3064.1559; 3071.0472; 3099.1419; 3131.6083; 3133.3683; 3135.4747; 3141.1069; 3144.5683; 3148.9825; 3150.5226; 3153.5039; 3154.0198; 3179.9533; 3188.8605; 3204.8158.

Cartesian Forces: Max 0.000001036 RMS 0.000000333

Item	Value	Threshold	Converged?
Maximum Force	0.000000	0.000450	YES
RMS Force	0.000000	0.000300	YES
Maximum Displacement	0.000074	0.001800	YES
RMS Displacement	0.000012	0.001200	YES

FILE:
Yb_Sq2_PhYbOTf2_TS_Neutral_Add56_B3LYP_M062x_Confx7.out

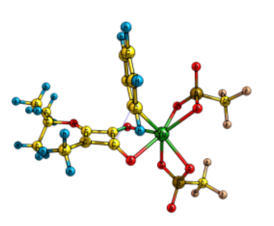

Conformer 14

E(RB3LYP/6-31G(d)/MWB59) = -2769.88315085
Convg = 0.8995D-08 -V/T = 2.0149

Temperature 195.150 Kelvin. Pressure 1.00000 Atm.
elec. and zero-point Energies= -2769.540234
elec. and thermal Energies= -2769.521691
elec. and thermal Enthalpies= -2769.521073
elec. and thermal Free Energies= -2769.581071

RB3LYP/6-31G(d)/MWB59/THF(IEFPCM) = -2769.90701015

RM06-2x/6-311+G(d,p)/MWB59/THF(IEFPCM) = -2769.71467590

Geometry at RB3LYP/6-31G(d)/MWB59
Charge = 0 Multiplicity = 1
Yb 0.9784334598 2.320691163 -0.4432702244
O 0.8662885269 2.6662274928 1.9028908988
S 0.3782519636 4.1007137098 1.8331106568
O -0.8362590997 4.4441644197 2.5509600729
O 0.4476904659 4.4536605402 0.3552604746
C 1.7492164685 5.0938320096 2.6194329167
F 1.7396347326 4.878742105 3.9338772356
F 2.9297578925 4.7188068742 2.1198209742
F 1.5532171363 6.387672637 2.3728381506
O 2.1544602283 3.4978323685 -2.1028136896
O 4.1351827286 2.2154624256 -3.0560561813
O 3.2786506504 1.929385834 -0.6810441993
S 3.4937604441 2.8150331307 -1.8998497814
C 4.6319364843 4.1726429813 -1.3130780016
F 4.8489355437 5.0177181289 -2.3184662403
F 4.0729813383 4.8276871624 -0.2941731741
F 5.7841839892 3.6345053657 -0.9181449878
C -1.1181050852 -1.3320296251 -0.0309459711
C -2.0687371731 -0.4657566835 -0.5687346337
C -0.1351998987 -0.2280771926 0.1441649619
C -1.1691494767 0.7116545168 -0.4007446802
O 1.0229800631 -0.0302646751 0.4958059909
O -1.3512295048 1.939516619 -0.1705112772
O -0.8890163264 -2.5897574396 0.2700239797
C -1.8411323936 -3.711978575 0.0879300369
C -3.4216422958 -0.4675205816 -1.177407418
H -4.0070318403 -1.3641734795 -0.9691496655
H -3.3258928995 -0.3593211515 -2.2674303799
H -3.9765818339 0.4087549846 -0.8223598807
C -3.0589858342 -3.4874431198 0.9863905535
H -3.627785105 -2.5976352645 0.7051006936
H -3.7306337753 -4.3494753622 0.9161279544
H -2.7480906458 -3.3802179817 2.0302128604
C -1.0238621884 -4.9117408536 0.5705276598
H -1.6234184632 -5.8246862156 0.498013187
H -0.12419663 -5.0362312066 -0.039511936
H -0.719490823 -4.7762494817 1.6125925649
C -2.1868404226 -3.8527351686 -1.3958918089
H -1.2748080034 -3.9644305958 -1.9904016496
H -2.8021751338 -4.7465818145 -1.5430660351
H -2.7469369665 -2.9976169742 -1.7811149982
C 0.0691353214 0.9355801173 -2.3253878745
C 0.8888193084 -0.0344994118 -2.9391589974
C -0.7835095206 1.6840743786 -3.1588696205
H 1.5587834091 -0.6465580421 -2.334347139
H -1.4641315885 2.4126214161 -2.7139918483
C 0.8916956133 -0.2128512155 -4.324238584
C -0.7855036948 1.5177787852 -4.5487651903
H 1.5512716158 -0.9483273375 -4.7791035582
H -1.4428233436 2.1190488524 -5.1728556751
C 0.0539964891 0.5662950381 -5.1303318038
H 0.0557472304 0.4276754204 -6.2085474423

Frequency: -240.9445; 8.3040; 16.2095; 18.8928; 26.9717; 28.5184; 32.7209; 36.3685; 38.2662; 48.2303; 56.7684; 58.3594; 65.7269; 67.1603; 81.7330; 85.0365; 90.8856; 106.4313; 115.5656; 130.5688; 150.4701; 153.8891; 159.8397; 167.0749; 172.9911; 179.0556; 182.1405; 193.8566; 204.1835; 217.9531; 223.6376; 231.4242; 244.3138; 249.1359; 249.9414; 259.7874; 267.3990; 290.4354; 303.7604; 307.7485; 312.1461; 319.2413; 322.5532; 345.4919; 362.1904; 365.5871; 373.3781; 397.8888; 404.2534; 422.4932; 451.1770; 480.6117; 488.9566; 490.1199; 510.1007; 530.2832; 536.2290; 543.7308; 558.3485; 558.5609; 573.4175; 578.2083; 592.6909; 623.5079; 626.2094; 637.1377; 640.3748; 652.1590; 715.5360; 723.2875; 750.2322; 762.6177; 765.4513; 767.4642; 792.7612; 841.1642; 877.3182; 933.8943; 934.5214; 938.8433; 950.3104; 965.3506; 980.8990; 983.6740; 988.6450; 1004.8967; 1008.5343; 1017.1319; 1024.0300; 1048.8905; 1054.7919; 1060.5648; 1066.4994; 1068.4219; 1072.5583; 1097.1377; 1138.0458; 1182.2590; 1192.0879; 1201.2281; 1204.0948; 1205.4414; 1215.0529; 1244.9655; 1262.4829; 1266.4910; 1267.9445; 1285.5850; 1294.3125; 1311.0472; 1319.2111; 1327.8043; 1361.1530; 1384.1883; 1433.8063; 1435.2107; 1439.3122; 1460.6485; 1466.1240; 1495.7544; 1502.6039; 1505.4018; 1514.0464; 1516.1601; 1517.3490; 1528.3668; 1529.5837; 1549.1132; 1589.2637; 1616.5335; 1620.2828; 1671.5214; 1805.8243; 3040.7574; 3066.5634; 3069.7875; 3076.0481; 3100.5397; 3134.3435; 3136.2401; 3136.9998; 3145.5822; 3146.4183; 3146.5517; 3152.8084; 3158.5493; 3167.5702; 3178.5355; 3187.5178; 3203.8719.

Cartesian Forces: Max 0.000001076 RMS 0.000000252

Item	Value	Threshold	Converged?
Maximum Force	0.000001	0.000450	YES
RMS Force	0.000000	0.000300	YES
Maximum Displacement	0.000039	0.001800	YES
RMS Displacement	0.000007	0.001200	YES

FILE: Yb_Sq2_PhYbOTf2_TS_Neutral_Add56_B3LYP_M062x_Confx7-.out

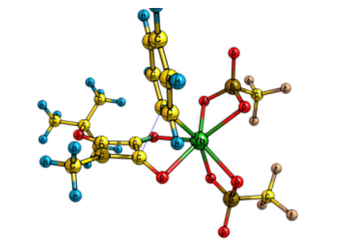

Conformer 15

E(RB3LYP/6-31G(d)/MWB59) = -2769.89175724
Convg = 0.4888D-08 -V/T = 2.0149

Temperature 195.150 Kelvin. Pressure 1.00000 Atm.
elec. and zero-point Energies= -2769.548803
elec. and thermal Energies= -2769.530237
elec. and thermal Enthalpies= -2769.529619
elec. and thermal Free Energies= -2769.589339

RB3LYP/6-31G(d)/MWB59/THF(IEFPCM) = -2769.91298598

RM06-2x/6-311+G(d,p)/MWB59/THF(IEFPCM) = -2769.71977323

Geometry at RB3LYP/6-31G(d)/MWB59
Charge = 0 Multiplicity = 1

```
Yb  0.883529339  2.291102566 -0.3534506916
O  0.9734779972  3.1245913937  1.8543066268
S  0.0907197408  4.3399315545  1.6116000142
O -1.0571248336  4.5179292957  2.4826540578
O -0.1451326754  4.3473859355  0.1131802187
C  1.2078732313  5.8044254495  1.9109313422
F  1.6315277561  5.7798092243  3.1738466425
F  2.2586936847  5.755468965  1.0891366634
F  0.5229736536  6.9240003737  1.6869369209
O  2.2313193222  3.6074338054 -1.7403756966
O  4.1036054142  2.3659782238 -2.9336625274
O  3.1330384883  1.6504762521 -0.6934943027
S  3.4850958864  2.75072075 -1.6779673164
C  4.7330466558  3.8002729043 -0.7686889211
F  4.9144349923  4.9410523729 -1.4295181431
F  4.291419946  4.0624502375  0.4641721115
F  5.8835883728  3.1339486662 -0.6926373659
C -0.7365033476 -1.627322838  0.1293105621
C -1.7721086025 -0.8806732697 -0.4215161403
C  0.0907679123 -0.4053032516  0.3378169237
C -1.0407379492  0.3965429995 -0.228206932
O  1.1835925005 -0.018428758  0.7453632903
O -1.3398428545  1.5921275383  0.0509143192
O -0.6530664103 -2.9246319989  0.314129315
C  0.5352114604 -3.5654108586  0.9520510919
C -3.087804603 -1.2133394074 -1.0084205071
H -3.31813569 -2.2771955079 -0.9088133244
H -3.0915881235 -0.9447878139 -2.0736542784
H -3.8799957082 -0.6240559086 -0.5305411754
C  1.7664208681 -3.3199452672  0.0784018652
H  2.0640295126 -2.2678115195  0.0779752206
H  2.6089381288 -3.8998854882  0.4700685769
H  1.5799461388 -3.6440160953 -0.9506516229
C  0.1389735936 -5.0410868077  0.9609671744
H  0.9396771351 -5.6370349191  1.4097358263
H -0.7750478386 -5.1936679353  1.5432659303
H -0.0326702408 -5.4021964506 -0.0578046411
C  0.6994489632 -3.0198710399  2.3715133709
H -0.2286961924 -3.135877233  2.9406200841
H  1.4858455178 -3.5825418917  2.8856783834
H  0.9950587793 -1.9671416087  2.3736366198
C  0.0766146521  0.8390707416 -2.2074648052
C  0.9491395472 -0.0040717141 -2.9278984059
C -0.8985458602  1.5397382466 -2.9449502464
H  1.7203420729 -0.567518082 -2.4029882508
H -1.6248759714  2.1624638984 -2.4188769002
C  0.8768078133 -0.1098168884 -4.3177287055
C -0.9724680249  1.4508897194 -4.3411441474
H  1.5739441506 -0.7496596436 -4.8538007405
H -1.7260572117  2.0134987323 -4.8874038494
C -0.0841296876  0.6217880813 -5.0260641682
H -0.1387901245  0.5403896299 -6.1086778001
```

Frequency: -245.1523; 11.5841; 20.6689; 23.3707; 26.8361; 28.7542; 29.7450; 36.2590; 40.1166; 50.3214; 52.3910; 58.2827; 61.9831; 68.7094; 76.0901; 80.4989; 85.8310; 105.6774; 114.9942; 128.9631; 138.0067; 138.8157; 155.6414; 163.7509; 171.5113; 176.8867; 194.6806; 202.2520; 208.6157; 212.2323; 217.4242; 223.6733; 245.9857; 250.4823; 263.7150; 269.9026; 273.9893; 294.7146; 303.8459; 308.3539; 320.4308; 321.6909; 325.6957; 344.1393; 361.6189; 364.7834; 370.9468; 397.8497; 400.0048; 424.3457; 451.2551; 489.3178; 490.6314; 492.0256; 502.5724; 530.4478; 534.7181; 538.3095; 558.2497; 558.6945; 573.7906; 578.9844; 600.3339; 622.9001; 626.0007; 636.8401; 639.3285; 644.2701; 715.7059; 721.9088; 733.0337; 753.1696; 765.3827; 766.6014; 791.6885; 861.8035; 875.7303; 935.1342; 938.7071; 942.3294; 950.4422; 965.0459; 980.0926; 983.3304; 987.6201; 1005.8965; 1008.7278; 1016.4242; 1032.6303; 1051.8635; 1055.7625; 1065.9919; 1066.5550; 1071.9756; 1073.3354; 1097.0188; 1122.9486; 1192.5599; 1200.8234; 1201.6555; 1204.3552; 1215.4245; 1220.3734; 1244.7627; 1260.0204; 1266.2118; 1268.6857; 1286.5596; 1303.1819; 1310.1866; 1319.7594; 1328.4663; 1361.5168; 1395.2383; 1434.7660; 1435.8210; 1442.7882; 1461.8353; 1465.4229; 1495.7165; 1500.6498; 1504.4264; 1513.8285; 1515.9365; 1516.3158; 1534.3607; 1535.1864; 1550.1139; 1592.6676; 1615.4084; 1621.2831; 1665.6813; 1796.5864; 3044.5105; 3063.6461; 3064.1082; 3070.9668; 3099.1251; 3131.5302; 3133.2575; 3135.4776; 3141.0692; 3144.4906; 3148.9823; 3150.6117; 3153.2650; 3153.9429; 3179.9175; 3188.8249; 3204.8120.

Cartesian Forces: Max 0.000010049 RMS 0.000001471

Item	Value	Threshold	Converged?
Maximum Force	0.000007	0.000450	YES
RMS Force	0.000001	0.000300	YES
Maximum Displacement	0.001199	0.001800	YES
RMS Displacement	0.000200	0.001200	YES

FILE: Yb_Sq2_PhYbOTf2_TS_Neutral_Add56_B3LYP_M062x_Confx8.out

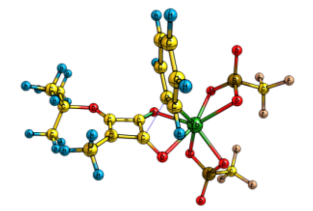

Conformer 16

E(RB3LYP/6-31G(d)/MWB59) = -2769.88315338
Convg = 0.2910D-08 -V/T = 2.0149

Temperature 195.150 Kelvin. Pressure 1.00000 Atm.
elec. and zero-point Energies= -2769.540211
elec. and thermal Energies= -2769.521685
elec. and thermal Enthalpies= -2769.521067
elec. and thermal Free Energies= -2769.580923

RB3LYP/6-31G(d)/MWB59/THF(IEFPCM) = -2769.90694418

RM06-2x/6-311+G(d,p)/MWB59/THF(IEFPCM) = -2769.71459020

Geometry at RB3LYP/6-31G(d)/MWB59
Charge = 0 Multiplicity = 1

```
Yb  1.0634542605  2.2112954114 -0.7756109723
O  1.019600668  2.6792877045  1.5514174943
S  0.573213426  4.1227164795  1.4190338426
O -0.6098837802  4.5413457163  2.1492958628
O  0.6117492377  4.3961422203 -0.0768372961
C  1.9955034962  5.1110925726  2.1152239749
F  2.0140844223  4.9677966331  3.4393231934
F  3.1497763335  4.670541099  1.6077760438
F  1.8343167494  6.3955249821  1.8029497703
O  2.2058237228  3.2646674142 -2.5389746621
O  4.1593318376  1.9098618708 -3.4466310996
O  3.3545466276  1.788633067 -1.0398400481
S  3.5463632082  2.5896208413 -2.3197213998
C  4.7033097292  3.9772392551 -1.8509753683
F  4.9068972204  4.7467329114 -2.9179977206
F  4.1674507368  4.7075942012 -0.8717210903
F  5.8593125352  3.4599810546 -1.4394354605
C -1.0959402203 -1.3632322788 -0.0960598081
C -2.0434689729 -0.507935253 -0.6562479705
C -0.0847477863 -0.2740774481 -0.0142963604
C -1.1145877658  0.6563687763 -0.5830892041
O  1.0879994911 -0.0841559719  0.290465001
O -1.2649285468  1.8992859412 -0.4204671319
O -0.8848064559 -2.6064047571  0.271236
C -1.8658323906 -3.7149103705  0.182072476
C -3.4133819435 -0.5135935828 -1.2255883151
H -4.0119868274 -1.3823183195 -0.9480108069
H -3.347345535 -0.4720909845 -2.3223743369
H -3.9382233345  0.3949768715 -0.9079337935
C -3.0514465259 -3.4106411672  1.0999918484
H -3.6100715332 -2.5265579627  0.7825899261
H -3.7424818327 -4.2601306343  1.1004929481
H -2.7073478016 -3.2490294195  2.1262470276
C -1.060738064 -4.9034217552  0.7106897366
H -1.6816314568 -5.8049021233  0.7082595929
H -0.1821826728 -5.0836069353  0.0840203341
H -0.7231994302 -4.7152108169  1.7341853604
C -2.2582637108 -3.932902086 -1.2806119829
H -1.3668872386 -4.1001960609 -1.8931455042
H -2.8972020257 -4.8189193862 -1.357971643
H -2.8105305868 -3.0883409161 -1.69900333
C  0.0739672058  0.739311826 -2.5496362369
C  0.8569305776 -0.2831803449 -3.1250507438
C -0.7850582051  1.4557967157 -3.4043678653
H  1.5295764038 -0.8736493973 -2.5019475462
H -1.4384909089  2.2242838705 -2.9867488046
C  0.8194172595 -0.543041578 -4.4966983152
C -0.8275063739  1.207661447 -4.7813262781
H  1.4513454926 -1.3185688879 -4.9234376314
H -1.4888302521  1.7852628119 -5.4232881587
C -0.0233601298  0.205297482 -5.3262908817
H -0.0532333777  0.0031841054 -6.3940084891
```

Frequency: -240.7183; 9.3734; 16.1875; 19.2767; 26.7909; 28.2907; 32.9298; 36.4299; 38.8168; 48.7874; 57.0431; 59.1735; 66.7480; 67.8689; 81.7104; 85.1971; 91.0547; 106.5449; 115.5571; 131.4219; 150.8300; 154.4009; 160.2361; 167.2241; 173.1533; 179.4102; 183.1626; 193.8244; 204.2962; 217.8091; 223.6387; 231.2211; 244.2068; 249.0149; 249.7757; 259.6179; 267.6898; 290.5482; 303.7877; 307.7179; 312.2531; 319.1481; 322.7715; 345.5145; 362.1241; 365.5641; 373.4600; 397.9509; 404.3832; 422.6952; 451.3338; 480.7153; 489.0853; 490.1611; 510.1372; 530.3846; 536.1444; 543.7057; 558.3025; 558.6138; 573.5382; 578.2630; 592.7278; 623.4088; 626.2508; 637.1163; 640.3072; 652.1492; 715.3571; 723.3474; 750.1445; 762.6014; 765.4247; 767.3293; 792.8701; 841.2442; 877.5308; 933.9539; 934.7575; 938.9481; 950.2441; 965.1920; 980.6451; 983.8298; 988.7683; 1004.9426; 1008.5307; 1017.1521; 1023.9925; 1049.2484; 1054.9283; 1060.6112; 1066.6435; 1068.5158; 1072.4820; 1097.3018; 1137.7355; 1182.2282; 1192.1208; 1200.9720; 1203.9597; 1205.3763; 1215.2250; 1245.0982; 1262.1681; 1266.6631; 1267.8841; 1285.6487; 1294.4049; 1311.2148; 1319.2162; 1327.8393; 1361.3042; 1384.4428; 1433.8865; 1435.3654; 1439.3501; 1460.7433; 1466.2265; 1495.9127; 1502.5969; 1505.4405; 1514.1604; 1516.1248; 1517.5535; 1528.1175; 1529.7397; 1549.1674; 1589.1289; 1616.6173; 1620.2875; 1671.5256; 1805.4760; 3040.7865; 3066.5819; 3069.7556; 3076.0588; 3100.5635; 3134.2753; 3136.2064; 3136.9971; 3145.6000; 3145.8916; 3146.5498; 3152.6783; 3158.5551; 3167.4760; 3178.5358; 3187.4893; 3203.8763.

Cartesian Forces: Max 0.000000832 RMS 0.000000220

Item	Value	Threshold	Converged?
Maximum Force	0.000001	0.000450	YES
RMS Force	0.000000	0.000300	YES
Maximum Displacement	0.000895	0.001800	YES
RMS Displacement	0.000125	0.001200	YES

FILE: Yb_Sq2_PhYbOTf2_TS_Neutral_Add56_B3LYP_M062x_Confx8-.out

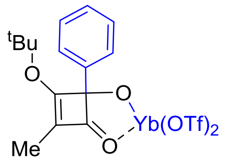 	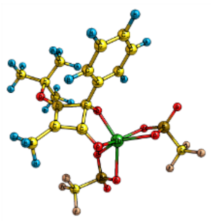	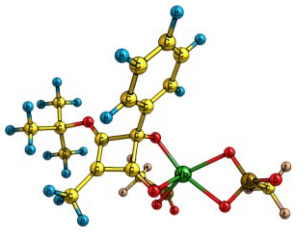	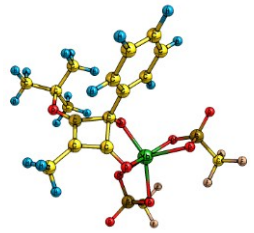
**C-1 addition product from $PhYb(OTf)_2$ + 1b**	*Conformer 1*	*Conformer 2*	*Conformer 3*
Lowest energy conformation is:<br>Conformer 1	E(RB3LYP/6-31G(d)/MWB59) = -2769.98001602<br>Convg = 0.1439D-08 -V/T = 2.0149<br><br>Temperature 195.150 Kelvin. Pressure 1.00000 Atm.<br>elec. and zero-point Energies= -2769.633746<br>elec. and thermal Energies= -2769.615393<br>elec. and thermal Enthalpies= -2769.614775<br>elec. and thermal Free Energies= -2769.675363<br><br>RB3LYP/6-31G(d)/MWB59/THF(IEFPCM) = -2770.00194721<br><br>RM06-2x/6-311+G(d,p)/MWB59/THF(IEFPCM) = -2769.80563726	E(RB3LYP/6-31G(d)/MWB59) = -2769.97454632<br>Convg = 0.6791D-08 -V/T = 2.0149<br><br>Temperature 195.150 Kelvin. Pressure 1.00000 Atm.<br>elec. and zero-point Energies= -2769.628159<br>elec. and thermal Energies= -2769.609897<br>elec. and thermal Enthalpies= -2769.609279<br>elec. and thermal Free Energies= -2769.669095<br><br>RB3LYP/6-31G(d)/MWB59/THF(IEFPCM) = -2769.99969138<br><br>RM06-2x/6-311+G(d,p)/MWB59/THF(IEFPCM) = -2769.80101577	E(RB3LYP/6-31G(d)/MWB59) = -2769.98014009<br>Convg = 0.2505D-08 -V/T = 2.0149<br><br>Temperature 195.150 Kelvin. Pressure 1.00000 Atm.<br>elec. and zero-point Energies= -2769.633906<br>elec. and thermal Energies= -2769.615531<br>elec. and thermal Enthalpies= -2769.614913<br>elec. and thermal Free Energies= -2769.675537<br><br>RB3LYP/6-31G(d)/MWB59/THF(IEFPCM) = -2770.00105376<br><br>RM06-2x/6-311+G(d,p)/MWB59/THF(IEFPCM) = -2769.80489480
	Geometry at RB3LYP/6-31G(d)/MWB59<br>Charge = 0 Multiplicity = 1<br>Yb -0.4457602676 0.5090640948 2.9020466415<br>O -0.709002274 2.8321377014 2.8407115772<br>S 0.6327130293 3.1896292553 3.4602210271<br>O 1.437504695 4.1948689123 2.7916626396<br>O 1.2802834667 1.8431080352 3.7437526424<br>C 0.1947847946 3.8489969752 5.1501445<br>F -0.498800979 4.97569271 5.0140059195<br>F -0.5442426092 2.9423567875 5.7954730618<br>F 1.3121758935 4.0825593259 5.8322291866<br>O -1.8183042214 -0.0697417233 4.7558496017<br>O -0.7337248981 -1.4966301569 6.5606355024<br>O 0.1020193796 -1.3790137388 4.1603545298<br>S -1.0458989443 -1.3095881877 5.1568169672<br>C -2.1500506139 -2.72307474 4.6423642921<br>F -3.2931690321 -2.6663619334 5.3181442741<br>F -2.4059407547 -2.6285478234 3.3281256497<br>F -1.5357143564 -3.8779578509 4.8855936786<br>C -0.7394938458 -1.7128552344 -0.5447973727<br>C -2.0845537166 -1.6722146994 -0.1832653398<br>C -1.8277392968 -0.4781042031 0.5435899354<br>C -0.3979215672 -0.2840949106 0.0079040708<br>O 0.4510604533 -0.1767564343 1.096591384<br>O -2.2857384756 0.1087443767 1.5621030087<br>O -0.1042717196 -2.7336668337 -1.0539351567<br>C 1.383352026 -2.867908086 -1.1608668595<br>C -3.2113528128 -2.6456302353 -0.2585482417<br>H -2.8621119183 -3.6095650293 -0.6407307771<br>H -4.0100043487 -2.2870808413 -0.918731592<br>H -3.6504050788 -2.8002889708 0.7336743266<br>C -0.2031398578 0.80898675 -1.0410016602<br>C 0.7695767876 1.7961498426 -0.8427129331<br>C -1.0010285431 0.8663116576 -2.1942297166<br>H 1.3957389161 1.7521409779 0.0412052932<br>H -1.7640184147 0.1124191334 -2.3672588845<br>C 0.9412916202 2.820749277 -1.7765848471<br>C -0.8285881372 1.8897546351 -3.1253488228<br>H 1.698596971 3.5802262687 -1.6033046302<br>H -1.4571657559 1.9210604063 -4.0113344563<br>C 0.1438037716 2.8716523132 -2.9197644811<br>H 0.2754517146 3.6703421854 -3.6445101928<br>C 1.9359718802 -3.0477305761 0.252814999<br>H 1.7575809111 -2.1541048379 0.85718452<br>H 1.47281399 -3.9111916322 0.7416589691<br>H 3.0162116723 -3.2230055084 0.2019722156<br>C 1.985800517 -1.6624812214 -1.8777471109<br>H 3.0361078053 -1.877671107 -2.1027243775<br>H 1.4689517191 -1.4673258821 -2.8227367481<br>H 1.9559945565 -0.7603779501 -1.2643982291<br>C 1.5254653814 -4.1408640982 -1.9949948334<br>H 2.5851298009 -4.3898681424 -2.1090185174<br>H 1.0229407152 -4.9815676914 -1.5069247467<br>H 1.0922219718 -4.0060663419 -2.9913278866<br><br>Frequency: 10.5834; 14.3853; 16.7080; 21.1662; 21.2293; 26.4628; 27.5711; 40.9927; 41.4885; 45.7971; 48.6277; 57.2528; 58.5985; 64.5594; 84.4037; 85.6754; 98.1684; 99.9706; 135.1526; 159.6780; 161.5930; 164.9924; 186.6374; 191.8146; 194.7849; 210.7602; 217.3410; 223.2418; 224.0988; 231.2907; 234.5385; 242.4816; 266.1857; 276.4264; 278.4389; 303.5564; 305.6140; 310.7190; 319.7382; 320.1562; 328.3589; 342.7682; 346.2639; 363.6323; 366.8247; 387.3228; 413.4390; 420.7995; 452.3676; 455.9712; 490.5667; 491.3312; 494.8651; 516.8097; 526.9753; 532.3002; 537.7158; 556.7962; 558.1783; 578.6030; 582.8646; 591.7060; 625.0235; 633.0187; 634.0990; 670.5627; 692.2492; 715.3527; 723.1361; 763.6996; 763.8899; 766.4294; 779.6570; 800.1525; 860.2865; 865.8161; 898.2050; 922.9479; 938.0988; 943.5872; 953.4124; 960.7831; 963.3373; 979.7151; 988.2647; 1006.0781; 1016.6655; 1042.2007; 1054.6258; 1059.2471; 1067.8806; 1071.0080; 1072.5555; 1074.3603; 1076.3854; 1110.9412; 1192.5907; 1195.5754; 1198.0287; 1199.5205; 1209.6211; 1215.3023; 1239.9644; 1242.4708; 1255.0131; 1258.4788; 1272.8416; 1273.7118; 1288.5637; 1303.0171; 1325.6326; 1333.4502; 1347.2513; 1364.7000; 1416.2149; 1434.0248; 1436.1067; 1458.2177; 1462.4900; 1493.8840; 1502.7433; 1508.5904; 1513.0260; 1514.7418; 1519.1075; 1531.3463; 1535.0189; 1540.6177; 1550.5104; 1572.2748; 1640.3217; 1659.4901; 1743.2500; 3053.9660; 3060.0714; 3063.9021; 3071.2884; 3111.2371; 3128.6670; 3131.2757; 3137.5248; 3138.8364; 3143.2380; 3151.7928; 3172.0810; 3185.3782; 3195.1873; 3203.5998; 3213.2806; 3232.7475.<br><br>Cartesian Forces: Max 0.000000596 RMS 0.000000223<br><br>Item Value Threshold Converged?<br>Maximum Force 0.000000 0.000450 YES<br>RMS Force 0.000000 0.000300 YES<br>Maximum Displacement 0.000013 0.001800 YES<br>RMS Displacement 0.000002 0.001200 YES	Geometry at RB3LYP/6-31G(d)/MWB59<br>Charge = 0 Multiplicity = 1<br>Yb -0.8267739233 0.6430580805 2.7768249607<br>O -1.7460653649 2.5905958826 3.7810246683<br>S -0.5212962843 3.4642934482 3.5939083442<br>O -0.7082141928 4.7919959907 3.0401983011<br>O 0.486122271 2.5537514823 2.9013098232<br>C 0.1680103966 3.6690893937 5.3154417269<br>F -0.6895518151 4.373460464 6.0492440246<br>F 0.3441597329 2.4645345924 5.8659902536<br>F 1.3356133731 4.3036979287 5.2490402003<br>O -1.9691697223 -1.0734749718 3.9167761005<br>O -1.1276043378 -1.641145267 6.2485141529<br>O 0.3030088444 -0.5327384586 4.4625693704<br>S -0.8303583599 -1.4709947233 4.8392135505<br>C -0.2838586952 -3.1360449644 4.1957879863<br>F -1.2804878755 -4.0090671302 4.3353274982<br>F 0.0354770602 -3.0368913691 2.8985820293<br>F 0.7779784061 -3.5488623927 4.8810555862<br>C -1.3457147007 -1.5309675056 -0.6308379907<br>C -2.6791837468 -1.1828291136 -0.3897601951<br>C -2.2031073796 -0.0303041409 0.3120965825<br>C -0.7388878281 -0.2021058322 -0.0989020246<br>O 0.0113423061 -0.2669186004 1.0632057632<br>O -2.6025640302 0.6798145075 1.2715850374<br>O -0.605774867 -2.5346118986 -1.007386949<br>C -1.0632824787 -3.9291479664 -1.2573587682<br>C -4.0674024999 -1.7245572715 -0.5188639904<br>H -4.1584129097 -2.7630388483 -0.1859945899<br>H -4.4271285676 -1.6746761986 -1.5539178053<br>H -4.741459445 -1.1191911221 0.0954286745<br>C -0.1541001627 0.7103604163 -1.1701611734<br>C 1.1120240125 1.2728248693 -0.9769840131<br>C -0.8569627249 1.0025738349 -2.3484981376<br>H 1.6544186139 1.0438730013 -0.0668842542<br>H -1.8423955917 0.5757753861 -2.515899109<br>C 1.6665086187 2.1129359344 -1.9452207767<br>C -0.30378299 1.8449736962 -3.3119292496<br>H 2.6506279208 2.5433308222 -1.780716219<br>H -0.8632096903 2.0681140169 -4.2165963113<br>C 0.9617061905 2.4027151648 -3.1137071629<br>H 1.3920084308 3.0603432715 -3.8640902981<br>C -2.050759848 -3.9444909232 -2.4244713739<br>H -2.9883383797 -3.4385880591 -2.1842188373<br>H -1.6094682523 -3.4666358545 -3.3049266199<br>H -2.2901382115 -4.9812721681 -2.6839428743<br>C -1.6360024507 -4.4989794162 0.0409441818<br>H -1.8535253701 -5.5639675402 -0.0927430574<br>H -0.9158323844 -4.3928106997 0.8574454705<br>H -2.5636028589 -4.0032002603 0.3371392227<br>C 0.241982257 -4.6309294076 -1.6356221844<br>H 0.0497232624 -5.6882301634 -1.8441089584<br>H 0.6814807315 -4.1741462082 -2.5275432488<br>H 0.9662540581 -4.5617021307 -0.8187234102<br><br>Frequency: 12.8556; 14.7688; 20.3238; 22.5505; 26.0406; 28.4784; 30.5406; 36.8398; 40.1710; 44.7976; 50.9420; 59.4508; 61.5728; 67.1175; 93.7410; 99.1299; 114.4817; 129.2741; 142.6767; 149.8831; 165.4367; 166.3046; 177.3787; 186.8734; 192.4149; 202.3805; 214.0107; 216.2997; 222.0024; 234.9366; 236.9311; 238.9094; 255.6662; 260.9555; 263.4296; 302.8832; 304.9858; 315.6253; 319.7763; 320.4434; 321.3771; 340.9162; 353.4929; 363.3326; 365.3041; 390.3842; 409.2029; 418.2516; 455.0075; 464.3461; 486.2445; 491.3453; 491.9238; 501.3151; 532.2894; 534.2465; 543.4325; 557.9433; 558.0578; 578.6147; 583.2915; 625.4110; 633.1853; 633.8586; 635.3670; 682.9704; 694.0131; 713.9586; 727.1512; 758.9810; 764.3355; 766.1304; 785.7739; 811.8169; 845.9651; 863.9817; 892.9994; 927.1793; 936.5114; 937.6063; 953.5786; 960.8140; 974.1454; 981.6420; 982.5377; 1004.7634; 1017.4826; 1036.5965; 1054.7009; 1060.3502; 1060.9000; 1067.3630; 1070.3336; 1070.6988; 1080.6654; 1110.0150; 1192.6909; 1197.4698; 1200.7559; 1201.6427; 1208.0601; 1210.9021; 1222.0115; 1241.1712; 1253.8744; 1258.0327; 1269.2467; 1271.1248; 1287.5177; 1296.2384; 1326.4127; 1333.6112; 1349.7850; 1365.3333; 1411.5198; 1434.1369; 1436.3684; 1452.5683; 1461.4993; 1495.1652; 1501.3199; 1510.4835; 1515.5399; 1518.0704; 1520.0651; 1528.9993; 1530.3329; 1542.4593; 1548.4155; 1573.6306; 1641.9820; 1661.4322; 1740.3582; 3053.0927; 3066.1326; 3070.4229; 3077.2042; 3113.0961; 3133.6329; 3134.4890; 3140.5105; 3145.0204; 3147.6403; 3159.8461; 3165.0586; 3182.3337; 3191.3840; 3200.4044; 3211.5159; 3237.1627.<br><br>Cartesian Forces: Max 0.000001406 RMS 0.000000483<br><br>Item Value Threshold Converged?<br>Maximum Force 0.000001 0.000450 YES<br>RMS Force 0.000000 0.000300 YES<br>Maximum Displacement 0.000020 0.001800 YES<br>RMS Displacement 0.000004 0.001200 YES	Geometry at RB3LYP/6-31G(d)/MWB59<br>Charge = 0 Multiplicity = 1<br>Yb -0.528599309 0.5777298767 2.8930451302<br>O -0.7058002053 2.9094595581 2.7456331637<br>S 0.6343108492 3.238172199 3.3818980435<br>O 1.4978126548 4.1771345576 2.690139963<br>O 1.2194845997 1.8819026229 3.7440252878<br>C 0.1856603557 3.9976606754 5.0261893993<br>F -0.4254843955 5.1605107698 4.8187229639<br>F -0.6335786839 3.1723542019 5.6840381969<br>F 1.2934860088 4.1872812277 5.7380967714<br>O -0.2002917781 -1.3713954067 4.120409707<br>O -2.1785116957 -2.2639275085 5.4389217343<br>O -1.8925313799 0.1842469437 4.8106170378<br>S -1.2825333019 -1.1539803129 5.1714466034<br>C -0.3244003648 -0.8238391678 6.737661832<br>F 0.337493712 -1.9236036342 7.0882634297<br>F 0.5438123151 0.1698715914 6.523703596<br>F -1.1694894542 -0.4757667012 7.7044070681<br>C -0.7463478065 -1.6918992815 -0.530529358<br>C -2.0985618743 -1.656044169 -0.1939137182<br>C -1.8597067228 -0.4587622603 0.5317605141<br>C -0.4234333346 -0.2562493402 0.0168298574<br>O 0.4100560995 -0.1282055798 1.114696807<br>O -2.3382273037 0.1312357888 1.5406687822<br>O -0.0975119771 -2.713046102 -1.0205258409<br>C 1.392184508 -2.838888864 -1.1052341683<br>C -3.2219334519 -2.630637953 -0.2957073382<br>H -2.8424436485 -3.6351217239 -0.5076220536<br>H -3.9187957159 -2.3581563087 -1.0977979697<br>H -3.791827009 -2.659435758 0.6392054608<br>C -0.2240452144 0.8276951942 -1.0416778756<br>C 0.7435674689 1.8201639423 -0.844720282<br>C -1.0109041084 0.8702623842 -2.2031286109<br>H 1.3608800532 1.788168298 0.0457918683<br>H -1.7697462228 0.1121607472 -2.375739013<br>C 0.9211433249 2.8352292746 -1.7878181797<br>C -0.8327858976 1.8843659301 -3.143390339<br>H 1.6742599227 3.5989835874 -1.6150553372<br>H -1.4530213159 1.9042065312 -4.0355704767<br>C 0.1345757338 2.8714547803 -2.939098173<br>H 0.2707155675 3.6627842375 -3.6710749375<br>C 1.9287818658 -2.9894215096 0.317903965<br>H 1.7393975712 -2.0852267291 0.9027905748<br>H 1.4630806235 -3.8449218211 0.8180152859<br>H 3.0102580331 -3.1611307706 0.2823797779<br>C 1.9939101834 -1.6417288765 -1.8365466314<br>H 3.0492739397 -1.8510090934 -2.042683539<br>H 1.4886255902 -1.4695127077 -2.7922189719<br>H 1.9467710412 -0.7283257847 -1.2411903585<br>C 1.5536678504 -4.1255152742 -1.9145118431<br>H 2.6164898406 -4.3680083567 -2.0121585463<br>H 1.052179918 -4.9611459452 -1.4168181152<br>H 1.1306275409 -4.0122299793 -2.9178841445<br><br>Frequency: 12.5434; 14.2415; 16.0968; 20.0211; 23.2328; 24.6067; 27.3105; 41.0149; 42.1536; 45.0894; 49.4445; 54.9654; 59.3879; 63.1474; 79.5443; 84.9045; 97.6669; 99.9442; 135.5496; 156.3478; 161.9605; 163.4671; 184.0516; 189.1673; 197.2596; 210.6997; 217.5981; 220.4306; 223.1375; 230.7405; 236.3091; 245.0805; 267.4529; 276.2039; 276.4259; 303.1622; 306.3189; 312.2777; 319.6421; 320.4350; 328.8199; 343.4412; 346.9523; 363.3714; 367.4612; 387.5798; 413.7317; 420.8088; 452.6240; 455.9456; 490.9187; 491.4225; 494.7586; 516.3260; 526.0953; 532.3282; 538.1897; 557.9762; 558.3536; 577.8280; 583.6382; 591.7261; 622.4109; 633.1095; 636.7909; 669.9374; 691.8528; 715.2416; 722.8074; 763.5290; 765.9149; 766.3266; 779.3063; 800.1994; 860.5001; 865.6171; 898.0193; 922.5675; 938.3354; 943.5654; 951.4982; 961.3561; 963.6937; 979.5477; 988.5042; 1005.8474; 1016.6523; 1041.7647; 1054.0317; 1060.4050; 1068.1861; 1068.6478; 1071.7925; 1074.4153; 1075.6114; 1110.9066; 1192.5062; 1196.3566; 1199.6835; 1201.8069; 1209.3283; 1215.4471; 1240.9556; 1249.1302; 1255.3146; 1257.8437; 1271.5198; 1272.7886; 1288.7063; 1303.0115; 1322.3878; 1331.3407; 1347.0167; 1364.4661; 1416.5422; 1434.1788; 1436.4187; 1458.2972; 1462.6353; 1493.7668; 1502.7107; 1507.5386; 1513.0937; 1515.8104; 1519.1859; 1531.4834; 1534.8117; 1540.3961; 1550.4127; 1568.2150; 1640.2680; 1659.3700; 1742.2041; 3052.8617; 3060.2078; 3063.8542; 3071.1531; 3111.2413; 3128.9130; 3131.1163; 3137.1288; 3138.9252; 3143.2548; 3152.3353; 3171.4502; 3185.3657; 3195.2356; 3203.7082; 3213.2246; 3233.9559.<br><br>Cartesian Forces: Max 0.000000807 RMS 0.000000323<br><br>Item Value Threshold Converged?<br>Maximum Force 0.000000 0.000450 YES<br>RMS Force 0.000000 0.000300 YES<br>Maximum Displacement 0.000020 0.001800 YES<br>RMS Displacement 0.000004 0.001200 YES
	FILE: Yb_Sq2_PhYbOTf2_Prod12_Neutral_B3LYP_M062x_Conf1.out	FILE: Yb_Sq2_PhYbOTf2_Prod12_Neutral_B3LYP_M062x_Conf1-.out	FILE: Yb_Sq2_PhYbOTf2_Prod12_Neutral_B3LYP_M062x_Conf2.out

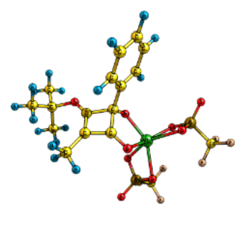

Conformer 4

E(RB3LYP/6-31G(d)/MWB59) = -2769.97426866
Convg = 0.9420D-08 -V/T = 2.0149

Temperature 195.150 Kelvin. Pressure 1.00000 Atm.
elec. and zero-point Energies= -2769.627952
elec. and thermal Energies= -2769.609648
elec. and thermal Enthalpies= -2769.609030
elec. and thermal Free Energies= -2769.669182

RB3LYP/6-31G(d)/MWB59/THF(IEFPCM) = -2769.99833640

RM06-2x/6-311+G(d,p)/MWB59/THF(IEFPCM) = -2769.80058643

Geometry at RB3LYP/6-31G(d)/MWB59
Charge = 0 Multiplicity = 1

```
Yb -0.6081344865  0.4818538248  2.9489739035
O  -0.5816114199  2.8236604012  2.831717601
S   0.7558129218  3.0258919148  3.5232322618
O   1.7198083811  3.9063350095  2.8908938586
O   1.2126815858  1.6174539554  3.8736669749
C   0.3081594964  3.7811405565  5.1696224634
F  -0.1983526956  4.9950699358  4.9713759535
F  -0.6000776557  3.0113542618  5.7763533568
F   1.3995778456  3.8629727349  5.9263585922
O  -0.5112020366 -1.5150662355  4.1422909884
O  -2.5973443154 -2.2277842648  5.3998667984
O  -2.0499195147  0.1915585795  4.8383217166
S  -1.5874491554 -1.2086870851  5.1766451982
C  -0.6341905077 -1.0159177754  6.7681141417
F  -0.0996386869 -2.1870549116  7.1058330402
F   0.3384405786 -0.1156001322  6.5949428274
F  -1.458461783 -0.6013688366  7.7270163515
C  -1.0619103725 -1.6851589671 -0.4832228924
C  -2.4065186018 -1.5646275035 -0.1133415946
C  -2.0597062188 -0.3710202361  0.5938280181
C  -0.6266073308 -0.2955594219  0.0621316515
O   0.2240897501 -0.2723704023  1.1549302203
O  -2.4892613161  0.2401007643  1.6088318895
O  -0.2129140936 -2.537644405 -0.9800371269
C  -0.4780869915 -3.9659668017 -1.3075950941
C  -3.6971585155 -2.3201689728 -0.146090683
H  -3.5859056761 -3.3750503961  0.1226090791
H  -4.1677324624 -2.2767341912 -1.1360525872
H  -4.3904129899 -1.866791772  0.5695975367
C  -0.281928  0.7253482388 -1.0159560167
C   0.9366822773  1.4090055326 -0.9496081423
C  -1.1556667792  0.9912246986 -2.0804392775
H   1.6124483622  1.1991483184 -0.1282528681
H  -2.1066489287  0.4693215018 -2.1489382011
C   1.275689138  2.344291273 -1.929672315
C  -0.8181120578  1.9293935731 -3.0552486211
H   2.2248065644  2.8691377607 -1.863492906
H  -1.509252992  2.1319723137 -3.8691964848
C   0.4004426606  2.608724433 -2.9835444146
H   0.6626155102  3.3409378898 -3.7424122068
C  -1.5696871675 -4.0558686079 -2.3744528706
H  -2.545953657 -3.739002955 -2.0020682775
H  -1.3077451007 -3.4415264998 -3.2418181614
H  -1.6636600044 -5.0944157586 -2.7091823066
C  -0.8161721003 -4.7089703527 -0.0151195796
H  -0.9290840979 -5.7777366624 -0.2258860114
H  -0.0129111872 -4.5857261021  0.7177253053
H  -1.7472537628 -4.3555058575  0.4345090486
C   0.8703722952 -4.421977494 -1.8669915909
H   0.8224975268 -5.4820067392 -2.1360862027
H   1.1327165252 -3.847101566 -2.7603621882
H   1.6606061977 -4.2856748589 -1.1229929218
```

Frequency: 11.2456; 15.0883; 20.7109; 23.2644; 24.7318; 26.5615; 31.8327; 32.0355; 41.8747; 43.0142; 49.7461; 54.0755; 56.9847; 64.8422; 90.4878; 101.1347; 121.0415; 130.9443; 143.1955; 146.6769; 161.4977; 164.8699; 172.4278; 185.0868; 196.2348; 202.4128; 215.7163; 216.7447; 219.8144; 233.1537; 235.7169; 244.8308; 253.6287; 261.6482; 263.1458; 303.2983; 306.4422; 318.7434; 319.7932; 320.8118; 322.1865; 339.5215; 353.2430; 363.0641; 367.5572; 387.4895; 408.6482; 418.2042; 454.4245; 463.6032; 484.7717; 491.0975; 491.5312; 503.4875; 531.2664; 533.6392; 542.5845; 558.0639; 558.3443; 578.0968; 584.0848; 622.4118; 633.2335; 635.6877; 636.5643; 682.4821; 692.4123; 714.6067; 726.5755; 760.4695; 765.8176; 766.1708; 784.8956; 812.5873; 844.9911; 864.8094; 893.1729; 927.6763; 936.5970; 937.0129; 951.7859; 962.9129; 974.6699; 981.7464; 982.4550; 1005.7040; 1017.4590; 1036.5637; 1055.0001; 1060.6257; 1061.3112; 1066.9978; 1070.6359; 1071.8129; 1080.6007; 1109.7216; 1192.4660; 1198.2529; 1200.3414; 1202.5732; 1207.6032; 1210.3678; 1222.5061; 1248.9934; 1254.5974; 1258.4351; 1270.2798; 1271.8651; 1288.1223; 1296.3699; 1322.1620; 1331.8432; 1349.4458; 1365.0005; 1412.8359; 1433.6388; 1435.8533; 1453.2427; 1460.6774; 1495.0472; 1501.0784; 1509.9229; 1514.5598; 1518.7712; 1520.0993; 1527.6281; 1530.7472; 1542.2612; 1548.0130; 1570.3726; 1641.9546; 1661.3182; 1739.0448; 3052.9562; 3065.9669; 3069.4712; 3075.5760; 3113.4306; 3133.5618; 3134.7639; 3137.6271; 3143.8058; 3147.4443; 3157.3251; 3167.3740; 3181.7112; 3191.2572; 3200.3613; 3211.2891; 3234.5610.

Cartesian Forces: Max 0.000001399 RMS 0.000000384

Item	Value	Threshold	Converged?
Maximum Force	0.000001	0.000450	YES
RMS Force	0.000000	0.000300	YES
Maximum Displacement	0.000013	0.001800	YES
RMS Displacement	0.000002	0.001200	YES

FILE: Yb_Sq2_PhYbOTf2_Prod12_Neutral_B3LYP_M062x_Conf2-.out

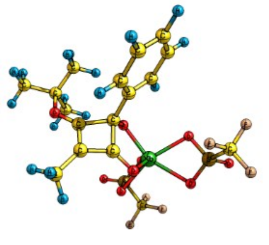

Conformer 5

E(RB3LYP/6-31G(d)/MWB59) = -2769.98106146
Convg = 0.9540D-08 -V/T = 2.0149

Temperature 195.150 Kelvin. Pressure 1.00000 Atm.
elec. and zero-point Energies= -2769.634779
elec. and thermal Energies= -2769.616433
elec. and thermal Enthalpies= -2769.615815
elec. and thermal Free Energies= -2769.676098

RB3LYP/6-31G(d)/MWB59/THF(IEFPCM) = -2770.00234102

RM06-2x/6-311+G(d,p)/MWB59/THF(IEFPCM) = -2769.80581818

Geometry at RB3LYP/6-31G(d)/MWB59
Charge = 0 Multiplicity = 1

```
Yb -0.1412278  0.6125570834  2.8395547507
O   1.1701208336  2.5366842844  2.7920570598
S   0.2253872266  3.4534314162  3.557518609
O   0.7904984285  4.2704310129  4.6142076172
O  -0.960667979  2.5708050728  3.8954967493
C  -0.4262623308  4.6053145917  2.2428183215
F   0.5717010105  5.3499651143  1.7737460381
F  -0.9490426055  3.8816071015  1.2460013267
F  -1.3675494289  5.3860841986  2.7653493727
O   1.1906210652 -0.4814809464  4.4210519267
O   0.4973789664 -2.8548319836  5.0164991605
O  -1.1062028196 -1.1006100496  4.1155773876
S   0.1342187425 -1.4530927131  4.9212876754
C  -0.2550745058 -0.8533857572  6.644850358
F   0.8197448284 -1.0014328697  7.4136359883
F  -0.596104807  0.4371506374  6.5928671123
F  -1.2671393103 -1.5630506292  7.1369135114
C  -0.8569013923 -1.5795937491 -0.5729830261
C  -2.1682319255 -1.2405192492 -0.2438345854
C  -1.671372899 -0.1209386307  0.4762303576
C  -0.2236617134 -0.2507691475 -0.0308979454
O   0.6084803339 -0.3106349817  1.0742782669
O  -2.0071608126  0.5747190282  1.4736738324
O  -0.449047182 -2.7214453967 -1.056011722
C   0.9752315552 -3.1812687102 -1.1210436019
C  -3.4798670637 -1.9413970876 -0.3463491624
H  -3.3325461551 -3.0056505826 -0.5550905221
H  -4.0976341299 -1.5231246832 -1.150490514
H  -4.0429984419 -1.840464511  0.5876553364
C   0.2218702464  0.7570149798 -1.08880797
C   1.3625159999  1.5376863316 -0.8645619941
C  -0.5040500944  0.9434009663 -2.2755557049
H   1.9282628312  1.3957070798  0.0491121302
H  -1.3942889122  0.3512203187 -2.4685078916
C   1.77079365  2.483355338 -1.808532169
C  -0.0953862038  1.8882229993 -3.2160167419
H   2.6585367998  3.0803552792 -1.6179991871
H  -0.6698543279  2.0204609947 -4.129011177
C   1.0449988747  2.6620751252 -2.9861969513
H   1.3624587659  3.3984229295 -3.7193996926
C   1.4388994466 -3.4609934888  0.3081021466
H   1.4591805216 -2.5404334665  0.8976411681
H   0.7784725388 -4.1858976039  0.7949175924
H   2.4504169051 -3.8811302741  0.2870044492
C   1.846191228 -2.1462166675 -1.8282346147
H   2.8300713048 -2.5885056331 -2.0191011363
H   1.4117656849 -1.8559843608 -2.7901627723
H   1.9969825413 -1.2513526185 -1.2219161854
C   0.8541256861 -4.4643239076 -1.9428130788
H   1.8357356369 -4.9408424585 -2.0279459112
H   0.1682350725 -5.1684031344 -1.4619237061
H   0.4844281155 -4.2499595916 -2.9507692817
```

Frequency: 13.7319; 15.0576; 19.0457; 21.2385; 22.5556; 27.3148; 29.4602; 41.6251; 43.0633; 46.4027; 47.2464; 52.5818; 59.8911; 65.1097; 78.4127; 83.1244; 98.3995; 100.5810; 135.4651; 158.8286; 162.1187; 164.5320; 187.5107; 188.9396; 195.2884; 210.0828; 216.7054; 222.6265; 225.4456; 231.1261; 237.9869; 241.6691; 267.1127; 275.6492; 276.7255; 303.6819; 305.8921; 310.6750; 319.8543; 320.2848; 329.7171; 343.0009; 346.8802; 365.2586; 366.4573; 387.8733; 414.0390; 421.2192; 451.8360; 455.7198; 490.6969; 491.3268; 493.7415; 517.2266; 527.0625; 532.3467; 538.1405; 557.5378; 558.1889; 578.7545; 583.3378; 591.6974; 625.2925; 633.1759; 634.1033; 670.4484; 692.4923; 714.7514; 722.9483; 764.6304; 765.3645; 766.4480; 779.6012; 799.9938; 859.8554; 865.6434; 898.3481; 922.9772; 937.9426; 943.3168; 952.9026; 960.4318; 963.8941; 979.5687; 988.0752; 1004.8794; 1016.5868; 1042.3118; 1054.0980; 1059.2340; 1067.5494; 1067.8048; 1071.9720; 1074.1778; 1075.5627; 1111.2809; 1192.7387; 1195.6356; 1199.0783; 1200.2161; 1210.8609; 1215.3336; 1241.3211; 1248.1766; 1254.8192; 1258.3213; 1271.6046; 1272.9563; 1288.6193; 1303.2392; 1325.0275; 1332.5200; 1347.3109; 1365.0484; 1416.4652; 1433.7719; 1435.8406; 1458.2525; 1462.2045; 1493.7788; 1502.2784; 1507.7390; 1513.0902; 1515.7317; 1519.0725; 1530.7930; 1535.0707; 1540.7593; 1550.5730; 1570.8330; 1640.0143; 1659.3553; 1744.4023; 3052.6902; 3061.3858; 3063.9832; 3071.1724; 3111.0505; 3129.9479; 3131.4598; 3137.0579; 3138.7962; 3143.6218; 3153.2181; 3170.3988; 3185.0115; 3193.7939; 3202.5959; 3212.6208; 3235.9008.

Cartesian Forces: Max 0.000000267 RMS 0.000000085

Item	Value	Threshold	Converged?
Maximum Force	0.000000	0.000450	YES
RMS Force	0.000000	0.000300	YES
Maximum Displacement	0.000019	0.001800	YES
RMS Displacement	0.000004	0.001200	YES

FILE: Yb_Sq2_PhYbOTf2_Prod12_Neutral_B3LYP_M062x_Conf3.out

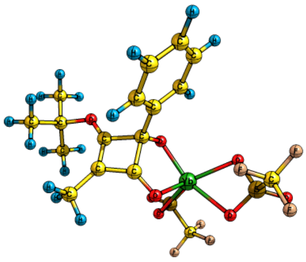

Conformer 6

E(RB3LYP/6-31G(d)/MWB59) = -2769.97522112
Convg = 0.7000D-09 -V/T = 2.0149

Temperature 195.150 Kelvin. Pressure 1.00000 Atm.
elec. and zero-point Energies= -2769.628865
elec. and thermal Energies= -2769.610569
elec. and thermal Enthalpies= -2769.609951
elec. and thermal Free Energies= -2769.670145

RB3LYP/6-31G(d)/MWB59/THF(IEFPCM) = -2769.99950000

RM06-2x/6-311+G(d,p)/MWB59/THF(IEFPCM) = -2769.80118136

Geometry at RB3LYP/6-31G(d)/MWB59
Charge = 0 Multiplicity = 1

```
Yb -0.2597916533  0.5144899271  2.9224395233
O   1.2585692198  2.2788908282  2.9921481066
S   0.3732945607  3.2895223613  3.7073861937
O   0.9557556311  4.0379585593  4.8047853728
O  -0.9209753731  2.5415119288  3.9622476429
C  -0.0672364601  4.5074891456  2.3653356513
F   1.0297090975  5.147186914  1.9689787548
F  -0.5951689135  3.8439343374  1.3293213701
F  -0.956552269  5.3796673964  2.8321892168
O   0.8856963454 -0.7495137791  4.5214233723
O  -0.0804800774 -3.0442813374  5.0436230319
O  -1.4529061652 -1.1106051313  4.1302458065
S  -0.2854796295 -1.6095563719  4.9657726789
C  -0.6646929177 -1.002125864  6.6885379626
F   0.3601766522 -1.2810096122  7.4886366075
F  -0.8611042872  0.3189185834  6.654204069
F  -1.7648245105 -1.6048554663  7.1332249705
C  -1.1765681526 -1.5291504069 -0.4957085751
C  -2.4632374294 -1.1168346271 -0.1305329268
C  -1.867230945 -0.0170505528  0.5638570024
C  -0.4512678685 -0.2621427579  0.0365117004
O   0.3769435353 -0.4182349196  1.135910108
O  -2.1509562524  0.6903724808  1.5654746352
O  -0.5310928903 -2.5510175397 -0.9793775736
C  -1.0944094735 -3.8974157034 -1.2764232284
C  -3.885678882 -1.5788173599 -0.1564737929
H  -4.0027105886 -2.6282692013  0.1304960688
H  -4.3356320367 -1.4529935157 -1.1489803043
H  -4.4658326859 -0.9751569156  0.5487446098
C   0.1123727606  0.6544805255 -1.0424560448
C   1.4263067807  1.1215023816 -0.9295798825
C  -0.6558324008  1.0423777516 -2.1502500661
H   2.0185156896  0.8182113538 -0.0737469128
H  -1.678619846  0.6896585736 -2.2546547628
C   1.9635168848  1.9612937518 -1.9079260507
C  -0.1196055445  1.8847198399 -3.1234002359
H   2.9860863185  2.3151145669 -1.8074113857
H  -0.7289304204  2.1819635637 -3.9728775806
C   1.1938074668  2.346420967 -3.0059809691
H   1.6115991258  3.0026570454 -3.7646647688
C  -2.1829475774 -3.7764077639 -2.3433508348
H  -3.0679836166 -3.2496783843 -1.9806950143
H  -1.7981221416 -3.2525986999 -3.2242336404
H  -2.4976194425 -4.778291935 -2.6542586868
C  -1.5789666387 -4.5233012117  0.0314098037
H  -1.9182883339 -5.5474755045 -0.1565947376
H  -0.7657578567 -4.5595895239  0.7627432304
H  -2.4115093754 -3.9695548041  0.4722321113
C   0.1249432466 -4.6415000877 -1.8228682084
H  -0.1478334044 -5.6727672225 -2.0687010868
H   0.5011262661 -4.1552373854 -2.7281333508
H   0.9278334571 -4.6601582792 -1.0802257308
```

Frequency: 11.0975; 13.6149; 20.1524; 21.7794; 26.1131; 28.3047; 30.7703; 32.6201; 41.9900; 43.2248; 49.4486; 51.5724; 58.8897; 64.5084; 92.3134; 100.6717; 121.9113; 131.6842; 143.4444; 145.6052; 162.1941; 163.9762; 179.6333; 185.4066; 193.2108; 202.7581; 212.3965; 217.2126; 225.5088; 233.3170; 237.9991; 241.0682; 252.6946; 261.6849; 264.0733; 303.7707; 306.0961; 316.7090; 320.0496; 320.4895; 320.8424; 339.8310; 353.0838; 365.2432; 366.4768; 389.6218; 408.8584; 418.5634; 454.3089; 463.7976; 485.4142; 490.7540; 491.4610; 501.7339; 532.2135; 533.7240; 543.0406; 557.4086; 558.2487; 578.9736; 583.5857; 625.1955; 633.2701; 633.8537; 635.5471; 683.1891; 693.7956; 714.3598; 727.6857; 759.6641; 765.1976; 766.3306; 785.5667; 812.6044; 845.0839; 864.9713; 893.0643; 927.5943; 936.4614; 936.8964; 953.7975; 961.4997; 975.0084; 981.6831; 983.6320; 1004.8705; 1017.4094; 1037.9229; 1055.2007; 1061.0356; 1061.2891; 1067.0888; 1070.0058; 1070.7087; 1080.9568; 1109.7778; 1192.4674; 1198.1455; 1199.5633; 1200.8070; 1208.8032; 1210.8519; 1222.1227; 1247.1878; 1254.0625; 1258.5207; 1271.3731; 1272.1860; 1288.0634; 1296.5176; 1324.7225; 1332.5882; 1349.6250; 1365.1620; 1412.4552; 1433.5647; 1435.8090; 1453.0414; 1460.6917; 1495.0590; 1501.1354; 1510.2647; 1514.5235; 1518.6043; 1520.3618; 1527.6288; 1530.7740; 1542.2799; 1548.1732; 1573.4779; 1641.6825; 1661.2456; 1741.6843; 3052.7388; 3066.0055; 3069.4524; 3075.5671; 3113.1157; 3133.2979; 3134.5689; 3137.8377; 3143.6556; 3147.8853; 3157.0199; 3166.9798; 3181.9032; 3190.8278; 3199.8945; 3211.0245; 3236.2618.

Cartesian Forces: Max 0.000001067 RMS 0.000000355

Item	Value	Threshold	Converged?
Maximum Force	0.000000	0.000450	YES
RMS Force	0.000000	0.000300	YES
Maximum Displacement	0.000006	0.001800	YES
RMS Displacement	0.000002	0.001200	YES

FILE: Yb_Sq2_PhYbOTf2_Prod12_Neutral_B3LYP_M062x_Conf3-.out

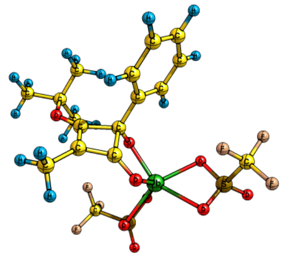

Conformer 7

E(RB3LYP/6-31G(d)/MWB59) = -2769.98002869
Convg = 0.2180D-08 -V/T = 2.0149

Temperature 195.150 Kelvin. Pressure 1.00000 Atm.
elec. and zero-point Energies= -2769.633722
elec. and thermal Energies= -2769.615397
elec. and thermal Enthalpies= -2769.614779
elec. and thermal Free Energies= -2769.674821

RB3LYP/6-31G(d)/MWB59/THF(IEFPCM) = -2770.00227581

RM06-2x/6-311+G(d,p)/MWB59/THF(IEFPCM) = -2769.80509726

Geometry at RB3LYP/6-31G(d)/MWB59
Charge = 0 Multiplicity = 1

```
Yb -0.3627975661  0.5295739791  2.9290937337
O   1.0895834761  2.3483082911  2.9218347933
S   0.1821916872  3.3428777348  3.6340527917
O   0.7591783883  4.1361496627  4.7017667589
O  -1.0769396212  2.5508397109  3.930619406
C  -0.3303526495  4.5149185134  2.2762300852
F   0.737225625  5.176237017  1.8364518111
F  -0.8649259697  3.8124525671  1.269946123
F  -1.23200226  5.371488445  2.7469437407
O  -1.5594061082 -1.0198233507  4.2286462748
O  -0.6007781199 -1.5280352572  6.5307486067
O   0.785054265 -0.6640617253  4.5805970902
S  -0.396664637 -1.4695626417  5.0966118459
C  -0.0504687188 -3.2106920973  4.5213554187
F  -1.0706114184 -3.9958393503  4.8578200378
F   0.0944790407 -3.2197170951  3.1896014408
F   1.0678739127 -3.6512705061  5.090091505
C  -1.02276779 -1.5716000497 -0.5809708782
C  -2.3280544604 -1.1829379551 -0.2848397145
C  -1.8127486485 -0.1051480268  0.4841910259
C  -0.3512150479 -0.2916833523  0.0426112441
O   0.4139024835 -0.4266532859  1.188455714
O  -2.1631969228  0.5877171165  1.4777946585
O  -0.6596453175 -2.718018266 -1.0904274964
C   0.7473764717 -3.1917710365 -1.2844940476
C  -3.6660119775 -1.8171456535 -0.4601528299
H  -3.5602907777 -2.8745669491 -0.7217094848
H  -4.2407715597 -1.3291687285 -1.2568178844
H  -4.2521144199 -1.7393440634  0.4619962659
C   0.2036816973  0.7226535538 -0.9548153064
C   1.4230362565  1.3581515052 -0.689614156
C  -0.4928767286  1.0528841935 -2.1279435014
H   1.9614870081  1.1072995909  0.2170528495
H  -1.4417587867  0.5728714656 -2.3510693527
C   1.937801446  2.3024112057 -1.5817581176
C   0.0207473791  1.9981992604 -3.0146548585
H   2.8851656918  2.7865354072 -1.3606878601
H  -0.5330851991  2.2457768318 -3.9163042806
C   1.2398476541  2.6257721569 -2.7454371194
H   1.639291212  3.3624052242 -3.4370760221
C   1.4249559197 -3.3123541686  0.0788089464
H   1.5144328013 -2.3404999592  0.5705995138
H   0.8602365942 -3.9843813333  0.7327096313
H   2.4282498083 -3.7315656922 -0.056054388
C   1.4830361008 -2.2462273674 -2.2319507679
H   2.4510119428 -2.6875745154 -2.4930534324
H   0.9148803321 -2.1014381449 -3.1565810373
H   1.6716174003 -1.2708313827 -1.7789955333
C   0.528634134 -4.5611134502 -1.9286689813
H   1.4953298319 -5.0406359355 -2.1117415785
H  -0.059797536 -5.2084372247 -1.2712216769
H   0.0033036805 -4.4643998682 -2.8841690067
```

Frequency: 12.6355; 15.9424; 18.5066; 20.5522; 26.2566; 29.2682; 31.3147; 40.7836; 43.8979; 49.1604; 53.0084; 54.1015; 57.5135; 68.8436; 72.7329; 82.7489; 102.0127; 105.6982; 135.4761; 159.6174; 162.0452; 166.0022; 185.5164; 189.3217; 195.1880; 212.2184; 213.5647; 214.0188; 225.6163; 231.2899; 238.2035; 239.4679; 264.1484; 269.7509; 278.1892; 304.4414; 305.0782; 313.2495; 319.6078; 319.7646; 329.3706; 343.5702; 349.9924; 364.4240; 366.1469; 384.9189; 416.1853; 421.1245; 453.0113; 453.1234; 490.5473; 491.0373; 495.2848; 515.8534; 525.2494; 532.0865; 538.0083; 557.3291; 557.3870; 576.9612; 585.6255; 592.2214; 622.7523; 633.0644; 636.0475; 670.6102; 691.8405; 715.0837; 723.2431; 763.0536; 764.8542; 765.2296; 778.9889; 801.0334; 861.7349; 866.8607; 896.6490; 923.3180; 938.6196; 943.1107; 951.8400; 960.6869; 963.6774; 980.1453; 989.8373; 1005.5334; 1016.7250; 1042.3143; 1055.1271; 1059.4644; 1068.0889; 1071.0162; 1072.2449; 1073.6444; 1076.3733; 1110.3664; 1192.8298; 1195.9693; 1199.0302; 1201.0140; 1209.7666; 1213.1130; 1243.3231; 1244.4540; 1247.4206; 1260.2605; 1271.6441; 1272.1161; 1289.6462; 1301.2195; 1327.9839; 1336.1928; 1347.3410; 1364.7892; 1414.2908; 1434.6333; 1440.7320; 1456.4875; 1465.2675; 1493.7859; 1503.4835; 1507.9247; 1513.9645; 1515.7202; 1520.4053; 1531.0230; 1533.5816; 1540.4295; 1550.5586; 1571.6216; 1639.7523; 1659.1531; 1745.1948; 3052.8836; 3061.9131; 3063.6922; 3070.6247; 3110.8465; 3129.6739; 3131.4738; 3137.0704; 3139.5086; 3143.2355; 3156.9634; 3165.4703; 3185.3616; 3194.0256; 3202.7658; 3213.0105; 3237.0264.

Cartesian Forces: Max 0.000000533 RMS 0.000000175

Item	Value	Threshold	Converged?
Maximum Force	0.000000	0.000450	YES
RMS Force	0.000000	0.000300	YES
Maximum Displacement	0.000006	0.001800	YES
RMS Displacement	0.000001	0.001200	YES

FILE: Yb_Sq2_PhYbOTf2_Prod12_Neutral_B3LYP_M062x_Conf4.out

C-2 addition product from $PhYb(OTf)_2$ + 1b

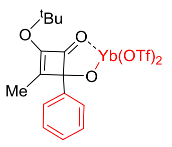

Lowest energy conformation is: Conformer 1

Conformer 8

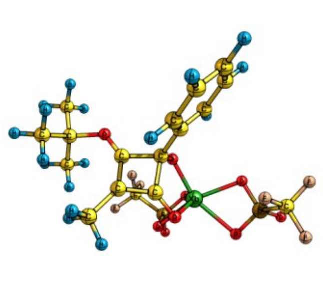

```
E(RB3LYP/6-31G(d)/MWB59) = -2769.97482206
 Convg = 0.5154D-08 -V/T = 2.0149

 Temperature 195.150 Kelvin. Pressure 1.00000 Atm.
     elec. and zero-point Energies= -2769.628409
     elec. and thermal Energies= -2769.610173
     elec. and thermal Enthalpies= -2769.609555
     elec. and thermal Free Energies= -2769.669175

RB3LYP/6-31G(d)/MWB59/THF(IEFPCM) =  -2769.99986523

RM06-2x/6-311+G(d,p)/MWB59/THF(IEFPCM) =  -2769.80129937
```

```
Geometry at RB3LYP/6-31G(d)/MWB59
Charge = 0 Multiplicity = 1
 Yb -0.4424100234  0.4676429323  2.9389739946
 O   1.1569227128  2.1490617797  3.1099485037
 S   0.3040389968  3.174093201  3.8449070221
 O   0.8968464171  3.8564890557  4.9786245706
 O  -1.0281587174  2.4772886172  4.046413162
 C  -0.0527888892  4.4563818079  2.5380958504
 F   1.0794664324  5.0610293181  2.188848206
 F  -0.5838838881  3.852529543  1.4673803153
 F  -0.9148399822  5.3494500224  3.0158156358
 O  -1.752034527 -1.1700771111  4.0107707257
 O  -0.9461734877 -1.9621596201  6.2894966075
 O   0.5717545118 -0.9093049178  4.5405469717
 S  -0.650832372 -1.7411174887  4.8872474264
 C  -0.2971016132 -3.413749509  4.1372208884
 F  -1.3825965159 -4.1795950967  4.2400930833
 F   0.018071621 -3.2707292523  2.8426973315
 F   0.7193207777 -3.9817921042  4.7783526105
 C  -1.3579196505 -1.4387999284 -0.5414823977
 C  -2.6311171392 -0.9578012694 -0.2176921709
 C  -1.9966477944  0.0950485304  0.5150918308
 C  -0.5807027182 -0.2165883277  0.0230241237
 O   0.2095835621 -0.4282948931  1.139736943
 O  -2.2703734809  0.7917339377  1.526906635
 O  -0.7541124214 -2.4971371536 -1.0015397366
 C  -1.3767717513 -3.8185495115 -1.2902616422
 C  -4.0760719855 -1.334611911 -0.3034275696
 H  -4.2685153903 -2.3748668168 -0.0237303015
 H  -4.4746633089 -1.1834064096 -1.314162223
 H  -4.6487253974 -0.6968343553  0.3773861818
 C   0.0568005106  0.6812366712 -1.0305006926
 C   1.3860637387  1.0876108699 -0.872112827
 C  -0.6577135311  1.112275761 -2.1580672949
 H   1.9365728581  0.7512103489 -0.0009908843
 H  -1.6914871043  0.8072513975 -2.2979055827
 C   1.9911654719  1.9101381251 -1.8251416477
 C  -0.0535486439  1.9371904476 -3.10600409
 H   3.0247705735  2.2168765169 -1.6891170319
 H  -0.6216674052  2.2682766399 -3.9713617412
 C   1.2748148286  2.3382974651 -2.9431545082
 H   1.7454402761  2.9810954849 -3.6821354177
 C  -2.4100367076 -3.6659753507 -2.4067222646
 H  -3.2714230987 -3.0693155538 -2.0988735661
 H  -1.956039569 -3.1993105514 -3.2867234854
 H  -2.7786227243 -4.6552936738 -2.6981297759
 C  -1.9523116402 -4.3853823073  0.0081083453
 H  -2.2920045176 -5.4126289273 -0.161471116
 H  -1.1901292229 -4.3982108129  0.7926332396
 H  -2.8056753049 -3.8070945422  0.3704574036
 C  -0.177025 -4.640782977 -1.7632816701
 H  -0.4966965708 -5.6583391075 -2.0096311259
 H   0.2720303618 -4.1912735024 -2.6541460684
 H   0.5853501236 -4.6936564486 -0.9805595269
```

Frequency: 13.2888; 14.5143; 20.9038; 24.8484; 26.0855; 29.3248; 31.2655; 37.6267; 43.9063; 44.2976; 50.0183; 57.2194; 60.6866; 69.3740; 94.4942; 99.0505; 112.2906; 130.6055; 142.9957; 149.0322; 161.2066; 167.4185; 185.1955; 186.9139; 191.6259; 204.1947; 211.9519; 214.7285; 225.8389; 235.5674; 238.2513; 239.1944; 255.9260; 262.1559; 263.6323; 303.0200; 305.0288; 315.0533; 319.9086; 320.1126; 321.3159; 341.0226; 354.1469; 363.2942; 365.8597; 390.9702; 408.7381; 418.4037; 455.0585; 464.2600; 486.3252; 490.6708; 491.7715; 501.5046; 531.7796; 534.8223; 543.3300; 557.3303; 557.9092; 577.1755; 585.8069; 622.2819; 633.2247; 634.9804; 635.0916; 683.0374; 694.0042; 714.1453; 727.0499; 759.1468; 764.1255; 765.0703; 785.7894; 811.7283; 845.6635; 864.7075; 892.9854; 927.2413; 936.6752; 937.5949; 952.3686; 961.6566; 974.5620; 982.0143; 982.6406; 1004.6787; 1017.4139; 1036.7101; 1054.3954; 1061.4126; 1061.7497; 1067.3887; 1070.0738; 1070.4063; 1080.4779; 1109.7103; 1192.4830; 1197.3496; 1199.3611; 1201.4921; 1208.3843; 1210.5339; 1222.5045; 1240.3486; 1246.8617; 1257.5984; 1269.5881; 1272.1993; 1287.6111; 1296.3129; 1327.8838; 1336.0880; 1349.3442; 1365.0464; 1411.5126; 1434.4133; 1436.4587; 1452.6791; 1461.5793; 1494.9402; 1501.5901; 1510.4391; 1515.6161; 1518.1001; 1519.9798; 1529.0282; 1530.6769; 1542.2693; 1548.7683; 1574.1443; 1641.7096; 1661.2286; 1740.5532; 3053.0643; 3066.1876; 3070.5091; 3077.2694; 3113.2980; 3133.7498; 3134.8313; 3140.6149; 3145.0422; 3147.7191; 3160.0085; 3164.9338; 3182.1967; 3191.1029; 3200.1090; 3211.1807; 3236.5956.

```
 Cartesian Forces:  Max     0.000000441 RMS     0.000000152

         Item               Value     Threshold  Converged?
 Maximum Force            0.000000     0.000450     YES
 RMS     Force            0.000000     0.000300     YES
 Maximum Displacement     0.000028     0.001800     YES
 RMS     Displacement     0.000005     0.001200     YES
```

FILE: Yb_Sq2_PhYbOTf2_Prod12_Neutral_B3LYP_M062x_Conf4-.out

Conformer 1

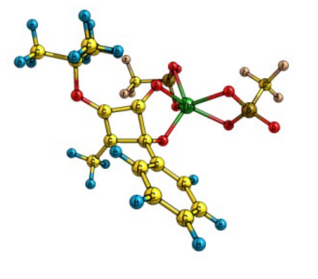

```
E(RB3LYP/6-31G(d)/MWB59) = -2769.97095774
 Convg = 0.7639D-08 -V/T = 2.0149

 Temperature 195.150 Kelvin. Pressure 1.00000 Atm.
     elec. and zero-point Energies= -2769.625100
     elec. and thermal Energies= -2769.606772
     elec. and thermal Enthalpies= -2769.606154
     elec. and thermal Free Energies= -2769.666542

RB3LYP/6-31G(d)/MWB59/THF(IEFPCM) =  -2769.99484681

RM06-2x/6-311+G(d,p)/MWB59/THF(IEFPCM) =  -2769.79782291
```

```
Geometry at RB3LYP/6-31G(d)/MWB59
Charge = 0 Multiplicity = 1
 Yb -1.0774448283  1.710304922  2.1514436976
 O  -2.0943908708  3.75637063  2.6307648895
 S  -0.8689379276  4.6319584303  2.4242768844
 O  -1.0310426095  5.8591479042  1.6698502746
 O   0.2084468044  3.6568933933  1.9772940926
 C  -0.3593411754  5.0858040683  4.1601465483
 F  -1.3001856385  5.8393361241  4.7189047011
 F  -0.2092618134  3.957785556  4.86895716
 F   0.7940879046  5.7455792562  4.1280195231
 O  -2.3805014742  0.6575465304  3.7934446408
 O  -1.2642762604  0.0074531963  5.9864887019
 O   0.0072882287  0.4451614595  3.8254551476
 S  -1.2475243061 -0.0258360053  4.5372939431
 C  -1.392293006 -1.8104667082  4.0144735185
 F  -2.5146773609 -2.3340244185  4.4949145532
 F  -1.4156307031 -1.8697240406  2.6710639426
 F  -0.3427291103 -2.4962348868  4.4599638494
 C  -0.1723780054 -1.1288043561 -0.778540176
 C  -1.4943487124 -0.8428648486 -1.0327437285
 C  -1.3981609559  0.6517058788 -0.660857024
 C  -0.0293329814  0.1777002174 -0.1486628593
 O   0.5852793071  0.6681321149  0.82440267
 O  -2.1232119954  1.0726265472  0.4484853353
 O   0.502904357 -2.260976105 -0.9409068976
 C   1.8263148192 -2.4856631573 -0.3152126454
 C  -2.6773004094 -1.6711654046 -1.367011888
 H  -2.4248316817 -2.7331254611 -1.4304560588
 H  -3.1030556193 -1.3467267049 -2.3260476276
 H  -3.4581206784 -1.5285244752 -0.6099786658
 C   2.8203149057 -1.4313849565 -0.809648835
 H   3.824860153 -1.6822646786 -0.452333748
 H   2.8449733459 -1.4079037604 -1.9043054736
 H   2.5805808897 -0.4329694038 -0.4336276882
 C   2.1977101106 -3.877373447 -0.828899136
 H   3.1726037068 -4.1759109363 -0.430869307
 H   1.4533231403 -4.6153404753 -0.513702949
 H   2.2512924342 -3.8845595076 -1.9221866626
 C   1.6770028971 -2.4766505526  1.2085341528
 H   1.4402414526 -1.477885959  1.5841518743
 H   0.8921334832 -3.1719211838  1.523248972
 H   2.6185013584 -2.7926149151  1.671027521
 C  -1.4496044172  1.6298906199 -1.8295635073
 C  -0.7071341049  1.4059318187 -2.9990811541
 C  -2.252641755  2.7733419427 -1.7463924383
 H  -0.0838648864  0.5203782747 -3.0883088505
 H  -2.8385663437  2.9420444506 -0.8499503719
 C  -0.7591967135  2.3134535884 -4.0564778862
 C  -2.3074778567  3.6788965825 -2.8084658774
 H  -0.1732798424  2.1283937527 -4.952909505
 H  -2.9353010804  4.5619598105 -2.7269539722
 C  -1.5604975659  3.4540454454 -3.9649452491
 H  -1.6009086086  4.1608198333 -4.7892164115
```

Frequency: 10.5910; 15.2678; 17.3732; 19.4463; 24.0336; 28.8676; 31.3868; 38.5710; 41.7085; 45.3449; 46.9059; 52.8520; 59.8003; 63.8365; 77.4947; 86.0752; 103.0539; 122.3535; 143.9051; 156.3161; 162.9907; 169.6863; 182.8338; 191.2747; 197.1413; 201.3020; 213.6984; 216.8826; 224.9471; 232.8725; 237.2990; 253.0847; 265.0042; 267.0457; 273.2057; 290.3523; 304.1421; 306.1950; 319.1524; 319.9809; 321.3202; 342.0564; 362.4699; 365.8059; 367.4246; 388.2236; 418.1690; 424.9014; 451.1865; 464.8012; 487.4962; 490.9030; 491.2221; 532.2755; 533.5454; 537.9312; 549.7070; 556.8436; 557.0479; 580.3187; 584.1009; 595.4442; 624.3369; 631.3116; 633.3836; 634.5106; 667.9187; 711.4424; 716.1176; 752.1014; 762.1920; 763.3371; 765.3266; 797.2832; 863.0222; 864.9056; 925.8152; 933.3308; 936.3992; 945.4564; 954.1131; 961.0873; 970.9834; 978.5919; 984.2633; 1005.2455; 1017.1414; 1029.8511; 1053.6620; 1058.6236; 1060.9850; 1064.4451; 1070.3894; 1074.2044; 1084.9404; 1109.3015; 1143.2370; 1193.3042; 1193.7349; 1196.7695; 1210.3390; 1211.2212; 1230.6977; 1237.2970; 1246.9253; 1268.5241; 1274.3257; 1277.5554; 1283.1563; 1300.0880; 1330.4738; 1337.6767; 1348.4209; 1365.2009; 1380.6294; 1430.2397; 1433.1777; 1433.5858; 1459.7335; 1494.0500; 1498.1017; 1503.4251; 1504.5910; 1515.9807; 1517.4704; 1534.3080; 1535.3853; 1542.2379; 1550.8949; 1614.8765; 1641.0586; 1660.8470; 1739.0099; 3044.2619; 3061.4279; 3062.5746; 3069.4912; 3099.7008; 3128.6368; 3130.7589; 3138.1198; 3141.9966; 3144.5943; 3148.3063; 3156.3262; 3184.1552; 3193.5465; 3202.1922; 3212.3940; 3233.4340.

```
 Cartesian Forces:  Max     0.000001949 RMS     0.000000675

         Item               Value     Threshold  Converged?
 Maximum Force            0.000000     0.000450     YES
 RMS     Force            0.000000     0.000300     YES
 Maximum Displacement     0.000010     0.001800     YES
 RMS     Displacement     0.000002     0.001200     YES
```

FILE: Yb_Sq2_PhYbOTf2_Prod56_Neutral_B3LYP_M062x_Conf1.out

Conformer 2

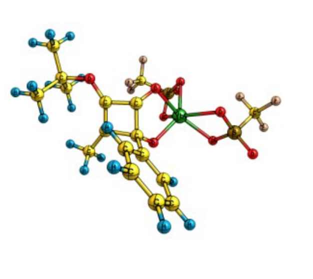

```
E(RB3LYP/6-31G(d)/MWB59) = -2769.96387234
 Convg = 0.9419D-08 -V/T = 2.0149

 Temperature 195.150 Kelvin. Pressure 1.00000 Atm.
     elec. and zero-point Energies= -2769.618049
     elec. and thermal Energies= -2769.599720
     elec. and thermal Enthalpies= -2769.599102
     elec. and thermal Free Energies= -2769.659395

RB3LYP/6-31G(d)/MWB59/THF(IEFPCM) =  -2769.98854585

RM06-2x/6-311+G(d,p)/MWB59/THF(IEFPCM) =  -2769.79145532
```

```
Geometry at RB3LYP/6-31G(d)/MWB59
Charge = 0 Multiplicity = 1
 Yb -1.1532467798  1.6274943127  2.1021564136
 O  -2.0799367404  3.6166414869  2.8840689376
 S  -0.9157065488  4.5276369895  2.5224310584
 O  -1.2190452708  5.7661042889  1.8316043294
 O   0.1011510335  3.5915684559  1.889491039
 C  -0.1625619063  4.9685283314  4.1717820014
 F  -1.0309095278  5.6916191794  4.872361369
 F   0.1227439715  3.8432905068  4.8347454778
 F   0.9532704339  5.6642434659  3.9731501165
 O  -2.4175216534  0.2640834132  3.5179253056
 O  -1.5350519315 -0.1607444925  5.8665864433
 O  -0.0654508639  0.5303473228  3.9086477992
 S  -1.2921867104 -0.1838812105  4.4371570008
 C  -1.0232870196 -1.9654065785  3.9529768129
 F  -2.0903941764 -2.6858447748  4.2879065623
 F  -0.8432623052 -2.0397889302  2.6228901583
 F   0.0551545946 -2.4402968719  4.5674816469
 C  -0.5776107672 -1.1657324537 -0.8956956754
 C  -1.8556549359 -0.716889385 -1.1637120489
 C  -1.5625349349  0.7592265329 -0.7625009552
 C  -0.284544614  0.1018229595 -0.2466067006
 O   0.3709719  0.4568639105  0.7561115938
 O  -2.247372549  1.2478736887  0.3419312521
 O   0.2173407397 -2.2300428915 -0.9101521103
 C  -0.1981093771 -3.5930204948 -1.2792559338
 C  -3.2065869155 -1.231378465 -1.5065683282
 H  -3.2544111874 -2.3093510666 -1.6665405084
 H  -3.5658562109 -0.7241357154 -2.4120695356
 H  -3.9027393114 -0.963928583 -0.7015466753
 C  -1.2391766987 -4.0951443148 -0.2741475963
 H  -1.451033968 -5.1541206841 -0.4562486153
 H  -0.8600237831 -3.9883623425  0.7467043246
 H  -2.1838124958 -3.5499802826 -0.3436515782
 C   1.1059772349 -4.3826306045 -1.1396482421
 H   0.9378102443 -5.43495909 -1.3901262497
 H   1.8712501634 -3.9820878628 -1.8115268775
 H   1.4823202584 -4.3233701912 -0.113964586
 C  -0.6882336483 -3.6180375023 -2.7297676408
 H  -1.5911598669 -3.0203840231 -2.8775714422
 H   0.0894440975 -3.2381564226 -3.4001640183
 H  -0.9189694395 -4.6475529765 -3.0238153099
 C  -1.4936521356  1.741313347 -1.9259880661
 C  -0.7392234547  1.4595117952 -3.0750632094
 C  -2.2009148791  2.9470457902 -1.8588640088
 H  -0.1862882172  0.5270668685 -3.1495235264
 H  -2.7906137799  3.1624260348 -0.9751153039
 C  -0.6866071224  2.3707902375 -4.1292126845
 C  -2.152454843  3.8558425646 -2.9183582521
 H  -0.0907777694  2.1415853701 -5.008769099
 H  -2.70674472  4.7879024172 -2.8499050977
 C  -1.3947821042  3.5726748915 -4.054976335
 H  -1.3535227554  4.2824823446 -4.8766346365
```

Frequency: 9.1527; 17.1813; 20.3085; 23.3879; 26.7459; 27.7740; 31.1226; 31.7474; 41.8277; 43.0121; 47.9891; 54.2495; 59.9475; 63.0257; 78.8153; 87.0977; 103.7635; 126.2952; 154.6699; 160.5730; 166.5273; 169.4671; 181.0601; 183.7819; 194.8284; 195.7876; 216.5716; 223.9488; 226.1808; 234.6428; 235.3058; 242.3163; 260.0844; 262.3171; 267.0884; 289.1932; 304.1882; 306.0171; 314.0408; 319.7089; 319.9570; 345.7297; 364.0516; 366.5535; 369.8979; 379.0581; 418.3463; 426.2975; 456.7065; 474.1093; 476.7081; 490.4805; 491.3205; 521.0008; 532.3386; 536.7447; 539.3029; 556.4781; 557.9898; 578.9904; 582.4794; 593.8155; 624.9999; 633.2702; 634.2084; 647.3636; 680.8792; 714.1863; 726.7249; 748.4828; 763.5228; 766.1816; 767.3936; 798.5021; 835.9799; 864.9101; 922.6175; 930.9237; 933.3445; 953.1725; 958.3852; 961.2227; 965.9342; 978.0445; 978.9658; 1005.4023; 1017.3847; 1031.8728; 1049.7182; 1057.9658; 1058.8233; 1060.3095; 1069.6913; 1073.7317; 1095.0316; 1113.3869; 1128.8735; 1192.9561; 1196.0245; 1199.0179; 1210.5328; 1214.4584; 1227.2902; 1237.3666; 1253.3721; 1263.5073; 1273.2630; 1274.4250; 1282.9110; 1292.9134; 1327.8521; 1335.2217; 1348.5441; 1364.7417; 1365.9845; 1429.5164; 1432.9092; 1434.0839; 1459.3004; 1494.2264; 1497.4367; 1503.4552; 1505.6962; 1516.7564; 1518.2769; 1528.9756; 1529.4032; 1542.2480; 1549.1820; 1618.8064; 1641.2708; 1660.8797; 1741.2056; 3046.3345; 3064.3209; 3067.6744; 3074.6510; 3097.0157; 3132.1106; 3137.1484; 3142.9077; 3144.5455; 3151.8739; 3153.3685; 3166.1899; 3183.6223; 3192.9983; 3201.7207; 3212.0625; 3235.0273.

```
 Cartesian Forces:  Max     0.000001424 RMS     0.000000414

         Item               Value     Threshold  Converged?
 Maximum Force            0.000000     0.000450     YES
 RMS     Force            0.000000     0.000300     YES
 Maximum Displacement     0.000011     0.001800     YES
 RMS     Displacement     0.000001     0.001200     YES
```

FILE: Yb_Sq2_PhYbOTf2_Prod56_Neutral_B3LYP_M062x_Conf1-.out

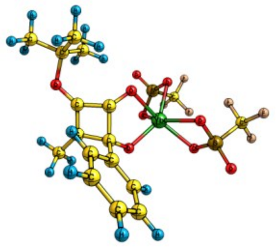

Conformer 3

E(RB3LYP/6-31G(d)/MWB59) = -2769.97077866
Convg = 0.8981D-08 -V/T = 2.0149

Temperature 195.150 Kelvin. Pressure 1.00000 Atm.
elec. and zero-point Energies= -2769.625070
elec. and thermal Energies= -2769.606668
elec. and thermal Enthalpies= -2769.606050
elec. and thermal Free Energies= -2769.667014

RB3LYP/6-31G(d)/MWB59/THF(IEFPCM) = -2769.99256083

RM06-2x/6-311+G(d,p)/MWB59/THF(IEFPCM) = -2769.79572450

Geometry at RB3LYP/6-31G(d)/MWB59
Charge = 0 Multiplicity = 1
Yb -1.0444196509 1.7291607671 2.1742335856
O -2.2387584066 3.5865392329 2.917918573
S -1.2426291022 4.6479112137 2.4715197297
O -1.7587524484 5.7827783783 1.7305512036
O -0.1052541881 3.8530871651 1.8527741713
C -0.5297869966 5.2981456498 4.068777584
F -1.4907070785 5.8968083081 4.7668047792
F -0.0324588295 4.2781591885 4.7754222908
F 0.4406304501 6.1650168773 3.7946792472
O 0.2766981153 1.0139319995 4.0334780867
O -0.2348445185 -1.3343678768 4.859954389
O -1.902481006 0.1058408404 3.5997687282
S -0.7192501661 -0.000182273 4.5541855331
C -1.3568524481 0.7403142475 6.1431835843
F -0.3588558209 0.8250801991 7.0188289537
F -1.84014952 1.9621215145 5.8966909148
F -2.3246395606 -0.0306340076 6.6310900314
C -0.1956305039 -1.1213022611 -0.7374657271
C -1.5052300057 -0.789628917 -1.0044477916
C -1.3580665555 0.7060772364 -0.6505612209
C -0.0207999749 0.1784798381 -0.1074380765
O 0.5872430959 0.6436747703 0.8870970543
O -2.0875327545 1.1739348373 0.4338989181
O 0.4426233285 -2.2743284148 -0.893035492
C 1.7517866447 -2.5452744123 -0.2527644451
C -2.716762845 -1.5803105356 -1.3265491243
H -2.5001381876 -2.6504707175 -1.3826431968
H -3.1369601292 -1.2479306436 -2.2852620451
H -3.4869020987 -1.4061384816 -0.5652122943
C 2.7797381126 -1.505932588 -0.7077357016
H 3.7723274169 -1.7956781136 -0.3465297138
H 2.8183762976 -1.4538262767 -1.801086426
H 2.5649349788 -0.5116245686 -0.306890938
C 2.0970249165 -3.9338811589 -0.7922819088
H 3.0540338864 -4.2683080483 -0.3796418864
H 1.326136573 -4.6582103056 -0.5113351985
H 2.1756344949 -3.9168136571 -1.8840034452
C 1.5742350545 -2.5673699623 1.267149677
H 1.3296797613 -1.5767519105 1.6594917237
H 0.7848441296 -3.2699444005 1.5530339271
H 2.5061418602 -2.8909158617 1.7432966754
C -1.3468237681 1.6633778639 -1.8382459127
C -0.5707057454 1.4028062356 -2.9778357701
C -2.1302837355 2.8225226573 -1.803652591
H 0.0378803639 0.504207262 -3.0300630044
H -2.740416934 3.0198891424 -0.9297379276
C -0.5703988613 2.2903355343 -4.0534410657
C -2.133510527 3.7075009199 -2.8841178901
H 0.0414108999 2.0770587591 -4.9260069418
H -2.7468458446 4.6033359583 -2.8401387955
C -1.353027424 3.4465759526 -4.010596191
H -1.353053745 4.1375708437 -4.8491596397

Frequency: 8.6375; 14.3930; 16.9878; 20.8289; 23.3650; 26.3776; 28.9330; 32.1345; 40.4487; 42.3468; 43.6893; 53.7978; 58.3634; 64.8788; 77.9226; 84.9741; 99.5775; 118.9656; 139.0307; 153.7161; 162.5751; 165.7763; 177.1430; 193.0178; 196.9440; 200.5516; 212.6504; 217.0828; 220.1594; 237.0411; 245.9038; 251.9410; 265.5568; 265.9217; 272.8452; 292.5450; 303.1975; 306.2639; 319.5136; 320.0516; 321.8801; 340.5753; 363.3858; 364.9141; 368.8790; 388.2754; 418.1815; 423.5014; 451.2260; 465.0881; 487.9129; 491.2721; 491.4882; 532.2468; 532.7194; 537.8705; 550.1245; 558.0379; 558.1309; 577.8902; 581.6137; 596.5259; 622.0680; 633.1767; 633.6588; 637.6950; 668.6796; 711.0225; 715.9430; 750.5596; 760.7746; 765.8962; 766.3000; 796.8868; 861.9476; 864.7819; 924.3354; 933.0040; 935.7417; 944.9080; 950.6081; 962.7002; 968.3962; 978.1836; 983.4973; 1005.2362; 1017.3326; 1029.5194; 1052.8091; 1058.1220; 1060.8093; 1063.1641; 1069.4525; 1070.9323; 1083.9195; 1108.8757; 1143.9069; 1192.9940; 1198.8033; 1201.4903; 1209.1489; 1211.0399; 1232.1946; 1247.8023; 1255.6871; 1266.2762; 1272.5309; 1273.9314; 1283.8696; 1299.5585; 1321.9867; 1332.4428; 1348.3534; 1364.9887; 1381.4917; 1429.5621; 1432.0772; 1433.1549; 1458.9482; 1494.0402; 1497.4367; 1502.6170; 1504.2005; 1515.9609; 1516.8285; 1532.6944; 1535.3795; 1542.1933; 1550.3341; 1603.5369; 1641.2597; 1660.8241; 1734.1815; 3044.7384; 3061.3741; 3063.1055; 3069.9238; 3100.6747; 3128.3024; 3131.6008; 3138.2568; 3142.2592; 3145.8390; 3148.9070; 3154.4568; 3183.7734; 3193.2937; 3202.0193; 3212.0539; 3235.6056.

Cartesian Forces: Max 0.000001229 RMS 0.000000367

Item	Value	Threshold	Converged?
Maximum Force	0.000000	0.000450	YES
RMS Force	0.000000	0.000300	YES
Maximum Displacement	0.000008	0.001800	YES
RMS Displacement	0.000002	0.001200	YES

FILE: Yb_Sq2_PhYbOTf2_Prod56_Neutral_B3LYP_M062x_Conf2.out

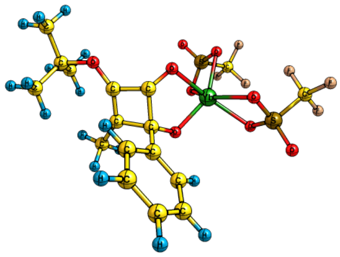

Conformer 4

E(RB3LYP/6-31G(d)/MWB59) = -2769.96364652
Convg = 0.1177D-08 -V/T = 2.0149

Temperature 195.150 Kelvin. Pressure 1.00000 Atm.
elec. and zero-point Energies= -2769.617907
elec. and thermal Energies= -2769.599529
elec. and thermal Enthalpies= -2769.598911
elec. and thermal Free Energies= -2769.659613

RB3LYP/6-31G(d)/MWB59/THF(IEFPCM) = -2769.98729251

RM06-2x/6-311+G(d,p)/MWB59/THF(IEFPCM) = -2769.79090350

Geometry at RB3LYP/6-31G(d)/MWB59
Charge = 0 Multiplicity = 1
Yb -1.0293287483 1.6922550478 2.1362527742
O -2.0275237913 3.647955789 2.9207197081
S -0.9393303917 4.6128874611 2.471771427
O -1.3526260763 5.8031414195 1.753303679
O 0.1060692074 3.7198597602 1.8254936656
C -0.1430365055 5.1688920149 4.0651316579
F -1.0316136014 5.8470529767 4.786393628
F 0.2647832829 4.0964792417 4.7507907459
F 0.9016551603 5.9431061252 3.7862993034
O 0.1935207325 0.830760794 3.9977111363
O -0.5706077563 -1.4509955782 4.8227562022
O -2.062688853 0.1527672248 3.5397389849
S -0.907288713 -0.074748593 4.5074108207
C -1.4815109441 0.7328645819 6.0878000965
F -0.4923193743 0.7098809163 6.9772307629
F -1.8264501823 1.999798999 5.8361241558
F -2.5339766034 0.0721003908 6.5630729547
C -0.5446535612 -1.1556208521 -0.8325112913
C -1.8309401825 -0.7077355803 -1.0618615597
C -1.5216899261 0.7742683017 -0.6965709734
C -0.2352239772 0.1166752702 -0.2023670057
O 0.4421240237 0.4818188742 0.7839424799
O -2.1763077523 1.2913039059 0.4118987402
O 0.246844011 -2.2209734199 -0.8629577749
C -0.1518751644 -3.5593696991 -1.3304001818
C -3.1929012041 -1.2286786956 -1.3463925964
H -3.2531553952 -2.3142451564 -1.4350538974
H -3.5670545805 -0.7772813918 -2.2754442296
H -3.8683713844 -0.9030270508 -0.5455736824
C -1.1429269364 -4.1646180754 -0.3321179084
H -1.3874059099 -5.1912611477 -0.6254154815
H -0.7036395159 -4.1887938002 0.669950841
H -2.077445021 -3.6002329398 -0.2799529144
C 1.1731128728 -4.32562012 -1.3025688595
H 1.013652437 -5.364515645 -1.6087871308
H 1.8972597098 -3.8681845074 -1.9837499755
H 1.5966723271 -4.3201410526 -0.2938318386
C -0.6968512554 -3.4822287434 -2.7594294549
H -1.632541705 -2.9216639427 -2.8247736142
H 0.0362652082 -3.0099549852 -3.4214704152
H -0.8898055998 -4.4936601033 -3.1324595834
C -1.4730802892 1.7322720997 -1.8818867384
C -0.743121747 1.4259584395 -3.0403976089
C -2.1759635338 2.9410449544 -1.824395817
H -0.1936629651 0.4909471871 -3.1078709931
H -2.7460434157 3.1757439024 -0.9327974723
C -0.7102279531 2.3159368324 -4.1134128158
C -2.1475924775 3.8283912971 -2.9026156745
H -0.1330087838 2.0677407895 -5.0002191218
H -2.6981736852 4.7631406515 -2.8411042245
C -1.4142470748 3.5207947462 -4.0487926978
H -1.3884532692 4.2139560403 -4.8851805334

Frequency: 10.1946; 14.3369; 19.3820; 22.1318; 24.5905; 26.4823; 27.7658; 30.5977; 40.8767; 43.4315; 45.7004; 54.7021; 56.0565; 64.5215; 78.9628; 84.6525; 104.2252; 124.8910; 151.6416; 163.2151; 164.0350; 166.6276; 178.0035; 181.2625; 194.8058; 198.3180; 217.5198; 219.8698; 224.4066; 235.4498; 236.4221; 244.9415; 260.7315; 262.2079; 269.2693; 291.5783; 303.1834; 306.2269; 316.3432; 319.6477; 320.3426; 343.8431; 363.2938; 366.4767; 369.5577; 379.5512; 418.3594; 426.8918; 455.8334; 473.9523; 477.2012; 491.1464; 491.3927; 517.5544; 532.3816; 536.7247; 541.2149; 557.8819; 558.1595; 578.2638; 581.6050; 596.8947; 622.0093; 633.3130; 637.1820; 647.6905; 679.7134; 714.0491; 724.9488; 749.3463; 765.7713; 766.0105; 766.2730; 799.1226; 835.4261; 864.5730; 921.6516; 930.9213; 932.5310; 950.7187; 958.3259; 962.4695; 964.7957; 977.6442; 978.9387; 1005.1485; 1017.3858; 1031.4101; 1049.6721; 1058.6719; 1058.9322; 1060.2834; 1069.0639; 1069.7747; 1095.0823; 1112.8863; 1129.4823; 1192.8439; 1199.2520; 1201.8629; 1210.4587; 1213.9790; 1228.5908; 1247.6324; 1255.4346; 1262.0633; 1271.5358; 1273.2658; 1283.1396; 1292.9898; 1323.4061; 1332.9476; 1348.4316; 1364.7280; 1366.9998; 1429.1431; 1432.5323; 1433.9995; 1459.1378; 1494.1703; 1496.9975; 1503.1342; 1505.9562; 1516.6937; 1517.9320; 1528.2496; 1530.0528; 1542.1198; 1549.3562; 1615.3840; 1641.2756; 1660.8044; 1739.4180; 3045.2236; 3064.1851; 3066.7871; 3073.3886; 3098.1416; 3131.5986; 3134.8255; 3141.8981; 3144.8276; 3151.3746; 3153.5427; 3166.4217; 3183.3277; 3192.7997; 3201.5446; 3211.8461; 3235.7026.

Cartesian Forces: Max 0.000001051 RMS 0.000000307

Item	Value	Threshold	Converged?
Maximum Force	0.000000	0.000450	YES
RMS Force	0.000000	0.000300	YES
Maximum Displacement	0.000005	0.001800	YES
RMS Displacement	0.000001	0.001200	YES

FILE: Yb_Sq2_PhYbOTf2_Prod56_Neutral_B3LYP_M062x_Conf2-.out

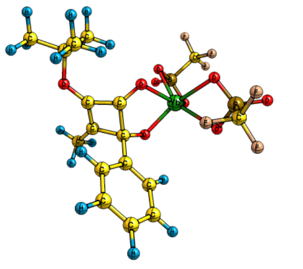

Conformer 5

E(RB3LYP/6-31G(d)/MWB59) = -2769.97174497
Convg = 0.4723D-08 -V/T = 2.0149

Temperature 195.150 Kelvin. Pressure 1.00000 Atm.
elec. and zero-point Energies= -2769.625905
elec. and thermal Energies= -2769.607564
elec. and thermal Enthalpies= -2769.606946
elec. and thermal Free Energies= -2769.667361

RB3LYP/6-31G(d)/MWB59/THF(IEFPCM) = -2769.99295491

RM06-2x/6-311+G(d,p)/MWB59/THF(IEFPCM) = -2769.79544438

Geometry at RB3LYP/6-31G(d)/MWB59
Charge = 0 Multiplicity = 1
Yb -1.2895219919 1.7043897424 2.129928931
O 0.2881188515 3.3286466596 2.8176941337
S -0.5611193588 4.5409617165 2.484527619
O -0.6398543135 5.6082547003 3.4628932097
O -1.8678642446 3.9541763508 1.9695303006
C 0.2374646381 5.2358125136 0.9487639412
F 1.4598257804 5.670169233 1.2414904923
F 0.317593605 4.2682108122 0.0256633341
F -0.4997409694 6.2371276953 0.4778260225
O -0.8149339993 -0.0772738327 3.5836418384
O -2.8139506224 -1.1230780264 4.7556184799
O -2.7879932636 1.2573913216 3.8571905993
S -2.048457312 0.0729285439 4.4597944917
C -1.3829296723 0.7333318388 6.0730815502
F -0.5962090755 -0.1833178151 6.6306568784
F -0.676806614 1.8423574074 5.8318775866
F -2.3969637692 1.0165965694 6.8843857262
C -0.1500932078 -1.1040676121 -0.7413489606
C -1.498928808 -0.9566777335 -0.9755915908
C -1.5434906227 0.551908973 -0.6523210543
C -0.1305477642 0.2234723731 -0.1442719425
O 0.4420451701 0.799409868 0.8101673553
O -2.2938828646 0.9390714635 0.450143312
O 0.6336758742 -2.1639013687 -0.8982321202
C 1.9706826679 -2.2546562354 -0.2662771707
C -2.600244517 -1.9091410325 -1.252750969
H -2.2427487313 -2.941519647 -1.2942337991
H -3.0828227173 -1.6576934078 -2.2067451322
H -3.370005976 -1.8237061515 -0.4760398673
C 2.8506529653 -1.091775676 -0.7330353187
H 3.8766651483 -1.2514806633 -0.3844138378
H 2.8691198291 -1.0370169325 -1.8267982153
H 2.5143654479 -0.1331341538 -0.3292402867
C 2.4943495237 -3.5887313424 -0.799712309
H 3.4874559176 -3.7929704411 -0.3873015405
H 1.8252017581 -4.4063555069 -0.5141399814
H 2.5689305083 -3.5669823706 -1.8916435011
C 1.8044270103 -2.2900031139 1.254756345
H 1.4090036159 -1.3464382759 1.6405065039
H 1.1325485851 -3.1028985855 1.5487873281
H 2.7771563577 -2.4630437256 1.728116392
C -1.6961446865 1.4810891449 -1.8524134928
C -0.963066287 1.2771064458 -3.0315195078
C -2.5863667588 2.5593850083 -1.787153367
H -0.2727557004 0.4414679329 -3.1070570632
H -3.1607280302 2.7127668204 -0.8807699188
C -1.1117652616 2.1396323363 -4.1171970938
C -2.7383224112 3.4189427061 -2.877647807
H -0.5332551364 1.9700811132 -5.0214909169
H -3.4357032531 4.2497373148 -2.8113678324
C -2.0014494695 3.2141673048 -4.0444741189
H -2.1188268444 3.8844987404 -4.8915766551

Frequency: 11.6850; 14.1317; 20.3361; 21.3170; 23.6378; 28.0369; 29.3906; 35.4097; 41.9512; 42.8826; 46.3304; 52.3269; 58.1888; 67.7746; 76.7535; 85.1487; 100.8285; 120.7441; 141.4008; 154.3167; 163.5723; 165.7419; 182.7386; 193.2203; 194.0385; 201.1269; 212.4751; 216.7720; 225.5286; 239.5966; 242.0101; 253.1426; 264.2564; 266.6588; 272.3367; 292.4538; 303.7993; 306.1570; 319.1252; 320.1172; 322.4936; 341.5792; 365.1144; 365.6624; 368.6317; 388.3906; 418.3249; 425.3853; 452.0261; 465.0188; 487.1348; 490.6967; 491.3865; 532.4925; 534.4528; 538.2581; 549.1364; 557.2239; 558.0431; 579.3426; 582.1979; 595.9250; 625.1627; 632.9978; 633.3394; 636.2124; 667.5493; 711.2375; 716.1389; 750.9992; 762.0863; 765.1040; 766.2257; 797.1676; 862.7262; 865.4287; 925.8674; 933.3472; 936.3643; 945.2908; 952.5148; 959.8891; 970.2414; 978.9511; 983.9315; 1004.7792; 1017.1704; 1029.3181; 1053.2721; 1057.0608; 1061.0120; 1064.6841; 1067.5953; 1070.7967; 1084.7549; 1109.0349; 1143.4952; 1193.1089; 1198.1851; 1199.4710; 1210.3397; 1211.7359; 1232.0308; 1246.1219; 1253.2899; 1266.9998; 1273.4703; 1274.3446; 1283.8924; 1299.4037; 1327.1022; 1334.4577; 1348.4478; 1365.1534; 1381.2508; 1430.0996; 1433.4522; 1433.5466; 1459.8554; 1493.9570; 1497.7161; 1502.6874; 1504.5381; 1515.8127; 1516.9866; 1533.5580; 1535.9068; 1542.1474; 1550.5896; 1608.5249; 1640.8970; 1660.6195; 1736.4990; 3044.5953; 3061.3339; 3062.0992; 3069.1453; 3100.3772; 3127.6299; 3130.2222; 3138.2484; 3141.8656; 3145.8186; 3150.2392; 3153.7295; 3183.9147; 3192.6845; 3201.6637; 3211.8487; 3236.4256.

Cartesian Forces: Max 0.000000533 RMS 0.000000204

Item	Value	Threshold	Converged?
Maximum Force	0.000000	0.000450	YES
RMS Force	0.000000	0.000300	YES
Maximum Displacement	0.000026	0.001800	YES
RMS Displacement	0.000004	0.001200	YES

FILE: Yb_Sq2_PhYbOTf2_Prod56_Neutral_B3LYP_M062x_Conf3.out

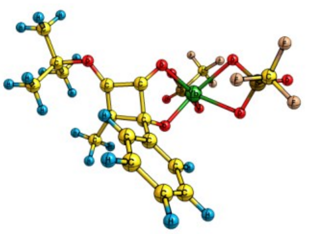

Conformer 6

E(RB3LYP/6-31G(d)/MWB59) = -2769.96455134
Convg = 0.1092D-08 -V/T = 2.0149

Temperature 195.150 Kelvin. Pressure 1.00000 Atm.
elec. and zero-point Energies= -2769.618724
elec. and thermal Energies= -2769.600378
elec. and thermal Enthalpies= -2769.599760
elec. and thermal Free Energies= -2769.660287

RB3LYP/6-31G(d)/MWB59/THF(IEFPCM) = -2769.98891655

RM06-2x/6-311+G(d,p)/MWB59/THF(IEFPCM) = -2769.79200759

Geometry at RB3LYP/6-31G(d)/MWB59
Charge = 0 Multiplicity = 1
Yb -1.2655881598 1.6505862931 2.0658570215
O 0.4548996141 3.1031545387 2.7916047253
S -0.2772656944 4.3974538032 2.4906743961
O -0.2568402443 5.4419602979 3.4963943236
O -1.6322654062 3.948623614 1.9626584239
C 0.5831211352 5.0553086528 0.9722227588
F 1.842247732 5.3605457087 1.2716214733
F 0.5668086882 4.1125179395 0.0208517908
F -0.0543563617 6.1371954861 0.5340878995
O -1.0135365515 -0.2247837838 3.4527433137
O -3.1214464035 -1.0501323959 4.6089670532
O -2.7973970008 1.3398941805 3.7992908063
S -2.2110789276 0.0491025397 4.3482028303
C -1.4547643363 0.5555867829 5.9768793785
F -0.7940139952 -0.476963644 6.4938080319
F -0.6091011659 1.569383655 5.7683612536
F -2.4179342071 0.9409451376 6.8083602693
C -0.4652821967 -1.1382613498 -0.9056219971
C -1.8089692149 -0.8819106154 -1.0982417191
C -1.7061928162 0.6293152386 -0.7418092464
C -0.3211418159 0.1708422327 -0.2898915904
O 0.331484687 0.6476308232 0.6624915389
O -2.3910059003 1.0470228924 0.3909977234
O 0.4696727918 -2.0794361692 -0.9595475424
C 0.2528198527 -3.463844722 -1.4117777048
C -3.0883024697 -1.59540145 -1.3471908228
H -2.9931859597 -2.6785721418 -1.4342234618
H -3.5480093377 -1.2067996813 -2.2661988369
H -3.7834480331 -1.3684228828 -0.529307831
C -0.6128771622 -4.1981899663 -0.3839680264
H -0.7179621755 -5.250625805 -0.6687988727
H -0.1454474719 -4.1544392385 0.604584852
H -1.6158453543 -3.7715157835 -0.3039900465
C 1.6738220687 -4.0332936589 -1.4208684407
H 1.6555369076 -5.0863104854 -1.7196754246
H 2.3056364759 -3.4818846084 -2.124018168
H 2.1209054217 -3.9607995858 -0.4249352568
C -0.3375201268 -3.474810981 -2.8248170064
H -1.3452160429 -3.0542159854 -2.8646736031
H 0.3017946789 -2.907018729 -3.5087436062
H -0.394279823 -4.5058096581 -3.1898028126
C -1.8341811016 1.5854903947 -1.9225522399
C -1.1426942797 1.3624771837 -3.1230694
C -2.6600070829 2.7106506617 -1.8160809354
H -0.4994908917 0.4930858455 -3.2282678119
H -3.1985777965 2.8804843399 -0.8908373548
C -1.2697869984 2.2510807089 -4.1902474927
C -2.7912507704 3.5962551462 -2.8883027638
H -0.7223247882 2.0674173371 -5.1109958301
H -3.4382640703 4.4638323933 -2.7900942529
C -2.0965907634 3.3714686542 -4.077124677
H -2.1967291888 4.0626581642 -4.9095642949

Frequency: 11.7100; 12.8774; 20.6085; 21.6981; 24.4641; 27.1870; 29.8158; 30.9304; 42.3610; 42.9200; 45.4467; 52.4314; 56.9716; 66.4260; 79.6359; 85.2173; 105.1184; 124.7636; 152.0282; 162.7451; 165.2457; 168.8747; 180.8445; 184.1866; 192.4119; 198.1740; 216.6780; 224.6437; 226.3118; 236.5387; 238.9346; 241.7057; 260.6677; 261.7064; 269.2177; 291.1691; 304.0725; 306.3024; 314.6043; 320.2373; 320.4432; 344.0681; 365.5634; 366.0148; 369.4925; 380.8289; 418.5442; 427.3395; 456.0507; 473.1791; 477.4724; 490.7069; 491.4029; 517.5394; 532.5546; 536.4826; 541.3508; 557.2411; 558.0820; 579.3752; 582.3386; 597.1030; 625.2251; 633.3779; 634.3955; 646.6738; 679.7135; 713.8530; 725.2992; 749.5685; 765.1321; 766.2919; 767.6124; 799.5722; 835.5889; 864.9568; 923.1055; 931.0919; 932.5820; 952.9864; 958.7883; 960.5658; 967.1249; 977.9866; 979.2291; 1004.5355; 1017.2660; 1031.5184; 1049.8130; 1058.6702; 1058.9882; 1060.4640; 1068.4961; 1069.2259; 1095.6168; 1113.4820; 1129.4109; 1192.9243; 1198.6170; 1199.9572; 1210.7389; 1214.0711; 1228.0018; 1246.4290; 1253.4615; 1261.9313; 1272.9316; 1273.7463; 1283.0586; 1292.9200; 1326.2233; 1333.9427; 1348.2395; 1364.7598; 1367.1170; 1429.7264; 1432.6990; 1434.0135; 1458.9634; 1494.0442; 1497.3039; 1503.3843; 1506.1433; 1516.8242; 1518.0663; 1528.2197; 1530.0279; 1542.0028; 1549.3257; 1619.2508; 1640.9072; 1660.6254; 1742.5513; 3045.0848; 3064.1322; 3066.7917; 3073.4569; 3097.7900; 3131.3245; 3134.9122; 3141.8626; 3144.6944; 3152.1884; 3153.6620; 3166.8063; 3183.4921; 3192.4159; 3201.3413; 3211.6584; 3236.7081.

Cartesian Forces: Max 0.000000706 RMS 0.000000256

Item	Value	Threshold	Converged?
Maximum Force	0.000000	0.000450	YES
RMS Force	0.000000	0.000300	YES
Maximum Displacement	0.000015	0.001800	YES
RMS Displacement	0.000004	0.001200	YES

FILE: Yb_Sq2_PhYbOTf2_Prod56_Neutral_B3LYP_M062x_Conf3-.out

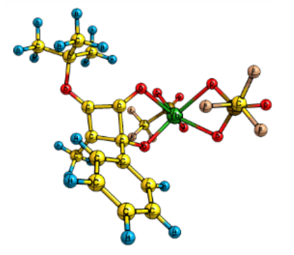

Conformer 7

E(RB3LYP/6-31G(d)/MWB59) = -2769.97105281
Convg = 0.1297D-08 -V/T = 2.0149

Temperature 195.150 Kelvin. Pressure 1.00000 Atm.
elec. and zero-point Energies= -2769.625260
elec. and thermal Energies= -2769.606899
elec. and thermal Enthalpies= -2769.606281
elec. and thermal Free Energies= -2769.666912

RB3LYP/6-31G(d)/MWB59/THF(IEFPCM) = -2769.99299973

RM06-2x/6-311+G(d,p)/MWB59/THF(IEFPCM) = -2769.79593709

Geometry at RB3LYP/6-31G(d)/MWB59
Charge = 0 Multiplicity = 1

```
Yb -1.1257852132  1.597050268  2.1587990608
O   0.4007543243  3.2591136126  2.8611931767
S  -0.4904403503  4.4485783494  2.555067402
O  -0.5924261994  5.4999130184  3.5478463334
O  -1.7831361502  3.8264098351  2.0449068562
C   0.2678653034  5.1885809065  1.0198570332
F   1.4797449097  5.6564522063  1.3035378147
F   0.3662345513  4.2361786755  0.082312212
F  -0.5055934338  6.1726682862  0.5712087609
O  -2.541264599  1.0488258298  3.9278254028
O  -1.5205210242 -0.2146354616  5.8857646242
O  -0.5453168523 -0.2199227013  3.5355733046
S  -1.751186829 -0.1383540568  4.4566191532
C  -2.7823979917 -1.6199719643  3.9821690205
F  -3.9394479969 -1.5815159412  4.6349723893
F  -3.0125618678 -1.5977948013  2.6635977543
F  -2.1204877331 -2.731749665  4.2953099484
C   0.0165008305 -1.1165027968 -0.7932138692
C  -1.3439739081 -1.0138912489 -0.9779717233
C  -1.4297035844  0.4901417801 -0.6400242295
C   0.0094434871  0.2044025737 -0.1811327973
O   0.5907280019  0.7849996531  0.7651065655
O  -2.1560752607  0.8421145941  0.4891500577
O   0.8307710138 -2.1467621929 -0.9862050725
C   2.1939315985 -2.1916620442 -0.4061050588
C  -2.4214645562 -2.0042556833 -1.214952208
H  -2.0229876969 -3.0160297918 -1.3291517016
H  -2.9893887368 -1.7363617245 -2.1155212225
H  -3.1291991861 -1.9890455379 -0.3767672641
C   3.0095217348 -0.988945847 -0.8884108531
H   4.053734701 -1.1140175472 -0.5823997297
H   2.982967349 -0.9181371862 -1.9810805027
H   2.6536393473 -0.0497410768 -0.4568635572
C   2.7494556255 -3.496798132 -0.9774475972
H   3.7637563967 -3.6674046811 -0.6031454825
H   2.1232065006 -4.3439672761 -0.6808243748
H   2.7842889422 -3.4574322302 -2.070866619
C   2.0857445013 -2.2529857793  1.1193338242
H   1.6611535499 -1.3339669321  1.5324420902
H   1.4643462763 -3.100777932  1.4259469049
H   3.0820259537 -2.385524285  1.5551382217
C  -1.6519921055  1.4231153551 -1.8261774127
C  -0.9669518028  1.2408103998 -3.0372797907
C  -2.5581619751  2.4841837684 -1.714504126
H  -0.2649674443  0.4190897452 -3.1487505938
H  -3.0954043647  2.6210647238 -0.7831004737
C  -1.1786531352  2.1073626844 -4.1092223253
C  -2.7728966209  3.3479936782 -2.7909602792
H  -0.6370484436  1.9544501597 -5.0389739184
H  -3.4816168792  4.1653778313 -2.6883062465
C  -2.0838721608  3.1646068285 -3.9901328208
H  -2.2502097967  3.8381987536 -4.8263720611
```

Frequency: 11.1872; 12.8829; 17.6289; 20.4341; 23.4594; 27.9354; 28.9398; 34.6417; 40.5544; 43.9013; 46.6879; 51.8988; 59.0974; 69.3623; 77.2532; 84.6425; 100.3383; 118.5433; 141.0119; 155.7703; 161.9801; 168.6439; 186.4047; 187.8455; 195.4169; 202.3142; 212.2764; 213.4393; 226.1096; 239.0599; 240.3751; 253.8763; 263.7744; 266.5697; 272.0470; 292.3792; 303.6732; 305.2563; 318.6463; 320.0403; 321.8214; 341.4327; 362.7510; 365.9582; 369.2426; 388.2500; 418.2195; 425.5513; 452.0304; 465.0239; 486.9106; 490.5579; 491.4129; 532.0550; 534.8759; 538.6571; 549.5215; 557.1145; 557.6663; 577.5109; 585.3014; 594.5614; 622.3014; 633.3359; 633.8556; 637.1268; 668.1195; 711.1657; 716.0559; 750.6789; 762.1651; 764.8071; 764.9927; 797.2265; 862.3715; 865.3784; 925.5408; 933.3593; 936.2141; 945.0576; 951.1385; 960.3518; 969.6275; 978.7789; 983.8089; 1004.6925; 1017.1555; 1030.1756; 1053.6432; 1057.7193; 1061.1098; 1064.1664; 1066.9507; 1070.7379; 1084.7775; 1108.9689; 1143.1146; 1193.0758; 1197.9355; 1199.5668; 1209.8722; 1211.8136; 1232.0601; 1245.4389; 1246.6571; 1266.3280; 1272.3533; 1274.1364; 1284.0204; 1299.3769; 1328.7757; 1337.1966; 1348.4502; 1365.1822; 1381.4425; 1430.1674; 1433.3879; 1433.5662; 1459.9360; 1493.9594; 1498.9323; 1502.3541; 1504.4801; 1515.6478; 1516.8937; 1533.3697; 1535.9302; 1542.2341; 1550.5698; 1608.7874; 1640.9313; 1660.6388; 1736.2529; 3045.8499; 3061.5625; 3062.1198; 3069.2013; 3100.6671; 3127.8503; 3130.0867; 3138.2126; 3141.9792; 3144.6959; 3150.6073; 3153.9231; 3184.0577; 3192.9020; 3201.9095; 3211.9166; 3236.7441.

Cartesian Forces: Max 0.000000827 RMS 0.000000301

Item	Value	Threshold	Converged?
Maximum Force	0.000000	0.000450	YES
RMS Force	0.000000	0.000300	YES
Maximum Displacement	0.000011	0.001800	YES
RMS Displacement	0.000002	0.001200	YES

FILE: Yb_Sq2_PhYbOTf2_Prod56_Neutral_B3LYP_M062x_Conf4.out

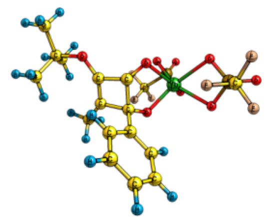

Conformer 8

E(RB3LYP/6-31G(d)/MWB59) = -2769.96353235
Convg = 0.8288D-08 -V/T = 2.0149

Temperature 195.150 Kelvin. Pressure 1.00000 Atm.
elec. and zero-point Energies= -2769.617755
elec. and thermal Energies= -2769.599387
elec. and thermal Enthalpies= -2769.598769
elec. and thermal Free Energies= -2769.659476

RB3LYP/6-31G(d)/MWB59/THF(IEFPCM) = -2769.98807436

RM06-2x/6-311+G(d,p)/MWB59/THF(IEFPCM) = -2769.79142574

Geometry at RB3LYP/6-31G(d)/MWB59
Charge = 0 Multiplicity = 1

```
Yb -0.9733053404  1.554265319  2.0776012327
O   0.7162620184  3.0676996852  2.7423257186
S  -0.0814790397  4.3338392117  2.4914892958
O  -0.0583939817  5.368465524  3.5067792489
O  -1.4398453616  3.8334046333  2.0195477897
C   0.6794482379  5.0430817945  0.9431636885
F   1.9380513359  5.3953984984  1.187350008
F   0.6557368886  4.1117722326 -0.0194464548
F  -0.0207278846  6.103338417  0.5499732143
O  -2.3351878896  1.1372083923  3.9295111632
O  -1.3574929171 -0.3034788535  5.7843038823
O  -0.5474108578 -0.360413089  3.3732079068
S  -1.6696753446 -0.1542622829  4.3764418485
C  -2.9111574553 -1.480028775  3.9463111301
F  -4.0011117153 -1.3239716192  4.6910608282
F  -3.2390216845 -1.3836154268  2.6515541337
F  -2.3725272198 -2.6760819125  4.1754037077
C  -0.2741385718 -1.1335503385 -1.0047303026
C  -1.6425516095 -0.9560846932 -1.0686389065
C  -1.5964555097  0.5575734857 -0.7074945496
C  -0.1532166927  0.1753040425 -0.3837572788
O   0.5483875974  0.6734762595  0.5220548212
O  -2.2013213005  0.9303365825  0.4837406421
O   0.7058816366 -2.0167702323 -1.1531842122
C   0.5344649463 -3.403500962 -1.61722919
C  -2.8940380347 -1.7498128125 -1.1833772141
H  -2.7394063401 -2.796674222 -1.4490243824
H  -3.5511592089 -1.2876273273 -1.9311909684
H  -3.429325749 -1.709553418 -0.2259486548
C  -0.2147983872 -4.2043722102 -0.5483079606
H  -0.2738416095 -5.2571099313 -0.8449788917
H   0.3149173629 -4.1461126643  0.4076216205
H  -1.2347532953 -3.8427989212 -0.3955827882
C   1.9805372971 -3.8915700852 -1.7366098632
H   1.9995861655 -4.9372618434 -2.0601009316
H   2.5308874849 -3.2902660499 -2.4667416364
H   2.4908614078 -3.8152009634 -0.7718463394
C  -0.1522999966 -3.4219165872 -2.985834944
H  -1.1781895239 -3.0473028247 -2.947865479
H   0.4105699288 -2.8143702799 -3.7019602596
H  -0.1876092755 -4.4487851256 -3.3648496472
C  -1.8821189602  1.5092288763 -1.8641573234
C  -1.3070436143  1.317880658 -3.1297768879
C  -2.7374987112  2.5996919429 -1.6674386451
H  -0.6431250472  0.4759874698 -3.3053355327
H  -3.1861380432  2.7453651026 -0.691556854
C  -1.5772035903  2.2032280339 -4.1727784471
C  -3.0117904401  3.4821007982 -2.714899028
H  -1.1188981172  2.0441909586 -5.1453373493
H  -3.6796133116  4.322576909 -2.545931506
C  -2.4326227024  3.2887660843 -3.9693046716
H  -2.6441472207  3.9774878581 -4.7827119287
```

Frequency: 11.5634; 12.4668; 17.9887; 21.5767; 24.5603; 26.8858; 28.8900; 30.6347; 40.8428; 43.6591; 44.9972; 51.9437; 58.4888; 68.0999; 71.1351; 86.1343; 102.6880; 125.4366; 153.6922; 161.5391; 167.6478; 171.4936; 182.8505; 186.7360; 188.6916; 199.1395; 213.1877; 224.7887; 227.3197; 237.6469; 238.4053; 239.9340; 260.4936; 260.8838; 269.8951; 290.4682; 303.5177; 305.3119; 313.8590; 320.0419; 320.2714; 344.2175; 363.1740; 366.0865; 369.8342; 381.1920; 418.1422; 427.2090; 456.4079; 473.0614; 478.2175; 490.6066; 491.5535; 517.4877; 532.1139; 536.9252; 542.3009; 557.2198; 557.5372; 577.4764; 585.1740; 594.7144; 622.5202; 633.4125; 636.2121; 647.4710; 679.9169; 713.3867; 725.2077; 748.7897; 764.5181; 765.0691; 767.5509; 799.7406; 835.5113; 864.4765; 922.5370; 931.2066; 932.5931; 951.6598; 958.2452; 961.0164; 966.3441; 977.5693; 979.3782; 1004.1860; 1017.2363; 1033.4701; 1051.0437; 1059.0093; 1059.5294; 1060.5267; 1067.8904; 1069.2223; 1096.1605; 1111.7740; 1129.0524; 1192.8559; 1198.5913; 1200.3851; 1210.7748; 1214.0923; 1228.1033; 1244.7232; 1246.3950; 1260.6140; 1271.1178; 1273.4620; 1282.9737; 1293.1809; 1328.4151; 1336.8242; 1348.1040; 1364.6924; 1366.8607; 1430.3049; 1432.5004; 1434.1281; 1458.9219; 1494.0278; 1498.6396; 1503.6381; 1505.3676; 1516.6196; 1518.8395; 1528.6287; 1529.6429; 1541.9771; 1549.6498; 1618.5533; 1640.9014; 1660.5568; 1741.2138; 3049.4543; 3064.2452; 3066.7367; 3073.4003; 3099.4773; 3131.7760; 3134.5025; 3142.2420; 3144.6645; 3151.6115; 3152.3190; 3164.8837; 3183.7010; 3192.6915; 3201.6784; 3211.7517; 3237.2511.

Cartesian Forces: Max 0.000000743 RMS 0.000000182

Item	Value	Threshold	Converged?
Maximum Force	0.000000	0.000450	YES
RMS Force	0.000000	0.000300	YES
Maximum Displacement	0.000052	0.001800	YES
RMS Displacement	0.000013	0.001200	YES

FILE: Yb_Sq2_PhYbOTf2_Prod56_Neutral_B3LYP_M062x_Conf4-.out

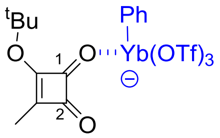	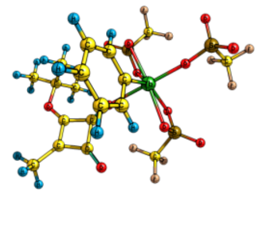	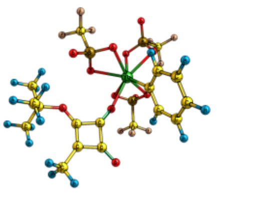	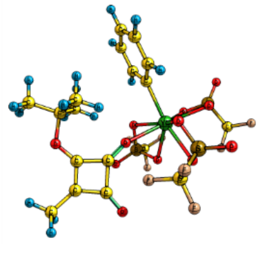
**Pre-reaction complex 1b•[PhYb(OTf)$_3$]$^-$:- chelation to C1 carbonyl only**	*Conformer 1*	*Conformer 2*	*Conformer 3*
Lowest energy conformation is: Conformer 11	E(RB3LYP/6-31G(d)/MWB59) = -3731.45894922<br>Convg = 0.6494D-08 -V/T = 2.0122<br><br>Temperature 195.150 Kelvin. Pressure 1.00000 Atm.<br>elec. and zero-point Energies= -3731.088057<br>elec. and thermal Energies= -3731.063681<br>elec. and thermal Enthalpies= -3731.063063<br>elec. and thermal Free Energies= -3731.137580<br><br>RB3LYP/6-31G(d)/MWB59/THF(IEFPCM) = -3731.52649885<br><br>RM06-2x/6-311+G(d,p)/MWB59/THF(IEFPCM) = -3731.36093368	E(RB3LYP/6-31G(d)/MWB59) = -3731.45054073<br>Convg = 0.8945D-08 -V/T = 2.0122<br><br>Temperature 195.150 Kelvin. Pressure 1.00000 Atm.<br>elec. and zero-point Energies= -3731.079200<br>elec. and thermal Energies= -3731.055170<br>elec. and thermal Enthalpies= -3731.054552<br>elec. and thermal Free Energies= -3731.128305<br><br>RB3LYP/6-31G(d)/MWB59/THF(IEFPCM) = -3731.52066154<br><br>RM06-2x/6-311+G(d,p)/MWB59/THF(IEFPCM) = -3731.35625207	E(RB3LYP/6-31G(d)/MWB59) = -3731.46302286<br>Convg = 0.2449D-09 -V/T = 2.0122<br><br>Temperature 195.150 Kelvin. Pressure 1.00000 Atm.<br>elec. and zero-point Energies= -3731.091937<br>elec. and thermal Energies= -3731.067777<br>elec. and thermal Enthalpies= -3731.067159<br>elec. and thermal Free Energies= -3731.140252<br><br>RB3LYP/6-31G(d)/MWB59/THF(IEFPCM) = -3731.52926629<br><br>RM06-2x/6-311+G(d,p)/MWB59/THF(IEFPCM) = -3731.36949938
	Geometry at RB3LYP/6-31G(d)/MWB59<br>Charge = -1 Multiplicity = 1<br>Yb 0.2409039003 2.3315007323 1.2057059747<br>O 2.3086731858 2.4376772762 -0.102237847<br>S 3.2885358711 2.1543245539 1.0048416721<br>O 4.348775695 1.1862592383 0.7172236369<br>O 2.466315145 1.9347993472 2.2442886153<br>C 4.1774921053 3.7659020117 1.3007130764<br>F 4.7755698373 4.1588149157 0.1724312649<br>F 3.326525112 4.7034693244 1.7051593006<br>F 5.1101989118 3.5823861519 2.24558085<br>O 0.0732123543 4.4739814093 1.0594546323<br>O -1.2176455611 6.4611213944 0.3020761165<br>O 1.3040960449 6.447120911 0.1766962874<br>S 0.0678752203 5.9859853535 0.8103523005<br>C 0.1614720123 6.5740888058 2.5726090448<br>F -0.8972480006 6.1522286571 3.2703855953<br>F 1.2758682816 6.1204834246 3.1645925823<br>F 0.1854263939 7.9170224525 2.5990544726<br>C 0.3636838096 -2.5309677489 0.6491282834<br>C -0.8973045981 -3.0905493575 0.7793999932<br>C -1.4939365263 -1.85492369 1.3318249005<br>C -0.0998558482 -1.196951839 1.1706213001<br>O 0.4147056898 -0.1085781593 1.3484707734<br>O -2.5792918404 -1.5212757422 1.7391877642<br>O 1.4579988635 -3.1017579792 0.2122652517<br>C 2.7645162379 -2.3760399181 0.0797718671<br>C -1.4629363965 -4.4248503354 0.4708692715<br>H -0.6838923084 -5.1372582224 0.1845541587<br>H -2.186116996 -4.3471178833 -0.3517229239<br>H -2.0123563899 -4.816630525 1.3356622216<br>C 2.6082610833 -1.2675180314 -0.9583021103<br>H 3.5653740254 -0.7543552832 -1.087993807<br>H 2.2941853232 -1.6850561597 -1.9209768445<br>H 1.883378846 -0.5114356102 -0.6520564568<br>C 3.6891965759 -3.4836777343 -0.4255772792<br>H 4.693334005 -3.0770581749 -0.5812859066<br>H 3.7539584093 -4.3007173819 0.3007918559<br>H 3.3274234605 -3.8884850529 -1.3763312348<br>C 3.2025162412 -1.8766060661 1.4553419283<br>H 2.5418353385 -1.0977538936 1.8396384305<br>H 3.2420411397 -2.7081813797 2.1682954107<br>H 4.197312945 -1.429577986 1.3769897716<br>C -0.7639725095 1.6153050706 -0.8643606709<br>C -0.0791897679 1.6600052959 -2.1015069732<br>C -2.0763313821 1.0881469654 -0.9217386225<br>H 0.9343804472 2.0585255897 -2.1378267688<br>H -2.6714298692 1.03126062 -0.0108331798<br>C -0.6481111278 1.2136648654 -3.3008864743<br>C -2.6597733405 0.6348391348 -2.1108860016<br>H -0.0806708513 1.2735920485 -4.22953793<br>H -3.676079813 0.2413402647 -2.1036998862<br>C -1.9443918515 0.6948040965 -3.309356885<br>H -2.3936184368 0.3468771888 -4.2384958373<br>O -2.4414572248 3.213224463 4.3576746285<br>S -1.6027725176 2.2779011774 3.6201026361<br>C -1.8719000173 0.637420468 4.4672238587<br>O -0.1121507222 2.469926746 3.6599235769<br>O -1.9457137719 2.0273658436 2.1720502522<br>F -1.0177892438 -0.289431573 4.0044558718<br>F -3.1210428104 0.2147257422 4.2743187914<br>F -1.6580831337 0.780626546 5.7822823821<br><br>Frequency: 8.1729; 12.9665; 15.1743; 18.3888; 21.0101; 22.9952; 26.0809; 29.6286; 34.1024; 37.0058; 39.2057; 45.7530; 46.5185; 48.7786; 51.3478; 54.3168; 55.7454; 61.1794; 68.5435; 74.2256; 77.0562; 81.0040; 84.7180; 88.9870; 91.4579; 114.6723; 123.7335; 135.3587; 141.3757; 144.9393; 148.1104; 166.0913; 179.2349; 183.7040; 186.9661; 189.6577; 190.6846; 201.6273; 209.5684; 212.3680; 214.4567; 216.6030; 217.5008; 221.9416; 253.4637; 269.1918; 272.4429; 292.0415; 300.5754; 305.2670; 307.2045; 321.5830; 322.1590; 329.1835; 329.3733; 339.9039; 350.4159; 352.5827; 355.9060; 394.7443; 396.1105; 449.6672; 454.8233; 474.0156; 478.8289; 488.9759; 490.6992; 492.1632; 508.9876; 524.9384; 528.8083; 553.7889; 556.4695; 558.0847; 570.9715; 573.4299; 575.3727; 596.4908; 600.7854; 616.3236; 625.0671; 627.6606; 633.8777; 643.7709; 647.9546; 719.9435; 731.8635; 737.5224; 759.5504; 759.9610; 764.7063; 777.5127; 786.8987; 846.1084; 871.1347; 915.2521; 937.0227; 941.2614; 960.5055; 967.6943; 975.9687; 976.9662; 983.4750; 985.5387; 986.7294; 989.7704; 1010.6695; 1032.4010; 1054.8363; 1064.5243; 1066.6977; 1084.3970; 1085.3274; 1085.5258; 1110.8389; 1128.7499; 1152.9128; 1183.6393; 1198.1431; 1208.8299; 1211.8632; 1220.7952; 1223.7921; 1226.4959; 1229.5444; 1237.5899; 1239.6232; 1245.8119; 1249.1528; 1260.4266; 1286.8173; 1292.0899; 1292.9083; 1299.6191; 1314.2837; 1333.1086; 1371.5123; 1398.1369; 1431.9064; 1441.0141; 1445.4011; 1458.1270; 1464.2023; 1499.1357; 1502.2232; 1505.9479; 1513.3648; 1517.1607; 1521.6882; 1528.0859; 1529.6656; 1549.8931; 1613.5338; 1625.0597; 1647.6221; 1812.7354; 1888.6256; 3044.7474; 3058.8638; 3066.2957; 3073.8279; 3097.1746; 3130.6980; 3136.6678; 3137.1038; 3138.3019; 3143.5833; 3145.5854; 3148.4025; 3159.9754; 3166.0088; 3173.8693; 3175.7201; 3176.9204.<br><br>Cartesian Forces: Max 0.000001260 RMS 0.000000289<br><br>Item Value Threshold Converged?<br>Maximum Force 0.000000 0.000450 YES<br>RMS Force 0.000000 0.000300 YES<br>Maximum Displacement 0.000094 0.001800 YES<br>RMS Displacement 0.000013 0.001200 YES	Geometry at RB3LYP/6-31G(d)/MWB59<br>Charge = -1 Multiplicity = 1<br>Yb 0.0640730214 2.1072841636 0.9742710455<br>O 1.9375298147 1.9607730048 -0.6504884882<br>S 2.7116871555 0.8780367727 0.0399959982<br>O 3.0892010522 -0.2895798262 -0.7643096496<br>O 2.0139356268 0.6109876943 1.3424850592<br>C 4.3442849325 1.6426223391 0.5107243853<br>F 5.001624125 2.0101413175 -0.59541283<br>F 4.1789154317 2.7011019166 1.296187634<br>F 5.0788677934 0.7251279551 1.162528577<br>O 0.1760845217 4.3758432414 0.0706041563<br>O 2.3726075491 5.5939614442 0.4399810767<br>O 1.4684315658 3.8280777775 2.0208373515<br>S 1.2116066083 4.9219296897 1.0148582421<br>C 0.3324627623 6.2546432106 1.9773264857<br>F 0.0034279492 7.2555593584 1.1484494887<br>F -0.7736180474 5.7908838063 2.55529485<br>F 1.1519863791 6.7326742311 2.9238517902<br>C -0.0552040562 -2.5409560647 0.9638369892<br>C -1.1152704268 -3.4068186139 0.7115747795<br>C -2.1318770431 -2.3367460666 0.8915264089<br>C -0.9625510339 -1.349067664 1.0904747165<br>O -0.8039805605 -0.1555253509 1.2006100575<br>O -3.3391943861 -2.3025416642 0.8725845678<br>O 1.2408480449 -2.4934808761 1.1046050195<br>C 2.1973589829 -3.5875112614 0.8193893225<br>C -1.3872043164 -4.8298484093 0.3748367239<br>H -1.3043435212 -5.4738968466 1.2606851379<br>H -0.7109364496 -5.2270328215 -0.3890205825<br>H -2.4166299487 -4.9100743959 0.0089632961<br>C 1.8593149002 -4.8199789049 1.6615356547<br>H 2.6659305476 -5.554401517 1.5605007864<br>H 1.7787704083 -4.5487204218 2.7193567102<br>H 0.9319439295 -5.3046541609 1.3507431167<br>C 3.5334892305 -2.986318124 1.2594252354<br>H 4.3361669717 -3.7115921663 1.084919069<br>H 3.7438392717 -2.075156728 0.6959559725<br>H 3.5091836136 -2.7412900035 2.3259268901<br>C 2.1803082661 -3.8462842814 -0.6881047207<br>H 1.2122009644 -4.2309000029 -1.0257337095<br>H 2.3961858604 -2.916270221 -1.2218048348<br>H 2.9457258359 -4.5868726133 -0.9458289779<br>C -1.3560547664 1.7722221093 -0.9443657745<br>C -0.9370331717 2.1414598056 -2.2432015814<br>C -2.675047154 1.269836647 -0.8587674678<br>H 0.0653920692 2.543213508 -2.3860898909<br>H -3.0719385371 0.9587635114 0.1081145749<br>C -1.7599051433 2.0194145103 -3.3690654786<br>C -3.5150123128 1.1448331388 -1.9712896793<br>H -1.3898241712 2.3168882399 -4.3501964289<br>H -4.5252413386 0.7542911571 -1.8508939948<br>C -3.0571442921 1.5191022067 -3.2367058364<br>H -3.7035097596 1.4229103264 -4.1081635298<br>O -1.4506761206 3.3125511237 4.7401675626<br>S -1.4436410709 2.3961974501 3.6056702729<br>C -2.863981345 1.2344853103 3.9436157047<br>O -0.246456656 1.5137430287 3.4170661638<br>O -1.7649329203 2.9707888485 2.2456337904<br>F -3.0619779696 0.3896120779 2.9261585619<br>F -3.9844633705 1.9381813023 4.1409424236<br>F -2.6024294761 0.5199354354 5.0480918506<br><br>Frequency: 3.8324; 13.6074; 15.1542; 19.4031; 23.6103; 25.4798; 27.9119; 31.3191; 32.2516; 34.9281; 38.8402; 43.3365; 44.0549; 48.1132; 48.8283; 55.2636; 57.2913; 71.7354; 77.4682; 83.8586; 88.0255; 89.2845; 97.0059; 116.2566; 126.8190; 133.6002; 135.8319; 139.4403; 142.6813; 148.3092; 149.7434; 163.4065; 174.0784; 179.1914; 186.8633; 203.0862; 205.5173; 208.2811; 212.0534; 214.1437; 217.6320; 220.6062; 226.6114; 229.2742; 247.1654; 256.6640; 274.9626; 293.9307; 303.3766; 306.2588; 308.6287; 323.0224; 323.8416; 324.9145; 334.5479; 350.1032; 352.0923; 354.0902; 357.4441; 393.6283; 394.0101; 449.7060; 451.3098; 478.9023; 482.8480; 489.2107; 490.3324; 491.2985; 522.5089; 524.2306; 528.0569; 557.7456; 557.9384; 558.6863; 569.8231; 571.8265; 572.2415; 595.7070; 618.5332; 624.9035; 625.9935; 632.2627; 634.4012; 646.4097; 649.4815; 715.9121; 735.5368; 759.4472; 761.9462; 762.7025; 763.6636; 779.6035; 787.7871; 843.1114; 866.9959; 911.0880; 936.0238; 938.6492; 972.8993; 974.1952; 975.6011; 978.9084; 982.6089; 985.2624; 987.8503; 996.9094; 1011.5898; 1031.5787; 1053.8384; 1063.5871; 1069.3627; 1083.3029; 1086.8565; 1093.9671; 1110.3740; 1121.2612; 1143.3337; 1182.8925; 1195.2474; 1207.0968; 1211.0779; 1212.2804; 1214.4403; 1224.8164; 1229.9963; 1230.3367; 1232.3450; 1238.1616; 1254.3965; 1258.0693; 1281.5592; 1287.8986; 1295.9736; 1296.2894; 1304.2540; 1311.0058; 1369.9347; 1388.4115; 1430.0976; 1434.0817; 1440.2099; 1458.7914; 1459.9808; 1500.3852; 1507.6267; 1513.2393; 1519.5570; 1521.8295; 1525.3188; 1529.1241; 1534.7353; 1554.8616; 1614.4613; 1625.0186; 1646.6295; 1849.4309; 1885.9409; 3040.3223; 3059.4850; 3062.6442; 3070.7095; 3099.9792; 3124.9388; 3126.5163; 3129.0038; 3134.1269; 3134.6082; 3141.3137; 3151.6058; 3153.8927; 3164.3651; 3168.8924; 3172.5987; 3174.3218.<br><br>Cartesian Forces: Max 0.000002949 RMS 0.000000544<br><br>Item Value Threshold Converged?<br>Maximum Force 0.000003 0.000450 YES<br>RMS Force 0.000000 0.000300 YES<br>Maximum Displacement 0.000281 0.001800 YES<br>RMS Displacement 0.000055 0.001200 YES	Geometry at RB3LYP/6-31G(d)/MWB59<br>Charge = -1 Multiplicity = 1<br>Yb 0.3358221977 2.4171805046 0.029963114<br>O 0.7216491583 1.9564199871 2.459131232<br>S -0.722236728 1.8649910635 2.8689787297<br>O -1.1246409773 0.6209113424 3.5336951535<br>O -1.5402454935 2.3074042833 1.6941486139<br>C -0.9516387239 3.184700567 4.1632777069<br>F -0.1621213447 2.9179815653 5.213600223<br>F -0.6462196202 4.3897029154 3.6872462724<br>F -2.2269441099 3.1882108154 4.573585066<br>O 0.5084028196 4.3897018813 -1.3834184614<br>O 1.6159079645 6.4468778976 -0.3834951395<br>O 0.562860457 4.6003457634 1.0060624603<br>S 0.609680926 5.3996002816 -0.2732977638<br>C -1.0320639513 6.2799171098 -0.3012919317<br>F -1.1795632225 6.9316458832 -1.459514995<br>F -2.0313934332 5.4042881093 -0.1622344661<br>F -1.0875132446 7.1640720329 0.7033330251<br>C -0.0472477683 -2.2949076125 1.3947315508<br>C -1.3476437665 -2.7413389464 1.5617829829<br>C -1.9464916462 -1.5056044726 1.0090769512<br>C -0.5020002007 -0.9773263645 0.8288281841<br>O 0.0494474365 0.0258543241 0.4188840502<br>O -3.0603512467 -1.1067971436 0.7770057242<br>O 1.0658196937 -2.9281829778 1.6707564821<br>C 2.4265991917 -2.308784875 1.5432122991<br>C -1.9413735522 -3.985932407 2.1041008467<br>H -1.17547542 -4.7320941855 2.3347378314<br>H -2.6570762115 -4.411750813 1.3899251506<br>H -2.5049227847 -3.7651750705 3.0201453659<br>C 2.6744806154 -1.910420939 0.0902134166<br>H 3.7076558852 -1.5655610131 -0.0189463832<br>H 2.5207575512 -2.7676802666 -0.5754755238<br>H 2.0369521604 -1.0823387394 -0.221801994<br>C 3.3332065419 -3.4658015878 1.9644037921<br>H 4.3780228183 -3.141405194 1.9307694044<br>H 3.102556246 -3.7892660933 2.9848037014<br>H 3.2134555378 -4.3211135339 1.2908495869<br>C 2.5264480716 -1.1375219006 2.5163664441<br>H 1.8422847034 -0.3260450081 2.263136102<br>H 2.3238323954 -1.4705861864 3.5403616416<br>H 3.537136951 -0.7193672573 2.4747729345<br>C 2.7037299922 1.9835734859 -0.0963174504<br>C 3.5538682106 2.1141359393 1.0272353528<br>C 3.3447757499 1.5597843411 -1.2841387302<br>H 3.1340258999 2.4400168009 1.9784334669<br>H 2.7574577276 1.4481658963 -2.194529224<br>C 4.9253023043 1.8336617399 0.9826584619<br>C 4.7135217205 1.2702276882 -1.3489090584<br>H 5.5378978776 1.9566070023 1.8760668004<br>H 5.1591365833 0.9467317326 -2.2896622754<br>C 5.5121126225 1.4025027359 -0.2101271156<br>H 6.5782737544 1.1838861388 -0.253989305<br>O -1.3560280025 2.5501638089 -3.8249459481<br>S -1.100866703 1.8041941664 -2.5979138732<br>C -1.8630050745 0.1342320259 -2.9305807806<br>O -1.74605104 2.2843905833 -1.330215159<br>O 0.3316788806 1.4929830018 -2.2482288104<br>F -1.6028493923 -0.7370444752 -1.9440413246<br>F -1.3622346001 -0.3657835591 -4.0695009257<br>F -3.1874703034 0.2544107619 -3.0578084696<br><br>Frequency: 11.2792; 14.4667; 17.1279; 19.0278; 26.2216; 27.1072; 28.5851; 32.1191; 35.8412; 37.4368; 41.5589; 43.7494; 47.5798; 51.7066; 58.4258; 59.7938; 63.6670; 66.7930; 72.0160; 74.7774; 75.7477; 81.8205; 87.8687; 93.3994; 111.3426; 124.7144; 132.2699; 137.8162; 139.5232; 144.5140; 145.7918; 166.8116; 178.2174; 178.6688; 186.0306; 187.5511; 201.6169; 209.5511; 210.9253; 212.4590; 214.3647; 217.9967; 218.9557; 227.5369; 247.7806; 265.8322; 268.9407; 290.7151; 304.1136; 305.9220; 308.7449; 320.4324; 324.3088; 325.6526; 329.3169; 347.5142; 348.2227; 350.7378; 357.9259; 393.7606; 395.8523; 449.4538; 452.3253; 477.1125; 487.9360; 490.8739; 491.1123; 491.5094; 520.6737; 523.6395; 529.3044; 556.0779; 558.0191; 558.6185; 568.2587; 569.4438; 577.3066; 594.0139; 602.1087; 622.3567; 626.9105; 632.9651; 634.3562; 645.0223; 648.2346; 719.5112; 731.9811; 739.0481; 759.4376; 761.3498; 763.3122; 782.3185; 787.3707; 845.5813; 870.9714; 915.9985; 936.6650; 940.5122; 959.9318; 976.4283; 978.6648; 979.9003; 984.7721; 986.4205; 987.5705; 989.0307; 1011.4038; 1033.0566; 1055.6799; 1062.7922; 1067.5242; 1084.1151; 1084.8540; 1086.4510; 1113.4241; 1118.9662; 1137.6542; 1182.4990; 1197.4752; 1213.6185; 1214.5532; 1216.5103; 1220.5925; 1226.2550; 1231.4184; 1236.5070; 1240.4109; 1243.6210; 1245.9149; 1253.8406; 1280.2390; 1292.6007; 1294.5004; 1297.9666; 1306.9156; 1318.1936; 1370.7077; 1397.6023; 1431.1133; 1441.7084; 1445.8127; 1457.6218; 1464.6330; 1500.3814; 1502.6625; 1506.2532; 1513.0138; 1515.5827; 1521.2729; 1528.0413; 1529.3678; 1547.8150; 1612.2284; 1623.7702; 1649.6856; 1821.9033; 1894.1987; 3044.3194; 3057.1960; 3062.4391; 3068.4721; 3096.4559; 3127.8527; 3131.5140; 3133.0901; 3133.9378; 3137.0235; 3141.1426; 3143.2212; 3160.1250; 3167.3613; 3175.1411; 3176.9800; 3181.8942.<br><br>Cartesian Forces: Max 0.000002026 RMS 0.000000763<br><br>Item Value Threshold Converged?<br>Maximum Force 0.000000 0.000450 YES<br>RMS Force 0.000000 0.000300 YES<br>Maximum Displacement 0.000040 0.001800 YES<br>RMS Displacement 0.000009 0.001200 YES
	FILE: Yb_Sq2_PhYbOTf3_GS_Anion_B3LYP_M062x_Add12_Conf1.out	FILE: Yb_Sq2_PhYbOTf3_GS_Anion_B3LYP_M062x_Add12_Conf1-_Y.out	FILE: Yb_Sq2_PhYbOTf3_GS_Anion_B3LYP_M062x_Add12_Conf1-1.out

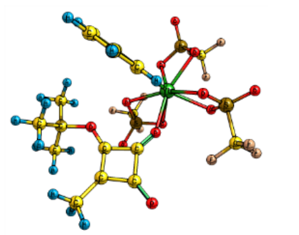

Conformer 4

E(RB3LYP/6-31G(d)/MWB59) = -3731.45748566
Convg = 0.1109D-08 -V/T = 2.0122

Temperature 195.150 Kelvin. Pressure 1.00000 Atm.
elec. and zero-point Energies= -3731.086181
elec. and thermal Energies= -3731.062120
elec. and thermal Enthalpies= -3731.061502
elec. and thermal Free Energies= -3731.134611

RB3LYP/6-31G(d)/MWB59/THF(IEFPCM) = -3731.52483470

RM06-2x/6-311+G(d,p)/MWB59/THF(IEFPCM) = -3731.36276349

Geometry at RB3LYP/6-31G(d)/MWB59
Charge = -1 Multiplicity = 1
Yb 0.033648416 2.0089219151 -0.1171801326
O 0.5700220288 1.10943553 2.166967562
S -0.8011725305 1.2452378679 2.7566446335
O -1.3804690483 0.0309999973 3.3445701437
O -1.6395833171 1.9947242145 1.7642409956
C -0.6021066434 2.4055305392 4.1996434457
F 0.2586293571 1.8730959893 5.081303341
F -0.1407815434 3.5923405265 3.8090719626
F -1.7845331176 2.5775996912 4.8063178395
O 0.851304565 4.0137370385 -1.2778755692
O 2.6994122137 5.3295292489 -0.1334986404
O 1.119237602 3.7699372123 1.0946507657
S 1.3823330336 4.7105848793 -0.0589343857
C 0.1915516399 6.1111879754 0.2418137887
F 0.2016271731 6.9491201583 -0.7998703677
F -1.0469916118 5.6414199079 0.4137254104
F 0.555493037 6.7838016576 1.3420420984
C -0.2380984962 -2.2296906643 0.9410357147
C -1.0703877381 -3.3266646229 0.7223082689
C -1.9965926392 -2.5505053927 -0.1381671079
C -1.0965208303 -1.3243127407 0.1131693693
O -1.0793214966 -0.1482202253 -0.1843568177
O -3.0043687159 -2.807642678 -0.7546241525
O 0.8539082912 -1.8530803748 1.5330226171
C 1.6811026961 -2.6522067599 2.4638499672
C -1.200186338 -4.7593709624 1.0961811874
H -0.246450713 -5.295938182 1.0867002452
H -1.8866055156 -5.2438107229 0.3931060644
H -1.6318142304 -4.8670035958 2.1003188721
C 0.7987226033 -3.1959717741 3.5896375449
H 1.4354712038 -3.6039567349 4.38237556
H 0.1925156339 -2.3872225337 4.0097637133
H 0.1352486616 -3.9970917743 3.2535332714
C 2.6686352709 -1.6133388622 2.997595037
H 3.3675109903 -2.0939364076 3.6916960639
H 3.2287124214 -1.1644989763 2.1730515645
H 2.1358944011 -0.8125608477 3.5163417025
C 2.4047922514 -3.7331883849 1.6585530184
H 1.7103801489 -4.4632668876 1.2313752715
H 2.9739732918 -3.2742513395 0.8440770514
H 3.1011069694 -4.2735736529 2.3097605517
C 2.0088496739 0.7653605229 -0.7011369596
C 3.1913494379 0.8546741549 0.0694622512
C 2.0632202748 -0.1496333848 -1.7766161024
H 3.2283506494 1.5421447308 0.9156014075
H 1.1988842046 -0.2506670886 -2.433875022
C 4.3347404733 0.091183267 -0.1985238255
C 3.1896524164 -0.9347169598 -2.0531251726
H 5.227524053 0.2079910797 0.4161516369
H 3.1802095324 -1.6282940962 -2.8941139542
C 4.3342955467 -0.8188315249 -1.2601447683
H 5.2193833693 -1.4156896245 -1.4762550472
O -1.9539141503 3.4880928138 -3.5385691826
S -1.8399389022 2.5010271413 -2.470566237
C -3.2600294199 1.3298388166 -2.7797067757
O -2.038061822 2.9693874344 -1.053889811
O -0.6140203028 1.6262483515 -2.4535559343
F -3.3795631522 0.4260485388 -1.8063870703
F -3.0646653149 0.6913495868 -3.9421590139
F -4.4012802616 2.0284922436 -2.8594796958

Frequency: 9.7317; 16.2030; 17.3413; 19.0897; 21.9209; 26.8843; 27.8862; 29.7422; 32.9429; 33.7996; 36.2734; 39.9502; 46.7909; 53.1867; 55.5643; 59.3258; 60.2239; 66.9645; 71.0583; 80.7348; 89.4160; 91.4818; 100.1614; 112.5828; 118.1393; 125.6706; 129.6209; 137.5819; 139.0007; 144.1018; 146.5005; 162.2121; 175.3541; 178.0865; 185.3593; 203.9374; 206.6427; 209.0568; 212.8785; 213.2062; 217.6535; 220.5883; 224.9236; 227.8722; 248.3560; 254.5711; 267.8140; 297.6805; 303.7773; 306.6078; 307.6665; 320.8534; 324.9442; 325.8226; 339.0292; 347.4968; 351.4130; 352.4460; 357.8111; 393.0456; 395.1303; 450.5114; 452.7959; 481.9953; 486.7774; 489.2631; 490.6994; 491.8005; 520.6169; 523.7252; 529.1666; 557.6947; 558.0869; 558.2986; 568.5227; 570.0698; 577.6051; 600.3306; 614.2190; 622.4503; 626.4297; 632.3516; 634.1216; 647.7240; 648.6224; 718.2316; 735.9476; 760.1818; 761.5799; 761.8708; 766.0951; 781.5094; 792.2922; 844.9100; 869.2438; 907.4254; 937.2327; 941.2396; 972.3923; 976.1545; 978.1704; 979.0800; 981.2863; 987.9629; 988.4051; 1000.0448; 1011.1006; 1033.6204; 1053.3077; 1064.3644; 1073.8001; 1082.2492; 1084.7183; 1108.8148; 1113.2205; 1117.4637; 1144.4289; 1181.9472; 1200.5406; 1212.0205; 1212.6203; 1214.5479; 1215.2778; 1223.1536; 1228.1243; 1230.0314; 1235.9252; 1243.8605; 1246.8587; 1250.8928; 1276.6037; 1287.3273; 1295.4145; 1296.4345; 1305.5638; 1316.4421; 1368.3605; 1396.4111; 1431.1186; 1437.6673; 1443.0032; 1457.5701; 1462.7989; 1499.3137; 1505.8133; 1513.1903; 1518.4413; 1520.0292; 1521.6845; 1528.1555; 1532.0854; 1551.0606; 1611.7943; 1622.5370; 1648.8394; 1839.4843; 1887.8592; 3042.9304; 3059.3937; 3062.5325; 3069.9576; 3101.8891; 3127.2553; 3130.3021; 3131.7404; 3134.1033; 3136.3895; 3142.2944; 3144.7480; 3148.9290; 3155.0079; 3155.4986; 3163.9769; 3172.0308.

Cartesian Forces: Max 0.000001139 RMS 0.000000456

Item	Value	Threshold	Converged?
Maximum Force	0.000000	0.000450	YES
RMS Force	0.000000	0.000300	YES
Maximum Displacement	0.000009	0.001800	YES
RMS Displacement	0.000002	0.001200	YES

FILE: Yb_Sq2_PhYbOTf3_GS_Anion_B3LYP_M062x_Add12_Conf1-1-_Y.out

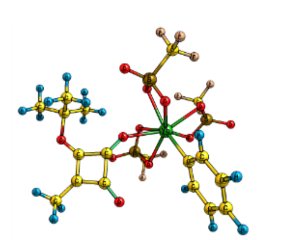

Conformer 5

E(RB3LYP/6-31G(d)/MWB59) = -3731.46192467
Convg = 0.4246D-08 -V/T = 2.0122

Temperature 195.150 Kelvin. Pressure 1.00000 Atm.
elec. and zero-point Energies= -3731.091074
elec. and thermal Energies= -3731.066731
elec. and thermal Enthalpies= -3731.066113
elec. and thermal Free Energies= -3731.140137

RB3LYP/6-31G(d)/MWB59/THF(IEFPCM) = -3731.52897506

RM06-2x/6-311+G(d,p)/MWB59/THF(IEFPCM) = -3731.36616874

Geometry at RB3LYP/6-31G(d)/MWB59
Charge = -1 Multiplicity = 1
Yb 0.1772565123 2.4881298381 0.4391263272
O 1.9268945595 2.338525379 -1.2676999557
S 3.1390275951 2.2164185529 -0.3847291417
O 4.0431529209 1.0965330552 -0.6567686582
O 2.6665879964 2.3922144812 1.0267668715
C 4.1778585202 3.7169001961 -0.7683123974
F 4.5743053711 3.6645636305 -2.0475899416
F 3.5115677769 4.8509182708 -0.5691317165
F 5.2648520897 3.7090036766 0.0185527305
O -1.0548351885 4.2175251326 1.6111484965
O -0.9695735985 6.5779037914 0.6734132331
O 0.796490697 4.8118082514 0.2056428426
S -0.251655343 5.3916710708 1.1186189723
C 0.7156690917 5.9316226519 2.6165051172
F -0.120622956 6.3685942124 3.5661691096
F 1.4286460286 4.9109210177 3.1039671458
F 1.5507862662 6.9243933901 2.2862102868
C 0.6019682282 -2.3754052608 0.8303678435
C -0.5914314552 -2.9399199432 0.413416825
C -1.0728271206 -1.693991517 -0.2215431675
C 0.2316462457 -1.0252874661 0.2803344593
O 0.7351967656 0.0845242748 0.2887176319
O -2.0390190008 -1.3638493278 -0.8620240384
O 1.575252349 -2.9601553775 1.4832636884
C 2.8785072461 -2.3064483503 1.8168533401
C -1.1960502994 -4.2835877453 0.5669056535
H -0.4450560123 -5.0370837711 0.8236744022
H -1.7131646893 -4.5778486256 -0.353492888
H -1.9498266161 -4.2661614632 1.3658771735
C 3.5941051348 -1.9566193206 0.5144641747
H 4.5937314242 -1.5707040452 0.7379081096
H 3.6989084135 -2.8457693092 -0.1180294169
H 3.0802447815 -1.1694245857 -0.0401626578
C 3.5991716073 -3.4276308579 2.5678137277
H 4.5990759843 -3.0907563043 2.8592181112
H 3.0483462133 -3.7034793896 3.4729658284
H 3.7022600672 -4.3167379593 1.9365821095
C 2.6401798673 -1.098115596 2.7192241401
H 2.1894595386 -0.261316396 2.1858103801
H 1.9948114285 -1.365203065 3.5614308754
H 3.6031785952 -0.7537150587 3.1125318559
C -1.3020793604 2.5191972278 -1.4220152798
C -0.8682796674 2.6797498623 -2.7583156356
C -2.7027615867 2.4318100558 -1.2477226508
H 0.1981773589 2.7521577179 -2.9684444719
H -3.1099719911 2.2963854266 -0.2460918138
C -1.7552423948 2.7497858003 -3.8384148443
C -3.6044258146 2.5025727904 -2.3148398061
H -1.3733624954 2.8741481035 -4.8517631887
H -4.675929969 2.4296533753 -2.1302994033
C -3.1315437711 2.6622878565 -3.6190687571
H -3.8277752039 2.7173123167 -4.4549365346
O -0.8366163432 -0.4702292311 3.2793872302
S -0.9083876508 0.9462207077 2.8986223172
C -1.9624788767 1.7217638933 4.2247798229
O -1.6109029411 1.2439318742 1.6037361051
O 0.3782361497 1.7117059007 2.8942109485
F -2.1170059375 3.028510315 4.0279292565
F -1.3801701812 1.5277886836 5.4176243042
F -3.1673008256 1.1360994122 4.2347135383

Frequency: 11.8782; 13.5323; 17.6163; 20.6368; 22.1419; 24.4649; 25.7307; 31.4826; 31.8293; 34.2085; 36.6607; 39.5352; 45.4493; 49.4846; 56.2234; 56.8086; 60.6941; 64.8612; 69.4866; 71.6504; 76.1285; 82.1025; 88.2795; 90.9694; 95.7343; 116.4451; 125.5481; 133.8680; 139.4389; 141.8318; 143.5235; 161.4429; 173.6732; 179.9310; 185.4795; 191.2016; 197.8539; 204.3184; 208.5106; 213.2631; 214.3706; 216.4550; 218.5438; 224.5434; 246.1563; 265.6127; 269.2508; 292.2691; 303.9183; 307.0384; 309.0904; 320.8337; 324.2352; 325.9628; 333.2817; 344.5931; 348.2085; 350.0382; 357.0870; 393.4996; 394.1717; 446.7616; 452.6522; 477.9306; 490.3420; 490.7542; 490.9994; 492.1165; 519.5663; 523.1288; 529.0148; 557.7763; 558.5835; 559.0508; 567.1185; 570.5491; 577.2092; 588.2382; 604.6726; 622.6311; 624.7392; 633.5541; 634.1939; 643.9648; 652.0382; 715.1068; 733.7798; 737.7719; 760.4145; 762.2231; 763.2539; 782.6816; 788.3102; 850.3451; 866.2363; 914.3825; 935.0574; 939.7906; 962.1461; 972.9856; 979.0984; 982.6709; 983.7834; 984.3153; 984.5691; 991.2008; 1012.2647; 1036.3422; 1052.8762; 1059.5453; 1067.2498; 1086.4812; 1086.4872; 1087.3501; 1116.3660; 1128.0072; 1144.0847; 1183.6909; 1199.3646; 1211.3065; 1212.8207; 1215.9752; 1225.9805; 1227.6593; 1229.7754; 1235.5993; 1238.4080; 1247.9546; 1254.0172; 1257.0160; 1275.5469; 1286.8511; 1292.1872; 1296.6667; 1296.9701; 1318.9358; 1370.5836; 1397.8078; 1430.2830; 1439.0922; 1445.1603; 1459.1308; 1461.7559; 1499.5460; 1503.6933; 1505.9199; 1512.7133; 1515.3868; 1522.9512; 1524.7146; 1531.5270; 1547.1647; 1615.4986; 1628.3571; 1648.5276; 1808.2542; 1893.3660; 3043.1934; 3057.1992; 3062.9632; 3072.5153; 3099.9685; 3127.8854; 3131.7619; 3133.5688; 3134.4467; 3135.0132; 3140.5138; 3144.0410; 3158.3772; 3165.1985; 3173.1588; 3174.7864; 3193.8312.

Cartesian Forces: Max 0.000006905 RMS 0.000001010

Item	Value	Threshold	Converged?
Maximum Force	0.000006	0.000450	YES
RMS Force	0.000001	0.000300	YES
Maximum Displacement	0.001158	0.001800	YES
RMS Displacement	0.000177	0.001200	YES

FILE: Yb_Sq2_PhYbOTf3_GS_Anion_B3LYP_M062x_Add12_Conf2.out

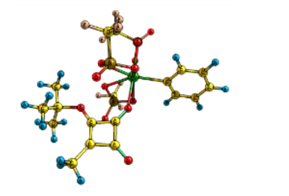

Conformer 6

E(RB3LYP/6-31G(d)/MWB59) = -3731.45884997
Convg = 0.2774D-08 -V/T = 2.0122

Temperature 195.150 Kelvin. Pressure 1.00000 Atm.
elec. and zero-point Energies= -3731.087721
elec. and thermal Energies= -3731.063420
elec. and thermal Enthalpies= -3731.062802
elec. and thermal Free Energies= -3731.136927

RB3LYP/6-31G(d)/MWB59/THF(IEFPCM) = -3731.52424621

RM06-2x/6-311+G(d,p)/MWB59/THF(IEFPCM) = -3731.36186878

Geometry at RB3LYP/6-31G(d)/MWB59
Charge = -1 Multiplicity = 1
Yb -0.2087417496 1.8538422219 0.2240215253
O 1.1814411543 1.0529597208 -1.6577267443
S 2.2781957714 0.382316745 -0.8810377324
O 2.5276248092 -1.033828359 -1.1762668273
O 2.0857819239 0.7419650418 0.5603744639
C 3.843851159 1.2413253799 -1.4114832872
F 4.0631381259 1.01046496 -2.7125323507
F 3.7742807308 2.5552328783 -1.2102107507
F 4.8762212591 0.7490569431 -0.7081764095
O -0.3390893244 3.8046248442 1.649841528
O 0.601653637 5.9893586588 0.7555904575
O 1.3343803292 3.7009858259 -0.0697314735
S 0.7999830069 4.573749894 1.0345526917
C 2.1304677613 4.5120173362 2.3370371428
F 1.7365828016 5.1895957981 3.4226731259
F 2.3861667537 3.2471004467 2.6874431437
F 3.2549934846 5.0661306661 1.8669292946
C -0.026839462 -2.4543815172 0.6645782669
C -0.525819319 -3.6306747844 0.111522306
C -1.4067873852 -2.8999312607 -0.8353112902
C -0.8440816384 -1.5923449422 -0.2385120533
O -0.95544125 -0.3928169793 -0.397508452
O -2.2032789812 -3.2288528602 -1.6823738545
O 0.7813585726 -1.9718432878 1.5576817641
C 1.6266041103 -2.7297789929 2.5015552924
C -0.3752437951 -5.1042915343 0.2321656374
H 0.5797126123 -5.442760281 -0.1908436827
H -1.1789408072 -5.5862386678 -0.3347817649
H -0.419157737 -5.4534008361 1.2692048778
C 0.7140997853 -3.4880974381 3.4677982575
H 1.3137761597 -3.9280724986 4.27252086
H -0.0168627007 -2.8019392919 3.9061093335
H 0.1727928253 -4.2996493068 2.9720271889
C 2.3855290031 -1.6123644976 3.2202547039
H 3.0527412906 -2.0441369972 3.9747471936
H 2.9720522226 -1.0311351878 2.5043647534
H 1.682821138 -0.9286454399 3.7030472203
C 2.5813869069 -3.627344407 1.7116323085
H 2.0641028262 -4.4655114185 1.2360223667
H 3.0874241006 -3.0419929266 0.9377300963
H 3.33581989 -4.0432645142 2.3887799803
C -1.6639718477 2.770105452 -1.4182005272
C -1.1583721465 3.3114547078 -2.6225269807
C -3.070204244 2.8117858586 -1.2801114462
H -0.0823707298 3.3121234527 -2.7994434792
H -3.5303339489 2.4105168871 -0.377412997
C -1.9828477431 3.8529465462 -3.615014988
C -3.9109378216 3.3484300303 -2.2615646761
H -1.5463266428 4.2596397468 -4.526998445
H -4.9903612992 3.3575402451 -2.1110215128
C -3.3678426032 3.8727778066 -3.4365983665
H -4.0164468509 4.2922581101 -4.2044537763
O -2.0838171054 -0.7012692552 3.0072369718
S -1.6223573672 0.6558698881 2.6956610817
C -2.15819301 1.6721582401 4.1619872921
O -2.248493197 1.3306970156 1.5075629852
O -0.1416512501 0.8531869149 2.58499377
F -1.8215152201 2.9516399677 4.0203829777
F -1.5731707166 1.1889006074 5.2696824575
F -3.4870871257 1.5838825723 4.306372351

Frequency: 9.8392; 16.4883; 17.0013; 18.8675; 20.7098; 24.8735; 27.1852; 27.6433; 31.4923; 34.5902; 34.6517; 36.1244; 43.4692; 48.2739; 49.8128; 51.0756; 56.2872; 63.1287; 70.7051; 73.5140; 83.1245; 86.7935; 97.3775; 98.1407; 118.1678; 122.2297; 129.5963; 134.6017; 138.2624; 139.7634; 141.7031; 153.6643; 172.4028; 177.1590; 190.5172; 191.2103; 201.4762; 205.4078; 214.3216; 214.9929; 216.6214; 218.9129; 220.3054; 230.9245; 238.7550; 249.0691; 257.3166; 296.0283; 304.0660; 307.3958; 308.8475; 321.2982; 325.6461; 326.0700; 338.0086; 347.2863; 349.3201; 350.9268; 356.8956; 393.0737; 394.9097; 447.8728; 453.0679; 481.9962; 487.4062; 490.4965; 490.7715; 491.4824; 518.7763; 521.9612; 528.4478; 557.9215; 558.4741; 559.0053; 566.3875; 569.1835; 576.1549; 601.3470; 612.6373; 622.9135; 624.8703; 633.3952; 634.2764; 647.4654; 652.1443; 715.3843; 737.4318; 760.2642; 762.1759; 762.4396; 767.2307; 783.6525; 793.3915; 847.1975; 866.7459; 912.3667; 937.2306; 940.3853; 971.0291; 977.4073; 978.7660; 983.3670; 985.0403; 987.4813; 989.9790; 1002.8447; 1012.0417; 1036.9406; 1053.4389; 1064.1808; 1074.4625; 1086.0038; 1087.0596; 1116.7845; 1120.3503; 1131.0050; 1146.1855; 1183.6823; 1198.3816; 1212.0538; 1212.4297; 1216.1253; 1217.6131; 1224.9614; 1228.3557; 1228.9871; 1235.0635; 1237.7016; 1252.8612; 1254.8232; 1274.8600; 1281.9099; 1287.1823; 1296.0092; 1296.6317; 1318.1483; 1370.1684; 1397.5254; 1430.8018; 1437.0031; 1442.9975; 1459.1142; 1461.6827; 1498.2220; 1505.5841; 1514.2724; 1517.7203; 1519.9161; 1522.8787; 1527.8473; 1529.7588; 1549.3367; 1615.3475; 1628.0597; 1654.3592; 1834.5879; 1888.7294; 3043.6621; 3061.2988; 3062.4001; 3070.5014; 3102.3222; 3126.5765; 3130.0165; 3133.8895; 3134.0028; 3140.8742; 3145.3511; 3148.2801; 3151.4922; 3153.4072; 3161.6968; 3165.9135; 3174.3950.

Cartesian Forces: Max 0.000004249 RMS 0.000000505

Item	Value	Threshold	Converged?
Maximum Force	0.000002	0.000450	YES
RMS Force	0.000000	0.000300	YES
Maximum Displacement	0.001358	0.001800	YES
RMS Displacement	0.000225	0.001200	YES

FILE: Yb_Sq2_PhYbOTf3_GS_Anion_B3LYP_M062x_Add12_Conf2-.out

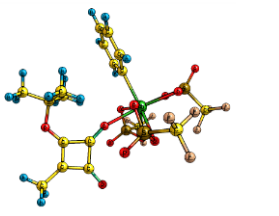

Conformer 7

E(RB3LYP/6-31G(d)/MWB59) = -3731.46375581
Convg = 0.5110D-08 -V/T = 2.0122

Temperature 195.150 Kelvin. Pressure 1.00000 Atm.
elec. and zero-point Energies= -3731.092595
elec. and thermal Energies= -3731.068409
elec. and thermal Enthalpies= -3731.067791
elec. and thermal Free Energies= -3731.141570

RB3LYP/6-31G(d)/MWB59/THF(IEFPCM) = -3731.52969490

RM06-2x/6-311+G(d,p)/MWB59/THF(IEFPCM) = -3731.36868458

Geometry at RB3LYP/6-31G(d)/MWB59
Charge = -1 Multiplicity = 1
Yb 0.3655705399 2.5356388548 0.2252875128
O 0.7103735407 2.2897813314 2.6540974235
S -0.7327233066 2.3592755291 3.0810049835
O -1.2269543786 1.2407517394 3.8849595007
O -1.5222942248 2.7444148372 1.8663703482
C -0.8258525758 3.8300308748 4.2208865392
F -0.0714364968 3.6005086942 5.305512692
F -0.3948459421 4.9395951545 3.6233603796
F -2.0950881753 4.0110167436 4.6107478759
O 0.4801772906 4.2802431046 -1.4462078318
O 1.5776618556 6.4799007568 -0.8037884232
O 0.6003625612 4.8500690492 0.8827387393
S 0.597100481 5.4465625221 -0.5004192081
C -1.0635648702 6.2820142599 -0.6184699852
F -1.2410830078 6.7706112989 -1.8517904454
F -2.0407931726 5.4096144324 -0.3524601014
F -1.1298969533 7.2898168331 0.2597273931
C -0.1894398493 -2.2258478311 1.3813215277
C -1.4935646935 -2.6570318376 1.5549804986
C -2.0745960633 -1.3202348432 1.2809291856
C -0.6220353972 -0.8189862054 1.0817806458
O -0.0454125762 0.215721965 0.8099603688
O -3.1813703307 -0.846856909 1.2367252386
O 0.9191266982 -2.9183614346 1.4777190509
C 2.2789075951 -2.3216251195 1.2612204653
C -2.1066624837 -3.9638198447 1.8900410532
H -1.3504060837 -4.7415839228 2.0302978883
H -2.7949614094 -4.2751309237 1.0938113042
H -2.7051342073 -3.8784705963 2.8060749145
C 2.3821092256 -1.8136577198 -0.1754677533
H 3.3979813969 -1.4491558982 -0.3580833035
H 2.166916258 -2.6218044682 -0.8837235062
H 1.7113412518 -0.9756746648 -0.3698578196
C 3.18740883 -3.5305407399 1.4881318283
H 4.2322566954 -3.2313782513 1.3584398146
H 3.0627458762 -3.9269611474 2.5013385885
H 2.9625212198 -4.3269257651 0.7708796464
C 2.5255861688 -1.2393230515 2.3103310355
H 1.8597601863 -0.3837231084 2.1894803988
H 2.4083041562 -1.650286023 3.3194974881
H 3.5462155904 -0.8583532286 2.2036871254
C 2.7437130718 2.1379729072 0.1845637166
C 3.5598139543 2.2769492498 1.3319796794
C 3.4276734078 1.7407636956 -0.9886511251
H 3.1061995448 2.581810468 2.2740707028
H 2.8678537804 1.613632464 -1.9142763646
C 4.9382441686 2.0292922776 1.3220328195
C 4.8048741417 1.4882923566 -1.0201394935
H 5.5220524246 2.1533486653 2.2342573095
H 5.283645529 1.1863822608 -1.9517914725
C 5.5685419681 1.6283402545 0.1414024434
H 6.640470744 1.4357884127 0.1248310241
O -1.5805666716 -0.2467314718 -2.2382508616
S -1.0300000396 1.1020195929 -2.0968861396
C -1.2851719826 1.8876167976 -3.7668128999
O 0.4522092104 1.1958000267 -1.8453106322
O -1.6920077242 2.0230448333 -1.1167766569
F -0.8199978042 3.1352969403 -3.7959253436
F -2.5936621699 1.9044751601 -4.055235147
F -0.6483237678 1.1654149659 -4.7001125791

Frequency: 6.8716; 14.0696; 17.3427; 18.8426; 24.5247; 25.7706; 27.8384; 29.7614; 30.2320; 36.1508; 38.4734; 40.1937; 46.4876; 48.3878; 56.4643; 59.7839; 61.6385; 63.9277; 72.2874; 76.2137; 82.4649; 83.8065; 86.9124; 92.2376; 111.0204; 126.4985; 135.5660; 139.7563; 140.5972; 144.3835; 145.9805; 163.6277; 179.4660; 180.1265; 186.0267; 188.2383; 204.9361; 207.7645; 209.6283; 214.1855; 216.0535; 217.1471; 217.7230; 228.4490; 253.3756; 269.2121; 270.2032; 290.5225; 304.5416; 307.4753; 308.3051; 321.0656; 324.8868; 325.1211; 329.4926; 349.3327; 350.1385; 350.2628; 357.5125; 394.2173; 395.4755; 448.7788; 453.2507; 476.7087; 490.1353; 490.9459; 491.0656; 492.0910; 520.1647; 523.3188; 529.1518; 557.9437; 558.2849; 558.7235; 567.3074; 570.3804; 577.4366; 596.1416; 601.6927; 622.6045; 625.4589; 634.3890; 634.4549; 647.4560; 648.2822; 718.0222; 731.0179; 738.8734; 760.9018; 762.3043; 762.6459; 784.1946; 786.3252; 845.1013; 869.6616; 915.6505; 936.9130; 939.2328; 958.7356; 977.2003; 977.7457; 980.4615; 983.2770; 985.1584; 985.9407; 989.4307; 1011.7198; 1032.8287; 1056.4360; 1063.4767; 1067.3257; 1084.7293; 1084.8457; 1088.6528; 1115.3309; 1120.9526; 1134.7792; 1182.6105; 1198.5392; 1213.7649; 1213.9933; 1215.5508; 1226.3703; 1229.7051; 1230.5908; 1237.2192; 1239.5026; 1242.3480; 1253.1192; 1253.5140; 1287.3381; 1292.0071; 1293.3001; 1294.5191; 1296.5633; 1318.5304; 1370.3146; 1397.7179; 1431.2254; 1441.8014; 1445.7880; 1457.7053; 1464.5835; 1501.1035; 1502.3250; 1506.4232; 1514.8139; 1514.9402; 1521.2909; 1529.0009; 1529.9940; 1548.1802; 1612.7341; 1624.0355; 1652.0199; 1827.8451; 1896.9485; 3044.2047; 3057.4586; 3063.6201; 3068.8568; 3095.7759; 3128.1914; 3132.0577; 3132.9760; 3134.3843; 3136.8483; 3141.1234; 3143.4653; 3162.3563; 3167.9252; 3175.1976; 3179.1708; 3182.0660.

Cartesian Forces: Max 0.000000649 RMS 0.000000279

Item	Value	Threshold	Converged?
Maximum Force	0.000000	0.000450	YES
RMS Force	0.000000	0.000300	YES
Maximum Displacement	0.000267	0.001800	YES
RMS Displacement	0.000057	0.001200	YES

FILE: Yb_Sq2_PhYbOTf3_GS_Anion_B3LYP_M062x_Add12_Conf2-1.out

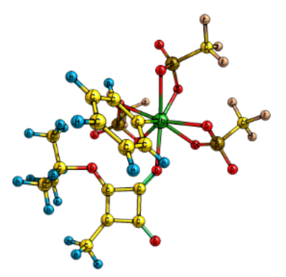

Conformer 8

E(RB3LYP/6-31G(d)/MWB59) = -3731.45745423
Convg = 0.8667D-09 -V/T = 2.0122

Temperature 195.150 Kelvin. Pressure 1.00000 Atm.
elec. and zero-point Energies= -3731.085592
elec. and thermal Energies= -3731.061889
elec. and thermal Enthalpies= -3731.061271
elec. and thermal Free Energies= -3731.132209

RB3LYP/6-31G(d)/MWB59/THF(IEFPCM) = -3731.52353703

RM06-2x/6-311+G(d,p)/MWB59/THF(IEFPCM) = -3731.36395186

Geometry at RB3LYP/6-31G(d)/MWB59
Charge = -1 Multiplicity = 1
Yb 0.2242848911 2.0834307751 0.4818284702
O 1.105084217 1.802429621 2.7976747458
S -0.142365974 1.2464240865 3.4228623017
O 0.0030609806 0.0647910232 4.2772063853
O -1.1709387033 1.1794268543 2.3289684423
C -0.7531769081 2.5684758097 4.5874142454
F 0.1807768803 2.8057061531 5.5160602904
F -1.0246257412 3.696545352 3.9381727403
F -1.8666054812 2.1350926927 5.1952595555
O 2.14474289 3.6187037542 0.5850558409
O 1.9989761709 5.4431363925 2.34900203
O -0.0291502898 4.3896944907 1.2511079459
S 1.4205753461 4.7951040752 1.1767019008
C 1.4859168804 6.0982546407 -0.1569395039
F 2.7329173954 6.5814825964 -0.2429501209
F 1.1396852166 5.5970442364 -1.3446260257
F 0.6553551945 7.1027844285 0.1506539813
C -0.2019351883 -2.1150132467 0.7843780991
C -0.9150494292 -3.2093668248 0.2976218892
C -1.7636790012 -2.3661301289 -0.5784280582
C -0.9769723157 -1.1449171419 -0.0608063995
O -0.955201466 0.0522958024 -0.2521271494
O -2.669787171 -2.5853704963 -1.3481546603
O 0.7563641382 -1.7789441316 1.5979682359
C 1.5786113783 -2.7119596291 2.4149364528
C -0.9824242961 -4.6901273381 0.4158554882
H -0.878201464 -5.0488881036 1.4440728264
H -0.1977981158 -5.1706757032 -0.1838911009
H -1.9466410027 -5.0262826622 0.0188063813
C 0.6792432799 -3.3286303366 3.4874828757
H 1.2858186725 -3.9417583213 4.1636053933
H 0.2022526416 -2.5333151607 4.0669315043
H -0.0974798547 -3.9701327932 3.0605620149
C 2.6204751699 -1.7865195738 3.043382969
H 3.288494881 -2.3764713052 3.6815537389
H 3.2102737798 -1.2945827314 2.2656468483
H 2.131907694 -1.0209510079 3.6495510937
C 2.250684098 -3.730535506 1.4920684321
H 1.5407484677 -4.4335171684 1.0495589304
H 2.7879860852 -3.2138623268 0.6901887218
H 2.9749120358 -4.3135538457 2.0718556879
C 2.0212421408 0.699904139 -0.3808977885
C 3.2802060023 0.6797216585 0.2641760464
C 1.9293542747 -0.1245878299 -1.5253848903
H 3.4406588807 1.3118818795 1.1370524576
H 1.0042083239 -0.1395527103 -2.1034961095
C 4.3482188378 -0.1155413972 -0.1698561484
C 2.9840339377 -0.9272336075 -1.979642763
H 5.2988224225 -0.0919539934 0.3632472002
H 2.8597790673 -1.5393079933 -2.8730822407
C 4.2015090399 -0.9333677397 -1.2945496538
H 5.0293962984 -1.5495377584 -1.6425158287
O -2.5035513001 2.2683482323 -2.7180377041
S -1.7693681082 2.9419021398 -1.6523524771
C -2.1384283544 4.7513386511 -1.925199836
O -0.2570593574 2.8470844372 -1.7093835779
O -2.1511242446 2.664143984 -0.2323181055
F -1.6519877535 5.5134251908 -0.9450052608
F -3.4672145644 4.9206389028 -1.9785866019
F -1.606984436 5.1474626483 -3.0888936535

Frequency: 10.9089; 18.0362; 19.1408; 23.3911; 27.2878; 29.1097; 35.4775; 36.1742; 39.1935; 44.3258; 49.0543; 51.1131; 53.2394; 58.6067; 65.0491; 66.4756; 72.1654; 75.0134; 79.4654; 80.6137; 90.5353; 95.1087; 99.9926; 113.1086; 124.5832; 128.0173; 131.8818; 136.3134; 145.9177; 146.5572; 152.8102; 165.4825; 173.4163; 181.9691; 183.4988; 202.9146; 207.5426; 208.5299; 215.1418; 215.5643; 221.1293; 223.7435; 229.1657; 235.7409; 249.4380; 253.4070; 278.1775; 298.6985; 303.1991; 307.4087; 308.8655; 321.3919; 323.5112; 326.6148; 340.2185; 350.4647; 351.9683; 357.5561; 358.8630; 395.8121; 396.6422; 452.0803; 452.9067; 481.7918; 486.0892; 489.8269; 490.1084; 491.2642; 523.3767; 523.9357; 529.4036; 558.0484; 558.4438; 558.8995; 570.0135; 571.9090; 575.2789; 602.8522; 610.7458; 620.9332; 624.4132; 634.3977; 635.4896; 645.5539; 647.1014; 719.8546; 735.1031; 760.3171; 761.1125; 762.7463; 763.6573; 783.1763; 790.4454; 838.3548; 869.6493; 908.0480; 937.8061; 941.5141; 970.3447; 974.3065; 976.9090; 980.2025; 982.1119; 986.7608; 989.9889; 998.3628; 1011.2994; 1031.2622; 1053.4556; 1066.7149; 1074.1495; 1083.2579; 1083.7074; 1102.9056; 1111.5311; 1118.7805; 1136.2260; 1182.0406; 1200.5029; 1208.8403; 1210.1703; 1212.0021; 1214.3309; 1224.4895; 1226.1304; 1228.7679; 1234.5631; 1246.9249; 1252.0662; 1258.0727; 1284.0234; 1291.0169; 1295.2543; 1296.6451; 1302.6951; 1312.9998; 1368.9717; 1392.2054; 1430.7854; 1436.9687; 1441.9186; 1457.4752; 1463.0207; 1501.1471; 1506.2917; 1514.4531; 1520.4950; 1521.9667; 1523.9924; 1530.9802; 1534.6498; 1556.1296; 1611.3785; 1622.1318; 1644.4887; 1838.8454; 1885.9033; 3042.6229; 3061.8245; 3063.1477; 3070.8255; 3101.6655; 3128.5906; 3128.9899; 3131.2468; 3135.5682; 3138.1330; 3143.2327; 3150.1271; 3155.9947; 3158.4225; 3161.4277; 3173.3554; 3174.0524.

Cartesian Forces: Max 0.000000900 RMS 0.000000224

Item	Value	Threshold	Converged?
Maximum Force	0.000001	0.000450	YES
RMS Force	0.000000	0.000300	YES
Maximum Displacement	0.000067	0.001800	YES
RMS Displacement	0.000012	0.001200	YES

FILE: Yb_Sq2_PhYbOTf3_GS_Anion_B3LYP_M062x_Add12_Conf2-1-_a.out

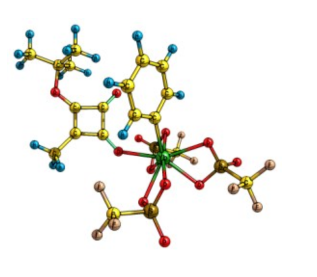

Conformer 9

E(RB3LYP/6-31G(d)/MWB59) = -3731.46166931
Convg = 0.5738D-08 -V/T = 2.0122

Temperature 195.150 Kelvin. Pressure 1.00000 Atm.
elec. and zero-point Energies= -3731.090340
elec. and thermal Energies= -3731.066345
elec. and thermal Enthalpies= -3731.065727
elec. and thermal Free Energies= -3731.138325

RB3LYP/6-31G(d)/MWB59/THF(IEFPCM) = -3731.53326029

RM06-2x/6-311+G(d,p)/MWB59/THF(IEFPCM) = -3731.36555211

Geometry at RB3LYP/6-31G(d)/MWB59
Charge = -1 Multiplicity = 1
Yb 3.0166472445 1.4037054766 -1.5072183197
O 1.6760034576 2.566840337 -3.1924766965
S 1.4077169449 3.8338439601 -2.4255342706
O 1.6258867315 5.1044967986 -3.1071733456
O 2.0066717263 3.6428701628 -1.065509524
C -0.4336323354 3.788486368 -2.1324190906
F -1.0921779606 3.9318490244 -3.2874836159
F -0.8084157828 2.6173546837 -1.5785742973
F -0.7955505468 4.7752773167 -1.3032730507
O 4.6035123581 0.6708846358 -3.2115743012
O 6.9068151538 1.7370353367 -3.1755549874
O 4.8165845708 2.8845690428 -2.3080893645
S 5.4600816381 1.9079675828 -3.2568869458
C 5.168911985 2.6200356104 -4.9549069898
F 5.6814300505 1.7920795527 -5.8765621146
F 3.8693305823 2.7834724181 -5.1976560438
F 5.7841455964 3.8058483268 -5.0532883563
C -0.6790954751 -1.3258874496 1.2562023745
C -0.7290452236 0.0517285017 1.1167154334
C 0.6315673019 0.1250090675 0.5969208497
C 0.7790901942 -1.3983413918 0.813039701
O 1.6278597952 -2.2450821754 0.7472590946
O 1.322935728 1.0364200478 0.164065564
O -1.627247452 -2.1430790939 1.644857634
C -1.4475897196 -3.6242557876 1.6498155071
C -1.7677203322 1.0867314417 1.3427316681
H -2.7502567343 0.6369236499 1.5136134745
H -1.8174045596 1.7618077284 0.4819841253
H -1.5109640968 1.7026470958 2.2145205196
C -1.1232638575 -4.0990530586 0.2327823266
H -0.1391246927 -3.7615203042 -0.0999249582
H -1.1268454615 -5.1948239085 0.2119809513
H -1.8756621652 -3.7411596136 -0.477283851
C -2.8250155278 -4.1100809904 2.1022732583
H -2.8330428765 -5.2035010013 2.1556488171
H -3.0712650328 -3.7122610912 3.092256958
H -3.5991068183 -3.7915130141 1.3967909832
C -0.3623546848 -3.993851589 2.6623234803
H -0.5917304119 -3.5697266581 3.6459850111
H -0.3182987214 -5.0841873099 2.7631057712
H 0.6201661936 -3.6455149406 2.3351690673
O 4.3982687134 -0.0387437144 -0.1827055449
S 4.5747529404 0.8144194904 1.0521996644
C 6.4075410856 1.1358048573 1.1129418934
O 4.257959936 0.1914641809 2.336251292
O 3.9514078488 2.1399293739 0.7463296831
F 6.6828875934 1.961196395 2.1331118259
F 6.8312123669 1.6921143521 -0.0212363093
F 7.0624972649 -0.0169277699 1.3051516939
C 1.8294082765 -0.5424690248 -2.3532353406
C 2.5089811825 -1.7684247544 -2.5385552804
C 0.4460045779 -0.5827983328 -2.6442599707
C 1.8705928001 -2.938698231 -2.9645501055
C -0.2152807795 -1.741988833 -3.0732391466
C 0.4987532883 -2.9318482565 -3.2337029264
H 3.5787819196 -1.8191318655 -2.3379234659
H -0.1495007742 0.3263927311 -2.5466239074
H 2.4425348503 -3.8581288172 -3.0882404235
H -1.2827868005 -1.7135098033 -3.2954641988
H -0.003005631 -3.8369950183 -3.5737704344

Frequency: 11.5246; 12.9047; 16.9425; 21.9046; 26.0623; 27.9315; 31.4743; 35.4375; 36.7494; 40.1378; 43.6464; 44.5017; 48.4692; 49.6437; 51.9691; 54.8242; 62.5900; 67.6206; 72.2956; 74.6352; 82.9704; 96.2438; 101.8747; 105.7391; 111.9559; 124.8384; 133.2014; 138.6326; 143.2396; 144.3816; 150.9874; 163.0040; 172.9042; 177.2271; 182.9674; 205.1759; 208.2657; 208.9824; 210.3535; 212.3431; 213.9111; 217.4343; 220.2167; 230.3296; 255.6030; 266.6953; 277.7525; 302.0057; 305.4397; 306.9986; 313.3891; 321.0240; 323.3835; 324.3779; 339.8195; 348.3554; 350.3553; 352.2513; 357.7321; 390.8938; 396.6072; 453.1374; 458.4182; 471.0610; 488.8001; 489.4036; 490.7461; 495.2548; 522.9293; 524.4055; 528.4999; 555.6575; 557.8869; 558.2765; 570.3651; 571.9965; 573.0237; 594.1323; 608.9864; 623.7456; 627.0326; 631.0848; 634.2464; 634.9642; 645.5341; 718.1693; 735.0598; 747.7212; 755.4430; 761.7113; 762.1871; 777.3543; 787.4293; 855.3550; 867.8001; 907.6632; 936.3974; 941.0253; 957.3065; 973.2547; 976.9704; 979.6757; 982.2230; 984.9025; 985.2488; 997.5950; 1011.6456; 1031.9788; 1062.8979; 1065.0855; 1069.9861; 1082.5219; 1083.9813; 1087.4551; 1111.4529; 1124.1751; 1141.6759; 1181.1234; 1195.2736; 1206.0780; 1212.4812; 1215.8819; 1223.1256; 1225.0104; 1230.7933; 1232.3599; 1238.2795; 1249.7725; 1252.9011; 1258.8678; 1287.3915; 1294.9176; 1298.0540; 1302.0219; 1305.8118; 1314.2814; 1369.4742; 1402.4903; 1431.8994; 1434.7527; 1449.3506; 1457.5696; 1460.9725; 1502.3246; 1506.0015; 1511.1638; 1513.9262; 1519.0701; 1520.8995; 1534.6207; 1537.8493; 1553.8540; 1611.2548; 1622.6446; 1638.6009; 1823.4915; 1886.8645; 3051.1996; 3057.3940; 3060.0374; 3067.2011; 3112.8076; 3122.4105; 3125.1203; 3127.7909; 3134.2583; 3138.2963; 3139.5184; 3145.8406; 3147.4380; 3159.2858; 3160.3657; 3166.5240; 3171.6160.

Cartesian Forces: Max 0.000006108 RMS 0.000001334

Item	Value	Threshold	Converged?
Maximum Force	0.000005	0.000450	YES
RMS Force	0.000001	0.000300	YES
Maximum Displacement	0.001654	0.001800	YES
RMS Displacement	0.000187	0.001200	YES

FILE: Yb_Sq2_PhYbOTf3_GS_Anion_B3LYP_M062x_Add12_Conf3.out

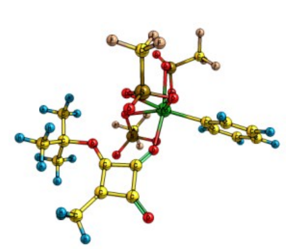

Conformer 10

E(RB3LYP/6-31G(d)/MWB59) = -3731.45825832
A.U. after 1 cycles Convg = 0.9487D-09 -V/T = 2.0122

Temperature 195.150 Kelvin. Pressure 1.00000 Atm.
elec. and zero-point Energies= -3731.086999
elec. and thermal Energies= -3731.062795
elec. and thermal Enthalpies= -3731.062177
elec. and thermal Free Energies= -3731.135666

RB3LYP/6-31G(d)/MWB59/THF(IEFPCM) = -3731.52301136

RM06-2x/6-311+G(d,p)/MWB59/THF(IEFPCM) = -3731.36082664

Geometry at Geometry at RB3LYP/6-31G(d)/MWB59
Charge = -1 Multiplicity = 1
Yb -1.4142805245 -2.0064315139 -3.2746721793
O -1.4785115358 -4.4604255151 -3.2402309332
S -0.0941443134 -4.7363112936 -2.719433342
O 0.0098953675 -5.5598923755 -1.5114147111
O 0.6364617826 -3.4294376748 -2.7070426103
C 0.7174590338 -5.7578645381 -4.0488858123
F 0.1035860324 -6.9458765221 -4.1374351532
F 0.6619746212 -5.1532784753 -5.2329439518
F 2.003639393 -5.9630266487 -3.7244447375
O -0.4016364657 -2.4273981039 -5.4121819874
O 0.4915557555 -0.433210554 -6.6994136304
O -1.4053752822 -0.2818463384 -5.0231779622
S -0.6404817596 -1.0770541546 -6.0430407994
C -1.8570716216 -1.4176690844 -7.4137095051
F -1.2472686984 -2.1163002289 -8.3816256331
F -2.9021187694 -2.1137272496 -6.9719397297
F -2.2862278822 -0.2569006178 -7.9252556438
C -0.0456670587 -2.7225436726 0.7814696247
C -0.5126987851 -3.1002832779 2.0378182469
C -1.9100765478 -3.2481665986 1.5575980025
C -1.4009038771 -2.8317951231 0.1615232242
O -1.8483210301 -2.6533353403 -0.9539314307
O -2.9592089832 -3.5532693253 2.0738160565
O 1.0135371842 -2.372338869 0.1185631955
C 2.3761269923 -2.1622583776 0.6506802408
C -0.014122154 -3.3419031569 3.4169479549
H 0.5351916598 -4.2906843128 3.4797264102
H -0.8729005966 -3.4121475881 4.093174589
H 0.6519423604 -2.5499375153 3.7746808992
C 2.3439833186 -0.9771101191 1.6178557026
H 3.3679055118 -0.7043111422 1.8973849055
H 1.8631160272 -0.1195713046 1.1388324098
H 1.7981777208 -1.2122767984 2.5371224666
C 3.1633121074 -1.8235515524 -0.6167404008
H 4.2109470625 -1.6287052764 -0.3613764782
H 3.1116127762 -2.6526579113 -1.3275102041
H 2.7367466123 -0.943223177 -1.1034606088
C 2.8802713374 -3.4616540236 1.2817528798
H 2.3483288998 -3.7101812069 2.2041220344
H 2.7687711169 -4.2916133218 0.5770398632
H 3.9426486687 -3.3569586834 1.528866904
C -3.7489175458 -2.1999122466 -3.7204593767
C -4.4592619352 -3.42154346 -3.7526438511
C -4.5313927761 -1.0417961759 -3.9371166119
H -3.9217867716 -4.3550261798 -3.589850156
H -4.0518713205 -0.0624836434 -3.9275231954
C -5.837795361 -3.4899867867 -3.9848348995
C -5.9106092771 -1.089401256 -4.1678631408
H -6.3416211779 -4.4563948831 -4.0007435054
H -6.4707541028 -0.1682932018 -4.3282034263
C -6.5710305691 -2.3199234167 -4.1936368794
H -7.6443341539 -2.3660341783 -4.3731692179
O -0.1893315705 0.6798527328 -0.2534180613
S -0.5173713405 0.4790619551 -1.6699831614
C -0.3795992169 2.1862304909 -2.4059078631
O -1.9170869954 0.0262416753 -1.9776773368
O 0.412867237 -0.3897835424 -2.4605179687
F -0.6684468822 2.1915444049 -3.70314187
F 0.8737978077 2.6346195865 -2.2358526715
F -1.2205470866 3.0118599548 -1.7676752101

Frequency: 13.5759; 14.8382; 18.5417; 21.0814; 23.7104; 25.5266; 28.2294; 28.9710; 31.1316; 32.7897; 34.7932; 35.7369; 41.2858; 47.8871; 53.4623; 55.6861; 57.5316; 64.0439; 76.9173; 79.9803; 84.3845; 87.7274; 98.5834; 102.6473; 118.3068; 121.8305; 131.3332; 135.4992; 138.9092; 143.6183; 150.6148; 156.3429; 166.9785; 172.0734; 190.2912; 192.9963; 202.4512; 205.4721; 213.3585; 215.1626; 216.7823; 218.6526; 221.8063; 229.2502; 241.4514; 250.3311; 258.7779; 296.4176; 304.8556; 307.5302; 308.4712; 323.0049; 325.2002; 325.6655; 338.4322; 347.5230; 349.5017; 350.4756; 356.8251; 394.1675; 395.4954; 447.9467; 451.9364; 481.1732; 487.0575; 490.5570; 491.2017; 491.6579; 519.4282; 522.6384; 528.2585; 558.6201; 558.7592; 559.0580; 566.8147; 569.6942; 574.1086; 600.7202; 612.5993; 624.9812; 626.0534; 633.5524; 634.4447; 647.0541; 651.5557; 716.1317; 737.1674; 762.3938; 762.5960; 762.7640; 766.6955; 783.4954; 792.8988; 845.4771; 867.3762; 912.5364; 936.7458; 939.6366; 972.3798; 977.5527; 979.6261; 982.7544; 985.6903; 986.2480; 989.3048; 1002.0394; 1012.0540; 1036.0415; 1053.4461; 1063.5458; 1073.7924; 1085.6742; 1087.1467; 1115.8378; 1116.3943; 1129.4058; 1144.2877; 1183.4420; 1199.3308; 1210.4347; 1211.5569; 1213.1569; 1216.0709; 1225.3741; 1227.5966; 1229.0056; 1232.5531; 1254.0790; 1255.0445; 1256.9854; 1276.6054; 1285.5797; 1287.0177; 1295.9895; 1296.3658; 1310.5629; 1370.2408; 1396.9146; 1430.4974; 1437.0306; 1442.7550; 1459.1165; 1461.6470; 1498.3748; 1506.1215; 1513.6326; 1518.4512; 1520.1760; 1522.6725; 1528.1470; 1529.9303; 1550.1625; 1615.0035; 1627.5395; 1653.3245; 1834.1459; 1888.5274; 3042.7603; 3060.6821; 3062.9670; 3070.5450; 3102.0000; 3125.7690; 3129.7536; 3132.7777; 3133.4518; 3141.1823; 3144.2955; 3151.3685; 3153.6402; 3154.3184; 3164.2139; 3164.7516; 3174.4162.

Cartesian Forces: Max 0.000000585 RMS 0.000000244

Item	Value	Threshold	Converged?
Maximum Force	0.000000	0.000450	YES
RMS Force	0.000000	0.000300	YES
Maximum Displacement	0.000039	0.001800	YES
RMS Displacement	0.000005	0.001200	YES

FILE: Yb_Sq2_PhYbOTf3_GS_Anion_B3LYP_M062x_Add12_Conf3-_Y.out

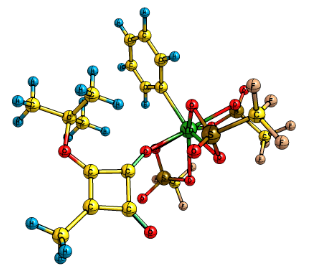

Conformer 11

E(RB3LYP/6-31G(d)/MWB59) = -3731.4637496
Convg = 0.6320D-08 -V/T = 2.0122

Temperature 195.150 Kelvin. Pressure 1.00000 Atm.
elec. and zero-point Energies= -3731.092625
elec. and thermal Energies= -3731.068414
elec. and thermal Enthalpies= -3731.067796
elec. and thermal Free Energies= -3731.14189

RB3LYP/6-31G(d)/MWB59/THF(IEFPCM) = -3731.52999159

RM06-2x/6-311+G(d,p)/MWB59/THF(IEFPCM) = -3731.36905881

Geometry at RB3LYP/6-31G(d)/MWB59
Charge = -1 Multiplicity = 1
Yb 0.449424657 2.6406693288 0.5756786203
O 0.0839647738 2.0446201875 2.9418163585
S -1.4222764449 2.0610124792 2.9327894733
O -2.0878031795 0.840647377 3.3908957871
O -1.8384427021 2.6231918827 1.6069541559
C -1.896361325 3.3513447619 4.1902817146
F -1.4832363613 2.9614308044 5.4049852712
F -1.3496819649 4.5326227849 3.9080004725
F -3.2297008233 3.4831343785 4.211652431
O 0.9701566806 4.6105406514 -0.7263478372
O 1.7658998234 6.68356806 0.5086437229
O 0.4085533027 4.8319682537 1.5971972276
S 0.7754610064 5.6247267287 0.3701411471
C -0.8117500398 6.4814112935 -0.0952707844
F -0.6496850738 7.1478623769 -1.2445578765
F -1.7934187377 5.5869089686 -0.2470251915
F -1.1589333901 7.3492016682 0.8627015187
C -0.2545485037 -2.2298552222 0.8891347883
C -1.53852146 -2.6725331374 0.6203234744
C -2.0568087376 -1.3088925491 0.352753898
C -0.6259298402 -0.7941994837 0.6504250011
O -0.0283923284 0.2634123357 0.6880568235
O -3.1169789259 -0.8281126733 0.0431261457
O 0.7996565371 -2.934785775 1.2200012839
C 2.1433638538 -2.3225421125 1.4876835977
C -2.1811822821 -4.007622136 0.6010425438
H -1.4734434849 -4.8012407926 0.8575285935
H -2.6018595276 -4.2119294985 -0.3918910932
H -3.0198712077 -4.0351001304 1.3084643895
C 2.6449438414 -1.6363849487 0.2186352524
H 3.658134431 -1.2583901449 0.38822598
H 2.6706830817 -2.3462414153 -0.6159298637
H 2.0340580965 -0.7760078602 -0.0580574402
C 2.9845102549 -3.5554201258 1.8206318644
H 4.0114335784 -3.2478652584 2.0416725227
H 2.5820994883 -4.0768214181 2.6955384349
H 3.0047692793 -4.2528638063 0.9765712015
C 2.03808536 -1.383939402 2.6880039722
H 1.410587423 -0.5153853045 2.4834952755
H 1.6434099898 -1.9191685172 3.5591046216
H 3.0328063232 -0.9992720661 2.93452282
C 2.7500959229 2.2369208944 1.1760674862
C 3.194079735 2.2204583419 2.5194339195
C 3.7567905498 1.9913884318 0.2126325059
H 2.4775856743 2.4025026055 3.3193493034
H 3.4932167481 1.9892893617 -0.8441623643
C 4.5235216279 1.9678171926 2.880268632
C 5.0914357572 1.7365577942 0.5521256723
H 4.8138843119 1.9686489609 3.9310090613
H 5.8288989929 1.5552759104 -0.2299552355
C 5.4811667239 1.719776707 1.8936816074
H 6.5175613191 1.5244130933 2.1659035103
O -0.5968321904 0.2553628113 -2.7138133135
S -0.1614717702 1.5656273428 -2.2285544733
C 0.0490596129 2.5881742446 -3.7716655185
O 1.1799131669 1.6137204671 -1.5447030456
O -1.112570347 2.3375771793 -1.3643799316
F 0.4571834016 3.8236988042 -3.4873192816
F -1.1197248039 2.655510658 -4.4236519835
F 0.9544694036 2.005609382 -4.5711058607

Frequency: 5.1668; 12.9707; 17.9556; 18.5699; 23.9448; 25.5219; 27.8171; 28.5551; 30.1380; 36.5130;38.3289; 39.7686; 44.6667; 47.9230; 56.1471; 59.4353; 61.3469; 63.6094; 72.0429; 76.6036; 82.5166; 8.8417; 86.4414; 92.7040; 110.9939; 126.5044; 135.3759; 139.6866; 140.5080; 144.3893; 145.4668; 164.012; 179.4435; 179.9827; 186.0495; 188.2030; 203.3877; 207.6152; 209.4848; 214.0478; 215.8649; 216.773; 217.9089; 228.2309; 252.1293; 269.0460; 269.7595; 290.2750; 304.4338; 307.3977; 308.2176; 321.088; 324.7496; 325.0006; 329.3707; 349.2637; 350.1572; 350.1819; 357.4821; 394.1634; 395.4436; 448.7579 453.0223; 476.7357; 490.1490; 490.9335; 490.9886; 491.7526; 520.1946; 523.3524; 529.0954; 557.9546;558.2839; 558.7233; 567.3065; 570.3858; 577.3781; 596.1355; 601.6360; 622.5715; 625.4157; 634.3690;
34.4385; 647.4900; 648.2562; 718.1389; 730.9869; 738.9168; 760.8956; 762.2932; 762.6308; 784.2115; 76.2214; 844.9213; 869.8541; 915.7058; 936.3953; 938.9094; 958.7120; 977.3677; 977.8098; 980.5050; 98.2863; 985.3128; 985.9472; 989.4570; 1011.7277; 1032.7685; 1056.3584; 1063.0942; 1067.4105; 1084.735; 1084.8016; 1088.6603; 1115.2930; 1121.0424; 1134.8825; 1182.6381; 1198.3103; 1213.7511; 1213.9851;1215.5156; 1226.4269; 1229.7250; 1230.6099; 1237.2794; 1239.5632; 1242.3297; 1253.1417; 1253.5393; 187.3908; 1292.0212; 1293.3259; 1294.4944; 1296.7904; 1318.5690; 1370.3812; 1397.7069; 1431.0150; 144.5471; 1445.7679; 1457.7190; 1464.1983; 1501.0845; 1502.2495; 1506.3979; 1514.7676; 1515.0066; 1521.977; 1528.9644; 1529.7228; 1548.3586; 1612.7005; 1624.0090; 1652.0349; 1827.8776; 1896.9384; 3044.162; 3057.4606; 3063.6584; 3068.8870; 3095.7076; 3128.1689; 3132.0557; 3132.9087; 3134.4548; 3136.8849 3141.1455; 3143.5427; 3162.4264; 3168.0460; 3175.2925; 3179.4233; 3182.2068.

Cartesian Forces: Max 0.000000433 RMS 0.000000187

Item	Value	Threshold	Converged?
Maximum Force	0.000000	0.000450	YES
RMS Force	0.000000	0.000300	YES
Maximum Displacement	0.000162	0.001800	YES
RMS Displacement	0.000032	0.001200	YES

FILE: Yb_Sq2_PhYbOTf3_GS_Anion_B3LYP_M062x_Add12_Conf3-1_Y.out

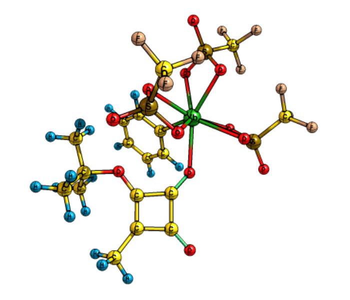

Conformer 12

E(RB3LYP/6-31G(d)/MWB59) = -3731.45749044
Convg = 0.2655D-08 -V/T = 2.0122

Temperature 195.150 Kelvin. Pressure 1.00000 Atm.
elec. and zero-point Energies= -3731.085585
elec. and thermal Energies= -3731.061913
elec. and thermal Enthalpies= -3731.061295
elec. and thermal Free Energies= -3731.131958

RB3LYP/6-31G(d)/MWB59/THF(IEFPCM) = -3731.52352489

RM06-2x/6-311+G(d,p)/MWB59/THF(IEFPCM) = -3731.36420569

Geometry at RB3LYP/6-31G(d)/MWB59
Charge = -1 Multiplicity = 1
Yb 0.0343521173 2.0998535661 0.7625584721
O -0.6935628143 3.3552632732 2.6331271748
S -2.1909431084 3.3881410974 2.3943011686
O -3.0354599953 2.9688941357 3.5076309228
O -2.4133590582 2.7663139321 1.051667934
C -2.5938577721 5.1984746203 2.1816193898
F -2.1999377993 5.8765028204 3.2672936571
F -2.001285528 5.7196646722 1.1067523217
F -3.9206289254 5.3350526534 2.0463510531
O -0.1503631312 4.1453237571 -0.5656042275
O 1.9776145458 4.9672257347 -1.6723839142
O 1.9440227793 3.6254991629 0.486434781
S 1.2805733337 4.6019581056 -0.4437952766
C 1.196254356 6.1885554448 0.5342844679
F 0.396693407 7.0631813662 -0.0896354731
F 0.7294498177 5.9763315512 1.7666346618
F 2.4241392123 6.7174727724 0.626535681
C -0.4385029135 -2.0456633111 1.3047700532
C -1.2875907107 -3.025401708 1.8166607195
C -2.1904113888 -2.0278206365 2.4401889344
C -1.2740958309 -0.9293862327 1.863080607
O -1.2281897148 0.2822638253 1.8487802823
O -3.1924869843 -2.0944499075 3.1131373464
O 0.6573157494 -1.875872324 0.6235404152
C 1.5902981916 -2.95188794 0.191443033
C -1.4626383036 -4.5019718467 1.8447649235
H -0.5423402559 -5.0429998908 2.0860118238
H -1.8202601599 -4.8766396249 0.8765577502
H -2.2223372928 -4.7470553843 2.5950103576
C 2.1380438102 -3.6433184178 1.4417369441
H 2.9028024569 -4.3705800565 1.146849291
H 2.5975752343 -2.9063912985 2.1080846823
H 1.3634945837 -4.1805771093 1.9963326412
C 2.6964192567 -2.1782167869 -0.5257496163
H 3.4637028063 -2.8819931754 -0.868633803
H 2.2900522423 -1.6442376004 -1.3872714308
H 3.1541237518 -1.4536826512 0.15257452
C 0.8627175815 -3.8837386998 -0.778832985
H 0.0798896385 -4.4705521234 -0.2912245464
H 0.4206700713 -3.2959348247 -1.588415793
H 1.5832275125 -4.5867763848 -1.2118172322
C 1.7362611221 1.0183579762 2.1078817428
C 1.5206092046 0.4708373922 3.3931136376
C 3.0631528992 0.9047168457 1.6301382535
H 0.532905767 0.5461914651 3.8499978979
H 3.317020708 1.3294308195 0.6593223495
C 2.5258728601 -0.1622326791 4.1356317699
C 4.0836902641 0.2738611179 2.352678361
H 2.3053137607 -0.5608984498 5.1259236404
H 5.0902762456 0.2148703022 1.9384289366
C 3.8164480501 -0.2720485737 3.6119129896
H 4.6061599516 -0.7577957696 4.1833437978
O 0.245621051 -0.7702920431 -2.4490067865
S -0.0131354038 0.5683207188 -1.9121735103
C -0.5524261803 1.5519845834 -3.4018295811
O 1.1511321004 1.3172111133 -1.3306634466
O -1.1394539586 0.7040603502 -0.9249985491
F -0.9249406127 2.7816343325 -3.0596649852
F -1.5857802525 0.9290003285 -3.9860036273
F 0.4586745308 1.6222580419 -4.2754704342

Frequency: 13.6184; 17.9428; 19.2147; 23.5847; 27.5036; 29.7213; 36.1106; 37.2424; 39.7873; 44.8570; 48.9128; 51.3766; 53.8305; 59.1754; 65.0641; 68.5133; 72.7919; 75.1706; 80.3491; 80.7226; 90.5830; 94.9792; 101.3903; 112.3093; 127.5408; 128.4036; 131.7605; 136.5184; 146.0033; 146.9792; 152.4837; 166.3063; 173.3225; 181.7957; 183.9958; 202.9572; 204.6819; 208.3675; 214.8437; 216.1260; 221.4423; 223.6006; 230.8360; 236.6934; 249.5333; 253.8722; 276.6862; 297.6972; 303.3462; 307.5399; 308.9833; 321.7091; 323.7115; 326.7221; 340.1154; 350.5769; 352.0408; 357.9596; 359.0891; 395.4597; 395.9852; 451.6377; 453.0601; 481.5404; 486.4600; 489.8880; 490.0888; 491.1886; 523.3355; 523.9376; 529.3825; 558.0755; 558.4263; 558.9086; 569.9422; 571.9483; 575.3271; 602.2907; 611.8855; 620.9144; 624.2496; 634.3820; 635.4416; 646.0296; 647.6057; 719.7883; 735.2298; 760.3052; 761.0609; 762.6098; 763.6061; 784.6593; 790.1848; 838.1852; 869.6397; 908.3646; 937.9234; 941.5842; 969.9708; 974.1048; 976.9675; 980.1785; 982.0511; 986.6022; 990.5707; 998.5267; 1011.3072; 1031.4912; 1053.5473; 1067.1275; 1073.9068; 1083.5073; 1083.8331; 1102.3773; 1111.3079; 1118.8445; 1135.6985; 1182.1123; 1199.7728; 1208.9802; 1210.0596; 1211.9466; 1214.3348; 1224.6996; 1226.0854; 1228.6814; 1234.7548; 1246.9056; 1252.1431; 1258.0397; 1284.0585; 1291.0173; 1295.3446; 1296.9452; 1302.6500; 1312.9990; 1369.1589; 1392.1011; 1431.0777; 1436.6496; 1441.4835; 1457.5567; 1462.7726; 1501.6008; 1506.6826; 1515.4309; 1520.5450; 1521.9181; 1523.6058; 1532.1764; 1533.6191; 1556.1072; 1611.4886; 1622.2861; 1643.7689; 1838.7815; 1885.8642; 3043.6838; 3061.1849; 3063.6498; 3071.4157; 3102.8061; 3128.2507; 3130.1874; 3131.7218; 3135.6462; 3138.8435; 3143.5050; 3148.7385; 3150.7712; 3160.4946; 3161.4934; 3173.5457; 3174.0762.

Cartesian Forces: Max 0.000010530 RMS 0.000001592

Item	Value	Threshold	Converged?
Maximum Force	0.000007	0.000450	YES
RMS Force	0.000001	0.000300	YES
Maximum Displacement	0.001034	0.001800	YES
RMS Displacement	0.000276	0.001200	YES

FILE: Yb_Sq2_PhYbOTf3_GS_Anion_B3LYP_M062x_Add12_Conf3-1-.out

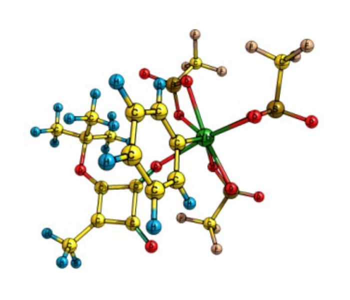

Conformer 13

E(RB3LYP/6-31G(d)/MWB59) = -3731.45746883
Convg = 0.7788D-09 -V/T = 2.0122

Temperature 195.150 Kelvin. Pressure 1.00000 Atm.
elec. and zero-point Energies= -3731.086265
elec. and thermal Energies= -3731.062283
elec. and thermal Enthalpies= -3731.061665
elec. and thermal Free Energies= -3731.133400

RB3LYP/6-31G(d)/MWB59/THF(IEFPCM) = -3731.52370463

RM06-2x/6-311+G(d,p)/MWB59/THF(IEFPCM) = -3731.36145999

Geometry at RB3LYP/6-31G(d)/MWB59
Charge = -1 Multiplicity = 1
Yb 0.3591414603 2.5555496087 1.1062125971
O 2.3751929557 2.9600464375 -0.270435302
S 3.3859698647 2.3407033742 0.6529219362
O 4.3355080631 1.392130461 0.066894696
O 2.624471357 1.8877660339 1.8661818827
C 4.45616145 3.7515425285 1.2378504892
F 5.070477255 4.3062030642 0.1854955896
F 3.7383426121 4.6802338223 1.864258823
F 5.3844988161 3.2768125665 2.0793559197
O 0.7928062247 4.7727081855 2.060040223
O -0.9631613676 6.5438262576 1.5842934325
O -0.4878252804 4.581326282 0.0425890133
S -0.0059474865 5.5753787541 1.064561174
C 1.2556098594 6.6179636199 0.1681227116
F 1.8323740115 7.462303321 1.0340798109
F 2.2054953968 5.8685302113 -0.39677193
F 0.6456123185 7.3310113172 -0.7875326256
C 0.2865267601 -2.3711785769 0.7336523509
C -0.9790650978 -2.9206234965 0.849056398
C -1.6008932857 -1.6453020143 1.2617440319
C -0.1925072292 -1.0020351469 1.1518434177
O 0.3201017623 0.0880186777 1.3100679476
O -2.713334536 -1.2770201097 1.5520982293
O 1.395228203 -2.9754710939 0.383112871
C 2.6818043636 -2.2461577753 0.1388213908
C -1.5306647811 -4.2774080179 0.62005779
H -0.7660929537 -4.9649945071 0.2469090012
H -2.3555420931 -4.2338565312 -0.1021538202
H -1.9489034042 -4.6845289539 1.549899373
C 2.4730478267 -1.2574876326 -1.0058155683
H 3.4080806807 -0.7263396822 -1.2044031297
H 2.1593861763 -1.7824879748 -1.9147638678
H 1.7226600782 -0.5004039662 -0.7678731107
C 3.6155352335 -3.3845306458 -0.2737597803
H 4.6061059695 -2.9787314602 -0.5020551512
H 3.7180299068 -4.1175019182 0.5335663767
H 3.2359558434 -3.8958618972 -1.1644741958
C 3.1481137681 -1.5940302151 1.4385834793
H 2.4866044861 -0.7868478045 1.7561458396
H 3.222129419 -2.343003089 2.2354553326
H 4.134839693 -1.14821135 1.281915709
C -0.5712383444 1.6533885488 -0.9406088564
C 0.0956955106 1.6574208006 -2.1865321019
C -1.87779151 1.1110153818 -0.9605510924
H 1.1005058686 2.0739999726 -2.2527010153
H -2.4631255781 1.0798195243 -0.0408183796
C -0.4811279931 1.1503545225 -3.3583654204
C -2.472951378 0.6042481912 -2.1220083494
H 0.0764194126 1.1739557604 -4.2949008161
H -3.4857320055 0.2034179927 -2.0847675481
C -1.7717032644 0.6182689751 -3.3305301183
H -2.2271829065 0.2250128762 -4.2383524771
O -1.7243277503 3.4466912345 4.6273165191
S -1.2322414214 2.4376474923 3.697826213
C -1.9663869773 0.8386209741 4.3347185717
O 0.24079416 2.1795680547 3.6239857511
O -1.7122187791 2.5425993924 2.2635039691
F -1.2899514589 -0.2233981923 3.8730244689
F -3.245354551 0.7363567007 3.9745957512
F -1.8882998508 0.825106318 5.6731216412

Frequency: 17.4810; 18.8890; 20.5376; 25.5672; 28.0282; 31.1040; 32.4174; 33.0580; 37.3214; 40.4823; 42.9809; 44.7974; 48.3157; 54.4988; 56.8121; 58.3283; 60.0476; 69.2463; 78.2361; 82.5570; 86.5125; 87.8650; 89.1478; 99.4386; 106.6753; 126.9137; 128.7017; 134.4449; 135.2514; 144.2681; 149.9979; 162.2445; 172.1497; 184.9591; 188.4655; 191.1083; 207.2842; 208.1696; 210.8729; 211.0958; 219.8211; 219.8666; 224.0384; 228.8634; 259.0599; 267.2111; 272.8765; 292.1691; 302.4165; 308.0093; 309.7349; 321.1198; 322.3211; 324.2340; 327.8537; 350.2267; 350.4825; 352.0554; 358.5200; 394.4432; 394.7408; 455.4160; 458.1593; 472.2162; 488.8920; 489.8136; 491.3932; 492.5565; 523.5797; 524.2515; 530.0646; 556.6505; 557.3113; 558.7743; 568.3360; 571.5922; 576.5958; 592.3841; 606.3380; 619.7155; 625.3614; 633.8466; 636.7688; 641.0134; 646.8843; 720.9195; 731.6623; 736.6600; 759.2456; 760.4856; 763.1301; 778.1284; 787.2203; 847.2797; 870.0323; 910.7569; 937.0505; 942.2942; 959.5909; 970.1109; 973.3622; 977.4399; 980.6254; 983.9884; 985.8370; 988.2816; 1010.2009; 1030.8227; 1056.8822; 1063.5735; 1066.5674; 1082.3225; 1082.8564; 1085.2194; 1105.1329; 1117.1915; 1136.0794; 1182.6872; 1199.4642; 1210.3409; 1210.7050; 1213.8860; 1220.8662; 1224.8719; 1229.4569; 1231.0091; 1246.5419; 1246.8733; 1249.3484; 1256.8818; 1284.9304; 1291.9740; 1292.9949; 1299.1350; 1305.2897; 1318.2292; 1370.0645; 1396.5556; 1431.2661; 1441.7968; 1445.3091; 1458.0111; 1464.4673; 1500.2763; 1503.2532; 1506.6504; 1514.2401; 1518.1827; 1520.9462; 1529.8814; 1532.0653; 1551.5398; 1613.1549; 1623.6375; 1649.2177; 1815.8516; 1884.1180; 3043.6708; 3057.6795; 3064.6014; 3071.1460; 3095.1925; 3129.1341; 3133.8410; 3134.9867; 3136.6684; 3139.6820; 3140.7559; 3146.7005; 3153.6667; 3160.8981; 3165.5023; 3173.5177; 3180.9633.

Cartesian Forces: Max 0.000001346 RMS 0.000000503

Item	Value	Threshold	Converged?
Maximum Force	0.000000	0.000450	YES
RMS Force	0.000000	0.000300	YES
Maximum Displacement	0.000080	0.001800	YES
RMS Displacement	0.000016	0.001200	YES

FILE: Yb_Sq2_PhYbOTf3_GS_Anion_B3LYP_M062x_Add12_Conf4.out

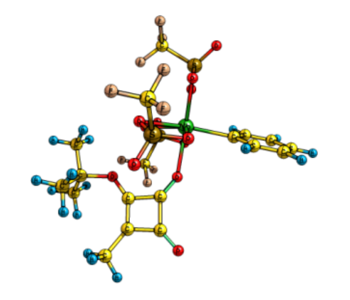

Conformer 14

E(RB3LYP/6-31G(d)/MWB59) = -3731.45825266
Convg = 0.6962D-09 -V/T = 2.0122

Temperature 195.150 Kelvin. Pressure 1.00000 Atm.
elec. and zero-point Energies= -3731.086814
elec. and thermal Energies= -3731.062795
elec. and thermal Enthalpies= -3731.062177
elec. and thermal Free Energies= -3731.134251

RB3LYP/6-31G(d)/MWB59/THF(IEFPCM) = -3731.52541666

RM06-2x/6-311+G(d,p)/MWB59/THF(IEFPCM) = -3731.36339142

Geometry at RB3LYP/6-31G(d)/MWB59
Charge = -1 Multiplicity = 1
Yb -0.0601178694 1.8598413416 0.2013324124
O 1.0608253127 0.9734675785 -1.8311411684
S 2.1898173921 0.228616192 -1.1807770842
O 2.3106098022 -1.1991396327 -1.5084023701
O 2.1762910003 0.5754953033 0.2752921997
C 3.7433856149 0.9871580402 -1.8742516216
F 3.801890205 0.7576774948 -3.1924651576
F 3.7876785658 2.2984041441 -1.6585997358
F 4.8082496518 0.4138146434 -1.2907419327
O 0.006153195 3.9042188804 1.5096037319
O 1.0286939739 5.9570223918 0.4151575656
O 1.5647919225 3.5747825267 -0.2852859726
S 1.1541118005 4.5539407061 0.7844772777
C 2.5583909081 4.4915804861 2.0073603919
F 2.2653228309 5.2397662672 3.0766078475
F 2.780190548 3.2345117437 2.4042392254
F 3.6742464687 4.9676753159 1.4390238913
C 0.0901746559 -2.4663079438 0.5311781818
C -0.359399228 -3.60282158 -0.1335341077
C -1.3268611301 -2.8302736213 -0.9555440862
C -0.8021017085 -1.5607105128 -0.2505924052
O -0.9664368951 -0.3575253488 -0.2941750036
O -2.1509707473 -3.1154703645 -1.7915468482
O 0.8893785744 -2.039758193 1.4621418402
C 1.8684757401 -2.8322549711 2.236477323
C -0.1059966783 -5.065640651 -0.1928630583
H 0.8099639455 -5.281094084 -0.7588561856
H -0.9382684381 -5.546618768 -0.7180400174
H 0.0003711841 -5.5235877194 0.7958855309
C 1.0976311926 -3.7580022414 3.1804373015
H 1.797837296 -4.266901271 3.8522020861
H 0.3957499279 -3.1756501077 3.7842628238
H 0.5373523562 -4.5252618022 2.6367311376
C 2.6112139173 -1.7430735715 3.0129139617
H 3.3649979027 -2.1994885555 3.6641402162
H 3.0965508097 -1.050759272 2.3204920956
H 1.9088430655 -1.1665821575 3.6205111258
C 2.8047458474 -3.5694006393 1.277223793
H 2.3123341569 -4.4102493036 0.7806899798
H 3.1771388047 -2.8813962291 0.5125769184
H 3.6565147926 -3.9686937333 1.8390059803
C -1.69736078 2.7636117844 -1.2685008329
C -1.6423729707 2.613542031 -2.6732395509
C -2.8040889931 3.4965354098 -0.781193689
H -0.8168229697 2.0596034725 -3.1198482674
H -2.9130258747 3.6503614542 0.2924638963
C -2.6114532478 3.1478089335 -3.5297440796
C -3.7820557026 4.0392921388 -1.6222127268
H -2.5274438124 3.0046789247 -4.6069532966
H -4.6168398736 4.5983870682 -1.2000445919
C -3.6885602103 3.8653110693 -3.0047813745
H -4.4461155487 4.2845448373 -3.6655979874
O -1.266222864 2.0583463922 4.2630952326
S -1.0970920792 1.2684744471 3.048249006
C -1.7273725862 -0.4178305578 3.547819976
O 0.2986231522 1.0084728483 2.563068092
O -1.9127380019 1.637456559 1.8418852704
F -1.8430127463 -1.2506149374 2.4998409052
F -2.9257997867 -0.3121705801 4.1294540462
F -0.8732166904 -0.9767251912 4.4279511346

Frequency: 13.6027; 16.9769; 19.0478; 23.7846; 25.4293; 27.7329; 31.6817; 34.1153; 34.5646; 39.6764; 45.0227; 47.8986; 49.8395; 55.6639; 57.6909; 59.0395; 65.2878; 73.2401; 75.5838; 79.8838; 84.9756; 92.4102; 96.8548; 99.8556; 112.9177; 120.8711; 130.1296; 133.3199; 135.6999; 137.4684; 143.3666; 158.1323; 171.8123; 177.2331; 190.4889; 202.9911; 203.7401; 210.4856; 213.2557; 215.1495; 218.1001; 220.1222; 220.3664; 229.9874; 242.5700; 252.8443; 265.5114; 297.2910; 303.5116; 304.8012; 308.7493; 320.6701; 325.3832; 326.0811; 337.7747; 346.6901; 348.8462; 349.7532; 357.2956; 393.5810; 398.0044; 448.0428; 453.8509; 481.4218; 487.0136; 488.3243; 490.9750; 491.9774; 519.4029; 521.5449; 528.7280; 555.5396; 557.9836; 559.2026; 567.1046; 567.6060; 576.3851; 597.4992; 613.3453; 622.0012; 626.2390; 631.4892; 634.1534; 646.5080; 652.0401; 716.2097; 737.9035; 754.3473; 760.8346; 763.0598; 766.5160; 781.8546; 792.3410; 844.8167; 867.8713; 913.9029; 937.0806; 941.9232; 973.9254; 976.0012; 977.8170; 981.8600; 985.3303; 988.5812; 989.7534; 1001.4166; 1011.8884; 1036.8459; 1052.7944; 1064.2015; 1075.0453; 1086.1352; 1086.8456; 1114.7726; 1118.8227; 1129.4409; 1147.9588; 1183.6744; 1193.7589; 1199.4212; 1211.2208; 1213.1763; 1215.5903; 1218.4572; 1225.3870; 1229.3075; 1233.9583; 1235.6223; 1240.8869; 1253.5265; 1274.2159; 1286.4982; 1296.3868; 1296.9268; 1307.1564; 1318.5777; 1370.9003; 1395.7408; 1431.8989; 1437.6318; 1442.2660; 1459.0198; 1462.8181; 1499.7448; 1505.7602; 1513.4610; 1517.8879; 1521.1090; 1523.1238; 1529.0215; 1531.2080; 1550.0890; 1615.2493; 1627.9866; 1654.2771; 1833.5774; 1888.9546; 3044.0666; 3062.6166; 3064.2280; 3071.8883; 3103.1009; 3127.0863; 3130.5146; 3134.9454; 3135.5687; 3142.4749; 3145.4492; 3151.3343; 3155.2507; 3156.1982; 3161.4569; 3165.6682; 3174.2671.

Cartesian Forces: Max 0.000000798 RMS 0.000000317

Item	Value	Threshold	Converged?
Maximum Force	0.000000	0.000450	YES
RMS Force	0.000000	0.000300	YES
Maximum Displacement	0.000063	0.001800	YES
RMS Displacement	0.000009	0.001200	YES

FILE: Yb_Sq2_PhYbOTf3_GS_Anion_B3LYP_M062x_Add12_Conf4-_Y.out

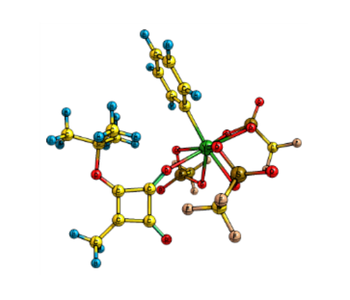

Conformer 15

E(RB3LYP/6-31G(d)/MWB59) = -3731.46305053
Convg = 0.2764D-08 -V/T = 2.0122

Temperature 195.150 Kelvin. Pressure 1.00000 Atm.
elec. and zero-point Energies= -3731.091858
elec. and thermal Energies= -3731.067755
elec. and thermal Enthalpies= -3731.067137
elec. and thermal Free Energies= -3731.139838

RB3LYP/6-31G(d)/MWB59/THF(IEFPCM) = -3731.52928062

RM06-2x/6-311+G(d,p)/MWB59/THF(IEFPCM) = -3731.36948681

Geometry at RB3LYP/6-31G(d)/MWB59
Charge = -1 Multiplicity = 1
Yb 0.4060742187 2.5363115085 0.3863098272
O 0.9128075997 1.8696902971 2.7437933721
S -0.5079118089 1.7800229102 3.2283740128
O -0.9081089461 0.4959418235 3.8136010063
O -1.3773259175 2.3364987985 2.1423856132
C -0.6259316444 2.99467984 4.6356046859
F 0.2128896614 2.6234300054 5.6134790895
F -0.3124615243 4.2267847525 4.2411658392
F -1.8756829301 2.9959404702 5.1182020695
O 0.552222819 4.6136189217 -0.8717042469
O 1.7688309888 6.5569821137 0.2244205969
O 0.7468466098 4.6263550588 1.5197091281
S 0.7427928827 5.5266304055 0.3084163863
C -0.873737892 6.4422600208 0.4478311669
F -1.069203941 7.1900235065 -0.6433286249
F -1.8866657455 5.5804475254 0.5742237681
F -0.8483609101 7.2419076437 1.5220759162
C -0.0341155092 -2.2605657324 1.3872024092
C -1.3349810251 -2.6896013774 1.5919896121
C -1.9295769744 -1.399506998 1.1757438604
C -0.4833972555 -0.8910784925 0.9561912515
O 0.0715919153 0.1295945492 0.5973731235
O -3.0432969973 -0.9579414398 1.0406798457
O 1.0748459902 -2.9393323773 1.5472246954
C 2.4427789898 -2.3426659995 1.3907466377
C -1.9314484138 -3.9607241382 2.0653903029
H -1.175251958 -4.741866017 2.1860172492
H -2.7005915406 -4.307483348 1.3641660614
H -2.4326175248 -3.8043539101 3.0296941314
C 2.6200207568 -1.8354245428 -0.0384602949
H 3.6537716354 -1.5039206397 -0.1790137013
H 2.4086263419 -2.6338892851 -0.7591758375
H 1.9867625087 -0.9725760028 -0.2481709747
C 3.3396818338 -3.5498928575 1.6667619992
H 4.389418924 -3.2477987347 1.597527711
H 3.1582320507 -3.9481086546 2.6705215024
H 3.1587282479 -4.3459822896 0.9366409868
C 2.6282296819 -1.2556219887 2.445643006
H 1.9539240281 -0.4107790995 2.2965814932
H 2.4729395029 -1.6648983212 3.4501019914
H 3.6462464764 -0.8589309697 2.3797029011
C 2.7503526018 2.0598365505 0.0890510572
C 3.6651977725 2.0764189188 1.1683690482
C 3.3120224537 1.7216693959 -1.1646091875
H 3.3084716472 2.3314163803 2.1658605233
H 2.6718636549 1.6999298656 -2.0454559249
C 5.0236122381 1.76875755 1.021574469
C 4.6663803496 1.4067922051 -1.3320966644
H 5.6883079156 1.8024793425 1.885059781
H 5.0495380373 1.1525596955 -2.3204236191
C 5.5306681255 1.4250540005 -0.2345007897
H 6.5861870201 1.1861282887 -0.3578248684
O -1.494255663 3.0219145491 -3.340885328
S -1.1903433828 2.1720835512 -2.1951920261
C -2.0145129414 0.5523944682 -2.6168703455
O -1.7504245312 2.560253127 -0.8573966071
O 0.2508372849 1.8020357758 -1.9557576937
F -1.7225629091 -0.4028164001 -1.7210478726
F -1.5924834741 0.1368912421 -3.8198491563
F -3.3403235748 0.7119769418 -2.6562387971

Frequency: 11.7930; 16.3930; 18.2306; 20.4679; 27.2362; 27.6472; 29.0824; 33.4791; 36.1153; 38.3225; 42.9125; 43.8476; 48.0150; 52.0361; 58.3978; 59.8257; 64.5191; 66.9380; 72.7296; 75.4798; 76.4021; 82.1317; 88.4508; 94.0324; 111.6054; 125.0539; 133.0173; 138.1540; 139.8865; 144.7099; 146.2300; 167.8404; 178.2942; 179.0475; 186.2666; 188.1303; 204.4293; 209.5585; 210.8955; 212.8819; 214.8566; 218.4007; 218.9360; 227.9316; 252.3417; 267.4363; 270.0109; 291.2905; 304.2246; 306.0178; 308.8331; 320.5140; 324.3269; 325.6660; 329.4711; 347.8082; 348.4254; 350.7734; 357.9740; 394.2370; 396.1586; 449.7539; 452.5822; 477.2131; 487.9558; 490.9958; 491.2260; 491.7757; 520.6265; 523.6465; 529.3522; 556.0856; 558.0670; 558.6300; 568.2074; 569.5242; 577.3647; 594.1539; 602.1346; 622.3651; 626.8904; 632.9578; 634.3822; 645.1816; 648.3521; 719.7130; 731.9857; 739.1315; 759.4065; 761.3758; 763.2862; 782.3355; 787.5183; 845.6606; 871.0444; 916.2947; 936.9913; 940.8077; 959.9485; 976.3894; 978.8605; 979.8608; 984.7788; 986.7755; 987.9018; 989.0767; 1011.4479; 1033.0907; 1055.6748; 1063.2350; 1067.5793; 1084.3442; 1085.1256; 1086.5139; 1113.3607; 1118.9741; 1137.6539; 1182.5364; 1197.5020; 1213.5965; 1214.5461; 1216.4528; 1220.5258; 1226.7172; 1231.3906; 1236.5044; 1240.4171; 1243.6246; 1245.9097; 1253.8344; 1280.2477; 1292.4439; 1294.6692; 1297.9543; 1306.9204; 1318.1723; 1371.2318; 1397.6243; 1431.2303; 1441.9654; 1445.8245; 1457.6428; 1464.9442; 1500.7826; 1502.6795; 1506.2817; 1513.7227; 1515.7325; 1521.4922; 1528.5329; 1529.8531; 1548.0660; 1612.2990; 1623.7926; 1649.6724; 1821.9206; 1894.1846; 3044.2981; 3057.1591; 3062.5481; 3068.5360; 3096.5226; 3127.8550; 3131.5687; 3133.1747; 3133.9110; 3136.9257; 3141.1624; 3143.2291; 3160.1297; 3167.5580; 3175.3056; 3177.3183; 3182.3187.

Cartesian Forces: Max 0.000016807 RMS 0.000003666

Item	Value	Threshold	Converged?
Maximum Force	0.000014	0.000450	YES
RMS Force	0.000002	0.000300	YES
Maximum Displacement	0.001648	0.001800	YES
RMS Displacement	0.000379	0.001200	YES

FILE: Yb_Sq2_PhYbOTf3_GS_Anion_B3LYP_M062x_Add12_Conf4-1.out

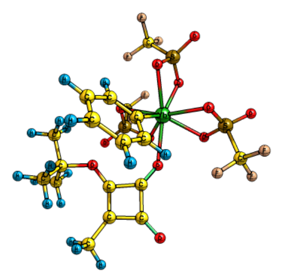

Conformer 16

E(RB3LYP/6-31G(d)/MWB59) = -3731.45703354
Convg = 0.2875D-08 -V/T = 2.0122

Temperature 195.150 Kelvin. Pressure 1.00000 Atm.
elec. and zero-point Energies= -3731.085304
elec. and thermal Energies= -3731.061557
elec. and thermal Enthalpies= -3731.060939
elec. and thermal Free Energies= -3731.131738

RB3LYP/6-31G(d)/MWB59/THF(IEFPCM) = -3731.52352741

RM06-2x/6-311+G(d,p)/MWB59/THF(IEFPCM) = -3731.36297080

Geometry at RB3LYP/6-31G(d)/MWB59
Charge = -1 Multiplicity = 1
Yb -0.1615984079 2.0748511155 0.2183399743
O 0.6596470931 1.6819609472 2.4760822837
S -0.6344661565 1.3647826999 3.1807797968
O -0.6446921978 0.1549971414 4.008538114
O -1.7262747033 1.5283290845 2.1723396237
C -0.8760672734 2.7465873159 4.4111629621
F 0.1832598258 2.805148537 5.2292894003
F -1.0249694007 3.9224246859 3.8051633132
F -1.9707897873 2.4940139354 5.1413959396
O -0.4826518641 4.3984062026 0.9194295244
O 1.0329389865 6.0990322298 -0.1964746495
O 1.6981098129 3.6820877692 0.2160310512
S 0.907960562 4.8965157251 0.617920968
C 1.6103503805 5.4001586872 2.2728968684
F 0.8295729476 6.3366238033 2.8270504569
F 1.7006016977 4.3641981359 3.1123711519
F 2.8366868315 5.9107765459 2.0957251463
C -0.2231942594 -2.1538482573 0.9143107578
C -0.8217034224 -3.330834193 0.4669510469
C -1.7816581415 -2.6033303176 -0.3987592244
C -1.1204879082 -1.2940303054 0.0775153129
O -1.235782324 -0.1031283494 -0.122507233
O -2.6798893001 -2.9332617911 -1.1383438633
O 0.7040895509 -1.6868471188 1.6952024292
C 1.5884395333 -2.4789085762 2.5859737387
C -0.7083722322 -4.8059608529 0.6138329681
H -0.5810491128 -5.1274423119 1.6522607999
H 0.1430377597 -5.196078804 0.0403441343
H -1.6157934726 -5.2678257449 0.2097154892
C 0.7225014044 -3.1292012883 3.6664608628
H 1.3665595655 -3.6224123712 4.4032195816
H 0.1299207556 -2.3618129265 4.1724588898
H 0.0452227849 -3.8845426208 3.2562069851
C 2.4986042516 -1.4074255056 3.1867604629
H 3.2016622543 -1.875780487 3.8849689101
H 3.0592073346 -0.9020317483 2.3962723981
H 1.9039115253 -0.6623123146 3.7193202201
C 2.3952348432 -3.4698881459 1.7450876418
H 1.7754647677 -4.2679620888 1.3282176943
H 2.8986384078 -2.9458571281 0.9261978333
H 3.1577804709 -3.9392151397 2.3768277853
C 1.6363132325 0.676188856 -0.6282295773
C 2.9058836295 0.7014645251 -0.005711697
C 1.5314358579 -0.2143270482 -1.7209220904
H 3.075874521 1.3819951658 0.8286959781
H 0.5960228053 -0.2711063391 -2.2800582191
C 3.9737219616 -0.1083819638 -0.4123118382
C 2.5847916919 -1.0340311525 -2.146837905
H 4.934005922 -0.0454233079 0.1000789155
H 2.4497182848 -1.6987917981 -3.0001667262
C 3.8145798208 -0.9906840275 -1.4856835984
H 4.6418962744 -1.6184646062 -1.8136059734
O -2.5413546288 4.2309022721 -2.5532083965
S -1.9421724091 3.0025714214 -2.0482425864
C -2.4294319281 1.6957721406 -3.2856626858
O -2.4118801443 2.4745585989 -0.7232529174
O -0.4343429048 2.9156052511 -2.0150908893
F -1.8760522014 0.5148806552 -2.9824983295
F -2.0215839323 2.0575032652 -4.5083661189
F -3.760117808 1.5570260024 -3.2969041434

Frequency: 14.3801; 19.1752; 20.6019; 26.5584; 27.6827; 31.5298; 34.2511; 36.3876; 39.0208; 40.8811; 47.4075; 51.1266; 53.9864; 61.1145; 63.7879; 65.8358; 67.9076; 70.9880; 76.9426; 81.4226; 88.4945; 94.0163; 97.3663; 107.3461; 123.8557; 130.5937; 135.0908; 138.9253; 143.1997; 147.8250; 153.4673; 164.1031; 174.8115; 179.2894; 185.3567; 205.3307; 208.2892; 209.0697; 211.6162; 215.0913; 218.1749; 222.2902; 224.7799; 232.3647; 247.2128; 253.1926; 273.6783; 296.9498; 301.8963; 305.6427; 308.4068; 321.8409; 321.9254; 325.6713; 338.4132; 349.6964; 351.6635; 354.7170; 357.9889; 394.7295; 395.2714; 451.5872; 455.4459; 481.6766; 485.5363; 489.6863; 490.4768; 491.1193; 522.9193; 524.1821; 530.0814; 556.9413; 557.9297; 558.8442; 569.5273; 570.9379; 577.3693; 602.1156; 611.3036; 619.2840; 624.5236; 634.6528; 636.5368; 645.0513; 647.1984; 720.2699; 735.4337; 759.6021; 759.9571; 761.9258; 765.1530; 783.3780; 789.0342; 841.2935; 869.4829; 907.6997; 937.8121; 941.6176; 973.9399; 976.4164; 978.0149; 978.6366; 982.2916; 987.9592; 989.5556; 999.5592; 1011.4674; 1031.1217; 1053.1736; 1066.8425; 1074.4335; 1083.1847; 1083.9649; 1108.6866; 1109.5690; 1119.1335; 1140.0331; 1181.9039; 1199.7510; 1211.0096; 1211.7184; 1214.2452; 1215.9094; 1224.3120; 1224.7856; 1229.2131; 1231.3212; 1238.5781; 1245.3182; 1254.5686; 1282.8470; 1290.6986; 1295.3922; 1296.9846; 1306.8449; 1318.8706; 1370.1093; 1394.9280; 1431.0106; 1435.7733; 1442.0739; 1457.6063; 1461.4783; 1500.8170; 1506.3737; 1514.4253; 1520.4808; 1521.1892; 1522.9401; 1530.1038; 1532.5776; 1553.2421; 1611.2972; 1622.0302; 1647.1123; 1837.7315; 1885.0417; 3043.0605; 3061.8706; 3062.4471; 3070.6926; 3102.2867; 3127.2589; 3128.9733; 3130.2325; 3133.4687; 3135.7212; 3144.7434; 3149.1384; 3153.6914; 3155.2841; 3156.7066; 3172.6595; 3173.1551.

Cartesian Forces: Max 0.000014865 RMS 0.000002347

Item	Value	Threshold	Converged?
Maximum Force	0.000007	0.000450	YES
RMS Force	0.000001	0.000300	YES
Maximum Displacement	0.001592	0.001800	YES
RMS Displacement	0.000230	0.001200	YES

FILE: Yb_Sq2_PhYbOTf3_GS_Anion_B3LYP_M062x_Add12_Conf4-1-.out

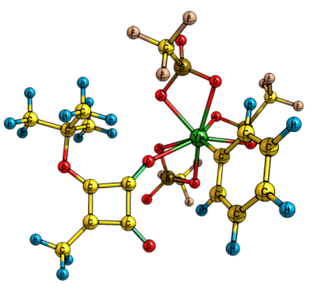

Conformer 17

E(RB3LYP/6-31G(d)/MWB59) = -3731.46010496
Convg = 0.1002D-08 -V/T = 2.0122

Temperature 195.150 Kelvin. Pressure 1.00000 Atm.
elec. and zero-point Energies= -3731.088845
elec. and thermal Energies= -3731.064748
elec. and thermal Enthalpies= -3731.064130
elec. and thermal Free Energies= -3731.136529

RB3LYP/6-31G(d)/MWB59/THF(IEFPCM) = -3731.52662334

RM06-2x/6-311+G(d,p)/MWB59/THF(IEFPCM) = -3731.36310057

Geometry at RB3LYP/6-31G(d)/MWB59
Charge = -1 Multiplicity = 1
Yb 0.3692019577 2.5273873767 0.4905269951
O 2.6497101845 1.6764486413 0.5701766337
S 3.2951389064 2.6573204056 -0.3764567131
O 4.5391835794 3.2872410308 0.0485855043
O 2.1913249352 3.5330163765 -0.8867416435
C 3.7761758998 1.6073057933 -1.8391137926
F 4.7632282287 0.7648176194 -1.4856607143
F 2.7394432453 0.8825426 -2.2747398529
F 4.2131521711 2.3823237076 -2.8364134383
O 1.0767520578 4.4690415185 1.8690217106
O -0.7893387819 6.1080143773 2.3798949948
O -0.7233375431 4.6682510239 0.2905328345
S 0.0107780982 5.4114126241 1.3781522657
C 0.9180856799 6.7791735183 0.4908283172
F 1.748121589 7.387210542 1.349387805
F 1.6225644066 6.3204642176 -0.5414918044
F 0.0292607599 7.6769717779 0.042720139
C 0.1480845313 -2.4221547819 1.0199950968
C -1.1429824005 -2.9145903139 1.0876186266
C -1.7601468521 -1.6121732173 0.752984486
C -0.3244575772 -1.0276016721 0.7000667559
O 0.2137139703 0.0445342008 0.5088484642
O -2.8810156288 -1.2055851995 0.5688797914
O 1.27234856 -3.0738972516 1.2013479093
C 2.6173959792 -2.4205429615 1.154008495
C -1.7205177857 -4.2443633345 1.3952464926
H -0.9465578995 -5.0138234837 1.4704854905
H -2.44513636 -4.5342993397 0.6244855101
H -2.2697847154 -4.2056945369 2.3452172855
C 2.8324994785 -1.8137202656 -0.2307119518
H 3.8537109063 -1.4281743355 -0.3060584807
H 2.6919852423 -2.5723556972 -1.0091527608
H 2.1609351103 -0.9745880024 -0.4130928937
C 3.5503400068 -3.6090263885 1.3903784072
H 4.5898728161 -3.2661236427 1.3854117161
H 3.3444016931 -4.0775430992 2.3583269921
H 3.429474545 -4.3624987134 0.6047921943
C 2.7167200538 -1.3996997408 2.2858719722
H 2.0353176291 -0.5580602529 2.1533038735
H 2.5128072251 -1.8782042521 3.2500744955
H 3.731266367 -0.9882951516 2.3125385336
C -0.6762602238 2.1074180764 -1.6285244527
C -0.2332812816 2.6769290903 -2.8450057403
C -1.858586801 1.3361299006 -1.7233593771
H 0.6632827988 3.295682158 -2.8494770929
H -2.2775207761 0.8693887298 -0.8318944642
C -0.9015925644 2.4884612181 -4.0600832794
C -2.5437287422 1.1404806814 -2.9284600838
H -0.5177623044 2.9481567912 -4.9708807345
H -3.453142855 0.5403755873 -2.9453141733
C -2.06354486 1.715049322 -4.1070675819
H -2.5897985568 1.5658397813 -5.0489723503
O -1.3653148032 0.1178990352 3.4769883888
S -0.8023710236 1.4254929583 3.1266278453
C -1.1650412606 2.5125996419 4.5943411723
O -1.4549919792 2.1434100537 1.9657253628
O 0.6746083245 1.4932784138 2.915743265
F -0.6976386376 3.7426751083 4.4057581721
F -0.5841128339 1.9847564478 5.6823514129
F -2.4865267016 2.5677234238 4.796135129

Frequency: 12.8321; 19.7153; 20.9003; 24.3817; 26.7313; 27.6830; 29.4741; 30.3300; 39.5295; 43.3012; 45.5368; 46.2167; 49.7609; 51.2939; 55.0549; 58.1723; 65.6705; 65.7865; 71.6301; 72.8435; 76.5378; 81.1221; 87.3256; 92.7048; 97.3152; 122.5679; 130.8493; 136.6337; 137.3157; 146.1209; 150.3024; 162.9926; 171.6101; 177.0155; 187.5544; 189.2162; 205.0254; 207.8647; 212.0804; 214.1461; 215.0650; 219.1969; 223.7817; 231.8668; 257.5230; 266.0384; 273.2544; 292.8048; 303.7958; 307.1548; 311.1021; 321.3854; 324.9615; 325.3279; 329.4467; 348.4794; 350.9340; 352.2728; 358.0869; 393.7614; 396.7655; 451.0421; 454.0240; 476.8409; 489.4385; 490.2178; 491.2898; 491.8161; 521.1196; 523.9106; 527.9997; 557.7921; 557.9749; 558.9327; 568.2967; 570.8019; 572.8387; 595.6636; 601.6341; 624.1965; 626.9616; 631.4771; 633.9232; 642.1904; 649.3848; 720.8196; 732.2475; 737.1357; 758.1668; 761.8849; 764.0295; 782.4091; 787.7521; 849.9285; 871.6773; 917.3007; 937.8696; 939.1187; 960.6923; 974.1831; 977.3470; 979.5567; 980.8046; 984.8302; 987.3739; 991.0626; 1011.1497; 1033.4005; 1056.0443; 1064.2062; 1065.6798; 1083.6461; 1085.1241; 1087.6273; 1108.6415; 1122.2529; 1137.2924; 1183.1269; 1200.1809; 1207.4057; 1210.6345; 1213.2306; 1224.8722; 1226.2710; 1230.5206; 1234.1892; 1240.3860; 1245.3185; 1255.0684; 1257.2985; 1283.5127; 1291.8152; 1293.4453; 1298.1743; 1304.4189; 1314.7763; 1371.4103; 1395.8930; 1431.5867; 1440.2047; 1444.6692; 1458.0608; 1463.5202; 1502.6062; 1502.9331; 1506.5168; 1514.9154; 1516.5718; 1523.5637; 1531.2066; 1533.9477; 1550.0667; 1615.0878; 1626.3887; 1651.6732; 1821.2013; 1888.1983; 3044.2509; 3057.8202; 3064.9299; 3070.1352; 3096.9757; 3127.6947; 3130.1032; 3134.5327; 3135.6450; 3136.0285; 3140.9679; 3145.2141; 3158.2117; 3164.3989; 3174.6919; 3182.1783; 3188.3035.

Cartesian Forces: Max 0.000000724 RMS 0.000000260

Item	Value	Threshold	Converged?
Maximum Force	0.000000	0.000450	YES
RMS Force	0.000000	0.000300	YES
Maximum Displacement	0.000089	0.001800	YES
RMS Displacement	0.000010	0.001200	YES

FILE: Yb_Sq2_PhYbOTf3_GS_Anion_B3LYP_M062x_Add12_Conf5.out

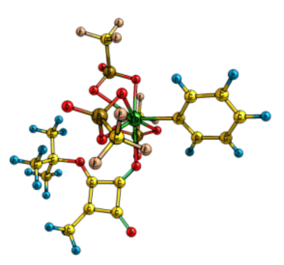

Conformer 18

E(RB3LYP/6-31G(d)/MWB59) = -3731.45556534
Convg = 0.8302D-08 -V/T = 2.0122

Temperature 195.150 Kelvin. Pressure 1.00000 Atm.
elec. and zero-point Energies= -3731.084094
elec. and thermal Energies= -3731.060110
elec. and thermal Enthalpies= -3731.059492
elec. and thermal Free Energies= -3731.131752

RB3LYP/6-31G(d)/MWB59/THF(IEFPCM) = -3731.52151540

RM06-2x/6-311+G(d,p)/MWB59/THF(IEFPCM) = -3731.36189588

Geometry at RB3LYP/6-31G(d)/MWB59
Charge = -1 Multiplicity = 1
Yb -0.1151494814 1.5012921942 0.1426358148
O 1.1386446947 -0.3665787716 -0.7761950953
S 2.0959335682 0.3765684321 -1.6810651275
O 3.5013511031 -0.0121944575 -1.5945415914
O 1.7390132114 1.8229415646 -1.5831660688
C 1.5617147668 -0.1434848472 -3.3888634924
F 1.8247959607 -1.4490050594 -3.55959412
F 0.2544866988 0.055151101 -3.5616448889
F 2.2377758847 0.5549422157 -4.3078913595
O 2.2211852886 1.7312327294 1.3445653256
O 2.0826985577 3.4879475733 3.164454485
O 0.6140912836 3.5203111119 1.1001772013
S 1.9270791802 3.1378931963 1.7558995429
C 3.1809030082 4.2039290182 0.8775923863
F 4.4139937939 3.80761965 1.2235115131
F 3.0567660231 4.1276596889 -0.4461392427
F 3.0188131937 5.4803384794 1.2529480851
C -0.2167816283 -2.4596423925 0.9684965999
C -0.6991158314 -3.7027765789 0.5644165886
C -1.819039469 -3.1052424546 -0.203892722
C -1.2675424528 -1.7214250931 0.1949186024
O -1.5003520319 -0.5527732875 -0.0473811165
O -2.7301314018 -3.5473273188 -0.8644586908
O 0.6974808364 -1.8815841908 1.6870742602
C 1.9019226515 -2.5079085286 2.2829988094
C -0.3891395761 -5.1500090016 0.7059334713
H 0.6710473472 -5.3755847179 0.5506539098
H -0.9792610468 -5.708600056 -0.0285456476
H -0.6643998595 -5.5203852168 1.7023898359
C 1.4804963418 -3.5890890955 3.2803107056
H 2.3633868903 -3.9362032916 3.8283739928
H 0.768519364 -3.1808790714 4.0046880666
H 1.0306795358 -4.4581518913 2.7940755026
C 2.560389832 -1.3293667525 3.0007533863
H 3.494303556 -1.6609281497 3.4684199245
H 2.7764834169 -0.5181881633 2.3016661328
H 1.8959965982 -0.9328847557 3.7738097466
C 2.7834624826 -3.0314754844 1.1477619017
H 2.2999343625 -3.8440327092 0.5952387711
H 3.0183733801 -2.2254844802 0.4470470955
H 3.7202682554 -3.4193000051 1.5633237011
C -1.4051307757 2.4680259385 -1.6184902327
C -0.8683795398 3.3841841831 -2.5524386257
C -2.784735468 2.1958510425 -1.7678296346
H 0.1906288209 3.6369587336 -2.5060751886
H -3.2723439355 1.5093351432 -1.0764180655
C -1.6387592328 3.9869158054 -3.5533804345
C -3.5690161681 2.778784805 -2.7692513941
H -1.1795670978 4.6917892327 -4.2463466571
H -4.6277851961 2.5325237525 -2.8464489214
C -2.9967576356 3.6820391562 -3.6677588007
H -3.6019494524 4.1432911452 -4.4470233792
O -2.7997257197 0.1737751127 3.1254825593
S -1.8642194038 1.1918464042 2.6479538688
C -1.8655081303 2.494134964 3.980714658
O -2.2152020783 1.9256130184 1.3854967633
O -0.4245247119 0.7736556229 2.5080532343
F -1.0967606704 3.5280803252 3.6501512934
F -1.4145008783 1.9591303668 5.1240974436
F -3.1193022294 2.9272139527 4.1746156574

Frequency: 15.0931; 17.9987; 20.4377; 22.2186; 25.9304; 26.9415; 28.9436; 31.8067; 32.3018; 35.4765; 37.9240; 39.2243; 49.5361; 55.1292; 56.1119; 57.9206; 65.4945; 66.2658; 72.0237; 82.1172; 84.0600; 98.1169; 100.8973; 102.5416; 117.6570; 121.2881; 128.2271; 130.3966; 141.2548; 144.6024; 149.8815; 165.3895; 173.9315; 177.3093; 190.0647; 200.3449; 205.6990; 209.5557; 214.0257; 216.7233; 222.8856; 225.2260; 227.3083; 230.7318; 251.2351; 253.5994; 264.8596; 299.9581; 304.4603; 307.3611; 310.1561; 323.3305; 325.0495; 328.3971; 342.7551; 349.7927; 351.2236; 351.7328; 358.2821; 393.9285; 397.0450; 446.7487; 450.2039; 480.2709; 486.9488; 489.8886; 491.0221; 491.5867; 520.3763; 521.9135; 526.4007; 558.2552; 558.9968; 559.1662; 568.2551; 569.8299; 571.5094; 596.3317; 615.3004; 622.1059; 625.7803; 631.2404; 634.3232; 646.9799; 650.9201; 714.5394; 735.7879; 760.8934; 761.3344; 762.8633; 764.5697; 784.7694; 792.5888; 841.8214; 866.2287; 911.5260; 936.5960; 937.7209; 971.6428; 972.0104; 977.0229; 981.0167; 982.5038; 983.9288; 985.5575; 1000.3296; 1012.3213; 1035.3405; 1053.8849; 1062.1308; 1070.3678; 1085.8188; 1087.0451; 1107.9934; 1113.8635; 1128.3378; 1132.9784; 1183.6090; 1199.7978; 1210.3560; 1212.3032; 1212.4524; 1213.0967; 1225.2054; 1229.1349; 1230.6847; 1233.1432; 1247.1251; 1253.7392; 1256.4206; 1283.4503; 1293.9705; 1295.7334; 1296.4922; 1300.5941; 1308.8900; 1369.6639; 1395.2034; 1431.1230; 1438.6208; 1441.8727; 1459.0275; 1463.7295; 1499.7900; 1507.8820; 1512.0466; 1518.5607; 1520.1199; 1522.7329; 1527.5481; 1532.3386; 1549.9330; 1615.1210; 1627.4375; 1647.8370; 1825.5682; 1884.6690; 3042.2106; 3060.8627; 3063.9357; 3071.2445; 3101.5840; 3124.5772; 3128.6332; 3131.0343; 3135.6335; 3140.8922; 3143.9228; 3154.0891; 3157.1976; 3157.6009; 3163.1478; 3168.3157; 3175.1145.

Cartesian Forces: Max 0.000000747 RMS 0.000000299

Item	Value	Threshold	Converged?
Maximum Force	0.000000	0.000450	YES
RMS Force	0.000000	0.000300	YES
Maximum Displacement	0.000003	0.001800	YES
RMS Displacement	0.000001	0.001200	YES

FILE: Yb_Sq2_PhYbOTf3_GS_Anion_B3LYP_M062x_Add12_Conf5-_Y.out

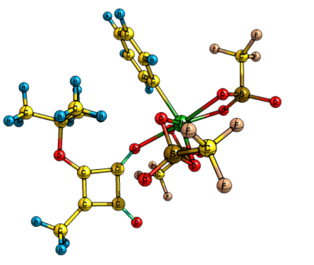

Conformer 19

E(RB3LYP/6-31G(d)/MWB59) = -3731.46166024
Convg = 0.2838D-08 -V/T = 2.0122

Temperature 195.150 Kelvin. Pressure 1.00000 Atm.
elec. and zero-point Energies= -3731.090537
elec. and thermal Energies= -3731.066403
elec. and thermal Enthalpies= -3731.065785
elec. and thermal Free Energies= -3731.138829

RB3LYP/6-31G(d)/MWB59/THF(IEFPCM) = -3731.52792060

RM06-2x/6-311+G(d,p)/MWB59/THF(IEFPCM) = -3731.36870306

Geometry at RB3LYP/6-31G(d)/MWB59
Charge = -1 Multiplicity = 1
Yb 0.2680032847 2.6076231024 0.9059347735
O -1.516509283 2.7097524473 2.6542656714
S -0.5963210133 2.8671655547 3.8274562342
O -0.8189587227 3.9828906605 4.7396753797
O 0.8002469952 2.6668724931 3.2930975045
C -0.8952776186 1.3649437072 4.894183113
F -2.1765565292 1.3179682286 5.2677810846
F -0.5937436467 0.2251401879 4.2502559001
F -0.1263806015 1.4355805518 5.9901777835
O 0.1606565744 4.9684216707 1.4156177097
O -0.8282177322 6.4801790957 -0.3685925316
O 0.2923597936 4.2614839316 -0.8709798412
S 0.1711068012 5.485116368 -0.0001257463
C 1.8065793455 6.3626859595 -0.1703390631
F 1.7991204704 7.4636834576 0.5918022555
F 2.8169333703 5.5813451544 0.2060948846
F 1.9844265906 6.7231106512 -1.4482607571
C 0.0899152757 -2.2922946712 1.3202767597
C -1.1624043321 -2.7847079519 1.6481694887
C -1.7346775803 -1.4411881971 1.8904068411
C -0.3492165614 -0.8656421645 1.5078316176
O 0.1799365329 0.2240228637 1.4024617081
O -2.7966433006 -1.0001328442 2.2526459794
O 1.1642539292 -2.9700878313 0.9995932978
C 2.4548663613 -2.3336288113 0.5756982569
C -1.7392804031 -4.1474551556 1.7235830533
H -0.9752710661 -4.9180627016 1.5855328597
H -2.508652136 -4.273092323 0.9503463158
H -2.2366630525 -4.299801824 2.6893952416
C 2.2240505595 -1.5771903847 -0.7294589507
H 3.1748710307 -1.1603827918 -1.0768524596
H 1.8320005887 -2.2505055363 -1.4998349369
H 1.5384909403 -0.736799812 -0.6086631025
C 3.3470755437 -3.557366499 0.3642442532
H 4.3372003248 -3.2334433967 0.0284115443
H 3.463950551 -4.1213187917 1.2958449949
H 2.9225943604 -4.221664731 -0.3957342212
C 2.9852126496 -1.4482117041 1.7007918515
H 2.3660309196 -0.5632606432 1.8522182392
H 3.0566435475 -2.0155124749 2.6360775988
H 3.9841764267 -1.0867801698 1.4365579219
C 2.6547863409 2.2955143448 0.7206686792
C 3.5364492738 2.2969655118 1.8267832927
C 3.2684713268 2.0512256256 -0.5310542957
H 3.1392299322 2.4885446717 2.8224732277
H 2.6552411276 2.0391940035 -1.4317076279
C 4.91173785 2.0606032261 1.7051824457
C 4.6410417438 1.8135363761 -0.6745913255
H 5.5481477283 2.0736134378 2.5901553441
H 5.0648134654 1.6352270634 -1.6631815987
C 5.4717027868 1.8124278455 0.449553376
H 6.5408183996 1.6311323055 0.346691925
O -1.6665377513 -0.319962699 -1.3876497932
S -1.2268267913 1.0770797883 -1.3085414242
C -1.7849342087 1.8381629745 -2.9146458593
O 0.2579394117 1.3004750063 -1.2528788635
O -1.8374133718 1.9225857953 -0.2301675305
F -1.4834732473 3.1324083017 -2.9698227131
F -3.1105343214 1.6916777594 -3.038703372
F -1.1846731902 1.2059783398 -3.9341385526

Frequency: 6.9688; 16.3726; 20.6127; 20.9645; 26.8461; 29.4574; 32.0355; 32.6411; 35.9038; 36.3137; 41.7226; 43.0745; 46.0864; 48.2627; 56.1367; 61.0033; 64.1658; 68.1040; 71.0938; 73.1889; 78.9878; 81.0483; 88.9310; 92.1009; 113.2185; 125.8032; 133.5174; 138.7550; 142.6009; 145.5090; 155.2979; 163.8570; 169.9088; 176.1762; 186.6933; 187.0628; 202.8548; 206.9867; 210.9341; 213.3245; 215.0665; 215.7788; 219.7835; 227.4068; 250.3554; 266.9010; 270.3591; 291.5837; 304.4265; 305.8219; 308.8255; 321.7254; 323.7179; 325.8149; 329.7745; 347.4241; 347.9226; 350.4031; 357.8595; 394.1299; 396.2288; 450.5881; 452.3316; 477.0894; 488.3504; 490.8218; 491.3142; 491.7483; 520.6528; 523.6689; 529.4053; 556.2494; 558.5958; 558.8560; 568.1763; 569.3927; 574.9921; 594.2607; 601.5551; 624.5080; 627.4608; 633.1793; 634.4875; 645.1628; 648.2265; 720.0916; 732.1632; 738.9138; 758.6592; 762.8855; 763.5054; 782.2975; 787.4923; 846.0444; 871.0142; 915.3417; 936.5067; 940.3633; 960.0075; 976.8802; 978.5931; 980.9568; 984.8276; 986.6992; 987.6874; 988.9260; 1011.3633; 1032.7235; 1055.4854; 1062.6692; 1067.5328; 1084.0255; 1084.5879; 1086.4422; 1112.1872; 1119.2379; 1137.4907; 1182.3300; 1197.3710; 1211.8097; 1212.6946; 1214.3064; 1218.2642; 1226.0117; 1231.0428; 1234.3688; 1241.6073; 1246.0216; 1253.1451; 1256.1718; 1280.8362; 1292.4754; 1294.2972; 1297.6672; 1306.7687; 1314.3568; 1370.2707; 1397.4694; 1431.0760; 1441.6293; 1445.6835; 1457.7013; 1464.5437; 1500.5488; 1502.6565; 1506.2327; 1514.0752; 1516.1792; 1520.8558; 1528.7915; 1529.6225; 1548.5852; 1611.8909; 1623.5182; 1649.4120; 1821.9724; 1894.3107; 3044.2528; 3057.0828; 3062.2468; 3068.0147; 3096.6667; 3127.5687; 3131.0425; 3132.0078; 3133.3153; 3136.8256; 3140.5228; 3142.6526; 3159.7542; 3168.0058; 3175.7283; 3176.4688; 3182.8584.

Cartesian Forces: Max 0.000024727 RMS 0.000003833

Item	Value	Threshold	Converged?
Maximum Force	0.000021	0.000450	YES
RMS Force	0.000002	0.000300	YES
Maximum Displacement	0.001595	0.001800	YES
RMS Displacement	0.000216	0.001200	YES

FILE: Yb_Sq2_PhYbOTf3_GS_Anion_B3LYP_M062x_Add12_Conf5-1.out

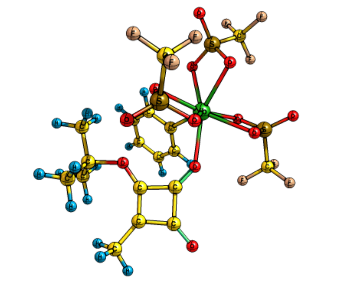

Conformer 20

E(RB3LYP/6-31G(d)/MWB59) = -3731.45822771
Convg = 0.7438D-08 -V/T = 2.0122

Temperature 195.150 Kelvin. Pressure 1.00000 Atm.
elec. and zero-point Energies= -3731.086638
elec. and thermal Energies= -3731.062737
elec. and thermal Enthalpies= -3731.062119
elec. and thermal Free Energies= -3731.133961

RB3LYP/6-31G(d)/MWB59/THF(IEFPCM) = -3731.52388284

RM06-2x/6-311+G(d,p)/MWB59/THF(IEFPCM) = -3731.36280061

Geometry at RB3LYP/6-31G(d)/MWB59
Charge = -1 Multiplicity = 1

```
Yb  0.2118057405  2.1202882323  0.8583156663
O  -1.6264053122  2.9984755175  2.2826060191
S  -0.7455455993  3.8161361666  3.1802660473
O  -1.1183970117  5.2020686896  3.4372171648
O   0.6714469731  3.544770447  2.7313355416
C  -0.8426698998  2.9853898214  4.8464889502
F  -2.1053807513  3.0058857708  5.2886974337
F  -0.430547286  1.7143538252  4.7771043564
F  -0.0673951288  3.6391042425  5.7211028831
O  -0.3973625091  4.1596142717 -0.3515690548
O   1.0333563631  4.7038766112 -2.3750525554
O   1.8910150011  3.4314740739 -0.3537510904
S   0.9589907448  4.4571942277 -0.9385585307
C   1.4721479308  6.070561385 -0.1584580224
F   0.6226597706  7.0358902993 -0.5334996779
F   1.478355848  5.9886257443  1.1703023363
F   2.7033816028  6.3929760824 -0.5801891734
C  -0.0909322522 -2.0885189285  1.4774864884
C  -0.6245038529 -3.0504534154  2.3346462671
C  -1.2210317565 -2.0305617596  3.2311617193
C  -0.6531005373 -0.9562730421  2.2821527379
O  -0.6538326558  0.2533339272  2.1896402617
O  -1.8604100322 -2.0685561378  4.257119981
O   0.6117532168 -1.9286936095  0.3969951007
C   1.2173856801 -3.0037839932 -0.4295077257
C  -0.7163442296 -4.5259502719  2.4932081102
H   0.2403487973 -5.0356396984  2.3416420756
H  -1.4357131885 -4.9576034439  1.7847589023
H  -1.0766801697 -4.7459384601  3.504011236
C   2.2860381397 -3.7104929767  0.40632248
H   2.819500162 -4.4325634913 -0.2221633945
H   3.0070921739 -2.9820602711  0.7904382148
H   1.8575718513 -4.2570122032  1.2515921392
C   1.8517130739 -2.221678891 -1.5796838648
H   2.357648116 -2.9183944989 -2.2577887958
H   1.0861554886 -1.6726355784 -2.1319201307
H   2.5799344406 -1.5049724089 -1.1921289379
C   0.1090558244 -3.9243694017 -0.9429700948
H  -0.3416217306 -4.5225681668 -0.146313788
H  -0.6684032543 -3.3269028566 -1.4278289876
H   0.5290166981 -4.6175635295 -1.6804340895
C   2.2268134569  0.9190744002  1.4847330954
C   2.4826630403  0.3908255108  2.7707502763
C   3.2475136905  0.684479267  0.5341040397
H   1.7608545297  0.5557112895  3.5724456772
H   3.1380160815  1.0861797706 -0.4731122745
C   3.6391015938 -0.3339768285  3.086316797
C   4.4113972199 -0.0385091037  0.825307169
H   3.7875684655 -0.7143900104  4.0970704755
H   5.1695644058 -0.1870354983  0.0560793907
C   4.6099915752 -0.5602178845  2.107515425
H   5.5155103895 -1.1170480634  2.3444784619
O  -1.4137083821 -0.7913084061 -1.8184077285
S  -1.1776919419  0.6066366769 -1.443621581
C  -1.8803880208  1.5799799248 -2.8697748271
O   0.2605685426  1.043277971 -1.3390176801
O  -1.8712931623  1.1185276001 -0.2193857288
F  -1.7784689343  2.8881034803 -2.6618022172
F  -3.1731425071  1.2592398842 -3.0224227889
F  -1.2219072308  1.2577237723 -3.990883158
```

Frequency: 13.1034; 16.6151; 17.4519; 25.2678; 27.5291; 30.4868; 31.2480; 36.3519; 36.8366; 38.0258; 42.2616; 44.9380; 51.0633; 52.4476; 55.0791; 59.7811; 64.1207; 67.7686; 75.3579; 79.8254; 83.1790; 86.3110; 93.9391; 110.5878; 126.3450; 133.5888; 135.4572; 140.0407; 141.1273; 147.4349; 153.5519; 163.2956; 175.8822; 178.0342; 183.5848; 202.2622; 206.8301; 210.2115; 212.9655; 213.9984; 218.1978; 220.1385; 225.5179; 232.3324; 247.6464; 252.9866; 273.5908; 296.9313; 303.5350; 306.6421; 308.9140; 322.4954; 323.0693; 324.0630; 338.7018; 349.9037; 352.6186; 354.1713; 358.1755; 394.2988; 394.8593; 451.2185; 454.6474; 481.6611; 486.1459; 489.2800; 489.5029; 490.7818; 522.9125; 524.6226; 528.8589; 557.2299; 558.0950; 558.6512; 570.6913; 572.1186; 573.6559; 603.3784; 611.2414; 625.0806; 626.1481; 631.8076; 634.9164; 645.2647; 646.8398; 720.7712; 735.7311; 760.1244; 762.2383; 763.2296; 764.7763; 784.3571; 788.9950; 839.9957; 870.4266; 908.7461; 937.6554; 941.2299; 975.8874; 977.7792; 978.1790; 979.5155; 982.5742; 985.5622; 987.9967; 999.7858; 1011.2746; 1031.3359; 1053.1743; 1065.5921; 1073.8501; 1083.0377; 1083.8894; 1108.5417; 1110.7328; 1118.0082; 1139.0982; 1181.8617; 1199.3878; 1210.2451; 1211.1799; 1212.1185; 1215.9450; 1224.5024; 1231.0151; 1232.1692; 1233.1178; 1238.2491; 1255.0713; 1258.4084; 1283.1608; 1290.4603; 1295.4135; 1296.7758; 1304.5731; 1312.6749; 1370.1306; 1395.1012; 1430.5785; 1435.4400; 1442.1235; 1457.5880; 1461.3889; 1500.4430; 1505.7841; 1514.6899; 1519.4707; 1521.0141; 1522.9562; 1530.3666; 1532.1146; 1552.9981; 1611.1887; 1622.0354; 1646.6257; 1837.7481; 1885.1422; 3043.4939; 3060.7549; 3063.9395; 3071.4171; 3102.5439; 3127.7418; 3128.2112; 3131.1866; 3135.0656; 3136.7063; 3144.9278; 3147.8168; 3149.2143; 3158.1104; 3158.9667; 3172.6183; 3173.4022.

Cartesian Forces: Max 0.000002097 RMS 0.000000757

Item	Value	Threshold	Converged?
Maximum Force	0.000001	0.000450	YES
RMS Force	0.000000	0.000300	YES
Maximum Displacement	0.000027	0.001800	YES
RMS Displacement	0.000006	0.001200	YES

FILE: Yb_Sq2_PhYbOTf3_GS_Anion_B3LYP_M062x_Add12_Conf5-1-.out

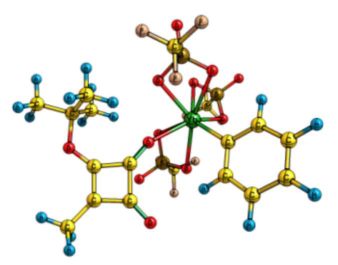

Conformer 21

E(RB3LYP/6-31G(d)/MWB59) = -3731.45655938
Convg = 0.5647D-08 -V/T = 2.0122

Temperature 195.150 Kelvin. Pressure 1.00000 Atm.
elec. and zero-point Energies= -3731.085300
elec. and thermal Energies= -3731.061213
elec. and thermal Enthalpies= -3731.060595
elec. and thermal Free Energies= -3731.132858

RB3LYP/6-31G(d)/MWB59/THF(IEFPCM) = -3731.52381519

RM06-2x/6-311+G(d,p)/MWB59/THF(IEFPCM) = -3731.36246939

Geometry at RB3LYP/6-31G(d)/MWB59
Charge = -1 Multiplicity = 1

```
Yb  0.3182438682  2.4473761414  0.3866828783
O   2.5935261164  1.5789991667  0.2647644945
S   3.1207887838  2.5000975231 -0.8086875487
O   4.4352968891  3.0954141728 -0.6094952483
O   1.9765696342  3.385348437 -1.2022875236
C   3.3367260621  1.3591409199 -2.2668581925
F   4.333097475  0.490000226 -2.0186257529
F   2.2178172147  0.6592400115 -2.4947797932
F   3.6428179546  2.0578433987 -3.3634329673
O  -0.4330970068  4.5597030771  0.9990852186
O   1.0861539058  6.5758924863  0.9696522654
O   1.8807849522  4.2493323055  1.6215459602
S   0.7969065575  5.2679942813  1.5474445225
C   0.352771153  5.6368326462  3.3236351183
F  -0.8434987204  6.2336200417  3.3874095185
F   0.3228694871  4.5229685056  4.0610309378
F   1.273646261  6.4619362866  3.8416003666
C   0.392485259 -2.489471348  1.0931055131
C  -0.8430867653 -3.1061799524  1.0129055308
C  -1.5141824129 -1.9085605562  0.462582454
C  -0.1484718516 -1.1804491462  0.5765493576
O   0.312465398 -0.0743430157  0.3765522319
O  -2.6238950907 -1.6412355213  0.0718171986
O   1.5269303427 -3.0049093278  1.5030637955
C   2.813467319 -2.2416186982  1.5337822924
C  -1.3416849059 -4.4567710988  1.3639922674
H  -0.5204571062 -5.1554335393  1.5497540387
H  -1.9802916844 -4.8490023336  0.5636310173
H  -1.9632364272 -4.4042916695  2.2679651272
C   3.1593246554 -1.7896960807  0.1164407105
H   4.146254797 -1.3173085125  0.1144707828
H   3.1861413043 -2.6487989751 -0.5637438843
H   2.4533996327 -1.0472054561 -0.2559531346
C   3.7947830403 -3.3029675517  2.0330569548
H   4.7971139348 -2.8695275922  2.1081185326
H   3.5003011261 -3.6689486587  3.0220588026
H   3.8346903134 -4.1529468519  1.3435875969
C   2.6888994813 -1.0887488594  2.5277823068
H   1.9686526467 -0.3371567408  2.2040433191
H   2.3957736948 -1.4640839794  3.5141059697
H   3.6570914686 -0.5859189225  2.6212260164
C  -0.9787424511  2.3040818609 -1.6176977451
C  -0.751034553  3.1627122171 -2.7195255374
C  -2.0608871008  1.4054042805 -1.7707710372
H   0.0665422843  3.8813674343 -2.6789884933
H  -2.3136105554  0.7186068017 -0.9652049169
C  -1.5334662111  3.1347419407 -3.8795625651
C  -2.8475879936  1.3566733446 -2.927489021
H  -1.3197789667  3.821748954 -4.6982645444
H  -3.6674465942  0.6421205243 -2.9961631918
C  -2.5870051927  2.2248260921 -3.9897381858
H  -3.1981345928  2.1945373951 -4.8909738742
O  -1.435714205 -0.1227272967  3.2047404892
S  -1.2096218488  1.2599091152  2.770533356
C  -2.0912279131  2.3037304931  4.0418054451
O  -1.799475444  1.675241517  1.4572616421
O   0.2215545181  1.7263770689  2.7703457567
F  -2.2078907154  3.5658549146  3.6296908893
F  -1.415290642  2.2770999079  5.1967054721
F  -3.316194516  1.8032001193  4.2461406325
```

Frequency: 13.3342; 14.0582; 23.4492; 25.1411; 27.5045; 29.6634; 31.0750; 34.3642; 36.9954; 39.4054; 47.4179; 49.1516; 52.0271; 52.7628; 57.8482; 60.7337; 65.1166; 67.5362; 75.8508; 76.1871; 81.2919; 83.8737; 86.6890; 90.3605; 98.7574; 112.8830; 120.2531; 128.4560; 136.6018; 144.6171; 153.7042; 162.0141; 170.0161; 180.2630; 188.5389; 189.6205; 202.3602; 203.7966; 214.7323; 216.3415; 217.4489; 221.9240; 225.8638; 232.5650; 253.2785; 263.9420; 270.9897; 293.0574; 304.2545; 307.9773; 311.8395; 322.0960; 324.5226; 326.9636; 329.5449; 348.4376; 349.4360; 352.2525; 359.9873; 393.1954; 396.6114; 446.7946; 454.1378; 477.0551; 488.7493; 490.3929; 490.8281; 491.3580; 520.5878; 522.6336; 528.2180; 557.4791; 557.9659; 558.8555; 567.8162; 569.8149; 574.2505; 593.1568; 602.5313; 617.2548; 623.5447; 633.7651; 634.9197; 641.4407; 650.7903; 718.4340; 732.1494; 736.7173; 757.0041; 759.3792; 762.6702; 783.2892; 787.4367; 849.2763; 870.3300; 918.0277; 937.2121; 938.3522; 960.4655; 966.4732; 977.4773; 978.5527; 981.1691; 984.6078; 988.1215; 990.4210; 1011.6599; 1034.0842; 1054.8509; 1064.0258; 1066.1883; 1084.8738; 1085.4919; 1089.0434; 1118.3133; 1122.7360; 1136.2746; 1183.2584; 1200.2781; 1206.7975; 1212.4512; 1215.6186; 1221.7171; 1225.5415; 1228.2479; 1230.2917; 1242.1293; 1246.2798; 1247.5033; 1252.1142; 1279.8022; 1292.0887; 1294.3784; 1298.5988; 1309.8133; 1317.7299; 1373.3142; 1395.7010; 1431.2510; 1438.5717; 1444.3296; 1457.9653; 1462.2574; 1501.5945; 1503.2476; 1506.3483; 1513.0123; 1514.7272; 1524.9366; 1530.0261; 1531.9281; 1547.1168; 1615.5077; 1626.9382; 1651.6700; 1820.6400; 1888.9059; 3043.9701; 3058.1163; 3065.3585; 3071.7713; 3098.3906; 3129.0556; 3131.2110; 3134.1783; 3135.5063; 3136.4925; 3141.0179; 3147.9807; 3162.5041; 3172.0801; 3183.1995; 3187.3801; 3190.7752.

Cartesian Forces: Max 0.000001054 RMS 0.000000431

Item	Value	Threshold	Converged?
Maximum Force	0.000000	0.000450	YES
RMS Force	0.000000	0.000300	YES
Maximum Displacement	0.000067	0.001800	YES
RMS Displacement	0.000015	0.001200	YES

FILE: Yb_Sq2_PhYbOTf3_GS_Anion_B3LYP_M062x_Add12_Conf6.out

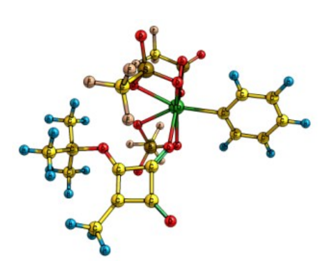

Conformer 22

E(RB3LYP/6-31G(d)/MWB59) = -3731.45825116
Convg = 0.5032D-09 -V/T = 2.0122

Temperature 195.150 Kelvin. Pressure 1.00000 Atm.
elec. and zero-point Energies= -3731.086801
elec. and thermal Energies= -3731.062783
elec. and thermal Enthalpies= -3731.062165
elec. and thermal Free Energies= -3731.134303

RB3LYP/6-31G(d)/MWB59/THF(IEFPCM) = -3731.52549980

RM06-2x/6-311+G(d,p)/MWB59/THF(IEFPCM) = -3731.36350747

Geometry at RB3LYP/6-31G(d)/MWB59
Charge = -1 Multiplicity = 1

```
Yb -0.0262683133  1.8285221349  0.0772727895
O   2.2641266064  0.7676057172  0.3403629588
S   2.6704842867  0.8924746714 -1.0983802763
O   3.9395179723  1.5459023452 -1.399921517
O   1.4491824734  1.329385494 -1.8564669183
C   2.961125806 -0.8701526473 -1.6455623789
F   3.8444997781 -1.4605828594 -0.8163172205
F   1.8337030168 -1.6008392087 -1.6347407641
F   3.461207043 -0.8952402017 -2.8845022387
O  -0.2298490373  3.6784949853  1.611398988
O   0.655972189  5.9418818487  0.8727556398
O   1.4739957844  3.7316997895 -0.0777764014
S   0.8971491339  4.519043919  1.0680549805
C   2.2041857473  4.4194482834  2.3920216785
F   1.7633937634  5.0149835381  3.5081973697
F   2.4944616348  3.1446091456  2.6702170885
F   3.3163966489  5.0392093974  1.9822939285
C  -0.099692559 -2.4899622129  0.5036502092
C  -0.8989082588 -3.5809033253  0.1769572716
C  -1.7105156354 -2.7925113072 -0.7864696727
C  -0.8532763709 -1.5688205152 -0.3968301528
O  -0.7938775956 -0.3799380991 -0.6409579794
O  -2.6258030699 -3.0443397819 -1.5336237691
O   0.921627622 -2.1087198754  1.2099722321
C   1.6871728285 -2.9126372372  2.1867040933
C  -1.0782790383 -5.0120506357  0.533656325
H  -0.1323143567 -5.558336153  0.6069526022
H  -1.7045434678 -5.4893451172 -0.2277959992
H  -1.5954596485 -5.1130051281  1.4970352752
C   0.7336581476 -3.4930058327  3.2325900879
H   1.3160911558 -3.8959851545  4.0686109807
H   0.0684679095 -2.7108621543  3.609921425
H   0.1233112883 -4.3070554368  2.8315871379
C   2.6150614925 -1.8623054989  2.800434422
H   3.2727497715 -2.3339462452  3.5390794378
H   3.2222821571 -1.3936836399  2.0216797018
H   2.0285295698 -1.0757680146  3.2817978243
C   2.4809822699 -3.9724773392  1.4192336662
H   1.8281039112 -4.7159214915  0.9509935379
H   3.0832542999 -3.4967513782  0.6399331316
H   3.1520746132 -4.5005164746  2.1057900326
C  -1.5159656577  2.7720465234 -1.5192048919
C  -1.0435183235  3.3612705636 -2.7146899861
C  -2.9195963378  2.7884058552 -1.3503946623
H   0.0280193766  3.3824639232 -2.9136591364
H  -3.3553031206  2.3508033549 -0.4523504795
C  -1.8973232244  3.9238582778 -3.6701046555
C  -3.7889013499  3.3451796995 -2.2951088625
H  -1.4861841576  4.3681722851 -4.5763274283
H  -4.864857514  3.3330767901 -2.1216539023
C  -3.2783977963  3.917017258 -3.4625426161
H  -3.9491108262  4.3527554446 -4.2018659594
O  -1.8462610987 -0.8938259293  2.7557848159
S  -1.3935218703  0.4842351792  2.5182697578
C  -1.901042316  1.4015040859  4.0581032635
O  -2.0479674759  1.2197705954  1.3854384163
O   0.0842971766  0.6831168961  2.3883119027
F  -1.558644586  2.6849833257  4.0000402336
F  -1.3018742309  0.838786724  5.1199432127
F  -3.227808575  1.3087144688  4.2157782533
```

Frequency: 11.9373; 17.3067; 18.6918; 23.9219; 25.3626; 27.9467; 31.8564; 34.1207; 34.6728; 39.9651; 44.7046; 47.7954; 50.0566; 55.6903; 57.5191; 58.5582; 65.0668; 72.8858; 77.2670; 79.7979; 85.1683; 92.2786; 97.5372; 99.1575; 112.1794; 120.0065; 130.2883; 133.2768; 135.4783; 137.7671; 142.9236; 158.9458; 171.9566; 177.1119; 190.4368; 203.2277; 204.1716; 210.1934; 213.2395; 215.3332; 217.9554; 219.9105; 220.4196; 230.3902; 243.1020; 253.6968; 267.6119; 297.2988; 303.4865; 304.7260; 308.7419; 320.5861; 325.0118; 326.3124; 337.7686; 346.6299; 348.8524; 349.8633; 357.2485; 393.5288; 398.0700; 447.9776; 453.9750; 482.0034; 487.2014; 488.2430; 490.8574; 492.0085; 519.3947; 521.5466; 528.6753; 555.5369; 557.9619; 559.2217; 567.0775; 567.5193; 576.3058; 597.6852; 613.2592; 621.9236; 626.2228; 631.4864; 634.1610; 646.5364; 652.0541; 716.1164; 737.9089; 754.3388; 760.8320; 763.0671; 766.6290; 781.8906; 792.3303; 845.1147; 867.7988; 913.9143; 936.9064; 942.4546; 973.8937; 976.0067; 977.7517; 981.7626; 985.2354; 988.5878; 989.7108; 1001.4911; 1011.8834; 1036.8459; 1052.7655; 1064.4467; 1075.1589; 1086.0644; 1086.7743; 1114.7259; 1118.9999; 1129.2902; 1147.9686; 1183.6667; 1193.7586; 1199.4085; 1211.2017; 1213.2207; 1215.6288; 1218.5221; 1225.1713; 1229.2950; 1233.9315; 1235.5989; 1240.9203; 1253.4837; 1274.1708; 1286.5131; 1296.2644; 1297.2269; 1307.1187; 1318.5687; 1370.5843; 1395.7599; 1432.1423; 1437.5925; 1442.3679; 1459.0050; 1463.0458; 1499.7636; 1505.8603; 1513.5053; 1518.0281; 1521.1238; 1522.9758; 1529.6143; 1531.2442; 1550.1447; 1615.2174; 1627.9758; 1654.3038; 1833.6323; 1888.9543; 3043.8719; 3062.9645; 3064.2697; 3071.9664; 3102.8324; 3126.9974; 3130.3616; 3134.9317; 3135.5928; 3142.4982; 3145.5961; 3151.9397; 3155.3238; 3156.3972; 3161.4881; 3165.4459; 3174.2456.

Cartesian Forces: Max 0.000001175 RMS 0.000000436

Item	Value	Threshold	Converged?
Maximum Force	0.000000	0.000450	YES
RMS Force	0.000000	0.000300	YES
Maximum Displacement	0.000054	0.001800	YES
RMS Displacement	0.000007	0.001200	YES

FILE: Yb_Sq2_PhYbOTf3_GS_Anion_B3LYP_M062x_Add12_Conf6-_Y.out

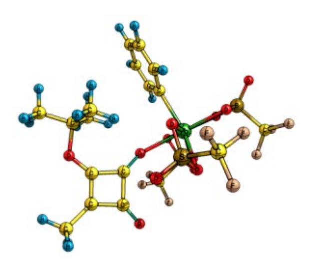

Conformer 23

E(RB3LYP/6-31G(d)/MWB59) = -3731.46302952
Convg = 0.4939D-09 -V/T = 2.0122

Temperature 195.150 Kelvin. Pressure 1.00000 Atm.
elec. and zero-point Energies= -3731.091873
elec. and thermal Energies= -3731.067753
elec. and thermal Enthalpies= -3731.067135
elec. and thermal Free Energies= -3731.139909

RB3LYP/6-31G(d)/MWB59/THF(IEFPCM) = -3731.52926963

RM06-2x/6-311+G(d,p)/MWB59/THF(IEFPCM) = -3731.36943449

Geometry at RB3LYP/6-31G(d)/MWB59
Charge = -1 Multiplicity = 1

```
Yb  0.4640153081  2.5343663928  0.5938986956
O  -1.8401946441  2.8944990461  1.4654247114
S  -1.4818121483  2.8984510841  2.9233885806
O  -1.9087270276  4.033695685  3.7334321162
O  -0.0354968385  2.4821375707  3.0014158934
C  -2.4126227345  1.4581893463  3.6579281665
F  -3.7250289137  1.6175253172  3.4654329839
F  -2.0362975992  0.2916986183  3.1123111817
F  -2.1725430234  1.3973313245  4.975721709
O   1.0197430469  4.2287478471 -1.0147697296
O   1.9257714388  6.4481623259 -0.1765185781
O   0.5111088693  4.8805568286  1.237546594
S   0.8859240426  5.4303546503 -0.1113839451
C  -0.6665386616  6.2638728969 -0.7166693971
F  -0.4727426937  6.7336430095 -1.955971556
F  -1.682414574  5.3965847683 -0.7370439905
F  -0.9788222655  7.2845620435  0.0887290067
C   0.0070643023 -2.3540319808  0.9168700432
C  -1.2674459058 -2.8318517326  0.6608341233
C  -1.8700592695 -1.4807395076  0.6122055598
C  -0.4511902032 -0.9214522464  0.881107399
O   0.0829021909  0.1626840764  1.0148820085
O  -2.9764719528 -1.0263109078  0.4606939985
O   1.1041160985 -3.0436234441  1.109428214
C   2.4584214197 -2.4194541283  1.2764964478
C  -1.8355457537 -4.1888391916  0.4833775019
H  -1.0932660917 -4.9670462398  0.6832723917
H  -2.2051670567 -4.3100679102 -0.5433178845
H  -2.6974922076 -4.3330809228  1.1466140344
C   2.8180557345 -1.676513704 -0.0070864432
H   3.8303832254 -1.2701689932  0.082995001
H   2.785150757 -2.3559394235 -0.8660748503
H   2.1572984814 -0.8291909192 -0.1971194634
C   3.3431236271 -3.6502610185  1.4776110681
H   4.3840573722 -3.3350380676  1.6005184821
H   3.0411311745 -4.2066028158  2.3714040143
H   3.2824077793 -4.3187342797  0.6124543947
C   2.4620045467 -1.5256138597  2.5143593379
H   1.8370200873 -0.6415147928  2.3830920901
H   2.129301006 -2.0869276075  3.39520705
H   3.4789226398 -1.1633460104  2.6958790304
C   2.7280980035  2.1733690227  1.339012711
C   3.1005222936  2.2107299631  2.703359892
C   3.7819572157  1.8846204232  0.4400420904
H   2.3455028298  2.4385787765  3.4544506276
H   3.5743572779  1.8417442451 -0.6287419281
C   4.407031651  1.9634687989  3.1430534619
C   5.0956253636  1.6384407469  0.8586425448
H   4.6419609134  2.004448848  4.2067025109
H   5.872989359  1.4268675639  0.1241143098
C   5.4134597174  1.6710719652  2.2191230981
H   6.4329485514  1.4826109284  2.5531014564
O  -0.4319054539 -0.3922583948 -2.2808571955
S  -0.075489375  1.0074333451 -2.0253133621
C   0.0397644547  1.7791748098 -3.7166895565
O   1.2673785503  1.2404309324 -1.3897755006
O  -1.0676858693  1.8383054013 -1.27029339
F   0.3357988927  3.0746705147 -3.6400504845
F  -1.1312951678  1.6361142057 -4.3514648839
F   0.9923400838  1.1555067232 -4.4247405811
```

Frequency: 11.9165; 16.3838; 17.6215; 20.2034; 27.2120; 27.5606; 28.8827; 32.9987; 36.3462; 38.2125; 42.4197; 43.9240; 48.7008; 52.1788; 58.3754; 59.9313; 64.0469; 67.0260; 72.2778; 74.8404; 76.0972; 81.9429; 88.2784; 93.6550; 111.5853; 125.0516; 132.7167; 137.7242; 140.0317; 144.5925; 145.9976; 166.9727; 178.3218; 178.8351; 186.0683; 187.8902; 202.7129; 209.2901; 210.9705; 212.5130; 214.1181; 217.6811; 218.9831; 227.4477; 252.4745; 266.4789; 269.8927; 291.2676; 304.1436; 306.0253; 308.6776; 320.4175; 324.3820; 325.4557; 329.4640; 347.6278; 348.1216; 350.7543; 357.9027; 393.9411; 396.2948; 449.6379; 452.4754; 477.1868; 487.9354; 490.8492; 491.1233; 491.5714; 520.6462; 523.6627; 529.3387; 556.0981; 558.0294; 558.5450; 568.2111; 569.5271; 577.3226; 593.9177; 602.3244; 622.3789; 626.8754; 632.9159; 634.4272; 645.0455; 648.2548; 719.7288; 731.9541; 739.1661; 759.4163; 761.3936; 763.2822; 782.3506; 787.4195; 845.6328; 871.0958; 916.2022; 937.1105; 940.6898; 959.8549; 976.4069; 979.1069; 979.7972; 984.6677; 986.6422; 987.8438; 988.9222; 1011.4199; 1033.0746; 1055.7698; 1063.0980; 1067.4276; 1084.1921; 1084.9258; 1086.3130; 1113.4757; 1118.9352; 1137.5088; 1182.5238; 1197.4442; 1213.5113; 1214.5340; 1216.4673; 1220.6088; 1226.4376; 1231.3809; 1236.5085; 1240.3616; 1243.6834; 1246.0442; 1253.9498; 1280.3112; 1292.5496; 1294.6121; 1297.8284; 1306.8717; 1318.1518; 1370.9232; 1397.5161; 1431.2491; 1441.8654; 1445.8214; 1457.6296; 1464.8043; 1500.7477; 1502.6551; 1506.2780; 1513.2637; 1515.9958; 1521.3664; 1528.1989; 1529.7227; 1548.1217; 1612.2378; 1623.7670; 1649.6458; 1821.8800; 1894.1585; 3044.3430; 3057.1764; 3062.6037; 3068.5253; 3096.4287; 3127.8657; 3131.6078; 3133.1899; 3133.9491; 3137.0849; 3141.1709; 3143.2995; 3160.2730; 3167.6687; 3175.3756; 3176.8831; 3182.7046.

Cartesian Forces: Max 0.000000939 RMS 0.000000362

Item	Value	Threshold	Converged?
Maximum Force	0.000000	0.000450	YES
RMS Force	0.000000	0.000300	YES
Maximum Displacement	0.000014	0.001800	YES
RMS Displacement	0.000001	0.001200	YES

FILE: Yb_Sq2_PhYbOTf3_GS_Anion_B3LYP_M062x_Add12_Conf6-1_Y.out

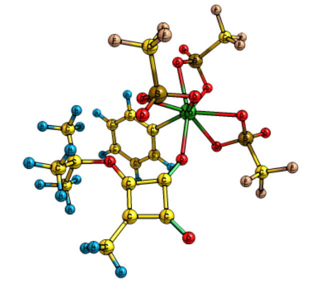

Conformer 24

E(RB3LYP/6-31G(d)/MWB59) = -3731.45748200
Convg = 0.1243D-09 -V/T = 2.0122

Temperature 195.150 Kelvin. Pressure 1.00000 Atm.
elec. and zero-point Energies= -3731.086195
elec. and thermal Energies= -3731.062121
elec. and thermal Enthalpies= -3731.061503
elec. and thermal Free Energies= -3731.134813

RB3LYP/6-31G(d)/MWB59/THF(IEFPCM) = -3731.52482730

RM06-2x/6-311+G(d,p)/MWB59/THF(IEFPCM) = -3731.36276695

Geometry at RB3LYP/6-31G(d)/MWB59
Charge = -1 Multiplicity = 1
Yb 0.2827863334 2.1924815273 0.6327918906
O -1.6882901486 3.6079229592 1.0802599948
S -1.4747141864 3.7680413366 2.561621322
O -1.4892099188 5.1189946868 3.1117342666
O -0.3095708765 2.881094276 2.9137221224
C -2.9572066844 2.9433569915 3.3402015191
F -4.0470109648 3.6925492751 3.1211707671
F -3.1682659536 1.7255480297 2.8392536758
F -2.7650100173 2.8403901141 4.662896597
O 1.4396775163 3.1906288087 -1.2147236633
O 3.160972411 5.0001306583 -0.7670750563
O 1.2686584953 4.4335580027 0.8312295692
S 1.8037041669 4.5081146361 -0.5693258747
C 0.7001546807 5.7354527693 -1.4331401751
F 1.0715064267 5.8507902895 -2.7153902065
F -0.5723106857 5.3317364502 -1.3875800265
F 0.8015139919 6.9316550155 -0.8447647153
C -0.2808508205 -2.0618743483 1.4815433222
C -1.1659193159 -2.9020005963 2.1560140563
C -2.0156054986 -1.7728355376 2.6072200312
C -1.0562194246 -0.8344813844 1.8483811484
O -0.9608548052 0.3521867085 1.614338025
O -3.0142626698 -1.6742072739 3.281551144
O 0.8086155031 -2.0482641759 0.7761183411
C 1.5576505699 -3.2219167485 0.2755755878
C -1.3899676782 -4.3448140333 2.4343350792
H -0.4688414563 -4.88700284 2.6692833709
H -1.8591188125 -4.8437840297 1.5757889446
H -2.0800219177 -4.4349366161 3.2804191364
C 2.2442524703 -3.8982544637 1.4638065194
H 2.8887142143 -4.7085309577 1.1045433798
H 2.8632406133 -3.1723337275 2.000390931
H 1.5236329619 -4.3295090431 2.1655066964
C 2.5851527391 -2.5801837941 -0.6580813118
H 3.2308917407 -3.3572984966 -1.0826713897
H 2.0825479375 -2.0447397101 -1.4673319983
H 3.1981000333 -1.8605332841 -0.1090989794
C 0.6087739813 -4.1362039201 -0.5025160225
H -0.0901248607 -4.667942392 0.1484545376
H 0.038519313 -3.5479360467 -1.2283613058
H 1.1941714428 -4.88751868 -1.0439661983
C 2.194303772 1.1758632739 1.6828651915
C 2.2260342489 0.7993278779 3.0444693679
C 3.3552050626 0.8492795367 0.9440806269
H 1.3783161928 1.045616703 3.6847885686
H 3.4078944151 1.1107147355 -0.1136695096
C 3.3100496299 0.1286109352 3.6247581952
C 4.4572836435 0.1934389592 1.5074505878
H 3.2844057707 -0.1422560481 4.6803973028
H 5.3358070075 -0.02226994 0.8990173109
C 4.4341986577 -0.1806459678 2.854580164
H 5.2872784944 -0.6898138499 3.3010350145
O -1.375337864 -0.9539098667 -1.6583976116
S -0.6979702565 0.3484358181 -1.6417110133
C -0.4679894253 0.7705040781 -3.440933283
O 0.6794986324 0.3738565306 -1.0512084366
O -1.4516922739 1.5075170989 -1.0604164536
F 0.0842225654 1.9731050458 -3.5916498997
F -1.6551195279 0.7569428467 -4.0625223226
F 0.3262968809 -0.1475445544 -4.0136538607

Frequency: 9.1322; 14.6613; 16.4834; 19.2370; 21.6100; 26.6604; 27.1174; 29.1976; 32.3333; 33.7073; 36.1017; 40.3257; 46.7968; 52.6521; 56.0329; 59.1218; 59.9556; 66.8804; 71.0082; 80.4101; 86.8635; 91.0256; 100.0226; 112.7782; 117.8174; 125.1782; 130.1848; 137.7440; 138.9018; 143.8753; 146.3179; 162.7799; 175.5821; 177.9473; 185.4157; 205.4501; 206.4217; 209.3343; 212.5543; 213.2798; 217.3238; 220.4822; 225.2458; 228.5136; 248.3191; 254.7574; 269.9788; 297.8025; 303.7517; 306.5092; 307.5805; 320.7615; 324.8332; 325.6152; 339.1672; 347.5227; 351.3800; 352.9983; 357.7600; 392.8842; 396.0326; 450.7533; 452.8451; 481.8261; 486.8810; 489.2828; 490.6947; 491.7685; 520.6886; 523.7114; 529.2006; 557.6829; 558.0614; 558.2723; 568.5815; 570.0972; 577.5611; 600.4342; 614.0720; 622.4455; 626.4174; 632.3495; 634.0947; 647.6787; 648.5564; 718.2795; 735.9350; 760.1763; 761.5353; 761.8231; 766.1376; 781.5060; 792.3115; 844.7256; 869.3328; 907.4152; 936.8807; 941.2314; 972.5164; 976.1084; 978.2040; 979.0728; 981.2998; 988.0827; 988.3472; 1000.1026; 1011.0164; 1033.6209; 1053.2787; 1064.6528; 1073.8596; 1082.1892; 1084.6466; 1108.7192; 1113.2829; 1117.4869; 1144.2182; 1181.9188; 1200.4961; 1212.0555; 1212.6581; 1214.4050; 1215.2670; 1223.0459; 1228.0429; 1230.0558; 1235.8724; 1243.8434; 1246.7987; 1250.9547; 1277.0114; 1287.0822; 1295.2474; 1296.4103; 1305.6209; 1316.4267; 1368.2880; 1396.4012; 1431.2948; 1437.6052; 1442.9260; 1457.6022; 1463.0429; 1499.5659; 1505.6675; 1513.0961; 1518.2926; 1519.9513; 1521.8243; 1528.3792; 1532.3053; 1550.9702; 1611.7309; 1622.5191; 1648.6149; 1839.3848; 1887.8071; 3042.8854; 3059.3575; 3062.2638; 3069.7701; 3101.9170; 3127.2285; 3130.1843; 3131.8204; 3133.9185; 3136.1612; 3141.9822; 3144.6764; 3148.6746; 3154.7557; 3155.2659; 3162.6557; 3172.0034.

Cartesian Forces: Max 0.000001271 RMS 0.000000455

Item	Value	Threshold	Converged?
Maximum Force	0.000000	0.000450	YES
RMS Force	0.000000	0.000300	YES
Maximum Displacement	0.000017	0.001800	YES
RMS Displacement	0.000003	0.001200	YES

FILE: Yb_Sq2_PhYbOTf3_GS_Anion_B3LYP_M062x_Add12_Conf6-1-_Yout

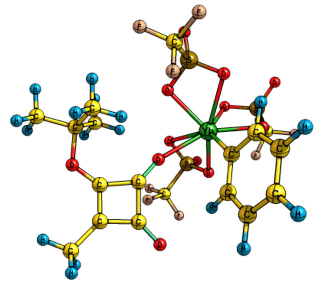

Conformer 25

E(RB3LYP/6-31G(d)/MWB59) = -3731.45550366
Convg = 0.8621D-08 -V/T = 2.0122

Temperature 195.150 Kelvin. Pressure 1.00000 Atm.
elec. and zero-point Energies= -3731.084286
elec. and thermal Energies= -3731.060219
elec. and thermal Enthalpies= -3731.059601
elec. and thermal Free Energies= -3731.132067

RB3LYP/6-31G(d)/MWB59/THF(IEFPCM) = -3731.52446902

RM06-2x/6-311+G(d,p)/MWB59/THF(IEFPCM) = -3731.36212067

Geometry at RB3LYP/6-31G(d)/MWB59
Charge = -1 Multiplicity = 1
Yb 0.3907681589 2.3976329839 0.7315180437
O 2.6360859118 1.4897187501 0.6337577499
S 3.2859639903 2.5402525074 -0.2380340406
O 4.552329524 3.0974992128 0.2186165302
O 2.1900134293 3.4743152648 -0.6465702783
C 3.7198989154 1.6196226961 -1.8002571209
F 4.7283641623 0.7624443551 -1.5572249034
F 2.6746041946 0.9199036053 -2.2577052634
F 4.1114011724 2.4807436916 -2.743262707
O -0.4400368 4.6352998845 0.3590420364
O 1.1560970873 6.5247785731 0.9435264026
O 1.1398345376 4.2808636341 2.1334410157
S 0.3993391566 5.3585210534 1.3829628317
C -0.8344658284 6.0467699189 2.6028586288
F -1.5131325683 7.046526684 2.0197447211
F -1.7006222882 5.1191388264 3.0045597121
F -0.1845237895 6.5328214456 3.667351912
C 0.2530292127 -2.6088083099 0.8479638159
C -1.0115222116 -3.16892917 0.7930639782
C -1.6926130059 -1.8622793607 0.691452515
C -0.2882343862 -1.2010924323 0.7432093544
O 0.1879594538 -0.0862408552 0.7320499925
O -2.8370551946 -1.484046997 0.6175862347
O 1.400382789 -3.2290585039 0.9733451743
C 2.7192564445 -2.5198444315 1.0024625819
C -1.5181277219 -4.5613556262 0.8444332884
H -0.7013271426 -5.2846453654 0.9227089083
H -2.1083993303 -4.7864359603 -0.0529190628
H -2.1905341465 -4.6910755466 1.7021625851
C 2.9073968919 -1.787825646 -0.322694529
H 3.9080346829 -1.348778081 -0.361189665
H 2.8024747107 -2.4832572062 -1.1631527333
H 2.197045165 -0.9684753192 -0.435351181
C 3.6987318568 -3.6856883562 1.1409793245
H 4.7231363943 -3.3013823326 1.1716925067
H 3.5104538389 -4.244477105 2.0636266832
H 3.6108223232 -4.3721606745 0.2923071039
C 2.7713392215 -1.6002046182 2.2210295377
H 2.0657361902 -0.7714000336 2.1504207877
H 2.57573672 -2.1686643467 3.1371241133
H 3.7706695042 -1.1594450198 2.2966406563
C -0.7598761259 1.9957247057 -1.337629199
C -0.3711472045 2.6230552866 -2.5445924229
C -1.9254999495 1.2001430748 -1.4243937022
H 0.5041055103 3.2713634885 -2.5518072598
H -2.3059678309 0.6926368936 -0.5383253819
C -1.0722669135 2.4601986689 -3.744535314
C -2.6453069509 1.0314811443 -2.6135114347
H -0.7299207824 2.9634971388 -4.6485858425
H -3.5411748238 0.4111518815 -2.6232744529
C -2.2158373477 1.6590859172 -3.7844048688
H -2.7678875866 1.5309153685 -4.7145274425
O -0.7572093943 2.7497896016 4.7308957104
S -0.6130561909 1.8353709382 3.6036982229
C -1.4961295325 0.3003988411 4.1918545804
O 0.7536382701 1.3858702419 3.2100183576
O -1.3430409733 2.2086146715 2.3282673315
F -1.320511126 -0.7346611027 3.3484522159
F -2.8050474174 0.5325420718 4.3095464989
F -1.0088582376 -0.0608300733 5.3866442783

Frequency: 8.5077; 17.5091; 20.5442; 21.3420; 26.1874; 27.3403; 28.3665; 37.1065; 40.6474; 42.9388; 44.8582; 51.3507; 53.0649; 56.5451; 58.2236; 61.1535; 61.9762; 71.7032; 74.8904; 78.0348; 78.9721; 83.8403; 86.4223; 89.0581; 94.9099; 122.3796; 127.1495; 132.7841; 135.6378; 146.3728; 147.6814; 164.9191; 167.6687; 178.9052; 186.5623; 189.8769; 206.1822; 208.0176; 209.8174; 215.7349; 220.5113; 223.0806; 225.0003; 232.3952; 255.3883; 264.2140; 275.8600; 291.5543; 303.2883; 306.5213; 308.5262; 322.1603; 324.5706; 325.1612; 327.5355; 348.3549; 351.0066; 353.6296; 359.1855; 393.7295; 396.3294; 450.4318; 454.7982; 476.2336; 487.6168; 489.0464; 489.9275; 491.4190; 521.6611; 523.2969; 528.8867; 555.0954; 557.5392; 557.6988; 566.5627; 570.0776; 574.3037; 596.7548; 602.0634; 621.2618; 624.3309; 633.5178; 634.8974; 640.3427; 649.3631; 720.3013; 731.9732; 736.6872; 757.6558; 758.0744; 761.5679; 781.3203; 788.1321; 848.0141; 871.1328; 916.9192; 937.8331; 940.7047; 959.1771; 970.2334; 973.0471; 977.2185; 979.6142; 983.3224; 986.1023; 989.9463; 1011.4600; 1033.0408; 1056.9479; 1063.4360; 1064.8762; 1082.1365; 1083.5751; 1087.0472; 1103.0572; 1118.9895; 1133.3753; 1183.0045; 1197.0741; 1204.4192; 1206.9893; 1209.3339; 1221.3450; 1223.6971; 1225.6279; 1230.7781; 1241.8812; 1244.5394; 1247.9908; 1254.9411; 1293.1238; 1294.1675; 1300.0794; 1306.1897; 1308.2303; 1318.0002; 1370.6307; 1397.0603; 1431.7990; 1440.4229; 1445.7842; 1457.9827; 1463.4828; 1502.7782; 1503.2243; 1506.6984; 1514.3739; 1515.0695; 1523.1893; 1530.1836; 1533.9803; 1548.3577; 1615.2938; 1626.1310; 1651.2936; 1828.2630; 1884.8663; 3044.6935; 3058.5171; 3066.1508; 3071.7562; 3095.8770; 3129.0142; 3131.4986; 3137.1427; 3137.3700; 3137.5418; 3146.0352; 3146.5167; 3159.8720; 3166.1636; 3175.6329; 3184.0982; 3188.1840.

Cartesian Forces: Max 0.000001062 RMS 0.000000494

Item	Value	Threshold	Converged?
Maximum Force	0.000001	0.000450	YES
RMS Force	0.000000	0.000300	YES
Maximum Displacement	0.000082	0.001800	YES
RMS Displacement	0.000012	0.001200	YES

FILE: Yb_Sq2_PhYbOTf3_GS_Anion_B3LYP_M062x_Add12_Conf7.out

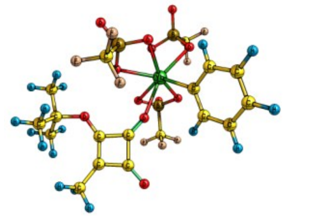

Conformer 26

E(RB3LYP/6-31G(d)/MWB59) = -3731.45003598
Convg = 0.5810D-08 -V/T = 2.0122

Temperature 195.150 Kelvin. Pressure 1.00000 Atm.
elec. and zero-point Energies= -3731.078731
elec. and thermal Energies= -3731.054707
elec. and thermal Enthalpies= -3731.054089
elec. and thermal Free Energies= -3731.126910

RB3LYP/6-31G(d)/MWB59/THF(IEFPCM) = -3731.52217862

RM06-2x/6-311+G(d,p)/MWB59/THF(IEFPCM) = -3731.35706240

Geometry at RB3LYP/6-31G(d)/MWB59
Charge = -1 Multiplicity = 1
Yb 0.1460401625 1.9295324558 0.6932863215
O 2.0233672046 0.3483661408 0.6349831457
S 2.7810615792 0.9556980637 -0.513460497
O 4.2287846513 1.0715635958 -0.3861082421
O 1.997759184 2.1536155446 -0.9562212589
C 2.5373600148 -0.2751424865 -1.8941412834
F 3.255142403 -1.3934045433 -1.6461211767
F 1.2501787063 -0.6274572978 -2.0075673507
F 2.9503375703 0.235638567 -3.0558090581
O 0.3270485012 4.3457915431 0.4457387279
O 2.6036626001 5.3493756402 0.9528991312
O 1.7243557634 3.238094063 2.0560314286
S 1.4563559879 4.5762308917 1.4146214552
C 0.716649257 5.6155609103 2.7744777326
F 0.3825051746 6.8168825439 2.2818501527
F -0.3690478127 5.0446405843 3.2927562222
F 1.6243579857 5.787135037 3.7454068782
C -0.0378001163 -2.6390635886 0.7825930803
C -1.055714541 -3.5821079022 0.6742926466
C -2.091617929 -2.5466899924 0.4232202716
C -0.9742491669 -1.487750139 0.5380682858
O -0.8758690728 -0.2844885632 0.472740785
O -3.2820661191 -2.5699332295 0.2188734415
O 1.239344692 -2.5094262976 1.0093941296
C 2.2118545339 -3.5990837041 1.2408667178
C -1.2824353585 -5.0493794247 0.761206066
H -1.3268642989 -5.3827620912 1.8068126908
H -0.5089457755 -5.6387853796 0.2590984272
H -2.2519947843 -5.2794235663 0.3060331737
C 1.7798704308 -4.4343191645 2.4477892568
H 2.5752357183 -5.1447825561 2.6983258182
H 1.613574942 -3.7883997468 3.3156417841
H 0.8704393353 -5.008889091 2.2573335004
C 3.4990660625 -2.8299358238 1.5452467038
H 4.3129291558 -3.5358596317 1.7439245786
H 3.7757751933 -2.1953876862 0.701324558
H 3.363242782 -2.1871237078 2.4192134738
C 2.3485215499 -4.4110758501 -0.0492506726
H 1.428424927 -4.9477655207 -0.298854093
H 2.602403617 -3.7474210203 -0.8803510204
H 3.1492234878 -5.1503845811 0.0627227843
C -1.2816171893 2.1599437837 -1.2293393246
C -0.89332545 2.9847678245 -2.3110336485
C -2.5631562465 1.5753634847 -1.3527285753
H 0.0780377761 3.4772445773 -2.2838490028
H -2.935470584 0.9215890484 -0.563706732
C -1.7079922061 3.2090460452 -3.4263861443
C -3.3961112767 1.7924083793 -2.4566717525
H -1.3616389969 3.8529492795 -4.2346732417
H -4.3777025013 1.3207358985 -2.500709848
C -2.9683278515 2.6115871302 -3.5034067653
H -3.609114729 2.7833840605 -4.367308185
O -1.1245035299 2.2700709896 4.7311466935
S -1.2261099719 1.6242953826 3.4272864002
C -2.7093729088 0.5050694313 3.5911026296
O -0.1068056462 0.7330052071 2.9881814461
O -1.568921492 2.501563665 2.2451734418
F -2.9991054151 -0.1030331184 2.4334244219
F -3.7745188258 1.2121928564 3.9806334075
F -2.4560509365 -0.4381746715 4.5121963586

Frequency: 8.4738; 12.7670; 17.9440; 23.0238; 25.7255; 25.8083; 29.3196; 31.5330; 32.9522; 38.3734; 40.7707; 45.3585; 46.8115; 51.0114; 56.6096; 61.7817; 66.6395; 68.8662; 73.6201; 81.2197; 83.5010; 90.2521; 96.5663; 104.2431; 119.9141; 124.1181; 128.9195; 137.8107; 143.4927; 148.7528; 151.0217; 163.3886; 170.3586; 180.5082; 187.5576; 197.9026; 205.8577; 209.4935; 214.3104; 215.6520; 217.0617; 220.6810; 226.2985; 232.4193; 247.2726; 251.9325; 265.3577; 295.2640; 302.5045; 305.0175; 308.1883; 322.0827; 322.6101; 324.4012; 335.9049; 349.8460; 352.0882; 352.2039; 357.3382; 394.3791; 396.4494; 448.3086; 451.3105; 480.9434; 483.6715; 488.9765; 489.2444; 491.0000; 522.2865; 523.4997; 528.7128; 555.3177; 557.6202; 557.9933; 569.1342; 571.1411; 575.9382; 598.9022; 611.1614; 617.0461; 624.9321; 632.7692; 634.7864; 646.4417; 649.2267; 715.1411; 735.3330; 754.4913; 758.5730; 761.7321; 764.0289; 780.4922; 788.4200; 844.6121; 866.4363; 910.7601; 935.2207; 937.2176; 972.5201; 974.8860; 975.1550; 977.1501; 982.6584; 983.0681; 986.5912; 998.0942; 1011.8126; 1032.5171; 1052.9835; 1062.0674; 1067.9802; 1084.0631; 1087.3713; 1094.3733; 1108.8047; 1123.5039; 1141.2030; 1182.9693; 1184.7178; 1196.7388; 1211.8228; 1212.2815; 1214.9870; 1222.5654; 1225.5157; 1226.5494; 1230.1574; 1239.4685; 1243.7483; 1253.3500; 1285.6423; 1295.3385; 1295.9884; 1304.9086; 1307.7610; 1318.2619; 1369.8662; 1389.4938; 1430.6654; 1435.4473; 1441.0940; 1458.6407; 1461.5016; 1500.7073; 1505.7435; 1513.3052; 1518.6694; 1519.4839; 1522.1979; 1529.3280; 1531.9645; 1550.3105; 1614.4711; 1625.7204; 1647.8040; 1850.3064; 1887.3767; 3041.8509; 3066.3972; 3067.4200; 3075.5313; 3101.0808; 3129.0950; 3132.4457; 3133.6424; 3136.1755; 3141.7555; 3145.7187; 3153.8493; 3156.0481; 3160.7551; 3163.5242; 3174.1284; 3176.8136.

Cartesian Forces: Max 0.000004552 RMS 0.000000925

Item	Value	Threshold	Converged?
Maximum Force	0.000008	0.000450	YES
RMS Force	0.000001	0.000300	YES
Maximum Displacement	0.001138	0.001800	YES
RMS Displacement	0.000194	0.001200	YES

FILE: Yb_Sq2_PhYbOTf3_GS_Anion_B3LYP_M062x_Add12_Conf7-.out

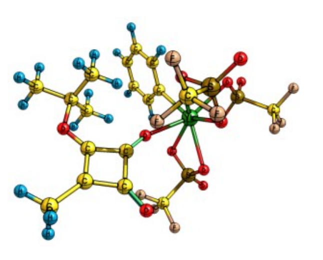

Conformer 27

E(RB3LYP/6-31G(d)/MWB59) = -3731.45972172
Convg = 0.4541D-09 -V/T = 2.0122

Temperature 195.150 Kelvin. Pressure 1.00000 Atm.
elec. and zero-point Energies= -3731.088564
elec. and thermal Energies= -3731.064509
elec. and thermal Enthalpies= -3731.063891
elec. and thermal Free Energies= -3731.136420

RB3LYP/6-31G(d)/MWB59/THF(IEFPCM) = -3731.52785328

RM06-2x/6-311+G(d,p)/MWB59/THF(IEFPCM) = -3731.36936874

Geometry at RB3LYP/6-31G(d)/MWB59
Charge = -1 Multiplicity = 1
Yb 0.3847759632 2.4233736926 0.4832920819
O -1.5354231787 2.6188239273 2.0507583788
S -0.7334194254 2.7977679101 3.3076674348
O -1.0051155668 3.9700472169 4.1315016664
O 0.6965843782 2.506649003 2.9372507916
C -1.2163052513 1.3705516002 4.4123568413
F -2.5436662036 1.3073606047 4.5330311634
F -0.7710163893 0.194896385 3.9311515651
F -0.6768803185 1.5436885375 5.6265885037
O 0.6230392149 4.0749726848 -1.2786243171
O 1.6249142609 6.3206353038 -0.6424598492
O 0.4883935194 4.765234734 1.0134886492
S 0.6236096879 5.2928271625 -0.3930803737
C -1.0191662782 6.1065621277 -0.726187291
F -1.076064746 6.5043509138 -2.002241106
F -2.0183558543 5.2529893172 -0.4901123225
F -1.1620537238 7.1748085368 0.0673315831
C 0.1731735974 -2.4277307419 1.3901516726
C -1.0681231407 -2.9076024236 1.776291395
C -1.701503258 -1.5711041681 1.7511008747
C -0.3223419829 -1.0055406766 1.3295788123
O 0.1669296523 0.0726170494 1.0619778823
O -2.8028096648 -1.1244883298 1.9575709065
O 1.2742187125 -3.1065710391 1.1921518265
C 2.5565588727 -2.484932062 0.714827675
C -1.5900137599 -4.2595230165 2.0876899832
H -0.8257873112 -5.0292428248 1.9466370488
H -2.4526556812 -4.4903371991 1.4499822609
H -1.9476074867 -4.301204285 3.124704859
C 2.3285955276 -1.8788414166 -0.6675962539
H 3.2790290775 -1.495422794 -1.0512714771
H 1.9494737217 -2.6366673316 -1.3626731968
H 1.6405305922 -1.0323113895 -0.6425088031
C 3.4819345282 -3.6998386063 0.6467030624
H 4.4715600316 -3.3846497373 0.301564137
H 3.59013364 -4.1630855419 1.6330736855
H 3.0927656124 -4.4489053422 -0.0509546668
C 3.0396448952 -1.4727330506 1.748817667
H 2.3831109076 -0.6040103962 1.8102415565
H 3.1247004743 -1.9428088375 2.7353075643
H 4.0238179976 -1.0933873787 1.4565689915
C 2.7667991592 2.0406346163 0.5097793724
C 3.5594986761 2.1740282543 1.6739227049
C 3.4727090664 1.6479486469 -0.6523510982
H 3.0882036177 2.4840357872 2.605401
H 2.9318100257 1.5373169605 -1.5916089645
C 4.9369737257 1.9210085438 1.6903424687
C 4.8490809506 1.388698782 -0.6566853177
H 5.503034345 2.0407567284 2.6141331195
H 5.347190663 1.0947239122 -1.580910097
C 5.589388439 1.519641942 0.5218555845
H 6.6611458298 1.3252936962 0.5257850858
O -1.1293904439 2.0185688785 -3.4200618161
S -0.9327707445 1.4054258818 -2.1114421352
C -1.7089422097 -0.2791011876 -2.3136490001
O -1.63287396 2.0125870903 -0.9306984767
O 0.4764314541 1.1168233928 -1.6702003571
F -1.5638961574 -1.0370404926 -1.2088358433
F -1.1260555471 -0.9258811781 -3.3323959856
F -3.0142735166 -0.1570256565 -2.5688959993

Frequency: 8.6303; 12.7832; 18.1141; 21.9429; 25.3897; 29.4688; 32.8963; 35.8699; 38.7141; 43.7844; 44.2288; 46.9765; 52.5858; 55.8130; 56.4385; 62.7753; 65.3562; 73.2598; 74.9945; 76.3116; 78.3907; 82.3925; 92.0973; 99.5521; 104.9724; 119.0222; 131.3415; 137.9657; 141.4174; 143.0239; 144.6800; 164.1843; 176.5000; 178.7950; 186.2216; 188.4539; 204.6537; 210.1104; 211.9078; 214.1729; 217.9786; 218.7084; 220.8335; 226.4029; 251.4972; 266.0869; 271.6413; 289.3970; 303.9463; 305.6578; 307.0607; 319.9211; 324.0588; 325.4552; 327.8915; 347.4238; 347.9279; 350.0722; 358.5889; 394.1986; 396.3790; 449.3060; 452.6755; 477.5188; 486.5692; 488.2914; 490.9858; 491.3798; 520.9930; 523.2512; 529.6714; 554.6744; 556.1401; 558.2865; 567.0355; 569.1433; 577.2693; 595.7422; 601.5057; 623.3313; 623.6177; 633.7175; 634.1991; 644.4552; 648.0802; 720.7413; 731.3798; 739.4826; 757.0930; 758.0162; 762.0034; 781.7384; 787.3710; 843.2092; 872.4508; 917.1918; 937.0409; 940.4019; 958.7701; 976.3868; 977.4923; 983.2242; 984.6872; 985.7278; 987.2048; 988.0326; 1011.1163; 1032.8161; 1056.1577; 1062.3956; 1066.3525; 1083.6927; 1084.4181; 1084.8913; 1111.4998; 1118.7972; 1132.5781; 1182.4244; 1193.3839; 1201.4396; 1207.5936; 1214.0276; 1218.9266; 1221.2580; 1226.4793; 1239.0694; 1241.0662; 1243.6882; 1245.4945; 1246.2824; 1293.5648; 1294.6395; 1299.1485; 1305.2962; 1308.6313; 1318.8580; 1371.2584; 1398.5849; 1431.4141; 1440.6466; 1446.5890; 1457.4693; 1463.4166; 1500.7813; 1502.7654; 1506.4441; 1513.2225; 1514.3869; 1521.0794; 1528.3105; 1529.0434; 1547.3797; 1611.4948; 1623.2048; 1649.9060; 1829.4645; 1893.2648; 3044.5297; 3057.7800; 3063.1369; 3068.2644; 3095.8334; 3128.8349; 3133.3376; 3133.7354; 3134.4866; 3137.8009; 3142.3954; 3143.6226; 3161.6679; 3168.3425; 3175.7203; 3177.0331; 3181.7606.

Cartesian Forces: Max 0.000000630 RMS 0.000000291

Item	Value	Threshold	Converged?
Maximum Force	0.000001	0.000450	YES
RMS Force	0.000000	0.000300	YES
Maximum Displacement	0.000008	0.001800	YES
RMS Displacement	0.000002	0.001200	YES

FILE: Yb_Sq2_PhYbOTf3_GS_Anion_B3LYP_M062x_Add12_Conf7-1_Y.out

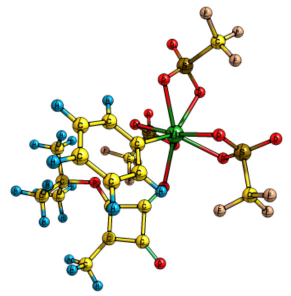

Conformer 28

E(RB3LYP/6-31G(d)/MWB59) = -3731.45491638
Convg = 0.4635D-08 -V/T = 2.0122

Temperature 195.150 Kelvin. Pressure 1.00000 Atm.
elec. and zero-point Energies= -3731.083093
elec. and thermal Energies= -3731.059457
elec. and thermal Enthalpies= -3731.058839
elec. and thermal Free Energies= -3731.128950

RB3LYP/6-31G(d)/MWB59/THF(IEFPCM) = -3731.52379884

RM06-2x/6-311+G(d,p)/MWB59/THF(IEFPCM) = -3731.36155503

Geometry at RB3LYP/6-31G(d)/MWB59
Charge = -1 Multiplicity = 1
Yb -0.1715420491 1.9781416113 0.4263579594
O -1.6631769421 1.966888238 2.5276959692
S -0.5264297266 1.7875304234 3.4785167233
O -0.3734845699 2.722866454 4.5860224789
O 0.7000044393 1.509242635 2.640634762
C -0.8774283119 0.1667159479 4.3389148796
F -1.8628548071 0.3297808784 5.2268571054
F -1.2467098537 -0.7974627621 3.478883975
F 0.2193320172 -0.2604410567 4.9948318175
O 1.8004398458 3.4134072233 0.3541264492
O 1.8362867885 5.3730298613 1.9659935614
O -0.2972703238 4.3454086843 1.0470729913
S 1.1636534387 4.674335942 0.8773637683
C 1.2241311192 5.8671304293 -0.5542478718
F 2.5023642075 6.1649658986 -0.8277119677
F 0.6587890899 5.349787807 -1.6433409445
F 0.5814572219 6.9938622633 -0.2212881546
C -0.0623175831 -2.2527805661 1.1363060767
C -0.6742327089 -3.4169594321 0.6749072852
C -1.7849067774 -2.6707481452 0.0353127705
C -1.1071636737 -1.3734876965 0.5181466111
O -1.3011877395 -0.17764999 0.4354499497
O -2.7770398074 -2.983392449 -0.5812510463
O 0.9475524579 -1.8131288988 1.8263495426
C 1.9856401906 -2.6308853288 2.4989223093
C -0.4767475983 -4.8902226022 0.6607103709
H 0.2878573656 -5.1767972155 -0.0735510261
H -1.416394871 -5.3661257786 0.3597469158
H -0.1705740884 -5.2951581542 1.6304459792
C 1.3206173233 -3.4111714466 3.6355553291
H 2.08596182 -3.9405242873 4.2137732969
H 0.7938663739 -2.7240432829 4.3032292287
H 0.6071099555 -4.1536717129 3.2654805479
C 2.9335431142 -1.56234245 3.0461523227
H 3.7442493603 -2.0407360874 3.6069345942
H 3.3579651415 -0.9806674193 2.2241158559
H 2.396000499 -0.8782526811 3.7070116896
C 2.6937331619 -3.5160239657 1.4726404745
H 2.0532828104 -4.3202803768 1.1017652613
H 3.0356321695 -2.9135852961 0.6252409661
H 3.5673935142 -3.980446569 1.9438955879
C 1.4737082948 0.48713999 -0.5941301988
C 2.8297007521 0.5273979757 -0.1920530952
C 1.1926744752 -0.4428458702 -1.6214276049
H 3.1361473708 1.2415038582 0.5717356239
H 0.1801070403 -0.512125332 -2.0229410273
C 3.8168344986 -0.2996737934 -0.7427368089
C 2.1620533374 -1.2815614019 -2.1881184675
H 4.8490971363 -0.2201874468 -0.4009457767
H 1.888378933 -1.9731247272 -2.9851867591
C 3.4849809796 -1.2180107947 -1.744561295
H 4.2488695432 -1.8564098222 -2.1862669602
O -2.6554341855 4.0368884537 -2.3287470229
S -2.0793808091 2.8181547613 -1.7739395943
C -2.7447957588 1.447470999 -2.8471173462
O -2.4583222127 2.425086644 -0.3752579842
O -0.5826828826 2.6465734452 -1.8585889506
F -2.2748543903 0.2571903771 -2.4499461994
F -2.3796310694 1.6488061269 -4.1190591181
F -4.0804604318 1.4255423294 -2.7783975216

Frequency: 13.6267; 17.0847; 23.4918; 30.0147; 31.8200; 34.7287; 35.1361; 40.4535; 45.7636; 49.4593; 49.6866; 53.8335; 54.2848; 55.2127; 64.0715; 68.9658; 73.2357; 76.5938; 77.3686; 80.5114; 87.0595; 93.5668; 105.6985; 109.5031; 125.2549; 132.1844; 135.3763; 138.6006; 144.2905; 150.9711; 157.2998; 163.5099; 174.4547; 178.9970; 181.9781; 205.4073; 208.3981; 210.9620; 211.1879; 214.1734; 217.0621; 218.3775; 223.6359; 230.0797; 249.4467; 255.2446; 275.0070; 299.3503; 302.0188; 304.6371; 307.4839; 321.2875; 322.2177; 323.3439; 338.6782; 349.5305; 352.8333; 354.2801; 358.0880; 395.2775; 398.6481; 452.6599; 457.0332; 482.3554; 484.2616; 488.8440; 489.3236; 490.5933; 522.3162; 525.4084; 530.1345; 555.5652; 556.8231; 558.1215; 567.0497; 573.2232; 576.3062; 599.2421; 611.2046; 618.8741; 625.6669; 634.1123; 636.7718; 644.2083; 647.0068; 722.2524; 736.0741; 754.8715; 759.7694; 762.2415; 765.5712; 782.1713; 787.5085; 842.8556; 871.4421; 908.5082; 936.8244; 942.0234; 974.8904; 976.8708; 978.2315; 979.8183; 982.7835; 984.8793; 989.0024; 999.5231; 1011.3607; 1030.0386; 1052.6168; 1065.7117; 1074.0239; 1082.3347; 1084.2405; 1106.3260; 1108.7077; 1116.6061; 1140.8597; 1181.7454; 1192.5163; 1200.9270; 1211.7149; 1212.2638; 1215.0860; 1216.7334; 1225.4561; 1229.0647; 1232.3477; 1233.5281; 1239.3775; 1254.4982; 1288.2999; 1294.1180; 1297.1220; 1305.1171; 1309.9829; 1320.6766; 1371.2725; 1393.9957; 1432.7013; 1437.6903; 1441.6308; 1457.0479; 1463.2580; 1501.8308; 1506.5170; 1514.1075; 1519.8170; 1520.8052; 1522.0741; 1530.8625; 1533.2586; 1551.9756; 1610.0427; 1620.7217; 1645.6605; 1832.2202; 1884.9865; 3043.5480; 3062.7961; 3065.6704; 3072.9899; 3102.7356; 3128.0247; 3128.2933; 3130.7565; 3135.1363; 3136.9224; 3146.7459; 3149.1997; 3155.6165; 3157.4227; 3160.8513; 3170.2172; 3172.6903.

Cartesian Forces: Max 0.000019337 RMS 0.000003429

Item	Value	Threshold	Converged?
Maximum Force	0.000013	0.000450	YES
RMS Force	0.000002	0.000300	YES
Maximum Displacement	0.001196	0.001800	YES
RMS Displacement	0.000192	0.001200	YES

FILE: Yb_Sq2_PhYbOTf3_GS_Anion_B3LYP_M062x_Add12_Conf7-1-.out

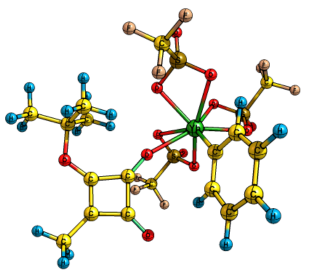

Conformer 29

E(RB3LYP/6-31G(d)/MWB59) = -3731.45438580
Convg = 0.6205D-08 -V/T = 2.0122

Temperature 195.150 Kelvin. Pressure 1.00000 Atm.
elec. and zero-point Energies= -3731.083175
elec. and thermal Energies= -3731.059093
elec. and thermal Enthalpies= -3731.058475
elec. and thermal Free Energies= -3731.131079

RB3LYP/6-31G(d)/MWB59/THF(IEFPCM) = -3731.52389765

RM06-2x/6-311+G(d,p)/MWB59/THF(IEFPCM) = -3731.36072526

Geometry at RB3LYP/6-31G(d)/MWB59
Charge = -1 Multiplicity = 1
Yb 0.3103914821 2.4406585202 0.5756176675
O 2.6033264632 1.6063554598 0.5634082688
S 3.2091682531 2.6241986301 -0.3719073102
O 4.4365204812 3.2842416338 0.0550715157
O 2.0725037003 3.4705117374 -0.856546016
C 3.7138593178 1.6284003253 -1.8645164555
F 4.7430033828 0.8226998757 -1.5464264178
F 2.7045692154 0.8681085522 -2.3057571822
F 4.1009327791 2.4462879152 -2.8477477287
O 0.9730681117 4.4355109228 1.8756218249
O -0.9253915773 6.0742440603 2.2731698672
O -0.7753517628 4.567734658 0.2345455048
S -0.0871541449 5.3534789193 1.3231883903
C 0.8373584066 6.7073550594 0.429567687
F 1.6286673633 7.3483902739 1.3005886204
F 1.5840404401 6.2359855899 -0.5666832934
F -0.0472355059 7.5815765943 -0.0719568331
C 0.2326642651 -2.5490317574 0.8410189821
C -1.0297544082 -3.1140647429 0.7902932395
C -1.7135064271 -1.8113799721 0.6544346648
C -0.3105346403 -1.1471450122 0.6935518711
O 0.1660400101 -0.032808803 0.6504055093
O -2.858547316 -1.4375529438 0.5716105728
O 1.3814371852 -3.1611888172 0.9932180377
C 2.6947564532 -2.4433262119 1.0407746779
C -1.5338941499 -4.5057864726 0.8733268857
H -0.719116482 -5.2219597022 1.0136046488
H -2.0834734453 -4.7682329467 -0.0397958843
H -2.2430360502 -4.6049787565 1.7049275656
C 2.9103755308 -1.7333150969 -0.2921068187
H 3.9078677969 -1.2856282681 -0.313222816
H 2.8327501355 -2.4439933596 -1.1226880073
H 2.1952598652 -0.9233758837 -0.437034798
C 3.6775517597 -3.600260471 1.2235282075
H 4.6988471171 -3.2092845367 1.270062735
H 3.4715303432 -4.1427182991 2.1521036526
H 3.6127618398 -4.3031412518 0.3862813283
C 2.713243849 -1.5011537918 2.2432193735
H 2.0072610248 -0.6756957374 2.1414289897
H 2.4970067762 -2.0533420968 3.1646240074
H 3.7094278692 -1.0562796305 2.3356298771
C -0.7852645204 1.9252759606 -1.5001850642
C -0.3585396182 2.4308093423 -2.7502142595
C -1.9801021626 1.1695757919 -1.5365976863
H 0.543956564 3.0384665989 -2.8004861987
H -2.3883271522 0.750455714 -0.6170141293
C -1.0526040754 2.1939807308 -3.9421517142
C -2.6923341228 0.9272324289 -2.7173049644
H -0.6801146067 2.604322356 -4.8807336397
H -3.6106675274 0.3415230904 -2.6874920118
C -2.2262730487 1.4370057663 -3.9307915075
H -2.7725217944 1.251315966 -4.854583331
O -0.7706805993 2.8565852663 4.6011087599
S -0.623452667 1.9286066234 3.4870932466
C -1.4483674636 0.3790490055 4.1221382464
O 0.7476309758 1.5096732836 3.071662177
O -1.3878251452 2.2562254711 2.219393335
F -1.2547272196 -0.6704078678 3.2984724454
F -2.7617998395 0.5714451619 4.2572152843
F -0.9301774826 0.0579737262 5.3154217831

Frequency: 9.8156; 17.8619; 19.3904; 19.9205; 21.7135; 28.2320; 30.2510; 34.9136; 39.6112; 41.1432; 45.8300; 46.3295; 51.9538; 54.3562; 57.3394; 61.7214; 64.4688; 71.6795; 73.7803; 76.8176; 80.7062; 81.9963; 87.9397; 91.3379; 97.4759; 122.5726; 132.5559; 133.3264; 137.4074; 145.0541; 149.9733; 160.5150; 169.7855; 177.3757; 188.0305; 190.0905; 205.8353; 208.9721; 209.5526; 216.5389; 217.8437; 220.6266; 223.6073; 232.0705; 255.7177; 264.6215; 276.0747; 291.6506; 302.8353; 306.3206; 308.4201; 321.7335; 323.4434; 325.8563; 327.7798; 348.9729; 350.7734; 352.1564; 358.8879; 393.8871; 396.9864; 452.1763; 454.8092; 476.4332; 487.6748; 489.5377; 489.9774; 492.1659; 520.8193; 523.8139; 529.3940; 554.8591; 557.7710; 557.9121; 565.1807; 570.7681; 574.2405; 597.6365; 601.4347; 619.4458; 625.2949; 634.0446; 636.1372; 641.1555; 649.1636; 720.8068; 732.1328; 736.9991; 757.3014; 757.9822; 761.4790; 781.2325; 788.3287; 848.5994; 871.0868; 916.3539; 937.7816; 940.7947; 959.6130; 969.1889; 975.0379; 977.2520; 980.1215; 984.9378; 986.7221; 988.9000; 1011.3842; 1032.9478; 1056.9118; 1063.7196; 1065.3967; 1082.6685; 1083.7050; 1087.2922; 1102.3862; 1120.4430; 1133.9442; 1183.0943; 1196.3077; 1202.5513; 1207.3964; 1209.4971; 1221.5296; 1224.0053; 1226.0440; 1229.4834; 1239.9474; 1245.0080; 1247.4341; 1255.9149; 1292.8084; 1293.7916; 1299.9558; 1303.5343; 1310.6776; 1320.4220; 1370.9950; 1397.0689; 1432.0413; 1440.6869; 1445.7087; 1458.0235; 1463.7900; 1502.9827; 1503.1640; 1506.6887; 1514.7256; 1515.1909; 1523.3007; 1530.9510; 1534.3139; 1548.8083; 1615.2253; 1626.1717; 1651.4793; 1827.2185; 1885.3785; 3044.6614; 3058.4734; 3065.7852; 3071.6567; 3095.8932; 3128.4819; 3131.2663; 3136.7170; 3137.0092; 3137.4219; 3144.9090; 3145.8522; 3158.5149; 3165.8383; 3175.6529; 3182.5337; 3188.9723.

Cartesian Forces: Max 0.000001069 RMS 0.000000446

Item	Value	Threshold	Converged?
Maximum Force	0.000000	0.000450	YES
RMS Force	0.000000	0.000300	YES
Maximum Displacement	0.000041	0.001800	YES
RMS Displacement	0.000008	0.001200	YES

FILE: Yb_Sq2_PhYbOTf3_GS_Anion_B3LYP_M062x_Add12_Conf8.out

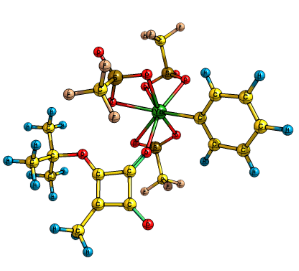

Conformer 30

E(RB3LYP/6-31G(d)/MWB59) = -3731.44967331
Convg = 0.6302D-08 -V/T = 2.0122

Temperature 195.150 Kelvin. Pressure 1.00000 Atm.
elec. and zero-point Energies= -3731.078562
elec. and thermal Energies= -3731.054426
elec. and thermal Enthalpies= -3731.053808
elec. and thermal Free Energies= -3731.127204

RB3LYP/6-31G(d)/MWB59/THF(IEFPCM) = -3731.52140969

RM06-2x/6-311+G(d,p)/MWB59/THF(IEFPCM) = -3731.35582578

Geometry at RB3LYP/6-31G(d)/MWB59
Charge = -1 Multiplicity = 1
Yb 0.0452271083 1.9173607463 0.3946357654
O 2.0892686787 0.4492789271 0.4321187735
S 2.7098317219 0.9931069029 -0.8182844477
O 4.1296101269 1.3267648691 -0.7942642197
O 1.7513801209 2.0146948247 -1.3629620666
C 2.5991973753 -0.4210554662 -2.0284052727
F 3.3922034391 -1.4335296621 -1.6166783922
F 1.3465945773 -0.8884185878 -2.1233817001
F 3.0061658227 -0.0356387764 -3.2401122768
O 1.5551397321 3.1177041666 1.9268376439
O 0.8325015729 5.4751378471 2.5373150322
O 0.2375810841 4.3461136785 0.3411717259
S 1.1853270226 4.5149879393 1.4982430025
C 2.7449304453 5.2007295784 0.7414277756
F 3.6922358233 5.3023272251 1.6836612931
F 3.1945547277 4.4165081333 -0.2393719565
F 2.5013965086 6.4206119609 0.2431444588
C 0.0260772628 -2.5539450269 0.6190260074
C -0.9286778879 -3.5614429841 0.5123602491
C -1.9492509135 -2.6404658631 -0.0503788185
C -0.9088189496 -1.5069799347 0.0823962255
O -0.8582432259 -0.3140480003 -0.1170013151
O -3.0870980651 -2.7702388385 -0.434687069
O 1.2512222106 -2.3186453978 0.9913387749
C 2.223854578 -3.3021215489 1.513207743
C -1.114913816 -5.0076661287 0.8036238507
H -0.2583345467 -5.6231301851 0.5114591295
H -2.0022235202 -5.3598519824 0.2662831555
H -1.2933891584 -5.1756731368 1.8741853928
C 1.6644902952 -3.962019594 2.7748095839
H 2.4433933155 -4.5787851921 3.2364701956
H 1.3596017009 -3.1979008291 3.4965187781
H 0.8095128986 -4.6095327548 2.5654522907
C 3.4308829679 -2.4230002102 1.8461093223
H 4.2364797989 -3.0420831897 2.2560327124
H 3.7905670726 -1.9136990182 0.9498690838
H 3.1573570091 -1.6584213052 2.5778754377
C 2.560030051 -4.29359407 0.3965690905
H 1.7045241615 -4.9196889741 0.1266212815
H 2.8903740377 -3.7509593589 -0.4937985795
H 3.3708643621 -4.9546674118 0.7216723954
C -1.4845959299 2.3740803494 -1.3936868517
C -1.117421619 3.1562025443 -2.5120343964
C -2.8257152177 1.9283548992 -1.3890677942
H -0.0998271295 3.5406364968 -2.583422038
H -3.1872732347 1.3164569763 -0.5614793113
C -2.0071482686 3.4696071467 -3.5457675249
C -3.7337203926 2.2346423614 -2.4091022616
H -1.6744460738 4.0756512181 -4.3885159207
H -4.7589259721 1.8684825958 -2.3564485744
C -3.3247467601 3.0084764029 -3.4973523623
H -4.0240189629 3.2496133986 -4.2968958074
O -1.2750855535 1.798697849 4.4544150851
S -1.3735080331 1.3620421229 3.0673596871
C -2.9048859322 0.2990985632 3.0252092805
O -0.2893259551 0.4824840031 2.5209270734
O -1.6430290742 2.4132720212 2.0188485099
F -3.1396586398 -0.1640778343 1.7879168819
F -3.9675011384 0.9992186567 3.4295567944
F -2.7424462394 -0.7535928595 3.8429680372

Frequency: 5.6707; 15.1796; 17.2012; 21.0674; 23.6749; 26.1874; 31.0400; 31.8438; 35.4297; 38.2577; 39.8334; 41.7722; 43.4201; 53.9455; 55.7749; 57.7127; 63.6000; 68.7894; 74.4830; 76.0483; 79.0037; 88.8516; 91.9220; 98.6822; 119.8694; 121.4980; 125.5024; 138.7802; 142.6247; 144.2024; 149.8515; 161.8997; 171.2221; 174.2183; 187.3216; 197.4736; 205.4432; 209.3048; 211.2118; 212.9604; 215.9899; 217.6241; 223.3527; 229.8832; 246.7129; 252.1275; 264.8496; 295.1463; 302.0458; 304.5833; 307.3720; 321.6719; 322.6426; 323.9766; 336.4149; 349.9583; 351.1693; 351.4098; 357.1199; 392.1273; 396.3430; 450.5347; 452.8581; 481.1840; 485.0002; 488.3998; 489.4553; 490.7269; 521.9987; 523.4517; 528.7430; 555.7452; 557.2737; 557.6628; 568.7826; 571.6439; 576.0595; 597.5675; 610.9656; 618.9476; 623.8175; 633.8743; 635.0013; 646.8720; 650.1351; 714.5223; 735.7961; 754.8355; 757.6170; 761.1129; 764.6282; 780.7885; 789.1896; 845.0267; 865.4961; 908.0957; 935.3253; 938.0150; 971.2118; 974.7658; 975.9619; 979.5422; 981.0750; 983.6425; 987.6054; 997.6999; 1012.4090; 1035.0583; 1052.7032; 1062.6197; 1068.9925; 1085.7306; 1086.6133; 1097.3964; 1110.0584; 1122.4693; 1143.4639; 1182.8077; 1189.8068; 1197.8781; 1212.3034; 1213.8776; 1215.0095; 1218.3412; 1222.9522; 1224.8040; 1231.8743; 1240.9784; 1243.4994; 1246.7676; 1285.6899; 1295.3747; 1297.3189; 1305.3865; 1309.1792; 1319.0479; 1370.1802; 1391.4382; 1430.4521; 1435.5672; 1441.4295; 1459.0630; 1461.6502; 1500.3359; 1505.2995; 1512.9287; 1517.2190; 1519.7351; 1523.3322; 1529.3188; 1531.0354; 1549.6371; 1615.2458; 1626.6823; 1648.7659; 1845.1240; 1888.5230; 3042.7838; 3066.0510; 3066.9946; 3075.1053; 3101.8436; 3128.9991; 3130.7792; 3133.3828; 3136.6465; 3136.8183; 3147.6458; 3149.6494; 3152.9803; 3157.3196; 3158.4209; 3172.7206; 3173.4202.

Cartesian Forces: Max 0.000000984 RMS 0.000000400

Item	Value	Threshold	Converged?
Maximum Force	0.000000	0.000450	YES
RMS Force	0.000000	0.000300	YES
Maximum Displacement	0.000138	0.001800	YES
RMS Displacement	0.000038	0.001200	YES

FILE: Yb_Sq2_PhYbOTf3_GS_Anion_B3LYP_M062x_Add12_Conf8-.out

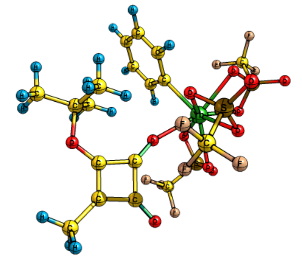

Conformer 31

E(RB3LYP/6-31G(d)/MWB59) = -3731.45931287
Convg = 0.1722D-08 -V/T = 2.0122

Temperature 195.150 Kelvin. Pressure 1.00000 Atm.
elec. and zero-point Energies= -3731.088210
elec. and thermal Energies= -3731.064197
elec. and thermal Enthalpies= -3731.063579
elec. and thermal Free Energies= -3731.135447

RB3LYP/6-31G(d)/MWB59/THF(IEFPCM) = -3731.52672129

RM06-2x/6-311+G(d,p)/MWB59/THF(IEFPCM) = -3731.36695792

Geometry at RB3LYP/6-31G(d)/MWB59
Charge = -1 Multiplicity = 1
Yb 0.2663577175 2.4932080745 0.679702148
O -1.6800786641 2.3284488825 2.2163625012
S -0.948906195 2.7131299496 3.4690951898
O -1.4855535962 3.8050103228 4.2722612303
O 0.5124107209 2.7596001489 3.1008336855
C -1.077205922 1.232498605 4.6001686468
F -2.3549715637 0.892577266 4.7751552286
F -0.4125725644 0.1709936346 4.1054990341
F -0.5390783103 1.532733523 5.7898913033
O -0.1925734025 4.815363087 1.0071297356
O -0.4770226995 6.2991123621 -1.0343656385
O 0.8659211585 4.1422705005 -1.0385051067
S 0.3441485419 5.3446039322 -0.3018606982
C 1.8677958305 6.2962924405 0.1939824884
F 1.5095966022 7.3899727607 0.8788360501
F 2.6687131321 5.5514524086 0.9577466294
F 2.5372983761 6.6737326631 -0.9024722937
C 0.227299116 -2.4328449485 1.3667240051
C -0.9652237944 -2.9500009546 1.8460322385
C -1.6012259569 -1.6220796166 1.9824905153
C -0.2806761955 -1.0132601707 1.4432121397
O 0.169659934 0.0830383577 1.1833058184
O -2.6709392251 -1.2035282724 2.3509915372
O 1.3042464643 -3.089295162 1.0172706543
C 2.5539395676 -2.4286305057 0.5086862839
C -1.4457131327 -4.3249779429 2.1216340702
H -0.6650497385 -5.0690855405 1.9391187846
H -2.3125552862 -4.5593571113 1.4902084018
H -1.7848714161 -4.4094167827 3.1617906815
C 2.2413791388 -1.7223561073 -0.8077090354
H 3.1679937214 -1.3134785103 -1.2229729503
H 1.8158858319 -2.4264689824 -1.5317928919
H 1.5593484754 -0.880653738 -0.6779898191
C 3.4727104692 -3.6316278162 0.2942433697
H 4.4385883803 -3.2883912374 -0.0893410799
H 3.643224461 -4.164922626 1.2354203504
H 3.0395481818 -4.329612849 -0.429952909
C 3.0993771657 -1.4934740043 1.5831533579
H 2.4525351928 -0.6297197236 1.7426091485
H 3.2375728613 -2.0326240923 2.527465094
H 4.0676574806 -1.0948728679 1.2648067744
C 2.6522237727 2.1408560078 0.820426212
C 3.4038829285 2.1539212574 2.0182212414
C 3.3996308554 1.8708061952 -0.3508895905
H 2.8991974323 2.3679246615 2.9595083123
H 2.8917981446 1.8575611941 -1.315372647
C 4.7823692567 1.9076443222 2.055524718
C 4.7769814089 1.6182107249 -0.3355149418
H 5.3165233387 1.9339226851 3.005450239
H 5.307729675 1.4214618529 -1.2673301014
C 5.4769026174 1.6325267257 0.8748454467
H 6.5496092002 1.4445243228 0.8952553248
O -1.0360427504 2.0789447901 -3.3477432522
S -0.9939942279 1.5865460235 -1.9765991855
C -1.9682357302 -0.0020538794 -2.04192845
O -1.6660174144 2.3884268629 -0.9018272229
O 0.3442690016 1.1680631685 -1.4237105894
F -2.1077864806 -0.5417887928 -0.8183916164
F -1.3363246257 -0.8932178896 -2.8209954046
F -3.1853205066 0.2230671616 -2.5439908733

Frequency: 13.5953; 15.9300; 19.0277; 26.3463; 26.8168; 30.5999; 32.4338; 36.2516; 40.4262; 42.1576; 45.5031; 50.4661; 53.2637; 56.1417; 59.6563; 61.4977; 65.9079; 70.2308; 73.2170; 75.9431; 78.5958; 81.5957; 87.6773; 95.4691; 106.3257; 121.9191; 130.4530; 134.8871; 140.6144; 145.2611; 156.4472; 167.3049; 169.5846; 179.1206; 186.8302; 187.8475; 203.0612; 208.3770; 211.9356; 214.2183; 217.0465; 221.4902; 222.9450; 226.2004; 252.0317; 265.9771; 270.3461; 290.0663; 303.2353; 306.2073; 307.5903; 321.2377; 323.3677; 324.9066; 328.2903; 345.9748; 349.4799; 351.1325; 359.0169; 393.7442; 395.8390; 450.7593; 452.7708; 477.2162; 487.8029; 489.0694; 490.5727; 490.8396; 521.4896; 523.7000; 529.6534; 555.9418; 556.5536; 558.2985; 568.1951; 569.4711; 576.3272; 596.5817; 599.3065; 620.2170; 624.2488; 633.8454; 634.5070; 641.7417; 647.8844; 720.7862; 731.0362; 738.4371; 757.1793; 757.8675; 761.6118; 781.9818; 787.5617; 843.4060; 872.0100; 914.0667; 935.9311; 940.2003; 958.0253; 975.2675; 976.3949; 978.8094; 982.3395; 984.5640; 986.1648; 987.1373; 1010.8776; 1032.6425; 1056.2582; 1060.7093; 1065.3550; 1082.7278; 1083.3833; 1084.3776; 1111.1086; 1118.3049; 1130.3873; 1182.2115; 1193.7133; 1203.9218; 1212.0180; 1214.1380; 1218.2496; 1219.3376; 1225.5658; 1235.3484; 1241.0552; 1245.1204; 1246.2946; 1247.1163; 1293.5649; 1294.6900; 1298.3824; 1308.0250; 1309.2043; 1320.0539; 1370.4856; 1397.9033; 1431.2980; 1439.6235; 1446.3121; 1457.4956; 1462.6066; 1500.1011; 1502.9059; 1506.4685; 1514.4849; 1514.7489; 1520.4744; 1528.3317; 1529.4971; 1548.2864; 1611.1203; 1622.6365; 1649.9133; 1828.8788; 1891.5014; 3044.4027; 3057.5712; 3062.6198; 3067.8059; 3095.5719; 3128.3407; 3131.6801; 3133.0231; 3133.5246; 3137.5635; 3141.5563; 3141.9421; 3156.6926; 3165.0543; 3173.7569; 3177.6006; 3180.7739.

Cartesian Forces: Max 0.000001129 RMS 0.000000410

Item	Value	Threshold	Converged?
Maximum Force	0.000000	0.000450	YES
RMS Force	0.000000	0.000300	YES
Maximum Displacement	0.000043	0.001800	YES
RMS Displacement	0.000004	0.001200	YES

FILE: Yb_Sq2_PhYbOTf3_GS_Anion_B3LYP_M062x_Add12_Conf8-1.out

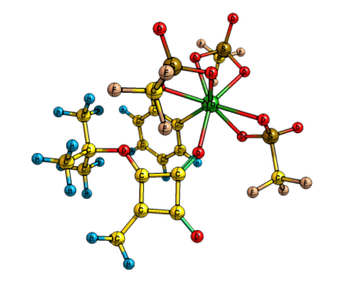

Conformer 32

E(RB3LYP/6-31G(d)/MWB59) = -3731.45544576
Convg = 0.3901D-08 -V/T = 2.0122

Temperature 195.150 Kelvin. Pressure 1.00000 Atm.
elec. and zero-point Energies= -3731.083903
elec. and thermal Energies= -3731.060079
elec. and thermal Enthalpies= -3731.059461
elec. and thermal Free Energies= -3731.131045

RB3LYP/6-31G(d)/MWB59/THF(IEFPCM) = -3731.52410889

RM06-2x/6-311+G(d,p)/MWB59/THF(IEFPCM) = -3731.36331374

Geometry at RB3LYP/6-31G(d)/MWB59
Charge = -1 Multiplicity = 1
Yb 0.1021910937 2.2193316299 0.7494114386
O -1.983088188 3.02837805 1.8428221946
S -1.3504377093 3.3889058392 3.1567686858
O -1.539457017 4.7439488149 3.6623865438
O 0.0605989984 2.8545813512 3.09886322
C -2.2026055729 2.3223096612 4.4299050841
F -3.5100096345 2.6159199371 4.4502341495
F -2.0593106757 1.0203353714 4.1757816368
F -1.6846899161 2.5806033429 5.6395486281
O 0.3204782373 4.6334174124 0.6662800632
O 1.0165304119 5.7193100271 -1.5222148018
O 1.4967393345 3.2960684258 -0.9368916849
S 1.2749028558 4.7193323013 -0.4948519932
C 2.8899278412 5.2205738773 0.2867599072
F 2.801416707 6.4767666973 0.7393898265
F 3.1893438343 4.4123280024 1.3065930046
F 3.8713589634 5.1569648508 -0.6222196601
C -0.0397557239 -2.0998434495 1.2927808476
C -0.4581180269 -2.9966556705 2.274310895
C -1.2749352028 -1.9731454317 2.9709649515
C -0.7905423537 -0.9599231387 1.9115644028
O -0.9218401604 0.2226730779 1.6748543308
O -1.9986616869 -1.9798602338 3.9390615058
O 0.6687764524 -2.0019921505 0.2088294574
C 1.3925755256 -3.0944684153 -0.4809081205
C -0.2769200623 -4.4126373289 2.688798333
H -0.3473750257 -5.1194136631 1.8559014123
H -1.0454556762 -4.6611355034 3.4287446347
H 0.7010190837 -4.5611451165 3.1656133559
C 2.4281493828 -3.7019860694 0.4663138203
H 3.0682598463 -4.3917767094 -0.0952524368
H 3.057218752 -2.9134843755 0.8916711978
H 1.9680765488 -4.2675929617 1.2809378207
C 2.0716028127 -2.3543036157 -1.634174344
H 2.6330851446 -3.0664376654 -2.2492045591
H 1.3260253912 -1.8505284035 -2.2535267165
H 2.7541450388 -1.5954636181 -1.2437674274
C 0.3625575884 -4.0981301723 -1.0049538407
H -0.1537187906 -4.624518559 -0.196619996
H -0.3826088776 -3.58108524 -1.6167067862
H 0.8634714828 -4.848073039 -1.6269435286
C 2.1150391979 1.0645471797 1.4514313075
C 2.3182050696 0.5569225143 2.7549646108
C 3.1500956094 0.7743418811 0.5314657432
H 1.5798806062 0.7679267861 3.5295487385
H 3.0681894547 1.14031962 -0.4917314997
C 3.4425690552 -0.1972919086 3.116089373
C 4.288354049 0.0327352369 0.8726796686
H 3.551069415 -0.563461321 4.1372013072
H 5.0651452846 -0.1506649619 0.1297852585
C 4.4363408893 -0.4659499386 2.171365493
H 5.3213253827 -1.0382448359 2.4466462474
O -0.7712007054 1.4525775173 -3.3548264542
S -0.9000201485 1.1054996168 -1.9440487885
C -2.0244227253 -0.3838864309 -1.9640149699
O -1.5873800053 2.0895784917 -1.0367750231
O 0.3155649242 0.6252258548 -1.2133428028
F -2.2950049853 -0.8286430757 -0.7265412433
F -1.4262583289 -1.3859883191 -2.6413225399
F -3.1788827131 -0.0947235942 -2.5704744065

Frequency: 6.7286; 17.7122; 21.3218; 25.2512; 27.4510; 30.1353; 33.1402; 34.6876; 37.2669; 43.4981; 46.5340; 48.1541; 54.1361; 58.2534; 59.9553; 64.9552; 67.2393; 70.7621; 72.6088; 84.3688; 90.8943; 92.6810; 98.4641; 109.3495; 121.3992; 127.2473; 129.7367; 139.6303; 143.8263; 148.2835; 150.7577; 164.6635; 168.8209; 176.8709; 186.0587; 204.8579; 207.9974; 211.3105; 214.4708; 214.8505; 217.4035; 220.6126; 224.2501; 228.6136; 251.2474; 257.8226; 271.9218; 299.4278; 302.4839; 306.0725; 306.6810; 320.7553; 324.0609; 324.6747; 339.7767; 349.0025; 351.5361; 351.9327; 358.3066; 394.6473; 398.2211; 449.9094; 452.7302; 482.2298; 485.2133; 488.2231; 489.4842; 491.0958; 521.3388; 523.8902; 529.6775; 555.7375; 557.6723; 557.9231; 568.1657; 569.8747; 577.8433; 598.1733; 612.2974; 619.6147; 624.7099; 633.8276; 635.2188; 646.0895; 647.8871; 719.6421; 735.1874; 754.1442; 760.5763; 761.6824; 765.4249; 782.0159; 787.5763; 843.6275; 870.2850; 907.8700; 937.2504; 941.1183; 974.1246; 975.8958; 978.2453; 978.8827; 982.8662; 986.8916; 987.7635; 997.9864; 1010.8933; 1031.4431; 1052.5812; 1064.7615; 1073.1547; 1081.9745; 1083.2629; 1105.3729; 1112.0652; 1115.8115; 1138.5238; 1181.4140; 1191.1627; 1200.5038; 1211.9759; 1212.7436; 1214.9232; 1219.2327; 1223.3320; 1229.8741; 1236.7658; 1238.1947; 1242.1142; 1245.2310; 1287.7331; 1295.6185; 1296.4511; 1304.9059; 1307.8150; 1320.0208; 1367.9194; 1395.4651; 1431.8936; 1437.3176; 1442.3055; 1457.4377; 1463.1342; 1500.4573; 1505.7947; 1513.9075; 1517.1626; 1518.9407; 1521.9729; 1530.2550; 1532.1645; 1551.5136; 1610.2093; 1620.8560; 1647.7483; 1837.4188; 1886.9369; 3043.9649; 3062.3481; 3064.6645; 3072.1334; 3103.1403; 3128.6425; 3130.5186; 3131.0506; 3135.3944; 3138.0073; 3146.6340; 3150.8455; 3151.5965; 3154.0554; 3160.2313; 3169.3908; 3171.6472.

Cartesian Forces: Max 0.000011452 RMS 0.000002384

Item	Value	Threshold	Converged?
Maximum Force	0.000011	0.000450	YES
RMS Force	0.000001	0.000300	YES
Maximum Displacement	0.001090	0.001800	YES
RMS Displacement	0.000162	0.001200	YES

FILE: Yb_Sq2_PhYbOTf3_GS_Anion_B3LYP_M062x_Add12_Conf8-1-.out

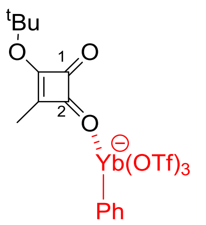

Pre-reaction complex 1b•[PhYb(OTf)$_3$]$^-$:- chelation to C2 carbonyl only

Lowest energy conformation is: Conformer 14

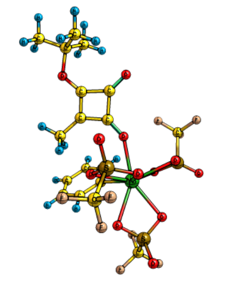

Conformer 1

E(RB3LYP/6-31G(d)/MWB59) = -3731.46899830
Convg = 0.3436D-09 -V/T = 2.0122

Temperature 195.150 Kelvin. Pressure 1.00000 Atm.
elec. and zero-point Energies= -3731.097254
elec. and thermal Energies= -3731.073420
elec. and thermal Enthalpies= -3731.072802
elec. and thermal Free Energies= -3731.145895

RB3LYP/6-31G(d)/MWB59/THF(IEFPCM) = -3731.53568523

RM06-2x/6-311+G(d,p)/MWB59/THF(IEFPCM) = -3731.37323648

Geometry at RB3LYP/6-31G(d)/MWB59
Charge = -1 Multiplicity = 1
Yb 0.3542165448 2.7768700064 1.3924204912
O 1.9310431778 2.0597051886 -0.5089358103
S 3.1937072842 2.3639525952 0.2431269149
O 4.1696695552 1.2778852023 0.3507015509
O 2.788541787 3.05209503 1.5173811198
C 4.0610271818 3.6776438031 -0.7529306055
F 4.354400498 3.1886572397 -1.9676653014
F 3.3027440629 4.7618125885 -0.8951655722
F 5.2025627314 4.0202666709 -0.1414023563
O 0.3867209671 4.6595065278 -0.151452572
O 0.8200211938 6.9790804969 0.7793896767
O -0.0607357724 5.0475304352 2.1734351538
S 0.0537620589 5.7407110999 0.8429672156
C -1.6978688541 6.2222512722 0.4316656291
F -1.7537292049 6.7034997574 -0.8169169593
F -2.5092335097 5.1660217293 0.5268603445
F -2.1168959032 7.1700580026 1.2785365904
C 1.6365167655 -2.4465158056 0.5641186409
C 1.1334716454 -1.2590743894 0.0439857831
C 1.2094854048 -0.5923216284 1.3464939655
C 1.7440303565 -1.8922442041 1.9660520899
O 2.0811696412 -2.2915214285 3.0545644204
O 0.9744851885 0.513420469 1.81334293
O 1.875360085 -3.5689906332 -0.0743475187
C 2.4483663522 -4.7619552908 0.6002778996
C 0.7017862635 -0.8559252759 -1.3111207344
H 0.9297493553 -1.6388930615 -2.0408640063
H 1.1937700248 0.0817750903 -1.5883014393
H -0.3753854249 -0.6489719751 -1.3197201239
C 3.8263287631 -4.41148816 1.165682985
H 4.3079828734 -5.3224731137 1.5385349323
H 4.4645163467 -3.9814781118 0.3866872818
H 3.7480417758 -3.7077704104 1.9979371559
C 2.5534348921 -5.7640132011 -0.5500226482
H 2.9675990926 -6.7090647088 -0.1839469828
H 1.5673507199 -5.960538629 -0.9829530473
H 3.2077657248 -5.3794683561 -1.3387907583
C 1.4775303114 -5.2465600228 1.6792261328
H 1.4114611875 -4.5311009661 2.5026534779
H 0.4805170157 -5.4081686022 1.2556015859
H 1.8335832371 -6.1992235688 2.0873487933
C -1.2971009655 1.8582174993 -0.1155914555
C -1.2906624037 2.1655848922 -1.4965828659
C -2.2612019994 0.9011347784 0.2773485499
H -0.5781049627 2.8989233596 -1.8734777365
H -2.3453359071 0.6316802847 1.3305217702
C -2.1574348407 1.5632938434 -2.4162469063
C -3.1336464594 0.2803024887 -0.6263558703
H -2.1119503993 1.8355623886 -3.4707313405
H -3.8584735288 -0.453108842 -0.2723191646
C -3.0819562805 0.608079058 -1.9840049009
H -3.7581337761 0.1327646998 -2.6932497978
O -1.1744594159 3.4555944342 5.2776327194
S -0.6464617768 2.5452834762 4.2674435593
C -0.7858376686 0.8637467925 5.0664201432
O 0.7912970272 2.6796862228 3.8689535534
O -1.4286437184 2.4011755251 2.9827694285
F -0.4789192106 -0.1212663834 4.2141148533
F -2.0431886726 0.6743311639 5.4930645505
F 0.0396856315 0.7943557381 6.1179338246

Frequency: 5.6856; 6.7765; 17.5452; 22.9729; 25.4444; 26.0661; 28.2850; 32.4852; 34.5250; 35.7920; 37.9383; 43.1873; 45.7045; 51.5421; 59.0562; 59.2685; 69.0014; 70.0759; 77.0515; 83.3976; 83.8683; 92.1977; 107.9509; 111.6569; 124.6208; 128.1852; 137.4479; 145.4960; 148.4127; 157.3278; 162.8560; 164.4535; 174.3398; 176.4336; 186.5416; 204.6706; 209.9327; 211.9638; 213.8040; 215.8894; 217.7950; 220.2380; 223.4279; 232.0806; 259.4982; 275.4455; 277.8337; 303.2031; 304.8053; 307.2931; 316.7967; 321.5126; 324.3721; 329.0076; 344.8376; 347.5684; 349.3627; 351.5764; 357.1618; 392.9308; 402.4698; 450.0223; 454.4667; 481.9644; 489.5649; 490.7466; 491.5694; 495.2357; 520.8483; 523.7257; 528.7639; 557.1627; 557.8047; 558.5513; 569.0835; 570.4301; 576.1874; 603.7659; 607.7374; 623.3362; 626.4965; 630.7297; 634.5126; 641.9662; 647.2822; 719.1949; 736.4969; 747.3378; 758.7544; 761.5513; 762.6428; 772.4048; 788.5984; 865.5638; 871.2960; 911.3951; 936.4755; 943.3330; 971.1568; 973.2859; 976.6031; 980.7010; 983.2618; 986.2930; 988.4691; 1004.9618; 1011.5718; 1032.9728; 1056.3741; 1069.1036; 1071.4964; 1082.7628; 1084.0531; 1098.7713; 1116.2732; 1119.8072; 1136.4598; 1182.1599; 1209.4412; 1212.9751; 1214.8392; 1215.2747; 1223.1110; 1229.4492; 1229.8767; 1235.9071; 1236.5085; 1241.7812; 1252.7586; 1255.4496; 1286.4778; 1289.4603; 1294.8675; 1304.7237; 1306.4606; 1315.3008; 1366.9357; 1399.7954; 1432.2645; 1434.0895; 1437.8315; 1458.2301; 1460.4081; 1491.6272; 1503.7439; 1504.1531; 1518.3876; 1518.7965; 1519.0878; 1535.7531; 1538.5833; 1555.5761; 1611.2892; 1622.4796; 1624.6192; 1815.6886; 1876.0648; 3055.3517; 3058.4283; 3059.7021; 3066.7775; 3121.8804; 3124.0489; 3126.7168; 3132.7720; 3136.9682; 3138.4329; 3140.9799; 3145.8733; 3153.6944; 3156.9527; 3160.8845; 3160.9684; 3173.7265.

Cartesian Forces: Max 0.000000520 RMS 0.000000181

Item	Value	Threshold	Converged?
Maximum Force	0.000000	0.000450	YES
RMS Force	0.000000	0.000300	YES
Maximum Displacement	0.000035	0.001800	YES
RMS Displacement	0.000007	0.001200	YES

FILE: Yb_Sq2_PhYbOTf3_GS_Anion_B3LYP_M062x_Add56_Conf1_Y.out

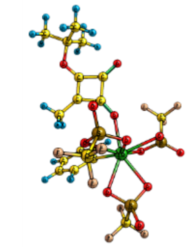

Conformer 2

E(RB3LYP/6-31G(d)/MWB59) = -3731.45910531
Convg = 0.7049D-08 -V/T = 2.0122

Temperature 195.150 Kelvin. Pressure 1.00000 Atm.
elec. and zero-point Energies= -3731.087397
elec. and thermal Energies= -3731.063594
elec. and thermal Enthalpies= -3731.062976
elec. and thermal Free Energies= -3731.135548

RB3LYP/6-31G(d)/MWB59/THF(IEFPCM) = -3731.52799243

RM06-2x/6-311+G(d,p)/MWB59/THF(IEFPCM) = -3731.36981361

Geometry at RB3LYP/6-31G(d)/MWB59
Charge = -1 Multiplicity = 1
Yb 0.3501715864 2.5119613939 1.1024005993
O 2.3076206859 2.3614184731 -0.3758993885
S 3.359588906 2.7232106573 0.6396870344
O 4.5067525672 1.8297575413 0.7653377978
O 2.6172775199 3.094632957 1.8945282373
C 4.0969214386 4.3214645892 0.0237681192
F 4.6701379814 4.1146827758 -1.1704496425
F 3.1794552796 5.2790359846 -0.0984776216
F 5.0383943014 4.7334937309 0.8856878992
O -1.6128978359 3.9853677016 1.2497606504
O -1.8036602497 5.7846726817 -0.5311669134
O 0.3950851044 4.7156095035 0.1531558942
S -1.0070588498 5.1443829327 0.5076834747
C -0.7516934943 6.4536957887 1.8081034206
F -1.9305151069 6.8082939546 2.3321389303
F 0.040718044 6.0009615147 2.7823912606
F -0.1830581597 7.5325766822 1.2536565963
C 0.9468585673 -2.8838873745 0.9704133286
C 0.0666014218 -1.8048563819 1.0505958506
C 1.1709300422 -0.8726545298 1.3454785924
C 2.1547418762 -2.0562070105 1.330661955
O 3.3201614629 -2.2752993219 1.5257880622
O 1.2859018876 0.3332093339 1.5108461547
O 0.9928649605 -4.1702370114 0.7126033354
C -0.1339404659 -5.0096106972 0.2507542098
C -1.3863606188 -1.5259810133 0.9198411494
H -2.0067838603 -2.3978372142 1.1419814059
H -1.617047194 -1.1796400588 -0.0953206728
H -1.6599922959 -0.7067755735 1.5903585248
C -1.1138469575 -5.2065576604 1.4099225303
H -1.9120014764 -5.8932199453 1.1070335628
H -0.5979490034 -5.6380469465 2.2737662637
H -1.5783050333 -4.2684121258 1.7207772282
C 0.56615344 -6.3277611983 -0.0915668624
H -0.168965148 -7.0657936369 -0.429219446
H 1.3012855662 -6.1770033846 -0.8879557209
H 1.084762732 -6.7265791864 0.785760966
C -0.771853797 -4.397914218 -0.9983487468
H -1.2645652281 -3.4444333823 -0.8016142242
H -0.0145151922 -4.2396214693 -1.7727629349
H -1.5250154906 -5.0882207083 -1.3940448417
C -0.7582713041 1.4325845238 -0.7575797188
C -0.0631947926 0.6677897084 -1.723592065
C -2.1665810363 1.4313096921 -0.8943890221
H 1.0265802477 0.6468702725 -1.7003890875
H -2.769695615 2.007694258 -0.1932534567
C -0.7110824667 -0.0662169196 -2.7258050465
C -2.8344436417 0.7090477839 -1.8911000009
H -0.1291684171 -0.6408269357 -3.4469498025
H -3.9220763209 0.740488763 -1.9535279707
C -2.1064375855 -0.0538647697 -2.8093554333
H -2.618640488 -0.6183691952 -3.587418659
O -2.1620233003 2.5761441307 4.5675862996
S -1.033162244 2.0306765233 3.8219751425
C -0.3405417933 0.712227358 4.9438849244
O 0.1359814256 2.9289700802 3.5430364053
O -1.32854404 1.2993340404 2.5399267647
F 0.765889112 0.1613688016 4.4272855029
F -1.2533389021 -0.2583829478 5.1156047185
F -0.0401206337 1.234831349 6.1375310695

Frequency: 4.9035; 11.2787; 19.3009; 22.4418; 23.4740; 25.1317; 28.1733; 28.9513; 34.4707; 36.4980; 40.3636; 50.0131; 53.6152; 61.5978; 62.6165; 65.9371; 67.5220; 70.6185; 75.5167; 80.0240; 89.0993; 96.1929; 107.7594; 115.5223; 127.3530; 131.9605; 138.8472; 143.5129; 146.0764; 149.6419; 154.5590; 170.9089; 181.2000; 183.6849; 184.3816; 192.5501; 209.6283; 212.0014; 212.5709; 213.5468; 217.0024; 219.8062; 220.7831; 236.1320; 237.9899; 261.5757; 287.7219; 294.3964; 304.3205; 305.7996; 307.2984; 321.5745; 324.3904; 326.1465; 346.4450; 348.9944; 352.2516; 353.9662; 358.4922; 393.7671; 399.0078; 449.8796; 450.4039; 483.4946; 488.7297; 490.4774; 491.2357; 491.9229; 520.4860; 524.0439; 528.9723; 557.2069; 558.3157; 558.4068; 568.3582; 570.8791; 576.4916; 599.5028; 605.6376; 622.5308; 625.3313; 631.8602; 634.1358; 646.8036; 649.1599; 721.3373; 737.6784; 757.9245; 761.3824; 761.7802; 767.3538; 779.1827; 785.1502; 848.7677; 873.5863; 914.5393; 932.4584; 935.4509; 976.6457; 976.8182; 978.5730; 979.5945; 981.3660; 984.0602; 985.6087; 1003.6959; 1011.0944; 1032.7842; 1052.0572; 1061.7561; 1064.3076; 1082.4591; 1083.1870; 1099.4252; 1112.6444; 1117.5605; 1131.3347; 1181.8910; 1202.4225; 1207.9724; 1212.2112; 1214.4918; 1214.9566; 1223.3863; 1225.7532; 1228.1757; 1235.7295; 1238.0295; 1245.1371; 1254.4963; 1284.9451; 1293.3448; 1294.0616; 1302.5389; 1306.6954; 1317.0550; 1368.1367; 1386.2655; 1431.7002; 1434.2018; 1440.6984; 1457.8257; 1459.5559; 1495.8851; 1503.9379; 1510.9021; 1516.2494; 1518.6518; 1520.7045; 1529.1156; 1531.7106; 1549.8092; 1610.2156; 1620.7020; 1621.9894; 1823.3311; 1898.6025; 3060.2685; 3061.9887; 3066.2860; 3072.8544; 3125.3503; 3129.5888; 3130.4401; 3133.0473; 3139.5037; 3142.0119; 3142.8719; 3153.6898; 3155.5913; 3161.6243; 3164.6197; 3173.4952; 3177.0379.

Cartesian Forces: Max 0.000004527 RMS 0.000000736

Item	Value	Threshold	Converged?
Maximum Force	0.000006	0.000450	YES
RMS Force	0.000001	0.000300	YES
Maximum Displacement	0.001183	0.001800	YES
RMS Displacement	0.000257	0.001200	YES

FILE: Yb_Sq2_PhYbOTf3_GS_Anion_B3LYP_M062x_Add56_Conf1-.out

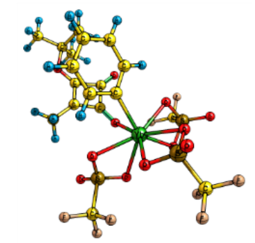

Conformer 3

E(RB3LYP/6-31G(d)/MWB59) = -3731.46890168
Convg = 0.5582D-08 -V/T = 2.0122

Temperature 195.150 Kelvin. Pressure 1.00000 Atm.
elec. and zero-point Energies= -3731.097123
elec. and thermal Energies= -3731.073312
elec. and thermal Enthalpies= -3731.072694
elec. and thermal Free Energies= -3731.145080

RB3LYP/6-31G(d)/MWB59/THF(IEFPCM) = -3731.53479014

RM06-2x/6-311+G(d,p)/MWB59/THF(IEFPCM) = -3731.37249453

GGeometry at RB3LYP/6-31G(d)/MWB59
Charge = -1 Multiplicity = 1

Yb	0.1782747345	2.435668216	-0.132526724
O	-0.5188674456	2.261099065	2.2812830357
S	-1.9996109574	2.3384792934	2.0438808131
O	-2.8112428696	1.2656908354	2.6263345632
O	-2.1979054217	2.6478646773	0.5892708142
C	-2.5632607577	3.8701156959	2.9441308079
F	-2.2901237795	3.7419979704	4.2513373932
F	-1.9585188989	4.9629391828	2.4824249514
F	-3.8879989047	4.0119522944	2.7950656953
O	1.3481990759	4.2028256541	-1.3709312708
O	2.2849023766	6.1354109561	-0.0126075773
O	0.3922193295	4.6445568868	0.7855618385
S	1.1491448578	5.2879807824	-0.3516701386
C	-0.1074438614	6.4271553409	-1.1225259616
F	0.3894092027	6.9630846305	-2.2423909073
F	-1.2249446924	5.7566149799	-1.4179596402
F	-0.4083911497	7.4135138729	-0.2673888278
C	0.9756216309	-2.4163000382	1.5027526205
C	0.2899016046	-1.3024699209	1.972250114
C	0.0190197168	-0.8250321452	0.6196661076
C	0.6976420209	-2.0814116595	0.0518189125
O	0.8959534812	-2.5841538978	-1.0269705542
O	-0.5089460448	0.1608098387	0.1183083817
O	1.5951629277	-3.337639926	2.1995143242
C	2.4150970595	-4.4009760231	1.5590108431
C	0.0010818409	-0.7731921891	3.3231231534
H	0.6190174203	0.1162391375	3.4944778025
H	0.2177200898	-1.5195589365	4.0932890355
H	-1.0404195242	-0.442143314	3.3924631815
C	1.4984234942	-5.3174994405	0.7465391377
H	2.0775668981	-6.1663369028	0.3655144307
H	0.6899745432	-5.7095559166	1.3733139443
H	1.0722824969	-4.7886195707	-0.1098166985
C	3.0050803142	-5.1242553239	2.7700892122
H	3.6306815239	-5.958483703	2.4363416323
H	3.6217276618	-4.4422075137	3.3637560848
H	2.2097978733	-5.5201391802	3.4101614191
C	3.5074553017	-3.7521388119	0.7062565721
H	3.0923319715	-3.292839662	-0.1938826133
H	4.0420532685	-2.9866259938	1.2776204658
H	4.225685427	-4.5189021205	0.3942723128
C	2.2524967979	1.5548883821	0.7693125368
C	2.6295100236	1.7342853621	2.1219400961
C	3.1509869095	0.779056299	-0.0015562293
H	1.9921795291	2.328238259	2.7775057167
H	2.9383999147	0.6131773116	-1.0570341254
C	3.7942970481	1.1822721302	2.67026395
C	4.3197824535	0.2163525422	0.5281546511
H	4.0412177617	1.3557049914	3.7177234114
H	4.9837312324	-0.3651621886	-0.1118766666
C	4.6455948012	0.4130724411	1.8728616762
H	5.5567203176	-0.0141157919	2.2898636889
O	0.1691446509	2.4772907874	-4.3683692049
S	-0.177560002	1.8384416386	-3.1035018593
C	-1.0726085738	0.2823001311	-3.6104113717
O	-1.1483286247	2.5498688696	-2.1988928228
O	0.9468562195	1.3666415725	-2.2230635503
F	-1.5395576167	-0.3870428536	-2.553672753
F	-0.2379540883	-0.5131711551	-4.292038676
F	-2.105496199	0.6017417206	-4.4039135609

Frequency: 8.5295; 13.9076; 14.9482; 20.7124; 26.2314; 27.2057; 28.6472; 30.1725; 35.1272; 36.3088; 40.7377; 42.6534; 50.8216; 52.5328; 56.3583; 60.7694; 69.2415; 71.9156; 79.5859; 80.1465; 83.5705; 93.0036; 110.8431; 116.7345; 124.8821; 134.6848; 137.4625; 141.9572; 143.5677; 145.7379; 154.4438; 174.4240; 178.6977; 181.4942; 186.7929; 208.1021; 209.4574; 213.1022; 213.8619; 214.6329; 217.3204; 218.8629; 220.0600; 241.4501; 256.5940; 267.3612; 276.5058; 299.2947; 303.9348; 307.1332; 311.2390; 321.0821; 324.9705; 328.0214; 340.5510; 347.1905; 348.5954; 351.3723; 357.8654; 396.1994; 399.7238; 452.2109; 454.8716; 475.5360; 489.3202; 491.0884; 491.7648; 494.4125; 520.6811; 523.6357; 529.1400; 557.6347; 558.0143; 558.8152; 568.7979; 570.3396; 577.4810; 594.4345; 612.9734; 621.9914; 625.6822; 631.4745; 634.0638; 640.5311; 646.0599; 722.3819; 736.6454; 746.1105; 760.0807; 761.1693; 761.7122; 773.1422; 788.0332; 861.6625; 873.2476; 915.8945; 937.4075; 943.7687; 970.3156; 977.2770; 979.5470; 981.3012; 987.3146; 988.0115; 989.4808; 1003.0835; 1010.3805; 1032.0076; 1056.6313; 1067.9012; 1072.0281; 1082.6997; 1083.8232; 1097.4647; 1113.9202; 1119.3584; 1138.8435; 1182.1036; 1208.1440; 1213.2111; 1214.2199; 1214.9913; 1225.0321; 1227.9387; 1230.3482; 1235.7304; 1242.2661; 1242.5736; 1252.8418; 1258.7615; 1282.8151; 1286.2254; 1292.4410; 1304.3627; 1305.7992; 1316.9061; 1369.8924; 1403.7383; 1431.9287; 1435.1378; 1443.7230; 1457.3837; 1461.1346; 1496.2102; 1503.5474; 1505.5407; 1516.3946; 1519.2948; 1519.7045; 1534.9154; 1537.8013; 1553.9264; 1611.0203; 1622.3479; 1625.5882; 1808.3505; 1875.4807; 3056.4768; 3056.5178; 3059.9761; 3066.7548; 3121.8619; 3122.8805; 3130.0942; 3133.1338; 3135.3078; 3139.4930; 3141.2413; 3143.4432; 3154.7565; 3155.0799; 3162.5162; 3166.0299; 3173.7327.

Cartesian Forces: Max 0.000005342 RMS 0.000000714

Item	Value	Threshold	Converged?
Maximum Force	0.000003	0.000450	YES
RMS Force	0.000000	0.000300	YES
Maximum Displacement	0.000566	0.001800	YES
RMS Displacement	0.000089	0.001200	YES

FILE: Yb_Sq2_PhYbOTf3_GS_Anion_B3LYP_M062x_Add56_Conf1-1.out

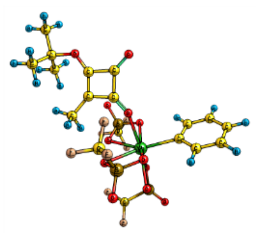

Conformer 4

E(RB3LYP/6-31G(d)/MWB59) = -3731.45921794
Convg = 0.6653D-08 -V/T = 2.0122

Temperature 195.150 Kelvin. Pressure 1.00000 Atm.
elec. and zero-point Energies= -3731.087533
elec. and thermal Energies= -3731.063578
elec. and thermal Enthalpies= -3731.062960
elec. and thermal Free Energies= -3731.135938

RB3LYP/6-31G(d)/MWB59/THF(IEFPCM) = -3731.52794108

RM06-2x/6-311+G(d,p)/MWB59/THF(IEFPCM) = -3731.36778371

Geometry at RB3LYP/6-31G(d)/MWB59
Charge = -1 Multiplicity = 1

Yb	0.9828975202	2.1122543294	0.050467109
O	1.0604066343	1.6876981169	2.5170625837
S	-0.3446747807	1.2074436024	2.7160071754
O	-0.513830676	-0.1203854237	3.3205048463
O	-1.0867930439	1.4435747012	1.432764893
C	-1.099832431	2.37301905	3.9575367617
F	-0.4486790086	2.2615458059	5.1229929606
F	-1.0423319232	3.6360879545	3.5466884373
F	-2.3848285055	2.0357238914	4.1495684625
O	0.4630232576	4.0294644271	-1.341573065
O	0.6345720544	6.3411851829	-0.3012799813
O	0.2937906412	4.2140690265	1.0444451435
S	0.1054431987	4.9858498172	-0.2348053959
C	-1.7428083525	5.1709087307	-0.3797051172
F	-2.0528887975	5.7648896774	-1.5373564739
F	-2.3381967153	3.9745537486	-0.332524207
F	-2.2036037498	5.9224470152	0.6289060936
C	0.257229384	-3.1696854975	1.2815325045
C	-0.3204217105	-2.0511730131	0.6824052156
C	0.9705372945	-1.3484876806	0.6540195464
C	1.6505734247	-2.5929241295	1.2442667272
O	2.7513262039	-2.9756456613	1.538142556
O	1.3442555368	-0.2263149732	0.340993055
O	-0.0364871346	-4.347743753	1.7786780802
C	-1.4015689251	-4.8702558279	2.0040267781
C	-1.6355598408	-1.5612733987	0.1935961264
H	-2.4810526327	-2.1533229509	0.5468394749
H	-1.6416075743	-1.5610450666	-0.9031459381
H	-1.7701916908	-0.5205291957	0.504372396
C	-2.1599090509	-3.9210558966	2.935338468
H	-3.1586161792	-4.3245880047	3.1355675527
H	-1.6303680237	-3.8228702449	3.8879946471
H	-2.2758831483	-2.9196384766	2.5163857128
C	-1.1274863113	-6.2054751948	2.701014508
H	-2.072814987	-6.7062240139	2.9351462669
H	-0.5342627947	-6.8622879682	2.0570262572
H	-0.5765472443	-6.0452890748	3.6326017549
C	-2.0869951902	-5.1026489841	0.6549475177
H	-2.2464120739	-4.1755058374	0.1015210245
H	-1.4838808038	-5.7743907696	0.0354316185
H	-3.0647685278	-5.5701377428	0.815350957
C	3.3076339645	2.5903280672	0.2355792235
C	3.999688672	2.581765661	1.468789152
C	4.0919346892	2.9170883386	-0.8951396387
H	3.4597693795	2.3370753321	2.3830166933
H	3.6270423088	2.9380939256	-1.8805703299
C	5.3642316025	2.8738257974	1.5730513421
C	5.4566648349	3.2149169917	-0.8095549522
H	5.854611443	2.8524186314	2.546204318
H	6.0189632942	3.4634619346	-1.7094612017
C	6.0999510682	3.1933192468	0.4297760547
H	7.1620832842	3.4230326293	0.5038410155
O	-0.3026616454	1.7922876426	-3.9835001626
S	0.1568206578	1.1347640809	-2.7649271408
C	0.2330462719	-0.6657464991	-3.2546524903
O	-0.7503689758	1.154453569	-1.5692790568
O	1.5390725808	1.4475825427	-2.2683939796
F	0.8056556695	-1.4263402098	-2.3060705319
F	0.928441937	-0.8123757664	-4.3858122913
F	-1.015021918	-1.1311790611	-3.4527681195

Frequency: 7.4826; 12.2602; 13.6721; 18.8029; 23.5124; 25.5351; 29.0373; 32.2052; 35.9610; 36.4030; 39.5728; 44.2222; 52.6016; 54.8541; 61.3812; 62.9945; 63.7828; 73.7598; 74.4083; 82.3503; 87.1560; 95.6549; 101.4536; 108.0007; 116.6736; 126.9160; 133.1498; 137.8367; 140.4921; 142.3811; 156.7146; 170.7687; 172.0534; 178.7168; 190.4654; 198.0823; 206.2340; 209.4564; 210.0719; 212.9580; 216.6427; 216.9240; 221.5228; 228.4679; 246.8394; 257.5951; 278.7177; 290.0843; 304.0135; 305.0651; 309.9347; 320.8049; 325.9455; 328.2210; 343.7965; 346.7659; 350.5498; 357.2598; 358.1063; 394.3245; 399.7038; 447.9117; 452.2814; 484.3408; 488.6090; 491.0516; 491.9290; 496.5404; 519.2677; 521.2137; 528.7255; 556.3654; 557.8034; 559.1330; 567.4204; 567.8087; 576.7826; 599.0387; 606.6600; 622.0625; 625.9478; 631.3288; 634.2010; 651.7037; 653.1986; 716.7189; 738.3063; 754.8765; 760.6795; 763.2509; 768.4754; 778.5110; 786.1793; 850.5583; 868.2651; 915.1770; 932.5018; 935.3071; 975.5368; 977.8176; 978.5481; 979.9216; 982.2440; 986.0144; 989.1236; 1006.7117; 1011.8823; 1036.4111; 1058.8043; 1062.9440; 1064.2741; 1086.4682; 1086.8292; 1099.6302; 1116.2769; 1128.9093; 1144.3217; 1183.6853; 1200.4465; 1208.3478; 1209.8667; 1211.8507; 1215.7031; 1215.8373; 1226.1546; 1228.8942; 1234.5579; 1238.1140; 1240.0159; 1255.9981; 1276.8647; 1285.2533; 1292.6153; 1295.9674; 1306.7196; 1318.1629; 1371.3767; 1389.0025; 1432.7020; 1435.6989; 1446.7637; 1458.8882; 1460.7153; 1500.0745; 1503.3695; 1513.2811; 1516.8302; 1523.1663; 1523.7639; 1528.5657; 1530.8887; 1549.9926; 1615.1339; 1624.3405; 1627.8929; 1823.2463; 1899.9578; 3061.7819; 3066.3661; 3066.4242; 3073.1540; 3127.1186; 3128.2227; 3132.9316; 3135.2289; 3138.6900; 3143.1363; 3143.7834; 3157.8935; 3160.4866; 3163.8624; 3168.0099; 3174.2211; 3174.5446.

Cartesian Forces: Max 0.000011547 RMS 0.000001986

Item	Value	Threshold	Converged?
Maximum Force	0.000012	0.000450	YES
RMS Force	0.000001	0.000300	YES
Maximum Displacement	0.001532	0.001800	YES
RMS Displacement	0.000374	0.001200	YES

FILE: Yb_Sq2_PhYbOTf3_GS_Anion_B3LYP_M062x_Add56_Conf1-1-.out

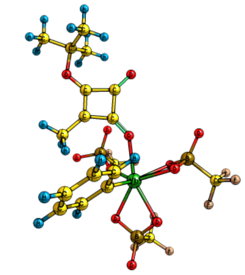

Conformer 5

E(RB3LYP/6-31G(d)/MWB59) = -3731.47061115
Convg = 0.1455D-09 -V/T = 2.0122

Temperature 195.150 Kelvin. Pressure 1.00000 Atm.
elec. and zero-point Energies= -3731.098989
elec. and thermal Energies= -3731.074960
elec. and thermal Enthalpies= -3731.074342
elec. and thermal Free Energies= -3731.148611

RB3LYP/6-31G(d)/MWB59/THF(IEFPCM) = -3731.53695024

RM06-2x/6-311+G(d,p)/MWB59/THF(IEFPCM) = -3731.37207255

Geometry at RB3LYP/6-31G(d)/MWB59
Charge = -1 Multiplicity = 1

Yb	0.3674263383	2.9646141602	0.7188437335
O	2.4399431976	3.2919482236	-0.5053617443
S	3.3181785475	3.6732261518	0.6595406052
O	4.5722380663	2.9471262856	0.8215240315
O	2.4070609371	3.7804486119	1.8551718682
C	3.847565406	5.426510986	0.3074671834
F	4.5365328667	5.4603112473	-0.8408929075
F	2.8043846472	6.2498568834	0.2102018121
F	4.6422158542	5.8531189697	1.2993195855
O	-1.7045289666	4.2048028513	1.0526430655
O	-2.1980964815	6.1603067801	-0.4955687544
O	0.1329163283	5.2274160543	-0.1086343645
S	-1.263630979	5.4851446137	0.3947357161
C	-1.0012332265	6.6404933766	1.8306622245
F	-2.1632184945	6.8807549601	2.4468767186
F	-0.1507457927	6.0897548926	2.707601728
F	-0.4895535202	7.7992571308	1.4015166363
C	1.0834488702	-2.2525757266	1.7519672959
C	0.2587378032	-1.2224257703	1.3208904768
C	1.3709649176	-0.2732709023	1.2736937329
C	2.3245263554	-1.3890464868	1.7224543143
O	3.5040012122	-1.5259086908	1.9376025879
O	1.5313553047	0.9075182778	0.991024037
O	0.74763805	-3.4880875721	2.0429513794
C	1.7287795515	-4.4790682534	2.5530757511
C	-1.1973797589	-1.1324179259	1.0664310632
H	-1.6264649375	-2.1276028314	0.9124307718
H	-1.4038395908	-0.4978646604	0.1987408986
H	-1.6957941175	-0.6741370182	1.9323838518
C	2.7985909547	-4.7265208053	1.4872030526
H	3.4474454314	-5.5496489233	1.8073895422
H	2.3359410463	-5.0074484021	0.5348574425
H	3.4233753652	-3.8417796873	1.3416936431
C	0.85560801	-5.7159145798	2.7686236162
H	1.4661730362	-6.5410465178	3.149621781
H	0.0620997279	-5.5062158989	3.4927548641
H	0.3922371439	-6.0311763832	1.8280912269
C	2.318803471	-3.973488798	3.8712901098
H	2.9580500184	-3.1016179639	3.7132822228
H	1.5215638973	-3.7115585646	4.574349093
H	2.9298657192	-4.763824184	4.3220017388
C	-0.6241116895	1.8531355152	-1.1601461909
C	0.1670684145	1.2225225763	-2.1490206848
C	-2.0224123479	1.7522065143	-1.3513606222
H	1.2541790062	1.2727105916	-2.0745475679
H	-2.6965354108	2.2267774807	-0.6375453719
C	-0.3835273924	0.5374333353	-3.2378063997
C	-2.5922018321	1.0660668829	-2.4317378534
H	0.2674331871	0.0708792474	-3.9768108589
H	-3.6755183547	1.0146861767	-2.5361364509
C	-1.7710116104	0.4531661407	-3.3805403076
H	-2.2067809458	-0.0793400834	-4.2244200039
O	0.5977972585	0.4479831917	4.0699429703
S	-0.570241667	1.3225935334	3.8854625858
C	-0.4087177726	2.6480597408	5.1781462675
O	-1.8984480848	0.7306954361	4.0867082024
O	-0.4989760366	2.1592630337	2.6208150354
F	-1.4187629964	3.5211990084	5.0836951914
F	0.7452344939	3.3088609441	5.0331444468
F	-0.4351909722	2.0939541078	6.40043736

Frequency: 8.0880; 9.0328; 13.5839; 17.9947; 20.2628; 23.1317; 24.4987; 25.7091; 29.1514; 32.6995; 37.3246; 38.9595; 40.5733; 43.9532; 48.0471; 53.3221; 60.1279; 67.8027; 76.2643; 80.8667; 84.4828; 86.2722; 104.2937; 113.5072; 118.8155; 131.2975; 138.8547; 142.2564; 148.0627; 152.0056; 159.6634; 168.0420; 178.8130; 182.7061; 189.3441; 195.0688; 210.3273; 211.6682; 212.2075; 215.3258; 217.8998; 220.0888; 221.5106; 241.5728; 257.5005; 270.6613; 276.0399; 302.8309; 304.3493; 307.3474; 315.6485; 322.0590; 324.2940; 330.6778; 342.2183; 346.6959; 347.1248; 353.4820; 358.1097; 393.2116; 402.3569; 447.3907; 454.4669; 486.3707; 486.6546; 489.7094; 490.5018; 494.9704; 507.2537; 524.3934; 528.6773; 554.9397; 557.3262; 558.0282; 569.3899; 570.4973; 577.8900; 599.5152; 607.3372; 617.3279; 622.1058; 629.7828; 633.7474; 642.9702; 649.3406; 720.5243; 736.1248; 748.0421; 760.5518; 761.0989; 761.6203; 773.6684; 789.5625; 865.6707; 873.3717; 915.1466; 936.3449; 943.1156; 972.1030; 973.1458; 974.2730; 975.5145; 987.2843; 988.1394; 993.9047; 1007.2295; 1010.8517; 1034.9766; 1062.8063; 1069.2471; 1071.3444; 1083.9031; 1084.9776; 1100.5805; 1109.3760; 1117.3859; 1157.5517; 1184.2890; 1210.1175; 1212.1968; 1214.8064; 1223.4234; 1227.9874; 1229.7978; 1231.4150; 1232.9892; 1239.2600; 1242.7028; 1254.3092; 1256.3236; 1286.3855; 1293.2091; 1296.8974; 1304.5309; 1305.7545; 1318.9639; 1368.5046; 1402.6167; 1431.7222; 1434.0255; 1449.1954; 1458.4116; 1460.3142; 1503.5221; 1505.8662; 1510.0681; 1517.6943; 1519.2413; 1520.5892; 1535.1566; 1537.9178; 1555.0020; 1613.4822; 1625.7331; 1628.6085; 1812.4655; 1877.3113; 3040.4840; 3057.2695; 3059.5619; 3066.5023; 3106.7311; 3122.5535; 3127.7341; 3135.0989; 3135.7046; 3138.7501; 3139.9878; 3141.2529; 3152.1264; 3156.7323; 3160.2063; 3162.0427; 3177.3972.

Cartesian Forces: Max 0.000001000 RMS 0.000000333

Item	Value	Threshold	Converged?
Maximum Force	0.000000	0.000450	YES
RMS Force	0.000000	0.000300	YES
Maximum Displacement	0.000030	0.001800	YES
RMS Displacement	0.000007	0.001200	YES

FILE: Yb_Sq2_PhYbOTf3_GS_Anion_B3LYP_M062x_Add56_Conf2_Y.out

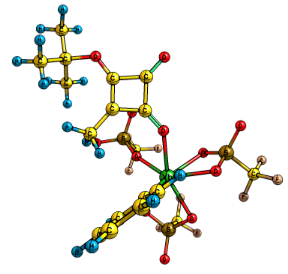

Conformer 6

E(RB3LYP/6-31G(d)/MWB59) = -3731.46229172
Convg = 0.3384D-08 -V/T = 2.0122

Temperature 195.150 Kelvin. Pressure 1.00000 Atm.
elec. and zero-point Energies= -3731.090852
elec. and thermal Energies= -3731.066784
elec. and thermal Enthalpies= -3731.066166
elec. and thermal Free Energies= -3731.139926

RB3LYP/6-31G(d)/MWB59/THF(IEFPCM) = -3731.52826227

RM06-2x/6-311+G(d,p)/MWB59/THF(IEFPCM) = -3731.36750415

Geometry at RB3LYP/6-31G(d)/MWB59
Charge = -1 Multiplicity = 1

Yb	0.4018004442	2.5249424383	0.6298230764
O	2.4461236103	2.9549223927	-0.5903707994
S	3.3800605824	3.0915036988	0.586590909
O	4.5979811889	2.2905754296	0.5735250836
O	2.5126761837	3.0368384798	1.8177911245
C	3.9891631899	4.8527950243	0.5125116341
F	4.6344068875	5.0514496938	-0.6442755305
F	2.9884976916	5.7279621809	0.6055923706
F	4.8425058509	5.0649477186	1.5242453212
O	-1.5855719855	3.7909009912	1.2547102009
O	-2.0224177178	5.9995341807	0.0732635221
O	0.2653270999	4.9023610775	0.202836777
S	-1.0972263342	5.1408834281	0.8000471047
C	-0.7251855041	6.0254951724	2.3954986666
F	-1.8530421721	6.2226764529	3.0859167455
F	0.1149128501	5.2910020942	3.1371019889
F	-0.1575852044	7.2097212503	2.143103257
C	0.8385759296	-2.6658878398	1.5400872773
C	0.081430803	-1.636719229	0.979927812
C	1.2644855604	-0.7794415926	0.8529396485
C	2.1460731877	-1.9352697377	1.3494770559
O	3.3095971474	-2.19331433	1.500995096
O	1.4646663365	0.3905030492	0.5430410502
O	0.7284861953	-3.8489914567	2.0871157158
C	-0.5241888595	-4.4675059745	2.5902176284
C	-1.3305280474	-1.3357333163	0.622898895
H	-1.8757898648	-2.2235742956	0.2901745945
H	-1.3543412995	-0.5885917159	-0.176780748
H	-1.8632308671	-0.9043416551	1.4799190077
C	-1.3027660211	-3.4677640923	3.4452305193
H	-2.0981664238	-3.9993133538	3.9796548869
H	-0.6474849839	-2.9934834851	4.1814283852
H	-1.7709201482	-2.6748936362	2.8611631591
C	0.0144464916	-5.6160375179	3.4475643631
H	-0.817601561	-6.2002119862	3.8545230646
H	0.6486497247	-6.2789507431	2.8503417807
H	0.6078129085	-5.2261870361	4.2799915
C	-1.3182930933	-5.0057709498	1.3986378062
H	-1.6599736699	-4.2028039406	0.7413997823
H	-0.7077494514	-5.6995386706	0.8112283112
H	-2.2029077558	-5.5434409756	1.7577195108
C	-0.711768473	1.8013168418	-1.3648455115
C	0.0123054545	1.2872462291	-2.4664282372
C	-2.1164760892	1.8383529043	-1.5301849001
H	1.100780688	1.2408679008	-2.4148292298
H	-2.7401633205	2.2355613091	-0.7286362589
C	-0.6072277236	0.8383169959	-3.6378453019
C	-2.7547990454	1.3886091891	-2.6929669747
H	-0.006447015	0.4543100217	-4.4618340653
H	-3.8402889209	1.4373109779	-2.7738940289
C	-1.9990340891	0.8845998301	-3.7534420829
H	-2.4881540842	0.536293731	-4.6617888199
O	0.5228847854	-0.2112082256	3.9918843814
S	-0.6270558699	0.6521305487	3.6855071031
C	-0.5882013407	2.0128595517	4.9523232217
O	-1.9661219148	0.0530512666	3.7907985912
O	-0.4546631919	1.4434641308	2.4045026438
F	-1.6171515317	2.8487760887	4.7746977103
F	0.5516405257	2.7069407733	4.8599442889
F	-0.6721249892	1.4853199955	6.1834513498

Frequency: 9.7981; 13.5059; 16.0472; 20.9553; 23.0748; 24.1765; 25.4972; 25.8747; 29.1995; 31.8136; 34.7646; 38.6089; 40.8914; 45.5252; 49.4874; 50.0649; 57.3771; 66.4430; 73.3289; 76.8826; 83.4454; 87.7986; 104.2830; 113.1852; 120.3647; 135.9182; 139.4026; 142.7275; 144.8194; 152.8094; 163.3795; 177.2749; 180.4886; 183.6508; 189.6110; 194.5897; 195.3906; 207.7347; 211.4266; 213.7671; 217.3204; 218.5433; 222.9835; 233.0278; 246.2824; 262.9385; 272.0152; 295.8053; 303.4608; 305.8425; 307.3838; 321.9696; 324.2720; 330.0382; 337.7211; 347.1875; 351.6662; 355.0278; 359.3187; 393.1102; 398.2311; 447.1289; 450.0006; 479.9731; 484.7155; 489.6525; 490.3492; 498.8938; 506.9386; 524.6787; 528.8641; 554.6469; 557.2986; 558.0357; 568.2466; 570.7196; 577.9227; 596.0564; 612.0478; 618.1582; 622.4637; 630.3001; 633.7037; 648.8549; 649.7749; 719.7362; 736.4381; 760.6076; 761.3571; 761.7155; 769.0106; 781.1233; 789.5031; 844.1251; 872.7135; 915.4050; 930.6882; 937.9153; 972.4811; 974.2520; 974.7337; 975.9854; 979.5260; 987.6634; 996.7299; 1005.7029; 1010.9309; 1035.1222; 1058.6010; 1064.6109; 1065.7145; 1084.2895; 1084.9867; 1095.5415; 1107.6768; 1116.4607; 1157.9674; 1184.3626; 1203.9002; 1211.0277; 1212.0281; 1214.0355; 1223.5330; 1226.9781; 1227.3482; 1231.7326; 1232.6852; 1241.6830; 1244.0367; 1254.3483; 1282.0302; 1290.4744; 1293.2745; 1295.0693; 1306.6323; 1319.2254; 1368.6436; 1394.1643; 1430.9042; 1434.5472; 1450.6558; 1458.5951; 1459.7108; 1501.2375; 1506.8401; 1510.8843; 1516.9199; 1519.7704; 1521.0153; 1529.1758; 1532.7799; 1546.0831; 1613.7126; 1621.6546; 1626.3963; 1812.2183; 1900.8557; 3051.2106; 3060.4509; 3065.0768; 3074.7710; 3115.7981; 3126.8993; 3135.1342; 3135.8849; 3139.0399; 3139.8373; 3140.6004; 3144.6198; 3152.3551; 3158.5673; 3160.3437; 3177.2661; 3185.8699.

Cartesian Forces: Max 0.000017587 RMS 0.000002185

Item	Value	Threshold	Converged?
Maximum Force	0.000012	0.000450	YES
RMS Force	0.000001	0.000300	YES
Maximum Displacement	0.001068	0.001800	YES
RMS Displacement	0.000201	0.001200	YES

FILE: Yb_Sq2_PhYbOTf3_GS_Anion_B3LYP_M062x_Add56_Conf2-.out

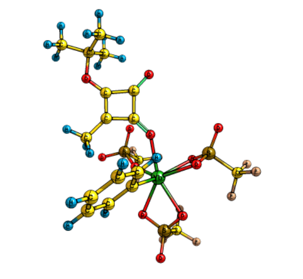

Conformer 7

E(RB3LYP/6-31G(d)/MWB59) = -3731.47061068
Convg = 0.3104D-09 -V/T = 2.0122

Temperature 195.150 Kelvin. Pressure 1.00000 Atm.
elec. and zero-point Energies= -3731.098991
elec. and thermal Energies= -3731.074960
elec. and thermal Enthalpies= -3731.074342
elec. and thermal Free Energies= -3731.148617

RB3LYP/6-31G(d)/MWB59/THF(IEFPCM) = -3731.53695874

RM06-2x/6-311+G(d,p)/MWB59/THF(IEFPCM) = -3731.37208679

Geometry at RB3LYP/6-31G(d)/MWB59
Charge = -1 Multiplicity = 1
Yb 0.3669232561 2.9648848194 0.7201261278
O 2.4432835386 3.2953941835 -0.4966056652
S 3.31724329 3.6761296498 0.6716892833
O 4.5713660956 2.950912855 0.837108283
O 2.4019941452 3.7812592914 1.864340265
C 3.846322887 5.4302452504 0.3233243558
F 4.5391470307 5.4658881756 -0.8226582795
F 2.8027739652 6.2528065992 0.2234250893
F 4.6372457582 5.8564435844 1.3183277917
O -1.707224144 4.2029327684 1.0483392994
O -2.1971526801 6.1599268114 -0.4991470578
O 0.1333054886 5.228483736 -0.105448658
S -1.2651566215 5.4844374872 0.393494556
C -1.0085772723 6.6382224668 1.8317271535
F -2.1728481389 6.8768282096 2.4442611222
F -0.1606685557 6.0870534491 2.7108922061
F -0.4963310593 7.7979104332 1.4057652588
C 1.084758799 -2.2526850237 1.750836436
C 0.2604979483 -1.2232073295 1.3172970711
C 1.3717490365 -0.2726847413 1.2751510656
C 2.3249215913 -1.3876673455 1.7266966564
O 3.5037285657 -1.5233009274 1.9462264267
O 1.5317636455 0.9085439407 0.9941127049
O 0.7493014667 -3.4887866648 2.0397032282
C 1.7296315035 -4.4789026871 2.5530694327
C -1.1947293272 -1.1346799648 1.0572892706
H -1.6220678219 -2.1302265111 0.9007885692
H -1.3985829211 -0.4996127026 0.1893593738
H -1.6969964803 -0.6777210523 1.9217167003
C 2.8038477249 -4.7244899997 1.4912063688
H 3.4524314167 -5.5470317053 1.813441895
H 2.345218977 -5.0054257039 0.5369202746
H 3.4281423167 -3.8389272585 1.3486203254
C 0.8570844759 -5.7168919974 2.7645475515
H 1.4671479403 -6.5415359443 3.1474012821
H 0.0605652657 -5.5085412172 3.4857564091
H 0.3976857045 -6.0321416207 1.8220639425
C 2.313956963 -3.9733816359 3.8738423653
H 2.95280528 -3.1006865102 3.7188034929
H 1.5136984486 -3.7127624491 4.5739528299
H 2.924178005 -4.7632681323 4.3264769164
C -0.6172752173 1.8547859055 -1.1635044217
C 0.1777416265 1.2263833057 -2.150708532
C -2.0148509866 1.7524480015 -1.3592224322
H 1.2645545704 1.2777505614 -2.0727740015
H -2.6917688792 2.225305039 -0.6469152158
C -0.368636217 0.5420450027 -3.2420892831
C -2.5804455969 1.0670263699 -2.442256156
H 0.2851848892 0.0772093498 -3.979649813
H -3.6633688631 1.0144979442 -2.5501075501
C -1.7555657592 0.4563196654 -3.3892714482
H -2.1880623239 -0.0756166695 -4.2351918392
O 0.5876452624 0.4445959045 4.0693670301
S -0.5806207468 1.3181681374 3.881422846
C -0.4256740987 2.6420925065 5.1764887176
O -1.908956561 0.7245382243 4.0766451425
O -0.50527588 2.1565987435 2.6181882268
F -1.4362423058 3.5143028499 5.079137617
F 0.7281608276 3.3042871179 5.0369982296
F -0.4564712483 2.0863374539 6.3979311628

Frequency: 8.0992; 9.0718; 13.5432; 18.0050; 20.2624; 23.0932; 24.4812; 25.6784; 29.1288; 32.6726; 37.3239; 38.9253; 40.5571; 43.9424; 48.0502; 53.3055; 60.1133; 67.7702; 76.2140; 80.8434; 84.4739; 86.2473; 104.2837; 113.5015; 118.7950; 131.2694; 138.8635; 142.2395; 148.0406; 151.9338; 159.6443; 167.8488; 178.7832; 182.6972; 189.3382; 195.0719; 210.3260; 211.6591; 212.2154; 215.3303; 217.8848; 220.0932; 221.5129; 241.5581; 257.4880; 270.6977; 276.0060; 302.8329; 304.3526; 307.3518; 315.6301; 322.0518; 324.2944; 330.6751; 342.2198; 346.7012; 347.1054; 353.4789; 358.1086; 393.2022; 402.3509; 447.3886; 454.4489; 486.3507; 486.6471; 489.7096; 490.5028; 494.9814; 507.2480; 524.3933; 528.6753; 554.9422; 557.3245; 558.0287; 569.3896; 570.4977; 577.8879; 599.5048; 607.3464; 617.3211; 622.1045; 629.7808; 633.7461; 642.9617; 649.3440; 720.5149; 736.1254; 748.0431; 760.5504; 761.1001; 761.6211; 773.6720; 789.5574; 865.6682; 873.3645; 915.1421; 936.3353; 943.1206; 972.0942; 973.1460; 974.2769; 975.5104; 987.2767; 988.1483; 993.9118; 1007.2297; 1010.8503; 1034.9767; 1062.7847; 1069.2486; 1071.3419; 1083.9060; 1084.9866; 1100.5662; 1109.3803; 1117.3924; 1157.5540; 1184.2867; 1210.1202; 1212.1978; 1214.8040; 1223.4322; 1227.9829; 1229.8009; 1231.4145; 1232.9921; 1239.2578; 1242.7035; 1254.3103; 1256.3206; 1286.3753; 1293.2077; 1296.8830; 1304.5239; 1305.7523; 1318.9658; 1368.5157; 1402.6143; 1431.7244; 1434.0320; 1449.1731; 1458.4099; 1460.3153; 1503.5279; 1505.8291; 1510.0478; 1517.7014; 1519.2411; 1520.5905; 1535.1640; 1537.9193; 1554.9934; 1613.4852; 1625.7323; 1628.6086; 1812.4710; 1877.3179; 3040.4639; 3057.2721; 3059.5625; 3066.5031; 3106.7242; 3122.5552; 3127.7346; 3135.0947; 3135.7047; 3138.7441; 3139.9749; 3141.2662; 3152.1180; 3156.7397; 3160.1991; 3162.0499; 3177.3938.

Cartesian Forces: Max 0.000000879 RMS 0.000000311

Item	Value	Threshold	Converged?
Maximum Force	0.000000	0.000450	YES
RMS Force	0.000000	0.000300	YES
Maximum Displacement	0.000188	0.001800	YES
RMS Displacement	0.000037	0.001200	YES

FILE: Yb_Sq2_PhYbOTf3_GS_Anion_B3LYP_M062x_Add56_Conf2_a.out

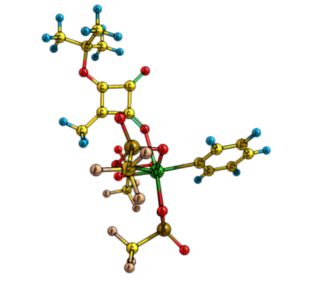

Conformer 8

E(RB3LYP/6-31G(d)/MWB59) = -3731.47132487
Convg = 0.3191D-08 -V/T = 2.0122

Temperature 195.150 Kelvin. Pressure 1.00000 Atm.
elec. and zero-point Energies= -3731.099593
elec. and thermal Energies= -3731.075629
elec. and thermal Enthalpies= -3731.075011
elec. and thermal Free Energies= -3731.148414

RB3LYP/6-31G(d)/MWB59/THF(IEFPCM) = -3731.53680159

RM06-2x/6-311+G(d,p)/MWB59/THF(IEFPCM) = -3731.37328480

Geometry at RB3LYP/6-31G(d)/MWB59
Charge = -1 Multiplicity = 1
Yb 0.9531980316 2.5181672142 -0.0441450013
O -0.1380515671 2.9490082431 2.151995691
S -1.4197608187 2.2115779528 1.9033759734
O -1.7788824825 1.1520143145 2.8525332566
O -1.4419855767 1.8310672473 0.4512465336
C -2.7673062712 3.4822577901 2.1084604481
F -2.7788605203 3.9181680884 3.3754063384
F -2.5924612812 4.5211774715 1.2948604402
F -3.9533388298 2.9151496252 1.838558305
O 1.001098678 3.5872082673 -2.2133324615
O 0.5297361991 6.0806530401 -2.337848252
O -0.2562442606 4.5951734141 -0.4330477642
S 0.1168487898 4.7604852048 -1.8812926628
C -1.4626464465 4.3939130556 -2.7982021409
F -1.2431058264 4.4353086829 -4.1184159121
F -1.9203171572 3.1795653365 -2.4749979647
F -2.3935895102 5.3042795466 -2.4868433331
C 0.5943206434 -2.5444239434 2.0120629959
C -0.0133007063 -1.5421028803 1.2662283362
C 1.0564369846 -0.5910103184 1.5675724414
C 1.7539810958 -1.662625652 2.4163011604
O 2.7498954977 -1.7605048904 3.0916462872
O 1.3340400362 0.5672383225 1.2845980126
O 0.1794242015 -3.7764452576 2.1989458928
C 0.9475165889 -4.7604260277 3.0030092457
C -1.2627533921 -1.4750829662 0.47865771
H -1.7181956227 -2.4650865386 0.3817076358
H -1.0565054882 -1.0492037316 -0.5086528017
H -1.9650551459 -0.7930633889 0.9717564052
C 2.3185283085 -4.980169706 2.3599132141
H 2.8304636758 -5.8046389673 2.8690300465
H 2.2102796301 -5.2447524898 1.3028835071
H 2.9457370697 -4.0896395658 2.4467188496
C 0.0746073531 -6.0119711045 2.9015519323
H 0.5331218623 -6.8324307196 3.4631014484
H -0.92196616 -5.8225457825 3.313120771
H -0.0348666119 -6.3229167194 1.8577132078
C 1.0493813789 -4.2639086106 4.4468999235
H 1.6819295368 -3.3755435305 4.5167328236
H 0.0556461875 -4.035140927 4.8464098392
H 1.4964896085 -5.0468949335 5.0698454718
C 2.7996282202 3.7049357886 0.8798196799
C 2.5989451221 4.8089034716 1.7405556404
C 4.1507797165 3.3749481629 0.6263746383
H 1.5827528415 5.1201048362 1.9836364902
H 4.3810857705 2.5354226811 -0.0287730131
C 3.6553663243 5.5311242514 2.3065480414
C 5.2204767807 4.0837315608 1.1849051074
H 3.450143164 6.3763818293 2.9632132563
H 6.2462060056 3.7905079494 0.9613498656
C 4.9754053624 5.1684209929 2.0297545185
H 5.80329746 5.7251040426 2.4669108864
O 2.1301004502 -1.2404606773 -1.5567726922
S 1.7899055418 0.1529056317 -1.8605624916
C 2.0632442117 0.3079343958 -3.6964959186
O 2.6583252391 1.2133340535 -1.2466568689
O 0.3635328307 0.5559483225 -1.6295851357
F 1.7838346303 1.5335717947 -4.1323474554
F 1.2740152704 -0.5704609386 -4.334367897
F 3.3406320336 0.023094496 -3.9828911679

Frequency: 6.8545; 12.2132; 13.4865; 20.1579; 23.7116; 25.6253; 27.3087; 30.8433; 32.5887; 35.8917; 36.4748; 37.4829; 45.8060; 50.6648; 53.5828; 62.8249; 69.4628; 71.6724; 76.7837; 82.8756; 85.0194; 88.7647; 98.5630; 110.2765; 117.6895; 128.5761; 135.8796; 139.7814; 142.2075; 145.7297; 153.2236; 158.0725; 173.4346; 179.3332; 190.4885; 205.2087; 205.8896; 212.2629; 214.4777; 215.9804; 217.7838; 218.2908; 221.1149; 235.3734; 258.7318; 273.7485; 276.3410; 303.4078; 306.2516; 307.9620; 313.1055; 321.4755; 326.3366; 328.5893; 343.6416; 346.4227; 347.0660; 350.1221; 356.8077; 393.5907; 401.6579; 447.3315; 454.3608; 481.9196; 490.8433; 491.0568; 492.2764; 494.9197; 518.5416; 521.9323; 528.5207; 557.7985; 558.6032; 559.1211; 566.5349; 569.6318; 576.8523; 603.2527; 606.7425; 622.5722; 625.7528; 632.4899; 634.2456; 642.1750; 651.5887; 715.4467; 737.4708; 748.7903; 760.2886; 762.2572; 762.9571; 775.2244; 788.8229; 866.1641; 866.7544; 912.7290; 936.2812; 943.1897; 971.9077; 973.1136; 979.2878; 982.9851; 985.2934; 987.5993; 989.8301; 1007.5583; 1012.0500; 1036.3634; 1061.1161; 1069.3031; 1070.5886; 1085.8305; 1086.5851; 1100.9021; 1118.1508; 1129.5348; 1143.8134; 1183.4082; 1209.9865; 1212.6010; 1213.2300; 1216.7166; 1224.9067; 1228.3066; 1229.1830; 1234.8859; 1237.5066; 1253.8049; 1255.7791; 1257.5624; 1278.1015; 1284.4509; 1286.3754; 1296.5923; 1304.5747; 1317.6662; 1369.9779; 1400.3503; 1431.9347; 1433.7226; 1442.3801; 1458.9595; 1460.2704; 1496.5882; 1503.7511; 1505.8473; 1518.3255; 1518.9302; 1522.5518; 1535.5432; 1538.0851; 1555.2991; 1615.1238; 1627.6212; 1627.8952; 1814.2384; 1876.9906; 3058.3934; 3059.1885; 3059.4652; 3066.5101; 3124.1927; 3126.2759; 3128.2317; 3134.0343; 3136.6634; 3137.8539; 3141.6602; 3146.6066; 3154.6305; 3157.2045; 3161.0110; 3163.2102; 3174.0401.

Cartesian Forces: Max 0.000005979 RMS 0.000001212

Item	Value	Threshold	Converged?
Maximum Force	0.000008	0.000450	YES
RMS Force	0.000001	0.000300	YES
Maximum Displacement	0.000851	0.001800	YES
RMS Displacement	0.000172	0.001200	YES

FILE: Yb_Sq2_PhYbOTf3_GS_Anion_B3LYP_M062x_Add56_Conf2-1.out

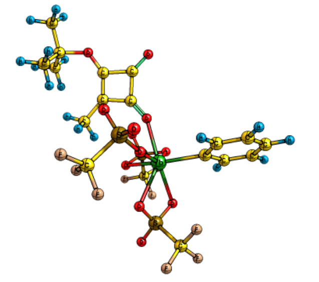

Conformer 9

E(RB3LYP/6-31G(d)/MWB59) = -3731.46319115
Convg = 0.6169D-09 -V/T = 2.0122

Temperature 195.150 Kelvin. Pressure 1.00000 Atm.
elec. and zero-point Energies= -3731.091620
elec. and thermal Energies= -3731.067501
elec. and thermal Enthalpies= -3731.066883
elec. and thermal Free Energies= -3731.141548

RB3LYP/6-31G(d)/MWB59/THF(IEFPCM) = -3731.53051590

RM06-2x/6-311+G(d,p)/MWB59/THF(IEFPCM) = -3731.36741392

Geometry at RB3LYP/6-31G(d)/MWB59
Charge = -1 Multiplicity = 1
Yb 0.6630234508 2.3969793212 0.4654398184
O -0.2663040178 3.0064720262 2.6623031875
S -1.6404358235 2.4232105908 2.4959111654
O -2.0817204407 1.4488060451 3.4951508339
O -1.7718927321 2.0039949527 1.0544732271
C -2.8068652925 3.8633280551 2.690644639
F -2.7040163555 4.3490389314 3.9347567849
F -2.5350405319 4.8315008638 1.8209280479
F -4.0636690465 3.439325705 2.4957355114
O -0.1021453803 4.6945778291 0.0956555312
O -1.8079649045 5.0609751286 -1.7484817803
O -0.2276013297 3.0746226932 -1.6737637892
S -0.5194071853 4.516399351 -1.3384676177
C 0.7530559922 5.462001996 -2.3164529938
F 0.7092877546 6.7569375308 -1.9815658981
F 1.9773907213 4.9894893013 -2.0737017093
F 0.4930412965 5.3443915074 -3.6245773243
C 0.0122318757 -2.6276419486 2.490586317
C -0.5439224835 -1.6029756195 1.7262056703
C 0.5323205069 -0.6816485984 2.1010215018
C 1.1470596422 -1.7695038632 2.9903160496
O 2.0775167402 -1.9145120741 3.7374855562
O 0.8302920694 0.4718432015 1.8129954249
O -0.151305707 -3.8890795338 2.8104575765
C -1.1662645249 -4.8004292885 2.2399918
C -1.6732712877 -1.3462948475 0.7968526187
H -2.5032635753 -2.0462301211 0.910980778
H -1.300734965 -1.4054240404 -0.2333821923
H -2.0441442374 -0.3272626745 0.9464128228
C -2.5493425806 -4.3857004541 2.7479827678
H -3.3037330115 -5.0931805846 2.3863992099
H -2.5692358831 -4.3906026465 3.8425420955
H -2.8327902306 -3.3879253429 2.4063770146
C -0.7571396912 -6.15164608 2.8323607382
H -1.4530608619 -6.9307696057 2.5039363728
H 0.2514765176 -6.4238456555 2.5069296088
H -0.7682783543 -6.1110563669 3.9259997201
C -1.0485683691 -4.8225867805 0.714373621
H -1.309226294 -3.8672455865 0.2563276039
H -0.0262114648 -5.0735126328 0.4154132024
H -1.7232075501 -5.5854270212 0.3101056067
C 2.7980047502 3.2888984518 0.9755981109
C 2.9520977462 4.4181137536 1.812179726
C 3.9989971158 2.7235687594 0.4873619212
H 2.0665355516 4.9068491721 2.2207490763
H 3.9571924725 1.8517566476 -0.1660804585
C 4.2035786812 4.9472129055 2.1441744216
C 5.2594246874 3.241172658 0.8073958729
H 4.2747810856 5.8188960296 2.7941620955
H 6.1586400415 2.7734461393 0.4079386332
C 5.3653240095 4.3572332777 1.6399167556
H 6.3432591258 4.7638220537 1.8934157478
O 3.0437969622 -0.6130556208 -2.1610486542
S 1.712559116 -0.505367831 -1.5662768371
C 0.5212330763 -0.4753495698 -2.9930877124
O 1.4394139567 0.8545109827 -0.9547657479
O 1.2343418519 -1.6174897445 -0.7240061444
F -0.7414642586 -0.3456120445 -2.5428567075
F 0.6010151104 -1.6281962893 -3.6783306681
F 0.7844103734 0.5342582191 -3.824235061

Frequency: 4.9084; 7.6008; 11.4900; 20.1227; 23.1984; 25.0880; 25.3498; 28.0071; 29.8156; 31.2698; 34.5630; 38.5202; 42.5216; 45.9895; 48.5076; 57.5216; 64.9144; 67.2675; 73.4256; 80.4052; 83.7458; 97.4304; 104.2714; 113.3896; 114.2224; 125.0390; 131.6104; 139.3039; 145.2959; 150.1320; 160.9605; 171.5863; 173.3783; 176.9435; 188.6353; 195.7202; 196.4426; 208.2106; 210.0670; 215.6009; 217.0200; 219.2531; 220.4531; 230.2730; 245.1813; 259.2569; 273.5924; 290.5972; 303.1579; 304.9893; 309.8781; 322.9230; 327.5278; 331.7201; 342.1096; 347.9249; 352.6177; 355.5585; 357.6246; 393.6419; 399.9614; 445.5004; 450.9226; 484.3665; 486.9219; 490.1093; 491.0383; 493.8303; 505.5701; 521.1012; 527.8331; 556.4375; 557.8629; 558.6951; 567.4882; 568.6235; 575.4114; 599.6704; 606.3473; 617.0213; 625.0225; 628.7194; 633.4308; 652.9920; 653.7046; 717.7239; 737.4367; 757.8579; 761.8638; 763.2253; 770.4408; 779.0244; 786.8807; 852.1682; 870.0801; 915.6675; 933.2021; 935.3704; 972.9303; 975.6934; 978.3735; 978.8143; 982.1727; 990.2648; 992.6318; 1010.6099; 1011.1805; 1037.3436; 1052.0309; 1062.5607; 1065.4420; 1086.0823; 1087.2980; 1104.7844; 1110.5466; 1126.2754; 1158.5370; 1184.5639; 1204.8665; 1208.9786; 1211.3337; 1214.4951; 1216.8483; 1224.0137; 1227.3123; 1232.5106; 1237.2120; 1244.7172; 1248.3101; 1258.9869; 1285.1052; 1289.0082; 1293.6381; 1296.0545; 1308.5354; 1312.8575; 1369.8971; 1391.2644; 1432.4498; 1434.5391; 1441.7490; 1459.1796; 1460.0790; 1501.2593; 1508.3921; 1514.2338; 1517.2834; 1522.5440; 1522.6526; 1529.4614; 1530.7474; 1549.6741; 1615.3297; 1624.2539; 1628.2081; 1820.5472; 1900.5677; 3061.6581; 3062.8900; 3066.5304; 3073.4769; 3126.8410; 3129.5728; 3132.9461; 3133.4173; 3139.5199; 3141.3697; 3142.4222; 3151.7062; 3153.8129; 3161.0610; 3165.0727; 3176.6441; 3177.5447.

Cartesian Forces: Max 0.000000412 RMS 0.000000166

Item	Value	Threshold	Converged?
Maximum Force	0.000000	0.000450	YES
RMS Force	0.000000	0.000300	YES
Maximum Displacement	0.000071	0.001800	YES
RMS Displacement	0.000018	0.001200	YES

FILE: Yb_Sq2_PhYbOTf3_GS_Anion_B3LYP_M062x_Add56_Conf2-1-_Y.out

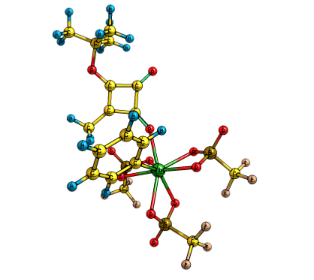

Conformer 10

E(RB3LYP/6-31G(d)/MWB59) = -3731.46725761
Convg = 0.8259D-08 -V/T = 2.0122

Temperature 195.150 Kelvin. Pressure 1.00000 Atm.
elec. and zero-point Energies= -3731.095641
elec. and thermal Energies= -3731.071792
elec. and thermal Enthalpies= -3731.071174
elec. and thermal Free Energies= -3731.143681

RB3LYP/6-31G(d)/MWB59/THF(IEFPCM) = -3731.53332990

RM06-2x/6-311+G(d,p)/MWB59/THF(IEFPCM) = -3731.36771328

Geometry at RB3LYP/6-31G(d)/MWB59
Charge = -1 Multiplicity = 1
Yb 0.8588984618 3.2141060645 0.9271049137
O 2.2044624186 4.2991887867 -0.7508160677
S 3.4677243001 4.509792207 0.051589888
O 4.7159684558 4.0578277706 -0.5545904582
O 3.1520695171 4.0546091374 1.4446535851
C 3.6570485856 6.3615237053 0.1678840195
F 3.784395239 6.8766777099 -1.062102701
F 2.6088927144 6.9278552837 0.7672516393
F 4.7612564571 6.6476804975 0.8710813903
O 0.262974046 5.4139208514 1.8300465645
O -2.192601631 6.048468888 1.8172721932
O -1.1562390953 4.4100869735 0.1764632704
S -1.0198059817 5.6141015414 1.0658834366
C -0.6637782538 7.0224145345 -0.1037508321
F -0.3117778136 8.1100455206 0.5941672836
F 0.3212450182 6.7129705062 -0.9494227757
F -1.7662853758 7.3030063448 -0.8114297399
C 2.3661422157 -2.0124339704 1.0310243221
C 1.3429057836 -1.0822555729 1.1674892867
C 2.2816200337 0.0421035097 1.1237664512
C 3.4433978449 -0.9534865187 0.9804111499
O 4.6460490779 -0.9165278596 0.8775341281
O 2.2250737108 1.2626297055 1.1726917314
O 2.2654562637 -3.3214747535 0.977304434
C 3.447077539 -4.2040711011 0.8088462491
C -0.1240692549 -1.2013485575 1.3048840275
H -0.4391737238 -2.2465580798 1.2331280873
H -0.6206410363 -0.6069576411 0.5284018877
H -0.4380852996 -0.7869593148 2.2704653135
C 4.1296492366 -3.888606906 -0.5239719495
H 4.915136105 -4.6287269864 -0.7141880854
H 3.408320888 -3.9365301188 -1.3465421878
H 4.5956152971 -2.9003057942 -0.5075847846
C 2.8121820212 -5.5952518141 0.7967679956
H 3.5884144494 -6.358079646 0.6773148475
H 2.2789070807 -5.784792532 1.7338182644
H 2.1019178195 -5.6884580123 -0.0309252519
C 4.3858847893 -4.0269912321 2.004299706
H 4.8456682882 -3.0356246423 2.0036908185
H 3.844368227 -4.1766567157 2.9444280673
H 5.1883208336 -4.7713619803 1.9498584976
C 0.1307289631 1.818643896 -0.9212997858
C 1.0088515573 1.2924521601 -1.8962971696
C -1.2251568135 1.4484340195 -1.0819089305
H 2.0686330661 1.5515558391 -1.8579163382
H -1.9674014964 1.8307139555 -0.3808646413
C 0.5851066297 0.4571094193 -2.9376983326
C -1.6713082703 0.6118294232 -2.1135880703
H 1.3044198541 0.0843598025 -3.6672414113
H -2.728297081 0.3570929513 -2.1922977433
C -0.7627443852 0.1076152036 -3.0478252598
H -1.1018201791 -0.5409590479 -3.8543441225
O -0.0195027191 0.6794021306 4.2044387081
S -0.0954866103 2.0108084454 3.5942355597
C -1.1694123139 3.0000480939 4.7501335666
O -0.8097705628 2.0830928383 2.2652539605
O 1.1728244207 2.7846397235 3.4517867248
F -1.3450938995 4.2382989032 4.295821887
F -0.5905828869 3.0528708957 5.9574853632
F -2.3630478601 2.4035047593 4.8705816598

Frequency: 7.7868; 13.1838; 18.1608; 23.4756; 26.8288; 27.0051; 30.1525; 31.5624; 33.6761; 36.7118; 39.1464; 41.6742; 47.6831; 51.0377; 55.9752; 63.8899; 65.2190; 73.4600; 74.6876; 80.9861; 84.6670; 86.9737; 102.4770; 110.0634; 123.9945; 129.1766; 138.7842; 142.3608; 144.2133; 152.2787; 163.5131; 172.1261; 176.6374; 179.5020; 183.8870; 205.9259; 209.0422; 211.1294; 212.4321; 215.1954; 217.0521; 218.5198; 219.3203; 234.6301; 258.1878; 274.1668; 275.5947; 301.8436; 306.4757; 306.7409; 311.2923; 321.6112; 323.6383; 324.6044; 344.1693; 346.7833; 350.5184; 351.0200; 357.2753; 391.7522; 401.4211; 454.0603; 456.2653; 483.8356; 489.6015; 490.5964; 491.6956; 494.5995; 522.1487; 524.5879; 529.2291; 557.7810; 558.0857; 558.7668; 569.5551; 571.9626; 575.3785; 602.0236; 605.3490; 621.3807; 627.0020; 634.5027; 635.0012; 641.7720; 645.9285; 718.3006; 734.3071; 747.3268; 760.7116; 761.5467; 763.2528; 771.9813; 788.6605; 865.9849; 869.1335; 906.3086; 936.1796; 943.0737; 969.1449; 972.5716; 975.8264; 979.1448; 980.3321; 987.2077; 988.2718; 1004.3143; 1011.2527; 1032.2596; 1064.9689; 1068.9450; 1070.9072; 1083.0016; 1083.8252; 1098.9545; 1115.5956; 1117.9641; 1142.7673; 1182.2342; 1210.1449; 1212.8984; 1214.3126; 1215.0610; 1222.8971; 1227.9687; 1229.6721; 1233.3135; 1246.2891; 1250.8115; 1254.0004; 1254.6883; 1283.2083; 1286.2121; 1293.7182; 1302.4607; 1304.4569; 1312.4456; 1367.6048; 1399.8352; 1432.0091; 1433.4758; 1444.7575; 1457.7954; 1459.9244; 1498.3806; 1503.8554; 1508.7433; 1518.6074; 1518.8710; 1519.8680; 1535.3449; 1538.3287; 1555.4286; 1612.5760; 1622.7455; 1626.9176; 1815.3057; 1874.2283; 3047.5339; 3058.4682; 3059.0737; 3066.4688; 3112.6473; 3123.7651; 3124.1349; 3125.9148; 3136.0430; 3137.0592; 3138.0095; 3138.4286; 3146.9536; 3156.5835; 3156.7318; 3160.3272; 3172.8394.

Cartesian Forces: Max 0.000001444 RMS 0.000000557

Item	Value	Threshold	Converged?
Maximum Force	0.000001	0.000450	YES
RMS Force	0.000000	0.000300	YES
Maximum Displacement	0.000028	0.001800	YES
RMS Displacement	0.000006	0.001200	YES

FILE: Yb_Sq2_PhYbOTf3_GS_Anion_B3LYP_M062x_Add56_Conf3.out

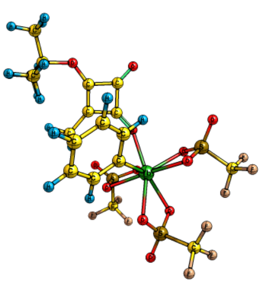

Conformer 11

E(RB3LYP/6-31G(d)/MWB59) = -3731.45789806
Convg = 0.5604D-08 -V/T = 2.0122

Temperature 195.150 Kelvin. Pressure 1.00000 Atm.
elec. and zero-point Energies= -3731.086361
elec. and thermal Energies= -3731.062528
elec. and thermal Enthalpies= -3731.061910
elec. and thermal Free Energies= -3731.134110

RB3LYP/6-31G(d)/MWB59/THF(IEFPCM) = -3731.52520104

RM06-2x/6-311+G(d,p)/MWB59/THF(IEFPCM) = -3731.36412709

Geometry at RB3LYP/6-31G(d)/MWB59
Charge = -1 Multiplicity = 1
Yb 0.9921371213 2.7991060615 1.0031917859
O 2.3733828062 3.6942730296 -0.7511206898
S 3.6608737405 3.8711623746 0.0215590675
O 4.8683077485 3.3089437414 -0.5738502027
O 3.3392404495 3.5164075551 1.4423696838
C 3.9651833314 5.7113883694 0.0283281297
F 4.0969521676 6.1477427697 -1.231244138
F 2.9668491267 6.3746813447 0.6133345029
F 5.099605572 5.966932536 0.6938521892
O 0.5580826083 5.0749555971 1.7901942742
O -1.8483071975 5.8756837451 1.773745813
O -0.9511258206 4.0883527703 0.207852127
S -0.719851277 5.3238832153 1.0316417752
C -0.2870679126 6.6430470823 -0.213861785
F 0.1590517605 7.734359872 0.4213644086
F 0.6531851506 6.2198867682 -1.0611628167
F -1.3817084593 6.9694829914 -0.9141944146
C 2.2220188127 -2.4672345524 0.7884514818
C 1.2685180134 -1.5366896715 1.1995161314
C 2.2561016773 -0.4464438991 1.1617397169
C 3.3460251588 -1.4621859688 0.7827617567
O 4.5302321556 -1.4931871154 0.5771559057
O 2.2217036415 0.7670291327 1.3155573783
O 2.36299636 -3.7285714484 0.4528489815
C 1.2646956327 -4.7021669664 0.2708734757
C -0.169485188 -1.4504865235 1.5543904076
H -0.6751848403 -2.4168819799 1.574042024
H -0.6778868859 -0.7946471604 0.8358719715
H -0.2729965388 -0.9743152805 2.5366509545
C 0.6613115168 -5.0375339824 1.637253272
H -0.1077220667 -5.8093813541 1.522236639
H 1.43573516 -5.4215222964 2.3092080487
H 0.197796028 -4.169650149 2.110518924
C 2.0011643174 -5.9160410503 -0.3024405003
H 1.2959738992 -6.7367050526 -0.4710080054
H 2.4763614458 -5.6622744015 -1.2548468792
H 2.7765290551 -6.2579516495 0.3902516121
C 0.2516891379 -4.1606574022 -0.7407932243
H -0.2660861074 -3.2656490936 -0.3918451913
H 0.7508567526 -3.9130591093 -1.6827431693
H -0.5032169638 -4.9283715889 -0.9437178949
C 0.1214360677 1.3635593542 -0.7623329549
C 0.9337384319 0.7052136757 -1.7139560394
C -1.2619677092 1.086424911 -0.8718644808
H 2.0100517149 0.8884406358 -1.7174673791
H -1.9569653869 1.574010631 -0.1881502459
C 0.4256881168 -0.1726696562 -2.6803869265
C -1.792921875 0.2098482765 -1.8274311116
H 1.0980254844 -0.6473335867 -3.3954880168
H -2.8674713449 0.0305977433 -1.8673517625
C -0.9463629263 -0.4313220996 -2.7362099579
H -1.3516250107 -1.1094770066 -3.4860050493
O -0.0236339389 0.513591137 4.4178081977
S -0.0083353566 1.8123510303 3.7370139135
C -0.9797172312 2.9409519997 4.8557449742
O -0.7407783992 1.8711709103 2.4172701208
O 1.3123836809 2.4777073688 3.5324702592
F -1.0672380595 4.1645047045 4.340330738
F -0.3758243331 3.0091522701 6.0498688118
F -2.2137184238 2.4469457087 5.0247245984

Frequency: 7.2477; 13.7527; 20.3838; 21.4895; 26.0705; 29.2585; 30.1515; 32.4641; 35.0629; 37.5436; 40.9312; 45.9052; 52.0756; 57.5039; 60.2756; 64.0484; 66.8125; 71.6980; 75.5040; 78.1197; 85.1426; 91.5550; 102.3698; 111.4469; 125.1275; 128.8839; 133.6191; 142.2478; 145.1594; 151.2398; 165.9193; 174.8804; 177.4511; 181.6096; 183.6325; 205.6292; 208.2929; 210.0836; 211.6856; 212.5857; 215.8916; 218.1406; 219.7791; 230.2622; 242.4264; 259.4599; 276.2580; 290.5632; 302.5354; 306.6300; 309.2904; 321.4598; 323.6723; 324.5275; 345.2431; 350.9845; 351.2874; 356.1014; 357.8510; 392.4638; 399.7040; 451.6778; 457.7631; 483.9484; 489.6043; 490.5979; 491.2938; 497.8476; 522.2968; 524.6774; 529.5235; 557.8467; 558.1354; 558.7887; 569.6086; 571.9887; 575.5044; 595.7386; 605.7932; 621.3690; 627.2248; 634.4012; 635.5851; 644.9190; 652.5740; 719.9464; 734.4925; 760.6963; 761.6038; 763.1189; 767.4367; 776.0016; 784.4868; 849.0757; 869.8685; 906.3569; 931.4818; 935.2341; 970.8860; 975.2354; 976.2213; 978.6076; 979.1622; 980.3331; 987.0189; 1006.8243; 1010.8415; 1031.2655; 1059.5879; 1062.8219; 1064.1085; 1082.2924; 1082.8807; 1098.7410; 1114.7422; 1117.0313; 1141.9542; 1181.6252; 1206.9144; 1209.8274; 1212.7235; 1214.0445; 1214.7804; 1222.7426; 1227.9264; 1229.9195; 1233.3465; 1246.6678; 1250.8604; 1254.7870; 1283.4178; 1284.5015; 1292.5643; 1293.0498; 1303.2387; 1313.0186; 1367.3571; 1387.0543; 1432.3399; 1434.2136; 1446.1689; 1457.3265; 1459.9001; 1499.5352; 1505.2509; 1509.7864; 1518.3238; 1519.0571; 1520.2737; 1529.0504; 1530.7504; 1549.3425; 1611.2254; 1621.1451; 1624.0069; 1819.5806; 1897.1692; 3048.9338; 3061.5775; 3066.1144; 3073.1777; 3107.0785; 3122.7070; 3128.5881; 3132.1594; 3136.0818; 3138.9730; 3142.1475; 3146.5697; 3156.1421; 3157.4356; 3167.5841; 3171.9938; 3175.5739.

Cartesian Forces: Max 0.000003372 RMS 0.000000752

Item	Value	Threshold	Converged?
Maximum Force	0.000003	0.000450	YES
RMS Force	0.000000	0.000300	YES
Maximum Displacement	0.000924	0.001800	YES
RMS Displacement	0.000195	0.001200	YES

FILE: Yb_Sq2_PhYbOTf3_GS_Anion_B3LYP_M062x_Add56_Conf3-.out

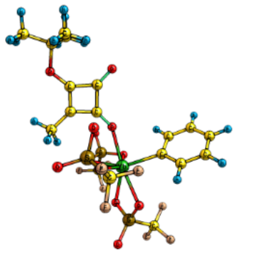

Conformer 12

E(RB3LYP/6-31G(d)/MWB59) = -3731.47076064
Convg = 0.3186D-08 -V/T = 2.0122

Temperature 195.150 Kelvin. Pressure 1.00000 Atm.
elec. and zero-point Energies= -3731.098997
elec. and thermal Energies= -3731.074954
elec. and thermal Enthalpies= -3731.074336
elec. and thermal Free Energies= -3731.148753

RB3LYP/6-31G(d)/MWB59/THF(IEFPCM) = -3731.53651042

RM06-2x/6-311+G(d,p)/MWB59/THF(IEFPCM) = -3731.37086646

Geometry at RB3LYP/6-31G(d)/MWB59
Charge = -1 Multiplicity = 1
Yb 0.0531786683 2.7981534612 1.0063237975
O 0.427870508 3.1117209733 3.3661555578
S -0.2982266825 1.9172100598 3.9335079619
O 0.4644922318 1.0402677225 4.8209020538
O -1.0295696081 1.2677609318 2.7959586364
C -1.6196380418 2.6523847138 5.022019686
F -1.0445254432 3.2708772946 6.0619347799
F -2.3696494064 3.5287698565 4.358171615
F -2.404227694 1.6691605328 5.4857929303
O -2.0488995441 3.8486701687 1.4815271
O -3.5246321568 4.6778422823 -0.4078851194
O -1.0208350914 4.3228545395 -0.6364053223
S -2.2082243555 4.6592801356 0.2164359962
C -1.9004338327 6.4060599679 0.7853164821
F -2.8988405145 6.8056629763 1.5828174861
F -0.7509246901 6.4786089947 1.4635675569
F -1.8364983754 7.224082034 -0.2710675379
C 2.2041170065 -2.1479674948 1.4217503849
C 1.0598286219 -1.3604843846 1.4491147527
C 1.841551653 -0.1251211792 1.4118947671
C 3.1306528439 -0.9544443077 1.3986346403
O 4.3212619592 -0.754666344 1.3871102555
O 1.6248236225 1.0813355302 1.4120876107
O 2.2818195257 -3.4581579601 1.4217876071
C 3.5794129486 -4.1776389091 1.3548071122
C -0.3850117628 -1.6823446674 1.476385361
H -0.545878288 -2.7082973888 1.8225101636
H -0.8070423456 -1.5905579323 0.465853858
H -0.9204596988 -0.9763631943 2.1177084913
C 4.2937299506 -3.8100417032 0.0525158292
H 5.1794608621 -4.4443129596 -0.067059113
H 3.6354721839 -3.9735024815 -0.8069257799
H 4.6235449967 -2.76836989 0.0599517838
C 3.1371366751 -5.6414803471 1.3569510746
H 4.0140185484 -6.295444288 1.3103091258
H 2.5768285624 -5.8749649333 2.2680599061
H 2.4984867935 -5.8527764473 0.4935071524
C 4.4092432045 -3.8404268392 2.5954830731
H 4.7296821854 -2.7956951042 2.585386423
H 3.8357910137 -4.0367312672 3.5077790011
H 5.3067565783 -4.4690471554 2.6142003763
C 1.9394230543 4.1982185131 0.6000670725
C 3.0051606525 4.2636547246 1.5275815456
C 2.1116111094 4.9691018768 -0.5726992847
H 2.942308711 3.6956656881 2.4560053872
H 1.327532753 4.9743948135 -1.3291630224
C 4.1515350449 5.0356164704 1.3116344449
C 3.2549250221 5.7408699547 -0.8101911977
H 4.9453614994 5.0561436595 2.0578219987
H 3.3439672562 6.3157062679 -1.7317164945
C 4.2813425883 5.7781601255 0.1356502224
H 5.1724387842 6.3787285428 -0.0412789627
O 1.2501863419 -0.2038861813 -1.5268753306
S -0.1149806641 0.3225538406 -1.6781848292
C -0.0782729943 1.3592926899 -3.2205613419
O -0.4623740236 1.3733299445 -0.6347603938
O -1.1999069536 -0.6457837573 -1.8734151595
F -1.2744478073 1.9083393537 -3.4475012701
F 0.2474371441 0.5886477164 -4.2708429132
F 0.8330942805 2.3323552096 -3.1072852039

Frequency: 8.1026; 8.8956; 12.2949; 18.2979; 19.6192; 20.9783; 24.5337; 26.9150; 28.7995; 33.5161; 34.5383; 36.9569; 40.9955; 46.2428; 49.3300; 55.9513; 63.4972; 67.8594; 78.2329; 80.0614; 87.3989; 89.9795; 95.4770; 108.5207; 111.2496; 124.3346; 131.3134; 144.8659; 153.0003; 156.0536; 162.0640; 171.3118; 173.9174; 179.4892; 194.2226; 196.1929; 203.5819; 209.0921; 215.0150; 217.7933; 218.8521; 219.7680; 224.0105; 240.2231; 259.3287; 272.8501; 276.1422; 302.6680; 305.3154; 307.1687; 315.2375; 321.5192; 328.5380; 330.3363; 342.7945; 347.1020; 347.6165; 352.3724; 356.3383; 393.9525; 402.7166; 445.2908; 454.5905; 486.4739; 489.7175; 491.0387; 491.3457; 495.2876; 507.3688; 522.1314; 527.7974; 556.1115; 557.6967; 558.8530; 568.9319; 569.5430; 576.6884; 601.7689; 606.9462; 617.8945; 622.5430; 628.3429; 634.0948; 643.6845; 651.2522; 715.6271; 736.1937; 749.3654; 761.1133; 761.7040; 762.8420; 774.8053; 789.9961; 866.0796; 867.8221; 913.2737; 936.5557; 943.2435; 972.8642; 974.8139; 976.2742; 977.3262; 984.4267; 988.4208; 989.3194; 1009.0034; 1012.0534; 1035.7498; 1064.7751; 1069.7230; 1071.6698; 1085.9933; 1087.1099; 1103.9600; 1118.8989; 1123.3091; 1153.5387; 1184.1624; 1209.8828; 1213.2306; 1215.4990; 1225.0001; 1226.9422; 1231.4509; 1232.1823; 1236.6654; 1240.7215; 1242.4496; 1256.7872; 1257.6990; 1286.4994; 1293.8340; 1296.6714; 1299.0315; 1304.6905; 1316.6370; 1369.9655; 1403.8013; 1432.0981; 1434.2181; 1450.1708; 1459.1550; 1460.4530; 1503.8319; 1507.2444; 1513.9086; 1518.1025; 1519.0678; 1522.6824; 1535.3156; 1538.1516; 1555.1689; 1615.3410; 1625.4783; 1627.9700; 1809.0798; 1876.6420; 3043.7887; 3057.9674; 3059.5122; 3066.5828; 3110.1100; 3123.5499; 3127.2664; 3136.2741; 3136.7616; 3138.5563; 3143.7404; 3150.4641; 3156.1081; 3157.0203; 3161.2882; 3163.7261; 3176.1749.

Cartesian Forces: Max 0.000011190 RMS 0.000002217

Item	Value	Threshold	Converged?
Maximum Force	0.000010	0.000450	YES
RMS Force	0.000001	0.000300	YES
Maximum Displacement	0.001284	0.001800	YES
RMS Displacement	0.000266	0.001200	YES

FILE: Yb_Sq2_PhYbOTf3_GS_Anion_B3LYP_M062x_Add56_Conf3-1.out

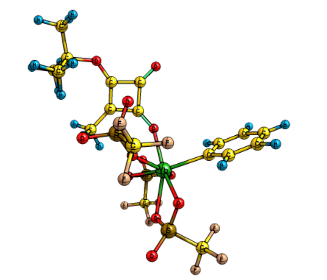

Conformer 13

E(RB3LYP/6-31G(d)/MWB59) = -3731.46216337
Convg = 0.6356D-08 -V/T = 2.0122

Temperature 195.150 Kelvin. Pressure 1.00000 Atm.
elec. and zero-point Energies= -3731.090622
elec. and thermal Energies= -3731.066506
elec. and thermal Enthalpies= -3731.065888
elec. and thermal Free Energies= -3731.140210

RB3LYP/6-31G(d)/MWB59/THF(IEFPCM) = -3731.52778238

RM06-2x/6-311+G(d,p)/MWB59/THF(IEFPCM) = -3731.36625542

Geometry at RB3LYP/6-31G(d)/MWB59
Charge = -1 Multiplicity = 1
Yb 0.144124636 2.4265988194 1.202573373
O 0.3056602629 2.94827264 3.5411378178
S -0.5526541141 1.864229262 4.1469050591
O 0.0542534255 1.048490056 5.1965915371
O -1.2086614901 1.1407049967 3.0081157796
C -1.9233276157 2.7885295826 5.0070822564
F -1.4157318056 3.4739261859 6.0401303601
F -2.5356535437 3.6371410169 4.1845308007
F -2.8209496283 1.9085383587 5.4733931308
O -1.9454492847 3.5929201244 1.3466856412
O -3.1727964275 4.292808382 -0.7598404489
O -0.6777632415 3.8294511858 -0.678803717
S -1.932328204 4.2867935773 0.0046734169
C -1.6069153928 6.0671667172 0.4445721173
F -2.6646954504 6.5754304187 1.0889251637
F -0.5333864196 6.1615906972 1.2351480604
F -1.3944131611 6.7790671222 -0.6676194463
C 2.0604122021 -2.5353538164 1.4551370128
C 0.9429204416 -1.7355013848 1.7003039322
C 1.7767973918 -0.534592023 1.8009541343
C 3.0343629321 -1.4014936393 1.66125284
O 4.2294513158 -1.284054881 1.7097045632
O 1.5694306499 0.6724556156 1.8899159167
O 2.4050287903 -3.7566213376 1.1386037771
C 1.5044878003 -4.8098814969 0.6042402674
C -0.5347918843 -1.8621900357 1.806316724
H -0.8414018676 -2.8285267366 2.2164292015
H -1.0051342138 -1.7414905321 0.8218121737
H -0.9222648894 -1.0627920462 2.4444494729
C 0.7018293964 -5.4029537383 1.7635367842
H 0.0961558314 -6.2413449 1.4018213098
H 1.37261821 -5.773949235 2.5454784092
H 0.0250730372 -4.6691457578 2.2072085685
C 2.5053785083 -5.8316488995 0.0578754078
H 1.9719287949 -6.7036841869 -0.334740125
H 3.098636347 -5.3922355584 -0.7495749928
H 3.1868793083 -6.1643054747 0.847290894
C 0.6350765051 -4.2445474152 -0.5191684755
H -0.1432466832 -3.5691111393 -0.1627282495
H 1.2447605698 -3.6954093743 -1.2424399808
H 0.1421022996 -5.0735959899 -1.039152362
C 2.1220003904 3.7099993627 0.869865057
C 3.0784531844 3.8500990464 1.9023292763
C 2.4597834849 4.3299569472 -0.3552692159
H 2.8853546379 3.398875959 2.87570588
H 1.7666903193 4.2714631192 -1.1938638701
C 4.2760863943 4.553107078 1.7347881335
C 3.6568953009 5.0301826243 -0.5440131576
H 4.9813023566 4.6361183871 2.5612364819
H 3.8753846518 5.4880900821 -1.5084149742
C 4.5710741728 5.1460195723 0.5049548777
H 5.5032117878 5.6916646965 0.3659055924
O 1.3958603275 -0.666950841 -1.359497775
S 0.0184299107 -0.155531552 -1.4338326486
C -0.04474904 0.8894600749 -2.9705126608
O -0.2901012227 0.8706188531 -0.3571626254
O -1.0610490447 -1.142610931 -1.5813347185
F -1.2549237133 1.4315745094 -3.1207825892
F 0.2185675557 0.1204154266 -4.039835449
F 0.8690705433 1.8630529429 -2.9099639596

Frequency: 6.0177; 13.1996; 14.4447; 18.3931; 20.1295; 25.3025; 25.5959; 27.4186; 29.2610; 31.2863; 34.7172; 36.2880; 42.9699; 47.7346; 49.7501; 55.0320; 61.7460; 63.4446; 74.9820; 78.1783; 79.6345; 86.9259; 97.1510; 109.0683; 112.9656; 125.4126; 130.9271; 143.7944; 150.3482; 153.6092; 168.0002; 173.1676; 179.9440; 181.0123; 194.3378; 195.4156; 196.5394; 205.5361; 208.1322; 212.5282; 217.1322; 219.4310; 222.0970; 233.3580; 247.7756; 263.6547; 278.1531; 294.2863; 303.4291; 306.8490; 308.5915; 321.4341; 327.3911; 330.1446; 339.2356; 347.4281; 350.1036; 354.7728; 357.5167; 393.8793; 399.0727; 445.1915; 450.1976; 480.5220; 487.9984; 490.7934; 491.0159; 499.5256; 506.2669; 522.4357; 527.8613; 556.1231; 557.6201; 558.8050; 567.8547; 569.9025; 576.6388; 596.5107; 612.4469; 619.0446; 622.5833; 628.6074; 634.0122; 649.3888; 651.5338; 715.3892; 736.4449; 761.2619; 762.3613; 762.6071; 769.8377; 781.9029; 790.5187; 844.8782; 867.7678; 913.7087; 930.5896; 938.0230; 973.0123; 975.2530; 976.3569; 977.8177; 981.7888; 984.8129; 989.9182; 1006.6698; 1011.9984; 1035.9740; 1058.8006; 1065.6676; 1066.9878; 1086.0555; 1087.2274; 1098.2828; 1118.5997; 1121.6218; 1152.0143; 1184.3943; 1203.8976; 1212.6581; 1213.1219; 1215.4167; 1223.3797; 1225.7556; 1231.6081; 1233.6888; 1236.8891; 1241.2688; 1245.0528; 1256.8197; 1282.3481; 1290.3344; 1294.9433; 1296.5298; 1296.8232; 1316.5669; 1369.9751; 1395.9358; 1431.1599; 1434.5472; 1453.6564; 1459.2452; 1459.7571; 1501.3782; 1508.6263; 1511.5171; 1518.2996; 1522.3415; 1522.7995; 1530.0544; 1533.1037; 1546.2801; 1615.2614; 1619.0391; 1628.2238; 1809.1657; 1900.5208; 3051.7473; 3060.8117; 3065.3364; 3074.8591; 3117.9483; 3127.3369; 3135.4224; 3137.6573; 3140.5284; 3144.4148; 3144.5909; 3148.5915; 3156.9380; 3159.6469; 3164.2094; 3176.7498; 3185.4253.

Cartesian Forces: Max 0.000000995 RMS 0.000000284

Item	Value	Threshold	Converged?
Maximum Force	0.000001	0.000450	YES
RMS Force	0.000000	0.000300	YES
Maximum Displacement	0.000041	0.001800	YES
RMS Displacement	0.000011	0.001200	YES

FILE: Yb_Sq2_PhYbOTf3_GS_Anion_B3LYP_M062x_Add56_Conf3-1-_Y.out

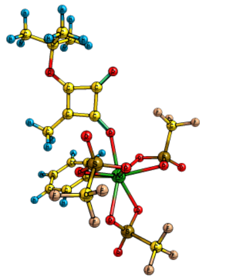

Conformer 14

E(RB3LYP/6-31G(d)/MWB59) = -3731.47039099
Convg = 0.8369D-09 -V/T = 2.0122

Temperature 195.150 Kelvin. Pressure 1.00000 Atm.
elec. and zero-point Energies= -3731.098674
elec. and thermal Energies= -3731.074827
elec. and thermal Enthalpies= -3731.074209
elec. and thermal Free Energies= -3731.147046

RB3LYP/6-31G(d)/MWB59/THF(IEFPCM) = -3731.53673319

RM06-2x/6-311+G(d,p)/MWB59/THF(IEFPCM) = -3731.37452585

Geometry at RB3LYP/6-31G(d)/MWB59
Charge = -1 Multiplicity = 1
Yb 0.3429176177 2.9046618747 0.9237573611
O 2.1552582003 2.2098379668 -0.7230544586
S 3.3207316062 2.555082133 0.1596006197
O 4.2910248859 1.4841261768 0.4015377973
O 2.7699003582 3.2682609784 1.3594148463
C 4.2806230885 3.8489102206 -0.7754008385
F 4.714217881 3.329539607 -1.9337029551
F 3.5329702539 4.9196283497 -1.0359908625
F 5.3429086272 4.2270567848 -0.0508284882
O -1.1988960361 4.8055281999 1.0284940562
O -1.365929934 6.2764179904 -1.0371006293
O 0.6339123905 4.8006117729 -0.5239911897
S -0.5298922912 5.6290077747 -0.0346612418
C 0.2879515048 7.0153440764 0.9023008624
F -0.6383348733 7.7653686198 1.5088337669
F 1.1231193608 6.5243486756 1.8231223453
F 0.9828768028 7.7869842466 0.055931259
C 2.1489598203 -2.2242787829 0.7619423286
C 1.5885365882 -1.1484005705 0.083069379
C 1.480108011 -0.3584026439 1.3117638227
C 2.0723901177 -1.533146798 2.1040481575
O 2.3406059969 -1.79224698 3.2525843273
O 1.1018199836 0.7584690966 1.6353066171
O 2.5519754335 -3.3709742675 0.26389846
C 3.1727661639 -4.428281281 1.1018766939
C 1.25039994 -0.9126980867 -1.3366534714
H 1.6198017032 -1.7281986708 -1.9658701894
H 1.6745567466 0.0432176045 -1.660425466
H 0.1640672874 -0.8192088817 -1.4574252886
C 4.4523900748 -3.8834272399 1.7399308367
H 4.9837473169 -4.6998882212 2.2419303544
H 5.1146603741 -3.4625509222 0.9762272844
H 4.2300434459 -3.1174267399 2.4867900749
C 3.4801525365 -5.5169010661 0.0728357778
H 3.9479232292 -6.3748251597 0.5666262188
H 2.5620051855 -5.8555955542 -0.4178051481
H 4.164497547 -5.1409555301 -0.6943085427
C 2.1580880786 -4.9096939456 2.1412472984
H 1.9474470156 -4.1313254235 2.8788454329
H 1.2247367816 -5.2142773507 1.6557583152
H 2.5654537231 -5.7772563784 2.6725879061
C -1.0424169303 1.7378573553 -0.6616888774
C -0.9000204483 1.9340536512 -2.0552180836
C -1.989491271 0.7566643379 -0.2879836957
H -0.1904528578 2.6774169 -2.4198610682
H -2.171728981 0.5694987102 0.7705815808
C -1.6260235379 1.2051499638 -3.0047323314
C -2.7201657362 0.0099139434 -1.2213003127
H -1.4813395635 1.3948245699 -4.0681571153
H -3.4375069167 -0.7382289497 -0.883361841
C -2.5370421308 0.2300567312 -2.5894570303
H -3.1034362882 -0.3438163698 -3.3215535522
O -1.7559101844 3.754452931 4.4976009106
S -0.7994922645 2.9412493904 3.7544734526
C -0.2664222391 1.6386167197 4.9800161177
O 0.4810954568 3.5885498459 3.3065082464
O -1.3148347568 2.1640215543 2.570971776
F 0.6966832129 0.8563390201 4.4896208925
F -1.3198507044 0.8728272414 5.3029576135
F 0.1880435009 2.233088497 6.0919690735

Frequency: 6.0669; 12.7430; 15.8604; 21.9867; 23.7530; 26.4142; 29.2625; 31.9078; 34.1805; 36.0169; 38.0242; 41.6705; 48.2150; 49.2157; 58.6592; 59.8504; 67.2683; 68.7743; 78.3908; 81.7803; 84.6623; 95.2471; 107.8788; 111.7420; 123.4858; 127.7769; 135.6500; 140.7183; 145.4422; 156.3889; 159.8935; 162.1763; 176.9745; 179.9599; 186.7854; 207.9672; 210.3526; 212.1347; 212.7926; 215.1900; 218.4834; 219.6486; 222.9552; 232.9192; 258.6391; 273.6791; 276.7152; 303.2679; 304.6747; 307.5559; 317.2769; 321.5570; 324.6236; 329.3081; 343.8598; 346.8831; 348.5264; 351.4138; 357.7174; 392.4458; 402.0416; 450.9805; 454.1823; 482.7676; 489.5589; 491.0741; 491.5986; 494.7539; 520.3261; 523.3978; 528.8793; 557.8007; 558.0017; 558.4738; 568.6353; 569.8881; 577.3040; 602.7545; 607.2673; 621.8535; 626.1335; 631.4049; 634.2977; 641.8020; 648.2461; 719.9881; 737.4377; 747.6075; 759.6087; 761.0312; 762.3165; 773.3414; 788.2949; 865.5741; 872.1211; 912.5965; 936.2187; 943.0377; 971.1066; 975.3684; 976.9470; 979.3497; 982.9572; 987.1668; 988.1690; 1005.4938; 1011.3549; 1034.2716; 1056.2957; 1068.9517; 1070.9495; 1082.7723; 1084.6313; 1098.4702; 1115.6035; 1117.9032; 1137.0944; 1182.6401; 1209.5883; 1212.7729; 1214.1655; 1215.6506; 1223.3302; 1227.9165; 1229.1804; 1235.2717; 1241.5536; 1244.7456; 1252.8970; 1253.8344; 1285.4353; 1286.6269; 1294.9287; 1304.5612; 1305.5653; 1316.9441; 1368.1825; 1399.7726; 1431.9540; 1433.7855; 1438.2276; 1458.2679; 1460.0577; 1491.2759; 1502.9003; 1503.8662; 1518.1905; 1518.9919; 1519.6558; 1535.3258; 1538.3166; 1555.2403; 1611.8750; 1623.2876; 1625.9790; 1816.9146; 1876.0615; 3054.4457; 3058.2886; 3059.5518; 3066.6832; 3120.2570; 3123.9470; 3126.6888; 3133.4747; 3136.8810; 3138.3121; 3139.6329; 3145.7311; 3152.6821; 3156.7570; 3158.1677; 3160.7443; 3173.9310.

Cartesian Forces: Max 0.000001247 RMS 0.000000465

Item	Value	Threshold	Converged?
Maximum Force	0.000000	0.000450	YES
RMS Force	0.000000	0.000300	YES
Maximum Displacement	0.000043	0.001800	YES
RMS Displacement	0.000009	0.001200	YES

FILE: Yb_Sq2_PhYbOTf3_GS_Anion_B3LYP_M062x_Add56_Conf4_Y.out

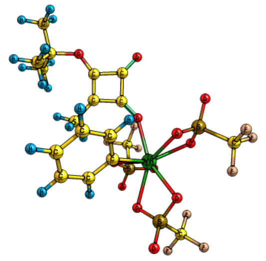

Conformer 15

E(RB3LYP/6-31G(d)/MWB59) = -3731.45909503
Convg = 0.1557D-08 -V/T = 2.0122

Temperature 195.150 Kelvin. Pressure 1.00000 Atm.
elec. and zero-point Energies= -3731.087381
elec. and thermal Energies= -3731.063573
elec. and thermal Enthalpies= -3731.062955
elec. and thermal Free Energies= -3731.135805

RB3LYP/6-31G(d)/MWB59/THF(IEFPCM) = -3731.52810983

RM06-2x/6-311+G(d,p)/MWB59/THF(IEFPCM) = -3731.36988551

Geometry at RB3LYP/6-31G(d)/MWB59
Charge = -1 Multiplicity = 1
Yb 0.7828841418 2.6476733758 0.7930195849
O 2.8107805789 2.4973340755 -0.5846888603
S 3.8021434146 2.9444737164 0.4576782247
O 4.9710332708 2.0987143166 0.6780715643
O 2.9909716177 3.3494201747 1.6581014767
C 4.5140507284 4.5359443806 -0.2038947368
F 5.1496117398 4.2916214477 -1.3588515572
F 3.5719244212 5.4529970083 -0.4159612364
F 5.3995309455 5.0228941804 0.6780653762
O -1.2318934342 4.0574393956 0.7891812119
O -1.3998714461 5.772665308 -1.0750570353
O 0.7991133779 4.8092819586 -0.249211377
S -0.6308213766 5.2041792062 0.0245435467
C -0.4768761668 6.5748333039 1.2768101891
F -1.6891096999 6.9114868971 1.7319273579
F 0.2838482609 6.1901724893 2.3041840909
F 0.0823216967 7.6479352965 0.7018980599
C 1.5653830033 -2.7313484317 1.0432990241
C 0.6525627646 -1.679418343 0.9672099922
C 1.7043685958 -0.6900785975 1.269065846
C 2.7199164399 -1.8368715275 1.4186859232
O 3.8741905164 -1.9998906741 1.7107441108
O 1.7715029841 0.5276258498 1.3516165806
O 1.6660252044 -4.032531315 0.9009844325
C 0.5947878491 -4.9475749385 0.4515086567
C -0.7920057413 -1.459054372 0.7009457588
H -1.4052337279 -2.3320887799 0.937764977
H -0.9476791505 -1.195351554 -0.3526843998
H -1.141159226 -0.6030748022 1.2847248447
C -0.4554166227 -5.0644307563 1.5588276475
H -1.2155160862 -5.7993888694 1.2718359709
H 0.0116582682 -5.3983932635 2.4909437843
H -0.9610110254 -4.1154242403 1.7491842826
C 1.3496730391 -6.2692566412 0.2841166559
H 0.6580446491 -7.0589435748 -0.0275601676
H 2.132883559 -6.171358244 -0.4737990891
H 1.8178409863 -6.5664568617 1.2276027565
C 0.0268469592 -4.4779903447 -0.889567137
H -0.5023916253 -3.5264919365 -0.817399464
H 0.8304326247 -4.370032822 -1.6250793292
H -0.680190069 -5.226240642 -1.2644574321
C -0.2028396624 1.4535104375 -1.0643266854
C 0.5604258809 0.6768843574 -1.9673957235
C -1.6028762249 1.394842721 -1.2602578237
H 1.6481467092 0.6964420302 -1.8967223718
H -2.2560050079 1.9762263802 -0.6098595945
C -0.01693644 -0.1196527015 -2.9649618355
C -2.2007142946 0.6093977608 -2.2534799731
H 0.6156812068 -0.7010778667 -3.6362031808
H -3.2850201861 0.5980425713 -2.3629730652
C -1.4068979174 -0.1618768627 -3.1079020304
H -1.8644266298 -0.7755372223 -3.8828265852
O -1.8958629352 2.7737589765 4.1269859217
S -0.7139189497 2.2339296975 3.4640917972
C -0.0373759388 0.9935313463 4.6808902633
O 0.4384292869 3.1582532757 3.2006825246
O -0.9205323659 1.4349938485 2.2053441213
F 1.1086330634 0.4536957502 4.2445578594
F -0.9281004285 0.0036275615 4.8579324358
F 0.1898602007 1.5822660933 5.8597782693

Frequency: 3.0640; 11.4994; 19.2293; 22.4234; 23.1337; 25.1986; 28.5033; 29.1845; 34.7173; 36.5739; 40.3615; 50.4685; 54.1788; 60.9756; 62.3165; 65.6020; 67.7308; 70.1501; 75.3185; 79.9765; 88.9409; 96.4958; 107.8215; 115.1620; 126.8483; 132.4179; 139.0315; 143.3710; 145.9755; 149.3953; 153.8493; 171.5115; 180.9879; 182.2520; 185.3435; 196.1224; 209.6847; 211.8569; 212.3015; 213.4346; 216.8051; 219.4979; 220.9631; 235.4845; 237.7156; 260.6238; 287.6099; 294.0716; 304.1716; 305.7611; 307.1783; 321.5786; 324.3639; 326.2925; 346.4588; 348.8823; 352.4314; 354.2992; 358.4287; 393.6506; 398.7652; 449.6592; 450.5344; 484.1477; 488.6606; 490.4223; 491.2078; 491.7188; 520.4217; 523.9912; 528.8954; 557.1928; 558.3195; 558.3933; 568.2628; 570.8526; 576.4597; 599.9924; 605.0035; 622.5181; 625.3617; 631.8490; 634.1925; 647.0191; 649.2201; 721.1931; 737.8335; 757.8341; 761.3773; 761.7691; 767.4374; 779.1699; 785.0101; 849.0095; 873.6314; 914.6061; 932.5162; 935.5761; 976.6981; 977.0286; 978.6310; 980.1725; 981.3974; 984.1027; 985.6432; 1003.8819; 1011.1228; 1032.9506; 1052.2464; 1062.2187; 1064.3056; 1082.5676; 1083.2912; 1099.7277; 1112.9015; 1117.5461; 1131.5009; 1181.9933; 1202.3029; 1208.1930; 1212.2131; 1214.5617; 1215.0174; 1223.3690; 1225.2105; 1228.0835; 1235.7045; 1238.0081; 1245.1037; 1254.4807; 1285.1614; 1293.2709; 1294.1065; 1302.4279; 1306.6370; 1317.0327; 1368.1229; 1386.1175; 1432.0827; 1434.2704; 1440.0290; 1457.9528; 1459.5973; 1496.1370; 1503.9720; 1511.0198; 1516.8047; 1518.7840; 1520.9066; 1528.9080; 1531.4249; 1550.0871; 1610.3596; 1620.9000; 1622.1963; 1824.2068; 1898.8000; 3059.8827; 3061.9580; 3066.2827; 3072.8107; 3125.4099; 3129.7010; 3130.3730; 3133.2205; 3139.5554; 3141.8414; 3142.8438; 3154.5033; 3155.8507; 3161.5167; 3164.4924; 3173.4926; 3176.6070.

Cartesian Forces: Max 0.000000777 RMS 0.000000294

Item	Value	Threshold	Converged?
Maximum Force	0.000000	0.000450	YES
RMS Force	0.000000	0.000300	YES
Maximum Displacement	0.000477	0.001800	YES
RMS Displacement	0.000097	0.001200	YES

FILE: Yb_Sq2_PhYbOTf3_GS_Anion_B3LYP_M062x_Add56_Conf4-_Y.out

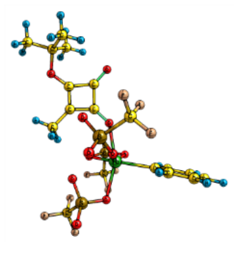

Conformer 16

E(RB3LYP/6-31G(d)/MWB59) = -3731.46874549
Convg = 0.1856D-09 -V/T = 2.0122

Temperature 195.150 Kelvin. Pressure 1.00000 Atm.
elec. and zero-point Energies= -3731.096996
elec. and thermal Energies= -3731.072941
elec. and thermal Enthalpies= -3731.072323
elec. and thermal Free Energies= -3731.146323

RB3LYP/6-31G(d)/MWB59/THF(IEFPCM) = -3731.53549125

RM06-2x/6-311+G(d,p)/MWB59/THF(IEFPCM) = -3731.37127552

Geometry at RB3LYP/6-31G(d)/MWB59
Charge = -1 Multiplicity = 1
Yb 0.8549181142 2.6273185724 1.0076577307
O 1.4309537614 2.9109072178 3.3696378997
S 0.3395197236 2.0885345311 3.9992682022
O 0.726442189 1.107482077 5.0095333697
O -0.5328642443 1.6103301161 2.873918386
C -0.6994386726 3.3440530364 4.9017109214
F -0.0181134878 3.8258835088 5.9481460183
F -1.0205515852 4.3574763345 4.0923318117
F -1.8233110532 2.7676595242 5.3447055271
O -1.6195135517 1.9196702273 0.0819463152
O -2.5527220424 3.91800964 -1.1729644751
O -0.8732522383 4.1419040776 0.6990272776
S -2.0097248734 3.3569075084 0.058506123
C -3.4002964208 3.5306120258 1.2895524885
F -4.4608800432 2.8366339592 0.8519464803
F -3.0593407104 3.069335554 2.4960717743
F -3.7455906094 4.818788433 1.403882975
C 1.1587860397 -2.6495466558 1.4682524187
C 0.4162980309 -1.4810828653 1.3393220713
C 1.6083991706 -0.6482580531 1.43219969
C 2.4691485012 -1.9049819291 1.5819749372
O 3.6377876292 -2.1759455663 1.7209681599
O 1.8649494454 0.5539505191 1.4186828487
O 0.7249439392 -3.8875295744 1.4645318254
C 1.6406734895 -5.0528322481 1.5726804861
C -1.0189243069 -1.192813377 1.1381570929
H -1.6182361069 -2.105140176 1.2069788742
H -1.1564257776 -0.7407580824 0.1485510873
H -1.3650345477 -0.4560405959 1.8703316923
C 2.6043533109 -5.0490572043 0.3842157801
H 3.1844057106 -5.9788585703 0.3846368053
H 2.0520374628 -4.9897605027 -0.559280351
H 3.3066073749 -4.214029787 0.4441121919
C 0.6706404462 -6.2327926125 1.5036464535
H 1.2246891424 -7.1744845939 1.5738663828
H -0.0494447319 -6.1903727707 2.3271358732
H 0.117999901 -6.2234177711 0.5589017147
C 2.3668643643 -5.0011893769 2.9185333606
H 3.0659315839 -4.1622460379 2.9601079884
H 1.6486571108 -4.9176650931 3.7411646589
H 2.9396388759 -5.9250377935 3.0576776015
C 2.5645406466 4.2676681251 0.7315157538
C 3.1559461513 4.9885152499 1.7955311385
C 3.0123107308 4.629532879 -0.5620677403
H 2.867741003 4.7599290717 2.8203531614
H 2.6120641607 4.1124285836 -1.4329639171
C 4.1121651055 5.9897928336 1.5939514307
C 3.9668191907 5.6287942411 -0.7822174447
H 4.5393275957 6.5161348936 2.4473683885
H 4.2788616723 5.8701091006 -1.7978761674
C 4.521550203 6.3157717271 0.2993066951
H 5.2650673262 7.094460563 0.1353443696
O 0.5119748346 1.0735706907 -3.3239044549
S 1.2309726617 0.8085456691 -2.0806478427
C 3.0342668875 0.8409967953 -2.5331828037
O 1.0384152578 -0.4989631473 -1.4259775293
O 1.1813837302 1.9669263458 -1.0939588658
F 3.8012538407 0.6798345437 -1.4465638034
F 3.3549936652 2.0012054196 -3.1180797953
F 3.3023071185 -0.1567290792 -3.3918695608

Frequency: 7.8425; 11.3302; 13.1914; 17.5160; 21.3056; 23.6816; 25.4524; 28.2687; 29.1494; 32.1564; 34.0852; 42.9303; 45.2792; 53.5786; 55.8849; 61.6358; 68.0387; 71.4753; 75.6090; 76.1718; 80.1888; 87.8850; 93.6840; 106.4218; 109.4271; 112.3603; 131.6699; 133.2154; 138.6682; 149.0924; 158.8904; 162.9685; 171.6703; 181.3418; 193.4055; 195.4635; 201.6478; 209.6805; 215.1560; 215.9958; 219.6111; 223.9074; 234.0337; 237.8716; 258.0377; 273.9887; 275.9077; 302.3133; 305.2318; 307.8446; 317.0390; 323.9208; 327.4779; 332.5958; 346.8853; 348.2484; 350.2143; 353.8500; 359.6250; 396.2818; 404.7656; 445.1734; 454.3295; 479.3938; 490.8445; 491.5865; 492.6878; 494.9980; 506.2748; 522.1669; 526.2726; 556.9763; 558.1004; 558.5942; 569.2046; 570.2873; 573.9366; 603.1959; 607.2475; 612.5895; 621.6372; 630.0765; 634.4182; 642.5506; 650.8688; 717.6348; 738.1106; 750.3595; 758.7416; 760.2125; 761.6274; 770.7292; 789.2127; 866.2172; 869.1145; 916.5289; 936.5616; 943.3189; 964.1014; 973.2043; 974.3204; 977.3617; 986.4838; 987.5671; 988.3497; 1012.0607; 1013.7742; 1034.9183; 1050.8713; 1070.1746; 1071.2429; 1085.9751; 1088.4263; 1102.6606; 1118.7000; 1129.7960; 1150.7463; 1184.4509; 1209.2145; 1216.0811; 1217.0193; 1224.5520; 1227.5480; 1228.3053; 1229.2062; 1232.5369; 1234.4260; 1242.8588; 1248.6242; 1262.8714; 1286.5888; 1294.1306; 1301.1200; 1305.1299; 1309.5158; 1317.2779; 1370.4622; 1402.4959; 1432.3561; 1434.0249; 1439.2180; 1458.8584; 1460.5212; 1499.9159; 1503.9830; 1505.7191; 1518.2949; 1518.9346; 1522.3423; 1535.4940; 1537.9926; 1555.1150; 1614.9112; 1623.6485; 1628.2881; 1802.1981; 1875.7034; 3058.6573; 3059.8737; 3060.9978; 3066.9857; 3124.4141; 3127.4413; 3128.3723; 3137.0327; 3138.5799; 3138.8939; 3148.4245; 3148.6156; 3156.9837; 3160.8704; 3165.1141; 3171.5414; 3178.6901.

Cartesian Forces: Max 0.000000823 RMS 0.000000372

Item	Value	Threshold	Converged?
Maximum Force	0.000000	0.000450	YES
RMS Force	0.000000	0.000300	YES
Maximum Displacement	0.000020	0.001800	YES
RMS Displacement	0.000005	0.001200	YES

FILE: Yb_Sq2_PhYbOTf3_GS_Anion_B3LYP_M062x_Add56_Conf4-1_Y.out

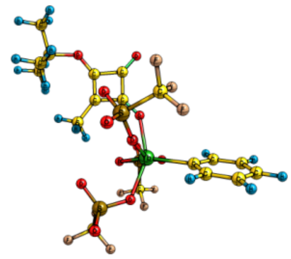

Conformer 17

E(RB3LYP/6-31G(d)/MWB59) = -3731.46010533
Convg = 0.7127D-08 -V/T = 2.0122

Temperature 195.150 Kelvin. Pressure 1.00000 Atm.
elec. and zero-point Energies= -3731.088825
elec. and thermal Energies= -3731.064537
elec. and thermal Enthalpies= -3731.063919
elec. and thermal Free Energies= -3731.138856

RB3LYP/6-31G(d)/MWB59/THF(IEFPCM) = -3731.52869368

RM06-2x/6-311+G(d,p)/MWB59/THF(IEFPCM) = -3731.36713388

Geometry at RB3LYP/6-31G(d)/MWB59
Charge = -1 Multiplicity = 1
Yb 0.8608536675 2.0900210553 1.241174603
O 1.2863020588 2.5308670058 3.5958543441
S 0.0824549936 1.8549062881 4.1986729419
O 0.3040795504 0.9567801229 5.3274476226
O -0.728143223 1.3292081156 3.0468334661
C -0.9231602882 3.2698057218 4.8753347354
F -0.299271458 3.7968643623 5.9356630088
F -1.0776283281 4.2201491353 3.9492977445
F -2.1271117508 2.8286345004 5.2553070794
O -2.3334281714 1.4674389719 0.0899833531
O -2.4931618038 3.6213441914 -1.2267005574
O -0.8487455954 3.3766736471 0.6390411144
S -2.178461437 2.9294069265 0.0206470419
C -3.3874327388 3.6137425111 1.2614689821
F -4.6383659035 3.3855663471 0.8307221849
F -3.242181362 3.0313784947 2.4583160369
F -3.2201922867 4.9341229794 1.4044150612
C 0.8985775493 -3.1667046486 1.5210389103
C 0.1926056536 -1.9617423937 1.5168432959
C 1.4101857811 -1.2057480265 1.7967019198
C 2.1989640046 -2.512179 1.9114871093
O 3.3125082014 -2.8799103954 2.1778439532
O 1.7003725274 -0.0092417158 1.8488364404
O 0.7548053549 -4.4452156047 1.2844310978
C -0.4149361401 -5.079234797 0.6266329912
C -1.1710042993 -1.4315345204 1.2706473116
H -1.9488313435 -2.1965222421 1.2960310992
H -1.2009448941 -0.9378862854 0.2913023647
H -1.4065044321 -0.6603021861 2.0108890751
C -1.5708336615 -5.1339488721 1.6277284116
H -2.4218641721 -5.6594313345 1.1806460845
H -1.2687276371 -5.6734923535 2.5313042219
H -1.9089008183 -4.1371701375 1.9198435574
C 0.1109334258 -6.4856755648 0.3287760897
H -0.6781102192 -7.0892269715 -0.1318430835
H 0.9614284746 -6.4398654751 -0.3580167039
H 0.4356617929 -6.9789957899 1.2503462385
C -0.7530719612 -4.3407118436 -0.6696252155
H -1.159353379 -3.343090503 -0.4994442498
H 0.137130906 -4.2340401645 -1.2963008617
H -1.5012665325 -4.9177930087 -1.224256646
C 2.6623414115 3.5914237046 0.9141397437
C 3.5978048619 3.9659484517 1.9050164877
C 2.8464618728 4.1880456484 -0.3550467394
H 3.5087102615 3.5505616697 2.9083205672
H 2.163367045 3.9457390516 -1.1703004727
C 4.6427436283 4.8614596732 1.6539208013
C 3.8813199605 5.0912960105 -0.6218116065
H 5.3434888014 5.1198601077 2.4472479064
H 3.9826643055 5.5285962451 -1.6142419539
C 4.7867634537 5.4295376777 0.3859387457
H 5.5963962124 6.1294490012 0.1853733617
O 0.7705855775 0.3533927953 -3.1225483663
S 1.2996369665 0.0877195375 -1.7880121671
C 3.1444474146 0.2868288157 -1.9237395661
O 1.1141276713 -1.2596685559 -1.2169877976
O 0.9655662397 1.1822874355 -0.7819917242
F 3.7182742091 0.1746039624 -0.7178382107
F 3.4547130335 1.4782722931 -2.4412119338
F 3.640941968 -0.6711770672 -2.7220042499

Frequency: 8.6908; 10.7355; 14.2861; 17.6096; 18.0700; 21.3592; 24.7008; 26.8844; 28.5636; 33.8860; 36.9476; 38.6439; 41.7400; 47.6425; 53.0162; 54.2256; 56.9144; 61.9191; 63.6961; 68.5692; 73.6979; 79.3202; 93.6263; 105.2608; 108.8390; 116.8294; 129.0874; 139.4542; 141.8307; 144.4391; 162.4885; 172.1626; 177.7671; 184.0510; 190.3175; 194.2340; 195.2684; 196.6990; 208.7790; 210.2407; 212.4194; 217.0646; 226.3080; 239.0253; 245.6682; 259.8875; 274.5184; 297.4091; 300.5184; 303.9321; 308.1757; 324.8397; 329.6964; 331.8735; 338.3075; 350.7611; 352.1739; 364.0129; 365.3207; 393.6119; 400.2843; 443.8836; 449.0950; 481.3885; 487.2228; 490.4740; 495.2595; 497.9277; 506.7247; 514.9069; 524.3289; 556.2555; 557.6025; 558.2162; 567.8320; 572.3959; 573.7598; 598.4805; 609.9310; 612.3436; 620.5274; 626.7352; 633.3551; 652.3462; 652.9732; 716.2490; 736.0320; 758.4219; 760.4260; 761.8638; 769.9097; 777.7561; 786.3423; 846.1772; 868.7135; 913.8198; 932.1852; 936.5229; 950.8931; 970.2041; 975.2703; 976.5337; 980.1425; 985.5046; 986.3202; 1011.6979; 1014.1934; 1036.9539; 1038.6609; 1061.3663; 1065.4264; 1085.4129; 1088.7638; 1104.3692; 1119.7835; 1139.6770; 1152.1142; 1185.3083; 1205.0346; 1215.0882; 1218.3975; 1221.8546; 1224.3301; 1224.7643; 1226.1274; 1228.3116; 1231.2089; 1241.0164; 1244.5784; 1246.9093; 1283.8513; 1294.2850; 1297.6176; 1305.2580; 1313.2649; 1321.5554; 1370.0978; 1396.9502; 1432.3563; 1432.7585; 1437.2612; 1459.6510; 1460.8826; 1499.6543; 1505.9120; 1509.0448; 1514.6446; 1520.3863; 1523.3081; 1528.7769; 1531.4168; 1547.1369; 1616.0272; 1616.7587; 1628.7235; 1799.9633; 1899.2096; 3061.5291; 3065.6834; 3067.4760; 3075.4471; 3128.1702; 3128.7931; 3135.5451; 3135.9739; 3140.4424; 3143.4895; 3144.2233; 3155.4589; 3156.7253; 3165.5206; 3166.5538; 3179.0504; 3183.5499.

Cartesian Forces: Max 0.000001563 RMS 0.000000305

Item	Value	Threshold	Converged?
Maximum Force	0.000001	0.000450	YES
RMS Force	0.000000	0.000300	YES
Maximum Displacement	0.000157	0.001800	YES
RMS Displacement	0.000028	0.001200	YES

FILE: Yb_Sq2_PhYbOTf3_GS_Anion_B3LYP_M062x_Add56_Conf4-1-_x.out

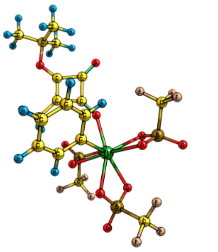

Conformer 18

E(RB3LYP/6-31G(d)/MWB59) = -3731.46851150
Convg = 0.4556D-08 -V/T = 2.0122

Temperature 195.150 Kelvin. Pressure 1.00000 Atm.
elec. and zero-point Energies= -3731.096774
elec. and thermal Energies= -3731.072941
elec. and thermal Enthalpies= -3731.072323
elec. and thermal Free Energies= -3731.144847

RB3LYP/6-31G(d)/MWB59/THF(IEFPCM) = -3731.53478195

RM06-2x/6-311+G(d,p)/MWB59/THF(IEFPCM) = -3731.36860426

Geometry at RB3LYP/6-31G(d)/MWB59
Charge = -1 Multiplicity = 1
Yb 0.4904248943 3.0325556739 0.7603718762
O 2.8645381321 3.4397632037 1.3332130126
S 3.2647685812 3.9927572825 -0.0027824789
O 4.0342298445 5.2308110057 -0.036295935
O 2.035696047 3.9075345686 -0.8744210419
C 4.4047815509 2.7074853293 -0.7249830485
F 5.5016796105 2.5952502311 0.0330450052
F 3.8021409519 1.512221781 -0.7871085164
F 4.7693748966 3.0664104841 -1.9623256124
O 0.3299588222 5.3277311536 1.6294639357
O -1.9719014272 6.3938883739 1.5636360147
O -1.2166153697 4.5868244542 -0.0495912959
S -0.8790267427 5.7541172855 0.8385601421
C -0.2533496609 7.0698565818 -0.3241122538
F 0.1872836583 8.1176292154 0.3843975706
F 0.7384836 6.6122870349 -1.0861969574
F -1.2569148605 7.476262626 -1.1145681114
C 0.8809424809 -2.3855788631 0.9853737262
C 0.0640361916 -1.2633538114 1.0540977321
C 1.2149504905 -0.356588217 1.0726297247
C 2.1531550124 -1.5707813833 1.0019918184
O 3.3425452937 -1.781035521 0.9721573526
O 1.4095699494 0.8495444687 1.1198588598
O 0.516457659 -3.6467120067 0.926450203
C 1.4989397704 -4.7558499468 0.8399265328
C -1.4014528811 -1.0765689361 1.0966976423
H -1.9203134043 -2.0354578473 1.0064756444
H -1.7151938883 -0.4036437709 0.2895485555
H -1.681408784 -0.5920273583 2.0396791951
C 2.3157809891 -4.6100057934 -0.4458949073
H 2.9432735166 -5.4982696889 -0.5807630431
H 1.6542115323 -4.5236577221 -1.314283182
H 2.9723946298 -3.7375963762 -0.402128889
C 0.5932566548 -5.9870662928 0.790763347
H 1.2016803371 -6.8949323788 0.7251376191
H -0.0261420989 -6.047286158 1.6913481684
H -0.0668716447 -5.9463001613 -0.0814703626
C 2.3756041138 -4.7548664739 2.094245677
H 3.0296002957 -3.8797262178 2.1202014191
H 1.7557820715 -4.7728634776 2.9969764905
H 3.0085227075 -5.6494937059 2.0963659486
C -0.392518749 1.7714597204 -1.1206086264
C 0.4020848522 1.0248834745 -2.0208352831
C -1.78381007 1.7158885522 -1.3717003156
H 1.4876388746 1.0317404215 -1.9101948516
H -2.4622895185 2.2815875002 -0.7329767194
C -0.1338633955 0.2740150162 -3.0747432816
C -2.3423896782 0.9685055173 -2.4170275335
H 0.5262354033 -0.2783982078 -3.7439962948
H -3.4221556495 0.9594822693 -2.566023219
C -1.5156962138 0.2384218092 -3.2748281044
H -1.9416008641 -0.3426785361 -4.0914820195
O -0.9767703236 0.7499970623 4.0063351011
S -0.7769169494 2.0688707835 3.3960929967
C -1.6980594944 3.2486967048 4.5038143473
O -1.4090838179 2.2616763943 2.0371286609
O 0.6171156452 2.5931342077 3.3106716704
F -1.6170292325 4.494726048 4.0450551099
F -1.1751596898 3.1988503333 5.7366192143
F -2.9867740287 2.887223585 4.5680123592

Frequency: 7.0783; 13.3866; 16.0542; 23.5434; 25.6086; 28.5225; 31.4415; 34.0819; 34.9544; 37.4556; 41.1191; 43.2518; 49.0792; 49.4755; 51.9228; 59.8579; 63.6431; 69.8735; 76.3330; 79.9375; 83.7947; 86.2664; 100.1929; 110.5037; 129.0619; 133.3142; 137.6202; 143.8786; 149.6814; 154.3616; 163.6315; 172.3735; 175.3117; 177.6534; 183.1217; 207.5985; 210.2230; 211.3278; 212.4704; 215.8731; 217.7474; 218.5432; 219.2862; 235.9391; 258.3302; 274.9214; 275.8146; 302.5829; 306.2393; 307.0428; 311.8318; 321.5326; 323.0387; 324.6375; 344.1997; 346.8214; 350.9193; 352.3501; 357.7044; 392.6924; 401.4449; 454.1435; 456.7869; 483.4457; 489.5099; 489.5827; 491.5018; 494.7716; 522.0479; 524.9309; 528.8178; 556.9256; 557.9532; 558.7396; 569.9792; 572.6605; 574.0611; 601.9631; 605.2430; 624.8090; 627.1317; 631.0945; 635.1456; 641.9597; 645.6691; 720.9837; 735.5648; 747.2842; 759.2881; 762.0851; 763.3166; 771.3503; 788.6749; 866.3995; 872.0759; 911.0828; 936.2373; 943.2529; 973.1294; 975.6308; 977.5184; 978.5976; 984.7930; 985.2285; 988.3824; 1004.8989; 1011.5987; 1032.0947; 1063.8759; 1069.1510; 1071.1296; 1083.3614; 1084.2121; 1099.1490; 1112.8939; 1119.2645; 1142.8323; 1182.3823; 1210.0616; 1212.4666; 1214.6422; 1216.7064; 1223.5690; 1228.0988; 1231.5905; 1233.2849; 1237.8187; 1253.1251; 1254.0604; 1254.4570; 1283.2060; 1286.1400; 1293.5451; 1304.4658; 1305.3927; 1313.3211; 1369.7273; 1399.5947; 1432.1246; 1433.5712; 1443.5517; 1457.9344; 1460.0216; 1497.8814; 1504.0312; 1507.8875; 1518.6994; 1518.8701; 1521.0931; 1535.4648; 1538.2639; 1555.4060; 1612.7108; 1623.0723; 1626.5782; 1815.0619; 1873.2078; 3048.9390; 3058.5905; 3059.1098; 3066.5394; 3114.6662; 3124.3283; 3126.0226; 3129.4054; 3137.2368; 3138.1049; 3138.4082; 3139.4751; 3149.3908; 3156.4975; 3157.7793; 3160.0608; 3173.3927.

Cartesian Forces: Max 0.000002149 RMS 0.000000507

Item	Value	Threshold	Converged?
Maximum Force	0.000002	0.000450	YES
RMS Force	0.000000	0.000300	YES
Maximum Displacement	0.001717	0.001800	YES
RMS Displacement	0.000401	0.001200	YES

FILE: Yb_Sq2_PhYbOTf3_GS_Anion_B3LYP_M062x_Add56_Conf5.out

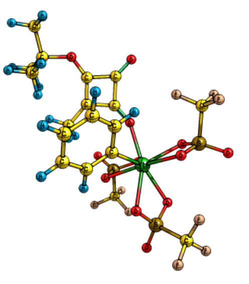

Conformer 19

E(RB3LYP/6-31G(d)/MWB59) = -3731.45944553
Convg = 0.7959D-08 -V/T = 2.0122

Temperature 195.150 Kelvin. Pressure 1.00000 Atm.
elec. and zero-point Energies= -3731.087893
elec. and thermal Energies= -3731.064046
elec. and thermal Enthalpies= -3731.063428
elec. and thermal Free Energies= -3731.135610

RB3LYP/6-31G(d)/MWB59/THF(IEFPCM) = -3731.52669864

RM06-2x/6-311+G(d,p)/MWB59/THF(IEFPCM) = -3731.36509444

Geometry at RB3LYP/6-31G(d)/MWB59
Charge = -1 Multiplicity = 1
Yb 0.514587744 2.7168906231 0.817361856
O 2.9262012849 2.9674158036 1.3088326877
S 3.3323525037 3.3967712305 -0.0702545701
O 4.1980698888 4.5623365252 -0.2039518687
O 2.0742254762 3.3597033601 -0.9044681861
C 4.3405289978 1.9764844289 -0.7348465133
F 5.4302718995 1.8014320459 0.0205397921
F 3.6287459582 0.8419583503 -0.7347619372
F 4.7221024136 2.2411959771 -1.9908525243
O 0.5643964169 5.0533858985 1.5665374697
O -1.6385359108 6.3112921806 1.4939157695
O -1.0825337445 4.3655322988 -0.0373646691
S -0.6239847112 5.5428658806 0.7801450069
C 0.0834342596 6.7382902237 -0.4627715503
F 0.6308679499 7.7778485806 0.1800603906
F 1.0121541234 6.1568351137 -1.219854274
F -0.9009031734 7.1904570626 -1.2525905037
C 0.6540822463 -2.6655456566 0.5625118335
C -0.1072742691 -1.584100736 1.004359492
C 1.0744293105 -0.709332009 1.0290847609
C 1.9534010123 -1.9027034356 0.62297929
O 3.1140270223 -2.1560519106 0.4343230909
O 1.2717151606 0.4812434423 1.2302034381
O 0.5504115869 -3.9116516583 0.1640278628
C -0.714677964 -4.6303572256 -0.1060038762
C -1.5084253612 -1.2277446316 1.3375714101
H -2.182530097 -2.0850902097 1.3704351835
H -1.880326843 -0.5067693269 0.5976107199
H -1.5324788293 -0.7172339407 2.307332535
C -1.4090727881 -4.9321556059 1.2243338135
H -2.3137912714 -5.5230945134 1.0436122792
H -0.7456919053 -5.5096083454 1.8764163288
H -1.7021515927 -4.022788918 1.7532102204
C -0.2172589242 -5.9231146568 -0.7584929962
H -1.0653155637 -6.5758283425 -0.9906925127
H 0.3187685114 -5.7031682881 -1.6867195708
H 0.4611560569 -6.4571444345 -0.0857997374
C -1.5738953134 -3.8282540526 -1.085954109
H -1.9378781454 -2.8885889303 -0.6676615487
H -1.0038749436 -3.5927695131 -1.9898664564
H -2.4450598027 -4.4266546546 -1.3749340503
C -0.5352716407 1.4519065317 -0.9849806635
C 0.167925636 0.6363542794 -1.9012972334
C -1.9398300895 1.4611807421 -1.1586623363
H 1.2572376745 0.591798035 -1.8524014758
H -2.5513971374 2.0840333534 -0.5056604369
C -0.4638996086 -0.1229194006 -2.894985827
C -2.5943449818 0.7070873106 -2.1417047539
H 0.1296524103 -0.7268684446 -3.5819651651
H -3.6798392764 0.750883689 -2.2315848224
C -1.8557654214 -0.0960419921 -3.0153968815
H -2.3559792863 -0.6787047837 -3.7877798463
O -1.0632460275 0.7203936729 4.1962047659
S -0.769332421 1.9835335567 3.5114044133
C -1.5550356515 3.2925988814 4.5777290197
O -1.4195038574 2.1637307416 2.1594786644
O 0.6613330287 2.3831451624 3.3651267026
F -1.3802140368 4.5023464993 4.0524208219
F -1.0049617317 3.2594507848 5.7990301075
F -2.867820254 3.0483134926 4.6900302112

Frequency: 8.0157; 16.4139; 17.3229; 22.9296; 25.0541; 27.4457; 31.5965; 34.4339; 37.3093; 38.3730; 43.2479; 44.6472; 49.2466; 52.2705; 59.3538; 61.0013; 63.9212; 70.4356; 72.8425; 76.1731; 82.9180; 91.2292; 101.7843; 111.7873; 127.2278; 132.7947; 138.7417; 139.9737; 148.5009; 155.1167; 166.7684; 175.1177; 177.7717; 180.0993; 183.4874; 204.7689; 207.9519; 209.3813; 211.4205; 212.6419; 215.3265; 218.5781; 219.4077; 230.5383; 241.9089; 259.7466; 274.5651; 290.0495; 303.3212; 306.5450; 309.5054; 321.4657; 323.0837; 324.4324; 344.3269; 351.2213; 352.3963; 354.7010; 358.0973; 392.9564; 398.9242; 450.8865; 458.0155; 483.0352; 489.3445; 489.5413; 490.8866; 497.3168; 522.3317; 524.9479; 528.9369; 556.9183; 557.9343; 558.7567; 570.1750; 572.6992; 574.1957; 594.6613; 606.4814; 624.8191; 627.1195; 631.5121; 634.9190; 644.5680; 652.3012; 721.6443; 735.5891; 759.6329; 762.0669; 763.1643; 767.5937; 775.9556; 784.0884; 848.7353; 872.0122; 909.7673; 931.1470; 935.4518; 975.4213; 975.9885; 976.7906; 978.3598; 978.7661; 984.4536; 985.4392; 1006.6161; 1010.9949; 1030.9318; 1059.3780; 1062.6949; 1063.9325; 1082.2448; 1082.8654; 1098.4140; 1112.0189; 1117.7971; 1141.7419; 1181.5345; 1206.9697; 1209.8185; 1212.3541; 1214.3204; 1215.4640; 1223.1194; 1229.1965; 1231.8393; 1233.5898; 1239.2312; 1253.0598; 1254.2559; 1283.6014; 1284.3294; 1292.5729; 1293.1275; 1305.4990; 1313.2755; 1369.1080; 1387.9700; 1432.2864; 1433.9413; 1445.9590; 1457.3179; 1459.6923; 1499.0091; 1505.2541; 1509.0652; 1516.9525; 1519.8912; 1520.6460; 1528.9191; 1531.3124; 1548.3724; 1611.0832; 1621.0021; 1623.9958; 1820.1733; 1895.4080; 3047.7763; 3061.4254; 3066.0283; 3073.5659; 3108.4728; 3128.4591; 3128.5388; 3133.0920; 3137.8519; 3139.1669; 3142.1778; 3148.3345; 3154.3860; 3157.2529; 3165.5417; 3172.0381; 3177.5475.

Cartesian Forces: Max 0.000000635 RMS 0.000000244

Item	Value	Threshold	Converged?
Maximum Force	0.000001	0.000450	YES
RMS Force	0.000000	0.000300	YES
Maximum Displacement	0.000180	0.001800	YES
RMS Displacement	0.000028	0.001200	YES

FILE: Yb_Sq2_PhYbOTf3_GS_Anion_B3LYP_M062x_Add56_Conf5-.out

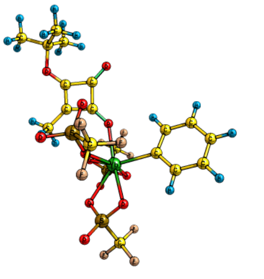

Conformer 20

E(RB3LYP/6-31G(d)/MWB59) = -3731.47014610
Convg = 0.2397D-09 -V/T = 2.0122

Temperature 195.150 Kelvin. Pressure 1.00000 Atm.
elec. and zero-point Energies= -3731.098268
elec. and thermal Energies= -3731.074269
elec. and thermal Enthalpies= -3731.073651
elec. and thermal Free Energies= -3731.148179

RB3LYP/6-31G(d)/MWB59/THF(IEFPCM) = -3731.53637318

RM06-2x/6-311+G(d,p)/MWB59/THF(IEFPCM) = -3731.36979294

Geometry at RB3LYP/6-31G(d)/MWB59
Charge = -1 Multiplicity = 1
Yb 0.4944697091 2.8240536358 1.6521457376
O -1.5891571943 1.9816414298 0.3610955098
S -2.541407823 2.8191201492 1.1617208297
O -3.5347712419 3.6105101442 0.4463183414
O -1.7219120991 3.5201987338 2.2186050574
C -3.5290070169 1.5795758602 2.1403074488
F -4.2194834174 0.7922159164 1.301996397
F -2.7236029583 0.8076747952 2.8809626317
F -4.3881609813 2.2073382533 2.9489537785
O 0.1158081775 4.4003557803 -0.2282458629
O 2.0396101438 5.9695605972 -0.7640062256
O 1.6824232206 4.9179565659 1.5188194685
S 1.1086473058 5.4351597842 0.2214143235
C 0.0666206277 6.8859056106 0.7515993725
F -0.6198655178 7.3570394717 -0.2954728561
F -0.7921380005 6.5235960521 1.7084323474
F 0.8562166127 7.8587509643 1.2249341062
C 0.4390738177 -2.2939990478 0.3190704577
C 0.2555904861 -0.9576634573 -0.0163301762
C 0.3014593311 -0.5427125943 1.3842571936
C 0.4619980923 -2.006806188 1.8032851475
O 0.5458946328 -2.6362830079 2.8300000926
O 0.2377085533 0.5062510696 2.0177752265
O 0.5371478202 -3.3224318994 -0.4880230058
C 0.8109586603 -4.6981878668 0.0044125303
C 0.1207906432 -0.2517017009 -1.3117380186
H -0.1818164566 -0.9474524985 -2.1006725581
H 1.0852273656 0.1901098454 -1.5972708943
H -0.6043952847 0.5632695569 -1.2317070779
C 2.1500660617 -4.7107100925 0.7443866131
H 2.4254035454 -5.7455702616 0.9773649255
H 2.9384443767 -4.2764197367 0.1213977784
H 2.0921957157 -4.1576185555 1.6848949371
C 0.8823504001 -5.4977760938 -1.2969110455
H 1.0805580695 -6.551520799 -1.0754692932
H -0.0624356901 -5.4293098387 -1.845862331
H 1.684408948 -5.119587293 -1.9383917711
C -0.3527160743 -5.159298917 0.884382969
H -0.3963165644 -4.5867216117 1.8142494056
H -1.3034535523 -5.0602245266 0.3494313519
H -0.2157393937 -6.2152071687 1.143753118
C 1.1236345314 2.9204231218 3.9590264143
C 1.7185031515 4.0642683405 4.5428782186
C 0.8785708172 1.8510345011 4.8537880869
H 1.9492162409 4.9241060195 3.9159546882
H 0.4345375834 0.929513508 4.4792379021
C 2.0391104954 4.1434289082 5.9025085882
C 1.1908609469 1.9126793711 6.2165015012
H 2.497876839 5.0473266672 6.3027846638
H 0.9829938466 1.0601445233 6.8626829901
C 1.7737316209 3.0644964715 6.7482564085
H 2.0208240349 3.1194713958 7.8075112241
O 3.232512294 -0.192033975 0.9973159616
S 3.322150114 1.0262280003 0.1764195225
C 4.7466791581 1.9962855558 0.8736540443
O 2.1689057928 1.9883534171 0.4242508987
O 3.6039818363 0.862374419 -1.2537291334
F 4.9059367625 3.139857138 0.2024837946
F 5.8737306274 1.2741364123 0.7685982861
F 4.534220362 2.2741866474 2.1636870052

Frequency: 5.1989; 8.1563; 14.5122; 17.9243; 18.9817; 20.6263; 24.3492; 27.4623; 29.9780; 31.8398; 33.6963; 37.0311; 43.0250; 44.1064; 53.5669; 56.4068; 63.5372; 68.4897; 72.7762; 81.2385; 87.4696; 89.1589; 99.1291; 106.0532; 114.9220; 126.5168; 138.2684; 148.8023; 153.7036; 159.5598; 163.2045; 172.9478; 178.1656; 178.8609; 193.5116; 194.6360; 209.1046; 210.5083; 211.9779; 214.9007; 218.1265; 219.5891; 223.2511; 244.8042; 259.1843; 271.6718; 277.5503; 302.1677; 304.5809; 307.4212; 321.6052; 323.2156; 326.2681; 330.9828; 341.3888; 347.3482; 349.8774; 353.3468; 357.0867; 395.9753; 403.5832; 446.0835; 454.6371; 487.5861; 489.0040; 489.8328; 490.3249; 494.9007; 507.6581; 522.6377; 527.0825; 555.7226; 557.7174; 557.9870; 568.4717; 570.8318; 575.3003; 599.7728; 609.2939; 619.5254; 622.5451; 627.1327; 634.6502; 643.5197; 649.8985; 717.6785; 738.0193; 748.6912; 759.4113; 761.2924; 762.4635; 773.8439; 790.4424; 864.6195; 868.7891; 916.2297; 936.5799; 942.8589; 973.3089; 974.2558; 974.6451; 976.2322; 986.5819; 987.1587; 988.3140; 1010.6842; 1012.0436; 1034.4595; 1065.1295; 1069.5352; 1071.6029; 1086.0045; 1087.8675; 1103.0053; 1112.8794; 1123.1154; 1151.3477; 1184.2169; 1208.8221; 1214.9142; 1217.0891; 1225.5561; 1227.8342; 1228.5854; 1234.5132; 1237.4139; 1240.9415; 1242.7338; 1243.7380; 1257.3190; 1286.6412; 1293.0705; 1298.1980; 1304.6359; 1308.8971; 1316.2737; 1370.1270; 1405.6530; 1432.2546; 1434.4785; 1449.1650; 1458.4639; 1460.8351; 1503.6883; 1506.2735; 1514.8571; 1517.3892; 1519.2892; 1521.8866; 1535.2848; 1538.0140; 1554.8304; 1614.0841; 1624.3409; 1628.1343; 1805.9133; 1876.4912; 3048.2099; 3057.6305; 3060.4475; 3067.2508; 3113.6660; 3123.0210; 3129.1791; 3136.2775; 3137.5382; 3139.8592; 3146.5331; 3151.3700; 3156.2330; 3161.7794; 3161.8849; 3169.6206; 3177.6282.

Cartesian Forces: Max 0.000001811 RMS 0.000000589

Item	Value	Threshold	Converged?
Maximum Force	0.000000	0.000450	YES
RMS Force	0.000000	0.000300	YES
Maximum Displacement	0.000081	0.001800	YES
RMS Displacement	0.000013	0.001200	YES

FILE: Yb_Sq2_PhYbOTf3_GS_Anion_B3LYP_M062x_Add56_Conf5-1_Y.out

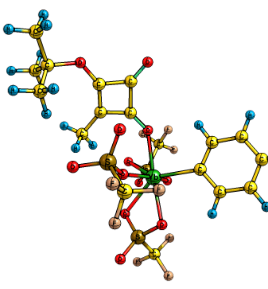

Conformer 21

E(RB3LYP/6-31G(d)/MWB59) = -3731.46213520
Convg = 0.2445D-08 -V/T = 2.0122

Temperature 195.150 Kelvin. Pressure 1.00000 Atm.
elec. and zero-point Energies= -3731.090413
elec. and thermal Energies= -3731.066388
elec. and thermal Enthalpies= -3731.065770
elec. and thermal Free Energies= -3731.139450

RB3LYP/6-31G(d)/MWB59/THF(IEFPCM) = -3731.52850539

RM06-2x/6-311+G(d,p)/MWB59/THF(IEFPCM) = -3731.36568459

Geometry at RB3LYP/6-31G(d)/MWB59
Charge = -1 Multiplicity = 1
Yb 0.1438470631 2.4053398482 1.7483914871
O -2.229051454 2.1260462662 0.7963881504
S -2.785679995 3.2954109304 1.5545980114
O -3.5998586908 4.2629973614 0.8296681881
O -1.6621322783 3.8292896177 2.4099112595
C -3.9386495242 2.5236914778 2.7969212405
F -4.9301010469 1.8876538531 2.155959606
F -3.2867907974 1.6382777656 3.5598532962
F -4.4675069392 3.466096051 3.5834643653
O 0.0233295868 3.8138938263 -0.3059675143
O 2.2498842441 4.7096180782 -1.1353120768
O 1.806545894 4.0829928704 1.2838296937
S 1.2900726554 4.5788746611 -0.0462908597
C 0.7277890089 6.3172574723 0.320221756
F 0.1131304938 6.8278350443 -0.7530968021
F -0.1158035012 6.3339714284 1.3546112995
F 1.7890051162 7.0798062921 0.6147365546
C -0.9635644935 -2.6789200715 0.9757337978
C -1.0587182219 -1.3648556685 0.5126793499
C -0.8616988333 -0.8341438841 1.8639954884
C -0.8846172919 -2.2604047344 2.4236055841
O -0.8776973513 -2.8163741221 3.4893592552
O -0.6839008151 0.2783427117 2.3537023095
O -0.9180856061 -3.917285977 0.5604437286
C -0.6557327144 -4.3655746119 -0.8327348596
C -1.2393272568 -0.6268578101 -0.7661171953
H -1.8140258124 -1.1972777919 -1.5004005497
H -0.266761971 -0.3781079395 -1.2093241462
H -1.7570366211 0.3170003371 -0.5699459399
C -1.9427252813 -4.2133729009 -1.6453170728
H -1.7933045014 -4.620607433 -2.6514198239
H -2.7641893205 -4.7600213621 -1.170519993
H -2.2379390395 -3.1667690687 -1.7497486382
C -0.3145047736 -5.8438608323 -0.6279707378
H -0.1379953989 -6.3232790937 -1.5965059519
H 0.5874308432 -5.9475380754 -0.0174811041
H -1.1358858086 -6.363737881 -0.1246053998
C 0.5352451712 -3.6067187276 -1.4172868414
H 0.3159942775 -2.5618680084 -1.6387387743
H 1.3845843358 -3.6273936208 -0.7284497658
H 0.8381240407 -4.0872219591 -2.3542922868
C 1.0323967519 2.4932321212 3.9652939373
C 1.9296994419 3.5054375828 4.3818995713
C 0.6639790993 1.5679951385 4.9714505473
H 2.2693330639 4.2473484544 3.6607090107
H -0.0147017499 0.751638005 4.7272847925
C 2.4173667596 3.5962957303 5.6900326094
C 1.1412228272 1.6434987265 6.2848338809
H 3.1078678235 4.3949119696 5.9603904555
H 0.82839561 0.9042447706 7.0217169363
C 2.0221497264 2.6629176783 6.6505775813
H 2.3984899308 2.7274336585 7.670506625
O 2.1717209159 -1.2318750605 1.1616031913
S 2.3147670354 -0.1393162055 0.1866583261
C 4.0001320428 0.5888171971 0.4816816787
O 1.4219962583 1.0511907445 0.4951996639
O 2.2749870998 -0.5006610096 -1.2378451518
F 4.2213047265 1.6051343399 -0.3545097283
F 4.9337457807 -0.3536876599 0.2733930388
F 4.1042649432 1.025659843 1.7398202434

Frequency: 7.8315; 13.0347; 18.5994; 19.9011; 22.2191; 24.5650; 26.0843; 27.4856; 29.8115; 31.6013; 33.9368; 37.0858; 43.0869; 44.7677; 53.1854; 57.3044; 63.2374; 66.5794; 71.8681; 77.2341; 78.7691; 87.7282; 96.3710; 113.6188; 124.9030; 127.3536; 138.2684; 147.5864; 150.8513; 154.2592; 169.2352; 174.5871; 178.1643; 184.7084; 191.3939; 193.5975; 195.0498; 207.7525; 210.8223; 213.2994; 214.2678; 220.4965; 222.4274; 234.9994; 249.8775; 263.5056; 278.4900; 298.6916; 303.6006; 306.5477; 307.6512; 322.1177; 324.9732; 330.6873; 339.5947; 347.7710; 352.5459; 355.6174; 360.6418; 396.3141; 398.4597; 446.4687; 449.9181; 480.3333; 486.7027; 489.5446; 490.3718; 500.2451; 507.2097; 522.8411; 526.9085; 555.3898; 557.7349; 558.1733; 568.4663; 571.0724; 574.6593; 596.2970; 612.1982; 620.1954; 622.5771; 627.4271; 634.6133; 648.5328; 650.3885; 718.4623; 738.4091; 759.8984; 761.4643; 763.0878; 769.3061; 782.2579; 789.3513; 843.1389; 869.5627; 917.3648; 930.1841; 937.6013; 974.6021; 975.0434; 975.4408; 976.7521; 980.8076; 987.7850; 988.3989; 1008.1192; 1011.9148; 1034.7662; 1058.4215; 1065.1231; 1066.5975; 1086.2487; 1088.0868; 1098.1699; 1111.9021; 1122.3142; 1151.1761; 1184.3946; 1202.7726; 1212.5778; 1214.4590; 1216.8551; 1224.0360; 1227.8836; 1231.0205; 1235.6320; 1237.3888; 1241.3812; 1245.1197; 1245.8141; 1282.1792; 1289.8410; 1293.0741; 1295.1662; 1308.6746; 1315.3973; 1370.3762; 1396.9981; 1431.2957; 1434.2848; 1451.4156; 1458.5139; 1459.6492; 1501.0259; 1508.4799; 1510.9512; 1515.9235; 1521.9578; 1523.2822; 1530.8071; 1532.3873; 1546.4476; 1613.8442; 1619.7053; 1628.2835; 1808.6169; 1900.0482; 3056.9766; 3061.1775; 3065.8375; 3075.8419; 3123.4512; 3127.7385; 3135.8371; 3138.2845; 3140.8902; 3145.0291; 3147.2508; 3148.6268; 3159.5083; 3162.2800; 3169.0962; 3177.6256; 3186.8222.

Cartesian Forces: Max 0.000009062 RMS 0.000001399

Item	Value	Threshold	Converged?
Maximum Force	0.000007	0.000450	YES
RMS Force	0.000001	0.000300	YES
Maximum Displacement	0.001008	0.001800	YES
RMS Displacement	0.000265	0.001200	YES

FILE: Yb_Sq2_PhYbOTf3_GS_Anion_B3LYP_M062x_Add56_Conf5-1-.out

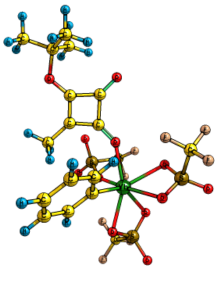

Conformer 22

E(RB3LYP/6-31G(d)/MWB59) = -3731.46961289
Convg = 0.1482D-08 -V/T = 2.0122

Temperature 195.150 Kelvin. Pressure 1.00000 Atm.
elec. and zero-point Energies= -3731.098048
elec. and thermal Energies= -3731.074035
elec. and thermal Enthalpies= -3731.073417
elec. and thermal Free Energies= -3731.147289

RB3LYP/6-31G(d)/MWB59/THF(IEFPCM) = -3731.53598178

RM06-2x/6-311+G(d,p)/MWB59/THF(IEFPCM) = -3731.37094993

Geometry at RB3LYP/6-31G(d)/MWB59
Charge = -1 Multiplicity = 1
Yb 0.3672672444 3.1155569972 0.6766595588
O 2.7312450601 3.5378622434 1.2438773572
S 3.1378244935 4.0482185315 -0.1095839976
O 3.9306864236 5.2681097898 -0.1788986405
O 1.9066186818 3.9553004408 -0.9789421488
C 4.246824881 2.7148883432 -0.792247971
F 5.3388131569 2.5946310038 -0.0295280401
F 3.6104254781 1.5347219476 -0.8201238244
F 4.6208098655 3.0271872594 -2.0386190024
O -1.3834748335 4.6332242158 0.0231868679
O -0.8176329739 7.1023486808 -0.0799928475
O 0.3462172995 5.4635005188 1.4698024355
S -0.8848527636 5.8812677195 0.7132693724
C -2.1650686635 6.2378243586 2.0213415949
F -3.3204523836 6.5699553879 1.4290225314
F -2.3695949082 5.1803835843 2.8044075903
F -1.75447665 7.2668910761 2.7735855292
C 0.7464244481 -2.1531289212 1.8442439555
C -0.0454029012 -1.0690359509 1.4843926088
C 1.1012124069 -0.1611624024 1.4802602297
C 2.012396235 -1.3315951349 1.8713942444
O 3.1861943045 -1.5132974645 2.0932566898
O 1.3229574747 1.0218442835 1.25149548
O 0.369249052 -3.3941873861 2.0494439428
C 1.3208749413 -4.4570332002 2.4609529016
C -1.4972005178 -0.919419795 1.2497294106
H -1.9842042083 -1.8964093256 1.1757331527
H -1.687921217 -0.33444824 0.3442902592
H -1.9303607629 -0.3690257958 2.0935727827
C 2.37023173 -4.648773636 1.3637772034
H 2.9917557485 -5.5190729198 1.6029799189
H 1.8880666406 -4.8290921038 0.3970194707
H 3.0258443308 -3.777843529 1.2865185912
C 0.4084635185 -5.6780689425 2.583386933
H 0.9943020331 -6.552024159 2.8862102185
H -0.3704222787 -5.5041909805 3.3325101903
H -0.074549006 -5.8974762232 1.6257746049
C 1.9427107496 -4.0849435526 3.8086944369
H 2.6069040012 -3.2222517847 3.7153513523
H 1.162576625 -3.8630853987 4.544252388
H 2.5350474929 -4.9286192848 4.1806402487
C -0.6272720236 1.7751876562 -1.064814095
C 0.1264170744 0.8865445785 -1.8667531872
C -2.0155324237 1.7916934492 -1.3350874246
H 1.2085509922 0.8318111044 -1.7365599275
H -2.659789312 2.469886816 -0.775252673
C -0.4480896656 0.0685694652 -2.846951504
C -2.6121861748 0.9759313268 -2.3050466799
H 0.1786107208 -0.594747533 -3.4429537409
H -3.6879124476 1.0232807505 -2.4723170291
C -1.8273494129 0.1056936472 -3.0655333665
H -2.283308385 -0.5289490676 -3.8237091564
O -0.4345024399 0.6737057755 3.9970272151
S -0.9314150688 2.0582906071 3.9129219576
C 0.3558058065 3.08151953 4.7840914545
O -2.2035805283 2.372439627 4.561760093
O -0.8313584358 2.6190297264 2.5008724542
F -0.0262119291 4.3597515552 4.85935246
F 1.5198354756 3.0188943315 4.1202236441
F 0.5459829291 2.6157474029 6.0270879256

Frequency: 7.5414; 11.4501; 12.4366; 16.1849; 20.6368; 25.6194; 28.8596; 29.7633; 31.5201; 32.7485; 37.2735; 43.0689; 44.5846; 46.6145; 51.4520; 53.8589; 57.6660; 68.8614; 76.5148; 78.1630; 81.3180; 87.6808; 102.9403; 109.9948; 122.7098; 128.4941; 136.2450; 143.9222; 147.3591; 149.9856; 157.4944; 163.7750; 176.5585; 186.3017; 189.4805; 197.1341; 210.3257; 212.5707; 213.4537; 215.8357; 216.6399; 217.6954; 220.2174; 233.5329; 258.4803; 274.2266; 276.1169; 301.8096; 303.3419; 305.7391; 314.8513; 322.1681; 322.9612; 332.8939; 338.1160; 346.6469; 347.6011; 352.9664; 357.6352; 392.4693; 402.8583; 452.1481; 454.4021; 477.0936; 489.1572; 489.5219; 490.8020; 495.2241; 507.0789; 524.6088; 529.0791; 555.4104; 556.8009; 558.2065; 566.2759; 571.3001; 576.5837; 604.0010; 606.3022; 620.5234; 621.9332; 631.7846; 634.8060; 642.9372; 647.8659; 721.2384; 736.5170; 747.9152; 759.0581; 760.0263; 762.3356; 772.1498; 788.7110; 866.7035; 873.6059; 914.1165; 936.3480; 943.4598; 970.5906; 973.0418; 973.3381; 977.2048; 984.7602; 986.7153; 988.5497; 1007.7519; 1011.8294; 1033.9586; 1050.8556; 1069.7581; 1071.3702; 1084.3230; 1085.0405; 1101.0062; 1107.8498; 1119.1582; 1152.7759; 1183.7077; 1209.6545; 1211.3809; 1215.2078; 1219.3957; 1224.0169; 1227.5817; 1229.6040; 1232.2051; 1240.5356; 1241.1983; 1253.8365; 1257.1361; 1286.3779; 1293.7308; 1304.8963; 1307.3067; 1311.1076; 1318.3995; 1369.6745; 1398.5726; 1431.9567; 1433.8882; 1438.6896; 1458.3807; 1460.1898; 1498.5199; 1504.0003; 1506.6508; 1518.4385; 1519.0969; 1521.7312; 1535.3840; 1538.4954; 1555.4161; 1613.3723; 1624.2398; 1624.9692; 1809.1193; 1873.8801; 3057.0693; 3058.3123; 3059.5744; 3066.6902; 3123.9524; 3124.8167; 3126.9208; 3130.3946; 3136.9147; 3138.6204; 3140.5779; 3144.1996; 3151.0715; 3156.5975; 3159.7109; 3160.4592; 3176.9661.

Cartesian Forces: Max 0.000000377 RMS 0.000000154

Item	Value	Threshold	Converged?
Maximum Force	0.000000	0.000450	YES
RMS Force	0.000000	0.000300	YES
Maximum Displacement	0.000010	0.001800	YES
RMS Displacement	0.000003	0.001200	YES

FILE: Yb_Sq2_PhYbOTf3_GS_Anion_B3LYP_M062x_Add56_Conf6_Y.out

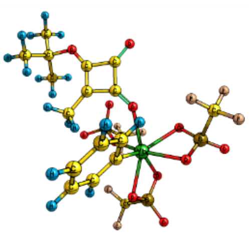

Conformer 23

E(RB3LYP/6-31G(d)/MWB59) = -3731.46041072
Convg = 0.8478D-08 -V/T = 2.0122

Temperature 195.150 Kelvin. Pressure 1.00000 Atm.
elec. and zero-point Energies= -3731.088854
elec. and thermal Energies= -3731.064929
elec. and thermal Enthalpies= -3731.064311
elec. and thermal Free Energies= -3731.137085

RB3LYP/6-31G(d)/MWB59/THF(IEFPCM) = -3731.52591590

RM06-2x/6-311+G(d,p)/MWB59/THF(IEFPCM) = -3731.36465307

Geometry at RB3LYP/6-31G(d)/MWB59
Charge = -1 Multiplicity = 1
Yb 0.5013618512 2.6267960214 0.5341877984
O 2.8836503009 2.7843246405 1.1953654831
S 3.3131257839 3.5898126195 0.0031516299
O 4.1553140515 4.757618917 0.2217083568
O 2.0729614989 3.7687372837 -0.8460736675
C 4.3543476535 2.4193306214 -1.0065809683
F 5.4343612132 2.0551491203 -0.3076467602
F 3.656747741 1.3227701561 -1.3282351419
F 4.7495518222 3.0227055249 -2.1338340224
O -1.1661722618 4.3376488717 0.2465818272
O -0.5658803701 6.7217600228 0.8765951885
O 0.5064076571 4.6811580561 1.9354825118
S -0.6824872491 5.3266031419 1.2815878372
C -2.0295142556 5.3157440228 2.5752620923
F -3.0744368845 6.0275595394 2.1313715515
F -2.4401222091 4.0726014838 2.8336576791
F -1.5760055287 5.8717388927 3.704152516
C 0.4773422139 -2.6252991703 1.3887736518
C -0.2128867069 -1.5005875323 0.9372452903
C 1.0373773959 -0.7496298594 0.7766152381
C 1.8311598386 -2.0082264215 1.1480940372
O 2.9751831917 -2.3743944862 1.208664361
O 1.3440283169 0.4098862842 0.518898925
O 0.2864715795 -3.8214549407 1.8857416072
C -0.9840825679 -4.3397141532 2.4509565863
C -1.6127194728 -1.0616063579 0.6974687598
H -2.2688904867 -1.8913541375 0.421055341
H -1.6295226731 -0.316748365 -0.1043391365
H -2.0292306136 -0.5792867375 1.5912247685
C -1.6192109076 -3.3034106506 3.3795894739
H -2.4138820302 -3.7854887691 3.9597768208
H -0.8786458264 -2.8990012974 4.0756441688
H -2.0649230794 -2.4648515765 2.8435363959
C -0.5028000252 -5.5580098757 3.2438956915
H -1.3593949363 -6.0817296415 3.6810524604
H 0.037044974 -6.252634158 2.5925691483
H 0.167319604 -5.2490215103 4.0517431307
C -1.8931264585 -4.7675981938 1.2971740254
H -2.190444037 -3.9186807343 0.6769138128
H -1.3865766707 -5.5006433588 0.6607727728
H -2.8044880837 -5.2273352766 1.6954777999
C -0.6441400972 1.7706330227 -1.4161901216
C 0.0312373294 1.0065033178 -2.3976336997
C -2.0189307548 1.9849717743 -1.6714075959
H 1.0981470321 0.8077590595 -2.2834428297
H -2.603024371 2.5862697907 -0.974784333
C -0.6021464913 0.4913604926 -3.5344747806
C -2.675413327 1.467227154 -2.794989655
H -0.0329550189 -0.0833404558 -4.2648633092
H -3.7382860169 1.6574966636 -2.9423145354
C -1.9664273386 0.7154110411 -3.734377002
H -2.4684700832 0.3147988811 -4.6136326087
O 0.3722974748 -0.1490357727 3.6910476965
S -0.6077973711 0.9477911207 3.6291548432
C -0.0352087112 2.1705262214 4.9096826023
O -2.0007689043 0.6260643145 3.9621722814
O -0.4997196449 1.7646659558 2.3521185073
F -0.8577375546 3.2223062236 4.9688372164
F 1.1981641274 2.5962027194 4.6249304375
F -0.0191334016 1.5729839848 6.1118341344

Frequency: 10.9644; 13.6838; 15.6100; 21.8905; 24.6913; 25.0098; 29.2401; 30.6012; 32.0131; 36.7134; 38.1594; 38.8979; 46.4911; 49.5312; 53.5805; 58.9299; 65.4119; 67.5477; 72.5746; 75.5267; 84.0774; 92.9942; 102.5308; 111.6340; 126.9358; 134.7945; 140.4321; 145.4657; 148.4687; 156.6875; 164.3582; 177.1461; 180.2956; 183.0771; 189.9076; 194.3197; 196.5514; 204.4618; 210.9269; 213.6759; 215.7628; 220.8314; 222.7327; 230.0034; 249.6203; 263.2192; 275.4152; 295.7720; 303.1690; 304.4692; 306.7709; 321.9118; 322.8689; 331.1715; 338.5799; 347.0651; 353.0759; 354.2340; 359.5209; 392.4600; 398.6398; 450.0741; 451.5466; 481.0030; 488.3007; 489.8362; 491.7003; 498.8850; 507.0479; 524.5299; 529.1348; 556.2827; 557.0717; 558.6325; 567.7851; 571.6037; 577.1569; 595.3935; 610.9315; 617.9464; 621.8986; 631.1337; 634.7982; 647.2809; 649.6473; 719.2215; 736.2176; 760.1445; 761.0013; 762.6264; 768.8559; 780.6036; 788.1637; 845.4589; 871.2555; 912.2930; 930.7814; 937.9927; 971.1489; 972.1905; 974.5925; 975.0050; 980.5532; 985.5521; 989.2148; 1006.8739; 1011.8270; 1033.2700; 1058.3369; 1062.5984; 1065.2470; 1084.3791; 1085.4231; 1099.0268; 1107.4958; 1118.2493; 1152.0699; 1183.8238; 1204.3687; 1209.0254; 1211.1099; 1215.3229; 1222.8163; 1224.8559; 1226.2408; 1232.6955; 1237.7076; 1242.0948; 1244.6032; 1249.2992; 1282.0533; 1292.9788; 1293.6682; 1296.0361; 1308.6252; 1318.9621; 1369.2909; 1393.9400; 1431.0657; 1434.8439; 1450.8785; 1458.6640; 1460.0621; 1500.6668; 1505.9912; 1509.7480; 1514.6568; 1519.4791; 1521.6081; 1528.9904; 1532.6620; 1546.0174; 1613.6148; 1621.7838; 1625.7185; 1812.0222; 1897.8393; 3051.5199; 3060.8646; 3065.3264; 3075.4014; 3115.4879; 3127.6675; 3130.0861; 3135.6295; 3140.1186; 3140.7686; 3141.9801; 3144.3657; 3151.6092; 3157.3471; 3160.1631; 3176.5336; 3183.6496.

Cartesian Forces: Max 0.000001331 RMS 0.000000443

Item	Value	Threshold	Converged?
Maximum Force	0.000001	0.000450	YES
RMS Force	0.000000	0.000300	YES
Maximum Displacement	0.000036	0.001800	YES
RMS Displacement	0.000007	0.001200	YES

FILE: Yb_Sq2_PhYbOTf3_GS_Anion_B3LYP_M062x_Add56_Conf6-_Y.out

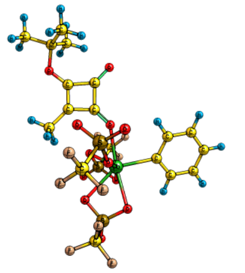

Conformer 24

E(RB3LYP/6-31G(d)/MWB59) = -3731.46831860
Convg = 0.7255D-08 -V/T = 2.0122

Temperature 195.150 Kelvin. Pressure 1.00000 Atm.
elec. and zero-point Energies= -3731.096551
elec. and thermal Energies= -3731.072587
elec. and thermal Enthalpies= -3731.071969
elec. and thermal Free Energies= -3731.145808

RB3LYP/6-31G(d)/MWB59/THF(IEFPCM) = -3731.53643170

RM06-2x/6-311+G(d,p)/MWB59/THF(IEFPCM) = -3731.37097160

Geometry at RB3LYP/6-31G(d)/MWB59
Charge = -1 Multiplicity = 1
Yb 0.7216269723 2.6483414334 0.6281355617
O -1.0664059317 1.8003905109 2.0863723338
S -1.3772259319 3.0891661517 2.8148739223
O -2.7815869874 3.4256245727 3.0071672667
O -0.4577031838 4.1245687331 2.2425750226
C -0.7060347052 2.7734510848 4.5231109699
F -1.3965480267 1.7802446986 5.1034344894
F 0.5819787584 2.4181744547 4.4606336191
F -0.8192038662 3.8733056596 5.2732459562
O 0.6687141627 4.6996476631 -0.6269320349
O -1.5883521691 5.8275337226 -0.8970444114
O -1.3004675272 3.3133578693 -0.7060419579
S -0.7563370754 4.6520381691 -1.1240752468
C -0.5921720266 4.5547422368 -2.9783613424
F -0.1327852064 5.7226488044 -3.4460661671
F 0.2466571223 3.5854748603 -3.3454098416
F -1.7947800699 4.3167914835 -3.5194934644
C 0.0232596218 -2.5116909569 1.8794108279
C -0.4135697562 -1.3387606452 1.2762607029
C 0.8208581602 -0.6355458501 1.6166226296
C 1.3415341376 -1.9044830262 2.3022936307
O 2.3310532941 -2.2551497503 2.8992906806
O 1.2906144191 0.4856493367 1.4563160625
O -0.6069255973 -3.6601429591 1.9649512709
C -0.0035275318 -4.8532086151 2.6128989411
C -1.6371707115 -0.9666780149 0.534053918
H -2.386691228 -1.761886916 0.5823762758
H -1.3653992056 -0.7887519557 -0.5137268835
H -2.0534402653 -0.0357113552 0.933314805
C 1.2734551371 -5.2426618111 1.8653595922
H 1.642960958 -6.1991435915 2.2520641791
H 1.0726800716 -5.3603473381 0.7954485904
H 2.0598351221 -4.4969339386 2.0050872595
C -1.0990461059 -5.9052417494 2.4360890575
H -0.7791437221 -6.8538354509 2.8793425627
H -2.0243857248 -5.5864222104 2.9265390615
H -1.3083597937 -6.0700720073 1.3744261978
C 0.2502532162 -4.5510606275 4.0913437573
H 1.0400035696 -3.8057089489 4.2138738802
H -0.6657367631 -4.1944438715 4.5744396669
H 0.5696341216 -5.4677069558 4.599904154
C 2.7622185658 3.4113325773 1.5765266936
C 2.9900580162 4.790928004 1.7894988967
C 3.8194871529 2.55592752 1.9632912163
H 2.2186598778 5.5092579715 1.5116834914
H 3.7149145408 1.4791120913 1.8316072447
C 4.1753831022 5.2848256271 2.3441502933
C 5.0136197433 3.0321508125 2.5168020714
H 4.3055904696 6.3567930592 2.4899895205
H 5.8020356337 2.334062413 2.7966868266
C 5.1951173728 4.4029025466 2.7104067285
H 6.1213462743 4.7803979358 3.1413032416
O 3.2097550271 0.0715792741 -1.9183938439
S 1.7583992109 0.2405215165 -1.8602192523
C 1.2139203729 0.5015896069 -3.617873919
O 1.3182440311 1.557831563 -1.2376770656
O 0.947308416 -0.9002112935 -1.3936089892
F -0.1019956645 0.7462007013 -3.6760828172
F 1.469537415 -0.607834051 -4.3313755403
F 1.8699821379 1.5239051566 -4.1756840701

Frequency: 6.9924; 11.0389; 12.6357; 15.7569; 17.5277; 21.2916; 25.0642; 30.5070; 32.4784; 35.5022; 36.9359; 44.6788; 51.7971; 53.5994; 57.9467; 61.1878; 66.3454; 71.1078; 73.0767; 80.7258; 83.7373; 88.1695; 96.1649; 111.3569; 115.1667; 118.2654; 126.0632; 141.4685; 145.3546; 151.1318; 155.0581; 165.6440; 172.8063; 177.4025; 194.2865; 197.7568; 207.7006; 211.9049; 215.3098; 216.4618; 217.1049; 221.5296; 237.5558; 242.7834; 258.8520; 274.5896; 276.8098; 303.7664; 307.4247; 310.1556; 318.5243; 323.3735; 324.0513; 332.5043; 345.6224; 347.6869; 351.2028; 355.3166; 364.1481; 393.2202; 403.1096; 445.0162; 454.2717; 479.3992; 485.9507; 489.4386; 490.0118; 494.8119; 503.8299; 521.9638; 527.3423; 556.7115; 557.7485; 558.0932; 569.0885; 569.6649; 574.9028; 602.9925; 606.3848; 614.2708; 620.0923; 631.4787; 633.8375; 641.8935; 652.1912; 715.3756; 736.6489; 747.6470; 759.8268; 760.0743; 762.3600; 773.0087; 789.2261; 865.8817; 867.8393; 913.4190; 936.5836; 943.3326; 967.8868; 971.9173; 972.8835; 975.9679; 984.8384; 987.4119; 988.7340; 1009.5247; 1011.8095; 1036.5083; 1057.4691; 1069.6524; 1071.7873; 1086.3274; 1087.1072; 1100.3360; 1109.7189; 1121.8113; 1149.8323; 1184.2685; 1209.3476; 1213.7287; 1215.0361; 1221.2104; 1225.2938; 1228.3595; 1230.3162; 1232.8084; 1239.6668; 1244.2337; 1254.5459; 1260.3007; 1286.6496; 1296.2546; 1304.9484; 1305.7542; 1307.3178; 1317.8185; 1369.9761; 1396.7523; 1432.1835; 1434.2740; 1439.4848; 1458.9419; 1460.4418; 1501.8462; 1504.1470; 1515.6747; 1518.3993; 1519.0177; 1522.7377; 1535.5742; 1538.2225; 1555.2850; 1615.0630; 1626.9234; 1628.1817; 1808.7102; 1874.9731; 3055.8302; 3058.5890; 3059.7868; 3066.9158; 3124.2914; 3124.3482; 3127.1045; 3137.0311; 3137.4795; 3138.6857; 3144.1863; 3145.0851; 3155.6796; 3156.8144; 3160.7939; 3162.5861; 3176.2867.

Cartesian Forces: Max 0.000008750 RMS 0.000001288

Item	Value	Threshold	Converged?
Maximum Force	0.000005	0.000450	YES
RMS Force	0.000001	0.000300	YES
Maximum Displacement	0.001774	0.001800	YES
RMS Displacement	0.000460	0.001200	YES

FILE: Yb_Sq2_PhYbOTf3_GS_Anion_B3LYP_M062x_Add56_Conf6-1_a.out

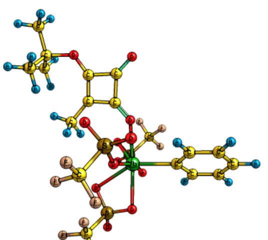

Conformer 25

E(RB3LYP/6-31G(d)/MWB59) = -3731.45908812
Convg = 0.3241D-08 -V/T = 2.0122

Temperature 195.150 Kelvin. Pressure 1.00000 Atm.
elec. and zero-point Energies= -3731.087573
elec. and thermal Energies= -3731.063445
elec. and thermal Enthalpies= -3731.062827
elec. and thermal Free Energies= -3731.137415

RB3LYP/6-31G(d)/MWB59/THF(IEFPCM) = -3731.52829283

RM06-2x/6-311+G(d,p)/MWB59/THF(IEFPCM) = -3731.36674982

Geometry at RB3LYP/6-31G(d)/MWB59
Charge = -1 Multiplicity = 1
Yb 0.6630669387 2.4961145158 0.3630146408
O -0.9199408113 2.0247264139 2.2127744103
S -1.0732846166 3.4437504236 2.7055013688
O -2.4193627398 3.9080451819 3.0134663159
O -0.209160569 4.2892736228 1.81505682
C -0.1704986419 3.4256285523 4.3343894261
F -0.8167855837 2.6312969619 5.1995788629
F 1.0733480812 2.9620785271 4.173213588
F -0.1133918873 4.6611112928 4.841335551
O 0.3321804254 4.2170045535 -1.2811207914
O -1.9630773551 5.2929126514 -1.3855174582
O -1.5896365633 2.8709589882 -0.7282218193
S -1.1548345554 4.0851412086 -1.5031513541
C -1.3149773875 3.5999369961 -3.2955769556
F -0.9202926962 4.6170287138 -4.0713825518
F -0.575063836 2.5276703884 -3.5813988488
F -2.598648233 3.3234220272 -3.5670234831
C -0.1693065198 -2.4366779554 2.1724947886
C -0.572673282 -1.2898196048 1.4901190231
C 0.7057328258 -0.6240222647 1.7664535882
C 1.1638593127 -1.8510604802 2.5653235027
O 2.1105960751 -2.2133334669 3.2118343151
O 1.2108528865 0.4525643318 1.4664673807
O -0.560897862 -3.6478962533 2.488940584
C -1.802289173 -4.303798275 2.0236622266
C -1.7115953076 -0.7620343384 0.696351795
H -2.6141651284 -1.3718537006 0.7569460776
H -1.3994315777 -0.7004583015 -0.3537045803
H -1.9516832829 0.2528435622 1.0322479446
C -3.0068549797 -3.6052087172 2.6593540132
H -3.9277453432 -4.126916557 2.3765838687
H -2.9231659776 -3.6233058981 3.7507858453
H -3.0996737634 -2.5647793557 2.3413641479
C -1.6477174667 -5.7215637261 2.5802612721
H -2.5223138194 -6.3249709055 2.3154717143
H -0.7545778397 -6.200627875 2.1678564243
H -1.5556800326 -5.6990115779 3.6705976561
C -1.8326718756 -4.3258229581 0.4937016685
H -1.8859988956 -3.3271719956 0.0568928885
H -0.9361289318 -4.8176657459 0.1038110159
H -2.7092377146 -4.8893786114 0.1555166446
C 2.8369522847 3.3269552258 0.8074669915
C 3.0834338619 4.6086024563 1.3509823096
C 3.9877528735 2.5593793971 0.5108060332
H 2.2417647053 5.2568154664 1.5950868304
H 3.8804789717 1.5650396919 0.0761542201
C 4.3743124872 5.0920155651 1.5895479318
C 5.2861269864 3.0278589124 0.7426358138
H 4.5165313155 6.0872551987 2.0100098906
H 6.1431444338 2.401550067 0.4976641958
C 5.483468918 4.2988293411 1.2862830003
H 6.491260109 4.6687552304 1.4695400225
O 0.8282570927 -1.3956753349 -1.228148353
S 1.5838877353 -0.262880417 -1.7955633756
C 1.103992528 -0.2399848379 -3.5909263578
O 3.0452946853 -0.3343711595 -1.7808726896
O 1.0296494346 1.0816225705 -1.3461717191
F 1.6187236342 0.8252308135 -4.2115654945
F -0.2298295932 -0.2172428737 -3.7233693981
F 1.5675995421 -1.3495174518 -4.1891905439

Frequency: 5.9907; 7.9632; 11.3424; 16.7942; 18.2948; 21.6247; 25.8864; 27.8629; 31.7353; 35.4578; 38.0549; 43.4833; 49.0791; 52.5591; 54.5974; 63.9162; 64.9519; 68.0655; 72.1368; 77.0077; 80.7691; 85.6127; 98.4293; 104.8059; 115.5494; 117.2065; 120.0919; 135.2423; 139.1014; 143.1733; 162.3282; 165.1728; 172.7245; 174.7640; 193.2607; 196.9157; 199.2004; 208.3028; 210.0029; 213.9544; 215.5379; 220.5691; 230.0611; 240.1827; 248.3907; 258.5184; 274.4063; 297.2067; 303.6499; 307.7628; 310.8451; 323.1925; 324.1429; 332.0856; 349.4795; 352.4806; 355.0830; 357.5982; 369.4411; 393.4108; 399.1921; 445.0468; 450.9520; 483.1070; 485.5658; 489.0169; 489.9426; 494.0940; 503.4015; 521.6868; 527.2084; 556.2537; 557.5387; 557.9032; 568.6622; 569.2882; 574.7772; 599.4242; 605.5863; 613.5959; 619.8782; 631.9944; 633.1726; 652.1586; 653.2181; 717.6467; 737.2007; 759.9251; 760.4404; 762.1558; 767.7732; 778.6467; 786.5450; 849.1975; 870.7752; 917.1993; 932.4302; 935.0849; 967.8619; 976.5209; 977.5092; 977.7336; 979.9286; 987.5812; 988.4722; 1011.0209; 1011.1700; 1037.1293; 1051.1285; 1062.2670; 1065.0705; 1085.5953; 1088.5742; 1103.2035; 1108.7146; 1121.9006; 1149.1443; 1184.6481; 1208.8194; 1213.6161; 1213.8445; 1215.5412; 1220.8929; 1226.5079; 1228.4919; 1231.9167; 1233.1775; 1241.4175; 1243.0946; 1253.4191; 1285.3678; 1292.9919; 1295.7178; 1303.1199; 1305.7721; 1317.3650; 1373.2041; 1387.0788; 1432.0654; 1432.8956; 1436.1431; 1458.7561; 1460.3479; 1501.0122; 1508.0026; 1512.9172; 1519.5786; 1523.0635; 1525.0950; 1529.1055; 1530.9406; 1550.5780; 1615.8691; 1623.6661; 1628.3230; 1814.4562; 1898.8321; 3059.9149; 3062.0612; 3066.8237; 3073.5818; 3122.1859; 3129.7037; 3132.6556; 3137.4904; 3139.6372; 3142.5378; 3143.7334; 3154.4823; 3156.8712; 3162.3955; 3167.0762; 3175.2733; 3176.5253.

Cartesian Forces: Max 0.000000763 RMS 0.000000325

Item	Value	Threshold	Converged?
Maximum Force	0.000001	0.000450	YES
RMS Force	0.000000	0.000300	YES
Maximum Displacement	0.000234	0.001800	YES
RMS Displacement	0.000036	0.001200	YES

FILE: Yb_Sq2_PhYbOTf3_GS_Anion_B3LYP_M062x_Add56_Conf6-1-.out

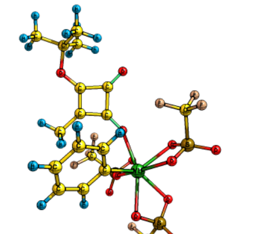

Conformer 26

E(RB3LYP/6-31G(d)/MWB59) = -3731.46303187
Convg = 0.5666D-08 -V/T = 2.0122

Temperature 195.150 Kelvin. Pressure 1.00000 Atm.
elec. and zero-point Energies= -3731.091532
elec. and thermal Energies= -3731.067566
elec. and thermal Enthalpies= -3731.066948
elec. and thermal Free Energies= -3731.140311

RB3LYP/6-31G(d)/MWB59/THF(IEFPCM) = -3731.53284997

RM06-2x/6-311+G(d,p)/MWB59/THF(IEFPCM) = -3731.36507088

Geometry at RB3LYP/6-31G(d)/MWB59
Charge = -1 Multiplicity = 1
Yb 0.4456596148 3.0359568552 0.8941008472
O 2.8332195546 3.168586902 1.5276546265
S 3.3531158561 3.4259118787 0.1431447406
O 4.3185360993 4.5001935538 -0.0451970978
O 2.1496758596 3.3920446191 -0.770407388
C 4.2811293247 1.8728641286 -0.3132862334
F 5.2642775951 1.6539699924 0.5646395357
F 3.4650334234 0.8070115728 -0.3211940883
F 4.8115459369 2.0057838153 -1.5353886126
O -1.0433806043 4.69812656 -0.0071542899
O -0.079179402 7.0086536039 -0.4133884592
O 0.7724605156 5.4282146286 1.3827419199
S -0.3604480421 5.9343377715 0.5307030315
C -1.5846397502 6.6603665381 1.7333632938
F -2.6666152751 7.0806410234 1.0628764476
F -1.9578908599 5.7596194728 2.639009952
F -1.0289906382 7.7071951589 2.3555047966
C 0.7496672062 -2.4187435251 1.521847893
C -0.1084431086 -1.3304385795 1.4173317223
C 0.9950339269 -0.3733824084 1.538119099
C 1.9792352262 -1.5488679389 1.6625483869
O 3.16698646 -1.709499127 1.8054621178
O 1.1401793631 0.8391428394 1.5419132422
O 0.4415462127 -3.6945054424 1.4879951041
C 1.4653804375 -4.7658051785 1.6048248439
C -1.5746130683 -1.2181051929 1.2549914958
H -2.0380404529 -2.208427184 1.2148675757
H -1.8148321953 -0.6615232057 0.342075691
H -2.0019547781 -0.653966967 2.090688609
C 2.4468341213 -4.6558751693 0.436232761
H 3.1189435375 -5.5212826233 0.4439409115
H 1.9100374359 -4.6490963436 -0.518279052
H 3.0591494088 -3.7542017782 0.5150664208
C 0.6174795077 -6.0344559445 1.5072154302
H 1.2599394857 -6.9179034736 1.5800419233
H -0.1179416779 -6.0714327389 2.3172095153
H 0.0835209374 -6.0701315436 0.5523189993
C 2.1578292415 -4.6570117477 2.9648737492
H 2.7720123522 -3.7552965199 3.027205946
H 1.4195504717 -4.6513699675 3.7737744265
H 2.8143871338 -5.5225149655 3.1082893856
C -0.7187729354 1.6955970421 -0.7446390432
C -0.0674769122 0.7630928544 -1.5850605185
C -2.1223047056 1.7624527942 -0.9024489458
H 1.0190273371 0.6686800873 -1.5406658747
H -2.6935010743 2.4767971722 -0.3084315308
C -0.7511210167 -0.0519595706 -2.4957886188
C -2.827926166 0.9519608781 -1.8011559205
H -0.2011691115 -0.7506330006 -3.1263199691
H -3.91109733 1.0390576343 -1.8819188416
C -2.1417053853 0.0346553851 -2.6019336441
H -2.6821415347 -0.5961423483 -3.3058419686
O -2.0249444728 3.662859026 4.716533704
S -0.9026195151 2.8474802353 4.2541107688
C -1.4448738102 1.0979578018 4.5850936274
O 0.3971384885 2.9696601283 4.916296103
O -0.7905427209 2.7994142179 2.7316760081
F -0.5086549377 0.2097599804 4.1955657236
F -2.5827378176 0.8096017564 3.925619182
F -1.663650526 0.9203561293 5.894450272

Frequency: 6.1139; 10.3777; 14.6685; 15.3233; 20.5919; 28.4748; 31.8257; 35.5127; 37.2586; 41.8825; 43.1260; 44.5362; 48.9706; 50.7913; 56.7047; 57.9459; 62.8033; 68.0324; 68.6649; 74.9182; 83.4098; 83.9051; 90.9995; 110.4835; 123.9826; 128.9643; 138.1586; 141.9074; 146.9553; 149.5281; 157.2183; 161.6866; 174.7077; 178.0140; 186.3163; 192.2960; 212.2378; 212.7540; 214.6900; 216.3173; 217.6798; 221.0029; 225.3003; 234.1580; 257.7537; 273.8240; 275.5353; 300.3671; 302.4333; 305.5224; 308.2900; 321.7038; 323.4203; 329.8551; 343.7518; 346.6049; 347.2749; 353.5086; 357.9481; 391.5986; 400.6298; 453.4220; 454.3744; 479.8303; 488.0781; 489.8525; 490.4352; 494.7608; 506.7649; 524.8817; 529.4907; 555.0085; 556.7877; 558.3842; 568.5446; 571.3870; 577.0026; 603.3730; 605.1632; 611.8846; 621.6296; 632.1458; 634.6728; 640.2747; 647.1762; 721.5693; 736.7169; 746.2743; 752.7683; 758.4281; 763.2912; 770.4151; 788.0644; 863.5387; 874.1218; 912.8824; 936.4454; 943.1079; 964.6377; 969.6823; 971.5456; 976.5506; 982.8894; 984.9883; 988.5002; 1000.9396; 1011.5458; 1033.6885; 1056.6949; 1068.3524; 1070.9585; 1083.4441; 1084.6468; 1095.6015; 1105.6531; 1118.8803; 1162.2392; 1183.2852; 1193.4030; 1207.8714; 1211.2775; 1212.5363; 1216.9718; 1222.6471; 1224.7237; 1234.5410; 1238.5414; 1242.5590; 1253.1684; 1256.4327; 1286.6596; 1294.5553; 1304.8764; 1307.5642; 1318.0419; 1330.9241; 1369.0159; 1401.1641; 1432.4202; 1433.9837; 1443.7818; 1458.3581; 1460.2933; 1496.4988; 1503.8966; 1507.9263; 1518.5419; 1518.6621; 1520.9452; 1535.3370; 1538.0462; 1555.2054; 1612.2257; 1623.0943; 1626.8762; 1820.9637; 1876.3851; 3059.4030; 3059.6441; 3062.5100; 3067.0912; 3125.0321; 3126.6537; 3128.2145; 3132.6157; 3137.0977; 3137.4851; 3138.8942; 3144.3919; 3148.8177; 3156.9639; 3157.6047; 3160.4139; 3176.7774.

Cartesian Forces: Max 0.000004399 RMS 0.000001041

Item	Value	Threshold	Converged?
Maximum Force	0.000004	0.000450	YES
RMS Force	0.000001	0.000300	YES
Maximum Displacement	0.000910	0.001800	YES
RMS Displacement	0.000181	0.001200	YES

FILE: Yb_Sq2_PhYbOTf3_GS_Anion_B3LYP_M062x_Add56_Conf7.out

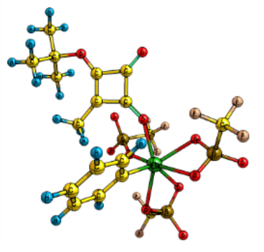

Conformer 27

E(RB3LYP/6-31G(d)/MWB59) = -3731.46040499
Convg = 0.5404D-08 -V/T = 2.0122

Temperature 195.150 Kelvin. Pressure 1.00000 Atm.
elec. and zero-point Energies= -3731.089035
elec. and thermal Energies= -3731.064942
elec. and thermal Enthalpies= -3731.064324
elec. and thermal Free Energies= -3731.138631

RB3LYP/6-31G(d)/MWB59/THF(IEFPCM) = -3731.52780105

RM06-2x/6-311+G(d,p)/MWB59/THF(IEFPCM) = -3731.36607414

Geometry at RB3LYP/6-31G(d)/MWB59
Charge = -1 Multiplicity = 1
Yb 0.5529506505 2.4563613725 0.7433236964
O 2.9075012809 2.4090605156 1.4865635582
S 3.4758111552 3.0742982439 0.2651793294
O 4.4367402319 4.15332148 0.4487679541
O 2.3058082105 3.3205706574 -0.6585013647
C 4.4255921874 1.723482284 -0.5989360358
F 5.4329811687 1.3072495972 0.1755297509
F 3.6281410068 0.6793947851 -0.8661140513
F 4.9259737967 2.1846409264 -1.7512447776
O -0.9166650775 4.3157701348 0.3136273615
O 0.0115379174 6.6465175141 0.6795419023
O 0.816595451 4.6030966854 1.9519255434
S -0.2868772003 5.3328163018 1.236167807
C -1.5841736825 5.6556012119 2.5363274398
F -2.6346485462 6.2647967294 1.9692948103
F -1.9969259799 4.5244916602 3.1054415314
F -1.0760225807 6.4598090257 3.4787524845
C 0.1245084148 -2.905952097 1.4204890336
C -0.4784727373 -1.718056031 1.0060997516
C 0.7919118985 -0.9901955507 1.1048724835
C 1.5001053458 -2.2956859875 1.4911140017
O 2.6108785963 -2.6887641754 1.7335899328
O 1.1562714738 0.171840097 0.9679328574
O -0.1418537965 -4.155026449 1.7172420858
C -1.491317116 -4.7494200918 1.848347395
C -1.8184833592 -1.1940335589 0.6412906355
H -2.48967726 -1.9597514538 0.2459786985
H -1.7130183025 -0.3974680457 -0.1018155443
H -2.278265503 -0.7482045415 1.5313507609
C -2.3470375649 -3.9094682388 2.798431174
H -3.2866992915 -4.4366357959 2.9973981898
H -1.8280905977 -3.7596415691 3.7500434496
H -2.5936211075 -2.928048521 2.3914934631
C -1.1813488266 -6.1163545586 2.4645390697
H -2.1063329062 -6.6889477311 2.5896494383
H -0.5030030741 -6.6840859575 1.8199764757
H -0.7087652796 -5.9974936467 3.444229094
C -2.0979243597 -4.9115869798 0.4526569433
H -2.2596219744 -3.950172212 -0.039680795
H -1.4403734464 -5.5159467911 -0.1806455365
H -3.0662858785 -5.4186731939 0.5267602695
C -0.5063469261 1.6284694147 -1.2600455716
C 0.167826526 0.7901465983 -2.1789851167
C -1.849637287 1.9206643971 -1.5929650564
H 1.2142044007 0.5344207395 -2.0047678205
H -2.4276762167 2.5809271507 -0.9461622944
C -0.4410179387 0.2723637583 -3.3281011748
C -2.4807386768 1.406059388 -2.7325825718
H 0.1252411718 -0.3635505675 -4.0083986451
H -3.519623116 1.6590635428 -2.9429637878
C -1.7761414978 0.5751753803 -3.6066559959
H -2.2584395545 0.1750710033 -4.4971441055
O -0.8484232373 -0.3020176984 3.6697195744
S -1.1218686312 1.1455416267 3.7269786492
C 0.2265232665 1.8428588034 4.8035924547
O -2.3841509088 1.5795381389 4.3247407067
O -0.8214092415 1.8358172504 2.4043764392
F 0.0290508878 3.1451446866 5.0255627396
F 1.4179007581 1.681857418 4.209696697
F 0.244437168 1.2033626367 5.9820428328

Frequency: 4.0429; 12.4411; 14.4203; 16.5317; 21.1840; 23.3847; 27.8990; 30.0640; 31.3540; 33.8962; 35.0270; 41.4021; 42.8068; 47.6999; 51.0174; 56.5403; 62.2203; 65.0615; 72.5266; 73.8568; 78.2272; 98.6326; 100.7270; 107.1434; 126.8523; 135.9307; 139.2742; 144.9831; 147.1970; 153.8543; 162.6409; 169.6182; 176.5519; 179.0463; 186.2789; 187.2589; 198.0040; 208.7018; 211.2122; 213.2420; 214.4921; 216.9625; 220.8742; 228.7938; 238.7357; 259.2566; 276.1885; 293.4944; 302.7515; 303.6211; 306.3261; 322.0741; 322.6700; 332.8330; 336.7207; 349.0108; 352.8931; 353.7500; 358.4408; 392.1900; 398.7983; 449.2260; 452.1530; 481.3348; 486.3078; 489.6833; 490.7673; 493.2848; 506.7887; 524.6070; 529.1172; 555.1893; 556.9074; 558.2616; 566.0484; 571.2952; 576.5784; 599.5995; 607.8897; 620.5076; 622.4576; 631.8547; 634.7102; 647.8369; 651.4063; 720.2246; 736.6207; 759.3676; 760.6024; 762.3172; 769.3435; 779.0663; 786.1464; 849.4818; 872.7142; 913.5544; 932.5369; 935.8358; 971.0078; 972.6248; 977.0817; 977.5658; 981.2850; 985.1984; 987.0023; 1008.3337; 1011.8255; 1033.9244; 1047.8570; 1062.0541; 1064.7584; 1084.3580; 1084.9852; 1103.2752; 1107.5562; 1118.3606; 1152.2290; 1183.6963; 1206.9786; 1210.7621; 1211.3383; 1215.2783; 1220.3652; 1223.9246; 1228.5136; 1229.4881; 1232.0979; 1241.4261; 1242.2935; 1253.3977; 1284.4611; 1293.3056; 1293.9911; 1307.0566; 1309.3247; 1318.4709; 1369.3295; 1390.0859; 1432.1546; 1433.5687; 1437.8430; 1458.5709; 1459.8979; 1499.7886; 1504.6743; 1511.0652; 1514.2723; 1520.1985; 1522.0231; 1529.1776; 1531.1416; 1547.8479; 1613.5330; 1620.7702; 1625.3197; 1817.0998; 1897.0680; 3061.0892; 3061.9498; 3066.4555; 3074.2190; 3125.9677; 3128.9030; 3131.6450; 3133.8948; 3139.5720; 3140.0199; 3142.6256; 3149.5333; 3151.4500; 3159.5016; 3163.1225; 3176.3351; 3181.9865.

Cartesian Forces: Max 0.000001666 RMS 0.000000346

Item	Value	Threshold	Converged?
Maximum Force	0.000001	0.000450	YES
RMS Force	0.000000	0.000300	YES
Maximum Displacement	0.000221	0.001800	YES
RMS Displacement	0.000047	0.001200	YES

FILE: Yb_Sq2_PhYbOTf3_GS_Anion_B3LYP_M062x_Add56_Conf7-_Y.out

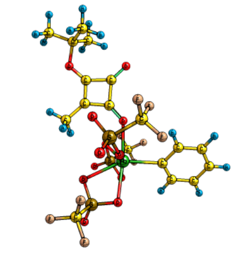

Conformer 28

E(RB3LYP/6-31G(d)/MWB59) = -3731.46906970
Convg = 0.5417D-08 -V/T = 2.0122

Temperature 195.150 Kelvin. Pressure 1.00000 Atm.
elec. and zero-point Energies= -3731.097427
elec. and thermal Energies= -3731.073403
elec. and thermal Enthalpies= -3731.072785
elec. and thermal Free Energies= -3731.146849

RB3LYP/6-31G(d)/MWB59/THF(IEFPCM) = -3731.53622730

RM06-2x/6-311+G(d,p)/MWB59/THF(IEFPCM) = -3731.37150091

Geometry at RB3LYP/6-31G(d)/MWB59
Charge = -1 Multiplicity = 1
Yb 0.8413481338 2.4038983995 0.9948043008
O -1.0199195351 1.711704701 2.438581523
S -1.4528798255 3.0817036125 2.9101413652
O -2.8821228417 3.3655015428 2.915639411
O -0.5286367509 4.0598759022 2.2491749119
C -0.9437164293 3.0905394007 4.7021647154
F -1.6869212407 2.2076539316 5.3851444615
F 0.3434356586 2.7545702427 4.8275990962
F -1.1306494244 4.3071257493 5.2241822717
O 0.7135382184 4.228241278 -0.5550786439
O -1.5952714606 5.0685151509 -1.1809591887
O -1.1125570626 2.6511088874 -0.5772108685
S -0.6461649459 3.962063485 -1.155494746
C -0.3064453102 3.5976984706 -2.9520364697
F 0.2281274043 4.6801023057 -3.531478118
F 0.5256575905 2.5701162414 -3.0944760037
F -1.4644535496 3.3085785458 -3.564775642
C -0.4299481734 -2.6488908525 1.7797877809
C -0.6437618416 -1.3895467939 1.2342603229
C 0.5825436867 -0.8613538588 1.8241736881
C 0.8599412249 -2.23272379 2.4498892011
O 1.7048205917 -2.7426739907 3.14654284
O 1.1722939388 0.2139056968 1.8454815284
O -1.1778652197 -3.7212643022 1.6585363264
C -0.8086610506 -5.0233151406 2.270110557
C -1.6825385495 -0.8111529434 0.3553739312
H -2.5123664049 -1.508461671 0.2094525458
H -1.2314558451 -0.5812052912 -0.6179214542
H -2.0567199891 0.1298479458 0.7728056491
C 0.523465282 -5.4968917289 1.6848259693
H 0.7279560437 -6.5176672195 2.0271104845
H 0.4823839069 -5.5040874836 0.5907033897
H 1.3498106833 -4.8605633863 2.0108874299
C -1.9641821139 -5.9245307311 1.8332311323
H -1.8158689325 -6.9362264809 2.2245808039
H -2.9175969264 -5.541453594 2.2109357206
H -2.0203692843 -5.9781697337 0.74143249
C -0.7604309975 -4.8717343248 3.7920661561
H 0.072252712 -4.2348662649 4.1007939381
H -1.6995343879 -4.451132163 4.1672244222
H -0.6201245073 -5.8568521497 4.2511611481
C 2.718293768 3.2785605722 2.1625478141
C 2.7637514121 4.6148872741 2.6228010944
C 3.8762027595 2.5089016618 2.421106467
H 1.9049840925 5.2659688442 2.4586889388
H 3.9205628255 1.4711001253 2.0902641471
C 3.872591211 5.1481837026 3.288307618
C 4.9935885064 3.0242929035 3.0879831254
H 3.8628853175 6.1850435438 3.6230146224
H 5.8636488608 2.3927341464 3.2649873331
C 4.9945862935 4.350380286 3.5251600311
H 5.8609105698 4.7584327229 4.0436380486
O 2.4281040133 0.3015631588 -2.8761114418
S 2.2046487043 0.0770556076 -1.4492190749
C 3.871390051 -0.3910088986 -0.7667700651
O 1.3163414185 -1.0321858073 -1.0521032651
O 1.9211083324 1.3532339625 -0.6740184989
F 3.7934393482 -0.6604504926 0.5463068517
F 4.7546551786 0.5946390252 -0.9494648434
F 4.3197319978 -1.4907938503 -1.3935634317

Frequency: 5.1052; 10.2069; 15.8549; 20.2098; 21.3496; 22.7107; 26.7627; 27.9047; 31.6234; 32.3800; 34.9959; 39.6788; 47.2640; 47.6204; 54.6698; 57.6546; 60.5852; 69.9405; 74.7798; 76.7750; 80.0902; 90.5550; 102.4499; 110.3487; 115.6375; 120.2628; 137.8922; 140.6942; 143.0687; 146.0238; 159.1150; 167.0534; 174.5442; 184.4297; 192.2038; 196.0967; 209.0253; 211.8712; 213.4828; 215.3314; 216.7107; 218.9942; 223.3849; 243.7304; 257.9032; 272.9872; 275.6927; 301.1322; 303.8755; 308.2224; 319.6687; 323.5612; 324.0388; 332.4650; 343.2232; 346.7803; 348.7134; 351.5344; 357.9638; 392.7947; 403.5257; 443.5719; 454.1690; 478.9188; 489.3804; 490.3336; 492.6286; 494.4293; 506.6651; 521.7377; 527.0847; 556.6146; 557.9929; 558.3705; 568.2417; 569.4980; 574.7023; 602.6397; 605.7499; 613.6859; 620.0700; 631.3554; 633.9020; 642.0643; 652.3315; 717.0167; 736.1542; 747.6059; 758.5549; 760.5316; 762.3548; 770.2193; 789.1570; 866.2618; 870.0758; 915.0265; 936.3314; 943.2469; 969.7760; 972.7567; 974.8808; 976.1636; 985.0120; 987.1272; 988.2595; 1011.3343; 1012.0152; 1036.6981; 1051.1681; 1069.7402; 1070.9841; 1085.9437; 1087.2298; 1099.1870; 1103.9300; 1121.2999; 1153.0067; 1184.6397; 1209.2762; 1209.6322; 1214.6870; 1217.7429; 1224.1895; 1226.3220; 1231.6354; 1233.9161; 1240.7913; 1243.4266; 1258.2150; 1261.1869; 1286.3441; 1295.9765; 1304.7203; 1305.0162; 1309.9386; 1316.6993; 1371.3174; 1397.5971; 1432.0386; 1433.7447; 1437.6046; 1459.0414; 1460.1954; 1502.5019; 1503.8897; 1511.2673; 1518.2644; 1518.9231; 1524.0835; 1535.3789; 1537.8122; 1555.0565; 1615.6926; 1628.0725; 1628.4476; 1808.0716; 1874.2919; 3057.0022; 3058.5728; 3059.7617; 3066.9082; 3123.3260; 3124.2767; 3127.1561; 3137.1954; 3138.1426; 3138.7482; 3144.2684; 3145.9244; 3155.9291; 3156.6408; 3160.4917; 3162.8985; 3177.2044.

Cartesian Forces: Max 0.000008084 RMS 0.000001252

Item	Value	Threshold	Converged?
Maximum Force	0.000006	0.000450	YES
RMS Force	0.000001	0.000300	YES
Maximum Displacement	0.001781	0.001800	YES
RMS Displacement	0.000311	0.001200	YES

FILE: Yb_Sq2_PhYbOTf3_GS_Anion_B3LYP_M062x_Add56_Conf7-1.out

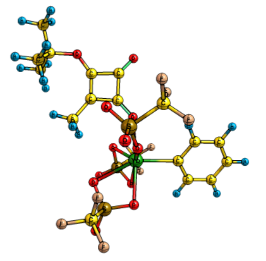

Conformer 29

E(RB3LYP/6-31G(d)/MWB59) = -3731.46029851
Convg = 0.6631D-08 -V/T = 2.0122

Temperature 195.150 Kelvin. Pressure 1.00000 Atm.
elec. and zero-point Energies= -3731.088748
elec. and thermal Energies= -3731.064681
elec. and thermal Enthalpies= -3731.064063
elec. and thermal Free Energies= -3731.137968

RB3LYP/6-31G(d)/MWB59/THF(IEFPCM) = -3731.52863189

RM06-2x/6-311+G(d,p)/MWB59/THF(IEFPCM) = -3731.36765855

Geometry at RB3LYP/6-31G(d)/MWB59
Charge = -1 Multiplicity = 1
Yb 0.7271098778 1.9813586834 1.2101241556
O -1.1199648168 1.6166796733 2.7835932302
S -1.3615867403 3.0565443386 3.1783905083
O -2.7447306064 3.5136241323 3.2210935542
O -0.3603590209 3.8693230726 2.4132303287
C -0.7704494755 3.1167834445 4.9442059858
F -1.5861507791 2.3837787792 5.7155283258
F 0.4684536292 2.6281662352 5.0440237025
F -0.7794997643 4.3794862129 5.3842015331
O 0.7304594166 3.7256092028 -0.4330740074
O -1.4907850215 4.8099100595 -0.9969607467
O -1.2700212147 2.382371388 -0.2899522844
S -0.6803108561 3.598678761 -0.955534925
C -0.4804073915 3.1117679803 -2.7439631502
F 0.1596675981 4.0859134192 -3.4022293185
F 0.2029589839 1.9772948651 -2.8696891727
F -1.6956760052 2.9492417629 -3.2896966405
C -1.0240441417 -2.9467372882 2.1146848509
C -1.1746992283 -1.6647876962 1.5874976206
C 0.111489839 -1.2369941083 2.1474422883
C 0.2919222964 -2.6238139493 2.7765091827
O 1.0802267901 -3.2118692419 3.4687764288
O 0.7760238484 -0.207007956 2.1123617696
O -1.6030456171 -4.1227658527 2.1643864371
C -2.8297468547 -4.5121078031 1.4346736715
C -2.0967460664 -0.8644891367 0.7429114567
H -3.076109347 -1.3227167692 0.5973312294
H -1.6269531464 -0.7178427991 -0.2379322705
H -2.2354184258 0.1268428161 1.1877764052
C -4.02212981 -3.7494485694 2.0173758482
H -4.9459349468 -4.0814701509 1.5310826458
H -4.1096365481 -3.9449080726 3.0910250905
H -3.9361509118 -2.6706092575 1.8723673805
C -2.937146491 -6.0051596885 1.7553739788
H -3.8292364794 -6.4266005997 1.2803487208
H -2.0579595067 -6.5415602328 1.385754981
H -3.0091818971 -6.1624924295 2.8360699272
C -2.6291150324 -4.296064999 -0.0669825859
H -2.499354953 -3.2448836186 -0.3297551669
H -1.7476058383 -4.8434747872 -0.4148428064
H -3.5038408458 -4.672932292 -0.6083690462
C 2.7473471135 2.6439341042 2.2707602621
C 2.9888221065 3.978725687 2.6704297909
C 3.8019710274 1.7356907265 2.5219156222
H 2.2181799625 4.732660355 2.5090411641
H 3.6944269449 0.689206513 2.2358649188
C 4.185060178 4.3830482456 3.2723762215
C 5.0039727266 2.1211615708 3.1263049737
H 4.3271363703 5.4238636668 3.5616696122
H 5.7883135567 1.3853272663 3.3006558613
C 5.199336274 3.4509907343 3.5044958633
H 6.1325381033 3.7582245751 3.974105407
O 1.9755468425 -0.3863813613 -2.6408098582
S 1.725064234 -0.5608514634 -1.2113283673
C 3.3144248633 -1.2391291681 -0.520573176
O 0.6969859235 -1.537825199 -0.8023865107
O 1.6119842898 0.7508660727 -0.4529493371
F 3.2016201935 -1.4775812741 0.7958500185
F 4.3211365461 -0.3827171024 -0.71543878
F 3.6106469929 -2.3968999854 -1.1324443994

Frequency: 6.8574; 9.6157; 16.6544; 20.6308; 22.2678; 23.5413; 25.4409; 28.5599; 30.6968; 32.8805; 35.7222; 39.8688; 45.4533; 49.0578; 55.7336; 59.2883; 63.1302; 67.2406; 71.2679; 75.2545; 79.6546; 97.2716; 104.9495; 109.4632; 115.6608; 120.6297; 133.2434; 139.5379; 142.9600; 154.7038; 160.5445; 168.5249; 175.3990; 183.4488; 192.5521; 194.2823; 200.9400; 209.0501; 210.8731; 212.9665; 215.2344; 218.4241; 223.7812; 230.1528; 249.0279; 258.9845; 272.6289; 296.2985; 301.7077; 304.4274; 308.4893; 323.6411; 324.1606; 332.6002; 342.3994; 350.3848; 354.0544; 355.9403; 362.6497; 393.1594; 399.5006; 443.6592; 450.7909; 485.6174; 488.6296; 489.3408; 490.7578; 495.3852; 506.4869; 521.7199; 527.0489; 556.6023; 558.0792; 558.3110; 568.1038; 569.2897; 574.6662; 599.2773; 605.1244; 613.6462; 620.3722; 631.5134; 633.8811; 652.5148; 652.6080; 717.3484; 736.2801; 758.7816; 760.8795; 762.2425; 768.1876; 777.6450; 783.3477; 849.4777; 870.4162; 915.4710; 932.7998; 935.2243; 970.0182; 975.0613; 976.0238; 978.1442; 980.1455; 985.1561; 987.5042; 1011.8369; 1012.2829; 1036.8438; 1047.7569; 1062.3660; 1065.1888; 1086.2372; 1087.5211; 1101.9735; 1105.9861; 1120.5142; 1153.0709; 1184.8306; 1208.0395; 1209.5592; 1213.7746; 1214.8146; 1218.9318; 1224.3896; 1226.5970; 1231.8740; 1235.1172; 1240.9663; 1244.1748; 1256.9989; 1285.4521; 1293.4710; 1295.9774; 1304.5516; 1308.8904; 1316.0913; 1371.6901; 1388.4240; 1431.0385; 1432.9137; 1435.9745; 1459.0980; 1460.2364; 1500.7648; 1506.8672; 1512.4001; 1518.7671; 1522.5521; 1524.3264; 1529.0833; 1530.6825; 1550.4855; 1615.7178; 1623.9674; 1628.4591; 1816.6824; 1897.8926; 3060.6864; 3062.0861; 3066.9612; 3073.7723; 3122.8878; 3129.7203; 3132.7684; 3138.4770; 3139.6707; 3142.3818; 3144.4422; 3155.8511; 3157.2458; 3163.1219; 3167.1392; 3175.6756; 3177.5785.

Cartesian Forces: Max 0.000001349 RMS 0.000000546

Item	Value	Threshold	Converged?
Maximum Force	0.000000	0.000450	YES
RMS Force	0.000000	0.000300	YES
Maximum Displacement	0.000055	0.001800	YES
RMS Displacement	0.000011	0.001200	YES

FILE: Yb_Sq2_PhYbOTf3_GS_Anion_B3LYP_M062x_Add56_Conf7-1-_Y.out

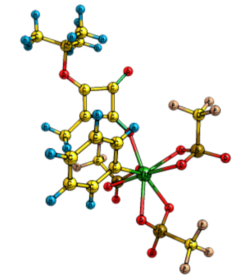

Conformer 30

E(RB3LYP/6-31G(d)/MWB59) = -3731.46356560
Convg = 0.7317D-08 -V/T = 2.0122

Temperature 195.150 Kelvin. Pressure 1.00000 Atm.
elec. and zero-point Energies= -3731.091937
elec. and thermal Energies= -3731.068123
elec. and thermal Enthalpies= -3731.067505
elec. and thermal Free Energies= -3731.139700

RB3LYP/6-31G(d)/MWB59/THF(IEFPCM) = -3731.53231207

RM06-2x/6-311+G(d,p)/MWB59/THF(IEFPCM) = -3731.36712894

Geometry at RB3LYP/6-31G(d)/MWB59
Charge = -1 Multiplicity = 1
O 2.658237259 3.2460770068 1.5636092492
S 3.2025298908 3.5944003135 0.2103354599
O 4.0919465333 4.7442486594 0.0976631129
O 2.0343822363 3.5157496083 -0.7419240112
C 4.259594227 2.1355105676 -0.2698287393
F 5.2668475077 1.9979102407 0.5996123733
F 3.5433694808 1.001776835 -0.2790374903
F 4.7678705184 2.3260242868 -1.4939370725
O 0.2569759453 5.3509289209 1.4825527668
O -1.9105368895 6.6046000694 1.0612445975
O -1.1746176117 4.5780003141 -0.2784102893
S -0.8185838733 5.7993871502 0.5270619559
C 0.0507658897 6.9246176363 -0.6783553899
F 0.5115392623 8.0021964352 -0.0302414595
F 1.0705125639 6.3051372961 -1.2711243593
F -0.8184296384 7.3257451706 -1.6168462249
C 0.5006497525 -2.4389784899 1.0515300172
C -0.3139119578 -1.3135363504 1.084753779
C 0.8304938202 -0.4200096779 1.2874471708
C 1.7650244079 -1.6398916765 1.2681253751
O 2.9471721485 -1.8621463886 1.3775744351
O 1.0256799693 0.7784513344 1.4255559614
O 0.1404718435 -3.6906894822 0.8803378604
C 1.123178379 -4.8035007409 0.8381096105
C -1.7750902716 -1.1244246903 0.9552083013
H -2.2860934415 -2.087307416 0.8618710051
H -1.9929830954 -0.503038967 0.0779918531
H -2.1684835102 -0.5860376336 1.8235462988
C 2.0829593811 -4.5866085181 -0.3336988573
H 2.7175279739 -5.4720904344 -0.4523277405
H 1.5260262669 -4.4377787979 -1.2646939583
H 2.7346399055 -3.7268326281 -0.1597910368
C 0.22490178 -6.0194467397 0.6082604072
H 0.8338099675 -6.9278332039 0.5563846744
H -0.4944840228 -6.1290125504 1.4261307399
H -0.3298883096 -5.9179840286 -0.3298381081
C 1.8491954659 -4.8887133167 2.1824350076
H 2.4969069668 -4.0225175145 2.3387845498
H 1.1294593882 -4.9591483457 3.0050209789
H 2.476711683 -5.7868992294 2.2004638226
C -0.5785424026 1.6438066886 -0.9881294644
C 0.2000490278 0.7572938843 -1.7671604028
C -1.9466840437 1.6986890037 -1.3451970008
H 1.2717763479 0.6764209393 -1.5764480463
H -2.6091688683 2.3766370557 -0.8074049186
C -0.3309213912 -0.0268678225 -2.7997520942
C -2.5010225993 0.9217160576 -2.3710357047
H 0.3176946664 -0.6899239346 -3.3729571728
H -3.5629052419 1.0012467161 -2.6036568868
C -1.6922367386 0.0473580605 -3.1027419127
H -2.1148799402 -0.5580553334 -3.9031815795
O -2.0616272685 3.6230054181 4.3177149183
S -1.2584576984 2.723035164 3.4988626236
C -1.4857362434 1.0652478329 4.3272040824
O 0.2180973996 2.9273482085 3.4338047215
O -1.7277849347 2.4846664918 2.0810514145
F -0.6422585508 0.1387506221 3.8359604393
F -2.7394773428 0.6222194571 4.1352047102
F -1.2643866792 1.1737150951 5.640587053
Yb 0.2964570842 2.9831979953 0.8497890943

Frequency: 7.7719; 8.8907; 15.7672; 24.8907; 30.2690; 32.2984; 34.0608; 35.1762; 37.0409; 40.0375; 43.6027; 46.2481; 50.7615; 53.1806; 56.4971; 60.9010; 68.5786; 72.6750; 77.2902; 82.0511; 86.9497; 87.8783; 101.9559; 104.9124; 121.1252; 132.0678; 133.5306; 140.7619; 147.1717; 151.7024; 159.0379; 168.5215; 172.9713; 177.9312; 181.8268; 202.6253; 209.4274; 210.7871; 213.1814; 215.3082; 217.8013; 218.3220; 218.7248; 238.7042; 258.3929; 274.3570; 276.3011; 301.5521; 304.4445; 306.1542; 310.4701; 320.6990; 322.6032; 323.7865; 344.3022; 346.6806; 349.6360; 352.1515; 357.4505; 393.0064; 401.1556; 453.9446; 457.8527; 480.2974; 489.0500; 489.5048; 491.0101; 494.7251; 521.9740; 525.1076; 529.7030; 556.5437; 557.0365; 558.0143; 567.0899; 572.8742; 576.2118; 601.0642; 605.5959; 618.7729; 625.2847; 634.3165; 636.3907; 641.4204; 645.2359; 722.5171; 736.2084; 745.9233; 755.4392; 758.8204; 761.9783; 769.3633; 788.2665; 864.7521; 873.6548; 912.7540; 936.2300; 943.1917; 970.5344; 974.7047; 977.2526; 979.2757; 984.7077; 984.9660; 988.3645; 1002.4291; 1011.7167; 1031.6395; 1056.4289; 1068.7388; 1070.7342; 1083.0182; 1084.2043; 1095.9445; 1109.8396; 1119.3756; 1141.7719; 1182.2397; 1203.4340; 1209.8126; 1212.6792; 1214.5160; 1215.9508; 1223.5301; 1225.9448; 1232.3312; 1236.8205; 1238.3804; 1252.4588; 1252.5698; 1286.3116; 1293.3060; 1304.5194; 1304.8642; 1309.3153; 1320.0678; 1370.2242; 1400.1263; 1432.2539; 1433.5945; 1444.2081; 1457.5468; 1460.1109; 1494.3646; 1503.9301; 1504.7899; 1518.4453; 1518.8833; 1521.0217; 1535.4155; 1538.1626; 1555.3679; 1611.7235; 1621.7780; 1626.0633; 1816.3818; 1873.1189; 3055.5327; 3058.5794; 3059.6331; 3066.7885; 3123.4636; 3124.1897; 3126.6340; 3126.7411; 3137.0065; 3138.7451; 3139.3839; 3145.2453; 3148.2262; 3156.5612; 3160.1806; 3160.2706; 3174.2229.

Cartesian Forces: Max 0.000000779 RMS 0.000000200

Item	Value	Threshold	Converged?
Maximum Force	0.000000	0.000450	YES
RMS Force	0.000000	0.000300	YES
Maximum Displacement	0.000071	0.001800	YES
RMS Displacement	0.000016	0.001200	YES

FILE: Yb_Sq2_PhYbOTf3_GS_Anion_B3LYP_M062x_Add56_Conf8_Y.out

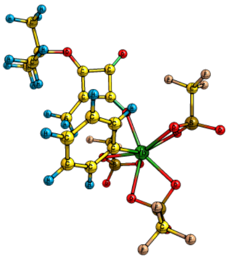

Conformer 31

E(RB3LYP/6-31G(d)/MWB59) = -3731.45787207
Convg = 0.1561D-09 -V/T = 2.0122

Temperature 195.150 Kelvin. Pressure 1.00000 Atm.
elec. and zero-point Energies= -3731.086288
elec. and thermal Energies= -3731.062473
elec. and thermal Enthalpies= -3731.061855
elec. and thermal Free Energies= -3731.134490

RB3LYP/6-31G(d)/MWB59/THF(IEFPCM) = -3731.52668814

RM06-2x/6-311+G(d,p)/MWB59/THF(IEFPCM) = -3731.36841817

Geometry at RB3LYP/6-31G(d)/MWB59
Charge = -1 Multiplicity = 1
Yb 0.4263389522 2.6763496836 0.9293297589
O 2.764224836 2.8861204153 1.7496682775
S 3.42381018 2.7991761347 0.4028324446
O 4.3440168206 3.8633176032 0.0177021909
O 2.3315604578 2.4336484782 -0.5726955047
C 4.5090927362 1.2834168613 0.4795950891
F 5.4586841583 1.4627986171 1.4075310078
F 3.815777929 0.1841284043 0.7827135881
F 5.0996271422 1.1013364653 -0.7108749667
O 0.9332873754 4.9705558399 0.3931420153
O -0.6767446833 6.7737636284 1.1723547608
O -1.3538599518 4.3580975461 0.7862472282
S -0.4665116963 5.5264442496 0.4494241401
C -0.8621231141 5.9244704951 -1.3278206767
F -0.1225412956 6.965512183 -1.7298754801
F -0.6055597736 4.8811546107 -2.119344658
F -2.1576101793 6.2472233134 -1.4389976297
C 0.1914917981 -2.7448882514 0.9099705338
C -0.5246939165 -1.5510782254 1.0032033961
C 0.7077748316 -0.7838762083 1.2623572322
C 1.5116936038 -2.0965443389 1.2351385152
O 2.6397732173 -2.4786172892 1.4025052328
O 0.9984571292 0.3945005418 1.4090059168
O 0.0484225168 -4.0247001355 0.6567927831
C -1.1934170208 -4.6926705687 0.2082228009
C -1.9252193643 -1.0690805482 0.9044223068
H -2.6595464442 -1.8473226401 1.1256940015
H -2.1197831951 -0.6760447009 -0.1015777317
H -2.0681620518 -0.2279593792 1.5887025182
C -2.1758865863 -4.7516040424 1.3803194463
H -3.0690963846 -5.3137477809 1.0863384689
H -1.7166184239 -5.2579392079 2.2354668268
H -2.4953094069 -3.7577983023 1.7008249273
C -0.6936668238 -6.0961921619 -0.1454357938
H -1.5312674087 -6.7200534304 -0.4745060202
H 0.0449766715 -6.0495274811 -0.9514072589
H -0.2263634885 -6.5684251414 0.7242529104
C -1.7524853814 -3.9913633647 -1.0314946852
H -2.0989382571 -2.9767415177 -0.8289116056
H -0.9908173292 -3.9420260558 -1.8161239812
H -2.6027863307 -4.5645802942 -1.4171586317
C -0.7474476841 1.6589317805 -0.9377187527
C -0.125927214 0.7612863235 -1.8369194589
C -2.1393993069 1.825243844 -1.1313213113
H 0.9515050565 0.6052375705 -1.7727060315
H -2.6867804882 2.5093997023 -0.4835195344
C -0.8271177796 0.0621344423 -2.8283767986
C -2.8604783882 1.140873241 -2.1179540951
H -0.2984615819 -0.6173381427 -3.4975042318
H -3.9327904747 1.3048722049 -2.2246446006
C -2.20554411 0.2450660978 -2.9692146526
H -2.7587921863 -0.2901230583 -3.7398058534
O -2.174851905 3.2177171487 4.3018766482
S -1.0957223182 2.5232003565 3.6102687061
C -0.5612760477 1.196014556 4.8056272983
O 0.1672385692 3.2784885334 3.3139802938
O -1.4412950991 1.7613656463 2.3587202691
F 0.4981656873 0.5198453734 4.34040401
F -1.5674449439 0.325000175 4.9912887938
F -0.2388195066 1.7370741003 5.9846248573

Frequency: 6.2129; 11.8122; 14.7223; 19.7757; 22.7028; 26.4444; 27.3588; 28.0882; 34.6231; 37.0407; 42.9739; 47.1759; 51.7542; 57.9977; 64.9637; 67.1364; 69.7732; 73.6521; 75.8651; 79.4055; 89.8171; 92.6623; 107.8628; 116.2499; 128.1852; 131.0951; 139.0398; 144.3841; 148.2510; 152.0038; 161.6366; 167.2577; 179.0003; 180.9366; 183.5301; 191.4160; 207.7260; 211.5044; 213.7346; 214.7975; 215.6499; 220.4826; 222.2717; 233.7476; 237.3200; 260.5728; 288.8084; 295.5114; 303.2698; 306.6941; 307.1554; 321.2948; 324.0850; 324.6014; 347.4979; 349.6634; 351.9271; 354.3656; 358.6209; 393.2625; 398.6609; 450.2038; 450.6757; 482.8798; 488.5446; 489.6722; 490.9210; 492.6804; 520.6135; 523.7796; 529.2782; 557.0981; 557.7042; 558.1589; 568.3545; 569.8646; 576.5188; 598.8315; 606.1259; 620.1577; 624.8517; 633.9199; 634.9812; 646.1344; 649.2444; 720.8280; 736.3175; 757.6255; 760.6681; 761.3193; 767.0742; 778.3371; 783.6697; 848.0643; 872.7195; 912.0022; 931.9945; 935.6236; 974.8482; 977.0382; 977.3121; 977.7165; 980.0425; 983.2317; 986.2353; 1003.1298; 1011.1984; 1031.8158; 1052.2320; 1061.8569; 1064.1756; 1081.9985; 1082.5776; 1100.8041; 1111.6749; 1116.0474; 1130.9423; 1181.5778; 1202.8719; 1207.2551; 1212.6606; 1213.8405; 1214.7169; 1222.5992; 1223.7021; 1230.2750; 1234.7263; 1238.9706; 1244.0621; 1244.6512; 1284.9051; 1293.1599; 1293.6828; 1305.1529; 1308.1868; 1319.0742; 1366.5091; 1387.2767; 1431.8215; 1434.4208; 1442.5522; 1457.5633; 1459.5533; 1495.2155; 1503.8596; 1510.2610; 1515.6341; 1517.7753; 1520.2844; 1528.8081; 1531.5034; 1549.1519; 1609.7643; 1620.0751; 1621.2039; 1822.2762; 1895.0577; 3058.0080; 3062.0495; 3066.4039; 3073.1392; 3123.5793; 3129.6985; 3130.9100; 3131.6372; 3139.6336; 3141.2679; 3142.0564; 3152.5769; 3153.2270; 3161.9450; 3164.7533; 3173.2771; 3177.5368.

Cartesian Forces: Max 0.000000220 RMS 0.000000080

Item	Value	Threshold	Converged?
Maximum Force	0.000000	0.000450	YES
RMS Force	0.000000	0.000300	YES
Maximum Displacement	0.000042	0.001800	YES
RMS Displacement	0.000009	0.001200	YES

FILE: Yb_Sq2_PhYbOTf3_GS_Anion_B3LYP_M062x_Add56_Conf8-_Y.out

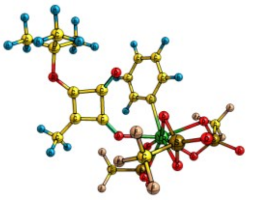

Conformer 32

E(RB3LYP/6-31G(d)/MWB59) = -3731.46011200
Convg = 0.7242D-09 -V/T = 2.0122

Temperature 195.150 Kelvin. Pressure 1.00000 Atm.
elec. and zero-point Energies= -3731.088711
elec. and thermal Energies= -3731.064816
elec. and thermal Enthalpies= -3731.064198
elec. and thermal Free Energies= -3731.136570

RB3LYP/6-31G(d)/MWB59/THF(IEFPCM) = -3731.53230303

RM06-2x/6-311+G(d,p)/MWB59/THF(IEFPCM) = -3731.36679810

Geometry at RB3LYP/6-31G(d)/MWB59
Charge = -1 Multiplicity = 1
Yb 0.5226575645 2.6865601515 -0.1264352057
O -1.8270964564 3.0922016221 -0.8602389294
S -2.3125429575 3.7168721572 0.4141652659
O -3.0353014201 4.9804046261 0.3253081363
O -1.1857233476 3.6084238917 1.4032938976
C -3.5914501904 2.5245352639 1.0814224838
F -4.4329227944 2.1452359233 0.1112959897
F -3.0138472323 1.4198461318 1.5934566127
F -4.2965080948 3.1073572069 2.0585026562
O 0.5673522905 5.0974537399 -0.4992461076
O 2.6300129646 6.0965707064 -1.5957634449
O 2.6256645128 3.8671157847 -0.3798797446
S 2.0627323891 5.2548421193 -0.5506184775
C 2.4779408446 6.1128382185 1.0498957146
F 1.9836939532 7.3572891521 1.037743846
F 1.958058682 5.4511979778 2.0872291122
F 3.8073220119 6.1788059775 1.1985591926
C -0.2906156938 -2.337877554 1.4878773734
C -1.3296953996 -1.5969272415 0.9498876426
C -0.4448624887 -0.5149031312 0.5294071585
C 0.7700098241 -1.3482507507 1.0155162788
O 1.9689737704 -1.3258287942 0.97229226
O -0.6439270645 0.5815040271 0.0239141845
O -0.3486317884 -3.4775641038 2.1322621313
C 0.8576785691 -4.1127468364 2.7399947178
C -2.7979066799 -1.7898764313 0.8521601379
H -3.1362737813 -2.604683015 1.4992541152
H -3.0834169632 -2.0280446645 -0.1810072302
H -3.3181148299 -0.8657321581 1.1204518003
C 1.8287924092 -4.5102777234 1.627298673
H 2.6522287737 -5.0917634914 2.0570586163
H 1.3250726832 -5.1338377213 0.8805649655
H 2.2548887622 -3.6297944465 1.1407370287
C 0.2581564795 -5.3410737329 3.4255183157
H 1.0504535915 -5.9139143608 3.918038689
H -0.4758841835 -5.0436788449 4.1812703262
H -0.2370880271 -5.9900355328 2.6957846804
C 1.4788385218 -3.1522100076 3.7550043524
H 1.9100376291 -2.2683069417 3.2792469839
H 0.7325562953 -2.8303634463 4.4887757964
H 2.2830689293 -3.668626146 4.2912319355
C 1.4279248771 1.8990509603 1.9765232236
C 0.6232581951 1.5302510605 3.0801344531
C 2.8122013143 1.6669241051 2.1453875708
H -0.4534216248 1.7003258214 3.0363272564
H 3.4959935169 1.9213510124 1.3361576912
C 1.1441953462 0.9640863962 4.2517773188
C 3.3549248212 1.1070693875 3.3073953406
H 0.4803929834 0.705072541 5.0775869564
H 4.4301223726 0.9472861854 3.3858363428
C 2.5195565066 0.7483298205 4.3694189561
H 2.9360253652 0.3172468794 5.2791201823
O 2.9026012999 1.6412681736 -3.5121450305
S 1.7502637658 1.4303171324 -2.6436042066
C 0.8684549617 -0.0225278468 -3.4107941153
O 0.700042071 2.5050843999 -2.5882641694
O 2.0018370337 1.004506077 -1.2258222178
F -0.2576938973 -0.3224151317 -2.7433258885
F 1.6645837718 -1.1020934505 -3.3938000116
F 0.5453641094 0.2519109919 -4.6802000677

Frequency: 9.3908; 16.0110; 16.7034; 21.0869; 23.2189; 26.0839; 28.5173; 32.2418; 36.4835; 38.1540; 44.1027; 46.9520; 47.8105; 53.1144; 59.1063; 61.0969; 67.9545; 72.9919; 74.3898; 80.8893; 86.3006; 88.7290; 97.1223; 114.8949; 115.5210; 123.2449; 133.4001; 141.0561; 146.1247; 151.0696; 156.0675; 161.8165; 167.7856; 177.6264; 186.7490; 204.8286; 209.6531; 211.2204; 213.3309; 214.6944; 216.3672; 219.0188; 225.5682; 231.0972; 257.4745; 269.6071; 277.0393; 302.2151; 306.1833; 307.0209; 319.7515; 321.1282; 323.8073; 327.5685; 340.3362; 348.7891; 349.3001; 350.6842; 357.7064; 392.0004; 396.5997; 451.2410; 453.1612; 470.4845; 488.9278; 489.9638; 490.7829; 495.0852; 521.5597; 522.8964; 529.2104; 555.8507; 556.7152; 557.7490; 568.6442; 569.7346; 577.5197; 595.5495; 610.4658; 618.9988; 621.8748; 632.9200; 634.1765; 634.5682; 646.7445; 717.7514; 733.7555; 746.8865; 754.4449; 756.8719; 760.8072; 777.1715; 786.7710; 852.8441; 867.9896; 907.5456; 936.5665; 940.7534; 954.2991; 970.7229; 977.5046; 978.7944; 983.3870; 985.9420; 986.9026; 992.5979; 1011.2544; 1032.5043; 1062.5208; 1065.0468; 1069.7517; 1082.3300; 1084.4431; 1087.8028; 1113.8189; 1127.8426; 1138.4016; 1181.3265; 1197.2388; 1205.2882; 1215.4697; 1217.5196; 1220.0477; 1220.7914; 1223.6437; 1233.8809; 1234.5766; 1235.2978; 1240.3190; 1259.2210; 1287.5699; 1295.9377; 1301.7991; 1306.6777; 1308.5153; 1320.3450; 1368.5744; 1402.0782; 1431.9456; 1435.1264; 1448.5954; 1457.8413; 1460.8753; 1502.6882; 1506.4536; 1512.7355; 1514.6168; 1519.2449; 1519.9649; 1534.5089; 1537.8899; 1554.4007; 1610.8959; 1622.6734; 1638.0077; 1820.7830; 1886.0894; 3051.2190; 3057.3146; 3059.7203; 3066.6450; 3114.9259; 3122.2197; 3126.0810; 3126.5610; 3134.2966; 3138.2441; 3140.3596; 3148.7583; 3152.7716; 3160.2574; 3160.5702; 3165.0243; 3171.9017.

Cartesian Forces: Max 0.000000729 RMS 0.000000208

Item	Value	Threshold	Converged?
Maximum Force	0.000000	0.000450	YES
RMS Force	0.000000	0.000300	YES
Maximum Displacement	0.000047	0.001800	YES
RMS Displacement	0.000010	0.001200	YES

FILE: Yb_Sq2_PhYbOTf3_GS_Anion_B3LYP_M062x_Add56_Conf8-1.out

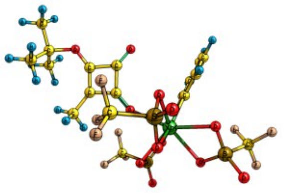

Conformer 33

E(RB3LYP/6-31G(d)/MWB59) = -3731.44950471
Convg = 0.6751D-08 -V/T = 2.0122

Temperature 195.150 Kelvin. Pressure 1.00000 Atm.
elec. and zero-point Energies= -3731.078156
elec. and thermal Energies= -3731.054143
elec. and thermal Enthalpies= -3731.053525
elec. and thermal Free Energies= -3731.126789

RB3LYP/6-31G(d)/MWB59/THF(IEFPCM) = -3731.52644256

RM06-2x/6-311+G(d,p)/MWB59/THF(IEFPCM) = -3731.36011706

Geometry at RB3LYP/6-31G(d)/MWB59
Charge = -1 Multiplicity = 1
Yb 0.5254703242 2.568433686 0.3026539517
O -1.8277004344 2.7560964883 -0.6362876594
S -2.4074401574 3.4870472688 0.5348899088
O -3.1700343431 4.7036159285 0.2769886466
O -1.3376201045 3.5361082556 1.5907489962
C -3.6881238579 2.3193740352 1.2455456364
F -4.44807405 1.8141716933 0.2579385847
F -3.1288691723 1.2873036043 1.9015440667
F -4.4813173315 2.9713649238 2.1032801241
O 0.5435487351 4.8779717633 -0.3478887804
O 2.3565668681 5.6591988973 -1.9450188952
O 2.606897253 3.6493893879 -0.4125159736
S 2.0085293197 5.0000526171 -0.6927899293
C 2.6908478416 6.1097316501 0.6401739925
F 2.2065038014 7.3490563393 0.4800645268
F 2.3543737298 5.6683741989 1.8520034112
F 4.0260418971 6.1542928306 0.5457601334
C -0.7335701076 -2.656758034 1.6166708593
C -1.5348180019 -1.7482838259 0.9355400125
C -0.5923564194 -0.6457585444 1.175960896
C 0.3237234989 -1.6196214811 1.9560297016
O 1.361838897 -1.6299010891 2.5522017839
O -0.6086838092 0.5267994683 0.8465482784
O -0.6561225455 -3.9184221936 1.9665247412
C -1.6436686044 -4.9678111732 1.6382754016
C -2.797785142 -1.6655664362 0.1527067381
H -3.5211664549 -2.4480861136 0.391938626
H -2.5681142559 -1.7211338033 -0.9192600008
H -3.2624129448 -0.6897879016 0.3230081201
C -2.9550410324 -4.6651465701 2.3671202012
H -3.672662549 -5.4733871604 2.1887124937
H -2.7818035533 -4.5906843555 3.445357771
H -3.4095849652 -3.7312057702 2.0284470307
C -0.9839062342 -6.2255210703 2.2089716425
H -1.6308316848 -7.0943682968 2.0488665052
H -0.0223810929 -6.4102784123 1.7204949
H -0.808456548 -6.1126852298 3.2831155971
C -1.7981701624 -5.080426795 0.1198525341
H -2.2526563746 -4.1915733942 -0.3221555963
H -0.8227486789 -5.2406742209 -0.3502318913
H -2.4381763993 -5.9368666683 -0.1188557552
C 1.569149075 2.4209813554 2.4513598083
C 0.9544882509 2.7774680473 3.6738768069
C 2.8914869298 1.9310851045 2.5636292594
H -0.061544922 3.1712487448 3.6641763346
H 3.4303399403 1.6351123557 1.6630716473
C 1.6000597611 2.6594681557 4.910460187
C 3.5529077222 1.8044059251 3.7899597928
H 1.0854788483 2.9523597848 5.8260468478
H 4.5720315782 1.4197931366 3.8240652872
C 2.906549805 2.1701159466 4.9729479373
H 3.4154006814 2.0756661392 5.9314910693
O 2.9000766471 0.8325562084 -2.8274812448
S 1.7074674849 0.858829418 -1.9889565172
C 0.7629636873 -0.6602965455 -2.5199915201
O 0.7239245352 1.9724051261 -2.1673982303
O 1.8900963925 0.6640988804 -0.5071748861
F -0.4351726618 -0.7326854098 -1.9030817287
F 1.4500828241 -1.7712053549 -2.2134386293
F 0.5483279187 -0.6399753273 -3.8394686013

Frequency: 6.0466; 11.2478; 19.1935; 20.2654; 24.3839; 26.7895; 27.7536; 29.1148; 31.0230; 33.0335; 37.0032; 42.7326; 51.2637; 54.6900; 60.9411; 64.2305; 66.0523; 71.7114; 73.9278; 79.0692; 83.6725; 92.5218; 95.6038; 107.3901; 121.6349; 128.7662; 129.5907; 137.0513; 141.1382; 154.9139; 160.8424; 167.6336; 174.6778; 177.9189; 183.6834; 189.5267; 205.8666; 210.8355; 213.2905; 214.0591; 217.6803; 221.7079; 223.8658; 235.3275; 238.0697; 259.6051; 268.6526; 294.8088; 302.3784; 305.6859; 307.1546; 321.7226; 323.9827; 324.9414; 336.7312; 347.1704; 349.0996; 352.4040; 358.0343; 394.2954; 399.7739; 449.0066; 449.3792; 481.0598; 484.2973; 488.3225; 490.8002; 491.3459; 520.6265; 522.2839; 528.9993; 555.1184; 557.0109; 558.4623; 567.5068; 568.6171; 575.7701; 600.4027; 603.5537; 618.6509; 622.1386; 632.4150; 634.4049; 650.4057; 652.9289; 716.9448; 736.6522; 753.5784; 753.8860; 761.5453; 767.8516; 775.3247; 786.6921; 848.9773; 867.5507; 912.3665; 933.7457; 935.0529; 969.8134; 973.2812; 976.2325; 979.3006; 980.6330; 983.8359; 987.0960; 998.6411; 1011.7361; 1035.3467; 1056.4126; 1062.0791; 1062.7926; 1084.8546; 1085.7651; 1086.4993; 1113.0665; 1130.6517; 1143.4207; 1182.8536; 1193.4190; 1203.7342; 1209.3656; 1213.1993; 1213.3608; 1216.3277; 1221.3804; 1225.4345; 1229.6064; 1232.9322; 1237.2382; 1248.9806; 1285.6079; 1294.8381; 1295.3331; 1306.9969; 1308.2087; 1318.6921; 1370.4433; 1384.1662; 1432.6335; 1433.8697; 1443.9808; 1458.7502; 1459.4303; 1500.9741; 1507.4293; 1512.7245; 1518.4025; 1521.4814; 1522.1407; 1528.5954; 1530.6644; 1550.6421; 1614.5766; 1627.0377; 1637.0875; 1844.3705; 1908.8708; 3059.5396; 3063.1740; 3066.8234; 3073.3917; 3125.4782; 3131.4517; 3131.8579; 3132.2578; 3139.4633; 3141.0152; 3143.9624; 3153.1024; 3154.3564; 3160.7009; 3160.9175; 3168.7195; 3172.1452.

Cartesian Forces: Max 0.000001138 RMS 0.000000458

Item	Value	Threshold	Converged?
Maximum Force	0.000001	0.000450	YES
RMS Force	0.000000	0.000300	YES
Maximum Displacement	0.000040	0.001800	YES
RMS Displacement	0.000009	0.001200	YES

FILE: Yb_Sq2_PhYbOTf3_GS_Anion_B3LYP_M062x_Add56_Conf8-1-_Y.out

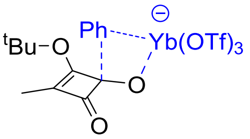	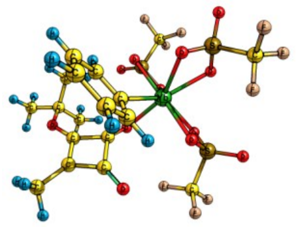	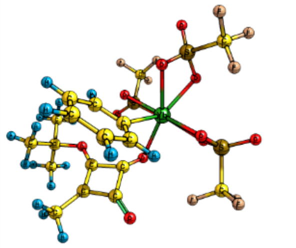	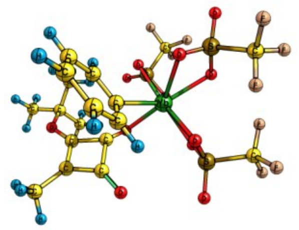
**Trapezoidal Transition State for [PhYb(OTf)$_3$]$^-$ addition to C-1 carbonyl of 1b**	*Conformer 1*	*Conformer 2*	*Conformer 3*
Lowest energy conformation is:<br>Conformer 14	E(RB3LYP/6-31G(d)/MWB59) = -3731.44997651<br>Convg = 0.4799D-08 -V/T = 2.0122<br><br>Temperature 195.150 Kelvin. Pressure 1.00000 Atm.<br>elec. and zero-point Energies= -3731.078850<br>elec. and thermal Energies= -3731.055672<br>elec. and thermal Enthalpies= -3731.055054<br>elec. and thermal Free Energies= -3731.124064<br><br>RB3LYP/6-31G(d)/MWB59/THF(IEFPCM) = -3731.51314208<br><br>RM06-2x/6-311+G(d,p)/MWB59/THF(IEFPCM) = -3731.35107377	E(RB3LYP/6-31G(d)/MWB59) = -3731.44975615<br>Convg = 0.5129D-08 -V/T = 2.0122<br><br>Temperature 195.150 Kelvin. Pressure 1.00000 Atm.<br>elec. and zero-point Energies= -3731.078586<br>elec. and thermal Energies= -3731.055357<br>elec. and thermal Enthalpies= -3731.054739<br>elec. and thermal Free Energies= -3731.124345<br><br>RB3LYP/6-31G(d)/MWB59/THF(IEFPCM) = -3731.51664446<br><br>RM06-2x/6-311+G(d,p)/MWB59/THF(IEFPCM) = -3731.35441890	E(RB3LYP/6-31G(d)/MWB59) = -3731.44928655<br>Convg = 0.7663D-08 -V/T = 2.0122<br><br>Temperature 195.150 Kelvin. Pressure 1.00000 Atm.<br>elec. and zero-point Energies= -3731.078174<br>elec. and thermal Energies= -3731.054987<br>elec. and thermal Enthalpies= -3731.054369<br>elec. and thermal Free Energies= -3731.123434<br><br>RB3LYP/6-31G(d)/MWB59/THF(IEFPCM) = -3731.51362671<br><br>RM06-2x/6-311+G(d,p)/MWB59/THF(IEFPCM) = -3731.35311068
	Geometry at RB3LYP/6-31G(d)/MWB59<br>Charge = -1 Multiplicity = 1<br>Yb 0.6462859425 2.1100292656 1.0769040344<br>O 2.5786416309 1.8594968485 -0.3707140512<br>S 3.6522461995 1.7000967575 0.6850000106<br>O 4.5646170439 0.56764269 0.5241975309<br>O 2.9689175067 1.8688443644 2.0042031341<br>C 4.7340453816 3.202773055 0.4713930117<br>F 5.3284767116 3.1515612832 -0.7283609628<br>F 4.0240456996 4.3235563487 0.5587568585<br>F 5.6784562477 3.203560655 1.4209766292<br>O 0.134101769 3.9179962936 -0.5057684895<br>O 1.5465800762 6.0214325451 -0.3227715902<br>O 1.1273501544 4.4344565943 1.6133429046<br>S 0.6604747548 5.0587924331 0.3222115944<br>C -0.8518244482 6.0426593101 0.7906947788<br>F -1.3702544479 6.6087247138 -0.3078921899<br>F -1.7754276291 5.2628733992 1.3520738804<br>F -0.509896027 7.0077038152 1.6524663288<br>C 0.0764999517 -2.0335872701 -0.1113670958<br>C -1.255768648 -2.3418323257 0.0560672152<br>C -1.5016163752 -1.2059888241 0.9583383472<br>C -0.015265236 -0.7942630051 0.7723509282<br>O 0.8180235925 -0.1690247002 1.4608520506<br>O -2.4277398084 -0.8316776111 1.6392012508<br>O 0.9501587851 -2.7356272891 -0.8043624235<br>C 2.4298846581 -2.6142683538 -0.7248795931<br>C -2.1231140999 -3.4414427854 -0.4329423486<br>H -1.5311975264 -4.2868531837 -0.7984016243<br>H -2.7565898042 -3.086416291 -1.2574191883<br>H -2.7951147181 -3.7849057508 0.3619902691<br>C 2.8659290083 -1.4011193458 -1.538381215<br>H 3.9586577126 -1.3494402517 -1.5700870852<br>H 2.4832790048 -1.474090778 -2.5619964936<br>H 2.5169698539 -0.469989266 -1.0935457814<br>C 2.8867486104 -3.915747376 -1.3898387144<br>H 3.9805675121 -3.946570271 -1.4282145986<br>H 2.5332296717 -4.7861947055 -0.8268850024<br>H 2.5014017855 -3.9830123252 -2.4125634375<br>C 2.8821343882 -2.5553472449 0.7337201715<br>H 2.5440598011 -1.6437426487 1.2292744808<br>H 2.5124451069 -3.427665601 1.2856898476<br>H 3.9761622311 -2.560526903 0.7704313199<br>C -0.6978615355 1.0534048674 -0.7955107633<br>C -0.0851945718 0.8734900929 -2.0552643821<br>C -2.1010322112 1.2245413463 -0.8111507395<br>H 0.9952832503 0.766797472 -2.1184793692<br>H -2.6325793317 1.3650714346 0.1267779201<br>C -0.8119149235 0.8670014183 -3.2506811398<br>C -2.8401341861 1.2249836593 -1.9978984919<br>H -0.2971262759 0.734688508 -4.2015616192<br>H -3.9187008014 1.3735317283 -1.9666326254<br>C -2.1973200569 1.0378497943 -3.2246449801<br>H -2.7698045232 1.0326874685 -4.1506465521<br>O -1.5334852031 3.5740311581 4.3502165798<br>S -1.0002130444 2.4509804853 3.5880232087<br>C -1.6677825953 0.9384981622 4.4585466423<br>O 0.4843621467 2.2443348227 3.554063533<br>O -1.4623936442 2.3256733748 2.1503844485<br>F -1.0391318708 -0.1690204034 4.0616449302<br>F -2.9768920565 0.8171077459 4.2315557692<br>F -1.4699976975 1.0850587462 5.7784020637<br><br>Frequency: -142.3232; 17.0263; 19.6327; 25.4321; 27.3240; 31.0599; 34.3557; 35.7927; 39.5122; 40.8132; 45.0549; 51.1635; 54.5830; 57.5530; 63.6965; 67.6766; 72.3238; 74.4047; 77.4067; 80.4844; 83.6021; 92.3554; 93.3516; 104.0239; 116.4249; 120.9918; 133.5200; 139.2035; 141.4569; 146.6456; 153.8375; 156.5186; 166.0894; 179.8124; 188.5775; 196.2580; 209.1409; 211.1781; 213.3112; 215.6169; 217.5620; 220.4927; 225.3762; 252.8687; 268.6864; 280.5678; 290.1009; 303.1044; 303.7460; 307.5011; 309.2123; 321.0869; 323.5863; 323.8060; 337.6133; 349.6872; 351.8360; 353.2049; 358.6237; 394.9387; 401.3795; 422.5955; 454.2122; 483.3080; 489.4604; 489.7580; 491.8556; 492.7979; 524.1779; 526.0363; 530.1420; 557.6047; 557.8831; 558.7452; 571.3174; 573.5058; 574.2520; 587.2866; 604.0224; 623.5174; 626.9819; 627.6398; 630.2784; 631.7593; 636.3465; 715.2744; 722.9173; 735.3560; 755.8678; 761.5818; 762.1520; 763.3335; 789.2759; 858.2788; 866.6604; 923.1633; 936.4080; 941.1749; 958.8591; 972.7401; 972.9662; 975.0943; 978.4445; 981.7428; 988.3023; 991.3252; 1006.8196; 1014.3112; 1053.2842; 1058.6114; 1067.2958; 1072.9039; 1075.7693; 1090.9061; 1104.6221; 1109.3672; 1132.9196; 1185.1948; 1203.7180; 1206.9722; 1211.8648; 1212.4133; 1223.3311; 1231.5999; 1233.3872; 1235.4175; 1250.3252; 1252.3265; 1253.3793; 1257.5949; 1289.8763; 1293.4175; 1298.3133; 1299.8277; 1306.1104; 1312.3177; 1368.1459; 1384.4453; 1429.9637; 1438.9322; 1442.0453; 1460.9263; 1463.4700; 1499.0119; 1504.7323; 1510.0111; 1515.1087; 1517.2858; 1519.5759; 1525.9655; 1530.0022; 1552.0901; 1613.7568; 1614.9367; 1620.6679; 1682.7055; 1871.2323; 3039.3362; 3055.3238; 3060.0965; 3071.1120; 3095.4552; 3125.4941; 3128.3327; 3129.8023; 3131.8096; 3139.5010; 3146.7291; 3157.5405; 3173.3027; 3176.4598; 3186.9197; 3194.8018; 3195.6940.<br><br>Cartesian Forces: Max 0.000001020 RMS 0.000000403<br><br>Item Value Threshold Converged?<br>Maximum Force 0.000001 0.000450 YES<br>RMS Force 0.000000 0.000300 YES<br>Maximum Displacement 0.000024 0.001800 YES<br>RMS Displacement 0.000004 0.001200 YES	Geometry at RB3LYP/6-31G(d)/MWB59<br>Charge = -1 Multiplicity = 1<br>Yb -3.8594158041 -2.9316782993 0.4838933294<br>O -2.5120117729 -4.6190426588 -0.6204365207<br>S -2.8974127705 -4.3389415173 -2.0549887741<br>O -1.8033588625 -4.1858262857 -3.0144168569<br>O -3.9513519276 -3.2788180339 -2.0109629286<br>C -3.7741787658 -5.8904571212 -2.5983262691<br>F -2.9093387994 -6.9153728255 -2.5926246443<br>F -4.7908856114 -6.1764448142 -1.7885918988<br>F -4.2378929672 -5.7271128175 -3.8445220668<br>O -4.3499440003 -4.8374381706 1.9675672706<br>O -5.9586112784 -6.581527518 1.0620744087<br>O -5.9567748451 -4.1532576641 0.3243371459<br>S -5.6959202622 -5.1851209285 1.3933653244<br>C -6.9122081787 -4.7855263509 2.7484872195<br>F -6.7295754756 -5.6338088065 3.7704056889<br>F -6.7518004717 -3.5385084441 3.1884985963<br>F -8.1602121926 -4.9305324314 2.2866183996<br>C 0.0080821744 -1.7187958157 0.1498734595<br>C 0.5747488326 -0.7023974363 0.9044439037<br>C -0.7449414913 -0.0971030143 1.2086918078<br>C -1.3852586724 -1.1928182836 0.3237635974<br>O -2.41926458 -1.3147827873 -0.3444133067<br>O -1.108181623 0.8824282441 1.8163661407<br>O 0.2929437288 -2.7674819864 -0.574902069<br>C 1.6381169368 -3.3132163354 -0.8382092159<br>C 1.9006329812 -0.20806208 1.3639232428<br>H 2.6711531398 -0.2369428856 0.5863340152<br>H 2.2626332239 -0.7966520176 2.217452095<br>H 1.7871977359 0.8263373646 1.7066840513<br>C 2.3656167632 -2.3536080881 -1.783530773<br>H 3.3485322158 -2.7619840693 -2.0444005201<br>H 1.79003048 -2.2199023409 -2.7046621592<br>H 2.5192082319 -1.3715678519 -1.3250327358<br>C 1.3209004636 -4.6390388203 -1.5333912492<br>H 2.2486204821 -5.1089555591 -1.8789195879<br>H 0.8155695083 -5.3198379984 -0.8420596348<br>H 0.6538689396 -4.4746975355 -2.3833607241<br>C 2.3898874981 -3.5502196459 0.4741013761<br>H 2.7483013548 -2.6229991379 0.9264780671<br>H 1.7446516472 -4.0610450024 1.1948230309<br>H 3.262851521 -4.1828131345 0.2776595751<br>C -2.0299985891 -2.8947142678 2.1922922253<br>C -1.1376889731 -3.991418571 2.2558625651<br>C -2.0807083193 -2.0817079078 3.3468165683<br>H -1.0645827573 -4.666538895 1.4045887246<br>H -2.751156994 -1.2248460573 3.3679023528<br>C -0.3602816673 -4.2650008672 3.3857045822<br>C -1.3019573607 -2.3356194348 4.4825866581<br>H 0.2939380771 -5.1368443616 3.4026698119<br>H -1.3752118792 -1.6817781984 5.3506134102<br>C -0.4338545343 -3.428560716 4.5044226038<br>H 0.1716084981 -3.6339022286 5.385782426<br>O -7.2469730167 -0.5981202397 1.3789197908<br>S -5.8345498009 -0.7014583862 1.0309030156<br>C -5.2105241656 1.0541657243 1.0759480156<br>O -5.4702812645 -1.2164186827 -0.3284776883<br>O -4.936759702 -1.4348146959 2.0058536916<br>F -3.9161748695 1.1173839005 0.7667449939<br>F -5.3911684136 1.5614207916 2.3022623166<br>F -5.903612864 1.792776394 0.197737853<br><br>Frequency: -99.4389; 14.5561; 16.2869; 24.0727; 25.8422; 27.7579; 32.1920; 34.2354; 37.1019; 41.0941; 42.0789; 48.1264; 49.8756; 54.9839; 57.0842; 65.8466; 68.5344; 74.4447; 75.3875; 78.6329; 86.1872; 87.8736; 96.1812; 116.4922; 129.3122; 134.6580; 135.8504; 144.3229; 144.8590; 148.4844; 155.6819; 156.9309; 167.7706; 179.2151; 189.4687; 203.0678; 209.3167; 210.4097; 212.1355; 214.1867; 218.6211; 221.4904; 223.9858; 236.1741; 252.5998; 264.4203; 291.7581; 300.3040; 303.6608; 306.8697; 308.8386; 321.0609; 323.2686; 323.5217; 334.8177; 349.9542; 351.5968; 352.8878; 358.4026; 395.5030; 397.9716; 421.8955; 454.6583; 482.9602; 484.1948; 488.9211; 489.4989; 491.1018; 523.2790; 525.4818; 529.5484; 557.7456; 557.9044; 558.5263; 570.8043; 573.1695; 573.8407; 587.4648; 608.2228; 625.2457; 626.7849; 630.8659; 631.6742; 635.6028; 647.3196; 715.6099; 731.0814; 760.5823; 761.8822; 762.1549; 762.3229; 765.7425; 783.0725; 848.3806; 869.2242; 918.9318; 935.7363; 939.5043; 973.1861; 974.2717; 976.2555; 979.8941; 982.4345; 984.2669; 987.3243; 994.6210; 1011.9541; 1014.2688; 1052.4816; 1061.6836; 1069.7178; 1075.4545; 1086.5169; 1100.9170; 1107.1178; 1113.0900; 1135.9729; 1183.9746; 1198.2579; 1211.8425; 1213.0483; 1213.2800; 1214.7047; 1223.3616; 1231.0611; 1231.8907; 1233.9056; 1250.6160; 1252.4834; 1253.8166; 1282.2378; 1291.6806; 1296.1542; 1298.4164; 1305.6977; 1312.3599; 1368.4486; 1387.6571; 1429.5073; 1436.9405; 1438.1509; 1459.2895; 1461.3341; 1500.5474; 1507.8207; 1514.0857; 1515.7783; 1517.2925; 1521.3376; 1527.4842; 1532.0144; 1548.8115; 1611.5860; 1618.8646; 1638.3884; 1727.6059; 1875.8879; 3041.0662; 3058.9550; 3062.8811; 3071.1296; 3099.4925; 3122.9499; 3128.0665; 3134.0827; 3138.7008; 3139.9855; 3141.0183; 3153.3894; 3161.5619; 3164.5313; 3169.4104; 3175.2608; 3183.9910.<br><br>Cartesian Forces: Max 0.000000672 RMS 0.000000255<br><br>Item Value Threshold Converged?<br>Maximum Force 0.000000 0.000450 YES<br>RMS Force 0.000000 0.000300 YES<br>Maximum Displacement 0.000046 0.001800 YES<br>RMS Displacement 0.000005 0.001200 YES	Geometry at RB3LYP/6-31G(d)/MWB59<br>Charge = -1 Multiplicity = 1<br>Yb 0.8387607194 2.1645474307 0.9917653947<br>O 2.8461437612 1.7877343675 -0.2977018094<br>S 3.8215239981 1.5241346705 0.8318776813<br>O 4.6118125329 0.2965128407 0.7340843256<br>O 3.0777578628 1.7840064776 2.1018894165<br>C 5.0828164808 2.8892838835 0.6823248672<br>F 5.7308068255 2.7639845666 -0.4841025903<br>F 4.5148736604 4.0893073501 0.7383657119<br>F 5.9690799532 2.7755697883 1.6814409046<br>O 0.5640065154 3.9610548195 -0.6863219597<br>O 2.2887888021 5.8225787239 -0.5740941157<br>O 1.6074961509 4.4197766322 1.4244910429<br>S 1.2546352587 5.0417376614 0.0956676132<br>C -0.0794256597 6.2868588517 0.4873277129<br>F -0.4331025992 6.9222566334 -0.6378894727<br>F -1.1604490443 5.7031603671 1.0072672961<br>F 0.3910326873 7.1864354129 1.3608766032<br>C 0.057281639 -1.954053172 -0.17830249<br>C -1.2835695074 -2.170055697 0.0515676852<br>C -1.4129813677 -0.9885051122 0.9232651099<br>C 0.0824475908 -0.6866726157 0.6679661964<br>O 0.9753811406 -0.1203253491 1.3351832619<br>O -2.2714661414 -0.5141068445 1.6297367532<br>O 0.8651388554 -2.7198865959 -0.885261533<br>C 2.3488885892 -2.7153067538 -0.7886225385<br>C -2.2387304585 -3.2272637274 -0.3631502737<br>H -1.7172401291 -4.1107827642 -0.7455106225<br>H -2.9013283856 -2.851774462 -1.1550427857<br>H -2.8789442461 -3.520084313 0.4771250876<br>C 2.895974273 -1.4993774057 -1.5276249092<br>H 3.9896610401 -1.5423470044 -1.5490312563<br>H 2.5219417306 -1.4799497244 -2.5566917396<br>H 2.6251143538 -0.5689436178 -1.0301601378<br>C 2.7136811859 -4.0126188581 -1.5151080519<br>H 3.8021250462 -4.1288482003 -1.540727094<br>H 2.2824210012 -4.8798705208 -1.0037699163<br>H 2.3418330076 -3.9959952782 -2.5449322789<br>C 2.7791088116 -2.7644044579 0.6776352484<br>H 2.5149215134 -1.8474276008 1.2078233088<br>H 2.3241305528 -3.6244516623 1.1830156454<br>H 3.8677292888 -2.8683348262 0.7298723691<br>C -0.5030447798 1.1319175962 -0.8991631162<br>C 0.1648248226 0.9396885609 -2.1283629908<br>C -1.8950881998 1.3634638018 -0.9797142089<br>H 1.2413752866 0.7858936686 -2.1384651776<br>H -2.4647230888 1.5193769152 -0.0670922835<br>C -0.5009467488 0.9794426698 -3.3582106389<br>C -2.5726079866 1.4086823651 -2.2013732211<br>H 0.0543783244 0.8363372635 -4.2842184744<br>H -3.6439512325 1.6036922106 -2.2218162559<br>C -1.8772622643 1.2089502032 -3.3973976156<br>H -2.4026843066 1.2391806259 -4.3503574849<br>O -2.0928391725 1.8626409227 4.0388450145<br>S -1.0731888219 2.5969850981 3.303627243<br>C -1.0874485113 4.287937913 4.0972979278<br>O -1.3113073425 2.8568491288 1.8329137363<br>O 0.351853298 2.1237157119 3.3951116443<br>F -0.1171273422 5.072545684 3.6288814329<br>F -0.9256346832 4.148753345 5.4201629094<br>F -2.2695973124 4.8740694258 3.8667538105<br><br>Frequency: -159.0864; 15.0541; 21.4678; 26.4434; 27.3261; 30.2554; 32.7187; 33.8706; 38.9255; 40.3176; 44.7232; 48.3447; 55.6338; 60.0272; 64.3137; 67.7113; 76.3204; 78.1181; 79.4741; 83.4189; 86.3863; 90.1120; 92.6674; 100.1273; 111.1374; 124.1864; 131.2732; 136.1153; 144.0428; 147.7312; 149.6355; 157.3707; 166.3072; 181.2033; 185.5528; 196.1888; 208.5563; 211.1434; 213.0674; 215.5342; 215.9929; 222.5317; 224.4759; 254.3141; 267.8259; 281.2810; 287.3638; 301.5951; 304.0138; 306.4658; 309.2179; 320.3529; 324.5729; 325.1375; 337.6061; 348.6453; 351.0537; 352.1858; 358.0919; 395.8316; 401.5808; 422.9729; 453.4529; 482.7238; 489.4467; 490.2342; 491.5009; 492.2310; 523.4366; 525.5682; 531.0331; 557.9594; 558.2176; 558.9425; 569.6433; 572.1894; 575.8659; 588.6809; 607.6943; 620.5568; 626.3743; 629.2803; 630.3449; 635.0575; 637.9711; 714.8515; 723.0047; 735.3527; 758.4794; 760.5522; 761.2534; 763.0394; 789.7567; 859.3203; 866.4595; 924.5811; 937.0759; 940.2680; 958.5005; 971.1297; 973.4867; 979.3125; 980.0349; 984.9987; 988.6112; 990.9322; 1006.3671; 1014.4804; 1054.4131; 1059.4304; 1067.1423; 1072.8231; 1075.9490; 1091.6183; 1103.3578; 1109.6589; 1130.7311; 1185.5833; 1204.1812; 1209.5451; 1211.1546; 1212.6567; 1223.4911; 1227.1663; 1229.3088; 1231.2204; 1246.5962; 1249.3836; 1253.1298; 1260.3920; 1290.1861; 1292.5898; 1298.5114; 1301.3197; 1306.2146; 1318.7477; 1368.6484; 1383.4745; 1429.7772; 1438.9283; 1440.7989; 1461.3503; 1462.6322; 1499.6574; 1504.8640; 1510.2563; 1514.9560; 1517.4975; 1518.8623; 1526.8940; 1530.1029; 1551.0394; 1613.7931; 1615.0959; 1621.0880; 1681.1102; 1872.4478; 3038.6353; 3055.0115; 3059.6252; 3070.7337; 3094.5383; 3124.5615; 3127.3494; 3129.0260; 3130.6620; 3138.0633; 3148.2650; 3158.9579; 3169.6417; 3177.5314; 3188.1937; 3196.0552; 3196.8220.<br><br>Cartesian Forces: Max 0.000001610 RMS 0.000000597<br><br>Item Value Threshold Converged?<br>Maximum Force 0.000000 0.000450 YES<br>RMS Force 0.000000 0.000300 YES<br>Maximum Displacement 0.000046 0.001800 YES<br>RMS Displacement 0.000009 0.001200 YES
	FILE: Yb_Sq2_PhYbOTf3_TS_Anion_B3LYP_M062x_Add12_Conf1.out	FILE: Yb_Sq2_PhYbOTf3_TS_Anion_B3LYP_M062x_Add12_Conf1-1.out	FILE: Yb_Sq2_PhYbOTf3_TS_Anion_B3LYP_M062x_Add12_Conf2.out

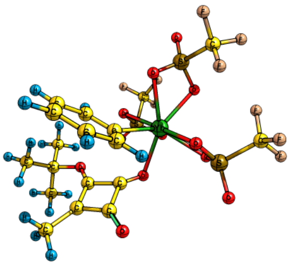

Conformer 4

E(RB3LYP/6-31G(d)/MWB59) = -3731.44882881
Convg = 0.9644D-08 -V/T = 2.0122

Temperature 195.150 Kelvin. Pressure 1.00000 Atm.
elec. and zero-point Energies= -3731.077597
elec. and thermal Energies= -3731.054431
elec. and thermal Enthalpies= -3731.053813
elec. and thermal Free Energies= -3731.122800

RB3LYP/6-31G(d)/MWB59/THF(IEFPCM) = -3731.51615430

RM06-2x/6-311+G(d,p)/MWB59/THF(IEFPCM) = -3731.35445414

Geometry at RB3LYP/6-31G(d)/MWB59
Charge = -1 Multiplicity = 1
Yb 0.6448952217 1.9613865694 0.9816864878
O 2.3319503024 0.8878051498 -0.3990070846
S 3.3370734344 0.5459502267 0.6760644589
O 3.8358948874 -0.8288472295 0.7035801358
O 2.8099507027 1.1315900448 1.9479602706
C 4.8305008114 1.5708498937 0.2398572998
F 5.3305234588 1.1529377514 -0.9320685836
F 4.5151163533 2.8607968116 0.1406754592
F 5.7677173908 1.420115307 1.1848803523
O 0.7551190629 3.6139947047 -0.8581121455
O 2.7255003839 5.2196033602 -0.8233769073
O 1.8058067649 4.0789429939 1.2464079462
S 1.577032828 4.6422761086 -0.1337393046
C 0.4123305962 6.0773650652 0.1204987179
F 0.1982180338 6.6875344008 -1.0528342175
F -0.759939467 5.667426008 0.6100937739
F 0.9558927532 6.957203931 0.9707305001
C -0.4317464543 -1.8374061517 0.0363264508
C -1.730001221 -2.3189641344 0.1035474465
C -2.1488832018 -1.2569364475 1.0527258232
C -0.6899575449 -0.7447312691 1.0303567873
O 0.0491124691 -0.1170405507 1.8003411462
O -3.1745794217 -0.9969230291 1.6347962391
O 0.7397720155 -2.0569255859 -0.500535428
C 1.0817718788 -3.1390765383 -1.442013696
C -2.5878544676 -3.4070730701 -0.4384706137
H -2.0909570737 -4.3825264242 -0.4675200512
H -2.9226871999 -3.170244203 -1.4572633321
H -3.4833656566 -3.4885866773 0.1874414604
C 1.0860233437 -4.461417284 -0.6703697444
H 1.3935546192 -5.2783252919 -1.3330059034
H 1.7897636839 -4.4098840076 0.1661619209
H 0.0945998459 -4.7029978989 -0.2745331028
C 2.4953797785 -2.7528954724 -1.8829929154
H 2.9140192581 -3.5396077978 -2.5207309127
H 2.4756722887 -1.814618834 -2.4451446076
H 3.1400736224 -2.6041391029 -1.0131422964
C 0.1197784192 -3.1407333516 -2.6330424025
H -0.8646686699 -3.5386912604 -2.3775272283
H -0.0066775269 -2.1254652003 -3.0203305877
H 0.5355323139 -3.7686945584 -3.4290879616
C -0.9588702633 1.1278175448 -0.7498016157
C -0.4616110438 0.7469364779 -2.0187482573
C -2.3407716342 1.4178896731 -0.6955833446
H 0.5989979707 0.5266614766 -2.1292313283
H -2.7853069635 1.721322436 0.2500604654
C -1.2758820665 0.6687102811 -3.152853165
C -3.1712568099 1.3361351836 -1.8202327359
H -0.8471534868 0.3966425452 -4.1175275247
H -4.2314078425 1.5699351028 -1.7322488286
C -2.6413014964 0.9564018774 -3.0545090719
H -3.2812784592 0.8937443616 -3.9332127824
O -1.9483986743 2.2612176559 4.2790751801
S -0.9561420125 2.8633356895 3.3966664287
C -0.8599027874 4.6375649001 3.9672512353
O -1.3134680219 2.9570330097 1.9270238015
O 0.4493272836 2.3482823262 3.4451786424
F 0.0758131573 5.3233481319 3.3092293054
F -0.5687771125 4.6609675023 5.2750647516
F -2.045615496 5.2319830048 3.7791407301

Frequency: -102.5943; 15.8518; 19.6221; 25.1471; 28.2338; 30.4496; 33.4234; 37.0546; 39.1923; 42.0583; 44.8953; 46.0142; 54.9810; 57.1854; 64.4714; 69.1999; 70.8978; 75.0872; 75.9231; 81.0751; 85.7719; 87.3168; 96.0304; 117.8274; 123.9039; 129.6545; 135.2237; 143.9550; 146.9854; 148.8118; 153.0317; 156.5089; 167.8474; 180.3294; 190.3755; 202.8218; 206.4544; 211.2984; 212.1180; 215.4355; 217.5263; 221.6802; 224.5690; 234.6965; 250.8392; 262.2321; 291.2883; 301.9276; 302.4509; 307.0560; 308.3381; 321.1153; 323.0595; 324.5839; 334.5759; 350.3426; 351.3295; 351.8575; 357.8987; 395.8619; 398.7168; 421.9219; 454.6109; 483.1855; 484.9206; 488.9428; 490.3618; 491.4865; 523.2155; 525.2494; 530.1530; 557.6781; 558.1791; 558.5918; 569.9197; 572.7426; 575.7279; 587.8081; 608.6792; 620.5904; 626.1888; 630.9398; 633.2356; 636.5970; 647.8284; 716.3209; 730.9262; 760.4577; 760.9011; 761.3818; 762.1269; 765.8355; 783.3752; 849.3800; 870.0250; 921.1120; 935.6872; 939.4881; 971.8027; 973.1139; 977.5206; 982.0329; 984.4418; 984.7540; 988.0338; 995.3847; 1011.6703; 1013.9173; 1052.3647; 1062.1276; 1069.5463; 1075.1985; 1086.7679; 1100.7907; 1110.9704; 1112.7547; 1136.4063; 1184.0093; 1197.0832; 1212.1325; 1213.4565; 1213.9683; 1215.1375; 1223.4902; 1227.3581; 1229.4152; 1232.2694; 1246.1472; 1250.1223; 1253.9043; 1281.9492; 1292.0454; 1296.5159; 1298.5729; 1305.1253; 1315.1385; 1368.8256; 1386.2089; 1429.5544; 1436.8678; 1437.8623; 1459.4297; 1461.0604; 1500.5823; 1507.7796; 1513.9780; 1515.9405; 1517.5304; 1521.0324; 1527.4641; 1531.8054; 1548.5421; 1611.6474; 1618.8920; 1637.8968; 1724.7374; 1879.1456; 3040.1608; 3059.1259; 3062.7688; 3070.9679; 3098.6746; 3122.8011; 3128.3816; 3133.7823; 3138.5972; 3139.8112; 3141.4750; 3153.7655; 3161.5067; 3164.5162; 3169.5175; 3175.9391; 3185.9266.

Cartesian Forces: Max 0.000002631 RMS 0.000000964

Item	Value	Threshold	Converged?
Maximum Force	0.000002	0.000450	YES
RMS Force	0.000000	0.000300	YES
Maximum Displacement	0.000215	0.001800	YES
RMS Displacement	0.000044	0.001200	YES

FILE: Yb_Sq2_PhYbOTf3_TS_Anion_B3LYP_M062x_Add12_Conf2-1.out

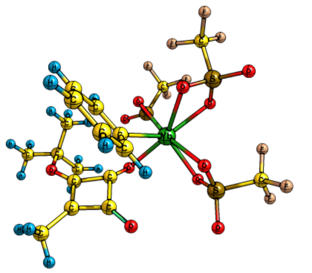

Conformer 5

E(RB3LYP/6-31G(d)/MWB59) = -3731.44882160
Convg = 0.6804D-08 -V/T = 2.0122

Temperature 195.150 Kelvin. Pressure 1.00000 Atm.
elec. and zero-point Energies= -3731.077676
elec. and thermal Energies= -3731.054514
elec. and thermal Enthalpies= -3731.053896
elec. and thermal Free Energies= -3731.123104

RB3LYP/6-31G(d)/MWB59/THF(IEFPCM) = -3731.51378916

RM06-2x/6-311+G(d,p)/MWB59/THF(IEFPCM) = -3731.35342256

Geometry at RB3LYP/6-31G(d)/MWB59
Charge = -1 Multiplicity = 1
Yb 0.536707854 2.1597617791 1.0535687971
O 2.5706983178 1.9518788809 -0.2257440313
S 3.5621898012 1.7403191445 0.9006378489
O 4.4322706051 0.5700312603 0.780012276
O 2.8105006473 1.9369930658 2.1753721001
C 4.7433867155 3.1811316104 0.7730465567
F 5.2764412902 3.212002867 -0.4558426288
F 4.1418606834 4.3443021294 1.0151942085
F 5.7285748868 3.0125729701 1.6658327804
O 1.2223441321 4.4494276951 1.400004319
O -0.3155634624 6.2554743053 0.5067440903
O -0.0965943057 3.949147787 -0.5414923543
S 0.5092666139 5.0848751611 0.2305784398
C 1.8724474819 5.7315767855 -0.8683084248
F 2.6468663772 6.579105328 -0.1790361406
F 2.6316395003 4.7406320995 -1.3434777173
F 1.3302636812 6.3875128155 -1.9031697142
C 0.0788565981 -2.0166902886 -0.1111962682
C -1.237788359 -2.3261661765 0.1489880304
C -1.4331376024 -1.1466922199 1.0116286243
C 0.0309558019 -0.7425173712 0.7238279258
O 0.8947993719 -0.111189693 1.3700989888
O -2.3092275388 -0.7224498094 1.7287568894
O 0.9275057792 -2.7267800951 -0.8288904243
C 2.4076862054 -2.6106319968 -0.7541100184
C -2.1240792736 -3.4521680611 -0.236917379
H -1.5496219655 -4.2991044148 -0.6260857998
H -2.8299743506 -3.1309955659 -1.0151775866
H -2.7222111607 -3.7838105961 0.6197840733
C 2.8521695673 -1.3471586503 -1.4822991465
H 3.9456181349 -1.3113111998 -1.5232149477
H 2.4589287692 -1.3389767612 -2.5042743205
H 2.5246022664 -0.4468267884 -0.9639482676
C 2.8583875845 -3.8673279696 -1.5032129825
H 3.9520099342 -3.9019537527 -1.5439239898
H 2.4995096142 -4.7708834022 -0.9988854323
H 2.4724712876 -3.8649815424 -2.5279943403
C 2.8609893833 -2.6449491635 0.7057943481
H 2.5386658094 -1.7546999854 1.2494427439
H 2.476179764 -3.5410314415 1.2067489286
H 3.954940204 -2.6706971894 0.7428240295
C -0.7127637367 1.0121587828 -0.8337331345
C -0.0263619654 0.888470762 -2.0613293377
C -2.118871165 1.1271697716 -0.9178994808
H 1.059389675 0.8242337333 -2.0681596753
H -2.7029398377 1.2294889154 -0.0066991383
C -0.6888705718 0.8844065854 -3.2939684775
C -2.7932587642 1.1263195632 -2.1416056637
H -0.1204082633 0.7967182467 -4.2189845007
H -3.8768519984 1.2323577218 -2.1649433543
C -2.0792904162 0.9972878482 -3.3365875401
H -2.6022737758 0.9924986223 -4.2913131906
O -2.4032335939 1.730137664 4.0788964168
S -1.3956371582 2.4962468244 3.3597543867
C -1.4062559859 4.1618366239 4.2010736603
O -1.639599607 2.7976951124 1.8987086978
O 0.036004236 2.0294989439 3.4288219207
F -0.4870008245 4.9785191508 3.6924310693
F -1.1600341067 3.991071612 5.5078485302
F -2.615165203 4.7194009114 4.0596795449

Frequency: -161.1884; 9.1635; 20.9624; 23.4531; 28.7272; 31.0784; 34.3562; 36.0749; 39.5731; 41.0859; 44.0511; 50.4151; 54.3302; 62.4825; 66.2497; 73.1688; 73.7992; 78.7988; 80.6925; 81.8347; 85.5193; 91.9815; 96.7219; 102.3495; 109.7971; 122.4734; 130.8594; 134.7187; 143.3962; 148.7993; 149.8509; 157.1710; 167.3239; 181.6142; 185.3739; 196.1306; 209.1333; 211.7850; 213.9900; 214.8942; 216.6008; 222.1804; 228.3051; 254.7196; 267.4926; 280.6111; 286.8769; 301.8303; 304.8365; 306.1780; 309.3600; 319.8413; 323.3903; 327.9475; 337.9961; 348.2476; 350.8887; 352.6893; 358.5797; 395.9723; 402.1280; 422.5450; 453.3064; 481.9034; 489.1227; 490.2324; 491.4729; 491.8659; 522.9160; 525.7206; 531.1446; 557.8715; 558.0820; 559.2026; 568.8283; 572.7645; 575.6736; 589.7599; 608.1957; 620.8042; 625.7852; 629.4958; 630.5700; 635.4801; 638.7061; 715.1549; 722.8571; 735.3344; 758.5380; 760.0486; 761.5873; 762.1263; 790.0079; 859.9785; 866.7493; 925.0367; 937.5869; 940.1641; 958.7352; 970.5025; 973.0496; 979.6248; 980.0979; 985.2597; 988.9188; 991.7627; 1006.2571; 1014.5640; 1054.7550; 1059.7574; 1067.1468; 1072.7856; 1076.3377; 1091.7474; 1099.2146; 1113.2328; 1130.4706; 1185.7594; 1204.5752; 1209.1758; 1211.1802; 1213.9061; 1223.6374; 1225.3566; 1228.3390; 1232.5495; 1245.3171; 1245.7402; 1254.4440; 1259.0567; 1289.6354; 1292.0749; 1298.7225; 1301.4331; 1308.5812; 1317.9014; 1368.8964; 1383.4922; 1429.7190; 1438.9454; 1440.4411; 1461.4603; 1462.5018; 1499.6124; 1505.0196; 1510.3779; 1514.8428; 1517.8242; 1518.5656; 1526.9070; 1530.0622; 1550.7490; 1614.1431; 1616.3780; 1621.7547; 1681.4519; 1871.6191; 3038.5967; 3055.0285; 3059.6057; 3070.6454; 3094.5193; 3124.3386; 3127.2807; 3128.9166; 3130.5379; 3137.8236; 3148.3144; 3159.4745; 3168.6343; 3177.6923; 3187.0380; 3195.5998; 3196.1504.

Cartesian Forces: Max 0.000002126 RMS 0.000000642

Item	Value	Threshold	Converged?
Maximum Force	0.000001	0.000450	YES
RMS Force	0.000000	0.000300	YES
Maximum Displacement	0.000279	0.001800	YES
RMS Displacement	0.000046	0.001200	YES

FILE: Yb_Sq2_PhYbOTf3_TS_Anion_B3LYP_M062x_Add12_Conf3.out

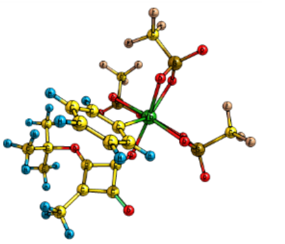

Conformer 6

E(RB3LYP/6-31G(d)/MWB59) = -3731.44874393
Convg = 0.7164D-08 -V/T = 2.0122

Temperature 195.150 Kelvin. Pressure 1.00000 Atm.
elec. and zero-point Energies= -3731.077495
elec. and thermal Energies= -3731.054326
elec. and thermal Enthalpies= -3731.053708
elec. and thermal Free Energies= -3731.122844

RB3LYP/6-31G(d)/MWB59/THF(IEFPCM) = -3731.51644465

RM06-2x/6-311+G(d,p)/MWB59/THF(IEFPCM) = -3731.35467940

Geometry at RB3LYP/6-31G(d)/MWB59
Charge = -1 Multiplicity = 1
Yb 0.3720089359 1.9956913584 1.0875533304
O 2.1425451356 1.141530562 -0.319460193
S 3.1637947559 0.780586339 0.736971206
O 3.7179263208 -0.5719063665 0.6731493763
O 2.6174356636 1.2614550618 2.0402199839
C 4.630610061 1.8710881185 0.361689205
F 5.0787713312 1.6026510155 -0.8735584806
F 4.3235158969 3.165401549 0.436695954
F 5.6083039304 1.6084027937 1.2394717227
O 1.6226091969 4.0799232266 1.2042826213
O 0.4991137665 6.2042545251 0.392672841
O -0.11289838 3.8799468928 -0.4371516999
S 0.9110608254 4.8342440623 0.1098772736
C 2.1721698123 5.0008994973 -1.2547175943
F 3.2525238705 5.6463042358 -0.7975764256
F 2.5472993781 3.8050062561 -1.7180517827
F 1.6446237143 5.7049310858 -2.2648166646
C -0.413613122 -1.8880519036 0.1185133617
C -1.6784299849 -2.4506832995 0.195473059
C -2.1573215629 -1.4156692875 1.1461276227
C -0.7329676159 -0.8147372428 1.1157309774
O -0.0308553726 -0.1397412026 1.8794537026
O -3.1944172117 -1.2187341059 1.7325706288
O 0.7644198668 -2.0334530976 -0.429448057
C 1.1635361403 -3.0943924998 -1.3729980295
C -2.4706129478 -3.5912339111 -0.3385761743
H -1.9132733786 -4.5332448147 -0.3726238824
H -2.8296308559 -3.3763822441 -1.354000052
H -3.3531544442 -3.7289341566 0.2958748417
C 1.2633808596 -4.4105587369 -0.5972771065
H 1.6128573672 -5.2090197221 -1.2616012292
H 1.9740628776 -4.3107754227 0.2289690116
H 0.2950605048 -4.7130950217 -0.1863894721
C 2.5432392858 -2.6197534032 -1.8344931333
H 3.0061922789 -3.3840902899 -2.4688333855
H 2.4544296231 -1.6918972048 -2.40736363
H 3.1857231298 -2.417033165 -0.9742393342
C 0.1889320153 -3.1631158053 -2.5518045107
H -0.7624035368 -3.6278762743 -2.2843968191
H -0.0119105954 -2.1592109711 -2.9374333634
H 0.6372063276 -3.761693081 -3.3528606275
C -1.1082335778 1.0349188343 -0.6859691403
C -0.5595015632 0.6961967619 -1.9453830804
C -2.509529217 1.2174475727 -0.6581721542
H 0.5161409114 0.5525745582 -2.0352203515
H -2.9934282843 1.4852192259 0.2788794861
C -1.3440356288 0.5601697263 -3.0959162941
C -3.3096206071 1.073430076 -1.7977884936
H -0.8772711936 0.324431899 -4.0526023954
H -4.3861371063 1.2243557833 -1.7296944741
C -2.7288533089 0.7404665315 -3.023100657
H -3.3455397656 0.6315037543 -3.9138458683
O -2.0840497421 2.0427876912 4.5245059087
S -1.2528998181 2.7221833274 3.5385323742
C -1.3640015884 4.5285063754 3.9816977462
O -1.7025788926 2.6760387416 2.0949478773
O 0.2168535484 2.4171437624 3.5158829771
F -0.6680688807 5.2750504099 3.1233210534
F -0.8787084018 4.7166199481 5.2159719855
F -2.645013747 4.9184016711 3.9581664278

Frequency: -98.0523; 14.0553; 17.1576; 23.1741; 29.6070; 31.1186; 32.7174; 37.7788; 38.9961; 41.2474; 44.7145; 48.3737; 55.7843; 57.4412; 65.7669; 66.8704; 70.2487; 74.2131; 74.9676; 81.0972; 87.3321; 89.2351; 95.6440; 114.9939; 124.1709; 129.5457; 135.8702; 142.9018; 146.1718; 148.5918; 154.8320; 157.5098; 167.6590; 180.4416; 190.6705; 204.6082; 206.5735; 210.3162; 212.2704; 214.1428; 219.7992; 221.7489; 223.2574; 235.8115; 252.3166; 263.6831; 291.3318; 301.2471; 302.3951; 307.0289; 308.0781; 320.3054; 322.3101; 325.0333; 334.3877; 350.0203; 350.6974; 352.9774; 358.0742; 395.7752; 399.4438; 423.1556; 454.7886; 483.1770; 484.7785; 488.7404; 490.1368; 491.3060; 522.9174; 525.6065; 530.1982; 557.2725; 557.8062; 558.7479; 569.3396; 573.5623; 575.6550; 587.6089; 608.9448; 620.0714; 626.9878; 631.0793; 633.0825; 636.0646; 647.9631; 717.6410; 731.5794; 760.0705; 761.0202; 761.3807; 762.0121; 765.8808; 783.2246; 849.2056; 871.1311; 921.9587; 935.5552; 939.7640; 972.7774; 974.6218; 975.4490; 982.6901; 984.5432; 985.0563; 989.4329; 995.2394; 1011.4554; 1014.1234; 1052.2384; 1061.7200; 1069.5292; 1075.5662; 1086.4249; 1100.3216; 1105.8566; 1118.9578; 1136.0838; 1183.9080; 1197.0705; 1212.1345; 1214.2995; 1214.5098; 1215.4230; 1223.6414; 1225.8935; 1228.7936; 1234.7887; 1242.6287; 1246.9948; 1254.7398; 1281.8762; 1291.3158; 1296.2346; 1298.2341; 1308.3806; 1315.6187; 1368.9002; 1386.1604; 1429.7389; 1436.6380; 1437.7865; 1459.3456; 1460.9877; 1500.8067; 1507.4006; 1513.8941; 1515.7673; 1517.5898; 1520.9572; 1527.8481; 1532.0744; 1548.8906; 1611.5113; 1618.6833; 1638.1640; 1726.5460; 1879.4432; 3040.2121; 3059.0160; 3062.6725; 3070.8150; 3098.7912; 3122.8925; 3128.2492; 3133.2011; 3137.3528; 3139.1866; 3141.2557; 3153.7616; 3163.4227; 3165.3624; 3169.2494; 3175.7634; 3186.4366.

Cartesian Forces: Max 0.000001211 RMS 0.000000453

Item	Value	Threshold	Converged?
Maximum Force	0.000001	0.000450	YES
RMS Force	0.000000	0.000300	YES
Maximum Displacement	0.000042	0.001800	YES
RMS Displacement	0.000010	0.001200	YES

FILE: Yb_Sq2_PhYbOTf3_TS_Anion_B3LYP_M062x_Add12_Conf3-1_l.out

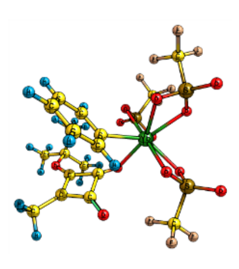

Conformer 7

E(RB3LYP/6-31G(d)/MWB59) = -3731.44847564
Convg = 0.8792D-08 -V/T = 2.0122

Temperature 195.150 Kelvin. Pressure 1.00000 Atm.
elec. and zero-point Energies= -3731.077272
elec. and thermal Energies= -3731.054178
elec. and thermal Enthalpies= -3731.053560
elec. and thermal Free Energies= -3731.121925

RB3LYP/6-31G(d)/MWB59/THF(IEFPCM) = -3731.51213370

RM06-2x/6-311+G(d,p)/MWB59/THF(IEFPCM) = -3731.35053651

Geometry at RB3LYP/6-31G(d)/MWB59
Charge = -1 Multiplicity = 1
Yb 0.6539596725 2.1445657189 1.0845255144
O 2.6005630931 1.9343736269 -0.3410618629
S 3.6633813918 1.6941328764 0.7116906367
O 4.5400000494 0.5429505336 0.4932077806
O 2.9833167686 1.8209718137 2.0344797883
C 4.8143405399 3.1579138613 0.5766535757
F 5.2890681777 3.2345636711 -0.6737819008
F 4.20374741 4.3007750332 0.8814887425
F 5.8413951141 2.9787912691 1.4177724517
O 1.3205525674 4.4367039518 1.5228435402
O -0.278690804 6.2766894844 0.8224998559
O -0.156124262 4.005096934 -0.3143990031
S 0.5168317926 5.1163048904 0.4421592157
C 1.7845107718 5.7907520794 -0.7500120587
F 2.6219483376 6.6129981984 -0.1056842926
F 2.4928253423 4.8078279761 -1.3144927151
F 1.1629433503 6.4785088505 -1.7166539297
C 0.0560631142 -1.9861769853 -0.1570276669
C -1.2821603707 -2.2754053223 -0.0033297028
C -1.5190353522 -1.1409592646 0.902926326
C -0.0251741295 -0.7507862958 0.7331846069
O 0.8090579817 -0.1422964689 1.435346402
O -2.4447998155 -0.7554405151 1.5779415892
O 0.9251236244 -2.6983336367 -0.8454859185
C 2.4051470494 -2.5905792057 -0.7603041373
C -2.1612068398 -3.3594987412 -0.5058909456
H -1.5786136197 -4.2101710842 -0.8740463828
H -2.7847310437 -2.9895426043 -1.331389236
H -2.842783619 -3.6996880123 0.2822735344
C 2.8537819694 -1.3738054417 -1.561453264
H 3.9468068284 -1.3328694153 -1.593215639
H 2.4688613827 -1.4319362572 -2.5851289237
H 2.5147553219 -0.4444006743 -1.1057503991
C 2.8531958186 -3.8896429463 -1.4358550468
H 3.9468434959 -3.9302137044 -1.4694659869
H 2.4890714806 -4.7621193734 -0.8829199612
H 2.4719634766 -3.9435999422 -2.460919575
C 2.8524554021 -2.5489654012 0.7004185264
H 2.5195460307 -1.6394576222 1.2032960339
H 2.4738243673 -3.4236763094 1.2424205052
H 3.9462405829 -2.5627682329 0.741315468
C -0.6503200217 1.1168357059 -0.8326890632
C -0.0109041588 0.95873627 -2.0818918448
C -2.0529716658 1.2882745939 -0.8755976763
H 1.0707026002 0.8533757724 -2.1242729628
H -2.6039301437 1.4187179691 0.0525690758
C -0.7123615665 0.9718072947 -3.2928766259
C -2.7668776877 1.3057349307 -2.0769096104
H -0.1773396303 0.855827148 -4.2347713936
H -3.8457621393 1.4541087987 -2.065964734
C -2.0981402974 1.1392841598 -3.2930395663
H -2.6512264249 1.148276912 -4.2307193685
O -1.5010547471 3.5782058496 4.4002664265
S -0.981165964 2.4737421316 3.6041267974
C -1.6432827655 0.941073942 4.4433423259
O 0.5042314073 2.2635435682 3.5502182281
O -1.4523060856 2.3862473572 2.167556819
F -1.0249592479 -0.1584287499 4.008454977
F -2.955538546 0.8309134516 4.2299850335
F -1.4276443649 1.0510885824 5.7637196172

Frequency: -143.2374; 18.2536; 21.6765; 27.5028; 28.7241; 34.2816; 35.3710; 39.1250; 41.3766; 42.1622; 49.3701; 52.6464; 57.5271; 65.0745; 66.7923; 72.5915; 73.2458; 74.2599; 79.3424; 82.7890; 85.1442; 93.5220; 96.4925; 104.3309; 114.5756; 119.1519; 129.8645; 137.0285; 141.9882; 146.7021; 151.5018; 156.9467; 163.6643; 181.5916; 188.7291; 196.7785; 208.4051; 210.4940; 212.2562; 215.6024; 218.1614; 223.0730; 224.1851; 252.9771; 268.2578; 281.1336; 289.7542; 302.1842; 303.6809; 306.7285; 308.6766; 320.5104; 322.5876; 325.7297; 337.7689; 349.5428; 350.7195; 352.8052; 358.4883; 395.2327; 402.3579; 422.3437; 454.4525; 483.3452; 489.5455; 490.9256; 492.3704; 492.7238; 524.1602; 525.2941; 531.4197; 557.3001; 558.0368; 559.2329; 570.4209; 570.8592; 578.5436; 587.8462; 603.9031; 619.4056; 623.4363; 627.5525; 630.2250; 633.6133; 639.7640; 715.4278; 723.0274; 735.4327; 755.9909; 759.8544; 761.6112; 762.3284; 789.4648; 858.8723; 866.8017; 923.3886; 936.6723; 941.2580; 959.0915; 972.5554; 973.0345; 973.5811; 978.4475; 984.2728; 988.8862; 992.2309; 1006.8571; 1014.3260; 1053.3035; 1058.5524; 1067.6633; 1072.9888; 1075.8943; 1090.7588; 1101.7774; 1114.5972; 1133.0714; 1185.1924; 1203.9374; 1206.9675; 1211.1931; 1214.0296; 1223.3994; 1226.6000; 1230.0533; 1231.6917; 1243.8071; 1251.7484; 1253.4861; 1257.4409; 1289.2663; 1293.3415; 1298.1582; 1299.9300; 1305.8220; 1318.8057; 1368.2800; 1384.4545; 1429.9597; 1438.8986; 1442.0936; 1460.8569; 1463.4018; 1498.7814; 1504.6975; 1509.9180; 1514.6406; 1517.3271; 1519.4379; 1525.2534; 1529.8402; 1551.5782; 1613.5912; 1614.9925; 1620.9102; 1682.6825; 1871.4132; 3039.3831; 3055.4012; 3060.2476; 3071.8371; 3095.5187; 3125.6996; 3128.5345; 3129.8820; 3132.3411; 3140.3577; 3146.4234; 3157.9546; 3173.2064; 3176.6017; 3186.7062; 3194.7188; 3196.6367.

Cartesian Forces: Max 0.000001034 RMS 0.000000371

Item	Value	Threshold	Converged?
Maximum Force	0.000000	0.000450	YES
RMS Force	0.000000	0.000300	YES
Maximum Displacement	0.000076	0.001800	YES
RMS Displacement	0.000012	0.001200	YES

FILE: Yb_Sq2_PhYbOTf3_TS_Anion_B3LYP_M062x_Add12_Conf4_x.out

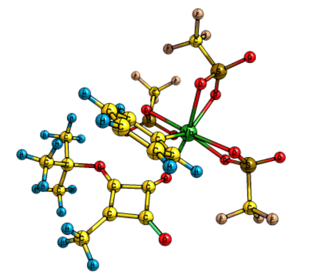

Conformer 8

E(RB3LYP/6-31G(d)/MWB59) = -3731.44835992
Convg = 0.9755D-08 -V/T = 2.0122

Temperature 195.150 Kelvin. Pressure 1.00000 Atm.
elec. and zero-point Energies= -3731.077147
elec. and thermal Energies= -3731.054003
elec. and thermal Enthalpies= -3731.053385
elec. and thermal Free Energies= -3731.122411

RB3LYP/6-31G(d)/MWB59/THF(IEFPCM) = -3731.51600901

RM06-2x/6-311+G(d,p)/MWB59/THF(IEFPCM) = -3731.35394913

Geometry at RB3LYP/6-31G(d)/MWB59
Charge = -1 Multiplicity = 1
Yb 0.4885643373 1.928619637 1.0947702969
O 2.2284821544 1.1655931441 -0.3955732267
S 3.2696635451 0.73601826 0.614840212
O 3.827191306 -0.6059443278 0.4430382585
O 2.7447844263 1.1195667483 1.9586643944
C 4.7251967062 1.8620136739 0.3024154481
F 5.1008585868 1.7622405856 -0.9805108154
F 4.4348802135 3.1338664384 0.5721967951
F 5.7475868879 1.4802067697 1.0801846938
O 1.6660013074 4.0332538509 1.294688822
O 0.4991878171 6.1176481333 0.44400177
O 0.0809854444 3.7886989512 -0.488745315
S 1.0034088591 4.7794160353 0.1619364769
C 2.359185598 5.0517327682 -1.0921862341
F 3.3829311461 5.6999767354 -0.522942826
F 2.8042799455 3.8942175873 -1.5910019298
F 1.885666187 5.7953549953 -2.1012563902
C -0.3206334803 -1.9175490275 -0.0090904291
C -1.5689974662 -2.5116656924 0.1017759628
C -2.0175756299 -1.5410743802 1.1297875889
C -0.6090219759 -0.9047935358 1.0588476444
O 0.1161503554 -0.2477305031 1.8146838138
O -3.0271694579 -1.4003137982 1.7789710978
O 0.8294498747 -2.0075583445 -0.6223247354
C 1.2028880562 -3.0087832337 -1.6402961535
C -2.3647329614 -3.6393483132 -0.4538231859
H -1.7903349006 -4.5644614677 -0.5693681847
H -2.7806556305 -3.3772381744 -1.4358158588
H -3.2097852468 -3.8326467205 0.2161504781
C 1.3723623205 -4.3593513726 -0.9396938294
H 1.7045096876 -5.1141077432 -1.6614699524
H 2.1227982962 -4.2836520637 -0.146743203
H 0.4336329627 -4.706460506 -0.4964174527
C 2.5454395397 -2.4760882981 -2.1454909094
H 2.9910049245 -3.1942809056 -2.8430292317
H 2.4060778069 -1.5218921793 -2.6623087648
H 3.2275244565 -2.3032347465 -1.3095448153
C 0.1696297275 -3.0416087573 -2.7694394951
H -0.7549202162 -3.5451798529 -2.4792840905
H -0.0750241279 -2.0247308183 -3.0901430635
H 0.5896775227 -3.5852656539 -3.6231585109
C -1.0975912856 1.0325671441 -0.6162964374
C -0.619074238 0.758894787 -1.9195372583
C -2.4974585224 1.1850413517 -0.4994987992
H 0.4525229274 0.6458105876 -2.0789351608
H -2.9318723667 1.4062002471 0.4737419785
C -1.4677576638 0.6530383933 -3.0263689008
C -3.362151683 1.0713670306 -1.5946567044
H -1.0547574193 0.4683707701 -4.0182846284
H -4.4350871672 1.1982821506 -1.457285079
C -2.8492066015 0.8003726783 -2.8644997364
H -3.5156879545 0.7152494225 -3.7212531096
O -1.1149094771 4.1264060943 4.3183807426
S -0.9548510069 2.8789502822 3.5819664667
C -2.0486315021 1.6621627094 4.4749169944
O 0.4021880738 2.2396485805 3.5498566875
O -1.455535886 2.8478963679 2.1534283454
F -2.0118218029 0.4616934617 3.8973953636
F -3.311027873 2.1089763057 4.4699725999
F -1.6373688368 1.5459650274 5.7453618144

Frequency: -97.2206; 12.6016; 19.3583; 25.2183; 28.3853; 29.3563; 34.0041; 37.3517; 38.4475; 40.1883; 46.4360; 49.6111; 54.2038; 57.9952; 67.2178; 69.0597; 71.1869; 74.0054; 76.9975; 82.6446; 87.1815; 91.4028; 96.7996; 112.0406; 128.0617; 130.7683; 136.3980; 144.0155; 145.8927; 147.0092; 153.3411; 157.5783; 167.2136; 179.6237; 190.5388; 204.4236; 208.4797; 210.6028; 212.4895; 214.2618; 220.5004; 223.0070; 224.1103; 235.5151; 252.9078; 263.9985; 291.6559; 299.1876; 302.7343; 305.8458; 308.3377; 320.9100; 322.2762; 325.3015; 334.6810; 350.2272; 351.0311; 352.6008; 358.3802; 395.5801; 397.4330; 422.1929; 454.7884; 482.8318; 484.1702; 489.4107; 490.7377; 491.5072; 523.2848; 524.6889; 530.6939; 557.4398; 558.0745; 559.0543; 569.7871; 570.6692; 578.0083; 587.0925; 608.4820; 619.0292; 625.0790; 631.0314; 633.7382; 638.6739; 647.4728; 715.8690; 731.4057; 759.5784; 760.7512; 761.2273; 762.0000; 765.7919; 783.2700; 847.8943; 869.3652; 918.3916; 935.8530; 940.0082; 972.4375; 974.0385; 974.6532; 979.1624; 984.2150; 984.8701; 986.9195; 994.3234; 1011.6201; 1015.1235; 1052.4530; 1061.7176; 1069.6381; 1075.4349; 1085.7999; 1100.4467; 1105.4026; 1116.8879; 1135.6399; 1183.9845; 1198.4616; 1211.8470; 1212.9558; 1214.1414; 1214.6721; 1222.7572; 1224.1735; 1228.6218; 1232.7072; 1243.2991; 1249.7402; 1254.3222; 1282.1272; 1291.2344; 1296.2305; 1298.3987; 1305.9599; 1318.6884; 1368.2923; 1387.3858; 1429.6657; 1436.9164; 1438.1694; 1459.2537; 1461.2485; 1500.8350; 1507.5446; 1514.0490; 1515.6795; 1517.5902; 1521.1784; 1527.6313; 1532.2608; 1548.9867; 1611.4896; 1618.9432; 1638.8502; 1729.6676; 1876.2489; 3040.8449; 3058.9819; 3062.7685; 3071.1156; 3099.3949; 3123.0418; 3128.1606; 3133.4898; 3137.5158; 3139.5087; 3141.1237; 3153.3878; 3163.3166; 3164.9635; 3165.8413; 3172.8202; 3182.3484.

Cartesian Forces: Max 0.000001156 RMS 0.000000308

Item	Value	Threshold	Converged?
Maximum Force	0.000001	0.000450	YES
RMS Force	0.000000	0.000300	YES
Maximum Displacement	0.000088	0.001800	YES
RMS Displacement	0.000019	0.001200	YES

FILE: Yb_Sq2_PhYbOTf3_TS_Anion_B3LYP_M062x_Add12_Conf4-1.out

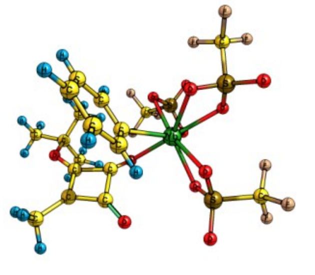

Conformer 9

E(RB3LYP/6-31G(d)/MWB59) = -3731.44491913
Convg = 0.7355D-08 -V/T = 2.0122

Temperature 195.150 Kelvin. Pressure 1.00000 Atm.
elec. and zero-point Energies= -3731.073625
elec. and thermal Energies= -3731.050563
elec. and thermal Enthalpies= -3731.049945
elec. and thermal Free Energies= -3731.118474

RB3LYP/6-31G(d)/MWB59/THF(IEFPCM) = -3731.51226347

RM06-2x/6-311+G(d,p)/MWB59/THF(IEFPCM) = -3731.35206585

Geometry at RB3LYP/6-31G(d)/MWB59
Charge = -1 Multiplicity = 1
Yb 0.3383422538 2.1801239107 1.3148285092
O 2.6246181216 2.3571506197 2.399365062
S 3.3586613004 2.5791725746 1.1167375926
O 4.1820081834 3.774121612 0.9701940192
O 2.3730794831 2.2898387157 0.0028267583
C 4.6030939116 1.1902494945 1.0296226577
F 5.6078323331 1.4410122362 1.876849398
F 4.0604472172 0.0108182002 1.3569246455
F 5.1029949882 1.0975664883 -0.2136214498
O 0.7857106855 4.5210053406 1.7399876125
O -1.0081639263 6.1552902469 1.0018329966
O -0.5392245179 3.9507396948 -0.1755101407
S -0.0502313527 5.1187455562 0.6334123376
C 1.1739462208 5.9894223208 -0.4721691927
F 1.8135217536 6.9371141941 0.2228953848
F 2.0670588219 5.1385726792 -0.9761324998
F 0.5129829279 6.5700159644 -1.4850493865
C 0.0808984899 -1.965042367 -0.0714740239
C -1.1902528519 -2.3368431471 0.2990815035
C -1.3330381338 -1.210944607 1.2432748803
C 0.083790766 -0.7416102661 0.8370425997
O 0.9853174795 -0.0887951809 1.4001176291
O -2.1537503227 -0.8489687043 2.0531322592
O 0.8745583011 -2.5839077919 -0.9283670969
C 2.3499367938 -2.4472381301 -0.9142164329
C -2.0884577113 -3.4562432078 -0.0766829944
H -1.5420903479 -4.2558169236 -0.5873447325
H -2.8784248112 -3.0963184294 -0.7500628853
H -2.5865102993 -3.8665594219 0.8094347977
C 2.7454291226 -1.0539337731 -1.3932665807
H 3.8361579011 -0.9753123072 -1.4461889625
H 2.3341070473 -0.8641427419 -2.3894522472
H 2.392241783 -0.2799694497 -0.712156029
C 2.7841680189 -3.5194287498 -1.9158114492
H 3.8751631705 -3.5207246015 -2.0099389411
H 2.4610965112 -4.5128892652 -1.587150299
H 2.3510946139 -3.3219493654 -2.901755394
C 2.8639958377 -2.7637807647 0.4922891353
H 2.5246688129 -2.0202417444 1.2167697624
H 2.5279519912 -3.7580364342 0.8076633556
H 3.9590762809 -2.7528926615 0.4951694584
C -0.9015468874 1.0295147016 -0.5666680177
C -0.2451143195 1.0185705566 -1.8162923623
C -2.3139453411 1.0220458893 -0.6081951536
H 0.842259906 1.0508770109 -1.8547932961
H -2.876957358 1.0353357492 0.3223912845
C -0.9413407248 1.0080960179 -3.0304698921
C -3.022725365 1.0110879089 -1.8119518759
H -0.3961184083 1.0113273642 -3.9734327797
H -4.1116836712 1.0215853821 -1.8022582397
C -2.3367281315 0.9971427224 -3.030482865
H -2.8863401283 0.9857730432 -3.9700099008
O -2.5041516347 1.373450767 4.3864618566
S -1.510242356 2.1946853901 3.7111630883
C -1.4129576517 3.7374874396 4.7578045248
O -1.8168036861 2.6826136107 2.3149016927
O -0.089485766 1.6888213965 3.6422232142
F -0.4533863612 4.5583294225 4.3393617835
F -1.1592519062 3.3849477082 6.0265875543
F -2.5887115357 4.3754902818 4.7151716671

Frequency: -147.6305; 16.5533; 19.5014; 23.3636; 28.2956; 31.8220; 37.3536; 38.3302; 42.5035; 45.0349; 48.9557; 50.3061; 56.8138; 59.4621; 63.2270; 69.6712; 75.9441; 80.0536; 83.2007; 85.5194; 89.0864; 90.0462; 94.6843; 104.9710; 115.0398; 120.4398; 129.4298; 134.0400; 141.1209; 145.6920; 150.3424; 157.9373; 167.4368; 181.7874; 185.4579; 196.2745; 209.2349; 214.4204; 215.1488; 216.9765; 222.5652; 224.2453; 227.3076; 257.8595; 279.1038; 286.9799; 300.1404; 304.2645; 306.0476; 308.4399; 311.0622; 319.5373; 324.0155; 325.6470; 337.1880; 349.2894; 350.1319; 352.8880; 358.7173; 397.4364; 401.3475; 420.8832; 457.5053; 479.2363; 488.0467; 489.1853; 490.0300; 490.4981; 522.7913; 525.5296; 530.8124; 557.0616; 557.7500; 558.0169; 567.5341; 572.1801; 575.5540; 588.9948; 607.0799; 624.1215; 625.6576; 629.6416; 630.7884; 635.5684; 640.4381; 715.3223; 723.1134; 734.1963; 757.2332; 757.9833; 761.7116; 761.9111; 789.2834; 864.3974; 867.2551; 923.9851; 937.1741; 938.8718; 958.7691; 970.1720; 972.6498; 977.6240; 979.2905; 983.7412; 986.5174; 991.2411; 1007.7364; 1014.4895; 1054.1884; 1059.5270; 1067.6827; 1073.0914; 1076.7681; 1091.1156; 1097.5119; 1107.0073; 1128.5400; 1185.7963; 1206.9318; 1208.1656; 1211.1274; 1212.4909; 1223.2505; 1224.4592; 1231.6162; 1232.5618; 1232.9683; 1241.6081; 1251.1079; 1259.1771; 1290.2324; 1299.3319; 1301.6854; 1306.0107; 1308.5610; 1318.8246; 1369.2627; 1380.1497; 1429.7072; 1436.9805; 1437.8151; 1460.2164; 1461.9639; 1502.7817; 1504.4133; 1509.8325; 1516.7480; 1518.3719; 1519.1602; 1532.1473; 1535.6487; 1554.0843; 1614.0184; 1620.3965; 1627.4962; 1688.6995; 1872.9221; 3039.3621; 3056.2860; 3061.3969; 3074.4962; 3095.5600; 3123.9266; 3127.9550; 3131.4039; 3131.5956; 3139.4985; 3148.0341; 3158.3532; 3163.4607; 3173.4810; 3181.4618; 3189.4901; 3190.2304.

Cartesian Forces: Max 0.000005874 RMS 0.000000684

Item	Value	Threshold	Converged?
Maximum Force	0.000004	0.000450	YES
RMS Force	0.000000	0.000300	YES
Maximum Displacement	0.000492	0.001800	YES
RMS Displacement	0.000082	0.001200	YES

FILE: Yb_Sq2_PhYbOTf3_TS_Anion_B3LYP_M062x_Add12_Conf5.out

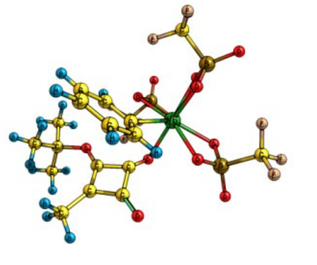

Conformer 10

E(RB3LYP/6-31G(d)/MWB59) = -3731.44733219
Convg = 0.4621D-08 -V/T = 2.0122

Temperature 195.150 Kelvin. Pressure 1.00000 Atm.
elec. and zero-point Energies= -3731.075982
elec. and thermal Energies= -3731.052850
elec. and thermal Enthalpies= -3731.052232
elec. and thermal Free Energies= -3731.121611

RB3LYP/6-31G(d)/MWB59/THF(IEFPCM) = -3731.51699601

RM06-2x/6-311+G(d,p)/MWB59/THF(IEFPCM) = -3731.35465199

Geometry at RB3LYP/6-31G(d)/MWB59
Charge = -1 Multiplicity = 1
Yb 0.4235092891 2.0640572533 1.4063015491
O 2.7739460574 1.6844210278 2.1849370334
S 3.3537933582 1.447490548 0.8261411646
O 4.5162976936 2.2250788577 0.4122924728
O 2.1867632517 1.3914711738 -0.1330276125
C 4.0034152618 -0.3006209689 0.8828737442
F 5.0157400735 -0.370487589 1.7568473835
F 3.0675585127 -1.1824579305 1.2432014415
F 4.467049379 -0.6485943554 -0.3325347873
O 1.6460225782 4.1780897364 1.109835052
O 0.3176594909 6.3418191403 1.1668378568
O -0.7012317152 4.0772181038 0.633863103
S 0.4881755556 5.0019888892 0.6185577921
C 0.838621167 5.2575050955 -1.192557185
F 1.9458419478 5.9934256102 -1.3423671394
F 1.0131760783 4.0826102405 -1.8071132199
F -0.1868840489 5.8977384188 -1.7679959168
C -0.2995909842 -1.8145987022 0.1969067956
C -1.5578258807 -2.3536607842 0.3991430682
C -1.8946154148 -1.3580810289 1.448449002
C -0.4678057648 -0.7774892635 1.2742871193
O 0.3420903234 -0.1719611379 1.9909192589
O -2.8509886665 -1.1849457457 2.1632152376
O 0.7964230842 -1.9684263757 -0.5066380964
C 1.0159957407 -2.991719376 -1.543937222
C -2.4482067356 -3.445213454 -0.0807633942
H -1.9323554213 -4.3969644633 -0.2442349582
H -2.9399503285 -3.163045254 -1.0215243913
H -3.2379417832 -3.5982211831 0.6632336303
C 1.1513689237 -4.3538630624 -0.8575770293
H 1.3788959983 -5.1260862121 -1.6011027163
H 1.9628692409 -4.3297430797 -0.1233193683
H 0.2302105341 -4.6418147611 -0.3426050972
C 2.3472310396 -2.5547592109 -2.1596405333
H 2.6699558101 -3.2854039718 -2.9093410418
H 2.2431318803 -1.5779318116 -2.6411433238
H 3.1170425226 -2.4690621629 -1.3897829679
C -0.0993405736 -2.9520449151 -2.5922414379
H -1.0334927001 -3.383160216 -2.2275669309
H -0.2922775858 -1.9209146547 -2.9027240354
H 0.2121702167 -3.5295636666 -3.4699940587
C -1.0472584365 1.1256310948 -0.3922264898
C -0.5046358179 0.9017855604 -1.6788161018
C -2.4521905994 1.271128804 -0.3333647806
H 0.5719416696 0.7881109127 -1.7909969815
H -2.9285125871 1.4466336743 0.6285895471
C -1.3006583751 0.8442623982 -2.8296171556
C -3.2613441917 1.2020040098 -1.4724012457
H -0.839829637 0.6997994875 -3.8069763863
H -4.3400872536 1.3223710174 -1.3820792343
C -2.6872959335 0.9859411513 -2.727779091
H -3.3120866137 0.9379603123 -3.6181872448
O -1.4489776075 1.4986915148 5.1453524018
S -1.1113547079 2.3424299161 4.0054231616
C -1.8149525653 4.0160859576 4.4264308704
O -1.7037731113 2.0002769047 2.6674800227
O 0.3522688296 2.6093042427 3.7467553086
F -1.5081795363 4.9162884903 3.4922249592
F -1.3210393178 4.4251401957 5.6025377045
F -3.1479650943 3.9280537365 4.5260918984

Frequency: -107.5088; 10.0700; 16.1655; 21.5565; 27.7057; 29.0621; 30.8206; 34.0200; 38.7065; 44.4071; 48.0429; 48.7245; 56.2247; 59.3746; 61.3265; 65.1558; 70.6446; 72.7289; 77.1706; 78.2016; 87.3056; 91.3466; 100.6225; 123.6999; 126.2379; 134.9697; 137.4689; 138.3054; 146.9944; 150.6063; 157.5023; 161.2301; 168.7361; 180.4394; 190.6367; 203.3942; 206.1286; 209.7975; 212.7676; 215.0235; 219.3395; 221.8181; 227.7492; 240.2125; 254.0426; 267.5523; 293.8700; 301.4675; 304.7328; 305.0262; 309.0858; 319.9273; 322.7781; 325.8917; 337.2324; 349.8591; 351.2216; 353.3478; 357.8057; 396.7907; 400.4846; 423.1857; 454.2688; 484.0725; 484.6798; 488.9322; 490.2268; 490.9520; 523.0708; 525.3549; 529.6989; 557.0613; 557.6183; 558.2500; 568.8117; 572.8463; 575.6128; 589.7433; 608.9888; 622.8592; 626.7073; 629.9542; 630.7419; 636.1384; 649.0912; 718.2117; 731.8779; 758.1094; 761.0483; 761.2944; 762.0117; 765.3499; 782.7831; 850.8450; 872.5828; 925.8366; 935.0353; 938.1360; 971.3009; 975.3716; 977.7712; 981.3881; 983.2635; 985.9796; 991.3314; 994.5364; 1011.1184; 1012.9930; 1052.4387; 1060.4590; 1065.7784; 1075.3786; 1086.1393; 1091.4452; 1106.8028; 1118.8005; 1131.8365; 1184.1608; 1196.6640; 1201.5379; 1213.8624; 1215.7224; 1216.4904; 1224.0980; 1225.0089; 1233.5209; 1235.7660; 1238.2374; 1239.5786; 1248.8523; 1282.4515; 1294.9393; 1298.8242; 1306.8835; 1308.5982; 1317.3388; 1369.9043; 1380.0994; 1429.4022; 1435.6013; 1437.0602; 1459.4952; 1459.7023; 1501.5032; 1507.5511; 1514.2550; 1516.2464; 1518.3749; 1522.2842; 1527.2611; 1532.7057; 1549.7362; 1611.3605; 1618.2077; 1636.5442; 1717.1090; 1881.0655; 3039.4357; 3060.7455; 3065.4838; 3072.7634; 3098.1347; 3123.2853; 3129.4172; 3134.6146; 3139.3364; 3140.8498; 3143.7632; 3154.7331; 3167.8780; 3170.1180; 3173.7235; 3180.0978; 3191.9510.

Cartesian Forces: Max 0.000005988 RMS 0.000000808

Item	Value	Threshold	Converged?
Maximum Force	0.000004	0.000450	YES
RMS Force	0.000000	0.000300	YES
Maximum Displacement	0.000198	0.001800	YES
RMS Displacement	0.000045	0.001200	YES

FILE: Yb_Sq2_PhYbOTf3_TS_Anion_B3LYP_M062x_Add12_Conf5-1.out

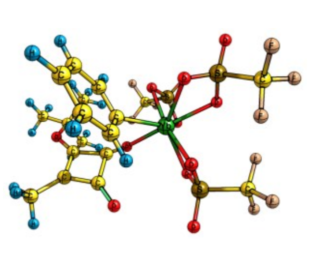

Conformer 11

E(RB3LYP/6-31G(d)/MWB59) = -3731.44415834
Convg = 0.8749D-08 -V/T = 2.0122

Temperature 195.150 Kelvin. Pressure 1.00000 Atm.
elec. and zero-point Energies= -3731.072849
elec. and thermal Energies= -3731.049804
elec. and thermal Enthalpies= -3731.049186
elec. and thermal Free Energies= -3731.117473

RB3LYP/6-31G(d)/MWB59/THF(IEFPCM) = -3731.51199332

RM06-2x/6-311+G(d,p)/MWB59/THF(IEFPCM) = -3731.35222178

GGeometry at RB3LYP/6-31G(d)/MWB59
Charge = -1 Multiplicity = 1
Yb 0.2287782624 2.0979043455 1.4781725984
O 2.5257661016 2.3649875273 2.5109401105
S 3.2056231275 2.7309145942 1.2306087775
O 3.943988454 3.9848044656 1.1518992879
O 2.2062737814 2.4543832771 0.1245824983
C 4.536472516 1.4392671529 1.0122626909
F 5.5518020904 1.7039004267 1.8424687549
F 4.0863145868 0.2060376448 1.2789099988
F 4.9972172029 1.4592258132 -0.2491317507
O -0.6950961504 3.8826960814 0.041362726
O 0.5629291885 6.0878887177 0.1021498104
O 0.5047510318 4.4463295679 2.0375836042
S -0.16067022 5.0490373554 0.8244966186
C -1.6666685262 5.9269902608 1.4913581116
F -2.3584337175 6.4525143174 0.4710779171
F -2.4651653039 5.1007837236 2.1695325031
F -1.2798514226 6.9182590181 2.3052281399
C 0.2178774664 -1.9743230577 -0.1270503982
C -1.0109844335 -2.4644821402 0.2502943302
C -1.2049639883 -1.42034833 1.2755508412
C 0.1576845615 -0.8173996958 0.8614247485
O 1.0194183841 -0.1241003119 1.4375597931
O -2.0181185344 -1.1828998898 2.1371767359
O 1.0267611627 -2.4757013785 -1.0436185815
C 2.4873765285 -2.2249965006 -1.0656787564
C -1.8354280846 -3.6206015759 -0.1786274519
H -1.2467102123 -4.3431152729 -0.7530485225
H -2.6666373082 -3.2767500995 -0.8094127392
H -2.278928185 -4.1222814164 0.6892413555
C 2.7571585853 -0.779397252 -1.4709011673
H 3.8358717831 -0.6112381882 -1.551262079
H 2.2983296877 -0.5654859709 -2.4410228758
H 2.3687694565 -0.0760772117 -0.734629068
C 2.9668845883 -3.1990610255 -2.1438940758
H 4.0508096101 -3.1090967077 -2.2707735083
H 2.7325042304 -4.2324474601 -1.8672149236
H 2.48594415 -2.9783677183 -3.1023171551
C 3.0723484061 -2.5834075304 0.3024395826
H 2.7014716362 -1.9144968525 1.0820293245
H 2.8259482885 -3.6185927188 0.5644249881
H 4.1625872153 -2.4849137765 0.2738743787
C -1.0067099557 0.9643072644 -0.4155466533
C -0.4047207745 1.0531234035 -1.6895456139
C -2.4145275599 0.8421941726 -0.4038684227
H 0.6736213643 1.1790116165 -1.768928971
H -2.9370763124 0.7748163535 0.5476430577
C -1.1471299723 1.0287904586 -2.8755895667
C -3.1695640686 0.8154844056 -1.5793936395
H -0.6434290734 1.1127951298 -3.8376559328
H -4.2545140631 0.7343042253 -1.5276899429
C -2.5359205108 0.9019084782 -2.822947397
H -3.1210211531 0.8786470936 -3.7406186906
O -2.3808582161 0.8546112022 4.6158430077
S -1.5404483892 1.8196869893 3.9226648584
C -1.6833473839 3.3623489832 4.9659742094
O -1.9457132342 2.2508502223 2.5327474237
O -0.060734755 1.5474779081 3.8302956421
F -0.8614806343 4.3242287005 4.5494225541
F -1.3831277805 3.0588764123 6.2362342523
F -2.9436310023 3.8136269484 4.9171415519

Frequency: -143.0594; 16.0204; 20.8908; 25.4424; 31.5969; 34.0261; 36.0984; 38.2839; 43.2202; 45.8173; 50.3563; 52.6867; 58.0271; 58.7227; 62.0509; 71.7946; 77.9059; 83.0496; 84.9100; 86.7040; 88.7055; 90.1764; 95.2432; 104.7129; 113.3662; 119.0572; 129.7881; 133.6634; 140.8623; 143.1798; 146.5762; 158.0803; 165.8997; 181.1608; 186.5327; 196.9114; 208.8793; 210.5389; 211.5198; 218.4102; 223.3972; 224.3146; 227.9034; 256.9312; 279.5983; 288.8112; 299.4416; 302.0201; 305.4626; 308.1741; 310.8111; 319.6393; 322.9558; 324.8533; 336.8930; 349.0474; 349.8325; 352.6597; 358.5245; 397.3703; 401.4718; 420.3798; 457.6914; 480.0852; 488.0040; 489.4712; 490.5729; 491.1893; 522.2064; 526.3566; 531.3618; 556.8623; 557.6936; 557.9034; 566.4220; 572.8033; 577.1472; 587.7544; 606.6899; 618.7376; 624.0041; 630.0096; 630.8385; 639.1750; 639.8168; 715.8761; 723.1878; 734.5479; 756.8510; 757.6264; 760.4921; 761.2189; 789.4307; 864.4876; 867.8336; 924.3381; 937.5793; 939.1121; 959.1385; 969.7649; 971.6150; 979.5485; 980.6410; 985.1822; 987.2398; 991.1901; 1007.8030; 1014.4069; 1054.1376; 1059.5458; 1067.8925; 1072.9378; 1076.8016; 1090.6497; 1100.4665; 1105.9240; 1128.5827; 1185.5573; 1206.8514; 1208.8897; 1212.4166; 1212.9601; 1222.7044; 1223.4866; 1226.6327; 1229.7543; 1231.9649; 1242.5031; 1249.6895; 1254.8707; 1290.6857; 1299.3796; 1301.0740; 1304.5526; 1312.9111; 1322.9453; 1368.8810; 1381.1135; 1429.8696; 1437.5005; 1437.9033; 1460.5812; 1461.7265; 1502.8871; 1504.3663; 1509.6420; 1516.5303; 1518.2706; 1519.2887; 1532.5309; 1535.2545; 1553.7267; 1613.8080; 1620.0709; 1627.5298; 1689.4549; 1874.0881; 3039.5769; 3056.4818; 3061.8179; 3075.7716; 3095.8573; 3124.4460; 3128.2942; 3131.7486; 3132.2429; 3140.6948; 3147.6434; 3157.3206; 3164.0440; 3172.8302; 3181.4644; 3190.1669; 3190.7055.

Cartesian Forces: Max 0.000001745 RMS 0.000000650

Item	Value	Threshold	Converged?
Maximum Force	0.000001	0.000450	YES
RMS Force	0.000000	0.000300	YES
Maximum Displacement	0.000098	0.001800	YES
RMS Displacement	0.000018	0.001200	YES

FILE: Yb_Sq2_PhYbOTf3_TS_Anion_B3LYP_M062x_Add12_Conf6.out

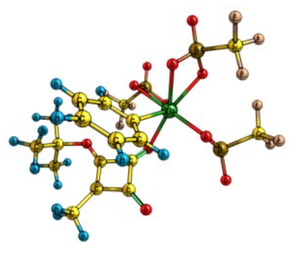

Conformer 12

E(RB3LYP/6-31G(d)/MWB59) = -3731.44580606
Convg = 0.7836D-08 -V/T = 2.0122

Temperature 195.150 Kelvin. Pressure 1.00000 Atm.
elec. and zero-point Energies= -3731.074572
elec. and thermal Energies= -3731.051423
elec. and thermal Enthalpies= -3731.050805
elec. and thermal Free Energies= -3731.119879

RB3LYP/6-31G(d)/MWB59/THF(IEFPCM) = -3731.51622074

RM06-2x/6-311+G(d,p)/MWB59/THF(IEFPCM) = -3731.35277196

Geometry at RB3LYP/6-31G(d)/MWB59
Charge = -1 Multiplicity = 1
Yb 0.150271657 1.9830745865 1.4222466442
O 2.5456180184 1.809834123 2.165021877
S 3.1312676006 1.7633542658 0.7897047282
O 4.2033562438 2.6921919683 0.4526870445
O 1.9615674168 1.6890878951 -0.1659270178
C 3.9461449613 0.0900915215 0.6708478337
F 4.9950149229 0.0443512424 1.5017088984
F 3.1090514179 -0.9027754161 0.9863413536
F 4.387581528 -0.1112811493 -0.5840693431
O -0.4893782029 3.7858630622 -0.1475484253
O 1.0021839701 5.8451550872 -0.1461436396
O 0.7441762863 4.2936142397 1.8434870186
S 0.161016899 4.9162455118 0.5988309133
C -1.2637473563 5.9579326952 1.2050971443
F -1.8070268937 6.6110265441 0.1688995711
F -2.2069075512 5.2051090879 1.7769236357
F -0.8235540911 6.8547889972 2.096379392
C -0.1930148721 -1.9742906604 0.138422696
C -1.3678576853 -2.6738977006 0.3650043427
C -1.7725318695 -1.7741297071 1.4750353117
C -0.4404854939 -1.0177741067 1.2697958374
O 0.287165777 -0.2893763382 1.9492707356
O -2.7086236268 -1.7372817985 2.2359249596
O 0.8780041701 -1.9560696255 -0.615590521
C 1.1790951398 -2.8923767893 -1.7151931129
C -2.1391054748 -3.8410894582 -0.1403539673
H -1.5170464572 -4.708481721 -0.3835479405
H -2.706271944 -3.5697519915 -1.0407361508
H -2.8667844296 -4.1337279495 0.6246489078
C 1.5235493484 -4.2538352077 -1.1052447835
H 1.8119429704 -4.9549956416 -1.896452646
H 2.3602870519 -4.1569483392 -0.4061763423
H 0.6732740667 -4.6835406222 -0.5671467336
C 2.4098422999 -2.2522947013 -2.3612183592
H 2.7917957599 -2.8989650284 -3.1586343392
H 2.153735652 -1.2786316541 -2.7892188843
H 3.1966859762 -2.0969020364 -1.6199109265
C 0.0185173161 -2.9537382471 -2.7115397598
H -0.8278924355 -3.5311526684 -2.3348057854
H -0.3300946376 -1.9451697801 -2.9521509878
H 0.3633378722 -3.4348233515 -3.6338014591
C -1.3305620066 0.9327988323 -0.2893404508
C -0.8538579808 0.783454196 -1.6133327174
C -2.7321960397 0.8368314581 -0.1325425508
H 0.2150138451 0.8666862491 -1.8072205663
H -3.1660739692 0.9520886305 0.8590618012
C -1.7058482273 0.5649066948 -2.7017137581
C -3.5978881129 0.604563377 -1.208359166
H -1.2981415835 0.4884534318 -3.7100554628
H -4.6720910982 0.5395501688 -1.0392586741
C -3.0862469691 0.4639851908 -2.5000206405
H -3.7544337253 0.2909417076 -3.342211904
O -2.0878785606 1.1817431346 4.9080014758
S -1.3689558061 2.1148955846 4.0490937521
C -1.6369348082 3.7763461611 4.856125344
O -1.8892327117 2.2885696573 2.6367904382
O 0.116395336 1.9679027955 3.9211325539
F -0.9330322681 4.7382625036 4.2586892609
F -1.2601515394 3.7034340393 6.1400824125
F -2.9373475435 4.0936110381 4.8063187059

Frequency: -81.7692; 13.2351; 15.1287; 25.7718; 27.4854; 32.2789; 33.7159; 34.7338; 41.5271; 44.0909; 45.8681; 52.3456; 54.8909; 57.3889; 62.3529; 68.2595; 72.0592; 75.4015; 76.4632; 81.4959; 86.0109; 89.1394; 97.5449; 117.3563; 124.0737; 128.5748; 137.5745; 143.9977; 147.1722; 150.9507; 154.5520; 158.2780; 166.7282; 180.5448; 190.1471; 203.8037; 207.3975; 208.7200; 211.9566; 212.5695; 218.5319; 221.0820; 226.4012; 237.0011; 252.8324; 267.8452; 291.2804; 293.4679; 301.6897; 304.4756; 307.4214; 320.5737; 322.9362; 324.2614; 334.7893; 349.8800; 350.8195; 351.6317; 357.8150; 396.6313; 397.9188; 424.2342; 454.5381; 483.0052; 484.5704; 489.6065; 490.6151; 491.7636; 523.5481; 525.2727; 530.5863; 557.2227; 557.9743; 558.5231; 568.8180; 571.6870; 577.4654; 585.9975; 604.1111; 618.5863; 625.3394; 631.2246; 633.8248; 639.6318; 647.7053; 716.9231; 732.7205; 758.6009; 760.1442; 760.5179; 761.2570; 765.5458; 783.3894; 849.7446; 871.2177; 918.9310; 935.5313; 938.9809; 972.1075; 973.3912; 975.5126; 980.3670; 982.6252; 984.8764; 987.5952; 994.7064; 1011.3428; 1016.0627; 1051.4634; 1060.8362; 1067.3569; 1075.9814; 1085.0850; 1096.2336; 1109.1979; 1111.6950; 1135.7303; 1183.3957; 1197.3828; 1204.4691; 1211.7118; 1213.6484; 1215.1600; 1223.7048; 1225.7393; 1226.9344; 1229.3418; 1236.5888; 1245.7805; 1253.0564; 1282.7505; 1295.0200; 1297.0459; 1304.4686; 1308.3503; 1320.0111; 1369.7921; 1382.6519; 1429.7788; 1435.8055; 1437.2971; 1458.5174; 1459.9217; 1501.4808; 1507.9432; 1513.6068; 1516.3536; 1518.1694; 1522.1616; 1527.3533; 1533.1850; 1549.8207; 1610.9116; 1618.1772; 1641.2778; 1740.2202; 1882.1372; 3042.0219; 3060.6646; 3066.1494; 3073.2401; 3100.9748; 3125.0114; 3129.7149; 3135.5757; 3136.0678; 3141.2747; 3142.5558; 3151.0068; 3163.0894; 3167.1436; 3169.8977; 3172.7715; 3182.1136.

Cartesian Forces: Max 0.000001224 RMS 0.000000511

Item	Value	Threshold	Converged?
Maximum Force	0.000000	0.000450	YES
RMS Force	0.000000	0.000300	YES
Maximum Displacement	0.000045	0.001800	YES
RMS Displacement	0.000006	0.001200	YES

FILE: Yb_Sq2_PhYbOTf3_TS_Anion_B3LYP_M062x_Add12_Conf6-1.out

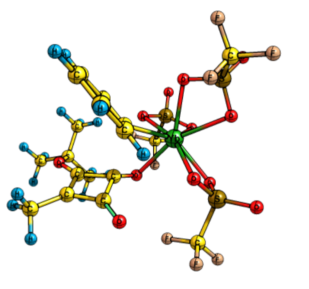

Conformer 13

E(RB3LYP/6-31G(d)/MWB59) = -3731.44700758
Convg = 0.7613D-08 -V/T = 2.0122

Temperature 195.150 Kelvin. Pressure 1.00000 Atm.
elec. and zero-point Energies= -3731.075741
elec. and thermal Energies= -3731.052739
elec. and thermal Enthalpies= -3731.052121
elec. and thermal Free Energies= -3731.120213

RB3LYP/6-31G(d)/MWB59/THF(IEFPCM) = -3731.51195215

RM06-2x/6-311+G(d,p)/MWB59/THF(IEFPCM) = -3731.35082577

Geometry at RB3LYP/6-31G(d)/MWB59
Charge = -1 Multiplicity = 1
Yb 0.4109424805 2.1639965558 1.5127846088
O 2.6276079629 3.090902875 1.9938437026
S 3.3479152272 2.6022514709 0.7695961965
O 4.1936097246 3.5354546607 0.0375562707
O 2.3405647682 1.8047739093 -0.0148887135
C 4.5263907198 1.3168350334 1.4320662635
F 5.5386749272 1.9086520553 2.0730188921
F 3.9068427168 0.4871277502 2.2799106426
F 5.0265297356 0.5907690999 0.4159249356
O 0.3158110412 4.0553358829 -0.0158384812
O 0.533642158 6.3841674855 0.9737934019
O -0.3075574777 4.3721302206 2.2792413272
S -0.1486755179 5.0969286658 0.9709565213
C -1.8957967893 5.4750510548 0.44719938
F -1.8993950817 5.9830474825 -0.791360645
F -2.6429516342 4.3665211109 0.4623821646
F -2.4344226961 6.3706797447 1.283185524
C 0.1432139412 -1.7901669623 -0.1551646727
C -1.1034065502 -2.3047902761 0.1106129704
C -1.3618460749 -1.3653441133 1.212733419
C 0.0237929464 -0.7084787675 0.9248721873
O 0.8620655311 -0.1102448979 1.635088559
O -2.2245339765 -1.2446317258 2.0506926155
O 0.9760678136 -2.2361278021 -1.0794930039
C 2.4558963799 -2.1320204028 -1.0652953772
C -1.8898346984 -3.4291664658 -0.4541557041
H -1.2548562672 -4.1147869485 -1.0249000254
H -2.6702739014 -3.0479024121 -1.1269058462
H -2.3976164362 -3.9835910727 0.3435664342
C 2.8580535949 -0.8633923494 -1.8127173195
H 3.9475612813 -0.8341961938 -1.9289663825
H 2.4040563752 -0.8533319958 -2.8093734892
H 2.5591582949 0.035739602 -1.2731132961
C 2.871158273 -3.3827575892 -1.8471258343
H 3.9610977022 -3.4123064011 -1.951440118
H 2.5460836375 -4.2915808637 -1.3294779312
H 2.4273367873 -3.3757835325 -2.848050279
C 2.9920042598 -2.177865252 0.3641373579
H 2.6655151113 -1.3173823918 0.9495466866
H 2.6699090994 -3.0963591803 0.8689404692
H 4.0868471574 -2.1696280964 0.3367972221
C -0.9865041746 1.1388107681 -0.3223557457
C -0.4841705877 1.1986277066 -1.641478567
C -2.3934133807 1.1473440244 -0.189199505
H 0.5908740131 1.2182261632 -1.80751114
H -2.8367145348 1.0928708759 0.8021608688
C -1.3234127523 1.2694138827 -2.75731294
C -3.2439339597 1.2234457636 -1.296818047
H -0.8977370986 1.3231720303 -3.7582551016
H -4.3233247954 1.2414168577 -1.1538265868
C -2.7100324991 1.2775454013 -2.5867999238
H -3.3687423273 1.3314997243 -3.451749828
O -1.5110886676 2.6607943896 5.1959836499
S -0.8698231928 1.7971244679 4.2124200127
C -0.997870862 0.0907670204 4.9621422246
O 0.5998437874 1.9873735405 3.94657976
O -1.5198068341 1.6942596951 2.8531137359
F -0.3065011212 -0.8147870595 4.2675810965
F -2.2779945578 -0.2817495764 5.025501858
F -0.4990974793 0.1356385332 6.2083414452

Frequency: -174.0006; 14.8966; 24.3821; 26.7494; 32.2848; 32.7529; 38.4366; 41.9880; 45.1000; 46.1182; 51.3998; 52.8956; 55.6716; 60.6368; 61.4583; 68.6607; 70.7718; 74.8618; 81.5835; 84.3189; 87.1179; 87.4194; 99.4717; 100.6537; 117.4626; 130.2542; 134.5438; 137.4868; 141.2352; 150.3270; 155.9690; 158.9330; 167.1826; 182.9222; 187.8169; 199.0486; 210.4127; 213.9391; 217.1722; 218.1193; 221.2915; 223.7566; 231.0708; 260.3325; 276.9559; 284.2830; 294.5392; 302.3154; 303.6806; 306.9753; 308.4348; 320.3469; 323.7738; 325.1246; 337.7685; 348.4825; 350.9574; 353.6039; 359.2312; 393.4346; 399.4257; 420.6212; 452.2129; 481.0099; 489.1524; 489.9295; 490.6210; 492.0601; 523.0068; 525.6874; 530.7749; 557.2239; 557.5661; 557.9668; 569.8469; 571.1293; 575.5562; 577.5472; 609.6788; 618.5690; 623.8793; 628.8065; 629.6699; 632.8631; 638.1209; 716.5157; 723.7228; 734.5221; 757.3734; 760.0812; 761.0419; 761.1658; 787.8071; 858.6639; 869.5209; 928.7200; 935.5215; 940.5859; 956.0919; 964.9927; 972.6341; 976.8119; 982.1219; 986.1925; 988.6062; 995.6220; 1007.8701; 1013.9786; 1048.9801; 1058.4203; 1068.6227; 1072.8052; 1074.5325; 1091.4180; 1103.6387; 1112.3559; 1129.0493; 1186.1564; 1204.5381; 1206.3077; 1209.0686; 1216.3615; 1223.1759; 1224.0467; 1231.3844; 1237.3415; 1237.5080; 1239.4446; 1249.8882; 1253.1258; 1288.1415; 1298.8591; 1301.8291; 1307.4093; 1309.6279; 1321.8555; 1369.2204; 1377.2210; 1429.8675; 1437.4120; 1442.2190; 1461.7839; 1463.8031; 1502.8324; 1504.4065; 1511.0493; 1516.5858; 1518.6589; 1522.2752; 1532.0457; 1535.9674; 1557.1655; 1608.2306; 1614.1477; 1620.4602; 1678.2124; 1869.7122; 3038.6765; 3053.9640; 3057.5840; 3064.2571; 3094.4917; 3119.9161; 3121.3811; 3126.2796; 3129.3208; 3132.9292; 3150.5651; 3161.2223; 3177.6922; 3179.2695; 3184.5976; 3188.7719; 3195.3181.

Cartesian Forces: Max 0.000001239 RMS 0.000000302

Item	Value	Threshold	Converged?
Maximum Force	0.000001	0.000450	YES
RMS Force	0.000000	0.000300	YES
Maximum Displacement	0.000069	0.001800	YES
RMS Displacement	0.000008	0.001200	YES

FILE: Yb_Sq2_PhYbOTf3_TS_Anion_B3LYP_M062x_Add12_Conf7_Y.out

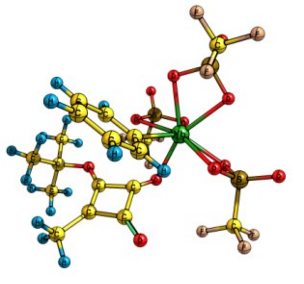

Conformer 14

E(RB3LYP/6-31G(d)/MWB59) = -3731.44876266
Convg = 0.7874D-08 -V/T = 2.0122

Temperature 195.150 Kelvin. Pressure 1.00000 Atm.
elec. and zero-point Energies= -3731.077421
elec. and thermal Energies= -3731.054307
elec. and thermal Enthalpies= -3731.053689
elec. and thermal Free Energies= -3731.122643

RB3LYP/6-31G(d)/MWB59/THF(IEFPCM) = -3731.51740803

RM06-2x/6-311+G(d,p)/MWB59/THF(IEFPCM) = -3731.35604577

Geometry at RB3LYP/6-31G(d)/MWB59
Charge = -1 Multiplicity = 1
Yb 0.345344342 1.961579463 1.4437342354
O 2.7202851197 2.2448272873 1.7018501928
S 3.2150336665 1.5977915825 0.4320062309
O 4.1963089973 2.3273418402 -0.362492146
O 2.0070176328 1.0506446545 -0.2654900941
C 4.1454532186 0.0995451971 1.0391126184
F 5.2309550941 0.4855190036 1.7199369983
F 3.3912930331 -0.6657715785 1.8307998492
F 4.547287837 -0.6404140448 -0.0127267681
O 0.5601536698 3.6886533036 -0.2681963051
O 1.1810689044 6.0382079804 0.4700124974
O 0.0980603104 4.3226750593 1.9990737958
S 0.3081134866 4.8811358392 0.6175770874
C -1.3830543432 5.4738043479 0.1103810668
F -1.3621206525 5.8688932272 -1.1687998773
F -2.2827292219 4.4941594369 0.2459336153
F -1.7504500946 6.5081511318 0.8757472238
C -0.1523163627 -1.8227636977 0.018394785
C -1.338087123 -2.5055556739 0.230120218
C -1.7082677239 -1.6456151022 1.3817323632
C -0.3651880472 -0.8995821475 1.186560052
O 0.4231547647 -0.2867803139 1.9233337115
O -2.6273746771 -1.634472683 2.1660377107
O 0.9208628654 -1.8042522048 -0.7308256323
C 1.1868405975 -2.6616150028 -1.8978586219
C -2.1318920797 -3.6416641381 -0.3121918088
H -1.5248580698 -4.5098409458 -0.5892855837
H -2.7012563002 -3.3332433266 -1.1994616434
H -2.8590093206 -3.9514009546 0.4467178416
C 1.4938654292 -4.0771531773 -1.4010585885
H 1.7589821542 -4.7205755008 -2.247645966
H 2.3362472409 -4.0610084733 -0.7022473901
H 0.6336339606 -4.5241488179 -0.8937911129
C 2.4325036466 -2.0064856245 -2.4999969793
H 2.7924328425 -2.5947387134 -3.3512429414
H 2.2040280823 -0.9918505811 -2.8379270973
H 3.2255367727 -1.9362892136 -1.7513851554
C 0.0163156041 -2.6125494151 -2.88324948
H -0.8501470199 -3.1769653103 -2.5318884328
H -0.2923030314 -1.577357843 -3.0559052787
H 0.331423438 -3.0474548019 -3.8384482945
C -1.3119725857 1.0443248637 -0.1916899455
C -0.9310609811 1.0009320789 -1.553779831
C -2.7015914565 1.0543550184 0.0622554685
H 0.1264231151 1.0090073226 -1.8106270875
H -3.0555549019 1.0883774529 1.0898650431
C -1.8680815724 0.980592549 -2.5918468455
C -3.6504630818 1.0257827617 -0.9669428966
H -1.5350706464 0.9749473204 -3.6295564884
H -4.7129529893 1.0385787247 -0.7281030992
C -3.2359035855 0.9843561619 -2.2993566812
H -3.9695614773 0.9654620595 -3.1035817581
O -1.3630215745 3.1869675582 5.0773440786
S -0.9016830609 2.1201215745 4.1969122782
C -1.3141875934 0.5582824301 5.1327610382
O 0.5687505944 2.0286033786 3.9006178376
O -1.6030221928 1.9673063955 2.8670981389
F -0.8878074289 -0.5346600076 4.5003513794
F -2.6394333253 0.4778026069 5.2991750347
F -0.7306236686 0.6078960183 6.3403734437

Frequency: -121.9438; 14.6852; 20.8317; 23.9964; 26.2932; 28.2730; 32.2212; 35.0150; 38.9429; 43.5060; 47.2538; 48.8718; 53.6318; 60.7529; 62.1739; 64.6488; 70.3580; 73.0984; 76.8532; 82.3424; 89.3991; 93.1636; 96.8540; 113.2173; 126.3434; 136.0529; 137.3332; 140.0307; 145.3955; 151.9372; 157.0203; 157.9672; 168.8032; 182.6093; 192.1722; 202.1348; 205.8602; 210.9491; 214.8470; 215.3458; 219.8432; 225.2911; 226.1391; 241.3151; 252.8513; 263.2396; 294.1859; 303.1731; 305.5716; 306.8919; 309.0101; 320.5003; 323.5189; 324.7794; 336.0670; 349.7870; 351.0102; 352.2588; 358.5633; 396.7918; 400.5184; 421.8618; 453.6130; 482.8363; 485.6690; 489.6083; 490.4937; 490.8936; 522.6088; 524.7374; 530.2005; 557.4831; 557.6935; 558.1582; 569.5021; 570.7329; 577.9830; 593.9724; 607.2279; 618.2751; 624.1376; 629.9573; 631.7416; 637.8367; 647.9189; 716.9101; 730.3941; 757.8121; 760.9377; 761.0654; 761.7267; 765.2338; 782.8928; 850.6297; 871.7926; 927.7265; 934.6879; 938.2026; 972.3530; 973.6972; 976.8488; 982.4271; 985.5766; 987.5861; 991.5370; 993.6664; 1011.0104; 1012.9644; 1052.8427; 1061.3162; 1067.0133; 1074.5763; 1086.7629; 1092.9771; 1109.1489; 1113.8765; 1131.5846; 1184.7591; 1197.2252; 1201.9204; 1209.9370; 1216.0586; 1216.3301; 1223.9395; 1226.1502; 1230.5296; 1236.5057; 1237.9552; 1243.0347; 1250.1427; 1282.2644; 1294.4727; 1300.1525; 1305.6511; 1307.4318; 1320.1173; 1369.4014; 1383.9588; 1429.4943; 1436.3004; 1437.9059; 1459.9871; 1460.3567; 1501.1593; 1507.4890; 1514.2091; 1515.9408; 1517.2754; 1520.6995; 1527.1681; 1532.0874; 1548.1178; 1612.0385; 1618.3600; 1635.6748; 1712.3362; 1874.8149; 3038.9920; 3060.0480; 3065.3742; 3072.4676; 3097.3539; 3122.6240; 3128.7429; 3135.6269; 3142.0108; 3143.1077; 3143.7165; 3156.7526; 3162.0674; 3164.4810; 3175.3601; 3182.4674; 3194.1819.

Cartesian Forces: Max 0.000010276 RMS 0.000001146

Item	Value	Threshold	Converged?
Maximum Force	0.000002	0.000450	YES
RMS Force	0.000000	0.000300	YES
Maximum Displacement	0.001037	0.001800	YES
RMS Displacement	0.000203	0.001200	YES

FILE: Yb_Sq2_PhYbOTf3_TS_Anion_B3LYP_M062x_Add12_Conf7-1_YY.out

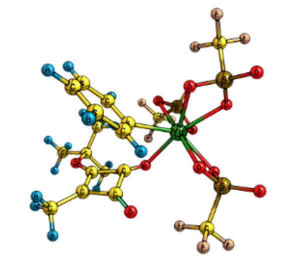

Conformer 15

E(RB3LYP/6-31G(d)/MWB59) = -3731.44324936
Convg = 0.8818D-08 -V/T = 2.0122

Temperature 195.150 Kelvin. Pressure 1.00000 Atm.
elec. and zero-point Energies= -3731.071872
elec. and thermal Energies= -3731.048865
elec. and thermal Enthalpies= -3731.048247
elec. and thermal Free Energies= -3731.116364

RB3LYP/6-31G(d)/MWB59/THF(IEFPCM) = -3731.50960424

RM06-2x/6-311+G(d,p)/MWB59/THF(IEFPCM) = -3731.34763072

Geometry at RB3LYP/6-31G(d)/MWB59
Charge = -1 Multiplicity = 1
Yb 0.3241006885 2.4609523094 1.2144192813
O 2.6787504065 2.7943221188 2.1002384198
S 3.3240356215 2.9013037875 0.7582761708
O 4.084914488 4.100448421 0.4273871598
O 2.2796999285 2.4585107155 -0.2468301179
C 4.617978937 1.5573057718 0.7543279202
F 5.6501189713 1.9330248525 1.5164948372
F 4.1338651997 0.404565263 1.2397200115
F 5.0625291316 1.3400450941 -0.4939745339
O 0.5890134604 4.8387731263 1.6536486641
O -1.3181262125 6.3588400945 0.9429209382
O -0.7725716354 4.1483000427 -0.1891086862
S -0.3179589899 5.366402878 0.5699884399
C 0.8180519644 6.2557519744 -0.6109347398
F 1.4327534026 7.260680323 0.0228694868
F 1.7361446181 5.4255103283 -1.1079468835
F 0.0984157851 6.7598905683 -1.6234285588
C 0.0183480169 -1.7629268998 0.0719741257
C -1.2959377534 -2.1086534002 0.28524729
C -1.5420367732 -0.9776883264 1.1955702027
C -0.0736755763 -0.5219630896 0.9509457113
O 0.7599592428 0.1692003411 1.5678595871
O -2.4564652666 -0.6312863711 1.9041898202
O 0.8877520663 -2.4321200789 -0.6622659723
C 2.356524711 -2.2663230116 -0.5804760538
C -2.1571573011 -3.2204076341 -0.1843954779
H -1.5637941957 -4.0452806919 -0.5916175088
H -2.833722668 -2.862685996 -0.9725307128
H -2.7860389251 -3.5924349081 0.6326511646
C 2.7457772043 -0.9446046039 -1.233112221
H 3.8350707562 -0.8478061138 -1.2622514113
H 2.3646186231 -0.9039879918 -2.2582138021
H 2.3514063214 -0.0914128213 -0.6812055317
C 2.8618336062 -3.4554604342 -1.4011766397
H 3.9561525899 -3.4433872513 -1.4375074305
H 2.5382389166 -4.4018861128 -0.9551756846
H 2.4800845599 -3.4057418621 -2.4260606386
C 2.8004222424 -2.3772828752 0.8794332863
H 2.4165408815 -1.5497256798 1.4797693488
H 2.4614938848 -3.3263234739 1.3103059058
H 3.8936024535 -2.3466815167 0.9327018185
C -0.8895826434 1.2661408471 -0.6582846067
C -0.2375509468 1.1071234335 -1.9009502848
C -2.3026762457 1.2797134278 -0.7043184115
H 0.8496430396 1.1212760698 -1.9447523629
H -2.8656091803 1.4059112943 0.2178644695
C -0.9353794655 0.9710429332 -3.1069942514
C -3.0143216663 1.1442460028 -1.8992646506
H -0.3905205997 0.8616146945 -4.0441236975
H -4.1031029681 1.1731708843 -1.8906392925
C -2.3309893642 0.9808606444 -3.1082427547
H -2.8815172492 0.8720230825 -4.0411517008
O -1.8033422769 3.6457643166 4.6244277703
S -1.2163354831 2.590214266 3.8091735719
C -1.6875064447 1.0125366618 4.6939860138
O 0.2780776041 2.5178588491 3.6887313799
O -1.7404490823 2.4486727116 2.3952688258
F -0.9785971004 -0.0290357347 4.2502558938
F -2.9892228896 0.7656701505 4.5420759189
F -1.4273334204 1.1671895991 6.0020451842

Frequency: -125.0759; 15.9109; 20.3728; 22.7463; 30.2831; 36.6249; 38.4141; 42.2777; 45.5005; 47.9393; 52.1343; 56.8323; 59.0594; 61.8210; 68.8737; 71.0347; 72.8047; 77.5695; 82.1080; 83.9024; 88.7997; 93.1775; 100.2280; 104.5587; 106.8189; 124.4567; 127.7631; 131.1288; 138.3538; 143.7584; 149.0379; 157.3242; 163.8646; 179.0985; 188.2770; 196.8986; 209.6497; 213.1424; 214.4277; 218.3881; 219.5620; 224.7516; 230.3817; 254.6915; 282.7185; 291.7071; 299.7490; 303.9492; 307.6123; 308.5815; 315.6299; 320.5368; 322.8407; 323.5555; 336.1550; 349.9268; 350.4793; 353.1690; 359.2223; 395.9795; 401.3491; 420.6133; 458.6920; 481.9314; 487.7319; 489.1061; 491.0411; 492.8570; 522.9208; 525.3090; 531.2343; 556.5993; 557.0831; 557.8936; 567.6153; 571.1664; 577.6070; 585.8520; 601.5974; 621.1652; 626.4818; 627.5865; 630.6291; 634.1968; 639.8578; 715.6980; 723.6449; 734.8305; 754.2797; 757.8509; 760.9324; 761.7618; 789.1732; 864.8592; 867.7641; 922.5141; 937.0890; 941.2197; 960.0999; 969.2910; 971.4963; 974.0306; 978.9801; 982.4180; 986.9291; 992.1445; 1008.1335; 1013.9153; 1053.0119; 1058.4494; 1068.3280; 1073.5314; 1076.4903; 1089.7489; 1101.0829; 1108.3301; 1133.7182; 1184.8838; 1205.8252; 1207.4222; 1212.7061; 1214.6406; 1220.4026; 1223.9300; 1229.2380; 1233.6876; 1234.3148; 1247.6951; 1250.5208; 1252.3533; 1292.5259; 1298.1421; 1299.5010; 1305.4169; 1309.8935; 1319.4724; 1369.6438; 1382.1446; 1430.2716; 1437.8360; 1438.9665; 1460.3581; 1461.8931; 1503.8371; 1504.1332; 1509.3164; 1516.3437; 1518.2881; 1520.0812; 1533.6454; 1535.6201; 1554.2705; 1612.8463; 1619.3179; 1624.0357; 1691.3910; 1875.4298; 3040.7469; 3056.6102; 3061.9102; 3077.7698; 3097.0708; 3124.8849; 3129.8032; 3131.9331; 3133.3056; 3144.0405; 3145.1018; 3156.1363; 3164.8859; 3173.9505; 3181.4330; 3188.3762; 3189.0846.

Cartesian Forces: Max 0.000000621 RMS 0.000000282

Item	Value	Threshold	Converged?
Maximum Force	0.000000	0.000450	YES
RMS Force	0.000000	0.000300	YES
Maximum Displacement	0.000019	0.001800	YES
RMS Displacement	0.000003	0.001200	YES

FILE: Yb_Sq2_PhYbOTf3_TS_Anion_B3LYP_M062x_Add12_Conf8_a.out

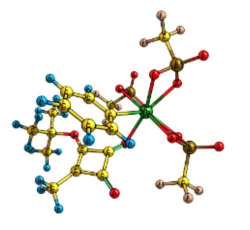

Conformer 16

E(RB3LYP/6-31G(d)/MWB59) = -3731.44612057
Convg = 0.7430D-08 -V/T = 2.0122

Temperature 195.150 Kelvin. Pressure 1.00000 Atm.
elec. and zero-point Energies= -3731.074910
elec. and thermal Energies= -3731.051730
elec. and thermal Enthalpies= -3731.051112
elec. and thermal Free Energies= -3731.120722

RB3LYP/6-31G(d)/MWB59/THF(IEFPCM) = -3731.51622739

RM06-2x/6-311+G(d,p)/MWB59/THF(IEFPCM) = -3731.35255911

Geometry at RB3LYP/6-31G(d)/MWB59
Charge = -1 Multiplicity = 1
Yb 0.215851803 2.3629404603 1.191535758
O 2.6095284479 2.1263003206 1.9645943364
S 3.1897620629 1.9738850405 0.5961048543
O 4.2933947044 2.8415483937 0.2002369808
O 2.0176483492 1.8797053092 -0.3552259896
C 3.9435663332 0.2685066541 0.5772012612
F 5.0135247048 0.2435004582 1.381902834
F 3.0812042095 -0.6671834199 0.9871267819
F 4.3406629035 -0.039271879 -0.6702846932
O 0.9794431977 4.6539532076 1.4376630232
O -0.5733188951 6.4954039615 0.6339944961
O -0.5604113563 4.1411895574 -0.3240328196
S 0.1735739336 5.2816202264 0.3272464563
C 1.4408021872 5.8115086708 -0.9330689661
F 2.2467388515 6.7376920311 -0.4003051275
F 2.1769994447 4.7760632062 -1.3393720013
F 0.8174667931 6.3418948277 -1.9948899932
C -0.2826919257 -1.7055157237 0.2374271217
C -1.5205438898 -2.287619526 0.4626589527
C -1.8907763971 -1.2759060716 1.4834527931
C -0.4950249376 -0.6403004072 1.2732844649
O 0.2581148496 0.1160130018 1.8869250847
O -2.851027657 -1.1106749989 2.1965458065
O 0.8149679039 -1.8347444363 -0.464763334
C 1.0817054408 -2.8798580881 -1.4718808698
C -2.3717300123 -3.4175334066 0.0044793696
H -1.8201285346 -4.3499886439 -0.1520342998
H -2.876540256 -3.161238944 -0.9364070044
H -3.1518258776 -3.5911176657 0.7538796245
C 1.2766762159 -4.2120599942 -0.742837075
H 1.5345178619 -4.9965503755 -1.4631391045
H 2.0883382009 -4.1323906152 -0.0126760518
H 0.3692298061 -4.5229614705 -0.2165298722
C 2.39244328 -2.3999738486 -2.0990049931
H 2.7503425151 -3.1372200894 -2.8257681002
H 2.2445295 -1.4438836372 -2.6095091645
H 3.1556931974 -2.2557032038 -1.3311359457
C -0.0340014893 -2.9228599192 -2.518885867
H -0.9428152617 -3.3976406764 -2.1440108575
H -0.2845759525 -1.910960628 -2.8501444541
H 0.3087697466 -3.5010312239 -3.3843840398
C -1.2300678532 1.2488656046 -0.50362897
C -0.7149924729 0.9490333332 -1.7872428799
C -2.6394602028 1.2146643357 -0.3940805039
H 0.362218711 0.9759562653 -1.9482954741
H -3.1051996529 1.4489633959 0.5615842984
C -1.5361321602 0.6463052213 -2.8800875412
C -3.475301034 0.8981463029 -1.471690933
H -1.0962925785 0.4537243837 -3.8592174848
H -4.5563823674 0.8862830171 -1.337854776
C -2.9250802859 0.6084945416 -2.7223053034
H -3.5694950387 0.3706680938 -3.567255929
O -1.7265637363 4.118524644 4.4925506656
S -1.2794056409 2.9336670279 3.7707752752
C -1.9726377381 1.5045976025 4.7511207107
O 0.1915195467 2.6643641701 3.6734322542
O -1.8255876652 2.7387521351 2.3708894669
F -1.4711446021 0.3398977874 4.3373554895
F -3.3038994103 1.4782164993 4.6342520408
F -1.6557657503 1.6685281395 6.0444091341

Frequency: -67.8727; 9.8357; 13.8349; 19.5062; 25.2179; 30.0439; 32.7811; 34.9120; 41.1158; 43.5170; 46.8790; 50.4684; 54.9663; 57.6147; 64.1782; 66.5263; 71.6513; 73.7901; 76.5983; 79.2603; 83.7230; 92.4039; 96.4675; 115.0232; 128.5316; 129.9271; 139.5027; 145.6424; 146.4588; 149.6126; 158.1116; 160.3155; 167.3893; 179.9750; 188.3582; 205.0357; 206.4396; 209.7024; 212.5911; 215.4086; 216.1661; 221.7796; 226.2105; 237.5174; 253.3455; 267.0457; 287.2489; 293.4769; 302.5598; 305.7811; 307.4526; 320.1980; 322.4707; 323.9505; 335.9683; 349.5798; 350.6850; 352.7070; 358.2083; 396.6242; 397.6627; 426.3093; 454.2596; 482.0970; 484.4753; 489.1361; 489.7910; 491.5043; 523.5031; 524.9672; 530.6378; 557.0767; 557.5442; 558.0744; 568.7586; 571.5125; 577.4998; 586.0812; 601.7016; 620.7913; 626.0595; 631.7520; 633.4737; 639.5184; 647.1290; 717.3307; 733.5267; 758.8492; 759.5989; 761.2176; 761.7720; 765.0124; 782.7966; 848.8548; 871.7510; 917.8767; 935.2973; 938.8690; 971.9928; 974.0121; 976.1604; 980.4143; 982.2732; 983.5605; 987.4532; 993.6996; 1011.2242; 1017.2841; 1050.8196; 1060.4057; 1067.3758; 1076.4639; 1084.5338; 1097.5703; 1104.2125; 1113.8991; 1136.4223; 1183.0292; 1199.0312; 1206.4268; 1211.3436; 1213.3122; 1215.2603; 1223.6864; 1225.8603; 1232.1954; 1233.4366; 1235.6507; 1247.1641; 1248.1627; 1282.5862; 1294.5403; 1296.1606; 1304.9780; 1308.3779; 1319.0295; 1368.9868; 1383.8215; 1429.9332; 1435.6317; 1437.1667; 1458.0521; 1459.8061; 1501.5354; 1507.3922; 1512.9702; 1516.0880; 1517.9025; 1521.2276; 1527.2350; 1532.8210; 1548.7768; 1610.3318; 1618.0948; 1642.2622; 1750.0779; 1879.6185; 3043.0836; 3060.8079; 3066.4637; 3073.4981; 3102.3451; 3125.8343; 3129.7454; 3133.6452; 3136.6794; 3141.5091; 3143.1774; 3150.2438; 3162.7071; 3165.4048; 3169.2250; 3171.4810; 3180.8275.

Cartesian Forces: Max 0.000000605 RMS 0.000000213

Item	Value	Threshold	Converged?
Maximum Force	0.000001	0.000450	YES
RMS Force	0.000000	0.000300	YES
Maximum Displacement	0.000101	0.001800	YES
RMS Displacement	0.000022	0.001200	YES

FILE: Yb_Sq2_PhYbOTf3_TS_Anion_B3LYP_M062x_Add12_Conf8-1.out

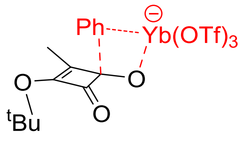

Trapezoidal Transition State for $[PhYb(OTf)_3]^-$ addition to C-2 carbonyl of 1b

Lowest energy conformation is:
Conformer 15

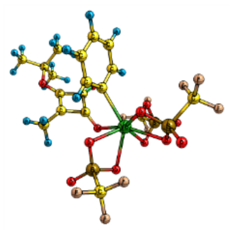

Conformer 1

E(RB3LYP/6-31G(d)/MWB59) = -3731.45905180
Convg = 0.4006D-08 -V/T = 2.0122

Temperature 195.150 Kelvin. Pressure 1.00000 Atm.
elec. and zero-point Energies= -3731.087554
elec. and thermal Energies= -3731.064513
elec. and thermal Enthalpies= -3731.063895
elec. and thermal Free Energies= -3731.133418

RB3LYP/6-31G(d)/MWB59/THF(IEFPCM) = -3731.52287791

RM06-2x/6-311+G(d,p)/MWB59/THF(IEFPCM) = -3731.35865975

Geometry at RB3LYP/6-31G(d)/MWB59
Charge = -1 Multiplicity = 1
Yb 0.6448042197 2.2081922113 1.2940172427
O 2.2233074779 1.0317361059 -0.2408881818
S 3.3794226632 0.9827617141 0.7213388694
O 4.0157655993 -0.3170359894 0.9297167078
O 2.9554990637 1.761296872 1.9344698856
C 4.6891064218 2.040545926 -0.0747370502
F 5.0656715051 1.487124242 -1.2360077916
F 4.224517344 3.2679694691 -0.3093765263
F 5.7551595756 2.1216085877 0.7301365145
O 0.7810258018 3.7880589618 -0.5533391589
O 2.3463568914 5.77582659 -0.3441189474
O 1.3645692136 4.5459862082 1.647444813
S 1.2486704386 4.9969814682 0.2177832252
C -0.2178766528 6.1461493809 0.1931333449
F -0.4261792453 6.5779593691 -1.058184824
F -1.3171426688 5.5237297816 0.6229776811
F 0.022581069 7.2017820386 0.9790170641
C -0.1010534345 -1.6195096788 0.2213208278
C -1.389413301 -2.0999083001 0.2640842938
C -1.8419734404 -1.0458910513 1.2442535815
C -0.4031193383 -0.4987606723 1.1433005323
O 0.3243075099 0.1012246942 1.9823367738
O -2.8319705198 -0.8189453426 1.8988758999
O -1.8920274584 -3.16298271 -0.3495344227
F -3.0897802125 2.161724467 4.6864248402
C -0.5736783556 1.0262164966 -1.7007697291
C -2.3839122906 1.5983046714 -0.2369125076
H 0.4714905564 0.7808183424 -1.875467788
H -2.7872844258 1.78613298 0.7547398601
C -1.4203315171 1.1388296017 -2.8061737021
C -3.2431241224 1.7164680836 -1.3363454955
H -1.0323464687 0.9699505287 -3.809799465
H -4.2848593686 1.9952300945 -1.1844096372
C -2.7644765381 1.4798149021 -2.6261869357
H -3.4281342756 1.569697622 -3.4843933235
O -1.0636359485 4.3814197581 4.4640350436
S -0.8078835531 3.1176160085 3.7833202013
C -1.7861227386 1.8616999954 4.7546891129
O 0.5938303535 2.5895086352 3.752176938
O -1.3363720783 2.9819804511 2.368909218
F -1.5994661737 0.6275374498 4.2898766009
C -1.0228053669 1.2523575872 -0.3805362556
F -1.4016549529 1.9027248291 6.0387022423
C -3.3244123522 -3.5091650458 -0.2688576123
C 1.141291667 -2.0686186958 -0.4486298587
H 2.0018917572 -1.9768951115 0.222181419
H 1.0481365153 -3.101190855 -0.8001814596
H 1.3546832566 -1.4210855379 -1.3076329655
C -4.1713591421 -2.3346943064 -0.767119517
H -3.8294430819 -2.0028205682 -1.7528530637
H -5.2177408977 -2.6502809582 -0.8523588112
H -4.1289253281 -1.4887796829 -0.0774112985
C -3.6721274808 -3.9102841561 1.1677514346
H -4.7017581574 -4.2846442171 1.2064286811
H -3.0051834338 -4.7071048877 1.5150617771
H -3.5966474642 -3.0541569291 1.8426877339
C -3.4248903978 -4.7054707446 -1.2180736676
H -3.1330612898 -4.4171951041 -2.233105826
H -2.7682708069 -5.5167281816 -0.8866416841
H -4.4539850396 -5.0789564534 -1.2439990368

Frequency: -200.8194; 8.5681; 19.0977; 21.3001; 24.6850; 29.0898; 30.5624; 34.3222; 35.5342; 37.7675; 42.5452; 44.9509; 50.8041; 57.6010; 61.4453; 66.1284; 71.0486; 73.6994; 79.2285; 80.9794; 89.5245; 99.5464; 113.6286; 119.1075; 123.6613; 128.3082; 137.3654; 141.9419; 144.1449; 152.1951; 156.6658; 162.0544; 171.9630; 181.0645; 190.5828; 207.0105; 208.7939; 212.7259; 215.5713; 217.1773; 218.4457; 220.0505; 226.3614; 259.1976; 269.3060; 275.8292; 298.2511; 304.5318; 307.3852; 310.4666; 320.1075; 324.2588; 325.1289; 336.6871; 347.2815; 351.8299; 352.9594; 356.3550; 364.6452; 397.1478; 402.1813; 430.1516; 454.2105; 489.0204; 490.0107; 490.2287; 491.1813; 500.9658; 522.9699; 525.4983; 529.3219; 557.4978; 558.0960; 558.6430; 562.7877; 571.3924; 573.1459; 575.7073; 599.1302; 624.1424; 626.5315; 629.5450; 630.4627; 634.6059; 644.8695; 718.1125; 720.7297; 738.2838; 761.4572; 761.7075; 762.1270; 763.3934; 787.6947; 863.9100; 868.2228; 927.2028; 934.4855; 940.9690; 967.8301; 972.4771; 977.3464; 983.4814; 983.8307; 985.0193; 990.7061; 995.8779; 1008.6062; 1014.3213; 1053.5279; 1065.9233; 1070.9732; 1073.3320; 1088.2969; 1093.5569; 1108.0903; 1109.9576; 1135.4938; 1185.5360; 1212.0099; 1213.6693; 1214.3873; 1215.4852; 1223.1916; 1229.3521; 1230.8952; 1234.9346; 1236.4119; 1245.6009; 1247.8364; 1251.4231; 1283.0847; 1294.7131; 1300.4769; 1301.9423; 1306.7357; 1313.5636; 1367.2969; 1381.1845; 1429.7142; 1432.7549; 1439.5734; 1458.8549; 1462.2540; 1496.7350; 1503.7357; 1506.3369; 1515.4615; 1516.5759; 1519.7404; 1534.7117; 1538.4834; 1554.5050; 1590.0158; 1613.6916; 1620.6790; 1695.4740; 1852.5152; 3053.9716; 3054.7988; 3057.1217; 3064.2678; 3116.1277; 3118.4085; 3125.1063; 3132.3531; 3135.1138; 3138.4622; 3149.1780; 3156.5004; 3159.9418; 3163.9217; 3177.9937; 3188.1066; 3197.9889.

Cartesian Forces: Max 0.000000716 RMS 0.000000326

Item	Value	Threshold	Converged?
Maximum Force	0.000000	0.000450	YES
RMS Force	0.000000	0.000300	YES
Maximum Displacement	0.000186	0.001800	YES
RMS Displacement	0.000027	0.001200	YES

FILE: Yb_Sq2_PhYbOTf3_TS_Anion_B3LYP_M062x_Add56_Conf1.out

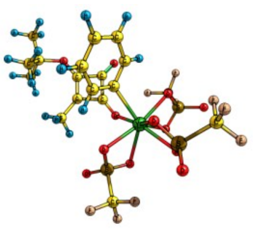

Conformer 2

E(RB3LYP/6-31G(d)/MWB59) = -3731.44925325
Convg = 0.6355D-08 -V/T = 2.0122

Temperature 195.150 Kelvin. Pressure 1.00000 Atm.
elec. and zero-point Energies= -3731.077966
elec. and thermal Energies= -3731.054868
elec. and thermal Enthalpies= -3731.054250
elec. and thermal Free Energies= -3731.123768

RB3LYP/6-31G(d)/MWB59/THF(IEFPCM) = -3731.51488719

RM06-2x/6-311+G(d,p)/MWB59/THF(IEFPCM) = -3731.35245066

Geometry at RB3LYP/6-31G(d)/MWB59
Charge = -1 Multiplicity = 1
Yb 0.6836650842 2.2794522663 1.2441481587
O 2.4294057885 1.4851301385 -0.2559056566
S 3.5497465998 1.3626846888 0.7516455163
O 4.2538287323 0.0802691211 0.7883956339
O 3.0289626636 1.9207735008 2.0380293062
C 4.8232313936 2.587136888 0.1611454915
F 5.2991778047 2.1960253257 -1.0293434433
F 4.292769527 3.8021208643 0.0387698968
F 5.8362099319 2.6350996122 1.0354606655
O 0.3425912815 3.9087843025 -0.5642234106
O 2.0024458009 5.8247705162 -0.7037694931
O 1.4661352556 4.5856396266 1.4449857019
S 1.0315562125 5.0773550683 0.0882002227
C -0.3345515872 6.2957761643 0.438746274
F -0.8252751399 6.7579967202 -0.7196731499
F -1.3213494663 5.7245374138 1.1287572101
F 0.1525026099 7.3254895197 1.1415926672
C 0.2463846981 -1.6516110641 0.254776248
C -1.01988174 -2.1978713313 0.3205408323
C -1.5211426524 -1.1675603539 1.2962242612
C -0.1207310018 -0.5316813444 1.1652237315
O 0.5963885001 0.1465444986 1.9485791678
O -2.5192043913 -1.0205393156 1.9520565059
O -1.756167378 -3.2019262114 -0.1300651289
F -3.0572541189 1.9494186557 4.5942865309
C -0.334866634 0.89953126 -1.7299272251
C -2.2459516503 1.3072140096 -0.3431088585
H 0.738493882 0.7837814637 -1.8671425229
H -2.7013368534 1.4854771502 0.6278429067
C -1.1529413138 0.8614336802 -2.8623914595
C -3.0777882565 1.2714061704 -1.4684338565
H -0.7146842994 0.7067592879 -3.8477420422
H -4.150141835 1.4236458708 -1.3563677086
C -2.5341593687 1.0398473261 -2.7331268449
H -3.1766815511 1.0110567504 -3.6114584352
O -1.2623185958 4.3708954854 4.337770615
S -0.8792247437 3.1216877846 3.6905073486
C -1.730855045 1.7949018998 4.6857680552
O 0.5696461506 2.7377503078 3.6795495392
O -1.3751978234 2.9025182144 2.275434169
F -1.4014523654 0.5783772027 4.256161484
C -0.8499494284 1.1212817481 -0.4327410035
F -1.370590433 1.9122916847 5.9720580502
C -1.3712554858 -4.1191727813 -1.210047628
C 1.57304041 -1.9007269911 -0.3668060955
H 2.3675623225 -1.5430701003 0.2958377648
H 1.7530612088 -2.9538425666 -0.5986790398
H 1.6670509462 -1.3222909886 -1.2949062439
C -0.3443343581 -5.1212243926 -0.6731093655
H -0.7532901974 -5.6591435953 0.1884715995
H -0.0912222251 -5.8526441467 -1.4490710276
H 0.5792012183 -4.627883114 -0.3604868981
C -0.8730600166 -3.3393112307 -2.4307914055
H -0.790062361 -4.0194998596 -3.2860875999
H -1.5762253922 -2.541115189 -2.6885541917
H 0.1071085093 -2.8880339473 -2.268048586
C -2.6943558612 -4.8232747348 -1.5281603132
H -3.0953286221 -5.3053264655 -0.6310008112
H -3.4334217722 -4.1022724979 -1.8907354359
H -2.5415037563 -5.5870543112 -2.2982440046

Frequency: -193.2231; 11.6321; 17.1077; 21.9274; 26.6270; 27.9397; 28.7207; 32.7048; 35.8225; 37.5660; 42.9635; 47.7812; 54.3483; 55.8544; 59.5549; 61.7914; 68.2460; 76.6043; 78.6977; 84.5333; 86.7695; 100.9559; 111.6224; 118.4189; 124.2651; 136.0223; 138.0348; 144.4715; 147.8042; 150.2448; 156.6601; 161.7028; 169.0590; 180.6120; 191.3601; 195.1835; 210.1410; 211.4965; 213.5118; 215.7165; 219.2599; 220.6410; 222.9420; 236.5660; 261.9894; 270.9167; 287.9651; 303.5375; 307.1092; 309.1995; 320.9740; 322.9002; 324.1632; 334.7199; 345.3179; 351.8851; 353.0335; 356.8432; 366.2151; 398.6279; 402.5879; 429.6582; 451.0892; 484.0938; 489.2346; 489.7639; 491.2811; 500.8525; 522.9523; 525.7503; 529.4483; 557.6712; 558.0655; 558.8112; 564.2051; 571.5294; 573.4549; 574.7252; 597.9263; 625.5582; 626.9103; 629.8408; 630.7144; 634.8108; 650.5856; 719.1487; 727.0037; 760.8876; 761.9872; 762.2810; 762.6067; 767.6310; 777.6445; 849.0896; 867.9702; 925.4853; 931.1153; 935.0509; 973.1961; 973.9032; 976.1152; 979.5042; 982.3138; 983.9885; 989.6926; 1000.0156; 1008.9985; 1014.0606; 1046.8034; 1060.7365; 1063.5129; 1073.3243; 1087.0698; 1093.1603; 1105.8350; 1111.6017; 1135.8056; 1185.1109; 1187.9527; 1211.8006; 1212.5354; 1213.4991; 1215.3734; 1222.5874; 1232.1649; 1233.4018; 1235.3908; 1250.3696; 1252.4382; 1253.0364; 1281.0163; 1291.9317; 1292.7701; 1301.0008; 1305.9427; 1312.4642; 1366.1049; 1368.9646; 1429.2420; 1432.9952; 1439.0135; 1457.8992; 1461.9636; 1498.2832; 1505.0676; 1511.1022; 1515.3269; 1516.3090; 1520.6260; 1528.1917; 1532.2032; 1548.6961; 1586.6768; 1613.4521; 1619.9731; 1699.1633; 1883.5451; 3053.6296; 3058.8756; 3062.3191; 3070.0094; 3114.7357; 3125.6218; 3130.5582; 3136.7835; 3140.5115; 3142.7342; 3146.8061; 3155.5089; 3159.3400; 3172.5815; 3175.1323; 3182.1789; 3194.9016.

Cartesian Forces: Max 0.000000843 RMS 0.000000351

Item	Value	Threshold	Converged?
Maximum Force	0.000000	0.000450	YES
RMS Force	0.000000	0.000300	YES
Maximum Displacement	0.000043	0.001800	YES
RMS Displacement	0.000007	0.001200	YES

FILE: Yb_Sq2_PhYbOTf3_TS_Anion_B3LYP_M062x_Add56_Conf1-1.out

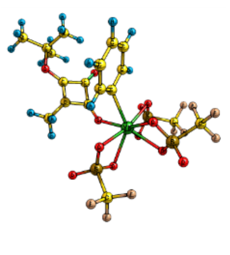

Conformer 3

E(RB3LYP/6-31G(d)/MWB59) = -3731.45828682
Convg = 0.2091D-08 -V/T = 2.0122

Temperature 195.150 Kelvin. Pressure 1.00000 Atm.
elec. and zero-point Energies= -3731.086793
elec. and thermal Energies= -3731.063786
elec. and thermal Enthalpies= -3731.063168
elec. and thermal Free Energies= -3731.131998

RB3LYP/6-31G(d)/MWB59/THF(IEFPCM) = -3731.52167168

RM06-2x/6-311+G(d,p)/MWB59/THF(IEFPCM) = -3731.35733427

Geometry at RB3LYP/6-31G(d)/MWB59
Charge = -1 Multiplicity = 1
Yb 0.7980465042 2.2382081861 1.1815547127
O 2.429612379 1.0320324409 -0.1778164291
S 3.4863709404 0.833958097 0.8837105655
O 3.9847375858 -0.5288828114 1.0658257953
O 3.0185735167 1.5842546928 2.0919518526
C 4.9488307892 1.7965037737 0.2481151958
F 5.3977219209 1.2329344006 -0.8818692208
F 4.6104380172 3.0607525419 -0.0025118256
F 5.9295012361 1.7766884295 1.1591117205
O 0.8739091181 3.7882328124 -0.7328578965
O 2.7811450694 5.4659217132 -0.7885278647
O 1.8995327082 4.4142492793 1.3439526845
S 1.6544734422 4.8872839843 -0.0651154212
C 0.4337256362 6.2876971916 0.1022803368
F 0.1930106576 6.8143921791 -1.1055606794
F -0.7203746405 5.86253303 0.6219899703
F 0.9429665339 7.2395372928 0.8940665286
C -0.1204265185 -1.6006922578 0.3094805964
C -1.4330481245 -2.0133477547 0.3017452437
C -1.8858549898 -0.875368123 1.1844929596
C -0.4159523228 -0.4114744348 1.1412753673
O 0.2893668675 0.2099214789 1.9829511805
O -2.898676843 -0.5532628701 1.7576900424
O -1.9548828072 -3.0867658878 -0.2757626302
F -0.4451384702 5.0800715766 5.3585314155
C -0.3073642672 0.9202725356 -1.8032898041
C -2.1647138296 1.689033785 -0.4969626348
H 0.7324155595 0.6134128297 -1.8965703659
H -2.6119668726 1.9719263947 0.4527728515
C -1.0826118487 0.9895767247 -2.963339736
C -2.9526850066 1.7646652712 -1.6520039964
H -0.6478095285 0.7269097695 -3.9268710185
H -3.9852820922 2.1042073011 -1.5843087559
C -2.4150512308 1.4075323008 -2.8899707433
H -3.0234859042 1.4637609842 -3.7909595713
O -1.773350948 2.5960437398 4.4774868634
S -0.8087953019 3.1948656826 3.5628406237
C -0.7661289953 4.9927280354 4.0607004251
O -1.181128694 3.2206945512 2.0938921995
O 0.6135308956 2.7282165492 3.6176143643
F 0.1261029616 5.6849673272 3.3514782626
C -0.8182644377 1.2661813067 -0.5325090821
F -1.9781468822 5.5340875309 3.8790035746
C -3.4065601342 -3.3546144497 -0.2614235386
C -4.1579901802 -2.1738384317 -0.8827773999
H -5.2124532304 -2.4424357869 -1.0160109147
H -4.1130548784 -1.2893018975 -0.2435193809
H -3.7391694081 -1.9220236123 -1.8625437577
C -3.5162196794 -4.6038240813 -1.1387080191
H -3.1511858872 -4.3975075101 -2.1500368882
H -2.9245500591 -5.423568473 -0.7178837005
H -4.5607462242 -4.9261370571 -1.2040701251
C -3.8579462599 -3.6425701683 1.1733995967
H -4.9070817434 -3.960520166 1.172098326
H -3.2569552803 -4.4479167508 1.6099410575
H -3.7751249828 -2.7491661852 1.7972473606
C 1.1332981679 -2.1458351286 -0.2605294118
H 1.9621150644 -2.0493434846 0.448367882
H 1.0106950751 -3.1934863738 -0.5534424748
H 1.4198338572 -1.5657750239 -1.146542268

Frequency: -199.3303; 15.3366; 19.1479; 23.9084; 25.7601; 30.4442; 32.3493; 35.5477; 36.0539; 39.5848; 44.1139; 49.9544; 56.1590; 59.3429; 63.7236; 68.9373; 72.8013; 74.2037; 78.9271; 82.6507; 88.4204; 100.2615; 112.9698; 116.0837; 120.6971; 124.8691; 132.7506; 141.8938; 143.1293; 151.4067; 153.4362; 159.0072; 168.9095; 181.8112; 191.1462; 207.1089; 210.5556; 212.0434; 215.4798; 216.0113; 217.8222; 220.7860; 224.8379; 257.8009; 268.6451; 276.1892; 297.0919; 303.5200; 306.6809; 309.6666; 320.8820; 323.7139; 324.7556; 336.2704; 347.0469; 351.3742; 352.5854; 355.8636; 364.7415; 397.1658; 402.5331; 429.7258; 454.2598; 489.0103; 489.5832; 490.5251; 491.4257; 500.3647; 522.8960; 525.4152; 529.7740; 557.6240; 558.3487; 558.6556; 562.7962; 569.9846; 573.1132; 577.5857; 599.4901; 620.7496; 626.5872; 629.4834; 632.1296; 636.4060; 644.5693; 718.6438; 720.3299; 738.6535; 760.5676; 761.3052; 762.2392; 763.7656; 787.8046; 863.9446; 868.6115; 926.4702; 934.4181; 941.1311; 967.2821; 971.8459; 977.2645; 983.7972; 984.4573; 984.9604; 990.2576; 997.5483; 1008.7719; 1014.0878; 1052.2430; 1065.9299; 1070.7958; 1073.3955; 1088.3021; 1093.2826; 1109.7528; 1111.0835; 1135.6508; 1185.2930; 1212.2367; 1213.7014; 1213.9967; 1215.3765; 1223.0802; 1228.1364; 1229.8779; 1230.6423; 1235.4152; 1245.2843; 1248.7891; 1253.6536; 1283.3472; 1293.7492; 1300.6929; 1301.9929; 1305.1724; 1315.1717; 1367.6947; 1381.8766; 1429.6789; 1432.7851; 1439.2900; 1458.9319; 1462.2818; 1498.2792; 1503.6008; 1507.4619; 1515.4251; 1516.6156; 1519.7241; 1534.7259; 1538.5801; 1554.6201; 1591.5737; 1613.6109; 1620.5692; 1696.9493; 1856.3350; 3053.6529; 3054.0163; 3056.9449; 3064.1353; 3114.0856; 3118.4774; 3124.8952; 3132.2233; 3134.9180; 3139.6769; 3148.0895; 3156.6965; 3158.7369; 3163.8138; 3175.6642; 3183.8383; 3195.0600.

Cartesian Forces: Max 0.000001508 RMS 0.000000682

Item	Value	Threshold	Converged?
Maximum Force	0.000000	0.000450	YES
RMS Force	0.000000	0.000300	YES
Maximum Displacement	0.000042	0.001800	YES
RMS Displacement	0.000006	0.001200	YES

FILE: Yb_Sq2_PhYbOTf3_TS_Anion_B3LYP_M062x_Add56_Conf2.out

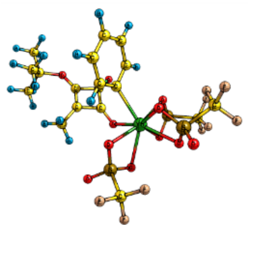

Conformer 4

E(RB3LYP/6-31G(d)/MWB59) = -3731.44815536
Convg = 0.4592D-09 -V/T = 2.0122

Temperature 195.150 Kelvin. Pressure 1.00000 Atm.
elec. and zero-point Energies= -3731.076841
elec. and thermal Energies= -3731.053789
elec. and thermal Enthalpies= -3731.053171
elec. and thermal Free Energies= -3731.122134

RB3LYP/6-31G(d)/MWB59/THF(IEFPCM) = -3731.51427648

RM06-2x/6-311+G(d,p)/MWB59/THF(IEFPCM) = -3731.35268783

Geometry at RB3LYP/6-31G(d)/MWB59
Charge = -1 Multiplicity = 1
Yb 0.8983855502 2.2878810969 1.1893614865
O 2.6594383241 1.2759441456 -0.1591876292
S 3.7155876567 1.1546784336 0.9150170051
O 4.331281229 -0.162460583 1.0781813603
O 3.1663559952 1.8343965129 2.1295981956
C 5.0957358534 2.253362772 0.3168569672
F 5.6071995028 1.7506524162 -0.8154690612
F 4.6525404839 3.4872405716 0.0823320929
F 6.062307559 2.3018745431 1.2420756069
O 0.8069971843 3.8261268104 -0.7375336564
O 2.5846492441 5.6382904009 -0.8426567255
O 1.822903038 4.5445616525 1.314242341
S 1.5166703949 4.9852358577 -0.0937814776
C 0.2004892463 6.2951574774 0.0853174339
F -0.0924061022 6.7975578765 -1.1214555357
F -0.9137066095 5.7911662535 0.6210485264
F 0.6501906519 7.2848586263 0.8667141966
C 0.2364722637 -1.6420708805 0.3711168886
C -1.0641575781 -2.1041020897 0.3793320874
C -1.5597961848 -0.9880815914 1.2599772804
C -0.1153052988 -0.4494240253 1.1924081269
O 0.5899275638 0.2188221084 1.996768731
O -2.5852568199 -0.739848436 1.836853449
O -1.8336333225 -3.087053664 -0.0639795416
F -0.5982648103 5.0587714551 5.3334981338
C -0.0548516072 0.8310375693 -1.7738722749
C -1.9956806241 1.4604431515 -0.515463766
H 1.0103515079 0.6197311124 -1.8445975656
H -2.4840254716 1.7197369294 0.4206195212
C -0.8120880291 0.8079306455 -2.9480419889
C -2.7662642957 1.4414584935 -1.6841044071
H -0.3374372977 0.5705247995 -3.8994844071
H -3.8263901558 1.6859674868 -1.6395537176
C -2.1778208958 1.1069807584 -2.9048074035
H -2.7730178419 1.089938741 -3.8161833774
O -1.7044244812 2.4708397689 4.4759454304
S -0.7891337925 3.1327517446 3.5548398541
C -0.8881801513 4.9343473836 4.0313600205
O -1.1576462763 3.1108878706 2.084736172
O 0.6661223369 2.7809956021 3.6165278882
F -0.0353127734 5.6823599531 3.3301372527
C -0.6176317186 1.1556439326 -0.5190063878
F -2.1326784404 5.3834654979 3.8218249068
C -1.4360703906 -4.0963134519 -1.0524773455
C -0.5128010673 -5.1185770468 -0.3819138822
H -0.2537108386 -5.9134233111 -1.0906459975
H 0.4161877319 -4.6602238262 -0.0344354052
H -1.0125305556 -5.5732765331 0.4797155411
C -2.7765753985 -4.7419743163 -1.4184443154
H -3.265935581 -5.1381859724 -0.5231525834
H -3.4431393378 -4.0050970727 -1.8769900752
H -2.6211062062 -5.5635093015 -2.1260672191
C -0.8095208673 -3.4315505828 -2.2823701911
H -0.706891265 -4.1730350649 -3.0828577707
H -1.4461233548 -2.6173252425 -2.6426428669
H 0.1813133445 -3.0222643451 -2.0783402276
C 1.5822100812 -2.0118596224 -0.1399131863
H 2.3519233411 -1.6779902945 0.5631154627
H 1.7023109052 -3.0848460716 -0.3110600556
H 1.7835984216 -1.4875582189 -1.0828651471

Frequency: -199.2069; 16.3494; 18.1400; 23.2090; 26.2274; 28.3306; 32.4113; 34.1146; 34.5504; 39.8843; 42.4549; 53.1424; 55.8972; 57.6974; 61.7545; 68.3863; 70.9076; 77.5611; 81.2329; 83.2177; 87.7077; 99.4916; 110.5801; 118.5915; 122.6799; 132.6116; 138.7367; 143.2821; 145.5206; 150.9616; 156.7299; 163.3606; 169.1541; 180.9083; 187.1455; 195.5071; 207.2768; 212.2140; 214.7940; 215.7093; 216.9296; 221.0708; 224.4533; 232.4535; 260.1864; 270.1645; 287.1783; 302.5898; 306.6590; 309.2405; 320.8991; 323.5503; 324.7300; 335.1066; 345.4444; 351.3383; 352.6561; 356.4915; 367.3572; 398.6355; 402.6802; 430.0686; 451.0649; 484.5681; 489.1788; 490.5450; 491.2808; 501.2680; 522.8883; 525.4364; 529.9689; 557.6787; 558.4109; 558.7795; 564.4364; 570.0340; 572.9753; 577.5472; 597.7144; 620.7694; 626.7309; 629.6407; 632.0881; 636.6448; 650.9368; 718.9670; 727.1818; 760.5119; 761.1261; 761.3911; 762.5222; 768.3175; 777.5107; 849.8416; 868.2071; 926.6129; 931.1702; 934.6993; 971.1512; 973.2296; 977.1366; 979.3948; 984.1138; 984.5612; 990.2681; 1000.7692; 1008.9851; 1013.9466; 1047.0750; 1060.8873; 1063.4017; 1073.2542; 1086.6714; 1093.1900; 1109.5060; 1110.9842; 1136.0523; 1185.1676; 1186.8460; 1212.0270; 1213.7367; 1213.8370; 1215.9163; 1222.6485; 1227.8673; 1229.9750; 1234.6490; 1245.8526; 1250.0359; 1253.8171; 1280.9117; 1292.5354; 1292.7559; 1301.5640; 1305.3867; 1315.6628; 1365.5460; 1368.3502; 1429.3429; 1432.7262; 1439.2689; 1457.7074; 1462.1829; 1498.2103; 1504.9724; 1510.6134; 1515.3469; 1516.3204; 1520.2924; 1527.8227; 1531.6409; 1548.4762; 1583.9409; 1613.5776; 1620.2447; 1697.8538; 1886.9792; 3054.2134; 3058.7278; 3062.1819; 3069.7655; 3115.3726; 3125.4926; 3130.1021; 3136.5283; 3140.4034; 3143.3215; 3146.8767; 3156.3344; 3159.6000; 3172.1818; 3175.2229; 3182.3074; 3195.3460.

Cartesian Forces: Max 0.000001198 RMS 0.000000419

Item	Value	Threshold	Converged?
Maximum Force	0.000000	0.000450	YES
RMS Force	0.000000	0.000300	YES
Maximum Displacement	0.000031	0.001800	YES
RMS Displacement	0.000006	0.001200	YES

FILE: Yb_Sq2_PhYbOTf3_TS_Anion_B3LYP_M062x_Add56_Conf2-1.out

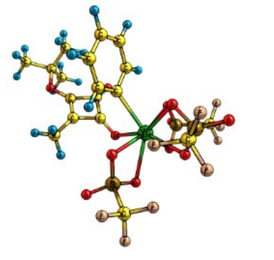

Conformer 5

E(RB3LYP/6-31G(d)/MWB59) = -3731.45819076
Convg = 0.4447D-08 -V/T = 2.0122

Temperature 195.150 Kelvin. Pressure 1.00000 Atm.
elec. and zero-point Energies= -3731.086781
elec. and thermal Energies= -3731.063727
elec. and thermal Enthalpies= -3731.063109
elec. and thermal Free Energies= -3731.132201

RB3LYP/6-31G(d)/MWB59/THF(IEFPCM) = -3731.52190874

RM06-2x/6-311+G(d,p)/MWB59/THF(IEFPCM) = -3731.35726436

Geometry at RB3LYP/6-31G(d)/MWB59
Charge = -1 Multiplicity = 1
Yb 0.4926721167 2.3952455118 1.2438254255
O 2.2292130446 1.4537204203 -0.150758983
S 3.2785332748 1.1855606361 0.9075421871
O 3.8094132527 -0.1774488927 0.9533482043
O 2.7783679696 1.7966683805 2.1737530345
C 4.7413571475 2.2185217343 0.3853159435
F 5.1538352137 1.8248728933 -0.8278149206
F 4.4390070872 3.5157102629 0.338310272
F 5.740255888 2.0389642792 1.2593199258
O 1.7347845396 4.4964845932 1.2130263491
O 0.5636200782 6.5662474564 0.3306269506
O -0.0630124412 4.1981184718 -0.3476523949
S 0.9755355046 5.1851352174 0.1082478236
C 2.1805746301 5.2780897313 -1.3123653075
F 3.2779436126 5.9452294796 -0.9343155545
F 2.5368554112 4.0574768838 -1.7237960784
F 1.6128030355 5.9267612019 -2.3373506507
C -0.1546920988 -1.5045258469 0.3030582313
C -1.4393253876 -1.9974776245 0.3167645302
C -1.9437025327 -0.8977901744 1.2197769077
C -0.5044492218 -0.3464144997 1.1565573286
O 0.178057256 0.3066034274 1.9925881143
O -2.9644550781 -0.6417203552 1.811792169
O -1.9053093594 -3.0949016325 -0.263281237
F -0.7693190843 5.281887373 5.2386448407
C -0.4437698488 1.0328682409 -1.7741268481
C -2.3846401475 1.6209075523 -0.4954927249
H 0.6196544571 0.8144749928 -1.8502618303
H -2.8706943673 1.855373342 0.4484460804
C -1.2008461402 1.0521546413 -2.949077574
C -3.1545266728 1.6423874286 -1.664002503
H -0.7274606174 0.8405607889 -3.9071035274
H -4.2134121566 1.8918030685 -1.6128437099
C -2.5653603742 1.3508074097 -2.8962161931
H -3.1600646415 1.3666789679 -3.807893921
O -1.9276850458 2.5522791424 4.6959196077
S -1.1276460269 3.2011169512 3.6648646723
C -1.2668929703 5.0242916109 4.0223724591
O -1.5967775135 3.0776776351 2.2330352927
O 0.3492427529 2.9269256213 3.6332479947
F -0.5958058624 5.7390227289 3.1179678294
C -1.006246454 1.3157361597 -0.5094850423
F -2.5551268634 5.3894031096 3.9969254054
C -3.3358349172 -3.4566697546 -0.2180029766
C -3.7340080234 -3.7846150037 1.2239872602
H -4.7595662648 -4.1713946469 1.2433922088
H -3.0712467339 -4.5516250528 1.6396562865
H -3.6962780417 -2.8924872239 1.8539770191
C -4.1764039432 -2.3222046655 -0.8107154599
H -4.1715346628 -1.4412666949 -0.1649481642
H -3.7992962389 -2.0375277098 -1.7983832393
H -5.2146516676 -2.6566696565 -0.9199939087
C -3.3848846949 -4.7034018675 -1.1042318409
H -4.407682812 -5.0920298738 -1.149263853
H -3.05718564 -4.4659131666 -2.1214908391
H -2.7321425872 -5.486502405 -0.7044509467
C 1.1163985901 -1.9606183528 -0.3051196567
H 1.9630334601 -1.7737885611 0.3629472321
H 1.070007819 -3.022636229 -0.5671805151
H 1.3120649923 -1.3871044558 -1.2203161853

Frequency: -198.0539; 14.4611; 17.2891; 22.6163; 26.0952; 30.6319; 32.6530; 34.2984; 37.3406; 37.8542; 43.5159; 52.4491; 54.5566; 57.4923; 65.4034; 66.8286; 71.3568; 72.4657; 79.8550; 81.4607; 87.6435; 98.3490; 110.7740; 114.6872; 118.8413; 124.8043; 130.9624; 141.9983; 142.9956; 150.7099; 153.6667; 157.1895; 169.9437; 181.3051; 190.9936; 207.7383; 210.1131; 211.8335; 214.4575; 215.7994; 217.0976; 220.7222; 223.8412; 257.6976; 268.4000; 275.6770; 296.6393; 303.5844; 306.6463; 309.1942; 320.2738; 322.5089; 325.4800; 335.6217; 346.6475; 350.6261; 353.0823; 355.9423; 364.0180; 397.3445; 403.5666; 428.9509; 454.3374; 488.7615; 489.0610; 490.2561; 491.3922; 499.6062; 522.5948; 525.5936; 529.7256; 557.3390; 557.7898; 558.8637; 561.8369; 569.3696; 573.6985; 577.4816; 599.3392; 620.3575; 626.8541; 629.7276; 632.1390; 635.7274; 644.5382; 719.2891; 720.5203; 738.8497; 760.1371; 761.5563; 761.8295; 763.8092; 787.6818; 863.7281; 869.8567; 927.1240; 934.4053; 941.0579; 967.8667; 973.8870; 975.0165; 984.1453; 984.9332; 986.2870; 991.1562; 997.2182; 1008.9020; 1013.8572; 1051.1457; 1065.8914; 1070.6382; 1073.5182; 1088.4748; 1092.9445; 1104.4923; 1117.9473; 1135.5506; 1185.3162; 1212.8382; 1214.1430; 1214.6095; 1215.3875; 1222.5844; 1227.9835; 1229.4309; 1231.2361; 1235.7149; 1242.5216; 1246.3927; 1253.9406; 1283.2292; 1290.9736; 1300.4708; 1301.6139; 1308.3609; 1315.4238; 1367.2874; 1381.9195; 1429.7148; 1432.6957; 1438.5362; 1458.9639; 1462.3607; 1499.9687; 1503.5212; 1509.3059; 1515.3130; 1516.3322; 1519.6204; 1534.7313; 1538.4089; 1554.4116; 1591.9527; 1613.8074; 1620.2428; 1697.3119; 1856.3409; 3050.6821; 3054.1570; 3057.0538; 3064.2214; 3110.4122; 3118.6202; 3125.0873; 3132.3039; 3135.0527; 3142.3854; 3147.4832; 3156.6950; 3158.3706; 3163.8265; 3174.9664; 3182.5539; 3194.0608.

Cartesian Forces: Max 0.000001381 RMS 0.000000430

Item	Value	Threshold	Converged?
Maximum Force	0.000000	0.000450	YES
RMS Force	0.000000	0.000300	YES
Maximum Displacement	0.000008	0.001800	YES
RMS Displacement	0.000002	0.001200	YES

FILE: Yb_Sq2_PhYbOTf3_TS_Anion_B3LYP_M062x_Add56_Conf3.out

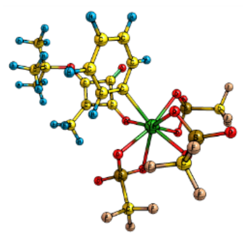

Conformer 6

E(RB3LYP/6-31G(d)/MWB59) = -3731.44811121
Convg = 0.6278D-09 -V/T = 2.0122

Temperature 195.150 Kelvin. Pressure 1.00000 Atm.
elec. and zero-point Energies= -3731.076735
elec. and thermal Energies= -3731.053718
elec. and thermal Enthalpies= -3731.053100
elec. and thermal Free Energies= -3731.121899

RB3LYP/6-31G(d)/MWB59/THF(IEFPCM) = -3731.51457564

RM06-2x/6-311+G(d,p)/MWB59/THF(IEFPCM) = -3731.35296234

Geometry at RB3LYP/6-31G(d)/MWB59
Charge = -1 Multiplicity = 1
Yb 0.5917660142 2.4362933436 1.2483734801
O 2.4226117771 1.6794390826 -0.1388986162
S 3.4854993983 1.5074333742 0.9254164559
O 4.1433652305 0.2003775951 0.9671534634
O 2.921254874 2.0596827504 2.1914682125
C 4.8472117848 2.6773495775 0.418932516
F 5.300304393 2.3338964817 -0.7950607571
F 4.4250473487 3.9406630931 0.3806183906
F 5.8546338564 2.5845494796 1.2967649332
O 1.625570074 4.6480751277 1.2086619938
O 0.26439528 6.5844582824 0.2967799858
O -0.1151286244 4.1611010929 -0.3692275728
S 0.8146109217 5.2498513593 0.0900900166
C 2.02101251 5.4554473536 -1.3176994532
F 3.0400998982 6.2333003415 -0.9318170452
F 2.5051556228 4.2756270116 -1.7171072579
F 1.4024753837 6.0371326992 -2.3535626496
C 0.2063008999 -1.5334450863 0.3579093602
C -1.0637971105 -2.0729017465 0.3877562187
C -1.6071409245 -0.9971152821 1.2898578741
C -0.197850205 -0.3731220453 1.2009197106
O 0.4832846498 0.3258104083 2.0000951268
O -2.6348487497 -0.815682388 1.8870729584
O -1.7828678332 -3.0954888952 -0.0505825865
F -0.9370555812 5.2326495496 5.2075899498
C -0.190041245 0.9506027196 -1.7490077423
C -2.1974454615 1.393243697 -0.515355357
H 0.8899593776 0.8280453498 -1.8038330298
H -2.7193697118 1.5998782524 0.4157236189
C -0.9246192595 0.8799485689 -2.9366219258
C -2.9450195627 1.3228989663 -1.696001574
H -0.4168076291 0.6964529304 -3.8829662042
H -4.022671547 1.4757053095 -1.6663067596
C -2.3112701133 1.0581352544 -2.9117722416
H -2.889005456 1.0030018874 -3.832815509
O -1.8411475875 2.4019797457 4.6922074748
S -1.1019144202 3.1115033635 3.6562760256
C -1.411070638 4.9173998659 3.9954399026
O -1.5514626597 2.9310500158 2.223817676
O 0.3944174857 2.9743612491 3.6306817502
F -0.8124430028 5.6832579059 3.0821276626
C -0.7983221441 1.2075659639 -0.4997561837
F -2.7279037868 5.1592963007 3.9706137768
C -1.3476536967 -4.0699947358 -1.0576722248
C -0.7893355976 -3.3576973874 -2.2937554299
H -0.6604948226 -4.084288282 -3.1040734712
H -1.4809381685 -2.5795957283 -2.6317977287
H 0.1796936441 -2.8921772538 -2.1063415675
C -0.3498411553 -5.040871269 -0.4182284282
H 0.5590136066 -4.5311531568 -0.0894764059
H -0.8007781805 -5.5303711897 0.4512094717
H -0.0620829742 -5.8139632206 -1.1398552783
C -2.654838693 -4.7918892176 -1.4011110447
H -2.4662800055 -5.5955897424 -2.1210618109
H -3.0990995345 -5.2256339459 -0.4997234783
H -3.3747674554 -4.0921819068 -1.8366236327
C 1.5584845958 -1.8135781491 -0.191588779
H 2.3242494781 -1.4073711459 0.4767277646
H 1.752399499 -2.8786495623 -0.3452473803
H 1.6865647293 -1.2997792133 -1.1532893486

Frequency: -199.4162; 16.3476; 17.6312; 22.8113; 25.4577; 30.4630; 31.7928; 36.2378; 38.0133; 38.4552; 44.6503; 53.1626; 56.6548; 57.3807; 67.3676; 68.0438; 70.2214; 74.1321; 78.0107; 85.5070; 90.6152; 101.6394; 112.8875; 117.1309; 122.9390; 131.1892; 141.0173; 143.6967; 144.9435; 152.6951; 157.0248; 165.3289; 170.1950; 180.9660; 187.4744; 195.9335; 208.0072; 211.8697; 214.4918; 215.1001; 217.2399; 220.5537; 224.3117; 232.5262; 259.8025; 270.6536; 287.1667; 302.8410; 306.7250; 308.8469; 320.2809; 322.4536; 325.8378; 335.4386; 344.7168; 350.7884; 353.1885; 356.7865; 367.6671; 398.5091; 404.4132; 430.0978; 451.3469; 484.0142; 488.8763; 490.3968; 491.4045; 501.4505; 522.6959; 525.7900; 530.0183; 557.5070; 557.8831; 559.0390; 564.2763; 569.4770; 573.6711; 577.4917; 597.9607; 620.5837; 626.9327; 629.7122; 631.8861; 635.9764; 650.5737; 721.0294; 727.2182; 760.2247; 761.1810; 761.7337; 761.8913; 768.9285; 777.7927; 849.7959; 870.2427; 928.2099; 931.1056; 934.7316; 973.4518; 974.1566; 974.8860; 979.4663; 984.2084; 987.4297; 992.7090; 1000.4737; 1008.8661; 1013.7728; 1047.6736; 1060.9102; 1063.5066; 1073.1150; 1086.7037; 1093.0839; 1103.6876; 1118.1180; 1135.7306; 1185.1228; 1187.1994; 1212.8114; 1214.1210; 1214.6625; 1216.1290; 1222.6043; 1227.7960; 1229.3963; 1235.7322; 1242.7294; 1246.5982; 1254.3708; 1281.1413; 1290.1933; 1292.6568; 1301.0422; 1308.7132; 1316.0180; 1365.7857; 1368.3445; 1429.3958; 1432.6199; 1439.6797; 1457.6492; 1462.2442; 1499.3223; 1505.9516; 1511.7883; 1515.3205; 1516.1664; 1521.3951; 1528.2048; 1531.5568; 1548.6161; 1583.0717; 1613.6275; 1619.9441; 1697.8100; 1887.1085; 3050.9967; 3058.6691; 3062.1037; 3069.6999; 3113.0282; 3125.5474; 3129.9586; 3136.4833; 3140.3481; 3142.8680; 3146.1579; 3155.9644; 3159.7051; 3172.5120; 3174.9552; 3181.8517; 3196.3424.

Cartesian Forces: Max 0.000000553 RMS 0.000000209

Item	Value	Threshold	Converged?
Maximum Force	0.000000	0.000450	YES
RMS Force	0.000000	0.000300	YES
Maximum Displacement	0.000057	0.001800	YES
RMS Displacement	0.000010	0.001200	YES

FILE: Yb_Sq2_PhYbOTf3_TS_Anion_B3LYP_M062x_Add56_Conf3-1.out

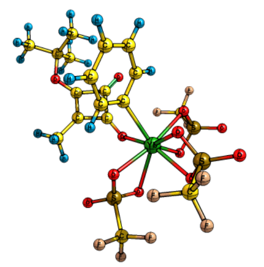

Conformer 7

E(RB3LYP/6-31G(d)/MWB59) = -3731.45765086
Convg = 0.4295D-08 -V/T = 2.0122

Temperature 195.150 Kelvin. Pressure 1.00000 Atm.
elec. and zero-point Energies= -3731.086288
elec. and thermal Energies= -3731.063239
elec. and thermal Enthalpies= -3731.062621
elec. and thermal Free Energies= -3731.131602

RB3LYP/6-31G(d)/MWB59/THF(IEFPCM) = -3731.52125600

RM06-2x/6-311+G(d,p)/MWB59/THF(IEFPCM) = -3731.35601492

Geometry at RB3LYP/6-31G(d)/MWB59
Charge = -1 Multiplicity = 1

```
Yb 0.6219965989 2.2884861608 1.2696525386
O 2.3116281442 1.4136321594 -0.2220020966
S 3.3857205111 1.079642711 0.791604152
O 3.905767211 -0.2878027403 0.7459700551
O 2.9239476555 1.6221616577 2.1030335221
C 4.842928635 2.1340067286 0.2967899029
F 5.1860249246 1.8582881035 -0.9688944001
F 4.5653730959 3.4335293836 0.3978087002
F 5.8800592726 1.851048891 1.0958716093
O 1.8080600147 4.4083787352 1.3221598563
O 0.58824632 6.4579758499 0.4610544857
O 0.0997802756 4.0926813559 -0.3331510287
S 1.0699592192 5.1059032332 0.2068000071
C 2.333172713 5.3064867495 -1.1520187284
F 3.3974653165 5.9740909214 -0.689982036
F 2.7358460955 4.1205490128 -1.6191463985
F 1.7928832158 6.0012858426 -2.1618626062
C -0.085981678 -1.5800188392 0.2101094745
C -1.3728636287 -2.0661099805 0.2621041585
C -1.8255909217 -1.0056139435 1.2361036657
C -0.3866569521 -0.4603672328 1.1292279049
O 0.3390883467 0.1641854951 1.9476775722
O -2.8164813919 -0.7700435754 1.8858678328
O -1.8737813757 -3.132245399 -0.3448067857
C -0.9701265778 1.2834164181 -0.4428192955
C -0.4706205568 1.040093593 -1.7417715604
C -2.3465568789 1.5829422004 -0.3528570419
H 0.5892277714 0.8317483732 -1.8769904407
H -2.7873572861 1.7898110987 0.6195983907
C -1.2848612372 1.0888644228 -2.876699264
C -3.1739999209 1.6354761362 -1.480472205
H -0.8589270277 0.9075586515 -3.8627807463
H -4.229218447 1.8811157302 -1.3699511439
C -2.6460325424 1.3803918692 -2.7479649479
H -3.2851210019 1.4203940797 -3.6283093591
O -1.0978730671 4.4968690129 4.4331443995
S -0.8360185577 3.2390426515 3.7451083994
C -1.7746827558 1.9625588157 4.7281739118
O 0.5772821154 2.7363365794 3.6940864237
O -1.3703103167 3.0986421112 2.3360140286
F -1.5816843527 0.7356990188 4.2458623189
F -3.0829715345 2.2454439045 4.6921075532
F -1.3611960007 1.9965565162 6.0027978039
C -3.3044226674 -3.4868945676 -0.2530139726
C -4.1604820459 -2.3208346722 -0.754879225
H -5.2051465325 -2.6435906022 -0.8338477104
H -4.1203191848 -1.4706859342 -0.0702894147
H -3.8245146342 -1.9924194101 -1.7437474815
C -3.4025174613 -4.6906977556 -1.1927695083
H -3.1181245771 -4.4080830693 -2.2114739663
H -2.7392824196 -5.4956003619 -0.8590392404
H -4.4294761423 -5.0704182142 -1.2102288118
C -3.6405379181 -3.879135173 1.1886622279
H -4.6674459054 -4.2597601415 1.2362553873
H -2.9665024677 -4.6689656726 1.5382198153
H -3.5671261657 -3.017454241 1.8567409555
C 1.1514685235 -2.0113473191 -0.4797512444
H 2.0317657563 -1.8489321823 0.1501135887
H 1.0907038509 -3.0623498864 -0.7803446487
H 1.298904131 -1.4025343152 -1.3812085163
```

Frequency: -191.4852; 14.6431; 18.4831; 23.1236; 27.8850; 29.5306; 31.9635; 35.3140; 36.5096; 39.3063; 42.4377; 49.8528; 55.4196; 57.7572; 65.9033; 69.9219; 71.1095; 78.0686; 79.0216; 82.4175; 88.0418; 96.7859; 110.5039; 113.7563; 117.1696; 123.6021; 132.4930; 139.3994; 146.3413; 151.5520; 153.2120; 155.3017; 170.0514; 180.8555; 191.1853; 206.7961; 209.4208; 210.5312; 213.7725; 216.0023; 216.8802; 221.5084; 224.3599; 258.0887; 268.0521; 275.4252; 296.5542; 303.7764; 306.0660; 309.2292; 320.3926; 322.4860; 325.5531; 335.2224; 346.1241; 350.9524; 352.4290; 355.4311; 363.1730; 396.6286; 402.0004; 427.9037; 454.3548; 488.5508; 489.6442; 490.7058; 491.6946; 499.0106; 523.0254; 524.9871; 530.5317; 557.3698; 558.0362; 558.9692; 561.6484; 570.1007; 571.2238; 578.8214; 599.3515; 619.1121; 625.5586; 629.7621; 632.1980; 638.8948; 644.4362; 718.3565; 720.3253; 738.5797; 759.8732; 761.4196; 761.9663; 762.6648; 787.8105; 863.5600; 868.5542; 924.4375; 934.7596; 941.1510; 968.4007; 972.8934; 974.5176; 982.4046; 984.7647; 985.2826; 990.1418; 997.0145; 1009.3241; 1014.0537; 1050.6230; 1065.9422; 1071.0949; 1073.2376; 1088.3449; 1092.7562; 1104.0831; 1116.6233; 1135.3947; 1185.1980; 1211.9080; 1212.8863; 1213.9542; 1215.2895; 1221.9399; 1226.7941; 1229.6891; 1231.7089; 1233.1031; 1242.9903; 1250.5649; 1253.5260; 1283.3901; 1290.7927; 1300.2176; 1301.3277; 1305.7785; 1318.1879; 1366.4875; 1383.1609; 1429.8763; 1433.0475; 1438.5524; 1459.0975; 1462.0653; 1500.2347; 1503.7118; 1509.1503; 1515.0582; 1516.1782; 1519.6555; 1534.7732; 1538.1711; 1554.1476; 1596.6560; 1613.6292; 1620.2273; 1698.9964; 1853.7839; 3050.2268; 3054.3347; 3057.4303; 3064.5752; 3110.0773; 3118.8427; 3125.7928; 3132.5682; 3135.5335; 3142.6558; 3147.2753; 3156.4470; 3157.8701; 3164.1133; 3172.8806; 3180.8625; 3190.0863.

Cartesian Forces: Max 0.000001093 RMS 0.000000417

Item	Value	Threshold	Converged?
Maximum Force	0.000000	0.000450	YES
RMS Force	0.000000	0.000300	YES
Maximum Displacement	0.000035	0.001800	YES
RMS Displacement	0.000006	0.001200	YES

FILE: Yb_Sq2_PhYbOTf3_TS_Anion_B3LYP_M062x_Add56_Conf4.out

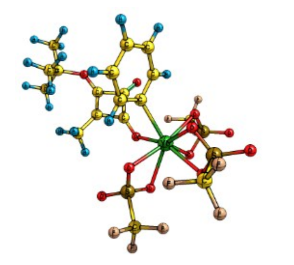

Conformer 8

E(RB3LYP/6-31G(d)/MWB59) = -3731.44789154
Convg = 0.7788D-08 -V/T = 2.0122

Temperature 195.150 Kelvin. Pressure 1.00000 Atm.
elec. and zero-point Energies= -3731.076491
elec. and thermal Energies= -3731.053506
elec. and thermal Enthalpies= -3731.052888
elec. and thermal Free Energies= -3731.121505

RB3LYP/6-31G(d)/MWB59/THF(IEFPCM) = -3731.51410825

RM06-2x/6-311+G(d,p)/MWB59/THF(IEFPCM) = -3731.35173706

Geometry at RB3LYP/6-31G(d)/MWB59
Charge = -1 Multiplicity = 1

```
Yb 0.6897855266 2.3377161398 1.2717104443
O 2.4873054692 1.6466708738 -0.1910059529
S 3.5695155795 1.4132114082 0.8414302117
O 4.2210531009 0.102780165 0.7985137577
O 3.0327895123 1.8982784319 2.1465761642
C 4.9261328436 2.6072644317 0.3792036573
F 5.3210959404 2.3705280079 -0.8793660169
F 4.5206713963 3.8728287735 0.4758162797
F 5.969104486 2.4242499497 1.1996472502
O 1.6647646897 4.5638196689 1.3258142051
O 0.2646937255 6.4797278959 0.4316690938
O 0.0200255348 4.0733155792 -0.3509799621
S 0.8793171507 5.1793399176 0.1949400738
C 2.1372707787 5.4945275086 -1.1468987742
F 3.1201940145 6.2718426787 -0.6759229154
F 2.6668745832 4.3519311992 -1.5949766229
F 1.5455426314 6.1211086584 -2.1724882757
C 0.2591794621 -1.6075288521 0.2564038513
C -1.0138806166 -2.1395924361 0.3112257802
C -1.5143646597 -1.0983621134 1.2758379023
C -0.1040718073 -0.4813840389 1.15980266
O 0.6111842053 0.1929446602 1.9473033921
O -2.519512564 -0.9337594671 1.9162306843
O -1.7567535888 -3.1370482082 -0.1423844467
C -0.7724435241 1.1748765249 -0.4609740674
C -0.2084459173 0.9527222333 -1.737547179
C -2.1718599137 1.3530597647 -0.423260962
H 0.8699991327 0.8407158854 -1.8344538676
H -2.6629214619 1.5356195225 0.5296028104
C -0.9844315761 0.9059056083 -2.8995890579
C -2.9614501314 1.3069819821 -1.5775751727
H -0.5097586214 0.749774134 -3.8677958731
H -4.0378924853 1.4541383106 -1.5057792893
C -2.3704152404 1.0746222751 -2.8211204949
H -2.9804108776 1.0387458028 -3.72207833
O -1.2955194312 4.3828119212 4.385910266
S -0.8905019239 3.1528806214 3.7171393296
C -1.6945328643 1.7914882551 4.7058314589
O 0.5700776323 2.8065545242 3.6871321799
O -1.3877171172 2.9406454948 2.3032185179
F -1.3414050032 0.5890533108 4.2549160974
F -3.0250922682 1.9143603611 4.6350845344
F -1.3184853356 1.9001937886 5.9882341152
C -1.3690114467 -4.0683948036 -1.2093587648
C -0.3573643902 -5.0747611024 -0.6520761174
H -0.1040335927 -5.8165372523 -1.4180647504
H 0.5679496146 -4.5871281969 -0.3357635177
H -0.7798471568 -5.5997801653 0.210918594
C -2.6953492468 -4.7627774027 -1.5350952822
H -3.1102745744 -5.2330017391 -0.6380180753
H -3.4236998803 -4.0381238293 -1.9117921631
H -2.5417080096 -5.5347869338 -2.2967590544
C -0.8500625529 -3.304644675 -2.4315822675
H -0.7655083057 -3.9930923564 -3.280095974
H -1.5419519384 -2.501340703 -2.7036074908
H 0.1332012795 -2.8626730734 -2.2621947629
C 1.5874591474 -1.8704113323 -0.3561260162
H 2.3801994696 -1.4848882376 0.2928406639
H 1.7733523081 -2.9302727915 -0.5506941072
H 1.6762563857 -1.3269224749 -1.3059017915
```

Frequency: -192.5689; 16.2058; 17.9537; 25.0168; 26.9258; 30.1521; 31.3346; 36.1166; 36.5476; 41.6622; 45.2863; 51.6552; 56.0296; 58.8897; 64.5905; 69.6393; 73.6497; 75.2989; 80.2177; 85.1960; 91.2629; 102.5005; 112.7381; 116.9690; 123.1625; 132.5550; 137.3715; 146.8292; 148.1157; 152.5800; 155.7478; 162.8989; 168.3909; 182.5755; 191.6883; 196.3663; 206.6965; 210.1535; 213.2029; 215.9098; 219.3746; 222.1108; 224.4910; 237.0688; 261.8489; 271.1726; 287.8828; 302.5695; 306.0539; 308.7438; 320.2899; 322.4820; 325.8753; 334.7706; 344.7216; 350.9622; 352.5541; 356.5102; 366.1424; 398.2798; 403.4442; 429.2707; 451.1524; 484.0655; 489.6227; 490.7137; 491.6997; 500.4042; 522.9856; 525.1567; 530.7969; 557.4335; 558.1464; 559.0952; 563.9328; 570.1758; 571.3749; 578.7562; 598.2929; 619.2266; 625.6641; 629.9066; 631.9996; 639.1712; 650.3962; 720.5306; 726.7385; 759.8762; 761.0505; 761.7345; 762.3617; 767.7415; 778.2720; 849.2798; 869.1682; 925.9688; 931.3390; 935.2806; 972.9136; 974.4412; 974.6604; 980.0245; 984.4851; 985.7376; 990.5829; 1000.2436; 1009.2522; 1013.8820; 1047.2372; 1061.1417; 1063.6719; 1073.1717; 1087.1447; 1093.1783; 1103.2864; 1116.9129; 1135.7175; 1185.1187; 1188.5240; 1211.3191; 1212.8159; 1214.3440; 1215.6046; 1222.3974; 1226.7173; 1229.6105; 1233.1068; 1242.9517; 1252.2424; 1253.9351; 1281.2848; 1290.0618; 1292.9625; 1300.5149; 1305.7232; 1318.2023; 1366.4340; 1369.2845; 1429.3821; 1433.0305; 1439.3648; 1457.8931; 1462.0042; 1499.7612; 1506.2437; 1512.0391; 1515.4660; 1516.3313; 1521.6072; 1528.5283; 1532.3069; 1548.8227; 1587.6533; 1613.5401; 1619.8503; 1699.9035; 1884.0584; 3050.6481; 3058.9488; 3062.3531; 3070.0236; 3112.5932; 3125.7693; 3130.5656; 3136.8501; 3140.5904; 3142.2936; 3145.9136; 3155.3912; 3159.4011; 3172.7681; 3173.1080; 3180.7012; 3193.3166.

Cartesian Forces: Max 0.000000650 RMS 0.000000265

Item	Value	Threshold	Converged?
Maximum Force	0.000001	0.000450	YES
RMS Force	0.000000	0.000300	YES
Maximum Displacement	0.000020	0.001800	YES
RMS Displacement	0.000005	0.001200	YES

FILE: Yb_Sq2_PhYbOTf3_TS_Anion_B3LYP_M062x_Add56_Conf4-1.out

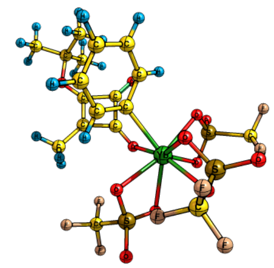

Conformer 9

E(RB3LYP/6-31G(d)/MWB59) = -3731.45535424
Convg = 0.4085D-08 -V/T = 2.0122

Temperature 195.150 Kelvin. Pressure 1.00000 Atm.
elec. and zero-point Energies= -3731.084006
elec. and thermal Energies= -3731.060901
elec. and thermal Enthalpies= -3731.060283
elec. and thermal Free Energies= -3731.129827

RB3LYP/6-31G(d)/MWB59/THF(IEFPCM) = -3731.52032286

RM06-2x/6-311+G(d,p)/MWB59/THF(IEFPCM) = -3731.35451071

Geometry at RB3LYP/6-31G(d)/MWB59
Charge = -1 Multiplicity = 1

```
Yb 1.0817791234 2.3117005992 0.4311575044
O 2.8630408496 2.0995328377 2.2065789506
S 3.7357720757 1.1655728446 1.4341938663
O 5.1495646324 1.4881766597 1.2783650029
O 2.9759698712 0.8142865912 0.1723888391
C 3.7453322285 -0.4167376496 2.4251945747
F 4.5549525846 -0.2797477536 3.4810009583
F 2.5222042609 -0.7334678059 2.8667209081
F 4.1970520003 -1.4279694217 1.6644617917
O 2.5110618604 4.2865464252 0.2588238547
O 2.0798293659 5.878254965 -1.6719850357
O 1.5109879248 3.402982031 -1.7333249333
S 2.377437317 4.5226152405 -1.2236588104
C 4.0698179004 4.1527614646 -1.9098781455
F 4.9602261241 5.0157509618 -1.4069876092
F 4.4465482599 2.9098220548 -1.6022913011
F 4.0498484848 4.2813742159 -3.243603176
C -0.1010604606 -1.6087320649 0.4597003785
C -1.4054583932 -1.8875593996 0.123248123
C -1.877790084 -0.4902868385 0.4510021911
C -0.3996967162 -0.1950694543 0.7837619533
O 0.13114056 0.6228272238 1.5801742173
O -2.9352213037 0.0853883292 0.5318607046
O -1.9201916121 -3.0381358753 -0.2839116636
F -0.896999222 6.3986493846 2.7529428196
C 0.9447156158 0.0485535792 -2.1621558348
C -1.1331060627 1.2289315841 -1.9864052861
H 1.9010144602 -0.2710710264 -1.7520589108
H -1.8411652679 1.8375082203 -1.4287292546
C 0.6449802057 -0.3020856691 -3.4816197626
C -1.4465356583 0.8853527363 -3.3066477379
H 1.3530410422 -0.8898744975 -4.0645994479
H -2.3800562154 1.2281031387 -3.7508356836
C -0.5601437616 0.1101684854 -4.0577072323
H -0.7987340648 -0.1590450078 -5.0851820335
O -2.3186561599 3.7579428691 2.4563942992
S -1.0807336942 3.9849152662 1.7208529908
C -0.9657689952 5.8361824538 1.5391377046
O -1.0124291877 3.4758003485 0.2970547651
O 0.2123334617 3.5719632868 2.3619266729
F 0.1113249109 6.1840334285 0.8366697577
C 0.0695425106 0.8229881899 -1.3689254124
F -2.0597884346 6.2915692083 0.9144031895
C 1.1348015305 -2.4256348241 0.5062838085
H 1.4685273326 -2.5620466827 1.5410309987
H 0.9713128234 -3.4087166096 0.0544245723
H 1.9480144838 -1.914316093 -0.0189611747
C -3.3308250575 -3.159997492 -0.7072076772
C -4.2437156605 -2.93908079 0.5022173929
H -5.2828770956 -3.1455774337 0.2209072076
H -3.9694468788 -3.6159709111 1.3189091194
H -4.1868116281 -1.9055315853 0.8528324381
C -3.6196370064 -2.1749483982 -1.8429256535
H -3.6145816086 -1.1420144157 -1.48811178
H -2.8774479532 -2.275573873 -2.6413478252
H -4.6105416553 -2.3843426964 -2.2624556657
C -3.3990229563 -4.6077588112 -1.1981521419
H -4.4148387348 -4.8427077202 -1.5328046465
H -2.7116123603 -4.763234821 -2.0359310986
H -3.1282489116 -5.3008320023 -0.3947086214
```

Frequency: -184.4720; 12.2456; 15.1155; 22.2402; 24.8582; 28.3372; 31.6553; 33.7826; 36.0540; 39.1800; 46.4082; 47.9927; 53.4842; 55.2359; 57.4517; 61.1771; 71.0610; 72.6881; 79.1239; 80.4681; 85.2188; 104.5428; 108.0840; 117.4070; 122.4043; 129.5170; 131.8500; 140.3421; 147.2274; 152.1116; 154.0270; 157.2256; 167.8048; 181.5082; 190.4872; 209.5334; 210.6936; 213.8693; 214.6513; 215.4405; 217.1290; 218.6533; 221.6171; 256.7778; 265.6077; 275.7359; 296.1178; 304.1691; 305.9447; 308.8952; 320.6325; 322.2814; 324.2483; 334.3006; 344.5943; 350.0897; 353.2014; 355.4489; 362.2385; 397.1676; 401.2893; 428.1308; 453.8166; 485.8978; 488.9621; 489.6411; 491.6425; 497.8407; 523.0203; 525.5949; 529.3136; 557.1822; 558.0236; 558.5668; 561.4229; 569.0976; 573.7256; 574.8396; 600.0849; 624.9765; 627.1233; 629.4614; 630.8992; 634.5806; 642.0619; 717.1938; 720.1620; 738.3239; 757.8317; 761.5100; 761.9236; 762.8615; 787.3503; 862.1012; 868.9930; 924.4376; 934.8255; 941.2252; 964.8804; 973.2855; 975.4981; 981.2075; 983.4231; 985.0947; 989.6285; 995.5036; 1008.9410; 1013.9191; 1058.8870; 1065.6234; 1070.8900; 1073.6097; 1087.4119; 1091.9030; 1104.7079; 1112.5433; 1135.6639; 1185.0815; 1210.8583; 1212.9220; 1213.7761; 1214.4203; 1222.2549; 1223.4541; 1228.8681; 1233.5315; 1235.5404; 1237.3030; 1244.9673; 1251.9491; 1283.5813; 1300.0663; 1301.3365; 1307.1441; 1307.9792; 1315.6058; 1367.2609; 1383.0353; 1429.8605; 1433.2941; 1440.1660; 1459.3040; 1462.1559; 1503.3985; 1503.6493; 1506.1369; 1515.0375; 1516.5009; 1519.6315; 1534.5628; 1537.8647; 1553.8908; 1600.1850; 1613.5488; 1619.9237; 1702.0294; 1859.0523; 3054.2022; 3057.3894; 3061.5636; 3064.5990; 3118.7930; 3124.2470; 3126.1744; 3132.2318; 3135.5573; 3141.4989; 3146.8673; 3155.8974; 3157.2758; 3164.5971; 3172.9264; 3181.0452; 3191.1437.

Cartesian Forces: Max 0.000001079 RMS 0.000000375

Item	Value	Threshold	Converged?
Maximum Force	0.000000	0.000450	YES
RMS Force	0.000000	0.000300	YES
Maximum Displacement	0.000018	0.001800	YES
RMS Displacement	0.000004	0.001200	YES

FILE: Yb_Sq2_PhYbOTf3_TS_Anion_B3LYP_M062x_Add56_Conf5.out

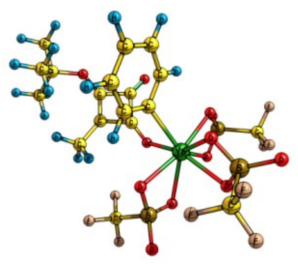

Conformer 10

E(RB3LYP/6-31G(d)/MWB59) = -3731.44459483
Convg = 0.9477D-08 -V/T = 2.0122

Temperature 195.150 Kelvin. Pressure 1.00000 Atm.
elec. and zero-point Energies= -3731.073268
elec. and thermal Energies= -3731.050175
elec. and thermal Enthalpies= -3731.049557
elec. and thermal Free Energies= -3731.118963

RB3LYP/6-31G(d)/MWB59/THF(IEFPCM) = -3731.51234692

RM06-2x/6-311+G(d,p)/MWB59/THF(IEFPCM) = -3731.35026303

Geometry at RB3LYP/6-31G(d)/MWB59
Charge = -1 Multiplicity = 1

```
Yb 1.1429626 2.344995239 0.461439713
O 2.9414746775 2.3327620361 2.2361965684
S 3.8827933999 1.4421753825 1.4948492056
O 5.2649040234 1.8708992571 1.3121617193
O 3.1477613988 0.9778425583 0.2543769766
C 4.0296886861 -0.0898433114 2.5531193162
F 4.8357017729 0.1591122476 3.5909573176
F 2.8419560561 -0.4869778135 3.0234686799
F 4.556732972 -1.0936519353 1.8303768014
O 2.4545551797 4.390230911 0.1925040097
O 1.9085206332 5.8789804501 -1.7907036439
O 1.4820693097 3.37465202 -1.7494106835
S 2.2890624996 4.5608574337 -1.2957494739
C 3.991001776 4.2625501757 -1.9934971361
F 4.8368806934 5.1927200077 -1.5357280667
F 4.4428441545 3.055193638 -1.6475367853
F 3.9460944187 4.3406170343 -3.3306150431
C 0.1884495589 -1.6351288676 0.6057471229
C -1.1164439833 -1.9823958397 0.3182389723
C -1.6550121044 -0.6124606129 0.6347946854
C -0.1866781276 -0.2272420242 0.9163471511
O 0.3423684428 0.6239093312 1.6782222358
O -2.7477062073 -0.1210050988 0.7310322013
O -1.8767077409 -2.9994149248 -0.0584340577
F -1.0716411964 6.3921609052 2.6087823219
C 1.1434397919 -0.0092246538 -2.0415347347
C -1.0253562014 0.9975550544 -1.8882267902
H 2.1296621124 -0.2254776287 -1.6345072323
H -1.7769180742 1.5685746316 -1.3483167766
C 0.8587890335 -0.4479815145 -3.3380461698
C -1.3264782009 0.5596657367 -3.1831143123
H 1.6099382123 -0.9953896789 -3.9066575432
H -2.2944783894 0.792884908 -3.623951706
C -0.3866269013 -0.1724448935 -3.9113027138
H -0.6151653415 -0.5129831653 -4.9197712584
O -2.3276173223 3.6546534435 2.4632470829
S -1.1161196534 3.9230145199 1.6991710076
C -1.1213172338 5.7665802295 1.4254279196
O -1.0285979084 3.3491208461 0.3012770918
O 0.2087760127 3.6254588748 2.3408451942
F -0.0776366207 6.1453099124 0.6888418845
C 0.2142530781 0.725832641 -1.2714930454
F -2.2499641369 6.1208684096 0.7972870186
C 1.5449468839 -2.2399174581 0.6911009625
H 1.7929450143 -2.4679193072 1.7353744156
H 1.6518842939 -3.1565212072 0.1073129919
H 2.2886299334 -1.5172018944 0.3447204098
C -1.3801917665 -4.289737954 -0.5505833188
C -0.4934901095 -4.0652287023 -1.7793178185
H -0.1948071651 -5.0313679678 -2.2017878704
H -1.0406345523 -3.5044314366 -2.5433049186
H 0.4117824122 -3.5016047102 -1.5454060668
C -0.6793761741 -5.0450458415 0.5836887219
H 0.2199366092 -4.5323971931 0.9306558636
H -1.3583477572 -5.1613063637 1.4348788134
H -0.385823079 -6.0432775083 0.2396616731
C -2.6725002355 -5.009878687 -0.9485833328
H -2.4449520177 -6.0076381735 -1.3390229519
H -3.3349147511 -5.1148784241 -0.0834708456
H -3.202067144 -4.4433985258 -1.7205042352
```

Frequency: -186.4166; 12.9323; 17.3976; 21.5939; 23.0978; 26.1294; 30.9928; 33.7259; 35.7012; 37.3205; 46.4631; 52.7670; 55.7268; 56.8550; 58.2086; 64.3510; 70.2426; 74.8546; 76.3748; 82.9854; 88.4594; 96.7095; 112.5422; 119.9801; 124.6769; 133.4185; 139.8390; 144.5260; 149.3990; 155.1237; 155.6356; 168.1402; 174.9962; 180.6037; 186.0984; 196.1388; 209.5583; 210.6173; 214.1588; 215.3524; 215.4328; 218.6285; 221.6048; 230.8833; 258.2935; 268.7983; 286.9318; 303.1684; 305.7817; 307.9874; 320.5397; 322.2526; 324.4846; 336.9604; 343.0811; 349.6297; 353.2646; 357.0682; 369.0596; 396.7321; 401.6755; 429.1963; 452.5345; 483.0593; 488.9620; 489.5212; 491.4881; 503.1036; 522.4678; 525.7916; 529.5488; 557.2727; 557.9244; 558.5003; 563.2776; 568.4350; 573.8316; 574.9991; 596.1789; 624.8861; 627.2258; 629.6525; 630.9661; 634.6043; 651.6209; 720.4413; 726.2890; 757.4718; 760.4453; 761.5063; 762.1204; 768.6421; 774.8624; 849.3385; 868.9183; 924.4815; 930.1166; 933.2810; 972.2617; 973.9824; 975.1451; 977.1401; 980.9128; 982.3797; 992.0297; 1000.7973; 1009.0560; 1013.6940; 1057.8541; 1062.3678; 1062.4781; 1073.3671; 1085.8034; 1092.3425; 1103.7201; 1112.1919; 1135.3002; 1184.0571; 1185.4876; 1208.9630; 1212.9222; 1214.1856; 1215.6026; 1222.9468; 1225.8683; 1233.8676; 1235.4257; 1236.3393; 1244.9091; 1251.3062; 1281.2135; 1291.4485; 1300.3828; 1307.2684; 1308.5512; 1316.1593; 1365.5739; 1369.5937; 1430.5543; 1432.6903; 1442.4562; 1458.8479; 1461.9789; 1500.1570; 1504.4178; 1512.0968; 1514.6318; 1516.8321; 1522.4910; 1528.3405; 1529.9043; 1549.0491; 1591.0720; 1613.4579; 1619.5175; 1701.2662; 1889.0734; 3059.3648; 3059.7264; 3063.2550; 3070.4188; 3124.3595; 3125.1374; 3131.3059; 3136.5500; 3141.5711; 3145.0336; 3155.1625; 3158.6197; 3164.3476; 3169.2576; 3172.5454; 3180.3086; 3194.1798.

Cartesian Forces: Max 0.000000893 RMS 0.000000265

Item	Value	Threshold	Converged?
Maximum Force	0.000001	0.000450	YES
RMS Force	0.000000	0.000300	YES
Maximum Displacement	0.000037	0.001800	YES
RMS Displacement	0.000007	0.001200	YES

FILE: Yb_Sq2_PhYbOTf3_TS_Anion_B3LYP_M062x_Add56_Conf5-1.out

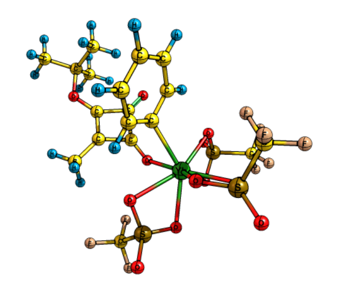

Conformer 11

E(RB3LYP/6-31G(d)/MWB59) = -3731.45650207
Convg = 0.9479D-08 -V/T = 2.0122

Temperature 195.150 Kelvin. Pressure 1.00000 Atm.
elec. and zero-point Energies= -3731.084996
elec. and thermal Energies= -3731.061956
elec. and thermal Enthalpies= -3731.061338
elec. and thermal Free Energies= -3731.130891

RB3LYP/6-31G(d)/MWB59/THF(IEFPCM) = -3731.52107613

RM06-2x/6-311+G(d,p)/MWB59/THF(IEFPCM) = -3731.35794903

Geometry at RB3LYP/6-31G(d)/MWB59
Charge = -1 Multiplicity = 1
Yb 0.3009998436 2.4277090748 1.5616350838
O 2.6690793533 2.743363986 1.7703303144
S 3.1582392487 2.0359274 0.5311777621
O 4.1456272784 2.7198024743 -0.294341867
O 1.9417568442 1.4732891468 -0.1428439824
C 4.0582811634 0.5441701314 1.1964831168
F 5.1867187091 0.9232217998 1.8053698928
F 3.3048186349 -0.1353041284 2.0651230202
F 4.3732553105 -0.2762338749 0.1776421419
O 0.4521372197 4.1162599978 -0.2075720338
O 1.1879779382 6.4615602526 0.4336783597
O 0.156183611 4.8163982762 2.0703150081
S 0.2948802939 5.3336823846 0.6645640188
C -1.4087583772 5.9644924833 0.2555478665
F -1.466068316 6.331478492 -1.0300480755
F -2.3240039338 5.0132233685 0.4753548745
F -1.6975511173 7.0246974186 1.0206412179
C -0.1016386294 -1.3110146168 0.0849503278
C -1.3147651422 -1.9496207009 0.1704460682
C -1.8015654643 -1.0646030115 1.2914193602
C -0.447088327 -0.3316597251 1.1493448082
O 0.2804907852 0.2342551241 2.0144082249
O -2.7523224768 -1.0333891034 2.03482827
O -1.7401928502 -3.0029742566 -0.5175219108
F -0.6472322436 3.831850304 6.4553134276
C -0.9921748687 1.4752060297 -1.458341411
C -2.7335165586 1.6313932592 0.186903843
H 0.0563914472 1.3773620436 -1.730801098
H -3.0715305726 1.6279036156 1.2199918197
C -1.9299036932 1.6464787627 -2.4789359784
C -3.6827823381 1.8058996881 -0.8278558177
H -1.6071006156 1.6645081427 -3.5188208064
H -4.7325904989 1.9418816151 -0.5725423594
C -3.2836318846 1.8065070608 -2.1653603423
H -4.0178370169 1.9401363126 -2.9578310843
O -1.3232018992 1.2215391783 5.202823568
S -0.9023701943 2.3094192943 4.3275024656
C -1.3192322105 3.8489011194 5.2953592892
O -1.6364713058 2.4717336265 3.0160838573
O 0.5604500468 2.4264530768 4.0013715838
F -1.0074300921 4.9598601197 4.6329009293
C -1.3602966173 1.4622863346 -0.0942893827
F -2.6340666627 3.8576041975 5.560662266
C 1.1193958827 -1.5505352037 -0.7195383514
H 2.0077191739 -1.5637896599 -0.0794081406
H 1.0489358322 -2.4955503815 -1.2673693233
H 1.2705608107 -0.731169673 -1.4310039616
C -3.1059629574 -3.5374637577 -0.3629983022
C -3.2710318225 -4.1195255202 1.0441041579
H -4.240508824 -4.6253603838 1.1221184903
H -2.4844615527 -4.8541167421 1.2491664504
H -3.2373568453 -3.3317072559 1.8006628293
C -3.1484228476 -4.6379068353 -1.4259815162
H -4.1229370822 -5.1372410328 -1.4113810681
H -2.987640586 -4.2163767562 -2.423563376
H -2.3711721931 -5.3858678694 -1.2372938067
C -4.1312103556 -2.4398119902 -0.6624093482
H -4.1280865013 -1.6681257282 0.1106441574
H -3.9233677362 -1.9718595208 -1.6302912667
H -5.1352372167 -2.8783888636 -0.6993112601

Frequency: -214.0146; 12.3982; 18.6153; 21.1950; 22.2215; 23.9372; 29.0817; 30.0991; 36.3285; 38.4548; 40.3279; 45.2461; 52.7526; 57.3473; 60.7360; 63.6498; 70.3549; 74.8083; 79.7670; 84.5457; 92.3192; 99.7389; 111.5998; 119.1072; 122.2833; 128.0585; 133.7631; 142.7239; 147.8631; 155.7270; 158.1544; 163.7134; 168.4935; 184.7583; 191.5801; 206.5851; 211.2546; 213.9256; 216.1575; 216.8424; 219.1650; 222.5405; 228.2946; 258.5908; 268.1687; 276.0453; 297.7679; 304.1714; 306.2243; 310.0314; 320.8396; 324.7665; 325.3924; 336.4213; 346.5730; 350.5593; 352.6588; 356.1606; 364.7308; 397.7554; 402.7634; 430.6998; 453.9264; 489.1810; 490.2048; 490.6697; 491.2358; 501.8950; 522.8352; 524.3466; 529.4713; 557.2400; 558.1306; 558.8415; 562.4664; 570.2537; 570.8091; 578.4407; 599.0022; 618.1790; 625.9858; 629.0233; 631.8554; 636.1468; 643.9742; 719.2932; 720.4326; 738.1939; 758.5011; 760.3620; 761.6558; 766.2199; 787.1957; 863.6864; 871.1972; 932.8380; 934.2410; 940.8913; 965.3684; 973.3057; 977.1021; 984.7940; 985.0188; 989.0517; 990.8102; 994.7831; 1009.1726; 1014.1360; 1055.4919; 1065.6206; 1070.7519; 1072.7892; 1087.1619; 1092.9396; 1106.4239; 1115.0183; 1130.1747; 1186.0408; 1207.3555; 1210.2252; 1215.9575; 1217.0720; 1222.4562; 1225.3937; 1227.4573; 1228.5978; 1233.4930; 1237.5526; 1242.6469; 1258.5772; 1283.0141; 1300.5140; 1303.2406; 1304.9215; 1308.1288; 1320.6983; 1367.6127; 1377.7788; 1429.3860; 1432.4912; 1437.9035; 1458.4990; 1462.7740; 1494.7849; 1503.5846; 1504.2492; 1515.4941; 1516.4640; 1519.7044; 1534.7258; 1538.2488; 1554.5527; 1583.2251; 1613.5784; 1621.1654; 1694.6415; 1855.5150; 3053.7113; 3056.5717; 3060.7030; 3063.7557; 3118.0968; 3123.9956; 3125.1018; 3131.9205; 3134.4374; 3138.1640; 3151.3290; 3156.6877; 3162.1669; 3163.3916; 3179.0623; 3188.4881; 3199.2650.

Cartesian Forces: Max 0.000001049 RMS 0.000000258

Item	Value	Threshold	Converged?
Maximum Force	0.000001	0.000450	YES
RMS Force	0.000000	0.000300	YES
Maximum Displacement	0.000071	0.001800	YES
RMS Displacement	0.000009	0.001200	YES

FILE:
Yb_Sq2_PhYbOTf3_TS_Anion_B3LYP_M062x_Add56_Conf6.out

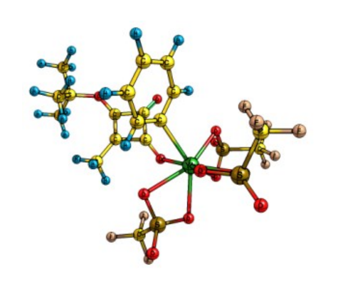

Conformer 12

E(RB3LYP/6-31G(d)/MWB59) = -3731.44583723
Convg = 0.9171D-08 -V/T = 2.0122

Temperature 195.150 Kelvin. Pressure 1.00000 Atm.
elec. and zero-point Energies= -3731.074477
elec. and thermal Energies= -3731.051397
elec. and thermal Enthalpies= -3731.050779
elec. and thermal Free Energies= -3731.120364

RB3LYP/6-31G(d)/MWB59/THF(IEFPCM) = -3731.51342238

RM06-2x/6-311+G(d,p)/MWB59/THF(IEFPCM) = -3731.35325895

Geometry at RB3LYP/6-31G(d)/MWB59
Charge = -1 Multiplicity = 1
Yb 0.3710784334 2.4942720277 1.5746944919
O 2.6879867489 3.0645168886 1.8355268363
S 3.2768120063 2.4231843241 0.6043835868
O 4.1989731692 3.2160359671 -0.1988860449
O 2.1449283187 1.7313217594 -0.0958597772
C 4.323022383 1.0373238474 1.2835908413
F 5.3857882117 1.5338904232 1.9246756272
F 3.6276937385 0.2694376384 2.1284782428
F 4.7529162925 0.2655054835 0.2691342963
O 0.3781050587 4.1988844123 -0.1872071251
O 0.8805335781 6.5995482428 0.4740220138
O -0.0143083813 4.8555846889 2.0888520387
S 0.0945315004 5.3913059542 0.6876143394
C -1.6546718414 5.8619584643 0.2559095206
F -1.7275239935 6.2280443483 -1.0292787247
F -2.4803401928 4.8286816942 0.4589951001
F -2.0519899731 6.8871633751 1.0200584183
C 0.2620307207 -1.2975485237 0.1181436771
C -0.9331154807 -1.9787541305 0.1892156518
C -1.4705646075 -1.1238182799 1.3049220936
C -0.155217045 -0.3188416498 1.1711446757
O 0.5532259157 0.3022162549 2.014041195
O -2.4280875622 -1.1710383053 2.0303857249
O -1.5941574706 -3.0047996613 -0.3315510646
F -0.8066024741 3.7860822524 6.442832399
C -0.737699598 1.4147235386 -1.4723056215
C -2.5456081721 1.4449577533 0.1068006457
H 0.324810044 1.4037581847 -1.7056689527
H -2.9205214188 1.4182101123 1.1266496398
C -1.6477757934 1.5020379242 -2.5283058912
C -3.4674116597 1.5354110126 -0.9433553878
H -1.2891338166 1.5421768198 -3.5559593154
H -4.5332902545 1.5859291772 -0.7273645294
C -3.0209668652 1.5559543836 -2.2652805107
H -3.7339207358 1.6237745249 -3.0850641092
O -1.1943754111 1.1209574042 5.1787611789
S -0.868625468 2.2474226117 4.3123863147
C -1.4580457389 3.7348884805 5.2721617217
O -1.58736955 2.3392854653 2.9854280902
O 0.5817828085 2.5115390989 4.015851873
F -1.2521782727 4.8730419036 4.6143615402
C -1.1542867227 1.3821744839 -0.1222445822
F -2.7706659384 3.6072349535 5.5159696494
C 1.5731799014 -1.3618776138 -0.5787749815
H 2.3707682354 -1.1153443668 0.1280434278
H 1.783923282 -2.338964202 -1.02032102
H 1.6239570363 -0.6016556109 -1.3673798663
C -1.1968687875 -3.7493869019 -1.5301833967
C -0.8639380682 -2.7918307384 -2.6790569586
H -0.7531711853 -3.3631162851 -3.6077332054
H -1.6696799081 -2.0633926317 -2.8139674044
H 0.0642878375 -2.2425370154 -2.5127042635
C -2.4626688887 -4.5502328536 -1.8540739096
H -2.2892340949 -5.2023771521 -2.7169386788
H -2.7507872176 -5.1694198401 -0.9987380968
H -3.2928847053 -3.8756894437 -2.0849185608
C -0.0430313469 -4.6936218617 -1.1759666817
H 0.8472408775 -4.1464818926 -0.856768592
H -0.3396249421 -5.3651377288 -0.3635913381
H 0.2271884634 -5.3021819845 -2.0465578632

Frequency: -213.6959; 12.6568; 17.8726; 22.0806; 22.6892; 24.4119; 29.1129; 31.1554; 34.9039; 36.7913; 40.8085; 43.4378; 55.1794; 56.1573; 61.9101; 66.6464; 70.7896; 75.2071; 80.7942; 85.6138; 93.6654; 95.9301; 106.6519; 112.4372; 124.1547; 132.7279; 138.8042; 145.7775; 151.0408; 155.0525; 164.8180; 167.5861; 169.7273; 184.3923; 188.2143; 195.3292; 209.7474; 211.6802; 214.1068; 217.3014; 218.3465; 220.8115; 227.4260; 234.3470; 260.7441; 272.2651; 289.8315; 303.0557; 306.3401; 308.2123; 320.7853; 324.2333; 325.0146; 336.5922; 345.6855; 350.4564; 352.7186; 356.4769; 367.7489; 400.1703; 403.8605; 431.6153; 451.4606; 483.7583; 489.2576; 490.1585; 491.1804; 504.1103; 522.6557; 524.5136; 529.6087; 557.2298; 558.1659; 558.8650; 565.5793; 570.3095; 571.2774; 578.4238; 595.8739; 618.0708; 626.0124; 629.0645; 631.8132; 636.2016; 650.8490; 720.2289; 729.0002; 758.2672; 759.9978; 760.4094; 761.7197; 770.4074; 774.7629; 848.8062; 871.3780; 930.1467; 933.7255; 934.2496; 970.0626; 972.8229; 977.4877; 977.9082; 985.0991; 988.7923; 995.4143; 997.5595; 1009.2247; 1014.1361; 1050.8156; 1059.7776; 1062.6262; 1073.0492; 1085.1443; 1093.1282; 1105.7108; 1114.9762; 1131.1835; 1181.5725; 1186.1240; 1208.7363; 1210.0927; 1216.4870; 1217.2443; 1223.6012; 1227.2151; 1228.3774; 1233.5661; 1237.2743; 1240.5191; 1258.4928; 1280.2414; 1291.9069; 1303.1960; 1305.7093; 1308.0516; 1320.7552; 1361.1462; 1369.2121; 1428.7443; 1432.4526; 1437.1915; 1457.4310; 1462.6867; 1495.4824; 1503.6581; 1505.5762; 1515.5787; 1516.8333; 1518.0075; 1527.4392; 1531.3304; 1548.2324; 1575.2156; 1613.5985; 1621.0268; 1695.9132; 1886.4378; 3058.3789; 3061.6777; 3065.2645; 3069.7723; 3124.8689; 3125.9012; 3129.6703; 3136.2603; 3140.2948; 3148.3546; 3150.5704; 3157.2654; 3162.8154; 3171.5168; 3179.1002; 3186.8583; 3201.0068.

Cartesian Forces: Max 0.000001097 RMS 0.000000330

Item	Value	Threshold	Converged?
Maximum Force	0.000001	0.000450	YES
RMS Force	0.000000	0.000300	YES
Maximum Displacement	0.000034	0.001800	YES
RMS Displacement	0.000007	0.001200	YES

FILE: Yb_Sq2_PhYbOTf3_TS_Anion_B3LYP_M062x_Add56_Conf6-1.out

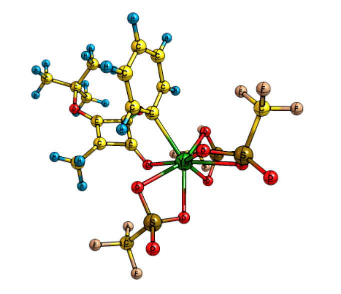

Conformer 13

E(RB3LYP/6-31G(d)/MWB59) = -3731.45743082
Convg = 0.9989D-08 -V/T = 2.0122

Temperature 195.150 Kelvin. Pressure 1.00000 Atm.
elec. and zero-point Energies= -3731.085896
elec. and thermal Energies= -3731.062888
elec. and thermal Enthalpies= -3731.062270
elec. and thermal Free Energies= -3731.131360

RB3LYP/6-31G(d)/MWB59/THF(IEFPCM) = -3731.52177853

RM06-2x/6-311+G(d,p)/MWB59/THF(IEFPCM) = -3731.35848353

Geometry at RB3LYP/6-31G(d)/MWB59
Charge = -1 Multiplicity = 1
Yb 0.4002138295 2.3882693828 1.5166724887
O 2.7780004719 2.6264089291 1.6833172017
S 3.2294205512 1.9797858993 0.3972361437
O 4.2053383081 2.6975042384 -0.413265451
O 1.9934271331 1.4647886138 -0.277256935
C 4.1343533292 0.449081534 0.960361583
F 5.2819399528 0.7877505083 1.5570027515
F 3.400995653 -0.272167738 1.8139158014
F 4.4145307341 -0.3196951856 -0.1077016735
O 0.4867542248 4.1208834549 -0.1797731568
O 1.3082350691 6.4364755231 0.4610966566
O 0.3471139503 4.7760730466 2.1263105025
S 0.4068920171 5.3233555342 0.7283194422
C -1.3046130479 5.9929108477 0.4269388661
F -1.4136077923 6.4134156213 -0.8395259266
F -2.2226506035 5.0452580836 0.647598192
F -1.5411193466 7.0239645583 1.2461437919
C -0.0600304335 -1.3409294861 -0.0015644904
C -1.278102114 -1.9641387216 0.1289384229
C -1.6988149118 -1.0902835798 1.2849572375
C -0.3445382159 -0.3733803247 1.0886916122
O 0.4239867592 0.1865872202 1.9192887159
O -2.6103446808 -1.0589562044 2.0772158309
O -1.7498153035 -2.9993971013 -0.5544712291
F -2.6212309768 1.0352602124 5.3603581094
C -0.9595442903 1.4757287815 -1.486829028
C -2.6730342756 1.5994500718 0.1884074928
H 0.0851298889 1.3954391213 -1.7787506592
H -2.9944623713 1.5916864951 1.2266248702
C -1.9175522156 1.6386332649 -2.4902476123
C -3.642673346 1.7645244737 -0.808340528
H -1.6128235107 1.6672193939 -3.5353639189
H -4.689300225 1.8864540228 -0.5334886597
C -3.2677002296 1.7768343773 -2.1529280637
H -4.0175814094 1.9042365608 -2.9316204047
O -1.1943615928 3.6731635753 5.1856560092
S -0.8011956858 2.600702651 4.2797904469
C -1.2938485167 1.0437490806 5.1834793178
O 0.6581110753 2.4313390995 3.9665635169
O -1.5249675912 2.5147494759 2.9545656953
F -0.9374684722 -0.05505435 4.5198984832
C -1.302515941 1.4505377338 -0.1162595282
F -0.6994655036 1.0303694496 6.3861963019
C 1.1211582885 -1.5778544539 -0.8636378452
H 2.0354216781 -1.6198462181 -0.2623630422
H 1.0136581087 -2.5068269616 -1.4322375815
H 1.2532038456 -0.7412783899 -1.55877355
C -3.1192801673 -3.5108299915 -0.3544842908
C -4.1335933666 -2.3885205318 -0.5924337383
H -4.086423011 -1.6324271588 0.1944529989
H -3.9541435429 -1.9043307382 -1.5578903407
H -5.1464403395 -2.8078301481 -0.5990212592
C -3.238135415 -4.1154023169 1.0477020934
H -4.2138706987 -4.6029893951 1.1570293989
H -2.4592723561 -4.8693203228 1.2070086822
H -3.1570809472 -3.3422417252 1.8157496989
C -3.2256070932 -4.5902973883 -1.4342222374
H -4.2080835515 -5.071787088 -1.3886147944
H -3.0975587719 -4.1532331926 -2.4298016378
H -2.4557940038 -5.3558221246 -1.2909587737

Frequency: -205.9016; 14.3515; 19.2148; 21.8392; 24.9726; 29.2613; 30.6667; 34.6273; 36.5663; 38.6092; 41.7870; 44.7627; 55.3769; 56.2502; 60.5667; 63.7291; 69.2933; 73.9843; 80.1820; 83.5736; 92.2505; 98.9705; 110.9130; 118.2317; 122.4204; 125.3327; 138.0575; 144.0139; 147.7366; 155.5976; 160.4168; 162.4551; 171.6035; 184.0680; 189.9258; 204.5965; 209.2611; 214.0112; 214.7538; 217.2229; 218.9504; 222.3300; 226.8335; 258.6021; 267.2436; 276.3359; 297.4877; 303.7774; 305.9318; 310.8479; 320.3959; 324.9798; 325.7472; 336.9025; 346.6469; 350.4540; 352.3352; 356.6236; 364.3264; 397.4344; 402.8091; 430.7793; 453.9200; 489.6724; 489.9067; 490.4421; 491.5145; 500.9534; 522.6714; 524.7952; 529.9812; 557.2303; 558.0266; 558.4577; 563.1941; 569.5370; 571.0601; 578.4765; 599.2787; 618.0883; 623.8529; 629.2019; 631.7570; 637.5007; 643.8293; 719.6018; 720.2613; 737.7873; 758.2217; 760.7297; 761.0575; 764.6958; 787.0579; 863.6560; 870.8595; 931.7668; 934.1058; 940.7554; 965.8780; 972.4858; 976.0519; 984.5451; 985.0402; 988.4950; 991.6628; 994.6902; 1009.2173; 1014.2826; 1055.0751; 1065.6659; 1070.7214; 1073.1580; 1087.1134; 1093.1239; 1107.7948; 1112.4754; 1131.3819; 1185.9591; 1207.5542; 1210.2796; 1215.3817; 1216.5137; 1222.9720; 1226.2662; 1227.2639; 1229.9477; 1234.8364; 1238.3669; 1240.5135; 1251.0392; 1283.1638; 1300.4004; 1302.6861; 1306.8190; 1307.9446; 1320.4884; 1367.6881; 1379.5209; 1429.5813; 1432.7338; 1437.9473; 1458.8245; 1462.5397; 1494.9120; 1503.5896; 1504.2435; 1515.3278; 1516.3615; 1519.7628; 1534.6814; 1538.0055; 1554.3089; 1589.0175; 1613.6050; 1620.7865; 1695.5852; 1852.6683; 3053.8798; 3057.0482; 3061.1813; 3064.2395; 3118.3247; 3125.0611; 3125.4165; 3132.1494; 3135.0814; 3139.2151; 3151.0731; 3156.1740; 3161.8702; 3163.4663; 3179.2866; 3189.6856; 3199.7902.

Cartesian Forces: Max 0.000000964 RMS 0.000000374

Item	Value	Threshold	Converged?
Maximum Force	0.000001	0.000450	YES
RMS Force	0.000000	0.000300	YES
Maximum Displacement	0.000032	0.001800	YES
RMS Displacement	0.000007	0.001200	YES

FILE:
Yb_Sq2_PhYbOTf3_TS_Anion_B3LYP_M062x_Add56_Conf7.out

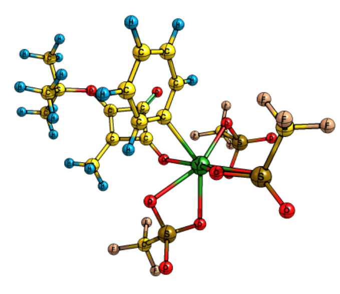

Conformer 14

E(RB3LYP/6-31G(d)/MWB59) = -3731.44712848
Convg = 0.7836D-08 -V/T = 2.0122

Temperature 195.150 Kelvin. Pressure 1.00000 Atm.
elec. and zero-point Energies= -3731.075780
elec. and thermal Energies= -3731.052718
elec. and thermal Enthalpies= -3731.052100
elec. and thermal Free Energies= -3731.121328

RB3LYP/6-31G(d)/MWB59/THF(IEFPCM) = -3731.51442628

RM06-2x/6-311+G(d,p)/MWB59/THF(IEFPCM) = -3731.35405577

Geometry at RB3LYP/6-31G(d)/MWB59
Charge = -1 Multiplicity = 1
Yb 0.4488860204 2.4543370588 1.5342271726
O 2.7798825643 2.9521413209 1.7844924234
S 3.3436782079 2.3722959751 0.5113972565
O 4.2610236727 3.2010679743 -0.2607781022
O 2.1974489788 1.7281664472 -0.2084680475
C 4.3934732499 0.9471772878 1.0986383285
F 5.4751512428 1.4042189587 1.7375034414
F 3.7141170582 0.1419876675 1.922631565
F 4.7930342353 0.2221061784 0.0381913563
O 0.398188571 4.1995325953 -0.1511047626
O 0.9657025339 6.5813052141 0.5211141241
O 0.1338261893 4.8212163776 2.1532967316
S 0.1742158181 5.3814814824 0.7600465518
C -1.5872723131 5.8781110299 0.415646496
F -1.7046917553 6.2929820662 -0.8520597265
F -2.4112238411 4.84230612 0.6088266759
F -1.9469342262 6.8757309535 1.2314176562
C 0.2968727778 -1.3297132139 0.0257934797
C -0.9029964374 -1.9979114074 0.1436067065
C -1.3754198362 -1.1510120662 1.2938993929
C -0.0606088915 -0.359714711 1.1056442197
O 0.6854242876 0.2580765936 1.9156935081
O -2.2952845586 -1.1955907898 2.0682459456
O -1.5993929756 -3.0078164392 -0.3600487631
F -2.5400234891 0.7538857016 5.2465288274
C -0.6926666338 1.4118434434 -1.5125187962
C -2.4913602718 1.3918160317 0.0757048778
H 0.368122267 1.4289841963 -1.7525022577
H -2.8616087065 1.3577013532 1.0968345537
C -1.610736016 1.4826461455 -2.563402198
C -3.4211168838 1.4630371051 -0.968602943
H -1.2584468257 1.5381385009 -3.592560941
H -4.4865578718 1.4886658609 -0.74603341
C -2.9832707492 1.4999723812 -2.293335673
H -3.7023630943 1.554801358 -3.1087255013
O -1.3947471002 3.5392866256 5.1463887124
S -0.8606183872 2.5201913357 4.2512156936
C -1.2165157837 0.9125267147 5.1304989326
O 0.6187906931 2.5071347654 3.9880711049
O -1.5239433678 2.3711056409 2.8998453918
F -0.7059221659 -0.1342184955 4.4843782733
C -1.1000682706 1.3619774633 -0.1599877399
F -0.6789911012 0.9616256349 6.3592336998
C 1.5713050552 -1.3933544977 -0.7356821752
H 2.4045333311 -1.1590316952 -0.0668747431
H 1.7538862931 -2.3657335404 -1.1995270794
H 1.5848436478 -0.6233653709 -1.5164519125
C -1.2797395715 -3.7231724132 -1.6004777576
C -0.1216376603 -4.6912830724 -1.3370857023
H 0.792846619 -4.1643336894 -1.0542708107
H -0.3815726642 -5.3816817226 -0.5279488878
H 0.0908107441 -5.2785321903 -2.2377620885
C -0.9968378158 -2.7370018836 -2.7382901635
H -0.9507095488 -3.282432555 -3.6877238563
H -1.7948003667 -1.9908625285 -2.8051325395
H -0.0503604703 -2.2086919972 -2.6112104749
C -2.573128261 -4.4977303692 -1.8749719181
H -2.4570533505 -5.1276381364 -2.7635551825
H -2.8228306898 -5.1369520932 -1.0222696377
H -3.4046458381 -3.8060304185 -2.0411265089

Frequency: -207.4656; 14.6510; 18.6576; 21.5606; 24.0881; 27.4578; 31.7038; 34.3473; 35.7136; 39.1240; 39.9549; 47.0136; 54.0636; 57.9448; 62.6032; 63.6849; 70.9113; 73.5499; 80.2133; 86.0282; 91.5179; 96.4065; 104.6516; 113.1977; 120.6821; 136.4533; 140.6351; 145.1024; 152.2839; 156.2690; 162.2891; 165.8701; 172.0336; 183.5575; 187.0023; 194.0409; 206.8483; 210.7070; 214.4118; 215.5269; 219.3063; 220.8436; 227.7678; 233.3785; 260.7981; 271.6872; 290.0132; 303.0052; 306.0412; 307.9781; 320.4509; 324.5638; 325.1836; 336.1545; 346.0911; 350.3512; 352.7435; 357.1474; 366.9937; 399.8143; 403.1342; 431.4157; 450.9941; 483.2210; 489.8074; 490.3572; 491.4949; 502.4636; 522.4580; 524.9915; 530.0770; 557.2326; 558.0164; 558.4452; 566.7082; 569.5475; 571.2479; 578.3771; 596.4407; 617.9394; 623.9554; 629.2073; 631.5406; 637.5568; 650.7415; 719.9506; 728.4120; 757.9760; 759.9871; 760.7803; 761.3890; 769.6695; 774.6095; 848.5073; 870.6485; 930.0807; 932.5227; 933.8703; 970.7176; 972.1804; 976.2701; 977.8916; 984.7798; 989.1741; 993.6301; 997.4315; 1009.1381; 1014.1890; 1049.9537; 1059.7067; 1062.5562; 1073.1255; 1085.5622; 1093.2873; 1106.7363; 1112.4489; 1132.3159; 1183.0491; 1186.0109; 1208.6747; 1209.1757; 1215.5381; 1216.6586; 1223.6740; 1226.6393; 1230.7234; 1234.9228; 1238.1304; 1239.3033; 1252.3758; 1280.2053; 1292.0530; 1302.4161; 1306.7863; 1307.7744; 1320.4038; 1363.4581; 1369.0182; 1428.8286; 1432.4165; 1436.7484; 1457.5652; 1462.4203; 1495.5920; 1503.7402; 1505.5168; 1515.2234; 1516.5664; 1517.8559; 1527.5067; 1530.9793; 1547.9617; 1580.5305; 1613.4904; 1620.4520; 1696.5523; 1882.8045; 3058.5778; 3061.7746; 3064.7190; 3069.9911; 3124.8863; 3125.9016; 3130.3216; 3136.5497; 3140.5905; 3148.8221; 3150.4426; 3157.1397; 3162.6847; 3171.0180; 3179.5140; 3188.2299; 3202.1676.

Cartesian Forces: Max 0.000000985 RMS 0.000000377

Item	Value	Threshold	Converged?
Maximum Force	0.000001	0.000450	YES
RMS Force	0.000000	0.000300	YES
Maximum Displacement	0.000079	0.001800	YES
RMS Displacement	0.000016	0.001200	YES

FILE: Yb_Sq2_PhYbOTf3_TS_Anion_B3LYP_M062x_Add56_Conf7-1.out

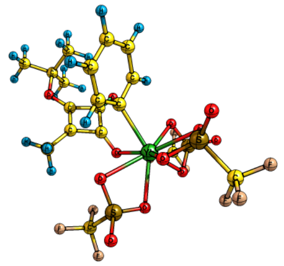

Conformer 15

E(RB3LYP/6-31G(d)/MWB59) = -3731.45762246
Convg = 0.1441D-08 -V/T = 2.0122

Temperature 195.150 Kelvin. Pressure 1.00000 Atm.
elec. and zero-point Energies= -3731.086110
elec. and thermal Energies= -3731.063050
elec. and thermal Enthalpies= -3731.062432
elec. and thermal Free Energies= -3731.131962

RB3LYP/6-31G(d)/MWB59/THF(IEFPCM) = -3731.52207118

RM06-2x/6-311+G(d,p)/MWB59/THF(IEFPCM) = -3731.35879862

Geometry at RB3LYP/6-31G(d)/MWB59
Charge = -1 Multiplicity = 1

```
Yb  0.2780976446  2.6694781232  1.0357944726
O   2.6357741993  2.8072973065  1.4890030512
S   3.1965583151  1.9936671775  0.3499356629
O   4.2852837051  2.5782987152 -0.4246746906
O   2.0255658183  1.425825959 -0.3938013017
C   3.9813235823  0.5260543156  1.193501062
F   5.0713722578  0.9133783525  1.8655646519
F   3.1408558442 -0.0690312241  2.0457259181
F   4.3467810042 -0.376845796  0.265128582
O  -0.7810154756  4.8377251198  0.6520367922
O  -0.6254965107  5.8728969938 -1.6613857879
O   0.9969874876  4.1246181069 -0.7932888384
S   0.0554002556  5.2686829785 -0.5233623014
C   1.151962575  6.6061820155  0.1679655086
F   0.4097937905  7.6581454002  0.5328414314
F   1.8191490732  6.1547256599  1.234611664
F   2.0313550013  6.9984322435 -0.7612018303
C  -0.1758103009 -1.2445644291  0.148239732
C  -1.4165956607 -1.8137628991  0.3014370461
C  -1.8838749696 -0.7466872299  1.2603848149
C  -0.4999748591 -0.0954172968  1.0344750534
O   0.2363844524  0.5730190776  1.8148698009
O  -2.8399804721 -0.5682120749  1.9767238891
O  -1.8744646459 -2.9442810929 -0.2240736119
F  -2.3206370693  1.9019514479  5.3470341653
C  -0.8464466112  1.3028755902 -1.8511234156
C  -2.6822958926  1.7380898691 -0.3627136552
H   0.2134611889  1.1427528655 -2.0365226393
H  -3.0808028859  1.9038717083  0.6349777471
C  -1.7143987294  1.3458468211 -2.945525554
C  -3.5600761212  1.7815957491 -1.4516898692
H  -1.3275537468  1.2085255313 -3.9539818297
H  -4.6179320937  1.9814361315 -1.289538956
C  -3.0784583778  1.5774032567 -2.746521633
H  -3.757614505  1.6127697648 -3.5962558043
O  -1.6518338002  4.6362086402  4.2409689007
S  -1.0317509067  3.4542174117  3.6539666155
C  -1.0530580389  2.1922927448  5.0269956498
O   0.425511369  3.5500576017  3.2675769513
O  -1.7247686825  2.790545676  2.4993988387
F  -0.4276144001  1.0683503798  4.6730952485
C  -1.3009594938  1.4957870146 -0.5275489444
F  -0.4424055666  2.7007336865  6.1072717524
C  -3.267280952 -3.388733575 -0.0302429596
C  -3.4902932656 -3.7411499672  1.4435543042
H  -4.4844395917 -4.1855954258  1.5698961398
H  -2.7444984833 -4.4698688016  1.7799855376
H  -3.4348315432 -2.8488109962  2.0719541816
C  -3.33860228 -4.6356786438 -0.9148937838
H  -4.3356272923 -5.0842975018 -0.8525428422
H  -3.1367700296 -4.3786923116 -1.9598138834
H  -2.6016393082 -5.3789841436 -0.5930740714
C  -4.2329187199 -2.3063613651 -0.5217025377
H  -4.2121130433 -1.4272714985  0.1262632689
H  -3.9813385103 -1.9993349245 -1.5421431671
H  -5.2551563966 -2.7022805067 -0.5222398335
C   1.0521571374 -1.6508890337 -0.57411738
H   1.9208838052 -1.6109365423  0.0912517883
H   0.9515870531 -2.6603790615 -0.984922724
H   1.2609886719 -0.9484910942 -1.3887263775
```

Frequency: -211.2205; 12.3399; 16.4947; 21.7754; 25.7773; 29.5679; 30.0430; 33.8428; 35.4328; 36.7368; 40.4773; 44.0447; 49.8530; 54.5121; 56.5759; 64.5311; 67.1948; 72.3744; 80.4229; 84.2487; 94.5105; 95.4976; 108.9467; 116.0759; 121.0997; 128.3095; 135.6775; 142.3862; 146.6000; 155.1924; 160.5989; 165.1253; 173.2648; 178.6182; 191.9745; 204.9459; 210.3464; 212.9578; 215.7458; 216.5433; 220.3911; 223.4021; 225.9177; 258.8745; 267.9238; 277.0028; 298.8224; 303.9482; 306.6183; 310.8695; 321.1850; 325.1619; 328.0057; 336.5536; 346.5521; 350.0139; 352.0096; 356.6474; 363.8960; 398.1187; 401.5925; 431.9668; 453.8383; 489.6060; 489.9754; 491.0874; 491.6588; 501.7777; 522.0767; 524.8376; 529.4235; 557.6463; 558.2603; 558.8024; 563.3540; 569.2029; 572.0709; 577.3364; 599.3240; 620.8347; 623.3614; 628.3942; 632.1818; 636.7532; 644.1823; 719.3505; 720.4386; 737.8451; 757.7207; 760.7844; 761.5842; 766.1924; 786.7894; 863.5976; 871.7417; 933.6117; 934.5529; 940.8681; 965.6403; 973.9579; 977.7991; 984.7503; 985.0229; 989.4070; 991.0120; 995.0135; 1010.4936; 1014.7272; 1055.1650; 1065.5537; 1070.4617; 1072.8996; 1087.0745; 1093.5367; 1109.4163; 1118.8362; 1129.1796; 1186.8609; 1207.2318; 1213.1922; 1215.0368; 1215.9449; 1222.4990; 1224.8728; 1226.8607; 1231.2823; 1237.8017; 1239.1475; 1239.8932; 1246.6268; 1282.9179; 1300.6057; 1303.5781; 1305.6052; 1308.1546; 1317.0745; 1367.9103; 1377.9260; 1429.4207; 1432.1537; 1437.7218; 1458.5106; 1463.0652; 1495.3548; 1503.4807; 1504.0830; 1515.5145; 1516.4773; 1519.7652; 1534.7029; 1538.3591; 1554.7203; 1584.1447; 1614.4412; 1621.1091; 1694.5775; 1853.0617; 3053.5886; 3056.8317; 3061.1027; 3064.0117; 3117.9498; 3124.5620; 3125.2147; 3131.9344; 3134.7505; 3139.3070; 3154.0191; 3156.3759; 3163.3457; 3164.7661; 3180.6432; 3188.8786; 3199.1720.

Cartesian Forces: Max 0.000000758 RMS 0.000000274

Item	Value	Threshold	Converged?
Maximum Force	0.000000	0.000450	YES
RMS Force	0.000000	0.000300	YES
Maximum Displacement	0.000022	0.001800	YES
RMS Displacement	0.000005	0.001200	YES

FILE: Yb_Sq2_PhYbOTf3_TS_Anion_B3LYP_M062x_Add56_Conf8.out

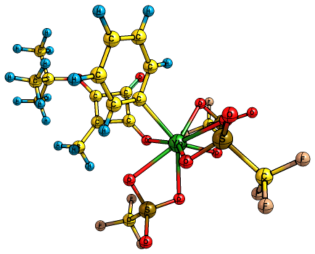

Conformer 16

E(RB3LYP/6-31G(d)/MWB59) = -3731.44718925
Convg = 0.7445D-08 -V/T = 2.0122

Temperature 195.150 Kelvin. Pressure 1.00000 Atm.
elec. and zero-point Energies= -3731.075833
elec. and thermal Energies= -3731.052719
elec. and thermal Enthalpies= -3731.052101
elec. and thermal Free Energies= -3731.121871

RB3LYP/6-31G(d)/MWB59/THF(IEFPCM) = -3731.51434212

RM06-2x/6-311+G(d,p)/MWB59/THF(IEFPCM) = -3731.35410046

Geometry at RB3LYP/6-31G(d)/MWB59
Charge = -1 Multiplicity = 1

```
Yb  0.3384660183  2.7116679797  1.0476548242
O   2.6579994694  3.1129182663  1.5300103145
S   3.3267513414  2.3714493486  0.3999733238
O   4.3518806197  3.0800660345 -0.357945488
O   2.2435545023  1.6690208523 -0.3602994887
C   4.2647498193  1.0071732252  1.2594956789
F   5.2878352203  1.5186146908  1.9530453677
F   3.4829826198  0.3152371891  2.0958684254
F   4.7515946449  0.1544092655  0.339752957
O  -0.932167659  4.7590856706  0.642769855
O  -0.8649198564  5.7930234774 -1.6754940457
O   0.9215843107  4.2256836166 -0.7846070137
S  -0.1349233115  5.2679716331 -0.5285793122
C   0.8119163662  6.7153727141  0.1627759641
F  -0.0379293166  7.6875461642  0.5139765024
F   1.5123955431  6.3418467219  1.2380972818
F   1.6546061165  7.1905222732 -0.7617920294
C   0.1821366141 -1.2546606327  0.1901294773
C  -1.0394958283 -1.8725108348  0.3432956633
C  -1.5563867512 -0.8351062904  1.3024873758
C  -0.212286041 -0.1058337933  1.0651062614
O   0.5080230787  0.614935904  1.8138140337
O  -2.5180370327 -0.7383146331  2.0186706985
O  -1.7319230387 -2.9433954585 -0.0240824831
F  -2.2310124144  1.6859815329  5.322569951
C  -0.5865135357  1.2224657478 -1.8424093669
C  -2.4989557803  1.4964730385 -0.4130455135
H   0.4891559575  1.1685779399 -1.9961840748
H  -2.9408460074  1.6309454034  0.570858911
C  -1.4219164399  1.1694112133 -2.9618236112
C  -3.3440331581  1.4433904787 -1.5268236224
H  -0.9938642053  1.0635990158 -3.9575280283
H  -4.4204279098  1.5398973236 -1.3968408172
C  -2.8076065808  1.2703116978 -2.8043169022
H  -3.4618901276  1.230580463 -3.6731882832
O  -1.8293538055  4.4676189752  4.2170828622
S  -1.0815683618  3.3560869668  3.6416144158
C  -0.9950370003  2.0965677444  5.0142809594
O   0.3644486169  3.6020632462  3.2783773028
O  -1.6821887653  2.6265245887  2.474792133
F  -0.2603896312  1.0390496528  4.6656828479
C  -1.0970879198  1.3834937422 -0.5346724981
F  -0.4454342288  2.6612241206  6.0997249003
C  -1.3579253279 -3.865183698 -1.1014691654
C  -0.983901388 -3.0933690305 -2.3710970438
H  -0.8892045603 -3.7948683244 -3.2077533133
H  -1.7611365274 -2.3630704136 -2.6172325031
H  -0.037017404 -2.5592691279 -2.2749653618
C  -2.6516947741 -4.6567255944 -1.3205816296
H  -2.4993318431 -5.4308522244 -2.0804537155
H  -2.9668104734 -5.1371218404 -0.3889228259
H  -3.4546920266 -3.991718821 -1.6529209517
C  -0.2421674848 -4.7913228384 -0.6058764905
H   0.6671377426 -4.2376798993 -0.3599726716
H  -0.5670200459 -5.3288746724  0.2910268725
H   0.0079755114 -5.5273472118 -1.3785099854
C   1.499532651 -1.471660288 -0.4622066039
H   2.2956753019 -1.1573774904  0.2187844807
H   1.6754634042 -2.5092777284 -0.7559651528
H   1.5928196017 -0.8348703846 -1.3500830801
```

Frequency: -214.0117; 11.0379; 15.8349; 22.1667; 24.4563; 28.6503; 29.2868; 31.8070; 33.9053; 36.2845; 42.3142; 43.6883; 51.6891; 53.9798; 57.5086; 65.0243; 70.1335; 72.3984; 80.2227; 82.2467; 92.7158; 97.4911; 104.0318; 113.3462; 121.0150; 135.9685; 139.3806; 141.9124; 152.7379; 159.2452; 163.2887; 166.9330; 174.1002; 178.5175; 187.8659; 196.8777; 205.3470; 212.5103; 213.6098; 214.7012; 219.2501; 223.0202; 225.3080; 233.7285; 260.6597; 273.9800; 291.0853; 303.9075; 306.5695; 307.1838; 320.6445; 324.5346; 327.6215; 336.8018; 345.9717; 350.0539; 352.1548; 357.2256; 367.0519; 399.9898; 402.9396; 432.7521; 451.1751; 482.8545; 489.7860; 491.0705; 491.6518; 503.2859; 521.7801; 524.9929; 529.4471; 557.6311; 558.2420; 558.7811; 567.3270; 569.1439; 572.2632; 577.1594; 596.5486; 620.6661; 623.3394; 628.5681; 632.1264; 636.8065; 650.8569; 720.2591; 729.1293; 757.4124; 759.7780; 761.0297; 761.5622; 771.2287; 774.0197; 848.6801; 872.0610; 929.8474; 933.6775; 935.3726; 970.3391; 973.7358; 978.0125; 978.1827; 985.2113; 990.1047; 995.2431; 996.9548; 1010.5987; 1014.6917; 1050.5093; 1060.0226; 1062.3234; 1072.7631; 1085.0963; 1093.8409; 1109.0806; 1118.8959; 1129.9932; 1181.9338; 1186.8566; 1208.4896; 1212.7156; 1215.1931; 1216.1683; 1223.0209; 1226.2475; 1232.0479; 1236.2132; 1238.8841; 1239.9724; 1246.8325; 1280.1559; 1291.9592; 1303.6096; 1305.6153; 1308.1675; 1316.9917; 1361.7701; 1368.8557; 1428.5990; 1432.3696; 1436.9963; 1457.3459; 1463.0868; 1496.3208; 1503.9498; 1505.8236; 1515.4746; 1516.8229; 1517.8561; 1527.5046; 1531.0985; 1547.9069; 1575.1636; 1614.3412; 1620.8822; 1695.4496; 1883.8035; 3058.3588; 3061.7149; 3066.0886; 3069.9015; 3124.8739; 3126.7667; 3129.9534; 3136.2445; 3140.4884; 3149.6016; 3153.3921; 3157.7931; 3165.6785; 3171.0173; 3180.7580; 3187.8946; 3201.7687.

Cartesian Forces: Max 0.000001424 RMS 0.000000455

Item	Value	Threshold	Converged?
Maximum Force	0.000000	0.000450	YES
RMS Force	0.000000	0.000300	YES
Maximum Displacement	0.000124	0.001800	YES
RMS Displacement	0.000017	0.001200	YES

FILE: Yb_Sq2_PhYbOTf3_TS_Anion_B3LYP_M062x_Add56_Conf8-1.out

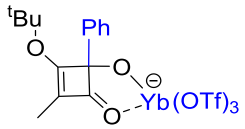

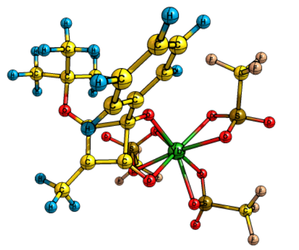

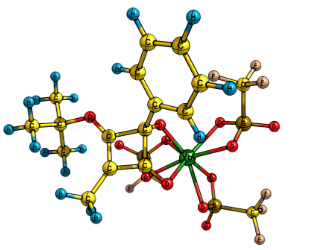

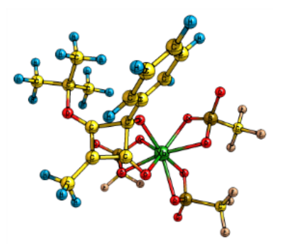

C-1 addition product from $[PhYb(OTf)_3]^-$ + 1b

Lowest energy conformation is: Conformer 15

Conformer 1

E(RB3LYP/6-31G(d)/MWB59) = -3731.53522843
Convg = 0.6057D-08 -V/T = 2.0122

Temperature 195.150 Kelvin. Pressure 1.00000 Atm.
elec. and zero-point Energies= -3731.160661
elec. and thermal Energies= -3731.137848
elec. and thermal Enthalpies= -3731.137230
elec. and thermal Free Energies= -3731.206751

RB3LYP/6-31G(d)/MWB59/THF(IEFPCM) = -3731.59848780

RM06-2x/6-311+G(d,p)/MWB59/THF(IEFPCM) = -3731.43416946

Geometry at RB3LYP/6-31G(d)/MWB59
Charge = -1 Multiplicity = 1

```
Yb  0.000327059 -0.0639837787  3.6181472302
O   1.5788615483  1.6063762006  4.4988997423
S   0.8616937594  2.8194230451  3.9828419536
O   0.5590922536  3.8985695826  4.9151962107
O  -0.2785704375  2.2991764306  3.1402941962
C   2.0702330781  3.5952538949  2.7770931796
F   2.8530647425  4.4613294843  3.4302873218
F   2.8555408656  2.6691388865  2.2109242987
F   1.4114329561  4.2504416675  1.8132129522
O  -0.4606955864  0.0966001142  6.0757090209
O  -2.6487442272 -0.960624454  6.8096659841
O  -2.2685901023  0.0043875  4.4961414031
S  -1.9568973887  0.0125422829  5.9701746843
C  -2.5696052769  1.6677944123  6.563684009
F  -2.2350771743  1.8373892034  7.8495088054
F  -2.0428008313  2.6589380855  5.8436434255
F  -3.9050700418  1.7170324333  6.4572050336
C   0.5339265037 -2.191982367  0.115872311
C  -0.7946639429 -2.4858962316  0.367792279
C  -0.9397442696 -1.2197231959  1.024013603
C   0.4471640314 -0.6920975754  0.6094807241
O   1.1198837424 -0.3160201739  1.7421805001
O  -1.6383209088 -0.7255737869  1.9265476026
O   1.4509679742 -3.0482473308 -0.2866135332
C   2.893877212 -2.7474639351 -0.4507592704
C  -1.6129534835 -3.7309545923  0.3021078173
H  -0.9971584015 -4.5809905242 -0.0089607683
H  -2.447184244 -3.6352407408 -0.4045923685
H  -2.0389302566 -3.9521286811  1.2876756957
C   3.0769328452 -1.7279113039 -1.5769539192
H   4.1451703535 -1.6229009011 -1.7992265519
H   2.5678773371 -2.059956191 -2.4887053794
H   2.6924541528 -0.7436488924 -1.301561097
C   3.4540009949 -4.1138755705 -0.8557903978
H   4.5360719606 -4.041800741 -1.0085773585
H   3.2620101678 -4.8511981078 -0.0704032919
H   2.9934229304 -4.4654922566 -1.7856954217
C   3.4900548195 -2.2772964819  0.8747204036
H   3.0431226845 -1.3372090834  1.2087119206
H   3.3236845479 -3.0218137667  1.6582110548
H   4.5700961002 -2.1326485297  0.7487650754
C   0.4594798154  0.3414466649 -0.5186291365
C  -0.2665399105  0.156656175 -1.7063295985
C   1.1908552002  1.5218466365 -0.3586018399
H  -0.8431774749 -0.7527890756 -1.8510983047
H   1.7475424475  1.6567733636  0.5572372801
C  -0.2599785692  1.1316971648 -2.7034847326
C   1.1991290361  2.4985860862 -1.3568545259
H  -0.8332001322  0.9728406438 -3.6142144734
H   1.7653010748  3.4138623479 -1.201808165
C   0.4739754687  2.3089619404 -2.5335845494
H   0.474644875  3.0708437441 -3.3095742792
O   1.9895563881 -3.7540051768  3.6420731285
S   1.2171774687 -2.7389295955  4.3584675625
C   1.0080052677 -3.4539892822  6.067945916
O  -0.1874416491 -2.4808939043  3.8705169059
O   1.8477544069 -1.391755578  4.5567877854
F   0.2870400493 -2.6570836494  6.8487371111
F   2.2172424272 -3.6316862699  6.6184204375
F   0.3989433258 -4.6443210707  5.9737426676
```

Frequency: 6.6831; 12.2486; 20.8684; 24.7910; 26.9798; 29.9790; 33.0033; 37.8223; 39.0343; 46.9020; 50.3047; 53.1477; 53.6359; 58.7796; 65.0560; 70.1080; 73.3235; 75.4942; 79.3131; 83.8025; 90.8132; 100.3836; 106.6775; 128.4088; 129.9427; 136.0851; 145.1665; 148.5121; 153.9948; 172.2311; 182.4433; 185.4257; 204.1751; 210.6535; 212.6976; 216.1929; 218.3101; 222.0924; 224.4958; 230.8049; 231.5527; 259.3282; 269.8548; 275.7161; 277.1889; 304.6018; 308.7424; 310.7123; 319.6157; 322.8817; 323.3403; 325.0883; 338.6091; 350.1192; 351.0579; 352.2396; 357.0641; 380.5712; 407.7434; 420.7693; 447.0161; 453.9117; 488.8388; 489.9571; 490.6326; 492.2205; 512.0591; 522.8340; 524.0467; 525.0127; 530.3010; 557.7349; 558.1669; 559.1892; 569.5292; 571.9416; 573.6474; 596.2488; 625.3121; 627.2823; 633.9000; 635.1281; 667.7412; 691.5925; 714.1169; 723.2766; 757.4143; 759.0481; 762.0820; 763.1720; 778.0540; 800.9629; 865.7015; 874.5615; 898.2967; 918.5772; 937.8875; 942.4398; 955.8042; 973.5783; 974.9238; 977.8608; 985.1321; 988.9661; 1001.8861; 1018.8711; 1044.2396; 1051.2682; 1072.2112; 1073.7334; 1075.7431; 1076.8879; 1104.4864; 1109.4375; 1115.9311; 1137.2190; 1186.1887; 1202.8281; 1207.8570; 1209.5355; 1211.7801; 1215.6643; 1226.0481; 1226.8644; 1230.2966; 1232.3037; 1236.2308; 1237.8190; 1247.2970; 1256.3469; 1261.9208; 1287.1071; 1291.4763; 1298.5395; 1305.7881; 1313.1034; 1346.6139; 1363.2615; 1391.8148; 1429.3773; 1440.8438; 1444.2006; 1469.3145; 1492.7970; 1503.5426; 1511.8737; 1515.5377; 1516.7413; 1521.7910; 1531.9116; 1534.0757; 1538.5715; 1553.6051; 1614.4522; 1639.9355; 1659.9146; 1766.7994; 3043.2450; 3053.2298; 3054.0840; 3061.7096; 3100.1312; 3117.0202; 3127.1269; 3128.4797; 3132.0686; 3139.5971; 3158.6459; 3162.2841; 3169.4448; 3182.3906; 3195.0026; 3202.1663; 3291.8673.

Cartesian Forces: Max 0.000000773 RMS 0.000000322

Item	Value	Threshold	Converged?
Maximum Force	0.000001	0.000450	YES
RMS Force	0.000000	0.000300	YES
Maximum Displacement	0.000078	0.001800	YES
RMS Displacement	0.000015	0.001200	YES

FILE: Yb_Sq2_PhYbOTf3_Prod_Anion_B3LYP_M062x_Add12_Conf1_a.out

Conformer 2

E(RB3LYP/6-31G(d)/MWB59) = -3731.52961031
Convg = 0.5458D-08 -V/T = 2.0122

Temperature 195.150 Kelvin. Pressure 1.00000 Atm.
elec. and zero-point Energies= -3731.155122
elec. and thermal Energies= -3731.132306
elec. and thermal Enthalpies= -3731.131688
elec. and thermal Free Energies= -3731.200882

RB3LYP/6-31G(d)/MWB59/THF(IEFPCM) = -3731.59676085

RM06-2x/6-311+G(d,p)/MWB59/THF(IEFPCM) = -3731.43340935

Geometry at RB3LYP/6-31G(d)/MWB59
Charge = -1 Multiplicity = 1

```
Yb  0.1216433542 -0.2087719383  3.5742442884
O   1.7538800379  1.5396128808  4.0557793774
S   0.9451138821  2.6930298749  3.5326177827
O   0.8110029525  3.8764450729  4.3740623135
O  -0.329092094  2.0892410559  2.9865666529
C   1.876864805  3.2947183133  2.0311586566
F   2.9609354725  3.9733752912  2.433049139
F   2.2642898395  2.2861313035  1.2563479311
F   1.0922941541  4.1193906586  1.3227618756
O  -0.0502924656  0.0919144392  6.0529043175
O  -2.1010163483 -0.9404823043  7.1348153563
O  -2.0345758373 -0.1764727324  4.719018726
S  -1.545251884 -0.0324057201  6.1360013031
C  -2.1285584862  1.6575507994  6.6575001566
F  -1.6732397768  1.9317618795  7.8871560963
F  -1.6927700905  2.595654753  5.8152158181
F  -3.4688453547  1.6846656314  6.6804480121
C   0.1675067185 -2.2997279273  0.0253808498
C  -1.1842135741 -2.44268285  0.2979337407
C  -1.1275767367 -1.1942769323  1.021161092
C   0.2912936333 -0.8396844937  0.5665792841
O   1.0734012311 -0.6183887139  1.6702854508
O  -1.7195018868 -0.6994927794  1.9972741853
O   1.2070628604 -2.9931161953 -0.3875412421
C   1.2569804721 -4.4515209212 -0.6149377195
C  -2.2872294508 -3.4498969948  0.234030624
H  -2.1629964935 -4.1770954274 -0.5734156304
H  -3.2432321733 -2.9369844384  0.0792860445
H  -2.3706997881 -3.999137905  1.180993969
C   0.6936001166 -5.2137640972  0.5851928453
H   0.9159459732 -6.280434257  0.4625559661
H   1.1565925558 -4.8662543058  1.5133957496
H  -0.3891596757 -5.1111692266  0.6748066502
C   2.7619447616 -4.7039401435 -0.7445899887
H   2.9495642575 -5.7611884189 -0.9617147273
H   3.1856633593 -4.1003611158 -1.5540708638
H   3.2681568651 -4.4401939974  0.1884679685
C   0.5330256313 -4.7615799385 -1.9283268223
H  -0.5303026303 -4.5125273737 -1.8725539828
H   0.9751669365 -4.1931484641 -2.7535111616
H   0.6185321168 -5.829539925 -2.1595456798
C   0.4447204015  0.2144796953 -0.5201952085
C   1.4002719924  0.0675051311 -1.5339755046
C  -0.3358552598  1.3779791329 -0.4892231559
H   2.0174074809 -0.8257493268 -1.5574351536
H  -1.0622450628  1.5143257239  0.3065572072
C   1.5664025851  1.058853422 -2.5029841765
C  -0.1694009656  2.3697038878 -1.4569259986
H   2.3136736363  0.9313188489 -3.2833026205
H  -0.7791335338  3.2689134681 -1.4150231456
C   0.7801272303  2.2124380818 -2.4691241833
H   0.9110041973  2.9863725986 -3.2220223443
O   2.0479556643 -3.9244360275  3.4808141352
S   1.3425090626 -2.8952598859  4.2479893656
C   1.2355923152 -3.6056889991  5.9679474402
O  -0.0911838763 -2.625726681  3.8625067294
O   2.0066882227 -1.5623893865  4.4108248891
F   0.5680416305 -2.79823211  6.7862562359
F   2.4726893344 -3.793351022  6.4479221525
F   0.6102327967 -4.7913519451  5.9173215263
```

Frequency: 14.6590; 15.7396; 17.5039; 23.7572; 27.4440; 31.1417; 32.3922; 33.6233; 35.1455; 36.3984; 46.0778; 51.7379; 52.6953; 58.0853; 59.4978; 68.4553; 73.6916; 81.1604; 84.6609; 90.1213; 98.1597; 112.1229; 126.0871; 129.5187; 135.4335; 138.4708; 148.2979; 153.6863; 155.3792; 167.4800; 177.0573; 180.3018; 205.3774; 207.3825; 211.1724; 215.4105; 218.0884; 219.2387; 220.7817; 224.5725; 232.2586; 245.8735; 258.5846; 272.3065; 291.6800; 304.1186; 306.7597; 309.3772; 310.6481; 321.3486; 323.1776; 324.8679; 330.7648; 350.3625; 351.4197; 352.8630; 356.5282; 368.3750; 399.9173; 411.9687; 437.9372; 459.2548; 474.9800; 489.5475; 490.1817; 491.9887; 515.7027; 522.9254; 525.3290; 529.3175; 547.0797; 557.7736; 558.5743; 559.0706; 570.2034; 573.2027; 574.0742; 625.3540; 626.8919; 631.6403; 632.3415; 636.8738; 676.0627; 681.5230; 710.7124; 724.9668; 761.3229; 761.6035; 762.2748; 772.0393; 792.3983; 804.9981; 855.1184; 861.0801; 898.1507; 932.4307; 935.7243; 938.7743; 967.5138; 968.5336; 973.9036; 977.2957; 983.5726; 983.9594; 984.9640; 1018.4485; 1035.6688; 1054.5969; 1061.5407; 1065.3546; 1070.8928; 1086.8216; 1106.1492; 1116.2472; 1118.5403; 1138.7425; 1189.4183; 1204.9861; 1207.1412; 1208.8673; 1211.1530; 1215.6625; 1216.8609; 1225.7743; 1228.4488; 1229.7666; 1232.6406; 1246.0601; 1257.2057; 1257.7041; 1278.2937; 1285.3362; 1287.7484; 1296.3561; 1304.6299; 1311.9748; 1342.2362; 1371.0607; 1388.0384; 1428.8784; 1436.7597; 1443.3058; 1462.4152; 1493.2612; 1502.7603; 1509.1352; 1516.6949; 1520.3574; 1522.8277; 1529.6414; 1534.7608; 1545.9959; 1554.5400; 1622.0465; 1639.5744; 1662.5516; 1761.0678; 3044.8974; 3056.4968; 3059.5479; 3067.3453; 3100.3609; 3122.1036; 3128.6138; 3129.7364; 3137.4891; 3145.2104; 3147.2218; 3167.6021; 3175.9984; 3181.6320; 3194.9219; 3210.7067; 3214.3477.

Cartesian Forces: Max 0.000000990 RMS 0.000000287

Item	Value	Threshold	Converged?
Maximum Force	0.000001	0.000450	YES
RMS Force	0.000000	0.000300	YES
Maximum Displacement	0.000071	0.001800	YES
RMS Displacement	0.000013	0.001200	YES

FILE: Yb_Sq2_PhYbOTf3_Prod_Anion_B3LYP_M062x_Add12_Conf1-1_a.out

Conformer 3

E(RB3LYP/6-31G(d)/MWB59) = -3731.53962984
Convg = 0.7584D-08 -V/T = 2.0122

Temperature 195.150 Kelvin. Pressure 1.00000 Atm.
elec. and zero-point Energies= -3731.165034
elec. and thermal Energies= -3731.142327
elec. and thermal Enthalpies= -3731.141709
elec. and thermal Free Energies= -3731.210018

RB3LYP/6-31G(d)/MWB59/THF(IEFPCM) = -3731.60214923

RM06-2x/6-311+G(d,p)/MWB59/THF(IEFPCM) = -3731.43995774

Geometry at RB3LYP/6-31G(d)/MWB59
Charge = -1 Multiplicity = 1

```
Yb -0.0472670279 -0.1518119347  3.6819920973
O  -0.5825996388  2.1583026945  3.2523065186
S   0.7663618316  2.7565155065  3.5779476087
O   1.3648019855  3.6316053178  2.5728081353
O   1.6024400079  1.6336111046  4.1171750361
C   0.4585702569  3.8882385691  5.0306164289
F  -0.4725106299  4.7925210537  4.7010835422
F   0.0510018552  3.2163899437  6.1064164808
F   1.5963749347  4.5296306053  5.3302164447
O  -0.4553280081  0.3022844491  6.0887013492
O  -2.4083390556 -0.9289841147  7.1337668256
O  -2.286103646 -0.3412726512  4.6686236615
S  -1.9233753975 -0.0271801899  6.0938455616
C  -2.7873696004  1.5876729655  6.4511116391
F  -2.3360259261  2.095213971  7.6057304372
F  -2.5854644177  2.4798958749  5.4785071739
F  -4.1049950469  1.3696820807  6.5630836085
C   0.515343611 -2.1634362537  0.0960704557
C  -0.8221239321 -2.4503090241  0.3077764921
C  -0.9549546577 -1.2274457901  1.0429380116
C   0.4431705291 -0.6926522767  0.6743257064
O   1.1053052421 -0.3922726384  1.8321914935
O  -1.6530938455 -0.7916056249  1.9779584022
O   1.4274953015 -3.0122681301 -0.3322323807
C   2.8805958137 -2.7285543223 -0.4370259583
C  -1.6586100134 -3.6752902065  0.1518461007
H  -1.0391082671 -4.5311706097 -0.1354952421
H  -2.4356130782 -3.5454081549 -0.6126002061
H  -2.1621883496 -3.910769094  1.096547082
C   3.1144822632 -1.6347649614 -1.4807772473
H   4.1903973299 -1.5381412198 -1.6667751379
H   2.624053782 -1.8885765245 -2.4271887108
H   2.7432698793 -0.6645285632 -1.1444680559
C   3.4269744628 -4.0734510646 -0.9242097918
H   4.5140240912 -4.012045511 -1.041866243
H   3.2002584742 -4.8617949952 -0.1998526977
H   2.9869876987 -4.3484163253 -1.8892602702
C   3.4454775504 -2.3652959774  0.9348076692
H   3.0079691865 -1.4402798821  1.3197045233
H   3.2440606254 -3.1574579788  1.6616116826
H   4.5314099435 -2.2356359344  0.8506363131
C   0.5009352826  0.4067062284 -0.3884700235
C  -0.1845339445  0.2976581827 -1.6091413312
C   1.2394955575  1.5674480175 -0.1336168056
H  -0.7657481472 -0.5950611595 -1.8241212635
H   1.7598154248  1.6602220105  0.8111106594
C  -0.1308912699  1.3255462468 -2.5499462227
C   1.2925333221  2.5972351831 -1.0760472046
H  -0.6722824731  1.2250182552 -3.4881709499
H   1.8596707472  3.4957962518 -0.8478029208
C   0.6103370096  2.4810877052 -2.2874580131
H   0.6480688136  3.28490765 -3.0191231998
O   2.1245639959 -3.7442014509  3.8641874756
S   1.2744519856 -2.7465461827  4.5121658192
C   1.0172601625 -3.4079455693  6.2353659375
O  -0.1194453143 -2.5705132231  3.9624203269
O   1.8305489954 -1.364616552  4.6990387955
F   0.2277213731 -2.6066490434  6.9456124239
F   2.2028278592 -3.5136903167  6.8507603409
F   0.4585125497 -4.6237880025  6.1651298762
```

Frequency: 15.4805; 20.0415; 21.4133; 24.4773; 28.1130; 32.3510; 35.2194; 37.7990; 44.6997; 47.5697; 49.5718; 53.0114; 58.2497; 60.6728; 68.9334; 70.8869; 75.1502; 75.5925; 82.0302; 85.3945; 91.4780; 98.7051; 106.6093; 130.3467; 133.3491; 139.3427; 143.4819; 150.6840; 151.9189; 174.8415; 183.9723; 185.1576; 205.2843; 208.7965; 212.6326; 216.8769; 218.6499; 218.7626; 222.8431; 226.6506; 231.6584; 258.8312; 269.4001; 279.0612; 280.8976; 302.7677; 307.7408; 308.7033; 321.5058; 321.9934; 324.3976; 325.3962; 339.8173; 350.0192; 350.6623; 353.2236; 357.2035; 381.3268; 408.8200; 422.3658; 448.4514; 453.5544; 489.2971; 490.1620; 491.1041; 492.2019; 514.0775; 522.8983; 524.4961; 524.8780; 531.2144; 558.3617; 558.8340; 559.1805; 569.4882; 572.4112; 575.4243; 596.0736; 621.0749; 627.2185; 633.1798; 637.3950; 666.1253; 692.7604; 714.5537; 723.0739; 758.6492; 760.3096; 761.7311; 762.7161; 778.2546; 801.2033; 865.4795; 873.9555; 898.7286; 917.8333; 937.8283; 942.1753; 955.3476; 972.2591; 973.5783; 977.4335; 986.7598; 987.8585; 1005.1576; 1017.0987; 1043.5632; 1050.5800; 1071.6839; 1073.5656; 1075.3708; 1077.7090; 1106.3747; 1112.4149; 1114.1269; 1136.4536; 1186.6346; 1202.7938; 1208.4156; 1210.8603; 1212.0468; 1214.2561; 1226.3786; 1228.5184; 1228.8348; 1232.9657; 1240.1703; 1246.9983; 1253.3634; 1255.2133; 1257.5301; 1286.7875; 1291.8451; 1296.9105; 1299.7895; 1311.0414; 1344.8540; 1362.2840; 1391.8401; 1429.6984; 1441.1653; 1444.2025; 1469.3104; 1491.8304; 1503.6194; 1511.2669; 1515.5553; 1517.0977; 1521.6676; 1531.8625; 1534.3927; 1537.8101; 1553.5017; 1610.7362; 1638.8215; 1658.8011; 1761.1438; 3041.8416; 3053.4195; 3054.1569; 3061.7800; 3098.5424; 3117.2863; 3127.2343; 3127.4509; 3130.6966; 3138.4710; 3156.6425; 3163.2085; 3168.0614; 3183.6937; 3196.1780; 3202.9541; 3257.4512.

Cartesian Forces: Max 0.000000649 RMS 0.000000292

Item	Value	Threshold	Converged?
Maximum Force	0.000000	0.000450	YES
RMS Force	0.000000	0.000300	YES
Maximum Displacement	0.000030	0.001800	YES
RMS Displacement	0.000006	0.001200	YES

FILE: Yb_Sq2_PhYbOTf3_Prod_Anion_B3LYP_M062x_Add12_Conf2_a.out

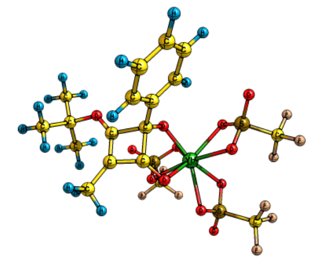

Conformer 4

E(RB3LYP/6-31G(d)/MWB59) = -3731.53267675
Convg = 0.5096D-08 -V/T = 2.0122

Temperature 195.150 Kelvin. Pressure 1.00000 Atm.
elec. and zero-point Energies= -3731.158091
elec. and thermal Energies= -3731.135402
elec. and thermal Enthalpies= -3731.134784
elec. and thermal Free Energies= -3731.203008

RB3LYP/6-31G(d)/MWB59/THF(IEFPCM) = -3731.59919090

RM06-2x/6-311+G(d,p)/MWB59/THF(IEFPCM) = -3731.43592688

Geometry at RB3LYP/6-31G(d)/MWB59
Charge = -1 Multiplicity = 1
Yb -0.0347469331 -0.2449012291 3.606436525
O -0.5803238778 2.0579625976 3.1571380625
S 0.7837883724 2.6532880217 3.4191929216
O 1.3482775295 3.5082671666 2.3791354911
O 1.6322176143 1.5324001531 3.9450609295
C 0.53787036 3.8067652938 4.8659681818
F -0.3868803549 4.7235914753 4.5525838632
F 0.1489836116 3.1544751983 5.9608913154
F 1.6941963976 4.432009512 5.1261773202
O -0.3333452862 0.2329302413 6.0389622313
O -2.2245569075 -1.0025241142 7.1882997237
O -2.2140972399 -0.462497821 4.7098331592
S -1.7938576665 -0.1155293873 6.1120085429
C -2.6648242579 1.4947655703 6.4706656387
F -2.1783060745 2.0305810098 7.5980124194
F -2.5093285984 2.3698531182 5.4741916576
F -3.9749916786 1.2641589522 6.6352704697
C 0.2382991915 -2.2777795179 0.0454336685
C -1.1160671447 -2.4809170724 0.275447393
C -1.1375275178 -1.2337446637 1.0034021095
C 0.2826052196 -0.8169792633 0.5962279777
O 1.0063957719 -0.5754218086 1.730019229
O -1.7830414213 -0.776580156 1.9628359546
O 1.3159724648 -2.9107973095 -0.3565660676
C 1.4266787133 -4.3608736889 -0.6051559277
C -2.1828345441 -3.5230070526 0.1544609563
H -1.8489248855 -4.5230816167 0.4483750526
H -2.5765134003 -3.5917258362 -0.8685792775
H -3.0136980679 -3.2467737527 0.8124373297
C 1.1099588055 -5.1293017181 0.6784488472
H 1.3097175863 -6.1960706254 0.5223064381
H 1.7260561567 -4.7710005313 1.507661042
H 0.064102761 -5.0226445742 0.9745523201
C 2.9053297992 -4.512456168 -0.973209965
H 3.1343106996 -5.5619007768 -1.1882262716
H 3.146198353 -3.9124899753 -1.8569184822
H 3.5361343941 -4.1777275799 -0.1447431761
C 0.5329375642 -4.7481250879 -1.7871742859
H -0.5283221828 -4.6289785849 -1.5573998422
H 0.7706917285 -4.1341145652 -2.6625121189
H 0.7026083032 -5.7989437733 -2.0484241626
C 0.4540927121 0.2353409329 -0.4994237299
C -0.2534589623 0.1748885477 -1.7096906414
C 1.3417657028 1.2952828591 -0.290369396
H -0.9493146624 -0.6406640241 -1.8894474507
H 1.8824566923 1.3438933314 0.6466513268
C -0.077277966 1.1539895918 -2.6871906157
C 1.5178311737 2.2760879868 -1.2686807078
H -0.638114162 1.0935984038 -3.617585066
H 2.2025532822 3.0987346982 -1.0793538085
C 0.8117273869 2.2101256389 -2.470352197
H 0.9463955939 2.9768260052 -3.2300788521
O 2.1459193359 -3.8301129385 3.6812269178
S 1.3042760839 -2.8504496567 4.3688485127
C 1.0997165548 -3.5451796051 6.0856441069
O -0.1063388953 -2.6822380447 3.8631222474
O 1.8549464243 -1.4698883752 4.5707805067
F 0.3165175076 -2.7669865229 6.8284472028
F 2.3000280341 -3.647396818 6.6718740468
F 0.5538703826 -4.7674718284 6.0076329881

Frequency: 15.4496; 19.6488; 23.3652; 24.3038; 29.4581; 31.9459; 33.4499; 37.4508; 40.8372; 45.0119; 46.3352; 50.2220; 57.3895; 64.2011; 70.8615; 72.1325; 74.6877; 83.8655; 86.4034; 93.0432; 99.2614; 106.6375; 127.7968; 131.1737; 133.0796; 140.3410; 142.4553; 145.1725; 154.7005; 167.9722; 176.6763; 181.9332; 190.9777; 207.3984; 209.8060; 215.8123; 218.7288; 218.9710; 222.4660; 227.0183; 238.3235; 250.8122; 261.0122; 262.7927; 283.0331; 302.7870; 307.4534; 308.8206; 320.4384; 321.5736; 323.5613; 325.1235; 335.7411; 350.2605; 353.1520; 354.2534; 357.2627; 392.6699; 396.9771; 420.8737; 455.6526; 459.4204; 484.0645; 489.1351; 491.1445; 492.0791; 499.2298; 522.7446; 524.1882; 527.5148; 534.4798; 558.2578; 558.7885; 559.0742; 569.5692; 572.0789; 575.4589; 620.9244; 626.7535; 633.3627; 636.6614; 637.7411; 684.6023; 692.0698; 713.0539; 728.6820; 754.1320; 760.1220; 761.5772; 762.2419; 784.4391; 811.2730; 857.7439; 862.3418; 896.1301; 919.8568; 934.4845; 937.4317; 967.3077; 971.6882; 973.5711; 977.7357; 979.8545; 986.9787; 1004.1433; 1017.4523; 1030.3305; 1051.9763; 1062.4190; 1068.4694; 1070.6315; 1084.5042; 1106.1515; 1112.9582; 1115.5750; 1138.9704; 1186.6737; 1204.2521; 1207.0126; 1210.1577; 1211.2744; 1212.6901; 1214.6431; 1226.1874; 1228.5789; 1231.9609; 1240.8127; 1245.6135; 1247.1056; 1252.3370; 1256.3783; 1283.9418; 1290.2844; 1295.5527; 1299.5786; 1311.6395; 1347.0367; 1363.0899; 1386.7596; 1429.8594; 1436.0667; 1444.4017; 1462.1587; 1493.0495; 1501.7199; 1512.7949; 1516.8837; 1520.1810; 1522.3318; 1529.5223; 1531.9439; 1539.6427; 1551.3871; 1613.6480; 1640.3899; 1660.4618; 1759.8171; 3038.1370; 3057.9989; 3060.7786; 3068.1556; 3100.0514; 3121.8458; 3126.3941; 3131.0659; 3141.0126; 3146.1971; 3159.1341; 3165.0196; 3168.2173; 3180.4856; 3192.1942; 3200.3849; 3252.7261.

Cartesian Forces: Max 0.000001104 RMS 0.000000421

Item	Value	Threshold	Converged?
Maximum Force	0.000000	0.000450	YES
RMS Force	0.000000	0.000300	YES
Maximum Displacement	0.000022	0.001800	YES
RMS Displacement	0.000005	0.001200	YES

FILE: Yb_Sq2_PhYbOTf3_Prod_Anion_B3LYP_M062x_Add12_Conf2-1_a.out

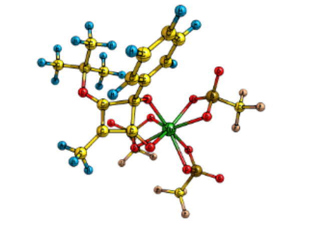

Conformer 5

E(RB3LYP/6-31G(d)/MWB59) = -3731.53988458
Convg = 0.7236D-08 -V/T = 2.0122

Temperature 195.150 Kelvin. Pressure 1.00000 Atm.
elec. and zero-point Energies= -3731.165293
elec. and thermal Energies= -3731.142575
elec. and thermal Enthalpies= -3731.141957
elec. and thermal Free Energies= -3731.210287

RB3LYP/6-31G(d)/MWB59/THF(IEFPCM) = -3731.60241857

RM06-2x/6-311+G(d,p)/MWB59/THF(IEFPCM) = -3731.44003061

Geometry at RB3LYP/6-31G(d)/MWB59
Charge = -1 Multiplicity = 1
Yb 0.093476254 -0.2042153452 3.7015562234
O -0.4999229322 2.0942353911 3.2192478663
S 0.8187533179 2.7223305159 3.5990818045
O 1.4445855009 3.6154674777 2.6270169986
O 1.6588799969 1.6126255497 4.1670590972
C 0.4129218011 3.8166033172 5.0517448788
F -0.3966383365 4.8052446123 4.6508943136
F -0.1876336886 3.126508037 6.0185692387
F 1.5429369921 4.3522777418 5.5324152686
O -2.1415137014 -0.1185238788 4.7055250368
O -2.376082586 1.0902927852 6.9250779809
O -0.2258595587 -0.0104600087 6.1552286277
S -1.729608889 0.0417675028 6.1429299553
C -2.2756601541 -1.5442813875 6.9588581758
F -3.6124013964 -1.5588305532 7.0536951467
F -1.8868983321 -2.6132924769 6.25905668
F -1.7564635209 -1.6212300134 8.191334211
C 0.6179469676 -2.1885056998 0.0813383051
C -0.6819599948 -2.5725814774 0.3617453698
C -0.8633278548 -1.3667195305 1.114454263
C 0.4719256436 -0.7308240945 0.6782858266
O 1.1726564004 -0.3943242582 1.8033408845
O -1.542278748 -0.985611992 2.0866851038
O 1.5630599803 -2.965166217 -0.4078107353
C 2.9870129883 -2.5811035679 -0.5746410761
C -1.4354952202 -3.8536483514 0.2364972139
H -0.7718535852 -4.660640716 -0.0909638135
H -2.2585484801 -3.7753836112 -0.4856195184
H -1.8709768777 -4.1312855274 1.2033570161
C 3.0969787226 -1.4575080223 -1.6069972004
H 4.1538383126 -1.2857523094 -1.841646734
H 2.5792088066 -1.7286379602 -2.5338243656
H 2.6788793021 -0.5198686632 -1.2352255799
C 3.5990084286 -3.8777254152 -1.1121542105
H 4.6725962859 -3.740471599 -1.2790650165
H 3.4606130021 -4.6911145771 -0.3932265442
H 3.1330849738 -4.1667199288 -2.0607967264
C 3.5898356225 -2.2022325724 0.7767582669
H 3.1111907052 -1.313845204 1.1973888989
H 3.4724391205 -3.0170946281 1.4968523222
H 4.660143996 -2.0007296087 0.6464772462
C 0.3960560405 0.377302676 -0.3742869117
C -0.345722729 0.2288211407 -1.5572166241
C 1.0670876565 1.584358436 -0.1489501854
H -0.8761449329 -0.7001119957 -1.7489285809
H 1.6319498399 1.7080890334 0.766141726
C -0.4130109617 1.2632084925 -2.49008609
C 0.9997956797 2.6201767631 -1.0837466003
H -0.9967777929 1.131286152 -3.39852998
H 1.5177801064 3.5533541626 -0.8785817756
C 0.2618835312 2.4647980876 -2.2575809158
H 0.2057260785 3.2732166815 -2.9829536711
O 2.362311177 -3.7309704413 3.6840353496
S 1.5562175303 -2.7611462068 4.4245752509
C 1.485494162 -3.4683928632 6.1498355213
O 0.1139883834 -2.6157387697 4.0033657085
O 2.0943258114 -1.3718207925 4.5844807154
F 0.8513310048 -2.6555436326 6.9936789977
F 2.7345652122 -3.6621196719 6.5947794643
F 0.8506004746 -4.648091105 6.121379944

Frequency: 14.9371; 19.0284; 23.0893; 25.3025; 29.2361; 32.4785; 32.8295; 39.6214; 42.8720; 50.1968; 52.0899; 53.0825; 57.9653; 62.9115; 66.1271; 71.4927; 73.3104; 74.7022; 75.9561; 83.0158; 92.1767; 99.1865; 106.6576; 130.6339; 132.8277; 137.6042; 142.0413; 150.5396; 152.5182; 174.1387; 183.2565; 187.6744; 206.6179; 208.5277; 213.8802; 216.0017; 217.6442; 218.4716; 221.6196; 225.5572; 231.9131; 258.3648; 270.4322; 281.2428; 282.7843; 302.5043; 307.5211; 308.6117; 320.7251; 322.0646; 324.3085; 325.3798; 339.5688; 350.3529; 350.7943; 353.1459; 357.2978; 381.7563; 409.1846; 422.4639; 447.4187; 453.9081; 489.0368; 490.5630; 490.9837; 491.8478; 514.4026; 522.5990; 524.1074; 525.1741; 531.1244; 558.1515; 558.5821; 559.0398; 569.5521; 572.4882; 575.3813; 595.8024; 621.2482; 627.0364; 633.2761; 636.8898; 666.5046; 692.4806; 714.6230; 723.2881; 758.6687; 760.1263; 761.6696; 762.8296; 778.1216; 801.1939; 865.5242; 873.9537; 898.6358; 917.7992; 938.0160; 942.1958; 955.3917; 972.1120; 975.1233; 976.3429; 986.9561; 988.4627; 1005.2215; 1016.9528; 1043.6327; 1050.4970; 1071.8822; 1073.8522; 1075.3094; 1077.5095; 1106.5420; 1108.7405; 1118.9007; 1136.7852; 1186.6454; 1202.8330; 1208.5360; 1211.6788; 1212.6691; 1215.0088; 1226.6931; 1228.4120; 1228.7317; 1234.5145; 1239.7631; 1245.0540; 1252.4349; 1255.5189; 1256.5135; 1286.9137; 1290.5157; 1297.6364; 1300.1644; 1311.3120; 1344.6990; 1362.1827; 1391.9876; 1429.6158; 1441.3148; 1444.2071; 1469.3839; 1491.8188; 1503.6178; 1511.2923; 1515.7890; 1517.0559; 1522.0905; 1532.0098; 1534.4726; 1537.8805; 1553.9527; 1610.8984; 1638.8852; 1658.7833; 1761.5328; 3041.7483; 3053.3192; 3054.1293; 3061.7848; 3098.2834; 3117.3377; 3127.0625; 3127.3351; 3130.7041; 3138.3355; 3156.6516; 3163.6023; 3168.2111; 3183.7112; 3196.0110; 3202.8588; 3258.0968.

Cartesian Forces: Max 0.000001344 RMS 0.000000466

Item	Value	Threshold	Converged?
Maximum Force	0.000000	0.000450	YES
RMS Force	0.000000	0.000300	YES
Maximum Displacement	0.000045	0.001800	YES
RMS Displacement	0.000008	0.001200	YES

FILE: Yb_Sq2_PhYbOTf3_Prod_Anion_B3LYP_M062x_Add12_Conf3_a.out

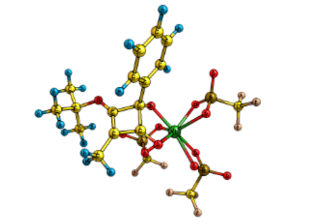

Conformer 6

E(RB3LYP/6-31G(d)/MWB59) = -3731.53264472
Convg = 0.6289D-08 -V/T = 2.0122

Temperature 195.150 Kelvin. Pressure 1.00000 Atm.
elec. and zero-point Energies= -3731.158154
elec. and thermal Energies= -3731.135416
elec. and thermal Enthalpies= -3731.134798
elec. and thermal Free Energies= -3731.203223

RB3LYP/6-31G(d)/MWB59/THF(IEFPCM) = -3731.59950873

RM06-2x/6-311+G(d,p)/MWB59/THF(IEFPCM) = -3731.43611944

Geometry at RB3LYP/6-31G(d)/MWB59
Charge = -1 Multiplicity = 1
Yb 0.0897313492 -0.2732897922 3.6235786532
O -0.509301923 2.0087957679 3.0665798188
S 0.8147566391 2.6437657805 3.4131099936
O 1.4304138519 3.5151539163 2.4168137631
O 1.6597635003 1.5439711377 3.9961458144
C 0.4287409343 3.7647459066 4.8504737339
F -0.3820210273 4.7495177264 4.4422107766
F -0.1650712843 3.0923293572 5.8348385042
F 1.5645619422 4.304591169 5.3118570038
O -2.0861587278 -0.1506689707 4.7518975399
O -2.190919325 1.0436784127 6.9884923304
O -0.0988096815 -0.0835432526 6.1053300426
S -1.5990103589 -0.0079737348 6.1678037013
C -2.1317991226 -1.5909395895 6.9985547002
F -3.4617466053 -1.5806027767 7.1663712582
F -1.8041911072 -2.6613445019 6.2696507644
F -1.5483542539 -1.6909099557 8.2003631607
C 0.30715131 -2.3044058404 0.0294595668
C -1.0152732149 -2.6000781074 0.3332003045
C -1.0756739838 -1.3692452096 1.0866400848
C 0.2889803239 -0.8540620208 0.6092023129
O 1.0601563914 -0.5868697228 1.7057854686
O -1.696397735 -0.9671227736 2.0860238649
O 1.3967888849 -2.8559091511 -0.452219718
C 1.5831096087 -4.2872856614 -0.7561239508
C -2.0196686501 -3.7056143028 0.2457731475
H -1.6104893233 -4.6867560451 0.5067773264
H -2.4578910616 -3.7852845773 -0.7581611678
H -2.8333817289 -3.4911908145 0.9468465768
C 1.3880148924 -5.1152553549 0.5146458395
H 1.6693027841 -6.1564003604 0.3167427266
H 2.006332368 -4.7285912212 1.3291283514
H 0.3506011417 -5.1070860636 0.8560084403
C 3.0452056758 -4.3319483496 -1.2092537335
H 3.3255561816 -5.3563510722 -1.4775694796
H 3.1984457997 -3.6864707931 -2.0802742752
H 3.7003178888 -3.9876549325 -0.4038387512
C 0.6492332224 -4.6937062616 -1.8999734457
H -0.402532716 -4.6456267082 -1.6084732788
H 0.800389549 -4.0412132556 -2.766636783
H 0.8654076198 -5.7243464623 -2.2038485015
C 0.3302188541 0.2246906321 -0.4736506693
C -0.4558135423 0.1481440209 -1.6334410161
C 1.1765147685 1.3248840309 -0.3053213453
H -1.1212738195 -0.6986108134 -1.7812632629
H 1.7789523973 1.3860553129 0.592424151
C -0.3972020484 1.1511101009 -2.6008939086
C 1.2354379081 2.3293084189 -1.2735587203
H -1.0183368227 1.0778567798 -3.4911886633
H 1.890002786 3.1824048192 -1.1149198404
C 0.4511911771 2.2473849872 -2.4247854875
H 0.4939704627 3.0325603621 -3.1763112495
O 2.3256466358 -3.8085351125 3.5138859666
S 1.556435983 -2.8384134078 4.294084914
C 1.5597064602 -3.5609903235 6.0143661704
O 0.0963801012 -2.6902717996 3.9401520338
O 2.1080657187 -1.4551981725 4.4432254974
F 0.9605265546 -2.7578112038 6.8921663152
F 2.8260911321 -3.7585797688 6.4055110532
F 0.9246583307 -4.741908691 6.0015919444

Frequency: 14.4027; 18.1392; 21.4960; 26.7876; 29.1166; 31.8070; 34.1190; 38.0540; 40.1708; 46.0406; 48.7858; 52.1592; 56.0831; 61.6927; 66.1053; 71.9038; 77.6399; 79.7689; 86.1624; 92.9410; 96.2909; 101.2454; 126.7582; 130.3990; 132.1651; 139.4471; 142.2780; 145.4033; 153.4515; 167.7724; 175.4572; 181.5913; 189.1483; 207.6759; 209.9827; 215.1645; 217.7061; 218.4272; 221.4652; 227.2519; 235.1131; 251.1913; 260.1887; 261.5244; 285.9670; 302.6079; 307.2169; 308.6760; 320.2844; 320.7055; 322.7664; 325.9549; 335.6189; 350.2171; 352.9406; 353.6189; 357.5615; 391.9407; 397.0523; 420.5909; 454.5303; 457.8334; 483.4314; 489.1533; 490.5495; 491.8747; 499.6388; 522.5159; 524.5424; 527.5044; 534.0376; 557.9789; 558.4780; 559.0868; 569.1766; 572.6931; 575.3676; 620.8076; 627.0268; 633.4212; 636.0747; 637.5994; 684.1844; 691.2252; 713.0565; 728.4494; 754.6761; 759.8176; 761.2800; 762.5504; 784.0104; 811.6601; 857.5374; 862.3069; 896.0493; 919.8548; 934.2956; 937.7118; 967.1882; 971.9563; 974.9735; 976.5655; 979.4475; 987.0786; 1003.9284; 1017.4251; 1030.5827; 1051.8326; 1061.9010; 1068.3444; 1070.1029; 1084.2798; 1106.0271; 1108.2050; 1121.4787; 1138.7483; 1186.5577; 1204.1332; 1207.0149; 1210.4487; 1212.1501; 1213.1725; 1215.2363; 1225.7831; 1227.1256; 1234.6700; 1240.8176; 1243.6626; 1245.9444; 1251.2712; 1255.3659; 1283.6829; 1289.1006; 1295.3669; 1302.2203; 1311.5276; 1347.0081; 1362.8798; 1386.6520; 1429.6476; 1435.8315; 1444.2822; 1461.9315; 1493.0289; 1501.4838; 1512.5332; 1516.9841; 1519.2892; 1521.9875; 1529.3811; 1531.5986; 1539.5047; 1550.4194; 1613.9936; 1640.4932; 1660.3993; 1760.7537; 3038.1941; 3058.0238; 3060.7955; 3068.3256; 3099.6642; 3122.0446; 3125.7670; 3131.3625; 3141.7587; 3145.9064; 3157.9260; 3165.2389; 3169.2877; 3180.6822; 3192.2157; 3200.6825; 3253.0301.

Cartesian Forces: Max 0.000000896 RMS 0.000000311

Item	Value	Threshold	Converged?
Maximum Force	0.000000	0.000450	YES
RMS Force	0.000000	0.000300	YES
Maximum Displacement	0.000018	0.001800	YES
RMS Displacement	0.000003	0.001200	YES

FILE: Yb_Sq2_PhYbOTf3_Prod_Anion_B3LYP_M062x_Add12_Conf3-1_a.out

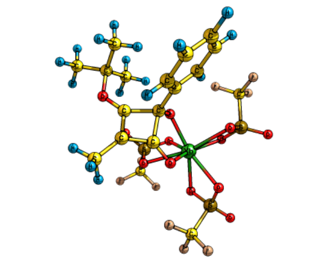

Conformer 7

E(RB3LYP/6-31G(d)/MWB59) = -3731.53685397
Convg = 0.2912D-08 -V/T = 2.0122

Temperature 195.150 Kelvin. Pressure 1.00000 Atm.
elec. and zero-point Energies= -3731.162399
elec. and thermal Energies= -3731.139592
elec. and thermal Enthalpies= -3731.138974
elec. and thermal Free Energies= -3731.208010

RB3LYP/6-31G(d)/MWB59/THF(IEFPCM) = -3731.60014507

RM06-2x/6-311+G(d,p)/MWB59/THF(IEFPCM) = -3731.43762911

Geometry at RB3LYP/6-31G(d)/MWB59
Charge = -1 Multiplicity = 1
Yb 0.2998879922 -0.150105306 3.8161913698
O 1.3698633611 1.6957365878 4.8893596573
S 0.9097225849 2.8141879772 3.9830697227
O 0.4165904113 4.0333809394 4.6099556028
O 0.0785241443 2.1708028461 2.9148283245
C 2.4764752482 3.3294965681 3.1124191963
F 3.2815188965 3.9792541846 3.9600281615
F 3.1318979545 2.2626437384 2.633456957
F 2.1821992145 4.1423122705 2.0868053781
O -1.7886828853 0.7220904861 4.7496574458
O -2.0558441919 0.7989945999 7.2721140532
O -0.4675731574 -0.7820739538 6.0803595233
S -1.7378497726 0.0215882383 6.0805094479
C -3.0796827299 -1.2659371339 5.9744118088
F -4.2700149266 -0.6749359195 5.8087171686
F -2.8633347155 -2.0951942636 4.9482322807
F -3.1097491381 -1.982674146 7.10639659
C 0.6273608133 -2.2707469424 0.2454865757
C -0.6462442274 -2.6477990396 0.6370762641
C -0.7770275608 -1.4214510654 1.3598045259
C 0.5227486667 -0.7934907614 0.8138766299
O 1.3021267634 -0.4285563542 1.8740118627
O -1.3961264304 -1.0089372189 2.3629143143
O 1.5359191868 -3.0675162873 -0.2762508088
C 2.9047617072 -2.6642914333 -0.6838754768
C -1.3964013691 -3.9367797045 0.6179358941
H -0.7901584781 -4.7286713356 0.1664758696
H -2.3323315839 -3.8560328563 0.0507796333
H -1.6552987019 -4.2381418739 1.6398863942
C 2.8081118611 -1.7155451136 -1.8808847843
H 3.812441072 -1.5304837021 -2.2791019792
H 2.1992193962 -2.1570551032 -2.6778196487
H 2.3737647319 -0.7534429909 -1.6006429573
C 3.5153837458 -4.0060516833 -1.0981113937
H 4.5468223718 -3.8569707703 -1.4347301276
H 3.5230713992 -4.6980582975 -0.2505174974
H 2.944241664 -4.4601030949 -1.9154347118
C 3.6624256141 -2.0562354973 0.4940467705
H 3.1964513391 -1.1345011075 0.8499321779
H 3.6944461546 -2.7515507292 1.3370717142
H 4.6895092147 -1.8362162792 0.1776209484
C 0.3766740894 0.2810426211 -0.2667493205
C -0.4959520847 0.1291933275 -1.3556605141
C 1.1277500786 1.457246458 -0.1672147973
H -1.0886781481 -0.776795197 -1.4497970585
H 1.7871750694 1.5755451379 0.681860792
C -0.6129929557 1.130514982 -2.3196541733
C 1.01468972 2.4576342684 -1.1348785964
H -1.2998130205 0.9978617463 -3.1526602635
H 1.5999122166 3.3681009092 -1.0307426094
C 0.1447718377 2.2998734718 -2.2146034966
H 0.0512759541 3.0821497744 -2.9642422338
O 2.9904850584 -3.3562406357 3.5916248539
S 2.0137358904 -2.5981246597 4.3742488041
C 2.0457119448 -3.4425963222 6.0373873296
O 0.5815801387 -2.6472948154 3.924673355
O 2.3293540613 -1.1544616397 4.6654817673
F 1.2477832145 -2.8479429966 6.9183822738
F 3.3001338696 -3.4319324347 6.5108770266
F 1.654972251 -4.7176197465 5.8871121165

Frequency: 11.3687; 19.7232; 21.8006; 23.5249; 24.3496; 27.6039; 29.5772; 34.8673; 44.7443; 49.4356; 50.6855; 53.0419; 57.5889; 61.0904; 64.1070; 65.3782; 71.5913; 75.7074; 78.5305; 81.4905; 92.8642; 100.8817; 108.0632; 121.7715; 126.8650; 134.5154; 144.0878; 152.9058; 157.5542; 170.1956; 185.2071; 192.4660; 207.0510; 209.4941; 211.4510; 212.8901; 215.4016; 220.1415; 222.5646; 226.0671; 231.6659; 261.4251; 268.0394; 273.5887; 287.0279; 302.4045; 306.6681; 308.5768; 321.0916; 321.9754; 323.8465; 327.0575; 339.6187; 350.8265; 350.9820; 352.5939; 356.3890; 381.7985; 406.4456; 422.2353; 443.3943; 454.1713; 488.6944; 489.5036; 491.2080; 492.1337; 511.5039; 522.4523; 523.7290; 524.0826; 530.5576; 557.2685; 557.8818; 559.3910; 569.2676; 570.9227; 578.1557; 595.5059; 617.9601; 625.7930; 633.6330; 634.3306; 666.8082; 690.7764; 715.1569; 722.7933; 756.5094; 757.5586; 760.1719; 762.0588; 777.9227; 801.3251; 866.2483; 873.6207; 896.7267; 917.1084; 937.2087; 942.3229; 953.8080; 973.6652; 974.8656; 978.0195; 987.0802; 987.4431; 1004.9435; 1018.0701; 1044.2352; 1051.2548; 1070.7942; 1073.2609; 1075.8893; 1077.1714; 1104.6602; 1110.9951; 1126.1220; 1133.7417; 1186.2307; 1202.3917; 1207.9572; 1209.8108; 1216.3231; 1217.9328; 1220.9413; 1226.4305; 1228.3133; 1232.3189; 1236.2907; 1236.9432; 1240.3474; 1257.0120; 1260.8997; 1286.4123; 1289.7532; 1298.6273; 1308.4661; 1320.9088; 1345.3792; 1362.1983; 1394.2323; 1429.1469; 1440.9850; 1444.6735; 1471.1746; 1492.2037; 1503.2534; 1512.1822; 1515.1371; 1516.9760; 1519.8251; 1530.2807; 1534.2239; 1538.1123; 1550.9266; 1604.2140; 1639.3674; 1659.2832; 1757.1172; 3043.7386; 3053.8928; 3054.5323; 3062.1572; 3099.4311; 3116.8853; 3127.4396; 3127.7141; 3135.6287; 3140.8869; 3160.3983; 3165.8854; 3169.1368; 3182.0853; 3193.9353; 3201.1970; 3272.0014.

Cartesian Forces: Max 0.000001091 RMS 0.000000340

Item	Value	Threshold	Converged?
Maximum Force	0.000001	0.000450	YES
RMS Force	0.000000	0.000300	YES
Maximum Displacement	0.000411	0.001800	YES
RMS Displacement	0.000081	0.001200	YES

FILE: Yb_Sq2_PhYbOTf3_Prod_Anion_B3LYP_M062x_Add12_Conf4_a.out

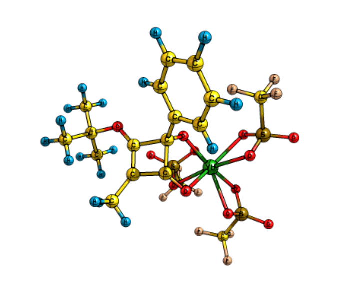

Conformer 8

E(RB3LYP/6-31G(d)/MWB59) = -3731.52834484
Convg = 0.7011D-08 -V/T = 2.0122

Temperature 195.150 Kelvin. Pressure 1.00000 Atm.
elec. and zero-point Energies= -3731.153862
elec. and thermal Energies= -3731.131103
elec. and thermal Enthalpies= -3731.130485
elec. and thermal Free Energies= -3731.198946

RB3LYP/6-31G(d)/MWB59/THF(IEFPCM) = -3731.59633539

RM06-2x/6-311+G(d,p)/MWB59/THF(IEFPCM) = -3731.43311633

Geometry at RB3LYP/6-31G(d)/MWB59
Charge = -1 Multiplicity = 1
Yb 0.3014848753 -0.2935726213 3.629479206
O 1.9090616987 1.4686170235 4.0816085545
S 1.0802933267 2.6182075041 3.577007363
O 0.9515811428 3.7956519766 4.4265110262
O -0.1961060322 2.002790945 3.0526473378
C 1.9820157837 3.2325695027 2.0626401326
F 3.0790835752 3.900103944 2.4476938554
F 2.3460172275 2.2310778297 1.266839345
F 1.1866141996 4.0697733322 1.3820980658
O -1.8433370939 -0.0900574662 4.7900686489
O -1.8703316666 1.1279665314 7.015578153
O 0.1606479872 -0.0967650688 6.1194481525
S -1.3329725488 0.0445122986 6.1996782183
C -1.9241357538 -1.5072264852 7.0493882685
F -3.2514888788 -1.4421100667 7.2265196895
F -1.6456285222 -2.5975904985 6.3289413897
F -1.33649861 -1.6201606752 8.2480666592
C 0.2645152357 -2.3220614879 0.0063961993
C -1.0678519702 -2.5239527857 0.3338345476
C -1.0186749483 -1.306515724 1.1095051629
C 0.365181339 -0.8838221617 0.6109723594
O 1.1881666065 -0.6789237234 1.6872817153
O -1.5856760548 -0.8737338245 2.1281246209
O 1.3008195033 -2.9597993079 -0.4955154444
C 1.3747822418 -4.4082281159 -0.7714230104
C -2.1614138178 -3.5390756097 0.2320907986
H -1.8329053819 -4.559511382 0.4535224518
H -2.6192337029 -3.5471822514 -0.766368254
H -2.9439066155 -3.2810161518 0.9537312436
C 1.1240367966 -5.193340121 0.5166240995
H 1.3092021236 -6.2584413374 0.333613113
H 1.7861976756 -4.8501991103 1.3162011977
H 0.0964233655 -5.0872127372 0.8705211456
C 2.826657337 -4.5761316127 -1.2284032609
H 3.0261427257 -5.6256117547 -1.4705699463
H 3.0246309233 -3.9690075259 -2.1181366475
H 3.5106857194 -4.2628348937 -0.4344847353
C 0.4064516731 -4.7625610111 -1.9038391567
H -0.636672198 -4.6221903951 -1.6117510368
H 0.6062615452 -4.1437480581 -2.7852738376
H 0.537019116 -5.8137276428 -2.1854891631
C 0.4347142894 0.216618191 -0.4386633854
C 1.379032876 0.1593301532 -1.4720668898
C -0.4083222383 1.3325535102 -0.3526836194
H 2.0443790789 -0.6964643313 -1.5376201732
H -1.1260154768 1.4013981206 0.4593397527
C 1.472925432 1.1920956503 -2.4065670438
C -0.3135233535 2.3661481714 -1.2859279923
H 2.2122129412 1.1344794315 -3.2026095778
H -0.9705797025 3.2281619075 -1.2010036341
C 0.6249277642 2.2982317122 -2.3179255184
H 0.6998344179 3.1049003017 -3.0435908025
O 2.3431223477 -3.9395440579 3.4469845743
S 1.6469227639 -2.9334529091 4.2504765758
C 1.6672876057 -3.655250421 5.9707909272
O 0.1864491799 -2.7119718457 3.9420456291
O 2.2728728626 -1.5798646278 4.3828150766
F 1.1315460906 -2.8248465196 6.8635477425
F 2.9339745885 -3.9116657485 6.3251093397
F 0.9779709396 -4.8055414196 5.9777732486

Frequency: 15.2057; 17.3351; 20.0339; 26.0309; 30.0910; 31.4090; 34.0765; 37.1682; 39.6400; 43.1126; 49.2910; 53.0801; 56.4486; 60.4390; 66.6621; 75.1435; 78.2422; 80.8776; 84.9676; 93.3931; 97.8613; 109.0897; 128.2437; 129.2652; 134.9647; 137.3494; 146.5430; 151.0663; 155.5455; 165.1453; 175.8282; 180.3282; 188.5315; 203.0916; 206.5823; 212.3485; 217.3716; 219.9262; 223.1855; 226.7713; 233.4706; 240.8795; 257.6179; 263.8492; 292.9472; 302.2403; 305.8466; 308.8706; 315.0859; 320.3291; 323.1585; 325.9819; 329.6496; 350.0242; 352.3877; 353.1554; 356.2089; 365.8658; 398.5437; 411.7183; 435.7958; 458.2576; 475.3524; 490.1614; 490.8200; 492.7089; 514.9935; 523.1283; 524.4632; 530.4805; 546.1441; 558.0081; 558.8537; 559.1595; 569.6134; 570.6122; 578.2584; 618.9649; 625.4304; 631.8811; 635.8852; 638.5754; 676.5988; 683.9212; 710.3238; 726.3061; 759.5434; 761.3332; 761.6217; 771.5267; 797.3679; 806.2130; 856.9490; 860.4665; 897.3467; 931.8121; 934.0169; 937.2718; 967.6212; 968.7984; 974.0938; 976.2469; 979.5970; 984.6973; 987.6875; 1018.4987; 1032.7180; 1054.4065; 1062.0829; 1068.4212; 1069.7275; 1087.0728; 1104.3839; 1116.5198; 1121.6759; 1138.4119; 1189.7018; 1204.5375; 1207.6134; 1208.8407; 1211.7650; 1215.6658; 1217.6834; 1225.3535; 1226.2874; 1227.1787; 1229.8203; 1242.8432; 1254.7241; 1256.5679; 1277.5599; 1284.6913; 1289.1907; 1295.9652; 1303.5081; 1317.4521; 1342.4093; 1371.6096; 1385.5438; 1429.9687; 1436.0229; 1443.8131; 1462.3120; 1493.4377; 1501.7263; 1512.8179; 1517.1632; 1520.3657; 1522.4128; 1529.6472; 1531.8285; 1546.1305; 1551.3849; 1619.2683; 1639.7631; 1662.5507; 1761.6201; 3037.4839; 3057.4787; 3060.7593; 3068.2167; 3098.8676; 3121.2414; 3125.0666; 3129.8513; 3141.3875; 3146.1596; 3159.8125; 3168.0010; 3169.7200; 3182.1934; 3195.5159; 3210.5550; 3215.5009.

Cartesian Forces: Max 0.000001547 RMS 0.000000513

Item	Value	Threshold	Converged?
Maximum Force	0.000000	0.000450	YES
RMS Force	0.000000	0.000300	YES
Maximum Displacement	0.000053	0.001800	YES
RMS Displacement	0.000008	0.001200	YES

FILE: Yb_Sq2_PhYbOTf3_Prod_Anion_B3LYP_M062x_Add12_Conf4-1_a.out

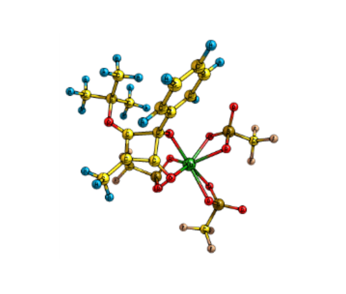

Conformer 9

E(RB3LYP/6-31G(d)/MWB59) = -3731.53724069
Convg = 0.7794D-09 -V/T = 2.0122

Temperature 195.150 Kelvin. Pressure 1.00000 Atm.
elec. and zero-point Energies= -3731.162609
elec. and thermal Energies= -3731.139886
elec. and thermal Enthalpies= -3731.139268
elec. and thermal Free Energies= -3731.207827

RB3LYP/6-31G(d)/MWB59/THF(IEFPCM) = -3731.60157686

RM06-2x/6-311+G(d,p)/MWB59/THF(IEFPCM) = -3731.43872560

Geometry at RB3LYP/6-31G(d)/MWB59
Charge = -1 Multiplicity = 1
Yb 0.115298077 -0.2672557914 3.7543146436
O -0.350326412 2.0546222128 3.2646898405
S 1.0092884356 2.6146066089 3.6104981247
O 1.6651477433 3.449374512 2.6052866217
O 1.795308829 1.4732190014 4.1884646621
C 0.694588608 3.7730420125 5.0365597329
F -0.0830065999 4.7822010926 4.6229214467
F 0.0984237638 3.1479493257 6.0468621858
F 1.8633169156 4.2750765941 5.4596416002
O -2.1390068421 -0.0453920477 4.6589800618
O -2.3906035113 1.4193373494 6.7138440348
O -0.276060443 0.1272682474 6.1715662508
S -1.7741853761 0.2536040375 6.0885518979
C -2.4388084063 -1.1912829844 7.0615578145
F -3.7789357342 -1.1815546347 7.0061693631
F -1.9979260654 -2.3531591775 6.5822767788
F -2.061522151 -1.0764548302 8.3418409956
C 0.7448550702 -2.2438705428 0.106279406
C -0.5286327602 -2.7017395634 0.3941416921
C -0.7721340506 -1.5115995539 1.1582334433
C 0.5263635278 -0.8050657717 0.7258079185
O 1.2144628909 -0.4574832634 1.8552868129
O -1.4685039497 -1.1712619242 2.1319636307
O 1.7323601611 -2.9647052323 -0.3894642332
C 3.1052582488 -2.4698357963 -0.6542553843
C -1.2148878116 -4.0195583402 0.2598064882
H -0.5058089798 -4.7951903366 -0.0481259676
H -2.0231556524 -3.9856968503 -0.4822040426
H -1.6610076896 -4.3136381224 1.2168994028
C 3.059912324 -1.3984567219 -1.7458363081
H 4.0822170311 -1.1612298535 -2.0621187317
H 2.5058862353 -1.7598736637 -2.619164161
H 2.5936650631 -0.4763594056 -1.3933898395
C 3.800600648 -3.7354736666 -1.1639679655
H 4.8511165714 -3.5198540567 -1.38575423
H 3.7602070032 -4.5240567264 -0.4060173004
H 3.3203884479 -4.1052576806 -2.076503053
C 3.7539905119 -1.9643861141 0.6346755433
H 3.1838396132 -1.1473232494 1.0854482358
H 3.8290685656 -2.7688486462 1.3710672878
H 4.7662605443 -1.6090207724 0.4081096844
C 0.3908007958 0.312095788 -0.3110123347
C -0.3969238072 0.1603279226 -1.4636792815
C 1.0551839148 1.5259570199 -0.1057096547
H -0.9224193862 -0.774517338 -1.6397413408
H 1.653187112 1.6555950264 0.7868605365
C -0.5159357675 1.1990125985 -2.3864404245
C 0.9368937417 2.5657752579 -1.0311808887
H -1.1358992083 1.0651708779 -3.2702366648
H 1.4509372286 3.5042089242 -0.8408672308
C 0.1533801746 2.4076076318 -2.1746348596
H 0.0568812488 3.2194347008 -2.8918678629
O 1.3819455387 -3.3795473097 6.3052435522
S 1.2897565413 -2.7775296867 4.9807603566
C 2.138087015 -4.0079575009 3.8605529927
O 2.0027251381 -1.4781348911 4.7445705994
O -0.0787902386 -2.6226731546 4.368048272
F 1.8434206964 -3.7787843361 2.5727031311
F 1.7396392521 -5.2491176072 4.166348179
F 3.4668004388 -3.9315936934 4.0124632452

Frequency: 13.7758; 19.8970; 23.3981; 26.3334; 27.5826; 28.4091; 33.3044; 34.8548; 41.7512; 47.5802; 51.1982; 53.5272; 55.5756; 63.4583; 64.8146; 65.4684; 69.9006; 71.7096; 78.0087; 80.5872; 94.3429; 98.8590; 115.9944; 132.0405; 134.4265; 143.2718; 146.3140; 150.8876; 154.1295; 174.1094; 182.3445; 185.8634; 207.3867; 210.6122; 214.6679; 216.2924; 217.2019; 219.8472; 224.1053; 225.1431; 232.9225; 256.2926; 271.7248; 284.1748; 286.6230; 303.6131; 308.1850; 309.2611; 321.3720; 322.6835; 324.0891; 324.5420; 338.8303; 350.7012; 352.3039; 352.8064; 357.3426; 382.1336; 409.5722; 422.6872; 441.9412; 454.3332; 488.7642; 490.2559; 490.4687; 491.7505; 513.2661; 524.2721; 524.5426; 525.1634; 530.3177; 557.3958; 558.4325; 558.8998; 570.5507; 572.1564; 572.7520; 597.3864; 625.0195; 627.2297; 633.0257; 634.7560; 666.9848; 691.8293; 715.8449; 724.2015; 758.0117; 758.6825; 761.7530; 763.1514; 778.0847; 801.4815; 866.7470; 874.7846; 897.2498; 919.6061; 937.1897; 942.6964; 956.3477; 973.1339; 974.7906; 976.7894; 983.4346; 989.2169; 1008.4274; 1017.2307; 1043.6925; 1050.6936; 1071.5553; 1074.0288; 1074.7393; 1078.4094; 1106.9039; 1108.0246; 1116.0026; 1135.0800; 1186.7072; 1203.5960; 1207.8639; 1211.3451; 1212.3196; 1215.1254; 1222.1966; 1228.5788; 1229.9556; 1232.0770; 1233.1526; 1240.6257; 1253.0001; 1253.7830; 1259.2784; 1286.2606; 1295.5402; 1297.0047; 1304.8847; 1314.3935; 1345.2117; 1362.5362; 1388.1291; 1429.3886; 1441.4072; 1443.1507; 1468.5269; 1491.7193; 1504.0637; 1510.7690; 1516.0205; 1518.0607; 1522.5413; 1531.1387; 1535.0526; 1538.2093; 1553.5236; 1613.2075; 1639.1215; 1658.7437; 1762.3460; 3041.5579; 3054.9428; 3056.1535; 3063.4456; 3097.6176; 3118.6839; 3125.7719; 3128.9348; 3131.0547; 3137.3071; 3157.5214; 3165.4305; 3168.8257; 3184.1904; 3196.1377; 3203.5502; 3262.4620.

Cartesian Forces: Max 0.000000624 RMS 0.000000286

Item	Value	Threshold	Converged?
Maximum Force	0.000000	0.000450	YES
RMS Force	0.000000	0.000300	YES
Maximum Displacement	0.000064	0.001800	YES
RMS Displacement	0.000013	0.001200	YES

FILE: Yb_Sq2_PhYbOTf3_Prod_Anion_B3LYP_M062x_Add12_Conf5_a.out

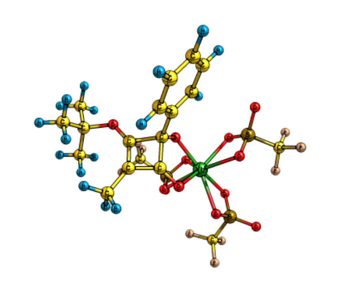

Conformer 10

E(RB3LYP/6-31G(d)/MWB59) = -3731.53090111
Convg = 0.6479D-08 -V/T = 2.0122

Temperature 195.150 Kelvin. Pressure 1.00000 Atm.
elec. and zero-point Energies= -3731.156230
elec. and thermal Energies= -3731.133552
elec. and thermal Enthalpies= -3731.132934
elec. and thermal Free Energies= -3731.201294

RB3LYP/6-31G(d)/MWB59/THF(IEFPCM) = -3731.59957558

RM06-2x/6-311+G(d,p)/MWB59/THF(IEFPCM) = -3731.43454465

Geometry at RB3LYP/6-31G(d)/MWB59
Charge = -1 Multiplicity = 1
Yb 0.0974443651 -0.2514628384 3.6927377464
O -0.5675322426 1.9834918332 3.0489707405
S 0.7466920135 2.6714980214 3.3308002417
O 1.3083121695 3.5139000839 2.2789416106
O 1.6390557796 1.6227255428 3.9349993486
C 0.366522569 3.8516891079 4.7224589285
F -0.4765718506 4.7959040325 4.2838853975
F -0.1864482181 3.2195006291 5.7540430963
F 1.5008709287 4.4404022217 5.1251525791
O -2.0913798058 -0.2293947609 4.7811617306
O -2.3602851807 1.2271817466 6.838897053
O -0.1540430308 0.1822423327 6.1453192163
S -1.6561917648 0.1336681205 6.1761193263
C -2.0663470984 -1.3682534561 7.1998719127
F -3.3969875917 -1.5335240601 7.2370966918
F -1.5114431691 -2.4692563054 6.6922396109
F -1.6211795625 -1.1937411556 8.4510855135
C 0.3506794619 -2.3915420228 0.1261369311
C -0.9818927634 -2.6608680039 0.4006836746
C -1.0337837485 -1.4282605762 1.1559965364
C 0.3461035097 -0.9359406731 0.6989366849
O 1.0977525992 -0.6530220131 1.8036890359
O -1.665772618 -1.0114928186 2.1395560645
O 1.439795377 -2.9632130289 -0.3385797957
C 1.5671441477 -4.3500543775 -0.8163042695
C -1.9951881182 -3.7585417718 0.3101549951
H -1.8987116379 -4.3619767067 -0.5979684563
H -3.0007585679 -3.3241662271 0.3187886149
H -1.9339126157 -4.4319021022 1.1752034628
C 1.0801519952 -5.3518117263 0.233136484
H 1.3455168371 -6.3650172693 -0.0903479702
H 1.555937174 -5.1634893505 1.1975421147
H -0.00157628 -5.3214255308 0.3695823654
C 3.0803252163 -4.4799965374 -1.0143600991
H 3.3304108104 -5.4749317433 -1.3983350721
H 3.4399601519 -3.727838729 -1.7234174829
H 3.5950636322 -4.3274533067 -0.0610557892
C 0.8227040815 -4.4740026001 -2.1489184754
H -0.2532118044 -4.3205142236 -2.0250123963
H 1.1949315508 -3.7324577774 -2.8631740746
H 0.9757031405 -5.4727819294 -2.5734730512
C 0.4211192494 0.1230330577 -0.4036386387
C -0.3620975495 0.0507539062 -1.565669206
C 1.3019716313 1.1980842897 -0.2514418512
H -1.0547575862 -0.7759331554 -1.7012907934
H 1.8994342333 1.2568631825 0.6498166409
C -0.2670038176 1.0337314735 -2.5506810532
C 1.3974052662 2.1824384232 -1.2370706701
H -0.8875524824 0.9652494136 -3.4417568793
H 2.0764423184 3.0180693339 -1.0894394579
C 0.6160439308 2.1047325495 -2.3904808633
H 0.6867003759 2.8748504373 -3.1553245371
O 1.6189272388 -3.3558604642 6.1286581887
S 1.5197404747 -2.6665126758 4.8472474047
C 2.5906384168 -3.6908209201 3.7145865174
O 2.0707718784 -1.2772940849 4.7419309284
O 0.1667111118 -2.6439275644 4.1786057893
F 2.6489919849 -3.1873509407 2.4784926371
F 2.0927070775 -4.9404365525 3.6371607859
F 3.8320667587 -3.7596120274 4.2092733131

Frequency: 14.6148; 17.9483; 20.3941; 26.3843; 29.7834; 32.0488; 34.3064; 38.1657; 40.8283; 43.0758; 48.9628; 50.6822; 54.0894; 54.7818; 62.7868; 71.2095; 77.7920; 79.8746; 83.9302; 93.5649; 100.7189; 116.3511; 131.2503; 134.9709; 138.4875; 143.5708; 147.8117; 150.5177; 153.5886; 163.3387; 178.9466; 181.9261; 201.4756; 207.7578; 210.1139; 212.3064; 215.0778; 217.1522; 218.4449; 222.8263; 236.5839; 257.2328; 259.5445; 272.3904; 288.7367; 302.7185; 306.1509; 308.0675; 319.9783; 320.8761; 322.7516; 325.4542; 334.9753; 350.0559; 352.5422; 353.3438; 357.4409; 392.8775; 398.0840; 420.9987; 453.2835; 457.8212; 482.1934; 489.6081; 490.6052; 492.6304; 500.3750; 524.3502; 524.8601; 528.3936; 533.9073; 558.0706; 558.2666; 558.9033; 570.4969; 572.8884; 573.6548; 624.5340; 627.3132; 632.7045; 633.9984; 641.9099; 677.2044; 689.0202; 713.2961; 725.9666; 754.6506; 758.1374; 761.3666; 762.7932; 782.8198; 809.3623; 858.0235; 862.5113; 898.4062; 920.1753; 935.1179; 937.1624; 967.1306; 970.3659; 974.6295; 977.0138; 980.6015; 984.1934; 1004.4082; 1017.5687; 1031.6550; 1052.4975; 1061.2558; 1065.1788; 1068.8755; 1084.1422; 1106.1739; 1108.3757; 1116.0577; 1138.1251; 1186.8267; 1197.3125; 1204.3208; 1207.6621; 1211.5304; 1213.8391; 1215.0033; 1225.9970; 1230.2362; 1233.1290; 1236.5437; 1238.8315; 1246.0812; 1248.5856; 1257.0479; 1284.4300; 1294.8031; 1301.0532; 1304.7236; 1313.8700; 1347.2177; 1363.0218; 1383.6921; 1429.3380; 1433.9142; 1443.4089; 1459.1843; 1493.0548; 1502.3287; 1510.2903; 1517.5510; 1521.4234; 1522.0840; 1529.0746; 1533.5765; 1539.2185; 1553.1413; 1621.5117; 1640.4037; 1660.2192; 1766.4081; 3043.1457; 3058.2937; 3062.5444; 3075.6130; 3101.1833; 3123.5199; 3126.5011; 3134.7268; 3142.9215; 3144.3988; 3155.7987; 3165.5092; 3181.1944; 3190.3697; 3192.6949; 3201.5302; 3252.0102.

Cartesian Forces: Max 0.000001312 RMS 0.000000521

Item	Value	Threshold	Converged?
Maximum Force	0.000000	0.000450	YES
RMS Force	0.000000	0.000300	YES
Maximum Displacement	0.000035	0.001800	YES
RMS Displacement	0.000005	0.001200	YES

FILE: Yb_Sq2_PhYbOTf3_Prod_Anion_B3LYP_M062x_Add12_Conf5-1_a.out

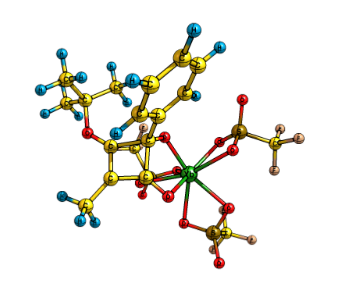

Conformer 11

E(RB3LYP/6-31G(d)/MWB59) = -3731.53489515
Convg = 0.7080D-08 -V/T = 2.0122

Temperature 195.150 Kelvin. Pressure 1.00000 Atm.
elec. and zero-point Energies= -3731.160336
elec. and thermal Energies= -3731.137566
elec. and thermal Enthalpies= -3731.136948
elec. and thermal Free Energies= -3731.205705

RB3LYP/6-31G(d)/MWB59/THF(IEFPCM) = -3731.59943944

RM06-2x/6-311+G(d,p)/MWB59/THF(IEFPCM) = -3731.43556619

Geometry at RB3LYP/6-31G(d)/MWB59
Charge = -1 Multiplicity = 1
Yb 0.1912955808 0.0067545789 3.6174508767
O 0.1691706291 2.3972862904 2.7746685291
S 1.4530184257 2.7639986547 3.4493866869
O 2.5067304943 3.3627743765 2.6314364668
O 1.8442610548 1.5818977286 4.307911018
C 1.014723183 4.0914606515 4.6828621092
F 0.4879792682 5.1408094736 4.0374589535
F 0.1373036916 3.6514636581 5.5838386463
F 2.1243981745 4.491444609 5.3200808352
O -1.495990627 -1.1084901598 5.0841441158
O -3.6369559143 0.1676789555 5.5661945782
O -1.4751207807 1.2828029546 4.8451106568
S -2.1789967716 0.1461288884 5.542360145
C -1.6559169928 0.3360841919 7.3192433702
F -2.1021468528 -0.6976244272 8.0421040644
F -0.3218758975 0.3848403745 7.4099967839
F -2.163118345 1.4676990315 7.8264220142
C 0.5125154912 -2.0957493266 0.1345200982
C -0.8226171251 -2.3097816059 0.4252770938
C -0.8672923544 -1.0419521815 1.0849413786
C 0.5393034016 -0.5889524292 0.626102424
O 1.2689199135 -0.2682725181 1.7329724364
O -1.5048327842 -0.5373403838 2.0342551178
O 1.3764553527 -3.0251518789 -0.2169016136
C 2.7438524046 -2.8026635849 -0.737789888
C -1.7080454582 -3.5094663025 0.4177412135
H -1.1865731386 -4.3719964089 -0.0093212013
H -2.6218160951 -3.3356678472 -0.1637424782
H -2.0113078367 -3.7595395597 1.4415633572
C 2.6039034921 -2.6315513576 -2.2537962511
H 3.5945685118 -2.5492722906 -2.7155079495
H 2.0864430428 -3.4905916221 -2.6946840536
H 2.0410133259 -1.7225364943 -2.4897817236
C 3.4452298962 -4.1147492098 -0.3756212286
H 4.4639885951 -4.1206479927 -0.7780730175
H 3.4954716573 -4.2275483209 0.7115359648
H 2.9014927636 -4.9706799027 -0.7892670233
C 3.4526370988 -1.6136862581 -0.0943062016
H 3.0231710608 -0.6584484042 -0.3999159765
H 3.4187479495 -1.6705412657 0.9936420983
H 4.5010498034 -1.6283102933 -0.4159506953
C 0.6121781961 0.4353768568 -0.5096725131
C -0.0719963217 0.247763396 -1.7220196279
C 1.3710137318 1.5993558481 -0.3403069786
H -0.6758184029 -0.6439053311 -1.8683577541
H 1.8870528421 1.7583064872 0.5977053005
C 0.0060014489 1.1981692158 -2.740585882
C 1.4497748778 2.5493831025 -1.3615427411
H -0.5349582244 1.0360965367 -3.6703787213
H 2.039864485 3.44874699 -1.2044041064
C 0.7709770456 2.3542547365 -2.5648933874
H 0.8306592877 3.0969371107 -3.3571783557
O 1.129344671 -3.5776230079 5.762927987
S 1.3912399296 -2.5782694499 4.7336725447
C 3.083666808 -3.0188982106 4.0797350032
O 1.5677235436 -1.1460913617 5.1823901809
O 0.5116153171 -2.5498502637 3.5251140333
F 3.5859156865 -2.0306894035 3.328540544
F 3.0078524091 -4.1303142848 3.3263629949
F 3.9249178243 -3.2492469823 5.094088522

Frequency: 14.6455; 18.0347; 21.0117; 25.4652; 28.1227; 29.8749; 32.3825; 36.5555; 39.0580; 46.5443; 52.5076; 53.4547; 57.7861; 62.9112; 65.9088; 70.9232; 72.7447; 73.0041; 80.5545; 82.8322; 88.1157; 92.0254; 101.5029; 117.4629; 128.7483; 129.6185; 135.6164; 153.0742; 165.2913; 171.1101; 183.5419; 191.0894; 205.8210; 208.2621; 211.1426; 213.5669; 218.0372; 224.1637; 227.9079; 233.2646; 236.6112; 265.0832; 273.7501; 282.5052; 290.4900; 304.3360; 306.4106; 308.4479; 320.6938; 323.4577; 325.6650; 327.4325; 338.6654; 348.9666; 350.8860; 352.2614; 356.8075; 386.8584; 403.8094; 422.6667; 448.4860; 456.7636; 485.0866; 490.0662; 491.3937; 493.0898; 514.4577; 522.2108; 523.9222; 527.9771; 529.8723; 557.5311; 558.0884; 559.2704; 569.7092; 571.0462; 577.1377; 594.0659; 620.7033; 622.9421; 631.9130; 633.5897; 665.8128; 694.2340; 713.2508; 722.3684; 755.9764; 756.3560; 760.4977; 761.6748; 779.5092; 799.4640; 865.9557; 872.4760; 895.6930; 913.7723; 937.2249; 943.1357; 951.7888; 972.1208; 973.6271; 978.0600; 985.1662; 986.1012; 1004.2427; 1016.4758; 1045.2649; 1049.1801; 1064.7552; 1072.7163; 1075.3197; 1078.6565; 1106.8246; 1114.8505; 1129.3240; 1134.2101; 1187.0666; 1202.8373; 1208.2145; 1210.2835; 1213.8416; 1217.3341; 1220.0888; 1224.1306; 1230.1542; 1232.3024; 1234.6011; 1237.2000; 1243.0160; 1252.9827; 1258.3770; 1283.4081; 1295.0176; 1300.7119; 1308.3588; 1317.3408; 1344.7481; 1362.6374; 1396.0921; 1429.8005; 1438.5279; 1445.4268; 1464.5341; 1491.0013; 1503.4151; 1511.6231; 1512.0881; 1514.6749; 1520.6455; 1528.6853; 1536.6513; 1538.2840; 1549.4882; 1603.9952; 1638.1918; 1657.7470; 1752.6000; 3045.6793; 3051.5328; 3059.3058; 3069.2014; 3101.9558; 3120.1276; 3127.8960; 3129.4213; 3132.6738; 3145.8226; 3154.3838; 3168.2081; 3182.8304; 3194.6542; 3201.3304; 3201.7084; 3262.6895.

Cartesian Forces: Max 0.000000915 RMS 0.000000345

Item	Value	Threshold	Converged?
Maximum Force	0.000001	0.000450	YES
RMS Force	0.000000	0.000300	YES
Maximum Displacement	0.000035	0.001800	YES
RMS Displacement	0.000006	0.001200	YES

FILE: Yb_Sq2_PhYbOTf3_Prod_Anion_B3LYP_M062x_Add12_Conf6_a.out

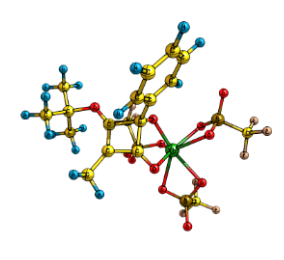

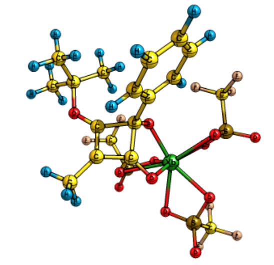

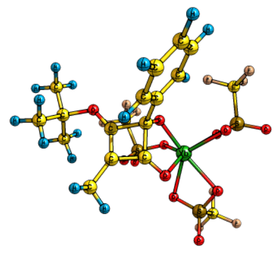

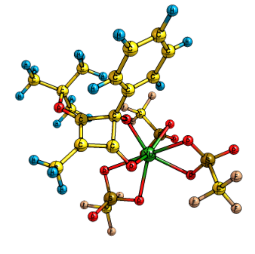

Conformer 12

E(RB3LYP/6-31G(d)/MWB59) = -3731.53013342
Convg = 0.7898D-08 -V/T = 2.0122

Temperature 195.150 Kelvin. Pressure 1.00000 Atm.
elec. and zero-point Energies= -3731.155822
elec. and thermal Energies= -3731.132863
elec. and thermal Enthalpies= -3731.132245
elec. and thermal Free Energies= -3731.202739

RB3LYP/6-31G(d)/MWB59/THF(IEFPCM) = -3731.59779480

RM06-2x/6-311+G(d,p)/MWB59/THF(IEFPCM) = -3731.43329047

Geometry at RB3LYP/6-31G(d)/MWB59
Charge = -1 Multiplicity = 1
Yb 0.221646059 -0.0405842556 3.4169597741
O 0.0951798014 2.2714165166 2.4671393295
S 1.3821884199 2.7318174864 3.0811032018
O 2.3864674022 3.3247722328 2.2012734469
O 1.8422360503 1.6141491598 3.9899021409
C 0.9181380268 4.109276774 4.2488860377
F 0.3319962623 5.0968228951 3.5590802315
F 0.0831534548 3.6872487812 5.1970728943
F 2.0260118917 4.589600439 4.8311287501
O -1.3383792864 -1.1807550582 4.9550966853
O -3.4911198951 0.0124385262 5.5738708583
O -1.4170546081 1.2130228678 4.738285587
S -2.0373858407 0.0482728886 5.4637717496
C -1.4140680101 0.2380976221 7.2077864663
F -1.7799614472 -0.8164031007 7.9448656382
F -0.0792272666 0.3316814696 7.2158792295
F -1.9264363118 1.3473697916 7.7573936792
C 0.2546206321 -2.2651176945 0.0172300576
C -1.0981569651 -2.3884770014 0.308148404
C -1.0276705314 -1.1070202346 0.9623616059
C 0.3873525818 -0.7715467853 0.4606094825
O 1.1827009234 -0.4812791333 1.5313430285
O -1.6055127608 -0.5769567701 1.9326061975
O 1.2861164909 -2.9781745102 -0.3689646342
C 1.3032362532 -4.4416553738 -0.555140233
C -2.216855225 -3.3819000766 0.2964481299
H -1.9116459125 -4.3853917025 0.6078549644
H -2.6775093682 -3.4676284817 -0.6969089488
H -2.9905607406 -3.0417031143 0.9929611484
C 0.9868143022 -5.1381300716 0.7692446787
H 1.0258531021 -6.2245126076 0.6267226576
H 1.7140163636 -4.8595515365 1.5339984433
H -0.0016900497 -4.8775305661 1.1503187534
C 2.7573977231 -4.6966718315 -0.9614444168
H 2.9236823539 -5.7682940773 -1.1161859842
H 2.9994385134 -4.166128862 -1.8881115981
H 3.4322423969 -4.3460271131 -0.1750268723
C 0.3476717917 -4.8239615277 -1.6894346829
H -0.697484616 -4.6460930767 -1.4264628458
H 0.581718032 -4.2508790624 -2.5929445925
H 0.4578425429 -5.8895698472 -1.9204233311
C 0.5361530238 0.1902883328 -0.7204638527
C -0.2261502987 0.058285133 -1.8909669297
C 1.4626240919 1.2344480174 -0.635131896
H -0.9536916769 -0.7447619826 -1.975743218
H 2.0435166768 1.3452877801 0.2716559141
C -0.0650506622 0.9501197765 -2.9514420225
C 1.6256312848 2.1257128518 -1.6975879729
H -0.6689952432 0.8343853733 -3.8489911721
H 2.3453629759 2.9352958848 -1.606099025
C 0.8649733673 1.9885233736 -2.8595366307
H 0.9900155787 2.6862655364 -3.6845543811
O 1.5129922967 -3.5151881028 5.6600146071
S 1.5927092818 -2.6341312708 4.4983516754
C 3.2415756741 -3.0396427137 3.727548163
O 1.7352193329 -1.1530477885 4.7917360943
O 0.5940585722 -2.7975316955 3.4078677822
F 3.4941366604 -2.2766237807 2.6640660734
F 3.2467940544 -4.3315964006 3.3317098194
F 4.222322446 -2.8755360985 4.6231249589

Frequency: 8.2922; 12.1619; 17.7611; 19.4206; 24.2965; 26.5067; 28.4141; 34.2784; 35.7937; 38.5494; 40.6186; 45.0769; 51.3203; 54.8672; 60.7222; 64.9332; 72.7967; 76.7635; 81.5606; 89.3192; 92.5861; 98.0281; 107.3821; 131.6347; 136.1708; 138.5283; 140.6603; 151.0775; 165.5262; 168.8322; 175.5569; 179.9227; 194.3751; 203.8945; 208.3246; 211.2957; 214.5894; 215.4859; 224.2135; 229.0349; 236.5732; 250.1615; 262.9610; 266.4981; 291.2760; 303.6749; 305.9642; 307.7765; 319.8979; 322.3494; 323.9947; 326.7979; 339.4137; 352.0445; 354.1161; 354.9302; 358.1656; 394.1702; 397.0321; 420.6477; 454.9136; 460.2501; 484.5834; 490.3233; 491.4513; 493.1726; 499.4211; 522.2887; 524.3703; 527.2416; 534.7478; 557.5765; 558.5272; 559.2747; 570.5964; 572.0840; 577.5596; 619.3743; 622.8598; 631.8947; 633.6891; 639.5049; 683.0133; 691.3985; 711.9950; 727.3847; 754.4699; 756.3213; 760.5405; 761.9736; 784.2462; 810.5789; 857.4197; 861.4975; 897.0082; 917.7523; 935.6102; 936.9898; 965.0246; 968.9844; 970.3346; 977.1063; 980.3822; 984.5961; 1001.3092; 1017.2441; 1028.2521; 1051.2790; 1062.9283; 1067.7880; 1071.8580; 1082.8582; 1107.0044; 1113.7792; 1127.2291; 1135.9198; 1186.7203; 1192.9048; 1204.7877; 1207.5535; 1212.4122; 1213.1981; 1217.1586; 1227.4212; 1230.6354; 1234.0149; 1236.5013; 1238.4681; 1245.5667; 1250.1873; 1254.1654; 1285.0948; 1293.5965; 1298.0331; 1305.9377; 1316.1120; 1346.8843; 1362.8339; 1389.5970; 1430.0416; 1435.8901; 1445.5150; 1462.2030; 1492.7234; 1501.5173; 1510.6676; 1513.5787; 1519.9685; 1521.8827; 1528.3798; 1531.0286; 1539.8569; 1549.1175; 1605.7284; 1640.5788; 1659.9772; 1751.9620; 3039.2271; 3058.2682; 3062.9731; 3073.3472; 3102.6289; 3123.2867; 3129.8276; 3132.2951; 3142.7375; 3157.5321; 3160.9641; 3165.4738; 3180.5165; 3187.6248; 3192.3262; 3199.7589; 3257.7717.

Cartesian Forces: Max 0.000001031 RMS 0.000000369

Item	Value	Threshold	Converged?
Maximum Force	0.000001	0.000450	YES
RMS Force	0.000000	0.000300	YES
Maximum Displacement	0.000083	0.001800	YES
RMS Displacement	0.000012	0.001200	YES

FILE: Yb_Sq2_PhYbOTf3_Prod_Anion_B3LYP_M062x_Add12_Conf6-1_a.out

Conformer 13

E(RB3LYP/6-31G(d)/MWB59) = -3731.53428613
Convg = 0.6155D-08 -V/T = 2.0122

Temperature 195.150 Kelvin. Pressure 1.00000 Atm.
elec. and zero-point Energies= -3731.159771
elec. and thermal Energies= -3731.136950
elec. and thermal Enthalpies= -3731.136332
elec. and thermal Free Energies= -3731.205394

RB3LYP/6-31G(d)/MWB59/THF(IEFPCM) = -3731.59990308

RM06-2x/6-311+G(d,p)/MWB59/THF(IEFPCM) = -3731.43530744

Geometry at RB3LYP/6-31G(d)/MWB59
Charge = -1 Multiplicity = 1
Yb 0.2281124259 -0.0406473115 3.6213289595
O 1.5396595845 1.6132119724 4.7365201511
S 1.2230844283 2.8239054661 3.888759368
O 0.8727243438 4.0574752477 4.5822573765
O 0.337757648 2.3457303849 2.7811206429
C 2.8525153384 3.1981513026 3.0628208483
F 3.7174345061 3.6998793062 3.9516604591
F 3.3860649799 2.090811141 2.5269030661
F 2.6708966998 4.0962082651 2.0826648473
O -1.3391836247 -1.0812181876 5.1955489496
O -3.6524976104 -0.0593800255 5.4181415877
O -1.6250665579 1.2271353065 4.5937273642
S -2.2061727337 0.1243543189 5.4377393741
C -1.7978792094 0.6275836111 7.1833453147
F -2.2038759662 -0.3210718082 8.0367686386
F -0.4795419314 0.7992999382 7.3234831106
F -2.4194775825 1.7738870206 7.4853660106
C 0.5396495828 -2.1721029151 0.1192122784
C -0.7829825782 -2.4154637871 0.4398270394
C -0.8400811665 -1.1533125407 1.1101345471
C 0.5492190066 -0.6716488118 0.6312795055
O 1.2923524701 -0.3470735256 1.7296301048
O -1.4743383099 -0.6582017756 2.064484483
O 1.4104077321 -3.0801002654 -0.2716063442
C 2.7502063806 -2.8249737876 -0.8438240802
C -1.6455144352 -3.6316389284 0.4374115453
H -1.1353560389 -4.4670614501 -0.0525890187
H -2.5954050807 -3.4535794785 -0.0810716098
H -1.8819574158 -3.9261056865 1.4671140984
C 2.5487954208 -2.6652665533 -2.3542322272
H 3.5187119807 -2.559390093 -2.853738249
H 2.0376575402 -3.5395577589 -2.7717105907
H 1.9536310138 -1.7723562681 -2.5722226176
C 3.4998578456 -4.1160194122 -0.5034076519
H 4.5048176387 -4.0946617687 -0.9386717911
H 3.5870429617 -4.2259649749 0.5814911996
H 2.9668127123 -4.9873239587 -0.8984116084
C 3.4496440767 -1.6130235592 -0.2333890655
H 2.9900562164 -0.6720989029 -0.540087064
H 3.4438917093 -1.658070266 0.8554060321
H 4.4894820881 -1.6078097549 -0.5818698425
C 0.5753981726 0.3703723222 -0.4903316769
C -0.1487413849 0.2009942465 -1.6816056033
C 1.3300015973 1.5370115819 -0.3249445894
H -0.7499106233 -0.6928620433 -1.8251999472
H 1.879133571 1.6667719068 0.5973543567
C -0.1143240569 1.1735761693 -2.681648443
C 1.3657939476 2.509543362 -1.3265622777
H -0.6865818641 1.0264552857 -3.5949337277
H 1.9526700991 3.4116574688 -1.1715037999
C 0.6455843477 2.3334406163 -2.5086510357
H 0.6696197033 3.0934240623 -3.2861033549
O 1.2944046866 -3.6105536019 5.7301831308
S 1.4918192575 -2.6523794621 4.6482922406
C 3.124586044 -3.1544292397 3.8902017811
O 1.733861839 -1.2109397201 5.0351830441
O 0.5191331561 -2.6452250632 3.5159327337
F 3.6267932074 -2.1811707029 3.1181951169
F 2.960575853 -4.2542113157 3.1340044928
F 4.0142478592 -3.4284364954 4.8513677482

Frequency: 13.6584; 18.3513; 20.1348; 22.4965; 24.8575; 28.6648; 33.9581; 36.9883; 42.5719; 46.9609; 48.9225; 51.6403; 57.6119; 61.8411; 67.1786; 70.0863; 71.8218; 72.6464; 78.3807; 81.2950; 85.7323; 90.4976; 103.2413; 112.2639; 119.9912; 129.2836; 143.2250; 156.0454; 167.6441; 168.1191; 183.5419; 190.2586; 205.4875; 206.8957; 213.1963; 214.6819; 216.7664; 223.7857; 228.2569; 233.3484; 234.9780; 264.9557; 271.2788; 282.9654; 289.0017; 304.4655; 305.9573; 307.4478; 320.7649; 323.9689; 324.9814; 327.0083; 338.6992; 348.9829; 350.9196; 352.0128; 357.0856; 386.6448; 404.0668; 421.8253; 446.8551; 456.7718; 484.5455; 489.9998; 491.0135; 493.0628; 512.8504; 521.7359; 524.2173; 527.3895; 530.1735; 557.3546; 558.0432; 558.5658; 569.0395; 572.2375; 576.9357; 594.4299; 618.6483; 623.4557; 631.3177; 634.1124; 665.9766; 692.9115; 713.0576; 722.4029; 755.6360; 756.4312; 757.2585; 761.1273; 778.8482; 799.6460; 866.3606; 873.3071; 895.2417; 914.4192; 937.3757; 943.6158; 953.0782; 971.6988; 973.2561; 978.3394; 985.0894; 986.5344; 1002.2956; 1017.8902; 1046.2019; 1049.6990; 1064.8064; 1072.0249; 1074.7766; 1078.7262; 1104.1977; 1114.5954; 1127.0749; 1136.6908; 1186.1532; 1201.4413; 1208.8661; 1209.4432; 1216.3507; 1216.8074; 1219.3014; 1220.2786; 1225.7426; 1230.1590; 1234.8394; 1236.5923; 1238.9713; 1240.4270; 1259.5427; 1283.6072; 1300.9878; 1307.9810; 1308.9178; 1319.5494; 1345.0304; 1362.4672; 1395.4209; 1429.8387; 1438.5388; 1445.0630; 1464.3628; 1491.7523; 1503.8512; 1511.4306; 1513.2582; 1514.8792; 1520.7662; 1528.6622; 1536.7924; 1537.7000; 1550.0870; 1607.9150; 1638.4988; 1658.6273; 1757.3338; 3046.5245; 3051.4979; 3059.9102; 3069.5392; 3103.1964; 3120.2469; 3127.6477; 3129.3845; 3132.1687; 3148.2893; 3153.4246; 3169.5351; 3182.4053; 3194.0597; 3201.3595; 3203.9161; 3278.1787.

Cartesian Forces: Max 0.000001050 RMS 0.000000434

Item	Value	Threshold	Converged?
Maximum Force	0.000001	0.000450	YES
RMS Force	0.000000	0.000300	YES
Maximum Displacement	0.000046	0.001800	YES
RMS Displacement	0.000010	0.001200	YES

FILE: Yb_Sq2_PhYbOTf3_Prod_Anion_B3LYP_M062x_Add12_Conf7_a.out

Conformer 14

E(RB3LYP/6-31G(d)/MWB59) = -3731.53081291
Convg = 0.3649D-09 -V/T = 2.0122

Temperature 195.150 Kelvin. Pressure 1.00000 Atm.
elec. and zero-point Energies= -3731.156392
elec. and thermal Energies= -3731.133450
elec. and thermal Enthalpies= -3731.132832
elec. and thermal Free Energies= -3731.202842

RB3LYP/6-31G(d)/MWB59/THF(IEFPCM) = -3731.59931751

RM06-2x/6-311+G(d,p)/MWB59/THF(IEFPCM) = -3731.43259738

Geometry at RB3LYP/6-31G(d)/MWB59
Charge = -1 Multiplicity = 1
Yb 0.2068807578 0.0518687852 3.3858928373
O 1.567372686 1.6947219596 4.4746665562
S 1.2474586278 2.8731167466 3.5860219756
O 0.9308939045 4.1422658354 4.2274349654
O 0.3130920516 2.3546904776 2.530061968
C 2.8492159087 3.1747409244 2.6814902482
F 3.7594763995 3.6790611664 3.521756966
F 3.326586451 2.0325566857 2.1681874574
F 2.652476121 4.0442205684 1.6822478647
O -1.2419515493 -1.1322195726 4.9177507017
O -3.570996379 -0.2144907413 5.3414795102
O -1.630339343 1.212290284 4.5364779576
S -2.1327154087 0.0203999835 5.305409859
C -1.6424931372 0.3800080612 7.0652439429
F -1.9724479662 -0.6515332861 7.8508169556
F -0.3232295077 0.5827385964 7.1466095493
F -2.2774544787 1.4752368429 7.5001807114
C 0.2279388584 -2.309507698 0.0406525494
C -1.1188991819 -2.4463775729 0.3581495059
C -1.0602124888 -1.1466505664 0.9756834689
C 0.3427360689 -0.8084751225 0.4524263381
O 1.1376398969 -0.4884377623 1.5171591878
O -1.6319303638 -0.5826196427 1.9300180735
O 1.2642235455 -3.0067421195 -0.3556298172
C 1.3045746029 -4.4663469125 -0.5757826227
C -2.2148462649 -3.4640078167 0.3902588498
H -1.876738108 -4.453124222 0.7127396438
H -2.6964558749 -3.5789248042 -0.5901625519
H -2.9799164474 -3.1295454195 1.098854941
C 1.0281265761 -5.2065427428 0.7332634318
H 1.0600635482 -6.2870439081 0.5490264521
H 1.7773155402 -4.9569997353 1.4860330348
H 0.0538796044 -4.9572364007 1.155304619
C 2.7564761763 -4.6787117843 -1.0136030537
H 2.9489878271 -5.7440776729 -1.180354467
H 2.9674263065 -4.133839976 -1.9396806437
H 3.4330435532 -4.3151341007 -0.2345701189
C 0.3325530645 -4.8365324408 -1.6998387838
H -0.7102293597 -4.6942802611 -1.4060809946
H 0.5301811823 -4.2297280595 -2.5899849692
H 0.4636856299 -5.8912162746 -1.9669844311
C 0.4719458336 0.1367124452 -0.7426356613
C -0.3247780097 0.0032607367 -1.8895386047
C 1.4150251291 1.1682963191 -0.695274593
H -1.0646470801 -0.7910121506 -1.9445187297
H 2.0234806237 1.2697455526 0.1940337793
C -0.1824581874 0.8830786207 -2.9626468713
C 1.5604802677 2.0466412767 -1.7708724404
H -0.8139474771 0.7678280043 -3.8409160934
H 2.2953347403 2.8460938298 -1.7120868215
C 0.7636442509 1.9095278715 -2.9083765612
H 0.8739902765 2.5975251863 -3.7434136394
O 1.8645180788 -3.4728180268 5.564519306
S 1.728046556 -2.7532880648 4.2989524305
C 3.342890032 -3.0672839801 3.4209943523
O 1.7492990673 -1.2379254461 4.4415428179
O 0.6862804087 -3.1847477292 3.3463801621
F 3.3905398601 -2.4726798626 2.2247185375
F 3.486821825 -4.3974588619 3.2250673535
F 4.3764438052 -2.6403350909 4.156083264

Frequency: 12.3428; 15.8445; 19.2559; 20.5339; 24.9415; 26.6901; 31.7363; 33.5116; 35.1511; 38.6986; 42.3750; 49.4196; 52.9769; 55.8287; 60.1336; 62.1678; 70.2922; 71.2343; 79.0447; 81.3637; 82.6464; 95.9637; 101.6888; 129.9243; 136.5081; 140.9110; 147.5624; 153.8498; 171.7062; 173.4599; 177.1311; 180.5960; 195.7469; 196.0792; 209.0594; 211.3737; 214.4468; 222.5050; 223.1945; 232.1604; 238.7250; 250.2933; 261.9925; 268.4429; 291.0609; 302.6963; 305.3456; 307.2278; 321.1441; 322.0188; 323.8569; 330.0727; 337.4408; 353.4565; 354.2781; 354.5336; 359.3238; 394.1292; 398.9557; 420.8523; 453.8990; 460.4419; 484.2445; 490.6105; 490.7587; 495.7475; 501.1860; 519.2174; 525.2193; 527.3017; 535.3809; 557.1980; 557.8194; 558.2003; 572.1685; 573.5796; 577.6147; 613.8402; 623.0962; 629.2155; 634.3225; 639.9490; 681.0759; 691.1924; 714.0473; 727.5875; 754.3551; 756.0332; 758.9802; 761.1642; 784.8425; 811.0561; 856.1265; 864.4488; 897.0002; 921.5234; 936.0768; 937.4918; 963.2202; 969.6840; 972.4516; 974.9741; 980.2916; 982.4462; 1007.7668; 1018.0669; 1031.2042; 1052.4727; 1063.2395; 1068.4918; 1071.9310; 1084.3726; 1105.6792; 1112.4700; 1120.6831; 1144.4551; 1186.9211; 1192.8008; 1204.8321; 1208.3831; 1212.9043; 1215.8735; 1216.4171; 1226.8752; 1231.4860; 1235.7570; 1237.2272; 1239.2239; 1239.9447; 1241.7496; 1247.9907; 1285.6887; 1293.2424; 1308.2392; 1309.7845; 1319.3810; 1348.0296; 1363.1893; 1392.9984; 1429.8762; 1435.6561; 1446.0877; 1462.4868; 1493.4608; 1501.3116; 1508.1467; 1513.2187; 1519.0128; 1522.0060; 1528.3504; 1530.7086; 1540.2361; 1547.8972; 1604.3547; 1640.9206; 1660.6366; 1754.9364; 3039.2647; 3057.9158; 3062.2266; 3072.2267; 3104.0286; 3123.2122; 3131.7903; 3132.4059; 3143.7071; 3155.5869; 3162.5243; 3167.4023; 3179.8885; 3191.5510; 3192.3133; 3199.5289; 3264.0999.

Cartesian Forces: Max 0.000000331 RMS 0.000000088

Item	Value	Threshold	Converged?
Maximum Force	0.000000	0.000450	YES
RMS Force	0.000000	0.000300	YES
Maximum Displacement	0.000021	0.001800	YES
RMS Displacement	0.000004	0.001200	YES

FILE: Yb_Sq2_PhYbOTf3_Prod_Anion_B3LYP_M062x_Add12_Conf7-1_a.out

Conformer 15

E(RB3LYP/6-31G(d)/MWB59) = -3731.54009255
Convg = 0.2408D-08 -V/T = 2.0122

Temperature 195.150 Kelvin. Pressure 1.00000 Atm.
elec. and zero-point Energies= -3731.165637
elec. and thermal Energies= -3731.142837
elec. and thermal Enthalpies= -3731.142219
elec. and thermal Free Energies= -3731.211585

RB3LYP/6-31G(d)/MWB59/THF(IEFPCM) = -3731.60232644

RM06-2x/6-311+G(d,p)/MWB59/THF(IEFPCM) = -3731.44182230

Geometry at RB3LYP/6-31G(d)/MWB59
Charge = -1 Multiplicity = 1
Yb 0.7491388042 -0.4601645895 3.6315217282
O 2.8859747622 -0.1537105642 4.6805411312
S 3.06935679 1.325370075 4.4346122156
O 3.4975257488 2.1458088184 5.5602414649
O 1.8722814776 1.7642326966 3.6440264262
C 4.4876860422 1.3989022432 3.2287602026
F 5.6053833139 0.9505109377 3.81444908
F 4.2400609026 0.6493986542 2.1485386931
F 4.6834441922 2.6645290624 2.837847186
O -1.0924034263 1.0922631959 3.3261826528
O -1.5381165155 2.8019088547 5.1499556228
O -0.3344409249 0.599175772 5.5562331108
S -1.3398983653 1.4065212816 4.7790345124
C -2.9616461956 0.5670896291 5.1462193172
F -3.9345079229 1.1153771952 4.4062092428
F -2.8902594682 -0.7374385866 4.8685878716
F -3.2687680375 0.7187087642 6.4409213781
C 0.7803811258 -2.2931492383 -0.0674203602
C -0.1907060665 -3.0769532017 0.5246950501
C -0.5096716496 -1.9825986051 1.3951149624
C 0.3601980823 -0.9517201641 0.6346238056
O 1.2269189266 -0.3534289417 1.5107385016
O -0.9406015382 -1.8449911942 2.5553380701
O 1.7434367644 -2.7026089141 -0.8731523127
C 3.0022695377 -1.9539622919 -1.0975685834
C -0.4945004132 -4.5365476216 0.5382211906
H 0.2157520217 -5.0846720603 -0.0895167059
H -1.5074923769 -4.7457097716 0.1717429725
H -0.4249105142 -4.9182842533 1.56391698
C 2.7306405068 -0.5029452959 -1.4952691604
H 3.6694978983 -0.0484024966 -1.8328301256
H 2.0092317812 -0.4493633467 -2.3172857673
H 2.3594419375 0.0805701685 -0.650752462
C 3.6407711309 -2.7330241406 -2.2495284688
H 4.6276620372 -2.3173021661 -2.4786400708
H 3.7616921892 -3.7872363852 -1.9799650695
H 3.0209224831 -2.6737466433 -3.1509338356
C 3.8326684253 -2.0526645031 0.1835884157
H 3.3090906261 -1.5726787751 1.0135352948
H 4.0211586031 -3.1002157192 0.4407103755
H 4.7960172489 -1.5487992978 0.042947148
C -0.3865839855 0.0352756874 -0.2669399046
C -1.1953499071 -0.3901982782 -1.3318281901
C -0.2815460947 1.4074683316 -0.0088803618
H -1.2953872805 -1.4512296974 -1.5444908976
H 0.3355214564 1.7343290139 0.8196946073
C -1.8768043465 0.5344789135 -2.1237661222
C -0.9655461133 2.3321894972 -0.8005843002
H -2.4979885276 0.1848263677 -2.9455526302
H -0.8764268904 3.3930893069 -0.5799197263
C -1.7635498523 1.901925428 -1.8617149847
H -2.2962888234 2.6235480637 -2.4767704009
O 0.4950196777 -4.6282265844 4.264695678
S 1.1662529141 -3.3542652522 4.5200663608
C 2.5380515407 -3.7869664717 5.7048180333
O 0.3860296151 -2.2777428162 5.2253259168
O 1.8497215678 -2.6932662737 3.3552446048
F 3.2238660694 -2.7066915625 6.0733419931
F 3.3759458109 -4.653542924 5.1157034436
F 2.0197918852 -4.3664434316 6.7962189892

Frequency: 11.8064; 17.3462; 21.0399; 23.0322; 27.1347; 28.1863; 29.5461; 33.5776; 34.4792; 42.9643; 44.7162; 48.3384; 49.1769; 51.5937; 62.4986; 64.0138; 70.3927; 75.7877; 85.3992; 89.4435; 96.9823; 103.7636; 123.0022; 124.0103; 132.3138; 133.7354; 144.9443; 148.9642; 161.6277; 171.1910; 183.6638; 189.2407; 204.0790; 208.5367; 213.2766; 216.0118; 218.6161; 220.1489; 222.5859; 226.1594; 234.4748; 267.0154; 271.4455; 286.5697; 294.0320; 303.2512; 306.8625; 307.7861; 321.6600; 322.7249; 325.3856; 326.8847; 339.2221; 345.9029; 351.7554; 352.5721; 356.7620; 382.4803; 402.9753; 421.5970; 448.9586; 455.7339; 486.3546; 489.0481; 490.2256; 490.8684; 518.3249; 522.3048; 524.3182; 524.4422; 531.1554; 557.4390; 557.6322; 558.5641; 570.0817; 571.5532; 577.3898; 594.8967; 619.8755; 625.7881; 633.1577; 633.8338; 670.1553; 693.2524; 712.1897; 722.4240; 760.2056; 760.4754; 761.6537; 762.7378; 777.0182; 799.4348; 862.0087; 873.5623; 901.0314; 915.3750; 937.3384; 942.8213; 955.8333; 969.5000; 974.8467; 977.0854; 984.2874; 986.0309; 1001.2066; 1016.6129; 1045.5679; 1048.9775; 1069.0218; 1072.3350; 1074.7593; 1079.4218; 1108.4330; 1108.9800; 1122.0475; 1127.9303; 1188.2281; 1203.8995; 1209.2906; 1212.6025; 1215.9214; 1217.7189; 1225.9527; 1229.2031; 1231.2676; 1234.9367; 1235.4014; 1238.0993; 1241.6181; 1253.5514; 1253.6277; 1285.3755; 1293.0080; 1300.2351; 1308.8999; 1319.3751; 1346.0113; 1363.6584; 1393.8686; 1428.6710; 1433.0809; 1446.7967; 1458.7717; 1492.4620; 1500.6084; 1513.7012; 1516.0385; 1517.3406; 1519.3990; 1529.3127; 1537.8072; 1539.4833; 1553.4490; 1617.1044; 1639.1826; 1659.1328; 1762.0110; 3042.6779; 3054.0972; 3056.0106; 3063.2305; 3101.2846; 3120.1022; 3123.2999; 3126.9435; 3128.0371; 3134.1550; 3163.7090; 3167.6610; 3169.1986; 3181.4750; 3193.4160; 3200.3613; 3244.1397.

Cartesian Forces: Max 0.000012247 RMS 0.000002071

Item	Value	ThresholSd	Converged?
Maximum Force	0.000009	0.000450	YES
RMS Force	0.000001	0.000300	YES
Maximum Displacement	0.001150	0.001800	YES
RMS Displacement	0.000208	0.001200	YES

FILE: Yb_Sq2_PhYbOTf3_Prod_Anion_B3LYP_M062x_Add12_Conf8_a.out

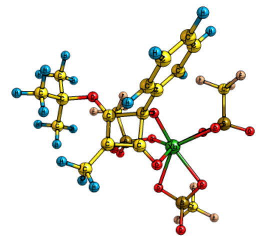

Conformer 16

E(RB3LYP/6-31G(d)/MWB59) = -3731.53166712
Convg = 0.3741D-08 -V/T = 2.0122

Temperature 195.150 Kelvin. Pressure 1.00000 Atm.
elec. and zero-point Energies= -3731.157008
elec. and thermal Energies= -3731.134228
elec. and thermal Enthalpies= -3731.133610
elec. and thermal Free Energies= -3731.202793

RB3LYP/6-31G(d)/MWB59/THF(IEFPCM) = -3731.59918586

RM06-2x/6-311+G(d,p)/MWB59/THF(IEFPCM) = -3731.43195413

Geometry at RB3LYP/6-31G(d)/MWB59
Charge = -1 Multiplicity = 1

```
Yb 0.1732735138 -0.2793610797 3.501561869
O 1.5294151213 1.2900603904 4.6972509753
S 1.1825839235 2.530600085 3.9086567835
O 0.8572664 3.7399994797 4.6528942766
O 0.2416099024 2.0839912448 2.8261184662
C 2.7685389548 2.9264241499 3.0128537427
F 3.6851855063 3.3662420855 3.8820015007
F 3.2500697913 1.8382880872 2.3965303141
F 2.5492582969 3.8768918303 2.0955794686
O -1.245465394 -1.595939566 4.9546969661
O -3.5825841064 -0.743945596 5.4635761852
O -1.6682961338 0.7646029305 4.7494931754
S -2.1479294208 -0.4874096067 5.4329190541
C -1.6464486798 -0.250800543 7.2103557096
F -1.9537296161 -1.3424892269 7.9202899406
F -0.3295438399 -0.0344284619 7.2955692819
F -2.2932445356 0.7994182285 7.730407131
C 0.2081300606 -2.4072596328 0.0155424119
C -1.134855661 -2.5845053496 0.3270494119
C -1.0929180545 -1.3261777827 1.0247010729
C 0.3000059163 -0.9311943994 0.5134612981
O 1.0964231177 -0.6635095602 1.5920218829
O -1.6635462536 -0.8322271381 2.0186137986
O 1.2568984971 -3.0626709912 -0.4156755838
C 1.3422700006 -4.505562443 -0.725296028
C -2.1939082506 -3.6401062812 0.3328777237
H -2.1305323251 -4.3153717791 -0.5260915748
H -3.1816961414 -3.166988543 0.3173535602
H -2.1403526757 -4.2417628763 1.249119479
C 0.7989227879 -5.3604785384 0.4201571312
H 1.0883324117 -6.4037778363 0.2475031159
H 1.209197862 -5.0348541639 1.3782564585
H -0.2893160339 -5.3265370097 0.4867865251
C 2.8539016764 -4.7058936752 -0.8696493614
H 3.0715789525 -5.7401352958 -1.1577241267
H 3.2591828009 -4.0350349214 -1.633929266
H 3.3507649768 -4.4917790108 0.0806221291
C 0.6181417193 -4.7490978494 -2.0520764273
H -0.4518409683 -4.5344306479 -1.9699553619
H 1.0371577896 -4.1156542823 -2.8409824989
H 0.7299115087 -5.7971572835 -2.3525414786
C 0.4060363491 0.0879763356 -0.6211296627
C -0.3897716482 0.0071813325 -1.7735271737
C 1.3260611114 1.1356050657 -0.5113051511
H -1.1113250489 -0.7990526821 -1.8760737036
H 1.9341279332 1.1959714193 0.3820558344
C -0.2690522036 0.9537602302 -2.7909796994
C 1.4499462826 2.0809344188 -1.5314756519
H -0.8993011794 0.8780335751 -3.6744326648
H 2.16743872 2.8911764043 -1.4249290423
C 0.6541730984 1.9958106338 -2.674768189
H 0.7478491352 2.7356062271 -3.4664485866
O 1.8811881453 -3.9601340207 5.3717098115
S 1.7363575175 -3.1237627151 4.1813190889
C 3.3508141496 -3.337248584 3.2723394614
O 1.737039798 -1.6290625337 4.4624899274
O 0.6972738098 -3.4769164758 3.1916587139
F 3.4506030724 -2.5203407199 2.221076674
F 3.4347587251 -4.6088283616 2.8178434803
F 4.3885150248 -3.1168989626 4.0886255149
```

Frequency: 13.2078; 17.7609; 19.9289; 23.1396; 27.1406; 29.1933; 31.6792; 34.7760; 38.5135; 41.0794; 48.3429; 49.1016; 53.6252; 58.7196; 60.0118; 60.6543; 69.9319; 71.1985; 78.2414; 82.9528; 90.8416; 92.4437; 116.7287; 138.4307; 141.6191; 148.5748; 153.9725; 156.1235; 172.6083; 174.3243; 176.9017; 188.2385; 196.9613; 205.1549; 209.4413; 211.4588; 214.3790; 222.3777; 222.9500; 232.1405; 233.5059; 255.4650; 261.4795; 274.2508; 290.0713; 303.0510; 305.1261; 307.1439; 320.4171; 321.7569; 323.8642; 330.9655; 338.2551; 353.5159; 354.2233; 354.5546; 358.9563; 393.2098; 399.8028; 421.2704; 456.4893; 461.5298; 483.8237; 490.5509; 490.7539; 495.7703; 501.0022; 518.8724; 525.2605; 528.0244; 537.4586; 557.2262; 557.8181; 558.2062; 571.6111; 573.4171; 577.5601; 614.0228; 623.1173; 629.2770; 634.3522; 639.0865; 679.3669; 691.0555; 714.3142; 725.3058; 753.8482; 754.8811; 759.0765; 761.1235; 785.0803; 807.9335; 853.9809; 865.1639; 897.8826; 922.0195; 936.8589; 940.7937; 964.0954; 969.9831; 972.1318; 974.9253; 982.5119; 984.2628; 1008.1219; 1018.1273; 1035.1699; 1052.7888; 1061.5358; 1065.5267; 1072.2854; 1084.7901; 1105.7159; 1112.1667; 1120.5938; 1142.5273; 1186.8031; 1190.3776; 1204.9726; 1211.1392; 1213.3787; 1215.9166; 1216.3898; 1227.1630; 1230.4012; 1237.1828; 1237.7367; 1239.3305; 1241.1920; 1241.5071; 1247.7441; 1285.6523; 1294.8668; 1306.4586; 1309.8229; 1319.3093; 1347.9868; 1363.2550; 1395.5326; 1429.6219; 1438.4573; 1444.4055; 1463.3983; 1493.3860; 1502.9782; 1509.8088; 1516.6076; 1518.2353; 1522.2934; 1528.3909; 1534.5881; 1540.2437; 1550.1515; 1606.5390; 1640.8369; 1660.5995; 1755.0783; 3047.2502; 3056.5566; 3062.1126; 3072.1990; 3106.6546; 3123.4938; 3124.7855; 3132.3273; 3137.1391; 3151.7099; 3155.0338; 3167.2623; 3179.8151; 3188.3859; 3191.8450; 3199.6150; 3263.4813.

Cartesian Forces: Max 0.000005388 RMS 0.000001183

Item	Value	Threshold	Converged?
Maximum Force	0.000005	0.000450	YES
RMS Force	0.000001	0.000300	YES
Maximum Displacement	0.001113	0.001800	YES
RMS Displacement	0.000218	0.001200	YES

FILE: Yb_Sq2_PhYbOTf3_Prod_Anion_B3LYP_M062x_Add12_Conf8-1_a.out

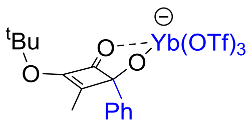

C-2 addition product from $[PhYb(OTf)_3]^-$ + 1b

Lowest energy conformation is: Conformer 3

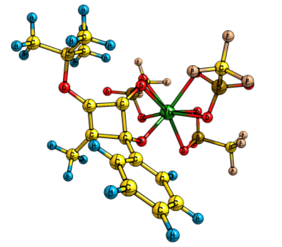

Conformer 1

E(RB3LYP/6-31G(d)/MWB59) = -3731.53054188
Convg = 0.8049D-08 -V/T = 2.0122

Temperature 195.150 Kelvin. Pressure 1.00000 Atm.
elec. and zero-point Energies= -3731.156653
elec. and thermal Energies= -3731.133699
elec. and thermal Enthalpies= -3731.133081
elec. and thermal Free Energies= -3731.203409

RB3LYP/6-31G(d)/MWB59/THF(IEFPCM) = -3731.59374199

RM06-2x/6-311+G(d,p)/MWB59/THF(IEFPCM) = -3731.42949974

Geometry at RB3LYP/6-31G(d)/MWB59
Charge = -1 Multiplicity = 1

```
Yb -0.2671193423 -0.4788944756 3.643246228
O 1.4398165323 1.3035439144 3.936005893
S 0.5247582201 2.4378768143 3.5809961514
O 0.4254256055 3.557290258 4.5103532755
O -0.7694224767 1.8027387434 3.1203589968
C 1.239443043 3.2002189571 2.0350023781
F 2.4897441369 3.6130104559 2.2834518954
F 1.265318142 2.3388403765 1.0166267894
F 0.498181857 4.2586456859 1.6816735884
O -0.5301203378 0.1458378046 5.9947741807
O -2.4792501426 -0.9678836221 7.1789753456
O -2.4641185743 -0.4432507273 4.6935817349
S -2.0125018001 -0.1143923859 6.0920951491
C -2.7645031092 1.5519944881 6.4565009242
F -2.3187808522 1.9932034786 7.639562397
F -2.4586358089 2.4437785865 5.5160636143
F -4.0988084678 1.4282582859 6.5180815879
C 0.6338732018 -2.2960725956 -0.1552215787
C -0.679900224 -2.6127277934 0.0583263182
C -1.0493846524 -1.2691330965 0.7357553421
C 0.4844713247 -1.0984546224 0.6850445764
O 1.1728590474 -0.4638292276 1.489237799
O -1.4311041557 -1.3144297349 2.0505376273
O 1.5942051712 -2.9871514767 -0.783877835
C 3.0184884906 -2.7407017792 -0.5063303796
C -1.4796671777 -3.85291714 -0.0958599856
H -0.8780533356 -4.6783089413 -0.488738522
H -2.3326514016 -3.6802679389 -0.766250181
H -1.8919481475 -4.1419426425 0.8787425727
C 3.3911086751 -1.3092662518 -0.909178844
H 4.4770853788 -1.1788913381 -0.8370576516
H 3.0904333367 -1.1101557926 -1.9441777908
H 2.9258473711 -0.5726106805 -0.2494646404
C 3.7098375698 -3.7606829973 -1.4149932368
H 4.7971467055 -3.6859390304 -1.3076497062
H 3.4034404711 -4.7778717378 -1.1500813767
H 3.4496424575 -3.5830035382 -2.464225573
C 3.3176465828 -3.0202033613 0.9687353026
H 2.8479895202 -2.284812591 1.6261869353
H 2.9642559976 -4.0159278401 1.255362794
H 4.4000262759 -2.9774595304 1.1390767459
C -1.9035310457 -0.3348057237 -0.1219302583
C -1.7173153806 -0.2204753476 -1.5081128226
C -2.9032487252 0.4326252006 0.4878461841
H -0.9491045788 -0.8105391542 -2.0007187609
H -3.0460978695 0.3408110288 1.5583212109
C -2.5091708166 0.6433679707 -2.2654166995
C -3.6972592339 1.2949611647 -0.2709637187
H -2.3479237394 0.7201243153 -3.3385501064
H -4.467155667 1.8844982851 0.2209937273
C -3.5047453535 1.4050952192 -1.6490677855
H -4.1227925614 2.078669096 -2.2382828168
O 1.4393656717 -4.3340954997 4.003632258
S 1.059966094 -3.0499775233 4.5883064912
C 1.7043561274 -3.1044854571 6.3356785342
O -0.4009487596 -2.737862043 4.7086296328
O 1.702339763 -1.8182652469 3.9933618746
F 1.4856644719 -1.9497631533 6.9610999659
F 3.0227832897 -3.346229239 6.314823025
F 1.0957075324 -4.0907160615 7.0046577292
```

Frequency: 8.2955; 14.9676; 20.8495; 23.7744; 25.2931; 28.5609; 29.3531; 30.6453; 35.1622; 37.0337; 38.9852; 44.5311; 47.4337; 50.0846; 54.1739; 64.8426; 67.6118; 72.6921; 83.9511; 86.4336; 91.8553; 103.2113; 126.8031; 129.5447; 134.5883; 139.5495; 143.5137; 147.7398; 155.1044; 160.8241; 171.9718; 180.4534; 198.0962; 207.0886; 214.2121; 215.0234; 216.9458; 221.0864; 221.8112; 225.4324; 254.1756; 259.9925; 267.8174; 273.7150; 281.5472; 296.1489; 304.0482; 306.8719; 308.9555; 321.7336; 323.9157; 324.6949; 342.3748; 352.0309; 352.9118; 357.4481; 364.2760; 385.0643; 412.2133; 422.3034; 450.3825; 452.9803; 483.9717; 489.4815; 490.4422; 491.9001; 524.8235; 524.9344; 529.4372; 540.3504; 542.4957; 558.0875; 558.2433; 558.8444; 570.5122; 571.9738; 573.5280; 588.2709; 625.3196; 626.6692; 633.3489; 634.9333; 637.3278; 665.3681; 709.8679; 715.5186; 750.3209; 761.1778; 762.1514; 762.9615; 765.8313; 796.7061; 863.4784; 864.9370; 918.8142; 932.0660; 935.2829; 943.9020; 966.2604; 970.8034; 972.9824; 976.2006; 979.2989; 983.0586; 1001.9010; 1016.5763; 1028.6485; 1057.2069; 1060.0716; 1064.1362; 1069.6832; 1081.7555; 1104.5549; 1106.7710; 1115.2156; 1128.4678; 1154.6037; 1187.9752; 1208.2923; 1211.4242; 1212.3581; 1213.1382; 1218.9488; 1231.0038; 1232.2717; 1235.0487; 1242.8768; 1247.5096; 1253.2988; 1255.8277; 1259.8061; 1280.5266; 1295.1950; 1299.5640; 1305.5932; 1312.6093; 1346.5940; 1362.5888; 1364.4313; 1427.2737; 1429.7558; 1433.3442; 1458.7682; 1492.3923; 1501.7595; 1503.8413; 1507.3747; 1516.9301; 1518.7258; 1534.7052; 1537.8782; 1539.9914; 1553.6988; 1639.2659; 1657.5766; 1662.8852; 1762.4223; 3037.5718; 3052.3840; 3055.2897; 3062.7965; 3095.2092; 3115.7421; 3124.3045; 3127.5964; 3131.4902; 3133.6986; 3154.0248; 3163.6039; 3167.3778; 3180.8765; 3193.1251; 3200.2279; 3240.7185.

Cartesian Forces: Max 0.000001005 RMS 0.000000290

Item	Value	Threshold	Converged?
Maximum Force	0.000001	0.000450	YES
RMS Force	0.000000	0.000300	YES
Maximum Displacement	0.000042	0.001800	YES
RMS Displacement	0.000008	0.001200	YES

FILE: Yb_Sq2_PhYbOTf3_Prod_Anion_B3LYP_M062x_Add56_Conf1.out

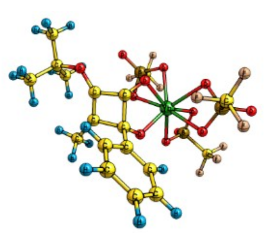

Conformer 2

E(RB3LYP/6-31G(d)/MWB59) = -3731.52353179
Convg = 0.6166D-09 -V/T = 2.0122

Temperature 195.150 Kelvin. Pressure 1.00000 Atm.
elec. and zero-point Energies= -3731.149658
elec. and thermal Energies= -3731.126734
elec. and thermal Enthalpies= -3731.126116
elec. and thermal Free Energies= -3731.196048

RB3LYP/6-31G(d)/MWB59/THF(IEFPCM) = -3731.58858886

RM06-2x/6-311+G(d,p)/MWB59/THF(IEFPCM) = -3731.42518540

Geometry at RB3LYP/6-31G(d)/MWB59
Charge = -1 Multiplicity = 1

```
Yb -0.1531611675 -0.4387785108 3.5088004926
O 1.3713643198 1.4821808095 3.6587566518
S 0.3293114143 2.5506117214 3.4964087447
O 0.3373405119 3.6694211142 4.431352084
O -0.9780416178 1.8261222435 3.2834446528
C 0.6886726031 3.3135240005 1.8352851326
F 1.8990173341 3.8855269066 1.8525093048
F 0.6615349392 2.3907440914 0.8687085444
F -0.2277878165 4.2509510709 1.561637343
O -0.2443868559 -0.0365222375 5.9277323948
O -1.8054985071 -1.5362187118 7.2516023138
O -2.1572645137 -0.9557109611 4.8065445327
S -1.6304625329 -0.578265751 6.1650021445
C -2.6450307172 0.8981657041 6.6799870455
F -2.1686114785 1.3862833593 7.8321967603
F -2.6154326096 1.8571928986 5.7561893706
F -3.9166262192 0.5158701736 6.8672984312
C 0.2627474671 -2.4202673189 -0.1396724844
C -1.0926589087 -2.4789981984 0.0753497822
C -1.2004543594 -1.0267701327 0.6537398664
C 0.3255936835 -1.1723913966 0.6179473859
O 1.1290799455 -0.6790503542 1.4207163876
O -1.5880413759 -0.9104637675 1.9580942173
O 1.3129003187 -3.1095976529 -0.5987886537
C 1.333324883 -4.5576959287 -0.8070876169
C -2.2124035509 -3.4562868009 0.0979819698
H -1.9585051696 -4.448003226 -0.2824614997
H -3.0551886733 -3.059444791 -0.4838578423
H -2.5626531314 -3.5567228951 1.13324014
C 0.9057004112 -5.2877177562 0.4696087382
H 1.0448127512 -6.3673116591 0.3377501147
H 1.511608861 -4.9546871465 1.3173986822
H -0.1440940826 -5.114103799 0.7171818589
C 2.813544923 -4.8266846788 -1.0978805917
H 2.9740052817 -5.8915283589 -1.2997671039
H 3.1435694816 -4.2514491363 -1.9694252971
H 3.4235608444 -4.5348314954 -0.2381263128
C 0.4749079336 -4.9125503593 -2.0276220018
H -0.5790615789 -4.666791142 -1.8736690835
H 0.8270403456 -4.3675329106 -2.9102193506
H 0.5426843955 -5.9865128483 -2.2364842066
C -1.8742393749 -0.0273748582 -0.2861196425
C -1.707046926 -0.0772224019 -1.6781832418
C -2.6856370307 0.9750401744 0.2592951885
H -1.0808242133 -0.8478949163 -2.1194870863
H -2.8056124499 1.0101813743 1.3357764975
C -2.335834349 0.854084468 -2.5057178181
C -3.3157688229 1.9055852951 -0.5693349586
H -2.1926026141 0.800832003 -3.5828603792
H -3.9400821926 2.6788972517 -0.1279304101
C -3.1451614164 1.8498912533 -1.9538864228
H -3.6355479671 2.5764191571 -2.5978297171
O 2.5233343872 -3.7187069533 3.2329892708
S 1.6514240243 -2.849430724 4.0245149343
C 1.6929982727 -3.585170399 5.7354357071
O 0.1875112287 -2.8438459401 3.6530211608
O 2.0484423113 -1.416911542 4.2003049231
F 0.9021024501 -2.9103159771 6.5666118558
F 2.9478980113 -3.5565874347 6.2031345247
F 1.283162312 -4.860563887 5.6814026073
```

Frequency: 13.6887; 14.2875; 19.7904; 21.5958; 26.2038; 28.5172; 30.9535; 32.2989; 33.6931; 37.0150; 39.0494; 46.3774; 47.3546; 56.5766; 61.1727; 65.7841; 67.4818; 73.1915; 78.4021; 87.4794; 90.5964; 101.2028; 123.1160; 130.4966; 136.2559; 143.3213; 144.1394; 150.5784; 170.6978; 173.3094; 175.4909; 185.1032; 191.6810; 209.1450; 211.1761; 212.0307; 214.6186; 217.1714; 220.1050; 227.6125; 239.7787; 252.0422; 265.5051; 267.4700; 277.9908; 291.4083; 303.1180; 306.4970; 308.1498; 321.0984; 322.7635; 323.4525; 348.1853; 351.6554; 352.5096; 356.8536; 367.2766; 381.9997; 413.0529; 422.1049; 456.9317; 464.3451; 476.3678; 489.1243; 489.9468; 491.1370; 521.5284; 523.7916; 525.5728; 529.8919; 539.0559; 557.1960; 558.0310; 558.2675; 571.0178; 573.2701; 573.7287; 581.1860; 625.1770; 627.0413; 633.4044; 633.9384; 648.8159; 685.3423; 711.7960; 730.4005; 742.6542; 760.8451; 762.2383; 762.5506; 770.5227; 794.3586; 840.1107; 864.0398; 913.1435; 930.1012; 932.8096; 953.3442; 962.4191; 970.9631; 977.0214; 977.3117; 979.9027; 983.9131; 1000.7199; 1016.9110; 1031.7802; 1052.9948; 1059.5209; 1061.9750; 1070.6551; 1094.8101; 1106.1957; 1111.4663; 1114.9687; 1134.8668; 1141.5896; 1187.6250; 1206.7236; 1212.0139; 1212.9298; 1216.3927; 1221.3842; 1231.3931; 1233.0540; 1235.0468; 1237.0976; 1246.8638; 1248.8573; 1252.7310; 1254.0102; 1280.3576; 1290.1319; 1293.1665; 1305.7319; 1314.4945; 1346.0210; 1351.1079; 1362.7436; 1425.6867; 1429.7259; 1434.8836; 1460.1467; 1492.3649; 1500.3996; 1503.9515; 1508.7846; 1518.8260; 1522.6837; 1529.1275; 1531.6495; 1539.6067; 1553.0870; 1638.9312; 1647.8834; 1659.4658; 1756.0625; 3041.8018; 3055.3335; 3058.0777; 3065.8285; 3094.5101; 3119.2636; 3127.8282; 3135.3938; 3141.9815; 3144.5954; 3151.3551; 3163.4078; 3166.6742; 3179.9396; 3192.6800; 3200.0811; 3244.9301.

Cartesian Forces: Max 0.000001316 RMS 0.000000503

Item	Value	Threshold	Converged?
Maximum Force	0.000000	0.000450	YES
RMS Force	0.000000	0.000300	YES
Maximum Displacement	0.000058	0.001800	YES
RMS Displacement	0.000011	0.001200	YES

FILE: Yb_Sq2_PhYbOTf3_Prod_Anion_B3LYP_M062x_Add56_Conf1-1.out

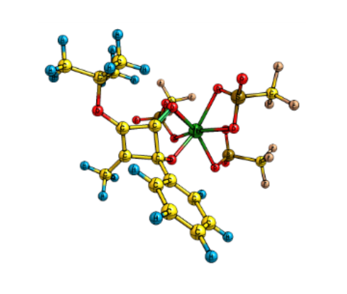

Conformer 3

E(RB3LYP/6-31G(d)/MWB59) = -3731.53132103
Convg = 0.6720D-08 -V/T = 2.0122

Temperature 195.150 Kelvin. Pressure 1.00000 Atm.
elec. and zero-point Energies= -3731.157326
elec. and thermal Energies= -3731.134458
elec. and thermal Enthalpies= -3731.133840
elec. and thermal Free Energies= -3731.203531

RB3LYP/6-31G(d)/MWB59/THF(IEFPCM) = -3731.59461331

RM06-2x/6-311+G(d,p)/MWB59/THF(IEFPCM) = -3731.43158265

Geometry at RB3LYP/6-31G(d)/MWB59
Charge = -1 Multiplicity = 1
Yb -0.3696168426 -0.55445376 3.7168258717
O -0.5529864918 1.7547917997 3.1242515966
S 0.8614439385 2.1884608176 3.4443573994
O 1.5937545208 2.8883267301 2.3947471407
O 1.5185606062 1.0143629605 4.1098580564
C 0.6704092438 3.471142112 4.7876183985
F -0.1035199364 4.469814879 4.3431591026
F 0.1280927427 2.9619520614 5.8935408037
F 1.8790823123 3.9650110569 5.0916196833
O -0.7890336157 0.1800610288 6.0298188255
O -2.921927552 -0.7167974392 7.0704089934
O -2.6344972117 -0.3311980246 4.5751109592
S -2.2870497571 0.0354943579 5.9944629895
C -2.9433669741 1.7703511156 6.1989351667
F -2.4783964461 2.2966988197 7.3391999785
F -2.5843146261 2.5562040321 5.1813136813
F -4.2821552038 1.7321965939 6.2564824155
C 0.6639987254 -2.2217730522 -0.1371664938
C -0.6228005236 -2.6347745842 0.0729687064
C -1.0783068319 -1.340877674 0.7922622638
C 0.4402616961 -1.0611594452 0.739150476
O 1.0922793389 -0.4015623568 1.5509555742
O -1.4516073903 -1.4487598773 2.1074649048
O 1.6645853176 -2.8226840427 -0.7961005331
C 3.0704224506 -2.4993459583 -0.5049128711
C -1.339553814 -3.9188310286 -0.1249731699
H -0.6874679525 -4.6857409255 -0.5542876901
H -2.2078449451 -3.7778257138 -0.7829821117
H -1.7230146275 -4.2743212715 0.8393862279
C 3.3521093087 -1.0260668467 -0.8209919143
H 4.4297750993 -0.8371148911 -0.7539575671
H 3.0256300816 -0.7830096259 -1.8386207831
H 2.8553905227 -0.3588172882 -0.1125173168
C 3.8244001581 -3.417488869 -1.470729097
H 4.9049930451 -3.2858821523 -1.3506970344
H 3.5774667432 -4.4659115438 -1.2738453839
H 3.5584707585 -3.188574844 -2.5085038691
C 3.3880571714 -2.8477999334 0.9517880157
H 2.8422280426 -2.2100033926 1.6512265694
H 3.1333346671 -3.892209893 1.1612276709
H 4.4596993604 -2.7110142244 1.1390065055
C -2.0047497303 -0.4477956182 -0.0337313578
C -1.8104699735 -0.246425296 -1.4087667552
C -3.084466704 0.1861019393 0.5930462549
H -0.9806011136 -0.7327728138 -1.9144294698
H -3.2339595516 0.0263654306 1.6549453254
C -2.6718661519 0.5735639891 -2.1381493331
C -3.9487027929 1.0033060283 -0.1385799705
H -2.5018748337 0.7213736232 -3.2024291259
H -4.7808545295 1.4885607212 0.3659390063
C -3.7471851186 1.2016819567 -1.5053782381
H -4.4194225039 1.840958088 -2.0730924486
O 0.9120665209 -4.5685420141 4.2474673912
S 0.7408051998 -3.196158154 4.712889754
C 1.5784743875 -3.1280687232 6.3748838581
O -0.6506603496 -2.6987576219 4.9605103021
O 1.4373116964 -2.1131296896 3.9196338789
F 1.5412558816 -1.8885971696 6.8678415153
F 2.8573554147 -3.5078115692 6.2507113348
F 0.9625854539 -3.9568674887 7.2252244021

Frequency: 12.6164; 13.0407; 19.7499; 23.3588; 25.3554; 27.6651; 30.7125; 33.1226; 36.0902; 37.2071; 45.9761; 49.6894; 53.1920; 55.3973; 62.1989; 68.0288; 71.3260; 73.1394; 84.6417; 85.7800; 89.6722; 102.8999; 127.7204; 130.4335; 133.3652; 139.1572; 143.7609; 146.1590; 148.5501; 161.5620; 174.6054; 180.1598; 197.9277; 207.5956; 210.9719; 216.0245; 219.0408; 220.0458; 223.4368; 230.9759; 252.9659; 261.2934; 267.9218; 273.1745; 282.6219; 297.4797; 303.1053; 307.0293; 309.1840; 321.5162; 323.9591; 326.8514; 342.9894; 351.7372; 353.4035; 357.3938; 364.5368; 384.9126; 412.2028; 422.0644; 450.2267; 453.6736; 485.3487; 490.2024; 490.5294; 491.7608; 523.8830; 524.7597; 529.4692; 540.3413; 542.9290; 558.2328; 558.4350; 558.9194; 569.3934; 572.0120; 575.4215; 590.6503; 621.2469; 623.9154; 633.3370; 636.0319; 638.3012; 665.4083; 710.3093; 716.0426; 751.9484; 760.1013; 760.9934; 762.0898; 765.6720; 796.7494; 863.8912; 865.1883; 919.1659; 932.5611; 935.2190; 944.3176; 966.6586; 969.1741; 971.1321; 976.1958; 980.8866; 983.8641; 1004.2879; 1016.3486; 1028.1425; 1057.9318; 1060.1722; 1065.0239; 1070.0847; 1082.0524; 1104.7631; 1106.9719; 1117.7084; 1125.7447; 1154.3080; 1187.9918; 1208.3462; 1210.4774; 1213.3417; 1215.3486; 1219.4316; 1225.8569; 1228.8261; 1236.9334; 1243.1931; 1245.5982; 1248.3888; 1254.2051; 1259.2596; 1281.4405; 1295.3718; 1301.9997; 1304.7507; 1317.1345; 1346.6105; 1362.8403; 1364.6249; 1427.5758; 1430.1605; 1433.9301; 1458.9181; 1492.4904; 1502.1752; 1503.7349; 1507.5797; 1516.2272; 1518.9781; 1534.6254; 1537.7923; 1540.1143; 1554.0093; 1639.4255; 1658.6512; 1665.6104; 1766.9210; 3037.5094; 3052.4793; 3054.4815; 3061.8380; 3094.8453; 3115.5278; 3120.6245; 3127.3102; 3130.7518; 3132.5851; 3157.2479; 3162.8324; 3167.3726; 3180.3848; 3192.8066; 3200.2662; 3236.2134.

Cartesian Forces: Max 0.000000579 RMS 0.000000224

Item	Value	Threshold	Converged?
Maximum Force	0.000001	0.000450	YES
RMS Force	0.000000	0.000300	YES
Maximum Displacement	0.000043	0.001800	YES
RMS Displacement	0.000008	0.001200	YES

FILE: Yb_Sq2_PhYbOTf3_Prod_Anion_B3LYP_M062x_Add56_Conf2.out

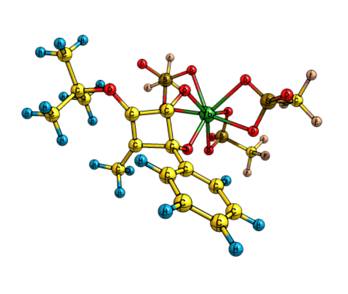

Conformer 4

E(RB3LYP/6-31G(d)/MWB59) = -3731.52252828
Convg = 0.8970D-08 -V/T = 2.0122

Temperature 195.150 Kelvin. Pressure 1.00000 Atm.
elec. and zero-point Energies= -3731.148608
elec. and thermal Energies= -3731.125736
elec. and thermal Enthalpies= -3731.125118
elec. and thermal Free Energies= -3731.194633

RB3LYP/6-31G(d)/MWB59/THF(IEFPCM) = -3731.58744319

RM06-2x/6-311+G(d,p)/MWB59/THF(IEFPCM) = -3731.42408990

Geometry at RB3LYP/6-31G(d)/MWB59
Charge = -1 Multiplicity = 1
Yb -0.2463137617 -0.3978635827 3.5019394708
O -1.067265654 1.8605525771 3.2289994511
S 0.2543697049 2.5886398283 3.3068976333
O 0.549770984 3.5401554728 2.2414125271
O 1.2809155783 1.5418736986 3.626391031
C 0.1290015476 3.6392491811 4.8427350892
F -0.8856384967 4.5040559102 4.7092779818
F -0.0755971347 2.898172388 5.9322125953
F 1.2661736071 4.3299867222 4.9984588025
O -0.3638574702 -0.0597742554 5.9221259077
O -1.9752534766 -1.5337401981 7.211221602
O -2.2876413695 -0.9132395795 4.7689164495
S -1.7656215607 -0.5702011114 6.1359996521
C -2.7465952982 0.9272810613 6.6587486367
F -2.2223064416 1.4401001719 7.7791374588
F -2.7463510517 1.8672655907 5.7113140119
F -4.0132967812 0.5641153256 6.9027374882
C 0.2296626407 -2.3841937147 -0.1277695591
C -1.1220196962 -2.4896192416 0.0928838094
C -1.2812148625 -1.0348623944 0.6524595258
C 0.2483430737 -1.1263064564 0.6155763363
O 1.0332395232 -0.596219035 1.4139334653
O -1.6684777571 -0.9165727913 1.957353791
O 1.3038999688 -3.0399703387 -0.5798097164
C 1.3658530158 -4.4840104602 -0.8037930492
C -2.2056930408 -3.5064148439 0.1333303189
H -1.9198440923 -4.4915111899 -0.2415971117
H -3.0672746381 -3.1446256305 -0.4437223247
H -2.5426960219 -3.6109328137 1.1725673145
C 0.9709742772 -5.2388710373 0.469149619
H 1.1422432394 -6.3124854229 0.3265041922
H 1.5720781935 -4.8944803066 1.3157940448
H -0.0816939531 -5.0997673272 0.7260579839
C 2.8509935851 -4.70558369 -1.1093134897
H 3.0417413972 -5.7633275525 -1.3219047676
H 3.15600879 -4.1130259374 -1.9783120892
H 3.4595947646 -4.4028241529 -0.2523141723
C 0.5087272684 -4.8531412374 -2.0210356648
H -0.5509704994 -4.6411746294 -1.8567980937
H 0.8372280806 -4.2894245569 -2.9009836388
H 0.6075549027 -5.9225402436 -2.2406417471
C -1.9899480495 -0.0702346956 -0.2975019702
C -1.8672738174 -0.1669011495 -1.691736758
C -2.7865337406 0.9485383732 0.2394065293
H -1.2513908283 -0.9495211931 -2.126497921
H -2.871826638 1.0227103984 1.316972356
C -2.5269179671 0.7328632569 -2.5300089462
C -3.4457742525 1.8486258264 -0.5999128324
H -2.4188316001 0.642559161 -3.6087916123
H -4.0560362582 2.6366180543 -0.1650442773
C -3.3212147763 1.7450207421 -1.9864999934
H -3.8346756761 2.4480000227 -2.6385576898
O 2.4770788809 -3.6396187454 3.2643106474
S 1.5895657355 -2.7782361123 4.0467529241
C 1.6373711597 -3.5041577746 5.7620542222
O 0.127061884 -2.7963215231 3.6697959467
O 1.9651092554 -1.3388055018 4.2129468989
F 0.8285405144 -2.8435410533 6.5873771054
F 2.8892350837 -3.4466302351 6.235534712
F 1.255245413 -4.7882394014 5.7116418886

Frequency: 11.7064; 18.6038; 20.4850; 23.9524; 25.8482; 28.4572; 30.2173; 31.5702; 34.5790; 37.7893; 41.5375; 47.5930; 55.5575; 61.8968; 64.8601; 67.0503; 71.1042; 78.5681; 80.3709; 84.7294; 91.5690; 101.8766; 123.9235; 129.4499; 131.8812; 142.4786; 143.6359; 148.4614; 166.8663; 174.4929; 179.5840; 184.0042; 190.6354; 206.8840; 210.1615; 211.2417; 213.6176; 217.2477; 219.9110; 228.6643; 239.1853; 251.5880; 265.3282; 266.8771; 277.7020; 291.1957; 302.2672; 306.2350; 307.8711; 321.1514; 322.4785; 324.3450; 347.8746; 351.3024; 351.3753; 356.4582; 368.1944; 382.5151; 413.5213; 421.9723; 457.0442; 464.3951; 477.5648; 489.5617; 490.9611; 491.7570; 520.0324; 523.6503; 525.5022; 530.4340; 539.6258; 557.8616; 558.1391; 558.5779; 570.4748; 572.6590; 574.1362; 582.9494; 621.8159; 626.7447; 633.4689; 637.1344; 648.9145; 685.0068; 711.0425; 729.7901; 742.3354; 760.9352; 761.7386; 762.4104; 771.1893; 794.3021; 840.2277; 862.0578; 912.6383; 930.0946; 932.7889; 952.9834; 962.7422; 969.4039; 975.7594; 978.0612; 979.6331; 986.5612; 997.5411; 1016.9964; 1032.2261; 1052.9944; 1059.6744; 1062.0318; 1070.7174; 1094.5728; 1106.8911; 1114.2178; 1114.8849; 1134.5232; 1141.7002; 1187.7888; 1207.5480; 1212.3110; 1212.6626; 1214.4338; 1221.9884; 1227.8852; 1229.7509; 1232.2114; 1244.9802; 1247.1876; 1248.6670; 1251.0720; 1254.9026; 1280.2643; 1289.8579; 1293.0385; 1303.6615; 1315.2956; 1346.0881; 1350.9574; 1363.1034; 1425.6247; 1429.6069; 1434.7806; 1460.0570; 1492.4071; 1500.4854; 1504.1608; 1508.8093; 1518.8440; 1522.7464; 1529.0234; 1531.4916; 1539.9826; 1553.1532; 1639.0570; 1647.3966; 1659.8063; 1754.8348; 3042.0550; 3055.4525; 3058.0694; 3065.7791; 3094.7544; 3119.3793; 3128.0590; 3135.4946; 3142.1600; 3144.3781; 3151.4146; 3163.5571; 3166.4114; 3180.0297; 3192.7395; 3199.9338; 3247.8418.

Cartesian Forces: Max 0.000001554 RMS 0.000000521

Item	Value	Threshold	Converged?
Maximum Force	0.000001	0.000450	YES
RMS Force	0.000000	0.000300	YES
Maximum Displacement	0.000094	0.001800	YES
RMS Displacement	0.000018	0.001200	YES

FILE: Yb_Sq2_PhYbOTf3_Prod_Anion_B3LYP_M062x_Add56_Conf2-1.out

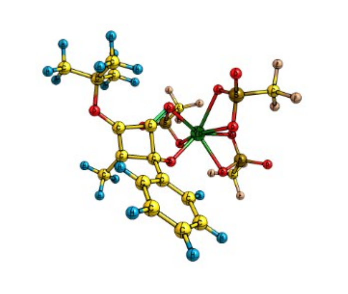

Conformer 5

E(RB3LYP/6-31G(d)/MWB59) = -3731.53091949
Convg = 0.3833D-08 -V/T = 2.0122

Temperature 195.150 Kelvin. Pressure 1.00000 Atm.
elec. and zero-point Energies= -3731.157000
elec. and thermal Energies= -3731.134032
elec. and thermal Enthalpies= -3731.133414
elec. and thermal Free Energies= -3731.203890

RB3LYP/6-31G(d)/MWB59/THF(IEFPCM) = -3731.59372669

RM06-2x/6-311+G(d,p)/MWB59/THF(IEFPCM) = -3731.43006721

Geometry at RB3LYP/6-31G(d)/MWB59
Charge = -1 Multiplicity = 1
Yb -0.2397999196 -0.5765574124 3.7607509979
O -0.4194158938 1.7426952291 3.1837538074
S 1.0062117677 2.1572957623 3.4773967539
O 1.7322524336 2.8499839734 2.4184638323
O 1.6598190181 0.9668521969 4.1215334169
C 0.8522089027 3.4289467462 4.8329840261
F 0.1837738037 4.4921546421 4.3675970206
F 0.2146940389 2.9443533479 5.8947197816
F 2.0802076798 3.8210131175 5.2041026761
O -2.4878126989 -0.125803376 4.5747060848
O -2.6622843504 1.6131760072 6.4151159539
O -0.6424120401 0.1127163902 6.0977192067
S -2.1253106953 0.3341055641 5.9629777158
C -2.9145728257 -0.9179651748 7.0975491278
F -4.2466930323 -0.7709549339 7.0561671719
F -2.6100795585 -2.1662371304 6.7484043826
F -2.4974459423 -0.7030093032 8.3531678337
C 0.6806788755 -2.2506560969 -0.1166647428
C -0.5848582488 -2.6976881418 0.146582028
C -1.047049557 -1.4130097515 0.8781786
C 0.4599156253 -1.0932226655 0.7640693593
O 1.1250351104 -0.4130635375 1.548244079
O -1.3620117352 -1.5229499507 2.2087193576
O 1.6708247431 -2.827075266 -0.8119113464
C 3.076819884 -2.4561106838 -0.5847663565
C -1.2723706379 -4.0024883238 -0.0173154185
H -0.6171015468 -4.7523973473 -0.4711362247
H -2.1710276402 -3.8889204301 -0.6388263608
H -1.6047497889 -4.3648949406 0.9632852083
C 3.2964696131 -0.9766667096 -0.9204800049
H 4.3694608167 -0.7532715136 -0.9028855563
H 2.917100104 -0.7505620202 -1.9235548358
H 2.8112939543 -0.3206999398 -0.1937417046
C 3.8172748453 -3.3557860378 -1.5780298875
H 4.8972761354 -3.1892082885 -1.5056167894
H 3.6127142023 -4.4102989983 -1.3656410196
H 3.4995911969 -3.1417797043 -2.6043632218
C 3.4689683395 -2.7845286406 0.8583897303
H 2.9323544376 -2.1610665709 1.5775859159
H 3.2591645417 -3.8356409397 1.0835756454
H 4.5424088484 -2.6102571095 0.9981649432
C -2.0274165136 -0.5457032348 0.0876654458
C -1.919198352 -0.3820241841 -1.3018534964
C -3.0665488892 0.1062008952 0.7632090186
H -1.1212626092 -0.8817511885 -1.8445390198
H -3.1515345605 -0.0213751435 1.8364950173
C -2.8256511237 0.4174678421 -1.9987151388
C -3.9746749686 0.9043366356 0.0642665334
H -2.7229206884 0.5349789154 -3.0753485191
H -4.7727735181 1.405292646 0.6068323828
C -3.8595532092 1.0641011381 -1.3174957331
H -4.5664228494 1.6881668538 -1.8595179977
O 1.1035364227 -4.5876017159 4.2210078832
S 0.9331376929 -3.2183421144 4.6962280656
C 1.8072527201 -3.1513498151 6.3389171725
O -0.4558677399 -2.7369776923 4.9871959398
O 1.5987719323 -2.12470797 3.8953291509
F 1.7591688151 -1.9162114433 6.8438366006
F 3.0887828814 -3.508941041 6.1867102485
F 1.2224931722 -3.9961310779 7.1972919466

Frequency: 9.4610; 14.0092; 18.3107; 22.0806; 25.0040; 25.5372; 27.8644; 28.5801; 35.2646; 38.7236; 39.1150; 45.8115; 49.0316; 53.1855; 59.6682; 61.5392; 68.2854; 73.4730; 83.1871; 85.6076; 89.1293; 103.0418; 120.7578; 132.3030; 138.6254; 142.5707; 144.4243; 145.4663; 147.3631; 162.1696; 172.2831; 180.4800; 198.4458; 206.4821; 213.4961; 215.1278; 216.5113; 218.0121; 221.2517; 229.3474; 254.3053; 259.9396; 267.3961; 272.0829; 282.2649; 297.7644; 303.9378; 307.5220; 309.3745; 321.7783; 323.9692; 324.9057; 342.7192; 351.3576; 353.3190; 357.4481; 364.7958; 384.5780; 412.3428; 422.6746; 450.4991; 453.5329; 484.5007; 489.2342; 490.6421; 491.4930; 524.2759; 524.4217; 529.1265; 540.9948; 542.6542; 558.0696; 558.5402; 558.7181; 570.3037; 571.1052; 574.7721; 589.9938; 622.6090; 626.8091; 633.1316; 634.6333; 637.1673; 665.5472; 710.3450; 716.3659; 751.0366; 761.4157; 761.9423; 762.3367; 766.1384; 796.8818; 864.3593; 865.6404; 920.2654; 931.7780; 935.1028; 944.1780; 966.7927; 970.4955; 971.4979; 977.0705; 980.6833; 984.9055; 1007.2825; 1016.0469; 1028.2910; 1057.9667; 1060.3080; 1064.6078; 1070.1474; 1082.2161; 1100.5996; 1107.9804; 1120.3830; 1126.7778; 1153.6018; 1188.2310; 1209.3982; 1209.8590; 1211.5283; 1216.8179; 1219.2932; 1228.6196; 1230.8225; 1236.7499; 1242.5342; 1243.4257; 1255.7689; 1259.0948; 1259.3020; 1281.2665; 1295.2839; 1301.9388; 1303.4532; 1315.7446; 1346.6725; 1363.2325; 1364.9234; 1427.5968; 1430.1632; 1433.6330; 1458.9131; 1492.3254; 1502.2782; 1503.4917; 1507.8677; 1516.0581; 1518.8457; 1534.4775; 1537.6970; 1540.3784; 1553.7635; 1639.2868; 1658.4163; 1665.3062; 1766.3006; 3037.5622; 3052.6037; 3054.4321; 3061.8377; 3094.8582; 3115.7142; 3120.4704; 3127.3627; 3130.6271; 3132.5029; 3157.4095; 3162.8930; 3167.1977; 3180.8538; 3193.1650; 3200.2203; 3237.7030.

Cartesian Forces: Max 0.000012845 RMS 0.000002198

Item	Value	Threshold	Converged?
Maximum Force	0.000011	0.000450	YES
RMS Force	0.000001	0.000300	YES
Maximum Displacement	0.001295	0.001800	YES
RMS Displacement	0.000247	0.001200	YES

FILE: Yb_Sq2_PhYbOTf3_Prod_Anion_B3LYP_M062x_Add56_Conf3.out

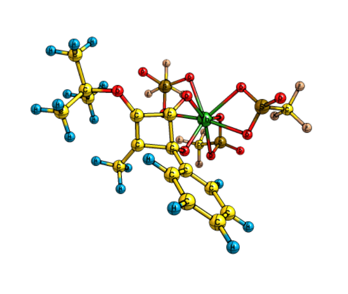

Conformer 6

E(RB3LYP/6-31G(d)/MWB59) = -3731.52230006
Convg = 0.3042D-08 -V/T = 2.0122

Temperature 195.150 Kelvin. Pressure 1.00000 Atm.
elec. and zero-point Energies= -3731.148421
elec. and thermal Energies= -3731.125526
elec. and thermal Enthalpies= -3731.124908
elec. and thermal Free Energies= -3731.194565

RB3LYP/6-31G(d)/MWB59/THF(IEFPCM) = -3731.58726340

RM06-2x/6-311+G(d,p)/MWB59/THF(IEFPCM) = -3731.42320916

Geometry at RB3LYP/6-31G(d)/MWB59
Charge = -1 Multiplicity = 1
Yb -0.1606843569 -0.3898776767 3.5660636
O -0.8489101018 1.8832508224 3.1610319963
S 0.4985327371 2.5551592529 3.2960051895
O 0.8985903812 3.468494814 2.2315447102
O 1.456051226 1.469934997 3.6954543627
C 0.3240746327 3.6296524272 4.807934594
F -0.5804774927 4.5890530423 4.5720872117
F -0.0665119886 2.914622774 5.8621969348
F 1.5016475778 4.2061145108 5.0834467009
O -2.2697198494 -0.415474024 4.837531918
O -2.2570999521 0.8183083722 7.0584998421
O -0.1812173769 -0.1783815688 5.9955923732
S -1.6745431533 -0.2079051143 6.2011476411
C -1.9961604677 -1.8084793928 7.102257942
F -3.303100673 -1.9069904935 7.3817336616
F -1.6371960153 -2.8618323886 6.3638536608
F -1.3076347889 -1.8285399137 8.2503764484
C 0.1948642767 -2.4076324665 -0.0807746017
C -1.1436761416 -2.5398449172 0.197003294
C -1.3037945533 -1.0931228726 0.7773299635
C 0.2235311401 -1.1535887707 0.6699047795
O 1.0349809217 -0.6107986505 1.4324037213
O -1.6341485184 -0.9920055426 2.0990514709
O 1.2604209065 -3.039662919 -0.5849535665
C 1.3277087956 -4.475595549 -0.8527903179
C -2.2089757652 -3.5741479651 0.2679194754
H -1.9311221877 -4.5434948303 -0.1515515343
H -3.1059470668 -3.2089159001 -0.2497828837
H -2.4866107496 -3.7143782525 1.3204438751
C 0.9905926231 -5.2719609842 0.4116273652
H 1.1863755977 -6.3366188815 0.2369170131
H 1.6046963029 -4.9324092943 1.2508730451
H -0.0579349611 -5.1693882393 0.700679904
C 2.8016650177 -4.6719818604 -1.2231170656
H 2.9934134766 -5.7202050122 -1.477694092
H 3.0666785978 -4.0482627881 -2.0833055537
H 3.4416013558 -4.3910107162 -0.3816116589
C 0.4267709536 -4.8193882746 -2.0455971635
H -0.6271178206 -4.6197264769 -1.8348678782
H 0.7169443275 -4.230313918 -2.9223750749
H 0.5240671171 -5.8819056747 -2.2970115787
C -2.0771145428 -0.1388609298 -0.132421904
C -1.9789933096 -0.1934798009 -1.5309214843
C -2.9132185899 0.8229010209 0.4473325517
H -1.3341772338 -0.9324783741 -1.9988514499
H -2.980500168 0.8625860987 1.5280576252
C -2.700025892 0.6933761602 -2.3314591447
C -3.6353939026 1.708967918 -0.3543241182
H -2.6090587041 0.6377177071 -3.4141522694
H -4.2766942755 2.4521166568 0.1135247691
C -3.5336038341 1.6486399162 -1.7453020246
H -4.0952366576 2.341301759 -2.3680339147
O 2.3716754753 -3.7726876061 3.2844379925
S 1.6013762973 -2.8359218919 4.1041109147
C 1.7859770745 -3.5057195338 5.8349017003
O 0.1129616776 -2.7966087367 3.8551237227
O 2.0591411688 -1.4141209252 4.1726522684
F 1.1849364014 -2.7262795374 6.7336627501
F 3.0879836849 -3.5897850968 6.1387510541
F 1.2496953458 -4.7328474882 5.8969042358

Frequency: 10.7568; 16.1198; 19.6749; 24.2865; 28.4637; 29.8632; 32.0268; 32.4984; 34.2381; 35.4393; 41.5869; 46.9629; 55.0977; 59.8615; 64.7745; 70.0639; 72.6426; 76.1785; 79.2046; 80.9349; 92.1363; 101.0014; 121.8681; 128.0611; 131.6678; 142.7749; 143.1067; 148.1875; 166.2667; 172.7593; 177.8061; 184.2995; 190.5527; 207.9046; 209.2993; 212.0844; 213.5649; 216.9308; 219.6353; 228.6755; 238.3641; 252.8421; 264.0663; 266.6098; 279.6739; 292.0199; 302.6294; 306.0914; 307.7172; 321.3657; 321.8016; 324.3399; 347.6420; 349.8881; 352.5674; 356.9395; 368.2612; 382.4612; 414.1524; 421.7529; 456.9117; 463.8815; 476.7575; 489.6468; 490.7028; 491.5887; 520.7062; 523.6015; 525.7026; 530.4649; 538.8863; 557.8269; 558.0830; 558.5319; 570.0930; 573.4922; 574.1410; 582.3393; 621.4456; 627.3368; 633.3633; 636.9487; 648.0139; 684.6074; 710.9316; 729.8061; 742.6800; 760.5641; 761.7344; 762.3292; 770.6025; 794.8886; 840.5421; 861.9594; 912.7382; 929.7729; 932.7263; 953.2349; 963.0850; 969.2660; 976.3194; 977.2359; 978.8631; 987.0771; 998.1100; 1016.8784; 1032.5923; 1052.7835; 1059.2066; 1061.6017; 1070.3902; 1094.7172; 1106.2156; 1109.4261; 1120.4768; 1134.9627; 1142.5140; 1187.6608; 1206.8094; 1212.4896; 1213.5898; 1215.0284; 1222.2527; 1227.2012; 1228.8996; 1233.8020; 1244.6151; 1245.6735; 1248.0605; 1250.2847; 1254.0756; 1279.7278; 1288.6745; 1292.7530; 1306.5759; 1315.4067; 1345.9439; 1350.5465; 1362.7615; 1425.6148; 1429.4779; 1434.7444; 1460.0234; 1492.4123; 1500.4031; 1504.3109; 1508.7612; 1518.6826; 1522.7105; 1528.9145; 1531.6909; 1539.6380; 1552.8693; 1639.1458; 1648.0633; 1659.7218; 1755.0695; 3042.6310; 3055.6046; 3058.1998; 3065.9584; 3094.8103; 3119.6381; 3128.5012; 3135.7686; 3141.7604; 3143.8395; 3150.9149; 3163.6071; 3166.4791; 3180.1796; 3192.7906; 3199.9106; 3246.6681.

Cartesian Forces: Max 0.000000478 RMS 0.000000179

Item	Value	Threshold	Converged?
Maximum Force	0.000000	0.000450	YES
RMS Force	0.000000	0.000300	YES
Maximum Displacement	0.000080	0.001800	YES
RMS Displacement	0.000012	0.001200	YES

FILE: Yb_Sq2_PhYbOTf3_Prod_Anion_B3LYP_M062x_Add56_Conf3-1_a.out

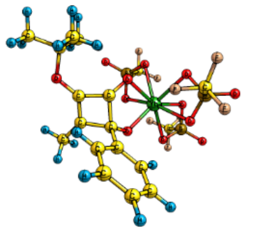

Conformer 7

E(RB3LYP/6-31G(d)/MWB59) = -3731.52997215
Convg = 0.7316D-08 -V/T = 2.0122

Temperature 195.150 Kelvin. Pressure 1.00000 Atm.
elec. and zero-point Energies= -3731.156067
elec. and thermal Energies= -3731.133203
elec. and thermal Enthalpies= -3731.132585
elec. and thermal Free Energies= -3731.201853

RB3LYP/6-31G(d)/MWB59/THF(IEFPCM) = -3731.59401571

RM06-2x/6-311+G(d,p)/MWB59/THF(IEFPCM) = -3731.43036471

Geometry at RB3LYP/6-31G(d)/MWB59
Charge = -1 Multiplicity = 1
Yb -0.1200223348 -0.4703857213 3.7574702258
O 1.411577026 1.4528883172 3.8750855669
S 0.379772312 2.5133700499 3.6182183729
O 0.35147687 3.6752121839 4.4971833519
O -0.9185121946 1.7778691474 3.3831436669
C 0.8121650876 3.2032202365 1.9416037815
F 2.0346991692 3.7491638299 1.9803103797
F 0.7991443622 2.2468234633 1.0066898539
F -0.0719039178 4.1485307921 1.6006845204
O -2.3033789899 -0.6292724782 4.8607156525
O -2.6480348078 0.9377887978 6.8303906135
O -0.3778183448 0.0505326149 6.1252389919
S -1.8728651498 -0.1311995676 6.2123646073
C -2.1094635349 -1.587941272 7.3539466936
F -3.4205540062 -1.8034356033 7.5295966148
F -1.5583937762 -2.6984117704 6.8580120359
F -1.5536181587 -1.3228159078 8.5431655788
C 0.6096617815 -2.3966901143 -0.0100013733
C -0.7288701976 -2.5864405561 0.205899752
C -0.976370184 -1.1992472004 0.8601657042
C 0.5646774237 -1.2076996526 0.8453838127
O 1.2965425601 -0.6798310746 1.6958372652
O -1.391227557 -1.1726191225 2.1605828839
O 1.5047820439 -3.1824656436 -0.6200363647
C 2.9488613666 -2.9077637621 -0.5413090774
C -1.6376115745 -3.7538159725 0.0899149894
H -1.1186555391 -4.639494399 -0.2892669934
H -2.4800388199 -3.518972256 -0.5748016257
H -2.0615002995 -3.9806560343 1.0758751932
C 3.2467194911 -1.5061791334 -1.0862728927
H 4.3309923409 -1.3650614168 -1.1602235634
H 2.815585278 -1.3796963387 -2.0858814337
H 2.8594726158 -0.7250994737 -0.426753795
C 3.5363153334 -3.9846605746 -1.4574099582
H 4.6278590187 -3.9012300004 -1.486059859
H 3.273895963 -4.9818469378 -1.0895205992
H 3.1506655053 -3.8792334811 -2.4773374818
C 3.4352072331 -3.0804181338 0.8989360776
H 3.034032336 -2.3057250468 1.556004847
H 3.1391824164 -4.0550155629 1.2973335223
H 4.5293338058 -3.0135324325 0.9289916843
C -1.7066539978 -0.2020373679 -0.0432134112
C -1.4734424708 -0.1338932659 -1.4251744118
C -2.6439777796 0.6691710943 0.5247616989
H -0.7538157522 -0.8047753154 -1.8865831997
H -2.8194767906 0.6143612712 1.5926107248
C -2.1574178973 0.7872463275 -2.2194270835
C -3.3303560712 1.5886376567 -0.2711500362
H -1.9607306791 0.8270236794 -3.288697559
H -4.0521255851 2.2592441613 0.1888700858
C -3.091095638 1.6528693755 -1.644880309
H -3.6250096401 2.3711204448 -2.2629212746
O 2.371049944 -3.8919611069 4.0315449918
S 1.5983258141 -2.8253293024 4.6658407499
C 1.751376125 -3.175758757 6.4920331976
O 0.1162908713 -2.8055927697 4.3865607671
O 2.0743506532 -1.4142035149 4.4911958572
F 1.2116812639 -2.2035843592 7.2257774614
F 3.0486178728 -3.285935628 6.8090756816
F 1.1380961181 -4.331557275 6.7777892554

Frequency: 13.0010; 16.9892; 22.3543; 23.7538; 28.0847; 30.6431; 31.6728; 32.4772; 38.4647; 38.9558; 44.1047; 48.2415; 54.4854; 60.0808; 62.5942; 67.9190; 70.0048; 77.9383; 82.2393; 85.0156; 92.1170; 101.8269; 125.9481; 126.7499; 132.4685; 136.9336; 143.2252; 145.7335; 150.7270; 161.2159; 169.7392; 182.8794; 198.0902; 208.3945; 211.3212; 214.5421; 215.5970; 217.0623; 219.1557; 221.7649; 255.0479; 262.2518; 266.5722; 273.2316; 283.6067; 299.1122; 302.8285; 306.4697; 308.4835; 321.3197; 322.1774; 324.6061; 341.1148; 350.9220; 352.2448; 357.1673; 362.5034; 385.2022; 414.5833; 422.9560; 451.0164; 452.3353; 482.7765; 489.8511; 490.7966; 491.8403; 524.4757; 524.9377; 529.5256; 534.0161; 547.1662; 557.1679; 558.2068; 558.7902; 570.4667; 571.8069; 577.6795; 588.9556; 619.9650; 625.8127; 632.9447; 633.2581; 639.1657; 671.7967; 709.0797; 714.8512; 745.7041; 760.2348; 760.6238; 762.0880; 762.2419; 796.6715; 863.4259; 866.1561; 916.2561; 931.8697; 935.2446; 943.4186; 963.1675; 970.4294; 974.1169; 977.3087; 979.4017; 987.0521; 1002.1512; 1016.6274; 1030.2448; 1054.8684; 1060.1858; 1064.5510; 1068.9188; 1082.2545; 1106.8894; 1109.3405; 1115.8998; 1133.7436; 1154.9214; 1187.9091; 1208.2195; 1211.1715; 1213.9613; 1216.4380; 1218.1842; 1226.7920; 1229.5621; 1234.2984; 1235.7733; 1246.2110; 1247.6006; 1254.2599; 1256.8663; 1281.1513; 1294.0611; 1297.2182; 1306.8170; 1319.7586; 1346.3440; 1362.7462; 1367.1471; 1426.7079; 1429.0211; 1432.4114; 1458.4642; 1492.2431; 1501.6673; 1503.4941; 1507.4837; 1515.7822; 1517.8657; 1533.9627; 1535.3493; 1539.6001; 1550.8576; 1639.2600; 1648.8803; 1659.6898; 1750.1463; 3038.1223; 3053.5187; 3056.7627; 3064.9214; 3096.0382; 3118.0201; 3128.0327; 3129.9706; 3131.7703; 3136.5254; 3149.9821; 3166.8567; 3167.6641; 3180.8140; 3193.2222; 3200.1229; 3246.1693.

Cartesian Forces: Max 0.000001502 RMS 0.000000402

Item	Value	Threshold	Converged?
Maximum Force	0.000001	0.000450	YES
RMS Force	0.000000	0.000300	YES
Maximum Displacement	0.000119	0.001800	YES
RMS Displacement	0.000023	0.001200	YES

FILE: Yb_Sq2_PhYbOTf3_Prod_Anion_B3LYP_M062x_Add56_Conf4.out

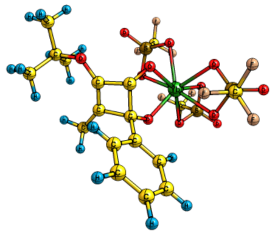

Conformer 8

E(RB3LYP/6-31G(d)/MWB59) = -3731.52261916
Convg = 0.8734D-08 -V/T = 2.0122

Temperature 195.150 Kelvin. Pressure 1.00000 Atm.
elec. and zero-point Energies= -3731.148663
elec. and thermal Energies= -3731.125835
elec. and thermal Enthalpies= -3731.125217
elec. and thermal Free Energies= -3731.194367

RB3LYP/6-31G(d)/MWB59/THF(IEFPCM) = -3731.58849346

RM06-2x/6-311+G(d,p)/MWB59/THF(IEFPCM) = -3731.42521689

Geometry at RB3LYP/6-31G(d)/MWB59
Charge = -1 Multiplicity = 1
Yb -0.0461857588 -0.4623375727 3.58312527
O 1.5324130321 1.4012565057 3.6632127388
S 0.520626705 2.5108684533 3.5968324803
O 0.6539571255 3.620177824 4.5321786597
O -0.8244145098 1.8343430131 3.4926133672
C 0.7704240439 3.2694993763 1.9136173224
F 1.9974650551 3.798479203 1.8323624219
F 0.6325153865 2.3510942854 0.9513254525
F -0.1316697508 4.2388360054 1.7159898599
O -2.1375270191 -0.6765305512 4.8554557021
O -2.2337764733 0.6359317849 7.0301640561
O -0.0785821793 -0.2256236276 6.0064554046
S -1.5665088353 -0.36863554 6.2108497673
C -1.7649648789 -1.9554163591 7.1724914482
F -3.0572171988 -2.1241941142 7.4860883695
F -1.3594447909 -3.0122300766 6.4628272444
F -1.0520856908 -1.8935200695 8.3040363595
C 0.2501311918 -2.4578677327 -0.096162079
C -1.1013313973 -2.4872934727 0.1469775847
C -1.1608185881 -1.0408991071 0.7453676903
C 0.3597388134 -1.2173569077 0.670788863
O 1.1919761873 -0.7452119016 1.4550425835
O -1.5107586933 -0.931007703 2.0615360478
O 1.2728266947 -3.1666987283 -0.5872507468
C 1.2548578731 -4.6128756062 -0.8079080326
C -2.2461632571 -3.4351303716 0.1724328235
H -2.024380903 -4.4277149082 -0.2255321099
H -3.0857105897 -3.0078039369 -0.3922766578
H -2.5860433388 -3.541153563 1.210595126
C 0.8310525179 -5.3446455595 0.4692012892
H 0.9523417545 -6.4252892724 0.3287160654
H 1.4510101621 -5.0267616424 1.3127755808
H -0.2135665374 -5.1577841666 0.7290875266
C 2.7229452741 -4.9148307456 -1.1265962649
H 2.8535905811 -5.9807639152 -1.3436352033
H 3.0524289751 -4.3373727452 -1.9968317826
H 3.3546116472 -4.6483906791 -0.2742405563
C 0.3674232269 -4.9358913737 -2.0164911283
H -0.6769326397 -4.6635226455 -1.8429041783
H 0.7189520278 -4.393368649 -2.9008304785
H 0.4033022191 -6.0096119836 -2.2343995135
C -1.8388944725 -0.016490985 -0.1636568258
C -1.6897153163 -0.0383724186 -1.558523229
C -2.636414537 0.979342319 0.4133680851
H -1.0748042956 -0.8040043932 -2.0239750717
H -2.7447631505 0.9916381644 1.4918127647
C -2.3219720056 0.9147684203 -2.3579798313
C -3.270646161 1.9313346184 -0.387425384
H -2.1923477768 0.8838158103 -3.4377077207
H -3.8842933432 2.6988121668 0.0782851608
C -3.1173442303 1.9040333124 -1.7748046659
H -3.6104733568 2.6474840802 -2.3969475576
O 2.4623492256 -3.852305647 3.2524451924
S 1.7166553833 -2.908629516 4.0869010701
C 1.915919548 -3.5853758083 5.8142764479
O 0.2242166086 -2.8528612035 3.8560522951
O 2.1897435716 -1.492827745 4.1589836799
F 1.413550769 -2.7564502381 6.7287930437
F 3.2193927973 -3.7655416626 6.0668071601
F 1.2923106884 -4.7679150373 5.9070749515

Frequency: 12.7496; 19.0169; 22.0902; 24.5828; 26.7621; 29.8530; 31.3378; 34.0558; 34.6928; 37.8116; 44.2065; 50.4304; 56.0829; 60.3116; 65.3352; 67.7363; 72.5753; 78.2081; 80.7267; 84.0920; 94.0379; 100.9219; 121.0350; 128.5388; 132.1277; 142.5841; 145.3967; 148.8623; 168.8603; 171.8969; 172.7308; 187.5425; 192.1912; 210.1377; 211.2753; 214.5471; 215.8807; 217.9532; 221.9436; 227.2281; 239.9738; 251.9779; 264.5566; 267.0326; 277.7940; 291.5318; 302.7202; 305.6382; 308.0016; 321.6081; 321.8432; 324.7317; 347.9727; 350.5653; 352.5862; 357.3622; 367.0916; 381.7895; 413.4450; 422.1112; 456.6591; 463.8225; 475.8784; 489.5630; 490.7921; 491.1972; 522.1532; 523.8585; 524.7791; 530.7078; 538.8649; 557.0204; 558.1385; 558.7255; 570.1514; 571.4224; 576.7476; 582.8357; 619.6266; 625.1871; 633.3270; 638.4695; 648.0901; 685.3464; 712.1410; 730.7923; 743.0845; 759.9662; 760.6523; 761.4758; 770.7081; 794.6412; 840.1774; 864.5351; 914.2441; 929.9903; 932.7742; 954.2266; 962.6277; 971.1665; 974.2817; 976.7677; 979.6962; 986.2719; 1002.2710; 1016.6851; 1032.0016; 1053.0922; 1059.2613; 1061.7481; 1070.6073; 1094.8825; 1106.1373; 1110.6206; 1117.5211; 1134.2858; 1141.7820; 1187.7350; 1206.6477; 1212.0997; 1214.4853; 1216.3869; 1221.3780; 1225.4022; 1228.8077; 1234.8066; 1236.6597; 1245.1973; 1247.8021; 1248.2664; 1253.2151; 1280.0493; 1289.2905; 1292.8140; 1307.1911; 1319.6672; 1345.8942; 1350.3091; 1362.7091; 1425.8694; 1429.8951; 1434.8416; 1459.9920; 1492.3970; 1500.3435; 1504.0237; 1508.9349; 1518.7982; 1522.9402; 1529.0288; 1531.8332; 1539.5465; 1553.2444; 1638.9371; 1650.3036; 1659.5250; 1758.5989; 3041.6730; 3055.3920; 3058.0378; 3065.7518; 3093.9300; 3119.4065; 3127.9690; 3134.9040; 3141.5443; 3144.3275; 3150.9311; 3162.3721; 3166.9000; 3180.4496; 3192.9320; 3200.0628; 3242.2888.

Cartesian Forces: Max 0.000001704 RMS 0.000000614

Item	Value	Threshold	Converged?
Maximum Force	0.000000	0.000450	YES
RMS Force	0.000000	0.000300	YES
Maximum Displacement	0.000043	0.001800	YES
RMS Displacement	0.000005	0.001200	YES

FILE: Yb_Sq2_PhYbOTf3_Prod_Anion_B3LYP_M062x_Add56_Conf4-1.out

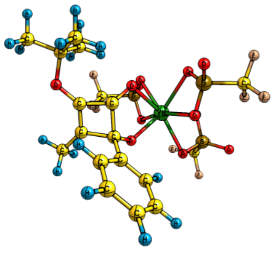

Conformer 9

E(RB3LYP/6-31G(d)/MWB59) = -3731.53086848
Convg = 0.8298D-09 -V/T = 2.0122

Temperature 195.150 Kelvin. Pressure 1.00000 Atm.
elec. and zero-point Energies= -3731.156854
elec. and thermal Energies= -3731.133980
elec. and thermal Enthalpies= -3731.133362
elec. and thermal Free Energies= -3731.202664

RB3LYP/6-31G(d)/MWB59/THF(IEFPCM) = -3731.59440302

RM06-2x/6-311+G(d,p)/MWB59/THF(IEFPCM) = -3731.43057713

Geometry at RB3LYP/6-31G(d)/MWB59
Charge = -1 Multiplicity = 1
Yb -0.0003674124 -0.4926254687 3.7507674044
O -0.524140954 1.7182947792 3.0132388001
S 0.8378407849 2.3551303596 3.1949202599
O 1.4013398583 3.05775981 2.046898584
O 1.6861812077 1.3262244413 3.8838671511
C 0.5550252024 3.6913775721 4.4635841728
F -0.2880659344 4.6052727765 3.9655086186
F 0.0461481773 3.1965470651 5.5885879214
F 1.7237038447 4.2885289432 4.7389005973
O -2.2063842589 -0.2583458692 4.7495573129
O -2.394880405 1.5161970158 6.5530500408
O -0.2685249018 0.2134019296 6.0908891889
S -1.7737470457 0.280309135 6.0887843865
C -2.3314129414 -0.9982332244 7.327472497
F -3.6710861539 -0.9853559922 7.3933995902
F -1.9302611082 -2.2228280491 6.9975134893
F -1.8386953281 -0.6867413772 8.5337649767
C 0.8174529661 -2.2342140604 -0.1324690165
C -0.4265033403 -2.7075750085 0.1811355968
C -0.8902179456 -1.4294665786 0.9280765522
C 0.604786462 -1.0843781388 0.7563417718
O 1.2895280581 -0.4041290588 1.5267837646
O -1.1447341209 -1.5417079251 2.2702716208
O 1.800411603 -2.8009343975 -0.845871761
C 3.1923579469 -2.3392261685 -0.7280587583
C -1.0807956908 -4.0344357445 0.0665022787
H -0.4377775614 -4.7620035023 -0.4386488722
H -2.0284865598 -3.9512942866 -0.4819776577
H -1.3219746116 -4.4074558411 1.0694274828
C 3.3008427404 -0.8724053229 -1.1593241464
H 4.3566677469 -0.5830987967 -1.2126437323
H 2.8565233896 -0.729767275 -2.1508639077
H 2.8130518333 -0.2037875833 -0.4457421149
C 3.9247167642 -3.2536768531 -1.7137951052
H 4.9940206652 -3.0176971309 -1.7250480841
H 3.8029937319 -4.3029984657 -1.4253725491
H 3.5284106013 -3.1255237872 -2.7268923838
C 3.6933781675 -2.5557901817 0.7030657648
H 3.1929411083 -1.8889877784 1.4096712163
H 3.5283084632 -3.5910136457 1.0173659023
H 4.7689349423 -2.3491600642 0.7522061844
C -1.9248635574 -0.5959095798 0.1708662254
C -1.8668282418 -0.4270699783 -1.2208861647
C -2.9679397995 0.0120604645 0.8798122894
H -1.0668672172 -0.8936421896 -1.7894699813
H -3.0124167241 -0.1179250916 1.9550849198
C -2.8262579456 0.3355072924 -1.8875577995
C -3.9297214663 0.7722124547 0.2112345856
H -2.7615620217 0.4587600916 -2.9664909773
H -4.7300622177 1.2399058686 0.7795090532
C -3.864304709 0.9379419392 -1.1730638507
H -4.612618607 1.5331614619 -1.6913570507
O 2.1708656481 -3.2799016036 6.1304483266
S 1.4792989592 -2.8940899769 4.906430077
C 1.7208171857 -4.3424007115 3.7588412881
O 2.0362234514 -1.7248679736 4.1313370753
O -0.0040335239 -2.6945620203 4.9573766037
F 1.1390507415 -4.120138871 2.5705872322
F 1.1866751075 -5.4452553189 4.2952595612
F 3.0293659461 -4.5518895089 3.5543965486

Frequency: 15.7836; 18.3045; 21.2893; 22.7045; 27.1384; 29.1325; 34.0962; 36.4386; 38.8261; 40.2603; 43.8575; 47.3920; 50.8112; 53.7765; 55.4100; 64.4745; 66.3197; 73.7040; 84.0814; 87.2320; 90.7872; 105.5350; 117.6670; 128.1567; 134.1959; 140.5944; 140.9835; 145.7336; 149.6267; 164.5015; 174.8858; 176.7510; 198.3505; 205.3217; 212.0227; 213.6339; 217.5621; 218.8061; 219.8401; 228.0459; 255.6379; 261.3087; 270.0104; 277.9181; 284.5841; 299.6235; 303.7906; 307.5326; 309.6695; 321.5722; 323.4294; 324.6148; 343.4815; 351.2520; 353.0101; 357.2595; 365.4087; 385.9673; 413.8630; 422.5547; 449.3133; 453.0829; 484.6366; 489.1277; 490.5388; 490.9954; 524.4791; 524.8875; 528.8957; 537.4521; 545.2689; 557.4063; 558.4207; 558.6600; 570.5308; 571.2052; 573.8693; 588.0255; 624.4486; 627.1917; 633.1816; 633.4914; 636.4060; 668.0944; 709.3095; 715.7750; 750.2269; 758.3389; 762.2531; 762.6178; 764.2878; 797.1482; 863.8524; 865.9959; 918.8510; 932.5782; 935.6390; 944.4855; 965.5368; 970.7329; 971.4409; 976.3155; 981.4523; 984.7960; 1006.2917; 1016.3694; 1029.8928; 1057.5703; 1060.6410; 1065.0140; 1070.4478; 1083.6641; 1101.7319; 1107.7704; 1119.1633; 1130.3918; 1153.5532; 1188.2262; 1209.1444; 1209.6175; 1211.4273; 1212.9150; 1216.3540; 1224.2288; 1229.5955; 1231.7171; 1235.5777; 1243.7804; 1256.5176; 1257.5723; 1259.3562; 1281.3421; 1296.2699; 1303.4355; 1305.5815; 1317.2018; 1346.7739; 1363.3564; 1365.4239; 1427.4836; 1429.8737; 1433.7525; 1458.7067; 1492.5011; 1502.8425; 1504.1123; 1507.4136; 1517.5664; 1519.3070; 1534.5369; 1538.0676; 1540.5738; 1554.6645; 1639.6748; 1657.7438; 1662.8431; 1761.2124; 3039.9433; 3053.4942; 3056.5496; 3064.3128; 3097.3816; 3117.6117; 3126.1299; 3128.2260; 3129.4587; 3133.9430; 3155.5185; 3161.3268; 3167.3064; 3181.0621; 3193.3026; 3200.3191; 3239.1115.

Cartesian Forces: Max 0.000000523 RMS 0.000000205

Item	Value	Threshold	Converged?
Maximum Force	0.000000	0.000450	YES
RMS Force	0.000000	0.000300	YES
Maximum Displacement	0.000032	0.001800	YES
RMS Displacement	0.000005	0.001200	YES

FILE: Yb_Sq2_PhYbOTf3_Prod_Anion_B3LYP_M062x_Add56_Conf5_a.out

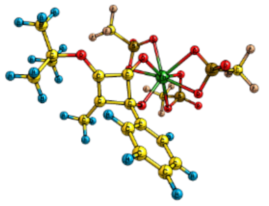

Conformer 10

E(RB3LYP/6-31G(d)/MWB59) = -3731.52138052
Convg = 0.5187D-09 -V/T = 2.0122

Temperature 195.150 Kelvin. Pressure 1.00000 Atm.
elec. and zero-point Energies= -3731.147470
elec. and thermal Energies= -3731.124588
elec. and thermal Enthalpies= -3731.123970
elec. and thermal Free Energies= -3731.193642

RB3LYP/6-31G(d)/MWB59/THF(IEFPCM) = -3731.58868014

RM06-2x/6-311+G(d,p)/MWB59/THF(IEFPCM) = -3731.42305662

Geometry at RB3LYP/6-31G(d)/MWB59
Charge = -1 Multiplicity = 1
Yb -0.1116109066 -0.4464531472 3.6171540613
O -0.9555561029 1.7685670384 3.1736103875
S 0.3540744372 2.5221278927 3.2204077699
O 0.6511380504 3.410867283 2.1022642204
O 1.3913964672 1.5105984196 3.6137118401
C 0.1869833002 3.6501236472 4.6940644913
F -0.7924573275 4.535659401 4.4677257094
F -0.0951395971 2.9623479615 5.7983754331
F 1.3358565785 4.3157645672 4.8752148904
O -2.2201898987 -0.6171182331 4.8588660843
O -2.3888446452 0.8018701971 6.9575452455
O -0.1957339924 -0.0744532488 6.0273641483
S -1.6839816489 -0.234309456 6.210610701
C -1.8660320941 -1.7715620598 7.2475063715
F -3.1688034202 -2.036966543 7.423196369
F -1.2885204044 -2.8198874445 6.6608894106
F -1.3020903233 -1.5751906819 8.4454765656
C 0.3383891075 -2.4704717403 -0.0688160518
C -0.9988194064 -2.640634949 0.189455886
C -1.1915263597 -1.2197566688 0.819176235
C 0.3370574213 -1.2348008194 0.7177649599
O 1.1287274329 -0.6772497833 1.4876063393
O -1.527684889 -1.1699880302 2.14134647
O 1.4225243228 -3.0661733621 -0.5838818607
C 1.456144653 -4.4307843995 -1.0981455045
C -2.0554630017 -3.6864011717 0.1916316415
H -1.7623064815 -4.6369241308 -0.259506808
H -2.9422240889 -3.3069824062 -0.333573927
H -2.3598024648 -3.8696878857 1.2300818803
C 1.1257763277 -5.4318347645 0.0151641149
H 1.2567872382 -6.4550317072 -0.3557728139
H 1.7895216771 -5.2831719049 0.8699726121
H 0.0978629622 -5.333223691 0.3701135047
C 2.9198495492 -4.5843179022 -1.5257457656
H 3.0973504787 -5.5862325834 -1.9315877512
H 3.1749070776 -3.8446715974 -2.2915389296
H 3.5787182364 -4.4293473655 -0.666016395
C 0.5271682193 -4.5575601908 -2.3118928117
H -0.5211526237 -4.3987609985 -2.046610345
H 0.8015684196 -3.8224355036 -3.0759924134
H 0.6157206666 -5.5584250647 -2.7498042146
C -1.9887359283 -0.2610433821 -0.066431598
C -1.8425134911 -0.2349732178 -1.4617251014
C -2.90176809 0.6152546223 0.5318094109
H -1.1382031724 -0.9071850002 -1.9445859355
H -3.0041700327 0.595264186 1.6101853365
C -2.5910631241 0.6480448194 -2.2407577629
C -3.6529026489 1.49623188 -0.2486721198
H -2.4606728963 0.6572746474 -3.3208007888
H -4.3541392101 2.1728073385 0.2337975566
C -3.5023392602 1.5170520232 -1.6362276153
H -4.0856471286 2.2067382051 -2.2420490298
O 1.9868541505 -3.3232363156 5.8908427943
S 1.7178092773 -2.6802723207 4.6092958829
C 2.7699526619 -3.6334920268 3.4010258587
O 2.1192702007 -1.2501891375 4.4276483601
O 0.3183861195 -2.8011599228 4.0528741781
F 2.6517714312 -3.1579373073 2.1563116742
F 2.3951492652 -4.9256264131 3.3939903985
F 4.0552216917 -3.5649692898 3.7662408277

Frequency: 7.7014; 17.2683; 20.6768; 24.9386; 26.9556; 31.4856; 32.5068; 34.7264; 36.5548; 38.2144; 43.8099; 45.2767; 52.0148; 57.7728; 60.7413; 66.4532; 74.4527; 77.2833; 80.5025; 85.2294; 94.6083; 102.0770; 121.8941; 127.4929; 133.7728; 140.4037; 144.9706; 148.3205; 166.7855; 170.6967; 177.3993; 183.5306; 187.1190; 209.6716; 211.4301; 211.8265; 214.3034; 217.1635; 218.3836; 230.6402; 236.6980; 255.3869; 262.2965; 265.8725; 280.9833; 293.4017; 303.6090; 306.0029; 308.0459; 321.8015; 322.4559; 323.7826; 345.4878; 351.2835; 352.8987; 357.3805; 367.9610; 382.2805; 414.1252; 421.9780; 456.2912; 461.4076; 477.0370; 489.5568; 489.8186; 491.5455; 516.7214; 524.1383; 525.8081; 530.3153; 539.2132; 557.8246; 557.9090; 558.5407; 570.2336; 573.5700; 574.0920; 581.4561; 625.7909; 627.4800; 633.2682; 634.4813; 643.1718; 682.7620; 711.2563; 726.9850; 744.0122; 759.1611; 762.0364; 762.6649; 769.4730; 796.3988; 841.3199; 861.7227; 913.6049; 929.1398; 929.8775; 954.6225; 964.9032; 968.9494; 975.0624; 976.3616; 977.5540; 983.3946; 999.3360; 1017.0227; 1032.7291; 1052.9812; 1058.5016; 1060.7866; 1069.1399; 1094.1978; 1106.3440; 1109.5531; 1114.8953; 1134.6569; 1141.2801; 1187.8334; 1204.8967; 1207.2012; 1212.6005; 1213.4668; 1223.8055; 1227.0004; 1231.9338; 1233.3816; 1235.6778; 1246.5437; 1248.3410; 1250.6332; 1255.0539; 1279.3328; 1290.7822; 1305.1390; 1306.2122; 1315.3791; 1345.5274; 1347.2736; 1362.9631; 1426.1839; 1429.3691; 1431.7717; 1457.0481; 1492.4592; 1500.1966; 1503.9263; 1509.3349; 1518.8744; 1521.5764; 1527.9459; 1530.5852; 1539.6482; 1552.2870; 1639.3736; 1652.4546; 1659.7798; 1759.1894; 3041.9735; 3057.0676; 3061.1169; 3072.2216; 3093.9958; 3122.0765; 3131.6664; 3139.3794; 3140.8120; 3146.9819; 3152.6640; 3166.6052; 3174.3471; 3180.4165; 3192.4550; 3199.6623; 3248.3119.

Cartesian Forces: Max 0.000000417 RMS 0.000000160

Item	Value	Threshold	Converged?
Maximum Force	0.000000	0.000450	YES
RMS Force	0.000000	0.000300	YES
Maximum Displacement	0.000157	0.001800	YES
RMS Displacement	0.000030	0.001200	YES

FILE: Yb_Sq2_PhYbOTf3_Prod_Anion_B3LYP_M062x_Add56_Conf5-1.out

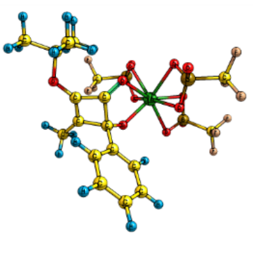

Conformer 11

E(RB3LYP/6-31G(d)/MWB59) = -3731.53101364
Convg = 0.8222D-08 -V/T = 2.0122

Temperature 195.150 Kelvin. Pressure 1.00000 Atm.
elec. and zero-point Energies= -3731.156902
elec. and thermal Energies= -3731.134153
elec. and thermal Enthalpies= -3731.133535
elec. and thermal Free Energies= -3731.202000

RB3LYP/6-31G(d)/MWB59/THF(IEFPCM) = -3731.59507581

RM06-2x/6-311+G(d,p)/MWB59/THF(IEFPCM) = -3731.43200465

Geometry at RB3LYP/6-31G(d)/MWB59
Charge = -1 Multiplicity = 1
Yb -0.1445864733 -0.4748251658 3.6888154868
O -0.6548874217 1.744743437 2.9807405065
S 0.7048260117 2.3798970108 3.1846225477
O 1.2713424206 3.1019383122 2.0505265583
O 1.5495934044 1.3461227167 3.8673790132
C 0.4191119481 3.7055683954 4.4679530723
F -0.5375437288 4.5410995043 4.0434681163
F 0.0560603629 3.1904288092 5.6425867308
F 1.5535551148 4.3991449455 4.6369135008
O -0.4947573014 0.2914556207 5.9901387451
O -2.4520781652 -0.7770567053 7.2035206313
O -2.3652101489 -0.464405031 4.6823602176
S -1.9696219816 -0.0084679176 6.0627061697
C -2.7914497786 1.6569042869 6.2523584308
F -2.2983410778 2.2847246887 7.3270813049
F -2.6023957309 2.4251679764 5.1767227801
F -4.1094008096 1.4806839999 6.4229282886
C 0.8106304399 -2.2054838237 -0.1660014474
C -0.4456884768 -2.6753213375 0.1000000651
C -0.9333350943 -1.3950851545 0.8263054986
C 0.5661097059 -1.0472632123 0.7052247164
O 1.2205099489 -0.3537670168 1.488642182
O -1.2365528752 -1.5036957538 2.1588936491
O 1.8177249635 -2.7757322513 -0.8424265295
C 3.2096717938 -2.341279079 -0.6462903526
C -1.1016190801 -3.9983765582 -0.0443946533
H -0.4394761346 -4.7301143975 -0.5178174117
H -2.0211997079 -3.9101445772 -0.6381837057
H -1.3932490325 -4.3690418996 0.9458161683
C 3.3684418253 -0.8751611436 -1.0641844921
H 4.4305339775 -0.604433132 -1.0566261545
H 2.9833507246 -0.7212786558 -2.0785929569
H 2.8525240913 -0.2011757226 -0.3758845141
C 3.9776965314 -3.2657360151 -1.5948205876
H 5.0500198557 -3.0485257861 -1.5480225482
H 3.8225129473 -4.3135704362 -1.3172915388
H 3.6384791548 -3.1277352325 -2.6271875741
C 3.6271833751 -2.5724795595 0.8089997353
H 3.1030401994 -1.8988161556 1.4914439366
H 3.4249431192 -3.605309497 1.1095220366
H 4.7023697037 -2.3878213016 0.9177545972
C -1.9416160658 -0.5645565522 0.0315746724
C -1.8164708293 -0.3706016931 -1.352431082
C -3.0296811227 0.0132791526 0.6964872235
H -0.9810445422 -0.8143006852 -1.8873465982
H -3.1243940521 -0.1388989319 1.7656504851
C -2.7540291446 0.3879174435 -2.053860355
C -3.9703189467 0.7682996817 -0.0070417617
H -2.6369132325 0.5317560063 -3.1257744829
H -4.807688714 1.2112141278 0.5268485253
C -3.8375954892 0.9599061094 -1.3831747041
H -4.5692305635 1.5513067912 -1.9288886772
O 2.0259783798 -3.2289233666 6.1064060981
S 1.3045656716 -2.8675547974 4.8929215168
C 1.4840871094 -4.3544538375 3.7833855588
O 1.8643981981 -1.735118626 4.0634588977
O -0.1715579358 -2.6297696503 4.9782602519
F 0.8954221006 -4.1464388223 2.5963336051
F 0.9238517942 -5.4243846926 4.3570374283
F 2.783522589 -4.6098024648 3.5695396486

Frequency: 17.8633; 18.9098; 24.1279; 25.7163; 27.2471; 33.1413; 35.0117; 37.0251; 37.2515; 46.2916; 47.9017; 49.8274; 53.5769; 55.7620; 65.0723; 67.3772; 72.2979; 79.0648; 82.9300; 90.1052; 91.9193; 105.4207; 123.2843; 126.7069; 132.0593; 138.3951; 141.1103; 146.0786; 150.3077; 165.2032; 174.0643; 179.5166; 198.1215; 204.1948; 212.6242; 213.6799; 218.7583; 220.1335; 224.5737; 230.5780; 254.1620; 262.2159; 270.2947; 277.7865; 283.8833; 298.6065; 303.1124; 306.6596; 309.4289; 321.4917; 323.2483; 326.2785; 343.5323; 351.5623; 353.2192; 357.2366; 365.3310; 385.7931; 413.4356; 422.1426; 449.2887; 453.0921; 485.4845; 489.9137; 490.4734; 491.3337; 524.0386; 524.6029; 529.6053; 538.1241; 544.5510; 557.3065; 558.3811; 559.0504; 569.7994; 570.8827; 576.1183; 588.5574; 620.1269; 624.6438; 633.3604; 634.8978; 638.4040; 666.9146; 709.5086; 715.7225; 751.5096; 758.3173; 759.9463; 761.0585; 764.4877; 797.1976; 863.4557; 865.5810; 918.5520; 931.9827; 935.4620; 944.5812; 965.9935; 969.1670; 970.7970; 974.7733; 981.3060; 984.3226; 1004.1511; 1016.5358; 1029.8152; 1058.0538; 1060.4330; 1064.8346; 1070.4057; 1083.5847; 1105.3715; 1106.9218; 1117.1475; 1128.2530; 1154.0525; 1188.0614; 1208.3678; 1210.7781; 1212.1957; 1213.0671; 1215.9589; 1224.1947; 1225.6417; 1229.2565; 1237.1472; 1243.6558; 1247.5537; 1253.4315; 1257.5960; 1281.0475; 1296.1563; 1302.9324; 1309.4751; 1319.8806; 1346.7440; 1362.9602; 1364.8937; 1427.5497; 1430.0856; 1433.6466; 1458.6416; 1492.6407; 1502.9481; 1504.0662; 1507.5897; 1517.6851; 1519.1306; 1534.6347; 1538.4359; 1540.3530; 1554.9374; 1639.7126; 1658.3732; 1663.8055; 1763.4398; 3039.9724; 3053.1903; 3056.3086; 3064.2500; 3097.8917; 3117.0303; 3125.8180; 3128.0312; 3129.8596; 3133.7645; 3155.5951; 3161.9340; 3167.5362; 3180.6532; 3192.8846; 3200.2734; 3237.5980.

Cartesian Forces: Max 0.000000957 RMS 0.000000425

Item	Value	Threshold	Converged?
Maximum Force	0.000000	0.000450	YES
RMS Force	0.000000	0.000300	YES
Maximum Displacement	0.000006	0.001800	YES
RMS Displacement	0.000001	0.001200	YES

FILE:
Yb_Sq2_PhYbOTf3_Prod_Anion_B3LYP_M062x_Add56_Conf6.out

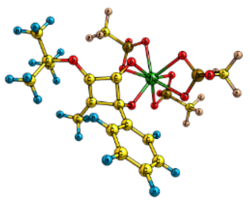

Conformer 12

E(RB3LYP/6-31G(d)/MWB59) = -3731.52067862
Convg = 0.7140D-08 -V/T = 2.0122

Temperature 195.150 Kelvin. Pressure 1.00000 Atm.
elec. and zero-point Energies= -3731.146632
elec. and thermal Energies= -3731.123867
elec. and thermal Enthalpies= -3731.123249
elec. and thermal Free Energies= -3731.192019

RB3LYP/6-31G(d)/MWB59/THF(IEFPCM) = -3731.58853784

RM06-2x/6-311+G(d,p)/MWB59/THF(IEFPCM) = -3731.42336536

Geometry at RB3LYP/6-31G(d)/MWB59
Charge = -1 Multiplicity = 1
Yb -0.2145192089 -0.4555418521 3.5367281213
O -1.1219771257 1.7571162726 3.1824906031
S 0.1714093133 2.5371863033 3.2108810585
O 0.4087438697 3.4667250137 2.111920309
O 1.2431572544 1.5393671386 3.5389463667
C 0.0389657888 3.6236051725 4.7211118583
F -1.0364311725 4.4146716508 4.6098303862
F -0.0675244389 2.9012654794 5.8364550829
F 1.1324731702 4.3926692298 4.8079719467
O -0.3885440522 -0.08551407 5.9421306425
O -2.0515085145 -1.5355030405 7.1948752934
O -2.3048668168 -0.8997787169 4.7489868469
S -1.8035822619 -0.5732832615 6.1277877277
C -2.767753227 0.9391804197 6.6397121775
F -2.2621371337 1.4328458476 7.7771289803
F -2.7284884103 1.8886486405 5.7018201743
F -4.0469703111 0.5980538892 6.8494415674
C 0.325403135 -2.4730222593 -0.1367579104
C -1.0239449095 -2.6124108551 0.073380104
C -1.2078172869 -1.1845585032 0.688322644
C 0.3225495923 -1.2327615301 0.6431998387
O 1.0958015878 -0.6880023268 1.4396864549
O -1.5880955146 -1.1204451456 1.9983290828
O 1.4136332704 -3.0951207915 -0.6093479939
C 1.443675916 -4.4740856344 -1.0853233511
C -2.1016749752 -3.6361759628 0.0438233849
H -1.8135800552 -4.5931631038 -0.3967977466
H -2.9633505373 -3.2397454332 -0.5097134168
H -2.4426676248 -3.8116413033 1.0722373801
C 1.0456776587 -5.4396653156 0.0370426685
H 1.1680051079 -6.4740949444 -0.3045126671
H 1.6780736899 -5.2857207404 0.9143730243
H 0.006818091 -5.3102812729 0.3473275785
C 2.9217002421 -4.664632245 -1.442993296
H 3.0986086344 -5.6804063182 -1.8130231398
H 3.2240609593 -3.951197957 -2.2164602245
H 3.5439091629 -4.4966899931 -0.5587902872
C 0.5663573897 -4.6151015258 -2.3353972627
H -0.4891820864 -4.4297522019 -2.1204709758
H 0.8883552648 -3.9052523502 -3.1048110588
H 0.6538230706 -5.6284727818 -2.7437809643
C -1.9496707533 -0.2111334417 -0.2282974439
C -1.7751895069 -0.2153748332 -1.620639881
C -2.8366710848 0.713133207 0.336255231
H -1.0901668126 -0.9245852149 -2.0775121776
H -2.9606662436 0.7178594235 1.4124228749
C -2.4710148155 0.6837220321 -2.4295528389
C -3.5341144583 1.6111989471 -0.4740296669
H -2.319953689 0.6676704167 -3.506809494
H -4.215170017 2.3252965145 -0.017388357
C -3.3563017351 1.6008287905 -1.8584417734
H -3.8984009189 2.3032042349 -2.4874904318
O 1.9257695288 -3.2554660704 5.861679286
S 1.6483882818 -2.636672239 4.5712168516
C 2.7224912942 -3.5908343311 3.3820681347
O 2.0234345809 -1.2013464456 4.3686048386
O 0.2524029291 -2.7892832219 4.0116557771
F 2.6009980388 -3.1424618688 2.1275873
F 2.3720207477 -4.8897553834 3.3985381135
F 4.0048610287 -3.4910444388 3.7504426487

Frequency: 9.3710; 20.6064; 23.0709; 26.3453; 27.8494; 30.2318; 34.0627; 36.3827; 37.5852; 43.2847; 45.6770; 51.6352; 59.4927; 64.4364; 66.6949; 70.5202; 73.9923; 82.6188; 83.2361; 87.5476; 96.5188; 102.0900; 123.0498; 127.3472; 131.8222; 138.9342; 142.6699; 149.9669; 163.3151; 170.5297; 179.5868; 185.1058; 188.0050; 207.6666; 209.4823; 211.5424; 214.9067; 218.2513; 220.1183; 230.9844; 236.6188; 254.3777; 263.6771; 265.6722; 280.0094; 293.0129; 302.2389; 305.4641; 307.6946; 321.1314; 322.8396; 324.2965; 346.0203; 351.0148; 351.8990; 357.2548; 368.3758; 382.8988; 413.9438; 421.8689; 456.6237; 462.1328; 477.4410; 489.8312; 490.9039; 491.9226; 517.8836; 523.9120; 525.6029; 531.1864; 539.1725; 557.7922; 558.2699; 558.6784; 569.0513; 572.2818; 576.4622; 582.8692; 619.8619; 626.0208; 633.4481; 638.8158; 643.8989; 683.0053; 711.0267; 727.3331; 743.6732; 758.9448; 760.6937; 761.7922; 770.2483; 796.3347; 841.2169; 861.3788; 913.8329; 929.1097; 930.2775; 955.1274; 964.8679; 968.9564; 974.3459; 975.8575; 977.1034; 986.3224; 997.8080; 1017.1256; 1032.9709; 1053.1005; 1058.8307; 1061.0685; 1069.3730; 1094.0313; 1106.6318; 1112.1495; 1113.8209; 1133.9442; 1141.1186; 1187.9465; 1204.0943; 1207.4922; 1212.2454; 1214.4486; 1223.5062; 1226.6630; 1227.3596; 1229.7424; 1235.3580; 1246.2943; 1247.1393; 1248.5108; 1252.8363; 1279.5878; 1291.0092; 1303.8013; 1307.0566; 1320.4769; 1345.7724; 1347.2326; 1363.1104; 1426.3204; 1429.6795; 1432.1662; 1457.4671; 1492.4785; 1500.3304; 1503.7578; 1509.5414; 1519.0527; 1521.7892; 1528.0574; 1531.0238; 1539.8603; 1552.6225; 1639.2757; 1652.8109; 1659.9278; 1759.9611; 3042.1658; 3057.0297; 3061.0885; 3072.5187; 3094.0137; 3121.9799; 3131.6189; 3139.6981; 3141.1297; 3146.9790; 3152.4565; 3166.8088; 3175.0795; 3180.4921; 3192.6877; 3199.8771; 3249.7337.

Cartesian Forces: Max 0.000001145 RMS 0.000000401

Item	Value	Threshold	Converged?
Maximum Force	0.000000	0.000450	YES
RMS Force	0.000000	0.000300	YES
Maximum Displacement	0.000079	0.001800	YES
RMS Displacement	0.000016	0.001200	YES

FILE: Yb_Sq2_PhYbOTf3_Prod_Anion_B3LYP_M062x_Add56_Conf6-1.out

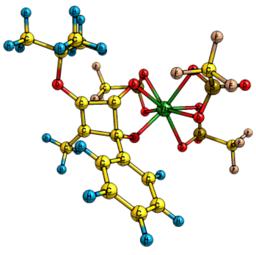

Conformer 13

E(RB3LYP/6-31G(d)/MWB59) = -3731.53032863
Convg = 0.7534D-08 -V/T = 2.0122

Temperature 195.150 Kelvin. Pressure 1.00000 Atm.
elec. and zero-point Energies= -3731.156267
elec. and thermal Energies= -3731.133458
elec. and thermal Enthalpies= -3731.132840
elec. and thermal Free Energies= -3731.201892

RB3LYP/6-31G(d)/MWB59/THF(IEFPCM) = -3731.59490429

RM06-2x/6-311+G(d,p)/MWB59/THF(IEFPCM) = -3731.43135921

Geometry at RB3LYP/6-31G(d)/MWB59
Charge = -1 Multiplicity = 1
Yb -0.0823553355 -0.474823729 3.6673503139
O 1.5578739326 1.3933588012 3.8451397261
S 0.598674824 2.4638451093 3.4190110121
O 0.4505403534 3.6354189807 4.2746004892
O -0.6674692658 1.7490229991 2.9986382118
C 1.2867309792 3.1613734238 1.8296989489
F 2.5216351195 3.6311601176 2.0522400511
F 1.3447455992 2.2470269307 0.858286409
F 0.5089168674 4.1711137061 1.4178019074
O -0.3449864435 0.2711854654 5.9838768039
O -2.2729026095 -0.8114321319 7.2360789259
O -2.2689306722 -0.4311444926 4.7244411618
S -1.8212090755 -0.0139894666 6.1022198425
C -2.607311033 1.655244491 6.3711453195
F -2.1679002346 2.1743620011 7.5243473008
F -2.3241411417 2.4970339743 5.3788511186
F -3.9387310913 1.5069154669 6.4454044252
C 0.8280553111 -2.2925605406 -0.1547121072
C -0.4530675878 -2.6969183807 0.1025918288
C -0.885784848 -1.3837532675 0.8084428739
C 0.6327238608 -1.1281382153 0.7165068418
O 1.3110012698 -0.4830375691 1.5229196882
O -1.2236766909 -1.4575936483 2.1330409126
O 1.8151524065 -2.9157360391 -0.8127172075
C 3.2150672338 -2.4876029435 -0.6699486628
C -1.168258243 -3.9911705781 -0.0204501144
H -0.5407692702 -4.7602978936 -0.4815096893
H -2.0841201571 -3.8711591342 -0.6142817845
H -1.4748883069 -4.33107509 0.9764385444
C 3.3724064475 -1.0313880881 -1.1232594904
H 4.4367956254 -0.7722227251 -1.1581379114
H 2.9541214973 -0.8940201124 -2.1268451156
H 2.8877185055 -0.3368230489 -0.4325851111
C 3.9466036697 -3.4363957345 -1.6233162272
H 5.0220320126 -3.2302537771 -1.6103482002
H 3.7884541807 -4.4772020465 -1.3223244517
H 3.5797870022 -3.3135058782 -2.6481085672
C 3.6772035094 -2.69268188 0.7756168179
H 3.1726296163 -2.0069906194 1.4608821063
H 3.4839307902 -3.7196799058 1.0997092707
H 4.7553279499 -2.5068896895 0.8478436546
C -1.8228316757 -0.5020975913 -0.0194046381
C -1.6751956241 -0.3565056296 -1.4072314969
C -2.8648043341 0.1790998978 0.6212689341
H -0.8757593606 -0.8806170453 -1.9240263306
H -2.9779046825 0.0641886426 1.693114686
C -2.5455455513 0.4546790718 -2.136074115
C -3.7376635971 0.9879932271 -0.1092672696
H -2.4127189997 0.5576658819 -3.2108986267
H -4.5392342593 1.5111039467 0.4065292756
C -3.5828717007 1.1303556071 -1.4891124187
H -4.2621351975 1.7628145116 -2.056046206
O 2.0052313371 -3.2479665778 6.1395596966
S 1.3399722292 -2.8702971337 4.8995689805
C 1.5731075086 -4.3412176696 3.7790536669
O 1.9305734211 -1.7247256841 4.1122472663
O -0.1409248433 -2.644556144 4.915376663
F 1.0153483592 -4.1256117523 2.5769967054
F 1.0095320146 -5.4255656819 4.3214116325
F 2.8809490378 -4.5790245302 3.5988378771

Frequency: 15.0247; 18.7292; 21.5304; 23.7874; 25.1529; 29.1541; 33.7381; 37.6157; 39.1011; 41.2296; 48.0586; 48.5649; 50.9460; 52.9986; 58.5601; 64.3045; 68.2536; 74.8391; 83.5176; 90.7186; 93.0778; 104.9098; 113.4541; 127.6265; 135.2693; 139.7607; 145.5533; 149.5009; 153.4572; 170.5395; 173.2968; 181.9200; 199.5310; 206.1311; 211.9227; 215.1783; 218.5370; 219.9972; 223.9847; 225.2545; 255.3123; 262.1794; 271.5215; 278.8044; 285.1162; 299.5639; 303.9216; 307.4696; 308.8086; 321.7229; 323.8490; 324.0909; 343.5110; 351.7675; 352.6012; 357.3620; 365.1365; 385.5214; 414.0275; 422.5832; 449.7708; 453.2458; 484.4708; 489.0082; 490.1794; 491.0267; 523.9776; 524.7627; 529.8273; 536.1870; 546.4145; 556.9520; 557.9920; 558.2236; 569.2222; 571.2754; 575.9368; 587.7967; 621.1486; 626.4575; 633.3286; 633.8854; 637.7856; 669.2866; 709.4120; 715.3527; 749.3359; 757.6502; 760.4729; 761.8606; 763.6825; 796.8687; 863.7727; 866.4707; 918.2524; 932.4211; 935.7749; 944.4416; 965.3621; 970.8634; 972.2785; 973.6951; 980.4094; 984.4254; 1004.0392; 1016.5547; 1030.5612; 1056.6865; 1060.6307; 1065.2725; 1070.3548; 1083.8714; 1102.7076; 1107.2418; 1117.2109; 1129.4553; 1153.9323; 1188.1564; 1208.4407; 1210.8147; 1210.9993; 1213.2571; 1215.3980; 1223.6097; 1231.0263; 1232.5030; 1236.9459; 1240.1282; 1246.0564; 1253.5541; 1256.7886; 1281.2316; 1296.0177; 1306.4626; 1307.8551; 1319.7333; 1346.4131; 1363.0504; 1366.2182; 1427.4981; 1429.8691; 1433.7731; 1458.7850; 1492.3628; 1502.3485; 1504.3614; 1506.8776; 1517.6557; 1519.4307; 1534.7824; 1538.3640; 1540.0302; 1554.8464; 1639.3950; 1657.1692; 1662.1479; 1761.1177; 3040.0334; 3053.5843; 3056.6507; 3064.5184; 3097.3294; 3117.4097; 3127.4553; 3128.5906; 3130.0501; 3134.7609; 3154.1247; 3162.0787; 3167.5687; 3181.2592; 3193.4986; 3200.5005; 3240.2113.

Cartesian Forces: Max 0.000001085 RMS 0.000000427

Item	Value	Threshold	Converged?
Maximum Force	0.000000	0.000450	YES
RMS Force	0.000000	0.000300	YES
Maximum Displacement	0.000052	0.001800	YES
RMS Displacement	0.000009	0.001200	YES

FILE:
Yb_Sq2_PhYbOTf3_Prod_Anion_B3LYP_M062x_Add56_Conf7.out

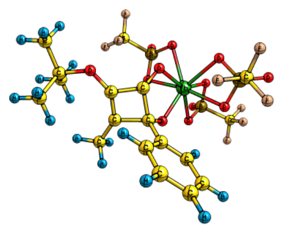

Conformer 14

E(RB3LYP/6-31G(d)/MWB59) = -3731.52137233
Convg = 0.7267D-08 -V/T = 2.0122

Temperature 195.150 Kelvin. Pressure 1.00000 Atm.
elec. and zero-point Energies= -3731.147369
elec. and thermal Energies= -3731.124555
elec. and thermal Enthalpies= -3731.123937
elec. and thermal Free Energies= -3731.192966

RB3LYP/6-31G(d)/MWB59/THF(IEFPCM) = -3731.58939605

RM06-2x/6-311+G(d,p)/MWB59/THF(IEFPCM) = -3731.42445345

Geometry at RB3LYP/6-31G(d)/MWB59
Charge = -1 Multiplicity = 1
Yb -0.1132753175 -0.5151576693 3.573116175
O 1.3673819911 1.4470289965 3.6665246031
S 0.3080438026 2.484048139 3.4298868347
O 0.2703248496 3.6448789317 4.3111727632
O -0.9775436287 1.7207677685 3.2222279241
C 0.6914325846 3.1798592698 1.7447806316
F 1.8848609578 3.7866395019 1.7670950341
F 0.7175304027 2.2136105177 0.8209816101
F -0.2423043166 4.0766131003 1.4034966467
O -0.2650434272 -0.1352041175 5.9795071221
O -1.977378683 -1.5402958829 7.2210986092
O -2.206634871 -0.8794477532 4.7812321574
S -1.6957061488 -0.576349527 6.1638493425
C -2.6041340832 0.9653148862 6.6857953703
F -2.1537434421 1.3655874781 7.8817133795
F -2.4363189167 1.9524413571 5.8068586516
F -3.9135311088 0.6939616924 6.7878587649
C 0.3949524019 -2.5078234585 -0.1259145866
C -0.9621103372 -2.5938626972 0.062195913
C -1.0944456011 -1.1726480725 0.7056985162
C 0.4335875361 -1.2844298333 0.679065823
O 1.2197247323 -0.7885040616 1.4945299563
O -1.4918977444 -1.1195072432 2.0102809732
O 1.4625227284 -3.165607719 -0.5973807622
C 1.4440385428 -4.5395770905 -1.0888221036
C -2.0815327311 -3.5699499377 -0.0036729501
H -1.8277195028 -4.5291209629 -0.4603045485
H -2.9176538655 -3.1261376438 -0.5604486645
H -2.4446176468 -3.7516172636 1.0160748805
C 0.9872666732 -5.5007392713 0.0147715294
H 1.0748463913 -6.5352666111 -0.3369584303
H 1.608977803 -5.3820796495 0.9051160441
H -0.0512652283 -5.3338394493 0.3077386946
C 2.9189570286 -4.7869797051 -1.4237069632
H 3.0601835163 -5.8046059598 -1.8038101643
H 3.2640649309 -4.0773993668 -2.1827259157
H 3.5318774348 -4.6558480587 -0.5268974339
C 0.583342752 -4.6295913732 -2.3550556259
H -0.466802848 -4.4010582525 -2.1557724661
H 0.9487105301 -3.9262051584 -3.1109313723
H 0.6341660874 -5.6414000139 -2.7733781758
C -1.7838832079 -0.1519427112 -0.2008102426
C -1.5555625099 -0.1052147852 -1.584481607
C -2.6788249362 0.763695959 0.3653903252
H -0.8651056215 -0.8084004083 -2.0425751316
H -2.8438871651 0.7270183517 1.4356283182
C -2.2045835459 0.8372845829 -2.3830008535
C -3.3304580353 1.7045246247 -0.4346606253
H -2.0115662434 0.8611395539 -3.4533925174
H -4.019625309 2.4101869342 0.023051228
C -3.0975792673 1.7461692966 -1.8104693624
H -3.6039103879 2.4815014143 -2.4316132836
O 1.921524027 -3.4021380868 5.8842050133
S 1.6691404134 -2.7687562991 4.5957949006
C 2.702550848 -3.7651762365 3.4048160626
O 2.101981226 -1.3498645637 4.3958720435
O 0.2676255394 -2.8639065007 4.0366451022
F 2.6094838571 -3.304124458 2.1520548321
F 2.291243091 -5.0461583037 3.4106582015
F 3.9865627338 -3.7284286907 3.7794674878

Frequency: 12.7006; 19.0373; 21.6848; 24.6786; 29.3532; 30.8375; 32.7477; 34.4990; 37.6750; 40.0737; 44.0302; 46.8343; 51.5925; 59.4650; 61.3457; 69.0854; 71.8532; 78.8588; 83.0003; 87.1857; 94.7157; 102.6630; 122.3971; 128.2738; 135.8268; 143.0667; 144.2151; 151.4047; 167.1272; 172.3764; 176.8556; 185.4296; 189.1097; 208.1602; 211.8012; 212.6081; 214.5508; 217.7267; 219.7149; 230.5687; 236.7852; 254.9057; 263.6979; 266.1187; 280.7147; 293.4073; 302.7978; 305.5518; 307.8149; 321.5269; 322.4669; 323.3209; 346.1957; 351.2939; 352.8336; 357.3469; 367.9095; 382.6860; 413.4241; 421.9313; 456.7481; 461.5060; 476.8767; 489.2917; 489.9825; 491.3863; 518.7966; 523.7157; 525.6254; 531.1278; 539.0197; 557.0062; 557.8919; 558.3104; 568.6091; 573.3909; 576.5778; 581.4282; 620.5370; 626.4541; 633.4142; 638.0512; 643.5320; 683.0589; 711.6495; 727.4188; 744.2034; 758.7309; 760.8353; 762.0097; 769.7340; 796.4130; 841.2259; 863.2501; 914.2921; 929.0527; 930.6145; 955.2180; 964.7733; 970.2907; 973.7456; 976.9037; 977.2963; 984.3284; 1000.9082; 1017.0293; 1032.6533; 1052.9893; 1058.7096; 1061.2242; 1069.2543; 1094.2864; 1105.9881; 1109.2660; 1114.0430; 1134.4759; 1141.2895; 1187.7933; 1204.2192; 1206.7744; 1212.4917; 1216.6607; 1223.1927; 1226.4917; 1231.4773; 1233.8849; 1235.3178; 1236.2350; 1246.5686; 1250.3756; 1252.5589; 1279.5960; 1291.0959; 1304.7894; 1308.9785; 1320.4197; 1345.4893; 1347.5017; 1362.7733; 1426.2699; 1429.7153; 1432.3151; 1457.5221; 1492.4200; 1500.3525; 1503.9583; 1509.4103; 1519.1680; 1521.9657; 1528.0780; 1531.2114; 1539.5389; 1552.7057; 1639.1006; 1652.7533; 1659.5437; 1760.4216; 3042.1202; 3056.9909; 3061.1041; 3072.6969; 3093.8901; 3121.8740; 3131.5567; 3139.8807; 3141.3411; 3146.9589; 3152.4553; 3166.9224; 3175.1912; 3180.3797; 3192.4817; 3199.7598; 3247.3798.

Cartesian Forces: Max 0.000000845 RMS 0.000000318

Item	Value	Threshold	Converged?
Maximum Force	0.000000	0.000450	YES
RMS Force	0.000000	0.000300	YES
Maximum Displacement	0.000038	0.001800	YES
RMS Displacement	0.000004	0.001200	YES

FILE: Yb_Sq2_PhYbOTf3_Prod_Anion_B3LYP_M062x_Add56_Conf7-1.out

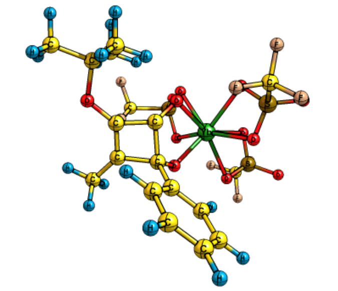

Conformer 15

E(RB3LYP/6-31G(d)/MWB59) = -3731.52931495
Convg = 0.4296D-08 -V/T = 2.0122

Temperature 195.150 Kelvin. Pressure 1.00000 Atm.
elec. and zero-point Energies= -3731.155276
elec. and thermal Energies= -3731.132439
elec. and thermal Enthalpies= -3731.131821
elec. and thermal Free Energies= -3731.201140

RB3LYP/6-31G(d)/MWB59/THF(IEFPCM) = -3731.59422596

RM06-2x/6-311+G(d,p)/MWB59/THF(IEFPCM) = -3731.43043683

Geometry at RB3LYP/6-31G(d)/MWB59
Charge = -1 Multiplicity = 1
Yb 0.0624416429 -0.5129601912 3.7420974515
O 1.7291997066 1.3285362308 3.8406949057
S 0.7658147528 2.419842621 3.4785459146
O 0.7031548235 3.5966197903 4.3356154124
O -0.5393313267 1.7291519765 3.1453301497
C 1.360531984 3.094881248 1.8417894133
F 2.6264646537 3.5150825151 1.970061017
F 1.3099657462 2.1806132807 0.8684907026
F 0.5950199688 4.1348904364 1.487092155
O -2.1231779939 -0.3754514263 4.80504269
O -2.361339132 1.481134923 6.5182504288
O -0.1742310868 0.2801616148 6.0499566538
S -1.6817020709 0.2617249788 6.0972896103
C -2.1150602063 -0.9752726381 7.4247543449
F -3.4492718418 -1.0995852083 7.486923495
F -1.5829218972 -2.1719145257 7.1893181272
F -1.6699815692 -0.5261929848 8.6062041796
C 0.8322816486 -2.3249448075 -0.122024594
C -0.4427872357 -2.7204941569 0.1761160867
C -0.8392441823 -1.4092613906 0.9081100601
C 0.6776770944 -1.1711766953 0.7701662634
O 1.3888618411 -0.5470087217 1.5653727706
O -1.1327604002 -1.4903727342 2.2423707445
O 1.7922918986 -2.9504026043 -0.8167298957
C 3.191945905 -2.5026987098 -0.7555554375
C -1.1744318958 -4.0069361004 0.0674552247
H -0.5665836649 -4.7826044815 -0.4087201506
H -2.1016983076 -3.8753626515 -0.5060693769
H -1.4639367654 -4.3446997627 1.070231189
C 3.3037136352 -1.0451742752 -1.2181728669
H 4.360886884 -0.7728543587 -1.3153466586
H 2.8258038421 -0.9142784269 -2.1956234145
H 2.8518341316 -0.3549526217 -0.5013388757
C 3.8802725082 -3.4429403929 -1.7487698631
H 4.9522221328 -3.2240951273 -1.7959488797
H 3.7515417476 -4.4854408507 -1.4396918937
H 3.4552082779 -3.3248526884 -2.7513529044
C 3.7400689513 -2.6989548237 0.6610372222
H 3.263107324 -2.0210314304 1.3732905998
H 3.5832228517 -3.72883409 0.9952412017
H 4.8171672481 -2.4943944958 0.6709860248
C -1.7925882738 -0.5092971603 0.1191819916
C -1.6983411416 -0.3644567678 -1.2733906809
C -2.7940154986 0.1913025099 0.8024697137
H -0.9308821483 -0.9031914701 -1.8226631588
H -2.867619363 0.078079858 1.8779750371
C -2.5811334493 0.4650608988 -1.9657415647
C -3.6787750724 1.0192619885 0.108364663
H -2.4900209868 0.5670066329 -3.0450267148
H -4.4470510936 1.5582316653 0.657251447
C -3.5772206233 1.1606766062 -1.2764871319
H -4.2656821532 1.8080542106 -1.8147815958
O 2.1745755238 -3.3029675194 6.1725144751
S 1.537206087 -2.9001741101 4.9251278283
C 1.824505834 -4.3327194582 3.7688000292
O 2.1189614968 -1.7191583187 4.1907597232
O 0.0503077905 -2.7079889597 4.911997606
F 1.2631776934 -4.1037773147 2.5709542949
F 1.2939339366 -5.4488276791 4.2802582298
F 3.1393803177 -4.5228910052 3.5885448074

Frequency: 12.3890; 19.0066; 21.0269; 23.4787; 25.3659; 27.5491; 33.6404; 35.5325; 38.2561; 39.1328; 45.9148; 48.7388; 52.2350; 55.2817; 56.5559; 65.7115; 66.3304; 76.5980; 84.0947; 89.0693; 92.0703; 105.6347; 113.7453; 130.3894; 136.0580; 140.0716; 143.8335; 150.8942; 152.4762; 163.4440; 176.6481; 181.0001; 199.6816; 206.7290; 212.5593; 214.5934; 217.0789; 219.1759; 220.0139; 225.5688; 256.2475; 261.7712; 271.1815; 279.7145; 285.6159; 300.2104; 303.0797; 308.1860; 308.3436; 322.0387; 322.8880; 324.3232; 343.6934; 350.9924; 352.1037; 357.1177; 364.8976; 385.8503; 414.4037; 423.0193; 449.7572; 453.1401; 483.7039; 489.7268; 490.1814; 491.2192; 523.6477; 524.9322; 530.0421; 535.2271; 546.8637; 557.0879; 557.9120; 558.3713; 568.0496; 572.1824; 576.7751; 588.2056; 620.5915; 624.9699; 633.1378; 633.6614; 639.3987; 670.4603; 709.3095; 715.4298; 748.3633; 758.1001; 759.8640; 762.0635; 763.4770; 796.7553; 864.1232; 866.9312; 918.5806; 932.0427; 935.9775; 944.4274; 965.0570; 970.7270; 971.3811; 976.3166; 981.6371; 985.5514; 1006.3693; 1016.3284; 1030.8991; 1056.2219; 1060.6641; 1065.3794; 1070.5827; 1084.3035; 1102.4857; 1107.9429; 1117.1062; 1131.1622; 1153.6396; 1188.3220; 1209.1110; 1209.8905; 1211.8600; 1213.6449; 1216.0826; 1224.1556; 1229.1434; 1232.4988; 1236.0626; 1237.3854; 1245.8834; 1256.0734; 1258.3457; 1281.2198; 1295.9937; 1303.3734; 1310.2202; 1320.9510; 1346.4515; 1363.2587; 1366.3816; 1427.4209; 1429.6657; 1433.7817; 1458.9106; 1492.2536; 1502.2563; 1504.2935; 1507.0127; 1517.4060; 1519.5790; 1534.6088; 1538.2108; 1540.1133; 1554.8500; 1639.3346; 1656.7640; 1661.7441; 1760.1083; 3039.2473; 3053.8888; 3056.7630; 3064.4585; 3096.0988; 3117.9685; 3127.4319; 3128.7989; 3129.7002; 3134.7879; 3153.7360; 3161.7878; 3167.3529; 3181.6160; 3193.6449; 3200.4825; 3240.2520.

Cartesian Forces: Max 0.000001297 RMS 0.000000412

Item	Value	Threshold	Converged?
Maximum Force	0.000000	0.000450	YES
RMS Force	0.000000	0.000300	YES
Maximum Displacement	0.000062	0.001800	YES
RMS Displacement	0.000015	0.001200	YES

FILE:
Yb_Sq2_PhYbOTf3_Prod_Anion_B3LYP_M062x_Add56_Conf8.out

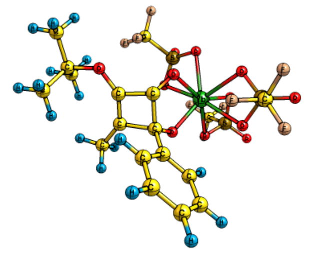

Conformer 16

E(RB3LYP/6-31G(d)/MWB59) = -3731.52166718
Convg = 0.7817D-09 -V/T = 2.0122

Temperature 195.150 Kelvin. Pressure 1.00000 Atm.
elec. and zero-point Energies= -3731.147682
elec. and thermal Energies= -3731.124867
elec. and thermal Enthalpies= -3731.124249
elec. and thermal Free Energies= -3731.193201

RB3LYP/6-31G(d)/MWB59/THF(IEFPCM) = -3731.58966670

RM06-2x/6-311+G(d,p)/MWB59/THF(IEFPCM) = -3731.42506926

Geometry at RB3LYP/6-31G(d)/MWB59
Charge = -1 Multiplicity = 1
Yb -0.0065792158 -0.5082819958 3.6323001551
O 1.470588272 1.4423926038 3.6196079899
S 0.3992147482 2.4896038451 3.5008961291
O 0.4600247237 3.6421550898 4.3902371863
O -0.9050100963 1.7356132489 3.4114551766
C 0.6252739399 3.1958001901 1.7915565551
F 1.8141258953 3.8063826 1.7074994606
F 0.5708343348 2.2330198057 0.8642979929
F -0.338066865 4.090469739 1.5396257455
O -2.0727756148 -0.8303816248 4.8951112015
O -2.3635040446 0.6585031019 6.9317890687
O -0.1009460123 -0.0635343091 6.0295244972
S -1.5701661466 -0.3414920068 6.2269706048
C -1.6164990706 -1.8419263103 7.3310441793
F -2.8906626025 -2.2172168379 7.517284918
F -0.9427998052 -2.8586979164 6.7934986348
F -1.0790254097 -1.5418274012 8.5198294387
C 0.3968614663 -2.513026279 -0.0721008364
C -0.9554006612 -2.5894780549 0.1508447683
C -1.0625808818 -1.1640305215 0.7900340225
C 0.4637364748 -1.2865574418 0.7261716103
O 1.2731695744 -0.7935840874 1.51986894
O -1.4270242644 -1.1002732487 2.1039459322
O 1.4484607807 -3.1793144339 -0.5672950521
C 1.4109990568 -4.5572241007 -1.0458358434
C -2.0823076272 -3.5585957454 0.1171816067
H -1.8426720299 -4.5260198283 -0.3296013963
H -2.9245961546 -3.1169898091 -0.432109155
H -2.4296717984 -3.7229923502 1.1452575405
C 0.9813236095 -5.5055761516 0.079405198
H 1.0511085294 -6.5438549553 -0.2652272853
H 1.6309377413 -5.3834186769 0.9492228463
H -0.0466077848 -5.3282761086 0.4022534242
C 2.8751158097 -4.8138643314 -1.4190421559
H 3.0022548928 -5.8361047863 -1.7916767192
H 3.2013637923 -4.1139219872 -2.1951607197
H 3.5131633569 -4.6750554603 -0.5411076379
C 0.5152712873 -4.6554254729 -2.2868931383
H -0.5282882057 -4.4219076603 -2.0607627644
H 0.8616583503 -3.9596928823 -3.0586069471
H 0.5516275716 -5.6709297001 -2.6977197296
C -1.7681091898 -0.1429745842 -0.1034156463
C -1.5762820194 -0.1049128407 -1.4928137601
C -2.6398444396 0.7829668453 0.4820703427
H -0.9041311934 -0.816103589 -1.9656039792
H -2.7783633316 0.7521143839 1.5564418203
C -2.2383169062 0.839462282 -2.2783696789
C -3.3043845145 1.7257392611 -0.3049690631
H -2.0737671777 0.8566192731 -3.3536426665
H -3.9749894052 2.4393444715 0.1676463679
C -3.1077562737 1.7588637336 -1.6866759054
H -3.6240670246 2.4956938663 -2.2977462689
O 2.0412760109 -3.4504187135 5.8595647047
S 1.7949177593 -2.7821332217 4.5865885601
C 2.8487434078 -3.7363261202 3.3792655737
O 2.2149844304 -1.353822798 4.4367532895
O 0.3989371119 -2.8738137355 4.0145277516
F 2.7416702992 -3.2634025736 2.1333864693
F 2.4661765089 -5.0268895244 3.3718612861
F 4.1318456267 -3.6751359927 3.7531371172

Frequency: 13.1806; 19.1575; 22.8613; 24.0558; 30.1926; 30.9409; 33.1436; 35.6193; 38.0959; 39.9356; 45.0719; 47.8135; 53.9307; 58.5000; 60.1734; 68.6129; 72.2568; 77.0358; 82.8541; 85.6622; 92.8350; 102.7365; 121.4519; 127.5263; 134.6255; 142.4541; 145.2212; 150.4291; 168.3216; 172.2241; 175.6428; 186.0887; 189.2884; 208.8082; 212.3694; 214.5271; 215.7709; 217.3814; 218.0661; 230.2921; 237.0925; 254.7046; 263.7725; 266.1982; 280.4176; 293.1005; 303.2177; 305.2353; 307.6557; 321.5324; 321.8942; 323.2801; 346.0751; 351.2378; 352.9056; 357.7363; 367.7787; 382.7005; 413.2508; 421.6224; 456.7536; 461.7495; 476.7569; 489.1229; 489.7475; 491.1868; 518.9429; 524.0334; 525.0645; 530.9795; 538.7779; 556.8306; 557.7773; 558.2894; 569.2563; 571.8623; 577.1612; 581.3971; 620.9137; 626.6097; 633.2704; 637.1415; 643.6071; 683.2325; 711.5596; 727.4278; 744.2619; 759.0210; 760.2734; 761.8057; 769.5232; 796.2739; 841.1865; 863.6109; 914.3487; 929.1750; 930.5900; 955.0436; 964.7546; 970.2914; 974.3981; 975.4470; 977.4838; 983.8441; 1001.5777; 1016.8180; 1032.5151; 1052.9988; 1058.6009; 1061.1939; 1069.2391; 1094.3521; 1106.0645; 1109.0480; 1113.2806; 1134.5393; 1141.0589; 1187.8374; 1202.0399; 1206.7224; 1213.2714; 1216.6859; 1223.8398; 1227.2352; 1232.1149; 1233.0957; 1235.6975; 1237.0423; 1246.6873; 1250.0374; 1250.4756; 1279.6881; 1291.0728; 1306.5168; 1307.6764; 1320.1611; 1345.3685; 1347.3562; 1362.7270; 1426.2883; 1429.7249; 1432.3486; 1457.5640; 1492.4029; 1500.4130; 1503.9673; 1509.4688; 1519.1188; 1522.0324; 1528.0797; 1531.2431; 1539.4802; 1552.7668; 1639.0545; 1653.3997; 1659.5732; 1761.2405; 3042.0491; 3057.0182; 3061.0797; 3072.3812; 3093.8121; 3121.9150; 3131.5458; 3139.9435; 3141.5857; 3146.7519; 3152.6715; 3166.8949; 3174.4870; 3180.6671; 3192.6092; 3199.7873; 3245.3758.

Cartesian Forces: Max 0.000001565 RMS 0.000000561

Item	Value	Threshold	Converged?
Maximum Force	0.000000	0.000450	YES
RMS Force	0.000000	0.000300	YES
Maximum Displacement	0.000042	0.001800	YES
RMS Displacement	0.000010	0.001200	YES

FILE: Yb_Sq2_PhYbOTf3_Prod_Anion_B3LYP_M062x_Add56_Conf8-1.out

REFERENCES AND NOTES FOR SUPPORTING INFORMATION

[1] M. Mohamed, T. P. Gonçalves, R. J. Whitby, D. C. Harrowven, H. F. Sneddon, *Chem. Eur. J.* **2011**, *17*, 13698–13705.

[2] *Preparation of squarate esters:* H. Liu, C. S. Tomooka, H. W. Moore, *Synth. Comm.* **1997**, *12*, 2177–2180.

[3] *Preparation of cyclobutenedione **1a**:* M. W. Reed, D. J. Pollart, S. T. Perri, L. D. Foland, H. W. Moore, *J. Org. Chem.* **1988**, 53, 2477–2482.

[4] *Preparation of cyclobutenedione **1b**:* A. Enhsen, K. Karabelas, J. M. Heerding, H. W. Moore, *J. Org. Chem.* **1990**, *55*, 1177–1185.

[5] L. S. Liebeskind, S. Iyer, C. F. Jewell Jr., *J. Org. Chem.*, **1986**, *51*, 3065–3067.

[6] B. M. Trost, O. R. Thiel, H.-C. Tsui, *J. Am. Chem. Soc.* **2003**, *125*, 13155–13164.

[7] 5*H*-furanone **29** was extremely sensitive towards aerial oxidation. To eliminate such side reactions, product solutions were collected under Ar. After evaporation of the solvent, the rotary evaporator was filled with Ar and the product was stored under Ar. Spectral analyses were conducted in degassed solvents (effected by immersion in a sonication bath under Ar for 30 min).

[8] C. M. Garner, B. C. Mossman, M. E. Prince, *Tetrahedron Lett.* **1993**, *34*, 4273–4276.

[9] G. B. Marini Bettòlo, C. G. Casinovi, C. Galeffi, *Tetrahedron Lett.* **1965**, *6*, 4857–4864.

[10] a) M. Dolg, H. Stoll, A. Savin, H. Preuss, *Theor. Chim. Acta* **1989**, *75*, 173–194; b) M. Dolg, H. Stoll, H. Preuss, *Theor. Chim. Acta* **1993**, *85*, 441–450.

[11] Y. Zhao, D. Truhlar, *Theor. Chem. Acc.* **2008**, *120*, 215−241.

[12] S. Miertuš, E. Scrocco, J. Tomasi, *Chem. Phys.* **1981**, *55*, 117–129.

[13] Gaussian 09, Revision A.02, M. J. Frisch, G. W. Trucks, H. B. Schlegel, G. E. Scuseria, M. A. Robb, J. R. Cheeseman, G. Scalmani, V. Barone, B. Mennucci, G. A. Petersson, H. Nakatsuji, M. Caricato, X. Li, H. P. Hratchian, A. F. Izmaylov, J. Bloino, G. Zheng, J. L. Sonnenberg, M. Hada, M. Ehara, K. Toyota, R. Fukuda, J. Hasegawa, M. Ishida, T. Nakajima, Y. Honda, O. Kitao, H. Nakai, T. Vreven, J. A. Montgomery, Jr., J. E. Peralta, F. Ogliaro, M. Bearpark, J. J. Heyd, E. Brothers, K. N. Kudin, V. N. Staroverov, R. Kobayashi, J. Normand, K. Raghavachari, A. Rendell, J. C. Burant, S. S. Iyengar, J. Tomasi, M. Cossi, N. Rega, J. M. Millam, M. Klene, J. E. Knox, J. B. Cross, V. Bakken, C. Adamo, J. Jaramillo, R. Gomperts, R. E. Stratmann, O. Yazyev, A. J. Austin, R. Cammi, C. Pomelli, J. W. Ochterski, R. L. Martin, K. Morokuma, V. G. Zakrzewski, G. A. Voth, P. Salvador, J. J. Dannenberg, S. Dapprich, A. D. Daniels, O. Farkas, J. B. Foresman, J. V. Ortiz, J. Cioslowski, and D. J. Fox, Gaussian, Inc., Wallingford CT, **2009**.

[14] a) J. J. P. Stewart, *J. Mol. Model*. **2013**, *19*, 1–32; b) J. D. L. Dutra, M. A. M. Filho, R. O. Freire, G. B. Rocha, A. M. Simas, J. J. P. Stewart, *J. Chem. Theory Comput.* DOI: 10.1021/ct 301012h. c) MOPAC2012, J. J. P. Stewart, Stewart Computational web: HTTP://OpenMOPAC.net.

[15] ChemCraft (G. A. Zhurko, http://www.chemcraftprog.com).

CONTINUATION OF REFERENCE 1 AND 5 FROM MAIN ARTICLE

[1] h) P. Wipf, C. R. Hopkins, *J. Org. Chem.* **1999**, *64*, 6881–6887; i) T. J. Onofrey, D. Gomez, M. Winters, H. W. Moore, *J. Org. Chem.* **1997**, *62*, 5658–5659; j) J. M. MacDougall, H. W. Moore, *J. Org. Chem.* **1999**, *64*, 7445–7450; k) Y. Xiong, H. W. Moore, *J. Org. Chem.* **1996**, *61*, 9168–9177; l) C. S. Tomooka, H. Liu, H. W. Moore, *J. Org. Chem.* **1996**, *61*, 6009–6012; m) K. H. Lee, H. W. Moore, *Tetrahedron Lett*. **1993**, *34*, 235–238; n) S. T. Perri, H. J. Dyke, H. W. Moore, *J. Org. Chem.* **1989**, *54*, 2032–2034; o) M. W. Reed, H. W. Moore, *J. Org. Chem.* **1988**, *53*, 4166–4171; p) M. W. Reed, H. W. Moore, *J. Org. Chem.* **1987**, *52*, 3491–3492.

[5] See also g) T. Hattori, M. Date, K. Sakurai, N. Morohashi, H. Kosugib, S. Miyanoa, *Tetrahedron Lett.* **2001**, *42*, 8035–8038; h) T. J. Greshock, R. L. Funk, *Org. Lett.* **2001**, *3*, 3511–3514; i) T. J. Blackburn, M. Helliwell, M. J. Kilner, A. T. L. Lee, E. J. Thomas, *Tetrahedron Lett.* **2009**, *50*, 3550–3554; j) K. Chibale, N. Greeves, L. Lyford, J. E. Pease, *Tetrahedron Asymm.* **1993**, *4*, 2407–2410; k) J. A. Bacigaluppo, M. I. Colombo, M. Gonzalez-Sierra, M. D. Preite, J. Zinczuk, E. A. Ruveda, H. Duddeck, M. Hiegemann, *J. Chem. Soc., Perkin Trans. 1* **1993**, 2009–2015; l) T. Laube, J. Schroder, R. Stehle, K. Seifert, *Tetrahedron* **2002**, *58*, 4299–4309; m) T. J. Donohoe, J. Y. K. Chiu, R. E. Thomas, *Org. Lett.* **2007**, *9*, 421–424; n) M. Sada, S. Matsubara, *Tetrahedron* **2011**, *67*, 2612–2616.
